# ﻿A taxonomic backbone for the Plumbaginaceae (Caryophyllales)

**DOI:** 10.3897/phytokeys.243.122784

**Published:** 2024-06-20

**Authors:** Maryam Malekmohammadi, Konstantina Koutroumpa, Manuel B. Crespo, Gianniantonio Domina, Nadja Korotkova, Hossein Akhani, Sabine von Mering, Thomas Borsch, Walter G. Berendsohn

**Affiliations:** 1 Botanischer Garten und Botanisches Museum Berlin, Freie Universität Berlin, Königin-Luise-Straße 6-8, 14195 Berlin, Germany Freie Universität Berlin Berlin Germany; 2 Halophytes and C4 Plants Research Laboratory, Department of Plant Sciences, School of Biology, College of Sciences, University of Tehran, P.O. Box 14155-6455, Tehran, Iran University of Tehran Tehran Iran; 3 Departamento de Ciencias Ambientales y Recursos Naturales (dCARN), Universidad de Alicante, Apdo. 99, 03080 Alicante, Spain Universidad de Alicante Alicante Spain; 4 Department of Agricultural, Food and Forest Sciences, University of Palermo, Viale delle Scienze, bldg. 4, 90128, Palermo, Italy University of Palermo Palermo Italy; 5 Current address: Museum für Naturkunde, Leibniz Institute for Evolution and Biodiversity Science, Invalidenstraße 43, 10115 Berlin, Germany Museum für Naturkunde Berlin Germany

**Keywords:** Caryophyllales, EDIT Platform, phylogenetic relationships, *Statice*, taxon concept, World Flora Online

## Abstract

A taxonomic backbone of the Plumbaginaceae is presented and the current state of knowledge on phylogenetic relationships and taxon limits is reviewed as a basis for the accepted taxon concepts. In total, 4,476 scientific names and designations are treated of which 30 are not in the family Plumbaginaceae. The Plumbaginaceae are subdivided in three tribes with 26 genera and 1,179 accepted species. Two subgenera, 17 sections, two subsections and 187 infraspecific taxa are accepted. At the species and infraspecific level 2,782 synonyms were assigned to accepted taxa, whereas 194 names were excluded from the core checklist (i.e., unplaced taxa, infrageneric subdivisions with still uncertain application, names of verified uncertain application, invalid horticultural names, excluded names from other families, other excluded designations, and unresolved names). The EDIT Platform for Cybertaxonomy was utilized as the tool to compile and manage the names and further taxonomic data under explicit taxon concepts. Secundum references are given in case taxon concepts were taken from the literature, whereas this study serves as reference for newly circumscribed taxa. The family’s division into the tribes Aegialitideae, Limonieae, and Plumbagineae departs from earlier two-subfamily classifications, prompted by recent phylogenetic findings that challenge the subfamilial affinity of *Aegialitis*. The genus *Acantholimon* was extended to include *Gladiolimon*, as currently available phylogenetic and morphological data support this merger. In *Limonium*, all accepted species could be assigned to sections and subsections or the “Mediterranean lineage”, respectively, making use of the phylogenetic distribution of their morphological characters and states. A new combination and/or status is proposed for *Dyerophytumsocotranum*, *Limoniumthymoides*, *Limonium×fraternum*, *Limonium×rossmaessleri*, and Limoniumsect.Jovibarba. Special attention is given to nomenclatural issues, particularly for *Staticenomenambiguum* to resolve the names under accepted names. The use of artificial groupings like “aggregates”, “complexes” and “species groups” in alpha-taxonomic treatments is discussed. The taxonomic backbone will receive continued updates and through the Caryophyllales Taxonomic Expert Network, it contributes the treatment of the Plumbaginaceae for the World Flora Online.

## ﻿Introduction

The Plumbaginaceae Juss. is a nearly cosmopolitan family of the order Caryophyllales that is most diverse in the northern hemisphere. The majority of its species are halophytes or psammophytes, growing on salty soils or in coastal habitats, while another large group of species are cold-adapted orophytes of arid regions. The generic concepts in this family have varied over time. The last family-wide synopsis accepted 29 genera (Hernández-Ledesma et al. 2015) compared to Kubitzki’s (1993) treatment with 27 accepted genera. Most of the species are concentrated in the large genera *Limonium* Mill. (ca. 600 spp.), *Acantholimon* Boiss. (ca. 200 spp.) and *Armeria* Willd. (ca. 100 spp.), whereas the other genera are small or monotypic, segregate genera (Kubitzki 1993).

Plumbaginaceae are monophyletic and sister to Polygonaceae (Lledó et al. 1998; Cuénoud et al. 2002; Schäferhoff et al. 2009; Yang et al. 2015; Yao et al. 2019). Plumbaginaceae has been divided into two subfamilies, Plumbaginoideae Burnett and Limonioideae Reveal (= Staticoideae), and three tribes, Plumbagineae Dumort. belonging to Plumbaginoideae, and Aegialitideae Z.X.Peng and Limonieae Reveal belonging to Limonioideae, based on molecular phylogenies (Lledó et al. 2001, 2005a; Koutroumpa et al. 2018). Compared to the Plumbagineae and Aegialitideae, the Limonieae stands out by more than 90 percent of the species diversity of the family. Among the large genera of Limonieae the monophyletic status of *Limonium* and *Armeria* was confirmed by investigations with dense taxon sampling (Lledó et al. 1998, 2005a; Malekmohammadi et al. 2017a; Koutroumpa et al. 2018), whereas *Acantholimon* turned out to be non-monophyletic as currently classified (Moharrek et al. 2017; Koutroumpa et al. 2018). *Plumbago*, the largest genus of the Plumbagineae, also appears as non-monophyletic (Lledó et al. 2001, 2005a; Koutroumpa et al. 2018).

**Figure 1.**
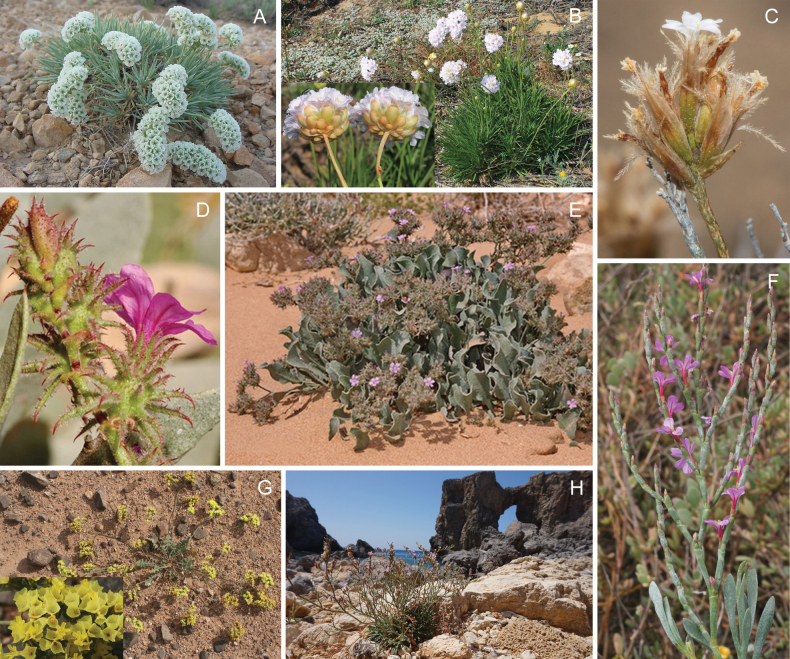
Morphological and habitat diversity in the family Plumbaginaceae. Limonieae: **A***Acantholimonpterostegium* Bunge **B***Armeriapungens* (Brot.) Hoffmanns. & Link **C***Bakerolimonplumosum* (Phil.) Lincz. **D***Ceratolimonfeei* (Girard) M.B.Crespo & Lledó **E***Ceratolimonweygandiorum* (Maire & Wilczek) M.B.Crespo & Lledó **F***Limoniastrummonopetalum* (L.) Boiss. **G***Limoniumbonduellei* (T.Lestib.) Kuntze **H***Limoniumvirgatum* (Willd.) Fourr. Photos **A** by Hossein Akhani **B, F, G** by Mario Martínez-Azorín **C** by Sergio Ibáñez **D, E** by José Quiles **H** by Konstantina Koutroumpa.

**Figure 2.**
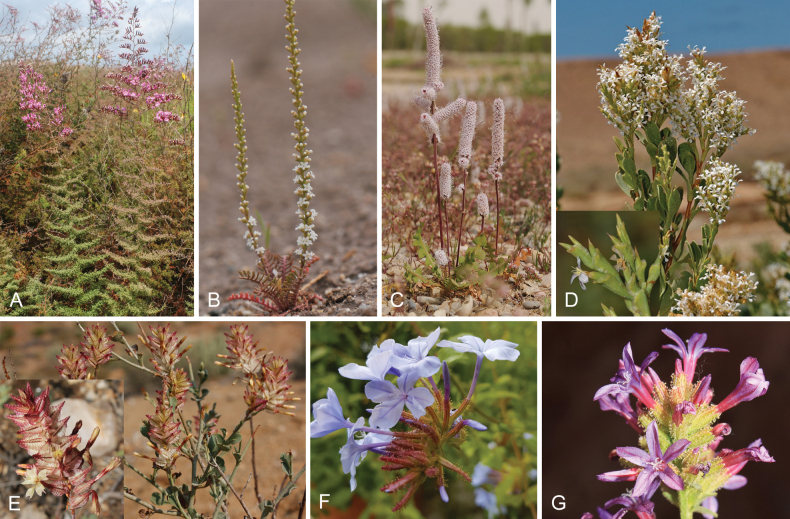
Morphological and habitat diversity in the family Plumbaginaceae. Limonieae: **A***Limoniumthymoides* (Girard) M.B.Crespo **B***Psylliostachysleptostachya* (Boiss.) Roshkova **C***Psylliostachysspicata* (Willd.) Nevski **D***Saharanthusifniensis* (Caball.) M.B.Crespo & Lledó. Plumbagineae: **E***Dyerophytumafricanum* (Lam.) Kuntze **F***Plumbagoauriculata* Lam. **G***Plumbagoeuropaea* L. Photos **A, E, G** by Mario Martínez-Azorín **B, C, F** by Hossein Akhani **D** by José Quiles.

The Plumbaginaceae are primarily perennial herbs and shrubs, rarely climbers, characterized by flowers that have stamens opposite the petals, a single basal anatropous ovule with curled funicle, an endotrophic transmitting tissue projecting inward from the base of the style, and salt (chalk) glands on leaves and stems (known as ‘Licopoli’ or ‘Mettenius’ organs). These traits are regarded as synapomorphies for the family (Labbe 1962; Kubitzki 1993; De Laet et al. 1995). Figures 1, 2 as well as two links of herbarium images of *Aegialitisannulata* R. Br. illustrate a segment of the morphological and ecological variability found within the family (https://herbarium.bgbm.org/object/B100518467; https://herbarium.bgbm.org/object/B100745686).

Estimates of species diversity have varied considerably, ranging from about 650 species (Kubitzki 1993) to 1000 (Hernández-Ledesma et al. 2015) or over 1000 species (Lledó et al. 1998), with the differences ascribed primarily to species number estimates in large genera with numerous microspecies. For example, estimated species numbers in *Limonium* vary from 350 (Hernández-Ledesma et al. 2015), 400–500 (Erben 1993; Brullo and Erben 2016), up to about 600 (Koutroumpa et al. 2018; Hassler 2023), and 708 (Govaerts 2023). Many of the recently described species in *Limonium* are small-ranged apomictic polyploids, for which taxonomic circumscriptions are particularly challenging. A prominent example of a significant increase in species number is found in a recent monograph of *Limonium* in Greece (Brullo and Erben 2016) that almost doubled the number of species previously known for the country by describing many new apomictic polyploid morphospecies.

This species backbone is part of the Global Caryophyllales Synthesis initiative, which aims at generating and maintaining a dynamic synthesis of data and knowledge on the species diversity of this order of flowering plants in a single open-access portal (Borsch et al. 2015, 2020; Arias et al. 2016). The Caryophyllales Network functions as a Taxonomic Expert Network dedicated to Caryophyllales within the World Flora Online (WFO) (http://www.worldfloraonline.org/) which acts as a community-driven authoritative source of information for the world’s plants (Wyse-Jackson and Miller 2015; Borsch et al. 2020). The WFO Plant List (https://wfoplantlist.org) now serves as the taxonomic backbone of the WFO and has replaced The Plant List (TPL 2013) that was the first consistent global species list for angiosperms, which is no longer curated (Schellenberger-Costa et al. 2023). Among the taxonomic backbones already published by the Caryophyllales Taxonomic Expert Network are the families Nepenthaceae (Berendsohn et al. 2018), Cactaceae (Korotkova et al. 2021), Aizoaceae (Berendsohn 2022), and the genus *Dianthus*, Caryophyllaceae (Fassou et al. 2022). The current work started with treatment of the names of *Limonium*. Considering that *Statice* is a rejected name with the respective species mostly belonging to *Limonium* or *Armeria* (Kuntze 1891) and that a considerable part of *Statice* names were still unresolved, it was mandatory to work in a broader scope including the subfamily Limonioideae to resolve these names. Finally, to accommodate recent phylogenetic results that inform changes at the circumscriptions of major taxonomic units below the family level (e.g., subfamilies and tribes), we extended the taxonomic treatment to the entire family.

The classification presented in this taxonomic backbone is built upon monophyletic groups, where possible. Our approach was to evaluate the available phylogenetic literature on the Plumbaginaceae. A detailed review on the state of knowledge as well as the evolution and diversity of Plumbaginaceae is provided.

The taxonomic backbone aims at including all validly published names and assigning them to the status as accepted names or synonyms. To be comprehensive, it also comprises (invalid) designations published in the literature or covered by online databases. This was deemed important when using the taxonomic backbone as a reference for name matching in meta-analysis of biodiversity data which also have to handle taxonomically less accurate sources.

## ﻿Materials and methods

### ﻿Informatics tools

The EDIT Platform for Cybertaxonomy (Ciardelli et al. 2009; Berendsohn 2010; BGBM 2011+, 2016+, Luther et al. 2019) (https://cybertaxonomy.eu/) was used to manage the taxonomic data, and to present them online as well as in text document format. The platform unites a set of open-source software tools that have been developed over the past 25 years at the Botanic Garden and Botanical Museum Berlin. This platform aims at including all aspects of taxonomic treatments and the workflows to create and maintain them (Borsch et al. 2015; Kilian et al. 2015; Henning et al. 2018). It provides the database system, editing tools, the online portal, and publication pipelines for this treatment. All sources of information can be cited for almost any item in the database, so that the information is transparent and appropriately credited.

The database component is structured according to the Common Data Model (CDM), a fully standard-based object-oriented data-model covering in detail the entire scope of taxonomic data (Müller et al. 2017). The principal software tool used in the context of the work presented here was the Taxonomic Editor, an operating-system independent frontend used to input and edit the taxonomic data in the online CDM database.

The appended taxonomic backbone was generated from an output of the EDIT platform using the functionalities of MS Access and MS Word processing software (Berendsohn et al. 2018). The contribution to the World Flora Online taxonomic backbone will be accomplished by submitting a WFO-DwCA (Darwin Core Archive) file generated from the EDIT platform.

### ﻿Data entry, sources of taxonomic information and editorial workflows

The terminology, editorial approaches, and the handling of source citations in the EDIT Platform are applied here as described in Berendsohn et al. (2018), Korotkova et al. (2021) and The Caryophyllales Network (2024+; https://caryophyllales.org/Editorial).

A list of Plumbaginaceae names was received from the World Flora Online (WFO 2017). This list was based on The Plant List 1.1 (TPL 2013) and included 2925 names with a unique WFO-identifier. The list was uploaded to the Tropicos (1991+) name matching service to obtain publication details, author and literature abbreviations were standardized and the names then matched with and imported into the EDIT Platform, resulting in a dataset comprising 2990 names. The first author then preliminarily placed the imported names not yet classified as either taxon names or synonyms. Afterwards, the names from the World Checklist of Vascular Plants (WCVP, obtained from Kew in December 2019) were matched with the records already present in the database and further names covered in the Euro+Med PlantBase were added manually. Newly published names or names that were missing from other databases were also entered manually.

Botanical literature, both in print and online, online databases, phylogenetic studies, monographs, regional or species group treatments and checklists as well as personal taxonomic knowledge of the authors were used to evaluate the taxon concepts at species level.

The taxonomic backbone includes a core part with accepted names and their synonyms. Names excluded from the core checklist were assigned to the following categories: “Unplaced taxa” currently contains only 2 invalidly published hybrid designations that were described by Pignatti and used in later publications. “Unplaced generic subdivisions” contains names of sections and subsections that we refrained from classifying awaiting further evidence from phylogenetic studies. “Names of verified uncertain application” lists names that probably will never be placed. The categories “Invalid horticultural names and combinations” and “Excluded designations” list designations that have been in use but which we did not want to include in the synonymy (e.g. erroneous author citations). “Excluded names” contains names outside the Plumbaginaceae that were erroneously part of the original WFO backbone. In contrast, the “unresolved names” offers a provisional category for practical reasons to accommodate names for which the correct application or status has yet to be determined. To classify the unresolved names in the correct place, further literature and/or herbarium revisions are required but this investigation exceeds the scope of this study. The circumscription of taxa is always indicated by a secundum “sec.” reference (Berendsohn 1995), a reference that indicates the circumscription of a taxon and its distinction from other taxa. The sec. references are either literature references, or original work done here, and then are referred as this publication. The “syn. sec.” reference of the synonyms refers to a reference stating the synonymy to the accepted name or to one of its synonyms. The sec. reference of the names that are excluded from the core taxonomic backbone is normally the source of the name, i.e., the dataset from where the name has been imported.

The authors collaborated both by exchange of corrections in the formatted output produced by the EDIT Platform and by using a preliminary password-protected online portal that gave direct access to the CDM database.

### ﻿Key literature sources

The Euro+Med Plant Base (2006+) as a continental-level checklist was used as primary source for taxon concepts at species level for many of the European, Mediterranean and North African Plumbaginaceae species (Plumbaginaceae treatment by Domina 2011+). The taxonomic treatment by Erben (1993) in the Flora Iberica was further considered for the species from the Iberian Peninsula and the Balearic Islands. Different literature was reviewed for the Italian species (e.g., Brullo 1988; Arrigoni 2015; Peruzzi et al. 2015; Brullo and Guarino 2017). A checklist of vascular plants of Greece (Dimopoulos et al. 2013, 2016) as well as the treatment by Brullo and Erben (2016) were the main source for species from Greece. Sell (2018) is used as the principal reference for the species of Great Britain.

The African species were treated based on the African Plant Database (http://africanplantdatabase.ch), floras and other literature (e.g., Lobin et al. 1995; Mucina and Hammer 2019).

Taxa from SW and Central Asia and Russia were treated according to relevant literature (e.g., Linczevski 1952; Rechinger and Schiman-Czeika 1974; Bokhari and Edmondson 1982) but also based on the personal knowledge of the first author on taxa from the Caucasus and Middle East.

The accepted species from SE Asia and China are based on literature and the online version of Flora of China (Peng and Kamelin 1996) (http://www.efloras.org).

The taxon concept of American taxa at species level were adopted from the Vascular Plants of the Americas online database (Ulloa Ulloa et al. 2018+) (https://www.tropicos.org/Project/VPA), Flora of North America (Morin 1993) (http://floranorthamerica.org/Plumbaginaceae) and the treatments by Luteyn (1976, 1990).

The majority of the *Statice* species were transferred to *Armeria*, *Limonium* or other Plumbaginaceae genera by Kuntze (1891). His treatment is the main source of nomenclatural information for *Statice* in our database together with other literature.

A total of 198 different literature references or online databases were used as secundum reference or in notes for Plumbaginaceae.

### ﻿Infrageneric taxa

Below the genus, we included the subgenera and sections of *Limonium* that had been revised in recent studies (e.g., Malekmohammadi et al. 2017a; Koutroumpa et al. 2018). A large group of *Limonium* species forming a well-supported clade were mostly not circumscribed at the sectional level due to the low internal phylogenetic resolution (Koutroumpa et al. 2018) and the difficulty in identifying diagnostic morphological characters that would be required to characterize sections. These species are currently classified under the non-formal “Mediterranean lineage” until further molecular and morphological data will allow their assignment to sections.

Though sections have been also described for *Armeria*, *Acantholimon* and *Goniolimon* (Boissier 1848; Bunge 1872; Linczevski 1952; Sauvage and Vindt 1952), only a small part of their species have been assigned into them. Furthermore, phylogenetic studies have shown that these sections are non-monophyletic (e.g., Fuertes Aguilar and Nieto Feliner 2003 for *Armeria*, and Moharrek et al. 2017 for *Acantholimon*). Therefore, sectional classifications of these genera were not included in the core checklist but were assigned to the “Unplaced generic subdivisions” category. Finally, aggregates, complexes and species groups that represent informal taxonomic units are not included in the checklist, but are mentioned in notes.

When necessary, all references to official herbaria of the type information follow the acronyms in Thiers (2024+).

### ﻿*Phylogenetic analysis*

We utilised Koutroumpa et al.’s (2018, 2021) ITS dataset (nrDNA) of the largest Plumbaginaceae phylogeny and added recently generated GenBank sequences for the two *Aegialitis* species to test the sister relationship of Aegialitideae. A Bayesian approach was employed using MrBayes v.3.2.7 (Ronquist et al. 2012), following the methodology described in Koutroumpa et al. (2018). The results from the nrDNA dataset were compare to previous phylogenetic inferences that relied only on chloroplast data for the genus (*rbcL*, *trnL-F* and *matK*; Lledó et al. 2001, 2005a; Koutroumpa et al. 2018, 2021). The new phylogenetic insights regarding the position of *Aegialitis* inform the subfamilial division of Plumbaginaceae.

## ﻿Results

### ﻿Taxonomic backbone

The taxonomic backbone, encompassing the taxa in the family and their synonyms as the core checklist, along with the lists of names and taxa not included in the core, is provided in the Suppl. material 1 as a static treatment. This list offers a snapshot reflecting the current state of knowledge. An online version is accessible through the Caryophyllales portal (https://caryophyllales.org). The taxonomic backbone comprises scientific names (both taxonomically accepted names and synonyms), author names standardized according to IPNI (2000+), and standardized nomenclatural citations. The URL of the protologue is provided where available, preferably connecting to the specific page of the protologue (e.g., links available online through the Biodiversity Heritage Library, BHL 2005+), otherwise a link to the entire publication is provided. Additional information, such as distribution area, common names, types, and the source of nomenclatural status, will be available for some taxa in the online portal. Discussion notes provide details on decisions regarding the status of a taxon, classifications into groups or aggregates by certain authors, and additional data like hybrid parents.

The taxonomic backbone is divided into a core part, encompassing all accepted taxa and their synonyms, and lists of names that could not be resolved or are excluded from the core part (“names of verified uncertain application”, “unresolved names”, “unplaced taxa”, “invalid horticultural names and combinations”, “excluded designations”, “excluded names non Plumbaginaceae” and “unplaced generic subdivisions”).

The Plumbaginaceae, as presented here, comprises 3 tribes, 26 accepted genera, 2 subgenera, 17 sections, 2 subsections, 1,179 accepted species, 105 subspecies, 79 varieties, 3 forms and 37 nothotaxa. The core checklist in the taxonomic backbone assigns 2,782 synonyms to accepted names, whereas 30 homotypic synonyms are found in non-core sections. A total of 4,446 scientific name records for Plumbaginaceae are included, incorporating 94 invalid designations and 70 illegitimate names). Table 1 shows the core database statistics including the number of taxa and synonyms assigned to each accepted genus.

Through a review of both old and recent literature, numerous hitherto unresolved names, could be placed. Some names require further revision, notably 45 names mostly from *Statice*. Five *Limonium* names are of verified uncertain application. A separate list contains 46 horticultural designations, including 19 synonyms, identified as *nomina nuda*. Twenty-eight names from the original WFO data set were excluded as they do not belong to the Plumbaginaceae. Most of these names are *Phlox* names from the Polemoniaceae family, relocated to a genus named *Armeria* in that family (with reference to Linnaeus 1737) by Kuntze (1891), with the argument that *Armeria* Willd. was an invalid synonym of *Statice*. The rest belong to other taxa, *Aegialitis* Trin. and *Plumbagoesquirolii* H.Lév., members of the Poaceae and Linaceae families, respectively.

New combinations for three *Limonium* names are implemented in the taxonomic novelties section of this paper, together with the new combinations Limoniumsect.Jovibarba and *Dyerophytumsocotranum*.

### ﻿Phylogenetic position of *Aegialitis* in the ITS tree

**Figure 3.**
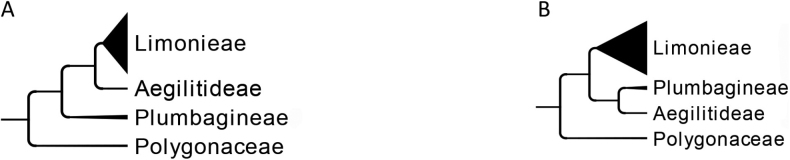
Topological incongruence in the sister relationships of the three tribes in Plumbaginaceae, using Polygonaceae as outgroup **A** plastid cpDNA tree (*rbc*L, *trn*L-F, *mat*K; Lledó et al. 2001, 2005a; Koutroumpa et al. 2018) **B** nrDNA tree, ITS (see Suppl. material 2), and 353 low copy nuclear loci (Baker et al. 2022).

In the ITS Bayesian tree, representatives of *Aegialitis* (Aegialitideae) form a well-supported clade (posterior probability [pp] = 1; Suppl. material 2) sister to the genera of Plumbagineae (Fig. 3; Suppl. material 2). However, the sister relationship between Plumbagineae and Aegialitideae received low support (pp = 0.63; Suppl. material 2). Plumbagineae and Limonieae are reciprocally monophyletic with the highest support (pp = 1; Suppl. material 2).

## ﻿Discussion

### ﻿Overall relationships and the division of Plumbaginaceae into three tribes

Molecular phylogenetic studies have shown that Plumbaginaceae are well supported as monophyletic and sister to Polygonaceae (e.g., Lledó et al. 1998; Cuénoud et al. 2002; Yang et al. 2015; Yao et al. 2019; Baker et al. 2022). Several studies provided insights into the phylogenetic relationships within Plumbaginaceae. The main ones include Lledó et al. (1998, 2001, 2005a), Fuertes Aguilar and Nieto Feliner (2003), Akhani et al. (2013), Moharrek et al. (2017), Malekmohammadi et al. (2017a) and Koutroumpa et al. (2018).

The family was divided into two well-supported clades assigned to subfamilies Plumbaginoideae and Limonioideae (= Staticoideae) (Lledó et al. 1998, 2001, 2005a; Malekmohammadi et al. 2017a; Koutroumpa et al. 2018). Plumbaginoideae comprised the tribe Plumbagineae, whereas Limonioideae was further divided into the tribes Limonieae and Aegialitideae, with the monogeneric Aegialitideae (genus *Aegialitis*) sister to the Limonieae clade with high support according to molecular phylogenies employing the chloroplast markers *rbcL*, *trnL-F* and *matK* (Lledó et al. 2001, 2005a; Koutroumpa et al. 2018, 2021). However, in a recent phylogenomic study for the angiosperm tree of life using the 353 nuclear bait set, *Aegialitis* was recovered sister to Plumbagineae clade, comprising *Plumbago* and *Ceratostigma* Bunge, with highest support (Baker et al. 2022). These results challenge the subfamilial classification of *Aegialitis* contradicting previous molecular studies that used two or three chloroplast markers for the genus (Lledó et al. 2001, 2005a; Koutroumpa et al. 2018, 2021). In order to explore whether there is an incongruence between chloroplast and nuclear data or the position of *Aegialitis* was affected by the very limited taxon sampling in Baker et al.’s (2022) phylogenomic study (only 11 genera of Plumbaginaceae), we inferred an ITS phylogeny adding *Aegialitis* to Koutroumpa et al.’s (2018, 2021) dataset of the largest Plumbaginaceae phylogeny. Our results confirmed Baker et al.’s (2022) topology, placing *Aegialitis* sister to the Plumbagineae genera (Fig. 3; Suppl. material 2), yet with low support, showing a conflict between nuclear and chloroplast genomes regarding the placement of *Aegialitis*. These results further indicate that either incomplete lineage sorting or reticulate evolution may have been implicated in the emergence of this lineage. *Aegialitis* was regarded as the “most primitive” and aberrant genus of Plumbaginaceae (Prakash and Lim 1995). It exhibits several autapomorphies, namely fleshy corolla, basifixed anthers, elongated fruit (capsule) with spongy mesocarp and seed two or three times longer than the calyx (e.g., Kubitzki 1993; Lledó et al. 2001). *Aegialitis* also exhibits intermediate features between the tribes Limonieae and Plumbagineae. Specifically, it has similar vegetative and chemical features to Limonieae (Boissier 1848; Maury 1886; Harborne 1967; Hanson et al. 1994; Lledó et al. 2001), but the same breeding system (‘Plumbago‐type’ pollen and monomorphic stigma) and similar anatomical characters to Plumbagineae (Maury 1886; Weber-El Ghobary 1984; Lledó et al. 2001). Taken together, in the present taxonomic treatment, we accept the classification of Plumbaginaceae into three distinct and monophyletic tribes: Aegialitideae, Limonieae and Plumbagineae. We abstain from dividing the family into the two subfamilies Limonioideae and Plumbaginoideae due to the incongruent placement of *Aegialitis* observed in phylogenetic studies. Our decision is also informed by the need for additional investigations including analyses of complete chloroplast and nuclear genomes, coupled with detailed morphological studies.

### ﻿Intergeneric relationships within Limonieae

Limonieae currently comprises 21 genera, namely *Acantholimon*, *Armeria*, *Bakerolimon* Lincz., *Bamiania* Lincz., *Bukiniczia* Lincz., *Cephalorhizum* Popov & Korovin, *Ceratolimon* M.B.Crespo & Lledó, *Chaetolimon* (Bunge) Lincz., *Dictyolimon* Rech.f., *Ghaznianthus* Lincz., *Goniolimon* Boiss., *Limoniastrum* Fabr., *Limonium*, *Limoniopsis* Lincz., *Myriolimon* Lledó, Erben & M.B.Crespo, *Muellerolimon* Lincz., *Neogontscharovia* Lincz., *Popoviolimon* Lincz., *Psylliostachys* (Jaub. & Spach) Nevski, *Saharanthus* M.B.Crespo & Lledó and *Vassilczenkoa* Lincz. These genera constitute five well-supported subclades in Limonieae: i) *Limonium*, ii) *Ceratolimon-Limoniastrum*, iii) *Armeria-Psylliostachys*, iv) *Bakerolimon-Muellerolimon-Myriolimon-Saharanthus*, and v) *Goniolimon-Acantholimons.l.* with *Acantholimons.l.* comprising the small genera *Bamiania*, *Bukiniczia*, *Chaetolimon*, *Cephalorhizum*, *Dictyolimon*, *Gladiolimon*, *Popoviolimon* and *Vassilczenkoa* (Malekmohammadi et al. 2017a; Moharrek et al. 2017; Koutroumpa et al. 2018). The sister relationships between these subclades remained largely unresolved (Koutroumpa et al. 2018). However, in the recent angiosperm phylogeny by Baker et al. (2022), the authors sampled representatives of eight Limonieae genera belonging to four out of the five aforementioned subclades (except iv) and found *Ceratolimon-Limoniastrum* sister to a clade comprising *Goniolimon-Acantholimons.l.*, *Limonium* and *Psylliostachys-Armeria*, with *Limonium* sister to *Psylliostachys-Armeria* subclade. All these relationships were highly supported, yet further sampling of genera under a phylogenomic approach is essential to draw clear conclusions regarding sister relationships within Limonieae. In addition, three small genera (*Ghaznianthus*, *Limoniopsis* and *Neogontscharovia*) have not been sampled yet in a phylogenetic framework.

Morphological data corroborate some of the inferred sister relationships of the genera within the five subclades. Specifically, *Ceratolimon* and *Limoniastrum* have stamen filaments adnate to the corolla up to the apex of the corolla tube, which is a synapomorphy within Plumbaginaceae (Lledó et al. 2000). *Armeria* and *Psylliostachys* share a unique calyx trait in which the rib‐like tissue fuses at the base of the calyx limb and is absent along the calyx tube (Crespo and Lledó 2000; Lledó et al. 2001). *Muellerolimon* and *Bakerolimon* (Malekmohammadi et al. 2017a; Koutroumpa et al. 2021) share distinctive pollen morphology and shrub habit with articulate, almost leafless stems (Baker 1953), whereas similar stem morphology is present in *Myriolimon* belonging to the same subclade (Lledó et al. 2003; Lledó et al. 2005b). The majority of small genera in Limonieae are found in the *Goniolimon-Acantholimons.l.* subclade and several of them have been previously segregated from these two genera. The monospecific genus *Ikonnikovia* Lincz., previously segregated from *Goniolimon* by Linczevski (1952), was found nested in *Goniolimon* and synonymised by Koutroumpa et al. (2018) on the basis of molecular and morphological data (i.e., styles free from the base, papillose or hairy in the lower part and capitate stigmata distinguish *Goniolimon* including *Ikonnikovia*, from the rest of Plumbaginaceae; Boissier 1848; Siebert and Voss 1896). The well-supported *Acantholimons.l.* clade comprise representatives of *Acantholimons.s.* placed in two subclades with small genera branching in-between them (Moharrek et al. 2017; Koutroumpa et al. 2018). One of the two subclades (clade B sensu Moharrek et al. 2017) is highly supported with the oligospecific genera *Dictyolimon* and *Bukiniczia* forming a monophyletic group sister to *Acantholimons.s.* species. The other subclade (clade A sensu Moharrek et al. 2017) is not highly supported and consists of the oligospecific genera *Vassilczenkoa*, *Chaetolimon*, *Popoviolimon*, *Cephalorhizum* and *Bamiania*, with the latter three forming a monophyletic group sister to *Acantholimons.s.* that includes *Gladiolimon* (Moharrek et al. 2017). The sister relationships between the lineages *Vassilczenkoa-Chaetolimon*, *Popoviolimon-Cephalorhizum-Bamiania* and *Acantholimons.s.* are not well resolved (Moharrek et al. 2017). Considering the phylogenetic results of Moharrek et al. (2017), Beshko et al. (2019) changed the circumscription of *Acantholimon* for Flora of Uzbekistan to include *Chaetolimon*, *Vassilczenkoa* and *Cephalorhizum*, and provided recombinations for their species under *Acantholimon*. Although a wider circumscription for *Acantholimon* including the smaller genera could avoid naming a non-monophyletic assemblage, the absence of morphological diagnostic characters for *Acantholimons.l.*, the unresolved relationships between *Acantholimons.s.* and some of the smaller genera, the non-comprehensive taxon sampling, and the few (two or three) molecular markers used in the phylogenetic studies hinder a formal revision in the circumscription of the genus. Therefore, in this taxonomic backbone, we adopt a more conservative approach by keeping *Bamiania*, *Bukiniczia*, *Chaetolimon*, *Cephalorhizum*, *Dictyolimon*, *Popoviolimon* and *Vassilczenkoa* separate from *Acantholimon* pending further molecular data and a detailed morphological analysis. However, we merge the previously segregated monospecific genus *Gladiolimon* following Rechinger and Schiman-Czeika (1974) back into *Acantholimon* as it is found nested into *Acantholimons.s.*, at a shallow phylogenetic node, in a well-supported clade sister to two species of Acantholimonsect.Acmostegia Bunge, with which it shares the morphological traits that were used for its segregation (Moharrek et al. 2017). Finally, in the *Limonium* clade of Limonieae, *Afrolimon* Lincz. and *Eremolimon* Lincz., two genera previously separated from *Limonium*, were found nested in the genus (Lledó et al. 2005a; Akhani et al. 2013; Malekmohammadi et al. 2017a) and were formally synonymised by Malekmohammadi et al. (2017a), and Akhani et al. (2013), respectively.

### ﻿Infrageneric relationships and genus concepts within Limonieae

The accepted name or synonym status and number of genera in Limonieae varied in different studies. Kubitzki (1993) accepted 22 genera in Limonieae including *Acantholimon*, *Armeria*, *Bakerolimon*, *Bamiania*, *Bukiniczia*, *Chaetolimon*, *Cephalorhizum*, *Dictyolimon*, *Ghaznianthus*, *Gladiolimon*, *Goniolimon*, *Ikonnikovia*, *Limoniastrum*, *Limoniopsis*, *Limonium*, *Muellerolimon*, *Neogontscharovia*, *Popoviolimon*, *Psylliostachys*, *Vassilczenkoa*, as well as *Afrolimon* and *Eremolimon*. The three genera *Ceratolimon*, *Myriolimon* and *Saharanthus* were described after the publication of Kubitzki (1993). *Ceratolimon* and *Saharanthus* were described by Crespo and Lledó (2000) based on phylogenetic results of Lledó et al. (2000). Lledó et al. (2005b) proposed the new name *Myriolimon* to replace their illegitimate *Myriolepis* (Boiss.) Lledó, Erben & M.B.Crespo, a combination that they had published before (Lledó et al. 2003), but that was considered homonymous with the earlier *Myrialepis* Becc. (Arecaceae) by the Committee for Spermatophyta and thus ratified at the XVII International Botanical Congress held in Vienna in July 2005 (Brummitt 2005). Molecular support for separation of *Myriolimon* was argued by Lledó et al. (2005a).

Hernández-Ledesma et al. (2015) accepted 24 genera in this tribe including the genera accepted by Kubitzki (1993) and the three described genera at that time, whereas *Eremolimon* was considered nested in *Limonium*. The 21 accepted genera in the current taxonomic backbone differ from Kubitzki (1993) with *Ikonnikovia* and *Afrolimon* being synonyms of *Goniolimon* (Koutroumpa et al. 2018) and *Limonium* (Malekmohammadi et al. 2017a), respectively. *Gladiolimon* is merged here in *Acantholimon* following Rechinger and Schiman-Czeika (1974) and the latest phylogenetic studies (Moharrek et al. 2017; Koutroumpa et al. 2018). Genera that are well supported as monophyletic are *Armeria* (e.g., Lledó et al. 1998, 2005a; Moharrek et al. 2017; Koutroumpa et al. 2018), *Ceratolimon* (e.g., Lledó et al. 2000, 2005a; Koutroumpa et al. 2018), *Dictyolimon* (Moharrek et al. 2017), *Goniolimon* (e.g., Moharrek et al. 2017; Koutroumpa et al. 2018), *Limoniastrum* (e.g., Lledó et al. 2000, 2005a; Koutroumpa et al. 2018), *Limonium* (e.g., Lledó et al. 2005a; Malekmohammadi et al. 2017a; Koutroumpa et al. 2018), *Myriolimon* (Malekmohammadi et al. 2017a) and *Psylliostachys* (Moharrek et al. 2017; Koutroumpa et al. 2018), *Acantholimon* is non-monophyletic (e.g., Moharrek et al. 2014, 2017; Koutroumpa et al. 2018). Monophyly has not been tested yet for *Bakerolimon*, *Cephalorhizum* and *Chaetolimon* as only one species per genus is sampled in available phylogenetic studies (e.g., Moharrek et al. 2017; Koutroumpa et al. 2018). *Limoniopsis*, *Ghaznianthus* and *Neogontscharovia* have not been sampled yet phylogenetically, and *Bamiania*, *Bukiniczia*, *Muellerolimon*, *Popoviolimon*, *Saharanthus* and *Vassilczenkoa* are monospecific genera. Below we discuss the infrageneric classifications and give some examples of recent studies on species delimitation within the large genera of Limonieae.

#### ﻿*Acantholimon*

It is the second largest genus in Plumbaginaceae and is highly diverse in the Irano-Turanian area. Fifteen sections were recognized by Rechinger and Schiman-Czeika (1974) based on morphological characteristics such as scape length and leaf and flower morphology (Rechinger and Schiman-Czeika 1974; Moharrek et al. 2017) and most of them were not monophyletic in the molecular phylogenetic trees (Moharrek et al. 2017; Koutroumpa et al. 2018). *Acantholimon* species and its related genera form a well-supported clade that is divided into two main subclades without recognized morphological synapomorphic characters (Moharrek et al. 2017; Koutroumpa et al. 2018). The moderately supported subclade A (sensu Moharrek et al. 2017) includes species from two large sections *Acantholimon* and *Armeriopsis* Boiss. as well as species from the small genera *Bamiania*, *Chaetolimon*, *Cephalorhizum*, *Popoviolimon* and *Vassilzenkoa*. The well-supported subclade B (sensu Moharrek et al. 2017) comprises species from *Acantholimon* sections *Acantholimon* and *Armeriopsis*, along with representatives from 12 other sections and *Gladiolimonspeciosissimum* (Aitch. & Hemsl.) Mobayen that is deeply nested in this clade and therefore merged in *Acantholimon* as mentioned above. In subclade B, sister to *Acantholimons.s.* are the small genera *Dictyolimon* and *Bukiniczia*. The monotypic section Bromeliopsis Rech.f. & Schiman-Czeika is missing from the phylogenetic sampling. The phylogenetic trees constructed from sampling of 197 individuals corresponding to a large part of the species of *Acantholimon* by Moharrek et al. (2017) only confirmed monophyly of sections *Platystegia* Rech.f. & Schiman-Czeika and *Pterostegia* Bunge, each with two species that appear in highly supported internal clades within subclade B. All other sections were non-monophyletic or their monophyly could not be tested, since only a single representative per section was sampled.

Regarding species monophyly in *Acantholimon*, 38 out of 121 species in the phylogeny were represented by multiple accessions (Moharrek et al. 2017), so that their monophyly could be tested. Seventeen of them were recovered as monophyletic, 16 were placed in polytomies with representatives of other species, and five species were non-monophyletic (Moharrek et al. 2017). Highly supported non-monophyly was found in only *Acantholimonfestucaceum* (Jaub. & Spach) Boiss. The authors did not present a corresponding matrix of morphological characters to further test species limits and to check for proper identification and application of names. However, their results show that species delimitations within this genus need a much more comprehensive taxon and character sampling to resolve evolutionary relationships at species level. In the absence of comprehensive phylogenetic studies at species level, we followed the morphology-based taxon concepts available through regional floras (e.g., Rechinger and Schiman-Czeika 1974, Flora Iranica; and Bokhari and Edmondson 1982, Flora of Turkey) and monographs (Mobayen 1964; Bunge 1872) as secundum references for the species of *Acantholimon*. The sectional classification within *Acantholimon* is not applied in this taxonomic backbone, as the sections mostly do not represent monophyletic entities. The expanded *Acantholimon* including *Gladiolimon* is consistent as to the variation of morphological characters. All species are pulvinate to densely branched cespitose subshrubs with linear acuminate leaves (Linczevski 1952; Kubitzki 1993). An expanded morphological description of *Acantholimon* to include *Gladiolimon* is given in the nomenclature novelty part of this paper.

#### ﻿*Armeria*

It is a diploid genus (2*n* = 2x = 18) (Nieto Feliner 1990; Tiburtini et al. 2023) with high diversity in the Mediterranean region, especially in the western Mediterranean, and it has been found to be monophyletic in all phylogenetic studies so far (Lledó et al. 1998, 2005a; Fuertes Aguilar and Nieto Feliner 2003; Moharrek et al. 2017; Koutroumpa et al. 2018). The estimated number of species has varied from just a few to about 120 species (Fuertes Aguilar and Nieto Feliner 2003). Bernis (1950) even proposed a single species with many subspecies, varieties and forms in the Iberian Peninsula. There are few comprehensive taxonomic or phylogenetic studies on this genus and most of the studies focused on certain geographic regions, for example the Iberian Peninsula (Bernis 1954; Nieto Feliner 1990). Other investigations addressed assumed species complexes such as *Armeriaarenaria* (Pers.) F.Dietr. and allies (Tauleigne-Gomes and Lefèbvre 2005), *A.maritima* Willd. (Lefèbvre and Vekemans 1995), *A.pubigera* Boiss. (Blanco-Dios 2007), or the *Armeriacanescens* aggregate, examined by Scassellati et al. (2013) with morphometrics. Hybridization, introgression, and reticulate evolution have been frequently considered as the major reason of complex and gradual morphological variation in *Armeria* (Bernis 1954, 1957; Pinto da Silva 1972; Nieto Feliner 1990, 1997; Nieto Feliner et al. 2001; Tauleigne-Gomes and Lefèbvre 2005, 2008; Villa-Machío et al. 2023) that resulted in describing artificial taxa and ecotypes and there are often conflicting views on which taxa to accept (Fuertes Aguilar and Nieto Feliner 2003; Tiburtini et al. 2023). From an evolutionary point of view, *Armeria* stands out as one of the groups of angiosperms with frequent homoploid hybrid speciation (Tauleigne-Gomes and Lefèbvre 2008; Nieto Feliner et al. 2017).

Fuertes Aguilar and Nieto Feliner (2003) generated an ITS data set of 133 accessions from 71 species, covering most of the geographical distribution of the genus *Armeria*. They found nine clades comprising species from mostly specific geographical areas, among others a southern Iberian Sierra Nevada clade, a Sardinia-Corsica clade, and a West-Mediterranean clade including the highest number of species among all these clades. In contrast, the *A.maritima-A.alpina* clade was found to unite plants from the European mountains, temperate to subarctic coastal areas in the northern hemisphere as well as the Mediterranean climate regions of California and Chile. Unlike the other clades that are not linked to hitherto recognized entities, all members of this *A.maritima-A.alpina* clade sensu Fuertes Aguilar and Nieto Feliner (2003) belong to the *A.maritima* and *A.alpina* species complexes (Lawrence 1940; Moore and Yates 1974; Lefèbvre and Vekemans 1995; Tauleigne-Gomes and Lefèbvre 2005, 2008). Based on morphological similarities, *A.alpina* was even considered a synonym of *A.maritima* by different authors (Bernis 1954; Pinto da Silva 1972). Through the consistent presence of additive polymorphic sites in certain taxa, Fuertes Aguilar and Nieto Feliner (2003) concluded that ancient hybridization events as earlier suggested (Nieto Feliner 1997; Fuertes Aguilar et al. 1999a, 1999b) indeed played a major role in the evolution of the genus. However, the ITS trees remained largely unresolved within these nine clades. Two sections have been described in this genus, A.sect.Macrocentron Boiss. with three subsections (*Astegiae* Boiss., *Microstegiae* Boiss. and *Macrostegiae* Boiss.) and A.sect.Plagiobasis Boiss. with two subsections (*Holotricae* Boiss. and *Pleurotrichae* Boiss.) (Boissier 1848), but none of them were monophyletic in the phylogenetic trees (Fuertes Aguilar et al. 1999a, 1999b; Fuertes Aguilar and Nieto Feliner 2003).

Recently, Tiburtini et al. (2023) employed an integrative taxonomic approach on the species of *Armeria* in Sardinia and Corsica and recognized five well-delimited, monophyletic and also geographically distinct endemic species on the basis of molecular phylogenetic trees, chromosome data and morphology. Based on their results, the authors for example suggested merging *A.multiceps* Wallr. and its subspecies into *A.leucocephala* Salzm. ex W.D.J.Koch, and disregarding recognition of the subspecies described in *A.leucocephala* and *A.sardoa* Spreng. This research demonstrates a significant taxonomic knowledge turnover (from 11 taxa formerly described for the islands only five could be upheld with altered circumscription) and underscores the value of detailed analyses of species limits using phylogenetic methods.

In our taxonomic backbone we build upon the published morphological or phylogenetic results and also regional Flora treatments that often offer insights from comprehensive investigations of specimens (e.g., Nieto Feliner 1987, *Armeria* in the Iberian Peninsula, and Pinto da Silva 1972, Flora Europaea; Tiburtini et al. 2023) as secundum references for the species of *Armeria* whereas the sectional classification is not applied here.

#### ﻿*Goniolimon*

This genus has been explored in the context of phylogenetic studies dedicated to other genera (Lledó et al. 2005a; Koutroumpa et al. 2018), confirming its monophyly with *Ikonnikovia* nested within it (Koutroumpa et al. 2018). Despite the extensive geographical distribution of this genus, ranging from North Africa (Algeria) and southeastern Europe to Mongolia and China, few studies on species limits have been undertaken. Recent research addressed *G.tataricum* (L.) Boiss. and allies in southeastern Europe (i.e., in Serbia: Buzurović et al. 2013 and in Croatia: Buzurović et al. 2016), and *G.speciosum* (L.) Boiss. of the Asian steppe (Volkova et al. 2017). In the latter phylogenetic study, monophyly of *G.speciosum* was either not resolved (ITS tree) or not supported (cpDNA tree). Buzurović et al. (2020) reconstructed a phylogenetic tree for seven species of this genus in the Balkans and Apennines using plastid loci and sampling multiple individuals per species. The resulting phylogenetic tree revealed two major unsupported clades with few well-supported subclades. Notably, three of these subclades included individuals from more than one species (sub-clades 2, 4, and 5 sensu Buzurović et al. 2020). Using morphological and phylogenetic data, Buzurović et al. (2020) presented a novel taxonomic classification for three closely related species: *G.italicum* Tammaro, Pignatti & G.Frizzi, *G.tataricum* and *G.dalmaticum* Rchb.f. that had been frequently confused with *G.tataricum* in Croatia. Buzurović et al. (2020) included *G.italicum* within *G.tataricum* and delineated four subspecies within *G.tataricum*. The relationships of *G.besserianum* (Schult.) Kusn. and *G.incanum* (L.) Hepper remained unclear due to lack of morphological data and statistical support in the presented phylogenetic trees.

Considering the non-monophyletic status of three out of the seven species studied by Buzurović et al. (2020) within a relatively small area in comparison to the extensive distribution range of *Goniolimon*, further investigations aiming at defining species boundaries within this genus appear necessary.

Although two sections and two subsections have been described for *Goniolimon* (Linczevski 1952), they only encompass a fraction of the currently recognized species. Also, due to the limited taxon sampling in existing phylogenetic studies, proposed infrageneric division cannot be adequately tested. Therefore, the sectional classification is not applied in this study. Here, we use the morphology-based treatments in Floras (e.g., Linczevski 1952) as well as the protologues from newly described species based on morphological (Buzurović et al. 2016) or molecular and morphological evidence (Buzurović et al. 2020) as secundum references.

#### ﻿*Limonium*

It is the largest and most diverse genus of the family Plumbaginaceae distributed worldwide (Kubitzki 1993) with c. 70% of its species being endemic in the Mediterranean area (Koutroumpa et al. 2018). The monophyly of *Limonium* is confirmed by multiple molecular phylogenetic studies (Lledó et al. 1998, 2005a; Malekmohammadi et al. 2017a; Koutroumpa et al. 2018). *Limonium* contains two well-supported monophyletic subgenera L.subg.Limonium and L.subg.Pterocladus (Spach) H.Arnaud (Lledó et al. 2005a; Malekmohammadi et al. 2017a; Koutroumpa et al. 2018). Twenty-five species are here classified under L.subg.Pterocladus and the rest are assigned to Limoniumsubg.Limonium.

Boissier (1848) provided the first infrageneric treatment for *Limonium* (under *Statice*) recognizing 13 sections and 10 subsections, which were mostly transferred to *Limonium* by Sauvage and Vindt (1952). Since Boissier (1848), some of the subsections were raised to sectional rank (e.g., Staticesect.Limoniumsubsect.Sarcophyllae raised to L.sect.Sarcophylla by Linczevski 1952), several new sections were described (e.g., L.sect.LimoniodendronSventenius (1960), L.sect.Nephrophyllum Rech.f. by Rechinger and Schiman-Czeika 1974), whereas some of Boissier’s sections were segregated from *Limonium* as independent genera (namely *Dictyolimon* by Rechinger and Schiman-Czeika (1974), *Psylliostachys* by Nevski (1937) and *Myriolimon* by Lledó et al. (2003, 2005b)). Sectional classification has been updated recently following molecular phylogenetic studies (Malekmohammadi et al. 2017a; Koutroumpa et al. 2018). The new Limoniumsect.Iranolimon M.Malekm., Akhani & Borsch was described by Malekmohammadi et al. (2017a) to accommodate species of an Irano-Turanian subclade previously classified under L.sect.Sarcophylla (Boiss.) Lincz. The latter section was originally described based on the woody habit of its species, which turned out to have convergently evolved in two unrelated lineages (Malekmohammadi et al. 2017a). Also, the new Limoniumsect.Circinaria (Boiss.) M.Malekm. was validated to include species previously assigned to *Afrolimon* that were found nested within *Limonium* (Lledó et al. 2005a; Malekmohammadi et al. 2017a). Koutroumpa et al. (2018) described the new monospecific section L.sect.Tenuiramosa Koutr. (*L.anthericoides* (Schltr.) R.A.Dyer) which is sister to L.sect.Pterocladus (Spach) Bokhari and both constitute L.subg.Pterocladus. Furthermore, Koutroumpa et al. (2018) amended L.sect.Limonium, L.sect.Nephrophyllum and L.sect.Sarcophylla, and published new combinations for L.sect.Pruinosa (Batt.) Koutr. and L.sect.Pterocladussubsect.Nobilia (Boiss.) Koutr.

The extensive sampling of Mediterranean endemics of *Limonium* in Koutroumpa et al.’s (2018) study of Plumbaginaceae revealed that they all belong to a large, well-supported internal clade, namely the “Mediterranean lineage”. Nevertheless, species relationships within the lineage remained largely unresolved. Only few species of the “Mediterranean lineage” were assigned to four morphologically well-defined sections (i.e., L.sect.Polyathrion (Boiss.) Sauvage & Vindt, L.sect.Pruinosa, L.sect.Siphonantha (Boiss.) Sauvage & Vindt and L.sect.Schizhymenium (Boiss.) Sauvage & Vindt), two of which were represented by multiple species in the phylogeny and recovered as monophyletic (Koutroumpa et al. 2018). A sectional classification for the remaining species within this lineage at the moment is difficult. Species of this lineage have diversified very recently (mostly during the Pleistocene; Lledó et al. 2005a; Koutroumpa et al. 2021) and to resolve their phylogenetic relationships, many molecular characters will be required. In addition, combined effects of polyploidy, apomixis and hybridization may have blurred species limits and make the identification of diagnostic morphological characters a difficult task. Therefore, all these species are provisionally assigned to the phylogenetically well-defined “Mediterranean lineage”. Apart from causing taxonomic complexity, polyploidy, apomixis and hybridization have been considered as the main factors for promoting speciation of *Limonium* in the Mediterranean region (Ingrouille 1984; Kubitzki 1993; Palacios et al. 2000; Lledó et al. 2005a). Indeed, Koutroumpa et al. (2021) found a significant shift in diversification rates for the “Mediterranean lineage” and showed that the turbulent geological history and climatic oscillations in the Mediterranean, combined with the significant role of apomixis triggered species radiation in *Limonium*.

**Figure 4.**
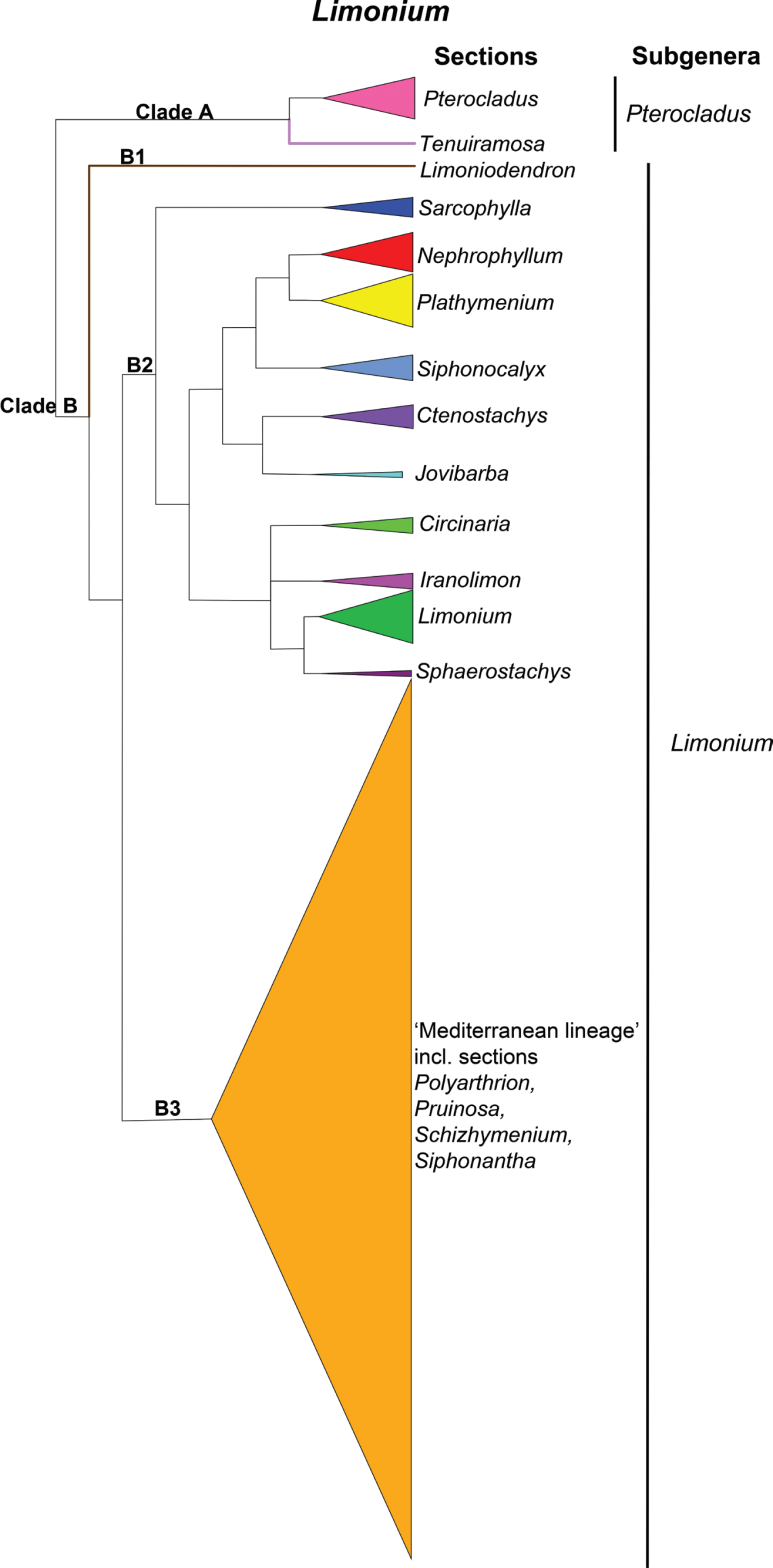
Phylogenetic relationships of the major clades in *Limonium* and the corresponding infrageneric units, following Koutroumpa et al. (2018). The size of triangles is proportional to the number of species assigned to the different sections and the “Mediterranean lineage”.

We follow the mentioned recent advances in the infrageneric classification of *Limonium* and assign taxa to major clades corresponding to the two subgenera, and further classify them to one of the 17 accepted sections and two subsections or the “Mediterranean lineage”. We achieved this by combining information from the latest phylogenetic analyses (Malekmohammadi et al. 2017a; Koutroumpa et al. 2018) with other data (e.g., morphology, chromosome counts, geographic distributions) obtained from an extensive literature search for *Limonium* taxa that were not yet sampled in a phylogenetic framework (see for example table S3 in Koutroumpa et al. 2018). As a result, we can summarize Limoniumsubg.Pterocladus to comprise L.sect.Tenuiramosa (one species) and L.sect.Pterocladus (24 species) that is further divided into L.subsect.Nobilia (18 species) and L.subsect.Odontolepidea (six species) (Fig. 4). Limoniumsubg.Limonium is divided into three distinct well-supported clades (B1, B2 and B3 sensu Koutroumpa et al. 2018; Fig. 4), with L.sect.Limoniodendron (one species; clade B1) being sister to a clade comprising mostly non-Mediterranean taxa (clade B2) and the “Mediterranean lineage” (clade B3). Clade B2 includes ten morphologically and phylogenetically well-defined sections (Fig. 4), namely L.sect.Circinaria (eight species), L.sect.Ctenostachys (Boiss.) Sauvage & Vindt (11 species), L.sect.Iranolimon (nine species), L.sect.Jovibarba (Boiss.) M.Malekm. & Koutr. (three species), L.sect.Limonium (25 species), L.sect.Nephrophyllum (16 species), L.sect.Plathymenium (Boiss.) Lincz. (28 species), L.sect.Sarcophylla (12 species), L.sect.Siphonocalyx Lincz. (12 species) and L.sect.Sphaerostachys (Boiss.) Bokhari (four species). The “Mediterranean lineage” (clade B3) comprise four small sections (Fig. 4) L.sect.Polyarthrion (four species), L.sect.Pruinosa (six species), L.sect.Schizhymenium (two species) and L.sect.Siphonantha (four species), whereas 479 species are assigned to this lineage but not classified further into sections or subsections due to the reasons explained above.

Only a few studies explore species limits and relationships in *Limonium* at shallow phylogenetic levels. A recent example is the phylogenomic investigation by Pina-Martins et al. (2023) on seven representatives of Limoniumsect.Limonium. The authors sampled multiple individuals per species and analyzed more than 10,000 SNPs obtained from genotyping by sequencing (GBS). The large amount of molecular data employed in the study could resolve species relationships that were previously mostly unresolved when only few molecular markers were used (see e.g., Malekmohammadi et al. 2017a, 2017b; Koutroumpa et al. 2018). Pina-Martins et al.’s (2023) phylogeny recovered *L.brasiliense* (Boiss.) Kuntze, *L.californicum* (Boiss.) A.Heller, *L.carolinianum* (Walter) Britton, *L.humile* Mill. and *L.narbonense* Mill. as monophyletic with high support. The widespread *L.vulgare* Mill. formed a large, well-supported clade with *L.maritimum* Caperta, Cortinhas, A.P.Paes, Guara, Esp.Santo & Erben nested in it. *Limoniummaritimum*, represented by a single population although it is widely distributed along the Portuguese coast (Cortinhas et al. 2015), differed from *L.vulgare* only by 34 out of 10,000 SNPs. Moreover, the populations of *L.vulgare* across its distributional range showed high genetic structure based on the phylogenetic and clustering analyses (Pina-Martins et al. 2023). *Limoniummaritimum* was described as separate species based on morphometric data, in which few diagnostic traits had slightly smaller yet largely overlapping size ranges compared to the closely related *L.vulgare* (Cortinhas et al. 2015). Taken together, the nested phylogenetic position of *L.maritimum* within *L.vulgare* and the low genetic and morphometric differentiation of *L.maritimum* compared to *L.vulgare*, question the recognition of the former as a separate species. Therefore, we consider *L.maritimum* as a synonym of *L.vulgare*.

Iamonico et al. (2022) examined species boundaries in four endemic species along the Tyrrhenian coast and Ponziane Archipelago (central Italy) combining molecular and morphometric data. They analyzed ITS sequences and found the same ribotype in all populations of the four species, except for two populations displaying individuals with dual ribotypes. This suggests a possible hybrid origin, though not addressed in the study. Morphometric analyses revealed that individuals from the two populations with dual ITS ribotypes were differentiated in morphospace, and at species level, *L.pandatariae* Pignatti was distinguished from *L.circaei* Pignatti, while *L.amynclaeum* Pignatti and *L.pontium* Pignatti occupied a central position relative to the other species, with considerable overlap among individuals. The authors proposed merging all species under *L.pontium* and recognizing the populations with mixed ITS genotypes as two different subspecies. However, considering the potential hybrid nature of these populations and the limitations of using a single molecular maker for species resolution within the “Mediterranean lineage” (see e.g., Koutroumpa et al. 2018), we suggest maintaining the four previously recognized species separate until further molecular studies provide clarity. Additionally, we propose synonymizing the newly described L.pontiumsubsp.terracinense Iberite, Iamonico, De Castro & Nicolella under *L.amynclaeum*.

We used phylogenetic studies for the circumscription of taxa in *Limonium*, where available (e.g., Akhani et al. 2013; Pina-Martins et al. 2023). As many species are of restricted range, in the absence of such studies it was considered adequate to use morphological circumscriptions from regional floras (e.g., Erben 1993, 2012), regional monographs (e.g., Brullo and Erben 2016, *Limonium* in Greece; Doğan et al. 2020, *Limonium* in Turkey), taxonomic revisions of specific species groups (e.g., Bogdanović and Brullo 2015, *Limoniumcancellatum* group), and protologues (Pignatti 1955, 1982) as secundum references for the taxa of *Limonium*.

### ﻿Intergeneric relationships within Plumbagineae

The tribe Plumbagineae comprises *Ceratostigma*, *Dyerophytum* Kuntze, *Plumbagella* Spach and *Plumbago*. *Ceratostigma* is sister to the rest of the genera (Koutroumpa et al. 2018). *Plumbago* is polyphyletic (Lledó et al. 2001, 2005a; Koutroumpa et al. 2018) with *Plumbagoeuropaea* L., the type species of the genus, sister to the monotypic *Plumbagella* and the tropical/subtropical species of *Plumbago* sister to *Dyerophytum*. All phylogenetic relationships within Plumbagineae are highly supported (Koutroumpa et al. 2018). *Plumbago* and *Plumbagella* are characterised by glandular calyces, a distinct diagnostic feature for the family (Kubitzki 1993). Although *Plumbago* forms a non-monophyletic assemblage, a formal revision of its generic circumscription would require a comprehensive taxon sampling in a phylogenetic framework (currently four out of 19 *Plumbago* species sampled in Koutroumpa et al. 2018) and a revision of the diagnostic characters of the well-supported phylogenetic groups and corresponding genera. Therefore, no changes in generic circumscriptions are advisable for the time being.

### ﻿Infrageneric relationships and genus concepts within Aegialitideae and Plumbagineae

The Aegialitideae comprise *Aegialitis* which is recovered as monophyletic with high support in our phylogenetic analysis in which we included sequences for its two species *A.annulata* (two accessions) and *A.rotundifolia* (four accessions) that are also recovered as reciprocally monophyletic with high support (Suppl. material 2). In Plumbagineae, highly supported monophyly is inferred for *Ceratostigma* (Koutroumpa et al. 2018; Zhao et al. 2023) and *Dyerophytum* (Koutroumpa et al. 2018). *Plumbago* is non-monophyletic (Lledó et al. 2001, 2005a; Koutroumpa et al. 2018) and *Plumbagella* is a monospecific genus, therefore, monophyly at the genus level cannot be tested.

To our knowledge, very few phylogenetic studies have explored species limits within the genera of Plumbagineae. Zhao et al. (2023) sampled whole plastid genomes of multiple individuals from five *Ceratostigma* species in China in a phylogenomic framework and tested for species limits. The inferred phylogeny resolved the interspecific relationships within *Ceratostigma* with high support in almost all clades and species’ monophyly was confirmed for four out of the five species, namely *C.griffithii* C.B.Clarke, *C.plumbaginoides* Bunge, *C.ulicinum* Prain and *C.willmottianum* Stapf. *Ceratostigmaminus* Stapf ex Prain was not monophyletic with its individuals found in two distinct clades corresponding to their geographical ranges. Individuals from Hengduan Mountains were sister to *C.plumbaginoides* and *C.willmottianum* and individuals from the Qinghai-Tibet Plateau were sister to *C.griffithii*. The authors attributed the non-monophyletic clustering of *C.minus* to genetic divergence promoted by geographical barriers in the two mountainous regions but they did not exclude a potential impact of hybridization and introgression to the observed topology. They also highlighted the necessity of comparing chloroplast genome phylogenies with those from nuclear genomes to further understand the evolutionary relationships between and within species.

Graham (2014) investigated the systematics of *Dyerophytum* using morphometric and molecular data (ITS and two cpDNA regions). She found *D.africanum* (Lam.) Kuntze highly supported as monophyletic and sister to a clade comprising *D.indicum* (Gibbs ex Wight) Kuntze, which was monophyletic in the ITS tree, *D.pendulum* (Balf.f.) Kuntze, and *D.socotranum*. The latter taxon was originally described as a variety of *D.indicum* (under the former generic name *Vogelia* Lam., i.e., Vogeliaindicavar.socotrana Balf.f.), but it was later unofficially raised to species level by J.R. Edmondson. This taxon was monophyletic in the ITS tree but formed a polytomy with *D.pendulum* in the cpDNA tree. In the morphometric analysis, individuals of all four *Dyerophytum* taxa were largely distinct. Consequently, we uphold all four taxa at the species level in this study and formally propose the new combination and status for *Dyerophytumsocotranum* (see Taxonomic and nomenclatural novelties).

For Plumbagineae and Aegialitideae, we follow floras and e-floras (e.g., Peng and Kamelin 1996, Flora of China) in addition to the recent phylogenetic studies (e.g., Zhao et al. 2023) as secundum references.

### ﻿Taxonomic history and nomenclature of *Statice* L. nom. rej.

Our current understanding of phylogenetic relationships and generic concept in the tribe Limonieae show that *Armeria* and *Limonium* are two well differentiated entities (e.g., Koutroumpa et al. 2018). However, the classification of these two genera is still historically linked via the name *Statice* and the names have been in part intermingled. Since the whole Plumbaginaceae were approached in this study, we also revisited the “Statice problem” and a few names could be resolved, resulting in three new combinations (see taxonomic and nomenclatural novelties below).

Tournefort (1694) considered *Limonium* and *Statice* as two distinct genera of sea-lavenders and thrifts, respectively. Linnaeus 1737, in his Genera Plantarum) and Species Plantarum (1753), combined them in the single genus *Statice* L. and regarded *Limonium* Tourn. as its synonym. Miller (1754, 1768) used the names *Limonium* Tourn. for sea-lavenders and *Statice* Tourn. for thrifts, based on the pre-Linnaean treatments. Willdenow (1809) separated the thrifts in the genus *Armeria* Willd. and called the sea-lavenders *Statice* L. (p.p.). This caused confusion in the circumscription of the three genera *Armeria*, *Limonium*, and *Statice* because following Willdenow’s publication, the names *Armeria* and *Statice* were both applied to the thrifts by different authors (Lawrence 1940) that led to the rejection of the name *Statice* as a *nomen ambiguum*. The name *Armeria* was conserved for the thrifts and *Limonium* for sea-lavenders in the International Botanical Congress of Cambridge 1930, following the report of the Editorial Committee for Nomenclature (see Bricquet 1935).

Rejection of *Statice* in favour of *Armeria* and *Limonium* caused part of the taxonomic and nomenclatural complexities in Plumbaginaceae. Only 18 species of *Statice* were described by Linnaeus (1753, 1762, 1767), 11 of which refer to species of *Limonium* and seven species are now synonyms of accepted species in *Acantholimon*, *Armeria*, *Goniolimon*, *Limoniastrum*, *Myriolimon* and *Psylliostachys*. Many new species were described under *Statice* before its rejection that are currently mostly synonyms of *Limonium* or *Armeria* but *Statice* names are spread over the Plumbaginaceae and can be found in synonymy status under 11 out of 26 genera of the family. This resulted in a multitude of required taxonomic recombination that is continuing even in this study. Kuntze (1891) recombined most of the described *Statice* species to their correct generic name. Despite several other works (Greuter and Raus 1987, 1989; Erben 2012), there are still 31 *Statice* names that have not yet been recombined or their status remains unresolved (classified under “unresolved names” in the database).

The comprehensive treatment in this study, attempting to completely cover *Statice* names, to clarify nomenclatural status and to highlight the still unresolved taxonomic and/or nomenclatural questions will facilitate future investigations.

### ﻿Classification of closely related and often apomictic species

Besides the accepted taxonomic subdivisions above species rank (e.g., subgenus, section, subsection), several informal terms are used by different authors to categorize species or infraspecies of similar morphology (Tutin et al. 1972; Greuter et al. 1984). These informal classifications mostly reflect the taxonomic complexity of species groups in which biological processes, such as apomixis, polyploidy and hybridization, play an important role in their speciation as demonstrated for example in *Limonium* (Koutroumpa et al. 2021).

Greuter et al. (1984) defined the term “aggregate” as an informal grouping for easily confused and morphologically allied and (probably) closely related species, the so-called “segregate species”. The following rule is given for naming aggregates: “an aggregate is designated by the oldest name, in terms of nomenclatural priority, of an included species, but without author citation”, yet this is not a formal rule and is not included in the International Code of Nomenclature for algae, fungi, and plants (Turland et al. 2018). In the Flora Europaea (Tutin et al. 1972), the term “group” was used for the same purpose, i.e., to group similar species that are difficult to distinguish. These groups, and not the individual species, were keyed out in the main identification key. A key to the component species within each group was provided along with descriptions for further study and identification. Furthermore, the term “complex” has also been used in literature, especially for *Limonium* and *Armeria* (e.g., Pignatti 1982; Baumbach and Hellwig 2007; Róis et al. 2018), to refer to morphologically related taxa, but its application sometimes lacks a global context.

Aggregates, complexes, and groups have been mostly used for *Limonium* in Plumbaginaceae due to the highly variable nature of its species, with several of them being polyploid and apomictic. These informal subdivisions contain many sexual and apomictic microspecies with narrow geographical distributions. For example, the *Limoniumbinervosum* aggregate is a complex assemblage of nine species and over 40 infraspecies in the British Isles (Ingrouille and Stace 1986) that are mostly raised to species level by Sell (2018) (e.g., *L.anglicum* (Ingr.) P.D.Sell), whereas Pignatti (1972) considered it as a group with only five species. *Limoniumbinervosum* (G.E.Sm.) C.E.Salmon is a widespread, apomictic, and variable species (Domina 2011+) but, has not been studied throughout its entire distribution area (Ingrouille and Stace 1986). Further examples of informal subdivisions in *Limonium* include the aggregates adopted by Greuter et al. (1989) to classify several Mediterranean species, the complexes recognized by Brullo and Guarino (2017) to classify species occurring in Italy and the groups adopted by Brullo and Erben (2016) for the Greek taxa.

All aggregates, complexes, and groups in *Limonium* are essentially regional classifications that refer to morphologically similar species and infraspecies in a restricted geographical area without considering the wider distribution of the group. It is therefore unclear which species should belong to a group, complex or aggregate when the range is expanded. In addition, the monophyly of these informal subdivisions in a phylogenetic framework has not yet been explored. Therefore, in our global Plumbaginaceae backbone we refrained from using aggregates, complexes or groups as ranks since they lack nomenclatural and phylogenetic status. A note is given in the database for each taxon that is part of a literature-based aggregate or group. These notes highlight the existing alpha-taxonomic confusion associated with the respective taxa.

### ﻿Comparison of different online sources

This database encompasses a total of 4,301 scientific names in the family Plumbaginaceae, surpassing the count in other online databases: Tropicos (1991+) assigns 2,067 names to Plumbaginaceae, IPNI (2000+) lists 3,200 names, the World Checklist of Vascular Plants (Govaerts 2023) 3,727 names, and the Catalogue of Life Checklist (COL 2024) contains 3,769 names. The Global Biodiversity Information Facility’s (GBIF) backbone largely follows COL (using a 2023 version).

With respect to the taxonomy, the Catalogue of Life (COL 2024), sourced from Hassler (2023) includes two subfamilies, 23 genera, 1,149 species, 100 subspecies, and 30 varieties in the accepted names and 166 names are considered “ambiguous synonyms”, of these 158 are unambiguously placed as synonyms in our treatment and four as misapplied names.

### ﻿Future updates and interaction with the World Flora Online

The database will be updated continuously according to newly published results of taxonomic and phylogenetic studies and published online in the Caryophyllales portal (https://caryophyllales.org). Future versions that significantly differ from this one will be published as further stable and citable versions. Adding further information such as distribution, common names, protologue link, type species, morphological description, species keys, molecular data, photographs or link to the photographs, cytological data, conservation status, etc. is a future goal for the Plumbaginaceae database, with the initial priority set to nomenclatural types, protologue links and geographical distribution.

Following publication, this information will be stored in ChecklistBank (Döring et al. 2022) (https://www.checklistbank.org/) and shared with the WFO Plant List, contributing to the development of the WFO Backbone. Regular updates will be consistently incorporated into WFO to keep the information current.

## ﻿Taxonomic and nomenclatural novelties

### ﻿ Dyerophytum socotranum

Taxon classificationPlantaeCaryophyllalesPlumbaginaceae

(Balf.f.) J.R.Edm., M.Malekm. & Koutr., comb. et stat. nov.

CB58565B-B7A0-55C2-9C38-0BAAFAAD129E

urn:lsid:ipni.org:names:77343843-1

≡ Vogeliaindicavar.socotrana Balf.f. in Proc. Roy. Soc. Edinburgh 12(113): 406. 1884, basionym. Lectotype (designated here by J.R. Edmondson): Yemen, Socotra, *Balfour 416* (E00068915); isolectotypes: E00068913, E00068914.

### ﻿ Limonium thymoides

Taxon classificationPlantaeCaryophyllalesPlumbaginaceae

(Girard) M.B.Crespo comb. nov.

A7B0CDE8-8A09-5BE4-9BED-278B4FF107BC

urn:lsid:ipni.org:names:77343844-1

≡ Statice thymoides Girard in Mém. Sect. Sci. Acad. Sci. Montpellier 1: 189. 1848, basionym. Lectotype (designated here by M.B. Crespo): Algéria. Alger [Algeria, Algiers], Durieu (MPU021644).
= Statice asparagoides Coss. & Durieu ex Batt., Fl. Algérie Dicot.: 727. 1890, **syn. nov.**
≡ Limonium asparagoides (Coss. & Durieu ex Batt.) Maire in Bull. Soc. His. Nat. Afrique N. 22: 55. 1931, **syn. nov.** Lectotype (designated here by M.B. Crespo): Algeria. Rochers maritimes à Nemours, ouest de Prov. d’Oran, June 1856, [Plantes d’Alger n° 131], *E. Bourgeau* (MPU 007820); isolectotypes: FI 000898, MPU 007818, MPU 007819.

### ﻿ Limonium × fraternum

Taxon classificationPlantaeCaryophyllalesPlumbaginaceae

(Sennen & Pau) M.B.Crespo comb. nov.

561F8A6F-DE22-5122-BC04-CCD8DAC84BAF

urn:lsid:ipni.org:names:77343845-1

≡ Statice × fraterna Sennen & Pau in Bull. Acad. Int. Geogr. Bot. 23: 47. 1913, pro sp., basionym. Lectotype (designated here by M.B. Crespo): Spain. Catalogne, [Lérida], Llano de Urgel au Prado de Monsoa [sic], 1 September 1911, Pl. Espagne n° 1222, Sennen (BC 54018); isolectotypes: ABH 42341, BC 54017; DAO 00455905, M, MA, FR, G, JE, RNG, etc.

#### Notes.

This name applies to the hybrid *L.hibericum* × *L.viciosoi*, sec. Erben (1993).

### ﻿ Limonium × rossmaessleri

Taxon classificationPlantaeCaryophyllalesPlumbaginaceae

(Willk.) M.B.Crespo stat. nov.

5EF0B8B1-FE34-56C8-B84F-4874B52C4380

urn:lsid:ipni.org:names:77343846-1

≡ Statice insignis var. rossmaessleri Willk. in Linnaea 30: 123. 1859, basionym.
≡ Limonium insigne var. rossmaessleri (Willk.) Pignatti in Collect. Bot. (Barcelona) 6: 295. 1962. Lectotype (designated here by M.B. Crespo): Spain. “H. M. Willkommii herbar. hispan. Statice Rossmaessleri n. sp.” [Regno Murcico apud Willkomm], Legit. *Rossmaessler* [Anno] 1853 (COI 00043402).

#### Notes.

This name applies to the hybrid *L.insigne* × *L.caesium*, sec. Erben (1993).

### ﻿ Limonium sect. Jovibarba

Taxon classificationPlantaeCaryophyllalesPlumbaginaceae

(Boiss.) M.Malekm. & Koutr. comb. nov.

E1775449-900B-58EE-8CE8-4B79933BA34E

urn:lsid:ipni.org:names:77343907-1

≡ Statice sect. Jovibarba Boiss. in Candolle, Prodr. 12: 665. 1848, basionym. Type: Limoniumjovibarba (Webb ex Boiss.) Kuntze, Revis. Gen. Pl. 2: 395. 1891.

### ﻿ Chaetolimon

Taxon classificationPlantaeCaryophyllalesPlumbaginaceae

(Bunge) Lincz. in Trudy Tadzikisk. Bazy 8: 586. 1940.

2D821C4B-24A7-5C0E-91AF-9B1E34661AF9

#### Type

**(designated here by M.Malekmohammadi).***Chaetolimonlimbatum* Lincz. in Trudy Tadzikisk. Bazy 8: 595. 1940.

### ﻿ Acantholimon

Taxon classificationPlantaeCaryophyllalesPlumbaginaceae

Boiss., Diagn. Pl. Orient. ser. 1, 7: 69. 1846 nom. cons.

14A17421-3E92-5304-B4F4-97A1F35D60B1

= Gladiolimon Mobayen, Rev. Taxon. Acanthol.: 296. 1964. Type: Gladiolimonspeciosissimum (Aitch. & Hemsl.) Mobayen, Rev. Taxon. Acanthol.: 297. 1964

#### Type.

*Acantholimonglumaceum* (Jaub. & Spach) Boiss., Diagn. Pl. Orient. ser. 1, 7: 75. 1846.

#### Emended diagnosis

**of *Acantholimon* (including *Gladiolimon*).** Laxly or densely branched, often with chalk protuberances, hemispherical or subspherical pulvinate subshrubs, usually forming thorny pincushions. Leaves, alternate, frequently spiny, linear-triangular, subcylindrical or linear, rarely flat and fairly broad, acuminate at apex. Inflorescence simple or branched spike, elongate or short, compact and capitate, paniculate, or subsessile. Spikes with one to numerous flowered spikelets, forming simple or compound panicles. Calyx broadly to narrowly infundibular or tubular, scarious, glabrous or hairy. Corolla longer than the calyx, petals slightly connate at base, white, pink, purple or red. Filaments of stamens distinct except at base, glabrous. Styles distinct from base, glabrous or rarely verrucose. Stigma capitate or oblong-capitate. Ovary narrowly linear-cylindrical or sub-ovoid. Fruit oblong-linear, not enlarged at the top, opening with a small round lid and with valves.

## Supplementary Material

XML Treatment for Dyerophytum socotranum

XML Treatment for Limonium thymoides

XML Treatment for Limonium × fraternum

XML Treatment for Limonium × rossmaessleri

XML Treatment for Limonium sect. Jovibarba

XML Treatment for Chaetolimon

XML Treatment for Acantholimon

## ﻿Additional information

### Author ORCIDs

Maryam Malekmohammadi https://orcid.org/0000-0003-4641-7795

Konstantina Koutroumpa https://orcid.org/0000-0001-9391-874X

Manuel B. Crespo https://orcid.org/0000-0002-3294-5637

Gianniantonio Domina https://orcid.org/0000-0003-4184-398X

Nadja Korotkova https://orcid.org/0000-0003-0106-3209

Hossein Akhani https://orcid.org/0009-0003-8484-973X

Sabine von Mering https://orcid.org/0000-0003-2982-7792

Thomas Borsch https://orcid.org/0000-0002-5724-2786

Walter Berendsohn https://orcid.org/0000-0001-9945-7606

### Data availability

All of the data that support the findings of this study are available in the main text or Supplementary Information.

## Supplementary materials

Supplementary material 1 Plumbaginaceae taxonomic backbone Data type pdf https://binary.pensoft.net/file/1075740This dataset is made available under the Open Database License (http://opendatacommons.org/licenses/odbl/1.0/). The Open Database License (ODbL) is a license agreement intended to allow users to freely share, modify, and use this Dataset while maintaining this same freedom for others, provided that the original source and author(s) are credited.Maryam Malekmohammadi, Konstantina Koutroumpa, Manuel B. Crespo, Gianniantonio Domina, Nadja Korotkova, Hossein Akhani, Sabine von Mering, Thomas Borsch, Walter G. Berendsohn

Supplementary material 2 Phylogenetic relationship of Plumbaginaceae using Polygonaceae outgroups Data type pdf Explanation note Phylogenetic relationship of Plumbaginaceae using Polygonaceae outgroups: Bayesian analysis of ITS sequences from Koutroumpa et al. (2018, 2021) with additional Aegialitis sequences from GenBank (highlighted in pink). Tree nodes display posterior probabilities. https://binary.pensoft.net/file/1075741This dataset is made available under the Open Database License (http://opendatacommons.org/licenses/odbl/1.0/). The Open Database License (ODbL) is a license agreement intended to allow users to freely share, modify, and use this Dataset while maintaining this same freedom for others, provided that the original source and author(s) are credited.Maryam Malekmohammadi, Konstantina Koutroumpa, Manuel B. Crespo, Gianniantonio Domina, Nadja Korotkova, Hossein Akhani, Sabine von Mering, Thomas Borsch, Walter G. Berendsohn
