# Supplementary material for: ﻿A taxonomic backbone for the Plumbaginaceae (Caryophyllales)
Source: PhytoKeys. 2024 Jun 20;243:67–103. doi: 10.3897/phytokeys.243.122784 (PMC11211657; doi:10.3897/phytokeys.243.122784)
Supplement: Supplementary material 1 — Plumbaginaceae taxonomic backbone [file phytokeys-243-067_article-122784__-s001.pdf]

Supplement 1: A taxonomic backbone for the Plumbaginaceae – Checklist

Introduction

This checklist attempts to cover all names that belong to the family, were effectively published, and cited in a taxonomic context. It includes a core part with accepted names and their synonyms. Names excluded from the core checklist were assigned to the following categories: "Unplaced taxa" currently contains only 2 invalidly published hybrid designations that were described by Pignatti and used in later publications. "Unplaced generic subdivisions" contains names of sections and subsections that we refrained from classifying awaiting further evidence from phylogenetic studies. "Names of verified uncertain application" lists names that probably will never be placed. The category "Invalid horticultural names and combinations" contains just that; "Excluded designations" likewise lists designations that have been in use but which we did not want to include in the synonymy (e.g. erroneous author citations). "Excluded names" contains names outside the Plumbaginaceae that were erroneously part of the original WFO backbone. In contrast, the "unresolved names" offers a provisional category for practical reasons to accommodate names for which the correct application or status still needs to be found out. To classify the unresolved names in the correct place, further literature and/or herbarium revisions are required but this investigation exceeds the scope of this study. The circumscription of taxa is always indicated by a secundum "sec." reference (Berendsohn 1995), a reference that indicates the circumscription of a taxon and its distinction from other taxa. The sec. references are either literature references, or original work done here, and then are referred as "Malekmohammadi et al. 2024". The "syn. sec." reference of the synonyms refers to a reference stating the synonymy to the accepted name or to one of its synonyms. The sec. reference of the names that are excluded from the core taxonomic backbone is normally the source of the name, i.e., the dataset from where the name has been imported.
An index to scientific names is provided following p. 102. All names are listed again with their World Flora Online name identifier and the verified URI of the original publication, where possible, following page 141. References cited as secundum references or in notes are listed on page 252.

The Core Checklist

***Plumbaginaceae* Juss., Gen. Pl.: 92. 1789, nom. cons.** Sec. APG IV [Angiosperm Phylogeny Group] (2016)

Type: *Plumbago* Tourn. ex L.
= *Armeriaceae* Horan., Prim. Lin. Syst. Nat.: 68. 1834 syn. sec. Malekmohammadi & al. (2024)
= *Limoniaceae* Ser., Fl. Pharm.: 456. 1851 syn. sec. Lledó & al. (2001)
= *Aegialitidaceae* Lincz. in Novosti Sist. Vyssh. Rast.: 173. 1968 syn. sec. Lledó & al. (2001)
= *Limoniaceae* Lincz. in Novosti Sist. Vyssh. Rast.: 174. 1968 syn. sec. Lledó & al. (2001)

Notes. – A cosmopolitan family of primarily perennial herbs, cushion form subshrubs and small shrubs, rarely climbers, mainly distributed in the temperate zones of the Northern Hemisphere, especially in the Mediterranean and Irano-Turanian regions but also in southern and eastern Africa, southern South America and Western Australia. Numbers given in the literature vary from 18 to 30 genera and about 650-1000 species (Kubitzki, 1993). In this checklist we accept 26 genera and 1,179 species. Plumbaginaceae predominantly occur in arid and saline environments, often in coastal habitats as perennial herbs and in the high mountains as cushion form plants. The family is characterized by flowers that have stamens opposite the petals (antipetalous) and a single basal anatropous ovule with curled funicle (Kubitzki, 1993).
Molecular studies based on different markers have shown that Plumbaginaceae are well supported as a monophyletic family sister to Polygonaceae within Caryophyllales (e.g., Lledó et al. 1998; Cuénoud & al. 2002; Hilu & al. 2003). Several studies provided data about the phylogenetic relationship within Plumbaginaceae. The main ones include Lledó et al. (1998, 2001, 2005), Aguilar & Feliner (2003), Akhani et al. (2013), Moharrek et al. (2017), Malekmohammadi et al. (2017) and Koutroumpa et. al. (2018). The family is currently divided into three morphologically distinct and monophyletic tribes: Aegialitideae, Limonieae and Plumbagineae. We abstain from futher dividing the family into the two subfamilies Limonioideae and Plumbaginoideae due to the incongruent placement of *Aegialitis* (comprosing Aegialitideae) observed in cpDNA vs. nrDNA molecular phylogenies (Lledo et al., 2001, 2005, Koutroumpa et al. 2018, Baker et al. 2022, current study).

***Aegialitideae* Z.X.Peng, Fl. Reipubl. Popularis Sin. 60(1): 1. 1987.** Sec. Malekmohammadi & al. (2024)

Type: *Aegialitis* R.Br.

***Aegialitis* R.Br., Prodr. Fl. Nov. Holland.: 426. 1810.** Sec. Malekmohammadi & al. (2024)

Type: *Aegialitis annulata* R.Br.

≡ *Aegialinites* C.Presl in Abh. Königl. Böhm. Ges. Wiss. ser. 5, 3: 533. 1845, nom. superfl. syn. sec. Malekmohammadi & al. (2024)

Notes. – A small genus with only two endangered woody mangrove species distributed in SE Asia to Australia-Papua New Guinea (Kubitzki 1993). These two species comprise a clade and are reciprocally monophyletic with highest support in the ITS phylogeny (current study).
Aegialitis exhibits several autapomorphies, namely fleshy corolla, basifixed anthers, elongated fruit (capsule) with spongy mesocarp and seed two or three times longer than the calyx (e.g., Kubitzki 1993; Lledó et al. 2001). *Aegialitis* also exhibits intermediate features between the tribes Limonieae and Plumbagineae. Specifically, it has similar vegetative and chemical features to Limonieae (Boissier 1848; Maury 1886; Harbone 1967; Hanson et al. 1994; Lledó et al. 2001), but the same breeding system ('Plumbago-type' pollen and monomorphic stigma) and similar anatomical characters to Plumbagineae (Maury 1886; Weber-El Ghobary 1984; Lledó et al. 2001). In molecular phylogenies employing cpDNA markers for *Aegialitis*, the genus is sister to tribe Limonieae (Lledó et al. 2001, 2005; Koutroumpa et al. 2018). This led Lledó et al. (2001) to classify *Aegialitis* comprising tribe Aegialitideae together with Limonieae in the subfamily Limonioideae. However, in molecular phylogenies employing nrDNA markers (Baker et al. 2022, current study), *Aegialitis* is sister to Plumbagineae. Currently the genus is classified in Aegialitideae and the subfamilial classification for Plumbaginaceae is abandoned.

***Aegialitis annulata* R.Br., Prodr. Fl. Nov. Holland.: 426. 1810.** Sec. Lledó & al. (2001)

≡ *Statice annulata* (R.Br.) Spreng., Syst. Veg., ed. 16, 1: 960. 1824 syn. sec. Malekmohammadi & al. (2024) ≡ *Aegialinites annulatus* (R.Br.) C.Presl in Abh. Königl. Böhm. Ges. Wiss. ser. 5, 3: 533. 1845 syn. sec. Malekmohammadi & al. (2024)

***Aegialitis rotundifolia* Roxb., Fl. Ind. 2: 111. 1824.** Sec. Malekmohammadi & al. (2024)

≡ *Aegialinites rotundifolius* (Roxb.) C.Presl in Abh. Königl. Böhm. Ges. Wiss. ser. 5, 3: 533. 1845 syn. sec. Malekmohammadi & al. (2024)
= *Aegialitis annulata* Kurz in J. Asiat. Soc. Bengal, Pt. 2, Nat. Hist. 46(2): 217. 1877 syn. sec. Malekmohammadi & al. (2024) [non *Aegialitis annulata* R.Br.]

Limonieae Reveal in Phytoneuron 33: 2. 2012. Sec. Malekmohammadi & al. (2024)

Type: *Limonium* Mill.
= *Armerieae* Dumort., Anal. Fam. Pl.: 27. 1829 syn. sec. Reveal (2012)
= *Staticeae* Bartl., Ord. Nat. Pl.: 127. 1830 syn. sec. Lledó & al. (2001)

Notes. – *Limonieae* is a phylogenetically well-supported clade (e.g., Malekmohammadi et al. 2017; Koutroumpa et al. 2018) with 21 accepted genera. The species of this tribe are diversified in temperate regions and are morphologically more variable compared to species in *Plumbagineae*. Most of the species are grouped in three genera, *Limonium* Mill., *Armeria* Willd., and *Acantholimon* Boiss., while the remaining species belong to monotypic or small genera (Kubitzki 1993), mostly segregated from *Limonium* and *Acantholimon*. The generic concepts of the small genera previously segregated from *Acantholimon* and their relationships need further studies to be clarified.

Acantholimon Boiss. in Boissier, Diagn. Pl. Orient. ser. 1, 7: 69. 1846, nom. cons. Sec. Moharrek & al. (2017)

Type: *Acantholimon glumaceum* (Jaub. & Spach) Boiss.
= *Statice* subg. *Armeriastrum* Jaub. & Spach in Ann. Sci. Nat., Bot., ser. 2, 20: 248. 1843 syn. sec. Linczevski (1952)
– *Armeriastrum* (Jaub. & Spach) Lindl., Veg. Kingd.: 641. 1846, nom. rej. syn. sec. Linczevski (1952)
= *Chomutowia* B.Fedtsch in Bot. Mater. Gerb. Glavn. Bot. Sada R.S.F.S.R. 3: 3. 1922 syn. sec. WFO 2018. Type: *Chomutowia ekatherinae* B.Fedtsch.
= *Gladiolimon* Mobayen, Revis. Taxon. Acanth.: 296. 1964 syn. sec. Rechinger & Schiman-Czeika (1974). Type: *Gladiolimon speciosissimum* (Aitch. & Hemsl.) Mobayen
= *Cephalorhizum* sect. *Sarcophyllastrum* Rech.f., Fl. Iran. 108: 19. 1974 syn. sec. Malekmohammadi & al. (2024)

Notes. – Large genus of predominantly cushion-forming subshrubs with about 300 currently accepted species (including many narrow endemics), distributed from south-eastern Europe to Central Asia, centred in the mountainous regions of Iran, Turkey and Afghanistan (Kubitzki 1993).
The study by Lledó et al. (2005) included only one representative of *Acantholimon*, which was recovered in a clade together with *Dictyolimon* and *Cephalorhizum*. Moharrek et al. (2014) studied 50 species of *Acantholimon* from Iran and demonstrated the unresolved position of *Popoviolimon turcomanicum* (Popov) Lincz. (= *Cephalorhizum turcomanicum* Popov), found either as sister to *Acantholimon* or nested within that genus, so monophyly of *Acantholimon* was uncertain. In Moharrek et al. (2017), 197 accessions of *Acantholimon* and other genera of *Limonioideae* were included in a broader study that shows that this genus is not monophyletic, with eight small genera, *Bamiania*, *Bukiniczia*, *Chaetolimon*, *Cephalorhizum*, *Dictyolimon*, *Popoviolimon* and *Vassilczenkoa*, branching in between the two major *Acantholimon* clades and *Gladiolimon* being well nested in *Acantholimon*. Following the latter result and morphological data, we merge here *Gladiolimon* in *Acantholimon*. Although a wider circumscription for *Acantholimon* including the other eight small genera could avoid naming a non-monophyletic assemblage, the absence of morphological diagnostic characters for *Acantholimon s.l.*, the unresolved relationships between *Acantholimon s.s.* and some of the smaller genera, the non-comprehensive taxon sampling and the few molecular markers used in the phylogenetic studies hinder for now a formal revision in the circumscription of the genus.

Sectional classifications of *Acantholimon* that were published by different authors based on alphataxonomic revisions (Rechinger and Schima-Czeika 1974; Davis and Bokhari 1982; Mobayen 1964) are not applied in this checklist due to incongruent results of the phylogenetic trees (Moharrek et al. 2017).

***Acantholimon acanthobryum* Rech.f. & Schiman-Czeika, Fl. Iran. 108: 35. 1974.** Sec. Rechinger & Schiman-Czeika (1974)

***Acantholimon acerosum* (Willd.) Boiss. in Boissier, Diagn. Pl. Orient. ser. 1, 7: 80. 1846.** Sec. Rechinger & Schiman-Czeika (1974)

= *Statice acerosa* Willd. in Neue Schriften Ges. Naturf. Freunde Berlin 3: 420. 1801 syn. sec. Boissier (1846); ≡ *Armeriastrum acerosum* (Boiss.) Kuntze, Revis. Gen. Pl. 2: 393. 1891 syn. sec. Malekmohammadi & al. (2024)
= *Acantholimon assyriacum* var. *micacme* Nábělek & Bornm. in Spisy Přír. Fak. Masarykovy Univ. 3: 420. 1929 syn. sec. Doğan & Akaydın (2007)
= *Acantholimon acerosum* var. *persicum* Mobayen, Revis. Taxon. Acanth.: 179. 1964 syn. sec. Malekmohammadi & al. (2024: 6 April 2022)

***Acantholimon acerosum* (Willd.) Boiss. subsp. *acerosum*.** Sec. Doğan & Akaydın (2007)

***Acantholimon acerosum* (Willd.) Boiss. var. *acerosum*.** Sec. Malekmohammadi & al. (2024)

***Acantholimon acerosum* var. *parvifolium* Bokhari in 30: 300. 1970.** Sec. Doğan & Akaydın (2007)

***Acantholimon acerosum* subsp. *brachystachyum* (Boiss.) Doğan & Akaydın in Bot. J. Linn. Soc. 154(3): 410. 2007.** Sec. Doğan & Akaydın (2007)

≡ *Acantholimon acerosum* var. *brachystachyum* Boiss., Fl. Orient. 4(2): 838. 1879 syn. sec. Doğan & Akaydın (2007)
= *Acantholimon phrygium* Boiss. in Boissier, Diagn. Pl. Orient. ser. 1, 7: 79. 1846 syn. sec. Doğan & Akaydın (2007)
= *Acantholimon pinardii* Boiss. in Boissier, Diagn. Pl. Orient. ser. 1, 7: 79. 1846 [as " *pinardi* "] syn. sec. POWO (2017+) ≡ *Armeriastrum pinardii* (Boiss.) Kuntze, Revis. Gen. Pl. 2: 393. 1891 syn. sec. POWO (2017+: 8 April 2022) ≡ *Acantholimon acerosum* var. *pinardii* (Boiss.) Mobayen, Revis. Taxon. Acanth.: 179. 1964 syn. sec. Malekmohammadi & al. (2024) − *Acantholimon pinardi* Boiss. in Boissier, Diagn. Pl. Orient. ser. 1, 7: 79. 1846, orth. var. syn. sec. Doğan & Akaydın (2007) − *Statice caryophyllacea* Boiss. in Boissier, Diagn. Pl. Orient. ser. 1, 7: 80. 1846, in sched., pro syn. syn. sec. Boissier (1846)
= *Acantholimon listoniae* Boiss. in Candolle, Prodr. 12: 631. 1848 syn. sec. Doğan & Akaydın (2007) ≡ *Armeriastrum listoniae* (Boiss.) Kuntze, Revis. Gen. Pl. 2: 393. 1891 syn. sec. Kuntze (1891)
= *Acantholimon tchihatcheffii* Fisch. & C.A.Mey. in Ann. Sci. Nat. Bot., ser. 4, 1: 30. 1854 syn. sec. Doğan & Akaydın (2007)

***Acantholimon acerosum* subsp. *longibracteolatum* Doğan & Akaydın in Bot. J. Linn. Soc. 154(3): 410. 2007.** Sec. Doğan & Akaydın (2007)

***Acantholimon acmostegium* Boiss. & Buhse in Nouv. Mém. Soc. Imp. Naturalistes Moscou 12: 182. 1860.** Sec. Rechinger & Schiman-Czeika (1974)

≡ *Armeriastrum acmostegium* (Boiss. & Buhse) Kuntze, Revis. Gen. Pl. 2: 393. 1891 syn. sec. Malekmohammadi & al. (2024)
= *Acantholimon giselae* Bornm. in Repert. Spec. Nov. Regni Veg. 41: 342. 1937 syn. sec. Rechinger & Schiman-Czeika (1974)

***Acantholimon aegaeum* F.K.Mey. in Haussknechtia, Mitt. Thüring. Bot. Ges. 3: 38. 1987.** Sec. Domina (2011+)

***Acantholimon afanassievii* Lincz. in Bot. Mater. Gerb. Bot. Inst. Komarova Akad. Nauk S.S.S.R. 22: 198. 1963.** Sec. Malekmohammadi & al. (2024)

***Acantholimon agropyroideum* Mobayen, Revis. Taxon. Acanth.: 301. 1964.** Sec. Mobayen (1964)

***Acantholimon ahangarense* Rech.f. & Schiman-Czeika, Fl. Iran. 108: 72. 1974.** Sec. Rechinger & Schiman-Czeika (1974)

***Acantholimon akaydinii* Özüdoğru in Phytotaxa 539(3): 267. 2022.** Sec. Malekmohammadi & al. (2024)

***Acantholimon alaicum* Czerniak., Flora URSS 18: 732. 1952.** Sec. Linczevski (1952)

***Acantholimon alatavicum* Bunge in Mém. Acad. Imp. Sci. Saint Pétersbourg, Sér. 7 18(7): 40. 1872.** Sec. Linczevski (1952)

= *Acantholimon hohenackeri* var. *subsessile* Trautv. in Bull. Soc. Imp. Naturalistes Moscou 39(4): 460. 1866 syn. sec. Bunge (1872) ≡ *Acantholimon alatavicum* var. *subsessile* (Trautv.) Herder in Bull. Soc. Imp. Naturalistes Moscou 41(2): 395. 1868 syn. sec. Linczevski (1952) ≡ *Armeriastrum subsessile* (Trautv.) Kuntze, Revis. Gen. Pl. 2: 393. 1891 syn. sec. Kuntze (1891)
− *Acantholimon alatavicum* var. *typicum* Regel in Trudy Imp. S.-Peterburgsk. Bot. Sada 6(2): 390. 1880, nom. inval. syn. sec. Linczevski (1952)
= *Acantholimon alatavicum* O.Fedtsch. & B.Fedtsch., Consp. Fl. Turkestanicae 5: 190. 1913 syn. sec. Rechinger & Schiman-Czeika (1974)

***Acantholimon alavae* Rech.f. & Schiman-Czeika, Fl. Iran. 108: 75. 1974.** Sec. Rechinger & Schiman-Czeika (1974)

***Acantholimon albanicum* O.Schwarz & F.K.Mey. in Haussknechtia, Mitt. Thüring. Bot. Ges. 3: 31. 1987.** Sec. Domina (2011+)

***Acantholimon albertii* Regel in Trudy Imp. S.-Peterburgsk. Bot. Sada 6: 389. 1879 [as " *alberti* "].** Sec. Linczevski (1952)

− *Acantholimon alberti* Regel in Trudy Imp. S.-Peterburgsk. Bot. Sada 6: 389. 1879, orth. var. syn. sec. Malekmohammadi & al. (2024)

***Acantholimon albocalycinum* Assadi & Mirtadz. in Iran. J. Bot. 11(2): 130. 2006.** Sec. Assadi & Mirtadzadidni (2006)

***Acantholimon alexandri* Fed. in Bot. Zhurn. S.S.S.R. 33: 34. 1948.** Sec. Linczevski (1952)

***Acantholimon alexeenkoanum* Czerniak. ex Ikonn. in Bot. Mater. Gerb. Bot. Inst. Komarova Akad. Nauk S.S.S.R. 22: 10. 1963.** Sec. Malekmohammadi & al. (2024)

***Acantholimon amoenum* Rech.f. & Schiman-Czeika, Fl. Iran. 108: 71. 1974.** Sec. Rechinger & Schiman-Czeika (1974)

***Acantholimon anatolicum* Yıld. in Ot Sist. Bot. Dergisi 16(1): 12. 2009.** Sec. Domina (2011+)

***Acantholimon anisophyllum* Rech.f. & Schiman-Czeika, Fl. Iran. 108: 68. 1974.** Sec. Rechinger & Schiman-Czeika (1974)

***Acantholimon annae* Lincz. in Bot. Mater. Gerb. Bot. Inst. Komarova Akad. Nauk S.S.S.R. 21: 491. 1961.** Sec. Linczevski (1961)

***Acantholimon antilibanoticum* Mouterde in Saussurea 4: 18. 1973.** Sec. Domina (2011+)

***Acantholimon anzobicum* Lincz., Fl. Tadzhikskoĭ SSR 8: 480, 30. 1986.** Sec. Linczevski (1986)

= *Acantholimon anzobicum* var. *albiflorum* Lincz., Fl. Tadzhikskoĭ SSR 8: 480. 1986 syn. sec. POWO (2017+: 7 april 2022)

***Acantholimon araxanum* Bunge in Mém. Acad. Imp. Sci. Saint Pétersbourg, Sér. 7 18(7): 33. 1872.** Sec. Linczevski (1952)

≡ *Armeriastrum araxanum* (Bunge) Kuntze, Revis. Gen. Pl. 2: 393. 1891 syn. sec. Kuntze (1891)
= *Acantholimon trautvetteri* Kusn., Fl. Caucas. Crit. 4(1): 183. 1902 syn. sec. Linczevski (1952)

***Acantholimon argyrostachyum* Rech.f. & Schiman-Czeika, Fl. Iran. 108: 70. 1974.** Sec. Rechinger & Schiman-Czeika (1974)

***Acantholimon aristulatum* Bunge in Mém. Acad. Imp. Sci. Saint Pétersbourg, Sér. 7 18(2): 60. 1872.** Sec. Bunge (1872)

≡ *Armeriastrum aristulatum* (Bunge) Kuntze, Revis. Gen. Pl. 2: 393. 1891 syn. sec. Kuntze (1891)

***Acantholimon armenum* Boiss. & A.Huet, Diagn. Pl. Orient. ser. 2, 4: 64. 1859.** Sec. Bunge (1872)

≡ *Armeriastrum armenum* (Boiss.) Kuntze, Revis. Gen. Pl. 2: 393. 1891 syn. sec. Kuntze (1891)
= *Acantholimon armenum* var. *puberulum* Trautv. in Trudy Imp. S.-Peterburgsk. Bot. Sada 2: 582. 1873 syn. sec. Linczevski (1952)
= *Acantholimon armenum* var. *typicum* Trautv. in Trudy Imp. S.-Peterburgsk. Bot. Sada 2: 581. 1873 syn. sec. Linczevski (1952)
= *Acantholimon baltanense* Boiss. & Hausskn. ex Boiss., Fl. Orient. 4(2): 838. 1879 syn. sec. Doğan & Akaydın (2007) ≡ *Armeriastrum baltanense* (Boiss. & Hausskn. ex Boiss.) Kuntze, Revis. Gen. Pl. 2: 394. 1891 syn. sec. Kuntze (1891)
= *Acantholimon armenum* var. *balansae* Kusn., Fl. Caucas. Crit. 4(1): 187. 1902, nom. illeg. syn. sec. Linczevski (1952) [non *Acantholimon armenum* var. *balansae* Boiss. & A.Huet] ≡ *Acantholimon balansae* (Kusn.) Grossh., Fl. Kavkaza 1(3): 217. 1932, nom. illeg. syn. sec. Linczevski (1952) [non *Acantholimon balansae* Boiss. ex Bunge]

***Acantholimon armenum* Boiss. & A.Huet var. *armenum*.** Sec. Bokhari & Edmondson (1982)

***Acantholimon armenum* var. *balansae* Boiss. & A.Huet in Boissier, Diagn. Pl. Orient. ser. 2, 4: 64. 1859.** Sec. Linczevski (1952)

= *Acantholimon balansae* Boiss. ex Bunge in Mém. Acad. Imp. Sci. Saint Pétersbourg, Sér. 7 18(2): 36. 1872 syn. sec. Linczevski (1952) ≡ *Armeriastrum balansae* (Boiss. ex Bunge) Kuntze, Revis. Gen. Pl. 2: 393. 1891 syn. sec. Kuntze (1891)
= *Acantholimon haussknechtii* Bunge in Mém. Acad. Imp. Sci. Saint Pétersbourg, Sér. 7 18(2): 37. 1872 [as " *hausknechti* "] syn. sec. Linczevski (1952) ≡ *Acantholimon caryophyllaceum* Hausskn. ex Bunge in Mém. Acad. Imp. Sci. Saint Pétersbourg, Sér. 7 18(2): 37. 1872, nom. illeg. syn. sec. Bunge (1872) ≡ *Armeriastrum hausknechtii* (Bunge) Kuntze, Revis. Gen. Pl. 2: 393. 1891 syn. sec. Kuntze (1891) − *Acantholimon hausknechti* Bunge in Mém. Acad. Imp. Sci. Saint Pétersbourg, Sér. 7, 18(2): 37. 1872, orth. var. syn. sec. Malekmohammadi & al. (2024) [is orthographic variant for *Acantholimon haussknechtii* Bunge]

***Acantholimon artosense* Doğan & Akaydın in Bot. J. Linn. Soc. 144(4): 498. 2004.** Sec. Doğan & Akaydın (2007)

***Acantholimon arundoscapum* Mobayen, Revis. Taxon. Acanth.: 298. 1964.** Sec. Mobayen (1964)

***Acantholimon aspadanum* Bunge in Mém. Acad. Imp. Sci. Saint Pétersbourg, Sér. 7 18(2): 50. 1872.** Sec. Bunge (1872)

≡ *Armeriastrum aspadanum* (Bunge) Kuntze, Revis. Gen. Pl. 2: 393. 1891 syn. sec. Kuntze (1891)

***Acantholimon asphodelinum* Mobayen, Revis. Taxon. Acanth.: 300. 1964.** Sec. Rechinger & Schiman-Czeika (1974)

***Acantholimon assadii* Mirtadz. & Bordbar in Phytotaxa 574(1): 100. 2022.** Sec. Bordbar & Mirtadzadini (2022)

***Acantholimon astragalinum* Mobayen, Revis. Taxon. Acanth.: 301. 1964.** Sec. Rechinger & Schiman-Czeika (1974)

***Acantholimon atrofuscum* Rech.f. in Biol. Skr. 13(4) (Symb. Afgan. 5): 176. 1963.** Sec. Rechinger & Schiman-Czeika (1974)

***Acantholimon atropatanum* Bunge in Mém. Acad. Imp. Sci. Saint Pétersbourg, Sér. 7 18(2): 29. 1872.** Sec. Rechinger & Schiman-Czeika (1974)

≡ *Armeriastrum atropatanum* (Bunge) Kuntze, Revis. Gen. Pl. 2: 393. 1891 syn. sec. Kuntze (1891)

***Acantholimon auganum* Bunge in Mém. Acad. Imp. Sci. Saint Pétersbourg, Sér. 7 18(2): 29. 1872.** Sec. Bunge (1872)

≡ *Armeriastrum auganum* (Bunge) Kuntze, Revis. Gen. Pl. 2: 393. 1891 syn. sec. Kuntze (1891)
= *Acantholimon munroanum* Aitch. & Hemsl. in J. Linn. Soc., Bot. 18: 76. 1881 ["1880"] syn. sec. POWO (2017+: 5 April 2022)

***Acantholimon aulieatense* Czerniak. in Bot. Mater. Gerb. Glavn. Bot. Sada R.S.F.S.R. 4: 65. 1923.** Sec. Linczevski (1952)

= *Acantholimon gramineum* Korovin in Trudy Turkestansk. Naucn. Obsc. I: 82. 1923 syn. sec. Linczevski (1952)

***Acantholimon austro-iranicum* Rech.f. & Schiman-Czeika, Fl. Iran. 108: 149. 1974.** Sec. Rechinger & Schiman-Czeika (1974)

***Acantholimon avanosicum* Doğan & Akaydın in Bot. J. Linn. Soc. 138(3): 365. 2002.** Sec. Doğan & Akaydın (2007)

***Acantholimon avenaceum* Bunge in Mém. Acad. Imp. Sci. Saint Pétersbourg, Sér. 7 18(7): 25. 1872.** Sec. Linczevski (1952)

≡ *Armeriastrum avenaceum* (Bunge) Kuntze, Revis. Gen. Pl. 2: 393. 1891 syn. sec. Kuntze (1891)
− *Acantholimon avenaceum* f. *simplicior* Bornm. ex Lincz. in Schischkin & Bobrov, Flora URSS 18: 327. 1952, nom. inval. syn. sec. Linczevski (1952)

***Acantholimon azizae* Mobayen, Revis. Taxon. Acanth.: 299. 1964.** Sec. Mobayen (1964)

***Acantholimon bakhtiaricum* Assadi in Iran. J. Bot. 10(1): 26. 2003.** Sec. Assadi (2003)

***Acantholimon balchanicum* Korovin in Trudy Turkestansk. Naucn. Obsc. I: 79. 1923.** Sec. Linczevski (1952)

***Acantholimon bashkaleicum* Doğan & Akaydın in Bot. J. Linn. Soc. 144(4): 498. 2004.** Sec. Doğan & Akaydın (2007)

***Acantholimon ×baubaschatense* Lazkov in Novosti Sist. Vyssh. Rast. 40: 230. 2009 ["2008"].** Sec. Malekmohammadi & al. (2024)

***Acantholimon birandii* Doğan & Akaydın in Nordic J. Bot. 21(5): 482. 2001.** Sec. Doğan & Akaydın (2007)

***Acantholimon blakelockii* Mobayen, Revis. Taxon. Acanth.: 309. 1964.** Sec. Rechinger & Schiman-Czeika (1974)

− *Acantholimon blakelackii* Mobayen, orth. var. syn. sec. Malekmohammadi & al. (2024) [is orthographic variant for *Acantholimon blakelockii* Mobayen]

***Acantholimon blandum* Czerniak. in Feddes Repert. Spec. Nov. Regni Veg. 27: 275. 1930.** Sec. Linczevski (1952)

***Acantholimon bodeanum* Bunge in Mém. Acad. Imp. Sci. Saint Pétersbourg, Sér. 7 18(2): 42. 1872.** Sec. Rechinger & Schiman-Czeika (1974)

≡ *Armeriastrum bodeanum* (Bunge) Kuntze, Revis. Gen. Pl. 2: 393. 1891 syn. sec. Kuntze (1891)

***Acantholimon bodeanum* Bunge subsp. *bodeanum*.** Sec. Malekmohammadi & al. (2024)

***Acantholimon bodeanum* subsp. *pilosum* Assadi, Fl. Iran 51: 162. 2005.** Sec. Assadi (2005)

***Acantholimon bonesseae* Parsa, Pl. Nov. Iranicae: 11. 1946.** Sec. Parsa (1946)

***Acantholimon borodinii* Krasn., List. Pl. Coll. E. Thian Shan: 128. 1886.** Sec. Linczevski (1952)

= *Acantholimon roborowskii* Czerniak. in Trudy Bot. Inst. Akad. Nauk S.S.S.R., ser. 1, Fl. Sist. Vyssh. Rast. 3: 267. 1937 syn. sec. Linczevski (1952)

***Acantholimon brachyphyllum* Boiss. in Candolle, Prodr. 12: 628. 1848.** Sec. Rechinger & Schiman-Czeika (1974)

≡ *Armeriastrum brachyphyllum* (Boiss.) Kuntze, Revis. Gen. Pl. 2: 394. 1891 syn. sec. Kuntze (1891)

***Acantholimon brachystachyum* Boiss. ex Bunge in Mém. Acad. Imp. Sci. Saint Pétersbourg, Sér. 7 18(2): 51. 1872.** Sec. Rechinger & Schiman-Czeika (1974)

≡ *Armeriastrum brachystachyum* (Boiss. ex Bunge) Kuntze, Revis. Gen. Pl. 2: 393. 1891 syn. sec. Kuntze (1891)
= *Acantholimon kurdicum* Bunge in Mém. Acad. Imp. Sci. Saint Pétersbourg, Sér. 7 18(2): 52. 1872 syn. sec. Rechinger & Schiman-Czeika (1974) ≡ *Armeriastrum kurdicum* (Bunge) Kuntze, Revis. Gen. Pl. 2: 393. 1891 syn. sec. Kuntze (1891)
= *Acantholimon brachystachyum* var. *brachyphyllum* Boiss., Fl. Orient. 4(2): 345. 1879 syn. sec. Rechinger & Schiman-Czeika (1974)

***Acantholimon bracteatum* (Girard) Boiss. in Boissier, Diagn. Pl. Orient. ser. 1, 7: 70. 1846.** Sec. Linczevski (1952)

≡ *Statice bracteata* Girard in Ann. Sci. Nat., Bot., ser. 3, 2: 330. 1844 syn. sec. Boissier (1848) ≡ *Armeriastrum bracteatum* (Boiss.) Kuntze, Revis. Gen. Pl. 2: 393. 1891 syn. sec. Kuntze (1891) ≡ *Acantholimon bracteatum* (Girard) Boiss. var. *bracteatum* syn. sec. Malekmohammadi & al. (2024)
= *Acantholimon splendidum* Bunge in Mém. Acad. Imp. Sci. Saint Pétersbourg, Sér. 7 18(2): 17. 1872 syn. sec. Linczevski (1952) ≡ *Acantholimon bracteatum* var. *splendidum* (Bunge) Boiss., Fl. Orient. 4(2): 827. 1879 syn. sec. Linczevski (1952) ≡ *Armeriastrum splendidum* (Bunge) Kuntze, Revis. Gen. Pl. 2: 394. 1891 syn. sec. Malekmohammadi & al. (2024)
= *Acantholimon bracteatum* var. *intermedium* Bordz. in Zhurn. Inst. Bot. UAN 3(11): 75. 1935 syn. sec. Malekmohammadi & al. (2024)

***Acantholimon brecklei* Rech.f. & Schiman-Czeika, Fl. Iran. 108: 48. 1974.** Sec. Rechinger & Schiman-Czeika (1974)

***Acantholimon bromifolium* Boiss. ex Bunge in Mém. Acad. Imp. Sci. Saint Pétersbourg, Sér. 7 18(2): 23. 1872.** Sec. Bunge (1872)

≡ *Armeriastrum bromifolium* (Boiss. ex Bunge) Kuntze, Revis. Gen. Pl. 2: 393. 1891 syn. sec. Kuntze (1891)
− *Acantholimon bromifolium* f. *breviscapa* Parsa, in sched. syn. sec. Malekmohammadi & al. (2024)

***Acantholimon bromifolium* var. *approximatum* Bornm., Abh. K. K. Zool.-Bot. Ges. Wien 9: 164. 1910.** Sec. Malekmohammadi & al. (2024)

Acantholimon bromifolium **Boiss. ex Bunge var. *bromifolium*.** Sec. Rechinger & Schiman-Czeika (1974)

Acantholimon bromifolium **var. *ilamicum* Mobayen, Revis. Taxon. Acanth.: 135. 1964.** Sec. Mobayen (1964)

Acantholimon bromifolium **var. *iranicum* (Bornm.) Rech.f. & Schiman-Czeika, Fl. Iran. 108: 44. 1974.** Sec. Rechinger & Schiman-Czeika (1974)

≡ *Acantholimon iranicum* Bornm. in Beih. Bot. Centralbl. Abt. 2, 22(2): 138. 1907 syn. sec. Rechinger & Schiman-Czeika (1974)

Acantholimon bromifolium **var. *lolioides* Rech.f. & Schiman-Czeika, Fl. Iran. 108: 44. 1974.** Sec. Rechinger & Schiman-Czeika (1974)

Acantholimon bromifolium **var. *platyphyllum* Bornm. in Beih. Bot. Centralbl. Abt. 2, 22(2): 137. 1907.** Sec. Rechinger & Schiman-Czeika (1974)

Acantholimon butkovii **Lincz. in Bot. Mater. Gerb. Bot. Inst. Komarova Akad. Nauk S.S.S.R. 21: 488. 1961.** Sec. Linczevski (1961)

Acantholimon cabulicum **Boiss. in Candolle, Prodr. 12: 623. 1848.** Sec. Rechinger & Schiman-Czeika (1974)

≡ *Armeriastrum cabulicum* (Boiss.) Kuntze, Revis. Gen. Pl. 2: 393. 1891 syn. sec. Kuntze (1891)

Acantholimon caesareum **Boiss. & Balansa in Boissier, Diagn. Pl. Orient. ser. 2, 4: 66. 1859.** Sec. Bunge (1872)

≡ *Armeriastrum caesareum* (Boiss. & Balansa) Kuntze, Revis. Gen. Pl. 2: 393. 1891 syn. sec. Kuntze (1891)
= *Acantholimon caesareum* var. *elongatum* Mobayen, Revis. Taxon. Acanth.: 210. 1964 syn. sec. Malekmohammadi & al. (2024)

Acantholimon calocephalum **Aitch. & Hemsl. in J. Linn. Soc., Bot. 18: 77. 1880 ["1881"].** Sec. Rechinger & Schiman-Czeika (1974)

Acantholimon calvertii **Boiss., Diagn. Pl. Orient. ser. 2, 4: 65. 1859.** Sec. Bokhari & Edmondson (1982)

≡ *Armeriastrum calvertii* (Boiss.) Kuntze, Revis. Gen. Pl. 2: 393. 1891 syn. sec. Kuntze (1891)
= *Acantholimon calvertii* var. *sanguineum* Mobayen, Revis. Taxon. Acanth.: 312. 1964 syn. sec. Doğan & Akaydın (2007)
= *Acantholimon glumaceum* var. *glabra* Mobayen, Revis. Taxon. Acanth.: 313. 1964 syn. sec. Doğan & Akaydın (2007)

Acantholimon calvertii **Boiss. var. *calvertii*.** Sec. Doğan & Akaydın (2007)

Acantholimon calvertii **var. *glabrum* Akaydın & Dogan in Bot. J. Linn. Soc. 154(3): 403. 2007.** Sec. Doğan & Akaydın (2007)

Acantholimon capitatum **Sosn. in Vĕstn. Tiflissk. Bot. Sada Ann. 11: 11. 1915.** Sec. Doğan & Akaydın (2007)

≡ *Acantholimon bracteatum* var. *capitatum* (Sosn.) Bokhari in 30: 300. 1970 syn. sec. Doğan & Akaydın (2007)
= *Acantholimon scabiosum* Mobayen, Revis. Taxon. Acanth.: 298. 1964 syn. sec. Bokhari & Edmondson (1982)
= *Acantholimon scabiosum* var. *nudicalyx* Mobayen, Revis. Taxon. Acanth.: 98. 1964 syn. sec. Malekmohammadi & al. (2024)

Acantholimon capitatum **Sosn. subsp. *capitatum*.** Sec. Doğan & Akaydın (2007)

Acantholimon capitatum **subsp. *sivasicum* Doğan & H.Duman in Bot. J. Linn. Soc. 154(3): 401. 2007.** Sec. Doğan & Akaydın (2007)

Acantholimon carinatum **Rech.f. & Schiman-Czeika, Fl. Iran. 108: 118. 1974.** Sec. Rechinger & Schiman-Czeika (1974)

Acantholimon caryophyllaceum **Boiss. in Boissier, Diagn. Pl. Orient. ser. 1, 7: 78. 1846.** Sec. Linczevski (1952)

≡ *Armeriastrum caryophyllaceum* (Boiss.) Kuntze, Revis. Gen. Pl. 2: 393. 1891 syn. sec. Kuntze (1891) ≡ *Acantholimon caryophyllaceum* Boiss. subsp. *caryophyllaceum* syn. sec. Malekmohammadi & al. (2024) − *Statice caryophyllacea* Boiss. & Hohen. ex Boiss. in Boissier, Diagn. Pl. Orient. ser. 1, 7: 79. 1846, in sched., pro syn. syn. sec. Boissier (1846)

Acantholimon catenatum **Rech.f. & Schiman-Czeika, Fl. Iran. 108: 71. 1974.** Sec. Rechinger & Schiman-Czeika (1974)

Acantholimon cephalotes **Boiss. in Candolle, Prodr. 12: 622. 1848.** Sec. Rechinger & Schiman-Czeika (1974)

≡ *Armeriastrum cephalotes* (Boiss.) Kuntze, Revis. Gen. Pl. 2: 393. 1891 syn. sec. Kuntze (1891)
− *Acantholimon cephalotum* St.-Lag. in Ann. Soc. Bot. Lyon 7: 118. 1880 syn. sec. Malekmohammadi & al. (2024) [is orthographic variant for *Acantholimon cephalotes* Boiss.]

Acantholimon cephalotoides **Rech.f. in Oesterr. Bot. Z. 95: 426. 1949.** Sec. Rechinger & Schiman-Czeika (1974)

Acantholimon chitralicum **Rech.f. & Schiman-Czeika, Fl. Iran. 108: 112. 1974.** Sec. Rechinger & Schiman-Czeika (1974)

Acantholimon chlorostegium **Rech.f. & Schiman-Czeika, Fl. Iran. 108: 139. 1974.** Sec. Rechinger & Schiman-Czeika (1974)

Acantholimon chrysostegium **Rech.f. & Schiman-Czeika, Fl. Iran. 108: 46. 1974.** Sec. Rechinger & Schiman-Czeika (1974)

Acantholimon collare **Köie & Rech.f. in Anz. Österr. Akad. Wiss., Math.-Naturwiss. Kl. 92: 273. 1955.** Sec. Rechinger & Schiman-Czeika (1974)

Acantholimon compactum **Korovin in Trudy Turkestansk. Naucn. Obsc. 1: 83. 1923.** Sec. Linczevski (1952)

Acantholimon confertiflorum **Bokhari in 30: 295. 1970.** Sec. Bokhari & Edmondson (1982)

Acantholimon cupreo-olivascens **Rech.f. & Schiman-Czeika, Fl. Iran. 108: 63. 1974.** Sec. Rechinger & Schiman-Czeika (1974)

***Acantholimon cymosum* Bunge in Mém. Acad. Imp. Sci. Saint Pétersbourg, Sér. 7 18(2): 15. 1872.** Sec. Rechinger & Schiman-Czeika (1974)

≡ *Armeriastrum cymosum* (Bunge) Kuntze, Revis. Gen. Pl. 2: 393. 1891 syn. sec. Kuntze (1891)
= *Acantholimon paniculatum* Rech.f. in Repert. Spec. Nov. Regni Veg. 48: 163. 1940 syn. sec. Rechinger & Schiman-Czeika (1974)

***Acantholimon damassanum* Mobayen, Revis. Taxon. Acanth.: 306. 1964.** Sec. Doğan & Akaydın (2007)

≡ *Acantholimon damassanum* var. *damassanum* syn. sec. Malekmohammadi & al. (2024: 7 April 2022)
= *Acantholimon damassanum* var. *lancibracteatum* Bokhari in 30: 300. 1970 syn. sec. Doğan & Akaydın (2007: 7 April 2022)

***Acantholimon davisii* Akaydın & M.B.Crespo in Phytotaxa 334(1): 80. 2018.** Sec. Akaydın & Crespo (2018)

= *Acantholimon caryophyllaceum* subsp. *parviflorum* Bokhari in 30: 299. 1970 syn. sec. Akaydın & Crespo (2018) ≡ *Acantholimon parviflorum* (Bokhari) Akaydın & Dogan in Bot. J. Linn. Soc. 154(3): 414. 2007, nom. illeg. syn. sec. Akaydın & Crespo (2018)

***Acantholimon demavendicum* Bornm. in Bull. Herb. Boissier, sér. 2, 8: 122. 1908.** Sec. Malekmohammadi & al. (2024)

− *Acantholimon demawendicum* Bornm., orth. var. syn. sec. Malekmohammadi & al. (2024) [is orthographic variant for *Acantholimon demavendicum* Bornm.]

***Acantholimon densiflorum* Assadi in Iran. J. Bot. 11(1): 32. 2005.** Sec. Assadi (2005)

***Acantholimon dianthifolium* Bokhari in 30: 296. 1970.** Sec. Rechinger & Schiman-Czeika (1974)

***Acantholimon diapensioides* Boiss. in Candolle, Prodr. 12: 624. 1848.** Sec. Bunge (1872)

≡ *Armeriastrum diapensioides* (Boiss.) Kuntze, Revis. Gen. Pl. 2: 393. 1891 syn. sec. Kuntze (1891)

***Acantholimon distachyum* Boiss. in Candolle, Prodr. 12: 623. 1848.** Sec. Bunge (1872)

≡ *Armeriastrum distachyum* (Boiss.) Kuntze, Revis. Gen. Pl. 2: 393. 1891 syn. sec. Kuntze (1891)

***Acantholimon distichum* Rech.f. & Schiman-Czeika, Fl. Iran. 108: 146. 1974.** Sec. Rechinger & Schiman-Czeika (1974)

***Acantholimon diversifolium* O.Schwarz & F.K.Mey. in Haussknechtia, Mitt. Thüring. Bot. Ges. 3: 43. 1987.** Sec. Domina (2011+)

***Acantholimon doganii* Bağcı, Doğu & Akaydın in Nordic J. Bot. 27(3): 228. 2009.** Sec. Domina (2011+)

***Acantholimon ecae* Aitch. & Hemsl. in Trans. Linn. Soc. London, Bot. 3(1): 84. 1888.** Sec. Rechinger & Schiman-Czeika (1974)

***Acantholimon edmondsonii* Rech.f. & Schiman-Czeika, Fl. Iran. 108: 122. 1974.** Sec. Rechinger & Schiman-Czeika (1974)

***Acantholimon ekatherinae* (B.Fedtsch.) Czerniak. in Schischkin & Bobrov, Flora URSS 18: 326. 1952.** Sec. Linczevski (1952)

≡ *Chomutowia ekatherinae* B.Fedtsch. in Bot. Mater. Gerb. Glavn. Bot. Sada R.S.F.S.R. 3: 3. 1922 syn. sec. Linczevski (1952)
= *Acantholimon tschimganicum* Korovin in Trudy Turkestansk. Nauchn. Obshch. 1: 79. 1923 syn. sec. Linczevski (1952)

***Acantholimon ekbergianum* Rech.f. & Schiman-Czeika, Fl. Iran. 108: 55. 1974.** Sec. Rechinger & Schiman-Czeika (1974)

***Acantholimon ekimii* Doğan & Akaydın in Bot. J. Linn. Soc. 154(3): 404. 2007.** Sec. Doğan & Akaydın (2007)

***Acantholimon embergeri* Mobayen, Revis. Taxon. Acanth.: 299. 1964.** Sec. Rechinger & Schiman-Czeika (1974)

− *Acantholimon eubergeri* Mobayen, Revis. Taxon. Acanth.: 299. 1964 syn. sec. Malekmohammadi & al. (2024) [is misspelling for *Acantholimon embergeri* Mobayen]

***Acantholimon erinaceum* (Jaub. & Spach) Lincz. in Schischkin & Bobrov, Flora URSS 18: 370. 1952.** Sec. Linczevski (1952)

≡ *Statice erinacea* Jaub. & Spach, Ill. Pl. Orient. 1(10): 163. 1844 syn. sec. Linczevski (1952) ≡ *Acantholimon roseum* var. *erinaceum* (Jaub. & Spach) Boiss. in Boissier, Diagn. Pl. Orient. ser. 1, 7: 71. 1846 syn. sec. Linczevski (1952) ≡ *Armeriastrum erinaceum* (Jaub. & Spach) Kuntze, Revis. Gen. Pl. 2: 393. 1891 syn. sec. Malekmohammadi & al. (2024)
= *Statice juniperina* Willd. ex Steud., Nomencl. Bot. 1: 811. 1821 syn. sec. Linczevski (1952)
= *Statice jaubertii* Girard in Ann. Sci. Nat., Bot., ser. 3, 2: 331. 1844 syn. sec. Linczevski (1952)
= *Statice pungens* Jaub. & Spach, Ill. Pl. Orient. 1(10): 163. 1844 syn. sec. Linczevski (1952) ≡ *Acantholimon roseum* var. *pungens* Boiss. in Boissier, Diagn. Pl. Orient. ser. 1, 7: 71. 1846 syn. sec. Linczevski (1952)
= *Acantholimon roseum* Boiss. in Boissier, Diagn. Pl. Orient. ser. 1, 7: 71. 1846 syn. sec. Linczevski (1952) ≡ *Statice rosea* (Boiss.) Boiss. in Candolle, Prodr. 12: 624. 1848 syn. sec. Boissier (1848)
= *Acantholimon rubellum* Boiss. ex Bunge in Mém. Acad. Imp. Sci. Saint Pétersbourg, Sér. 7 18(2): 66. 1872 syn. sec. Linczevski (1952)
= *Acantholimon roseum* var. *pubescens* Czebnjak. in Acta Horti Petrop. 44: 102. 1931 syn. sec. Linczevski (1952)
= *Acantholimon purpureum* Parsa, Pl. Nov. Iranicae: 11. 1946 syn. sec. Rechinger & Schiman-Czeika (1974)
= *Acantholimon parsianum* Lincz. in Bot. Mater. Gerb. Bot. Inst. Komarova Akad. Nauk S.S.S.R. 14: 279. 1951 syn. sec. Rechinger & Schiman-Czeika (1974)

***Acantholimon erythraeum* Bunge in Trudy Imp. S.-Peterburgsk. Bot. Sada 3: 99. 1875.** Sec. Linczevski (1952)

***Acantholimon eschkerense* Boiss. & Hausskn. ex Boiss., Fl. Orient. 4(2): 844. 1879.** Sec. Rechinger & Schiman-Czeika (1974)

≡ *Armeriastrum eschkerense* (Boiss. & Hausskn. ex Boiss.) Kuntze, Revis. Gen. Pl. 2: 394. 1891 syn. sec. Kuntze (1891)

***Acantholimon esfandiarii* Rech.f. & Schiman-Czeika, Fl. Iran. 108: 140. 1974.** Sec. Rechinger & Schiman-Czeika (1974)

***Acantholimon evrenii* Doğan & Akaydın in Bot. J. Linn. Soc. 149(3): 352. 2005.** Sec. Doğan & Akaydın (2007)

***Acantholimon fasciculare* Boiss. in Candolle, Prodr. 12: 626. 1848.** Sec. Bunge (1872)

≡ *Armeriastrum fasciculare* (Boiss.) Kuntze, Revis. Gen. Pl. 2: 393. 1891 syn. sec. Kuntze (1891)
= *Acantholimon tragacanthinum* Griff. ex Bunge, nom. illeg., in syn. syn. sec. Bunge 1872 in Mém. Acad. Imp. Sci. Saint Pétersbourg, Sér. 7 18(2): 64. 1872, nom. illeg. syn. sec. Bunge (1872) − *Acantholimon tragacanthium* Griff. ex Bunge in Mém. Acad. Imp. Sci. Saint Pétersbourg, Sér. 7 18(2): 64. 1872, orth. var. syn. sec. Bunge (1872)
= *Acantholimon parsanum* Mobayen, Revis. Taxon. Acanth.: 310. 1964 syn. sec. Rechinger & Schiman-Czeika (1974)

***Acantholimon faustii* Trautv. in Trudy Imp. S.-Peterburgsk. Bot. Sada 2: 482. 1873.** Sec. Rechinger & Schiman-Czeika (1974)

≡ *Armeriastrum faustii* (Trautv.) Kuntze, Revis. Gen. Pl. 2: 394. 1891 syn. sec. Kuntze (1891) ≡ *Acantholimon bodeanum* var. *faustii* (Trautv.) Mobayen, Revis. Taxon. Acanth.: 223. 1964 syn. sec. Malekmohammadi & al. (2024)

***Acantholimon fedorovii* Tamamsch. & Mirzoeva in Dokl. Akad. Nauk Armenii 21(4): 184. 1955.** Sec. Domina (2011+)

***Acantholimon festucaceum* (Jaub. & Spach) Boiss. in Boissier, Diagn. Pl. Orient. ser. 1, 7: 76. 1846.** Sec. Bunge (1872)

≡ *Statice festucacea* Jaub. & Spach, Ill. Pl. Orient. 1(10): 163. 1844 syn. sec. Boissier (1846) ≡ *Armeriastrum festucaceum* (Jaub. & Spach) Kuntze, Revis. Gen. Pl. 2: 393. 1891 syn. sec. Kuntze (1891)
= *Acantholimon flexuosum* var. *laxiflorum* Mobayen, Revis. Taxon. Acanth.: 145. 1964 syn. sec. Rechinger & Schiman-Czeika (1974)

***Acantholimon festucaceum* (Jaub. & Spach) Boiss. var. *festucaceum*.** Sec. Malekmohammadi & al. (2024)

***Acantholimon festucaceum* var. *laxiflora* Boiss. in Boissier, Diagn. Pl. Orient. ser. 1, 7: 77. 1846.** Sec. Malekmohammadi & al. (2024)

***Acantholimon fetisowi* Regel, Decas Pl. Nov.: 8. 1882.** Sec. Linczevski (1952)

− *Acantholimon fetissovii* Regel, orth. var. syn. sec. Linczevski (1952) [is orthographic variant for *Acantholimon fetisowi* Regel]
= *Acantholimon murorum* Korovin in Bot. Mater. Gerb. Glavn. Bot. Sada R.S.F.S.R. 3: 191. 1922 syn. sec. Malekmohammadi & al. (2024: 4 April 2022)
= *Acantholimon marmoreum* Korovin in Trudy Turkestansk. Naucn. Obsc. 1: 80. 1923 syn. sec. Linczevski (1952)

***Acantholimon flabellum* Assadi in Phytotaxa 227(1): 99. 2015.** Sec. Mahmoodi & al. (2015)

− *Acantholimon flabellum* Assadi in Iran. J. Bot. 11(1): 32. 2005, nom. inval. syn. sec. Mahmoodi & al. (2015)

***Acantholimon flexuosum* Boiss. ex Bunge in Mém. Acad. Imp. Sci. Saint Pétersbourg, Sér. 7 18(2): 51. 1872.** Sec. Rechinger & Schiman-Czeika (1974)

≡ *Armeriastrum flexuosum* (Boiss. ex Bunge) Kuntze, Revis. Gen. Pl. 2: 393. 1891 syn. sec. Kuntze (1891)

***Acantholimon fominii* Kusn., Delect. Pl. Jurjev. 3: 50. 1900.** Sec. Linczevski (1952)

***Acantholimon gabrieljaniae* Mirzoeva in Biol. Zhurn. Armenii 34(9): 973. 1981 [as " *gabrieljanii* "].** Sec. Malekmohammadi & al. (2024)

− *Acantholimon gabrieljanii* Mirzoeva in Biol. Zhurn. Armenii 34(9): 973. 1981, orth. var. syn. sec. Malekmohammadi & al. (2024)

***Acantholimon gadukense* Mobayen, Revis. Taxon. Acanth.: 309. 1964.** Sec. Rechinger & Schiman-Czeika (1974)

***Acantholimon gaudanense* Czerniak. in Trudy Bot. Sada Akad. Nauk S.S.S.R. 44: 99. 1931.** Sec. Linczevski (1952)

***Acantholimon gemicianum* Kaptaner İğci, Körüklü & Aytaç in Ann. Bot. Fenn. 54(1-3): 83. 2017.** Sec. Malekmohammadi & al. (2024)

***Acantholimon genistoides* (Jaub. & Spach) Boiss. in Boissier, Diagn. Pl. Orient. ser. 1, 7: 76. 1846.** Sec. Bunge (1872)

≡ *Statice genistoides* Jaub. & Spach, Ill. Pl. Orient. 1(10): 162. 1844 syn. sec. Boissier & Heldreich (1846) ≡ *Armeriastrum genistoides* (Jaub. & Spach) Kuntze, Revis. Gen. Pl. 2: 393. 1891 syn. sec. Kuntze (1891)

***Acantholimon genistioides* var. *khossrovii* Mobayen, Revis. Taxon. Acanth.: 243. 1964.** Sec. Mobayen (1964)

***Acantholimon genistoides* (Jaub. & Spach) Boiss. var. *genistoides*.** Sec. Malekmohammadi & al. (2024)

***Acantholimon ghoranum* Rech.f. & Schiman-Czeika, Fl. Iran. 108: 123. 1974.** Sec. Rechinger & Schiman-Czeika (1974)

***Acantholimon gilliatii* Turrill in Bull. Misc. Inform. Kew 1930(9): 441. 1930.** Sec. Rechinger & Schiman-Czeika (1974)

***Acantholimon gillii* Rech.f. & Köie in Biol. Skr. 13(4) (Symb. Afgan. 5): 162. 1963.** Sec. Rechinger & Schiman-Czeika (1974)

***Acantholimon glabratum* Assadi in Iran. J. Bot. 11(1): 35. 2005.** Sec. Assadi (2005)

***Acantholimon glabratum* Assadi subsp. *glabratum*.** Sec. Assadi (2005)

***Acantholimon glabratum* subsp. *kashanense* Batuli & Assadi, Fl. Iran 51: 83. 2005.** Sec. Assadi (2005)

***Acantholimon glumaceum* (Jaub. & Spach) Boiss. in Boissier, Diagn. Pl. Orient. ser. 1, 7: 75. 1846.** Sec. Bunge (1872)

≡ *Statice glumacea* Jaub. & Spach, Ill. Pl. Orient. 1(10): 162, 166, t. 91. 1844 syn. sec. Boissier & Heldreich (1846) ≡ *Armeriastrum glumaceum* (Boiss.) Kuntze, Revis. Gen. Pl. 2: 393. 1891 syn. sec. Kuntze (1891)
= *Acantholimon hohenackeri* Ledeb., Fl. Ross. 3: 470. 1847–1849 syn. sec. Linczevski (1952) ≡ *Statice hohenackeri* Ledeb., Fl. Ross. (Ledeb.) 3: 469. 1847–1849 syn. sec. Bunge (1872)
= *Statice ararati* Planch. in Fl. Serres Jard. Eur. 7: 95. 1851–1852 syn. sec. Linczevski (1952)
= *Acantholimon glumaceum* var. *breviscapum* Trautv. in Trudy Imp. S.-Peterburgsk. Bot. Sada 2: 582. 1873 syn. sec. Linczevski (1952)
− *Acantholimon ararati* hort. ex Jahand. in Rev. Hort. (Paris) 25: 346. 1936, pro syn. syn. sec. POWO (2017+: 6 April 2022)
− *Acantholimon glumaceum* var. *typicum* Trautv. in Trudy Imp. S.-Peterburgsk. Bot. Sada 4: 180. 1876, nom. inval. syn. sec. Linczevski (1952)

***Acantholimon glutinosum* Rech.f. & Köie in Biol. Skr. 13(4) (Symb. Afgan. 5): 166. 1963.** Sec. Rechinger & Schiman-Czeika (1974)

***Acantholimon goeksunicum* Doğan & Akaydın in Ann. Bot. Fenn. 40(1): 57. 2003.** Sec. Doğan & Akaydın (2007)

***Acantholimon gontscharovii* Czerniak. in Trudy Bot. Sada Akad. Nauk S.S.S.R. 44: 95. 1931.** Sec. Linczevski (1952)

***Acantholimon gorganense* Mobayen, Revis. Taxon. Acanth.: 306. 1964.** Sec. Rechinger & Schiman-Czeika (1974)

***Acantholimon gracillimum* Rech.f. & Schiman-Czeika, Fl. Iran. 108: 88. 1974.** Sec. Rechinger & Schiman-Czeika (1974)

***Acantholimon graecum* F.K.Mey. in Haussknechtia, Mitt. Thüring. Bot. Ges. 3: 20. 1987.** Sec. Domina (2011+)

***Acantholimon grammophyllum* Rech.f. & Köie in Biol. Skr. 13(4) (Symb. Afgan. 5): 180. 1963.** Sec. Rechinger & Schiman-Czeika (1974)

***Acantholimon griffithianum* Boiss. in Candolle, Prodr. 12: 623. 1848.** Sec. Bunge (1872)

≡ *Armeriastrum griffithianum* (Boiss.) Kuntze, Revis. Gen. Pl. 2: 393. 1891 syn. sec. Kuntze (1891)

***Acantholimon gulistanum* Bunge in Mém. Acad. Imp. Sci. Saint Pétersbourg, Sér. 7 18(2): 67. 1872.** Sec. Rechinger & Schiman-Czeika (1974)

≡ *Armeriastrum gulistanum* (Bunge) Kuntze, Revis. Gen. Pl. 2: 393. 1891 syn. sec. Kuntze (1891)
= *Acantholimon gulistanum* var. *glabrescens* Mobayen, Revis. Taxon. Acanth.: 281. 1964 syn. sec. Rechinger & Schiman-Czeika (1974)

***Acantholimon haesarense* Bornm. ex Rech.f. & Schiman-Czeika, Fl. Iran. 108: 121. 1974.** Sec. Rechinger & Schiman-Czeika (1974)

***Acantholimon halophilum* Bokhari in 30: 297. 1970.** Sec. Doğan & Akaydın (2007)

***Acantholimon halophilum* var. *coloratum* Doğan & Akaydın in Bot. J. Linn. Soc. 154(3): 412. 2007.** Sec. Doğan & Akaydın (2007)

***Acantholimon halophilum* Bokhari var. *halophilum*.** Sec. Doğan & Akaydın (2007)

***Acantholimon hamadannicum* Assadi & Mahmoodi in Iran. J. Bot. 27(2): 72. 2021.** Sec. Mahmoodi & Assadi (2021)

***Acantholimon hariabense* Rech.f. & Köie in Biol. Skr. 13(4) (Symb. Afgan. 5): 170. 1963.** Sec. Rechinger & Schiman-Czeika (1974)

***Acantholimon hedinii* Ostenf. in Hedin, S. Tibet Bot. 6(3): 48. 1922.** Sec. Linczevski (1952)

= *Acantholimon diapensioides* var. *longifolium* O.Fedsch in Trudy Imp. S.-Peterburgsk. Bot. Sada 21(1): 407. 1903 syn. sec. Linczevski (1952)

***Acantholimon heratense* Bunge in Mém. Acad. Imp. Sci. Saint Pétersbourg, Sér. 7 18(2): 55. 1872.** Sec. Bunge (1872)

≡ *Armeriastrum heratense* (Bunge) Kuntze, Revis. Gen. Pl. 2: 393. 1891 syn. sec. Kuntze (1891)

***Acantholimon heweri* Rech.f. & Schiman-Czeika, Fl. Iran. 108: 152. 1974.** Sec. Rechinger & Schiman-Czeika (1974)

***Acantholimon hilariae* Ikonn. in Bot. Mater. Gerb. Bot. Inst. Komarova Akad. Nauk S.S.S.R. 22: 12. 1963.** Sec. Rechinger & Schiman-Czeika (1974)

***Acantholimon hindukushum* Mobayen, Revis. Taxon. Acanth.: 308. 1964.** Sec. Rechinger & Schiman-Czeika (1974)

***Acantholimon hissaricum* Lincz. in Schischkin & Bobrov, Flora URSS 18: 736. 1952.** Sec. Linczevski (1952)

***Acantholimon hohenackeri* (Jaub. & Spach) Boiss. in Boissier, Diagn. Pl. Orient. ser. 1, 7: 75. 1846.** Sec. Linczevski (1952)

≡ *Statice hohenackeri* Jaub. & Spach, Ill. Pl. Orient. 1(10): 162, 167, t. 92. 1844 syn. sec. Boissier & Heldreich (1846)
– *Statice echinus* sensu Hohen. (1838), non L., err. sec. Malekmohammadi & al. (2024)
– *Statice echinus* sensu Marschall von Bieberstein (1808), non L., err. sec. Malekmohammadi & al. (2024)
= *Statice juniperifolia* Pall. ex Steud., Nomencl. Bot., ed. 2, 2: 633. 1841 syn. sec. Malekmohammadi & al. (2024: 8 April 2022)
= *Statice aciphylla* Jaub. & Spach, Ill. Pl. Orient. 1(10): 162. 1844 syn. sec. Bunge (1872)
= *Statice horrida* Girard in Ann. Sci. Nat., Bot., ser. 3, 2: 331. 1844 syn. sec. Bunge (1872)
= *Statice tenuifolia* Jaub. & Spach, Ill. Pl. Orient. 1(10): 162. 1844 syn. sec. Bunge (1872) ≡ *Armeriastrum tenuifolium* (Jaub. & Spach) Kuntze, Revis. Gen. Pl. 2: 393. 1891 syn. sec. Kuntze (1891)

***Acantholimon homophyllum* Rech.f. & Schiman-Czeika, Fl. Iran. 108: 56. 1974.** Sec. Rechinger & Schiman-Czeika (1974)

***Acantholimon hormozganense* Assadi in Iran. J. Bot. 10(2): 154. 2004.** Sec. Assadi (2004)

***Acantholimon horridum* Bunge in Mém. Acad. Imp. Sci. Saint Pétersbourg, Sér. 7 18(2): 64. 1872.** Sec. Rechinger & Schiman-Czeika (1974)

≡ *Armeriastrum horridum* (Bunge) Kuntze, Revis. Gen. Pl. 2: 393. 1891 syn. sec. Kuntze (1891)

***Acantholimon hoshapicum* Doğan & Akaydın in Bot. J. Linn. Soc. 144(4): 501. 2004.** Sec. Doğan & Akaydın (2007)

***Acantholimon huetii* Boiss., Diagn. Pl. Orient. ser. 2, 4: 65. 1859.** Sec. Bunge (1872)

≡ *Armeriastrum huetii* (Boiss.) Kuntze, Revis. Gen. Pl. 2: 393. 1891 syn. sec. Kuntze (1891)

***Acantholimon huetii* var. *breviscapum* Akaydın & Dogan in Bot. J. Linn. Soc. 154(3): 404. 2007.** Sec. Doğan & Akaydın (2007)

***Acantholimon huetii* Boiss. var. *huetii*.** Sec. Doğan & Akaydın (2007)

***Acantholimon hyalinum* Rech.f. & Köie in Biol. Skr. 13(4) (Symb. Afgan. 5): 168. 1963.** Sec. Rechinger & Schiman-Czeika (1974)

***Acantholimon hypochaerum* Bokhari in Notes Roy. Bot. Gard. Edinburgh 32(1): 74. 1972.** Sec. Bokhari & Edmondson (1982)

= *Acantholimon hypochoerum* var. *erythraeum* Mobayen, Revis. Taxon. Acanth.: 197. 1964 syn. sec. Malekmohammadi & al. (2024)
− *Acantholimon hypochoerum* Mobayen, Revis. Taxon. Acanth.: 303. 1964, nom. inval. syn. sec. Bokhari & Edmondson (1982)
− *Acantholimon sintenisii* Hausskn., nom. inval. syn. sec. Bokhari & Edmondson (1982)

***Acantholimon hystrix* Stapf in Denkschr. Acad. Wien Kl. 1: 71. 1885.** Sec. Rechinger & Schiman-Czeika (1974)

***Acantholimon ibrahimii* Akaydın in Phytotaxa 340(1): 49. 2018.** Sec. Akaydın (2018)

***Acantholimon iconicum* Boiss. & Heldr. ex Boiss., Fl. Orient. 4(2): 839. 1879.** Sec. Doğan & Akaydın (2007)

≡ *Armeriastrum iconicum* (Boiss. & Heldr.) Kuntze, Revis. Gen. Pl. 2: 394. 1891 syn. sec. Kuntze (1891)
= *Acantholimon kotschyi* var. *iconicum* Boiss. in Candolle, Prodr. 12: 628. 1848 syn. sec. Doğan & Akaydın (2007)

***Acantholimon incomptum* Boiss. & Buhse in Nouv. Mém. Soc. Imp. Naturalistes Moscou 12: 183. 1860.** Sec. Malekmohammadi & al. (2024)

≡ *Acantholimon scorpius* var. *incomptum* (Boiss. & Buhse) Boiss., Fl. Orient. 4(1): 183. 1875 syn. sec. Malekmohammadi & al. (2024) ≡ *Armeriastrum incomptum* (Boiss. & Buhse) Kuntze, Revis. Gen. Pl. 2: 393. 1891 syn. sec. Kuntze (1891)

***Acantholimon inerme* Rech.f. & Köie in Biol. Skr. 13(4) (Symb. Afgan. 5): 151. 1963.** Sec. Rechinger & Schiman-Czeika (1974)

***Acantholimon jarmilae* Halda in Acta Mus. Richnov., Sect. Nat., 7(2): 71. 2000.** Sec. Malekmohammadi & al. (2024)

***Acantholimon kandaharense* Rech.f. in Biol. Skr. 13(4) (Symb. Afgan. 5): 166. 1963.** Sec. Rechinger & Schiman-Czeika (1974)

***Acantholimon karabajeviorum* Lazkov in Turczaninowia 14(1): 5. 2011.** Sec. Malekmohammadi & al. (2024)

***Acantholimon karadarjense* Lincz. in Novosti Sist. Vyssh. Rast. 28: 129. 1991.** Sec. Malekmohammadi & al. (2024)

***Acantholimon karamanicum* Akaydın & Dogan in Israel J. Pl. Sci. 50(1): 68. 2002.** Sec. Doğan & Akaydın (2007)

***Acantholimon karatavicum* Pavlov in Vestnik Akad. Nauk Kazak. SSR 1949(1): 35. 1949.** Sec. Linczevski (1952)

***Acantholimon karelinii* (Stschegl.) Bunge in Mém. Acad. Imp. Sci. Saint Pétersbourg, Sér. 7 18(2): 58. 1872.** Sec. Linczevski (1952)

≡ *Statice karelinii* Stschegl. in Bull. Soc. Imp. Naturalistes Moscou 24(2): 475. 1851 syn. sec. Linczevski (1952) ≡ *Armeriastrum karelinii* (Bunge) Kuntze, Revis. Gen. Pl. 2: 393. 1891 syn. sec. Kuntze (1891)
= *Acantholimon szovitzii* Boiss. & Buhse in Nouv. Mém. Soc. Imp. Naturalistes Moscou 12: 184. 1860 syn. sec. Linczevski (1952)

***Acantholimon kaschgaricum* Lincz. in Novosti Sist. Vyssh. Rast. 17: 209. 1980.** Sec. Linczevski & N.I.Akshigitova (1980)

***Acantholimon katrantavicum* Lincz. in Bot. Mater. Gerb. Bot. Inst. Komarova Akad. Nauk S.S.S.R. 21: 493. 1961.** Sec. Linczevski (1961)

***Acantholimon kermanense* Assadi & Mirtadz. in Iran. J. Bot. 11(2): 130. 2006.** Sec. Assadi & Mirtadzadidni (2006)

***Acantholimon khorassanicum* Czerniak. in Trudy Bot. Sada Akad. Nauk S.S.S.R. 44: 96. 1931.** Sec. Rechinger & Schiman-Czeika (1974)

≡ *Acantholimon avenaceum* var. *khorassanicum* (Czerniak.) Mobayen, Revis. Taxon. Acanth.: 151. 1964 syn. sec. Malekmohammadi & al. (2024)
= *Acantholimon khorassanicum* var. *kopetdaghense* Czerniak. in Trudy Bot. Sada Akad. Nauk S.S.S.R. 44: 97. 1931 syn. sec. Malekmohammadi & al. (2024)

***Acantholimon kjurendaghi* Mesczer. in Izv. Akad. Nauk Turkmensk SSR, Ser. Biol. Nauk, 4: 80. 1968.** Sec. Memariani & al. (2016)

Notes. – There are two names with same scpecies epithet and different author names in Tropicos as *Acantholimon kjurendaghi*. The author name abbreviation as Meszer. is not correct.

***Acantholimon knorringianum* Lincz. in Schischkin & Bobrov, Flora URSS 18: 740. 1952.** Sec. Linczevski (1952)

***Acantholimon koeiei* Rech.f. & Schiman-Czeika, Fl. Iran. 108: 67. 1974.** Sec. Rechinger & Schiman-Czeika (1974)

***Acantholimon koelzii* Rech.f. & Köie in Biol. Skr. 13(4) (Symb. Afgan. 5): 157. 1963.** Sec. Rechinger & Schiman-Czeika (1974)

***Acantholimon koeycegizicum* Doğan & Akaydın in Ann. Bot. Fenn. 40(1): 54. 2003.** Sec. Doğan & Akaydın (2007)

***Acantholimon kokandense* Bunge in Trudy Imp. S.-Peterburgsk. Bot. Sada 3: 99. 1875.** Sec. Linczevski (1952)

***Acantholimon komarovii* Czerniak., Flora URSS 18: 741. 1952.** Sec. Linczevski (1952)

***Acantholimon korolkovii* (Regel) Korovin, Fl. URSS 18: 363. 1952.** Sec. Linczevski (1952)

≡ *Acantholimon alatavicum* var. *korolkowi* Regel in Trudy Imp. S.-Peterburgsk. Bot. Sada 6(2): 390. 1880 syn. sec. Linczevski (1952)

***Acantholimon korovinii* Czerniak. in Trudy Imp. S.-Peterburgsk. Bot. Sada 44: 94. 1931 [as " *korovini* "].** Sec. Linczevski (1952)

− *Acantholimon korovini* Czerniak. in Trudy Imp. S.-Peterburgsk. Bot. Sada 44: 94. 1931, orth. var. syn. sec. Malekmohammadi & al. (2024)

***Acantholimon kotschyi* (Jaub. & Spach) Boiss. in Boissier, Diagn. Pl. Orient. ser. 1, 7: 74. 1846.** Sec. Doğan & Akaydın (2007)

≡ *Statice kotschyi* Jaub. & Spach, Ill. Pl. Orient. 1: 162. 1844 syn. sec. Boissier (1846) ≡ *Armeriastrum kotschyi* (Boiss.) Kuntze, Revis. Gen. Pl. 2: 393. 1891 syn. sec. Kuntze (1891) ≡ *Acantholimon kotschyi* subsp. *kotschyi* syn. sec. Bokhari & Edmondson (1982)
= *Acantholimon kotschyi* var. *libanoticum* Boiss. in Candolle, Prodr. 12: 628. 1848 syn. sec. Malekmohammadi & al. (2024)
= *Acantholimon kotschyi* var. *cataonicum* Bunge in Mém. Acad. Imp. Sci. Saint Pétersbourg, Sér. 7 18(2): 35. 1872 syn. sec. Malekmohammadi & al. (2024)
= *Acantholimon breviscapum* Boiss. & Hausskn. ex Boiss., Fl. Orient. 4(2): 840. 1879 syn. sec. Bunge (1872)
= *Acantholimon kotschyi* subsp. *laxispicatum* Bokhari in 30: 299. 1970 syn. sec. Domina (2011+)

***Acantholimon kuramense* Lincz., Fl. Tadzhikskoĭ SSR 8: 480, 16. 1986.** Sec. Linczevski (1986)

***Acantholimon laevigatum* (T.X.Peng) Kamelin in Novon 3(3): 261. 1993.** Sec. Malekmohammadi & al. (2024)

≡ *Acantholimon alatavicum* var. *laevigatum* T.X.Peng in Guihaia 3(4): 291. 1983 syn. sec. Malekmohammadi & al. (2024)

***Acantholimon langaricum* O.Fedtsch. & B.Fedtsch. in Bot. Zhurn. (St. Petersburg) 1906: 194. 1907.** Sec. Linczevski (1952)

***Acantholimon latifolium* Boiss., Diagn. Pl. Orient. ser. 2, 4: 61. 1859.** Sec. Bunge (1872)

≡ *Armeriastrum latifolium* (Boiss.) Kuntze, Revis. Gen. Pl. 2: 393. 1891 syn. sec. Kuntze (1891)

***Acantholimon laxiflorum* Boiss. ex Bunge in Mém. Acad. Imp. Sci. Saint Pétersbourg, Sér. 7 18(2): 28. 1872.** Sec. Doğan & Akaydın (2007)

≡ *Armeriastrum laxiflorum* (Boiss.) Kuntze, Revis. Gen. Pl. 2: 393. 1891 syn. sec. Kuntze (1891) ≡ *Acantholimon venustum* var. *laxiflorum* (Boiss. ex Bunge) Bokhari in Notes Roy. Bot. Gard. Edinburgh 32(1): 70. 1972 syn. sec. Doğan & Akaydın (2007)
− *Acantholimon laxiflorum* Boiss., Fl. Orient. 4(2): 832. 1879, nom. inval. syn. sec. Malekmohammadi & al. (2024)

***Acantholimon laxiusculum* F.O.Khass. & I.I.Malzev in Uzb. Biol. Zhurn. 1990(5): 64. 1990.** Sec. Malekmohammadi & al. (2024)

***Acantholimon laxum* Czerniak. in Bot. Mater. Gerb. Glavn. Bot. Sada R.S.F.S.R. 4: 67. 1924.** Sec. Linczevski (1952)

= *Acantholimon talassicum* Korovin in Trudy Turkestansk. Naucn. Obsc. I: 83. 1923 syn. sec. Linczevski (1952)

***Acantholimon lepturoides* (Jaub. & Spach) Boiss. in Boissier, Diagn. Pl. Orient. ser. 1, 7: 77. 1846.** Sec. Bunge (1872)

≡ *Statice lepturoides* Jaub. & Spach, Ill. Pl. Orient. 1: 163. 1844 syn. sec. Boissier & Heldreich (1846) ≡ *Armeriastrum lepturoides* (Boiss.) Kuntze, Revis. Gen. Pl. 2: 393. 1891 syn. sec. Kuntze (1891)

***Acantholimon leucacanthum* (Jaub. & Spach) Boiss. in Boissier, Diagn. Pl. Orient. ser. 1, 7: 81. 1846.** Sec. Bunge (1872)

≡ *Statice leucacantha* Jaub. & Spach, Ill. Pl. Orient. 1(10): 164. 1844 syn. sec. Boissier (1846) ≡ *Armeriastrum leucacanthum* (Boiss.) Kuntze, Revis. Gen. Pl. 2: 393. 1891 syn. sec. Kuntze (1891)
= *Acantholimon scorpius* var. *leucacanthum* Boiss., Fl. Orient. 4(2): 850. 1879 syn. sec. Rechinger & Schiman-Czeika (1974)
= *Acantholimon leucacanthum* var. *orshanum* Mobayen, Revis. Taxon. Acanth.: 271. 1964 syn. sec. Rechinger & Schiman-Czeika (1974)

***Acantholimon leucochlorum* Rech.f. & Schiman-Czeika, Fl. Iran. 108: 106. 1974.** Sec. Rechinger & Schiman-Czeika (1974)

***Acantholimon libanoticum* Boiss. in Candolle, Prodr. 12: 630. 1848.** Sec. Bunge (1872)

≡ *Armeriastrum libanoticum* (Boiss.) Kuntze, Revis. Gen. Pl. 2: 393. 1891 syn. sec. Kuntze (1891)
= *Acantholimon caryophyllaceum* var. *brachystachyum* Boiss. in Boissier, Diagn. Pl. Orient. ser. 1, 7: 79. 1846 syn. sec. Bunge (1872)

***Acantholimon linczevskianum* Lazkov in Novosti Sist. Vyssh. Rast. 36: 38. 2004.** Sec. Malekmohammadi & al. (2024)

***Acantholimon linczevskii* Pavlov in Vestnik Akad. Nauk Kazak. SSR No. 8(113): 133. 1954.** Sec. Malekmohammadi & al. (2024)

Acantholimon litvinovii **Lincz. in Bot. Mater. Gerb. Bot. Inst. Komarova Akad. Nauk S.S.S.R. 21: 496. 1961.** Sec. Linczevski (1961)

Acantholimon longiflorum **Boiss., Diagn. Pl. Orient. ser. 2, 4: 62. 1859.** Sec. Bunge (1872)

≡ *Armeriastrum longiflorum* (Boiss.) Kuntze, Revis. Gen. Pl. 2: 394. 1891 syn. sec. Kuntze (1891)

Acantholimon longiscapum **Bokhari in Notes Roy. Bot. Gard. Edinburgh 31(2): 346. 1972.** Sec. Bokhari (1972)

Acantholimon lycaonicum **Boiss. & Heldr. in Candolle, Prodr. 12 12: 627. 1848.** Sec. Bunge (1872)

≡ *Armeriastrum lycaonicum* (Boiss. & Heldr.) Kuntze, Revis. Gen. Pl. 2: 394. 1891 syn. sec. Kuntze (1891) ≡ *Acantholimon androsaceum* subsp. *lycaonicum* (Boiss. & Heldr.) Bokhari in 30: 299. 1970 syn. sec. Domina (2011+) ≡ *Acantholimon ulicinum* subsp. *lycaonicum* (Boiss. & Heldr.) Bokhari & J.R.Edm., Fl. Turkey 7: 498. 1983 syn. sec. Domina (2011+) ≡ *Acantholimon echinus* subsp. *lycaonicum* (Boiss. & Heldr.) Papan. & Kokkini in Strid, Mount. Fl. Gr. 1: 748. 1986 syn. sec. Domina (2011+)
= *Acantholimon lycaonicum* var. *cataonicum* (Bunge) Mobayen, Revis. Taxon. Acanth.: 211. 1964 syn. sec. Malekmohammadi & al. (2024)

Acantholimon lycaonicum **subsp. *cappadocicum* Doğan & Akaydın in Bot. J. Linn. Soc. 154(3): 412. 2007.** Sec. Doğan & Akaydın (2007)

Acantholimon lycaonicum **Boiss. & Heldr. subsp. *lycaonicum*.** Sec. Doğan & Akaydın (2007)

Acantholimon lycopodioides **(Girard) Boiss. in Candolle, Prodr. 12: 632. 1848.** Sec. Linczevski (1952)

≡ *Statice lycopodioides* Girard in Ann. Sci. Nat., Bot., sér. 3, 2: 330. 1844 syn. sec. Boissier (1848) ≡ *Armeriastrum lycopodioides* (Girard) Kuntze, Revis. Gen. Pl. 2: 393. 1891 syn. sec. Kuntze (1891)
= *Acantholimon tibeticum* Hook.f. & Thomson ex C.B.Clarke, Fl. Brit. India 3(9): 479. 1882 syn. sec. Bunge (1872)

Acantholimon macranthum **Rech.f. & Köie in Biol. Skr. 13(4) (Symb. Afgan. 5): 171. 1963.** Sec. Rechinger & Schiman-Czeika (1974)

= *Acantholimon macranthum* subsp. *dubium* Rech.f. & Köie in Biol. Skr. 13(4) (Symb. Afgan. 5): 173. 1963 syn. sec. Rechinger & Schiman-Czeika (1974)

Acantholimon macropetalum **Rech.f. & Schiman-Czeika, Fl. Iran. 108: 66. 1974.** Sec. Rechinger & Schiman-Czeika (1974)

Acantholimon macrostachyum **Rech.f. & Schiman-Czeika, Fl. Iran. 108: 73. 1974.** Sec. Rechinger & Schiman-Czeika (1974)

Acantholimon majewianum **Regel in Trudy Imp. S.-Peterburgsk. Bot. Sada 6: 391. 1879.** Sec. Linczevski (1952)

− *Acantholimon maewskianum* Regel in Trudy Imp. S.-Peterburgsk. Bot. Sada 6: 391. 1879, orth. var. syn. sec. Malekmohammadi & al. (2024) − *Acantholimon majewianum* O.Fedtsch. & B.Fedtsch., Consp. Fl. Turkestanicae 5: 189. 1913, nom. inval. syn. sec. Linczevski (1952: 250, as "Majevianum")
= *Acantholimon bobrovii* Czerniak. in Trudy Bot. Sada Akad. Nauk S.S.S.R. 44: 102. 1931 syn. sec. Linczevski (1952)

Acantholimon manakyanii **Ogan. in Takhtajania 1: 56. 2011.** Sec. Oganesian (2011)

Acantholimon margaritae **Korovin, Fl. URSS 18: 735. 1952.** Sec. Linczevski (1952)

Acantholimon melananthum **(Boiss.) Boiss. in Boissier, Diagn. Pl. Orient. ser. 1, 7: 74. 1846.** Sec. Bunge (1872)

≡ *Armeriastrum melananthum* (Boiss.) Kuntze, Revis. Gen. Pl. 2: 394. 1891 syn. sec. Kuntze (1891) − *Statice melanantha* Boiss., Pl. Pers. Aust. Exsic.: 2. 1845, nom. nud. syn. sec. Boissier (1846)

Acantholimon mikeschinii **Lincz. in Schischkin & Bobrov, Flora URSS 18: 733. 1952.** Sec. Linczevski (1952)

Acantholimon minshelkense **Pavlov in Vestnik Akad. Nauk Kazak. SSR 1949(1): 35. 1950.** Sec. Linczevski (1952)

Acantholimon mirtadzadinii **Assadi in Iran. J. Bot. 10(1): 28. 2003.** Sec. Assadi (2005)

Acantholimon mishaudaghense **Mobayen, Revis. Taxon. Acanth.: 303. 1964.** Sec. Rechinger & Schiman-Czeika (1974)

Acantholimon mobayenii **Assadi & Ghahr., Fl. Iran 51: 113. 2005.** Sec. Assadi (2005)

Acantholimon modestum **Bornm. ex Rech.f. & Schiman-Czeika, Fl. Iran. 108: 140. 1974.** Sec. Rechinger & Schiman-Czeika (1974)

Acantholimon moradii **Assadi in Phytotaxa 227(1): 99. 2015.** Sec. Mahmoodi & al. (2015)

− *Acantholimon moradii* Assadi in Iran. J. Bot. 11(1): 37. 2005, nom. inval. syn. sec. Mahmoodi & al. (2015)

Acantholimon muchamedshanovii **Lincz. in Bot. Mater. Gerb. Bot. Inst. Komarova Akad. Nauk S.S.S.R. 21: 485. 1961.** Sec. Linczevski (1961)

Acantholimon multiflorum **(Bokhari) Doğan & Akaydın in Bot. J. Linn. Soc. 154(3): 414. 2007.** Sec. Doğan & Akaydın (2007)

≡ *Acantholimon spirizianum* var. *multiflorum* Bokhari in Notes Roy. Bot. Gard. Edinburgh 32(1): 70. 1972 syn. sec. Doğan & Akaydın (2007)

Acantholimon muradicum **O.Schwarz & F.K.Mey. in Haussknechtia, Mitt. Thüring. Bot. Ges. 3: 35. 1987.** Sec. Domina (2011+)

Acantholimon nabievii **Lincz. in Bot. Mater. Gerb. Bot. Inst. Komarova Akad. Nauk S.S.S.R. 21: 495. 1961.** Sec. Linczevski (1961)

Acantholimon narynense **Lazkov in Bot. Zhurn. (Moscow & Leningrad) 90(12): 1895. 2005.** Sec. Malekmohammadi & al. (2024)

Acantholimon nawaricum **Rech.f. & Schiman-Czeika, Fl. Iran. 108: 109. 1974.** Sec. Rechinger & Schiman-Czeika (1974)

Acantholimon nigricans **Mobayen, Revis. Taxon. Acanth.: 300. 1964.** Sec. Rechinger & Schiman-Czeika (1974)

Acantholimon nikitinii **Lincz. in Schischkin & Bobrov, Flora URSS 18: 742. 1952.** Sec. Linczevski (1952)

Acantholimon nuratavicum **Zakirov, Fl. URSS 18: 736. 1952.** Sec. Linczevski (1952)

Acantholimon oliganthum **Boiss. in Boissier, Diagn. Pl. Orient. ser. 1, 7: 77. 1846.** Sec. Bunge (1872)

≡ *Armeriastrum oliganthum* (Boiss.) Kuntze, Revis. Gen. Pl. 2: 394. 1891 syn. sec. Kuntze (1891) − *Statice oligantha* Boiss., Pl. Pers. Aust. Exsic.: 1845, nom. nud., pro syn. syn. sec. Boissier (1846)

Notes. – *Statice oligantha* Boiss. nom. nud. is an invalid designation listed on top of the map of mount “Kuh Delu” in the illustration part of the Plantarum Persiae Australis (Boissier, 1845) and later listed as a synonym of *Acantholimon oliganthum* Boiss. (Boissier, 1846).

Acantholimon olivieri **(Jaub. & Spach) Boiss. in Boissier, Diagn. Pl. Orient. ser. 1, 7: 80. 1846.** Sec. Rechinger & Schiman-Czeika (1974)

≡ *Statice olivieri* Jaub. & Spach, Ill. Pl. Orient. 1(10): 163, 168, t. 93. 1844 syn. sec. Boissier & Heldreich (1846) ≡ *Armeriastrum olivieri* (Boiss.) Kuntze, Revis. Gen. Pl. 2: 394. 1891 syn. sec. Kuntze (1891)
= *Acantholimon venustum* var. *olivieri* Boiss., Fl. Orient. 4(2): 832. 1879 syn. sec. Malekmohammadi & al. (2024)

Acantholimon ophiocladum **Rech.f. & Schiman-Czeika, Fl. Iran. 108: 126. 1974.** Sec. Rechinger & Schiman-Czeika (1974)

Acantholimon pamiricum **Czerniak., Flora URSS 18: 738. 1952.** Sec. Rechinger & Schiman-Czeika (1974)

= *Acantholimon alatavicum* O.Fedtsch. & B.Fedtsch., Consp. Fl. Turkestanicae 5: 190. 1913 syn. sec. Rechinger & Schiman-Czeika (1974)

Acantholimon parviflorum **Regel, Descr. Pl. Nov. Rar. Fedtsch.: 73. 1882.** Sec. Linczevski (1952)

Acantholimon pavlovii **Lincz. in Novosti Sist. Vyssh. Rast. 17: 208. 1980.** Sec. Linczevski & N.I.Akshigitova (1980)

Acantholimon peculiare **Rech.f. in Oesterr. Bot. Z. 95: 426. 1949.** Sec. Rechinger & Schiman-Czeika (1974)

Acantholimon petraeum **Boiss. ex Bunge in Mém. Acad. Imp. Sci. Saint Pétersbourg, Sér. 7 18(2): 28. 1872.** Sec. Bunge (1872)

≡ *Armeriastrum petraeum* (Boiss. ex Bunge) Kuntze, Revis. Gen. Pl. 2: 394. 1891 syn. sec. Kuntze (1891)

Acantholimon petuniiflorum **Mobayen, Revis. Taxon. Acanth.: 305. 1964.** Sec. Rechinger & Schiman-Czeika (1974)

Acantholimon physostegium **Rech.f. & Schiman-Czeika, Fl. Iran. 108: 77. 1974.** Sec. Rechinger & Schiman-Czeika (1974)

Acantholimon podlechii **Rech.f. & Schiman-Czeika, Fl. Iran. 108: 124. 1974.** Sec. Rechinger & Schiman-Czeika (1974)

Acantholimon poliochlorum **Rech.f. & Schiman-Czeika, Fl. Iran. 108: 51. 1974.** Sec. Rechinger & Schiman-Czeika (1974)

Acantholimon polystachyum **Boiss. in Candolle, Prodr. 12: 624. 1848.** Sec. Bunge (1872)

≡ *Armeriastrum polystachyum* (Boiss.) Kuntze, Revis. Gen. Pl. 2: 394. 1891 syn. sec. Kuntze (1891)

Acantholimon popovii **Czerniak. in Trudy Bot. Inst. Akad. Nauk S.S.S.R., ser. 1, Fl. Sist. Vyssh. Rast. 3: 264. 1937.** Sec. Malekmohammadi & al. (2024)

Acantholimon procumbens **Czerniak. in Trudy Bot. Sada Akad. Nauk S.S.S.R. 44: 97. 1931.** Sec. Linczevski (1952)

Acantholimon pskemense **Lincz. in Schischkin & Bobrov, Flora URSS 18: 734. 1952.** Sec. Linczevski (1952)

Acantholimon pterostegium **Bunge in Mém. Acad. Imp. Sci. Saint Pétersbourg, Sér. 7 18(2): 15. 1872.** Sec. Linczevski (1952)

≡ *Armeriastrum pterostegium* (Bunge) Kuntze, Revis. Gen. Pl. 2: 394. 1891 syn. sec. Kuntze (1891)

Acantholimon puberulum **Boiss. & Balansa in Boissier, Diagn. Pl. Orient. ser. 2, 4: 62. 1859.** Sec. Bunge (1872)

≡ *Armeriastrum puberulum* (Boiss. & Balansa) Kuntze, Revis. Gen. Pl. 2: 394. 1891 syn. sec. Kuntze (1891) ≡ *Acantholimon echinus* var. *puberulum* Boiss., Fl. Orient. 4(2): 841. 1879 syn. sec. Malekmohammadi & al. (2024: 5 April 2022)
= *Acantholimon cataonicum* Bunge in Mém. Acad. Imp. Sci. Saint Pétersbourg, Sér. 7 18(2): 43. 1872 syn. sec. Bokhari & Edmondson (1982: page 496) ≡ *Armeriastrum cataonicum* (Bunge) Kuntze, Revis. Gen. Pl. 2: 393. 1891 syn. sec. Malekmohammadi & al. (2024)
= *Acantholimon puberulum* var. *glabrum* Bokhari in 30: 300. 1970 syn. sec. Doğan & Akaydın (2007)

Acantholimon puberulum **subsp. *longiscapum* (Bokhari) Doğan & Akaydın in Bot. J. Linn. Soc. 154(3): 408. 2007.** Sec. Doğan & Akaydın (2007)

≡ *Acantholimon puberulum* var. *longiscapum* Bokhari in 30: 300. 1970 syn. sec. Doğan & Akaydın (2007)

Acantholimon puberulum **subsp. *peronini* (Boiss.) Akaydın & Dogan in Bot. J. Linn. Soc. 154(3): 408. 2007 [as " *peroninii* "].** Sec. Doğan & Akaydın (2007)

≡ *Acantholimon peronini* Boiss., Fl. Orient. 4(2): 842. 1879 syn. sec. Doğan & Akaydın (2007) ≡ *Armeriastrum peronini* (Boiss.) Kuntze, Revis. Gen. Pl. 2: 394. 1891 [as " *peroninii* "] syn. sec. Kuntze (1891) − *Acantholimon peroninii* Boiss., orth. var. syn. sec. Doğan & Akaydın (2007) − *Armeriastrum peroninii* (Boiss.) Kuntze, orth. var. syn. sec. Doğan & Akaydın (2007) − *Acantholimon puberulum* subsp. *peroninii* (Boiss.) Akaydın & Dogan, orth. var. syn. sec. Doğan & Akaydın (2007)

***Acantholimon puberulum* Boiss. & Balansa subsp. *puberulum*.** Sec. Doğan & Akaydın (2007)

***Acantholimon pulchellum* Korovin in Bot. Mater. Gerb. Glavn. Bot. Sada R.S.F.S.R. 3: 191. 1922.** Sec. Linczevski (1952)

= *Acantholimon kutschanense* Rech.f. in Oesterr. Bot. Z. 95: 424. 1949 syn. sec. Linczevski (1952)

***Acantholimon purpureum* Korovin in Trudy Turkestansk. Naucn. Obsc. I: 81. 1923.** Sec. Linczevski (1952)

***Acantholimon quettense* Rech.f. & Schiman-Czeika, Fl. Iran. 108: 104. 1974.** Sec. Rechinger & Schiman-Czeika (1974)

− *Acantholimon quettensis* Rech.f. & Schiman-Czeika, orth. var. syn. sec. Brenan (1981) [is orthographic variant for *Acantholimon quettense* Rech.f. & Schiman-Czeika]

***Acantholimon quinquelobum* Bunge in Mém. Acad. Imp. Sci. Saint Pétersbourg, Sér. 7 18(2): 56. 1872.** Sec. Doğan & Akaydın (2007)

≡ *Armeriastrum quinquelobum* (Bunge) Kuntze, Revis. Gen. Pl. 2: 394. 1891 syn. sec. Kuntze (1891)

***Acantholimon quinquelobum* var. *curviflorum* (Bunge) Doğan & Akaydın in Bot. J. Linn. Soc. 154(3): 402. 2007.** Sec. Doğan & Akaydın (2007)

≡ *Acantholimon curviflorum* Bunge in Mém. Acad. Imp. Sci. Saint Pétersbourg, Sér. 7 18(2): 57. 1872 syn. sec. Doğan & Akaydın (2007) ≡ *Armeriastrum curviflorum* (Bunge) Kuntze, Revis. Gen. Pl. 2: 393. 1891 syn. sec. Kuntze (1891)
= *Acantholimon straussii* Bornm. in Beih. Bot. Centralbl. Abt. 2, 22(2): 140. 1907 syn. sec. Rechinger & Schiman-Czeika (1974) ≡ *Acantholimon incomptum* var. *straussii* Bornm. in Beih. Bot. Centralbl. Abt. 2, 22(2): 140. 1907 syn. sec. Rechinger & Schiman-Czeika (1974)

***Acantholimon quinquelobum* Bunge var. *quinquelobum*.** Sec. Doğan & Akaydın (2007)

***Acantholimon raddeanum* Czerniak. in Feddes Repert. Spec. Nov. Regni Veg. 27: 276. 1930.** Sec. Linczevski (1952)

***Acantholimon raikoviae* Czerniak. ex Lincz., Fl. Tadzhikskoĭ SSR 8: 481, 20. 1986.** Sec. Linczevski (1986)

***Acantholimon reflexifolium* Bokhari in 30: 297. 1970.** Sec. Bokhari & Edmondson (1982)

***Acantholimon restiaceum* Bunge in Mém. Acad. Imp. Sci. Saint Pétersbourg, Sér. 7 18(2): 22. 1872.** Sec. Rechinger & Schiman-Czeika (1974)

≡ *Armeriastrum restiaceum* (Bunge) Kuntze, Revis. Gen. Pl. 2: 394. 1891 syn. sec. Kuntze (1891)
= *Acantholimon cleistocalyx* Hand.-Mazz. in Verh. Zool.-Bot. Ges. Wien 72: 33. 1923 syn. sec. Rechinger & Schiman-Czeika (1974)

***Acantholimon revolutum* Rech.f. & Köie in Biol. Skr. 13(4) (Symb. Afgan. 5): 153. 1963.** Sec. Rechinger & Schiman-Czeika (1974)

***Acantholimon rhodopolium* Rech.f. & Schiman-Czeika, Fl. Iran. 108: 141. 1974.** Sec. Rechinger & Schiman-Czeika (1974)

***Acantholimon riyatguelii* Yıldırım in Phytotaxa 175(2): 74. 2014.** Sec. Yıldırım & Crespo (2014)

***Acantholimon rubricosum* Mobayen, Revis. Taxon. Acanth.: 302. 1964.** Sec. Mobayen (1964)

***Acantholimon rudbaricum* Bornm. in Bull. Herb. Boissier, sér. 2, 8: 123. 1908.** Sec. Rechinger & Schiman-Czeika (1974)

= *Acantholimon truncatum* subsp. *rudbaricum* Bornm. in Bull. Herb. Boissier, sér. 2, 8: 123. 1908 syn. sec. Malekmohammadi & al. (2024)

***Acantholimon ruprechtii* Bunge in Mém. Acad. Imp. Sci. Saint Pétersbourg, Sér. 7 18(2): 20. 1872 [as " *ruprechti* "].** Sec. Linczevski (1952)

≡ *Acantholimon latifolium* Rupr. in Mém. Acad. Imp. Sci. St.-Pétersbourg, Sér. 7, 14(4): 69. 1869 syn. sec. Bunge (1872) ≡ *Armeriastrum ruprechtii* (Bunge) Kuntze, Revis. Gen. Pl. 2: 394. 1891 syn. sec. Kuntze (1891) − *Acantholimon ruprechti* Bunge in Mém. Acad. Imp. Sci. Saint Pétersbourg, Sér. 7, 18(2): 20. 1872, orth. var. syn. sec. Linczevski (1952)

***Acantholimon saadii* Assadi & Zeraatkar in Iran. J. Bot. 26: 2. 2020.** Sec. Assadi & Zeraatkar (2020)

***Acantholimon sackenii* Bunge in Mém. Acad. Imp. Sci. Saint Pétersbourg, Sér. 7 18(2): 68. 1872 [as " *sackeni* "].** Sec. Linczevski (1952)

− *Acantholimon sackeni* Bunge in Mém. Acad. Imp. Sci. Saint Pétersbourg, Sér. 7, 18(2): 68. 1872, orth. var. syn. sec. Malekmohammadi & al. (2024)
= *Acantholimon hohenackeri* var. *virens* Rupr. in Mém. Acad. Imp. Sci. Saint Pétersbourg, Sér. 7, 14(4): 69. 1869 syn. sec. Linczevski (1952)
= *Acantholimon desertorum* Regel in Trudy Imp. S.-Peterburgsk. Bot. Sada 6: 391. 1879 syn. sec. Linczevski (1952)

***Acantholimon sahendicum* Boiss. & Buhse in Nouv. Mém. Soc. Imp. Naturalistes Moscou 12: 183. 1860.** Sec. Bunge (1872)

≡ *Armeriastrum sahendicum* (Boiss. & Buhse) Kuntze, Revis. Gen. Pl. 2: 394. 1891 syn. sec. Kuntze (1891) ≡ *Acantholimon glumaceum* var. *sahendicum* (Boiss. & Buhse) Kusn., Fl. Caucas. Crit. 4(1): 193. 1902 syn. sec. Linczevski (1952)

***Acantholimon salangense* Bokhari in Notes Roy. Bot. Gard. Edinburgh 31(2): 346. 1972.** Sec. Rechinger & Schiman-Czeika (1974)

− *Acantholimon salangensis* Bokhari, orth. var. syn. sec. Malekmohammadi & al. (2024) [is orthographic variant for *Acantholimon salangense* Bokhari]

***Acantholimon sarawschanicum* Regel in Izv. Imp. Obshch. Lyubit. Estestv. Moskovsk. Univ. 34(2): 73. 1882.** Sec. Linczevski (1952)

− *Acantholimon saravschanicum* Regel, orth. var. syn. sec. Malekmohammadi & al. (2024) [is orthographic variant for *Acantholimon sarawschanicum* Regel]

***Acantholimon sarytavicum* Lincz. in Novosti Sist. Vyssh. Rast. 28: 130. 1991.** Sec. Malekmohammadi & al. (2024)

***Acantholimon saxifragiforme* Hausskn. & Sint. ex Bokhari in Notes Roy. Bot. Gard. Edinburgh 32(1): 74. 1972.** Sec. Bokhari & Edmondson (1982)

= *Acantholimon saxifragiforme* Hausskn. ex Mobayen, Revis. Taxon. Acanth.: 307. 1964, nom. illeg. syn. sec. Bokhari & Edmondson (1982)

***Acantholimon scabrellum* Boiss. & Hausskn. ex Boiss., Fl. Orient. 4(2): 845. 1879.** Sec. Rechinger & Schiman-Czeika (1974)

≡ *Armeriastrum scabrellum* (Boiss. & Hausskn. ex Boiss.) Kuntze syn. sec. Rechinger & Schiman-Czeika (1974)

***Acantholimon scabrellum* var. *kandilum* Mobayen, Revis. Taxon. Acanth.: 241. 1964.** Sec. Mobayen (1964)

***Acantholimon scabrellum* Boiss. & Hausskn. ex Boiss. var. *scabrellum*.** Sec. Mobayen (1964)

***Acantholimon schachimardanicum* Lincz. in Bot. Mater. Gerb. Bot. Inst. Komarova Akad. Nauk S.S.S.R. 21: 492. 1961.** Sec. Linczevski (1961)

***Acantholimon schahrudicum* Bunge in Mém. Acad. Imp. Sci. Saint Pétersbourg, Sér. 7 18(2): 65. 1872.** Sec. Bunge (1872)

≡ *Armeriastrum schahrudicum* (Bunge) Kuntze, Revis. Gen. Pl. 2: 394. 1891 syn. sec. Kuntze (1891)
− *Acantholimon shahrudicum* Bunge, orth. var. syn. sec. Malekmohammadi & al. (2024) [is misspelling for *Acantholimon schahrudicum* Bunge]

***Acantholimon schemachense* Grossh. in Trud. Geobot. Obsl. Pastb. S. S. R. Azerb., Baku (Trans. Geobot. Invest. Pasturelands, Azerb.) Ser. A. No., 7: 99. 1931.** Sec. Linczevski (1952)

***Acantholimon schirazianum* Boiss. in Boissier, Diagn. Pl. Orient. ser. 1, 7: 72. 1846.** Sec. Bunge (1872)

≡ *Armeriastrum schirazianum* (Boiss.) Kuntze, Revis. Gen. Pl. 2: 394. 1891 syn. sec. Kuntze (1891)
− *Statice erinacea* Boiss., in sched. syn. sec. Bunge (1872)
− *Statice schirasiana* Boiss., in sched. syn. sec. Malekmohammadi & al. (2024)

***Acantholimon schizostegium* Rech.f. & Schiman-Czeika, Fl. Iran. 108: 76. 1974.** Sec. Rechinger & Schiman-Czeika (1974)

***Acantholimon scirpinum* Bunge in Mém. Acad. Imp. Sci. Saint Pétersbourg, Sér. 7 18(2): 22. 1872.** Sec. Rechinger & Schiman-Czeika (1974)

≡ *Armeriastrum scirpinum* (Bunge) Kuntze, Revis. Gen. Pl. 2: 394. 1891 syn. sec. Kuntze (1891)

***Acantholimon sclerophyllum* Rech.f. & Schiman-Czeika, Fl. Iran. 108: 119. 1974.** Sec. Rechinger & Schiman-Czeika (1974)

***Acantholimon scorpius* (Jaub. & Spach) Boiss. in Boissier, Diagn. Pl. Orient. ser. 1, 7: 81. 1846.** Sec. Bunge (1872)

≡ *Statice scorpius* Jaub. & Spach, Ill. Pl. Orient. 1(10): 163. 1844 syn. sec. Boissier (1846)
= *Statice ferox* Jaub. & Spach, Ill. Pl. Orient. 1(10): 163. 1844 syn. sec. Bunge (1872) ≡ *Acantholimon ferox* (Jaub. & Spach) Boiss. in Boissier, Diagn. Pl. Orient. ser. 1, 7: 81. 1846 syn. sec. Malekmohammadi & al. (2024) ≡ *Armeriastrum ferox* (Boiss.) Kuntze, Revis. Gen. Pl. 2: 393. 1891 syn. sec. Malekmohammadi & al. (2024)
= *Statice spachii* Girard in Ann. Sci. Nat., Bot., ser. 3, 2: 322. 1844, nom. superfl. syn. sec. Bunge (1872)
= *Acantholimon scorpioideum* St.-Lag. in Ann. Soc. Bot. Lyon 7: 118. 1880 syn. sec. Malekmohammadi & al. (2024)
− *Acantholimon scorpius* var. *balutchii* Mobayen, Revis. Taxon. Acanth.: 267. 1964, nom. inval. syn. sec. Rechinger & Schiman-Czeika (1974)

***Acantholimon senganense* Bunge in Mém. Acad. Imp. Sci. Saint Pétersbourg, Sér. 7 18(2): 29. 1872.** Sec. Rechinger & Schiman-Czeika (1974)

≡ *Armeriastrum senganense* (Bunge) Kuntze, Revis. Gen. Pl. 2: 394. 1891 syn. sec. Kuntze (1891)
= *Acantholimon senganense* var. *glaucum* Parsa, Fl. Iran 4: 910. 1949 syn. sec. Malekmohammadi & al. (2024)

***Acantholimon senganense* Bunge subsp. *senganense*.** Sec. Assadi (2005)

***Acantholimon senganense* subsp. *tehranense* Assadi, Fl. Iran 51: 157. 2005.** Sec. Assadi (2005)

***Acantholimon serotinum* Rech.f. & Schiman-Czeika, Fl. Iran. 108: 96. 1974.** Sec. Rechinger & Schiman-Czeika (1974)

***Acantholimon sirchense* Assadi & Mirtadz. in Iran. J. Bot. 11(2): 134. 2006.** Sec. Assadi & Mirtadzadidni (2006)

***Acantholimon solidum* Rech.f. & Köie in Biol. Skr. 13(4) (Symb. Afgan. 5): 159. 1963.** Sec. Rechinger & Schiman-Czeika (1974)

***Acantholimon sorchense* Rech.f. in Oesterr. Bot. Z. 95: 425. 1949.** Sec. Rechinger & Schiman-Czeika (1974)

***Acantholimon speciosissimum* Aitch. & Hemsl. in Trans. Linn. Soc. London, Bot. 3(1): 85. 1888.** Sec. Rechinger & Schiman-Czeika (1974)

≡ *Gladiolimon speciosissimum* (Aitch. & Hemsl.) Mobayen, Revis. Taxon. Acanth.: 297. 1964 syn. sec. Rechinger & Schiman-Czeika (1974)

Notes. – Rechinger & Schiman-Czeika (1974) considered *Gladiolimon* as part of *Acantholimon* and the molecular phylogenetic studies by Moharrek et al. (Bot. J. Linn. Soc. 184: 2017) confirms that the monotypic *Gladiolimon* should be included in *Acantholimon*.

***Acantholimon spinicalyx* Köie & Rech.f. in Anz. Österr. Akad. Wiss., Math.-Naturwiss. Kl. 92: 274. 1956.** Sec. Rechinger & Schiman-Czeika (1974)

***Acantholimon spirizianum* Mobayen, Revis. Taxon. Acanth.: 304. 1964.** Sec. Bokhari & Edmondson (1982)

≡ *Acantholimon spirizianum* subsp. *spirizianum* syn. sec. Malekmohammadi & al. (2024)

***Acantholimon squarrosum* Pavlov in Vestnik Akad. Nauk Kazak. SSR 1949(1): 36. 1951.** Sec. Linczevski (1952)

= *Acantholimon iskanderi* Lipsky ex O.Fedtsch. & B.Fedtsch., Consp. Fl. Turkestanicae 5: 190. 1913 syn. sec. Linczevski (1952)

***Acantholimon stanjukoviczii* Lincz. ex Ikonn. in Bot. Zhurn. (Moscow & Leningrad) 86(10): 92. 2001.** Sec. Malekmohammadi & al. (2024)

***Acantholimon stapfianum* Rech.f. & Schiman-Czeika, Fl. Iran. 108: 148. 1974.** Sec. Rechinger & Schiman-Czeika (1974)

***Acantholimon stenorhaphium* Rech.f. in Biol. Skr. 13(4) (Symb. Afgan. 5): 177. 1963.** Sec. Rechinger & Schiman-Czeika (1974)

***Acantholimon stereophyllum* Rech.f. & Schiman-Czeika, Fl. Iran. 108: 37. 1974.** Sec. Rechinger & Schiman-Czeika (1974)

***Acantholimon stocksii* Boiss., Diagn. Pl. Orient. ser. 2, 4: 63. 1859.** Sec. Bunge (1872)

≡ *Armeriastrum stocksii* (Boiss.) Kuntze, Revis. Gen. Pl. 2: 394. 1891 syn. sec. Kuntze (1891)
= *Acantholimon stocksii* var. *auriculatum* Mobayen, Revis. Taxon. Acanth.: 268. 1964 syn. sec. Rechinger & Schiman-Czeika (1974)

***Acantholimon strictiforme* Nikitina ex Lazkov in Bot. Zhurn. (Moscow & Leningrad) 90(12): 1895. 2005.** Sec. Malekmohammadi & al. (2024)

***Acantholimon strictum* Czerniak., Flora URSS 18: 734. 1952.** Sec. Linczevski (1952)

***Acantholimon strigillosum* Bokhari in 30: 299. 1970.** Sec. Bokhari & Edmondson (1982)

***Acantholimon stroterophyllum* Rech.f. & Schiman-Czeika, Fl. Iran. 108: 138. 1974.** Sec. Rechinger & Schiman-Czeika (1974)

***Acantholimon subavenaceum* Lincz. in Bot. Mater. Gerb. Bot. Inst. Komarova Akad. Nauk S.S.S.R. 21: 489. 1961.** Sec. Linczevski (1961)

***Acantholimon subflavescens* Rech.f. & Schiman-Czeika, Fl. Iran. 108: 52. 1974.** Sec. Rechinger & Schiman-Czeika (1974)

***Acantholimon subsimile* Rech.f. & Schiman-Czeika, Fl. Iran. 108: 113. 1974.** Sec. Rechinger & Schiman-Czeika (1974)

***Acantholimon subulatum* Boiss. in Candolle, Prodr. 12: 629. 1848.** Sec. Bunge (1872)

≡ *Armeriastrum subulatum* (Boiss.) Kuntze, Revis. Gen. Pl. 2: 394. 1891 syn. sec. Kuntze (1891)
= *Acantholimon leptostachyum* Aitch. & Hemsl. in J. Linn. Soc., Bot. 18: 76. 1880 ["1881"] syn. sec. Rechinger & Schiman-Czeika (1974)
= *Acantholimon nuristanicum* Kitam. in Acta Phytotax. Geobot. 16(5): 133. 1956 syn. sec. POWO (2017+: 5 April 2022)

***Acantholimon takhtajanii* Ogan. in Takhtajania 1: 54. 2011.** Sec. Oganesian (2011)

***Acantholimon talagonicum* Boiss. in Candolle, Prodr. 12: 625. 1848.** Sec. Bunge (1872)

≡ *Armeriastrum talagonicum* (Boiss.) Kuntze, Revis. Gen. Pl. 2: 394. 1891 syn. sec. Kuntze (1891)
= *Acantholimon microstegium* Bornm. in Repert. Spec. Nov. Regni Veg. 8: 547. 1910 syn. sec. Rechinger & Schiman-Czeika (1974) ≡ *Acantholimon talagonicum* var. *microstegium* (Bornm.) Mobayen, Revis. Taxon. Acanth.: 75. 1964 syn. sec. Rechinger & Schiman-Czeika (1974)

***Acantholimon tarbagataicum* Gamajunova in Bot. Mater. Gerb. Bot. Inst. Komarova Akad. Nauk S.S.S.R. 13: 204. 1950.** Sec. Linczevski (1952)

***Acantholimon taschkurganicum* Lincz. & N.I.Akshigitova in Novosti Sist. Vyssh. Rast. 17: 210. 1980.** Sec. Linczevski & N.I.Akshigitova (1980)

***Acantholimon taschkurganicum* var. *escaposum* Lincz. & N.I.Akshigitova in Novosti Sist. Vyssh. Rast. 17: 210. 1980.** Sec. Linczevski & N.I.Akshigitova (1980)

***Acantholimon taschkurganicum* Lincz. & N.I.Akshigitova var. *taschkurganicum*.** Sec. Linczevski & N.I.Akshigitova (1980)

***Acantholimon tataricum* Boiss. in Candolle, Prodr. 12: 623. 1848.** Sec. Linczevski (1952)

≡ *Armeriastrum tartaricum* (Boiss.) Kuntze, Revis. Gen. Pl. 2: 394. 1891 syn. sec. Kuntze (1891)
= *Acantholimon iskanderi* Lipsky ex O.Fedtsch. & B.Fedtsch., Consp. Fl. Turkestanicae 5: 190. 1913 syn. sec. Linczevski (1952)

***Acantholimon tenuiflorum* Boiss. in Boissier, Diagn. Pl. Orient. ser. 1, 7: 78. 1846.** Sec. Bunge (1872)

≡ *Armeriastrum tenuiflorum* (Boiss.) Kuntze, Revis. Gen. Pl. 2: 394. 1891 syn. sec. Kuntze (1891)
= *Statice echinus* L., Sp. Pl.: 276. 1753 syn. sec. Bunge (1872) ≡ *Limonium echinus* (L.) Chaz. in Miller, Suppl. Dict. Jard. 2: 34. 1790 syn. sec. Malekmohammadi & al. (2024) ≡ *Acantholimon echinus* (L.) Bunge in Mém. Acad. Imp. Sci. Saint Pétersbourg, Sér. 7 18(2): 46. 1872 syn. sec. Bokhari & Edmondson (1982) ≡ *Acantholimon echinus* (L.) Boiss., Fl. Orient. 4(2): 810. 1879, nom. illeg. syn. sec. Doğan & Akaydın (2007) ≡ *Armeriastrum echinus* (L.) Kuntze, Revis. Gen. Pl. 2: 393. 1891 syn. sec. Malekmohammadi & al. (2024)

***Acantholimon termei* Rech.f. & Schiman-Czeika, Fl. Iran. 108: 120. 1974.** Sec. Rechinger & Schiman-Czeika (1974)

***Acantholimon tianschanicum* Czerniak. in Trudy Bot. Inst. Akad. Nauk S.S.S.R., ser. 1, Fl. Sist. Vyssh. Rast. 3: 262. 1937.** Sec. Linczevski (1952)

= *Acantholimon diapensioides* Herder non. Boiss. syn. sec. Linczevski 1952 in Bull. Soc. Imp. Naturalistes Moscou 41(1-2): 394. 1868 syn. sec. Linczevski (1952)

***Acantholimon titovii* Lincz. in Schischkin & Bobrov, Flora URSS 18: 740. 1952.** Sec. Linczevski (1952)

***Acantholimon tomentellum* Boiss. in Boissier, Diagn. Pl. Orient. ser. 1, 7: 72. 1846.** Sec. Bunge (1872)

≡ *Armeriastrum tomentellum* (Boiss.) Kuntze, Revis. Gen. Pl. 2: 394. 1891 syn. sec. Kuntze (1891) − *Statice tomentella* Boiss., Pl. Pers. aust.: 2. 1845, nom. nud. syn. sec. Malekmohammadi & al. (2024)

***Acantholimon tragacanthinum* (Jaub. & Spach) Boiss. in Boissier, Diagn. Pl. Orient. ser. 1, 7: 81. 1846.** Sec. Bunge (1872)

≡ *Statice tragacanthina* Jaub. & Spach, Ill. Pl. Orient. 1: 164. 1844 syn. sec. Boissier (1846) ≡ *Armeriastrum tragacanthium* (Boiss.) Kuntze, Revis. Gen. Pl. 2: 394. 1891 syn. sec. Kuntze (1891)
= *Statice spachii* Girard in Ann. Sci. Nat., Bot., ser. 3, 2: 322. 1844, nom. superfl. syn. sec. Bunge (1872)

***Acantholimon tricolor* Rech.f. & Köie in Biol. Skr. 13(4) (Symb. Afgan. 5): 162. 1963.** Sec. Rechinger & Schiman-Czeika (1974)

***Acantholimon truncatum* Bunge in Mém. Acad. Imp. Sci. Saint Pétersbourg, Sér. 7 18(2): 56. 1872.** Sec. Rechinger & Schiman-Czeika (1974)

≡ *Armeriastrum truncatum* (Bunge) Kuntze, Revis. Gen. Pl. 2: 394. 1891 syn. sec. Kuntze (1891)

***Acantholimon tulakense* Rech.f. & Schiman-Czeika, Fl. Iran. 108: 61. 1974.** Sec. Rechinger & Schiman-Czeika (1974)

− *Acantholimon tulakensis* Rech.f. & Schiman-Czeika, orth. var. syn. sec. Brenan (1981) [is orthographic variant for *Acantholimon tulakense* Rech.f. & Schiman-Czeika]

***Acantholimon turcicum* Doğan & Akaydın in Israel J. Pl. Sci. 51(3): 232. 2003.** Sec. Doğan & Akaydın (2007)

***Acantholimon ulicinum* (Willd. ex Schult.) Boiss. in Candolle, Prodr. 12: 627. 1848.** Sec. Bunge (1872)

≡ *Statice ulicina* Willd. ex Schult. in Roemer & Schultes, Syst. Veg. ed. 15[bis] 6: 798. 1820 syn. sec. Boissier (1848) ≡ *Acantholimon libanoticum* var. *ulicinum* (Willd. ex Schult.) Boiss. in Candolle, Prodr. 12: 627. 1848 syn. sec. Bokhari & Edmondson (1982) ≡ *Armeriastrum ulicinum* (Boiss.) Kuntze, Revis. Gen. Pl. 2: 394. 1891 syn. sec. Kuntze (1891)
= *Statice androsacea* Jaub. & Spach, Ill. Pl. Orient. 1(10): 161, 164, t. 89. 1844 syn. sec. Bokhari & Edmondson (1982) ≡ *Acantholimon androsaceum* (Jaub. & Spach) Boiss. in Boissier, Diagn. Pl. Orient. ser. 1, 7: 73. 1846 syn. sec. Bokhari & Edmondson (1982) ≡ *Acantholimon androsaceum* var. *olympicum* Boiss. in Boissier, Diagn. Pl. Orient. ser. 1, 7: 73. 1846 syn. sec. Domina (2011+) ≡ *Acantholimon olympicum* (Boiss.) F.K.Mey. in Haussknechtia, Mitt. Thüring. Bot. Ges. 3: 24. 1987 syn. sec. Malekmohammadi & al. (2024: 30 March 2023)
= *Statice pauciflora* Jaub. & Spach, Ill. Pl. Orient. 1(10): 162. 1844 syn. sec. Bokhari & Edmondson (1982) ≡ *Acantholimon androsaceum* var. *majus* Boiss. in Boissier, Diagn. Pl. Orient. ser. 1, 7: 73. 1846 syn. sec. Bokhari & Edmondson (1982: 6 April 2022)
= *Statice tournefortii* Jaub. & Spach, Ill. Pl. Orient. 1: 165. t. 90. 1844 syn. sec. Bokhari & Edmondson (1982) ≡ *Acantholimon tournefortii* (Jaub. & Spach) Boiss. in Boissier, Diagn. Pl. Orient. ser. 1, 7: 74. 1846 syn. sec. Bokhari & Edmondson (1982)
= *Acantholimon androsaceum* var. *latifolium* Boiss. in Boissier, Diagn. Pl. Orient. ser. 1, 7: 73. 1846 syn. sec. Bunge (1872: 6 April 2022)
= *Acantholimon echinus* f. *disticum* Mobayen, Revis. Taxon. Acanth.: 216. 1964 syn. sec. POWO (2017+: 6 April 2022)
= *Acantholimon echinus* var. *glaberrimum* Mobayen, Revis. Taxon. Acanth.: 216. 1964 syn. sec. POWO (2017+: 6 April 2022)
= *Acantholimon trojanum* F.K.Mey. in Haussknechtia, Mitt. Thüring. Bot. Ges. 3: 27. 1987 syn. sec. Domina (2011+)
= *Acantholimon androsaceum* (Jaub. & Spach) Boiss. var. *androsaceum* syn. sec. Bunge (1872: 7 April 2022)

***Acantholimon ulicinum* var. *creticum* (Boiss.) Bokhari & J.R.Edm., Fl. Turkey 7: 498. 1982.** Sec. Bokhari & Edmondson (1982)

≡ *Acantholimon androsaceum* var. *creticum* Boiss. in Boissier, Diagn. Pl. Orient. ser. 1, 7: 73. 1846 syn. sec. Bokhari & Edmondson (1982) ≡ *Acantholimon creticum* (Boiss.) Rech.f. in Oesterr. Bot. Z. 84: 180. 1935 syn. sec. Bokhari & Edmondson (1982) ≡ *Acantholimon androsaceum* var. *creticum* (Boiss.) Critop., Problems Balkan Fl. and Veg.: 173. 1975 syn. sec. Malekmohammadi & al. (2024) ≡ *Acantholimon echinus* subsp. *creticum* (Boiss.) Papan. & Kokkini, Mountain Fl. Greece 1: 748. 1986 syn. sec. Malekmohammadi & al. (2024) − *Acantholimon ulicinum* var. *creticum* (Boiss.) Greuter, Colloque OPTIMA Crète Guide Excurs.: 27. 1975, nom. inval. syn. sec. Malekmohammadi & al. (2024)

***Acantholimon ulicinum* var. *purpurascens* (Bokhari) Bokhari & J.R.Edm., Fl. Turkey 7: 498. 1984.** Sec. Bokhari & Edmondson (1982)

≡ *Acantholimon androsaceum* var. *purpurascens* Bokhari in 30: 300. 1970 syn. sec. Bokhari & Edmondson (1982)

***Acantholimon ulicinum* (Willd. ex Schult.) Boiss. var. *ulicinum*.** Sec. Bokhari & Edmondson (1982)

= *Statice histrix* Jaub. & Spach, Ill. Pl. Orient. 1(10): 162. 1844 syn. sec. POWO (2017+: 28 apr 2024) ≡ *Acantholimon hystrix* (Jaub. & Spach) Boiss. in Boissier, Diagn. Pl. Orient. ser. 1, 7: 75. 1846 syn. sec. Bokhari & Edmondson (1982) − *Statice hystrix* Jaub. & Spach, Ill. Pl. Orient. 1(10): 162, t. 89. 1844, orth. var. syn. sec. Malekmohammadi & al. (2024)

***Acantholimon vacillans* Rech.f. & Schiman-Czeika, Fl. Iran. 108: 89. 1974.** Sec. Rechinger & Schiman-Czeika (1974)

***Acantholimon varivtzevae* Czerniak., Flora URSS 18: 738. 1952.** Sec. Linczevski (1952)

***Acantholimon vedicum* Mirzoeva in Dokl. Akad. Nauk Armenii 21(4): 183. 1955.** Sec. Domina (2011+)

***Acantholimon velutinum* Czerniak., Flora URSS 18: 739. 1952.** Sec. Linczevski (1952)

= *Acantholimon alatavicum* var. *puberulum* Bunge ex Regel in Trudy Imp. S.-Peterburgsk. Bot. Sada 6(2): 390. 1880 syn. sec. Linczevski (1952)

***Acantholimon venustum* (Fenzl ex Boiss.) Boiss. in Boissier, Diagn. Pl. Orient. ser. 1, 7: 80. 1846.** Sec. Rechinger & Schiman-Czeika (1974)

≡ *Statice venusta* Fenzl ex Boiss., Fl. Orient. 4(2): 832. 1879 syn. sec. Boissier & Heldreich (1846)
= *Statice dianthifolia* Jaub. & Spach, Ill. Pl. Orient. 1(10): 162. 1844 syn. sec. Boissier & Heldreich (1846) ≡ *Armeriastrum dianthifolium* (Jaub. & Spach) Kuntze, Revis. Gen. Pl. 2: 393. 1891 syn. sec. Kuntze (1891)

***Acantholimon venustum* var. *assyriacum* (Boiss.) Boiss., Fl. Orient. 4(2): 832. 1879.** Sec. Bokhari & Edmondson (1982)

≡ *Acantholimon assyriacum* Boiss. in Boissier, Diagn. Pl. Orient. ser. 1, 7: 81. 1846 syn. sec. Boissier (1879) ≡ *Armeriastrum assyriacum* (Boiss.) Kuntze, Revis. Gen. Pl. 2: 393. 1891 syn. sec. Kuntze (1891)

***Acantholimon venustum* (Fenzl ex Boiss.) Boiss. var. *venustum*.** Sec. Bokhari & Edmondson (1982)

***Acantholimon virens* Czerniak., Flora URSS 18: 737. 1952.** Sec. Linczevski (1952)

***Acantholimon viscidulum* Boiss. in Boissier, Diagn. Pl. Orient. ser. 1, 7: 71. 1846.** Sec. Bunge (1872)

≡ *Armeriastrum viscidulum* (Boiss.) Kuntze, Revis. Gen. Pl. 2: 394. 1891 syn. sec. Kuntze (1891)

***Acantholimon vvedenskyi* Lincz. in Bot. Mater. Gerb. Bot. Inst. Komarova Akad. Nauk S.S.S.R. 21: 486. 1961.** Sec. Linczevski (1961)

***Acantholimon wendelboi* Rech.f. & Schiman-Czeika, Fl. Iran. 108: 47. 1974.** Sec. Rechinger & Schiman-Czeika (1974)

= *Acantholimon movdarinum* Parsa, Pl. Nov. Iranicae: 10. 1946 syn. sec. Assadi (2005)

***Acantholimon wiedemannii* Bunge in Mém. Acad. Imp. Sci. Saint Pétersbourg, Sér. 7 18(2): 45. 1872 [as " *wiedemanni* "].** Sec. Doğan & Akaydın (2007)

≡ *Armeriastrum wiedemannii* (Bunge) Kuntze, Revis. Gen. Pl. 2: 394. 1891 syn. sec. Kuntze (1891) − *Acantholimon wiedemanni* Bunge in Mém. Acad. Imp. Sci. Saint Pétersbourg, Sér. 7, 18(2): 45. 1872, orth. var. syn. sec. Malekmohammadi & al. (2024)

***Acantholimon wilhelminae* Rech.f. & Schiman-Czeika, Fl. Iran. 108: 44. 1974.** Sec. Rechinger & Schiman-Czeika (1974)

***Acantholimon xanthacanthum* Rech.f. & Köie in Biol. Skr. 13(4) (Symb. Afgan. 5): 173. 1963.** Sec. Rechinger & Schiman-Czeika (1974)

***Acantholimon yamense* Turrill in Bull. Misc. Inform. Kew 1930(9): 442. 1930.** Sec. Rechinger & Schiman-Czeika (1974)

≡ *Acantholimon gilliatii* var. *yamense* (Turrill) Mobayen, Revis. Taxon. Acanth.: 148. 1964 syn. sec. Rechinger & Schiman-Czeika (1974)

***Acantholimon yildizelicum* Akaydın in Nordic J. Bot. 22(6): 680. 2004.** Sec. Doğan & Akaydın (2007)

***Acantholimon zaeifii* Assadi in Iran. J. Bot. 10(2): 154. 2004.** Sec. Assadi (2005)

***Acantholimon zakirovii* Beshko in Turczaninowia 18(1): 19. 2015.** Sec. Malekmohammadi & al. (2024)

***Acantholimon zaprjagaevii* Lincz. in Schischkin & Bobrov, Flora URSS 18: 732. 1952.** Sec. Linczevski (1952)

***Armeria* Willd., Enum. Pl. 1: 333. 1809, nom. cons.** Sec. Koutroumpa & al. (2018)

Type: *Armeria vulgaris* Willd.
= *Statice* L., Sp. Pl.: 274. 1753, nom. ambig. syn. sec. Koutroumpa & al. (2018). Lectoype: *Statice armeria* L.
= *Polyanthemum* Medik., Staatsw. Vorles. Churpf. Phys.-Oek. Ges. 1: 228. 1791 syn. sec. Gams (1927)
= *Reverchonia* Gand. in Kuntze in Post, Lex. Gen. Phan.: 481. 1903 syn. sec. Bernis (1954)
= *Statice* (L.) Mill., Dict. Abridg. ed. 4: without pagination. 1754 syn. sec. Bernis (1954)

Notes. – Genus of about 100 species, found in temperate regions of the Northern hemisphere and in South America (Chile, Tierra del Fuego) with the Iberian Peninsula as its centre of distribution (Nieto Feliner 1990). Aguilar & Feliner (2003) carried out a phylogenetic analysis using ITS marker sequencing 133 accessions from 71 species. Their study resulted in a phylogenetic tree with large polytomic and low-supported clades that reflects reticulate and concerted evolution and hybridization events in this genus. Although only a very small number of *Armeria* species are used in the published phylogenetic studies, these studies always provided a monophyletic clade (Lledó et al. 1998, 2005; Aguilar & Feliner 2003; Moharrek et al, 2017; Koutroumpa et al. 2018) and thus *Armeria* is for the time being considered to be monophyletic.

***Armeria alboi* (Bernis) Nieto Fel. in Anales Jard. Bot. Madrid, ser. 2, 44: 333. 1987.** Sec. Domina (2011+)

≡ *Armeria maritima* var. *alboi* Bernis, Rev. Gen. Armeria Comp.: 8. 1951 [as " *albi* "] syn. sec. Nieto Feliner (1987) ≡ *Armeria pseudarmeria* var. *alboi* Bernis in Anales Inst. Bot. Cavanilles 12(2): 135. 1955 syn. sec. Malekmohammadi & al. (2024) ≡ *Armeria pseudarmeria* subsp. *alboi* (Bernis) Malag. in Acta Phytotax. Barcinon. 1: 24. 1968 ["1969"] syn. sec. Malekmohammadi & al. (2024) − *Armeria maritima* var. *albi* Bernis, orth. var. syn. sec. Domina (2011+) [is orthographic variant for *Armeria maritima* var. *albi* Bernis] − *Armeria albi* (Bernis) Nieto, orth. var. syn. sec. Malekmohammadi & al. (2024)

***Armeria alliacea* (Cav.) Hoffmanns. & Link, Fl. Portug. 1: 441. 1817.** Sec. Nieto Feliner (1987)

= *Statice alliacea* Cav., Icon. 2: 6. 1793 syn. sec. Sampaio (1913); ≡ *Armeria plantaginea* subsp. *alliacea* (Cav.) Malag. in Acta Phytotax. Barcinon. 1: 25. 1968 ["1969"] syn. sec. Malekmohammadi & al. (2024)

= *Armeria alliacea* Mutel, A., Fl. Franç. 3: 86. 1836 syn. sec. Malekmohammadi & al. (2024)
= *Armeria allioides* Boiss., Voy. Bot. Espagne 2: 524. 1841, nom. illeg. syn. sec. Pignatti (1972)
= *Armeria montana* Ray ex Wallr., Beitr. Bot. 2: 210. 1844 syn. sec. Domina (2011+)
= *Armeria rigida* subsp. *latifolia* Wallr. in Beitr. Bot. 2: 200. 1844 syn. sec. Malekmohammadi & al. (2024)
= *Armeria tenuis* var. *humilis* Wallr. in Beitr. Bot. 2: 184. 1844 syn. sec. Malekmohammadi & al. (2024)
= *Armeria plantaginea* var. *brachylepis* Boiss. in Candolle, Prodr. 12: 683. 1848 syn. sec. Malekmohammadi & al. (2024)
= *Armeria plantaginea* var. *brachyphylla* Boiss. in Candolle, Prodr. 12: 683. 1848 syn. sec. Malekmohammadi & al. (2024)
= *Armeria plantaginea* var. *leucantha* Boiss. in Candolle, Prodr. 12: 683. 1848 syn. sec. Malekmohammadi & al. (2024) ≡ *Statice plantaginea* var. *leucantha* (Boiss.) F.T.Hubb. in Rhodora 18(211): 157. 1916 syn. sec. Bailey (1916)
= *Armeria plantaginea* var. *longibracteata* Boiss. in Candolle, Prodr. 12: 683. 1848 syn. sec. Malekmohammadi & al. (2024)
= *Armeria plantaginea* var. *scorzonerifolia* Boiss. in Candolle, Prodr. 12: 683. 1848 syn. sec. Malekmohammadi & al. (2024)
= *Armeria leucantha* (Boiss.) Mathon in Bull. Mus. Natl. Hist. Nat. 21: 134. 1849 syn. sec. POWO (2017+)
= *Armeria seticeps* Rchb. f. in Fl. Germ. Excurs. 17: 68. 1855, nom. illeg. syn. sec. Arrigoni (2015) [non *Armeria seticeps* Rchb.]
= *Armeria bupleuroides* Cutanda, Fl. Madrit.: 561. 1861 syn. sec. Daveau (1888: Synonym of Armeria plantaginea Willd.)
= *Armeria plantaginea* subsp. *castellana* (Boiss. & Reut. ex Leresche) Nyman, Consp. Fl. Eur. Suppl. 2(2): 265. 1890 syn. sec. Domina (2011+)
= *Statice dianthodes* Voss, Vilm. Blumengärtn., ed. 3, 1: 618. 1895 syn. sec. Malekmohammadi & al. (2024)
= *Armeria montana* var. *stenophylla* Rouy in Naturaliste II, 14: 166. 1906 syn. sec. Malekmohammadi & al. (2024)
= *Armeria bupleuroides* var. *reducta* Rouy, Fl. France 2: 174. 1908 syn. sec. Malekmohammadi & al. (2024)
= *Armeria alliacea* var. *allioides* Pau in Repert. Spec. Nov. Regni Veg. 8: 136. 1910 syn. sec. Malekmohammadi & al. (2024)
= *Statice plantaginea* proles *rigida* (Wallr.) Samp., Herb. Port.: 101. 1913 syn. sec. Sampaio (1913: synonym of Armeria rigida Wallr.) ≡ *Statice rigida* (Wallr.) Samp., Fl. Port.: 442. 1947 syn. sec. Domina (2011+) ≡ *Armeria plantaginea* subsp. *rigida* (Wallr.) Malag. in Acta Phytotax. Barcinon. 1: 25. 1968 ["1969"] syn. sec. Malekmohammadi & al. (2024)
= *Armeria alliacea* var. *heterophylla* Pau ex Viciosa in Bol. Real Soc. Esp. Hist. Nat. 12: 139. 1916 syn. sec. Malekmohammadi & al. (2024)
= *Armeria sabulosa* var. *serpentini* Legrand in Bull. Géogr. Bot. 29: 31. 1919 syn. sec. Malekmohammadi & al. (2024)
= *Armeria matritensis* Pau in Bol. Soc. Ibér. Ci. Nat. 22: 98. 1923 syn. sec. Malekmohammadi & al. (2024)
= *Armeria montana* f. *sicorisiensis* Sennen, Exsicc. (Pl. Esp.) 1926: 5886. 1926 syn. sec. Malekmohammadi & al. (2024)
= *Statice allioides* Braun-Blanq. in Bull. Soc. Bot. France 77: 288. 1930 syn. sec. Malekmohammadi & al. (2024)
= *Armeria pseudarmeria* subsp. *allioides* (Boiss.) Malag. in Acta Phytotax. Barcinon. 1: 24. 1968 ["1969"] syn. sec. Malekmohammadi & al. (2024)
− *Armeria longearistata* Bourg. ex Reut. in Bull. Soc. Bot. France 2: 643. 1855, in sched. syn. sec. Malekmohammadi & al. (2024)
− *Armeria segoviensis* Gand., Fl. Eur. 19: 8. 1890, nom. inval. syn. sec. Malekmohammadi & al. (2024)

***Armeria alliacea* (Cav.) Hoffmanns. & Link subsp. *alliacea*.** Sec. Domina (2011+)

= *Statice sabulosa* Fourr. in Ann. Soc. Linn. Lyon, sér. 2, 17: 141. 1869 syn. sec. Malekmohammadi & al. (2024)
= *Armeria sicorisiana* Sennen in Bull. Soc. Bot. France 73: 674. 1927 syn. sec. Malekmohammadi & al. (2024)
= *Armeria maritima* var. *rumelicina* Bernis, Rev. Gen. Armeria Comp.: 2. 1951 syn. sec. Domina (2011+) ≡ *Armeria alliacea* var. *rumelicina* (Bernis) O.Bolòs & Vigo in Collect. Bot. (Barcelona) 11: 30. 1979 syn. sec. Domina (2011+: synomym of Armeria maritima var. rumelica)
= *Armeria alliacea* var. *javalambrica* Bernis in Anales Inst. Bot. Cavanilles 12(2): 212. 1955 syn. sec. Malekmohammadi & al. (2024) ≡ *Armeria maritima* subvar. *javalambrica* Bernis in Anales Inst. Bot. Cavanilles 12(2): 212. 1955 syn. sec. Malekmohammadi & al. (2024: 28 April 2022)
= *Armeria plantaginea* subsp. *sicorisiensis* (Sennen) Malag. in Acta Phytotax. Barcinon. 1: 25. 1968 ["1969"] syn. sec. Malekmohammadi & al. (2024: 7 May 2022) ≡ *Armeria alliacea* var. *sicorisiensis* (Sennen) O.Bolòs & Vigo in Collect. Bot. (Barcelona) 11: 30. 1979 syn. sec. Malekmohammadi & al. (2024)
= *Armeria alliacea* var. *palearensis* O.Bolòs & Vigo in Collect. Bot. (Barcelona) 11: 30. 1979 syn. sec. POWO (2017+)
= *Armeria alliacea* subsp. *loscosii* Romo in Estud. Mus. Cienc. Nat. Álava 10-11: 198. 1996 syn. sec. POWO (2017+: 9 May 2022)
− *Armeria trigoloides* Ebel, Armeriae: 34. 1840, orth. var. syn. sec. Malekmohammadi & al. (2024) [is misspelling for *Armeria trigonoides* Ebel] − *Armeria trigonoides* Ebel, Armeriae: 34. 1840, pro syn. syn. sec. Malekmohammadi & al. (2024)
= *Armeria cephalotes* Schousb., Iagttag. Vextrig. Marokko: 146. 1800 syn. sec. Domina (2011+)
= *Statice scorzonerifolia* Link in J. Bot. (Schrader) 1800(1): 60. 1801 syn. sec. Malekmohammadi & al. (2024)
= *Armeria scorzonerifolia* Willd., Enum. Pl.: 334. 1809 syn. sec. POWO (2017+)
= *Armeria leucantha* Salzm. ex Boiss., Voy. Bot. Espagne 2: 525. 1841 syn. sec. POWO (2017+: 9 May 2022)
= *Armeria juncea* Wallr., Beitr. Bot. 2: 212. 1844 syn. sec. POWO (2017+) ≡ *Statice juncea* (Wallr.) Samp., Herb. Port.: 101. 1913 syn. sec. Govaerts, R. (ed.) (2023)
= *Armeria rigida* Wallr., Beitr. Bot. 2: 199. 1844, nom. illeg. syn. sec. Domina (2011+)
= *Armeria stenophylla* Girard in Ann. Sci. Nat., Bot., sér. 3, 2: 323. 1844 syn. sec. POWO (2017+)
= *Armeria dianthoides* Hornem. & Spreng. ex Boiss. in Candolle, Prodr. 12: 683. 1848 syn. sec. POWO (2017+: 9 May 2022)
= *Armeria sabulosa* Jord. ex Boreau, Fl. Centre France ed. 3, 2: 537. 1857 syn. sec. POWO (2017+: 9 May 2022)

***Armeria alliacea* subsp. *matritensis* (Pau) Borja, Rivas Goday & Rivas Mart. in Anales Inst. Bot. Cavanilles 25: 154. 1969.** Sec. Pignatti (1972)

≡ *Statice alliacea* var. *matritensis* Pau in Bol. Soc. Ibér. Ci. Nat. 22: 98. 1923 syn. sec. Malekmohammadi & al. (2024) ≡ *Armeria maritima* var. *matritensis* (Pau) Bernis in Anales Inst. Bot. Cavanilles 12(2): 206. 1955 syn. sec. Malekmohammadi & al. (2024) ≡ *Armeria daveaui* subsp. *matritensis* (Bernis) Franco, Nova Fl. Portugal 2: 562. 1984 syn. sec. Malekmohammadi & al. (2024)

***Armeria alpina* Willd., Enum. Pl.: 333. 1809.** Sec. Nieto Feliner (1987)

≡ *Armeria vulgaris* var. *alpina* (Willd.) F.Petri, Gen. Armeniae: 41. 1863 syn. sec. Arrigoni (2015) ≡ *Armeria maritima* var. *alpina* (Willd.) G.H.M.Lawr. in Gentes Herbarum 4: 406. 1940 syn. sec. Arrigoni (2015) ≡ *Armeria pocutica* subsp. *alpina* (Willd.) P.Silva in Bot. Jour. Linn. Soc. 64: 376. 1971 syn. sec. Arrigoni (2015: 29 April 2022)
= *Statice montana* Mill., Gard. Dict., ed. 8: Statice n.º 2. 1768 syn. sec. Gams (1927) ≡ *Statice armeria* var. *alpina* DC., Fl. Franç. ed. 3, 3: 419. 1805 syn. sec. Arrigoni (2015) ≡ *Statice alpina* (DC.) Poir., Encycl. Suppl. 5: 234. 1817 syn. sec. Arrigoni (2015) ≡ *Armeria elongata* var. *alpina* (DC.) Ces., Comp. Fl. Ital. 2: 289. 1886 syn. sec. Arrigoni (2015) ≡ *Armeria maritima* var. *alpina* (DC.) Bernis in Anales Inst. Bot. Cavanilles 14: 335. 1957 syn. sec. Arrigoni (2015) ≡ *Armeria maritima* subsp. *alpina* (DC.) P.Silva in Bot. J. Linn. Soc. 64(4): 376. 1971 syn. sec. Arrigoni (2015)
= *Armeria cantabrica* var. *montana* Rouy in Ill. Pl. Eur. 2: 14. 1895 syn. sec. POWO (2017+)
= *Armeria vulgaris* var. *alpina* Fiori, Fl. Anal. Ital. 2: 334. 1902 syn. sec. Arrigoni (2015)
= *Armeria alpina* f. *basitricha* Simonk. in Magyar Bot. Lapok 6: 14. 1907 syn. sec. POWO (2017+)
= *Armeria alpina* var. *nana* Bolzon in Bull. Soc. Bot. Ital. 1910: 74. 1910 syn. sec. POWO (2017+)
= *Armeria alpina* var. *eliator* Conill & Despaty in Bull. Soc. Bot. France 67: 146. 1920 syn. sec. POWO (2017+)
= *Armeria alpina* var. *pumila* Fuss ex Jáv., Magyar Fl.: 816. 1925 syn. sec. Domina (2011+) ≡ *Armeria alpina* f. *pumila* (Fuss ex Jáv.) Novák in Comm. Reg. Soc. Sci. Bohem. Mat. Nat. 28: 22. 1939 syn. sec. Domina (2011+) ≡ *Armeria alpina* subsp. *pumila* Fuss ex Jáv. syn. sec. Domina (2011+)
= *Armeria alpina* f. *glabra* Novák in Comm. Reg. Soc. Sci. Bohem. Mat. Nat. 28: 22. 1939 syn. sec. POWO (2017+)
= *Armeria alpina* f. *hirsuta* Novák in Comm. Reg. Soc. Sci. Bohem. Mat. Nat. 28: 22. 1939 syn. sec. POWO (2017+)
= *Armeria alpina* f. *hirtifolia* Novák in Comm. Reg. Soc. Sci. Bohem. Mat. Nat. 28: 23. 1939 syn. sec. POWO (2017+)
− *Armeria alpina* Hoppe ex Ebel, Armeriae: 26. 1840, nom. inval. syn. sec. IPNI (2000+)

***Armeria alpina* Willd. subsp. *alpina*.** Sec. Domina (2011+)

= *Armeria montana* (Mill.) F.Herm., Fl. Deutschl. Fennoskand.: 368. 1912, nom. illeg. syn. sec. Malekmohammadi & al. (2024)

***Armeria alpina* subsp. *bubanii* (G.H.M.Lawr.) Rivas Mart. in Itinera Geobot. 15: 698. 2002.** Sec. Domina (2011+)

≡ *Armeria bubanii* G.H.M.Lawr. in Gentes Herbarum 4: 413. 1940 syn. sec. Domina (2011+) − *Armeria alpina* subsp. *bubanii* (G.H.M.Lawr.) Malag. in Acta Phytotax. Barcinon. 1: 22. 1968 ["1969"], nom. inval. syn. sec. Domina (2011+)
= *Armeria alpina* subvar. *confusa* Bernis in Anales Inst. Bot. Cavanilles 14: 266. 1957 syn. sec. Malekmohammadi & al. (2024) ≡ *Armeria alpina* subsp. *confusa* (Bernis) Malag. in Acta Phytotax. Barcinon. 1: 22. 1968 ["1969"] syn. sec. Malekmohammadi & al. (2024)

***Armeria alpina* subsp. *halleri* (Wallr.) Nyman, Consp. Fl. Eur. 3: 616. 1881.** Sec. Domina (2011+)

= *Armeria halleri* Wallr., Beitr. Bot. 2: 194. 1844 syn. sec. Nyman (1881); ≡ *Armeria vulgaris* var. *halleri* (Wallr.) F.Petri, Gen. Armeniae: 40. 1863 syn. sec. Nyman (1881: 9 May 2022) ≡ *Armeria maritima* subsp. *halleri* (Wallr.) Rothm. in Feddes Repert. Spec. Nov. Regni Veg. 67: 9. 1963 syn. sec. Domina (2011+);
= *Statice halleri* Garcke, Fl. N. Mitt.-Deutschland, ed. 2: 273. 1851 syn. sec. Malekmohammadi & al. (2024: 14 April 2022)
= *Armeria bottendorfensis* A.G.Schulz in Mitth. Thüring. Bot. Vereins, n.s. n.f. 42: 33. 1936 syn. sec. Domina (2011+: 4 May 2022) ≡ *Statice bottendorfensis* (A.G.Schulz) O.Schwarz in Mitth. Thüring. Bot. Vereins, n.f. 43: 33. 1936 syn. sec. Domina (2011+: 4 May 2022) ≡ *Armeria maritima* subsp. *bottendorfensis* (A.G.Schulz) Rothm. syn. sec. Domina (2011+) ≡ *Armeria maritima* subsp. *hornburgensis* (A.G.Schulz) Rothm. syn. sec. Domina (2011+)
= *Armeria vulgaris* subsp. *serpentini* (Gauckler) Holub in Folia Geobot. Phytotax. 12: 428. 1977 syn. sec. POWO (2017+) ≡ *Armeria maritima* subsp. *serpentini* (Gauckler) Rothm. syn. sec. Domina (2011+) ≡ *Armeria maritima* var. *serpentini* Gauckler syn. sec. Malekmohammadi & al. (2024)

***Armeria alpina* subsp. *occasiana* (Bernis) Rivas Mart. in Itinera Geobot. 15: 698. 2002.** Sec. Domina (2011+)

≡ *Armeria maritima* f. *occasiana* Bernis in Anales Inst. Bot. Cavanilles 14: 334. 1957 syn. sec. Domina (2011+) ≡ *Armeria maritima* var. *occasiana* (Bernis) O.Bolòs & Vigo in Collect. Bot. (Barcelona) 11: 31. 1979 syn. sec. Domina (2011+: 4 May 2022)

***Armeria alpinifolia* Pau & Font Quer, Iter Marocc. 1927: 473. 1928.** Sec. Malagarriga (1968)

≡ *Statice alpinifolia* (Pau & Font Quer) Maire in Jahandiez & al., Cat. Pl. Maroc 3: 566. 1934 syn. sec. Maire (1934)

***Armeria apollinaris* Sennen & Mauricio, Diagn. Nouv.: 242. 1936.** Sec. Malekmohammadi & al. (2024)

≡ *Statice alliacea* var. *apollinaris* (Sennen & Mauricio) Maire, Cat. Pl. Maroc 4: 1092. 1941 syn. sec. Malekmohammadi & al. (2024)

***Armeria arcuata* Welw. ex Boiss. & Reut., Pugill. Pl. Afr. Bor. Hispan.: 101. 1852.** Sec. Pignatti (1972)

= *Statice juniperifolia* Samp. syn. sec. Domina (2011+)
– *Statice juniperifolia* sensu auct., err. sec. Domina (2011+)

***Armeria arenaria* (Pers.) F.Dietr. in Nachtr. Vollst. Lex. Gärtn. 1: 313. 1815.** Sec. Tiburtini & al. (2022)

= *Statice plantaginea* var. *minor* Gaudin, Fl. Helv. 2: 455. 1828 syn. sec. Malekmohammadi & al. (2024)

– *Armeria alliacea* sensu auct., err. sec. Nieto Feliner (1987)

***Armeria arenaria* subsp. *anomala* (Bernis) Catalán ex Uribe-Ech., Claves Ilustr. Fl. País Vasco y Territor. Limítr.: 767. 1999.** Sec. Malekmohammadi & al. (2024)

≡ *Armeria maritima* var. *anomala* Bernis, Rev. Gen. Armeria Comp.: 11. 1951 syn. sec. Malekmohammadi & al. (2024)

***Armeria arenaria* (Pers.) F.Dietr. subsp. *arenaria*.** Sec. Tiburtini & al. (2022)

= *Statice plantaginea* All., Fl. Pedem. 2: 90. 1785 syn. sec. Arrigoni (2015) ≡ *Armeria plantaginea* (All.) Willd., Enum. Pl.: 334. 1809 syn. sec. Domina (2011+) ≡ *Armeria vulgaris* var. *plantaginea* (Willd.) F.Petri, Gen. Armeriae: 39. 1863 syn. sec. Malekmohammadi & al. (2024) ≡ *Armeria alliacea* subsp. *plantaginea* (All.) O.Bolòs & Vigo in Collect. Bot. (Barcelona) 11: 30. 1979 syn. sec. Malekmohammadi & al. (2024) ≡ *Armeria alliacea* var. *plantaginea* (All.) O.Bolòs & Vigo in Collect. Bot. (Barcelona) 11: 30. 1979 syn. sec. Malekmohammadi & al. (2024) ≡ *Armeria plantaginea* subsp. *plantaginea* syn. sec. Domina (2011+) ≡ *Armeria vulgaris* subsp. *plantaginea* (All.) Syme, Sowerby, nom. illeg. syn. sec. Arrigoni (2015)
= *Armeria montcaunica* Pau ex Losa in Bol. Soc. Ibér. Ci. Nat. 29: 98. 1930 syn. sec. POWO (2017+) ≡ *Armeria plantaginea* subsp. *montcaunica* (Pau ex Losa) Malag. in Acta Phytotax. Barcinon. 1: 25. 1968 ["1969"] syn. sec. Malekmohammadi & al. (2024)
= *Armeria petri-ludovicii* Sennen, Diagn. Nouv.: 28. 1936 syn. sec. Malekmohammadi & al. (2024)
= *Armeria sennenii* G.H.M.Lawr. in Gentes Herbarum 4: 417. 1940 syn. sec. Malekmohammadi & al. (2024)
= *Armeria plantaginea* subsp. *piorum* (Sennen) Malag. in Acta Phytotax. Barcinon. 1: 25. 1968 ["1969"] syn. sec. Malekmohammadi & al. (2024)
− *Armeria piorum* Sennen, Bull. Soc. Bot. France 73: 674, nomen. 1927, in sched. syn. sec. Arrigoni (2015)
− *Statice major* Garsault, Fig. Pl. Méd. 4: t. 571. 1764, nom. inval. syn. sec. Domina (2011+)

***Armeria arenaria* subsp. *bilbilitana* (Bernis) Nieto Fel. in Anales Jard. Bot. Madrid, ser. 2, 44: 341. 1987.** Sec. Nieto Feliner (1987)

≡ *Armeria maritima* var. *bilbilitana* Bernis, Rev. Gen. Armeria Comp.: 10. 1951 syn. sec. Nieto Feliner (1987) ≡ *Armeria alliacea* var. *bilbilitana* (Bernis) O.Bolòs & Vigo, Fl. Països Catalans 3: 97. 1995 syn. sec. POWO (2017+: 9 May 2022)

***Armeria arenaria* subsp. *bupleuroides* (Godr. & Gren.) Greuter & Raus in Willdenowia 19(1): 39. 1989.** Sec. Malekmohammadi & al. (2024)

≡ *Armeria bupleuroides* Gren. & Godr., Fl. France 2: 736. 1853 syn. sec. Greuter & Raus (1989) ≡ *Armeria plantaginea* subsp. *bupleuroides* (Gren. & Godr.) Nyman, Consp. Fl. Eur. 3: 616. 1881 syn. sec. Malekmohammadi & al. (2024) ≡ *Armeria plantaginea* var. *bupleuroides* (Gren. & Godr.) G.H.M.Lawr. in Gentes Herbarum 4: 410. 1940 syn. sec. Domina (2011+) ≡ *Armeria alliacea* subsp. *bupleuroides* (Gren. & Godr.) O.Bolòs & Vigo in Collect. Bot. (Barcelona) 11: 30. 1979 syn. sec. Domina (2011+: synonym of Armeria abupleoroides according to O.Bolòs & Vigo (1979)) ≡ *Armeria alliacea* var. *bupleuroides* (Gren. & Godr.) O.Bolòs & Vigo in Collect. Bot. (Barcelona) 11: 30. 1979 syn. sec. Malekmohammadi & al. (2024)
= *Armeria alliacea* subvar. *sicorisiensis* (Sennen) Bernis in Anales Inst. Bot. Cavanilles 12(2): 185. 1955 syn. sec. Malekmohammadi & al. (2024)
− *Statice alliacea* Willd., Sp. Pl., ed. 4, 1(2): 1523. 1798, nom. inval. syn. sec. Nyman (1881: 616)

***Armeria arenaria* subsp. *burgalensis* (Sennen & Elias) Uribe-Ech. in Estud. Mus. Cienc. Nat. Álava 6: 53. 1991.** Sec. Malekmohammadi & al. (2024)

≡ *Armeria burgalensis* Sennen & Elias, Exsicc. (Pl. Espagne) 1921: n.° 4341. 1921. syn. sec. Malekmohammadi & al. (2024) ≡ *Armeria plantaginea* subsp. *burgalensis* (Sennen & Elias) Malag. in Acta Phytotax. Barcinon. 1: 25. 1968 ["1969"] syn. sec. Malekmohammadi & al. (2024)

***Armeria arenaria* subsp. *confusa* (Bernis) Nieto Fel. in Anales Jard. Bot. Madrid, ser. 2, 44: 341. 1987.** Sec. Nieto Feliner (1987)

≡ *Armeria maritima* subvar. *confusa* Bernis in Anales Inst. Bot. Cavanilles 14: 266. 1957 syn. sec. Nieto Feliner (1987)

***Armeria arenaria* subsp. *madoricola* M.B.Crespo & Mateo in Flora Montiber., 49-55, 46: 50. 2010.** Sec. Malekmohammadi & al. (2024)

= *Armeria alpina* f. *conquensis* Bernis in Anales Inst. Bot. Cavanilles 14: 261. 1957 [as " *conchensis* "] syn. sec. Malekmohammadi & al. (2024) − *Armeria alpina* f. *conchensis* Bernis, orth. var. syn. sec. Malekmohammadi & al. (2024)

***Armeria arenaria* subsp. *marginata* (Levier) Arrigoni in Fl. Medit. 25(Special issue): 15. 2015.** Sec. Tiburtini & al. (2022)

≡ *Armeria majellensis* var. *marginata* Levier in Atti Soc. Tosc. Sci. Nat. Pisa Processi Verbali 6: 157. 1887 syn. sec. Tiburtini & al. (2022: 10 May 2022) ≡ *Armeria vulgaris* var. *marginata* (Levier) Fiori, Fiori e Paol. Fl. Anal. Ital. 2: 234. 1902 syn. sec. Arrigoni (2015) ≡ *Armeria maritima* subvar. *marginata* (Levier) Bernis in Anales Inst. Bot. Cavanilles 11(2): 247. 1954 syn. sec. Arrigoni (2015) ≡ *Armeria marginata* (Levier) Bianchini in Giorn. Bot. Ital. 111: 49. 1977 syn. sec. Tiburtini & al. (2022).
= *Armeria plantaginea* Bertol., Mant. Pl. Fl. Alp. Apuan.: 20. 1832 syn. sec. Arrigoni (2015)
= *Armeria arenaria* subsp. *apennina* Arrigoni in Fl. Medit. 25(Special issue): 13, fig. 1. 2015 syn. sec. Tiburtini & al. (2022)

***Armeria arenaria* subsp. *pradetensis* Médail, Baumel & Auda in Bot. J. Linn. Soc. 159(2): 265. 2009.** Sec. Malekmohammadi & al. (2024)

***Armeria arenaria* subsp. *praecox* (Jord.) Kerguélen ex Greuter, Burdet & G.Long in Med–Checkl. 4: 309. 1989.** Sec. Tiburtini & al. (2022)

≡ *Armeria praecox* Jord. In Boreau, Fl. Centre France, ed. 3, 2: 537. 1857 syn. sec. Tiburtini & al. (2022) ≡ *Armeria plantaginea* subsp. *praecox* (Jord.) Nyman, Consp. Fl. Eur. 3: 616. 1881 syn. sec. Arrigoni (2015) ≡ *Armeria alliacea* subsp. *praecox* (Jord.) Jovet & R.Vilm., Fl. Descr. France Suppl.: 410. 1977 syn. sec. Domina (2011+) − *Armeria arenaria* subsp. *praecox* (Jord.) Kerguélen, Lejeunia nov. ser., 120: 49. 1987, nom. inval. syn. sec. Tiburtini & al. (2022)

***Armeria arenaria* subsp. *segoviensis* (Gand. ex Bernis) Nieto Fel. in Anales Jard. Bot. Madrid, ser. 2, 44: 340. 1987.** Sec. Nieto Feliner (1987)

≡ *Armeria maritima* var. *segoviensis* Gand. ex Bernis in Anales Inst. Bot. Cavanilles 12(2): 164, 167. 1955 syn. sec. Nieto Feliner (1987) ≡ *Armeria plantaginea* subsp. *segoviensis* (Gand. ex Bernis) Rivas Mart. in Anales Inst. Bot. Cavanilles 21: 250. 1963 syn. sec. Nieto Feliner (1987)
= *Statice lacaitae* Villar in Cavanillesia 2: 79. 1929 syn. sec. Nieto Feliner (1987) ≡ *Armeria segoviensis* subsp. *lacaitae* (Villar) Rivas Mart., Fern.Gonz. & Sánchez Mata n Opusc. Bot. Pharm. Complut. 2: 104. 1986 syn. sec. Govaerts, R. (ed.) (2023) ≡ *Armeria lacaitae* (Villar) Rivas Mart. in Lagascalia 15(Extra): 115. 1988 syn. sec. Domina (2011+)
= *Armeria plantaginea* subsp. *oretana* (Bernis) Malag. in Acta Phytotax. Barcinon. 1: 25. 1968 ["1969"] syn. sec. Malekmohammadi & al. (2024) ≡ *Armeria duriensis* subsp. *oretana* (Bernis) Franco, Nova Fl. Portugal 2: 560. 1984 syn. sec. Malekmohammadi & al. (2024)
– *Armeria plantaginea* sensu auct., err. sec. Nieto Feliner (1987)

***Armeria arenaria* subsp. *vestita* (Willk.) Nieto Fel. in Anales Jard. Bot. Madrid, ser. 2, 44: 341. 1987.** Sec. Nieto Feliner (1987)

≡ *Armeria vestita* Willk. in Willkomm & Lange, Prodr. Fl. Hispan. 2: 366. 1868 syn. sec. Nieto Feliner (1987) ≡ *Armeria longiaristata* subsp. *vestita* (Willk.) Nyman, Consp. Fl. Eur. 3: 615. 1881 syn. sec. Domina (2011+)

***Armeria asperrima* (Sennen) Sennen, Bull. Soc. Bot. France 73: 673, nomen. 1927.** Sec. Sennen (1927)

− *Armeria rigida* f. *asperrima* Sennen, Pl. Espagne 1918, n.° 3.472. 1918, nom. inval. syn. sec. Malekmohammadi & al. (2024)

***Armeria aspromontana* Brullo, Scelsi & Spamp. in Edinburgh J. Bot. 54(1): 91. 1997.** Sec. Arrigoni (2015)

***Armeria atlantica* Pomel, Nouv. Mat. Fl. Atl. 1: 134. 1874.** Sec. Domina (2011+)

≡ *Statice plantaginea* var. *atlantica* (Pomel) Maire in Jahandiez & al., Cat. Pl. Maroc 3: 566. 1934 syn. sec. Malekmohammadi & al. (2024)
= *Statice plantaginea* subsp. *medians* Maire in Bull. Soc. Hist. Nat. Afrique N. 20: 29. 1929 syn. sec. Malekmohammadi & al. (2024)
= *Statice longearistata* var. *cuspidata* Faure & Maire in Bull. Soc. Hist. Nat. Afrique N. 22: 304. 1931 syn. sec. Malekmohammadi & al. (2024) ≡ *Statice plantaginea* var. *cuspidata* (Faure & Maire) Maire, Cat. Pl. Maroc 4: 1092. 1941 syn. sec. Malekmohammadi & al. (2024)
= *Statice plantaginea* subsp. *leucantha* (Boiss.) Maire in Jahandiez & al., Cat. Pl. Maroc 3: 566. 1934 syn. sec. Domina (2011+) ≡ *Armeria plantaginea* subsp. *leucantha* (Boiss.) Sauvage & Vindt in Trav. Inst. Sci. Chérifien, Sér. Bot. 4: 41. 1952 syn. sec. Domina (2011+)
= *Statice plantaginea* var. *subcuspidata* Maire, Cat. Pl. Maroc 4: 1092. 1941 syn. sec. Malekmohammadi & al. (2024)
= *Armeria fibrosa* Pomel, Nouv. Mat. Fl. Atl. 1: 133. 1874 syn. sec. POWO (2017+: 8 May 2022)

***Armeria australis* Boiss., Voy. Bot. Espagne 2: 526. 1845.** Sec. Malekmohammadi & al. (2024)

***Armeria beirana* Franco, Nova Fl. Portugal 2: 561. 1984.** Sec. Nieto Feliner (1987)

= *Armeria scorzonerifolia* Link in J. Bot. (Schrader) 3: 60. 1800 syn. sec. POWO (2017+: 8 May 2022)
= *Armeria beirana* subvar. *monchiquensis* Bernis in Bol. Soc. Brot., sér. 2, 23: 249. 1950 syn. sec. Nieto Feliner (1987) ≡ *Armeria beirana* subsp. *monchiquensis* (Bernis) Franco, Nova Fl. Portugal 2: 562. 1984 syn. sec. POWO (2017+: 8 May 2022) ≡ *Armeria beirana* var. *subrinhoi* Bernis in Anales Inst. Bot. Cavanilles 12(2): 149. 1955 syn. sec. Nieto Feliner (1987)
= *Armeria beirana* subvar. *sublittorea* Bernis in Bol. Soc. Brot., sér. 2, 23: 250. 1950 syn. sec. Nieto Feliner (1987) ≡ *Armeria duriensis* subsp. *sublittorea* (Bernis) Franco, Nova Fl. Portugal 2: 550. 1984 syn. sec. Nieto Feliner (1987)
= *Armeria maritima* f. *anisophylla* Bernis in Bol. Soc. Brot., sér. 2, 23: 248. 1950 syn. sec. Malekmohammadi & al. (2024) ≡ ?*Armeria carpetana* subsp. *anisophylla* (Bernis) Franco, Nova Fl. Portugal 2: 562. 1984 syn. sec. POWO (2017+)
– *Armeria plantaginea* sensu auct., non (All.) Willd., err. sec. Nieto Feliner (1987)

***Armeria belgenciensis* Donad. ex Kerguélen in Lejeunia 120: 49. 1987.** Sec. Domina (2011+)

= *Armeria filicaulis* Rouy, Fl. France 10: 169. 1908 syn. sec. Malekmohammadi & al. (2024)
= *Armeria arenaria* subsp. *peirescii* Baumel, Auda & Médail in Bot. J. Linn. Soc. 159(2): 265. 2009 syn. sec. Baumel & al. (2020)
= *Armeria belgenciensis* Donad., Bull. Soc. Bot. France 116: 514 in clavi, 519, in obs. 1970 syn. sec. Malekmohammadi & al. (2024)
= *Armeria belgenciensis* Donad. ex Guin. & Vilmorin, Fl. France [M. Guinochet & R. de Vilmorin] 1: 338, nom nov., without replaced synonym ref. 1973 syn. sec. Malekmohammadi & al. (2024)

***Armeria berlengensis* Daveau in Bol. Soc. Brot. 2: 24. 1884.** Sec. Nieto Feliner (1987)

≡ *Statice welwitschii* var. *berlengensis* (Daveau) Samp., Fl. Port.: 439. 1947 syn. sec. Malekmohammadi & al. (2024)

***Armeria bigerrensis* (C.Vicioso & Beltrán) Pau ex Rivas Mart. in Anales Inst. Bot. Cavanilles 21: 249. 1963.** Sec. Nieto Feliner (1987)

≡ *Armeria caespitosa* var. *bigerrensis* Vicioso & Beltrán in Bol. Real Soc. Esp. Hist. Nat. 13: 311. 1913 syn. sec. Domina (2011+) ≡ *Armeria splendens* subsp. *bigerrensis* (Vicioso & Beltrán) P.Silva in Bot. J. Linn. Soc. 64: 377. 1971 syn. sec. Malekmohammadi & al. (2024) − *Armeria biguerrensis* (C.Vicioso & Beltrán) Pau ex Rivas Mart., orth. var. syn. sec. Nieto Feliner (1987)

***Armeria bigerrensis* (C.Vicioso & Beltrán) Pau ex Rivas Mart. subsp. *bigerrensis*.** Sec. Domina (2011+)

***Armeria bigerrensis* subsp. *losae* (Bernis) Rivas Mart., T.E.Díaz, Fern.Prieto, Loidi & Penas, Veg. Picos Europa: 256. 1984.** Sec. Nieto Feliner (1987)

≡ *Armeria maritima* f. *losae* Bernis in Anales Inst. Bot. Cavanilles 14: 332. 1957 syn. sec. Malekmohammadi & al. (2024)

***Armeria bigerrensis* subsp. *microcephala* (Willk.) Nieto Fel. in Anales Jard. Bot. Madrid, ser. 2, 44: 343. 1987.** Sec. Nieto Feliner (1987)

≡ *Armeria alpina* var. *microcephala* Willk. in Willkomm & Lange, Prodr. Fl. Hispan. 2: 368. 1868 syn. sec. Nieto Feliner (1987) ≡ *Armeria alpina* subsp. *microcephala* (Willk.) Malag. in Acta Phytotax. Barcinon. 1: 23. 1968 ["1969"] syn. sec. Malekmohammadi & al. (2024)

***Armeria bourgaei* Boiss. ex Merino, Fl. Galicia 3: 585. 1909.** Sec. Malekmohammadi & al. (2024)

≡ *Armeria filicaulis* var. *bourgaei* (Boiss. ex Merino) Pau in Mem. Mus. Ci. Barcelona, ser. bot., 1(3): 32. 1925 syn. sec. POWO (2017+) ≡ *Armeria pseudarmeria* subsp. *bourgaei* (Boiss. ex Merino) Malag. in Acta Phytotax. Barcinon. 1: 26. 1968 syn. sec. POWO (2017+)
= *Armeria duriaei* subsp. *bourgaei* Boiss. ex Nyman, Consp. Fl. Eur. 3: 616. 1883 syn. sec. POWO (2017+) ≡ *Armeria maritima* subsp. *bourgaei* (Boiss. ex Nyman) Bernis in Bol. Soc. Brot., sér. 2, 23: 232. 1950 syn. sec. Domina (2011+) ≡ *Armeria maritima* var. *bourgaei* (Boiss. ex Nyman) Bernis, Rev. Gen. Armeria Comp.: 3. 1951

***Armeria bourgaei* Boiss. ex Merino subsp. *bourgaei*.** Sec. Nieto Feliner (1987)

= *Armeria mariae* Sennen, Diagn. Nouv.: 222. 1936 syn. sec. Nieto Feliner (1987)

***Armeria bourgaei* subsp. *lanceobracteata* (G.H.M.Lawr.) Nieto Fel. in Anales Jard. Bot. Madrid, ser. 2, 44: 336. 1987.** Sec. Nieto Feliner (1987)

≡ *Armeria lanceobracteata* G.H.M.Lawr. in Gentes Herbarum 4: 412. 1940 syn. sec. Nieto Feliner (1987)
− *Armeria duriaei* var. *seticaulis* Debeaux ex E.Rev. in Bull. Acad. Int. Geogr. Bot. 15: 160. 1905, nom. inval. syn. sec. Nieto Feliner (1987)

***Armeria bourgaei* subsp. *willkommiana* (Bernis) Nieto Fel. in Anales Jard. Bot. Madrid, ser. 2, 44: 336. 1987.** Sec. Nieto Feliner (1987)

≡ *Armeria maritima* var. *willkommiana* Bernis, Rev. Gen. Armeria Comp.: 13. 1951 syn. sec. Nieto Feliner (1987)

***Armeria brutia* Brullo, Gangale & Uzunov in Bot. Jahrb. Syst. 125: 465. 2004.** Sec. Arrigoni (2015)

***Armeria caballeroi* (Bernis) Donad. in Saussurea 11: 82. 1980.** Sec. Nieto Feliner (1987)

≡ *Armeria maritima* var. *caballeroi* Bernis, Rev. Gen. Armeria Comp.: 12. 1951 syn. sec. Nieto Feliner (1987)

***Armeria caespitosa* (Ortega) Boiss. in Candolle, Prodr. 12: 679. 1848.** Sec. Daveau (1888)

≡ *Statice caespitosa* Ortega, Fl. Españ. 6: 334. 1784 syn. sec. Boissier (1848);

***Armeria canescens* (Host) Boiss. in Candolle, Prodr. 12: 686. 1848.** Sec. Dimopoulos & al. (2013)

≡ *Statice canescens* Host, Fl. Austriaca 1: 407. 1827 syn. sec. Nyman (1881) ≡ *Armeria canescens* (Host) Ebel, Armeriae: 28. 1840 syn. sec. Arrigoni (2015) ≡ *Armeria maritima* var. *canescens* (Host) Bernis in Anales Inst. Bot. Cavanilles 11(2): 246. 1954 syn. sec. Arrigoni (2015)
= *Armeria denticulata* Portensch., Enum. Pl. Dalmatia: t. 6. 1824, nom. illeg. syn. sec. Nyman (1881)
= *Armeria gracilis* var. *humilis* Ten., Fl. Napol. 5: 223. 1835 syn. sec. Malekmohammadi & al. (2024)
= *Armeria petteriana* C.Presl in Abh. Königl. Böhm. Ges. Wiss. ser. 5, 3: 535. 1845 syn. sec. POWO (2017+: 9 May 2022)
= *Armeria canescens* var. *latifolia* Vis., Fl. Dalmat. 2: 6. 1847 syn. sec. Malekmohammadi & al. (2024) ≡ *Armeria canescens* f. *latifolia* (Vis.) Novák in Comm. Reg. Soc. Sci. Bohem. Mat. Nat. 27: 7. 1938 syn. sec. Malekmohammadi & al. (2024)
= *Armeria vulgaris* Vis., Fl. Dalmat. 2: 6. 1847 syn. sec. Nyman (1881)
= *Armeria orphanidis* Boiss., Diagn. Pl. Orient. ser. 2, 4: 71. 1859 syn. sec. POWO (2017+: 9 May 2022) ≡ *Armeria majellensis* subsp. *orphanidis* (Boiss.) Nyman, Consp. Fl. Eur. 3: 614. 1881 syn. sec. Malekmohammadi & al. (2024)
= *Armeria majellensis* var. *brachyphylla* Boiss., Fl. Orient. 4(2): 873. 1879 syn. sec. Malekmohammadi & al. (2024) ≡ *Armeria canescens* var. *brachyphylla* (Boiss.) G.H.M.Lawr. in Gentes Herbarum 4: 411. 1940 syn. sec. Malekmohammadi & al. (2024)
= *Armeria majellensis* var. *leucantha* Boiss., Fl. Orient. 4(2): 873. 1879 syn. sec. Malekmohammadi & al. (2024) ≡ *Armeria canescens* var. *leucantha* (Boiss.) G.H.M.Lawr. in Gentes Herbarum 4: 412. 1940 syn. sec. Malekmohammadi & al. (2024)
= *Armeria alpina* var. *lancifolia* Freyn in Verh. K.K. Zool.-Bot. Ges. Wien 38: 38. 1888 syn. sec. Malekmohammadi & al. (2024)
= *Armeria majellensis* var. *elatior* Levier ex Arch. in Atti Soc. Tosc. Sci. Nat. Pisa Processi Verbali 6: 157. 1888 syn. sec. Malekmohammadi & al. (2024)
= *Armeria canescens* f. *dasypoda* Murb. in Acta Univ. Lund. 27: 51. 1891 syn. sec. Malekmohammadi & al. (2024)
= *Armeria argyrocephala* f. *longifolia* Hausskn. in Mitth. Thüring. Bot. Vereins, n.f. 12: 54. 1897 syn. sec. Malekmohammadi & al. (2024)

= *Armeria argyrocephala* f. *minor* Hausskn. in Mitth. Thüring. Bot. Vereins, n.f. 12: 54. 1897 syn. sec. Malekmohammadi & al. (2024)
= *Armeria lacmonica* Hausskn. in Mitth. Thüring. Bot. Vereins n.f., 11: 55. 1897 syn. sec. Malekmohammadi & al. (2024)
= *Armeria majellensis* var. *stenophylla* Beck in Wiss. Mitt. Bosnien & Herzegovina 5: 485. 1897 syn. sec. Malekmohammadi & al. (2024)
= *Armeria argyrocephala* var. *graeca* Beck in Ann. K. K. Naturhist. Hofmus. 13(1): 17. 1898 syn. sec. Malekmohammadi & al. (2024)
= *Armeria canescens* f. *albanica* Beck in Ann. K. K. Naturhist. Hofmus. 13(1): 15. 1898 syn. sec. Malekmohammadi & al. (2024)
= *Armeria dalmatica* Beck in Ann. K. K. Naturhist. Hofmus. 13(1): 16. 1898 syn. sec. Pignatti (1972)
= *Armeria vulgaris* f. *tenorei* Fiori, Fl. Italia 2: 334. 1902 syn. sec. Malekmohammadi & al. (2024) ≡ *Armeria tenorei* (Fiori) Lacaita in Nuovo Giorn. Bot. Ital. n.s., 25: 15. 1918 syn. sec. POWO (2017+: 9 May 2022)
= *Armeria canescens* f. *dasyphylla* Rohlena in Repert. Spec. Nov. Regni Veg. 3: 148. 1906 syn. sec. Malekmohammadi & al. (2024)
= *Armeria pantocsekii* (Strobl) K.Malý in Glasn. Zemaljsk. Muz. Bosni Hercegovini 40: 113. 1928 syn. sec. Domina (2011+) ≡ *Armeria canescens* f. *pantoscekii* (Strobl) Novák in Comm. Reg. Soc. Sci. Bohem. Mat. Nat. 27: 8. 1938 syn. sec. Malekmohammadi & al. (2024)
= *Statice petteriana* (C.Presl) Degen, Fl. Veleb. 2: 539. 1937 syn. sec. Malekmohammadi & al. (2024)
= *Armeria canescens* f. *albiflora* Novák in Comm. Reg. Soc. Sci. Bohem. Mat. Nat. 27: 9. 1938 syn. sec. Malekmohammadi & al. (2024)
= *Armeria canescens* f. *angustifolia* Novák in Comm. Reg. Soc. Sci. Bohem. Mat. Nat. 27: 8. 1938 syn. sec. Malekmohammadi & al. (2024)
= *Armeria canescens* f. *dolichophylla* Novák in Comm. Reg. Soc. Sci. Bohem. Mat. Nat. 27: 16. 1938 syn. sec. Malekmohammadi & al. (2024)
= *Armeria canescens* f. *microphylla* Novák in Comm. Reg. Soc. Sci. Bohem. Mat. Nat. 27: 16. 1938 syn. sec. Malekmohammadi & al. (2024)
= *Armeria canescens* f. *platyphylla* Novák in Comm. Reg. Soc. Sci. Bohem. Mat. Nat. 27: 17. 1938 syn. sec. Malekmohammadi & al. (2024)
= *Armeria canescens* f. *pseudodalmatica* Novák in Comm. Reg. Soc. Sci. Bohem. Mat. Nat. 27: 17. 1938 syn. sec. Malekmohammadi & al. (2024)
= *Armeria canescens* f. *pubiscapa* Novák in Comm. Reg. Soc. Sci. Bohem. Mat. Nat. 27: 8. 1938 syn. sec. Malekmohammadi & al. (2024)
= *Armeria canescens* f. *rosea* Beck ex Novak in Comm. Reg. Soc. Sci. Bohem. Mat. Nat. 27: 12. 1938 syn. sec. Malekmohammadi & al. (2024)
= *Armeria canescens* f. *submajellensis* Novák in Comm. Reg. Soc. Sci. Bohem. Mat. Nat. 27: 9. 1938 syn. sec. Malekmohammadi & al. (2024)
= *Armeria canescens* var. *dalmatica* (Beck) Novák in Comm. Reg. Soc. Sci. Bohem. Mat. Nat. 27: 11. 1938 syn. sec. Malekmohammadi & al. (2024) ≡ *Armeria canescens* subsp. *dalmatica* (Beck) Trinajstić in Suppl. Fl. Anal. Jugosl 7: 6. 1980 syn. sec. Arrigoni (2015)
= *Armeria maritima* subsp. *smolikana* Babal. in Willdenowia 14(1): 61. 1984 syn. sec. Domina (2011+)
− *Armeria vulgaris* Moris ex Nyman, Consp. Fl. Eur. 3: 615. 1881, nom. inval. syn. sec. Malekmohammadi & al. (2024)

***Armeria canescens* (Host) Boiss. subsp. *canescens*.** Sec. Pignatti (1972)

= *Armeria denticulata* Tratt., Portenschl., Enum. Pl. Dalmatia: t. 6. 1824, nom. illeg. syn. sec. Malekmohammadi & al. (2024)

***Armeria canescens* subsp. *nebrodensis* (Guss.) P.Silva in Bot. J. Linn. Soc. 64: 376. 1971.** Sec. Pignatti (1972)

≡ *Statice nebrodensis* Guss., Fl. Sicul. Syn. 1: 366. 1843 syn. sec. Domina (2011+) ≡ *Armeria nebrodensis* (Guss.) Boiss. in Candolle, Prodr. 12: 685. 1848 syn. sec. Domina (2011+) ≡ *Armeria sardoa* var. *nebrodensis* (Guss.) Parl., Fl. Ital. 8: 596. 1889 syn. sec. Malekmohammadi & al. (2024)
= *Armeria alpina* Guss., Fl. Sicul. Prodr. 1: 378. 1827, nom. illeg. syn. sec. Malekmohammadi & al. (2024)
= *Armeria heterophylla* Wallr., Beitr. Bot. 2: 188. 1844 syn. sec. Malekmohammadi & al. (2024)
= *Armeria sicula* Heldr. ex Boiss. in Candolle, Prodr. 12: 685. 1848 syn. sec. Nyman (1881)

***Armeria cantabrica* Boiss. & Reut. ex Willk. & Lange, Prodr. Fl. Hispan. 2(2): 366. 1868.** Sec. Nieto Feliner (1987)

≡ *Statice cantabrica* (Boiss. & Reut. ex Willk. & Lange) P.Fourn., Quatre Fl. France: 724. 1937 syn. sec. Domina (2011+) ≡ *Armeria alpina* subsp. *cantabrica* (Boiss. & Reut. ex Willk. & Lange) Malag. in Acta Phytotax. Barcinon. 1: 23. 1968 ["1969"] syn. sec. Malekmohammadi & al. (2024)
= *Armeria vasconica* Sennen ex Losa in Bol. Soc. Iber. Ci. Nat. 29(6-8): 97. 1930 syn. sec. Domina (2011+) ≡ *Armeria alpina* subsp. *vasconica* (Sennen ex Losa) Malag. in Acta Phytotax. Barcinon. 1: 22. 1968 ["1969"] syn. sec. Malekmohammadi & al. (2024) ≡ *Armeria cantabrica* var. *vasconica* (Sennen ex Losa) López Fern. in Anales Inst. Bot. Cavanilles 29: 62. 1972 syn. sec. Malekmohammadi & al. (2024) ≡ *Armeria alpina* subsp. *vasconica* (Sennen ex Losa) Rivas Mart. & al., Veg. Picos Europa: 256. 1984 syn. sec. Domina (2011+) ≡ *Armeria cantabrica* subsp. *vasconica* (Sennen ex Losa) Uribe-Ech. in Estud. Mus. Cienc. Nat. Álava 6: 54. 1991 syn. sec. Domina (2011+) − *Armeria vasconica* Sennen, nom. inval. syn. sec. Nieto Feliner (1987)

= *Armeria maritima* f. *legionensis* Bernis in Anales Inst. Bot. Cavanilles 14: 333. 1957 syn. sec. Malekmohammadi & al. (2024) ≡ *Armeria maritima* subsp. *legionensis* (Bernis) M.Laínz in Bol. Inst. Estud. Adsturianos, Supl. Ci. 22: 22. 1976 syn. sec. Domina (2011+) ≡ *Armeria bigerrensis* subsp. *legionensis* (Bernis) Rivas Mart. & al., Veg. Picos Europa: 256. 1984 syn. sec. Malekmohammadi & al. (2024)
= *Armeria maritima* subvar. *gracilifolia* Bernis in Anales Inst. Bot. Cavanilles 14: 272. 1957 syn. sec. Domina (2011+) ≡ *Armeria cantabrica* subsp. *gracilifolia* (Bernis) Donad. in Saussurea 11: 77. 1980 syn. sec. Domina (2011+)
= *Armeria cantabrica* subvar. *asturica* (Boiss. & Reut. ex Willk. & Lange) Bernis syn. sec. Nieto Feliner (1987)

***Armeria capitella* Pau in Anales Soc. Esp. Hist. Nat. 1921(Extr): 296. 1921.** Sec. Nieto Feliner (1990)

≡ *Statice capitella* (Pau) Font Quer & Rothm., Sched. Fl. Iber Select., Cent. 2-3: 168. 1935 syn. sec. Nieto Feliner (1990: 3 May 2022) ≡ *Armeria undulata* var. *capitella* (Pau) Rivas Goday & Bellot in Anales Jard. Bot. Madrid 6(2): 150. 1948 syn. sec. Nieto Feliner (1990) ≡ *Armeria alliacea* subsp. *capitellata* (Pau) Rivas Mart. in Bull. Soc. Échange Pl. Vasc. Eur. Occid. Bassin Médit. 17: 44. 1979 syn. sec. Nieto Feliner (1990)

***Armeria caput-alba* (Rothm.) Rothm. in Cavanillesia 7: 118. 1935.** Sec. Malekmohammadi & al. (2024)

***Armeria cariensis* Boiss. in Candolle, Prodr. 12: 677. 1848.** Sec. Bokhari & Edmondson (1982)

= *Armeria alpina* Friv. ex Griseb., Spic. Fl. Rumel. 2: 296. 1846 ["1844"], nom. illeg. syn. sec. POWO (2017+: 8 May 2022)
– *Armeria alliacea* sensu Grisebach (1846), non (Cav.) Hoffmanns. & Link, err. sec. Malekmohammadi & al. (2024)

***Armeria cariensis* Boiss. var. *cariensis*.** Sec. Bokhari & Edmondson (1982)

***Armeria cariensis* var. *rumelica* (Boiss.) Boiss., Fl. Orient. 4(2): 874. 1879.** Sec. Bokhari & Edmondson (1982)

≡ *Armeria rumelica* Boiss. in Candolle, Prodr. 12: 677. 1848 syn. sec. Boissier (1879) ≡ *Statice rumelica* (Boiss.) Degen & Dörfl. in Denkschr. Kaiserl. Akad. Wiss., Wien. Math.-Naturwiss. Kl. 64: 734. 1897 syn. sec. POWO (2017+)
= *Armeria scorzonerifolia* Friv. ex Nyman, Consp. Fl. Eur. 3: 615. 1881 syn. sec. POWO (2017+)
= *Armeria majellensis* var. *rhodopaea* Velen. in Sitzungsber. Königl. Böhm. Ges. Wiss. Prag, Math.-Naturwiss. Cl. 29: 25. 1894 syn. sec. POWO (2017+) ≡ *Armeria rumelica* var. *rhodopaea* (Velen.) Beck in Ann. K. K. Naturhist. Hofmus. 13(1): 20. 1898 syn. sec. POWO (2017+) ≡ *Armeria rhodopea* Velen. in Sitzungsber. Königl. Böhm. Ges. Wiss., Math.-Naturwiss. Cl. 1902(27): 14. 1903 ["1902"] syn. sec. POWO (2017+) ≡ *Armeria rumelica* f. *rhodopaea* (Velen.) Hayek in Repert. Spec. Nov. Regni Veg. Beih. 30(2): 12. 1928 syn. sec. Govaerts, R. (ed.) (2023) ≡ *Armeria rumelica* f. *rhodopaea* (Velen.) Ančev in Fl. Narodna Republ. Bulg. 8: 348. 1982, nom. superfl. syn. sec. POWO (2017+: 7 May 2022)
= *Statice rumelica* var. *tempskyana* Degen & Dörfl. in Denkschr. Kaiserl. Akad. Wiss., Wien. Math.-Naturwiss. Kl. 64: 734. 1897 syn. sec. POWO (2017+) ≡ *Armeria rumelica* var. *tempskyana* (Degen & Dörfl.) Vandas, Reliq. Forman.: 502. 1909 syn. sec. POWO (2017+) − *Armeria rumelica* f. *temskyana* (Degen & Dörfl.) Micevski, Fl. Republ. Makedonija 1(3): 478. 1996, nom. inval. syn. sec. POWO (2017+)
= *Armeria adamovicii* Halácsy in Oesterr. Bot. Z. 56: 279. 1906 syn. sec. Domina (2011+) ≡ *Armeria rumelica* f. *adamovicii* (Halácsy) Hayek in Repert. Spec. Nov. Regni Veg. Beih. 30(2): 12. 1928 syn. sec. POWO (2017+) ≡ *Armeria rumelica* var. *adamovicii* (Halácsy) Novák in Comm. Reg. Soc. Sci. Bohem. Mat. Nat. 28: 19. 1939 syn. sec. POWO (2017+)
= *Armeria rumelica* f. *isophylla* Stoj. & Jordanov ex Novák in Comm. Reg. Soc. Sci. Bohem. Mat. Nat. 28: 12. 1939 syn. sec. POWO (2017+)
= *Armeria rumelica* f. *setacea* Novák in Comm. Reg. Soc. Sci. Bohem. Mat. Nat. 28: 12. 1939 syn. sec. POWO (2017+)
= *Armeria rumelica* var. *pseudocanescens* (Halácsy) Novák in Comm. Reg. Soc. Sci. Bohem. Mat. Nat. 28: 15. 1939 syn. sec. POWO (2017+)

***Armeria cariensis* var. *thessala* Boiss., Fl. Orient. 4(2): 874. 1879.** Sec. Bokhari & Edmondson (1982)

≡ *Armeria thessala* (Boiss.) Boiss. & Heldr., Diagn. Pl. Orient. ser. 2, 4: 70. 1859 syn. sec. Boissier (1879)

***Armeria ×carnotana* Blanco-Dios in Acta Bot. Malac. 37: 171. 2012.** Sec. Malekmohammadi & al. (2024)

Notes. – [A. beirana × A. pubigena]

***Armeria castellana* Boiss. & Reut. ex Rothm. in Bol. Real Soc. Esp. Hist. Nat. 34: 154. 1934.** Sec. Malekmohammadi & al. (2024)

≡ *Statice castellana* (Boiss. & Reut. ex Leresche) Rothm. in Bol. Real Soc. Esp. Hist. Nat. 34: 154. 1934 syn. sec. Malekmohammadi & al. (2024) ≡ *Armeria pseudarmeria* subsp. *castellata* (Boiss. & Reut. ex Leresche) Malag. in Acta Phytotax. Barcinon. 1: 24. 1968 ["1969"] syn. sec. Malekmohammadi & al. (2024)
− *Armeria castellana* Boiss. & Reut. ex Leresche in Deux Excurs. Bot.: 188. 1881, nom. inval. syn. sec. Malekmohammadi & al. (2024)

***Armeria castrovalnerana* Alejandre, Barredo & M.J.Escal. in Flora Montiber. 54: 123. 2013.** Sec. Malekmohammadi & al. (2024)

***Armeria castroviejoi* Nieto Fel. in Anales Jard. Bot. Madrid, ser. 2, 44: 330. 1987.** Sec. Nieto Feliner (1987)

***Armeria choulettiana* Pomel, Nouv. Mat. Fl. Atl. 1: 135. 1874.** Sec. Malekmohammadi & al. (2024)

≡ *Statice plantaginea* subsp. *choulettiana* (Pomel) Maire in Bull. Soc. Hist. Nat. Afrique N. 25: 309. 1934 syn. sec. Domina (2011+) ≡ *Armeria plantaginea* subsp. *choulettiana* (Pomel) Sauvage & Vindt in Trav. Inst. Sci. Chérifien, Sér. Bot. 4: 40. 1952 syn. sec. Domina (2011+)
= *Armeria longevaginata* Batt., Fl. Algérie [1](4): 738. 1890 syn. sec. POWO (2017+: 9 May 2022) − *Armeria longivaginata* Batt., Fl. Algérie [1](4): 738. 1890 syn. sec. Malekmohammadi & al. (2024) [is misspelling for *Armeria longevaginata* Batt.]
= *Statice villosa* var. *zaianica* Emberger, L. & Maire in Mém. Soc. Sci. Nat. Maroc 22: 42. 1930 ["1929"] syn. sec. Malekmohammadi & al. (2024) ≡ *Statice plantaginea* var. *zaianica* (Emberger, L. & Maire) Maire in Bull. Soc. Hist. Nat. Afrique N. 29: 435. 1938 syn. sec. Malekmohammadi & al. (2024)

= *Statice plantaginea* var. *barbata* Maire in Bull. Soc. Hist. Nat. Afrique N. 25: 309. 1934 syn. sec. Malekmohammadi & al. (2024)
= *Statice plantaginea* var. *brachylepis* (Batt.) Maire in Jahandiez & al., Cat. Pl. Maroc 3: 566. 1934 syn. sec. Malekmohammadi & al. (2024)
= *Statice plantaginea* var. *djurdjurae* Maire in Bull. Soc. Hist. Nat. Afrique N. 25: 309. 1934 syn. sec. Malekmohammadi & al. (2024)
= *Statice plantaginea* var. *microcephala* Maire in Bull. Soc. Hist. Nat. Afrique N. 30: 356. 1939 syn. sec. Malekmohammadi & al. (2024)
− *Armeria chouletteana* Pomel, Nouv. Mat. Fl. Atl.: 135. 1874 syn. sec. Malekmohammadi & al. (2024) [is misspelling for *Armeria choulettiana* Pomel]

***Armeria ciliata* (Lange) Nieto Fel. in Ruizia 2: 71. 1985.** Sec. Domina (2011+)

≡ *Armeria duriaei* var. *ciliata* Lange in Vidensk. Meddel. Dansk Naturhist. Foren. Kjøbenhavn 1881: 101. 1882 syn. sec. Nieto Feliner (1985) ≡ *Armeria pseudarmeria* subsp. *ciliata* (Lange) Malag. in Acta Phytotax. Barcinon. 1: 25. 1968 ["1969"] syn. sec. Malekmohammadi & al. (2024)

***Armeria ×cintrana* Taul.Gomes in Acta Bot. Malac. 36: 183. 2011.** Sec. Malekmohammadi & al. (2024)

***Armeria colorata* Pau in Mem. Mus. Ci. Nat. Barcelona, Ser. Bot., 1(1): 66. 1922.** Sec. Pignatti (1972)

***Armeria curvifolia* Bertero, Mercurio Chileno: 563. 1829.** Sec. POWO (2017+)

= *Statice caespitosa* Poir., Encycl. 7: 396. 1806 [as " *cespitosa* "], nom. illeg. syn. sec. Malekmohammadi & al. (2024) [non *Statice caespitosa* Ortega] − *Statice cespitosa* Poir., orth. var. syn. sec. Malekmohammadi & al. (2024) [is misspelling for *Statice caespitosa* Poir.]
= *Armeria macloviana* Cham. in Linnaea 6: 567. 1831 syn. sec. POWO (2017+: 10 May 2022) ≡ *Statice macloviana* (Cham.) Macloskie, Rep. Princeton Univ. Exped. Patagonia, Botany 8: 655. 1905 syn. sec. Malekmohammadi & al. (2024) ≡ *Armeria chilensis* subsp. *macloviana* (Cham.) Reiche, Fl. Chile 6: 104. 1911 syn. sec. Malekmohammadi & al. (2024) ≡ *Armeria chilensis* var. *macloviana* (Cham.) Reiche, Fl. Chile 6: 104. 1911 syn. sec. Malekmohammadi & al. (2024) ≡ *Armeria elongata* var. *macloviana* (Cham.) Skottsb. in Kongl. Svenska Vetensk. Acad. Handl., n.s., 56: 285. 1916 syn. sec. POWO (2017+: 9 May 2022) ≡ *Armeria maritima* var. *macloviana* (Cham.) G.H.M.Lawr. in Gentes Herbarum 4: 407. 1940 syn. sec. POWO (2017+: 9 May 2022)
= *Armeria curvifolia* Colla in Mem. Reale Accad. Sci. Torino 39: 5. 1835, nom. illeg. syn. sec. Malekmohammadi & al. (2024: 9 May 2022) ≡ *Armeria chilensis* var. *curvifolia* (Bertero) Boiss. in Candolle, Prodr. 12: 682. 1848 syn. sec. POWO (2017+: 9 May 2022) ≡ *Armeria maritima* var. *curvifolia* (Bertero) G.H.M.Lawr. in Gentes Herbarum 4: 407. 1940 syn. sec. POWO (2017+)
= *Armeria andicola* Gay ex Boiss. in Candolle, Prodr. 12: 682. 1848 syn. sec. POWO (2017+: 9 May 2022)
= *Armeria andina* Poepp. ex Boiss. in Candolle, Prodr. 12: 682. 1848 syn. sec. POWO (2017+: 9 May 2022) ≡ *Armeria chilensis* subsp. *andina* (Poepp. ex Boiss.) Reiche, Fl. Chile 6: 104. 1911 syn. sec. Malekmohammadi & al. (2024) ≡ *Armeria chilensis* var. *andina* (Poepp. ex Boiss.) Reiche, Fl. Chile 6: 104. 1911 syn. sec. Malekmohammadi & al. (2024) ≡ *Armeria macloviana* subsp. *andina* (Poepp. ex Boiss.) Iversen in Biol. Meddel. Kongel. Danske Vidensk. Selsk. 15(8): 18. 1940 syn. sec. POWO (2017+) ≡ *Armeria maritima* subsp. *andina* (Poepp. ex Boiss.) D.M.Moore & B.Yates in Bot. Not. 127: 191. 1974 syn. sec. POWO (2017+: 9 may 2022)
= *Armeria androsacea* Boiss. in Candolle, Prodr. 12: 679. 1848 syn. sec. POWO (2017+: 9 May 2022)
= *Armeria brachyphylla* Boiss. in Candolle, Prodr. 12: 682. 1848 syn. sec. POWO (2017+: 9 May 2022)
= *Armeria brevifolia* Kunze ex Boiss. in Candolle, Prodr. 12: 682. 1848 syn. sec. POWO (2017+: 9 May 2022)
= *Armeria chilensis* Boiss. in Candolle, Prodr. 12: 681. 1848 syn. sec. POWO (2017+: 9 May 2022) ≡ *Statice chilensis* (Boiss.) Macloskie, Rep. Princeton Univ. Exped. Patagonia, Botany 8: 655. 1905 syn. sec. Malekmohammadi & al. (2024)
= *Armeria chilensis* var. *brevifolia* Boiss. in Candolle, Prodr. 12: 682. 1848 syn. sec. POWO (2017+: 9 May 2022)
= *Armeria chilensis* var. *magellanica* Boiss. in Candolle, Prodr. 12: 682. 1848 syn. sec. POWO (2017+: 9 May 2022)
= *Armeria maculata* Poepp. ex Boiss. in Candolle, Prodr. 12: 682. 1848 syn. sec. POWO (2017+: 9 May 2022)
= *Armeria meridionalis* Poepp. ex Boiss. in Candolle, Prodr. 12: 682. 1848 syn. sec. POWO (2017+)
= *Armeria scabriuscula* Kunze ex Boiss. in Candolle, Prodr. 12: 682. 1848 syn. sec. POWO (2017+: 9 May 2022)
= *Armeria aegialea* Phil. in Anales Univ. Chile 91: 246. 1895 syn. sec. POWO (2017+: 9 May 2022)
= *Armeria delfinii* Phil. in Anales Univ. Chile 91: 246. 1895 syn. sec. POWO (2017+: 9 May 2022)
= *Armeria exaristata* Phil. in Anales Univ. Chile 91: 245. 1895 syn. sec. POWO (2017+: 9 May 2022)
= *Armeria patagonica* Phil. in Anales Univ. Chile 91: 244. 1895 syn. sec. POWO (2017+: 9 may 2022)
= *Armeria tenuifolia* Phil. in Anales Univ. Chile 91: 245. 1895 syn. sec. POWO (2017+: 9 May 2022)
= *Armeria bella* Albov in Revista Mus. La Plata, Secc. Bot. 7: 385. 1896 syn. sec. POWO (2017+: 9 May 2022) ≡ *Statice bella* (Albov) Macloskie, Rep. Princeton Univ. Exped. Patagonia, Botany 8: 655. 1905 syn. sec. Malekmohammadi & al. (2024) ≡ *Armeria chilensis* var. *bella* (Albov) Reiche, Fl. Chile 6: 104. 1911 syn. sec. POWO (2017+: 9 May 2022) ≡ *Armeria elongata* f. *bella* (Albov) Skottsb. in Kongl. Svenska Vetensk. Acad. Handl., n.s., 56: 286. 1916 syn. sec. POWO (2017+: 9 May 2022)
= *Statice punicea* Rendle in J. Bot. 42: 369. 1904 syn. sec. Malekmohammadi & al. (2024)
= *Armeria chilensis* var. *genuina* Reiche, Fl. Chile 6: 104. 1911 syn. sec. POWO (2017+: 9 May 2022)
= *Armeria elongata* var. *chilensis* (Boiss.) Skottsb. in Kongl. Svenska Vetensk. Acad. Handl. n.s., 56(5): 286. 1916 syn. sec. POWO (2017+: 9 May 2022)
= *Armeria maritima* var. *andina* (Poepp. ex Boiss.) G.H.M.Lawr. in Gentes Herbarum 4: 407. 1940 syn. sec. POWO (2017+)
= *Armeria maritima* var. *magellanica* (Boiss.) G.H.M.Lawr. in Gentes Herbarum 4: 407. 1940 syn. sec. Malekmohammadi & al. (2024)

= *Armeria maritima* var. *patagonica* (Phil.) G.H.M.Lawr. in Gentes Herbarum 4: 407. 1940 syn. sec. POWO (2017+)
= *Armeria maritima* var. *goodalliana* T.R.Dudley in Rhodora 83(836): 488. 1981 syn. sec. POWO (2017+)
− *Armeria chilensis* var. *majellanica* Boiss. in Candolle, Prodr. 12: 682. 1848 syn. sec. Malekmohammadi & al. (2024) [is misspelling for *Armeria chilensis* var. *magellanica* Boiss.]
− *Armeria scabra* Kunze ex Boiss. in Candolle, Prodr. 12: 682. 1848, nom. inval. syn. sec. POWO (2017+: 9 May 2022)

Armeria daveaui **(Cout.) P.Silva in Anuário Soc. Brot. 38: 170. 1972.** Sec. Domina (2011+)

≡ *Armeria alliacea* var. *daveaui* Cout., Fl. Portugal: 473. 1913 syn. sec. Domina (2011+) ≡ *Armeria langei* subsp. *deveaui* (Cout.) P.Silva in Agron. Lusit. 30: 219. 1970 syn. sec. Malekmohammadi & al. (2024); ≡ *Armeria daveaui* (Cout.) Rivas Mart. in Itinera Geobot. 18(2): 481. 2011, nom. superfl. syn. sec. Malekmohammadi & al. (2024) ≡ *Armeria daveaui* (Cout.) P.Silva subsp. *daveaui* syn. sec. Domina (2011+)
= *Armeria villosa* Cout. in Ann. Sci. Nat., Bot., ser. 3, 2: 323. 1844 syn. sec. Pignatti (1972)

Armeria denticulata **(Bertol.) DC., Cat. Pl. Horti Monsp.: 7. 1813.** Sec. Pignatti (1972)

≡ *Statice denticulata* Bertol., Rar. Lig. [Ital.] Pl. 2: 34. 1806 syn. sec. Arrigoni (2015) ≡ *Armeria denticulata* (Bertol.) Bertol., Amoen. Ital.: 77. 1819 syn. sec. Arrigoni (2015) ≡ *Armeria vulgaris* var. *denticulata* (Bertol.) Fiori in Fiori & Bèg., Fl. Anal. Ital. 2: 334. 1902 syn. sec. Pignatti (1972) ≡ *Armeria maritima* var. *denticulata* (Bertol.) Bernis in Anales Inst. Bot. Cavanilles 11(2): 252. 1954 syn. sec. Arrigoni (2015)

Armeria duriaei **Boiss. in Candolle, Prodr. 12: 684. 1848.** Sec. Nieto Feliner (1987)

≡ *Statice nebrodensis* var. *duriaei* (Boiss.) Samp., Fl. Port.: 441. 1947 syn. sec. Sampaio (1913)
= *Armeria juniperifolia* J.Gay ex Boiss. in Candolle, Prodr. 12: 684. 1848 syn. sec. Nyman (1881)
= *Statice asturiana* Rothm. in Bol. Real Soc. Esp. Hist. Nat. 34: 154. 1934 syn. sec. Malekmohammadi & al. (2024)

Armeria ebracteata **Pomel, Nouv. Mat. Fl. Atl. 1: 132. 1874.** Sec. Domina (2011+)

≡ *Statice ebracteata* (Pomel) Maire in Bull. Soc. Hist. Nat. Afrique N. 22: 56. 1931 syn. sec. Domina (2011+)
= *Armeria lachnolepis* Pomel, Nouv. Mat. Fl. Atl. 1: 134. 1874 syn. sec. Domina (2011+) ≡ *Statice lachnolepis* (Pomel) Maire in Bull. Soc. Hist. Nat. Afrique N. 22: 304. 1931 syn. sec. Malekmohammadi & al. (2024)
= *Armeria ebracteata* var. *laevis* Maire in Mém. Soc. Sci. Nat. Maroc 21-22: 11. 1929 syn. sec. Malekmohammadi & al. (2024) ≡ *Statice ebracteata* var. *laevis* (Maire) Maire in Jahandiez & al., Cat. Pl. Maroc 3: 567. 1934 syn. sec. Malekmohammadi & al. (2024)

Armeria eriophylla **Willk. in Bol. Soc. Brot. 2: 145. 1884.** Sec. Nieto Feliner (1987)

≡ *Statice juncea* race *eriophylla* (Willk.) Samp., Herb. Port.: 101. 1913 syn. sec. Sampaio (1913) ≡ *Statice eriophylla* (Willk.) Samp., Herb. Portug. 1: 9. 1914 syn. sec. Domina (2011+) ≡ *Armeria pseudarmeria* subsp. *eriophylla* (Willk.) Malag. in Acta Phytotax. Barcinon. 1: 25. 1968 ["1969"] syn. sec. Malekmohammadi & al. (2024)
= *Statice echinata* Schult. in Roemer & Schultes, Syst. Veg. ed. 15[bis] 6: 799. 1820 syn. sec. Malekmohammadi & al. (2024)
− *Armeria eriophila* Willk. in Bol. Soc. Brot. 2: 145. 1884 syn. sec. Malekmohammadi & al. (2024) [is misspelling for *Armeria eriophylla* Willk.]

Armeria euscadiensis **Donad. & Vivant in Bull. Soc. Bot. France 123: 562. 1976.** Sec. Nieto Feliner (1987)

≡ *Armeria cantabrica* var. *maritima* Rouy, Ill. Pl. Eur. 2: 14. 1895 syn. sec. Nieto Feliner (1987)

Armeria filicaulis **(Boiss.) Boiss., Voy. Bot. Espagne 2: 527. 1845.** Sec. Nieto Feliner (1987)

≡ *Statice filicaulis* Boiss., Elench. Pl. Nov.: 80. 1838 syn. sec. Domina (2011+) ≡ *Statice littoralis* subsp. *filicaulis* (Boiss.) P.Fourn., Quatre Fl. France: 724. 1937 syn. sec. Domina (2011+)
= *Armeria juniperifolia* Ebel, Armeriae: 30. 1840 syn. sec. POWO (2017+: 8 May 2022)
= *Armeria tenuis* Wallr., Beitr. Bot. 2: 184. 1844 syn. sec. Nyman (1881)
= *Armeria tenuis* var. *elata* Wallr., Beitr. Bot. 2: 184. 1844 syn. sec. Malekmohammadi & al. (2024: 8 May 2022)
= *Armeria filicaulis* var. *major* Boiss., Voy. Bot. Espagne 2: 527. 1845 syn. sec. Malekmohammadi & al. (2024: 8 May 2022)
= *Armeria filicaulis* var. *bourgeaui* Pau in Mem. Mus. Ci. Nat. Barcelona, Ser. Bot., 1(1): 66. 1922 syn. sec. Malekmohammadi & al. (2024: 8 May 2022)
= *Armeria filicaulis* var. *maroccana* Pau & Font Quer, Iter Marocc. 1928: 310. 1929 syn. sec. Malekmohammadi & al. (2024: 8 May 2022) ≡ *Statice filicaulis* var. *maroccana* (Pau & Font Quer) Maire in Jahandiez & al., Cat. Pl. Maroc 3: 566. 1934 syn. sec. Malekmohammadi & al. (2024: 8 May 2022) ≡ *Armeria maritima* var. *maroccana* (Font Quer) G.H.M.Lawr. in Gentes Herbarum 4: 405. 1940 syn. sec. Malekmohammadi & al. (2024)
= *Statice filicaulis* (Boiss.) Jahand. & Maire in Jahandiez & al., Cat. Pl. Maroc 3: 566. 1934 syn. sec. Malekmohammadi & al. (2024: 8 May 2022)
= *Armeria littoralis* var. *ancarensis* (Merino) G.H.M.Lawr. in Gentes Herbarum 4: 413. 1940 syn. sec. Malekmohammadi & al. (2024: 8 May 2022)
= *Armeria filicaulis* subsp. *willkommiana* (Bernis) Molero in Collect. Bot. (Barcelona) 16: 154. 1985 syn. sec. Malekmohammadi & al. (2024)
= *Armeria littoralis* H.J.Coste syn. sec. Domina (2011+)

Armeria filicaulis **subsp.** ***alfacarensis*** **Nieto Fel., Gut.Larena & Fuertes in Anales Jard. Bot. Madrid 61(1): 45. 2004.** Sec. Domina (2011+)

Armeria filicaulis **(Boiss.) Boiss. subsp.** ***filicaulis*****.** Sec. Domina (2011+)

= *Armeria ancarensis* Merino in Bol. Soc. Esp. Hist. Nat. 3: 155. 1903 syn. sec. POWO (2017+: 9 May 2022) ≡ *Statice ancarensis* (Merino) Rothm. in Cavanillesia 7: 118. 1935 syn. sec. Malekmohammadi & al. (2024: 9 May 2022)

***Armeria filicaulis* subsp. *nevadensis* Nieto Fel., Rosselló & Fuertes in Anales Jard. Bot. Madrid 56(1): 163. 1998.** Sec. Domina (2011+)

***Armeria filicaulis* subsp. *trevenqueana* Nieto Fel. in Anales Jard. Bot. Madrid 47(1): 271. 1990 ["1989"].** Sec. Domina (2011+)

***Armeria filicaulis* subsp. *valentina* (Pau ex C.Vicioso) Mateo in Toll Negre 6: 21. 2005.** Sec. Domina (2011+)

≡ *Armeria filicaulis* var. *valentina* Pau ex C.Vicioso in Bol. Real Soc. Esp. Hist. Nat. 16: 139. 1916 syn. sec. Domina (2011+)

***Armeria fontqueri* Pau in Treb. Inst. Catalana Hist. Nat. 1915: 30. 1916.** Sec. Nieto Feliner (1987)

≡ *Statice dertosensis* Font Quer & Rothm., Sched. Fl. Iber Select., Cent. 2-3: 171. 1935 syn. sec. Nieto Feliner (1987) ≡ *Armeria alpina* subsp. *fontqueri* (Pau) Malag. in Acta Phytotax. Barcinon. 1: 22. 1968 ["1969"] syn. sec. Domina (2011+)

***Armeria gaditana* Boiss. in Candolle, Prodr. 12: 675. 1848.** Sec. Pignatti (1972)

≡ *Statice major* race *gaditana* (Boiss.) Samp., Herb. Port.: 101. 1913 syn. sec. Sampaio (1913) ≡ *Statice gaditana* (Boiss.) Jahand. & Maire in Jahandiez & al., Cat. Pl. Maroc 3: 565. 1934 syn. sec. Domina (2011+) − *Statice gaditana* (Boiss.) Samp., Fl. Port.: 442. 1947, nom. inval. syn. sec. Domina (2011+: 26 April 2022)
− *Statice pseudoarmeria* Cav., Icon. 1: 38. 1791, nom. inval. syn. sec. Boissier (1848)

***Armeria genesiana* Nieto Fel. in Anales Jard. Bot. Madrid, ser. 2, 44: 332. 1987.** Sec. Nieto Feliner (1987)

***Armeria genesiana* subsp. *belmonteae* (P.Silva) Nieto Fel. in Anales Jard. Bot. Madrid, ser. 2, 44: 333. 1987 [as " *belmontei* "].** Sec. Nieto Feliner (1987)

≡ *Armeria langei* subsp. *belmonteae* P.Silva in Lazaroa 5: 181. 1984 [as " *belmontei* "] syn. sec. Nieto Feliner (1987) − *Armeria langei* subsp. *belmontei* P.Silva in Lazaroa 5: 181. 1984, orth. var. syn. sec. Malekmohammadi & al. (2024) − *Armeria genesiana* subsp. *belmontei* (P.Silva) Nieto Fel. in Anales Jard. Bot. Madrid, ser. 2, 44: 333. 1987, orth. var. syn. sec. Malekmohammadi & al. (2024)

Notes. – This name was published honouring a woman, Dr. Dolores Belmonte, the epithet is to be corrected to “belmonteae” in all cases without any authority correction

***Armeria genesiana* Nieto Fel. subsp. *genesiana*.** Sec. Nieto Feliner (1987)

***Armeria girardii* (Bernis) Litard., Prodr. Fl. Corse 3(2): 4. 1955.** Sec. Pignatti (1972)

≡ *Armeria maritima* var. *girardii* Bernis in Anales Inst. Bot. Cavanilles 11(2): 253. 1954 syn. sec. Domina (2011+) − *Armeria girardi* (Bernis) Litard., Prodr. Fl. Corse 3(2): 4. 1955, orth. var. syn. sec. Malekmohammadi & al. (2024) [is misspelling for *Armeria girardii* (Bernis) Litard.]
= *Armeria juncea* Girard in Ann. Sci. Nat., Bot., sér. 3, 2: 324. 1844, nom. illeg. syn. sec. Pignatti (1972) ≡ *Statice juncea* F.T.Hubb. in Rhodora 18(211): 157. 1916 syn. sec. Malekmohammadi & al. (2024)
= *Armeria filicaulis* var. *minor* Boiss., Voy. Bot. Espagne 2: 527. 1845 syn. sec. Malekmohammadi & al. (2024)
= *Statice littoralis* subsp. *juncea* P.Fourn. syn. sec. Domina (2011+)
− *Armeria setacea* Delile ex Nyman, Consp. Fl. Eur. 3: 616. 1881, nom. inval. syn. sec. Malekmohammadi & al. (2024)

***Armeria godayana* Font Quer, Fl. Hisp. Herb. 5: 6. 1948.** Sec. Nieto Feliner (1987)

≡ *Armeria alpina* subsp. *godayana* (Font Quer) Malag. in Acta Phytotax. Barcinon. 1: 22. 1968 ["1969"] syn. sec. Malekmohammadi & al. (2024: 7 May 2022)

***Armeria gracilis* Ten., Syll. Pl. Fl. Neapol.: 158. 1831.** Sec. Arrigoni (2015)

≡ *Armeria canescens* subsp. *gracilis* (Ten.) Bianchini in Giorn. Bot. Ital. 111: 49. 1977 syn. sec. Arrigoni (2015)
= *Armeria majellensis* var. *subalpina* Levier in Arcang. Atti Soc. Tosc. Sci. Nat. Proc. Verb. 6: 154. 1888 syn. sec. Arrigoni (2015)
= *Armeria majellensis* subsp. *ausonia* Bianchini in Giorn. Bot. Ital. 111: 49. 1977 syn. sec. Arrigoni (2015)

***Armeria gracilis* Ten. subsp. *gracilis*.** Sec. Arrigoni (2015)

***Armeria gracilis* subsp. *majellensis* (Boiss.) Arrigoni in Fl. Medit. 25(Special issue): 22. 2015.** Sec. Arrigoni (2015)

= *Armeria majellensis* Boiss. in Candolle, Prodr. 12: 685. 1848 syn. sec. Arrigoni (2015); ≡ *Armeria alpina* Ten. ex Boiss. in Candolle, Prodr. 12: 685. 1848, nom. illeg. syn. sec. Arrigoni (2015) ≡ *Statice majellensis* (Boiss.) F.T.Hubb. in Rhodora 18(211): 157. 1916 syn. sec. Malekmohammadi & al. (2024: 5 May 2022) ≡ *Armeria maritima* var. *majellensis* (Boiss.) Bernis in Anales Inst. Bot. Cavanilles 11(2): 247. 1954 syn. sec. Arrigoni (2015) ≡ *Armeria majellensis* subsp. *majellensis* syn. sec. Malekmohammadi & al. (2024) ≡ *Statice majellensis* subsp. *majellensis* syn. sec. Malekmohammadi & al. (2024)

***Armeria grajoana* Casim.-Sor.Solanas & Cabezudo in Acta Bot. Malac. 40: 60. 2015.** Sec. Cabezudo & al. (2015)

***Armeria helodes* F.Martini & Poldini in Candollea 42: 537. 1987.** Sec. Arrigoni (2015)

***Armeria hirta* Willd., Enum. Pl.: 333. 1809.** Sec. Nieto Feliner (1987)

≡ *Statice hirta* (Willd.) Steud., Nomencl. Bot. 1: 811. 1821 syn. sec. Malekmohammadi & al. (2024) ≡ *Statice hirta* (Willd.) Rothm. in Cavanillesia 7: 117. 1935 syn. sec. Malekmohammadi & al. (2024)
= *Armeria plantaginea* Boiss., Voy. Bot. Espagne 2: 526. 1841 syn. sec. POWO (2017+: 9 May 2022)
= *Armeria glauca* Wallr., Beitr. Bot. 2: 215. 1844 syn. sec. Domina (2011+) ≡ *Armeria hirta* var. *glauca* (Wallr.) Bernis in Anales Inst. Bot. Cavanilles 12(2): 113. 1955 syn. sec. Malekmohammadi & al. (2024)

= *Armeria baetica* Boiss., Voy. Bot. Espagne 2: 749. 1845 [as " *boetica* "] syn. sec. Domina (2011+) ≡ *Statice baetica* (Boiss.) Font Quer & Rothm., Sched. Fl. Iber. Select. Cent. 2-3: n. 165. 1935 syn. sec. Domina (2011+) ≡ *Statice hirta* subsp. *baetica* (Boiss.) Rothm. in Cavanillesia 7: 118. 1935 syn. sec. Malekmohammadi & al. (2024) − *Armeria boetica* Boiss., Voy. Bot. Espagne 2: 749. 1845, orth. var. syn. sec. Malekmohammadi & al. (2024) [is misspelling for *Armeria baetica* Boiss.] − *Reverchonia baetica* (Boiss.) Gand., Fl. Eur. 19: 19. 1890, nom. inval. syn. sec. Nieto Feliner (1987)
= *Armeria grosii* Pau in Mem. Mus. Ci. Nat. Barcelona, Ser. Bot., 1(1): 67. 1922 syn. sec. POWO (2017+: 9 May 2022)
= *Statice caput-alba* Rothm. in Cavanillesia 7: 118. 1935 syn. sec. Malekmohammadi & al. (2024)

***Armeria hirta* Willd. var. *hirta*.** Sec. Malekmohammadi & al. (2024)

***Armeria hirta* var. *perplexans* Bernis in Anales Inst. Bot. Cavanilles 11(2): 199. 1954.** Sec. Bernis (1954)

***Armeria hispalensis* Pau, Not. Bot. Fl. Esp. 6: 89. 1895.** Sec. Nieto Feliner (1987)

≡ *Armeria hirta* subsp. *hispalensis* (Pau) Malag. in Acta Phytotax. Barcinon. 1: 23. 1968 ["1969"] syn. sec. Malekmohammadi & al. (2024)

***Armeria humilis* (Link) Schult. in Roemer & Schultes, Syst. Veg. ed. 15[bis] 6: 722. 1820.** Sec. Pignatti (1972)

***Armeria humilis* (Link) Schult. subsp. *humilis*.** Sec. Pignatti (1972)

= *Statice juniperifolia* Vahl, Symb. Bot. 1: 25. 1790, nom. illeg. syn. sec. Boissier (1848)
= *Statice caespitosa* Cav., Icon. 1: 38. 1791 syn. sec. Malekmohammadi & al. (2024) ≡ *Armeria cespitosa* (Cav.) Boiss. in Candolle, Prodr. 12: 679. 1848 syn. sec. Malekmohammadi & al. (2024)
= *Armeria willkommii* Henriq. in Bol. Soc. Brot. 3: 199. 1884 syn. sec. Daveau (1888: synonym of Statice humilis Link)
= *Armeria caespitosa* var. *isernii* Vicioso & Beltrán in Bol. Real Soc. Esp. Hist. Nat. 13: 310. 1913 syn. sec. Malekmohammadi & al. (2024) ≡ *Statice isernii* (Vicioso & Beltrán) Font Quer & Rothm., Sched. Fl. Iber Select., Cent. 2-3: 174. 1935 syn. sec. Malekmohammadi & al. (2024) ≡ *Armeria juniperifolia* var. *isernii* (Vicioso & Beltrán) G.H.M.Lawr. in Gentes Herbarum 4: 408. 1940 syn. sec. Malekmohammadi & al. (2024)
= *Armeria juniperifolia* var. *bigerrensis* (Vicioso & Beltrán) G.H.M.Lawr. in Gentes Herbarum 4: 408. 1940 syn. sec. Malekmohammadi & al. (2024)
= *Armeria cespitosa* subsp. *cespitosa* syn. sec. Malekmohammadi & al. (2024)
= *Armeria juniperifolia* Hoffmanns. & Link syn. sec. Malekmohammadi & al. (2024)
− *Armeria juniperifolia* (Vahl) Hoffmanns. & Link in Flora 6: 711. 1823, nom. rej. syn. sec. Malekmohammadi & al. (2024: synonym of Armeria willkommii)

***Armeria humilis* subsp. *odorata* (Samp.) P.Silva in Bot. J. Linn. Soc. 64: 377. 1971.** Sec. Pignatti (1972)

≡ *Armeria willkommii* var. *odorata* Samp. in Ann. Sci. Nat. (Oporto) 8: 15. 1903 syn. sec. Nieto Feliner (1987) ≡ *Statice humilis* var. *odorata* (Samp.) Samp., Man. Fl. Port.: 364. 1912 syn. sec. Pignatti (1972: 26 April 2022) ≡ *Armeria maritima* subsp. *odorata* (Samp.) Bernis syn. sec. Malekmohammadi & al. (2024)
= *Armeria odorata* Samp. syn. sec. Malekmohammadi & al. (2024)

***Armeria icarica* Edm. in 38: 59. 1980.** Sec. Bokhari & Edmondson (1982)

***Armeria johnsenii* Papan. & Kokkini in Willdenowia 12: 221. 1982.** Sec. Domina (2011+)

***Armeria langei* Boiss. ex Lange in Vidensk. Meddel. Dansk Naturhist. Foren. Kjøbenhavn 1861: 59. 1861.** Sec. Nieto Feliner (1987)

≡ *Statice langei* (Boiss.) Rothm. in Bol. Real Soc. Esp. Hist. Nat. 34: 153. 1934 syn. sec. Malekmohammadi & al. (2024) ≡ *Armeria langei* Boiss. subsp. *langei* syn. sec. Malekmohammadi & al. (2024)
= *Armeria eriophylla* var. *marizii* Daveau in Bol. Soc. Brot. 6: 174. 1888 syn. sec. Malekmohammadi & al. (2024) ≡ *Statice filicaulis* var. *marizii* (Daveau) Samp., Fl. Port.: 441. 1947 syn. sec. Sampaio (1913: synonym of Armeria eriophylla var. marizii Daveau)

***Armeria leonis* Sennen, Diagn. Nouv.: 287. 1936.** Sec. Malekmohammadi & al. (2024)

≡ *Statice leonis* Sennen, Diagn. Nouv.: 287. 1936 syn. sec. Malekmohammadi & al. (2024: 14 April 2022)
= *Armeria maritima* var. *viciosoi* Bernis, Rev. Gen. Armeria Comp.: 10. 1951 syn. sec. Malekmohammadi & al. (2024)

***Armeria leucocephala* Salzm. ex W.D.J.Koch in Flora 6: 712. 1823.** Sec. Tiburtini & al. (2023)

≡ *Statice leucocephala* (Salzm. ex W.D.J.Koch) F.T.Hubb. in Rhodora 18(211): 157. 1916 syn. sec. Bailey (1916) ≡ *Armeria maritima* var. *leucocephala* (Salzm. ex W.D.J.Koch) Bernis, Rev. Gen. Armeria Comp.: C. 1951 syn. sec. Malekmohammadi & al. (2024)
= *Armeria juniperifolia* W.D.J.Koch in Flora 6: 711. 1823 syn. sec. POWO (2017+: 4 May 2022)
= *Statice leucantha* Pouzolz in Mém. Soc. Linn. Paris 4: 560. 1826 syn. sec. Malekmohammadi & al. (2024: 4 May 2022)
= *Statice pubescens* Salis in Flora 17(2): 13. 1834 syn. sec. Domina (2011+) ≡ *Statice armeria* var. *pubescens* Salis in Flora 17(2): 13. 1834 syn. sec. Malekmohammadi & al. (2024) ≡ *Armeria leucocephala* var. *pubescens* (Salis) G.H.M.Lawr. in Gentes Herbarum 4: 417. 1940 syn. sec. Malekmohammadi & al. (2024) ≡ *Armeria leucocephala* subsp. *pubescens* (Salis) Arrigoni in Webbia 25: 160. 1970 syn. sec. Tiburtini & al. (2023)
= *Armeria alliacea* var. *pubescens* Mutel, A., Fl. Franç. 3: 86. 1836 syn. sec. Pignatti (1972)
= *Armeria multiceps* Wallr., Beitr. Bot. 2: 196. 1844 syn. sec. Tiburtini & al. (2023) ≡ *Armeria leucocephala* var. *multiceps* (Wallr.) Mori, Fl. Ital. 8: 592. 1889 syn. sec. Malekmohammadi & al. (2024) ≡ *Armeria leucocephala* subsp. *multiceps* (Wallr.) Arcang., Comp. Fl. Ital. ed. 2: 460. 1894 syn. sec. Malekmohammadi & al. (2024) ≡ *Armeria maritima* var. *multiceps* (Wallr.) Bernis, Rev. Gen. Armeria Comp.: C. 1951 syn. sec. Malekmohammadi & al. (2024)
= *Armeria kochii* Boiss. in Candolle, Prodr. 12: 686. 1848 syn. sec. Malekmohammadi & al. (2024)
= *Armeria leucocephala* var. *alpina* Boiss. in Candolle, Prodr. 12: 687. 1848 syn. sec. Malekmohammadi & al. (2024)
= *Armeria leucocephala* var. *procera* Boiss. in Candolle, Prodr. 12: 687. 1848 syn. sec. Malekmohammadi & al. (2024)

= *Statice montana* Soleirol ex Boiss. in Candolle, Prodr. 12: 686. 1848 syn. sec. Boissier (1848)
= *Armeria thomasii* Nyman, Consp. Fl. Eur. 3: 615. 1881 syn. sec. Domina (2011+) ≡ *Armeria leucocephala* subsp. *thomasii* Nyman, Consp. Fl. Eur. 3: 651. 1881 syn. sec. Domina (2011+)
= *Armeria leucocephala* var. *glabra* Fiori, Fl. Italia 2: 235. 1902 syn. sec. Malekmohammadi & al. (2024)
= *Armeria leucocephala* var. *hirticula* Boenn. in Bull. Soc. Bot. France 67: 263. 1920 syn. sec. Malekmohammadi & al. (2024)
= *Statice pusilla* (Salis) P.Fourn., Quatre Fl. France: 723. 1937 syn. sec. Malekmohammadi & al. (2024)
= *Armeria leucocephala* var. *kochii* (Boiss.) G.H.M.Lawr. in Gentes Herbarum 4: 416. 1940 syn. sec. Malekmohammadi & al. (2024)
= *Armeria leucocephala* subsp. *breviaristata* Arrigoni in Webbia 25: 159. 1970 syn. sec. Domina (2011+)
= *Armeria multiceps* subsp. *meridionalis* Arrigoni in Webbia 25: 152. 1970 syn. sec. Tiburtini & al. (2023)
= *Armeria leucocephala* subsp. *leucocephala* syn. sec. Domina (2011+)
= *Armeria multiceps* subsp. *multiceps* syn. sec. Domina (2011+)

Armeria linkiana **Nieto Fel. in Anales Jard. Bot. Madrid, ser. 2, 44: 332. 1987.** Sec. Domina (2011+)

≡ *Armeria littoralis* Hoffmanns. & Link, Fl. Portug. 1: 441. 1817 syn. sec. Nieto Feliner (1987) ≡ *Armeria pseudarmeria* subsp. *littoralis* (Hoffmanns. & Link) Malag. in Acta Phytotax. Barcinon. 1: 26. 1968 ["1969"] syn. sec. Malekmohammadi & al. (2024)
= *Statice littoralis* Poir., Encycl. Suppl. 5: 235. 1817 syn. sec. Malekmohammadi & al. (2024)
= *Armeria littoralis* var. *hispida* Daveau in Bol. Soc. Brot. 6: 173. 1888 syn. sec. Malekmohammadi & al. (2024)
= *Statice littoralis* P.Fourn., Quatre Fl. France: 724. 1937, nom. illeg. syn. sec. Domina (2011+)
− *Armeria microcephala* Welw., nom. inval. syn. sec. Malekmohammadi & al. (2024: synonym of Armeria littoralis Hoffmanns. & Link)

Armeria littoralis **Willd., Enum. Pl.: 333. 1809.** Sec. Domina (2011+)

≡ *Statice littoralis* (Willd.) Steud. syn. sec. Domina (2011+) ≡ *Statice littoralis* (Willd.) Steud. subsp. *littoralis* syn. sec. Domina (2011+)
= *Armeria neglecta* Girard in Ann. Sci. Nat., Bot., sér. 3, 2: 324. 1844 syn. sec. Domina (2011+) ≡ *Statice neglecta* (Girard) Samp., Herb. Port.: 100. 1913 syn. sec. Sampaio (1913)
= *Statice davaei* (Samp.) Samp., Fl. Port.: 441. 1947 syn. sec. Domina (2011+) − *Statice littoralis* var. *davaei* Samp., Herb. Port.: 101. 1913, nom. inval. syn. sec. Malekmohammadi & al. (2024: 26 April 2022)

Armeria macrophylla **Boiss. & Reut., Pugill. Pl. Afr. Bor. Hispan.: 100. 1852.** Sec. Pignatti (1972)

≡ *Statice macrophylla* (Boiss. & Reut.) Font Quer & Rothm., Sched. Fl. Iber Select., Cent. 2-3: 167. 1935 syn. sec. Malekmohammadi & al. (2024) ≡ *Statice macrophylla* (Boiss. & Reut.) Rothm. in Ind. Sem. Ann., Coll. Agron. Nat. Lusit.: 6. 1939 ["1940"] syn. sec. Malekmohammadi & al. (2024) ≡ *Armeria pinifolia* subsp. *macrophylla* (Boiss. & Reut.) Bernis syn. sec. Malekmohammadi & al. (2024)
= *Armeria baetica* var. *stenophylla* Boiss. in Candolle, Prodr. 12: 677. 1848 syn. sec. Daveau (1888)
= *Armeria pinifolia* Bourg. ex Nyman, Consp. Fl. Eur. 3: 614. 1881 syn. sec. Daveau (1888)

Armeria macropoda **Boiss. in Candolle, Prodr. 12: 688. 1848.** Sec. Pignatti (1972)

≡ *Armeria morisii* var. *macropoda* (Boiss.) Parl., Fl. Ital. 8: 600. 1889 syn. sec. Pignatti (1972) ≡ *Armeria maritima* var. *macropoda* (Boiss.) Bernis in Anales Inst. Bot. Cavanilles 11(2): 246. 1954 syn. sec. Arrigoni (2015)
= *Armeria scorzonerifolia* Ten., Syll. Pl. Fl. Neapol.: 160. 1831 syn. sec. Nyman (1881)
= *Armeria garganica* Arrigoni in Fl. Medit. 25(Special issue): 25. 2015 syn. sec. Malekmohammadi & al. (2024)
− *Statice alliacea* Guss. ex Nyman, Consp. Fl. Eur. 3: 614. 1881, in sched. syn. sec. Nyman (1881) [non *Statice alliacea* Cav.]

Armeria maderensis **Lowe in Trans. Cambridge Philos. Soc. 6: 12. 1833.** Sec. Domina (2011+)

≡ *Armeria morisii* var. *maderensis* (Lowe ex Boiss.) G.H.M.Lawr. in Gentes Herbarum 4: 418. 1940 syn. sec. Malekmohammadi & al. (2024) ≡ *Armeria maritima* subsp. *maderensis* (Loew) Bernis syn. sec. Domina (2011+)

Armeria magna **Sennen in Bull. Soc. Bot. France 73: 673. 1927.** Sec. Sennen (1927)

≡ *Armeria plantaginea* subsp. *magna* (Sennen) Malag. in Acta Phytotax. Barcinon. 1: 25. 1968 ["1969"] syn. sec. Malekmohammadi & al. (2024)
− *Armeria rigida* f. *elongata* Sennen, in sched. syn. sec. Malagarriga (1968)

Armeria malacitana **Nieto Fel. in Anales Jard. Bot. Madrid, ser. 2, 44: 338. 1987.** Sec. Nieto Feliner (1987)

≡ *Armeria filicaulis* var. *longifolia* Willk., Suppl. Prodr. Fl. Hispan.: 139. 1893 syn. sec. Nieto Feliner (1987) ≡ *Armeria pseudarmeria* subsp. *longifolia* (Willk.) Malag. in Acta Phytotax. Barcinon. 1: 24. 1968 ["1969"] syn. sec. Malekmohammadi & al. (2024)

Armeria malinvaudii **H.J.Coste & Soulié in Bull. Soc. Bot. France 58: 362. 1911.** Sec. Domina (2011+)

≡ *Statice malinvaudii* (H.J.Coste & Soulié) P.Fourn., Quatre Fl. France: 723. 1937 syn. sec. Malekmohammadi & al. (2024)

Armeria maritima **(Mill.) Willd., Enum. Pl.: 333. 1809.** Sec. Bokhari & Edmondson (1982)

≡ *Statice maritima* Mill., Gard. Dict., ed. 8: Statice no. 3. 1768 syn. sec. Bokhari & Edmondson (1982) ≡ *Statice armeria* var. *maritima* (Mill.) Wahlenb., Fl. Suec. 1: 191. 1824 syn. sec. Gams (1927: synonym of Statice maritima Mill.)
= *Statice armeria* L., Sp. Pl.: 274. 1753 syn. sec. Bokhari & Edmondson (1982)
= *Statice capitata* Lam., Fl. Franç. 3: 63. 1779 syn. sec. Malekmohammadi & al. (2024)
= *Statice armeria* var. *pubescens* Sowerby ex Sm., Engl. Bot.: t. 226. 1795 syn. sec. Malekmohammadi & al. (2024)
= *Statice linearifolia* Laterr., Fl. Bordel., ed. 2: 189. 1821, nom. illeg. syn. sec. Boissier (1848)

= *Statice pulchella* Gray, Nat. Arr. Brit. Pl. 2: 296. 1821 syn. sec. Malekmohammadi & al. (2024)
= *Statice armeria* var. *arenaria* Dumort., Fl. Belg.: 27. 1827 syn. sec. Malekmohammadi & al. (2024)
= *Statice armeria* var. *caudiculata* Dumort., Fl. Belg.: 27. 1827 syn. sec. Malekmohammadi & al. (2024)
= *Statice armeria* var. *minor* Dumort., Fl. Belg.: 27. 1827 syn. sec. Malekmohammadi & al. (2024)
= *Statice armeria* var. *mixta* Dumort., Fl. Belg.: 27. 1827 syn. sec. Malekmohammadi & al. (2024)
= *Statice armeria* var. *hortensis* Gaudin, Fl. Helv. 2: 454. 1828 syn. sec. Malekmohammadi & al. (2024)
= *Armeria pubescens* B.Heyne ex Steud., Nomencl. Bot., ed. 2, 1: 133. 1840 syn. sec. Malekmohammadi & al. (2024)
= *Armeria campestris* var. *linkii* Wallr., Beitr. Bot. 2: 204. 1844 syn. sec. Malekmohammadi & al. (2024)
= *Armeria maritima* subsp. *anglica* Wallr., Beitr. Bot. 2: 186. 1844 syn. sec. Malekmohammadi & al. (2024)
= *Armeria maritima* subsp. *belgica* Wallr., Beitr. Bot. 2: 186. 1844 syn. sec. Malekmohammadi & al. (2024)
= *Armeria maritima* subsp. *gallica* Wallr., Beitr. Bot. 2: 186. 1844 syn. sec. Malekmohammadi & al. (2024)
= *Armeria maritima* subsp. *germanica* Wallr., Beitr. Bot. 2: 186. 1844 syn. sec. Malekmohammadi & al. (2024)
= *Armeria maritima* subsp. *itala* Wallr., Beitr. Bot. 2: 186. 1844 syn. sec. Malekmohammadi & al. (2024)
= *Armeria maritima* var. *hortensis* Wallr., Beitr. Bot. 2: 186. 1844 syn. sec. Malekmohammadi & al. (2024)
= *Armeria maritima* var. *sylvestris* Wallr., Beitr. Bot. 2: 186. 1844 syn. sec. Malekmohammadi & al. (2024)
= *Armeria intermedia* Link ex Boiss. in Candolle, Prodr. 12: 681. 1848 syn. sec. POWO (2017+: 9 May 2022)
= *Armeria pubigera* var. *scotica* Boiss. in Candolle, Prodr. 12: 678. 1848 syn. sec. POWO (2017+: 9 May 2022)
= *Armeria maritima* var. *linkii* Gren & Godr., Fl. France 2: 733. 1853 syn. sec. Daveau (1888)
= *Armeria vulgaris* f. *longiscapa* F.Petri, Gen. Armeniae: 40. 1863 syn. sec. Malekmohammadi & al. (2024)
= *Armeria vulgaris* subsp. *maritima* (Mill.) F.Petri, Gen. Armeniae: 41. 1863 syn. sec. Malekmohammadi & al. (2024)
= *Armeria vulgaris* var. *calaminaria* F.Petri, Gen. Armeniae: 40. 1863 syn. sec. Malekmohammadi & al. (2024)
= *Armeria vulgaris* var. *eifeliaca* F.Petri, Gen. Armeniae: 40. 1863 syn. sec. Malekmohammadi & al. (2024)
= *Armeria vulgaris* var. *longiinvolucrata* F.Petri, Gen. Armeniae: 41. 1863 syn. sec. Malekmohammadi & al. (2024)
= *Armeria vulgaris* var. *palatina* F.Petri, Gen. Armeriae: 39. 1863 syn. sec. Malekmohammadi & al. (2024)
= *Armeria vulgaris* var. *pubescens* (Sowerby) F.Petri, Gen. Armeniae: 40. 1863 syn. sec. Malekmohammadi & al. (2024)
= *Armeria vulgaris* var. *rugica* F.Petri, Gen. Armeniae: 40. 1863 syn. sec. Malekmohammadi & al. (2024)
= *Armeria maritima* var. *duriuscula* (Bab.) Bab., Man. Brit. Bot. ed. 6: 681. 1867 syn. sec. POWO (2017+)
= *Armeria maritima* var. *pubigera* (Boiss.) Bab., Man. Brit. Bot. ed. 6: 681. 1867 syn. sec. Malekmohammadi & al. (2024)
= *Armeria vulgaris* var. *planifolia* Syme, Engl. Bot. ed. 3B 8: 158. 1868 syn. sec. Malekmohammadi & al. (2024)
= *Armeria vulgaris* f. *purpurea* T.Marsson, Fl. Neu-Vorpommern: 379. 1869 syn. sec. POWO (2017+: 9 May 2022)
= *Armeria vulgaris* var. *glabra* T.Marsson, Fl. Neu-Vorpommern: 379. 1869 syn. sec. Malekmohammadi & al. (2024)
= *Armeria maritima* var. *planifolia* (Syme) Bab., Man. Brit. Bot. ed. 8: 295. 1881 syn. sec. Malekmohammadi & al. (2024)
= *Armeria planifolia* Nyman, Consp. Fl. Eur. 3: 616. 1881 syn. sec. POWO (2017+: 9 May 2022)
= *Armeria vulgaris* var. *maritima* (Mill.) T.Marsson in Meddel. Grønland 3: 683. 1891, nom. illeg. syn. sec. Malekmohammadi & al. (2024)
= *Armeria vulgaris* var. *maritima* Rosenv., Meddel. Gronland 3: 683. 1891 syn. sec. Malekmohammadi & al. (2024)
= *Armeria vulgaris* var. *alba* L.H.Bailey, Stand. Cycl. Hort. 1: 395. 1914 syn. sec. POWO (2017+: 9 May 2022) ≡ *Statice armeria* var. *alba* (L.H.Bailey) F.T.Hubb. in Rhodora 18(211): 156. 1916 syn. sec. Malekmohammadi & al. (2024)
= *Armeria vulgaris* var. *grandiflora* L.H.Bailey, Stand. Cycl. Hort. 1: 395. 1914 syn. sec. POWO (2017+: 9 May 2022) ≡ *Statice armeria* var. *grandiflora* (L.H.Bailey) F.T.Hubb. in Rhodora 18(211): 157. 1916 syn. sec. Malekmohammadi & al. (2024)
= *Armeria vulgaris* var. *rubra* hort. ex L.H.Bailey, Stand. Cycl. Hort. 1: 395. 1914 syn. sec. Malekmohammadi & al. (2024)
= *Armeria vulgaris* var. *splendens* L.H.Bailey, Stand. Cycl. Hort. 1: 395. 1914 syn. sec. POWO (2017+: 9 May 2022) ≡ *Statice armeria* var. *splendens* (L.H.Bailey) F.T.Hubb. in Rhodora 18(211): 157. 1916 syn. sec. Malekmohammadi & al. (2024)
= *Armeria elongata* var. *maritima* (Mill.) Skottsb. in Kongl. Svenska Vetensk. Acad. Handl. n.s., 56(5): 286. 1916 syn. sec. POWO (2017+: 9 May 2022)
= *Statice armeria* var. *purpurea* (L.H.Bailey) F.T.Hubb. in Rhodora 18(211): 157. 1916 syn. sec. Malekmohammadi & al. (2024)
= *Statice armeria* var. *rubra* (L.H.Bailey) F.T.Hubb. in Rhodora 18(211): 157. 1916 syn. sec. Berendsohn (2021)
= *Statice planifolia* (Syme) Druce in Rep. Bot. Soc. Exch. Club Brit. Isles 4(6): 203. 1917 syn. sec. Malekmohammadi & al. (2024)
= *Statice pubescens* (Sowerby) Druce, Rep. Bot. Soc. Exch. Club Brit. Isles 6(3): 504. 1922 syn. sec. Malekmohammadi & al. (2024)
= *Armeria vulgaris* f. *ambifaria* (Focke) W.F.Christ. in Bot. Arch. 31: 254. 1931 syn. sec. Malekmohammadi & al. (2024)
= *Armeria maritima* var. *alpinifolia* (Pau & Font Quer) G.H.M.Lawr. in Gentes Herbarum 4: 406. 1940 syn. sec. Malekmohammadi & al. (2024)
= *Armeria maritima* var. *ambifaria* (Focke) G.H.M.Lawr. in Gentes Herbarum 4: 405. 1940 syn. sec. Malekmohammadi & al. (2024)
= *Armeria maritima* var. *maria* G.H.M.Lawr. in Gentes Herbarum 4: 405. 1940 syn. sec. Malekmohammadi & al. (2024)
= *Armeria vulgaris* subsp. *intermedia* (T.Marsson) Nordh., Norsk Fl.: 498. 1940 syn. sec. Malekmohammadi & al. (2024: 7 May 2022)
= *Armeria maritima* subvar. *elegans* Bernis in Anales Inst. Bot. Cavanilles 14: 347. 1957 syn. sec. Malekmohammadi & al. (2024)
= *Armeria maritima* subsp. *elegans* (Bernis) Malag. in Acta Phytotax. Barcinon. 1: 23. 1968 ["1969"] syn. sec. Malekmohammadi & al. (2024)

≡ *Armeria maritima* var. *scotica* (Boiss.) P.D.Sell, Fl. Gr. Brit. Ireland 1: 687. 2018 syn. sec. Malekmohammadi & al. (2024: 9 May 2022)
= *Androsace macloviana* Cham. syn. sec. Malekmohammadi & al. (2024)
− *Taxanthema armeria* Neck., Elem. Bot. 1: 115. 1790, op. utique oppr. syn. sec. Malekmohammadi & al. (2024)
− *Armeria armeria* (L.) H.Karst., Deut. Fl. ed. 2, 2: 489. 1895, nom. inval. syn. sec. POWO (2017+)
− *Armeria maritima* subsp. *eifeliaca* (Petri) Lefebvre in New Phytol. 73(1): 210. 1974, nom. inval. syn. sec. Malekmohammadi & al. (2024)

***Armeria maritima* subsp. *azorica* Franco, Nova Fl. Portugal 2: 563. 1984.** Sec. Domina (2011+)

***Armeria maritima* subsp. *barcensis* (Simonk.) P.Silva in Bot. J. Linn. Soc. 64: 376. 1971.** Sec. Pignatti (1972)

≡ *Armeria barcensis* Simonk. in Magyar Bot. Lapok 6: 13. 1907 syn. sec. Domina (2011+) ≡ *Armeria maritima* var. *barcensis* (Simonk.) G.H.M.Lawr. in Gentes Herbarum 4: 407. 1940 syn. sec. Malekmohammadi & al. (2024: 4 May 2022) ≡ *Armeria alpina* subsp. *barcensis* (Simonk.) Jáv. syn. sec. Domina (2011+)

***Armeria maritima* subsp. *californica* (Boiss.) Porsild in Bull. Natl. Mus. Canada 135: 174. 1955.** Sec. Malekmohammadi & al. (2024)

≡ *Armeria andina* var. *californica* Boiss. in Candolle, Prodr. 12: 682. 1848 syn. sec. POWO (2017+: 9 May 2022) ≡ *Armeria arctica* var. *californica* (Boiss.) S.F.Blake in Rhodora 19: 18. 1917 syn. sec. Malekmohammadi & al. (2024) ≡ *Statice arctica* var. *californica* (Boiss.) S.F.Blake in Rhodora 19(217): 9. 1917 syn. sec. Malekmohammadi & al. (2024) ≡ *Armeria macloviana* subsp. *californica* (Boiss.) Iversen in Biol. Meddel. Kongel. Danske Vidensk. Selsk. 15(8): 18. 1940 syn. sec. POWO (2017+: 10 May 2022) ≡ *Armeria maritima* var. *californica* (Boiss.) G.H.M.Lawr. in Gentes Herbarum 4(11): 406. 1940 syn. sec. POWO (2017+) ≡ *Armeria arctica* subsp. *californica* (Boiss.) Abrams, Ill. Fl. Pacific States 3: 345. 1951 syn. sec. POWO (2017+: 9 May 2022) ≡ *Statice arctica* subsp. *californica* (Boiss.) A.E.Murray in Kalmia 12: 25. 1982 syn. sec. Malekmohammadi & al. (2024)
= *Armeria sanguinolenta* Wallr., Beitr. Bot. 2: 207. 1844 syn. sec. Malekmohammadi & al. (2024)

***Armeria maritima* subsp. *depilata* (Bernis) Malag. in Acta Phytotax. Barcinon. 1: 23. 1968 ["1969"].** Sec. Malagarriga (1968)

≡ *Armeria maritima* var. *depilata* Bernis in Anales Inst. Bot. Cavanilles 14: 351. 1957 syn. sec. Malagarriga (1968)

***Armeria maritima* subsp. *elongata* (Hoffm.) Bonnier, Fl. Ill. France 9: 54. 1927.** Sec. Pignatti (1972)

≡ *Armeria elongata* Hoffm., Deutschl. Fl. 1: 150. 1791 syn. sec. Malekmohammadi & al. (2024) ≡ *Statice elongata* Hoffm., Deutschl. Fl. 3: 150. 1800 syn. sec. Arrigoni (2015) ≡ *Armeria elongata* (Hoffm.) W.D.J.Koch, Deutschl. Fl. 2: 487. 1826 syn. sec. Arrigoni (2015: 27 April 2022) ≡ *Armeria vulgaris* var. *elongata* (Hoffm.) Mert. & W.D.J.Koch, Deutschl. Fl. ed. 3, 2: 487. 1826 syn. sec. Malekmohammadi & al. (2024) ≡ *Armeria vulgaris* subsp. *elongata* (Hoffm.) F.Petri, Gen. Armeniae: 39. 1863 syn. sec. Malekmohammadi & al. (2024) ≡ *Armeria vulgaris* var. *elongata* (Hoffm.) F.Petri, Gen. Armeniae: 39. 1863 syn. sec. Malekmohammadi & al. (2024) ≡ *Armeria vulgaris* var. *elongata* (Hoffm.) T.Marsson, Fl. Neu-Vorpommern: 378. 1869 syn. sec. Malekmohammadi & al. (2024) ≡ *Armeria maritima* f. *elongata* (Hoffm.) Blytt, Norges Fl. 2: 532. 1874 syn. sec. Malekmohammadi & al. (2024) ≡ *Statice armeria* subsp. *elongata* (Hoffm.) P.Fourn., Quatre Fl. France: 723. 1937 syn. sec. Malekmohammadi & al. (2024) ≡ *Statice vulgaris* var. *elongata* (Hoffm.) Samp., Fl. Port.: 440. 1947 syn. sec. Malekmohammadi & al. (2024: 26 April 2022) − *Statice maritima* var. *elongata* (Hoffm.) Samp., Herb. Port.: 100. 1913, nom. inval. syn. sec. Malekmohammadi & al. (2024: 26 April 2022)
= *Statice armeria* subsp. *major* Ehrh. in Hannover. Mag. 18: 220. 1780 syn. sec. Malekmohammadi & al. (2024)
= *Statice linearifolia* Loisel., Fl. Gall.: 180. 1806 syn. sec. Malekmohammadi & al. (2024)
= *Statice armeria* var. *tenuifolia* DC., Fl. Franç., ed. 3, 5: 379. 1815 syn. sec. Malekmohammadi & al. (2024)
= *Armeria elongata* W.D.J.Koch, Deutschl. Fl., ed. 3, 2: 487. 1826 syn. sec. Malekmohammadi & al. (2024)
= *Statice armeria* var. *elongata* Dumort., Fl. Belg.: 27. 1827 syn. sec. Malekmohammadi & al. (2024)
= *Armeria campestris* var. *hoffmannii* Wallr., Beitr. Bot. 2: 204. 1844 syn. sec. Malekmohammadi & al. (2024)
= *Armeria elongata* (Hoffm.) M.Loehr, Enum. Fl. Deutschland: 556. 1852, nom. illeg. syn. sec. Pignatti (1972)
= *Armeria vulgaris* var. *intermedia* T.Marsson, Fl. Neu-Vorpommern: 379. 1869 syn. sec. POWO (2017+: 9 May 2022) ≡ *Armeria intermedia* (T.Marsson) Szafer in Acta Soc. Bot. Poloniae 17: 9. 1946, nom. illeg. syn. sec. Malekmohammadi & al. (2024) ≡ *Armeria maritima* subsp. *intermedia* (T.Marsson) C.Lefèbvre ex Buttler, Ber. Bot. Arbeitsgem. Südwestdeutschl. 8: 33. 2017 syn. sec. POWO (2017+) ≡ *Armeria elongata* var. *intermedia* T.Marsson syn. sec. Malekmohammadi & al. (2024)
= *Armeria ambifaria* Focke in Abh. Naturwiss. Vereins Bremen 17: 445. 1903 syn. sec. POWO (2017+: 4 May 2022)
= *Armeria vulgaris* f. *persicina* W.F.Christ. in Bot. Arch. 31: 253. 1931 syn. sec. Malekmohammadi & al. (2024)
= *Armeria elongata* f. *rostellata* Szafer in Acta Soc. Bot. Poloniae 17: 13. 1946 syn. sec. Malekmohammadi & al. (2024)
= *Armeria baltica* Tzvelev, Konspekt Fl. Vost. Evr. 1: 335. 2012 syn. sec. POWO (2017+: 9 May 2022)

***Armeria maritima* subsp. *fontqueri*.** Sec. Domina (2011+)

***Armeria maritima* subsp. *interior* (Raup) A.E.Porsild in Bull. Natl. Mus. Canada 135: 172. 1955.** Sec. Malekmohammadi & al. (2024)

≡ *Statice interior* Raup in J. Arnold Arbor. 17(4): 289. 1936 syn. sec. Malekmohammadi & al. (2024) ≡ *Armeria maritima* var. *interior* (Raup) G.H.M.Lawr. in Gentes Herbarum 4(11): 405. 1940 syn. sec. Malekmohammadi & al. (2024) ≡ *Armeria maritima* subsp. *interior* (Raup) Lefebvre & Vekem. in Canad. J. Bot. 73: 1595. 1995 syn. sec. Malekmohammadi & al. (2024)

***Armeria maritima* subsp. *juniperifolia* (Bernis) Malag. in Acta Phytotax. Barcinon. 1: 23. 1968 ["1969"].** Sec. Malagarriga (1968)

≡ *Armeria maritima* subvar. *juniperifolia* Bernis in Anales Inst. Bot. Cavanilles 14: 352. 1957 syn. sec. Malagarriga (1968)

***Armeria maritima* (Mill.) Willd. subsp. *maritima*.** Sec. Pignatti (1972)

= *Statice vulgaris* var. *majuscula* (Samp.) Pires de Lima, Fl. Port.: 440. 1947 syn. sec. Malekmohammadi & al. (2024: 26 April 2022) ≡ *Armeria maritima* subsp. *majuscula* (Samp.) Malag. in Acta Phytotax. Barcinon. 1: 23. 1968 ["1969"] syn. sec. Malekmohammadi & al. (2024)
= *Statice armeria* subsp. *armeria* syn. sec. Domina (2011+)
− *Statice vulgaris* Hill, Brit. Herb.: 345. 1756, op. utique oppr. syn. sec. Govaerts, R. (ed.) (2023)
− *Statice maritima* var. *majuscula* Samp., Herb. Port.: 100. 1913, nom. inval. syn. sec. Malekmohammadi & al. (2024: 26 April 2022)
− *Armeria maritima* var. *scotica* (Boiss.) Bab., Man. Brit. Bot., ed. 6, 281. 1867 syn. sec. POWO (2017+: 9 May 2022) [is later isonym of *Armeria maritima* var. *scotica* (Boiss.) P.D.Sell]
= *Armeria vulgaris* Willd., Enum. Pl.: 333. 1809 syn. sec. Domina (2011+) ≡ *Armeria vulgaris* Willd. subsp. *vulgaris* syn. sec. Domina (2011+)
= *Statice pubescens* Sm. ex Schult. in Roemer & Schultes, Syst. Veg. ed. 15[bis] 6: 772. 1820 syn. sec. Govaerts, R. (ed.) (2023)
= *Statice vulgaris* Sweet, Hort. Brit.: 332. 1826 syn. sec. Domina (2011+)
= *Armeria pubescens* Link in Repert. Nat. Cur. Berol. 1: 180. 1829 syn. sec. Domina (2011+) ≡ *Armeria maritima* var. *pubescens* (Link) Bab., Man. Brit. Bot. ed. 6: 252. 1867 syn. sec. IPNI (2000+: 17 may 2024) ≡ *Armeria pubescens* Link subsp. *pubescens* syn. sec. Domina (2011+)
= *Armeria montana* G.Don ex Loudon, Hort. Brit.: 114. 1830 syn. sec. Malekmohammadi & al. (2024)
= *Armeria maritima* var. *pubescens* (Sowerby) Rchb., Fl. Germ. Excurs. 1: 190. 1831 syn. sec. POWO (2017+: 16 may 2024)
= *Armeria campestris* Wallr., Beitr. Bot. 2: 203. 1844 syn. sec. POWO (2017+: 9 May 2022)
= *Armeria duriuscula* Bab. in Ann. Mag. Nat. Hist. II, 3: 436. 1849 syn. sec. POWO (2017+: 9 May 2022)
= *Armeria laucheana* J.N.Haage & E.Schmidt, Nursery Cat. (Haage): 2. 1875 syn. sec. Govaerts, R. (ed.) (2023) ≡ *Armeria maritima* f. *laucheana* (J.N.Haage & E.Schmidt) Voss, Vilm. Blumengärtn., ed. 3, 1: 617. 1896 syn. sec. Govaerts, R. (ed.) (2023) ≡ *Armeria vulgaris* var. *laucheana* (J.N.Haage & E.Schmidt) L.H.Bailey, Stand. Cycl. Hort. 1: 395. 1914 syn. sec. Govaerts, R. (ed.) (2023) ≡ *Statice armeria* var. *laucheana* (J.N.Haage & E.Schmidt) F.T.Hubb. in Rhodora 18(211): 157. 1916 syn. sec. Malekmohammadi & al. (2024)
= *Statice armeria* subsp. *maritima* (Mill.) P.Fourn., Quatre Fl. France: 723. 1937 syn. sec. POWO (2017+: 9 May 2022)
= *Statice vulgaris* var. *majuscula* (Samp.) Pires de Lima, Fl. Port.: 440. 1947 syn. sec. Malekmohammadi & al. (2024: 26 April 2022) ≡ *Armeria maritima* subsp. *majuscula* (Samp.) Malag. in Acta Phytotax. Barcinon. 1: 23. 1968 ["1969"] syn. sec. Malekmohammadi & al. (2024)

***Armeria maritima* (Mill.) Willd. var. *maritima*.** Sec. Domina (2011+)

***Armeria maritima* var. *profilicaulis* Bernis.** Sec. Domina (2011+)

***Armeria maritima* subsp. *miscella* (Merino) Malag. in Acta Phytotax. Barcinon. 1: 23. 1968 ["1969"].** Sec. Pignatti (1972)

≡ *Armeria miscella* Merino, Fl. Galicia 3: 584. 1909 syn. sec. Malekmohammadi & al. (2024)

***Armeria maritima* subsp. *planifolia* (Syme) Á.Löve & D.Löve.** Sec. Domina (2011+)

***Armeria maritima* subsp. *purpurea* (W.D.J.Koch) Á.Löve & D.Löve in Bot. Not. 114: 54. 1961.** Sec. Pignatti (1972)

≡ *Armeria purpurea* W.D.J.Koch in Flora 6(2): 710. 1823 syn. sec. Domina (2011+) ≡ *Armeria vulgaris* var. *purpurea* (W.D.J.Koch) Mert. & W.D.J.Koch, Deutschl. Fl. ed. 3, 2: 488. 1826 syn. sec. Malekmohammadi & al. (2024) ≡ *Statice purpurea* (W.D.J.Koch) W.D.J.Koch, Syn. Fl. Germ. Helv. 1: 595. 1837 syn. sec. Domina (2011+) ≡ *Armeria elongata* var. *purpurea* (W.D.J.Koch) Boiss. in Candolle, Prodr. 12: 681. 1848 syn. sec. Malekmohammadi & al. (2024) ≡ *Armeria vulgaris* subsp. *purpurea* (W.D.J.Koch) Nyman, Consp. Fl. Eur. 3: 616. 1881 syn. sec. Domina (2011+) ≡ *Armeria alpina* var. *purpurea* (W.D.J.Koch) E.Baumann, Vegetationsbilder 9. 1911 syn. sec. Malekmohammadi & al. (2024) ≡ *Armeria maritima* var. *purpurea* (W.D.J.Koch) G.H.M.Lawr. in Gentes Herbarum 4(11): 405. 1940 syn. sec. Malekmohammadi & al. (2024)
= *Armeria rhenana* Gremli, Excursionsfl. Schweiz ed. 4: 350. 1881 syn. sec. POWO (2017+: 9 May 2022)
= *Armeria vulgaris* var. *purpurea* hort. ex L.H.Bailey, Stand. Cycl. Hort. 1: 395. 1914 syn. sec. Malekmohammadi & al. (2024)
= *Armeria vulgaris* subsp. *rhenana* (Gremli) Nyman syn. sec. Domina (2011+)
= *Armeria campestris* var. *kochii* Wallr., Beitr. Bot. 2: 204. 1844, nom. superfl. syn. sec. Govaerts, R. (ed.) (2023)

***Armeria maritima* subsp. *sibirica* (Turcz. ex Boiss.) Nyman, Consp. Fl. Eur. 3: 616. 1881.** Sec. Pignatti (1972)

≡ *Armeria sibirica* Turcz. ex Boiss. in Candolle, Prodr. 12: 678. 1848 syn. sec. Malekmohammadi & al. (2024) ≡ *Statice sibirica* (Turcz. ex Boiss.) Ledeb., Fl. Ross. 3: 357. 1849 syn. sec. Malekmohammadi & al. (2024) ≡ *Armeria maritima* var. *sibirica* (Turcz. ex Boiss.) A.Blytt, Norges Fl. 2: 532. 1874 syn. sec. Malekmohammadi & al. (2024) ≡ *Armeria elongata* var. *sibirica* (Turcz. ex Boiss.) Hartm., Handb. Skand. Fl. ed. 11: 135. 1879 syn. sec. Malekmohammadi & al. (2024) ≡ *Armeria vulgaris* var. *sibirica* (Turcz. ex Boiss.) Rosenv. in Meddel. Grønland 3: 683. 1891 syn. sec. POWO (2017+: 9 May 2022) ≡ *Armeria scabra* subsp. *sibirica* (Turcz. ex Boiss.) Hyl. in Uppsala Univ. Arsskr. 1945(7): 257. 1945 syn. sec. Domina (2011+) ≡ *Armeria labradorica* subsp. *sibirica* (Turcz. ex Boiss.) Kamelin in Novon 3(3): 261. 1993 syn. sec. Malekmohammadi & al. (2024)
= *Armeria scabra* Pall. ex Schult. in Roemer & Schultes, Syst. Veg. ed. 15[bis] 6: 776. 1820 syn. sec. Malekmohammadi & al. (2024)

= *Armeria vulgaris* f. *arctica* Cham. in Linnaea 6: 566. 1831 syn. sec. Malekmohammadi & al. (2024) ≡ *Armeria arctica* (Cham.) Wallr., Beitr. Bot. 2: 193. 1844 syn. sec. Malekmohammadi & al. (2024: 5 May 2022) ≡ *Armeria vulgaris* subsp. *arctica* (Cham.) Nyman, Consp. Fl. Eur. 3: 616. 1881 syn. sec. Malekmohammadi & al. (2024: 6 May 2022) ≡ *Statice arctica* (Cham.) S.F.Blake in Rhodora 19(217): 8. 1917 syn. sec. Malekmohammadi & al. (2024) ≡ *Armeria vulgaris* subsp. *arctica* (Cham.) Hultén, Fl. Aleut. Isl.: 275. 1937 syn. sec. Malekmohammadi & al. (2024) ≡ *Armeria scabra* subsp. *arctica* (Cham.) Iversen in Biol. Meddel. Kongel. Danske Vidensk. Selsk. 15(8): 18. 1940 syn. sec. Malekmohammadi & al. (2024) ≡ *Armeria maritima* subsp. *arctica* (Cham.) Hultén, Fl. Alaska Yukon 8: 1295. 1948 syn. sec. Malekmohammadi & al. (2024) ≡ *Armeria maritima* var. *arctica* (Cham.) Bernis in Anales Inst. Bot. Cavanilles 11(2): 259. 1954 syn. sec. Malekmohammadi & al. (2024)
= *Armeria alpina* Turcz. in Bull. Soc. Imp. Naturalistes Moscou 11: 99. 1838, nom. illeg. syn. sec. Malekmohammadi & al. (2024)
= *Armeria labradorica* Wallr., Beitr. Bot. 2: 185. 1844 syn. sec. Malekmohammadi & al. (2024) ≡ *Armeria vulgaris* var. *labradorica* (Wallr.) F.Petri, Gen. Armeniae: 41. 1863 syn. sec. POWO (2017+) ≡ *Statice labradorica* (Wallr.) F.T.Hubb. & S.F.Blake in Rhodora 19(217): 6. 1917 syn. sec. Malekmohammadi & al. (2024) ≡ *Armeria scabra* subsp. *labradorica* (Wallr.) Iversen in Biol. Meddel. Kongel. Danske Vidensk. Selsk. 15(8): 18. 1940 syn. sec. Domina (2011+) ≡ *Armeria maritima* var. *labradorica* (Wallr.) G.H.M.Lawr. in Gentes Herbarum 4(11): 405. 1940 syn. sec. Malekmohammadi & al. (2024) ≡ *Armeria maritima* subsp. *labradorica* (Wallr.) Hultén, Fl. Alaska Yukon 8: 1296. 1948 syn. sec. Domina (2011+) ≡ *Armeria scabra* var. *labradorica* (Wallr.) Tzvelev in Fl. Vostoch. Evropy 9: 163. 1996 syn. sec. Malekmohammadi & al. (2024) − *Armeria labradorica* var. *genuina* Malte in Rhodora 36: 185. 1934, nom. inval. syn. sec. Govaerts, R. (ed.) (2023)
= *Statice scabra* Pall. ex Boiss. in Candolle, Prodr. 12: 681. 1848 syn. sec. Boissier (1848)
= *Armeria elongata* var. *scabra* (Pall. ex Schult.) Regel, Index Seminum (LE) 1866: 30. 1868 syn. sec. Malekmohammadi & al. (2024)
= *Statice labradorica* f. *glabriscapa* S.F.Blake in Rhodora 19(217): 7, f. 1. 1917 syn. sec. Malekmohammadi & al. (2024)
= *Statice labradorica* f. *pubiscapa* S.F.Blake in Rhodora 19(217): 7. 1917 syn. sec. Malekmohammadi & al. (2024)
= *Statice labradorica* var. *submutica* S.F.Blake in Rhodora 19(217): 7. 1917 syn. sec. Malekmohammadi & al. (2024)
= *Armeria labradorica* f. *glabriscapa* H.F.Lewis, Canad. Field-Naturalist 46: 40. 1932 syn. sec. Malekmohammadi & al. (2024)
= *Armeria labradorica* var. *submutica* H.F.Lewis, Canad. Field-Naturalist 46(4): 92. 1932 syn. sec. Malekmohammadi & al. (2024)
= *Armeria labradorica* f. *glabriscapa* (S.F.Blake) Malte in Rhodora 36(425): 185. 1934 syn. sec. Malekmohammadi & al. (2024)
= *Armeria labradorica* f. *pubiscapa* Malte in Rhodora 36: 185. 1934 syn. sec. Malekmohammadi & al. (2024)
= *Armeria scabra* var. *glabricalyx* Tzvelev in Fl. Vostoch. Evropy 9: 163. 1996 syn. sec. Malekmohammadi & al. (2024)
– *Armeria arctica* sensu Sternberg, non (Cham.) Wallr., err. sec. Domina (2011+)

***Armeria masguindalii* (Pau) Nieto Fel. in Lagascalia 18: 236. 1996.** Sec. Domina (2011+)

≡ *Armeria plantaginea* var. *masguindalii* Pau, Iter Marocc. 1930: 510. 1932 syn. sec. Domina (2011+) ≡ *Statice plantaginea* var. *masguindalii* (Pau) Maire in Jahandiez & al., Cat. Pl. Maroc 3: 566. 1934 syn. sec. Malekmohammadi & al. (2024)
= *Armeria alliacea* var. *yebalica* Pau, Iter Marocc. 1928: 311. 1929 syn. sec. Malekmohammadi & al. (2024) ≡ *Statice allioides* var. *yebalica* (Pau) Maire in Bull. Soc. Hist. Nat. Afrique N. 22: 304. 1931 syn. sec. Malekmohammadi & al. (2024)

***Armeria mauritanica* Wallr., Beitr. Bot. 2: 217. 1844.** Sec. Bernis (1954)

= *Statice mauritanica* (Wallr.) F.T.Hubb. in Rhodora 18(211): 157. 1916 syn. sec. Malekmohammadi & al. (2024: 6 May 2022);
= *Statice lusitanica* Poir., Voy. Barbarie 2: 141. 1789 syn. sec. Bernis (1954)
= *Statice cephalotes* Poir., Encycl. 7: 394. 1806 syn. sec. Bernis (1954)
= *Armeria cephalotes* Hook. in Bot. Mag. 70: t. 4128. 1844, nom. illeg. syn. sec. Bailey (1916) [non *Armeria cephalotes* Schousb.]
= *Armeria formosa* Heynh. in Alph. Aufz. Gew.: 45. 1846 syn. sec. POWO (2017+: 6 May 2022)
= *Armeria baetica* var. *africana* Boiss. in Candolle, Prodr. 12: 676. 1848 syn. sec. Malekmohammadi & al. (2024)
= *Armeria boissieriana* Coss., Notes Pl. Crit.: 44. 1849 syn. sec. Bernis (1954) ≡ *Armeria gaditana* var. *boissieriana* (Coss.) G.H.M.Lawr. in Gentes Herbarum 4: 397. 1940 syn. sec. Malekmohammadi & al. (2024) ≡ *Armeria mauritanica* var. *boissieriana* (Coss.) Quézel & Santa syn. sec. Malekmohammadi & al. (2024: 6 May 2022)
= *Armeria alliacea* Webb ex Ball in J. Linn. Soc., Bot. 16: 560. 1878, nom. illeg. syn. sec. POWO (2017+: 6 May 2022)
– *Armeria baetica* sensu auct., non Boiss., err. sec. Domina (2011+)

***Armeria merinoi* (Bernis) Nieto Fel. & Silva Pando in Anales Jard. Bot. Madrid 44: 328. 1987.** Sec. Nieto Feliner (1987)

≡ *Armeria maritima* var. *merinoi* Bernis, Rev. Gen. Armeria Comp.: 12. 1951 syn. sec. Nieto Feliner (1987)

***Armeria montiberica* García Cardo, Fabado & Mateo in Flora Montiber. 86: 88. 2023.** Sec. Malekmohammadi & al. (2024)

***Armeria morisii* Boiss. in Candolle, Prodr. 12: 687. 1848.** Sec. Tiburtini & al. (2023)

= *Armeria latifolia* Moris, Stirp. Sard. Elench. 3: 10. 1829 syn. sec. Nyman (1881)
= *Statice cephalotes* Bertol., Fl. Ital. 3: 511. 1837 syn. sec. Boissier (1848)
= *Statice plantaginea* Guss., Fl. Sicul. Syn. 1: 366. 1843 syn. sec. Nyman (1881)

= *Armeria gussonei* Boiss. in Candolle, Prodr. 12: 687. 1848 syn. sec. Pignatti (1972) ≡ *Armeria morisii* var. *gussonei* (Boiss.) Parl., Fl. Ital. 8: 600. 1889 syn. sec. Malekmohammadi & al. (2024) ≡ *Armeria maritima* var. *morisii* (Boiss.) Bernis, Rev. Gen. Armeria Comp. 1951 syn. sec. Malekmohammadi & al. (2024)

***Armeria mulleri* A.Huet in Ann. Sci. Nat., Bot., sér. 3, 19: 255. 1853.** Sec. Nieto Feliner (1987)

≡ *Statice muelleri* (A.Huet) P.Fourn., Quatre Fl. France: 723. 1937 syn. sec. Malekmohammadi & al. (2024: 4 May 2022) ≡ *Armeria alpina* subsp. *muelleri* (A.Huet) Malag. in Acta Phytotax. Barcinon. 1: 22. 1968 ["1969"] syn. sec. Malekmohammadi & al. (2024: 7 May 2022) ≡ *Armeria maritima* subsp. *mulleri* (A.Huet) O.Bolòs & Vigo in Collect. Bot. (Barcelona) 11: 31. 1979 syn. sec. Malekmohammadi & al. (2024) − *Armeria muelleri* A.Huet in Ann. Sci. Nat., Bot., sér. 3, 19: 255. 1853, orth. var. syn. sec. Malekmohammadi & al. (2024);
= *Armeria maritima* subvar. *salvadorii* Bernis in Anales Inst. Bot. Cavanilles 14: 323. 1957 syn. sec. Nieto Feliner (1987)

***Armeria nuriensis* Sennen in Bull. Soc. Bot. France 73: 674. 1927.** Sec. Sennen (1927)

***Armeria orophila* Sennen in Bull. Soc. Bot. France 73: 674. 1927.** Sec. Sennen (1927)

***Armeria pauana* (Bernis) Nieto Fel. in Anales Jard. Bot. Madrid, ser. 2, 44: 333. 1987.** Sec. Nieto Feliner (1987)

≡ *Armeria maritima* var. *pauana* Bernis, Rev. Gen. Armeria Comp.: 8. 1951 syn. sec. Nieto Feliner (1987) ≡ *Armeria pseudarmeria* var. *pauana* Bernis in Anales Inst. Bot. Cavanilles 12(2): 133. 1955 syn. sec. Malekmohammadi & al. (2024) ≡ *Armeria pseudarmeria* subsp. *pauana* (Bernis) Malag. in Acta Phytotax. Barcinon. 1: 24. 1968 ["1969"] syn. sec. Malekmohammadi & al. (2024)

***Armeria* ×*pilariae* Sánchez Gullón, Muñoz Rodr. & Polo Ávila in Bouteloua 23: 171. 2016.** Sec. Malekmohammadi & al. (2024)

***Armeria pinifolia* (Brot.) Hoffmanns. & Link, Fl. Portug. 1: 437. 1817.** Sec. Pignatti (1972)

≡ *Statice pinifolia* Brot., Fl. Lusit. 1: 486. 1804 syn. sec. Daveau (1888)
= *Statice capillifolia* Poir., Encycl. 7: 397. 1806 syn. sec. Domina (2011+)
= *Armeria cephalotus* Boiss. in Candolle, Prodr. 12: 675. 1848 syn. sec. Nyman (1881)
= *Armeria lusitanica* Link ex Boiss. in Candolle, Prodr. 12: 675. 1848 syn. sec. Daveau (1888)
= *Statice pinifolia* var. *capillifolia* Samp., Fl. Port.: 439. 1947 syn. sec. Malekmohammadi & al. (2024: 26 April 2022)

***Armeria platyphylla* (Daveau) Franco, Nova Fl. Portugal 2: 563. 1984.** Sec. Domina (2011+)

≡ *Armeria welwitschii* var. *platyphylla* Daveau in Bol. Soc. Brot. 6: 167. 1888 syn. sec. Domina (2011+) ≡ *Statice welwitschii* var. *platyphylla* (Daveau) Samp., Fl. Port.: 439. 1947 syn. sec. Malekmohammadi & al. (2024: 26 April 2022)

***Armeria pocutica* Pawł., Fragm. Florist. Geobot. 8: 399. 1962.** Sec. Pignatti (1972)

***Armeria pseudarmeria* (Murray) Mansf. in Repert. Spec. Nov. Regni Veg. 47: 140. 1939.** Sec. Pignatti (1972)

≡ *Statice pseudarmeria* Murray, Syst. Veg., ed. 14: 300. 1784 syn. sec. Domina (2011+)
= *Armeria pseudarmeria* Murray, Syst. Veg., ed. 14: 300. 1784 syn. sec. Malekmohammadi & al. (2024)
= *Statice pseud-armeria* Murray, Syst. Veg., ed. 14: 300. 1784 syn. sec. Malekmohammadi & al. (2024)
= *Statice cephalotes* Aiton, Hort. Kew. 1: 383. 1789 syn. sec. Domina (2011+)
= *Statice variabilis* Salisb., Prodr. Stirp. Chap. Allerton: 176. 1796 syn. sec. Malekmohammadi & al. (2024)
= *Armeria pseudoarmeria* Brot., Fl. Lusit. 1: 488. 1804 syn. sec. Malekmohammadi & al. (2024) ≡ *Statice pseudoarmeria* Brot., Fl. Lusit. 1: 488. 1804 syn. sec. Malekmohammadi & al. (2024)
= *Armeria latifolia* Willd., Enum. Pl.: 334. 1809 syn. sec. Sampaio (1913)
= *Armeria cephalotes* Hoffmanns. & Link, Fl. Portug. 1: 440. 1817, nom. illeg. syn. sec. Domina (2011+)
= *Armeria longifolia* Desf., Tabl. École Bot., ed. 3 (Cat. Pl. Horti Paris.): 79. 1829 syn. sec. Malekmohammadi & al. (2024: synonym of Armeria cephalotes Hoffmanns. & Link)
= *Armeria plantaginea* Webb, Iter Hispan.: 18. 1838 syn. sec. POWO (2017+: 9 may 2022)
= *Armeria globosa* Link ex Boiss. in Candolle, Prodr. 12: 684. 1848 syn. sec. Malekmohammadi & al. (2024)
= *Armeria grandiflora* Boiss. in Candolle, Prodr. 12: 684. 1848 syn. sec. Malekmohammadi & al. (2024)
= *Armeria formosa* hort. ex H.Vilm., Fl. Pleine Terre: 848. 1863 syn. sec. Malekmohammadi & al. (2024)
= *Statice formosa* hort. ex E.Vilm., Fl. Pleine Terre: 1103. 1870 syn. sec. Malekmohammadi & al. (2024)
= *Armeria japonica* Rippa in Boll. Soc. Naturalisti Napoli 18: 173. 1905 syn. sec. Malekmohammadi & al. (2024)
= *Statice major* Samp., Herb. Port.: 101. 1913 syn. sec. Malekmohammadi & al. (2024)
= *Armeria arborea* L.H.Bailey, Stand. Cycl. Hort. 6: 3229. 1917 syn. sec. Malekmohammadi & al. (2024)
− *Armeria pseud-armeria* Murray, Syst. Veg., ed. 14: 300. 1784 syn. sec. Malekmohammadi & al. (2024) [is misspelling for *Armeria pseudarmeria* Murray]

***Armeria pseudarmeria* subsp. *provillosa* (Bernis) Malag. in Acta Phytotax. Barcinon. 1: 23. 1968 ["1969"].** Sec. Malagarriga (1968)

≡ *Armeria pseudarmeria* var. *provillosa* Bernis in Anales Inst. Bot. Cavanilles 12(2): 126. 1955 syn. sec. Malagarriga (1968)

***Armeria pseudarmeria* (Murray) Mansf. subsp. *pseudarmeria*.** Sec. Malekmohammadi & al. (2024)

***Armeria pseudarmeria* subsp. *sobrinhoi* (Bernis) Malag. in Acta Phytotax. Barcinon. 1: 24. 1968 ["1969"].** Sec. Malagarriga (1968)

≡ *Armeria pseudarmeria* var. *sobrinhoi* Bernis syn. sec. Malagarriga (1968)
= *Armeria sobrinhoi* subvar. *monchiquensis* Bernis in Bol. Soc. Brot., sér. 2, 23: 23. 1950 syn. sec. Malagarriga (1968) ≡ *Armeria pseudarmeria* subsp. *monchiquensis* (Bernis) Malag. in Acta Phytotax. Barcinon. 1: 24. 1968 ["1969"] syn. sec. Malekmohammadi & al. (2024)

***Armeria pseudarmeria* subsp. *tejedensis* (Bernis) Malag. in Acta Phytotax. Barcinon. 1: 24. 1968 ["1969"].** Sec. Malagarriga (1968)

≡ *Armeria pseudarmeria* subvar. *tejedensis* Bernis in Anales Inst. Bot. Cavanilles 12(2): 123. 1955 syn. sec. Malagarriga (1968)

***Armeria pubigera* (Desf.) Boiss. in Candolle, Prodr. 12: 678. 1848.** Sec. Pignatti (1972)

≡ *Statice pubigera* Desf., Tabl. École Bot., ed. 3: 391. 1829 syn. sec. Daveau (1888) ≡ *Statice vulgaris* var. *pubigera* (Desf.) Pires de Lima in Sampaio, Fl. Port.: 440. 1947 syn. sec. Malekmohammadi & al. (2024: 26 April 2022) ≡ *Armeria maritima* subsp. *pubigera* (Desf.) Malag. in Acta Phytotax. Barcinon. 1: 23. 1968 ["1969"] syn. sec. Malekmohammadi & al. (2024) − *Statice maritima* var. *pubigera* (Desf.) Samp., Herb. Port.: 100. 1913, nom. inval. syn. sec. Malekmohammadi & al. (2024: 26 April 2022)
= *Armeria expansa* Wallr., Beitr. Bot. 2: 197. 1844 syn. sec. POWO (2017+) ≡ *Armeria pubescens* subsp. *expansa* (Wallr.) Nyman, Consp. Fl. Eur. 3: 616. 1881 syn. sec. Domina (2011+)
= *Armeria hirta* Pourr. ex Willk. & Lange, Prodr. Fl. Hispan. 2(2): 369. 1868 syn. sec. Malekmohammadi & al. (2024) ≡ *Armeria pubigera* var. *hirta* Lange ex Daveau in Bol. Soc. Brot. 6: 170. 1888 syn. sec. Malekmohammadi & al. (2024: 25 April 2022)
= *Armeria langeana* Henriq. in Bol. Soc. Brot. 5: 7. 1887 syn. sec. Domina (2011+) ≡ *Statice langeana* (Henriq.) Rothm., Index Seminum (Coll. Agron. Nat. Lusit.) 1939: 6. 1940 syn. sec. Malekmohammadi & al. (2024) ≡ *Statice vulgaris* var. *langeana* (Henriq.) Samp., Fl. Port.: 440. 1947 syn. sec. Malekmohammadi & al. (2024: 26 April 2022) − *Statice maritima* var. *longeana* (Henriq.) Samp., Herb. Port.: 100. 1913, nom. inval. syn. sec. Malekmohammadi & al. (2024: 26 April 2022)
= *Armeria henriquesii* Daveau in Bol. Soc. Brot. 6: 170. 1888 syn. sec. Malekmohammadi & al. (2024)
= *Armeria langeana* var. *genuina* Daveau in Bol. Soc. Brot. 6: 170. 1888 syn. sec. Malekmohammadi & al. (2024: 25 April 2022)
= *Armeria langeana* var. *glabra* Daveau in Bol. Soc. Brot. 6: 170. 1888 syn. sec. Malekmohammadi & al. (2024: 25 April 2022)
= *Armeria pubigera* var. *glabrescens* Lange ex Daveau in Bol. Soc. Brot. 6: 170. 1888 syn. sec. Malekmohammadi & al. (2024: 25 April 2022) ≡ *Armeria maritima* subsp. *glabrescens* (Lange ex Daveau) Malag. in Acta Phytotax. Barcinon. 1: 23. 1968 ["1969"] syn. sec. Malekmohammadi & al. (2024)
= *Armeria portensis* G.H.M.Lawr. in Gentes Herbarum 4: 414. 1940 syn. sec. Domina (2011+)
= *Armeria parvula* Franco, Nova Fl. Portugal 2: 562. 1984 syn. sec. Domina (2011+)
= *Armeria cantabrica* Rouy syn. sec. Malekmohammadi & al. (2024)

***Armeria pubinervis* Boiss. in Candolle, Prodr. 12: 688. 1848.** Sec. Pignatti (1972)

≡ *Statice pubinervis* (Boiss.) Vines & Druce, Morison. Herb.: 195. 1914 syn. sec. Malekmohammadi & al. (2024: 4 May 2022) ≡ *Statice cantabrica* subsp. *pubinervis* (Boiss.) P.Fourn., Quatre Fl. France: 724. 1937 syn. sec. Malekmohammadi & al. (2024: 4 May 2022) ≡ *Armeria alpina* subsp. *pubinervis* (Boiss.) Malag. in Acta Phytotax. Barcinon. 1: 22. 1968 ["1969"] syn. sec. Malekmohammadi & al. (2024)
= *Armeria pubinervis* subsp. *orissonensis* Donad. in Anales Jard. Bot. Madrid 41: 2980. 1985 syn. sec. Malekmohammadi & al. (2024: synonym of Armeria pubinervis Boiss.)

***Armeria pungens* (Brot.) Hoffmanns. & Link, Fl. Portug. 1: 439. 1817.** Sec. Nieto Feliner (1987)

≡ *Statice pungens* Brot., Fl. Lusit. 1: 487. 1804 syn. sec. Daveau (1888)
= *Statice fasciculata* Vent., Descr. Pl. Nouv.: t. 38. 1801 syn. sec. Domina (2011+) ≡ *Armeria fasciculata* (Vent.) Willd., Enum. Pl.: 334. 1809 syn. sec. Pignatti (1972)
= *Armeria fruticosa* G.Lodd., Bot. Cab. 16(9): t. 1587. 1830 syn. sec. POWO (2017+)
= *Armeria berlengensis* var. *villosa* Daveau in Bol. Soc. Brot. 2: 25. 1884 syn. sec. Malekmohammadi & al. (2024)
= *Armeria fasciculata* var. *intermedia* Daveau in Bol. Soc. Brot. 6: 163. 1888 syn. sec. Malekmohammadi & al. (2024)
= *Armeria pungens* var. *major* Daveau in Bol. Soc. Brot. 6: 163. 1888 syn. sec. Malekmohammadi & al. (2024) ≡ *Armeria pungens* subsp. *major* (Daveau) Franco, Nova Fl. Portugal 2: 564. 1984 syn. sec. Malekmohammadi & al. (2024)
= *Statice fasciculata* var. *pungens* Samp., Fl. Port.: 439. 1947 syn. sec. Malekmohammadi & al. (2024: 25 April 2022)
= *Armeria pungens* var. *aciculifolia* Franco, Nova Fl. Portugal 2: 564. 1984 syn. sec. Malekmohammadi & al. (2024)

***Armeria pungens* subsp. *bernisiana* Malag. in Acta Phytotax. Barcinon. 1: 26. 1968 ["1969"].** Sec. Malagarriga (1968)

***Armeria pungens* (Brot.) Hoffmanns. & Link var. *pungens*.** Sec. Nieto Feliner (1987)

***Armeria pungens* var. *velutina* Cout., Fl. Portugal: 470. 1913.** Sec. Malekmohammadi & al. (2024)

***Armeria quichiotis* (Gonz.Albo) A.W.Hill, Index Kew. Suppl. 9: 25. 1938.** Sec. Domina (2011+)

≡ *Statice quichiotis* Gonz.Albo in Bol. Real Soc. Esp. Hist. Nat. 34: 163. 1934 syn. sec. Domina (2011+)

***Armeria rivasmartinezii* Sard.Rosc. & Nieto Fel. in Collect. Bot. (Barcelona) 23: 98. 1998.** Sec. Domina (2011+)

***Armeria rothmaleri* Nieto Fel. in Anales Jard. Bot. Madrid, ser. 2, 44: 339. 1987.** Sec. Nieto Feliner (1987)

***Armeria rouyana* Daveau in Bol. Soc. Brot. 6: 166. 1888.** Sec. Nieto Feliner (1987)

≡ *Statice rouyana* (Daveau) Samp., Herb. Port.: 100. 1913 syn. sec. Sampaio (1913: 27 April 2022)

***Armeria rouyana* subsp. *littorea* (Bernis) Malag. in Acta Phytotax. Barcinon. 1: 23. 1968 ["1969"].** Sec. Malagarriga (1968)

≡ *Armeria maritima* f. *littorea* Bernis in Bol. Soc. Brot., sér. 2, 23: 254. 1950 syn. sec. Malekmohammadi & al. (2024) ≡ *Armeria rouyana* f. *littorea* (Bernis) Bernis in Anales Inst. Bot. Cavanilles 12(2): 82. 1955 syn. sec. Malagarriga (1968)
= *Armeria ×nieto-felineri* Rivas Mart. & al., Itinera Geobot. 3: 137. 1990 syn. sec. POWO (2017+: 9 may 2022)

***Armeria rouyana* Daveau subsp. *rouyana*.** Sec. Nieto Feliner (1987)

***Armeria ruscinonensis* Girard in Ann. Sci. Nat., Bot., ser. 3, 2: 323. 1844.** Sec. Domina (2011+)

≡ *Statice ruscinonensis* (Girard) P.Fourn., Quatre Fl. France: 723. 1937 syn. sec. Domina (2011+) ≡ *Armeria maritima* var. *ruscinonensis* (Girard) G.H.M.Lawr. in Gentes Herbarum 4: 407. 1940 syn. sec. Malekmohammadi & al. (2024) ≡ *Armeria canescens* subsp. *ruscinonensis* (Girard) Malag. in Acta Phytotax. Barcinon. 1: 23. 1968 ["1969"] syn. sec. Malekmohammadi & al. (2024) ≡ *Armeria alliacea* subsp. *ruscinonensis* (Girard) O.Bolòs & Vigo in Collect. Bot. (Barcelona) 11: 31. 1979 syn. sec. Malekmohammadi & al. (2024)
= *Armeria maritima* Girard ex Boiss. in Candolle, Prodr. 12: 680. 1848 syn. sec. Malekmohammadi & al. (2024)
= *Statice majellensis* subsp. *malinvaudii* (H. J. Coste & Soulié) P.Fourn., Quatre Fl. France: 723. 1937 syn. sec. Domina (2011+)
− *Statice majellensis* subsp. *eumajellensis* P.Fourn., Quatre Fl. France: 723. 1937, nom. inval. syn. sec. Domina (2011+)

***Armeria ruscinonensis* subsp. *littorifuga* (Bernis) Malag., Sin. Fl. Iber. 61: 97. 1976.** Sec. Nieto Feliner (1987)

≡ *Armeria maritima* subvar. *littorifuga* Bernis in Anales Inst. Bot. Cavanilles 12(2): 219. 1955 syn. sec. Malekmohammadi & al. (2024) ≡ *Armeria ruscinonensis* subvar. *littorifuga* Bernis in Anales Inst. Bot. Cavanilles 12(2): 219. 1955 syn. sec. Malekmohammadi & al. (2024) ≡ *Armeria canescens* subsp. *littorifuga* (Bernis) Malag. in Acta Phytotax. Barcinon. 1: 23. 1968 ["1969"] syn. sec. Malekmohammadi & al. (2024) ≡ *Armeria alliacea* var. *littorifuga* (Bernis) O.Bolòs & Vigo in Collect. Bot. (Barcelona) 11: 31. 1979 syn. sec. Malekmohammadi & al. (2024: 6 May 2022)
= *Armeria foucaudii* Beck in Ann. K. K. Naturhist. Hofmus. 13(1): 16. 1898 syn. sec. Nieto Feliner (1987)

***Armeria ruscinonensis* Girard subsp. *ruscinonensis*.** Sec. Domina (2011+)

***Armeria ×salmantica* (Bernis) Nieto Fel. in Anales Jard. Bot. Madrid, ser. 2, 44: 329. 1987.** Sec. Nieto Feliner (1987)

≡ *Armeria maritima* var. *salmantica* Bernis, Rev. Gen. Armeria Comp.: 9. 1951 syn. sec. Nieto Feliner (1987)

***Armeria sampaioi* (Bernis) Nieto Fel. in Anales Jard. Bot. Madrid, ser. 2, 44: 328. 1987.** Sec. Nieto Feliner (1987)

≡ *Armeria maritima* var. *sampaioi* Bernis, App. Armeria Port. 1950 syn. sec. Nieto Feliner (1987) ≡ *Armeria pseudarmeria* subsp. *sampaioi* (Bernis) Malag. in Acta Phytotax. Barcinon. 1: 24. 1968 ["1969"] syn. sec. Malekmohammadi & al. (2024) ≡ *Armeria pseudarmeria* var. *sampaioi* Bernis syn. sec. Malagarriga (1968)
= *Armeria maritima* subvar. *protypica* Bernis in Bol. Soc. Brot., sér. 2, 23: 251. 1950 syn. sec. Nieto Feliner (1987)

***Armeria sancta* Janka in Természetrajzi Füz. 6: 165. 1882.** Sec. Pignatti (1972)

***Armeria sardoa* Spreng., Syst. Veg., ed. 16, 4(2): 127. 1827.** Sec. Tiburtini & al. (2023)

≡ *Armeria vulgaris* var. *sardoa* (Spreng.) Fiori, Fl. Italia 2: 335. 1902 syn. sec. Arrigoni (2015) ≡ *Armeria maritima* var. *sardoa* (Spreng.) Bernis, Rev. Gen. Armeria Comp. 1951 syn. sec. Malekmohammadi & al. (2024: 6 May 2022)
= *Armeria tenuis* Balb. ex Boiss. in Candolle, Prodr. 12: 685. 1848 syn. sec. Malekmohammadi & al. (2024: 6 May 2022)
= *Armeria sardoa* subsp. *genargentea* Arrigoni in Webbia 25: 166. 1970 syn. sec. Tiburtini & al. (2023)
= *Armeria sardoa* Spreng. subsp. *sardoa* syn. sec. Tiburtini & al. (2023)
= *Armeria scabra* Willd. syn. sec. Domina (2011+)

***Armeria saviana* Selvi in Nordic J. Bot. 27: 126. 2009.** Sec. Arrigoni (2015)

***Armeria seticeps* Rchb., Icon. Fl. Germ. Helv. 5: 54. 1827.** Sec. Malekmohammadi & al. (2024)

≡ *Armeria alpina* var. *seticeps* (Rchb.) Ebel, Armeriae: 27. 1840 syn. sec. POWO (2017+: 8 May 2022)

***Armeria simplex* Pomel, Nouv. Mat. Fl. Atl. 1: 132. 1874.** Sec. Domina (2011+)

≡ *Armeria gaditana* var. *simplex* (Pomel) G.H.M.Lawr. in Gentes Herbarum 4: 397. 1940 syn. sec. Malekmohammadi & al. (2024: 6 May 2022) ≡ *Statice simplex* (Pomel) Santa, Monde Pl. 46: 28. 1951 syn. sec. Malekmohammadi & al. (2024: 6 May 2022) ≡ *Armeria mauritanica* var. *simplex* (Pomel) Faure, Exsicc Pl. Alger.: 1930 syn. sec. Malekmohammadi & al. (2024)
= *Armeria mauritanica* Boiss. in Candolle, Prodr. 12: 674. 1848 syn. sec. Malekmohammadi & al. (2024: 6 May 2022)
= *Armeria mauritanica* var. *calva* Boiss. in Candolle, Prodr. 12: 677. 1848 syn. sec. Malekmohammadi & al. (2024: 6 May 2022)
= *Armeria mauritanica* var. *ciliolata* Boiss. in Candolle, Prodr. 12: 677. 1848 syn. sec. Malekmohammadi & al. (2024)
= *Armeria mauritanica* var. *minor* Batt., Fl. Algérie [1](4): 736. 1890 syn. sec. Malekmohammadi & al. (2024)
= *Armeria amplifoliata* Pau, Iter Marocc. 1930: 513. 1932 syn. sec. Domina (2011+) ≡ *Statice amplifoliata* (Pau) Maire in Jahandiez & al., Cat. Pl. Maroc 3: 565. 1934 syn. sec. Domina (2011+) ≡ *Armeria mauritanica* var. *amplifoliata* (Pau) G.H.M.Lawr. in Gentes Herbarum 4: 399. 1940 syn. sec. Malekmohammadi & al. (2024)
= *Armeria gaditiana* var. *chamaeropicola* Pau, Iter Marocc. 1930: 511. 1932 syn. sec. Malekmohammadi & al. (2024: 6 May 2022) ≡ *Statice gaditana* var. *chamaeropicola* (Pau) Maire in Jahandiez & al., Cat. Pl. Maroc 3: 565. 1934 syn. sec. Malekmohammadi & al. (2024: 6 May 2022) ≡ *Armeria mauritanica* var. *chamaeropicola* (Pau) Bernis in Anales Inst. Bot. Cavanilles 11(2): 237. 1954 syn. sec. Malekmohammadi & al. (2024: 6 May 2022) ≡ *Armeria tingitana* var. *chamaeropicola* (Pau) Donad. in Bull. Soc. Bot. France 120: 149. 1973 syn. sec. Malekmohammadi & al. (2024: 6 May 2022)
= *Statice mauritanica* var. *safiensis* Maire in Jahandiez & al., Cat. Pl. Maroc 3: 565. 1934 syn. sec. Malekmohammadi & al. (2024: 6 May 2022)
= *Statice mauritanica* var. *soloitana* Maire in Bull. Soc. Hist. Nat. Afrique N. 29: 435. 1938 syn. sec. Malekmohammadi & al. (2024: 6 May 2022)
= *Armeria maghrebensis* Donad. in Bull. Soc. Bot. France 119: 491. 1973 syn. sec. Domina (2011+)
= *Armeria maghrebensis* var. *ebracteolata* Donad. in Bull. Soc. Bot. France 119: 492. 1973 syn. sec. Malekmohammadi & al. (2024: 6 May 2022)

= *Armeria maghrebensis* var. *mamorensis* Donad. in Bull. Soc. Bot. France 119: 493. 1973 syn. sec. Malekmohammadi & al. (2024: 6 May 2022)
= *Armeria mauritanica* var. *safiensis* Maire syn. sec. Malekmohammadi & al. (2024: 6 May 2022)
= *Armeria mauritanica* var. *soloitana* Maire syn. sec. Malekmohammadi & al. (2024: 6 May 2022)
– *Armeria boissieriana* sensu auct., non Coss., err. sec. Domina (2011+)

***Armeria soleirolii* (Duby) Godr., Fl. France 2: 737. 1853.** Sec. Tiburtini & al. (2023)

≡ *Statice soleirolii* Duby, Bot. Gall.: 1032. 1830 syn. sec. Tiburtini & al. (2023) ≡ *Armeria leucocephala* var. *soleirolii* (Duby) Boiss. in Candolle, Prodr. 12: 687. 1848 syn. sec. Malekmohammadi & al. (2024: 6 May 2022) ≡ *Armeria leucocephala* subsp. *soleirolii* (Duby) Arcang., Comp. Fl. Ital.: 578. 1882 syn. sec. Malekmohammadi & al. (2024: 6 May 2022) ≡ *Armeria maritima* var. *soleirolii* (Duby) Bernis, Rev. Gen. Armeria Comp. 1951 syn. sec. Malekmohammadi & al. (2024: 6 May 2022)
= *Armeria glaucescens* Desf., Tabl. École Bot., ed. 3: 79. 1829 syn. sec. Malekmohammadi & al. (2024)
= *Armeria fasciculata* var. *aristata* Mutel, A., Fl. Franç. 3: 86. 1836 syn. sec. Malekmohammadi & al. (2024: 6 May 2022)

***Armeria spinulosa* Boiss. in Candolle, Prodr. 12: 675. 1848.** Sec. Bernis (1954)

≡ *Statice spinulosa* (Boiss.) Maire in Bull. Soc. Hist. Nat. Afrique N. 29: 435. 1938 syn. sec. Malekmohammadi & al. (2024) ≡ *Armeria gaditana* var. *spinulosa* (Boiss.) G.H.M.Lawr. in Gentes Herbarum 4: 397. 1940 syn. sec. Bernis (1954) ≡ *Armeria hirta* subsp. *spinulosa* (Boiss.) Bernis in Anales Inst. Bot. Cavanilles 11(2): 198. 1954 syn. sec. Bernis (1954)

***Armeria splendens* (Lag. & Rodr.) Webb, Iter Hispan.: 18. 1838.** Sec. Pignatti (1972)

≡ *Statice splendens* Lag. & Rodr. in Anales Ci. Nat. 5(15): 274. 1802 syn. sec. Malagarriga (1968) ≡ *Armeria caespitosa* var. *splendens* (Lag. & Rodr.) Vicioso & Beltrán in Bol. Real Soc. Esp. Hist. Nat. 13: 311. 1913 syn. sec. Malekmohammadi & al. (2024: 6 May 2022) ≡ *Armeria juniperifolia* var. *splendens* (Lag. & Rodr.) G.H.M.Lawr. in Gentes Herbarum 4: 408. 1940 syn. sec. Malekmohammadi & al. (2024: 6 May 2022) ≡ *Armeria australis* var. *splendens* (Lag. & Rodr.) Boiss., Voy. Bot. Espagne 2: 527. 1845 syn. sec. Malekmohammadi & al. (2024: 6 May 2022) ≡ *Armeria splendens* subsp. *splendens* syn. sec. Pignatti (1972)
= *Armeria splendens* Boiss., Voy. Bot. Espagne 2: 749. 1845, nom. illeg. syn. sec. Malagarriga (1968)
= *Statice juniperifolia* Boiss. in Candolle, Prodr. 12: 680. 1848 syn. sec. Malekmohammadi & al. (2024: Statice juniperifolia)

***Armeria sulcitana* Arrigoni in Webbia 25: 169. 1970.** Sec. Tiburtini & al. (2023)

***Armeria tingitana* Boiss. & Reut., Pugill. Pl. Afr. Bor. Hispan.: 102. 1852.** Sec. Domina (2011+)

≡ *Armeria gaditiana* var. *tingitana* (Boiss. & Reut.) Ball in J. Linn. Soc., Bot. 16: 560. 1878 syn. sec. Bernis (1954) ≡ *Statice gaditana* var. *tingitana* (Boiss. & Reut.) Ball in J. Linn. Soc., Bot. 16: 560. 1878 syn. sec. Malekmohammadi & al. (2024: 6 May 2022) ≡ *Armeria hirta* var. *tingitana* (Boiss. & Reut.) Bernis in Anales Inst. Bot. Cavanilles 11(2): 237. 1954 syn. sec. Malekmohammadi & al. (2024: 6 May 2022)

***Armeria trachyphylla* Lange, Overs. Kongel. Danske Vidensk. Selsk. Forh. Medlemmers Arbeider 1893: 199. 1893.** Sec. Domina (2011+)

≡ *Armeria alpina* subsp. *trachyphylla* (Lange) Malag. in Acta Phytotax. Barcinon. 1: 22. 1968 ["1969"] syn. sec. Malekmohammadi & al. (2024)

***Armeria transmontana* (Samp.) G.H.M.Lawr. in Agron. Lusit. 12: 303. 1950.** Sec. Nieto Feliner (1987)

≡ *Statice transmontana* Samp., Fl. Port.: 442. 1947 syn. sec. Domina (2011+) ≡ *Armeria pseudarmeria* subsp. *transmontana* (Samp.) Malag. in Acta Phytotax. Barcinon. 1: 25. 1968 ["1969"] syn. sec. Malekmohammadi & al. (2024)
= *Armeria carpetana* Villar in Bol. Real Soc. Esp. Hist. Nat. 16: 403. 1916 syn. sec. POWO (2017+: 6 May 2022) ≡ *Statice carpetana* (Villar) Villar in Cavanillesia 2: 79. 1929 syn. sec. Malekmohammadi & al. (2024: 6 May 2022)
= *Armeria duriensis* Franco, Nova Fl. Portugal 2: 559. 1984 syn. sec. POWO (2017+: 6 May 2022)
= *Armeria duriensis* subsp. *monticola* Franco, Nova Fl. Portugal 2: 560. 1984 syn. sec. POWO (2017+)
= *Armeria carpetana* subsp. *carpetana* syn. sec. Malekmohammadi & al. (2024)

***Armeria transmontana* subsp. *aristulata* (Bernis) Bernis, Nova Fl. Portugal 2: 561. 1984.** Sec. Malekmohammadi & al. (2024)

≡ *Armeria maritima* f. *aristulata* Bernis in Bol. Soc. Brot., ser. 2, 23: 248. 1949 syn. sec. Malekmohammadi & al. (2024: 8 May 2022)
= *Armeria beirana* subsp. *altimontana* Franco, Nova Fl. Portugal 2: 562. 1984 syn. sec. Malekmohammadi & al. (2024: 8 May 2022)
= *Armeria transmontana* subsp. *pseudotransmontana* Franco, Nova Fl. Portugal 2: 561. 1984 syn. sec. Malekmohammadi & al. (2024: 8 May 2022)

***Armeria transmontana* (Samp.) G.H.M.Lawr. subsp. *transmontana*.** Sec. Nieto Feliner (1987)

***Armeria trianoi* Nieto Fel. in Bot. J. Linn. Soc. 135: 75. 2001.** Sec. Domina (2011+)

***Armeria trojana* Bokhari & Quézel in Candollea 25: 376. 1970.** Sec. Bokhari & Edmondson (1982)

***Armeria undulata* (Bory & Chaub.) Boiss. in Candolle, Prodr. 12: 685. 1848.** Sec. Pignatti (1972)

≡ *Statice undulata* Bory & Chaub., Nouv. Fl. Pélop.: 20. t. 11. 1838 syn. sec. Boissier (1848)
= *Armeria argyrocephala* Wallr., Beitr. Bot. 2: 206. 1844 syn. sec. Domina (2011+)
= *Armeria undulata* f. *brevifolia* Novák in Comm. Reg. Soc. Sci. Bohem. Mat. Nat. 28: 8. 1939 syn. sec. POWO (2017+: 6 May 2022)

= *Armeria undulata* f. *longifolia* Novák in Comm. Reg. Soc. Sci. Bohem. Mat. Nat. 28: 8. 1939 syn. sec. POWO (2017+: 6 May 2022)

***Armeria vandasii* Hayek in Repert. Spec. Nov. Regni Veg. 21: 257. 1925.** Sec. Pignatti (1972)

***Armeria velutina* Welw. ex Boiss. & Reut., Pugill. Pl. Afr. Bor. Hispan.: 100. 1852.** Sec. Bernis (1954)

≡ *Statice velutina* (Welw. ex Boiss. & Reut.) Samp., Herb. Port.: 100. 1913 syn. sec. Sampaio (1913)

***Armeria villosa* Girard in Ann. Sci. Nat., Bot., sér. 3, 2: 323. 1844.** Sec. Nieto Feliner (1987)

≡ *Statice villosa* (Girard) Jahand. & Maire in Jahandiez & al., Cat. Pl. Maroc 3: 567. 1934 syn. sec. Malekmohammadi & al. (2024) ≡ *Armeria pseudarmeria* subsp. *villosa* (Girard) Malag. in Acta Phytotax. Barcinon. 1: 24. 1968 ["1969"] syn. sec. Malekmohammadi & al. (2024)
= *Armeria allioides* Willk. & Lange, Prodr. Fl. Hispan. 2(2): 366. 1868 syn. sec. Domina (2011+)

***Armeria villosa* subsp. *alcaracensis* Nieto Fel. in Anales Jard. Bot. Madrid, ser. 2, 44: 337. 1987.** Sec. Nieto Feliner (1987)

***Armeria villosa* subsp. *bernisii* Nieto Fel. in Anales Jard. Bot. Madrid, ser. 2, 44: 337. 1987.** Sec. Nieto Feliner (1987)

= *Armeria maritima* var. *allioides* Bernis, nom. illeg. syn. sec. Nieto Feliner (1987)

***Armeria villosa* subsp. *carratracensis* (Bernis) Nieto Fel. in Anales Jard. Bot. Madrid, ser. 2, 44: 337. 1987.** Sec. Domina (2011+)

≡ *Armeria maritima* subvar. *carratracensis* Bernis in Anales Inst. Bot. Cavanilles 12(2): 122. 1955 syn. sec. Nieto Feliner (1987) ≡ *Armeria pseudarmeria* subvar. *carratracensis* Bernis in Anales Inst. Bot. Cavanilles 12(2): 122. 1955 syn. sec. Malagarriga (1968) ≡ *Armeria pseudarmeria* subsp. *carratracensis* (Bernis) Malag. in Acta Phytotax. Barcinon. 1: 24. 1968 ["1969"] syn. sec. Malekmohammadi & al. (2024) ≡ *Armeria carratracensis* (Bernis) Rivas Mart. in Lagascalia 15(Extra): 115. 1988 syn. sec. Domina (2011+)

***Armeria villosa* subsp. *enritrianoi* Blanca, Cueto, J.Fuentes & S.Tello in Acta Bot. Malac. 42: 288. 2017.** Sec. Blanca & al. (2017)

***Armeria villosa* subsp. *longiaristata* (Boiss. & Reut.) Nieto Fel. in Anales Jard. Bot. Madrid, ser. 2, 44: 336. 1987.** Sec. Blanca & al. (2017)

≡ *Armeria longiaristata* Boiss. & Reut., Pugill. Pl. Afr. Bor. Hispan.: 103. 1852 syn. sec. Nieto Feliner (1987) ≡ *Statice longiaristata* (Boiss. & Reut.) Maire in Bull. Soc. Hist. Nat. Afrique N. 22: 304. 1931 syn. sec. Malekmohammadi & al. (2024: 4 May 2022) − *Armeria villosa* subsp. *longiristata* (Boiss. & Reut.) Nieto Fel. in Anales Jard. Bot. Madrid, ser. 2, 44: 336. 1987, orth. var. syn. sec. Malekmohammadi & al. (2024) [is misspelling for *Armeria villosa* subsp. *longiaristata* (Boiss. & Reut.) Nieto Fel.]
= *Armeria duriaei* Cout. in Bull. Soc. Brot. 2: 145. 1884, nom. illeg. syn. sec. Daveau (1888) [non *Armeria duriaei* Boiss.]

***Armeria villosa* subsp. *provillosa* (Bernis) Nieto Fel. in Anales Jard. Bot. Madrid, ser. 2, 44: 337. 1987.** Sec. Domina (2011+)

≡ *Armeria maritima* var. *provillosa* Bernis, Rev. Gen. Armeria Comp.: 7. 1951 syn. sec. Nieto Feliner (1987)

***Armeria villosa* subsp. *serpentinicola* Cabezudo, Casim.-Sor.Solanas & Pérez Lat. in Acta Bot. Malac. 40: 64. 2015.** Sec. Cabezudo & al. (2015)

***Armeria villosa* Girard subsp. *villosa*.** Sec. Domina (2011+)

***Armeria welwitschii* Boiss. in Candolle, Prodr. 12: 676. 1848.** Sec. Nieto Feliner (1987)

≡ *Statice welwitschii* (Boiss.) Samp., Herb. Port.: 100. 1913 syn. sec. Domina (2011+) ≡ *Statice welwitschii* (Boiss.) F.T.Hubb. ex L.H.Bailey in Rhodora 18(211): 158. 1916 syn. sec. Malekmohammadi & al. (2024: 6 May 2022)
= *Statice armeria* Brot., Fl. Lucit. 1: 489. 1804 syn. sec. Daveau (1888)
= *Armeria littoralis* Boiss. in Candolle, Prodr. 12: 676. 1848 syn. sec. Nyman (1881)
= *Armeria cinerea* Boiss. & Welw. ex Boiss. & Reut., Pugill. Pl. Afr. Bor. Hispan.: 101. 1852 syn. sec. Domina (2011+) ≡ *Statice welwitschii* var. *cinerea* (Boiss. & Reut.) Samp., Fl. Port.: 439. 1947 syn. sec. Malekmohammadi & al. (2024: 26 April 2022) ≡ *Armeria welwitschii* var. *cinerea* (Boiss. & Welw.) Govaerts, World Checkl. Seed Pl. 1(1): 9. 1995 syn. sec. Malekmohammadi & al. (2024: 6 May 2022)
= *Armeria welwitschii* var. *longibracteata* Daveau in Bol. Soc. Brot. 6: 167. 1888 syn. sec. Malekmohammadi & al. (2024: 6 May 2022)
= *Armeria welwitschii* var. *stenophylla* Daveau in Bol. Soc. Brot. 6: 167. 1888 syn. sec. Malekmohammadi & al. (2024: 6 May 2022)
= *Armeria welwitschii* var. *diversifolia* Franco, Nova Fl. Portugal 2: 563. 1984 syn. sec. Malekmohammadi & al. (2024)

Bakerolimon Lincz. in Novosti Sist. Vyssh. Rast. 4: 175. 1968. Sec. Malekmohammadi & al. (2017)

Type: *Bakerolimon plumosum* (Phil.) Lincz.

Notes. – With two shrubby species, distributed in the deserts of Chile and Peru (Kubitzki 1993), *Bakerolimon* is placed in a clade with *Muellerolimon*, *Myriolimon* and *Saharanthus* based on latest phylogenetic studies (Malekmohammadi et al. 2017; Koutroumpa et al. 2018, 2021). The relation is further supported by morphological data (Baker 1953).

***Bakerolimon peruvianum* (Kuntze) Lincz. in Novosti Sist. Vyssh. Rast. 4: 175. 1968.** Sec. Malekmohammadi & al. (2024)

≡ *Limonium peruvianum* Kuntze, Revis. Gen. Pl. 2: 395. 1891 syn. sec. POWO (2017+)

***Bakerolimon plumosum* (Phil.) Lincz. in Novosti Sist. Vyssh. Rast. 4: 175. 1968.** Sec. Zuloaga & al. (2019)

≡ *Statice plumosa* Phil., Fl. Atacam: 46. 1860 syn. sec. Zuloaga & al. (2019) ≡ *Limonium plumosum* (Phil.) Kuntze, Revis. Gen. Pl. 2: 396. 1891 syn. sec. Zuloaga & al. (2019)

***Bamiania* Lincz. in Bot. Zhurn. (Moscow & Leningrad) 56(11): 1634. 1971.** Sec. Malekmohammadi & al. (2024)

Type: *Bamiania pachycorma* (Rech.f.) Lincz.

Notes. – A monotypic genus from Afghanistan, which is phylogenetically placed in a well-supported clade with *Popoviolimon* and *Cephalorhizum* in the study by Moharrek et al. (2017).

***Bamiania pachycorma* (Rech.f.) Lincz. in Bot. Zhurn. (Moscow & Leningrad) 56(11): 1635. 1971.** Sec. Malekmohammadi & al. (2024)

≡ *Cephalorhizum pachycormum* Rech.f. in Anz. Österr. Akad. Wiss., Math.-Naturwiss. Kl. 101: 402. 1964 syn. sec. Linczevski (1971)

Distribution (general). – Afghanistan.

***Bukiniczia* Lincz. in Bot. Zhurn. (Moscow & Leningrad) 56(11): 1634. 1971.** Sec. Malekmohammadi & al. (2024)

Type: *Bukiniczia cabulica* (Boiss.) Lincz.
= *Aeoniopsis* Rech.f., Fl. Iran. 108: 24. 1974 syn. sec. Malekmohammadi & al. (2024). Type: *Aeoniopsis cabulica* (Boiss.) Rech.f.

Notes. – A monotypic genus comprising *Bukiniczia cabulica* distributed in Afghanistan and Pakistan. The genus is sister to *Dictyolimon* with which is morphologically related and they both form a well-supported clade (Moharrek et al. 2017; Koutroumpa et al. 2018).

***Bukiniczia cabulica* (Boiss.) Lincz. in Bot. Zhurn. (Moscow & Leningrad) 56(11): 1634. 1971.** Sec. Moharrek & al. (2017)

≡ *Statice cabulica* Boiss. in Candolle, Prodr. 12: 666. 1848 syn. sec. Rechinger & Schiman-Czeika (1974: synonym of Aeoniopsis cabulica) ≡ *Limonium cabulicum* (Boiss.) Kuntze, Revis. Gen. Pl. 2: 395. 1891 syn. sec. Kuntze (1891: synonym of Statice cabulica Boiss.) ≡ *Goniolimon cabulicum* (Boiss.) Mobayen, Revis. Taxon. Acanth.: 311. 1964 syn. sec. Malekmohammadi & al. (2024) ≡ *Aeoniopsis cabulica* (Boiss.) Rech.f., Fl. Iran. 108: 25. 1974 syn. sec. Malekmohammadi & al. (2024)

Notes. – The species was represented by two individuals in the phylogeny of Moharrek et al. (2017) and its monophyly was highly supported.

***Cephalorhizum* Popov & Korovin in Trudy Turkestansk. Nauchn. Obshch. 1: 39. 1923.** Sec. Malekmohammadi & al. (2024)

Type: not designated
= *Cephalorrhizum* Popov & Korovin, orth. var. syn. sec. Malekmohammadi & al. (2024)

Notes. – Four species from Afghanistan and Central Asia. One species was sampled (*Cephalorhizum coelicolor*) and found in a well-supported clade together with *Popoviolimon* and *Bamiania* in Moharrek et al. (2017).

***Cephalorhizum coelicolor* (Rech.f.) Rech.f. in Anz. Österr. Akad. Wiss., Math.-Naturwiss. Kl. 101: 403. 1964.** Sec. Rechinger & Schiman-Czeika (1974)

≡ *Limonium coelicolor* Rech.f. in Biol. Skr. 13(4) (Symb. Afghan. 5): 136. 1963 syn. sec. Malekmohammadi & al. (2024)

Notes. – Species monophyly was tested and confirmed in Moharrek et al.'s (2017) phylogeny by sampling two individuals of the species.

***Cephalorhizum micranthum* Lincz., Fl. Tadzhikskoĭ SSR 8: 481. 1986.** Sec. Malekmohammadi & al. (2024)

***Cephalorhizum oopodum* Popov & Korovin in Trudy Turkestansk. Nauchn. Obshch. 1: 39. 1923.** Sec. Malekmohammadi & al. (2024)

≡ *Acantholimon oopodum* (Popov & Korovin) Sennikov, Fl. Uzbekist. (Toshkent) 3: 24. 2019 syn. sec. Malekmohammadi & al. (2024)

***Cephalorhizum popovii* Lincz. in Novosti Sist. Vyssh. Rast. 1: 263. 1964.** Sec. Malekmohammadi & al. (2024)

***Ceratolimon* M.B.Crespo & Lledó in Bot. J. Linn. Soc. 132: 169. 2000.** Sec. Koutroumpa & al. (2018)

Type: *Ceratolimon feei* (Girard) M.B.Crespo & Lledó
– *Bubania* Girard in Mém. Sect. Sci. Acad. Sci. Montpellier, 1: 182. 1848, nom. superfl. syn. sec. Crespo & Lledó (2000). Type: *Bubania monopetala* (L.) Girard
= *Limoniastrum* sect. *Bubania* Batt., Fl. Algérie [1](4): 726. 1890 syn. sec. Crespo & Lledó (2000)
≡ *Limoniastrum* subg. *Bubania* (Batt.) Maire in Bull. Soc. Hist. Nat. Afrique N. 27: 247. 1936 syn. sec. Crespo & Lledó (2000)

Notes. – A genus segregated from *Limoniastrum* including three species of dwarf shrubs with rosulate leaves and long spiny inner floral bract, which show disjunct distributions on the Atlantic and Indian edges of the Sahara Desert (Lledó and Crespo

2000). In phylogenetic studies its species composed a well-supported monophyletic group that is sister to *Limoniastrum* (Crespo and Lledó 2000; Koutroumpa et al. 2018).

***Ceratolimon feei* (Girard) M.B.Crespo & Lledó in Bot. J. Linn. Soc. 132: 170. 2000.** Sec. Crespo & Lledó (2000)

≡ *Bubania feei* Girard in Mém. Sect. Sci. Acad. Sci. Montpellier, 1: 185. 1848 syn. sec. Crespo & Lledó (2000) ≡ *Limoniastrum feei* (Girard) Hook.f. ex Pax, Nat. Pflanzenfam. 4(1): 125. 1889 syn. sec. Crespo & Lledó (2000)

***Ceratolimon feei* var. *feei*.** Sec. Crespo & Lledó (2000)

***Ceratolimon feei* var. *grandiflorum* (Maire & Wilcz.) M.B.Crespo & Lledó in Bot. J. Linn. Soc. 132: 171. 2000.** Sec. Crespo & Lledó (2000)

≡ *Limoniastrum feei* var. *grandiflorum* Maire & Wilcz. in Bull. Soc. Hist. Nat. Afrique N. 26: 129. 1935 syn. sec. Crespo & Lledó (2000)

Distribution (general). – This variety grows together with *Ceratolimon* var. *feei*, but is more common in the southwestern littoral mountains of Morocco in the Saharan Province of the Saharo-Arabian Region (Crespo and Lledó 2000).

***Ceratolimon migiurtinum* (Chiov.) M.B.Crespo & Lledó in Bot. J. Linn. Soc. 132: 171. 2000.** Sec. Crespo & Lledó (2000)

≡ *Bubania migiurtina* Chiov., Fl. Somala 1: 212. 1929 syn. sec. Crespo & Lledó (2000) ≡ *Limoniastrum migiurtinum* (Chiov.) Chiov. ex Maire in Bull. Soc. Hist. Nat. Afrique N. 27: 248. 1936 syn. sec. Crespo & Lledó (2000) − *Limoniastrum migiurtinum* Chiov., Fl. Somala 1: 212. 1929, nom. inval. syn. sec. Crespo & Lledó (2000)
= *Limoniastrum rechingeri* J.R.Edm. in Ann. Naturhist. Mus. Wien, B 98(Suppl.): 400. 1996 syn. sec. Crespo & Lledó (2000) ≡ *Ceratolimon rechingeri* (J.R.Edm.) M.B.Crespo & Lledó in Bot. J. Linn. Soc. 132: 171. 2000 syn. sec. Thulin (2006: 8 July 2022)

***Ceratolimon weygandiorum* (Maire & Wilczek) M.B.Crespo & Lledó in Bot. J. Linn. Soc. 132: 172. 2000.** Sec. Crespo & Lledó (2000)

≡ *Limoniastrum weygandiorum* Maire & Wilczek in Bull. Soc. Hist. Nat. Afrique N. 26: 129. 1935 syn. sec. Crespo & Lledó (2000)

Chaetolimon (Bunge) Lincz. in Trudy Tadzikisk. Bazy 8: 586. 1940. Sec. Malekmohammadi & al. (2024)

Type: *Chaetolimon sogdianum* Lincz.
≡ *Acantholimon* sect. *Chaetolimon* Bunge in Mém. Acad. Imp. Sci. Saint Pétersbourg, Sér. 7 18(2): 68. 1872 syn. sec. Moharrek & al. (2017)

Notes. – Comprising two species distributed in Kazakhstan, Kirgizstan, Tadzhikistan, Turkmenistan and Uzbekistan, the genus represented by *Chaetolimon setiferum* in Moharrek et al. (2017), is morphologically related and phylogenetically sister to *Vassilczenkoa*.

***Chaetolimon limbatum* Lincz. in Trudy Tadzikisk. Bazy 8: 595. 1940.** Sec. Malekmohammadi & al. (2024)

≡ *Acantholimon limbatum* (Lincz.) Sennikov, Fl. Uzbekist. 3: 27. 2019 syn. sec. Malekmohammadi & al. (2024)

***Chaetolimon setiferum* (Bunge) Lincz. in Trudy Tadzikisk. Bazy 8: 591. 1940.** Sec. Malekmohammadi & al. (2024)

≡ *Acantholimon setiferum* Bunge in Mém. Acad. Imp. Sci. Saint Pétersbourg, Sér. 7 18(2): 68. 1872 syn. sec. Malekmohammadi & al. (2024) ≡ *Armeriastrum setiferum* (Bunge) Kuntze, Revis. Gen. Pl. 2: 394. 1891 syn. sec. POWO (2017+) ≡ *Cephalorhizum setiferum* (Bunge) Popov & Korovin in Trudy Turkestansk. Nauchn. Obshch. 1: 39. 1923 syn. sec. POWO (2017+)

Dictyolimon Rech.f., Fl. Iran. 108: 21. 1974. Sec. Malekmohammadi & al. (2024)

Type: *Dictyolimon macrorrhabdos* (Boiss.) Rech.f.
= *Statice* sect. *Schizopetalum* Boiss., Diagn. Pl. Orient. 2(4): 67. 1859 syn. sec. Rechinger & Schiman-Czeika (1974)
≡ *Statice* subg. *Schizopetalum* (Boiss.) Melchior in Notizbl. Bot. Gart. Berlin 14: 373. 1940 syn. sec. Rechinger & Schiman-Czeika (1974)

Notes. – Three species distributed in Afghanistan, Pakistan and India. The monophyly of the genus was tested and confirmed in Moharrek et al. (2017) by sampling *Dictyolimon macrorrhabdos* and *D. griffithii* which formed a well-supported clade sister to *Bukiniczia*.

***Dictyolimon gilesii* (Hemsl.) Rech.f. in Biol. Skr. 13(4) (Symb. Afgan. 5): 138. 1963.** Sec. Rechinger & Schiman-Czeika (1974)

≡ *Statice gilesii* Hemsl. in Hookers Icon. Pl. 18: t. 1737. 1888 syn. sec. Rechinger & Schiman-Czeika (1974)

***Dictyolimon griffithii* (Aitch. & Hemsl.) Rech.f., Fl. Iran. 108: 23. 1974.** Sec. Rechinger & Schiman-Czeika (1974)

≡ *Statice griffithii* Aitch. & Hemsl. in J.Linn.Soc., Bot. 19: 175. 1882 syn. sec. Rechinger & Schiman-Czeika (1974) ≡ *Limonium griffithii* (Aitch. & Hemsl.) Kuntze, Revis. Gen. Pl. 2: 395. 1891 syn. sec. Rechinger & Schiman-Czeika (1974) ≡ *Goniolimon griffithianum* (Aitch. & Hemsl.) Mobayen, Revis. Taxon. Acanth.: 311. 1964 syn. sec. Rechinger & Schiman-Czeika (1974)

***Dictyolimon macrorrhabdos* (Boiss.) Rech.f., Fl. Iran. 108: 22. 1974.** Sec. Rechinger & Schiman-Czeika (1974)

≡ *Statice macrorrhabdos* Boiss., Diagn. Pl. Orient. ser. 2, 4: 67. 1859 syn. sec. Rechinger & Schiman-Czeika (1974) ≡ *Limonium macrorhabdon* (Boiss.) Kuntze, Revis. Gen. Pl. 2: 395. 1891 syn. sec. Rechinger & Schiman-Czeika (1974)

= *Statice macrorrhabdos* var. *thomsonii* C.B.Clarke, Fl. Brit. Ind. 3: 480. 1882 [as " *thomsoni* "] syn. sec. Rechinger & Schiman-Czeika (1974) ≡ *Dictyolimon thomsonii* (C.B.Clarke) Rech.f., Fl. Iran. 108: 22. 1974 syn. sec. Rechinger & Schiman-Czeika (1974) − *Statice macrorrhabdos* var. *thomsoni* C.B.Clarke, orth. var. syn. sec. Rechinger & Schiman-Czeika (1974)

Notes. – Monophyly of the species was comfirmed by Moharrek et al. (2017) by sampling two individuals of the species.

Ghaznianthus **Lincz. in Novosti Sist. Vyssh. Rast. 16: 167. 1979.** Sec. Kubitzki (1993)

Type: *Ghaznianthus rechingeri* (Freitag) Lincz.

Notes. – Monotypic genus from Afghanistan. No sequence data are available yet.

Ghaznianthus rechingeri **(Freitag) Lincz. in Novosti Sist. Vyssh. Rast. 16: 167. 1979.** Sec. Malekmohammadi & al. (2024)

≡ *Acantholimon rechingeri* Freitag in Ann. Naturhist. Mus. Wien 75: 67. 1972 syn. sec. Malekmohammadi & al. (2024)

Goniolimon **Boiss. in Candolle, Prodr. 12: 632. 1848.** Sec. Koutroumpa & al. (2018)

Type: *Goniolimon tataricum* (L.) Boiss.
≡ *Statice* sect. *Goniolimon* (Boiss.) Hook., Gen. Pl. 2: 626. 1876 syn. sec. Koutroumpa & al. (2018)
= *Statice* sect. *Tropidice* Griseb., Spic. Fl. Rumel. 2(5-6): 299-300. 1846 syn. sec. Malekmohammadi & al. (2024)
= *Ikonnikovia* Lincz. in Schischkin & Bobrov, Flora URSS 18: 745. 1952 syn. sec. Koutroumpa & al. (2018). Type: *Ikonnikovia kaufmanniana* (Regel) Lincz.

Notes. – Containing about 20 species with perennial habit and rosulate leaves distributed from Mongolia to Italy and mostly diversified in Central Asia (Kubitzki 1993; Linczevski 1952). In phylogenetic studies *Goniolimon* was recovered as sister to the *Acantholimon s.l.* clade (Moharrek et al. 2017) and its status as a monophyletic genus is followed here with *Ikonnikovia* nested in it (Koutroumpa et al. 2018).

Goniolimon africanum **Buzurović, Bogdanović & Brullo in Phytotaxa 349(3): 288. 2018.** Sec. Malekmohammadi & al. (2024)

Goniolimon besserianum **Nyman, Consp. Fl. Eur. 3: 613. 1881.** Sec. Linczevski (1952)

= *Statice incana* M.Bieb., Fl. Taur.-Caucas. 1: 251. 1808 syn. sec. Boissier (1848: non L.)
= *Statice graminifolia* Besser, Enum. Pl.: 44. 1822 syn. sec. Malekmohammadi & al. (2024: 30 March 2023)
= *Statice besseriana* Schult. ex Rchb., Iconogr. Bot. Pl. Crit. 8: f. 962. 1830 syn. sec. Boissier (1848) ≡ *Limonium besserianum* (Schult. ex Rchb.) Kuntze, Revis. Gen. Pl. 2: 395. 1891 syn. sec. Linczevski (1952)
= *Goniolimon tataricum* var. *angustifolium* Boiss. in Candolle, Prodr. 12: 633. 1848 syn. sec. Linczevski (1952) ≡ *Limonium tataricum* var. *angustifolium* (Boiss.) F.T.Hubb. in Rhodora 18(211): 159. 1916 syn. sec. Bailey (1916) − *Statice tatarica* var. *angustifolium* hort. ex F.T.Hubb. in Rhodora 18(211): 159. 1916, nom. nud. syn. sec. Bailey (1916)
= *Goniolimon tataricum* var. *besserianum* O.Fedtsch. & B.Fedtsch., Consp. Fl. Turkestanicae 5: 180. 1913 syn. sec. Malekmohammadi & al. (2024)
− *Statice incana* var. *hybrida* hort. ex F.T.Hubb. in Rhodora 18(211): 159. 1916, nom. nud. syn. sec. Bailey (1916)

Goniolimon callicomum **(C.A.Mey.) Boiss. in Candolle, Prodr. 12: 633. 1848.** Sec. Linczevski (1952)

≡ *Statice callicoma* C.A.Mey. in Mém. Acad. Imp. Sci. St.-Pétersbourg, Divers Savans 4: 212. 1841 syn. sec. Linczevski (1952) ≡ *Limonium callicomum* (C.A.Mey.) Kuntze, Revis. Gen. Pl. 2: 395. 1891 syn. sec. Linczevski (1952)
= *Statice argentea* Pall. ex Siev. in Neueste Nord. Beytr. Phys. Geogr. Erd-Völkerbeschreib. 7: 282. 1796 syn. sec. Linczevski (1952)
= *Statice incana* Ledeb., Fl. Alt. 1: 435. 1829 syn. sec. Linczevski (1952: non L.)
= *Statice albicans* Ledeb., Fl. Ross. 3(1,9): 465. 1849 syn. sec. Malekmohammadi & al. (2024: 10 May 2022)

Goniolimon callicomum **(C.A.Mey.) Boiss. var. *callicomum*.** Sec. Linczevski (1952)

Goniolimon callicomum **var. *gorczakovskyi* (Knjaz.) Knjaz. & Golovanov, Turczaninowia 22(4): 35. 2019.** Sec. Malekmohammadi & al. (2024)

≡ *Goniolimon gorczakovskyi* Knjaz. in Novosti Sist. Vyssh. Rast. 45: 52. 2014 syn. sec. Malekmohammadi & al. (2024)

Goniolimon caucasicum **Klokov in Fl. URSR 8: 522. 1958.** Sec. Malekmohammadi & al. (2024)

= *Goniolimon tataricum* var. *puberulum* Trautv. in Bull. Acad. Sci. Pétersb. 14: 250. 1856 syn. sec. Linczevski (1952)

Goniolimon cuspidatum **Gamajun. in Vestn. Akad. Nauk Kazakhsk. S.S.R. 1: 79. 1951.** Sec. Linczevski (1952)

Goniolimon dalmaticum **(C.Presl) Rchb., Icon. Fl. Germ. Helv. 17: 61. 1855.** Sec. Pignatti (1972)

≡ *Statice dalmatica* C.Presl in Abh. Königl. Böhm. Ges. Wiss. ser. 5, 3: 535. 1845 syn. sec. Nyman (1881) ≡ *Limonium dalmaticum* (C.Presl) Kuntze, Revis. Gen. Pl. 2: 395. 1891 syn. sec. Kuntze (1891)
= *Statice incana* Vis., Fl. Dalmat. 2: 7. 1847 syn. sec. Nyman (1881)
= *Statice collina* Nyman, Consp. Fl. Eur. 3: 613. 1881 syn. sec. Nyman (1881)

Goniolimon dshungaricum **(Regel) O.Fedtsch. & B.Fedtsch., Consp. Fl. Turkestanicae 5: 179. 1913.** Sec. Linczevski (1952)

≡ *Statice dschungarica* Regel in Trudy Imp. S.-Peterburgsk. Bot. Sada 6(2): 386. 1880 syn. sec. Linczevski (1952)
= *Statice speciosa* var. *lanceolata* Regel in Trudy Imp. S.-Peterburgsk. Bot. Sada, prepr. 6: 387, 389. 1879 syn. sec. Linczevski (1952) ≡ *Goniolimon speciosum* var. *lanceolatum* (Regel) O.Fedtsch. & B.Fedtsch., Consp. Fl. Turkestanicae 5: 179. 1913 syn. sec. Linczevski (1952)

= *Goniolimon tarbagataicum* Gamajun. in Vestn. Akad. Nauk Kazakhsk. S.S.R. 1: 80. 1951 syn. sec. Linczevski (1952)

***Goniolimon elatum* (Fisch. ex Spreng.) Boiss. in Candolle, Prodr. 12: 634. 1848.** Sec. Linczevski (1952)

≡ *Statice elata* Fisch. ex Spreng., Syst. Veg., ed. 16, 1: 957. 1824 syn. sec. Linczevski (1952) ≡ *Taxanthema elata* (Fisch. ex Spreng.) Sweet, Hort. Brit.: 332. 1826 syn. sec. Malekmohammadi & al. (2024) ≡ *Limonium elatum* (Fisch. ex Spreng.) Kuntze, Revis. Gen. Pl. 2: 395. 1891 syn. sec. Linczevski (1952)
= *Statice flexuosa* Less. in Linnaea 9(2): 196. 1834 syn. sec. Boissier (1848)

***Goniolimon eximium* (Schenk) Boiss. in Candolle, Prodr. 12: 634. 1848.** Sec. Linczevski (1952)

≡ *Statice eximia* Schrenk, Enum. Pl. Nov. 1: 13. 1841 syn. sec. Linczevski (1952) ≡ *Limonium eximium* (Schrenk ex Fisch. & C.A.Mey.) H.Arnaud in Ser., Fl. Jard. 3: 302. 1849 syn. sec. Linczevski (1952) − *Limonium eximium* (Schrenk) Kuntze, Revis. Gen. Pl. 2: 395. 1891 syn. sec. Linczevski (1952) [is later isonym of *Limonium eximium* (Schrenk ex Fisch. & C.A.Mey.) H.Arnaud]
= *Statice eximia* var. *turkestanica* Regel in Gartenflora 37: 194, 266. 1888 syn. sec. Linczevski (1952)

***Goniolimon graminifolium* (Aiton) Boiss. in Candolle, Prodr. 12: 633. 1848.** Sec. Linczevski (1952)

≡ *Statice graminifolia* Aiton, Hort. Kew. 1: 383. 1789 syn. sec. Nyman (1881) ≡ *Taxanthema graminifolia* (Aiton) Sweet, Hort. Brit. 2: 332. 1826 syn. sec. Malekmohammadi & al. (2024) ≡ *Limonium graminifolium* (Aitch.) Kuntze, Revis. Gen. Pl. 2: 395. 1891 syn. sec. Linczevski (1952)
= *Statice desertorum* Trautv., Pl. Imag. Descr. Fl. Russ. 17: t. 11. 1844 syn. sec. Malekmohammadi & al. (2024) ≡ *Goniolimon tataricum* var. *desertorum* Trautv. in Bull. Acad. Sc. Peters. 14: 251,252. 1856 syn. sec. Linczevski (1952) ≡ *Limonium desertorum* (Trautv.) Kuntze, Revis. Gen. Pl. 2: 395. 1891 syn. sec. Kuntze (1891) ≡ *Goniolimon desertorum* (Trautv.) Klokov, Ind. Sem. Hort. Bot. Charcov.: 8. 1927 syn. sec. Linczevski (1952)
= *Goniolimon tataricum* var. *laxiflorum* Boiss. in Candolle, Prodr. 12: 633. 1848 syn. sec. Linczevski (1952)
= *Goniolimon tataricum* var. *graminifolium* Trautv. in Bull. Acad. Sc. Peters. 14: 251,252. 1856 syn. sec. Linczevski (1952)
= *Statice graminifolia* var. *desertorum* Regel in Trudy Imp. S.-Peterburgsk. Bot. Sada 6(2). 1880 syn. sec. Linczevski (1952)

***Goniolimon heldreichii* Halácsy in Verh. K.K. Zool.-Bot. Ges. Wien 36: 241. 1886.** Sec. Pignatti (1972)

− *Statice heldreichii* Halácsy in Verh. K.K. Zool.-Bot. Ges. Wien 36: 241. 1886, nom. inval. syn. sec. Malekmohammadi & al. (2024)

***Goniolimon incanum* (L.) Hepper, Fl. Turkey 10: 212. 1988.** Sec. Greuter, Burdet & G.Long 1989: Med. Checklist 4

≡ *Statice incana* L., Mant. Pl.: 59. 1767 syn. sec. Malekmohammadi & al. (2024) ≡ *Limonium incanum* (L.) Chaz. in Miller, Suppl. Dict. Jard. 2: 34. 1790 syn. sec. Govaerts, R. (ed.) (2023) ≡ *Taxanthema incana* (L.) Sweet, Hort. Brit.: 332. 1826 syn. sec. Malekmohammadi & al. (2024: 10 May 2022) ≡ *Limonium incanum* (L.) Kuntze, Revis. Gen. Pl. 2: 395. 1891, nom. superfl. syn. sec. Malekmohammadi & al. (2024)
= *Statice incana* d'Urv. ex Boiss. in Candolle, Prodr. 12: 672. 1848 syn. sec. Boissier (1848)
= *Statice trigonoides* Boiss. in Candolle, Prodr. 12: 633. 1848 syn. sec. Boissier (1848)
= *Limonium trigonodes* Kuntze, Revis. Gen. Pl. 2: 395. 1891 syn. sec. Kuntze (1891)
= *Statice speciosa* Forssk., Fl. Aegypt.-Arab.: 14. 1775 syn. sec. Nyman (1881)
= *Statice trigonoides* Pall., Reise Russ. Reich. 3(2): 637. 1776 syn. sec. Malekmohammadi & al. (2024)
= *Statice tatarica* var. *trigonoides* Poir., Encycl. 7: 400. 1817 syn. sec. Kuntze (1891)
= *Statice collina* Griseb., Spic. Fl. Rumel. 2: 300. 1846 ["1844"] syn. sec. Greuter, Burdet & G.Long 1989: Med. Checklist 4 ≡ *Goniolimon collinum* (Griseb.) Boiss. in Candolle, Prodr. 12: 633. 1848 syn. sec. Greuter, Burdet & G.Long 1989: Med. Checklist 4 ≡ *Limonium collinum* (Griseb.) F.T.Hubb. ex L.H.Bailey in Rhodora 18(211): 158. 1916 syn. sec. Greuter, Burdet & G.Long 1989: Med. Checklist 4
= *Statice besseriana* Friv. ex Boiss. in Candolle, Prodr. 12: 633. 1848 syn. sec. Boissier (1848)

***Goniolimon kaufmannianum* (Regel) Voss, Vilm. Blumengärtn., ed. 3, 1: 614. 1895.** Sec. Linczevski (1952)

≡ *Statice kaufmanniana* Regel in Trudy Imp. S.-Peterburgsk. Bot. Sada 6(2): 300. 1880 syn. sec. Malekmohammadi & al. (2024) ≡ *Limonium kaufmannianum* (Regel) Kuntze, Revis. Gen. Pl. 2: 395. 1891 syn. sec. Malekmohammadi & al. (2024) ≡ *Goniolimon kaufmannianum* (Regel) O.Fedtsch. & B.Fedtsch., Consp. Fl. Turkestanicae 5: 180. 1913 syn. sec. Malekmohammadi & al. (2024) ≡ *Ikonnikovia kaufmanniana* (Regel) Lincz. in Schischkin & Bobrov, Flora URSS 18: 381, pl. 19, f. 3. 1952 syn. sec. Malekmohammadi & al. (2024)
= *Ikonnikovia kaufmanniana* var. *latifolia* Z.B.Kubanskaya ex Lincz. in Schischkin & Bobrov, Flora URSS 18: 382. 1952 syn. sec. Linczevski (1952)

***Goniolimon krylovii* A.V.Grebenjuk, Konspekt Fl. Aziatsk. Rossii: 116. 2012.** Sec. Malekmohammadi & al. (2024)

= *Statice speciosa* var. *multicaulis* Krylov, Fl. Altai Gov. Tomsk 4: 1078. 1907 syn. sec. Malekmohammadi & al. (2024)

***Goniolimon orthocladum* Rupr. in Mém. Acad. Imp. Sci. Saint Pétersbourg, Sér. 7, 14(4): 69. 1869.** Sec. Linczevski (1952)

= *Goniolimon speciosum* var. *alpinum* Herd. in Bull. Soc. Nat. Mosc. 41: 396. 1868 syn. sec. Linczevski (1952)
= *Statice speciosa* var. *crispa* Regel in append. syn. sec. Malekmohammadi & al. 2024 in Trudy Imp. S.-Peterburgsk. Bot. Sada 6(2): 387,389. 1880 syn. sec. Malekmohammadi & al. (2024)
= *Statice speciosa* var. *lepidota* Regel in Trudy Imp. S.-Peterburgsk. Bot. Sada 6(2): 387,389. 1880 syn. sec. Linczevski (1952)
= *Goniolimon speciosum* var. *crispum* O.Fedtsch & B.Fedtsch., Rast. Turk. 5: 179. 1913 syn. sec. Linczevski (1952)

***Goniolimon rubellum* (S.G.Gmel.) Klokov in Grossheim, Opred. Rast. Kauk.: 593. 1949.** Sec. Linczevski (1952)

≡ *Statice rubella* S.G.Gmel., Reise Russland 2: 199. 1774 syn. sec. Malekmohammadi & al. (2024)
= *Goniolimon tataricum* var. *rubellum* Trautv. in Bull. Acad. Sc. Petersb. 14: 251. 1856 syn. sec. Linczevski (1952)

= *Goniolimon orae-syvashicae* Klokov, Ind. Sem. Hort. Bot. Charcov.: 8. 1927 syn. sec. Linczevski (1952)

***Goniolimon sartorii* Boiss., Diagn. Pl. Orient. ser. 2, 4: 67. 1859.** Sec. Pignatti (1972)

≡ *Statice sartorii* (Boiss.) Nyman, Syll. Fl. Eur. Suppl.: 25. 1865 syn. sec. Nyman (1881) ≡ *Limonium sartorii* (Nyman) Kuntze, Revis. Gen. Pl. 2: 396. 1891 syn. sec. Nyman (1881)

***Goniolimon sewerzowii* Herder in Bull. Soc. Imp. Naturalistes Moscou 41(1): 396. 1868.** Sec. Linczevski (1952)

≡ *Statice sewerzowii* (Herder) Regel, Trudy Imp. S.-Peterburgsk. Bot. Sada 6: 386. 1879 syn. sec. Linczevski (1952) ≡ *Limonium sewerzowii* (Herder) Kuntze, Revis. Gen. Pl. 2: 396. 1891 syn. sec. Linczevski (1952)
= *Statice alatavica* Regel & Schmalh. in Trudy Imp. S.-Peterburgsk. Bot. Sada 5: 259. 1877 syn. sec. Linczevski (1952) ≡ *Statice sewerzowii* var. *alatavica* Regel, Trudy Imp. S.-Peterburgsk. Bot. Sada 6: 387. 1879 syn. sec. Linczevski (1952)
− *Goniolimon severzovii* Herder in Bull. Soc. Imp. Naturalistes Moscou 41(1): 396. 1868 syn. sec. Malekmohammadi & al. (2024) [is misspelling for *Goniolimon sewerzowii* Herder]
− *Goniolimon sewerzovii* Herder in Bull. Soc. Imp. Naturalistes Moscou 41(1): 396. 1868 syn. sec. Malekmohammadi & al. (2024) [is misspelling for *Goniolimon sewerzowii* Herder]
− *Statice sewerzowii* var. *typica* Regel in Trudy Imp. S.-Peterburgsk. Bot. Sada 6: 387. 1879, nom. inval. syn. sec. Linczevski (1952)

***Goniolimon speciosum* (L.) Boiss. in Candolle, Prodr. 12: 634. 1848.** Sec. Linczevski (1952)

≡ *Statice speciosa* L., Sp. Pl.: 275. 1753 syn. sec. Linczevski (1952) ≡ *Limonium speciosum* (L.) Chaz. in Miller, Suppl. Dict. Jard. 2: 34. 1790 syn. sec. Malekmohammadi & al. (2024) ≡ *Limoniastrum speciosum* (L.) Moench, Suppl. Meth.: 149. 1802 syn. sec. Malekmohammadi & al. (2024) ≡ *Taxanthema speciosa* (L.) Sweet, Hort. Brit.: 333. 1826 syn. sec. Malekmohammadi & al. (2024) ≡ *Limonium speciosum* (L.) Kuntze, Revis. Gen. Pl. 2: 396. 1891 syn. sec. Linczevski (1952) ≡ *Goniolimon speciosum* (L.) Boiss. var. *speciosum* syn. sec. Malekmohammadi & al. (2024)
= *Statice conspicua* Sims, Bot. Mag. 39: tab. 1629. 1814 syn. sec. Linczevski (1952) ≡ *Taxanthema conspicua* (Sims) Sweet, Hort. Brit.: 333. 1829 syn. sec. Malekmohammadi & al. (2024)
= *Statice ochrantha* Kar. & Kir. in Bull. Soc. Imp. Naturalistes Moscou 14: 730. 1841 syn. sec. Kuntze (1891) ≡ *Limonium ochranthum* (Kar. & Kir.) Kuntze, Revis. Gen. Pl. 2: 396. 1891 syn. sec. Malekmohammadi & al. (2024)
= *Statice speciosa* var. *stricta* Regel in Trudy Imp. S.-Peterburgsk. Bot. Sada 6(2): 387. 1880 syn. sec. Malekmohammadi & al. (2024)
= *Statice speciosa* var. *typica* Regel in append. syn. sec. Linczevski 1952 in Trudy Imp. S.-Peterburgsk. Bot. Sada 6(2): 387. 1880 syn. sec. Linczevski (1952)
= *Goniolimon speciosum* var. *typicum* O.Fedtsch & B.Fedtsch, Consp. Fl. Turkestanicae 5: 179. 1913 syn. sec. Malekmohammadi & al. (2024)
= *Goniolimon crispum* (Regel) Lipsch., Fl. URSS 18: 394. 1952 syn. sec. Malekmohammadi & al. (2024)
= *Goniolimon strictum* (Regel) Lincz. in Schischkin & Bobrov, Flora URSS 18: 395. 1952 syn. sec. Malekmohammadi & al. (2024) ≡ *Goniolimon speciosum* var. *strictum* (Regel) T.H.Peng, Fl. Reipubl. Popularis Sin. 60(1): 24. 1987 syn. sec. Malekmohammadi & al. (2024)
= *Goniolimon speciosum* var. *genuinum* Herd. in Bull. Soc. Nat. Mosc. 41: 395. 1868 syn. sec. Linczevski (1952) − *Statice speciosa* var. *genuina* Kryl., Fl. Alt. 4: 389. 1907, nom. inval. syn. sec. Linczevski (1952)

***Goniolimon tataricum* (L.) Boiss. in Candolle, Prodr. 12: 632. 1848.** Sec. Linczevski (1952)

≡ *Statice tatarica* L., Sp. Pl.: 275. 1753 syn. sec. Linczevski (1952) ≡ *Limonium tataricum* (L.) Mill., Gard. Dict., ed. 8: no. 5. 1768 syn. sec. Linczevski (1952) ≡ *Taxanthema tatarica* (L.) Sweet, Hort. Brit.: 333. 1826 syn. sec. Malekmohammadi & al. (2024)
= *Statice terekiensis* Gueldenst., Reis. Russland (Gueldenst.) 2: 33. 1791 syn. sec. Malekmohammadi & al. (2024)
= *Statice triquetra* Boeber, N. Nord. Beitr. 6: 261. 1793 syn. sec. Malekmohammadi & al. (2024)
= *Limonium latifolium* Moench, Methodus: 623. 1794 syn. sec. Malekmohammadi & al. (2024)
= *Statice trigona* Pall., Tabl. Phys. Topogr. Taur.: 49. 1795 syn. sec. Malekmohammadi & al. (2024)
= *Goniolimon serbicum* Vis. in Mem. Reale Ist. Veneto Sci. 10: 440. 1861 syn. sec. Buzurović & al. (2013) ≡ *Statice serbica* (Vis.) Nyman, Syll. Fl. Eur. Suppl.: 25. 1865 syn. sec. Buzurović & al. (2013) ≡ *Limonium serbicum* (Nyman) Kuntze, Revis. Gen. Pl. 2: 396. 1891 syn. sec. Buzurović & al. (2013)
= *Goniolimon beckerianum* Janka in Természetrajzi Füz. 6: 169. 1882 syn. sec. Malekmohammadi & al. (2024) ≡ *Statice incana* Becker, Pl. exs. e Sarepta al Wolgam inferiorem, nom. illeg. syn. sec. Malekmohammadi & al. (2024)
= *Goniolimon collinum* var. *bulgaricum* Novák in Izv. Bulg. Bot. Druzh. 7: 58. 1936 syn. sec. Malekmohammadi & al. (2024) ≡ *Goniolimon tataricum* f. *bulgaricum* (Novák) Ančev, Fl. Nar. Republ. Bulgariya 8: 351. 1982 syn. sec. Malekmohammadi & al. (2024)
= *Goniolimon tataricum* f. *ciliatum* Ančev, Fl. Nar. Republ. Bulgariya 8: 484. 1982 syn. sec. Malekmohammadi & al. (2024)
= *Goniolimon tataricum* var. *kluchoricum* Tzvelev, Konspekt Fl. Kavkaza 3(2): 279. 2012 syn. sec. POWO (2017+)
− *Statice tatarica* var. *typica* Regel in Trudy Imp. S.-Peterburgsk. Bot. Sada 6(2): 388. 1880, nom. inval. syn. sec. Linczevski (1952)

***Goniolimon tataricum* subsp. *croaticum* Buzurović & Bogdanović in Pl. Syst. Evol. 306(2, 29): 17. 2020.** Sec. Buzurović & Bogdanović (2020)

***Goniolimon tataricum* subsp. *graecum* Buzurović in Pl. Syst. Evol. 306(2, 29): 17. 2020.** Sec. Buzurović & Bogdanović (2020)

***Goniolimon tataricum* subsp. *italicum* (Tammaro, Pignatti & Frizzi) Buzurović in Pl. Syst. Evol. 306(2, 29): 18. 2020.** Sec. Buzurović & Bogdanović (2020)

≡ *Goniolimon italicum* Tammaro, Pignatti & Frizzi in Webbia 36(1): 39. 1982 syn. sec. Buzurović & Bogdanović (2020)

***Goniolimon tataricum* (L.) Boiss. subsp. *tataricum*.** Sec. Buzurović & Bogdanović (2020)

***Goniolimon tataricum* var. *platypterum* (Klokov) Tzvelev, Konspekt Fl. Kavkaza 3(2): 279. 2012.** Sec. Malekmohammadi & al. (2024)

≡ *Goniolimon platypterum* Klokov, Fl. URSR 8: 522. 1958 syn. sec. POWO (2017+)

***Goniolimon tauricum* Klokov, Fl. RSS Ucr. 8: 521. 1958.** Sec. Pignatti (1972)

≡ *Goniolimon tataricum* var. *tauricum* (Klokov) Tzvelev, Fl. Vostoch. Evropy 9: 161. 1996 syn. sec. Tzvelev (2012)
= ?*Goniolimon glaberrimum* Klokov, Fl. URSR 8: 523. 1958 syn. sec. Tzvelev (2012)

Limoniastrum Heist. ex Fabr., Enum. Meth. Pl. Hort. Helmstad.: 25. 1759. Sec. Koutroumpa & al. (2018)

Type (statement): *Limoniodes monopetalum* Kuntze.
≡ *Limoniodes* Siegesb. ex Kuntze, Revis. Gen. Pl. 2: 394. 1891, nom. superfl. syn. sec. Ferrer-Gallego, P. P. & al. (2014). Type (statement): Limoniodes monopetalum Kuntze.
= *Bubania* Girard in Mém. Sect. Sci. Acad. Sci. Montpellier, 1: 182. 1848, nom. superfl. syn. sec. Crespo & Lledó (2000). Type: *Bubania monopetala* (L.) Girard

Notes. – Two perennial species with alternate leaves, densely covered with chalk depositions, and closed sheath; inner bract smooth; and stamens adnate to the corolla throat. Distributed in coastal areas of the Mediterranean region and subdesert areas of northern Africa (Crespo and Lledó 2000). In phylogenetic trees this genus forms a monophyletic clade that is sister to *Ceratolimon* (Crespo and Lledó et al. 2000; Koutroumpa et al. 2018).

***Limoniastrum guyonianum* Durieu ex Boiss. in Candolle, Prodr. 12: 689. 1848.** Sec. Lledó & al. (2000)

≡ *Limoniodes guyonianum* Siegesb. ex Kuntze, Revis. Gen. Pl. 2: 394. 1891, nom. illeg. syn. sec. Ferrer-Gallego, P. P. & al. (2014)
= *Limoniastrum ouarglense* Pomel, Nouv. Mat. Fl. Atl.: 126. 1874 syn. sec. Crespo & Lledó (2000)

***Limoniastrum monopetalum* (L.) Boiss., Candolle, Prodr. 12. 1848: 689.** Sec. Lledó & al. (2000)

≡ *Statice monopetala* L., Sp. Pl.: 276. 1753 syn. sec. Crespo & Lledó (2000) ≡ *Limonium monopetalum* (L.) Hill, Hort. Kew.: 183. 1768 syn. sec. Crespo & Lledó (2000) ≡ *Taxanthema monopetala* (L.) Sweet, Hort. Brit.: 333. 1826 syn. sec. Crespo & Lledó (2000) ≡ *Bubania monopetala* (L.) Girard in Mem. Acad. Montp. (Sect. Sc.) 1: 187. 1848 syn. sec. Crespo & Lledó (2000) ≡ *Limoniodes monopetalum* Kuntze, Revis. Gen. Pl. 2. 1891, nom. illeg. syn. sec. Crespo & Lledó (2000)
= *Limonium siculum* Mill., Gard. Dict., ed. 8: no. 7. 1768 syn. sec. Malekmohammadi & al. (2024)
= *Limoniastrum articulatum* Moench, Methodus: 423. 1794, nom. illeg. syn. sec. Malekmohammadi & al. (2024)
= *Statice scabra* Pers., Syn. Pl. 1: 333. 1805 syn. sec. POWO (2017+: 14 April 2022)
= *Statice denudata* Regel & Körn., Index Seminum 1857: 37. 1858 syn. sec. Malekmohammadi & al. (2024)
= *Limoniastrum majus* Lanza in Lav. Reale Ist. Bot. Palermo 3: 51. 1932 syn. sec. Crespo & Lledó (2000)
= *Limoniastrum multiflorum* C.Bonhomme & P.Fourn. in Monde Pl. 38: 4, 13. 1937 syn. sec. Malekmohammadi & al. (2024: acc. 11 jan 2021) ≡ *Limoniastrum monopetalum* subsp. *multiflorum* Bonhomme & P.Fourn. in Monde Pl. Rev. 38: 3. 1937 syn. sec. Domina (2011+)

Limoniopsis Lincz. in Schischkin & Bobrov, Flora URSS 18: 744. 1952. Sec. Kubitzki (1993)

Type: *Limoniopsis owerinii* (Boiss.) Lincz.

Notes. – Two perennial species with fleshy basal rosette leaves. Distributed in the Caucasus and Eastern Turkey (Kubitzki 1993). None of the species of this genus have been sampled in molecular studies.

***Limoniopsis davisii* Bokhari in 30: 303. 1970.** Sec. Bokhari & Edmondson (1982)

***Limoniopsis owerinii* (Boiss.) Lincz. in Schischkin & Bobrov, Flora URSS 18: 377. 1952.** Sec. Bokhari & Edmondson (1982)

≡ *Statice owerinii* Boiss., Fl. Orient. 4(2): 870. 1879 syn. sec. Linczevski (1952) ≡ *Limonium owerinii* (Boiss.) Kuntze, Revis. Gen. Pl. 2: 396. 1891 syn. sec. Linczevski (1952)

Limonium Mill., Gard. Dict. Abr., ed. 4, 2: [s. p.]. 1754, nom. cons. Sec. Malekmohammadi & al. (2017)

Type: *Limonium vulgare* Mill.
= *Plegorhiza* Molina, Sag. Stor. Nat. Chili: 164. 1782 syn. sec. Kuntze (1891). Type: *Plegorhiza guaicuru* Molina
– *Statice* L., Sp. Pl.: 274. 1753, nom. ambig. syn. sec. Kuntze (1891). Lectoype: *Statice armeria* L.
= *Taxanthema* Neck. ex R.Br., Prodr. Fl. Nov. Holland.: 426. 1810 syn. sec. Lindley (1846). Type: *Taxanthema australis* R.Br.
= *Linczevskia* Tzvelev in Takhtajan, Konspekt Fl. Kavkaza 3(2): 283. 2012 syn. sec. Malekmohammadi & al. (2024). Type: *Linczevskia sinuata* (L.) Tzvelev
= *Eurychiton* Nimmo, Cat. Pl. Bombay: Addend. 1839 syn. sec. Lindley (1846). Type: *Eurychiton adensis* Nimmo
= *Afrolimon* Lincz. in Novosti Sist. Vyssh. Rast. 16: 168. 1979 syn. sec. Malekmohammadi & al. (2017). Type: *Afrolimon peregrinum* (P.J.Bergius) Lincz.
= *Eremolimon* Lincz. in Novosti Sist. Vyssh. Rast. 22: 200. 1985 syn. sec. Akhani & al. (2013). Type: *Eremolimon sogdianum* (Ikonn.-Gal.) Lincz.
– *Limonium* Tourn. ex Mill., Gard. Dict., ed. 6,: 1771, nom. inval. syn. sec. Lindley (1846)

Notes. – *Limonium* is the largest genus of the family *Plumbaginaceae* with more than 650 species. Most of the species are halophytes and grow in coastal habitats. *Limonium* species are distributed almost worldwide and the Mediterranean basin is considered as its centre of diversity (Kubitzki 1993) with about 70 percent of all *Limonium* species being endemic there (Koutroumpa et al. 2018). Polyploidy, apomixis and frequent hybridisation are considered the main factors accounting for the taxonomic complexities in this genus (Kubitzki 1993; Lledó et al. 2005; Malekmohammadi et al. 2017; Koutroumpa et al. 2018). Koutroumpa et al. (2021) showed that geoclimatic changes (the Messinian Salinity Crisis, onset of Mediterranean climate and Plio-Pleistocene sea-level fluctuations) and apomixis are the main drivers of increased diversification rates in the "Mediterranean lineage" of *Limonium*.
The monophyly of *Limonium* with high support was first proven by Malekmohammadi et al. (2017), including *Afrolimon*, *Eremolimon* and *Linczevskia* nested in it. Inclusion of *Eremolimon* in *Limonium* was confirmed before by morphological and phylogenetic studies of Akhani et al. (2013). The nested position of *Afrolimon* into *Limonium* was first shown in the phylogenetic study by Lledó et al. (2005) and later confirmed by Malekmohammadi et al. (2017) and Koutroumpa et al. (2018).
Limonium is divided in two subgenera, *L.* subgen. *Limonium* and *L.* subgen. *Pterocladus* (Spach) H.Arnoud, that constitute the two major highly supported clades in molecular phylogenetic studies (Lledó et al. 2005; Malekmohammadi et al. 2017; Koutroumpa et al. 2018).
The *Limonium* sect. *Iranolimon* was described for species of an Irano-Turanian subclade that were classified under *L.* sect. *Sarcophylla* based on their common woody habitat (Malekmohammadi et al. 2017). Koutroumpa et al. (2018) sampled about one third of the all *Limonium* species (201 species) plus 84 species of other genera of *Plumbaginaceae* and sister family *Polygonaceae* in a phylogenetic framework. The authors described the new section *L.* sect. *Tenuiramosa* within *L.* subg. *Pterocladus* as a monotypic section (*L. anthericoides* (Schltr.) R.A.Dyer) and modified the circumscriptions of some sections: *L.* sect. *Limonium* (= *L.* sect. *Limonium* subsect. *Genuinae* sensu Boissier 1848), *L.* sect. *Sarcophylla* (Boiss.) Lincz., *L.* sect. *Nephrophyllum* Rech.f., and published new combinations for *L.* sect. *Pruinosa*, *L.* sect. *Pteroclados* subsect. *Odontolepidea* and *L.* subsect. *Nobilia* (Koutroumpa et al. 2018).
However, although several updates on the sectional and subsectional classification of *Limonium* have been made, many species are currently classified under the non-formal, yet phylogenetically well-defined, "Mediterranean lineage" until further molecular and morphological data could inform their assignment into newly described sections.

***Limonium* subg. *Limonium*.** Sec. Pignatti (1971)

Type: *Limonium vulgare* Mill.
≡ *Statice* subg. *Limonium* Spach, Hist. Nat. Vég. 10: 345. 1841 syn. sec. Malekmohammadi & al. (2024)

Limonium "Mediterranean lineage". Sec. Koutroumpa & al. (2018)

Notes. – The extensive sampling of Mediterranean endemics in Koutroumpa et al.'s (2018) study of *Plumbaginaceae* revealed a large, well-supported clade of *Limonium*, namely the "Mediterranean lineage". Nevertheless, species relationships within the lineage remained largely unresolved. Only few species of the "Mediterranean lineage" have been assigned into four morphologically well-defined sections (i.e., *Polyarthrion, Pruinosa, Siphonantha* and *Schizhymenium*; Koutroumpa et al. 2018). A sectional classification for the remaining species within this lineage is difficult, partially due to the combined effect of polyploidy, apomixis and hybridization present in many Mediterranean endemics that blur species limits and make the identification of diagnostic morphological characters very challenging. Therefore, these species are currently assigned to the phylogenetically well-defined "Mediterranean lineage" but their classification at the sectional level is pending further studies aiming at improving phylogenetic resolution and reviewing morphological diagnostic characters.

***Limonium* sect. *Polyarthrion* (Boiss.) Sauvage & Vindt in Trav. Inst. Sci. Chérifien, Sér. Bot. 4: 75. 1952.** Sec. Koutroumpa & al. (2018)

Type: *Limonium caesium* (Girard) Kuntze
≡ *Statice* sect. *Polyarthrion* Boiss. in Candolle, Prodr. 12: 667. 1848 syn. sec. Koutroumpa & al. (2018)

Notes. – The section comprises species with numerous sterile, articulate branches in the lower part of stem and large spikelets with pink corollas. Two of the species (*Limonium caesium* and *L. insigne*) are endemic to Spain and *L. ornatum* is endemic to Morocco. The monophyly of the section is strongly supported in the ITS tree of Koutroumpa et al. (2018).

***Limonium caesium* (Girard) Kuntze, Revis. Gen. Pl. 2: 395. 1891.** Sec. Pignatti (1972)

≡ *Statice caesia* Girard in Ann. Sci. Nat., Bot., sér. 3, 2: 325. 1844 syn. sec. Kuntze (1891)
= *Statice elegans* Coss. ex Nyman, Consp. Fl. Eur. 3: 613. 1881 syn. sec. Domina (2011+) [is replaced synonym for *Statice caesia* var. *major* Rouy] ≡ *Statice caesia* var. *major* Rouy in Rev. Sci. Nat. (Montpellier), sér. 3, 3: 63. 1883 syn. sec. Malekmohammadi & al. (2024)
− *Statice pruinosa* Cav. ex Willk. & Lange, Prodr. Fl. Hispan. 2(2): 372. 1868, nom. nud. syn. sec. Boissier (1848)

***Limonium insigne* (Coss.) Kuntze, Revis. Gen. Pl. 2: 395. 1891.** Sec. Domina (2011+)

≡ *Statice insignis* Coss., Notes Pl. Crit.: 177. 1852 syn. sec. Kuntze (1891)
= *Limonium insigne* subsp. *carthaginiense* Pignatti, Collect. Bot. (Barcelona) 6(6): 295. 1962 syn. sec. Domina (2011+)
− *Limonium insigne* subsp. *carthaginensis* Pignatti, orth. var. syn. sec. Malekmohammadi & al. (2024) [is misspelling for *Limonium insigne* subsp. *carthaginiense* Pignatti]

***Limonium insigne* subsp. *insigne*.** Sec. Malekmohammadi & al. (2024)

***Limonium insigne* var. *insigne*.** Sec. Pignatti (1962)

***Limonium insigne* var. *paui* Pignatti, Collect. Bot. (Barcelona) 6(6): 295. 1962.** Sec. Pignatti (1962)

− *Statice paui* Vicioso, nom. nud. syn. sec. Pignatti (1962)

***Limonium ornatum* (Ball) Kuntze, Revis. Gen. Pl. 2: 396. 1891.** Sec. Domina (2011+)

≡ *Statice ornata* Ball in J. Linn. Soc., Bot. 16: 559. 1878 syn. sec. Kuntze (1891)
= *Statice laeta* Ball in J. Bot. 13: 176. 1875 syn. sec. Maire (1934)

***Limonium ×rossmaessleri* (Willk.) M.B.Crespo, Phytokeys: 2024.** Sec. Malekmohammadi & al. (2024)

≡ *Statice insigne* var. *rossmaessleri* Willk. in Linnaea 30: 123. 1859 syn. sec. Malekmohammadi & al. (2024) ≡ *Limonium insigne* var. *rossmaessleri* (Willk.) Pignatti, Collect. Bot. (Barcelona) 6(6): 295. 1962 syn. sec. Malekmohammadi & al. (2024)

Notes. – This name applies to the hybrid *L. insigne* × *L. caesium*, sec. Erben (1993).

Limonium sect. *Pruinosa* (Batt.) Koutr. in Ecol. Evol. 8(24): 12420. 2018 [as " *Pruinosum* "]. Sec. Koutroumpa & al. (2018)

Type: *Limonium pruinosum* (L.) Chaz.
≡ *Statice* subsect. *Pruinosae* Batt., Fl. Algérie 1: 727. 1888 syn. sec. Koutroumpa & al. (2018)
– *Statice* subsect. *Hyalolepideae* Boiss. in Candolle, Prodr. 12: 659. 1848 syn. sec. Malekmohammadi & al. (2024)
≡ *Limonium* subsect. *Pruinosa* (Batt.) Sauvage & Vindt in Trav. Inst. Sci. Chérifien, Sér. Bot. 4: 58. 1952 syn. sec. Koutroumpa & al. (2018)
≡ *Limonium* sect. *Pruinosum* (Batt.) Koutr., orth. var. syn. sec. Malekmohammadi & al. (2024)

Notes. – This section is characterized by stems and branches covered by calcariferous tubercles with a punctuate depression in the centre, numerous sterile branches, one-flowered spikelets, calyces with membranous limbs, and deciduous leaves. Its representatives occur in North Africa, with *L. tuberculatum* and *L. pruinosum* extending their distributions into Macaronesia and Saharo-Arabia, respectively. The section was recovered as monophyletic in Koutroumpa et al.'s (2018) phylogenetic study.

***Limonium alleizettei* (Pau) Brullo in Mitt. Bot. Staatssamml. München 28: 493. 1989.** Sec. Domina (2011+)

≡ *Statice alleizettei* Pau in Cavanillesia 2: 92. 1929 syn. sec. Domina (2011+) ≡ *Limonium pruinosum* subsp. *alleizettei* (Pau) Maire in Bull. Soc. Hist. Nat. Afrique N. 22: 304. 1931 syn. sec. Domina (2011+)
− *Limonium alleizettii* Balls in Gard. Chron. ser. 3, 101: 72. 1937, nom. inval. syn. sec. Malekmohammadi & al. (2024)

***Limonium fesianum* Erben in Sendtnera 7: 77. 2001.** Sec. Domina (2011+)

***Limonium oudayense* Sauvage & Vindt in Compt.-Rend. Séances Soc. Sci. Nat. Maroc 20: 75. 1954.** Sec. Domina (2011+)

***Limonium pruinosum* (L.) Chaz. in Miller, Suppl. Dict. Jard. 2: 36. 1790.** Sec. Domina (2011+)

≡ *Statice pruinosa* L., Mant. Pl.: 59. 1767 syn. sec. Malekmohammadi & al. (2024) − *Limonium pruinosum* (L.) Kuntze, Revis. Gen. Pl. 2: 396. 1891 syn. sec. Del Guacchio & al. (2018) [is later isonym of *Limonium pruinosum* (L.) Chaz.]
= *Statice aphylla* Forssk., Fl. Aegypt.-Arab.: 60. 1775 syn. sec. Boissier (1848)
= *Statice tubiflora* Sieber ex Schult. in Roemer & Schultes, Syst. Veg. ed. 15bis 6: 798. 1820 syn. sec. Boissier (1848)
= *Statice pruinosa* var. *hirtiflora* Cavara & Grande in Bull. Orto Bot. Univ. Napoli 9 (1): 49. 1928 syn. sec. Cuccuini & al. (2016) ≡ *Limonium pruinosum* var. *hirtiflorum* (Cavara) Täckh. ex Feinbrun, Fl. Palaest. 3: 11. 1978 syn. sec. Malekmohammadi & al. (2024)
= *Limonium pruinosum* var. *glabrum* Maire & Weiller in Bull. Soc. Hist. Nat. Afrique N. 30: 291. 1939 syn. sec. Malekmohammadi & al. (2024)

Notes. – *Statice aphylla* Forssk. and *Statice tubiflora* Sieber ex Schult. are synonyms of *Statice pruinosa* L. according to Boissier (Prod. 12: 662. 1848).

***Limonium thymoides* (Girard) M.B.Crespo, Phytokeys: 2024.** Sec. Domina (2011+)

≡ *Statice thymoides* Girard in Mém. Sect. Sci. Acad. Sci. Montpellier, 1: 189. 1848 syn. sec. Malekmohammadi & al. (2024)
= *Statice asparagoides* Coss. & Durieu ex Batt., Fl. Algérie [1](4): 727. 1890 syn. sec. Maire (1934) ≡ *Limonium asparagoides* (Coss. & Durieu ex Batt.) Maire in Bull. Soc. Hist. Nat. Afrique N. 22: 55. 1931 syn. sec. Domina (2011+)

***Limonium tuberculatum* (Boiss.) Kuntze, Revis. Gen. Pl. 2: 396. 1891.** Sec. Domina (2011+)

≡ *Statice tuberculata* Boiss. in Candolle, Prodr. 12: 662. 1848 syn. sec. Kuntze (1891)
− *Statice manricarum* Bolle in Bot. Jahrb. Syst. 14: 250. 1892, pro syn. syn. sec. Domina (2011+)
− *Statice manricarum* Bolle ex Linding. in Abh. Auslandsk., Reihe C, Naturwiss. 8(2): 254. 1926, pro syn. syn. sec. Malekmohammadi & al. (2024) [is orthographic variant for *Statice manriqueorum* Bolle ex Linding.]
− *Statice manricarum* Bolle ex Pitard & Proust, orth. var. syn. sec. Malekmohammadi & al. (2024) [is orthographic variant for *Statice manriquorum* Bolle ex Pitard & Proust]
− *Statice manriquorum* Bolle ex Pitard & Proust, Iles Canaries: 320. 1908, nom. nud. syn. sec. Domina (2011+)
− *Statice manriqueorum* Bolle ex Linding. in Abh. Auslandsk., Reihe C, Naturwiss. 8(2): 254. 1926, nom. inval. syn. sec. G.Kunkel & Sunding (1967)

Limonium sect. *Schizhymenium* (Boiss.) Sauvage & Vindt in Trav. Inst. Sci. Chérifien, Sér. Bot. 4: 73. 1952. Sec. Malekmohammadi & al. (2017)

Type: *Limonium echioides* (L.) Mill.
≡ *Statice* sect. *Schizhymenium* Boiss. in Candolle, Prodr. 12: 665. 1848 syn. sec. Malekmohammadi & al. (2017)

Notes. – The section encompasses annual herbs bearing characteristic subtubular calyces with limbs lacerating in maturity and ribs forming hooked barbs (Bokhari 1972). Both species of the section are widespread in the Mediterranean and were sampled in the phylogeny of Malekmohammadi et al. (2017) forming a well-supported monophyletic group.

***Limonium avei* (De Not.) Brullo in Willdenowia 17: 17. 1988.** Sec. Malekmohammadi & al. (2024)

≡ *Statice avei* De Not., Prosp. Fl. Ligust.: 54. 1846 syn. sec. Brullo (1988)
= *Statice echioides* subsp. *exaristata* Murb. in Acta Univ. Lund. 35: 1. 1899 syn. sec. Brullo (1988) ≡ *Limonium echioides* subsp. *exaristatum* (Murb.) Maire in Jahandiez & al., Cat. Pl. Maroc 3: 571. 1934 syn. sec. Domina (2011+)
= *Statice exaristata* Murb., Contr. Fl. Nord-Ouest Afrique ser. 1, 3: 1. 1899 syn. sec. Malekmohammadi & al. (2024) ≡ *Statice echioides* subsp. *exaristata* (Murb.) Pamp., Pl. Tripol.: 188. 1914 syn. sec. Malekmohammadi & al. (2024) ≡ *Statice echioides* subsp. *exaristata* (Murb.) Hayek, Prodr. Fl. Penins. Balcan 1: 10. 1928 syn. sec. Malekmohammadi & al. (2024) ≡ *Limonium exaristatum* (Murb.) P.Fourn., Quatre Fl. France: 720. 1937 syn. sec. Brullo (1988)
= *Limonium longispicatum* Erben in Mitt. Bot. Staatssamml. München 14: 555. 1978 syn. sec. Brullo (1988)

***Limonium echioides* (L.) Mill., Gard. Dict., ed. 8: no. 11. 1768 [as " *echoideum* "].** Sec. Brullo (1988)

≡ *Statice echioides* L., Sp. Pl.: 275. 1753 syn. sec. Iamonico (2024) ≡ *Statice aspera* Lam., Fl. Franç. 3: 64. 1779 syn. sec. Iamonico (2024) ≡ *Statice sibirica* Dum.Cours. in Bot. Cult. 1: 661. 1802 syn. sec. Iamonico (2024)
≡ *Taxanthema echioides* (L.) Sweet, Hort. Brit.: 333. 1826 syn. sec. Iamonico (2024)
≡ *Statice echioides* L. subsp. *echioides* syn. sec. Domina (2011+)
− *Limonium echoideum* (L.) Mill., Gard. Dict., ed. 8: no. 11. 1768, orth. var. syn. sec. Brullo (1988)
= *Statice aristata* Sm., Fl. Graec. Prodr. 1(1): 213. 1806 syn. sec. Iamonico (2024)
= *Statice echioides* var. *segobricensis* Pau, Not. Bot. Fl. Españ 1: 23. 1887 syn. sec. Erben (1993)
− *Limonium echioides* subsp. *eu-echioides* Maire in Jahandiez & al., Cat. Pl. Maroc 3: 571. 1934, nom. inval. syn. sec. Malekmohammadi & al. (2024)

Limonium sect. *Siphonantha* (Boiss.) Sauvage & Vindt in Trav. Inst. Sci. Chérifien, Sér. Bot. 4: 76. 1952. Sec. Koutroumpa & al. (2018)

Type: *Limonium tubiflorum* (Delile) Kuntze
≡ *Statice* sect. *Siphonantha* Boiss. in Candolle, Prodr. 12: 668. 1848 syn. sec. Koutroumpa & al. (2018)

Notes. – Section characterized by densely branched stems, scorpioid-corymbiform inflorescences formed by flowers bearing large corollas with apically rounded corolla lobes, and membranous calyx limbs deeply divided into five lobes ending with an awn (Boissier 1848; Boulos 2000). This morphologically distinct section occurs in North Africa and one representative (*Limonium tubiflorum*) was sampled in a phylogenetic framework and recovered sister to representatives of *L.* sect. *Pruinosa* (Koutroumpa et al. 2018).

***Limonium asperrimum* Maire in Bull. Soc. Hist. Nat. Afrique N. 29: 434. 1938.** Sec. Domina (2011+)

***Limonium maroccanum* (Batt. & Trab.) Domina in Willdenowia 41(1): 131. 2011.** Sec. Domina (2011+)

≡ *Statice tubiflora* var. *maroccana* Batt. & Trab. in Bull. Soc. Hist. Nat. Afrique N. 9: 15. 1918 syn. sec. Domina (2011+) ≡ *Limonium tubiflorum* subsp. *maroccanum* (Batt. & Trab.) Maire & Weiller in Bull. Soc. Hist. Nat. Afrique N. 27: 244. 1936 syn. sec. Domina (2011+)

***Limonium tubiflorum* (Delile) Kuntze, Revis. Gen. Pl. 2: 396. 1891.** Sec. Malekmohammadi & al. (2024)

≡ *Statice tubiflora* Delile, Descr. Egypte, Hist. Nat.: 215. 1814 syn. sec. Kuntze (1891)
= *Statice squamata* Poir., Encycl. Suppl. 5: 237. 1817 syn. sec. Boissier (1848)

***Limonium zanonii* (Pamp.) Domina in Willdenowia 41(1): 131. 2011.** Sec. Domina (2011+)

≡ *Statice tubiflora* var. *zanonii* Pamp. in Nuovo Giorn. Bot. Ital., n.s., 24: 148. 1917 syn. sec. Domina (2011+) ≡ *Limonium tubiflorum* var. *zanonii* (Pamp.) Maire in Bull. Soc. Hist. Nat. Afrique N. 29: 435. 1938 syn. sec. Malekmohammadi & al. (2024) ≡ *Limonium tubiflorum* subsp. *zanonii* (Pamp.) Brullo in Webbia 33: 142. 1978 syn. sec. Domina (2011+)

***Limonium ×abnorme* (Rouy) P.Fourn., Quatre Fl. France: 722. 1937.** Sec. Fournier (1937)

≡ *Statice ×abnormis* Rouy in Rev. Bot. Syst. Geogr. Bot. 1(12): 182. 1904 syn. sec. Fournier (1937)

***Limonium acuminatum* L.Bolus in S. African Gard. 24: 124, 129. 1934.** Sec. Malekmohammadi & al. (2024)

***Limonium acutifolium* (Badarò ex Rchb.) C.E.Salmon in J. Bot. 62. 1924: 336.** Sec. Peruzzi & al. (2015)

≡ *Statice acutifolia* Badarò ex Rchb., Iconogr. Bot. Pl. Crit. 3(1): 23. 1825 syn. sec. C.E.Salmon in J. Bot. 62. 1924 (1924) ≡ *Limonium minutum* subsp. *acutifolium* (Rchb.) P.Fourn., Quatre Fl. France: 721. 1937 syn. sec. Malekmohammadi & al. (2024)
= *Statice minuta* Moritzi ex Rchb., Iconogr. Bot. Pl. Crit. 3: 23. 1825 syn. sec. Malekmohammadi & al. (2024)
= *Statice rupicola* Badarò ex Rchb., Fl. Germ. Excurs. 1: 191. 1831 syn. sec. Malekmohammadi & al. (2024) ≡ *Limonium rupicola* (Badarò ex Rchb.) Kuntze, Revis. Gen. Pl. 2: 396. 1891 syn. sec. Malekmohammadi & al. (2024)
= *Statice minuta* var. *acutifolia* Boiss. in Candolle, Prodr. 12: 655. 1848 syn. sec. Malekmohammadi & al. (2024)
= *Statice virgata* var. *pumila* Boiss. in Candolle, Prodr. 12: 654. 1848 syn. sec. Malekmohammadi & al. (2024)

Notes. – The combination *Statice minuta* var. *acutifolia* (Rchb.) Boissier was decribed by Boissier (Prod. 12: 655 & 673. 1848) with *Statice rupicula* Badarò and *Statice acutifolia* Rchb. considered as its synonys. [Boissier (1848)]

***Limonium acutifolium* subsp. *acutifolium*.** Sec. Malekmohammadi & al. (2024)

***Limonium acutifolium* (Badarò ex Rchb.) C.E.Salmon subsp. *acutifolium*.** Sec. Peruzzi & al. (2015)

***Limonium admirabile* Terrones, J.Moreno, M.Á.Alonso, Juan & M.B.Crespo in Phytotaxa 333 (1): 45. 2018.** Sec. Moreno & al. (2018)

***Limonium aegaeum* Erben & Brullo in Phytotaxa 240: 44. 2016.** Sec. Domina (2011+)

***Limonium aegusae* Brullo in Bot. Not. 133(3): 291. 1980.** Sec. Peruzzi & al. (2015)

***Limonium afrum* (Pignatti) Domina in Willdenowia 41(1): 131. 2011.** Sec. Domina (2011+)

≡ *Limonium delicatulum* subsp. *afrum* Pignatti, Collect. Bot. (Barcelona) 6(6): 308. 1962 syn. sec. Domina (2011+)

***Limonium albarracinense* Pau ex Ferrer-Gallego, P. P. & R.Roselló in Novon 26(1): 29. 2018.** Sec. Malekmohammadi & al. (2024)

***Limonium albidum* (Guss.) Pignatti in Bot. J. Linn. Soc. 64(4): 365. 1971.** Sec. Peruzzi & al. (2015)

≡ *Statice albida* Guss., Fl. Sicul. Syn. 1: 369. 1843 syn. sec. Pignatti (1971) ≡ *Statice psiloclada* var. *albida* (Guss.) Boiss. in Candolle, Prodr. 12: 651. 1848 syn. sec. Malekmohammadi & al. (2024: revision, 2022);

Notes. – Listed as a species in the *Limonium albidum* group by Pignatti (1972).

***Limonium albomarginatum* Brullo in Willdenowia 17: 15. 1988.** Sec. Domina (2011+)

***Limonium ×albuferae* Ferrer-Gallego, P. P., R.Roselló, M.Rosato, Rosselló & E.Laguna in Phytotaxa 252(2): 115. 2016.** Sec. Malekmohammadi & al. (2024)

Notes. – This taxon is here regarded as a hybrid of *L. angustebracteatum* × *L. girardianum*.

***Limonium album* (Coincy) Sennen, Diagn. Nouv.: 72. 1936.** Sec. Domina (2011+)

≡ *Statice alba* Coincy in J. Bot. (Morot) 9: 334. 1895 syn. sec. Domina (2011+)

***Limonium alcudianum* Erben in Mitt. Bot. Staatssamml. München 28: 313. 1989.** Sec. Domina (2011+)

***Limonium algarvense* Erben in Mitt. Bot. Staatssamml. München 14: 503. 1978.** Sec. Domina (2011+)

***Limonium algusae* (Brullo) Greuter in Willdenowia 16(2): 449. 1987.** Sec. Domina (2011+)

≡ *Limonium oleifolium* subsp. *algusae* Brullo in Bot. Not. 133(3): 289. 1980 syn. sec. Domina (2011+)

***Limonium alicunense* Gómiz in Anales Jard. Bot. Madrid 53: 255. 1996.** Sec. Malekmohammadi & al. (2024)

***Limonium altum* P.D.Sell, Fl. Gr. Brit. Ireland 1: 689. 2018.** Sec. Sell & Murrell (2018)

***Limonium alutaceum* (Steven) Kuntze, Revis. Gen. Pl. 2: 395. 1891.** Sec. Domina (2011+)

≡ *Statice alutacea* Steven in Bull. Soc. Imp. Naturalistes Moscou 30(1): 367. 1857 syn. sec. Domina (2011+) ≡ *Limonium tomentellum* subsp. *alutaceum* (Steven) Moysiyenko, Ecofl. Ukrayini 6: 9. 2010 syn. sec. Didukh Ya. & al. (2010)
= *Statice oblongifolia* Kotov in Trudy Silsko-Gosp. Bot. I 3: 165. 1927 syn. sec. Didukh Ya. & al. (2010) ≡ *Limonium oblongifolium* (Kotov) Loscot & Trautv., nom. illeg. syn. sec. Didukh Ya. & al. (2010)

***Limonium ×ambiguum* (Rouy) P.Fourn., Quatre Fl. France: 722. 1937.** Sec. Malekmohammadi & al. (2024)

≡ *Statice ×ambigua* Rouy in Rev. Bot. Syst. Geogr. Bot. 1(12): 181. 1904 syn. sec. Malekmohammadi & al. (2024)

***Limonium ammochostianum* Erben, Christodoulou, Hand & Kefalas in Fl. Medit. 32: 37. 2022.** Sec. Erben & al. (2022)

– *Limonium ocymifolium* subsp. *bellidifolium* sensu auct. cypr., non (Sm.) Meikle, err. sec. Erben & al. (2022)

***Limonium ammophilon* (Papatsou & Phitos) Domina in Willdenowia 41(1): 131. 2011.** Sec. Dimopoulos & al. (2016)

≡ *Limonium graecum* subsp. *ammophilon* Papatsou & Phitos in 34: 203. 1975 syn. sec. Domina (2011+)
= *Limonium rhodense* M.B.Crespo & Pena-Martín in Phytotaxa 94(2): 35. 2013 syn. sec. Koutroumpa & al. (2018)

***Limonium amopicum* Erben & Brullo in Phytotaxa 240: 67. 2016.** Sec. Domina (2011+)

***Limonium ampuriense* Arrigoni & Diana in Boll. Soc. Sarda Sci. Nat. 25: 165. 1986.** Sec. Peruzzi & al. (2015)

***Limonium amynclaeum* Pignatti in Webbia 36(1): 49. 1982.** Sec. Pignatti (1982)

= *Limonium pontium* subsp. *terracinense* Iberite, Iamonico, De Castro, Nicolella Iamonico, De Castro & Nicolella in Plants 11, 3163: 16. 2022 syn. sec. Malekmohammadi & al. (2024: 23 Nov. 2022)

***Limonium anglicum* (Ingr.) P.D.Sell, Fl. Gr. Brit. Ireland 1: 688. 2018.** Sec. Sell & Murrell (2018)

≡ *Limonium binervosum* subsp. *anglicum* Ingr. in Bot. J. Linn. Soc. 92(3): 188. Apr 1986 syn. sec. Sell & Murrell (2018)

***Limonium angustibracteatum* Erben in Mitt. Bot. Staatssamml. München 14: 512. 1978.** Sec. Domina (2011+)

≡ *Limonium delicatulum* subsp. *angustibracteatum* (Erben) Rivas Mart. & M.J.Costa, Stud. Bot. (Salamanca) 3: 15. 1984 syn. sec. Domina (2011+) − *Limonium angustebracteatum* Erben, orth. var. syn. sec. Malekmohammadi & al. (2024) − *Limonium delicatulum* subsp. *angustebracteatum* (Erben) Rivas Mart. & M.J.Costa, orth. var. syn. sec. Malekmohammadi & al. (2024)
= *Limonium delicatulum* subsp. *valentinum* Pignatti, Collect. Bot. (Barcelona) 6(6): 306. 1962 syn. sec. Erben (1978)

***Limonium antipaxorum* R.Artelari, Biosyst. Meleti Genous Limonium: 32. 1984; Bot. Chronika 4(1–2): 22. 1984.** Sec. Domina (2011+)

***Limonium antonii-llorensii* L.Llorens in Lazaroa 8: 31. 1986.** Sec. Domina (2011+)

***Limonium aphroditae* R.Artelari & Georgiou in Bot. J. Linn. Soc. 131(4): 401. 1999.** Sec. Domina (2011+)

***Limonium apulum* Brullo in Giornale Botanico Italiano 124 (1): 75. 1990.** Sec. Peruzzi & al. (2015)

***Limonium aragonense* (Debeaux ex Willk.) Font Quer in Collect. Bot. (Barcelona) 1: 300. 1947.** Sec. Ferrer-Gallego, P. P. & al. (2018)

≡ *Statice aragonensis* Debeaux ex Willk., Suppl. Prodr. Fl. Hispan.: 326. 1893 syn. sec. Ferrer-Gallego, P. P. & al. (2018) ≡ *Limonium aragonense* (Debeaux ex Willk.) Pignatti, Collect. Bot. (Barcelona) 6(6): 301. 1962 syn. sec. Ferrer-Gallego, P. P. & al. (2018) − *Limonium minutum* subsp. *aragonense* De Litard., nom. inval. syn. sec. Ferrer-Gallego, P. P. & al. (2018)
= *Statice monregalensis* Pau, Notas Bot. Fl. Esp.: 89. 1895 syn. sec. Pignatti (1962)
= *Statice monrealensis* Pau in Bol. Soc. Aragonesa Ci. Nat. 9: 236. 1910 syn. sec. Pignatti (1962)
= *Limonium stephanii* Sennen, Diagn. Nouv.: 262. 1936 syn. sec. Erben (1993) ≡ *Statice stephani* Sennen, Diagn. Nouv.: 262. 1936 syn. sec. Sennen (1936)

***Limonium archaeothirae* Erben & Brullo in Phytotaxa 240: 165. 2016.** Sec. Domina (2011+)

− *Limonium archeothirae* Erben & Brullo, orth. var. syn. sec. Domina (2011+) [is misspelling for *Limonium archaeothirae* Erben & Brullo]

***Limonium arcuatum* R.Artelari, Biosyst. Meleti Genous Limonium: 27. 1984.** Sec. Domina (2011+)

***Limonium arenosum* Erben in Mitt. Bot. Staatssamml. München 14: 550. 1978.** Sec. Domina (2011+)

***Limonium artelariae* Koutr. in Willdenowia 54: 71. 2024.** Sec. Koutroumpa (2024)

***Limonium articulatum* (Loisel.) Kuntze, Revis. Gen. Pl. 2: 395. 1891.** Sec. Domina (2011+)

≡ *Statice articulata* Loisel., Fl. Gall., ed. 2, 1: 225. t. 6. 1828 syn. sec. Domina (2011+)

***Limonium artruchium* Erben in Mitt. Bot. Staatssamml. München 28: 361. 1989.** Sec. Domina (2011+)

***Limonium astypaleanum* Erben & Brullo in Phytotaxa 240: 168. 2016.** Sec. Domina (2011+)

− *Limonium astipaleanum* Erben & Brullo, orth. var. syn. sec. Domina (2011+) [is misspelling for *Limonium astypaleanum* Erben & Brullo]

***Limonium athinense* Erben & Brullo in Phytotaxa 240: 56. 2016.** Sec. Domina (2011+)

***Limonium atticum* Erben & Brullo in Phytotaxa 240: 152. 2016.** Sec. Domina (2011+)

***Limonium aucheri* (Girard) Greuter & Raus in Willdenowia 19(1): 39. 1989.** Sec. Domina (2011+)

≡ *Statice aucheri* Girard in Ann. Sci. Nat., Bot., sér. 3, 2: 328. 1844 syn. sec. Domina (2011+)
= *Statice bellidifolia* Sm., Fl. Graec. Prodr. 1(1): 211. 1806 syn. sec. Domina (2011+) ≡ *Statice ocymifolia* var. *bellidifolia* (Sibth. & Sm.) Boiss., Fl. Orient. 4(2): 861. 1879 syn. sec. Malekmohammadi & al. (2024) ≡ *Limonium ocymifolium* var. *bellidifolium* (Sm.) Rech.f. in Denkschr. Akad. Wiss. Wien, Math.-Naturwiss. Kl. 105(1): 427. 1943 syn. sec. Malekmohammadi & al. (2024) ≡ *Limonium ocymifolium* subsp. *bellidifolium* (Sm.) Meikle, Fl. Cyprus: 1070. 1985 syn. sec. Domina (2011+)
= *Statice bellidifolia* Auct. ex Boiss. in Candolle, Prodr. 12: 648. 1848 syn. sec. Malekmohammadi & al. (2024)

***Limonium auriculae-ursifolium* (Pourr.) Druce, List Brit. Pl., ed. 2: 77. 1928.** Sec. Del Guacchio & al. (2019)

≡ *Statice auriculae-ursifolia* Pourr. in Mém. Acad. Sci. Toulouse 3: 330. 1788 syn. sec. Malekmohammadi & al. (2024)
= *Statice willdenowii* Loisel., Fl. Gall.: 224. 1806 syn. sec. Del Guacchio & al. (2019)
= *Statice willdenovii* Poir., Encycl. Suppl. 5: 236. 1817, nom. illeg. syn. sec. Del Guacchio & al. (2019) ≡ *Limonium willdenowii* (Poir.) P.Fourn., Quatre Fl. France: 721. 1937 syn. sec. Del Guacchio & al. (2019)
= *Statice lychnidifolia* Girard in Ann. Sci. Nat., Bot., sér. 2 17: 18, t. 3. 1842 syn. sec. Del Guacchio & al. (2019) ≡ *Limonium lychnidifolium* (Girard) Kuntze, Revis. Gen. Pl. 2: 395. 1891, nom. illeg. syn. sec. Del Guacchio & al. (2019)
= *Limonium binervosum* subsp. *lychnidifolium* P.Fourn., Quatre Fl. France: 722. 1937 syn. sec. Del Guacchio & al. (2019)
= *Limonium girardianum* subsp. *willdenowii* P.Fourn., Quatre Fl. France: 721. 1937 syn. sec. Domina (2011+)

***Limonium auriculifolium* (Vahl) Druce in J. Linn. Soc., Bot. 35: 77. 1901.** Sec. Del Guacchio & al. (2019)

= *Statice auriculifolia* Vahl, Symb. Bot. 1: 25. 1790 syn. sec. Del Guacchio & al. (2019); ≡ *Taxanthema auriculifolia* (Vahl) Sweet, Hort. Brit.: 332. 1826 syn. sec. Malekmohammadi & al. (2024)
= *Statice auriculifolia* Brot., Fl. Lusit. 1: 489. 1804, nom. illeg. syn. sec. Del Guacchio & al. (2019)
= *Statice auriculifolia* DC., Fl. Franç. ed. 3, 3: 431. 1805, nom. illeg. syn. sec. Del Guacchio & al. (2019)
= *Statice auriculifolia* Poir., Encycl. 7: 401. 1806, nom. illeg. syn. sec. Del Guacchio & al. (2019)
= *Statice auriculifolia* Benth., Cat. Pl. Pyrénées: 123. 1826, nom. illeg. syn. sec. Del Guacchio & al. (2019)
= *Limonium nydeggeri* Erben in Sendtenera 6: 103. 1999 syn. sec. Del Guacchio & al. (2019)

***Limonium aurigniense* (Ingr.) P.D.Sell, Fl. Gr. Brit. Ireland 1: 690. 2018.** Sec. Sell & Murrell (2018)

≡ *Limonium binervosum* var. *aurigniense* Ingr. in Bot. J. Linn. Soc. 92(3): 190. Apr 1986 syn. sec. Sell & Murrell (2018)

***Limonium avenaceum* (C.H.Wright) R.A.Dyer in Bull. Misc. Inform. Kew 1932(3): 155. 1932.** Sec. Malekmohammadi & al. (2024)

≡ *Statice avenacea* C.H.Wright in Thiselton-Dyer, Flora Capensis 4(1.3): 423. 1906 syn. sec. R.A.Dyer (1961) ≡ *Limonium scabrum* var. *avenaceum* (C.H.Wright) R.A.Dyer in Bothalia 7(3): 491. 1961 syn. sec. R.A.Dyer (1961)

Notes. – This species is accepted based on the personal comnunication with Mucina.

***Limonium balearicum* (Pignatti) Brullo in Bot. Not. 133(3): 288. 1980.** Sec. Domina (2011+)

≡ *Limonium gougetianum* subsp. *balearicum* Pignatti in Arch. Bot. (Forlì) 31: 87. 1955 syn. sec. Domina (2011+) ≡ *Limonium minutiflorum* subsp. *balearicum* Pignatti in Arch. Bot. (Forlì) 31: 85. 1955 syn. sec. Brullo (1980) ≡ *Limonium girardianum* subsp. *balearicum* (Pignatti) O.Bolòs, Vigo, Masalles & Ninot, Fl. Man. Països Catalans, ed. 1: 1214. 1990 syn. sec. Domina (2011+)

***Limonium barceloi* Gil & L.Llorens in Anales Jard. Bot. Madrid 49(1): 51. 1991.** Sec. Malekmohammadi & al. (2024)

***Limonium battandieri* Greuter & Raus in Willdenowia 16(2): 449. 1987.** Sec. Greuter & Raus (1987)

= *Statice gummifera* f. *corymbulosa* (Coss.) Batt., Fl. Algérie [1](4): 730. 1890 syn. sec. Malekmohammadi & al. (2024)
= *Statice gummifera* subsp. *cymulifera* (Boiss.) Batt., Fl. Algérie [1](4): 730. 1890 syn. sec. Domina (2011+)
= *Statice alba* Pau, Font.-Quer, It. Maroc: 476. 1927 syn. sec. Maire (1934)
− *Limonium gummiferum* subsp. *battandieri* Sauvage & Vindt in Trav. Inst. Sci. Chérifien, Sér. Bot. 4: 67. 1952, nom. inval. syn. sec. Greuter & Raus (1987)
– *Limonium gummiferum* subsp. *cymuliferum* sensu Battandier (1890), err. sec. Greuter & Raus (1987)
– *Statice gummifera* subsp. *cymulifera* sensu Battandier (1890), err. sec. Greuter & Raus (1987)

***Limonium bianorii* (Sennen & Pau) Erben in Mitt. Bot. Staatssamml. München 28: 350. 1989.** Sec. Domina (2011+)

≡ *Statice bianorii* Sennen & Pau in Bull. Acad. Int. Geogr. Bot. 23: 47. 1913 syn. sec. Domina (2011+) − *Limonium duriusculum* subsp. *bianorii* (Sennen & Pau) Malag., Subesp. Variación Geogr.: 13. 1973, nom. inval. syn. sec. Malekmohammadi & al. (2024)
− *Limonium duriusculum* var. *robustior* Pignatti, nom. inval. syn. sec. Erben (1989)

***Limonium biflorum* (Pignatti) Pignatti in Bot. J. Linn. Soc. 64(4): 368. 1971.** Sec. Erben (1993)

≡ *Limonium ovalifolium* f. *biflorum* Pignatti in Arch. Bot. (Forlì) 31: 95. 1955 syn. sec. Erben (1993) ≡ *Limonium ovalifolium* subsp. *biflorum* (Pignatti) Pignatti, Collect. Bot. (Barcelona) 6(6): 319. 1962 syn. sec. Pignatti (1971) ≡ *Limonium delicatulum* subsp. *biflorum* (Pignatti) O.Bolòs, Vigo, Masalles & Ninot, Fl. Man. Països Catalans, ed. 1: 1214. 1990 syn. sec. Erben (1993)
= *Statice lychnidifolia* Porta in Nuovo Giorn. Bot. Ital. 19: 317. 1887 syn. sec. Wangerin (1921)
= *Statice ovalifolia* var. *balleariea* Wanger in Repert. Spec. Nov. Regni Veg. 17: 400. 1921 syn. sec. Pignatti (1971)

Notes. – Listed as a species in the *Limonium delicatulum* group by Pignatti (1972).

***Limonium billardierei* (Girard) Kuntze, Revis. Gen. Pl. 2: 395. 1891.** Sec. Malekmohammadi & al. (2024)

≡ *Statice billardierei* Girard in Ann. Sci. Nat., Bot., sér. 3, 2: 325. 1844 syn. sec. Kuntze (1891)
= *Statice equisetina* var. *depauperata* Boiss. in Candolle, Prodr. 12: 658. 1848 syn. sec. Malekmohammadi & al. (2024) ≡ *Limonium equisetinum* var. *depauperatum* (Boiss.) Steenis, Fl. Males., Ser. 1, Spermat. 4(2): 112. 1949 syn. sec. Malekmohammadi & al. (2024) ≡ *Limonium depauperatum* (Boiss.) R.A.Dyer in Bothalia 7(3): 490. 1961 syn. sec. Malekmohammadi & al. (2024)

Notes. – *Limonium depauperatum* and *L. billardierei* (or billardieri) are identical taxonomic concepts and the *L. billardierei* is the oldest name (personal com. with Lodislav Mucina).

***Limonium binervosum* (G.E.Sm.) C.E.Salmon in J. Bot. 45: 24. 1907.** Sec. Pignatti (1972)

≡ *Statice binervosa* G.E.Sm., Engl. Bot. Suppl. 1: t. 2663. 1830 syn. sec. C.E.Salmon (1907)
= *Statice cordata* G.E.Sm., Cat. Pl. South Kent: 18, t. 2. 1826 syn. sec. C.E.Salmon (1907)
= *Statice lanceolata* Rchb., Iconogr. Bot. Pl. Crit. 8: 7, t. 719, f. 961. 1830 syn. sec. Boissier (1848)
= *Statice spathulata* Hook., Brit. Fl. (Hooker): 145. 1830 syn. sec. Pignatti (1972)
= *Statice bubanii* Girard in Ann. Sci. Nat., Bot., sér. 3, 2: 326. 1844 syn. sec. C.E.Salmon (1907)
= *Statice occidentalis* J.Lloyd, Fl. Loire-Inf.: 212. 1844 syn. sec. C.E.Salmon (1907) ≡ *Statice binervosa* var. *occidentalis* (J.Lloyd) Syme, Engl. Bot., ed. 3B, 7: 163. 1867 syn. sec. Ingrouille, M.J. & Stace (1986) ≡ *Limonium occidentale* (J.Lloyd) Kuntze, Revis. Gen. Pl. 2: 395. 1891 syn. sec. C.E.Salmon (1907) ≡ *Limonium binervosum* subsp. *occidentale* (J.Lloyd) P.Fourn., Quatre Fl. France: 722. 1937 syn. sec. Domina (2011+)
= *Statice bayonnensis* Gren. ex Boiss. in Candolle, Prodr. 12: 649. 1848 syn. sec. C.E.Salmon (1907)

Notes. – Listed as a species in the *Limonium binervosum* aggregate (Ingrouille and Stace 1986) and *L. binervosum* group (Pignatti, 1972).

***Limonium binervosum* subsp. *binervosum*.** Sec. Malekmohammadi & al. (2024)

***Limonium binervosum* var. *humilis* C.E.Salmon in J. Bot. 45: 25. 1907.** Sec. Pignatti (1972)

= *Statice reticulata* Hook., Fl. scotica: 97. 1821 syn. sec. C.E.Salmon (1907)

***Limonium bocconei* (Lojac.) Litard., Prodr. Fl. Corse 3(2): 16. 1955.** Sec. Domina (2011+)

≡ *Statice bocconei* Lojac., Fl. Sicul., Fl. Sicul. 2(2): 25. 1907 syn. sec. Peruzzi & al. (2015)
= *Statice cordata* Guss., Fl. Sicul. Prodr. 1: 382. 1827, nom. illeg. syn. sec. Brullo & Guarino (2017)
= *Statice ambigua* Tineo ex Lojac., Fl. Sicul. 2(2): 26. 1907 syn. sec. Brullo (1980)
− *Statice cordata* var. *genuina* Boiss. in Candolle, Prodr. 12: 656. 1848, nom. inval. syn. sec. Malekmohammadi & al. (2024)

Notes. – Listed as a species in the *Limonium cosyrense* group (Pignatti, 1972).

***Limonium boirae* L.Llorens & Tébar in Anales Jard. Bot. Madrid 45: 177. 1988.** Sec. Domina (2011+)

***Limonium boitardii* Maire in Bull. Soc. Hist. Nat. Afrique N. 28: 368. 1937.** Sec. Malekmohammadi & al. (2024)

***Limonium bollei* (Webb ex Wangerin) Erben in Sendtnera 7: 84. 2001.** Sec. Domina (2011+)

≡ *Statice bollei* Webb ex Wangerin in Repert. Spec. Nov. Regni Veg. 17: 401. 1921 syn. sec. Erben (2001)
= *Limonium ovalifolium* subsp. *canariense* Pignatti, Collect. Bot. (Barcelona) 6(6): 317. 1962 syn. sec. Erben (2001)

***Limonium bolosii* Gil & L.Llorens in Anales Jard. Bot. Madrid 49(1): 54. 1991.** Sec. Malekmohammadi & al. (2024)

***Limonium bonafei* Erben in Mitt. Bot. Staatssamml. München 30: 465. 1991.** Sec. Domina (2011+)

***Limonium bonifaciense* Arrigoni & Diana in Candollea 48(2): 638. 1993.** Sec. Domina (2011+)

***Limonium bonnetii* (Sennen) Erben in Mitt. Bot. Staatssamml. München 28: 413. 1989.** Sec. Erben (1989)

− *Statice bonnetii* Sennen in Bol. Soc. Ibér. Ci. Nat. 35: 29. 1936 syn. sec. Erben (1989) [is misspelling for *Statice bonnetii* Sennen]
= *Limonium willdenowii* Pignatti in Arch. Bot. (Forlì) 31: 95. 1955 syn. sec. Erben (1989)

***Limonium bosanum* Arrigoni & Diana in Boll. Soc. Sarda Sci. Nat. 24: 281. 1985.** Sec. Peruzzi & al. (2015)

≡ *Limonium acutifolium* subsp. *bosanum* (Arrigoni & Diana) Arrigoni in Parlatorea 7: 19. 2005 syn. sec. Peruzzi & al. (2015)

***Limonium britannicum* Ingr. in Bot. J. Linn. Soc. 92(3): 199. Apr 1986.** Sec. Ingrouille, M.J. & Stace (1986)

Notes. – Listed as a species in the *Limonium binervosum* aggregate (Ingrouille and Stace 1986).

***Limonium brusnicense* (Trinajstić) Bogdanović & Brullo in Phytotaxa 215(1): 11. 2015.** Sec. Bogdanović & Brullo (2015)

≡ *Limonium vestitum* subsp. *brusnicense* Trinajstić, Suppl. Fl. Anal. Jugosl. Suppl. 7: 7. 1980 syn. sec. Bogdanović & Brullo (2015)

***Limonium brutium* Brullo in Fl. Medit. 2: 109. 1992.** Sec. Peruzzi & al. (2015)

***Limonium busianum* Bogdanović & Brullo in Phytotaxa 215(1): 11. 2015.** Sec. Bogdanović & Brullo (2015)

***Limonium byzacium* Brullo in Mitt. Bot. Staatssamml. München 28: 419. 1989.** Sec. Domina (2011+)

***Limonium calabrum* Brullo in Bot. Not. 133(3): 288. 1980.** Sec. Peruzzi & al. (2015)

***Limonium calanchicola* Erben in Sendtnera 8: 27. 2002.** Sec. Domina (2011+)

***Limonium calcarae* (Tod. ex Janka) Pignatti in Bot. J. Linn. Soc. 64(4): 364. 1971.** Sec. Peruzzi & al. (2015)

≡ *Statice dictyoclada* subsp. *calcarae* (Tod. ex Janka) Nyman, Consp. Fl. Eur. 3: 611. 1881 syn. sec. Domina (2011+) ≡ *Statice calcarae* Tod. ex Janka in Természetrajzi Füz. 6: 175. 1882 syn. sec. Pignatti (1971) ≡ *Statice minuta* var. *calcarae* (Tod. ex Janka) Fiori, Nuov. Fl. Italia 2: 232. 1926 syn. sec. Malekmohammadi & al. (2024)
= *Statice dichotoma* Guss., Fl. Sicul. Syn. 1: 372. 1843, nom. illeg. syn. sec. Malekmohammadi & al. (2024) [non *Statice dichotoma* Cav.]

***Limonium calcicola* P.D.Sell, Fl. Gr. Brit. Ireland 1: 688. 2018.** Sec. Sell & Murrell (2018)

***Limonium calliopsium* Alf.Mayer, Libri Botanici 15: 96. 1995.** Sec. Domina (2011+)

***Limonium cambrense* (Ingr.) P.D.Sell, Fl. Gr. Brit. Ireland 1: 691. 2018.** Sec. Sell & Murrell (2018)

≡ *Limonium procerum* subsp. *cambrense* Ingr. in Bot. J. Linn. Soc. 92(3): 198. Apr 1986 syn. sec. Sell & Murrell (2018)

***Limonium camposanum* Erben in Mitt. Bot. Staatssamml. München 28: 313. 1989.** Sec. Domina (2011+)

***Limonium cancellatum* (Bernh. ex Bertol.) Kuntze, Revis. Gen. Pl. 2: 395. 1891.** Sec. Bogdanović & Brullo (2015)

≡ *Statice cancellata* Bernh. ex Bertol., Fl. Ital. 3: 525. 1839 syn. sec. Bogdanović & Brullo (2015); ≡ *Statice minuta* var. *cancellata* (Bernh. ex Bertol.) Fiori, Fl. Italia 2: 332. 1902 syn. sec. Bogdanović & Brullo (2015)
= *Statice rorida* Vis., Stirp. Dalmat. Spec.: 48. 1826 syn. sec. Bogdanović & Brullo (2015)
= *Statice pubescens* W.D.J.Koch, Syn. Fl. Germ. Helv. 1(2): 595. 1837 syn. sec. Boissier (1848)
= *Statice minuta* var. *pubescens* Boiss. in Candolle, Prodr. 12: 655. 1848 syn. sec. Boissier (1848)
= *Statice tineoi* J.Woods, Tourists Fl.: 306. 1850 syn. sec. Malekmohammadi & al. (2024)
= *Statice furfuracea* Rchb., Icon. Fl. Germ. Helv. 17: 64. 1854–1855 syn. sec. Boissier (1848)
= *Statice articulata* Schimp. ex Nyman, Consp. Fl. Eur. 3: 612. 1881 syn. sec. Nyman (1881)
= *Statice minuta* Tomm. ex Nyman, Consp. Fl. Eur. 3: 612. 1881 syn. sec. Nyman (1881)
= *Statice calcarae* Tod. ex Lojac., Fl. Sicul. 2(2): 24. 1907 syn. sec. Malekmohammadi & al. (2024)
= *Statice cordata* Hayek, Prodr. Fl. Penins. Balcan 1: 9. 1928 syn. sec. Malekmohammadi & al. (2024)
− *Statice cancellata* Bernh. ex Mutel, A., Fl. Franç. 3: 88. 1836, nom. inval. syn. sec. Malekmohammadi & al. (2024)

***Limonium cantianum* (Ingr.) P.D.Sell, Fl. Gr. Brit. Ireland 1: 689. 2018.** Sec. Sell & Murrell (2018)

≡ *Limonium binervosum* subsp. *cantianum* Ingr. in Bot. J. Linn. Soc. 92(3): 188. Apr 1986 syn. sec. Sell & Murrell (2018)

***Limonium ×capdeperae* Pignatti in Arch. Bot. (Forlì) 31: 97. 1955.** Sec. Pignatti (1955)

***Limonium capitis-eliae* Erben in Sendtnera 7: 65. 2001.** Sec. Peruzzi & al. (2015)

***Limonium capitis-marci* Arrigoni & Diana in Boll. Soc. Sarda Sci. Nat. 27: 259. 1990.** Sec. Peruzzi & al. (2015)

***Limonium caprariae* Rizzotto in Webbia 53(2): 255. 1999.** Sec. Peruzzi & al. (2015)

***Limonium caprariense* (Font Quer & Marcos) Pignatti in Arch. Bot. (Forlì) 31: 77. 1955.** Sec. Pignatti (1972)

≡ *Limonium minutum* subsp. *caprariense* Font Quer & Marcos in Cavanillesia 8: 37. 1936 syn. sec. Pignatti (1972) ≡ *Limonium caprariense* subsp. *caprariense* syn. sec. Pignatti (1972)
– *Statice minuta* sensu auct., non Desf., err. sec. Domina (2011+)

***Limonium carisae* Erben in Sendtnera 7: 73. 2001.** Sec. Peruzzi & al. (2015)

***Limonium carminis* B.Díez & Erben in Flora Montiber. 82: 113. 2022.** Sec. Malekmohammadi & al. (2024)

***Limonium carpathum* (Rech.f.) Rech.f. in Denkschr. Akad. Wiss. Wien, Math.-Naturwiss. Kl. 105(1): 427. 1943.** Sec. Domina (2011+)

≡ *Statice carpatha* Rech.f. in Ann. Naturhist. Mus. Wien 47: 147. 1936 syn. sec. Rechinger (1943)

***Limonium carpetanicum* Erben in Mitt. Bot. Staatssamml. München 27: 388. 1988.** Sec. Domina (2011+)

***Limonium carregadorense* Erben in Mitt. Bot. Staatssamml. München 28: 313. 1989.** Sec. Domina (2011+)

***Limonium carthaginense* (Rouy) C.E.Hubb. & Sandwith in Bull. Misc. Inform. Kew 1928: 150. 1928.** Sec. Domina (2011+)

≡ *Statice carthaginensis* Rouy in Rev. Bot. Syst. Geogr. Bot. 1(12): 182. 1904 syn. sec. Domina (2011+)
= *Limonium calaminare* Pignatti in Bot. J. Linn. Soc. 65(4): 353. 1972 syn. sec. Domina (2011+)

Notes. – *Statice ×carthaginensis* was described as a hybrid between *Statice virgata* and *Statice pubescens* (Rouy, 1904). However, it is here regardeda a proper species, not related to any of those putative parents.

***Limonium carvalhoi* Rosselló, L.Sáez & Carvalho, A.C. in Anales Jard. Bot. Madrid 56(1): 24. 1998.** Sec. Malekmohammadi & al. (2024)

***Limonium ×castellonense* Erben in Mitt. Bot. Staatssamml. München 16(Suppl.): 552. 1980.** Sec. Malekmohammadi & al. (2024)

Notes. – This taxon is a hybrid of *L. angustebracteatum* × *L. dufourii* according to Erben (1993).

***Limonium catalaunicum* (Willk. & Costa) Pignatti, Collect. Bot. (Barcelona) 6(6): 300. 1962.** Sec. Domina (2011+)

≡ *Statice catalaunica* Willk. & Costa in Linnaea 30: 122. 1860 syn. sec. Domina (2011+) ≡ *Statice duriuscula* var. *catalaunica* Costa, Fl. Catal.: 209. 1864 syn. sec. Erben (1993) ≡ *Statice duriuscula* subsp. *catalaunica* (Willk. & Costa) Nyman, Consp. Fl. Eur. 3: 611. 1881 syn. sec. Domina (2011+) ≡ *Limonium catalaunicum* subsp. *catalaunicum* syn. sec. Malekmohammadi & al. (2024)
= *Statice flexuosa* Sennen in Butl. Inst. Catalana Hist. Nat. 32: 104. 1932 syn. sec. Malekmohammadi & al. (2024) ≡ *Limonium flexuosum* Sennen, Diagn. Nouv.: 73. 1936 syn. sec. Malekmohammadi & al. (2024)
= *Statice urgellensis* Sennen in Butl. Inst. Catalana Hist. Nat. 32: 104. 1932 syn. sec. Malekmohammadi & al. (2024)
= *Limonium ×multirameum* Sennen, Diagn. Nouv.: 97. 1936 syn. sec. Malekmohammadi & al. (2024) [is alternative name for *Statice multiramea* Sennen] ≡ *Statice multiramea* Sennen, Diagn. Nouv.: 97. 1936 syn. sec. Malekmohammadi & al. (2024) ≡ *Statice ×multiramea* Sennen, Diagn. Nouv.: 97. 1936 syn. sec. Malekmohammadi & al. (2024)

***Limonium catanense* (Tineo ex Lojac.) Brullo in Bot. Not. 133(3): 290. 1980.** Sec. Domina (2011+)

≡ *Statice catanensis* Tineo ex Lojac., Fl. Sicul. 2(2): 27. 1907 syn. sec. Domina (2011+)

Notes. – Extincted (Rossi et al. 2013)

***Limonium catanzaroi* Brullo in Bot. Not. 133(3): 289. 1980.** Sec. Peruzzi & al. (2015)

***Limonium cazzae* Bogdanović & Brullo in Phytotaxa 215(1): 14. 2015.** Sec. Bogdanović & Brullo (2015)

***Limonium cedrorum* Domina & Raimondo in Nordic J. Bot. 31(2): 194. 2012.** Sec. Domina (2011+)

***Limonium celticum* (Ingr.) P.D.Sell, Fl. Gr. Brit. Ireland 1: 691. 2018.** Sec. Sell & Murrell (2018)

≡ *Limonium britannicum* subsp. *celticum* Ingr. in Bot. J. Linn. Soc. 92(3): 203. Apr 1986 syn. sec. Sell & Murrell (2018) ≡ *Limonium britannicum* var. *celticum* Ingr. in Bot. J. Linn. Soc. 92(3): 203. Apr 1986 syn. sec. Malekmohammadi & al. (2024)

***Limonium cephalonicum* R.Artelari, Biosyst. Meleti Genous Limonium: 57. 1984.** Sec. Domina (2011+)

***Limonium cercinense* Brullo in Mitt. Bot. Staatssamml. München 28: 419. 1989.** Sec. Domina (2011+)

***Limonium chersonesum* Erben & Brullo in Phytotaxa 240: 122. 2016.** Sec. Domina (2011+)

***Limonium circaei* Pignatti in Webbia 36(1): 50. 1982.** Sec. Pignatti (1982)

***Limonium clupeanum* Brullo in Mitt. Bot. Staatssamml. München 28: 419. 1989.** Sec. Domina (2011+)

***Limonium cofrentanum* Erben in Mitt. Bot. Staatssamml. München 28: 313. 1989.** Sec. Domina (2011+)

***Limonium ×coincyi* Sennen, Diagn. Nouv.: 73. 1936.** Sec. Pignatti (1972)

= *Statice ×coincyi* Sennen, Diagn. Nouv.: 73. 1936, nom. altern. syn. sec. Pignatti (1972)

Notes. – This is a hybrid of *L. album* × *L. supinum* according to Erben (1993)

***Limonium comosum* Erben in Sendtnera 7: 80. 2001.** Sec. Domina (2011+)

***Limonium confertum* Brullo in Mitt. Bot. Staatssamml. München 28: 419. 1989.** Sec. Domina (2011+)

***Limonium confusum* (Gren. & Godr.) Fourr. in Ann. Soc. Linn. Lyon, sér. 2, 17: 141. 1869.** Sec. Domina (2011+)

≡ *Statice confusa* Gren. & Godr., Fl. France 2: 743. 1853 syn. sec. Pignatti (1971) ≡ *Limonium ramosissimum* subsp. *confusum* (Gren. & Godr.) Pignatti in Bot. J. Linn. Soc. 64(4): 366. 1971 syn. sec. Domina (2011+) − *Limonium confusum* (Gren. & Godr.) Kuntze, Revis. Gen. Pl. 2: 395. 1891, comb. inval. syn. sec. Malekmohammadi & al. (2024)
= *Statice virgata* Herb. ex Nyman, Consp. Fl. Eur. 3: 611. 1881 syn. sec. Nyman (1881)

Notes. – *Statice virgata* Herb. is synonym of *Statice confusa* according to Nyman (1881)

***Limonium connivens* Erben in Mitt. Bot. Staatssamml. München 28: 313. 1989.** Sec. Domina (2011+)

***Limonium contortirameum* (Mabille) Erben in Mitt. Bot. Staatssamml. München 30: 478. 1991.** Sec. Domina (2011+)

≡ *Statice contortiramea* Mabille in Feuille Naturalistes 7: 112. 1877 syn. sec. Erben (1991)

***Limonium contractum* Erben & Brullo in Phytotaxa 240: 116. 2016.** Sec. Domina (2011+)

***Limonium coombense* (Ingr.) P.D.Sell, Fl. Gr. Brit. Ireland 1: 690. 2018.** Sec. Sell & Murrell (2018)

≡ *Limonium britannicum* subsp. *coombense* Ingr. in Bot. J. Linn. Soc. 92(3): 201. Apr 1986 syn. sec. Sell & Murrell (2018) ≡ *Limonium britannicum* var. *coombense* Ingr. in Bot. J. Linn. Soc. 92(3): 202. Apr 1986 syn. sec. Malekmohammadi & al. (2024)

Limonium cophanense **C.Brullo, Brullo, Cambria, del Galdo & Ilardi in Phytotaxa 255(2): 153. 2015.** Sec. Domina (2011+)

Limonium cordatum **(L.) Mill., Gard. Dict., ed. 8: no. 10. 1768.** Sec. Del Guacchio & al. (2018)

= *Statice cordata* L., Sp. Pl.: 275. 1753 syn. sec. Pignatti (1971); ≡ *Taxanthema cordata* (L.) Sweet, Hort. Brit.: 332. 1826 syn. sec. Malekmohammadi & al. (2024)
= *Statice obtusa* Dum.Cours. in Bot. Cult. 1: 660. 1802 syn. sec. Schultes (1820)
= *Statice pubescens* DC., Fl. Franç., ed. 3, 5: 380. 1815 syn. sec. Pignatti (1971) ≡ *Limonium pubescens* (DC.) P.Fourn., Quatre Fl. France: 721. 1937 syn. sec. Domina (2011+)
= *Statice minuta* Vis., Fl. Dalmat. 3: 356. 1851 syn. sec. Nyman (1881)

Limonium cordovillense **Stübing & Cirujano in Anales Jard. Bot. Madrid 55(2): 471. 1997.** Sec. Malekmohammadi & al. (2024)

Limonium ×coriacifolium **Sennen, Diagn. Nouv.: 98. 1936.** Sec. Serra Laliga (2007)

≡ *Statice coriacifolia* Sennen, Diagn. Nouv.: 98. 1936 syn. sec. Serra Laliga (2007) ≡ *Limonium ×coriacifolium* (Sennen) M.B.Crespo & Serra in Ruizia 19: 250. 2007, nom. superfl. syn. sec. Serra Laliga (2007)

Notes. – This is a hybrid of *L. cossonianum ×L. supinum* according to Erben (1993).

Limonium corinthiacum **(Boiss. & Heldr.) Kuntze, Revis. Gen. Pl. 2: 395. 1891.** Sec. Dimopoulos & al. (2013)

≡ *Statice corinthiaca* Boiss. & Heldr. in Boissier, Diagn. Pl. Orient. ser. 2, 4: 69. 1859 syn. sec. Kuntze (1891)

Limonium cornarianum **Kypr. & R.Artelari in Phyton (Horn) 38(1): 144. 1998.** Sec. Koutroumpa (2024)

Limonium cornubiense **(Ingr.) P.D.Sell, Fl. Gr. Brit. Ireland 1: 691. 2018.** Sec. Sell & Murrell (2018)

≡ *Limonium procerum* var. *cornubiense* Ingr. in Bot. J. Linn. Soc. 92(3): 197. Apr 1986 syn. sec. Sell & Murrell (2018)

Limonium cornusianum **Arrigoni & Diana in Boll. Soc. Sarda Sci. Nat. 25: 169. 1986.** Sec. Peruzzi & al. (2015)

≡ *Limonium acutifolium* subsp. *cornusianum* (Arrigoni & Diana) Arrigoni in Parlatorea 7: 19. 2005 syn. sec. Peruzzi & al. (2015)

Limonium coronense **R.Artelari, Biosyst. Meleti Genous Limonium: 71. 1984.** Sec. Domina (2011+)

Limonium corsicum **Erben in Mitt. Bot. Staatssamml. München 30: 174. 1991.** Sec. Domina (2011+)

Limonium corymbulosum **(Boiss.) Kuntze, Revis. Gen. Pl. 2: 395. 1891.** Sec. Malekmohammadi & al. (2024)

≡ *Statice corymbulosa* Boiss. in Candolle, Prodr. 12: 658. 1848 syn. sec. Malekmohammadi & al. (2024) ≡ *Limonium scabrum* var. *corymbulosum* (Boiss.) R.A.Dyer in Bothalia 7(3): 491. 1961 syn. sec. Malekmohammadi & al. (2024)
= *Statice scabra* Krauss in Flora 28: 72. 1845 syn. sec. Malekmohammadi & al. (2024)

Notes. – *Statice scabra* sensu Krauss (loc. cit.) is included in synonym of *Statice corymbulosa* by Boissier (1848). However, according to Mucina (pers. comm.) there is no certainty that both names apply to the same entity. Further check is needed to clarify this point.

Limonium cossonianum **(Nyman) Kuntze, Revis. Gen. Pl. 2: 395. 1891.** Sec. Erben (1993)

≡ *Statice gummifera* var. *corymbulosa* Coss., Notes Pl. Crit.: 175. 1852 syn. sec. Erben (1993) ≡ *Statice corymbulosa* (Coss.) Nyman, Consp. Fl. Eur. 3: 609. 1881 syn. sec. Kuntze (1891) ≡ *Limonium gummiferum* var. *corymbulosum* (Coss.) Maire in Jahandiez & al., Cat. Pl. Maroc 3: 570. 1934 syn. sec. Maire (1934) ≡ *Limonium cymuliferum* var. *corymbulosum* Pignatti, Collect. Bot. (Barcelona) 6(6): 315. 1962 syn. sec. Malekmohammadi & al. (2024)
= *Statice sanjurjensis* Sennen in Bull. Soc. Hist. Nat. Afrique N. 23: 275. 1932 syn. sec. Dobignard & Chatelain, C. (2013) ≡ *Limonium sanjurjoi* Sennen & Mauricio, Cat. Fl. Rif Orient.: 97. 1933 syn. sec. Dobignard & Chatelain, C. (2013) [is alternative name for *Statice sanjurjensis* Sennen]
= *Limonium faustii* Sennen & Mauricio, Cat. Fl. Rif Orient.: 97. 1933 syn. sec. Sennen (1936) [is alternative name for *Statice faustii* Sennen & Mauricio] ≡ *Statice faustii* Sennen & Mauricio, Cat. Fl. Rif Orient.: 97. 1933 syn. sec. Sennen (1936)
= *Limonium gavilae* Sennen & Mauricio, Cat. Fl. Rif Orient.: 97. 1933 syn. sec. Sennen (1936) [is alternative name for *Statice gavilae* Sennen & Mauricio] ≡ *Statice gavilae* Sennen & Mauricio, Cat. Fl. Rif Orient.: 97. 1933 syn. sec. Sennen (1936)
= *Limonium gomezi-jordanae* Sennen & Mauricio, Cat. Fl. Rif Orient.: 97. 1933 syn. sec. Dobignard & Chatelain, C. (2013) [is alternative name for *Statice gomezi-jordanae* Sennen] ≡ *Statice gomezi-jordanae* Sennen, Diagn. Nouv.: 165. 1936 syn. sec. Dobignard & Chatelain, C. (2013)
= *Limonium molesii* Sennen & Mauricio, Cat. Fl. Rif Orient.: 97. 1933 syn. sec. Dobignard & Chatelain, C. (2013) [is alternative name for *Statice molesii* Sennen] ≡ *Statice molesii* Sennen, Diagn. Nouv.: 164. 1936 syn. sec. Dobignard & Chatelain, C. (2013)
= *Statice sanjurjoi* Sennen & Mauricio, Diagn. Nouv.: 134. 1936 syn. sec. Malekmohammadi & al. (2024)

Notes. – *Statice scabra* Krauss is synonym of *Statice corymbulosa* Boiss.
Limonium corymbulosum (Boiss.) Kuntze and *Statice corymbulosa* Boiss. are synonym of *Limonium scabrum* var. *corymbulosum* (Boiss.) R.A.Dyer

Limonium costae **(Willk.) Pignatti, Collect. Bot. (Barcelona) 6(6): 302. 1962.** Sec. Pignatti (1972)

≡ *Statice costae* Willk. in Linnaea 30: 121. 1859 syn. sec. Pignatti (1962) ≡ *Statice girardiana* subsp. *costae* (Willk.) Nyman, Consp. Fl. Eur. 3: 610. 1881 syn. sec. Domina (2011+)

Notes. – Listed as a species in the *Limonium delicatulum* group by Pignatti (1972).

Limonium cosyrense **(Guss.) Kuntze, Revis. Gen. Pl. 2: 395. 1891.** Sec. Domina (2011+)

≡ *Statice cosyrensis* Guss., Fl. Sicul. Prodr. Suppl. 1: 90. 1832 syn. sec. Domina (2011+); ≡ *Statice cosyrensis* subsp. *cosyrensis* syn. sec. Domina (2011+)
= *Statice dichotoma* Sm., Fl. Graec. Prodr. 1(1): 212. 1806, nom. illeg. syn. sec. Rechinger (1943) [non *Statice dichotoma* Cav.]
= *Statice gracilis* Tineo, Fl. Sicul. Syn. 2: 807. 1845 syn. sec. Boissier (1848) ≡ *Statice tineoi* var. *gracilis* (Tineo) J.Woods, Tourist's Fl.: 306. 1850 syn. sec. Malekmohammadi & al. (2024)
= *Statice pygmaea* Tineo, Fl. Sicul. Syn. 2: 807. 1845 syn. sec. Boissier (1848) ≡ *Statice tineoi* var. *pygmaea* (Tineo) J.Woods, Tourist's Fl.: 306. 1850 syn. sec. Malekmohammadi & al. (2024) ≡ *Statice dictyoclada* subsp. *pygmaea* (Tineo) Nyman, Consp. Fl. Eur. 3: 611. 1881 syn. sec. Domina (2011+)
= *Statice articulata* Vis., Fl. Dalmat. 2: 7. 1847 syn. sec. Nyman (1881)
= *Statice cordata* var. *maior* Boiss. in Candolle, Prodr. 12: 656. 1848 syn. sec. Rechinger (1943) ≡ *Limonium cosyrense* var. *maior* (Boiss.) Rech.f. in Denkschr. Akad. Wiss. Wien, Math.-Naturwiss. Kl. 105(1): 427. 1943 syn. sec. Malekmohammadi & al. (2024)
= *Statice dictyoclada* var. *pygmaea* Boiss. in Candolle, Prodr. 12: 654. 1848 syn. sec. Boissier (1848)

Notes. – Listed as a species in the *Limonium cosyrense* group (Pignatti, 1972).

***Limonium crateriforme* Erben & Brullo in Phytotaxa 240: 146. 2016.** Sec. Domina (2011+)

***Limonium creticum* R.Artelari in Candollea 44(2): 415. 1989.** Sec. Domina (2011+)

= *Limonium rigidum* Alf.Mayer, Libri Botanici 15: 93. 1995 syn. sec. Brullo (2016)

***Limonium croaticum* Bogdanović & Brullo in Phytotaxa 215(1): 14. 2015.** Sec. Bogdanović & Brullo (2015)

***Limonium cumanum* (Ten.) Kuntze, Revis. Gen. Pl. 2: 395. 1891.** Sec. Domina (2011+)

= *Statice cumana* Ten., Fl. Napol. 3: 351. 1829 syn. sec. Kuntze (1891); ≡ *Statice minuta* var. *cumana* (Ten.) Fiori, Fl. Italia 2: 332. 1902 syn. sec. Malekmohammadi & al. (2024) ≡ *Limonium cumanum* (Ten.) Kuntze var. *cumanum* syn. sec. Malekmohammadi & al. (2024)
= *Statice cumana* var. *glabrescens* Lacaita, Sched. Fl. Ital. Exs. ser. 3,: 137. 1917 syn. sec. Valariello & al. (2016) ≡ *Limonium cumanum* var. *glabrescens* (Lacaita) Vallariello, Iamonico & Del Guacchio in Phytotaxa 263(2): 133. 2016 syn. sec. Valariello & al. (2016)
= *Limonium johannis* Pignatti in Bot. J. Linn. Soc. 64(4): 362. 1971 syn. sec. Malekmohammadi & al. (2024: 15 Sep. 2022)

***Limonium cunicularium* Arrigoni & Diana in Boll. Soc. Sarda Sci. Nat. 27: 263. 1990.** Sec. Domina (2011+)

***Limonium cuspidatum* (Delort.) Erben in Mitt. Bot. Staatssamml. München 14: 472. 1978.** Sec. Domina (2011+)

≡ *Statice cuspidata* Delort. in Arch. Fl. France Allem.: 339. 1855 syn. sec. Erben (1978)
– *Statice globulariifolia* sensu auct., non Desf., err. sec. Domina (2011+)
– *Limonium confusum* subsp. *psilocladum* sensu auct., non (Boiss.) P.Fourn., err. sec. Domina (2011+)
– *Statice psiloclada* sensu auct., non Boiss., err. sec. Domina (2011+)
– *Limonium confusum* subsp. *raddianum* sensu auct., non (Boiss.) P.Fourn., err. sec. Domina (2011+)

***Limonium cymuliferum* (Boiss.) Sauvage & Vindt in Trav. Inst. Sci. Chérifien, Sér. Bot. 4: 68. 1952.** Sec. Domina (2011+)

≡ *Statice cymulifera* Boiss., Pugill. Pl. Afr. Bot. Hispan.: 104. 1852 syn. sec. Sauvage & Vindt (1952) ≡ *Statice gummifera* var. *cymulifera* (Boiss.) Batt., Fl. Algérie [1](4): 730. 1890 syn. sec. Malekmohammadi & al. (2024) ≡ *Limonium cymuliferum* (Boiss.) Sauvage & Vindt var. *cymuliferum* syn. sec. Domina (2011+)
= *Statice lepidorachis* Pomel, Nouv. Mat. Fl. Atl.: 127. 1874 syn. sec. Dobignard & Chatelain, C. (2013)
= *Statice lingua* Pomel in Bull. Soc. Sci. Phys. Algérie 11: 129. 1874 syn. sec. Domina (2011+) ≡ *Limonium linguum* (Pomel) Pons, Quézel, Quezel & Santa, Nouv. Fl. Alger. 2: 734. 1963 syn. sec. Domina (2011+)
= *Statice sebkarum* f. *glomerata* Batt., Fl. Algérie [1](4): 730. 1890 syn. sec. Malekmohammadi & al. (2024)
= *Statice sebkarum* f. *macrolepis* Batt., Fl. Algérie [1](4): 730. 1890 syn. sec. Malekmohammadi & al. (2024)
= *Limonium cymuliferum* var. *sebkarum* (Pomel) Sauvage & Vindt in Trav. Inst. Sci. Chérifien, Sér. Bot. 4: 69. 1952 syn. sec. Malekmohammadi & al. (2024)
= *Limonium cymuliferum* var. *uniflorum* Pignatti, Collect. Bot. (Barcelona) 6(6): 314. 1962 syn. sec. Malekmohammadi & al. (2024)
= *Statice corymbulosa* Coss. syn. sec. Domina (2011+)
– *Limonium cossonianum* sensu Greuter & al. (1989), non (Nyman) Kuntze, err. sec. Sáez & al. (2002)

***Limonium cymuliferum* subsp. *cymuliferum*.** Sec. Domina (2011+)

***Limonium cymuliferum* subsp. *mauritii* Sennen, Cat. Fl. Rif Orient.: 98. 1933.** Sec. Sennen & Mauricio (1933)

≡ *Statice cymulifera* subsp. *mauritii* Sennen, Cat. Fl. Rif Orient.: 98. 1933 syn. sec. Sennen & Mauricio (1933)

***Limonium cyprium* (Meikle) Hand in Willdenowia 33(2): 315. 2003.** Sec. Domina (2011+)

≡ *Limonium albidum* subsp. *cyprium* Meikle in Ann. Mus. Goulandris 6: 88. 1983 syn. sec. Malekmohammadi & al. (2024)

***Limonium cyrenaicum* (Rouy) Brullo in Webbia 33(1): 144. 1978.** Sec. Brullo (1978)

≡ *Statice cyrenaica* Rouy in Rev. Bot. Syst. Geogr. Bot. 1(11): 155. 1903 syn. sec. Brullo (1978)

***Limonium cyrtostachyum* (Girard) Brullo in Bot. Not. 133(3): 288. 1980.** Sec. Brullo (1980)

≡ *Statice cyrtostachya* Girard in Ann. Sci. Nat., Bot., sér. 3, 2: 328. 1844 syn. sec. Brullo (1980)

***Limonium cythereum* R.Artelari & Georgiou in Bot. J. Linn. Soc. 131(4): 404. 1999.** Sec. Domina (2011+)

***Limonium damboldtianum* Phitos & R.Artelari in Bot. Chron. 1: 18. 1981.** Sec. Domina (2011+)

***Limonium daveaui* Erben in Mitt. Bot. Staatssamml. München 14: 477. 1978.** Sec. Domina (2011+)

= *Statice confusa* var. *intermedia* Daveau in Bol. Soc. Brot. 6: 181. 1888 syn. sec. Erben (1978)
= *Limonium globulariifolium* subsp. *lusitanicum* Pignatti, Collect. Bot. (Barcelona) 6(6): 326. 1962 syn. sec. Erben (1978)

***Limonium decumbens* (Boiss.) Kuntze, Revis. Gen. Pl. 2: 395. 1891.** Sec. Bredenkamp (2003)

≡ *Statice decumbens* Boiss. in Candolle, Prodr. 12: 659. 1848 syn. sec. Kuntze (1891)

***Limonium delicatulum* (Girard) Kuntze, Revis. Gen. Pl. 2: 395. 1891.** Sec. Domina (2011+)

= *Statice delicatula* Girard in Ann. Sci. Nat., Bot., sér. 3, 2: 327. 1844 syn. sec. Kuntze (1891);
= *Statice globulariifolia* var. *glauca* Boiss., Voy. Bot. Espag. 2: 531. 1839 syn. sec. Boissier (1848)
= *Statice pyrrholepis* Pomel, Nouv. Mat. Fl. Atl. 1: 131. 1874 syn. sec. Dobignard & Chatelain, C. (2013)
= *Limonium delicatulum* var. *typicum* Maire in Jahandiez & al., Cat. Pl. Maroc 3: 570. 1934 syn. sec. Maire (1934)

Notes. – Listed as a species in the Limonium delicatulum group (Pignatti, 1972).

***Limonium delicatulum* subsp. *delicatulum*.** Sec. Domina (2011+)

***Limonium delicatulum* subsp. *eu-delicatulum* Maire in Jahandiez & al., Cat. Pl. Maroc 3: 570. 1934.** Sec. Maire (1934)

***Limonium delicatulum* subsp. *formenterae* (L.Llorens) O.Bolòs, Vigo, Masalles & Ninot, Fl. Man. Països Catalans, ed. 1: 1214. 1990.** Sec. Domina (2011+)

≡ *Limonium formenterae* L.Llorens in Lazaroa 8: 72. 1986 syn. sec. Domina (2011+)

***Limonium delicatulum* subsp. *orientale* Pignatti, Collect. Bot. (Barcelona) 6(6): 309. 1962.** Sec. Domina (2011+)

***Limonium densiflorum* (Guss.) Kuntze, Revis. Gen. Pl. 2: 395. 1891.** Sec. Domina (2011+)

≡ *Statice densiflora* Guss., Fl. Sicul. Prodr. Suppl. 1: 86. 1832 syn. sec. Domina (2011+) ≡ *Statice oleifolia* var. *densiflora* (Guss.) Fiori, Fl. Italia 2: 329. 1901 syn. sec. Erben & al. (2018)
= *Statice densiflora* var. *obtusifolia* Guss., Fl. Sicul. Syn. 1: 368. 1843 syn. sec. Erben & al. (2018)
= *Statice oxylepis* Boiss. in Candolle, Prodr. 12: 647. 1848 syn. sec. Domina (2011+)
= *Statice spathulata* Jan ex Nyman, Consp. Fl. Eur. 3: 610. 1881 syn. sec. Nyman (1881)
= *Statice willdenowiana* Herb. ex Nyman, Consp. Fl. Eur. 3: 610. 1881 syn. sec. Nyman (1881)
− *Limonium motianum* Brullo ex Erben, Del Guacchio & P.Caputo in Phytotaxa, ser. 3, 369: 187. 2018, pro syn. syn. sec. Domina (2011+)

***Limonium densissimum* (Pignatti) Pignatti in Bot. J. Linn. Soc. 64(4): 367. 1971.** Sec. Domina (2011+)

≡ *Limonium confusum* subsp. *densissimum* Pignatti in Collect. Bot. (Barcelona) 3: 379. 1953 syn. sec. Malekmohammadi & al. (2024)
= *Limonium globulariifolium* subsp. *tommasinii* Pignatti, Collect. Bot. (Barcelona) 6(6): 327. 1962 syn. sec. Domina (2011+) ≡ *Limonium ramosissimum* subsp. *tommasinii* (Pignatti) Pignatti in Bot. J. Linn. Soc. 64(4): 366. 1971 syn. sec. Domina (2011+) ≡ *Limonium tommasinii* (Pignatti) Brullo, Flora d'Italia 2: 34. 2017 syn. sec. Brullo & Guarino (2017)
= *Limonium castellonense* Socorro & S.Tárrega in Anales Jard. Bot. Madrid 40: 83. 1983 syn. sec. Malekmohammadi & al. (2024)
= *Limonium neocastellonense* Fern.Casas in Fontqueria 4: 37. 1983 syn. sec. Malekmohammadi & al. (2024)
= *Statice confusa* Rchb.f. syn. sec. Domina (2011+)

***Limonium devoniense* (Ingr.) P.D.Sell, Fl. Gr. Brit. Ireland 1: 690. 2018.** Sec. Sell & Murrell (2018)

≡ *Limonium procerum* subsp. *devoniense* Ingr. in Bot. J. Linn. Soc. 92(3): 197. Apr 1986 syn. sec. Sell & Murrell (2018)

***Limonium dianium* Pignatti in Webbia 36(1): 51. 1982.** Sec. Domina (2011+)

***Limonium dichotomum* (Cav.) Kuntze, Revis. Gen. Pl. 2: 395. 1891.** Sec. Domina (2011+)

≡ *Statice dichotoma* Cav. in Icon. 1: 37. 1791 syn. sec. Kuntze (1891) ≡ *Taxanthema dichotoma* (Cav.) Sweet, Hort. Brit.: 333. 1826 syn. sec. Domina (2011+) − *Taxanthema dichotomum* (Cav.) Sweet, Hort. Brit.: 333. 1826, orth. var. syn. sec. Malekmohammadi & al. (2024)
− *Statice dichotoma* Willk. in Flora 35: 290. 1852, nom. inval. syn. sec. Malekmohammadi & al. (2024) [is later isonym of *Statice dichotoma* Cav.]

Notes. – Willkomm (Flora 35: 290. 1852) did not describe *Statice dichotoma* as a new species. He described the features of *Statice dichotoma* Cav.

***Limonium dictyophorum* (Tausch) Degen, Fl. Veleb. 2: 540. 1937.** Sec. Bogdanović & Brullo (2015)

≡ *Statice dictyophora* Tausch, Syll. Pl. Nov. 2: 254. 1828 syn. sec. Bogdanović & Brullo (2015)
= *Statice cancellata* var. *glabra* Boiss. in Candolle, Prodr. 12: 656. 1848 syn. sec. Malekmohammadi & al. (2024)
= *Statice anfracta* C.E.Salmon, C.E.Salmon in J. Bot. 60. 1922: 345 syn. sec. C.E.Salmon in J. Bot. 62. 1924 (1924) ≡ *Limonium anfractum* (C.E.Salmon) C.E.Salmon in J. Bot. 62. 1924: 336 syn. sec. Bogdanović & Brullo (2015)

***Limonium didimense* Doğan & Akaydın in Ot Sist. Bot. Dergisi 24(2): 12. 2017.** Sec. Doğan & Akaydın (2017)

***Limonium diomedeum* Brullo in Willdenowia 17: 11. 1988.** Sec. Domina (2011+)

***Limonium dissitiflorum* (Boiss.) Kerguélen, Index Synonym. Fl. France (Coll. Patrim. Nat.) 8: 14. 1993.** Sec. Malekmohammadi & al. (2024)

≡ *Statice minuta* var. *dissitiflora* Boiss. in Candolle, Prodr. 12: 655. 1848 syn. sec. Malekmohammadi & al. (2024) ≡ *Limonium minutum* var. *dissitiflorum* (Boiss.) C.E.Salmon in J. Bot. 62. 1924: 355 syn. sec. Malekmohammadi & al. (2024)

***Limonium divaricatum* (Rouy) Brullo in Bot. Not. 133(3): 286. 1980.** Sec. Domina (2011+)

≡ *Statice virgata* var. *divaricata* Rouy, Fl. France 10: 147. 1908 syn. sec. Brullo (1980) ≡ *Limonium virgatum* subsp. *divaricatum* Pignatti in Arch. Bot. (Forlì) 31: 75. 1955 syn. sec. Brullo (1980) ≡ *Limonium graecum* subsp. *divaricatum* (Pignatti) Pignatti in Bot. J. Linn. Soc. 64(4): 366. 1971 syn. sec. Brullo (1980)
= *Statice virgata* var. *reticulata* Boiss. in Candolle, Prodr. 12: 654. 1848 syn. sec. Domina (2011+)

***Limonium dodartiforme* Ingr. in Bot. J. Linn. Soc. 92(3): 208. Apr 1986.** Sec. Ingrouille, M.J. & Stace (1986)

Notes. – Listed as a species in the *Limonium binervosum* aggregate (Ingrouille and Stace 1986).

***Limonium dodartii* (Girard) Kuntze, Revis. Gen. Pl. 2: 395. 1891.** Sec. Domina (2011+)

≡ *Statice dodartii* Girard in Ann. Sci. Nat., Bot., sér. 2, 17: 31. 1842 syn. sec. Kuntze (1891); ≡ *Statice binervosa* var. *dodarti* (Girard) Syme, Engl. Bot., ed. 3B, 7: 163. 1867 syn. sec. Malekmohammadi & al. (2024) ≡ *Limonium auriculifolium* var. *dodarti* (Girard) Druce in J. Linn. Soc., Bot. 35: 77. 1901 syn. sec. Malekmohammadi & al. (2024) ≡ *Limonium binervosum* subsp. *dodartii* (Girard) P.Fourn., Quatre Fl. France: 722. 1937 syn. sec. Domina (2011+) ≡ *Statice binervosa* var. *dodarti* (Girard) Hook syn. sec. Ingrouille, M.J. & Stace (1986)
= *Statice dichotoma* Mutel, A., Fl. Franç. 5: 171. 1838 syn. sec. Nyman (1881)
= *Statice binervosa* var. *intermedia* Syme, Engl. Bot., ed. 3, 7: 163. 1867 syn. sec. Domina (2011+)

***Limonium doerfleri* (Halácsy) Rech.f. in Denkschr. Akad. Wiss. Wien, Math.-Naturwiss. Kl. 105(1): 427. 1943.** Sec. Domina (2011+)

≡ *Statice doerfleri* Halácsy in Allg. Bot. Z. Syst. 5: 1. 1899 syn. sec. Domina (2011+) ≡ *Limonium ramosissimum* subsp. *doerfleri* (Halácsy) Pignatti in Bot. J. Linn. Soc. 64(4): 366. 1971 syn. sec. Domina (2011+)

***Limonium ×dolcheri* Pignatti in Webbia 36(1): 55. 1982.** Sec. Pignatti (1982)

≡ *Limonium ×dolcheri* Pignatti ex Dolcher & Pignatti in Boll. Soc. Sarda Sci. Nat. 8: 90. 1971 syn. sec. Pignatti (1982)

***Limonium dolihiense* Erben & Brullo in Phytotaxa 240: 54. 2016.** Sec. Domina (2011+)

***Limonium donegalense* (Ingr.) P.D.Sell, Fl. Gr. Brit. Ireland 1: 691. 2018.** Sec. Sell & Murrell (2018)

≡ *Limonium recurvum* var. *donegalense* Ingr. in Bot. J. Linn. Soc. 92: 213. 1985 syn. sec. Sell & Murrell (2018)

***Limonium doriae* (Sommier) Pignatti in Webbia 36(1): 55. 1982.** Sec. Domina (2011+)

≡ *Statice doriae* Sommier in Bull. Soc. Bot. Ital. 1902: 211. 1902 syn. sec. Domina (2011+)

***Limonium dragonericum* Erben in Mitt. Bot. Staatssamml. München 28: 345. 1989.** Sec. Domina (2011+)

***Limonium dregeanum* (C.Presl) Kuntze, Revis. Gen. Pl. 2: 395. 1891.** Sec. Bredenkamp (2003)

≡ *Statice dregeana* C.Presl in Abh. Königl. Böhm. Ges. Wiss. ser. 5, 3: 535. 1845 syn. sec. Kuntze (1891)

***Limonium dubium* (Andrews ex Guss.) Litard. in Candollea 11: 212. 1948.** Sec. Erben (2001)

≡ *Statice dubia* Andrews ex Guss., Fl. Sicul. Prodr. Suppl. 1: 89. 1832 syn. sec. Erben (2001) ≡ *Statice dictyoclada* var. *dubia* (Andrews ex Guss) Boiss. in Candolle, Prodr. 12: 654. 1848 syn. sec. Erben (2001)
= *Limonium oleifolium* var. *majus* (Guss.) Pignatti in Tutin & al., Flora Europaea 3 3: 46. 1972 syn. sec. Pignatti (1972)
= *Limonium pignattii* Brullo & Di Martino ex Brullo in Bot. Not. 133(3): 286. 1980 syn. sec. Domina (2011+)
− *Limonium pignattii* Brullo & Di Martino in Boll. Ist. Bot. Giard. Col. Palermo 26: 11. 1974, nom. inval. syn. sec. Brullo (1980)

Notes. – *Statice dubia* Andrews ex Guss. is synonym of *Statice dictyoclada* var. *dubia* Boissi. according to Boissier (1848)

***Limonium dufourii* (Girard) Kuntze, Revis. Gen. Pl. 2: 395. 1891.** Sec. Domina (2011+)

≡ *Statice dufourii* Girard in Ann. Sci. Nat., Bot., sér. 2 17: 36. 1842 syn. sec. Kuntze (1891)

***Limonium durieui* (Girard) Kuntze, Revis. Gen. Pl. 2: 395. 1891.** Sec. Domina (2011+)

≡ ?*Statice durieui* Girard in Mém. Sect. Sci. Acad. Sci. Montpellier, 1: 187. 1848 syn. sec. Kuntze (1891) − *Limonium duriaei* (Girard) Kuntze, orth. var. syn. sec. Malekmohammadi & al. (2024) [is misspelling for *Limonium durieui* (Girard) Kuntze]
= *Statice ciliata* (Lange) Rothm. in Bol. Real Soc. Esp. Hist. Nat. 34: 154. 1934 syn. sec. Malekmohammadi & al. (2024)

***Limonium duriusculum* (Girard) Fourr. in Ann. Soc. Linn. Lyon, sér. 2, 17: 141. 1869.** Sec. Domina (2011+)

≡ *Statice duriuscula* Girard in Ann. Sci. Nat., Bot., ser. 3, 2: 327. 1844 syn. sec. Domina (2011+) ≡ *Limonium confusum* subsp. *duriusculum* (Girard) P.Fourn., Quatre Fl. France: 721. 1937 syn. sec. Domina (2011+) − *Limonium duriusculum* (Girard) Kuntze, Revis. Gen. Pl. 2: 395. 1891, comb. inval. syn. sec. Malekmohammadi & al. (2024) [is later isonym of *Limonium duriusculum* (Girard) Fourr.]
= *Statice willdenowiana* Rchb., Iconogr. Bot. Pl. Crit. 2: 55. 1824 syn. sec. Boissier (1848)
– *Limonium longispicatum* sensu auct., non Erben, err. sec. Domina (2011+)

***Limonium duriusculum* subsp. *cavanillesii* (Erben) O.Bolòs, Vigo, Masalles & Ninot, Fl. Man. Països Catalans, ed. 1: 1214. 1990.** Sec. Bolòs & al. (1990)

≡ *Limonium cavanillesii* Erben in Mitt. Bot. Staatssamml. München 16(Suppl.): 556. 1980 syn. sec. Bolòs & al. (1990) − *Limonium durisculum* subsp. *cavanillesii* (Erben) O.Bolòs, Vigo, Masalles & Ninot, orth. var. syn. sec. Malekmohammadi & al. (2024)
= *Statice duriuscula* var. *valentina* Sennen & Pau in Bull. Géogr. Bot. 23: 46. 1913 syn. sec. Malekmohammadi & al. (2024) − *Statice valentina* Sennen & Pau in Bol. Soc. Ibér. Ci. Nat. 35(1-2): 32. 1936, nom. nud. syn. sec. Malekmohammadi & al. (2024)

***Limonium duriusculum* subsp. *companyonis* (Gren. & Billot) O.Bolòs, Vigo, Masalles & Ninot, Fl. Man. Països Catalans, ed. 1: 1214. 1990.** Sec. Bolòs & al. (1990)

≡ *Statice companyonis* Gren. & Billot in F.Schultz, Arch. Fl. Fr. et Allem.: 338. 1855 syn. sec. Kuntze (1891) = *Limonium companyonis* (Gren. & Billot) Kuntze, Fl. Man. Països Catalans, ed. 1: 39. 1990 syn. sec. Domina (2011+);

***Limonium duriusculum* subsp. *duriusculum*.** Sec. Domina (2011+)

***Limonium dyeri* Lincz. in Novosti Sist. Vyssh. Rast. 8: 214. 1971.** Sec. Malekmohammadi & al. (2024)

= *Limonium membranaceum* R.A.Dyer in Bothalia 7(3): 490. 1961 syn. sec. Malekmohammadi & al. (2024)

***Limonium ebusitanum* (Font Quer) Font Quer in Cavanillesia 8: 37. 1936.** Sec. Domina (2011+)

≡ *Statice ebusitana* Font Quer in Butl. Inst. Catalana Hist. Nat. 25: 100. 1925 syn. sec. Domina (2011+) ≡ *Limonium inarimense* subsp. *ebusitanum* (Font Quer) Pignatti in Arch. Bot. (Forlì) 31: 84. 1955 syn. sec. Domina (2011+)

***Limonium ejulabilis* Rosselló, Mus & J.X.Soler in Anales Jard. Bot. Madrid 51(2): 201. 1994.** Sec. Rosselló & al. (1994)

***Limonium elaphonisicum* Alf.Mayer, Libri Botanici 15: 96. 1995.** Sec. Domina (2011+)

***Limonium elfahsianum* Brullo & Giusso in Bocconea 19: 143. 2006.** Sec. Domina (2011+)

***Limonium emarginatum* (Willd.) Kuntze, Revis. Gen. Pl. 2: 395. 1891.** Sec. Domina (2011+)

≡ *Statice emarginata* Willd., Enum. Pl.: 335. 1809 syn. sec. Kuntze (1891) ≡ *Taxanthema emarginata* (Willd.) Sweet, Hort. Brit.: 332. 1826 syn. sec. Malekmohammadi & al. (2024) ≡ *Statice spathulata* var. *emarginata* (Willd.) Boiss. in Candolle, Prodr. 12: 650. 1848 syn. sec. Erben (1993) ≡ *Limonium spathulatum* var. *emarginatum* (Willd.) C.E.Hubb. & Sandwith in Bull. Misc. Inform. Kew 1928: 151. 1928 syn. sec. Malekmohammadi & al. (2024)

***Limonium equisetinum* (Boiss.) R.A.Dyer in Bull. Misc. Inform. Kew 1932(3): 155. 1932.** Sec. Bredenkamp (2003)

≡ *Statice equisetina* Boiss. in Candolle, Prodr. 12: 658. 1848 syn. sec. Malekmohammadi & al. (2024)
= *Statice scabrida* C.Presl in Abh. Königl. Böhm. Ges. Wiss. ser. 5, 3: 535. 1845 syn. sec. Malekmohammadi & al. (2024)

***Limonium ×erectiflorum* (B.Fedtsch. & Gontsch.) A.V.Grebenjuk in Novosti Sist. Vyssh. Rast. 37: 163. 2005.** Sec. Malekmohammadi & al. (2024)

≡ *Statice ×erectiflora* B.Fedtsch. & Gontsch. in Trudy Bot. Sada Akad. Nauk S.S.S.R. 41: 98. 1929 syn. sec. Malekmohammadi & al. (2024)

***Limonium erectum* Erben in Mitt. Bot. Staatssamml. München 14: 431. 1978.** Sec. Domina (2011+)

***Limonium ×escarrei* L.Llorens & Tébar in Anales Jard. Bot. Madrid 45(1): 173. 1988.** Sec. Bolòs & al. (1990)

≡ *Limonium minutum* subsp. *escarrei* (L.Llorens & Tébar) O.Bolòs, Vigo, Masalles & Ninot, Fl. Man. Països Catalans, ed. 1: 1214. 1990 syn. sec. Bolòs & al. (1990)
= *Limonium ×virgolsii* Pignatti in Arch. Bot. (Forlì) 31: 97. 1955 syn. sec. Pignatti (1955)

Notes. – Without latin description

***Limonium estevei* Fern.Casas in Cuad. Ci. Biol. 1(1): 23. 1971.** Sec. Domina (2011+)

= *Limonium almeriense* Pount in Bull. Soc. Bot. France 120: 341. 1973 syn. sec. Erben (1993)

***Limonium etruscum* Arrigoni & Rizzotto in Webbia 39(1): 129. 1985.** Sec. Domina (2011+)

***Limonium ×eugeniae* Sennen, Diagn. Nouv.: 98. 1936, nom. altern.** Sec. Sennen (1936)

≡ *Statice eugeniae* Sennen, Diagn. Nouv.: 98. 1936 syn. sec. Malekmohammadi & al. (2024) ≡ *Limonium tunetanum* subsp. *eugeniae* (Sennen) Pignatti in Nuovo Giorn. Bot. Ital. nov. ser. 60: 334. 1953 syn. sec. Domina (2011+)

Notes. – *Limonium ×eugeniae* is the hybrid *L. delicatulum* × *L. supinum* according to Erben (1993).

***Limonium flagellare* (Lojac.) Brullo in Bot. Not. 133(3): 284. 1980.** Sec. Domina (2011+)

≡ *Statice flagellaris* Lojac., Fl. Sicul. 2(2): 27. 1907 syn. sec. Brullo (1980)

***Limonium florentinum* Arrigoni & Diana in Candollea 48(2): 650. 1993.** Sec. Domina (2011+)

***Limonium fontqueri* (Pau) L.Llorens ex Greuter in Folia Bot. Misc. 4: 56. 1984.** Sec. Domina (2011+)

≡ *Statice fontqueri* Pau in Butl. Inst. Catalana Hist. Nat. 14: 142. 1914 syn. sec. Domina (2011+) ≡ *Limonium virgatum* subsp. *fontqueri* (Pau) O.Bolòs, Vigo, Masalles & Ninot, Fl. Man. Països Catalans, ed. 1: 1214. 1990 syn. sec. Domina (2011+)

***Limonium formosum* Bartolo, Brullo & Giusso in Bocconea 16(2): 537. 2003.** Sec. Domina (2011+)

***Limonium fradinianum* (Pomel) Erben in Fl. Medit. 22: 64. 2012.** Sec. Erben & al. (2012)

≡ *Statice fradiniana* Pomel, Nouv. Mat. Fl. Atl.: 128. 1874 syn. sec. Erben & al. (2012) ≡ *Statice oleifolia* var. *fradiniana* (Pomel) Batt., Fl. Algérie [1](4): 731. 1890 syn. sec. Battandier (1890)

Notes. – *Statice fradiniana* Pomel is synonym of *Statice oleifolia* var. *fradiniana* according to Batt. (1890)

***Limonium fragile* Erben & Brullo in Phytotaxa 240: 52. 2016.** Sec. Brullo (2016)

***Limonium ×fraternum* (Sennen & Pau) M.B.Crespo, Phytokeys: 2024.** Sec. Malekmohammadi & al. (2024)

≡ *Statice fraterna* Sennen & Pau in Bull. Acad. Int. Geogr. Bot. 23: 47. 1913 syn. sec. Malekmohammadi & al. (2024)

Notes. – *Limonium ×fraternum* taxon is a hybrid of *L. hibericum* × *L. viciosoi* according to Erben (1993).

***Limonium frederici* (Barbey) Rech.f. in Denkschr. Akad. Wiss. Wien, Math.-Naturwiss. Kl. 105(1): 427. 1943.** Sec. Domina (2011+)

≡ *Statice frederici* Barbey, Karpathos: 127. t. 13. 1895 syn. sec. Rechinger (1943)

Notes. – Limonium frederici is listed as a species in the *Limonium albidum* group (Pignatti, 1972).

***Limonium furfuraceum* (Lag.) Kuntze, Revis. Gen. Pl. 2: 395. 1891.** Sec. Domina (2011+)

≡ *Statice furfuracea* Lag., Gen. Sp. Pl.: 13. 1816 syn. sec. Kuntze (1891)
= *Statice pruinosa* Forssk. ex Schult. in Roemer & Schultes, Syst. Veg. ed. 15[bis] 6: 794. 1820 syn. sec. Malekmohammadi & al. (2024)

***Limonium furnarii* Brullo in Bot. Not. 133(3): 286. 1980.** Sec. Domina (2011+)

***Limonium galilaeum* Domina, Danin & Raimondo in Fl. Medit. 16: 133. 2006.** Sec. Malekmohammadi & al. (2024)

***Limonium gallicum* (Pignatti) Domina in Willdenowia 41(1): 131. 2011.** Sec. Domina (2011+)

≡ *Limonium delicatulum* subsp. *gallicum* Pignatti, Collect. Bot. (Barcelona) 6(6): 316. 1962 syn. sec. Domina (2011+) ≡ *Limonium ovalifolium* subsp. *gallicum* Pignatti, Collect. Bot. (Barcelona) 6(6): 316. 1962 syn. sec. Domina (2011+)

***Limonium gallurense* Arrigoni & Diana in Boll. Soc. Sarda Sci. Nat. 25: 177. 1986.** Sec. Domina (2011+)

***Limonium geronense* Erben in Mitt. Bot. Staatssamml. München 14: 467. 1978.** Sec. Domina (2011+)

***Limonium gibertii* (Sennen) Sennen, Diagn. Nouv.: 271. 1936.** Sec. Domina (2011+)

≡ *Statice gibertii* Sennen in Ann. Soc. Linn. Lyon, ser. 2, 69: 113. 1923 [as " *giberti* "] syn. sec. Domina (2011+) − *Limonium giberti* (Sennen) Sennen, orth. var. syn. sec. Domina (2011+) − *Statice giberti* Sennen, orth. var. syn. sec. Domina (2011+)

Notes. – Listed as a species in the *Limonium delicatulum* group (Pignatti, 1972).

***Limonium ginae* P.D.Sell, Fl. Gr. Brit. Ireland 1: 690. 2018.** Sec. Sell & Murrell (2018)

***Limonium ginzbergeri* Bogdanović & Brullo in Phytotaxa 215(1): 16. 2015.** Sec. Bogdanović & Brullo (2015)

***Limonium girardianum* (Guss.) Fourr. in Ann. Soc. Linn. Lyon, sér. 2, 17: 141. 1869.** Sec. Domina (2011+)

≡ *Statice girardiana* Guss., Fl. Sicul. Syn. 1: 368. 1843 syn. sec. Domina (2011+) ≡ *Limonium girardianum* (Guss.) Kuntze, Revis. Gen. Pl. 2: 395. 1891, comb. illeg. syn. sec. Malekmohammadi & al. (2024) ≡ *Statice girardiana* subsp. *girardiana* syn. sec. Domina (2011+)
= *Statice densiflora* Girard in Ann. Sci. Nat., Bot., sér. 2 17: 25. 1842 syn. sec. Domina (2011+)
= *Limonium densiflorum* Maire & Petitm., Matér. Étude Fl. Géogr. Bot. Orient 4: 186. 1908 syn. sec. Malekmohammadi & al. (2024)

***Limonium ×glaucophyllum* Pignatti in Collect. Bot. (Barcelona) 3: 380. 1953.** Sec. Sáez & al. (1998)

***Limonium glomeratum* (Tausch) Erben in Mitt. Bot. Staatssamml. München 16(Suppl.): 561. 1980.** Sec. Domina (2011+)

≡ *Statice glomerata* Tausch, Syll. Pl. Nov. 2: 255. 1828 syn. sec. Domina (2011+)

***Limonium gorgonae* Pignatti in Webbia 36(1): 51. 1982.** Sec. Domina (2011+)

= *Limonium savianum* Pignatti in Webbia 36(1): 52. 1982 syn. sec. Peruzzi & al. (2015)

***Limonium ×gougemolsii* Pignatti in Arch. Bot. (Forlì) 31: 100. 1955.** Sec. Pignatti (1955)

Notes. – without Latin description

***Limonium gougetianum* (Girard) Kuntze, Revis. Gen. Pl. 2: 395. 1891.** Sec. Domina (2011+)

≡ *Statice gougetiana* Girard in Ann. Sci. Nat., Bot., ser. 3, 2: 328. 1844 syn. sec. Kuntze (1891)
= *Statice minuta* Desf., Fl. Atlant. 1: 275. 1798 syn. sec. Boissier (1848)
= *Statice dichotoma* Rchb., Iconogr. Bot. Pl. Crit. 8: 7, t. 718. 1830, nom. illeg. syn. sec. Boissier (1848) [non *Statice dichotoma* Cav.]
= *Statice scopoliana* Bertol., Fl. Ital. 3: 528. 1837 syn. sec. Boissier (1848)

***Limonium grabusae* Erben & Brullo in Phytotaxa 240: 135. 2016.** Sec. Domina (2011+)

***Limonium graecum* (Poir.) Kuntze, Revis. Gen. Pl. 2: 395. 1891.** Sec. Domina (2011+)

≡ *Statice graeca* Poir., Encycl. Suppl. 5: 237. 1817 syn. sec. Kuntze (1891); ≡ *Limonium graecum* (Poir.) Rech.f. in Denkschr. Akad. Wiss. Wien, Math.-Naturwiss. Kl. 105(1): 427. 1943, comb. illeg. syn. sec. Malekmohammadi & al. (2024) ≡ *Limonium graecum* subsp. *graecum* syn. sec. Domina (2011+)
= *Statice graeca* Boiss. in Candolle, Prodr. 12: 650. 1848 syn. sec. Malekmohammadi & al. (2024)
= *Statice graeca* var. *microphylla* Boiss. in Candolle, Prodr. 12: 650. 1848 syn. sec. Malekmohammadi & al. (2024)
= *Statice verrucosa* Willd. ex Boiss. in Candolle, Prodr. 12: 650. 1848 syn. sec. Malekmohammadi & al. (2024)

***Limonium grandicaule* (Ingr.) P.D.Sell, Fl. Gr. Brit. Ireland 1: 690. 2018.** Sec. Sell & Murrell (2018)

≡ *Limonium britannicum* var. *grandicaule* Ingr. in Bot. J. Linn. Soc. 92(3): 202. Apr 1986 syn. sec. Sell & Murrell (2018)

***Limonium greuteri* Erben in Willdenowia 36(1): 145. 2006.** Sec. Domina (2011+)

***Limonium grosii* L.Llorens in Lazaroa 8: 76-78. 1985.** Sec. Malekmohammadi & al. (2024)

≡ *Limonium girardianum* subsp. *grosii* (L.Llorens) O.Bolòs, Vigo, Masalles & Ninot, Fl. Man. Països Catalans, ed. 1: 1214. 1990 syn. sec. Domina (2011+)

***Limonium gueneri* Doğan, H.Duman & Akaydın in Ann. Bot. Fenn. 45: 390. 2008.** Sec. Domina (2011+)

***Limonium gummiferum* (Durieu ex Boiss. & Reut.) Kuntze, Revis. Gen. Pl. 2: 395. 1891.** Sec. Domina (2011+)

≡ *Statice gummifera* Durieu ex Boiss. & Reut., Pugill. Pl. Afr. Bot. Hispan.: 104. 1852 syn. sec. Kuntze (1891)
– *Statice globulariifolia* sensu auct., non Desf., err. sec. Maire (1934)

***Limonium gummiferum* subsp. *gummiferum*.** Sec. Domina (2011+)

= *Limonium gummiferum* subsp. *eu-gummiferum* Maire, Bull. Soc. Hist. Nat. Afrique N. 1923 syn. sec. Maire (1934)

− *Limonium gummiferum* var. *typicum* Maire in Jahandiez & al., Cat. Pl. Maroc 3: 570. 1934, nom. inval. syn. sec. Malekmohammadi & al. (2024)

***Limonium gummiferum* var. *muticum* Maire & Sennen, Bull. Soc. Hist. Nat. Afrique N. 24: 222. 1932.** Sec. Maire (1934)

***Limonium gymnesicum* Erben in Mitt. Bot. Staatssamml. München 22: 204. 1986.** Sec. Domina (2011+)

***Limonium halophilum* Pignatti ex Brullo in Bot. Not. 133(3): 290. 1980.** Sec. Domina (2011+)

≡ *Limonium ramosissimum* subsp. *siculum* Pignatti in Bot. J. Linn. Soc. 64(4): 366. 1971 syn. sec. Domina (2011+)

***Limonium helenae* Erben & Brullo in Phytotaxa 240: 163. 2016.** Sec. Domina (2011+)

***Limonium heraionense* Erben & Brullo in Phytotaxa 240: 175. 2016.** Sec. Domina (2011+)

***Limonium hermaeum* (Pignatti) Pignatti in Bot. J. Linn. Soc. 64(4): 365. 1971.** Sec. Pignatti (1972)

≡ *Limonium tenuiculum* subsp. *hermaeum* Pignatti in Nuovo Giorn. Bot. Ital. 67: 18. 1960 syn. sec. Pignatti (1971)

***Limonium heterospicatum* Erben in Mitt. Bot. Staatssamml. München 28: 383. 1989.** Sec. Domina (2011+)

***Limonium hibericum* Erben in Mitt. Bot. Staatssamml. München 27: 385. 1988.** Sec. Domina (2011+)

= *Statice duriuscula* var. *procera* Willk. in Willkomm & Lange, Prodr. Fl. Hispan. 2: 376. 1868 syn. sec. Erben (1988) ≡ *Limonium catalaunicum* subsp. *procerum* (Willk.) Pignatti, Collect. Bot. (Barcelona) 6(6): 300. 1962 syn. sec. Erben (1988)

***Limonium hibernicum* (Ingr.) P.D.Sell, Fl. Gr. Brit. Ireland 1: 691. 2018.** Sec. Sell & Murrell (2018)

≡ *Limonium procerum* var. *hibernicum* Ingr. in Bot. J. Linn. Soc. 92(3): 195. Apr 1986 syn. sec. Sell & Murrell (2018)

***Limonium hierapetrae* Rech.f. in Denkschr. Akad. Wiss. Wien, Math.-Naturwiss. Kl. 105 (2,1): 104. 1943.** Sec. Koutroumpa (2024)

= *Limonium chrisianum* Brullo & Guarino in Flora Medit. 10: 269. 2000 syn. sec. Koutroumpa (2024)
= *Limonium minoicum* Erben & Brullo in Phytotaxa 240: 107. 2016 syn. sec. Koutroumpa (2024)

***Limonium himariense* F.K.Mey. in Haussknechtia Beih. 15: 113. 2011.** Sec. F.K.Mey. (2011)

***Limonium hipponense* Brullo in Mitt. Bot. Staatssamml. München 28: 432. 1989.** Sec. Domina (2011+)

***Limonium hyblaeum* Brullo in Bot. Not. 133(3): 282. 1980.** Sec. Domina (2011+)

= *Statice bellidifolia* Guss., FI. Sic. Syn. 1: 369. 1843 syn. sec. Domina (2011+)
= *Statice psiloclada* var. *gracilis* Boiss. in Candolle, Prodr. 12: 651. 1848 syn. sec. Domina (2011+)
= *Statice psiloclada* var. *spathulaefolia* Lojac., FI. Sic. 2(2): 18. 1907 syn. sec. Domina (2011+)

***Limonium ikaricum* Erben & Brullo in Phytotaxa 240: 124. 2016.** Sec. Domina (2011+)

***Limonium ilergabonum* López-Alvarado, Cobacho, Arán & L.Sáez in Phytotaxa 331(2): 201. 2017.** Sec. López-Alvarado & al. (2017)

***Limonium ilvae* Pignatti in Webbia 36(1): 51. 1982.** Sec. Peruzzi & al. (2015)

***Limonium inarimense* (Guss.) Pignatti in Arch. Bot. (Forlì) 31: 84. 1955.** Sec. Domina (2011+)

≡ *Statice inarimensis* Guss., Enum. Pl. Inarim.: 267. 1855 syn. sec. Domina (2011+) ≡ *Statice minuta* var. *inarimensis* (Guss.) Fiori, Fl. Italia 2: 331. 1902 syn. sec. Valariello & al. (2016)

***Limonium inexpectans* L.Sáez & Rosselló in Anales Jard. Bot. Madrid 54: 286. 1996.** Sec. Sáez & Rosselló (1996)

***Limonium insulare* (Bég. & Landi) Arrigoni & Diana in Boll. Soc. Sarda Sci. Nat. 27: 275. 1990.** Sec. Domina (2011+)

≡ *Statice laeta* subsp. *insularis* Bég. & Landi in Arch. Bot. Sist. 7: 57. 1931 syn. sec. Arrigoni & Diana (1990) ≡ *Limonium laetum* subsp. *insulare* (Bég. & Landi) Atzei & V.Picci in Arch. Bot. Biogeogr. Ital. 53: 25. 1977 syn. sec. Domina (2011+)

***Limonium intercedens* P.D.Sell, Fl. Gr. Brit. Ireland 1: 687. 2018.** Sec. Sell & Murrell (2018)

***Limonium ×interjectum* J.X.Soler & Rosselló in Anales Jard. Bot. Madrid 55(1): 11. 1997.** Sec. Malekmohammadi & al. (2024)

= *Statice virgata* var. *macroclada* Pau in Actas Soc. Esp. Hist. Nat. 27: 199. 1898 syn. sec. Soler & Rosselló (1997)
= *Limonium dianiae* (Pau) A.Barber, M.B.Crespo & Lledó, Contr. Coneix. Fl. Fitogeogr. Lit. Comarca Marina Alta: 126. 1999 syn. sec. Malekmohammadi & al. (2024) − *Statice dianiae* Pau in Actas Soc. Esp. Hist. Nat. 27: 199. 1898, nom. inval. syn. sec. Soler & Rosselló (1997)

Notes. – This name was described as a possible hybrid *L. girardianum* × *L. virgatum* (Soler & Rosselló 1997), but it grows in a very small area where *L. girardianum* does not occur. Conversely, *L. interjectum* is always found together with *L. rigualii* and *L. virgatum*, and it is most probably the hybrid *L. rigualii* × *L. virgatum*.

***Limonium intermedium* (Guss.) Brullo in Bot. Not. 133(3): 283. 1980.** Sec. Domina (2011+)

≡ *Statice intermedia* Guss., Fl. Sicul. Prodr. Suppl. 1: 87. 1832 syn. sec. Brullo (1980) ≡ *Statice psiloclada* var. *intermedia* (Guss.) Boiss. in Candolle, Prodr. 12: 651. 1848 syn. sec. Peruzzi & al. (2015)

Notes. – Extinct in the willd (Rossi et al. 2013)

***Limonium intricatum* Brullo in Mitt. Bot. Staatssamml. München 28: 419. 1989.** Sec. Domina (2011+)

***Limonium ionicum* Brullo in Bot. Not. 133(3): 288. 1980.** Sec. Domina (2011+)

= *Limonium punicum* Brullo in Mitt. Bot. Staatssamml. München 28: 419. 1989 syn. sec. Domina (2011+)

***Limonium irtaense* Ferrer-Gallego, P. P., A.Navarro, P.Pérez, R.Roselló, Rosselló, M.Rosato & E.Laguna in Phytotaxa 234(3): 264. 2015.** Sec. Malekmohammadi & al. (2024)

***Limonium isidorum* Erben & Brullo in Phytotaxa 240: 165. 2016.** Sec. Brullo (2016)

***Limonium issaeum* Bogdanović & Brullo in Phytotaxa 215(1): 17. 2015.** Sec. Bogdanović & Brullo (2015)

***Limonium istriacum* Bogdanović & Brullo in Phytotaxa 215(1): 18. 2015.** Sec. Bogdanović & Brullo (2015)

***Limonium ithacense* R.Artelari in Bot. Chronika 4(1–2): 26. 1984.** Sec. Domina (2011+)

***Limonium jankae* (Lojac.) Giardina & Raimondo in Bocconea 20: 12. 2007.** Sec. Domina (2011+)

≡ *Statice jankae* Lojac., Fl. Sicul. 2(2): 24. 1907 syn. sec. Giardina & al. (2007)

***Limonium japygicum* (E.Groves) Pignatti, Galasso & Nicolella in Inform. Bot. Ital. 46(1): 81. 2014.** Sec. Domina (2011+)

≡ *Statice cancellata* var. *japygica* E.Groves in Nuovo Giorn. Bot. Ital. 19: 184. 1887 syn. sec. Pignatti (1971) − *Limonium japygicum* (E.Groves) Pignatti in Bot. J. Linn. Soc. 64(4): 364. 1971, comb. inval. syn. sec. Malekmohammadi & al. (2024)

***Limonium kairouanum* Brullo in Mitt. Bot. Staatssamml. München 28: 438. 1989.** Sec. Domina (2011+)

***Limonium kardamylii* R.Artelari & Kamari in Phyton (Horn) 35(1): 132. 1995.** Sec. Domina (2011+)

***Limonium karpasiticum* Kefalas, Erben, Christodoulou & Hand in Fl. Medit. 32: 37. 2022.** Sec. Erben & al. (2022)

***Limonium kelseyanum* (Ingr.) P.D.Sell, Fl. Gr. Brit. Ireland 1: 691. 2018.** Sec. Sell & Murrell (2018)

≡ *Limonium britannicum* var. *kelseyanum* Ingr. in Bot. J. Linn. Soc. 92(3): 201. Apr 1986 syn. sec. Sell & Murrell (2018)

***Limonium kerryense* (Ingr.) P.D.Sell, Fl. Gr. Brit. Ireland 1: 691. 2018.** Sec. Sell & Murrell (2018)

≡ *Limonium recurvum* var. *kerryense* Ingr. in Bot. J. Linn. Soc. 92(3): 212. Apr 1986 syn. sec. Sell & Murrell (2018)

***Limonium kirikosicum* Erben & Brullo in Phytotaxa 240: 172. 2016.** Sec. Domina (2011+)

***Limonium korakonisicum* Valli in Phytotaxa 217(1): 65. 2015.** Sec. Malekmohammadi & al. (2024)

***Limonium korbousense* Brullo in Mitt. Bot. Staatssamml. München 28: 419. 1989.** Sec. Domina (2011+)

***Limonium kraussianum* (Buchinger ex Boiss.) Kuntze, Revis. Gen. Pl. 2: 395. 1891.** Sec. Malekmohammadi & al. (2024)

− *Statice kraussiana* Buchinger ex Boiss. in Flora 28: 73. 1845, nom. nud. syn. sec. Kuntze (1891)
= *Statice aretiifolia* Fr. ex Boiss. in Candolle, Prodr. 12: 657. 1848 syn. sec. Malekmohammadi & al. (2024) ≡ *Statice linifolia* var. *aretiifolia* Boiss. in Candolle, Prodr. 12: 657. 1848 syn. sec. Malekmohammadi & al. (2024)

Notes. – *Statice aretiifolia* and *S. linifolia* var. *aretiifolia* are placed in synonymy of *L. kraussianum* based on personal comunication with Dr. Ladislav Mucina.

***Limonium ksamilum* Bogdanović, Shuka, Giusso & Brullo in Phytotaxa 554(1): 86. 2022.** Sec. Bogdanović & al. (2022)

***Limonium lacertosum* Brullo in Mitt. Bot. Staatssamml. München 28: 419. 1989.** Sec. Domina (2011+)

***Limonium lacinium* Arrigoni in Boll. Soc. Sarda Sci. Nat. 22: 227. 1983.** Sec. Domina (2011+)

***Limonium laetum* (Nyman) Pignatti in Bot. J. Linn. Soc. 64(4): 365. 1971.** Sec. Domina (2011+)

≡ *Statice australis* Moris, Diagn. Stirp. Sard.: 1. 1857, nom. illeg. syn. sec. Malekmohammadi & al. (2024) ≡ *Statice duriuscula* subsp. *laeta* Nyman, Consp. Fl. Eur. 3: 611. 1881 syn. sec. Pignatti (1971)
= *Statice laeta* Moris, Fl. Sardoa 3: 42. 1859 syn. sec. Pignatti (1971)
= *Limonium turritanum* Diana in Boll. Soc. Sarda Sci. Nat. 17: 267. 1978 syn. sec. Domina (2011+)

***Limonium lagostanum* Bogdanović & Brullo in Phytotaxa 215(1): 18. 2015.** Sec. Bogdanović & Brullo (2015)

***Limonium lambinonii* Erben in Sendtnera 8: 25. 2002.** Sec. Domina (2011+)

***Limonium lanceolatum* (Hoffmanns. & Link) Franco, Nova Fl. Portugal 2: 564. 1984.** Sec. Domina (2011+)

≡ *Statice lanceolata* Hoffmanns. & Link, Fl. Portug. 2 (Lief. 16): 446, t. 78. 1813 syn. sec. Malekmohammadi & al. (2024) ≡ *Limonium ovalifolium* subsp. *lanceolatum* (Hoffmanns. & Link) Franco syn. sec. Domina (2011+) − *Statice laneolata* Hoffmanns. & Link, orth. var. syn. sec. Domina (2011+) [is misspelling for *Statice lanceolata* Hoffmanns. & Link]
= *Statice ovalifolia* var. *lanceolata* Rouy in Rev. Bot. Syst. Geogr. Bot. 1(11): 163. 1903 syn. sec. Malekmohammadi & al. (2024)

***Limonium lanfrancoi* Agius, M.E.Galea, Cambria, del Galdo & Brullo, Phytotaxa 622(2): 102. 2023.** Sec. Agius & al. (2023)

***Limonium latibracteatum* Erben in Mitt. Bot. Staatssamml. München 14: 516. 1978.** Sec. Domina (2011+)

≡ *Limonium delicatulum* subsp. *latibracteatum* (Erben) Castrov. & Cirujano in Anales Jard. Bot. Madrid 37: 214. 1980 syn. sec. Domina (2011+) − *Limonium latebracteatum* Erben, orth. var. syn. sec. Domina (2011+) [is misspelling for *Limonium latibracteatum* Erben] − *Limonium delicatulum* subsp. *latebracteatum* (Erben) Castrov. & Cirujano, orth. var. syn. sec. Domina (2011+) [is misspelling for *Limonium delicatulum* subsp. *latibracteatum* (Erben) Castrov. & Cirujano]

***Limonium lausianum* Pignatti in Bot. J. Linn. Soc. 64(4): 369. 1971.** Sec. Domina (2011+)

Notes. – This species is listed as a species in the *Limonium delicatulum* group (Pignatti, 1972).

***Limonium laxiusculum* Franco, Nova Fl. Portugal 2: 564. 1984.** Sec. Domina (2011+)

***Limonium legrandii* (Gaut. & Timb.-Lagr.) Erben in Mitt. Bot. Staatssamml. München 14: 475. 1978.** Sec. Domina (2011+)

≡ *Statice legrandii* Gaut. & Timb.-Lagr. in Bull. Soc. Sci. Phys. Nat. Toulouse 4: 40. 1878 syn. sec. Erben (1978)

***Limonium leonardi-llorensii* L.Sáez, Á.C.Carvalho & Rosselló in Anales Jard. Bot. Madrid 56(1): 34. 1998.** Sec. Malekmohammadi & al. (2024)

***Limonium leprosorum* Bogdanović & Brullo in Phytotaxa 215(1): 19. 2015.** Sec. Bogdanović & Brullo (2015)

***Limonium letourneuxii* (Coss. ex Batt.) Greuter & Raus in Willdenowia 16(2): 450. 1987.** Sec. Domina (2011+)

≡ *Statice letourneuxii* Coss. ex Batt., Fl. Algérie [1](4): 734. 1890 syn. sec. Greuter & Raus (1987) − *Limonium letourneuxii* (Coss. ex Batt.) A.Pons & Quézel, Nouv. Fl. Alger. 2: 732. 1963, comb. inval. syn. sec. Greuter & Raus (1987)

***Limonium liberianum* Bogdanović & Brullo in Phytotaxa 215(1): 20. 2015.** Sec. Bogdanović & Brullo (2015)

***Limonium liburnicum* Bogdanović & Brullo in Phytotaxa 215(1): 20. 2015.** Sec. Bogdanović & Brullo (2015)

***Limonium lilybaeum* Brullo in Bot. Not. 133(3): 290. 1980.** Sec. Domina (2011+)

***Limonium linifolium* (L.f.) Chaz. in Miller, Suppl. Dict. Jard. 2: 35. 1790.** Sec. Malekmohammadi & al. (2024)

≡ *Statice linifolia* L.f., Suppl. Pl.: 187. 1782 ["1781"] syn. sec. Kuntze (1891) ≡ *Limonium linifolium* (L.f.) Kuntze, Revis. Gen. Pl. 2: 395. 1891, nom. illeg. syn. sec. Bredenkamp (2003)
= *Statice linifolia* var. *brachyphylla* Boiss. in Candolle, Prodr. 12: 657. 1848 syn. sec. Malekmohammadi & al. (2024)
= *Statice linifolia* var. *robusta* C.H.Wright in Thiselton-Dyer, Flora Capensis 4(1.3): 421. 1906 syn. sec. Malekmohammadi & al. (2024)

Notes. – According to Mucina (pers. comm.), *Statice linifolia* sensu Drege ex Boiss. is a synonym of *Limonium pedicellatum*, which is clearly distinct from both *L. scabrum* and *L. dregeanum*.

***Limonium linifolium* (L.f.) Chaz. var. *linifolium*.** Sec. Malekmohammadi & al. (2024)

***Limonium linifolium* var. *maritimum* (Eckl. & Zeyh. ex Boiss.) R.A.Dyer in Bothalia 7(3): 490. 1961.** Sec. Malekmohammadi & al. (2024)

≡ *Statice linifolia* var. *maritima* Eckl. & Zeyh. ex Boiss. in Candolle, Prodr. 12: 657. 1848 syn. sec. R.A.Dyer (1961)

***Limonium lobetanicum* Erben in Mitt. Bot. Staatssamml. München 28: 313. 1989.** Sec. Domina (2011+)

***Limonium loganicum* Ingr. in Bot. J. Linn. Soc. 92(3): 205. Apr 1986.** Sec. Ingrouille, M.J. & Stace (1986)

Notes. – Listed as a species in the *Limonium binervosum* aggregate (Ingrouille and Stace 1986).

***Limonium lojaconoi* Brullo in Bot. Not. 133(3): 286. 1980.** Sec. Domina (2011+)

≡ *Statice sicula* Tineo ex Lojac., Fl. Sicul. (Lojacono) 2(2): 20. 1907 syn. sec. Brullo (1980) − *Limonium lajaconi* Brullo, orth. var. syn. sec. Malekmohammadi & al. (2024) [is misspelling for *Limonium lojaconoi* Brullo]

***Limonium longibracteatum* Erben in Mitt. Bot. Staatssamml. München 27: 393. 1988.** Sec. Domina (2011+)

***Limonium lopadusanum* Brullo in Bot. Not. 133(3): 281. 1980.** Sec. Domina (2011+)

***Limonium lovricii* Bogdanović & Brullo in Phytotaxa 215(1): 21. 2015.** Sec. Bogdanović & Brullo (2015)

***Limonium lowei* R.Jardim, M.Seq., Capelo, J.C.Costa & Rivas Mart., Silva Lusitana 15(2): 277. 2007.** Sec. Domina (2011+)

≡ *Statice pyramidata* Lowe in Trans. Cambridge Philos. Soc. 4(1): 18. 1831 syn. sec. Domina (2011+) ≡ *Statice ovalifolia* var. *pyramidata* (Lowe) Menezes, Fl. Arch. Madeira: 108. 1914 syn. sec. Domina (2011+) ≡ *Limonium ovalifolium* subsp. *pyramidatum* (Lowe) A.Hansen & Sunding, Fl. Macaronesia, ed. 2, 1: 92. 1979 syn. sec. Domina (2011+)

***Limonium ×lucentinum* Pignatti & Freitag in Bot. J. Linn. Soc. 64(4): 363. 1971.** Sec. Erben (1993)

≡ *Limonium furfuraceum* subsp. *lucentinum* (Pignatti & Freitag) O.Bolòs & Vigo, Fl. Països Catalans 3: 75. 1995 syn. sec. Malekmohammadi & al. (2024)

***Limonium magallufianum* L.Llorens in Lazaroa 8: 47. 1986.** Sec. Domina (2011+)

***Limonium majoricum* Pignatti in Arch. Bot. (Forlì) 31: 89. 1955.** Sec. Domina (2011+)

≡ *Limonium girardianum* subsp. *majoricum* (Pignatti) O.Bolòs, Vigo, Masalles & Ninot, Fl. Man. Països Catalans, ed. 1: 1214. 1990 syn. sec. Domina (2011+)

***Limonium majus* (Boiss.) Erben in Mitt. Bot. Staatssamml. München 14: 553. 1978.** Sec. Domina (2011+)

≡ *Statice globulariifolia* var. *major* Boiss., Voy. Bot. Espagne 2: 531. 1841 [as " *globulariaefolia* "] syn. sec. Erben (1978)
− *Statice globulariaefolia* var. *major* Boiss., Voy. Bot. Espagne 2: 531. 1841, orth. var. syn. sec. Malekmohammadi & al. (2024)

***Limonium malacitanum* B.Díez, Trab. Monogr. 2: 124. 1981.** Sec. Domina (2011+)

***Limonium malfatanicum* Erben in Sendtnera 7: 69. 2001.** Sec. Domina (2011+)

***Limonium mansanetianum* M.B.Crespo & Lledó, Gén. Limonium Comun. Valenciana: 97. 1998.** Sec. Domina (2011+)

***Limonium marisolii* L.Llorens in Lazaroa 8: 51. 1986.** Sec. Domina (2011+)

***Limonium mateoi* Erben & Arán in Anales Jard. Bot. Madrid 62(1): 3. 2005.** Sec. Domina (2011+)

***Limonium mazarae* Pignatti ex Brullo in Bot. Not. 133(3): 283. 1980.** Sec. Domina (2011+)

***Limonium meandrinum* Erben & Brullo in Phytotaxa 240: 170. 2016.** Sec. Domina (2011+)

***Limonium medium* (Ingr.) P.D.Sell, Fl. Gr. Brit. Ireland 1: 691. 2018.** Sec. Sell & Murrell (2018)

≡ *Limonium procerum* var. *medium* Ingr. in Bot. J. Linn. Soc. 92(3): 195. Apr 1986 syn. sec. Sell & Murrell (2018)

***Limonium melancholicum* Brullo, Marcenò & S.Romano in Candollea 51(1): 100. 1996.** Sec. Domina (2011+)

***Limonium melitense* Brullo in Willdenowia 17: 11. 1988.** Sec. Domina (2011+)

***Limonium menigense* Brullo in Mitt. Bot. Staatssamml. München 28: 419. 1989.** Sec. Domina (2011+)

***Limonium merxmuelleri* Erben in Mitt. Bot. Staatssamml. München 16(Suppl.): 1. 1980.** Sec. Domina (2011+)

≡ *Limonium merxmuelleri* subsp. *merxmuelleri* syn. sec. Peruzzi & al. (2015)

***Limonium messeniacum* R.Artelari & Kamari in Bot. Chron. (Patras) 13: 45. 2000.** Sec. Domina (2011+)

***Limonium microcycladicum* Erben & Brullo in Phytotaxa 240: 59. 2016.** Sec. Domina (2011+)

***Limonium migjornense* L.Llorens in Lazaroa 8: 57. 1986.** Sec. Domina (2011+)

≡ *Limonium delicatulum* subsp. *migjornense* (L.Llorens) O.Bolòs, Vigo, Masalles & Ninot, Fl. Man. Països Catalans, ed. 1: 1214. 1990 syn. sec. Domina (2011+)

***Limonium milovicii* Bogdanović & Brullo in Phytotaxa 215(1): 22. 2015.** Sec. Bogdanović & Brullo (2015)

***Limonium minoricense* Erben in Mitt. Bot. Staatssamml. München 28: 368. 1989.** Sec. Domina (2011+)

***Limonium minus* (Boiss.) Erben in Mitt. Bot. Staatssamml. München 27: 382. 1988.** Sec. Domina (2011+)

≡ *Statice globulariifolia* var. *minor* Boiss., Voy. Bot. Espagne 2: 531. 1841 [as " *globulariaefolia* "] syn. sec. Domina (2011+) ≡ *Statice delicatula* var. *minor* (Boiss.) Bég. & A.Vacc., Contr. Fl. Lib. 2: 29. 1912 syn. sec. Malekmohammadi & al. (2024) − *Statice globulariaefolia* var. *minor* Boiss., Voy. Bot. Espagne 2: 531. 1841, orth. var. syn. sec. Malekmohammadi & al. (2024)

***Limonium minutiflorum* (Guss.) Kuntze, Revis. Gen. Pl. 2: 395. 1891.** Sec. Domina (2011+)

≡ *Statice minutiflora* Guss., Fl. Sicul. Prodr. Suppl. 1: 89. 1832 syn. sec. Domina (2011+) ≡ *Statice minutiflora* subsp. *minutiflora* syn. sec. Domina (2011+)
= *Statice minuta* Ten. ex Nyman, Consp. Fl. Eur. 3: 610. 1881 syn. sec. Boissier (1848)

Notes. – *Statice minuta* Ten. is synonym of *Statice minutiflora* Guss. according to Boissier (Prod. 12: 652. 1848)

***Limonium minutum* (L.) Chaz. in Miller, Suppl. Dict. Jard. 2: 35. 1790.** Sec. Domina (2011+)

≡ *Statice minuta* L., Mant. Pl.: 59. 1767 syn. sec. Domina (2011+) ≡ *Taxanthema minuta* (L.) Sweet, Hort. Brit.: 333. 1826 syn. sec. Malekmohammadi & al. (2024) ≡ *Statice virgata* var. *minuta* (L.) Knoche, Fl. Balearica 2: 277. 1922 syn. sec. Erben (1993) − *Limonium minutum* (L.) Fourr. in Ann. Soc. Linn. Lyon, sér. 2, 17: 141. 1869, nom. inval. syn. sec. Malekmohammadi & al. (2024) [is later isonym of *Limonium minutum* (L.) Chaz.] − *Limonium minutum* (L.) Kuntze, Revis. Gen. Pl. 2: 395. 1891, comb. inval. syn. sec. Malekmohammadi & al. (2024)
= *Limonium minutum* subsp. *minutum* syn. sec. Domina (2011+)
= *Statice pubescens* Rchb., Iconogr. Bot. Pl. Crit. 2: 79. 1824 syn. sec. Nyman (1881)
= *Statice minuta* f. *puberula* C.E.Salmon in J. Bot. 53: 242. 1915 syn. sec. Malekmohammadi & al. (2024) ≡ *Limonium minutum* f. *puberulum* C.E.Salmon in J. Bot. 62. 1924: 335 syn. sec. Malekmohammadi & al. (2024)
= *Limonium minutum* var. *medense* O.Bolòs & Vigo, Sist. Nat. Illes Medes: 148. 1984 syn. sec. Domina (2011+)

Notes. – *Statice pubescens* Rchb. is synonym of *Statice minuta* L. according to Nyman (Conspectus florae europaeae 3: 612. 1881)

***Limonium monolithicum* Erben & Brullo in Phytotaxa 240: 69. 2016.** Sec. Domina (2011+)

***Limonium montis-christi* Rizzotto in Webbia 53(2): 269. 1999.** Sec. Domina (2011+)

= *Statice minuta* var. *sommierana* Fiori, Fl. Anal. Ital. 2: 331. 1902 syn. sec. Domina (2011+)

***Limonium morisianum* Arrigoni in Boll. Soc. Sarda Sci. Nat. 17: 177. 1978.** Sec. Domina (2011+)

***Limonium mouterdei* Domina, Erben & Raimondo in Fl. Medit. Fl. Medit. 18: 335. 2008.** Sec. Domina (2011+)

= *Limonium graeca* Post, Fl. Syria, ed. 2, 1: 412-414. 1932 syn. sec. Domina & al. (2008)
= *Limonium graecum* Mouterde, Nouvelle Flore du Liban 3: 14-17. 1978 syn. sec. Domina & al. (2008)

***Limonium mucronulatum* (H.Lindb.) Greuter & Raus in Willdenowia 19(1): 40. 1989.** Sec. Domina (2011+)

≡ *Statice mucronulata* H.Lindb. in Acta Soc. Sci. Fenn., Ser. B, Opera Biol. 2(7): 26. 1946 syn. sec. Greuter & Raus (1989)

***Limonium multiceps* (Pomel) Erben in Fl. Medit. 22: 64. 2012.** Sec. Domina (2011+)

≡ *Statice multiceps* Pomel in Bull. Soc. Sci. Phys. Algérie 11: 128. 1874 syn. sec. Erben & al. (2012) ≡ *Limonium gougetianum* var. *multiceps* (Pomel) Maire in Bull. Soc. Hist. Nat. Afrique N. 29: 434. 1938 syn. sec. Erben & al. (2012) ≡ *Limonium gougetianum* subsp. *multiceps* (Pomel) Quézel & Santa ex Greuter & Raus in Willdenowia 16(2): 450. 1987 syn. sec. Erben & al. (2012)

***Limonium multiflorum* Erben in Mitt. Bot. Staatssamml. München 14: 497. 1978.** Sec. Domina (2011+)

= *Statice densiflora* var. *lusitanica* Daveau in Bol. Soc. Brot. 6: 183. 1888 syn. sec. Erben (1978) ≡ *Limonium dodartii* subsp. *lusitanicum* (Daveau) Franco, Nova Fl. Portugal 2: 564. 1984 syn. sec. Domina (2011+)
= *Limonium binervosum* subsp. *multiflorum* Pignatti, Collect. Bot. (Barcelona) 6(6): 320. 1962, nom. illeg. syn. sec. Erben (1978) ≡ *Limonium auriculae-ursifolium* subsp. *multillorum* (Pignatti) Pignatti in Bot. J. Linn. Soc. 64(4): 367. 1971 syn. sec. Erben (1978)

***Limonium multiforme* (Martelli) Pignatti in Webbia 36(1): 52. 1982.** Sec. Pignatti (1982)

≡ *Statice minuta* var. *multiformis* Martelli, Riv. Sp. Ital. Statice: 13. 1887, nom. illeg. syn. sec. Domina (2011+) − *Limonium multiforme* (Martelli) Pignatti in Bot. J. Linn. Soc. 64(4): 364. 1971, nom. nud. syn. sec. Peruzzi & al. (2015)
= *Limonium argentarium* Pignatti in Webbia 36(1): 50. 1982 syn. sec. Domina (2011+)
= *Limonium herculis* Pignatti in Webbia 36(1): 51. 1982 syn. sec. Domina (2011+)
= *Limonium trojae* Pignatti in Webbia 36(1): 53. 1982 syn. sec. Domina (2011+)

Notes. – Listed as a species in the *Limonium cosyrense* group (Pignatti, 1972).

***Limonium multifurcatum* Erben in Sendtnera 7: 61. 2001.** Sec. Domina (2011+)

***Limonium muradense* Erben in Mitt. Bot. Staatssamml. München 30: 648. 1991.** Sec. Domina (2011+)

***Limonium mutabile* (Ingr.) P.D.Sell, Fl. Gr. Brit. Ireland 1: 691. 2018.** Sec. Sell & Murrell (2018)

≡ *Limonium paradoxum* var. *mutabile* Ingr. in Bot. J. Linn. Soc. 92(3): 192. Apr 1986 syn. sec. Sell & Murrell (2018)

***Limonium mutatum* (Ingr.) P.D.Sell, Fl. Gr. Brit. Ireland 1: 690. 2018.** Sec. Sell & Murrell (2018)

≡ *Limonium binervosum* subsp. *mutatum* Ingr. in Bot. J. Linn. Soc. 92(3): 189. Apr 1986 syn. sec. Sell & Murrell (2018)

***Limonium naniforme* P.D.Sell, Fl. Gr. Brit. Ireland 1: 691. 2018.** Sec. Sell & Murrell (2018)

***Limonium neapolense* Brullo in Mitt. Bot. Staatssamml. München 28: 419. 1989.** Sec. Domina (2011+)

***Limonium ×neumanii* C.E.Salmon in J. Bot. 42: 361. 1904.** Sec. Stace (1975)

= *Statice ×neumani* Rouy, Ill. Pl. Eur.: t. 497. 1905 syn. sec. Stace (1975)

***Limonium normannicum* Ingr. in Watsonia 15(3): 223. 1985.** Sec. Domina (2011+)

= *Statice lychnidifolia* var. *corymbosa* Boiss., Prodr. 12: 647. 1848 syn. sec. Domina (2011+)
= *Limonium lychnidifolium* var. *corymbosum* (Boiss.) C.E.Salmon in J. Bot. 39: 192-195. 1901 syn. sec. Ingrouille, M.J. (1985)

***Limonium nymphaeum* Erben in Mitt. Bot. Staatssamml. München 17: 494. 1981.** Sec. Peruzzi & al. (2015)

≡ *Limonium acutifolium* subsp. *nymphaeum* (Erben) Arrigoni in Parlatorea 7: 19. 2005 syn. sec. Peruzzi & al. (2015)

***Limonium obesifolium* P.D.Sell, Fl. Gr. Brit. Ireland 1: 689. 2018.** Sec. Sell & Murrell (2018)

***Limonium oblanceolatum* Brullo in Mitt. Bot. Staatssamml. München 28: 419. 1989.** Sec. Domina (2011+)

***Limonium obtusifolium* (Rouy) Erben in Mitt. Bot. Staatssamml. München 14: 449. 1978.** Sec. Domina (2011+)

≡ *Statice acutifolia* var. *obtusifolia* Rouy, Fl. France 10: 149. 1908 syn. sec. Erben (1978) ≡ *Limonium acutifolium* var. *obtusifolium* (Rouy) C.E.Salmon in J. Bot. 62. 1924: 336 syn. sec. C.E.Salmon in J. Bot. 62. 1924 (1924) ≡ *Limonium acutifolium* subsp. *obtusifolium* (Rouy) Diana in Boll. Soc. Sarda Sci. Nat. 17: 280. 1978 syn. sec. Domina (2011+)

***Limonium ocymifolium* (Poir.) Kuntze, Revis. Gen. Pl. 2: 396. 1891.** Sec. Domina (2011+)

≡ *Statice ocymifolia* Poir., Encycl. Suppl. 5: 238. 1817 syn. sec. Kuntze (1891) ≡ *Statice ocymifolia* subsp. *ocymifolia* syn. sec. Domina (2011+)

***Limonium oligotrichum* Erben & Brullo in Phytotaxa 240: 65. 2016.** Sec. Domina (2011+)

***Limonium omissae* Bogdanović & Brullo in Phytotaxa 215(1): 22. 2015.** Sec. Bogdanović & Brullo (2015)

***Limonium optimae* Raimondo in Fl. Medit. 3: 13. 1993.** Sec. Domina (2011+)

***Limonium opulentum* (Lojac.) Brullo in Giorn. Bot. Ital. n.s., 114: 45. 1980.** Sec. Domina (2011+)

≡ *Statice opulenta* Lojac., Fl. Sicul. 2(2): 23. 1907 syn. sec. Brullo (1980) ≡ *Limonium oleifolium* subsp. *opulentum* (Lojac.) Brullo in Bot. Not. 133(3): 289. 1980 syn. sec. Domina (2011+) ≡ *Limonium opulentum* (Lojac.) Greuter in Willdenowia 16(2): 450. 1987 syn. sec. Domina (2011+)

***Limonium orellii* Erben in Mitt. Bot. Staatssamml. München 30: 462. 1991.** Sec. Domina (2011+)

***Limonium oristanum* Alf.Mayer, Libri Botanici 15: 68. 1995.** Sec. Malekmohammadi & al. (2024)

≡ *Limonium merxmuelleri* subsp. *oristanum* (Alf.Mayer) Arrigoni in Parlatorea 7: 19. 2005 syn. sec. Malekmohammadi & al. (2024: Priliminary revision, 2022)

***Limonium ovalifolium* (Poir.) Kuntze, Revis. Gen. Pl. 2: 396. 1891.** Sec. Domina (2011+)

≡ *Statice ovalifolia* Poir., Encycl. Suppl. 5: 237. 1817 syn. sec. Domina (2011+) ≡ *Limonium ovalifolium* subsp. *ovalifolium* syn. sec. Domina (2011+)
= *Statice hybrida* Mont. ex J.Lloyd, Fl. Loire-Inf.: 211. 1844 syn. sec. Malekmohammadi & al. (2024)
= *Statice mucosa* Salzm. ex Boiss. in Candolle, Prodr. 12: 646. 1848 syn. sec. Malekmohammadi & al. (2024)
= *Statice ovalifolia* var. *minor* Boiss. in Candolle, Prodr. 12: 646. 1848 syn. sec. Malekmohammadi & al. (2024)
= *Statice ovalifolia* var. *major* Rouy in Rev. Bot. Syst. Geogr. Bot. 1(11): 163. 1903 syn. sec. Malekmohammadi & al. (2024)
= *Statice ovalifolia* var. *nana* Rouy in Rev. Bot. Syst. Geogr. Bot. 1(11): 163. 1903 syn. sec. Malekmohammadi & al. (2024)
= *Statice ovalifolia* var. *normalis* Rouy, Fl. France 10: 159. 1908 syn. sec. Malekmohammadi & al. (2024)
= *Statice ovalifolia* var. *paniculata* Rouy, Fl. France 10: 159. 1908 syn. sec. Malekmohammadi & al. (2024)
= *Limonium ovalifolium* subsp. *lusitanicum* Pignatti, Collect. Bot. (Barcelona) 6(6): 318. 1962 syn. sec. Domina (2011+)

Notes. – *Statice hybrida* Mont. ex J.Lloyd is synonym of *Statice ovalifolia* var. *minor* Boiss. according to Boissier (1848)

***Limonium pachynense* Brullo in Bot. Not. 133(3): 292. 1980.** Sec. Domina (2011+)

***Limonium palmare* (Sm.) Rech.f. in Denkschr. Akad. Wiss. Wien, Math.-Naturwiss. Kl. 105 (2,1): 105. 1943.** Sec. Domina (2011+)

≡ *Statice palmaris* Sm., Fl. Graec. 3: 91. 1821 syn. sec. Rechinger (1943) ≡ *Statice graeca* var. *palmaris* (Sm.) Hay, Prodr. Fl. Pen. Balc. 2: 8. 1931 syn. sec. Rechinger (1943) ≡ *Limonium graecum* var. *palmare* (Sm.) Rech.f. in Denkschr. Akad. Wiss. Wien, Math.-Naturwiss. Kl. 105(1): 427. 1943 syn. sec. Rechinger (1943)

***Limonium pandatariae* Pignatti in Webbia 36(1): 54. 1982.** Sec. Domina (2011+)

≡ *Limonium pontium* subsp. *pandatariae* (Pignatti) Iamonico, Iberite, De Castro & Nicolella in Plants 11(22), 3163: 16. 2022 syn. sec. Malekmohammadi & al. (2024: 23 Nov. 2022)

Notes. – Listed as a species in the Limonium albidum group (Pignatti, 1972).

***Limonium panormitanum* (Tod.) Pignatti in Bot. J. Linn. Soc. 64(4): 365. 1971.** Sec. Domina (2011+)

≡ *Statice panormitana* Tod., Index Seminum (PAL) 1857: 45. 1857 syn. sec. Pignatti (1971) ≡ *Statice minutiflora* subsp. *panormitana* (Tod.) Nyman, Consp. Fl. Eur. 3: 611. 1881 syn. sec. Domina (2011+)

***Limonium paradoxum* Pugsley in J. Bot. 69: 46. 1931.** Sec. Ingrouille, M.J. & Stace (1986)

Notes. – Listed as a species in the *Limonium binervosum* aggregate by Ingrouille and Stace (1986) and in the *L. binervosum* group by Pignatti (1972).

***Limonium paralimniticum* Christodoulou, Erben, Hand & Kefalas in Fl. Medit. 32: 37. 2022.** Sec. Erben & al. (2022)

***Limonium paramedium* (Ingr.) P.D.Sell, Fl. Gr. Brit. Ireland 1: 691. 2018.** Sec. Sell & Murrell (2018)

≡ *Limonium procerum* var. *paramedium* Ingr. in Bot. J. Linn. Soc. 92(3): 197. Apr 1986 syn. sec. Sell & Murrell (2018)

***Limonium parosicum* Erben & Brullo in Phytotaxa 240: 119. 2016.** Sec. Domina (2011+)

***Limonium parvibracteatum* Pignatti in Bot. J. Linn. Soc. 64(4): 367. 1971.** Sec. Domina (2011+)

= *Limonium supinum* Erben in Mitt. Bot. Staatssamml. München 14: 546. 1978 syn. sec. Erben (1993)

***Limonium parvifolium* (Tineo) Pignatti in Bot. J. Linn. Soc. 64(4): 364. 1971.** Sec. Domina (2011+)

≡ *Statice parvifolia* Tineo, Fl. Sicul. Syn. 2: 806. 1845 syn. sec. Pignatti (1971) ≡ *Statice tineoi* var. *parvifolia* (Tineo) J.Woods, Tourist's Fl.: 306. 1850 syn. sec. Malekmohammadi & al. (2024)

***Limonium parvum* Ingr. in Bot. J. Linn. Soc. 92(3): 204. Apr 1986.** Sec. Ingrouille, M.J. & Stace (1986)

Notes. – Listed as a species in the Limonium binervosum aggregate (Ingrouille and Stace 1986).

***Limonium patrimoniense* Arrigoni & Diana in Candollea 48(2): 663. 1993.** Sec. Domina (2011+)

***Limonium paui* Cámara & Sennen, Diagn. Nouv.: 269. 1936.** Sec. Domina (2011+)

≡ *Statice paui* Cámara & Sennen, Diagn. Nouv.: 269. 1936 syn. sec. Malekmohammadi & al. (2024)

***Limonium pavonianum* Brullo in Willdenowia 17: 13. 1988.** Sec. Erben & al. (2018)

= *Statice densiflora* var. *humilis* Guss., Fl. Sicul. Syn. 1: 368. 1843 syn. sec. Erben & al. (2018)

***Limonium pedicellatum* (Wallr. ex Boiss.) Kuntze, Revis. Gen. Pl. 2: 396. 1891.** Sec. Malekmohammadi & al. (2024)

≡ *Statice pedicellata* Wallr. ex Boiss. in Candolle, Prodr. 12: 658. 1848 syn. sec. Kuntze (1891)
= *Statice linifolia* Drege ex Boiss. in Candolle, Prodr. 12: 658. 1848 syn. sec. Malekmohammadi & al. (2024)

Notes. – *Limonium pedicellatum* is accepted based on personal comnunication with Ladislav Mucina.; According to Mucina (pers. comm.), *Statice linifolia* sensu Drege ex Boiss. is a synonym of *Limonium pedicellatum*, which is clearly distinct from both *L. scabrum* and *L. dregeanum*.

***Limonium pelagosae* Bogdanović & Brullo in Phytotaxa 215(1): 23. 2015.** Sec. Bogdanović & Brullo (2015)

***Limonium penicillatum* Adamson in J. S. African Bot. 7: 202. 1941.** Sec. Adamson & al. (1941)

***Limonium pericotii* (O.Bolòs & Vigo) Greuter & Raus in Willdenowia 19(1): 40. 1989.** Sec. Domina (2011+)

≡ *Limonium minutum* subsp. *pericotii* O.Bolòs & Vigo in J. Ros et al., Sist. Nat. Illes Medes: 148. 1984 syn. sec. Domina (2011+)

***Limonium perplexum* L.Sáez & Rosselló in Anales Jard. Bot. Madrid 57(1): 48. 1999.** Sec. Malekmohammadi & al. (2024)

***Limonium pescadense* Greuter & Raus in Willdenowia 16(2): 450. 1987.** Sec. Domina (2011+)

− *Statice psiloclada* var. *genuina* Boiss. in Candolle, Prodr. 12: 651. 1848, nom. inval. syn. sec. Greuter & Raus (1987)

***Limonium peucetium* Pignatti in Webbia 36(1): 53. 1982.** Sec. Domina (2011+)

Notes. – Possibly extinct (Rossi et al., 2013).

***Limonium pharense* (Ingr.) P.D.Sell, Fl. Gr. Brit. Ireland 1: 691. 2018.** Sec. Sell & Murrell (2018)

≡ *Limonium britannicum* var. *pharense* Ingr. in Bot. J. Linn. Soc. 92(3): 203. Apr 1986 syn. sec. Sell & Murrell (2018)

***Limonium pharosianum* Bogdanović & Brullo in Phytotaxa 215(1): 24. 2015.** Sec. Bogdanović & Brullo (2015)

***Limonium phitosianum* R.Artelari in Mitt. Bot. Staatssamml. München 20: 430. 1984.** Sec. Domina (2011+)

***Limonium pigadiense* (Rech.f.) Rech.f. in Denkschr. Akad. Wiss. Wien, Math.-Naturwiss. Kl. 105(1): 427. 1943.** Sec. Domina (2011+)

≡ *Statice pigadiensis* Rech.f. in Repert. Spec. Nov. Regni Veg. 43: 147. 1938 syn. sec. Malekmohammadi & al. (2024)

***Limonium pinillense* Roselló, Stübing, Peris J.B. & Cirujano in Anales Jard. Bot. Madrid 55(2): 475. 1997.** Sec. Malekmohammadi & al. (2024)

***Limonium planesiae* Pignatti in Webbia 36(1): 52. 1982.** Sec. Domina (2011+)

***Limonium plurisquamatum* Erben in Mitt. Bot. Staatssamml. München 14: 480. 1978.** Sec. Erben (1993)

= *Limonium auriculae-ursifolium* subsp. *lusitanicum* (Pignatti) Pignatti in Bot. J. Linn. Soc. 64(4): 367. 1971 syn. sec. Erben (1993)

***Limonium poimenum* Ilardi, Brullo, D.Cusimano & G.Giusso in Phytotaxa 188(5): 268. 2014.** Sec. Peruzzi & al. (2015)

Limonium pomelianum **(Rouy) Erben in Fl. Medit. 22: 64. 2012.** Sec. Domina (2011+)

≡ *Statice pomeliana* Rouy in Rev. Bot. Syst. Geogr. Bot. 1(11): 155. 1903 syn. sec. Malekmohammadi & al. (2024) ≡ *Statice delicatula* f. *pomeliana* Rouy in Rev. Bot. Syst. Geogr. Bot. 1(11): 155. 1903 syn. sec. Domina (2011+)
= *Statice leptostachys* Pomel, Nouv. Mat. Fl. Atl.: 311. 1875 syn. sec. Domina (2011+) ≡ *Limonium delicatulum* var. *leptostachys* (Pomel) Maire in Jahandiez & al., Cat. Pl. Maroc 3: 570. 1934 syn. sec. Malekmohammadi & al. (2024)

Limonium pomoense **Bogdanović & Brullo in Phytotaxa 215(1): 24. 2015.** Sec. Bogdanović & Brullo (2015)

Limonium pontium **Pignatti in Bot. J. Linn. Soc. 64(4): 364. 1971.** Sec. Iamonico & al. (2022)

≡ *Limonium pontium* subsp. *pontium* syn. sec. Malekmohammadi & al. (2024: 23 Nov. 2022)

Notes. – Listed as a species in the *Limonium cosyrense* group (Pignatti, 1972).

Limonium ponzoi **(Fiori & Bég.) Brullo in Bot. Not. 133(3): 284. 1980.** Sec. Domina (2011+)

≡ *Statice ambigua* var. *ponzoi* Fiori & Bég., Sched. Fl. It. 3, 29-30: 384. 1927 syn. sec. Brullo (1980)

Limonium portlandicum **(Ingr.) P.D.Sell, Fl. Gr. Brit. Ireland 1: 689. 2018.** Sec. Sell & Murrell (2018)

≡ *Limonium recurvum* subsp. *portlandicum* Ingr. in Bot. J. Linn. Soc. 92(3): 211. Apr 1986 syn. sec. Sell & Murrell (2018) ≡ *Limonium recurvum* var. *portlandicum* Ingr. in Bot. J. Linn. Soc. 92(3): 212. Apr 1986 syn. sec. Malekmohammadi & al. (2024)

Limonium portopetranum **Erben in Mitt. Bot. Staatssamml. München 30: 471. 1991.** Sec. Domina (2011+)

Limonium portovecchiense **Erben in Sendtnera 7: 53. 2001.** Sec. Domina (2011+)

Limonium postii **Domina, Erben & Raimondo in Fl. Medit. Fl. Medit. 18: 333. 2008.** Sec. Domina (2011+)

Limonium procerum **(C.E.Salmon) Ingr. in Bot. J. Linn. Soc. 92(3): 193. Apr 1986.** Sec. Domina (2011+)

≡ *Limonium occidentale* var. *procerum* C.E.Salmon in J. Bot. 41: 72. 1903 syn. sec. Ingrouille, M.J. & Stace (1986)

Notes. – Listed as a species in the *Limonium binervosum* aggregate (Ingrouille and Stace 1986).

Limonium proliferum **(d'Urv.) Erben & Brullo in Phytotaxa 240: 135. 2016.** Sec. Domina (2011+)

≡ *Statice prolifera* d'Urv. in Mém. Soc. Linn. Paris 1: 291. 1822 syn. sec. Brullo (2016);

Limonium protohermaeum **Arrigoni & Diana in Boll. Soc. Sarda Sci. Nat. 24: 277. 1985.** Sec. Domina (2011+)

Limonium pseudarticulatum **Erben in Mitt. Bot. Staatssamml. München 28: 313. 1989.** Sec. Domina (2011+)

≡ *Limonium articulatum* subsp. *pseudarticulatum* (Erben) O.Bolòs & Vigo, Fl. Països Catalans 3: 94. 1995 syn. sec. Malekmohammadi & al. (2024)

Limonium pseudebusitanum **Erben in Mitt. Bot. Staatssamml. München 28: 313. 1989.** Sec. Domina (2011+)

≡ *Limonium minutum* var. *pseudebusitanum* (Erben) O.Bolòs & Vigo, Fl. Països Catalans 3: 94. 1995 syn. sec. Malekmohammadi & al. (2024)

Limonium ×pseudoconfusum **(Rouy) P.Fourn., Quatre Fl. France: 722. 1937.** Sec. Fournier (1937)

≡ *Statice ×pseudoconfusa* Rouy in Rev. Bot. Syst. Geogr. Bot. 1(12): 182. 1904 syn. sec. Fournier (1937)
= *Statice ×girardianoides* H.Lév. in Bull. Acad. Int. Geogr. Bot. 22: 55. 1917 syn. sec. Malekmohammadi & al. (2024)

Limonium pseudodictyocladum **(Pignatti) L.Llorens in Lazaroa 8: 60. 1986 ["1985"].** Sec. Erben (1993)

≡ *Limonium virgatum* subsp. *pseudodictyocladum* (Pignatti) O.Bolòs, Vigo, Masalles & Ninot, Fl. Man. Països Catalans, ed. 1: 1214. 1990 syn. sec. Malekmohammadi & al. (2024) − *Limonium virgatum* subsp. *pseudodictyocladum* Pignatti in Nuovo Giorn. Bot. Ital. n.s. 66: 562. 1959, nom. inval. syn. sec. Greuter & Raus (1987) − *Limonium oleifolium* subsp. *pseudodictyocladum* (Pignatti) Pignatti in Bot. J. Linn. Soc. 64(4): 366. 1971, nom. inval. syn. sec. Greuter & Raus (1987)
− *Limonium pseudodictyocladum* Greuter & Raus in Willdenowia 16(2): 450. 1987, nom. inval. syn. sec. Malekmohammadi & al. (2024)

Limonium ×pseudodivaricatum **Pignatti in Arch. Bot. (Forlì) 31: 100. 1955.** Sec. Pignatti (1955)

Notes. – Without latin description

Limonium pseudolaetum **Arrigoni & Diana in Boll. Soc. Sarda Sci. Nat. 27: 267. 1990.** Sec. Domina (2011+)

Limonium pseudominutum **Erben in Mitt. Bot. Staatssamml. München 27: 397. 1988.** Sec. Domina (2011+)

≡ *Limonium minutum* subsp. *pseudominutum* (Erben) O.Bolòs & Vigo in Fl. Països Catalans 3: 77. 1995 syn. sec. Malekmohammadi & al. (2024)
= *Statice minuta* var. *microphylla* Boiss. in Candolle, Prodr. 12: 655. 1848 syn. sec. Erben (1988)
= *Limonium minutum* var. *microphyllum* C.E.Salmon in J. Bot. 62. 1924: 335 syn. sec. Malekmohammadi & al. (2024)

Notes. – *Statice minuta* var. *microphylla* Boiss. is synonym of *Limonium minutum* var. *microphyllum* according to Salmon (J. Bot. 62: 335. 1924)

Limonium pseudoparadoxum **(Ingr.) P.D.Sell, Fl. Gr. Brit. Ireland 1: 691. 2018.** Sec. Sell & Murrell (2018)

≡ *Limonium recurvum* var. *pseudoparadoxum* Ingr. in Bot. J. Linn. Soc. 92(3): 214. Apr 1986 syn. sec. Sell & Murrell (2018)

Limonium ×pseudosmithii **Pignatti in Arch. Bot. (Forlì) 31: 97. 1955.** Sec. Pignatti (1955)

Notes. – Without Latin description

Limonium pseudotranswallianum **(Ingr.) P.D.Sell, Fl. Gr. Brit. Ireland 1: 691. 2018.** Sec. Sell & Murrell (2018)

≡ *Limonium recurvum* subsp. *pseudotranswallianum* Ingr. in Bot. J. Linn. Soc. 92(3): 212. Apr 1986 syn. sec. Sell & Murrell (2018)

***Limonium pujosii* Sauvage & Vindt in Compt.-Rend. Séances Soc. Sci. Nat. Maroc 19: 115. 1953.** Sec. Domina (2011+)

***Limonium pulviniforme* Arrigoni & Diana in Boll. Soc. Sarda Sci. Nat. 25: 173. 1986.** Sec. Domina (2011+)

***Limonium pusillum* Erben & Brullo in Phytotaxa 240: 116. 2016.** Sec. Domina (2011+)

***Limonium pylium* R.Artelari, Biosyst. Melet. Gen. Limonium: 37. 1984.** Sec. Domina (2011+)

***Limonium pyramidatum* Brullo in Mitt. Bot. Staatssamml. München 28: 419. 1989.** Sec. Domina (2011+)

***Limonium quesadense* Erben in Mitt. Bot. Staatssamml. München 27: 390. 1988.** Sec. Domina (2011+)

***Limonium quinnii* M.B.Crespo & Pena-Martín in Phytotaxa 94(2): 31. 2013.** Sec. Domina (2011+)

− *Limonium ladikanum* Erben & Brullo, nom. nud. syn. sec. Malekmohammadi & al. (2024)

***Limonium racemosum* (Lojac.) Diana in Boll. Soc. Sarda Sci. Nat. 17: 272. 1978.** Sec. Domina (2011+)

≡ *Statice racemosa* Lojac. in Boll. Reale Orto Bot. Palermo 5: 99. 1906 syn. sec. Malekmohammadi & al. (2024)

***Limonium raddianum* (Boiss.) Pignatti ex Brullo in Webbia 33(1): 153. 1978.** Sec. Domina (2011+)

≡ *Statice raddiana* Boiss. in Candolle, Prodr. 12: 653. 1848 syn. sec. Brullo (1978)
– *Statice globulariifolia* sensu auct., non Desf., err. sec. Domina (2011+)

***Limonium ramosissimum* (Poir.) Maire in Bull. Soc. Hist. Nat. Afrique N. 27: 244. 1936.** Sec. Domina (2011+)

≡ *Statice ramosissima* Poir., Voy. Barbarie 2: 142. 1789 syn. sec. Malekmohammadi & al. (2024)
= *Statice globulariifolia* Desf., Fl. Atlant. 1: 274. 1798 [as " *globulariaefolia* "] syn. sec. Domina (2011+) ≡ *Limonium globulariifolium* (Desf.) Kuntze, Revis. Gen. Pl. 2: 395. 1891 syn. sec. Domina (2011+) ≡ *Limonium globulariifolium* (Desf.) Kuntze subsp. *globulariifolium* syn. sec. Domina (2011+) − *Statice globulariaefolia* Desf., Fl. Atlant. 1: 274. 1798, orth. var. syn. sec. Malekmohammadi & al. (2024)
= *Taxanthema globulariifolia* Sweet, Hort. Brit.: 332. 1826 syn. sec. Malekmohammadi & al. (2024)
= *Statice laxissima* Rouy, Illustr. Pl. Eur. Rar.: t. 296. 1899 syn. sec. Domina (2011+)

***Limonium ramosissimum* subsp. *provinciale* (Pignatti) Pignatti in Bot. J. Linn. Soc. 64(4): 366. 1971.** Sec. Domina (2011+)

≡ *Limonium globulariifolium* subsp. *provinciale* Pignatti, Collect. Bot. (Barcelona) 6(6): 326. 1962 syn. sec. Domina (2011+)
= *Statice bellidifolia* Bertol., Fl. Ital. 3: 524. 1837 syn. sec. Boissier (1848)
= *Statice psiloclada* Boiss. in Candolle, Prodr. 12: 651. 1848 syn. sec. Domina (2011+) ≡ *Limonium psilocladum* (Boiss.) Kuntze, Revis. Gen. Pl. 2: 396. 1891 syn. sec. Malekmohammadi & al. (2024) ≡ *Limonium confusum* subsp. *psilocladum* (Boiss.) P.Fourn. syn. sec. Domina (2011+)
= *Statice delicatula* subsp. *raddiana* (Boiss.) Rouy in Rev. Bot. Syst. Geogr. Bot. 1(11): 155. 1903 syn. sec. Domina (2011+) ≡ *Limonium confusum* subsp. *raddianum* (Boiss.) P.Fourn. syn. sec. Domina (2011+)

Notes. – *Statice bellidifolia* Bertol. is synonym of *Statice psiloclada* Boissi. according to Boissier (Prod. 12: 651. 1848)

***Limonium ramosissimum* subsp. *ramosissimum*.** Sec. Domina (2011+)

***Limonium recticaule* Erben & Brullo in Phytotaxa 240: 144. 2016.** Sec. Domina (2011+)

***Limonium recurviforme* (Ingr.) P.D.Sell, Fl. Gr. Brit. Ireland 1: 689. 2018.** Sec. Sell & Murrell (2018)

≡ *Limonium recurvum* var. *recurviforme* Ingr. in Bot. J. Linn. Soc. 92(3): 212. Apr 1986 syn. sec. Sell & Murrell (2018)

***Limonium recurvum* C.E.Salmon in J. Bot. 41: 67. 1903.** Sec. Domina (2011+)

≡ *Statice recurva* (C.E.Salmon) C.E.Salmon in J. Bot. 51: 95. 1913 syn. sec. Ingrouille, M.J. & Stace (1986)

Notes. – Listed as a species in the *Limonium binervosum* aggregate (Ingrouille and Stace 1986) and *L. binervosum* group (Pignatti, 1972).

***Limonium recurvum* subsp. *crigyllensis* I.Rees in Brit. Irish Bot. 4(1): 4. 2021**

***Limonium recurvum* subsp. *humile* (Girard) Ingr. in Bot. J. Linn. Soc. 92(3): 213. Apr 1986.** Sec. Ingrouille, M.J. & Stace (1986)

≡ *Statice dodartii* var. *humile* Girard in Ann. Sci. Nat., Bot., sér. 2, 17: 33. 1842 syn. sec. Malekmohammadi & al. (2024) ≡ *Limonium recurvum* var. *humile* (Girard) Ingr. in Bot. J. Linn. Soc. 92(3): 213. Apr 1986 syn. sec. Malekmohammadi & al. (2024)

***Limonium recurvum* subsp. *recurvum*.** Sec. Domina (2011+)

***Limonium remotispiculum* (Lacaita) Pignatti in Bot. J. Linn. Soc. 64(4): 365. 1971.** Sec. Domina (2011+)

≡ *Statice remotispicula* Lacaita in Nuovo Giorn. Bot. Ital. 16: 168. 1884 syn. sec. Pignatti (1971) ≡ *Statice minuta* var. *remotispicula* (Lacaita) Fiori, Fl. Italia 2: 331. 1902 syn. sec. Valariello & al. (2016)

***Limonium reticulatum* (L.) Mill., Gard. Dict., ed. 8: no. 9. 1768.** Sec. Malekmohammadi & al. (2024)

= *Statice reticulata* L., Sp. Pl.: 275. 1753 syn. sec. Schultes (1820); ≡ *Taxanthema reticulata* (L.) Sweet, Hort. Brit.: 333. 1826 syn. sec. Malekmohammadi & al. (2024)

***Limonium retirameum* Greuter & Raus in Willdenowia 16(2): 451. 1987.** Sec. Domina (2011+)

≡ *Limonium retirameum* subsp. *retirameum* syn. sec. Malekmohammadi & al. (2024)
= *Statice dictyoclada* var. *reticulata* Boiss. in Candolle, Prodr. 12: 654. 1848 syn. sec. Peruzzi & al. (2015)

***Limonium retusum* L.Llorens in Lazaroa 8: 80. 1986.** Sec. Domina (2011+)

≡ *Limonium delicatulum* subsp. *retusum* (L.Llorens) O.Bolòs, Vigo, Masalles & Ninot, Fl. Man. Països Catalans, ed. 1: 1214. 1990 syn. sec. Domina (2011+)

***Limonium revolutum* Erben in Mitt. Bot. Staatssamml. München 14: 451. 1978.** Sec. Domina (2011+)

≡ *Limonium minutum* subsp. *revolutum* (Erben) O.Bolòs & Vigo, Sist. Nat. Illes Medes: 148. 1984 syn. sec. Domina (2011+)
= *Limonium emporitanum* Fern.Casas & Molero in Fontqueria 3: 19. 1983 syn. sec. Domina (2011+)

***Limonium rigualii* M.B.Crespo & Erben in Mitt. Bot. Staatssamml. München 30: 459. 1991.** Sec. Domina (2011+)

***Limonium roridum* (Sibth. & Sm.) Brullo & Guarino in Flora Medit. 10: 267. 2000.** Sec. Domina (2011+)

≡ *Statice rorida* Sibth. & Sm., Fl. Graec. 3: 91. 1821 syn. sec. Brullo & Guarino (2000)
= *Statice hyssopifolia* Girard in Ann. Sci. Nat., Bot., ser. 3, 2: 329. 1844 syn. sec. Brullo (2016) ≡ *Statice graeca* var. *hyssopifolia* (Girard) Boiss. in Candolle, Prodr. 12: 650. 1848 syn. sec. Malekmohammadi & al. (2024) ≡ *Limonium hyssopifolium* (Girard) Rech.f. in Denkschr. Akad. Wiss. Wien, Math.-Naturwiss. Kl. 105(1): 427. 1943 syn. sec. Brullo (2016) ≡ *Limonium graecum* var. *hyssopifolium* (Girard) Bokhari in Notes Roy. Bot. Gard. Edinburgh 32(1): 62. 1972 syn. sec. Brullo (2016)
= *Statice oliveriana* Andrz. ex Boiss. in Candolle, Prodr. 12: 650. 1848 syn. sec. POWO (2017+)
= *Statice spathulata* Heldr. ex Nyman, Consp. Fl. Eur. 3: 611. 1881 syn. sec. Malekmohammadi & al. (2024)

***Limonium rosselloi* Ferrer-Gallego, P. P., R.Roselló & E.Laguna in Collect. Bot. (Barcelona) 32: 34. 2013.** Sec. Malekmohammadi & al. (2024)

***Limonium rubescens* Brullo in Mitt. Bot. Staatssamml. München 28: 419. 1989.** Sec. Domina (2011+)

***Limonium ruizii* (Font Quer) Fern.Casas in Candollea 29: 330. 1974.** Sec. Domina (2011+)

≡ *Statice ruizii* Font Quer, Butl. Inst. Catalana Hist. Nat. 33: 111. 1933 syn. sec. Domina (2011+) ≡ *Limonium aragonense* subsp. *ruizii* (Font Quer) Fern.Casas & Muñoz Garm. in Garmendia, Exsicc. 1: 8. 1978 syn. sec. Domina (2011+)

***Limonium rungsii* Sauvage & Vindt in Compt.-Rend. Séances Soc. Sci. Nat. Maroc 16: 51. 1950.** Sec. Domina (2011+)

***Limonium sabulicola* P.D.Sell, Fl. Gr. Brit. Ireland 1: 687. 2018.** Sec. Sell & Murrell (2018)

***Limonium salmonis* (Sennen & Elías) Pignatti, Collect. Bot. (Barcelona) 6(6): 321. 1962.** Sec. Pignatti (1972)

≡ *Statice salmonis* Sennen & Elías in Bol. Soc. Iber. Ci. Nat. 35(1-2): 30. 1936 syn. sec. Pignatti (1962)

Notes. – Listed as a species in the *Limonium binervosum* group (Pignatti, 1972).

***Limonium samium* Erben & Brullo in Phytotaxa 240: 75. 2016.** Sec. Domina (2011+)

***Limonium sanctamargaritense* P.D.Sell, Fl. Gr. Brit. Ireland 1: 688. 2018.** Sec. Sell & Murrell (2018)

***Limonium santapolense* Erben in Mitt. Bot. Staatssamml. München 27: 392. 1988.** Sec. Domina (2011+)

≡ *Limonium delicatulum* subsp. *santapolense* (Erben) O.Bolòs, Vigo, Masalles & Ninot, Fl. Man. Països Catalans, ed. 1: 1214. 1990 syn. sec. Domina (2011+)

***Limonium saracinatum* R.Artelari, Biosyst. Melet. Gen. Limonium: 42. 1984.** Sec. Domina (2011+)

***Limonium sardoum* (Pignatti) Erben in Mitt. Bot. Staatssamml. München 16(Suppl.): 561. 1980.** Sec. Malekmohammadi & al. (2024)

≡ *Limonium virgatum* subsp. *sardoum* Pignatti in Nuovo Giorn. Bot. Ital. n.s. 66: 562. 1959 syn. sec. Peruzzi & al. (2015) ≡ *Limonium oleifolium* subsp. *sardoum* (Pignatti) Pignatti in Bot. J. Linn. Soc. 64(4): 366. 1971 syn. sec. Peruzzi & al. (2015)
= *Limonium caralitanum* Erben in Sendtnera 7: 57. 2001, nom. illeg. syn. sec. Peruzzi & al. (2015) ≡ *Limonium retirameum* subsp. *caralitanum* (Erben) Arrigoni in Parlatorea 7: 19. 2005 syn. sec. Peruzzi & al. (2015)

***Limonium sarniense* (Ingr.) P.D.Sell, Fl. Gr. Brit. Ireland 1: 690. 2018.** Sec. Sell & Murrell (2018)

≡ *Limonium binervosum* var. *sarniense* Ingr. in Bot. J. Linn. Soc. 92(3): 190. Apr 1986 syn. sec. Sell & Murrell (2018) ≡ *Limonium binervosum* subsp. *sarniense* Ingr. in Bot. J. Linn. Soc. 92(3): 190. Apr 1986 syn. sec. Sell & Murrell (2018)

***Limonium sartorianum* Erben & Brullo in Phytotaxa 240: 110. 2016.** Sec. Domina (2011+)

***Limonium saxicola* Erben in Mitt. Bot. Staatssamml. München 28: 402. 1989.** Sec. Domina (2011+)

***Limonium saxonicum* (Ingr.) P.D.Sell, Fl. Gr. Brit. Ireland 1: 687. 2018.** Sec. Sell & Murrell (2018)

≡ *Limonium binervosum* subsp. *saxonicum* Ingr. in Bot. J. Linn. Soc. 92(3): 189. Apr 1986 syn. sec. Sell & Murrell (2018)

***Limonium scabrum* (Thunb.) Kuntze, Revis. Gen. Pl. 2: 396. 1891.** Sec. Bredenkamp (2003)

≡ *Statice scabra* Thunb., Prodr. Pl. Cap. 1: 54. 1794 syn. sec. Kuntze (1891) ≡ *Taxanthema scabra* (Thunb.) Sweet, Hort. Brit.: 332. 1826 syn. sec. Bredenkamp (2003)
= *Statice cinerea* Poir., Encycl. 7: 407. 1806 syn. sec. Malekmohammadi & al. (2024)
= *Statice scabra* Drege ex Boiss. in Candolle, Prodr. 12: 666. 1848 syn. sec. Malekmohammadi & al. (2024)
= *Limonium scabrum* (Thunb.) Kuntze var. *scabrum* syn. sec. Malekmohammadi & al. (2024)

Notes. – Although Boissier (Prod. 12: 666. 1848) considers *Statice cinerea* Poir. a synonym of *Statice purpurata* L., it is closer to the aggregate of *Limonium scabrum* (sensu lato) according to Mucina (pers. comm.). The identity of *Statice scabra* sensu Drege in Boiss., which Boissier (Prod. 12: 666. 1848) regarded as a synonym of *L. purpuratum*, still remains uncertain and needs to be clarified.

***Limonium schinousae* Erben & Brullo in Phytotaxa 240: 150. 2016.** Sec. Domina (2011+)

***Limonium scopulorum* M.B.Crespo & Lledó in Folia Geobot. 44(2): 178. 2009.** Sec. Domina (2011+)

***Limonium scorpioides* Erben in Mitt. Bot. Staatssamml. München 28: 387. 1989.** Sec. Erben (1993)

= *Limonium caprariense* subsp. *multiflorum* Pignatti in Arch. Bot. (Forlì) 31: 77. 1955 syn. sec. Erben (1989)

***Limonium sebkarum* (Pomel) Maire in Bull. Soc. Hist. Nat. Afrique N. 25: 308. 1934.** Sec. Domina (2011+)

≡ *Statice sebkarum* Pomel, Nouv. Mat. Fl. Atlant.: 130. 1874 syn. sec. Domina (2011+) ≡ *Limonium gummiferum* subsp. *sebkarum* (Pomel) Maire, Cat. Pl. Maroc 4: 1094. 1941 syn. sec. Domina (2011+)
= *Statice sebkarum* var. *mauritii* Sennen, Diagn. Nouv.: 241. 1936 syn. sec. Malekmohammadi & al. (2024)

***Limonium secundirameum* (Lojac.) Greuter & Raus in Willdenowia 16(2): 451. 1987.** Sec. Domina (2011+)

≡ *Statice secundiramea* Lojac., Fl. Sicul. 2(2): 21. 1907 syn. sec. Greuter & Raus (1987) − *Limonium secundirameum* (Lojac.) Brullo in Bot. Not. 133(3): 292, 293. 1980, comb. inval. syn. sec. Greuter & Raus (1987) − *Limonium secundirameum* (Lojac.) Pignatti, Fl. Italia 2: 317. 1982, comb. inval. syn. sec. Greuter & Raus (1987)

***Limonium selinuntinum* Brullo in Bot. Not. 133(3): 291. 1980.** Sec. Domina (2011+)

***Limonium ×sennenii* (Rouy) P.Fourn., Quatre Fl. France: 722. 1937.** Sec. Fournier (1937)

≡ *Statice ×sennenii* Rouy in Rev. Bot. Syst. Geogr. Bot. 1(12): 181. 1904 syn. sec. Fournier (1937)

Notes. – This is the hybrid *Limonium duriusculum* × *Limonium virgatum*

***Limonium sercquense* (Ingr.) P.D.Sell, Fl. Gr. Brit. Ireland 1: 690. 2018.** Sec. Sell & Murrell (2018)

≡ *Limonium binervosum* var. *sercquense* Ingr. in Bot. J. Linn. Soc. 92(3): 191. Apr 1986 syn. sec. Sell & Murrell (2018)

***Limonium serpentinicum* R.Pino, Silva Pando & J.J.Pino in Novon 24(4): 382. 2016.** Sec. Malekmohammadi & al. (2024)

***Limonium serratum* Brullo in Mitt. Bot. Staatssamml. München 28: 419. 1989.** Sec. Domina (2011+)

***Limonium sibthorpianum* (Guss.) Kuntze, Revis. Gen. Pl. 2: 396. 1891.** Sec. Domina (2011+)

≡ *Statice sibthorpiana* Guss., Fl. Sicul. Prodr. Suppl. 1: 87. 1832 syn. sec. Kuntze (1891)
= *Statice gussoneana* Steud., Nomencl. Bot., ed. 2, 2: 633. 1841 syn. sec. Boissier (1848)

Notes. – *Statice gussoneana* Steud. is synonym of *Statice sibthorpiana* Guss. (as *S. gussoniana*) according to Boissier (Prod. 12: 652. 1848)

***Limonium sieberi* (Boiss.) Kuntze, Revis. Gen. Pl. 2: 396. 1891.** Sec. Domina (2011+)

≡ *Statice sieberi* Boiss., Voy. Bot. Espagne 2: 530, in nota. 1841 syn. sec. Kuntze (1891) ≡ *Statice graeca* var. *sieberi* (Boiss.) Boiss. in Candolle, Prodr. 12: 650. 1848 syn. sec. Boissier (1848)
= *Statice spathulata* Sieber ex Boiss. in Candolle, Prodr. 12: 650. 1848 syn. sec. Nyman (1881)
= *Statice virgata* Orph. ex Nyman, Consp. Fl. Eur. 3: 610. 1881 syn. sec. Nyman (1881)
= *Limonium runemarkii* Rech.f. in Bot. Jahrb. Syst. 80: 370. 1961 syn. sec. Domina (2011+)

Notes. – *Statice spathulata* Sieber ex Boiss. and *Statice virgata* Orph. ex. Nyman are synonym of *Statice sieberi* Boiss. according to Nyman (Consp. Fl. Eur. 3: 610. 1881)
Statice sieberi Boiss. is synonym of *Statice graeca* var. *sieberi* (Boiss.) Boiss. according to Boissier (Prodr. 12: 650. 1848)

***Limonium silvestrei* Aparicio in Ann. Bot. Fenn. 42(5): 372. 2005.** Sec. Domina (2011+)

***Limonium sirinicum* Erben & Brullo in Phytotaxa 240: 179. 2016.** Sec. Domina (2011+)

***Limonium sitiacum* Rech.f. in Denkschr. Akad. Wiss. Wien, Math.-Naturwiss. Kl. 105 (2,1): 103. 1943.** Sec. Domina (2011+)

***Limonium soboliferum* Erben in Mitt. Bot. Staatssamml. München 28: 313. 1989.** Sec. Domina (2011+)

***Limonium sommierianum* (Fiori) Arrigoni in Carta Veg. Isola Giannutri: 7. 1981.** Sec. Domina (2011+)

≡ *Statice minuta* var. *sommieriana* Fiori in Fl. Anal. Ital. 2: 331. 1902 syn. sec. Domina (2011+) ≡ *Statice sommieriana* (Fiori) Sommier in Nuovo Giorn. Bot. Ital. nov. ser. 10: 174. 1903 syn. sec. Domina (2011+)

***Limonium sougiae* Erben & Brullo in Phytotaxa 240: 133. 2016.** Sec. Domina (2011+)

***Limonium spathulatum* (Desf.) Kuntze, Revis. Gen. Pl. 2: 396. 1891.** Sec. Domina (2011+)

≡ *Statice spathulata* Desf., Fl. Atlant. 1: 275. 1798 syn. sec. Kuntze (1891) ≡ *Taxanthema spathulata* (Desf.) Sweet, Hort. Brit. 2: 332. 1826 syn. sec. Malekmohammadi & al. (2024) ≡ *Statice spathulata* var. *spathulata* syn. sec. Domina (2011+)
= *Statice cordata* Poir., Voy. Barbarie 2: 142. 1789 syn. sec. Boissier (1848)
= *Statice glauca* Pers., Syn. Pl. 1: 333. 1805 syn. sec. Malekmohammadi & al. (2024)
= *Taxanthema willdenowiana* Sweet, Hort. Brit. 2: 332. 1826 syn. sec. Malekmohammadi & al. (2024)
= *Statice spathulata* Willd. ex Boiss. in Candolle, Prodr. 12: 650-652. 1848 syn. sec. Malekmohammadi & al. (2024)
= *Limonium spathulatum* subsp. *rusicadense* (Maire) Quézel & Santa, Nouv. Fl. Algérie: 736. 1963 syn. sec. Domina (2011+)

Notes. – *Statice cordata* Poir. is synonym of *Statice spathulata* Desf. according to Boissier (Prodr. 12: 650. 1848).
Statce spathulata Will. is synonym of *Taxanthema willdenowiana* Sweet according to Sweet (Hort. Brit.: 332. 1826)

***Limonium spreitzenhoferi* Erben & Brullo in Phytotaxa 240: 131. 2016.** Sec. Domina (2011+)

***Limonium squarrosum* Erben in Mitt. Bot. Staatssamml. München 28: 313. 1989.** Sec. Domina (2011+)

***Limonium stenophyllum* Erben in Mitt. Bot. Staatssamml. München 28: 313. 1989.** Sec. Domina (2011+)

***Limonium stenotatum* (Rech.f.) Erben & Brullo in Phytotaxa 240: 141. 2016.** Sec. Domina (2011+)

≡ *Limonium graecum* var. *stenotatum* Rech.f. in Denkschr. Akad. Wiss. Wien, Math.-Naturwiss. Kl. 105 (2,1): 105. 1943 syn. sec. Domina (2011+)

***Limonium steppicum* Sefi, Ghrabi-Gammar & Brullo in Phytotaxa 446(5): 281. 2020.** Sec. Sefi & al. (2020)

Limonium strictissimum **(Salzm.) Arrigoni in Boll. Soc. Sarda Sci. Nat. 20: 240. 1981 ["1980"].** Sec. Domina (2011+)

≡ *Statice articulata* var. *strictissima* Salzm. in Flora 4: 108. 1821 syn. sec. Domina (2011+)
= *Statice dictyoclada* Boiss. in Candolle, Prodr. 12: 654. 1848 syn. sec. Domina (2011+) ≡ *Limonium dictyocladum* (Boiss.) Kuntze, Revis. Gen. Pl. 2: 395. 1891 syn. sec. Domina (2011+)
– *Limonium articulatum* subsp. *dictyocladum* sensu auct., err. sec. Domina (2011+)
– *Limonium oleifolium* subsp. *dictyocladum* sensu auct., err. sec. Domina (2011+)
– *Limonium virgatum* subsp. *dictyocladum* sensu auct., non (Arcang.) Pignatti, err. sec. Domina (2011+)
– *Limonium dictyocladum* sensu auct., non (Boiss.) Kuntze, err. sec. Domina (2011+)
– *Statice dictyoclada* sensu auct., non Boiss., err. sec. Domina (2011+)

Limonium subanfractum **Trinajstić, Suppl. Fl. Anal. Jugosl. 7: 6. 1980.** Sec. Domina (2011+)

Limonium subglabrum **Erben in Mitt. Bot. Staatssamml. München 14: 522. 1978.** Sec. Domina (2011+)

Limonium subnudum **Bogdanović & Brullo in Phytotaxa 215(1): 26. 2015.** Sec. Bogdanović & Brullo (2015)

Limonium subrotundifolium **(Bég. & A.Vacc.) Brullo in Webbia 33(1): 145. 1978.** Sec. Domina (2011+)

≡ *Statice delicatula* var. *subrotundifolia* Bég. & A.Vacc., Sec. Contr. Fl. Lib.: 29. 1913 syn. sec. Domina (2011+)

Limonium sucronicum **Erben in Mitt. Bot. Staatssamml. München 28: 313. 1989.** Sec. Domina (2011+)

Limonium sulcitanum **Arrigoni in Boll. Soc. Sarda Sci. Nat. 20: 233. 1981 ["1980"].** Sec. Malekmohammadi & al. (2024)

≡ *Limonium merxmuelleri* subsp. *sulcitanum* (Arrigoni) Arrigoni in Parlatorea 7: 19. 2005 syn. sec. Malekmohammadi & al. (2024: 2022)

Limonium supinum **(Girard) Pignatti, Collect. Bot. (Barcelona) 6(6): 309. 1962.** Sec. Domina (2011+)

≡ *Statice supina* Girard in Ann. Sci. Nat., Bot., ser. 3, 2: 326. 1844 syn. sec. Pignatti (1962)
= *Statice salsuginosa* Boiss. in Candolle, Prodr. 12: 653. 1848 syn. sec. Domina (2011+) ≡ *Limonium salsuginosum* (Boiss.) Kuntze, Revis. Gen. Pl. 2: 396. 1891 syn. sec. Pignatti (1962)
= *Statice salsuginosa* var. *hirtula* Willk. in Willkomm & Lange, Prodr. Fl. Hispan. 2: 376. 1868 syn. sec. Erben (1993)
= *Statice diegoi* Sennen, Butl. Inst. Catalana Hist. Nat. 32: 110. 1932 syn. sec. Malekmohammadi & al. (2024)
= *Statice hieronymi* Sennen in Bol. Soc. Ibér. Ci. Nat. 35: 28. 1936 syn. sec. Pignatti (1962)

Notes. – *Statice globulariaefolia* var. *minor* Boiss. is synonym of *Statice salsuginosa* Boiss. according to Boissier (Prodr. 12: 653. 1848)

Limonium supinum **var. *diegoi*** **(Sennen) Pignatti, Collect. Bot. (Barcelona) 6(6): 309. 1962.** Sec. Domina (2011+)

≡ *Limonium diegoi* Sennen, Diagn. Nouv.: 80. 1936 syn. sec. Domina (2011+)

Limonium supinum **var. *supinum*.** Sec. Malekmohammadi & al. (2024)

Limonium syracusanum **Brullo in Bot. Not. 133(3): 284. 1980.** Sec. Domina (2011+)

Limonium tabernense **Erben in Mitt. Bot. Staatssamml. München 14: 543. 1978.** Sec. Domina (2011+)

≡ *Limonium cossonianum* f. *tabernense* (Erben) G.Kunkel, Florula Desierto Almeriense: 201. 1988 syn. sec. Malekmohammadi & al. (2024)

Limonium tabulare **Bogdanović & Brullo in Phytotaxa 215(1): 26. 2015.** Sec. Bogdanović & Brullo (2015)

Limonium tacapense **Brullo in Mitt. Bot. Staatssamml. München 28: 466. 1989.** Sec. Domina (2011+)

Limonium taenari **Erben & Brullo in Phytotaxa 240: 148. 2016.** Sec. Domina (2011+)

Limonium tamarindanum **Erben in Mitt. Bot. Staatssamml. München 28: 313. 1989.** Sec. Domina (2011+)

Limonium tarcoense **Arrigoni & Diana in Candollea 48(2): 652. 1993.** Sec. Domina (2011+)

Limonium tauromenitanum **Brullo in Bot. Not. 133(3): 288. 1980.** Sec. Domina (2011+)

Limonium tenoreanum **(Guss.) Pignatti in Bot. J. Linn. Soc. 64(4): 365. 1971.** Sec. Domina (2011+)

≡ *Statice tenoreana* Guss., Enum. Pl. Inarim.: 268. 1855 syn. sec. Pignatti (1971)

Limonium tenuicaule **Erben in Mitt. Bot. Staatssamml. München 28: 313. 1989.** Sec. Domina (2011+)

Limonium tenuiculum **(Tineo ex Guss.) Pignatti in Bot. J. Linn. Soc. 64(4): 365. 1971.** Sec. Domina (2011+)

≡ *Statice tenuicula* Tineo ex Guss., Fl. Sicul. Prodr. Suppl. 1: 90. 1832 syn. sec. Pignatti (1971) ≡ *Statice cumana* var. *tenuicula* (Tineo ex Cuss.) Boiss. in Candolle, Prodr. 12: 657. 1848 syn. sec. Boissier (1848) ≡ *Limonium tenuiculum* (Tineo ex Guss.) Desole & Pignatti in Nouv. Giorn. Bot. Ital., n. s. 67: 18. 1961 syn. sec. Domina (2011+) − *Limonium tenuiculum* (Tineo ex Cuss.) Desole in Webbia 15: 557. 1960, comb. inval. syn. sec. Malekmohammadi & al. (2024) − *Limonium tenuicolum* (Tineo ex Guss.) Pignatti, orth. var. syn. sec. Domina (2011+)
= *Statice divaricata* Cav. ex Schult. in Roemer & Schultes, Syst. Veg. ed. 15bis 6: 847. 1820 syn. sec. Domina (2011+)

Limonium tenuifolium **(Bertol. ex Moris) Erben in Mitt. Bot. Staatssamml. München 17: 505. 1981.** Sec. Peruzzi & al. (2015)

≡ *Statice tenuifolia* Bertol. ex Moris, Stirp. Sard. Elench. 2: 8. 1828 syn. sec. Peruzzi & al. (2015) ≡ *Limonium acutifolium* subsp. *tenuifolium* (Bertol. ex Moris) Arrigoni in Parlatorea 7: 19. 2005 syn. sec. Malekmohammadi & al. (2024)

Limonium teuchirae **Brullo in Webbia 33(1): 148. 1978.** Sec. Domina (2011+)

Limonium thaenicum **Brullo in Mitt. Bot. Staatssamml. München 28: 469. 1989.** Sec. Domina (2011+)

Limonium tharrosianum **Arrigoni & Diana in Boll. Soc. Sarda Sci. Nat. 24: 285. 1985.** Sec. Peruzzi & al. (2015)

≡ *Limonium acutifolium* subsp. *tharrosianum* (Arrigoni & Diana) Arrigoni in Parlatorea 7: 19. 2005 syn. sec. Malekmohammadi & al. (2024)
= *Limonium sinisicum* Erben in Mitt. Bot. Staatssamml. München 22: 210. 1986 syn. sec. Domina (2011+)

***Limonium thiniense* Erben in Mitt. Bot. Staatssamml. München 17: 485. 1981.** Sec. Domina (2011+)

≡ *Limonium duriusculum* subsp. *thiniense* (Erben) O.Bolòs, Vigo, Masalles & Ninot, Fl. Man. Països Catalans, ed. 1: 1214. 1990 syn. sec. Domina (2011+)

***Limonium thirae* Erben & Brullo in Phytotaxa 240: 142. 2016.** Sec. Domina (2011+)

***Limonium tibulatium* Pignatti in Webbia 36(1): 55. 1982.** Sec. Domina (2011+)

= *Limonium ×tibulatium* Pignatti in Boll. Soc. Sarda Sci. Nat. 8: 91. 1971 syn. sec. Pignatti (1982)

Notes. – First described by Pignatti in 1971 without Latin description, as a possible hybrid (*L. acutifolium* x *L. articulatum*).

***Limonium tigulianum* Arrigoni & Diana in Boll. Soc. Sarda Sci. Nat. 28: 323. 1991.** Sec. Malekmohammadi & al. (2024)

≡ *Limonium merxmuelleri* subsp. *tigulianum* (Arrigoni & Diana) Arrigoni in Parlatorea 7: 19. 2005 syn. sec. Malekmohammadi & al. (2024: 2022)

***Limonium tineoi* (Lojac.) Giardina & Raimondo in Phytotaxa 196(1): 126. 30 Jan 2015.** Sec. Peruzzi & al. (2015)

≡ *Statice gussonei* Tineo ex Lojac., Fl. Sicul. 2(2): 18. 1907 syn. sec. Peruzzi & al. (2015) ≡ *Statice tineoi* Lojac., Fl. Sicul. 2(2): 18. 1907 syn. sec. Peruzzi & al. (2015) − *Limonium gussonei* (Tineo ex Lojac.) Giardina & Raimondo in Bocconea 20: 12. 2007, nom. inval. syn. sec. Peruzzi & al. (2015)

***Limonium tobarrense* J.Moreno, Terrones, M.Á.Alonso, Juan & M.B.Crespo in Phytotaxa 257(1): 65. 2016.** Sec. J.Moreno & al. (2016)

***Limonium todaroanum* Raimondo & Pignatti in Webbia 39(2): 417. 1986.** Sec. Domina (2011+)

***Limonium toletanum* Erben in Mitt. Bot. Staatssamml. München 28: 313. 1989.** Sec. Domina (2011+)

***Limonium tournefortii* (Girard) Erben in Mitt. Bot. Staatssamml. München 14: 514. 1978.** Sec. Domina (2011+)

≡ *Statice delicatula* var. *tournefortii* (Girard) Boiss. in Candolle, Prodr. 12: 653. 1848 syn. sec. Domina (2011+) ≡ *Limonium ilerdense* Erben in Mitt. Bot. Staatssamml. München 27: 386. 1988 syn. sec. Domina (2011+) − *Statice tournefortii* Girard in Ann. Sci. Nat., Bot., ser. 3, 2: 326. 1844, nom. inval. syn. sec. Domina (2011+) − *Limonium delicatulum* subsp. *tournefortii* (Girard) Pignatti, Collect. Bot. (Barcelona) 6(6): 305. 1962, comb. inval. syn. sec. Domina (2011+)

***Limonium transcanalis* (Ingr.) P.D.Sell, Fl. Gr. Brit. Ireland 1: 691. 2018.** Sec. Sell & Murrell (2018)

≡ *Limonium britannicum* subsp. *transcanalis* Ingr. in Bot. J. Linn. Soc. 92(3): 202. Apr 1986 syn. sec. Sell & Murrell (2018)

***Limonium transwallianum* (Pugsley) Pugsley in J. Bot. 62: 277. 1924.** Sec. Domina (2011+)

≡ *Statice transwalliana* Pugsley in J. Bot. 62: 133. 1924 syn. sec. Malekmohammadi & al. (2024)

Notes. – Listed as a species in the *Limonium binervosum* aggregate (Ingrouille and Stace 1986) and *L. binervosum* group (Pignatti, 1972).

***Limonium tremolsii* (Rouy) P.Fourn. ex Erben in Mitt. Bot. Staatssamml. München 14: 444. 1978.** Sec. Domina (2011+)

≡ *Statice tremolsii* Rouy in Bull. Soc. Bot. France 41: 325. 1894 syn. sec. Domina (2011+) ≡ *Limonium minutum* subsp. *tremolsii* (Rouy) P.Fourn., Quatre Fl. France: 721. 1937 syn. sec. Domina (2011+) − *Limonium tremolsii* (Rouy) P.Fourn., Quatre Fl. France: 721, in clavi. 1937, nom. inval. syn. sec. Erben (1978)

***Limonium trinajsticii* Bogdanović & Brullo in Phytotaxa 215(1): 27. 2015.** Sec. Bogdanović & Brullo (2015)

***Limonium tritonianum* Brullo in Mitt. Bot. Staatssamml. München 28: 419. 1989.** Sec. Domina (2011+)

***Limonium tunetanum* (Barratte & Bonnet) Maire in Mém. Soc. Hist. Nat. Afrique N. 3: 166. 1933.** Sec. Erben (2001)

≡ *Statice tunetana* Barratte & Bonnet, Ill. Fl. Tunisie: t. 15. 1895 syn. sec. Erben (2001)

***Limonium tyrrhenicum* Arrigoni & Diana in Boll. Soc. Sarda Sci. Nat. 24: 273. 1985.** Sec. Domina (2011+)

***Limonium ugijarense* Erben in Mitt. Bot. Staatssamml. München 28: 313. 1989.** Sec. Domina (2011+)

***Limonium ursanum* Erben in Mitt. Bot. Staatssamml. München 22: 214. 1986.** Sec. Brullo & Guarino (2017)

= *Limonium coralliforme* Alf.Mayer, Libri Botanici 15: 66. 1995 syn. sec. Erben (2001)

***Limonium usticanum* Giardina & Raimondo in Phytotaxa 196(1): 126. 30 Jan 2015.** Sec. Peruzzi & al. (2015)

− *Limonium usticanum* Giardina & Raimondo in Bocconea 20: 12. 2007, nom. inval. syn. sec. Peruzzi & al. (2015)
= *Statice ambigua* f. *major* Lojac., Fl. Sicul. 2(2): 27. 1907 syn. sec. Peruzzi & al. (2015)

***Limonium vaccarii* Pignatti ex Brullo in Webbia 33: 148. 1978.** Sec. Cuccuini & al. (2016)

= *Statice delicatula* Bég. & Vacc., Min. Colon., Monogr. Rapp. Colon. 7: 1-40. 1913, nom. illeg. syn. sec. Cuccuini & al. (2016)
= *Limonium sibthorpianum* subsp. *vaccarii* Pignatii ex Brullo in Webbia 33(1): 137-158. 1978 syn. sec. Cuccuini & al. (2016)

***Limonium ×valentinum* (Huter, Porta & Rigo) M.B.Crespo & Lledó, Gén. Limonium Comun. Valenciana: 98. 1998.** Sec. Crespo & Lledó (1998)

≡ *Statice ×valentina* Huter, Porta & Rigo in Oesterr. Bot. Z. 57: 431. 1907 syn. sec. Crespo & Lledó (1998)

Notes. – This is the hybrid of *Limonium girardianum* Kuntze (Guss.) Fourr. × *Limonium dufourii* (Girard) Kuntze

***Limonium validum* Erben in Mitt. Bot. Staatssamml. München 28: 313. 1989.** Sec. Erben (1993)

***Limonium vanandense* Erben & Brullo in Phytotaxa 240: 73. 2016.** Sec. Domina (2011+)

***Limonium velutinum* Bogdanović & Brullo in Phytotaxa 215(1): 27. 2015.** Sec. Bogdanović & Brullo (2015)

***Limonium vestitum* (C.E.Salmon) C.E.Salmon in J. Bot. 62. 1924: 336.** Sec. Bogdanović & Brullo (2015)

≡ *Statice vestita* C.E.Salmon, C.E.Salmon in J. Bot. 61. 1923: 97 syn. sec. C.E.Salmon in J. Bot. 62. 1924 (1924) ≡ *Limonium vestitum* subsp. *vestitum* syn. sec. Malekmohammadi & al. (2024)

***Limonium viciosoi* (Pau) Erben in Mitt. Bot. Staatssamml. München 14: 437. 1978.** Sec. Domina (2011+)

≡ *Statice viciosoi* Pau, Not. Bot. Fl. Españ. 6: 88. 1895 syn. sec. Domina (2011+) ≡ *Limonium catalaunicum* subsp. *viciosoi* (Pau) Pignatti, Collect. Bot. (Barcelona) 6(6): 300. 1962 syn. sec. Erben (1978)

***Limonium vigoi* L.Sáez, Curcó & Rosselló in Anales Jard. Bot. Madrid 56(2): 270. 1998.** Sec. Sáez & al. (1998)

***Limonium viniolae* Arrigoni & Diana in Boll. Soc. Sarda Sci. Nat. 27: 271. 1990.** Sec. Domina (2011+)

***Limonium ×viretianum* Pignatti in Arch. Bot. (Forlì) 31: 99. 1955.** Sec. Pignatti (1955)

***Limonium ×virgatoformis* (Rouy) B.Bock in Bull. Soc. Bot. Centre-Ouest 42: 264. 2012.** Sec. Malekmohammadi & al. (2024)

≡ *Statice ×virgatoformis* Rouy in Rev. Bot. Syst. Geogr. Bot. 1(12): 181. 1904 syn. sec. Malekmohammadi & al. (2024)

Notes. – This is the hybrid *Limonium echioides* × *Limonium virgatum*

***Limonium virgatum* (Willd.) Fourr. in Ann. Soc. Linn. Lyon, sér. 2, 17: 141. 1869.** Sec. Malekmohammadi & al. (2024)

≡ *Statice virgata* Willd., Enum. Pl.: 336. 1809 syn. sec. Malekmohammadi & al. (2024) ≡ *Taxanthema virgata* (Willd.) Sweet, Hort. Brit.: 333. 1826 syn. sec. POWO (2017+)
= *Statice cordata* Desf., Fl. Atlant. 1: 273. 1798 syn. sec. Boissier (1848)
= *Statice oleifolia* Willd., Sp. Pl., ed. 4, 1(2): 1525. 1798 syn. sec. Schultes (1820)
= *Statice oleifolia* Pourr. ex DC., Fl. Franç. ed. 3, 3: 422. 1805 syn. sec. Schultes (1820)
= *Statice oleifolia* Sm., Fl. Graec. Prodr. 1(1): 212. 1806 syn. sec. Boissier (1848)
= *Statice viminea* Schrad. ex Hornem., Hort. Bot. Hafn.: 37. 1819 syn. sec. Boissier (1848)
= *Taxanthema viminea* Sweet, Hort. Brit.: 333. 1826 syn. sec. Malekmohammadi & al. (2024)
= *Statice smithii* Ten., Fl. Napol. 3: 350. 1829 syn. sec. Domina (2011+)
= *Statice cosyrensis* subsp. *melia* Nyman, Consp. Fl. Eur. 3: 612. 1881 syn. sec. Pignatti (1971)
= *Statice melia* Nyman, Consp. Fl. Eur. 3: 612. 1881 syn. sec. Brullo (2016) ≡ *Limonium melium* (Nyman) Pignatti in Bot. J. Linn. Soc. 64(4): 365. 1971 syn. sec. Brullo (2016)
= *Statice catanensis* Janka in Természetrajzi Füz. 6: 174. 1882 syn. sec. Domina (2011+)
= *Statice virgata* var. *tenia* Heldr., Herb. Graec. Norm.: no. 1683. 1901 syn. sec. Brullo (2016) ≡ *Limonium tenium* (Heldr.) Rech.f. in Bot. Jahrb. Syst. 80(3): 371. 1961 syn. sec. Domina (2011+)
= *Statice tremolsii* var. *delilei* (Aubouy) Rouy in Bull. Soc. Bot. France 49: 301. 1902 syn. sec. POWO (2017+) ≡ *Statice delilei* Aubouy ex Rouy, Fl. France 10: 150. 1908 syn. sec. Domina (2011+)
− *Statice taubertii* Hausskn. in Mitth. Thüring. Bot. Vereins, n.f. 12: 53. 1897, nom. inval. syn. sec. Malekmohammadi & al. (2024)

Notes. – *Statice cordata* Desf., *Statice oleifolia* Sm. and *Statice viminea* Schrad. ex Hornem. are synonym of *Statice virgata* Willd. according to Boissier (Prod. 12: 654. 1848).

***Limonium ×virgitanum* Pignatti in Arch. Bot. (Forlì) 31: 99. 1955.** Sec. Pignatti (1955)

Notes. – Without Latin description.

***Limonium ×virgutiflorum* Pignatti in Arch. Bot. (Forlì) 31: 99. 1955.** Sec. Pignatti (1955)

Notes. – Without Latin description.

***Limonium vravronense* Erben & Brullo in Phytotaxa 240: 154. 2016.** Sec. Brullo (2016)

***Limonium wessexense* (Ingr.) P.D.Sell, Fl. Gr. Brit. Ireland 1: 690. 2018.** Sec. Sell & Murrell (2018)

≡ *Limonium procerum* var. *wessexense* Ingr. in Bot. J. Linn. Soc. 92(3): 197. Apr 1986 syn. sec. Sell & Murrell (2018)

***Limonium wiedmannii* Erben in Mitt. Bot. Staatssamml. München 22: 206. 1986.** Sec. Erben (1993)

***Limonium woolacombense* P.D.Sell, Fl. Gr. Brit. Ireland 1: 690. 2018.** Sec. Sell & Murrell (2018)

***Limonium xerocamposicum* Erben & Brullo in Phytotaxa 240: 56. 2016.** Sec. Domina (2011+)

***Limonium xerophilum* Brullo in Mitt. Bot. Staatssamml. München 28: 419. 1989.** Sec. Domina (2011+)

***Limonium xiliense* Erben & Brullo in Phytotaxa 240: 126. 2016.** Sec. Domina (2011+)

***Limonium zacynthium* R.Artelari in Mitt. Bot. Staatssamml. München 20: 429. 1984.** Sec. Domina (2011+)

***Limonium zankii* Bogdanović & Brullo in Phytotaxa 215(1): 28. 2015.** Sec. Bogdanović & Brullo (2015)

***Limonium zembrae* Pignatti in Webbia 36(1): 54. 1982.** Sec. Domina (2011+)

***Limonium zeraphae* Brullo in Bot. Not. 133(3): 285. 1980.** Sec. Domina (2011+)

***Limonium zeugitanum* Brullo in Mitt. Bot. Staatssamml. München 28: 419. 1989.** Sec. Domina (2011+)

***Limonium* sect. *Circinaria* (Boiss.) M.Malekm. in Taxon 66(5): 1142. 2017.** Sec. Malekmohammadi & al. (2017)

Type: *Limonium peregrinum* (P.J.Bergius) R.A.Dyer
≡ *Statice* sect. *Circinaria* Boiss. in Candolle, Prodr. 12: 666. 1848 syn. sec. Malekmohammadi & al. (2017)

= *Afrolimon* Lincz. in Novosti Sist. Vyssh. Rast. 16: 168. 1979 syn. sec. Malekmohammadi & al. (2017). Type: *Afrolimon peregrinum* (P.J.Bergius) Lincz.

Notes. – The section is endemic to South Africa and characterized by large flowers with circinate styles and capitate stigmata (Baker 1953; Boissier 1848). Monophyly of the section was tested and confirmed in Lledó et al.'s (2005) and Koutroumpa et al.'s (2018) phylogenetic studies by sampling two and three species, respectively.

***Limonium amoenum* (C.H.Wright) R.A.Dyer in Bull. Misc. Inform. Kew 1932(3): 155. 1932.** Sec. Malekmohammadi & al. (2024)

≡ *Statice amoena* C.H.Wright in Thiselton-Dyer, Flora Capensis 4(1.3): 420. 1906 syn. sec. Malekmohammadi & al. (2024) ≡ *Afrolimon amoenum* (C.H.Wright) Lincz. in Novosti Sist. Vyssh. Rast. 16: 168. 1979 syn. sec. Malekmohammadi & al. (2024)

***Limonium capense* (L.Bolus) L.Bolus in S. African Gard. 24: 129. 1934.** Sec. Malekmohammadi & al. (2024)

≡ *Statice capensis* L.Bolus in Ann. Bolus Herb. 1: 193. 1915 syn. sec. Malekmohammadi & al. (2024) ≡ *Afrolimon capense* (L.Bolus) Lincz. in Novosti Sist. Vyssh. Rast. 16: 168. 1979 syn. sec. Malekmohammadi & al. (2017)

***Limonium dagmariae* Mucina in Phytotaxa 403(2): 75. 2019 [as " *dagmarae* "].** Sec. Mucina & Hammer (2019)

− *Limonium dagmarae* Mucina, Plants of the Greater Cape Floristic Region, The extra Cape Flora 2: 439. 2013, nom. nud. syn. sec. Mucina & Hammer (2019) − *Limonium dagmarae* Mucina in Phytotaxa 403(2): 75. 2019, orth. var. syn. sec. Mucina & Hammer (2019)

***Limonium longifolium* (Thunb.) R.A.Dyer in Bothalia 7(3): 490. 1961.** Sec. Mucina & Hammer (2019)

≡ *Statice longifolia* Thunb., Prodr. Pl. Cap. 1: 54. 1794 syn. sec. R.A.Dyer (1961) ≡ *Statice purpurata* var. *longifolia* (Thunb.) Boiss. in Candolle, Prodr. 12: 667. 1848 syn. sec. R.A.Dyer (1961) ≡ *Limonium purpuratum* var. *longifolium* (Thunb.) F.T.Hubb. in Rhodora 18(211): 158. 1916 syn. sec. Malekmohammadi & al. (2017) ≡ *Afrolimon longifolium* (Thunb.) Lincz. in Novosti Sist. Vyssh. Rast. 16: 168. 1979 syn. sec. Malekmohammadi & al. (2017)
= *Limonium fergusoniae* L.Bolus in S. African Gard. 24: 124. 1934 syn. sec. Mucina & Hammer (2019)

***Limonium namaquanum* L.Bolus in S. African Gard. 24: 124. 1934.** Sec. Malekmohammadi & al. (2024)

≡ *Afrolimon namaquanum* (L.Bolus) Lincz. in Novosti Sist. Vyssh. Rast. 16: 168. 1979 syn. sec. Malekmohammadi & al. (2024)

***Limonium peregrinum* (P.J.Bergius) R.A.Dyer in Bothalia 7(3): 490. 1961.** Sec. Bredenkamp (2003)

≡ *Statice peregrina* P.J.Bergius, Descr. Pl. Cap.: 80. 1767 syn. sec. R.A.Dyer (1961) ≡ *Afrolimon peregrinum* (P.J.Bergius) Lincz. in Novosti Sist. Vyssh. Rast. 16: 168. 1979 syn. sec. Malekmohammadi & al. (2024)
= *Statice purpurata* Willd., Sp. Pl., ed. 4, 1(2): 1528. 1798 syn. sec. Boissier (1848)
= *Statice rosea* Sm. in Rees, Cycl. 34: no. 20. 1816 syn. sec. Malekmohammadi & al. (2024) ≡ *Limonium roseum* (Sm.) Kuntze, Revis. Gen. Pl. 2: 396. 1891 syn. sec. Malekmohammadi & al. (2024)
= *Statice rytidophylla* Hook. in Bot. Mag. 70: t. 4055. 1843 syn. sec. Boissier (1848) ≡ *Limonium rytidophyllum* (Hook.) H.Arnaud in Ser., Fl. Jard. 3: 301. 1849 syn. sec. Malekmohammadi & al. (2024)
= *Statice dickensonii* Poit. in Rev. Hort. (Paris), sér. 2 2: 425. 1844 syn. sec. Malekmohammadi & al. (2024)
= *Statice dickinsonii* hort. Angl. ex F.Cels, Ann. Fl. Pomone ser. 2, 2: 179, t. 21. 1844 syn. sec. Malekmohammadi & al. (2024)

Notes. – *Statice purpurata* Will. is synonym of *Statice rosea* Sm. and *Statice rytidophylla* Hokk. according to Boissier (Prod. 12: 667. 1848)

***Limonium purpuratum* (L.) Chaz. in Miller, Suppl. Dict. Jard. 2: 35. 1790.** Sec. Malekmohammadi & al. (2017)

≡ *Statice purpurata* L., Mant. Pl.: 59. 1767 syn. sec. Bailey (1916) ≡ *Taxanthema purpurata* (L.) Sweet, Hort. Brit.: 332. 1826 syn. sec. Malekmohammadi & al. (2024) ≡ *Limonium purpuratum* (L.) F.T.Hubb. in Rhodora 18(211): 158. 1916, comb. illeg. syn. sec. Malekmohammadi & al. (2024) ≡ *Afrolimon purpuratum* (L.) Lincz. in Novosti Sist. Vyssh. Rast. 16: 168. 1979 syn. sec. Malekmohammadi & al. (2017)
− *Statice purpurata* var. *genuina* Boiss. in Candolle, Prodr. 12: 666. 1848, nom. inval. syn. sec. Malekmohammadi & al. (2024)

Notes. – The identity of *Statice scabra* sensu Drege in Boiss., considered to be a synonym of *L. purpuratum* by Boissier (Prod. 12: 666. 1848), still remains uncertain (personal com. with Lodislav Mucina).

***Limonium teretifolium* L.Bolus in S. African Gard. 24: 124. 1934.** Sec. Malekmohammadi & al. (2017)

≡ *Afrolimon teretifolium* (L.Bolus) Lincz. in Novosti Sist. Vyssh. Rast. 16: 168. 1979 syn. sec. Malekmohammadi & al. (2017)

Limonium sect. *Ctenostachys* (Boiss.) Sauvage & Vindt in Trav. Inst. Sci. Chérifien, Sér. Bot. 4: 52. 1952. Sec. Malekmohammadi & al. (2017)

Type: *Limonium mucronatum* (L.f.) Chaz.
≡ *Statice* sect. *Ctenostachys* Boiss. in Candolle, Prodr. 12: 639. 1848 syn. sec. Malekmohammadi & al. (2017)

Notes. – The section consists of perennial herbs usually with crispate-winged or angled stems, compact, spreading-scorpioid spikes, and funnel-shaped, often coloured and shortly lobed calyces (Boissier 1848). The species are distributed in Macaronesia and Morocco. The monophyly of the section was confirmed by Koutroumpa et al. (2018).

***Limonium bahamense* (Griseb.) Britton in Bull. New York Bot. Gard. 4: 142. 1906.** Sec. Malekmohammadi & al. (2024)

≡ *Statice bahamensis* Griseb., Fl. Brit. W. I.: 389. 1861 syn. sec. Britton (1906)

***Limonium braunii* (Bolle) A.Chev. in Rev. Bot. Appl. Agric. Trop. 15: 928. 1935.** Sec. Hansen, A. & Sunding (1993)

≡ *Statice braunii* Bolle, Index Seminum (B), App. 1861: 4. 1861 syn. sec. Chevalier (1935)

***Limonium brunneri* (Webb) Kuntze, Revis. Gen. Pl. 2: 395. 1891.** Sec. Hansen, A. & Sunding (1993)

≡ *Statice brunneri* Webb, Niger Fl.: 170. 1849 syn. sec. Kuntze (1891)

***Limonium chazaliei* (H.Boissieu) Maire in Bull. Soc. Hist. Nat. Afrique N. 27: 67, 247. 1936.** Sec. Domina (2011+)

≡ *Statice chazaliei* H.Boissieu in J. Bot. (Morot) 10: 220. 1896 syn. sec. Malekmohammadi & al. (2024)
= *Limonium mauritanicum* Hutch. & Dalziel, Fl. W. Trop. Afr. 2: 188. 1931 syn. sec. Malekmohammadi & al. (2024)

***Limonium chrysopotamicum* Maire in Bull. Soc. Hist. Nat. Afrique N. 30: 355. 1940.** Sec. Domina (2011+)

***Limonium fallax* (Coss. ex Wangerin) Maire in Jahandiez & al., Cat. Pl. Maroc 3: 568. 1934.** Sec. Maire (1934)

≡ *Statice fallax* Coss. ex Wangerin in Repert. Spec. Nov. Regni Veg. 17: 398. 1921 syn. sec. Maire (1934)
= *Limonium ifniense* Caball. in Trab. Mus. Nac. Ci. Nat., Ser. Bot., 28: 11. 1935 [as " *ifniensis* "] syn. sec. Emberger & Maire (1941) ≡ *Statice ifniensis* Caball. in Trab. Mus. Nac. Ci. Nat., Ser. Bot., 28: 11. 1935 syn. sec. Emberger & Maire (1941) − *Limonium ifniensis* Caball., orth. var. syn. sec. Malekmohammadi & al. (2024) [is misspelling for *Limonium fallax* (Coss. ex Wangerin) Maire]

***Limonium haitiense* S.F.Blake in J. Wash. Acad. Sci. 21: 13. 1931.** Sec. Malekmohammadi & al. (2024)

≡ *Limonium bahamense* var. *haitiense* (S.F.Blake) Alain in Mem. New York Bot. Gard. 21(2): 144. 1971 syn. sec. Malekmohammadi & al. (2024) − *Limonium haitense* S.F.Blake, orth. var. syn. sec. Malekmohammadi & al. (2024) [is misspelling for *Limonium haitiense* S.F.Blake] − *Limonium bahamense* var. *haitense* (S.F.Blake) Alain, orth. var. syn. sec. Malekmohammadi & al. (2024)

***Limonium mucronatum* (L.f.) Chaz. in Miller, Suppl. Dict. Jard. 2: 36. 1790.** Sec. Malekmohammadi & al. (2024)

≡ *Statice mucronata* L.f., Suppl. Pl.: 187. 1782 syn. sec. Kuntze (1891) ≡ *Taxanthema mucronata* (L.f.) Sweet, Hort. Brit.: 333. 1826 syn. sec. Malekmohammadi & al. (2024) ≡ *Limonium mucronatum* (L.f.) Kuntze, Revis. Gen. Pl. 2: 395. 1891 syn. sec. Malekmohammadi & al. (2024)
= *Statice laeta* Salisb., Prodr. Stirp. Chap. Allerton: 177. 1796 syn. sec. Malekmohammadi & al. (2024)
= *Statice crispa* Pers., Syn. Pl. 1: 334. 1805 syn. sec. Malekmohammadi & al. (2024) ≡ *Limonium crispum* (Pers.) H.Arnaud in Ser., Fl. Jard. 3: 305. 1849 syn. sec. Malekmohammadi & al. (2024)
− *Statice crispa* L. ex B.D.Jacks., Index Linn. Herb.: 141. 1912, nom. inval. syn. sec. Malekmohammadi & al. (2024)

Notes. – *Statice crispa* L. ex B.D.Jacks. was indicated as a manuscript name by Jackson (1912) in his Index to the Linnean Herbarium. The specimen Jackson based his name on exists in the Linnean herbarium and was examined by Malekmohammadi. It is considered to belong to *L. mucronatum* (L.f.) Chaz.

***Limonium papillatum* (Webb & Berthel.) Kuntze, Revis. Gen. Pl. 2: 396. 1891.** Sec. Malekmohammadi & al. (2024)

≡ *Statice papillata* Webb & Berthel., Hist. Nat. Iles Canaries 3: 177. 1846 syn. sec. Kuntze (1891)
= *Statice pruinosa* Webb & Berthel. ex Boiss. in Candolle, Prodr. 12: 640. 1848 syn. sec. Boissier (1848)

Notes. – Statice pruinosa Webb & Berthol. ex Boiss. is synonym of Statice papillata Webb. & Berthel. according to Boissier (Prod. 12: 640. 1848)

***Limonium papillatum* var. *callibotryum* Svent., Index Seminum Hortus Acclim. Pl. Arautap., Pars Quarta: 43-60. 1969.** Sec. Domina (2011+)

***Limonium papillatum* var. *papillatum*.** Sec. Domina (2011+)

***Limonium pectinatum* (Aiton) Kuntze, Revis. Gen. Pl. 2: 394. 1891.** Sec. Malekmohammadi & al. (2024)

≡ *Statice pectinata* Aiton, Hort. Kew. 1: 385. 1789 syn. sec. Kuntze (1891) ≡ *Taxanthema pectinata* (Aiton) Sweet, Hort. Brit.: 333. 1826 syn. sec. Malekmohammadi & al. (2024)
= *Statice pectinata* var. *incompta* Webb & Berthel., Hist. Nat. Iles Canaries (Phytogr.) 3: 178. 1846 syn. sec. Malekmohammadi & al. (2024) ≡ *Limonium pectinatum* var. *incomptum* (Webb & Berthel.) G.Kunkel & Sunding, Cuad. Bot. Cuadernos Bot., Las Palmas 2: 15. 1967 syn. sec. Malekmohammadi & al. (2024)

***Limonium pectinatum* var. *corculum* (Webb & Berthel.) G.Kunkel & Sunding, Cuad. Bot. Cuadernos Bot., Las Palmas 2: 15. 1967.** Sec. Domina (2011+)

≡ *Statice pectinata* var. *corculum* Webb & Berthel., Hist. Nat. Iles Canaries 3: 178. 1846 syn. sec. G.Kunkel & Sunding (1967) ≡ *Limonium corculum* (Webb & Berthel.) Kuntze, Revis. Gen. Pl. 2: 395. 1891 syn. sec. Domina (2011+)
= *Statice corculum* Christ in Bot. Jahrb. Syst. 9(2): 141. 1887 syn. sec. Domina (2011+)

***Limonium pectinatum* var. *divaricatum* (Pit.) G.Kunkel & Sunding, Cuad. Bot. Cuadernos Bot., Las Palmas 2: 15. 1967.** Sec. Domina (2011+)

≡ *Statice pectinata* var. *divaricata* Pit., Iles Canaries: 320. 1908 syn. sec. G.Kunkel & Sunding (1967)

***Limonium pectinatum* var. *pectinatum*.** Sec. Domina (2011+)

***Limonium pectinatum* var. *solandri* (Webb & Berthel.) Kuntze, Revis. Gen. Pl. 2: 394. 1891.** Sec. Domina (2011+)

≡ *Statice pectinata* var. *solandri* Webb & Berthel. in Phytogr. Canar. 3(1): 178. 1846 syn. sec. Kuntze (1891) ≡ *Limonium solandri* (Webb & Berthel.) G.Kunkel in Cuad. Bot. Canaria 25: 45. 1975 syn. sec. Domina (2011+)
= *Statice humboldtii* Bolle, Append. Pl. Nov. Hort. Berol. 1861: 4. 1861 syn. sec. Domina (2011+) ≡ *Limonium humboldtii* (Bolle) Kuntze, Revis. Gen. Pl. 2: 395. 1891 syn. sec. Domina (2011+)

***Limonium trachycladum* Maire & Wilczek in Bull. Soc. Hist. Nat. Afrique N. 27: 246. 1936.** Sec. Domina (2011+)

≡ *Limonium fallax* var. *trachycladum* (Maire & Wilczek) Maire in Bull. Soc. Hist. Nat. Afrique N. 29: 433. 1938 syn. sec. Domina (2011+)

***Limonium* sect. *Iranolimon* M.Malekm., Akhani & Borsch in Taxon 66(5): 1142. 2017.** Sec. Malekmohammadi & al. (2017)

Type: *Limonium iranicum* (Bornm.) Lincz.
– *Statice* subsect. *Sarcophyllae* Boiss. in Candolle, Prodr. 12: 663. 1848 syn. sec. Malekmohammadi & al. (2024)

Notes. – Species of *L.* sect. *Iranolimon* are mostly distributed in the Irano-Turanian region and the monophyly of the section is well-supported (Malekmohammadi et al. 2017; Koutroumpa et al. 2018).

***Limonium anatolicum* Hedge in 23: 556. 1961.** Sec. Malekmohammadi & al. (2024)

***Limonium carnosum* (Boiss.) Kuntze, Revis. Gen. Pl. 2: 395. 1891.** Sec. Akhani & al. (2013)

≡ *Statice carnosa* Boiss. in Candolle, Prodr. 12: 663. 1848 syn. sec. Kuntze (1891) ≡ *Statice suffruticosa* var. *carnosa* (Boiss.) Kusn., Mat. Fl. Kavk. 5(1): 222. 1903 syn. sec. Linczevski (1952)

***Limonium failachicum* Erben & Mucina in Folia Geobot. 41(2): 229. 2006.** Sec. Malekmohammadi & al. (2024)

***Limonium gabrieli* (Bornm.) Rech.f., Fl. Iran. 108: 10. 1974.** Sec. Malekmohammadi & al. (2024)

≡ *Statice gabrieli* Bornm. in Repert. Spec. Nov. Regni Veg. 36: 170. 1934 syn. sec. Malekmohammadi & al. (2024)

***Limonium iranicum* (Bornm.) Lincz. in Schischkin & Bobrov, Flora URSS 18: 461. 1952.** Sec. Malekmohammadi & al. (2024)

≡ *Statice leptophylla* var. *iranica* Bornm. in Beih. Bot. Centralbl. Abt. 2, 22(2): 140. 1907 syn. sec. Linczevski (1952)

***Limonium kobstanicum* Tzvelev, Konspekt Flory Kavkaza 3(2): 282. 2012.** Sec. Malekmohammadi & al. (2024)

***Limonium leptophyllum* (Schrenk) Kuntze, Revis. Gen. Pl. 2: 395. 1891.** Sec. Linczevski (1952)

≡ *Statice leptophylla* Schrenk in Bull. Cl. Phys.-Math. Acad. Imp. Sci. Saint-Pétersbourg 3: 211. 1845 syn. sec. Kuntze (1891)
= *Statice suffruticosa* var. *leptophylla* Trautv. in Bull. Soc. Nat. Mosc. XL, 3: 96. 1867 syn. sec. Linczevski (1952)

***Limonium palmyrense* (Post) Dinsm., Pl. Post. & Dinsm. Fasc. 1: 12. 1932.** Sec. Malekmohammadi & al. (2024)

≡ *Statice palmyrensis* Post, Pl. Postianae 3: 16. 1892 syn. sec. Malekmohammadi & al. (2024)

***Limonium suffruticosum* (L.) Kuntze, Revis. Gen. Pl. 2: 396. 1891.** Sec. Malekmohammadi & al. (2024)

≡ *Statice suffruticosa* L., Sp. Pl.: 276. 1753 syn. sec. Kuntze (1891) ≡ *Taxanthema suffruticosa* (L.) Sweet, Hort. Brit.: 333. 1826 syn. sec. Malekmohammadi & al. (2024)
= *Statice fruticosa* Lepech., Reise Versch. Prov. Russ. Reich. 1: 254. 1774 syn. sec. Malekmohammadi & al. (2024)
= *Statice glauca* Less. in Linnaea 9: 196. 1834 syn. sec. Boissier (1848)
= *Limonium lessingianum* Lincz. in Novosti Sist. Vyssh. Rast. 8: 213. 1971 syn. sec. Malekmohammadi & al. (2024)
− *Statice suffruticosa* var. *typica* Trautv. in Bull. Soc. Nat. Mosc. 40(3): 95. 1867, nom. inval. syn. sec. Linczevski (1952)

Notes. – Statice glauca Less. is synonym of Statice suffruticosa according to Boissier (Prod. 12: 663. 1848).

***Limonium* sect. *Jovibarba* (Boiss.) M.Malekm. & Koutr., Phytokeys: 2024.** Sec. Malekmohammadi & al. (2024)

Type: *Limonium jovibarba* (Webb ex Boiss.) Kuntze
≡ *Statice* sect. *Jovibarba* Boiss. in Candolle, Prodr. 12: 665. 1848 syn. sec. Malekmohammadi & al. (2017)

Notes. – A small section of species endemic to Cape Verde that are closely related to species of *Limonium</I> sect. <i>Ctenostachys*.

***Limonium jovibarba* (Webb ex Boiss.) Kuntze, Revis. Gen. Pl. 2: 395. 1891.** Sec. Hansen, A. & Sunding (1993)

≡ *Statice jovibarba* Webb ex Boiss. in Candolle, Prodr. 12: 665. 1848 syn. sec. Kuntze (1891)

***Limonium lobinii* N.Kilian & T.Leyens in Willdenowia 24(1-2): 59. 1994.** Sec. Malekmohammadi & al. (2024)

***Limonium sundingii* Leyens, Lobin, N.Kilian & Erben in Willdenowia 25(1): 208. 1995.** Sec. Malekmohammadi & al. (2024)

***Limonium* sect. *Limoniodendron* Svent., Addit. Fl. Canar. 1: 38. 1960.** Sec. Koutroumpa & al. (2018)

Type: *Limonium dendroides* Svent.

Notes. – A monotypic section accommodating *Limonium dendroides* which is endemic to La Gomera (Canary Islands), and has a unique arborescent habit with woody stems up to 3 m tall (Sventenius 1960). Its phylogenetic placement as an isolated lineage sister to all other species of *Limonium* subg. *Limonium* is in agreement with its morphological distinctiveness (Lledó et al. 2005, Koutroumpa et al. 2018).

***Limonium dendroides* Svent., Addit. Fl. Canariensem 1: 38. 1960.** Sec. Domina (2011+)

***Limonium* sect. *Limonium*.** Sec. Koutroumpa & al. (2018)

Type: *Limonium vulgare* Mill.
= *Statice* subsect. *Genuinae* Boiss. in Candolle, Prodr. 12: 643. 1848 syn. sec. Koutroumpa & al. (2018)

Notes. – This section is distinguished by its large broad leaves with pinnate venation, tall stems with few or no sterile branches, large inflorescences, and calyces with short denticulate limbs bearing up to 10 lobes, with short lobes placed between larger lobes (Boissier 1848). The section had a much wider circumscription before (e.g., Boissier 1848) but was recently revised in the light of new phylogenetic results to comprise only species traditionally assigned to *Limonium* subsect. *Genuinae* (*Limonium vulgare* clade; Koutroumpa et al. 2018). Species of the section occur in both the Old (Irano-Turanian, Mediterranean, Euro-Siberian, and Macaronesian regions) and New World (North and South America) growing often in salt marshes and saline steppes.

***Limonium angustifolium* (Tausch) Turrill in Bull. Misc. Inform. Kew 1937(4): 208. 1937.** Sec. Malekmohammadi & al. (2024)

≡ *Statice angustifolia* Tausch, Syll. Pl. Nov. 2: 254. 1828 syn. sec. Rechinger (1943) ≡ *Limonium angustifolium* (Tausch) Degen, Fl. Veleb. 2: 540. 1937 syn. sec. Domina (2011+) ≡ *Limonium vulgare* subsp. *angustifolium* (Tausch) P.Fourn., Quatre Fl. France: 720. 1937 syn. sec. Malekmohammadi & al. (2024) ≡ *Statice limonium* subsp. *angustifolia* (Tausch) Rouy syn. sec. Malekmohammadi & al. (2024)
= *Statice gmelinii* W.D.J.Koch, Syn. Fl. Germ. Helv., ed. 2: 684. 1844 syn. sec. Boissier (1848)
= *Statice limonium* var. *macroclada* Boiss. in Candolle, Prodr. 12: 645. 1848 syn. sec. Bailey (1916) ≡ *Limonium vulgare* var. *macroclada* (Boiss.) F.T.Hubb. in Rhodora 18(211): 159. 1916 syn. sec. Rechinger (1943)
= *Statice boissieri* Lafont in Actes Soc. Linn. Bordeaux 27: 156. t. 9. f. 7. 1869 syn. sec. Malekmohammadi & al. (2024: 12 April 2022)
= *Statice gaillardotii* Lafont in Actes Soc. Linn. Bordeaux 27: 158. t. 9. f. 9. 1869 syn. sec. Malekmohammadi & al. (2024)
= *Statice limonioides* Bernh. ex Link. syn. sec. Gams (1927)
− *Statice lanceolata* hort. ex E.Vilm., Fl. Pleine Terre, ed. 3: 1099. 1870, nom. inval. syn. sec. Vilmorin (1870)

***Limonium asterotrichum* (C.E.Salmon) C.E.Salmon in J. Bot. 62. 1924: 336.** Sec. Domina (2011+)

≡ *Statice asterotricha* C.E.Salmon in J. Bot. 55: 33. 1917 syn. sec. C.E.Salmon in J. Bot. 62. 1924 (1924)

***Limonium brasiliense* (Boiss.) Kuntze, Revis. Gen. Pl. 2: 395. 1891.** Sec. Zuloaga & al. (2019)

≡ *Statice brasiliensis* Boiss. in Candolle, Prodr. 12: 644. 1848 syn. sec. Zuloaga & al. (2019) ≡ *Limonium brasiliense* var. *brasiliense* syn. sec. Malekmohammadi & al. (2024)
= *Statice marginata* Schott ex Steud., Nomencl. Bot., ed. 2, 2: 633. 1841 syn. sec. Boissier (1848)
= *Statice brasiliense* var. *antarctica* Boiss. in Candolle, Prodr. 12: 644. 1848 syn. sec. Zuloaga & al. (2019)
= *Limonium brasiliense* (Boiss.) Small in Bull. Torrey Bot. Club 24: 488. 1897 syn. sec. Malekmohammadi & al. (2024)
= *Statice patagonica* Speg. in Revista Fac. Agron. Univ. Nac. La Plata 3: 548. 1897 syn. sec. Zuloaga & al. (2019) ≡ *Limonium brasiliense* var. *patagonicum* (Speg.) Burkart, Fl. Il. Entre Ríos 5: 25. 1979 syn. sec. Zuloaga & al. (2019)
= *Limonium brasiliensis* A.Heller, Cat. N. Amer. Pl.: 6. 1898 syn. sec. Malekmohammadi & al. (2024)
= *Limonium brasiliense* Small, Fl. S.E. U.S.: 900. 1903 syn. sec. Malekmohammadi & al. (2024)
= *Limonium patagonicum* Macloskie, Rep. Princeton Univ. Exped. Patagonia, Botany 8: 656. 1905 syn. sec. Malekmohammadi & al. (2024)
= *Statice uruguayensis* Arechav. in Anales Mus. Nac. Montevideo 7: 24. 1909 syn. sec. Zuloaga & al. (2019) ≡ *Statice brasiliense* var. *uruguayensis* (Arechav.) Hauman in Anales Mus. Nac. Hist. Nat. Buenos Aires 24: 410. 1913 syn. sec. Zuloaga & al. (2019)
= *Statice brasiliensis* var. *patagonica* Hosseus, Physis (Buenos Aires) 1: 539. 1913 syn. sec. Malekmohammadi & al. (2024)
= *Statice americana* Larrañaga, Escritos D.A.Larrañaga 2: 125. 1923 syn. sec. Malekmohammadi & al. (2024)

***Limonium brevipetiolatum* R.Artelari & Erben in Mitt. Bot. Staatssamml. München 22: 507. 1986.** Sec. Domina (2011+)

***Limonium bulgaricum* Ančev, Fl. Narodna Republ. Bulg. 8: 483, 360. 1982.** Sec. Domina (2011+)

***Limonium bungei* (Claus) Gamajun. in Izv. Kazakhsk. Fil. Akad. Nauk S.S.S.R. 1: 10. 1944.** Sec. Domina (2011+)

≡ *Statice bungei* Claus, Beitr. Pflanzenk. Russ. Reiches 8: 308. 1851 syn. sec. Domina (2011+)
= *Statice gracilis* Fisch. ex Boiss. in Candolle, Prodr. 12: 660. 1848 syn. sec. Linczevski (1952)
= *Statice gmelini* var. *steiroclada* Trautv. in Bull. Acad. Sci. Pétersb. 14: 254. 1856 syn. sec. Linczevski (1952)
= *Statice membranacea* Czern. ex Trautv. in Trudy Imp. S.-Peterburgsk. Bot. Sada 9: 117. 1884 syn. sec. Linczevski (1952) ≡ *Limonium membranaceum* (Czern. ex Trautv.) Klokov, Fl. URSR 8: 170. 1958 syn. sec. Domina (2011+)
= *Statice gmelini* f. *steiroclada* Wangerin in Z. Naturwiss. 82: 442. 1912 syn. sec. Linczevski (1952)

***Limonium californicum* (Boiss.) A.Heller, Cat. N. Amer. Pl.: 6. 1898.** Sec. Morin (2005)

≡ *Statice californica* Boiss. in Candolle, Prodr. 12: 643. 1848 syn. sec. Malekmohammadi & al. (2024) ≡ *Statice limonium* var. *californica* (Boiss.) A.Gray, Rep. Colorado R. 4: 19. 1861 syn. sec. Blake (1916) ≡ *Limonium commune* var. *californicum* (Boiss.) Greene, Man. Bot. San Francisco Bay: 235. 1894 syn. sec. Morin (2005) ≡ *Limonium commune* subsp. *californicum* (Boiss.) A.E.Murray in Kalmia 12: 21. 1982 syn. sec. Malekmohammadi & al. (2024) ≡ *Limonium californicum* (Boiss.) A.Heller var. *californicum* syn. sec. Malekmohammadi & al. (2024)
= *Statice limonium* Rattan, Calif. Fl.: 72. 1879 syn. sec. Blake (1916) [non *Statice limonium* Pall.]
= ?*Limonium mexicanum* S.F.Blake in Rhodora 18(207): 59. 1916 syn. sec. Morin (2005) ≡ *Limonium commune* var. *mexicanum* (S.F.Blake) Jeps., Fl. Calif. 3(1): 77. 1939 syn. sec. Malekmohammadi & al. (2024) ≡ *Limonium californicum* var. *mexicanum* (S.F.Blake) Munz, Aliso 4(1): 96. 1958 syn. sec. Morin (2005)
− *Limonium californicum* Small, Bull. Torr. Club 24: 318. 1898 syn. sec. Malekmohammadi & al. (2024) [is later isonym of *Limonium californicum* (Boiss.) A.Heller]

***Limonium carolinianum* (Walter) Britton in Mem. Torrey Bot. Club 5(17): 255. 1894.** Sec. J.L.Luteyn (1976)

≡ *Statice caroliniana* Walter, Fl. Carol.: 118. 1788 syn. sec. Blake (1916) ≡ *Statice limonium* var. *carolinianum* (Walter) A.Gray, Manual (ed. 2): 270. 1856 syn. sec. J.L.Luteyn (1976) ≡ *Taxanthema caroliniana* (Walter) Sweet, Hort. Brit.: 332. 1826 syn. sec. Malekmohammadi & al. (2024) ≡ *Limonium carolinianum* (Walter) Britton var. *carolinianum* syn. sec. Malekmohammadi & al. (2024) − *Taxanthema carolinianum* (Walter) Sweet, Hort. Brit.: 332. 1826, orth. var. syn. sec. J.L.Luteyn (1976) [is orthographic variant for *Taxanthema caroliniana* (Walter) Sweet]
= *Statice limonium* Bigelow, Fl. Bost., ed. 1: 75. 1814 syn. sec. Malekmohammadi & al. (2024) [non *Statice limonium* Pall.]
= *Statice brasiliense* var. *angustata* A.Gray, Syn. Fl. N. Amer. 2(1): 54. 1878 [as " *brasiliensis* "] syn. sec. Malekmohammadi & al. (2024) ≡ *Limonium carolinianum* var. *angustatum* (A.Gray) S.F.Blake in Rhodora 25(292): 56. 1923 syn. sec. J.L.Luteyn (1976) ≡ *Limonium angustatum* (A. Gray) Small in Bull. Torrey Bot. Club 24(11): 488. 1897 syn. sec. J.L.Luteyn (1976) ≡ *Statice angustata* (Small) Wangerin in Z. Naturwiss. 82: 439. 1911 syn. sec. J.L.Luteyn (1976) ≡ *Limonium nashii* var. *angustatum* (A.Gray) H.E.Ahles in J. Elisha Mitchell Sci. Soc. 80(2): 173. 1964 syn. sec. J.L.Luteyn (1976) − *Limonium augustatum* (A.Gray) Small, orth. var. syn. sec. Malekmohammadi & al. (2024) [is misspelling for *Limonium angustatum* (A. Gray) Small] − *Statice brasiliense* var. *augustata* A.Gray, orth. var. syn. sec. Malekmohammadi & al. (2024) [is misspelling for *Statice brasiliense* var. *angustata* A.Gray]
= *Statice brasiliensis* A.Gray, Syn. Fl. N. Amer. 2(1): 54. 1878 syn. sec. J.L.Luteyn (1976)
= *Statice brasiliensis* Chapm., FI. S. U. S., ed. 2: 634. 1883 syn. sec. Malekmohammadi & al. (2024)
= *Statice lefroyi* Hemsl. in J. Bot. 21: 105. 1883 syn. sec. J.L.Luteyn (1976) ≡ *Limonium lefroyi* (Hemsl.) Britton in J. New York Bot. Gard. 6: 154. 1905 syn. sec. J.L.Luteyn (1976)
= *Limonium nashii* Small in Bull. Torrey Bot. Club 24(11): 491. 1897 syn. sec. J.L.Luteyn (1976) ≡ *Statice nashii* (Small) Wangerin in Z. Naturwiss. 82: 440. 1911 syn. sec. J.L.Luteyn (1976) ≡ *Limonium carolinianum* var. *nashii* (Small) B.Boivin in Naturaliste Canad. 93(5): 643. 1966 syn. sec. J.L.Luteyn (1976)
= *Statice endlichiana* Wangerin in Z. Naturwiss. 82: 441. 1910 syn. sec. J.L.Luteyn (1976) ≡ *Limonium endlichianum* (Wangerin) S.F.Blake in Rhodora 18(207): 60. 1916 syn. sec. J.L.Luteyn (1976)
= *Limonium obtusilobum* S.F.Blake in Rhodora 18(207): 63. 1916 syn. sec. J.L.Luteyn (1976) ≡ *Limonium carolinianum* var. *obtusilobum* (S.F.Blake) H.E.Ahles in J. Elisha Mitchell Sci. Soc. 80(2): 173. 1964 syn. sec. J.L.Luteyn (1976)
= *Limonium trichogonum* S.F.Blake in Rhodora 18(207): 61. 1916 syn. sec. J.L.Luteyn (1976) ≡ *Limonium nashii* var. *trichogonum* S.F.Blake in Rhodora 25(292): 58. 1923 syn. sec. J.L.Luteyn (1976) ≡ *Limonium carolinianum* var. *trichogonum* (S.F.Blake) B.Boivin in Naturaliste Canad. 93(5): 643. 1966 syn. sec. J.L.Luteyn (1976)
= *Statice tracyi* Gand. in Bull. Soc. Bot. France 66(5-6): 221. 1919 syn. sec. J.L.Luteyn (1976)
= *Limonium trichogonum* f. *albiflorum* House in Bull. New York State Mus. Nat. Hist. 243-244: 54. 1923 syn. sec. Malekmohammadi & al. (2024)
= *Limonium nashii* f. *albiflorum* House in Bull. N.Y. State Museum 254: 562. 1924 syn. sec. J.L.Luteyn (1976)
= *Limonium carolinianum* var. *compactum* Shinners in Field & Lab. 24(3): 105. 1956 syn. sec. J.L.Luteyn (1976)
− *Statice brasiliensis* var. *angustata* A.Gray, Syn. Fl. N. Amer. 2(1): 54. 1878 syn. sec. Malekmohammadi & al. (2024) [is misspelling for *Statice brasiliense* var. *augustata* A.Gray]
− *Statice caroliniana* var. *albiflora* Raf., Med. Fl. 2: 94. 1830, nom. nud. syn. sec. J.L.Luteyn (1976)
− *Statice caroliniana* var. *cespitosa* Raf., Med. Fl. 2: 94. 1830, nom. nud. syn. sec. J.L.Luteyn (1976)
− *Statice caroliniana* var. *longifolia* Raf., Med. Fl. 2: 94. 1830, nom. nud. syn. sec. J.L.Luteyn (1976)
− *Statice caroliniana* var. *pumila* Raf., Med. Fl. 2: 94. 1830, nom. nud. syn. sec. J.L.Luteyn (1976)
− *Statice caroliniana* var. *ramosissima* Raf., Med. Fl. 2: 94. 1830, nom. nud. syn. sec. J.L.Luteyn (1976)

***Limonium compactum* Erben & Brullo in Phytotaxa 240: 32. 2016.** Sec. Domina (2011+)

***Limonium coriarium* H.Arnaud in Ser., Fl. Jard. 3: 303. 1849.** Sec. Mabberley & Malécot (2023)

≡ *Statice coriaria* Pall. ex M.Bieb., Fl. Taur.-Caucas. 1: 249. 1808, nom. superfl. syn. sec. Mabberley & Malécot (2023) ≡ *Statice coriaria* Pall., Tabl. Phys. Topogr. Taur.: 49. 1795 syn. sec. Schultes (1820) ≡ *Statice latifolia* Sm. in Trans. Linn. Soc. London 1: 250. 1791 syn. sec. Mabberley & Malécot (2023) ≡ *Taxanthema latifolia* (Sm.) Sweet, Hort. Brit.: 332. 1826 syn. sec. Malekmohammadi & al. (2024) ≡ *Limonium latifolium* (Sm.) Kuntze, Revis. Gen. Pl. 2: 395. 1891, nom. illeg. syn. sec. Mabberley & Malécot (2023) ≡ *Limonium platyphyllum* Lincz. in Novosti Sist. Vyssh. Rast. 1: 266. 1964, nom. illeg. syn. sec. Mabberley & Malécot (2023) ≡ *Limonium gerberi* Soldano in Atti Soc. Ital. Sci. Nat. Mus. Civico Storia Nat. Milano 131(15): 254. 1991, nom. illeg. syn. sec. Mabberley & Malécot (2023) [is replaced synonym for *Limonium latifolium* (Sm.) Kuntze]
= *Statice coriacea* hort. ex Schult. in Roemer & Schultes, Syst. Veg. ed. 15bis 6: 778. 1820 syn. sec. Malekmohammadi & al. (2024)

***Limonium effusum* (Boiss.) Kuntze, Revis. Gen. Pl. 2: 395. 1891.** Sec. Malekmohammadi & al. (2024)

≡ *Statice effusa* Boiss. in Candolle, Prodr. 12: 646. 1848 syn. sec. Kuntze (1891)

***Limonium gmelini* (Willd.) Kuntze, Revis. Gen. Pl. 2: 395. 1891 [as " *gmelinii* "].** Sec. Malekmohammadi & al. (2017)

≡ *Statice gmelini* Willd., Sp. Pl. 1(2): 1524. 1798 syn. sec. Malekmohammadi & al. (2017) − *Limonium gmelinii* (Willd.) Kuntze, Revis. Gen. Pl. 2: 395. 1891, orth. var. syn. sec. Malekmohammadi & al. (2024) [is misspelling for *Limonium gmelini* (Willd.) Kuntze] − *Statice gmelini* var. *typica* Trautv. in Bull. Acad. Sci. Pétersb. 14: 252. 1856, nom. inval. syn. sec. Linczevski (1952)
= *Statice limonium* Pall., Tabl. Phys. Topogr. Taur.: 49. 1795 syn. sec. Schultes (1820)
= *Statice glauca* Willd. ex Schult., Syst. Veg. ed. 15bis 6: 799. 1820 syn. sec. Didukh Ya. & al. (2010)
= *Statice gmelinii* var. *scoparia* (Pall. ex Willd.) Schmalh., Syst. Veg. ed. 15[bis] 6: 799. 1820 syn. sec. Linczevski (1952)
= *Taxanthema gmelini* Sweet, Hort. Brit.: 332. 1826 syn. sec. Malekmohammadi & al. (2024)
= *Taxanthema scoparia* Sweet, Hort. Brit.: 332. 1826 syn. sec. Malekmohammadi & al. (2024)
= *Statice gmelini* var. *genuina* Boiss. in Candolle, Prodr. 12: 645. 1848 syn. sec. Linczevski (1952)

= *Statice emarginata* Schur, Enum. Pl. Transsilv.: 558. 1866 syn. sec. Malekmohammadi & al. (2024)
= *Statice gmelini* subsp. *genuina* Wangerin in Z. Naturwiss. 82: 441. 1912 syn. sec. Linczevski (1952)
= *Statice hypanica* Klokov, Vizn. rosl. URSR: 708. 1950, nom. illeg. syn. sec. Malekmohammadi & al. (2024)
= *Limonium hypanicum* Klokov in Fl. URSR 8: 524. 1958 syn. sec. Malekmohammadi & al. (2024) ≡ *Limonium gmelinii* var. *hypanicum* Pawł., Fl. Polska 10: 32. 1963 syn. sec. Didukh Ya. & al. (2010) ≡ *Limonium gmelinii* subsp. *hypanicum* (Klokov) Sóo, Acta Bot. Acad. Sci. Hung. 14 (1-2): 156. 1968 syn. sec. Didukh Ya. & al. (2010) ≡ *Limonium tomentellum* subsp. *hypanicum* (Klokov) Moysiyenko, Ecofl. Ukrayini 6: 8. 2010 syn. sec. Malekmohammadi & al. (2024)
− *Statice gmelinii* Willd., Sp. Pl. 1(2): 1524. 1798, orth. var. syn. sec. Malekmohammadi & al. (2024) [is misspelling for *Statice gmelini* Willd.]

Limonium guaicuru **(Molina) Kuntze, Revis. Gen. Pl. 2: 395. 1891.** Sec. Zuloaga & al. (2019)

≡ *Plegorhiza guaicuru* Molina, Sag. Stor. Nat. Chili 164: 351. 1782 syn. sec. Kuntze (1891) ≡ *Statice guaicuru* (Molina) Larrañaga, Escritos D. A. Larrañaga 1: 82. 1922 syn. sec. Malekmohammadi & al. (2024) ≡ *Statice guaicuru* (Molina) I.M.Johnst. in Contr. Gray Herb. 70: 92. 1924 syn. sec. Malekmohammadi & al. (2024)
= *Plegorhiza adstringens* Willd., Sp. Pl. 2(1): 487. 1799, nom. illeg. syn. sec. Kuntze (1891)
= *Statice chilensis* Phil. in Anales Univ. Chile 1861: 58. 1861 syn. sec. Kuntze (1891)
− *Plegorhiza quicura* Steud., Nomencl. Bot., ed. 2, 2: 354. 1841, pro syn. syn. sec. Zuloaga & al. (2019)

Limonium hirsuticalyx **Pignatti in Bot. J. Linn. Soc. 64(4): 361. 1971.** Sec. Domina (2011+)

= *Statice gmelinii* var. *limonioides* Wangerin in Z. Naturwiss. 82: 442. 1911 syn. sec. Pignatti (1971)

Limonium humile **Mill., Gard. Dict., ed. 8: no. 4. 1768.** Sec. Domina (2011+)

≡ *Statice humilis* (Mill.) C.E.Salmon in J. Bot. 51: 93. 1913 syn. sec. Malekmohammadi & al. (2024) ≡ *Limonium vulgare* subsp. *humile* (Mill.) Gams in Hegi, Illustrierte Flora von Mittel-Europa 5(3,97-106): 1883. 1927 syn. sec. Domina (2011+)
= *Statice limonoides* Biv. ex Link, Enum. Hort. Berol. Alt. 1: 295. 1821 syn. sec. Malekmohammadi & al. (2024)
= *Statice bahusiensis* Fr., Novit. Fl. Suec. Mant. 1(1-3): 10. 1832 syn. sec. Gams (1927) ≡ *Statice limonium* subsp. *bahusiensis* Syme, Engl. Bot., ed. 3B, 7: 162. 1867 syn. sec. Becherer (1928) ≡ *Limonium vulgare* subsp. *bahusiense* (Fr.) Bech. in Repert. Spec. Nov. Regni Veg. 25: 14. 1928 syn. sec. Domina (2011+) − *Statice limonium* subsp. *bahusiensis* (Fr.) Hook.f., Student. Fl. Brit. Isl.: 306. 1870, nom. inval. syn. sec. Domina (2011+)
= *Statice rariflora* Drejer, Fl. Excurs. Hafn.: 121. 1838 syn. sec. Gams (1927) ≡ *Statice limonium* subsp. *rariflora* (Drejer) Hook.f., Student. Fl. Brit. Isl., ed. 3: 259. 1884 syn. sec. Becherer (1928) ≡ *Limonium rariflorum* (Drejer) Kuntze, Revis. Gen. Pl. 2: 395. 1891 syn. sec. Malekmohammadi & al. (2024)
= *Statice crouanii* Lenorm. ex Nyman, Consp. Fl. Eur. 3: 609. 1881 syn. sec. Gams (1927)

Notes. – Rouy described Statice Limonium subsp. remotifolia (Rouy 1904 & 1908).

Limonium hungaricum **Klokov, Fl. URSR 8: 525. 1958.** Sec. Domina (2011+)

≡ *Limonium gmelinii* subsp. *hungaricum* (Klokov) Soó syn. sec. Domina (2011+)

Limonium limbatum **Small in Bull. Torrey Bot. Club 25(6): 317. 1898.** Sec. Morin (2005)

≡ *Statice limbata* (Small) K.Schum., Just's Bot. Jahresber. 26(1): 390. 1900 syn. sec. Morin (2005)
= *Limonium limbatum* var. *glabrescens* Correll in Rhodora 68(776): 425. 1966 syn. sec. Morin (2005)

Limonium marmarisense **Doğan & Akaydın in Ot Sist. Bot. Dergisi 24(2): 16. 2017.** Sec. Doğan & Akaydın (2017)

Limonium meyeri **(Boiss.) Kuntze, Revis. Gen. Pl. 2: 395. 1891.** Sec. Malekmohammadi & al. (2024)

≡ *Statice meyeri* Boiss. in Candolle, Prodr. 12: 645. 1848 syn. sec. Kuntze (1891) ≡ *Limonium scoparium* var. *meyeri* (Boiss.) Tzvelev, Konspekt Fl. Kavkaza 3(2): 281. 2012 syn. sec. Malekmohammadi & al. (2024)
= *Statice scoparia* Pall. ex Willd., Sp. Pl. 1(2): 1524. 1794 syn. sec. Linczevski (1952) ≡ *Limonium scoparium* (Pall. ex Willd.) H.Arnaud in Ser., Fl. Jard. 3: 303. 1849 syn. sec. Malekmohammadi & al. (2024)
= *Statice gmelini* var. *laxiflora* Boiss. in Candolle, Prodr. 12: 646. 1848 syn. sec. Malekmohammadi & al. (2024) ≡ *Statice laxiflora* (Boiss.) Novopokr. in Izv. Bot. Sada Akad. Nauk S.S.S.R. 30: 239. 1932 ["1931"] syn. sec. Linczevski (1952) − *Statice gmelinii* var. *laxiflora* Boiss. in Candolle, Prodr. 12: 646. 1848, orth. var. syn. sec. Linczevski (1952)
= *Statice scoparia* C.A.Mey. ex Boiss. in Candolle, Prodr. 12: 645. 1848 syn. sec. Linczevski (1952) [non *Statice scoparia* Pall. ex Willd.]
= *Statice obovata* Ledeb., Fl. Ross. 3: 468. 1849 syn. sec. Linczevski (1952) ≡ *Limonium obovatum* (Ledeb.) Kuntze, Revis. Gen. Pl. 2: 395. 1891 syn. sec. Linczevski (1952)
= *Statice gmelinii* var. *scoparia* Trautv. in Bull. Acad. Sci. Pétersb. 14: 263. 1856 syn. sec. Malekmohammadi & al. (2024)
= *Limonium gmelini* f. *laxiflorum* C.E.Salmon in Journ. of Bot. 47: 288. 1909 syn. sec. Linczevski (1952)
= *Limonium gmelini* var. *meyeri* C.E.Salmon in Journ. of Bot. 47: 288. 1909 syn. sec. Linczevski (1952)
= *Statice gmelini* subsp. *scoparia* Wangerin in Z. Naturwiss. 82: 442. 1912 syn. sec. Malekmohammadi & al. (2024)
= *Statice gmelini* var. *grandis* Popov ex Andross., Tr. Turkmensk. bot. sada 1: 52. 1941 syn. sec. Linczevski (1952)
= *Limonium tanaiticum* Gamajun. in Izv. Kazakhsk. Fil. Akad. Nauk S.S.S.R. 1: 9. 1944 syn. sec. Linczevski (1952)
= *Limonium scoparium* Klokov in Grossheim, Opred. Rast. Kauk.: 594. 1949 syn. sec. Malekmohammadi & al. (2024)
= *Limonium neoscoparium* Klokov, Fl. RSS Ucr. 8: 525. 1958 syn. sec. Tzvelev (2012)
= *Statice scoparia* M.Bieb., Fl. Taur.-Caucas. 1: 249. 1808, nom. illeg. syn. sec. Malekmohammadi & al. (2024) [non *Statice scoparia* Pall. ex Willd.] − *Limonium scoparium* (M.Bieb.) Stankov, Syst. Classif. Vasc. Pl. Eur. Russ.: 741. 1949, comb. inval. syn. sec. Linczevski (1952)

Limonium narbonense **Mill., Gard. Dict., ed. 8: no 2. 1768.** Sec. Dimopoulos & al. (2013)

= *Statice lespinassi* Lafont in Actes Soc. Linn. Bordeaux 27: 156. 1869 syn. sec. Malekmohammadi & al. (2024)

= *Statice nigricans* Lafont in Actes Soc. Linn. Bordeaux 27: 155. 1869 syn. sec. Malekmohammadi & al. (2024)
= *Statice scoparia* Rchb. ex Nyman, Consp. Fl. Eur. 3: 609. 1881 syn. sec. Nyman (1881) [non *Statice scoparia* Pall. ex Willd.]
= *Statice spinulosa* Janka in Természetrajzi Füz. 6: 170. 1882 syn. sec. Malekmohammadi & al. (2024)
= *Statice limonium* subsp. *aggregata* (Rouy) Rouy in Rev. Bot. Syst. Geogr. Bot. 1(11): 169. 1903 syn. sec. Malekmohammadi & al. (2024) ≡ *Statice aggregata* Rouy, Fl. France 10: 162. 1908 syn. sec. Malekmohammadi & al. (2024)
= *Statice remotiflora* Rouy in Rev. Bot. Syst. Geogr. Bot. 1(12): 179. 1904 syn. sec. Dimopoulos & al. (2013) ≡ *Statice limonium* subsp. *remotiflora* (Rouy) Rouy in Rev. Bot. Syst. Geogr. Bot. 1(12): 179. 1904 syn. sec. Dimopoulos & al. (2013)
= *Statice glauca* (Boiss.) Cuatrec. in Trab. Mus. Ci. Nat. Barcelona 12: 383. 1929 syn. sec. Malekmohammadi & al. (2024)
= *Statice serotina* Rchb., Fl. Germ. Excurs. 1: 191. 1830–1832 syn. sec. Domina (2011+) ≡ *Statice limonium* subsp. *serotina* (Rchb.) Nyman, Consp. Fl. Eur. 3: 611. 1881 syn. sec. Domina (2011+) ≡ *Limonium serotinum* (Rchb.) Pignatti in Giorn. Bot. Ital. 107(5): 220. 1973 syn. sec. Domina (2011+) ≡ *Limonium serotinum* (Rchb.) Erben in Mitt. Bot. Staatssamml. München 14: 411. 1978, nom. illeg. syn. sec. Domina (2011+) ≡ *Limonium vulgare* subsp. *serotinum* (Rchb.) Gams in Hegi, Illustrierte Flora von Mittel-Europa 5(3,97-106): 1882. 1927 syn. sec. Dimopoulos & al. (2013)
= *Statice barulensis* A.Bruni, Descr. Bot. Barletta: 169. 1857 syn. sec. Malekmohammadi & al. (2024: 12 April 2022)
= *Statice brunii* Guss. ex A.Bruni in Lez. Elem. Agric.: 87. 1868 syn. sec. Domina (2011+)
= *Limonium mareoticum* El Garf ex Hadidi & Fayed, Taeckholmia 15: 114. 1995 syn. sec. Malekmohammadi & al. (2024)
– *Statice behen* sensu auct., non Drejer, err. sec. Domina (2011+)

Notes. – *Statice scoparia* Rchb. is synonym of *Statice serotina* Rchb. according to Nyman (Consp. Fl. Eur. 3: 609. 1881)

***Limonium pagasaeum* Erben & Brullo in Phytotaxa 240: 34. 2016.** Sec. Domina (2011+)

***Limonium sareptanum* (A.K.Becker) Gams in Hegi, Illustrierte Flora von Mittel-Europa 5(3,97-106): 1880. 1927.** Sec. Domina (2011+)

≡ *Statice sareptana* A.K.Becker in Bull. Soc. Imp. Naturalistes Moscou 31(1): 12, 60. 1858 syn. sec. Gams (1927)
= *Statice gmelini* Rchb., Icon. Crit. Cent. 3: 37. 1825 syn. sec. C.E.Salmon (1911)
= *Statice ×intermedia* Czern., Konsp. rast. okr. Khar'kova: 51. 1859 syn. sec. C.E.Salmon (1911)
= *Statice tomentella* subsp. *sareptana* Nyman, Consp. Fl. Eur. 3: 609. 1881 syn. sec. C.E.Salmon (1911)
= *Limonium tomentellum* var. *sareptanum* C.E.Salmon in J. Bot. 49: 76. 1911 syn. sec. Linczevski (1952)
= *Statice maeotica* Klokov, Vizn. rosl. URSR: 707. 1950 syn. sec. Linczevski (1952)

***Limonium tomentellum* (Boiss.) Kuntze, Revis. Gen. Pl. 2: 396. 1891.** Sec. Domina (2011+)

≡ *Statice tomentella* Boiss. in Candolle, Prodr. 12: 645. 1848 syn. sec. Kuntze (1891) ≡ *Statice gmelinii* var. *tomentella* (Boiss.) Trautv., Bull. Acad. Sci. Petersb. 14: 225. 1856 syn. sec. C.E.Salmon (1911) ≡ *Statice gmelinii* subsp. *tomentella* (Boiss.) Wangerin in Z. Naturwiss. 82: 443. 1912 syn. sec. Tzvelev (2012)
= *Statice donetzica* Klokov, Vizn. rosl. URSR: 708. 1950 syn. sec. Linczevski (1952) ≡ *Limonium donetzicum* Klokov in Fomin, Fl. RSS Ucr. 8: 523. 1958 syn. sec. Pignatti (1972) ≡ *Limonium tomentellum* subsp. *donetzicum* (Klokov) Moysiyenko, Ecoflora of Ukraine: 8. 2010 syn. sec. Malekmohammadi & al. (2024)
= *Limonium dubium* Gamajun. ex Klokov in Novosti Sist. Vyssh. Nizsh. Rast. 1977: 64. 1978 syn. sec. Linczevski (1952)
= *Statice gmelini* M.Bieb., Fl. Taur.-Caucas. 1: 250. 1808 syn. sec. C.E.Salmon (1911)
= *Statice tschurjukiensis* Klokov in Okhor. Pamyat. Prir. Ukr. 1: 60, in adnot. 1927 syn. sec. Malekmohammadi & al. (2024) ≡ *Limonium tschurjukiense* (Klokov) Lavr. ex Klokov, Fl. RSS Ucr. 8: 163. 1958 syn. sec. Malekmohammadi & al. (2024)
= *Statice czurjukiensis* Klokov, Vizn. rosl. URSR: 708. 1950 syn. sec. Linczevski (1952) ≡ *Limonium czurjukiense* (Klokov) Lavrenko, Descr. Veg. URSS 2: 669. 1956 syn. sec. Pignatti (1972)
= *Statice donetzica* Klokov, Vizn. rosl. URSR: 708. 1950 syn. sec. Linczevski (1952) ≡ *Limonium donetzicum* Klokov in Fomin, Fl. RSS Ucr. 8: 523. 1958 syn. sec. Pignatti (1972) ≡ *Limonium tomentellum* subsp. *donetzicum* (Klokov) Moysiyenko, Ecoflora of Ukraine: 8. 2010 syn. sec. Malekmohammadi & al. (2024)

***Limonium vanense* Kit Tan & Sorger in 41: 533. 1984.** Sec. Domina (2011+)

***Limonium vulgare* Mill., Gard. Dict., ed. 8, n. 1. 1768.** Sec. Domina (2011+)

= *Statice limonium* L., Sp. Pl.: 274. 1753 syn. sec. Malekmohammadi & al. (2024) ≡ *Taxanthema limonium* (L.) Sweet, Hort. Brit.: 332. 1826 syn. sec. Malekmohammadi & al. (2024) − *Limonium limonium* (L.) A.Lyons, Pl. Nam.: 225, in obs. 1900, nom. inval. syn. sec. Malekmohammadi & al. (2024)
= *Statice maritima* Lam., Fl. Franç. 3: 64. 1779 syn. sec. Schultes (1820)
= *Statice behen* Drejer, Fl. Excurs. Hafn.: 122. 1838 syn. sec. Domina (2011+) ≡ *Statice limonium* subsp. *behen* Syme, Engl. Bot., ed. 3B, 7: 161. 1867 syn. sec. Becherer (1928) ≡ *Limonium behen* (Drejer) Kuntze, Revis. Gen. Pl. 2: 395. 1891 syn. sec. Malekmohammadi & al. (2024) ≡ *Limonium vulgare* subsp. *behen* (Drejer) Bech. in Repert. Spec. Nov. Regni Veg. 25: 14. 1928 syn. sec. Malekmohammadi & al. (2024)
= *Statice drepanensis* Tineo ex Guss., Fl. Sicul. Syn. 2: 805. 1845 syn. sec. Malekmohammadi & al. (2024)
= *Statice limonium* var. *puberula* Regel, Index Seminum (LE, Petropolitanus) 1860: 34. 1860 syn. sec. Malekmohammadi & al. (2024)
= *Statice longidentata* Lafont in Actes Soc. Linn. Bordeaux 27: 156. 1869 syn. sec. Malekmohammadi & al. (2024)
= *Statice limonia* St.-Lag. in Ann. Soc. Bot. Lyon 7: 135. 1880 syn. sec. Malekmohammadi & al. (2024)
= *Statice scanica* Warm., Dansk Plantev. 1: 209. 1906 syn. sec. Malekmohammadi & al. (2024)
= *Statice limonium* var. *longidentata* (Lafont) Rouy, Fl. France 10: 161. 1908 syn. sec. Malekmohammadi & al. (2024: 12 April 2022)

= *Limonium maritimum* Caperta, Cortinhas, A.P.Paes, Guara, Esp.Santo & Erben in Ann. Bot. (Oxford) 115(3): 383. 2015 syn. sec. Malekmohammadi & al. (2024)
= *Limonium commune* Gray, Nat. Arr. Brit. Pl. 2: 296. 1821 syn. sec. Malekmohammadi & al. (2024)
= *Limonium commune* var. *minus* Gray, Nat. Arr. Brit. Pl. 2: 296. 1822 ["1821"] syn. sec. Malekmohammadi & al. (2024)
= *Limonium commune* var. *obtusum* Gray, Nat. Arr. Brit. Pl. 2: 296. 1822 ["1821"] syn. sec. Malekmohammadi & al. (2024)
= *Limonium commune* var. *serotinum* Gray, Nat. Arr. Brit. Pl. 2: 296. 1822 ["1821"] syn. sec. Malekmohammadi & al. (2024)
= *Statice limonium* var. *minor* Roth, Enum. Pl. Phaen. Germ. 1(1): 1012. 1827 syn. sec. Malekmohammadi & al. (2024)
= *Statice pseudolimonium* Rchb., Fl. Germ. Excurs. 1: 191. 1831 syn. sec. Erben (1993) ≡ *Limonium vulgare* subsp. *pseudolimonium* (Rchb.) Gams in Hegi, Illustrierte Flora von Mittel-Europa 5(3,97-106): 1883. 1927 syn. sec. Becherer (1928)
= *Statice behen* Drejer, Fl. Excurs. Hafn.: 122. 1838 syn. sec. Domina (2011+) ≡ *Statice limonium* subsp. *behen* Syme, Engl. Bot., ed. 3B, 7: 161. 1867 syn. sec. Becherer (1928) ≡ *Limonium behen* (Drejer) Kuntze, Revis. Gen. Pl. 2: 395. 1891 syn. sec. Malekmohammadi & al. (2024) ≡ *Limonium vulgare* subsp. *behen* (Drejer) Bech. in Repert. Spec. Nov. Regni Veg. 25: 14. 1928 syn. sec. Malekmohammadi & al. (2024)
= *Limonium vulgare* f. *pyramidale* C.E.Salmon syn. sec. Gams (1927)
= *Statice limonium* var. *behen* Rouy syn. sec. Gams (1927)
= *Statice limonium* var. *pseudolimonium* Rouy syn. sec. Domina (2011+)
= *Statice limonium* var. *scanica* Fries syn. sec. Gams (1927)
− *Limonium limonium* Druce in Rep. Bot. Soc. Exch. Club Brit. Isles 7(3): 583, 687. 1924, nom. inval. syn. sec. Malekmohammadi & al. (2024)
− *Statice limonium* var. *genuina* Boiss., nom. inval. syn. sec. Gams (1927)
− *Statice limonium* var. *typica* Rouy, nom. inval. syn. sec. Gams (1927)

Notes. – *Limonium maritimum* Caperta is described as a segregated species from *Limonium vulgare* from Portugal coast based on the morphological and karyological studies.
In another study SNP data is used to text the application of this data to understanding the genetic structure in *L. vulgare* complex and differentiation between *L. maritimum* and *L. vulgare* (Pina-Martins et al., BMC Plant Biology (2023) 23:34). The authors address that "a set of 34 SNPs were found to be fully segregated between *L. vulgare* and *L. maritimum*, two of which are potentially linked to proteins that might be involved in the speciation process".
In the PCA graphs of published in this article the *L. maritimum* specimens are very close to the *L. vulgare* and in the phylogenetic reconstructed from the Maximum Likelihood they are nested within it. This result shows that *L. vulgare* is not monophyletic without merging *L. maritimum* within it. Accordingly, the segregation of *L. maritimum* from *L. vulgare* is not accepted in this checklist.

***Limonium vulgare* var. *album* F.T.Hubb. in Rhodora 18(211): 159. 1916, nom. nud.** Sec. Bailey (1916)

− *Statice limonium* var. *alba* hort. ex F.T.Hubb. in Rhodora 18(211): 159. 1916, nom. nud. syn. sec. Bailey (1916)

Limonium sect. *Nephrophyllum* Rech.f., Fl. Iran. 108: 8. 1974. Sec. Koutroumpa & al. (2018)

Type: *Limonium reniforme* (Girard) Lincz.
= *Statice* subsect. *Hyalolepideae* Boiss. in Candolle, Prodr. 12: 659. 1848 syn. sec. Koutroumpa & al. (2018)

Notes. – An expanded circumscription for this section that includes also species of the "*Limonium bellidifolium* complex" is currently accepted based on phylogenetic results by Koutroumpa et al. (2018) who also expanded description of the section. The species belonging here are distributed in the Irano-Turanian area, apart from *L. bellidifolium* that spread towards the Euro-Siberian and Mediterranean regions.

***Limonium afghanicum* Erben & Podlech in Mitt. Bot. Staatssamml. München 16: 547. 1980.** Sec. Malekmohammadi & al. (2024)

***Limonium bellidifolium* (Gouan) Dumort., Fl. Belg.: 27. 1827.** Sec. Domina (2011+)

≡ *Statice limonium* var. *bellidifolia* Gouan, Fl. Monsp.: 231. 1764 syn. sec. Domina (2011+) ≡ *Statice bellidifolia* (Gouan) DC., Fl. Franç. ed. 3, 3: 421. 1805 syn. sec. Domina (2011+) ≡ *Taxanthema bellidifolia* (Gouan) Sweet, Hort. Brit.: 332. 1826 syn. sec. POWO (2017+)
= *Statice bellidifolia* var. *divaricata* DC., Fl. Franç., ed. 3, 5: 379. 1815 syn. sec. POWO (2017+)
= *Statice airoides* Tausch, Syll. Pl. Nov. 2: 255. 1828 syn. sec. Malekmohammadi & al. (2024)
= *Statice dichotoma* Duby, Bot. Gall.: 388. 1828, nom. illeg. syn. sec. POWO (2017+) [non *Statice dichotoma* Cav.]
= *Statice diffusa* Laterr. ex Boiss. in Candolle, Prodr. 12: 661. 1848 syn. sec. Malekmohammadi & al. (2024)
= *Limonium danubiale* Klokov in Fl. URSR 8: 526. 1958 syn. sec. POWO (2017+)

***Limonium bellidifolium* var. *bellidifolium*.** Sec. Malekmohammadi & al. (2024)

***Limonium bellidifolium* var. *prostratum* (Beauverd) Rech.f. in Denkschr. Akad. Wiss. Wien, Math.-Naturwiss. Kl. 105(1): 427. 1943.** Sec. Rechinger (1943)

≡ *Statice bellidifolia* var. *prostrata* Beauverd in Bull. Soc. Bot. Genève 28: 153. 1938 syn. sec. Rechinger (1943)

***Limonium caspium* (Willd.) Gams in Hegi, Illustrierte Flora von Mittel-Europa 5(3,97-106): 1880. 1927.** Sec. Akhani & al. (2013)

≡ *Statice caspia* Willd., Enum. Pl. Horti Berol. 1: 336. 1809 syn. sec. Gams (1927) ≡ *Taxanthema caspia* (Willd.) Sweet, Hort. Brit.: 332. 1826 syn. sec. Malekmohammadi & al. (2024) ≡ *Limonium caspium* (Willd.) P.Fourn., Quatre Fl. France: 720. 1937 syn. sec. Akhani & al. (2013) ≡ *Limonium bellidifolium* subsp. *caspium* (Willd.) P.Fourn. syn. sec. Malekmohammadi & al. (2024)

= *Statice oleifolia* Host, Syn. Pl.: 177. 1797 syn. sec. Malekmohammadi & al. (2024: 14 April 2022)
= *Statice caspia* Pollini, Fl. Veron. 1: 418, 611. 1822 syn. sec. Malekmohammadi & al. (2024: 14 April 2022)
= *Statice patens* Fisch. ex Boiss. in Candolle, Prodr. 12: 661. 1848 syn. sec. Malekmohammadi & al. (2024) ≡ *Statice caspia* var. *patens* (Fisch. ex Boiss.) Boiss. in Candolle, Prodr. 12: 661. 1848 syn. sec. Malekmohammadi & al. (2024) ≡ *Limonium caspium* var. *patens* (Fisch. ex Boiss.) A.V.Grebenjuk, Konspekt Fl. Aziatsk. Rossii: 117. 2012 syn. sec. Malekmohammadi & al. (2024)
= *Statice caspia* var. *urumiensis* Bornm. in Verh. K. K. Zool.-Bot. Ges. Wien 60: 165. 1910 syn. sec. Akhani & al. (2013)
= *Limonium adilguneri* Yıld. & Doğru-Koca in Ot Sist. Bot. Dergisi 13(1): 13. 2006 syn. sec. Malekmohammadi & al. (2024)
= *Limonium smithii* Akaydın in World Appl. Sci. J. 2(4): 406. 2007 syn. sec. Malekmohammadi & al. (2024)
– *Limonium bellidifolium* sensu auct., non (Guan) Dumort., err. sec. Akhani & al. (2013)

***Limonium coralloides* (Tausch) Lincz. in Schischkin & Bobrov, Flora URSS 18: 451. 1952.** Sec. Peng & Kamelin (1996)

≡ *Statice coralloides* Tausch, Syll. Pl. Nov. 2: 255. 1828 syn. sec. Linczevski (1952)
= *Statice aphylla* Poir., Encycl. 7(1): 408. 1806 syn. sec. Linczevski (1952)
= *Statice decipiens* Ledeb., Fl. Altaic. 1: 433. 1829 syn. sec. Linczevski (1952) ≡ *Limonium decipiens* (Ledeb.) Kuntze, Revis. Gen. Pl. 2: 395. 1891 syn. sec. Linczevski (1952)
− *Statice reticulata* Sievers ex Boiss. in Candolle, Prodr. 12: 661. 1848, nom. inval. syn. sec. Malekmohammadi & al. (2024)

***Limonium cretaceum* Cherkasova in Byull. Moskovsk. Obshch. Isp. Prir., Otd. Biol. 75(4): 100. 1970.** Sec. Abdulina (1999)

***Limonium dubyi* (Gren. & Godr.) Kuntze, Revis. Gen. Pl. 2: 395. 1891.** Sec. Domina (2011+)

≡ *Statice dubyi* Gren. & Godr., Fl. France 2: 750. 1853 syn. sec. Kuntze (1891) ≡ *Limonium bellidifolium* subsp. *dubyi* (Gren. & Godr.) P.Fourn., Quatre Fl. France: 720. 1937 syn. sec. Domina (2011+)
= *Statice dubyeana* Bubani, Fl. Pyren. 1: 196. 1897 syn. sec. Malekmohammadi & al. (2024)

Notes. – *Statice dubyi* Godr. is synonym of *Statice dubyeana* Bubani according to Bubani (1897)

***Limonium iconium* (Boiss. & Heldr.) Kuntze, Revis. Gen. Pl. 2: 395. 1891 [as " *iconicum* "].** Sec. Bokhari & Edmondson (1982)

≡ *Statice iconia* Boiss. & Heldr. in Candolle, Prodr. 12 12: 661. 1848 syn. sec. Kuntze (1891) − *Limonium iconicum* (Boiss. & Heldr.) Kuntze, Revis. Gen. Pl. 2: 395. 1891, orth. var. syn. sec. Malekmohammadi & al. (2024)

***Limonium kimmericum* (Lipsky) Klokov, Fl. URSR 8: 527. 1958.** Sec. Malekmohammadi & al. (2024)

≡ *Statice caspia* var. *kimmerica* Lipsky in Mat. Fl. Kavk. 4(1): 219. 1902 syn. sec. Malekmohammadi & al. (2024) − *Limonium cimmericum* (Lipsky) Klokov, Fl. URSS 8: 527. 1958, orth. var. syn. sec. Malekmohammadi & al. (2024) [is orthographic variant for *Limonium kimmericum* (Lipsky) Klokov]

***Limonium macrorrhizum* (Ledeb.) Kuntze, Revis. Gen. Pl. 2: 395. 1891.** Sec. Domina (2011+)

≡ *Statice macrorrhiza* Ledeb., Fl. Altaic. 1: 434. 1829 syn. sec. Kuntze (1891)

***Limonium myrianthum* (Schrenk ex Fisch. & C.A.Mey.) Kuntze, Revis. Gen. Pl. 2: 395. 1891.** Sec. Linczevski (1952)

≡ *Statice myriantha* Schrenk ex Fisch. & C.A.Mey., Enum. Pl. Nov. 1: 14. 1841 syn. sec. Kuntze (1891)
= *Statice latissima* Kar. & Kir. in Bull. Soc. Imp. Naturalistes Moscou 14(4): 729. 1841 syn. sec. Linczevski (1952)

***Limonium otolepis* (Schrenk) Kuntze, Revis. Gen. Pl. 2: 396. 1891.** Sec. Akhani & al. (2013)

≡ *Statice otolepis* Schrenk in Bull. Cl. Phys.-Math. Acad. Imp. Sci. Saint-Pétersbourg 1: 362. 1843 syn. sec. Kuntze (1891)

***Limonium perfoliatum* (C.A.Mey. ex Boiss.) Kuntze, Revis. Gen. Pl. 2: 396. 1891.** Sec. Akhani & al. (2013)

≡ *Statice perfoliata* C.A.Mey. ex Boiss. in Candolle, Prodr. 12: 663. 1848 syn. sec. Kuntze (1891)

***Limonium popovii* Kubansk. in Fl. Kazakhst. 7: 84, 473. 1964.** Sec. Lazkov & Sultanova (2011)

***Limonium reniforme* (Girard) Lincz. in Schischkin & Bobrov, Flora URSS 18: 456. 1952.** Sec. Akhani & al. (2013)

≡ *Statice reniformis* Girard in Ann. Sci. Nat., Bot., ser. 3, 2: 325. 1844 syn. sec. Linczevski (1952)
= *Statice perfoliata* var. *reniformis* Boiss. in Candolle, Prodr. 12: 663. 1848 syn. sec. Akhani & al. (2013)

***Limonium tamaricoides* Bokkari in 30: 301. 1970.** Sec. Domina (2011+)

***Limonium tianschanicum* Lincz. in Novosti Sist. Vyssh. Rast. 8: 209. 1971.** Sec. Lazkov & Sultanova (2011)

Limonium sect. *Plathymenium* (Boiss.) Lincz. in Schischkin & Bobrov, Flora URSS 18: 420. 1952. Sec. Malekmohammadi & al. (2017)

Type: *Limonium flexuosum* (L.) Chaz.
≡ *Statice* sect. *Plathymenium* Boiss. in Candolle, Prodr. 12: 640. 1848 syn. sec. Malekmohammadi & al. (2017)

Notes. – *Limonium* sect. *Plathymenium* is characterized by caudex bearing hyaline to brown or black scales, capitate inflorescences and funnel-form calyces with broad limbs, strongly oblique at base (e.g., Boissier 1848; Linczevski 1952). Species of this section occur Central and East Asia and Oceania. The monophyly of this section is well-established (Lledó et al. 2005; Malekmohammadi et al. 2017; Koutroumpa et al. 2018). However, the subdivision of this section into subsections Chrysanthae and Rhodanthae (under *Statice*) proposed by Boissier (1848) on the basis of corolla color (i.e., yellow and reddish, respectively) is not confirmed by the phylogenetic topology (Koutroumpa et al. 2018).

***Limonium aureum* (L.) Chaz. in Miller, Suppl. Dict. Jard. 2: 35. 1790.** Sec. Malekmohammadi & al. (2024)

≡ *Statice aurea* L., Sp. Pl.: 276. 1753 syn. sec. Del Guacchio & al. (2018) ≡ *Limonium aureum* (L.) Hill ex Kuntze, Revis. Gen. Pl. 2: 395. 1891 syn. sec. Del Guacchio & al. (2018); − *Limonium aureum* (L.) Hill in Veg. Syst. 12: 37. 1767, comb. inval. syn. sec. Del Guacchio & al. (2018)
= *Limonium erythrorrhizum* Ikonn.-Gal. ex Lincz. in Novosti Sist. Vyssh. Rast. 8: 211. 1971 syn. sec. Peng & Kamelin (1996)
= *Limonium erythrorrhizum* var. *eriocalyx* Lincz. in Novosti Sist. Vyssh. Rast. 8: 212. 1971 syn. sec. Malekmohammadi & al. (2024)

***Limonium aureum* var. *aureum*.** Sec. Malekmohammadi & al. (2024)

***Limonium aureum* var. *maduoensis* Y.H.Wu in J. Wuhan Bot. Res. 24(4): 323. 2006.** Sec. Wu & Yang (2006)

***Limonium australe* (R.Br.) Kuntze, Revis. Gen. Pl. 2: 395. 1891.** Sec. Malekmohammadi & al. (2024)

≡ *Taxanthema australis* R.Br., Prodr. Fl. Nov. Holland.: 426. 1810 syn. sec. Boissier (1848) ≡ *Statice australis* (R.Br.) Spreng., Syst. Veg., ed. 16, 1: 959. 1824 syn. sec. Malekmohammadi & al. (2024)
= *Statice taxanthema* Schult. in Roemer & Schultes, Syst. Veg. ed. 15[bis] 6: 798. 1820 syn. sec. Boissier (1848)

***Limonium australe* var. *australe*.** Sec. Malekmohammadi & al. (2024)

***Limonium australe* var. *baudinii* (Lincz.) A.M.Gray in Kanunnah 4: 117. 2011.** Sec. Gray, A.M. & Duretto, M.F. (2011)

≡ *Limonium baudinii* Lincz. in Novosti Sist. Vyssh. Rast. 23: 107. 1986 syn. sec. Gray, A.M. & Duretto, M.F. (2011)

***Limonium bicolor* (Bunge) Kuntze, Revis. Gen. Pl. 2: 395. 1891.** Sec. Peng & Kamelin (1996)

≡ *Statice bicolor* Bunge, Enum. Pl. China Bor.: 55. 1833 syn. sec. Peng & Kamelin (1996)
= *Statice bungeana* Boiss. in Candolle, Prodr. 12: 642. 1848 syn. sec. Peng & Kamelin (1996)
= *Statice varia* Hance in J. Bot. 20(238): 290. 1882 syn. sec. Peng & Kamelin (1996)
= *Statice sinensium* Gand. in Bull. Soc. Bot. France 66: 221. 1919 syn. sec. Peng & Kamelin (1996)
= *Statice florida* Kitag. in Bot. Mag. (Tokyo) 48(566): 107. 1934 syn. sec. Peng & Kamelin (1996)

***Limonium chrysocomum* (Kar. & Kir.) Kuntze, Revis. Gen. Pl. 2: 395. 1891.** Sec. Peng & Kamelin (1996)

≡ *Statice chrysocoma* Kar. & Kir. in Bull. Soc. Imp. Naturalistes Moscou 15: 429. 1842 syn. sec. Kuntze (1891) ≡ *Limonium chrysocomum* (Kar. & Kir.) Kuntze subsp. *chrysocomum* syn. sec. Malekmohammadi & al. (2024)
= *Statice schrenkiana* Fisch. & C.A.Mey. in Bull. Cl. Phys.-Math. Acad. Imp. Sci. Saint-Pétersbourg 1: 362. 1843 syn. sec. Peng & Kamelin (1996) ≡ *Limonium schrenkianum* (Fisch. & C.A.Mey.) Kuntze, Revis. Gen. Pl. 2: 396. 1891 syn. sec. Peng & Kamelin (1996)
= *Statice halochrysa* Fisch. ex Boiss. in Candolle, Prodr. 12: 641. 1848 syn. sec. Linczevski (1952)
= *Statice chrysocephala* Regel in Trudy Imp. S.-Peterburgsk. Bot. Sada 6(2): 383. 1880 syn. sec. Peng & Kamelin (1996) ≡ *Limonium chrysocephalum* (Regel) Lincz. in Schischkin & Bobrov, Flora URSS 18: 434. 1952 syn. sec. Peng & Kamelin (1996) ≡ *Limonium chrysocomum* var. *chrysocephalum* (Regel) T.H.Peng, Fl. Reipubl. Popularis Sin. 60(1): 40. 1987 syn. sec. Peng & Kamelin (1996) ≡ *Limonium semenowii* var. *chrysocephalum* (Regel) Grubov in Novon 4(1): 31. 1994 syn. sec. Peng & Kamelin (1996)
= *Statice sedoides* Regel in Trudy Imp. S.-Peterburgsk. Bot. Sada 6(2): 384. 1880 syn. sec. Peng & Kamelin (1996) ≡ *Limonium sedodes* (Regel) Kuntze, Revis. Gen. Pl. 2: 396. 1891 syn. sec. Peng & Kamelin (1996) ≡ *Limonium chrysocomum* var. *sedoides* (Regel) T.H.Peng, Fl. Reipubl. Popularis Sin. 60(1): 40. 1987 syn. sec. Peng & Kamelin (1996) ≡ *Limonium semenowii* var. *sedoides* (Regel) Grubov in Novon 4(1): 31. 1994 syn. sec. Peng & Kamelin (1996)
= *Limonium chrysocomum* var. *pubescens* Lincz. in Schischkin & Bobrov, Flora URSS 18: 432. 1952 syn. sec. Peng & Kamelin (1996)

***Limonium congestum* (Ledeb.) Kuntze, Revis. Gen. Pl. 2: 395. 1891.** Sec. Linczevski (1952)

≡ *Statice congesta* Ledeb., Fl. Altaic. 1: 437. 1829 syn. sec. Kuntze (1891)
= *Limonium iljinii* Sobolevsk. in Bot. Mater. Gerb. Bot. Inst. Komarova Akad. Nauk S.S.S.R. 14: 48. 1951 syn. sec. Linczevski (1952)

***Limonium dichroanthum* (Rupr.) Ikonn.-Gal. ex Lincz. in Schischkin & Bobrov, Flora URSS 18: 428. 1952.** Sec. Peng & Kamelin (1996)

≡ *Statice dichroantha* Rupr. in Mém. Acad. Imp. Sci. Saint Pétersbourg, Sér. 7 14(4): 69. 1869 syn. sec. Peng & Kamelin (1996)

***Limonium dielsianum* (Wangerin) Kamelin in Novon 3(3): 261. 1993.** Sec. Peng & Kamelin (1996)

≡ *Statice dielsiana* Wangerin in Repert. Spec. Nov. Regni Veg. 17: 399. 1921 syn. sec. Peng & Kamelin (1996) ≡ *Limonium aureum* var. *dielsianum* (Wangerin) T.H.Peng, Fl. Reipubl. Popularis Sin. 60(1): 38. 1987 syn. sec. Peng & Kamelin (1996)

***Limonium fischeri* (Trautv.) Lincz. in Schischkin & Bobrov, Flora URSS 18: 427. 1952.** Sec. Malekmohammadi & al. (2024)

≡ *Statice fischeri* Trautv. in Trudy Imp. S.-Peterburgsk. Bot. Sada 2: 481. 1873 syn. sec. Linczevski (1952)
= *Statice nuda* Grossh., Fl. Kavk., ed. 1, 3: 219. 1932 syn. sec. Linczevski (1952)

***Limonium flexuosum* (L.) Chaz. in Miller, Suppl. Dict. Jard. 2: 34. 1790.** Sec. Del Guacchio & al. (2018)

≡ *Statice flexuosa* L., Sp. Pl.: 276. 1753 syn. sec. Del Guacchio & al. (2018) ≡ *Taxanthema flexuosa* (L.) Sweet, Hort. Brit.: 333. 1826 syn. sec. Del Guacchio & al. (2018) − *Limonium flexuosum* (L.) Kuntze, Revis. Gen. Pl. 2: 395. 1891 syn. sec. Del Guacchio & al. (2018) [is later isonym of *Limonium flexuosum* (L.) Chaz.]
= *Statice davurica* Pall., Reise Russ. Reich. 3: 320. 1776 syn. sec. Linczevski (1952)
= *Statice rosea* Pall., Reise Russ. Reich. 3: 260. 1776 syn. sec. Linczevski (1952)

***Limonium franchetii* (Debeaux) Kuntze, Revis. Gen. Pl. 2: 395. 1891.** Sec. Peng & Kamelin (1996)

≡ *Statice franchetii* Debeaux in Actes Soc. Linn. Bordeaux 31: 348. 1876 syn. sec. Kuntze (1891)
= *Statice tchefouensis* Gand. in Bull. Soc. Bot. France 66: 221. 1919 syn. sec. Peng & Kamelin (1996)
= *Limonium subviolaceum* Q.Z.Han & S.D.Zhao, Fl. Pl. Herb. Chin. Bor.-Orient. 7: 255. 1981 syn. sec. Peng & Kamelin (1996)
= *Limonium teretiscaposum* S.D.Zhao, Fl. Pl. Herb. Chin. Bor.-Orient. 7: 255. 1981 syn. sec. Peng & Kamelin (1996)
= *Limonium teretiscaposum* var. *microphyllum* S.D.Zhao, Fl. Pl. Herb. Chin. Bor.-Orient. 7: 255. 1981 syn. sec. Malekmohammadi & al. (2024)

***Limonium gobicum* Ikonn.-Gal. in Trudy Bot. Inst. Akad. Nauk S.S.S.R., ser. 1, Fl. Sist. Vyssh. Rast. 2: 260. 1936.** Sec. Malekmohammadi & al. (2024)

− *Statice gobica* Ikonn.-Gal. in Trudy Bot. Inst. Akad. Nauk S.S.S.R., ser. 1, Fl. Sist. Vyssh. Rast. 2: 260. 1936, nom. inval. syn. sec. Malekmohammadi & al. (2024)

***Limonium grubovii* Lincz. in Bot. Zhurn. (Moscow & Leningrad) 56(11): 1635. 1971.** Sec. Linczevski (1971)

***Limonium hoeltzeri* (Regel) Ikonn.-Gal., Fl. URSS 18: 426. 1952.** Sec. Malekmohammadi & al. (2024)

≡ *Statice hoeltzeri* Regel in Trudy Imp. S.-Peterburgsk. Bot. Sada 5: 259. 1877 syn. sec. Linczevski (1952)
= *Limonium amblyolobum* Ikonn.-Gal. in Trudy Bot. Inst. Akad. Nauk S.S.S.R., ser. 1, Fl. Sist. Vyssh. Rast. 2: 270. 1936 syn. sec. Linczevski (1952) − *Statice amblyoloba* Ikonn.-Gal. in Trudy Bot. Inst. Akad. Nauk S.S.S.R., ser. 1, Fl. Sist. Vyssh. Rast. 2: 270. 1936, nom. inval. syn. sec. Linczevski (1952)
– *Statice tenella* sensu Linczevski (1952), non Regel, err. sec. Malekmohammadi & al. (2024)

***Limonium kaschgaricum* (Rupr.) Ikonn.-Gal. in Trudy Bot. Inst. Akad. Nauk S.S.S.R., ser. 1, Fl. Sist. Vyssh. Rast. 2: 255. 1936.** Sec. Linczevski (1952)

≡ *Statice kaschgarica* Rupr. in Mém. Acad. Imp. Sci. Saint Pétersbourg, Sér. 7 14(4): 69. 1869 syn. sec. Malekmohammadi & al. (2024)

***Limonium klementzii* Ikonn.-Gal. in Trudy Bot. Inst. Akad. Nauk S.S.S.R., ser. 1, Fl. Sist. Vyssh. Rast. 2: 261. 1936.** Sec. Linczevski (1952)

− *Statice klementzii* Ikonn.-Gal. in Trudy Bot. Inst. Akad. Nauk S.S.S.R., ser. 1, Fl. Sist. Vyssh. Rast. 2: 261. 1936, nom. inval. syn. sec. Malekmohammadi & al. (2024)

***Limonium lacostei* (Danguy) Kamelin in Novon 3(3): 261. 1993.** Sec. Peng & Kamelin (1996)

≡ *Statice lacostei* Danguy in J. Bot. (Morot), ser. 2, 1(3): 53. 1908 syn. sec. Malekmohammadi & al. (2024)
= *Limonium roborowskii* Ikonn.-Gal. in Trudy Bot. Inst. Akad. Nauk S.S.S.R., ser. 1, Fl. Sist. Vyssh. Rast. 2: 255. 1936 syn. sec. Peng & Kamelin (1996)
− *Statice roborowskii* Ikonn.-Gal. in Trudy Bot. Inst. Akad. Nauk S.S.S.R., ser. 1, Fl. Sist. Vyssh. Rast. 2: 255. 1936, nom. inval. syn. sec. Peng & Kamelin (1996)

***Limonium leptolobum* (Regel) Kuntze, Revis. Gen. Pl. 2: 395. 1891.** Sec. Linczevski (1952)

≡ *Statice leptoloba* Regel in Trudy Imp. S.-Peterburgsk. Bot. Sada 6(2): 385. 1880 syn. sec. Kuntze (1891)
= *Statice leptoloba* var. *subaphylla* Regel, Fl. XXX: 164. 1881 syn. sec. Linczevski (1952)
− *Statice juncea* Tatarinow ex Wlangali in Beitr. Z. Kenntn. Russ Reich. XX: 251. 1856, nom. nud. syn. sec. Linczevski (1952)

***Limonium michelsonii* Lincz. in Schischkin & Bobrov, Flora URSS 18: 747. 1952.** Sec. Linczevski (1952)

= *Statice leptoloba* Michelson in Tr. pochv.-bot. eksp. Peresel upr. II, 4: 54. 1913 syn. sec. Linczevski (1952)
= *Statice amblyoloba* Popov in Trudy Alma-Atinskogo Gosudastvennogo Zapovednika 3: 37. 1940 syn. sec. Linczevski (1952)

***Limonium nudum* (Boiss. & Buhse) Kuntze, Revis. Gen. Pl. 2: 395. 1891.** Sec. Malekmohammadi & al. (2024)

= *Statice nuda* Boiss. & Buhse in Nouv. Mém. Soc. Imp. Naturalistes Moscou 12: 184. 1860 syn. sec. Kuntze (1891);

***Limonium potaninii* Ikonn.-Gal. in Trudy Bot. Inst. Akad. Nauk S.S.S.R., ser. 1, Fl. Sist. Vyssh. Rast. 2: 256. 1936.** Sec. Peng & Kamelin (1996)

≡ *Limonium aureum* var. *potaninii* (Ikonn.-Gal.) T.H.Peng, Fl. Reipubl. Popularis Sin. 60(1): 38. 1987 syn. sec. Peng & Kamelin (1996)
− *Statice potaninii* Ikonn.-Gal. in Trudy Bot. Inst. Akad. Nauk S.S.S.R., ser. 1, Fl. Sist. Vyssh. Rast. 2: 256. 1936, nom. inval. syn. sec. Malekmohammadi & al. (2024)

***Limonium rezniczenkoanum* Lincz. in Schischkin & Bobrov, Flora URSS 18: 434. 1952.** Sec. Peng & Kamelin (1996)

***Limonium semenowii* (Herder) Kuntze, Revis. Gen. Pl. 2: 396. 1891.** Sec. Malekmohammadi & al. (2024)

≡ *Statice semenovii* Herder in Bull. Soc. Imp. Naturalistes Moscou 41(2): 398. 1868 syn. sec. Kuntze (1891) ≡ *Limonium chrysocomum* var. *semenovii* (Herder) T.H.Peng, Fl. Reipubl. Popularis Sin. 60(1): 40. 1987 syn. sec. Malekmohammadi & al. (2024) ≡ *Limonium chrysocomum* subsp. *semenovii* (Herder) Kamelin in Novon 3(3): 261. 1993 syn. sec. Malekmohammadi & al. (2024)

***Limonium senkakuense* T.Yamaz. in J. Jap. Bot. 66(3): 131. 1991.** Sec. Malekmohammadi & al. (2024)

***Limonium sinense* (Girard) Kuntze, Revis. Gen. Pl. 2: 396. 1891.** Sec. Peng & Kamelin (1996)

≡ *Statice sinensis* Girard in Ann. Sci. Nat., Bot., sér. 3, 2: 329. 1844 syn. sec. Peng & Kamelin (1996)

= *Statice fortuni* Lindl. in Edwards's Bot. Reg. 36: 63. 1845 syn. sec. Boissier (1848) ≡ *Limonium fortunei* (Lindl.) H.Arnaud in Ser., Fl. Jard. 3: 301. 1849 syn. sec. Malekmohammadi & al. (2024) − *Statice fortunei* Lindl., orth. var. syn. sec. Malekmohammadi & al. (2024) [is misspelling for *Statice fortuni* Lindl.]
= *Limonium sinense* var. *spinulosum* Y.Huang in Bull. Bot. Res., Harbin 17(4): 361. 1997 syn. sec. Malekmohammadi & al. (2024)

***Limonium solanderi* Lincz. in Novosti Sist. Vyssh. Rast. 23: 105. 1986.** Sec. Malekmohammadi & al. (2024)

***Limonium tenellum* (Turcz.) Kuntze, Revis. Gen. Pl. 2: 396. 1891.** Sec. Peng & Kamelin (1996)

≡ *Statice tenella* Turcz. in Bull. Soc. Imp. Naturalistes Moscou 5: 203. 1832 syn. sec. Kuntze (1891) ≡ *Limonium tenellum* (Turcz.) Ikonn.-Gal. in Trudy Bot. Inst. Akad. Nauk S.S.S.R., ser. 1, Fl. Sist. Vyssh. Rast. 2: 272. 1936, comb. illeg. syn. sec. Malekmohammadi & al. (2024)

***Limonium tetragonum* (Thunb.) Bullock in Kew Bull. 3(3): 368. 1948.** Sec. Bullock (1948)

≡ *Statice tetragona* Thunb., Prodr. Pl. Cap. 1: 54. 1794 syn. sec. Bullock (1948) ≡ *Taxanthema tetragona* (Thunb.) Sweet, Hort. Brit.: 332. 1826 syn. sec. Malekmohammadi & al. (2024)
= *Statice limonium* Thunb., Fl. Jap.: 129. 1784 syn. sec. Boissier (1848)
= *Statice japonica* Siebold & Zucc., Fl. Jap. Fam. Nat. 2: 72. 1846 syn. sec. Baker (1953) ≡ *Limonium japonicum* (Siebold & Zucc.) Kuntze, Revis. Gen. Pl. 2: 395. 1891 syn. sec. Baker (1953)

Notes. – *Statice limonium* sensu Thunb. is synonym of *Statice japonica* Siebold & Zucc. according to Boissier (Prod. 12: 642. 1848)

***Limonium wrightii* (Hance) Kuntze, Revis. Gen. Pl. 2: 396. 1891.** Sec. Peng & Kamelin (1996)

≡ *Statice wrightii* Hance in Ann. Sci. Nat., Bot., sér. 5 5: 236. 1866 syn. sec. Kuntze (1891)
= *Limonium wrightii* var. *roseum* H.Hara in Acta Bot. Taiwan. 1: 7. 1947 syn. sec. Peng & Kamelin (1996)

***Limonium wrightii* var. *luteum* (H.Hara) H.Hara, Enum. Spermatoph. Jap. 1: 99. 1948.** Sec. Peng & Kamelin (1996)

≡ *Limonium arbusculum* var. *luteum* H.Hara in J. Jap. Bot. 21(1-2): 19. 1947 syn. sec. Peng & Kamelin (1996)

***Limonium wrightii* (Hance) Kuntze var. *wrightii*.** Sec. Peng & Kamelin (1996)

***Limonium wrightii* f. *albescens* Hatus. in Mem. Natl. Sci. Mus. (Tokyo) 7: 118. 1974.** Sec. Malekmohammadi & al. (2024)

***Limonium wrightii* f. *albolutescens* Hatus. in Mem. Natl. Sci. Mus. (Tokyo) 7: 118. 1974.** Sec. Malekmohammadi & al. (2024)

***Limonium wrightii* f. *arbusculum* (Maxim.) Hatus. in Mem. Natl. Sci. Mus. (Tokyo) 7: 118. 1974.** Sec. Malekmohammadi & al. (2024)

≡ *Statice arbuscula* Maxim., Decas. Pl. Nov.: 8. 1882 syn. sec. Malekmohammadi & al. (2024) ≡ *Limonium arbusculum* (Maxim.) Makino, Ill. Fl. Jap.: 228. 1940 syn. sec. Malekmohammadi & al. (2024)

Limonium sect. *Sarcophylla* (Boiss.) Lincz. in Schischkin & Bobrov, Flora URSS 18: 457. 1952 [as " *Sarcophyllum* "]. Sec. Malekmohammadi & al. (2017)

Type: *Limonium axillare* (Forssk.) Kuntze
≡ *Limonium* sect. *Sarcophyllum* (Boiss.) Lincz., orth. var. syn. sec. Malekmohammadi & al. (2024)
= *Statice* subsect. *Sarcophyllae* Boiss. in Candolle, Prodr. 12: 663. 1848 syn. sec. Malekmohammadi & al. (2017)

Notes. – The section comprises Sudano-Zambezian/Saharo-Arabian species that are shrublets with woody caudex and fleshy, mostly cauline leaves, on woody branches, alternate and often spirally arranged. The monophyly and circumscription of this section have been established based on phylogenetic studies by Malekmohammadi et al. (2017) and Koutroumpa et al. (2018).
Limonium sect. *Sarcophylla* is the correct name of this section, opposite to the wrong choice by Linczcevski. When Boissier described his *Statice* sect. *Sarcophyllae*, he based that name on the adjective “sarcophyllus, -a, -um” and correctly chose the feminine plural case to build the sectional name “Sarcophyllae”. Since it is the basionym Linczevski used for his combination in *Limonium*, the name “*L.* sect. *Sarcophyllum*” is orthographically incorrect, since represents the neuter singular case, and therefore it is corrected here to “*L.* sect. *Sarcophylla*”, which is the preceptive neuter plural case for the sectional name (agreeing with the original choice by Boissier).

***Limonium axillare* (Forssk.) Kuntze, Revis. Gen. Pl. 2: 395. 1891.** Sec. Cufodontis (1960)

≡ *Statice axillaris* Forssk., Fl. Aegypt.-Arab.: 58. 1775 syn. sec. Kuntze (1891);
= *Statice attenuata* Schult. in Roemer & Schultes, Syst. Veg. ed. 15[bis] 6: 798. 1820 syn. sec. Cufodontis (1960)
= *Eurychiton adensis* Nimmo, Cat. Pl. Bombay: Addend. 1839 syn. sec. Malekmohammadi & al. (2024) ≡ *Statice adensis* (Nimmo) Boiss. in Candolle, Prodr. 12: 670. 1848 syn. sec. Malekmohammadi & al. (2024)
= *Statice arabica* Jaub. & Spach, Ill. Pl. Orient. 1(9): 156, t. 85. 1844 syn. sec. Cufodontis (1960) ≡ *Limonium arabicum* (Jaub. & Spach) Kuntze, Revis. Gen. Pl. 2: 395. 1891 syn. sec. Malekmohammadi & al. (2024)
= *Statice bovei* Jaub. & Spach, Ill. Pl. Orient. 1(9): 157, t. 86. 1844 syn. sec. Boissier (1848)
= *Statice arabica* f. *glomerata* N.Terrac., (ign.). 1894 syn. sec. Cufodontis (1960) ≡ *Statice axillaris* var. *glomerata* (N.Terrac.) Fiori, (ign.). 1912 syn. sec. Cufodontis (1960)
= *Statice axillaris* f. *attenuata* N.Terrac., (ign.). 1894 syn. sec. Cufodontis (1960) ≡ *Statice axillaris* var. *attenuata* (N.Terrac.) Fiori, (ign.). 1912 syn. sec. Cufodontis (1960)
= *Limonium wendelboi* Bokhari in 40(1): 93. 1982 syn. sec. Akhani & al. (2013)

− *Taxanthema attenuata* R.Br., Voy. Abyss.: Append. p. lxiv as 'Taxantemum attenuatum'. 1814, nom. inval., nom. nud. syn. sec. Boissier (1848)
− *Statice lanceolata* Edgew. in J. Asiat. Soc. Bengal 16(2): 1218. 1847, nom. inval. syn. sec. Malekmohammadi & al. (2024: 14 April 2022)
− *Statice axillaris* var. *ellenbeckii* Engl., (ign.). 1904, nom. nud. syn. sec. Cufodontis (1960)

Notes. – *Statice bovei* Jaub. & Spach is synonym of *Statice axillaris* according to Boissier in Candolle, Prodr. 12: 664 & 673 (1848).

***Limonium cylindrifolium* (Forssk.) Verdc. ex Cufod., Bull. Jard. Bot. État Brux. 30(Suppl.): 661. 1960.** Sec. Cufodontis (1960)

≡ *Statice cylindrifolia* Forssk., Fl. Aegypt.-Arab.: 59. 1775 syn. sec. Cufodontis (1960)
= *Limonium fruticosum* Mill., Gard. Dict., ed. 8: no. 12. 1768 syn. sec. Kuntze (1891)
= *Statice cylindrica* Steud., Nomencl. Bot.: 810, sphalm. 1821 syn. sec. Boissier (1848)
= *Statice teretifolia* Baker ex Oliv. in Bull. Misc. Inform. Kew 93: 334. 1894 syn. sec. Malekmohammadi & al. (2024) ≡ *Limonium teretifolium* (Baker ex Oliv.) Cufod., Bull. Jard. Bot. État Brux. 30(Suppl.): 662. 1960 syn. sec. Malekmohammadi & al. (2024)

***Limonium guigliae* Raimondo & Domina in Pl. Biosystems 143(3): 504. 2009.** Sec. Raimondo & Domina (2009)

***Limonium kossmatii* (R.Wagner & Vierh.) Verdc. & Hemming ex Cufod., Bull. Jard. Bot. Natl. Belg. Suppl. 30(4): 662. 1960.** Sec. Malekmohammadi & al. (2024)

≡ *Statice kossmatii* R.Wagner & Vierh. in Oesterr. Bot. Z. 55: 89. 1905 syn. sec. Cufodontis (1960)

***Limonium maurocordatae* (Schweinf. & Volkens) Cufod., Bull. Jard. Bot. État Brux. 30(Suppl.): 662. 1960.** Sec. Thulin (2006)

≡ *Statice maurocordatae* Schweinf. & Volkens, Liste Pl. Somalis: 9. 1897 syn. sec. Malekmohammadi & al. (2024)
= *Limonium distichum* Wilmot-Dear in Kew Bull. 31: 847. 1976 syn. sec. Thulin (2006)

***Limonium milleri* Ghaz. & J.R.Edm. in Edinburgh J. Bot. 60(1): 15. 2003.** Sec. Malekmohammadi & al. (2024)

***Limonium paulayanum* (Vierh.) Ghaz. & J.R.Edm. in Edinburgh J. Bot. 60(1): 16. 2003.** Sec. Malekmohammadi & al. (2024)

≡ *Statice paulayana* Vierh. in Oesterr. Bot. Z. 55: 89. 1905 syn. sec. Ghazanfar & Edmondson (2003)
= *Statice axillaris* Balf.f. in Trans. Roy. Soc. Edinb. 31: 148. 1888 syn. sec. Ghazanfar & Edmondson (2003)

***Limonium sarcophyllum* Ghaz. & J.R.Edm. in Edinburgh J. Bot. 60(1): 13. 2003.** Sec. Malekmohammadi & al. (2024)

***Limonium sokotranum* (Vierh.) Radcl.-Sm. in Kew Bull. 25(2): 188. 1971.** Sec. Malekmohammadi & al. (2024)

≡ *Statice sokotrana* Vierh. in Oesterr. Bot. Z. 55: 89. 1905 syn. sec. Malekmohammadi & al. (2024)

***Limonium somalorum* (Vierh.) Hutch. & E.A.Bruce in Bull. Misc. Inform. Kew 1941(2): 158. 1942.** Sec. Cufodontis (1960)

≡ *Statice somalorum* Vierh. in in Denkschr. Akad. Wien, Math. Nat. 71: 400. 1907 syn. sec. Malekmohammadi & al. (2024)

***Limonium stocksii* (Boiss.) Kuntze, Revis. Gen. Pl. 2: 396. 1891.** Sec. Malekmohammadi & al. (2024)

≡ *Statice stocksii* Boiss. in Candolle, Prodr. 12: 664. 1848 syn. sec. Kuntze (1891)

***Limonium xipholepis* (Baker) Hutch. & E.A.Bruce in Bull. Misc. Inform. Kew 1941(2): 158. 1942.** Sec. Thulin (2006)

≡ *Statice xipholepis* Baker in Bull. Misc. Inform. Kew 105: 218. 1895 syn. sec. Cufodontis (1960)
= *Statice carinensis* Chiov., Fl. Somala 1: 210. 1929 syn. sec. Cufodontis (1960) ≡ *Limonium carinense* (Chiov.) Verdc. & Hemming ex Cufod. in Bull. Jard. Bot. Natl. Belg. 39(Suppl.): 29. 1969, not defined syn. sec. Thulin (2006)
= *Statice nogalensis* Chiov., Fl. Somala: 211. 1929 syn. sec. Thulin (2006) ≡ *Limonium nogalense* (Chiov.) Verdc. & Hemming ex Cufod. in Bull. Jard. Bot. Natl. Belg. 39(Suppl.): 29. 1969 syn. sec. Thulin (2006)

***Limonium* sect. *Siphonocalyx* Lincz. in Schischkin & Bobrov, Flora URSS 18: 749. 1952.** Sec. Malekmohammadi & al. (2017)

Type: *Limonium sogdianum* (Popov) Ikonn.-Gal.
= *Eremolimon* Lincz. in Novosti Sist. Vyssh. Rast. 22: 200. 1985 syn. sec. Malekmohammadi & al. (2017). Type: *Eremolimon sogdianum* (Ikonn.-Gal.) Lincz.

Notes. – It consists of species from Central Asia growing on gypsum and saline soils and its monophyly has been tested and confirmed by Malekmohammadi et al. (2017; "*Limonium sogdianum* clade").

***Limonium botschantzevii* (Lincz.) M.Malekm., Akhani & Borsch in Taxon 66(5): 1142. 2017.** Sec. Malekmohammadi & al. (2017)

≡ *Eremolimon botschantzevii* Lincz. in Novosti Sist. Vyssh. Rast. 22: 201. 1985 syn. sec. Malekmohammadi & al. (2017)

***Limonium callianthum* (T.X.Peng) Kamelin in Novon 3(3): 262. 1993.** Sec. Peng & Kamelin (1996)

≡ *Limonium drepanostachyum* subsp. *callianthum* T.X.Peng in Guihaia 3(4): 292. 1983 syn. sec. Peng & Kamelin (1996)

***Limonium chodshamumynense* Lincz. & Czukav. in Novosti Sist. Vyssh. Rast. 21: 135. 1984.** Sec. Malekmohammadi & al. (2024)

***Limonium drepanostachyum* Ikonn.-Gal. in Trudy Bot. Inst. Akad. Nauk S.S.S.R., ser. 1, Fl. Sist. Vyssh. Rast. 2: 267. 1936.** Sec. Malekmohammadi & al. (2017)

≡ *Statice drepanostachya* Ikonn.-Gal. in Trudy Bot. Inst. Akad. Nauk S.S.S.R., ser. 1, Fl. Sist. Vyssh. Rast. 2: 267. 1936 syn. sec. Malekmohammadi & al. (2017) ≡ *Eremolimon drepanostachyum* (Ikonn.-Gal.) Lincz. in Novosti Sist. Vyssh. Rast. 22: 203. 1985 syn. sec. Malekmohammadi & al. (2017)
= *Statice flexuosa* O.Fedtsch. & B.Fedtsch., Perech. rast. Turk. 5: 184. 1913 syn. sec. Linczevski (1952)

***Limonium fajzievii* Zakirov ex Lincz. in Schischkin & Bobrov, Flora URSS 18: 748. 1952.** Sec. Malekmohammadi & al. (2024)

≡ *Eremolimon fajzievii* (Zakirov ex Lincz.) Lincz. in Novosti Sist. Vyssh. Rast. 22: 203. 1985 syn. sec. Malekmohammadi & al. (2024)

***Limonium ferganense* Ikonn.-Gal. in Trudy Bot. Inst. Akad. Nauk S.S.S.R., ser. 1, Fl. Sist. Vyssh. Rast. 2: 262. 1936.** Sec. Malekmohammadi & al. (2024)

= *Statice gracilis* O.Fedtch. & B.Fedtsch., Perech. rast. Turk. 5: 183. 1913 syn. sec. Linczevski (1952)
= *Statice ferganensis* Ikonn.-Gal. in Trudy Bot. Inst. Akad. Nauk S.S.S.R., ser. 1, Fl. Sist. Vyssh. Rast. 2: 262. 1936 syn. sec. Malekmohammadi & al. (2024)
= *Limonium komarovii* Ikonn.-Gal. ex Lincz. & Czukav. in Novosti Sist. Vyssh. Rast. 21: 131. 1984 syn. sec. Malekmohammadi & al. (2024) − *Limonium komarovii* Ikonn.-Gal., nom. inval. syn. sec. Malekmohammadi & al. (2024)

***Limonium jarmolenkoi* (Lincz.) M.Malekm., Akhani & Borsch in Taxon 66(5): 1142. 2017.** Sec. Malekmohammadi & al. (2017)

≡ *Eremolimon jarmolenkoi* Lincz. in Novosti Sist. Vyssh. Rast. 22: 203. 1985 syn. sec. Malekmohammadi & al. (2017)

***Limonium kurgantjubense* (Lincz.) M.Malekm., Akhani & Borsch in Taxon 66(5): 1142. 2017.** Sec. Malekmohammadi & al. (2017)

≡ *Eremolimon kurgantjubense* Lincz. in Novosti Sist. Vyssh. Rast. 22: 204. 1985 syn. sec. Malekmohammadi & al. (2017)

***Limonium narynense* Lincz. in Novosti Sist. Vyssh. Rast. 22: 206. 1985.** Sec. Lazkov & Sultanova (2011)

***Limonium ovczinnikovii* Lincz. & Czukav. in Novosti Sist. Vyssh. Rast. 21: 132. 1984.** Sec. Malekmohammadi & al. (2024)

***Limonium piptopodum* Nevski in Trudy Bot. Inst. Akad. Nauk S.S.S.R., ser. 1, Fl. Sist. Vyssh. Rast. 4: 313. 1937.** Sec. Malekmohammadi & al. (2017)

≡ *Eremolimon piptopodum* (Nevski) Lincz. in Novosti Sist. Vyssh. Rast. 22: 206. 1985 syn. sec. Malekmohammadi & al. (2017)

***Limonium sogdianum* (Popov) Ikonn.-Gal. in Trudy Bot. Inst. Akad. Nauk S.S.S.R., ser. 1, Fl. Sist. Vyssh. Rast. 2: 268. 1936.** Sec. Malekmohammadi & al. (2017)

≡ *Statice sogdiana* Popov in Trav. Turkest. Univ., Tashkent 4: 35, in obs. 1922 syn. sec. Malekmohammadi & al. (2024) ≡ *Eremolimon sogdianum* (Ikonn.-Gal.) Lincz. in Novosti Sist. Vyssh. Rast. 22: 206. 1985 syn. sec. Malekmohammadi & al. (2017)
= *Statice spiridonowii* B.Fedtsch. in Trudy. Glavn. Bot. Sada 35: 210. 1921 syn. sec. Linczevski (1952)

Limonium sect. *Sphaerostachys* (Boiss.) Bokhari in Notes Roy. Bot. Gard. Edinburgh 32(1): 59. 1972. Sec. Malekmohammadi & al. (2017)

Type: *Limonium globuliferum* (Boiss. & Heldr. ex Boiss.) Kuntze
≡ *Statice* subsect. *Sphaerostachys* Boiss. in Candolle, Prodr. 12: 664. 1848 syn. sec. Linczevski (1952)

Notes. – A section with four species distributed in Turkey (Inner Anatolia) and Syria, which is characterized by stems without sterile branches, inflorescences of globose or congested spikes and flowers with densely pilose, obconical calyces with ribs terminating well below the margin (Boissier 1848; Bokhari 1972; Bokhari & Edmondson 1982). The section is well-supported as monophyletic and sister to *Limonium* sect. *Limonium* (Malekmohammadi et al. 2017; Koutroumpa et al. 2018).

***Limonium davisii* Doğan in Pl. Syst. Evol. 306: 17. 2020.** Sec. Doğan & al. (2020)

***Limonium globuliferum* (Boiss. & Heldr. ex Boiss.) Kuntze, Revis. Gen. Pl. 2: 395. 1891.** Sec. Malekmohammadi & al. (2024)

≡ *Statice globulifera* Boiss. & Heldr. ex Boiss. in Candolle, Prodr. 12: 665. 1848 syn. sec. Kuntze (1891)

***Limonium lilacinum* (Boiss. & Balansa) Wagenitz in Willdenowia 3(2): 265. 1962.** Sec. Bokhari & Edmondson (1982)

≡ *Statice lilacina* Boiss. & Balansa in Boissier, Diagn. Pl. Orient. ser. 2, 4: 68. 1859 syn. sec. Wagenitz (1962) ≡ *Statice gmelinii* var. *lilacina* (Boiss. & Balansa) Boiss., Fl. Orient. 4(2): 859. 1879 syn. sec. Wagenitz (1962)

***Limonium lilacinum* var. *laxiflorum* Doğan & Akaydın in in Pl. Syst. Evol. 306: 16. 2020.** Sec. Doğan & al. (2020)

***Limonium lilacinum* var. *lilacinum*.** Sec. Doğan & al. (2020)

***Limonium pycnanthum* (K.Koch) Kuntze, Revis. Gen. Pl. 2: 396. 1891.** Sec. Malekmohammadi & al. (2024)

≡ *Statice pycnantha* K.Koch in Linnaea 21: 716. 1848 syn. sec. Kuntze (1891)
= *Statice balansae* Boiss., Diagn. Pl. Orient. ser. 2, 4: 69. 1859 syn. sec. Malekmohammadi & al. (2024)

Limonium subg. *Pterocladus* (Spach) H. Arnaud in Ser., Fl. Jard. 3: 295. 1849. Sec. Lledó & al. (2005)

Lectoype: *Limonium sinuatum* (L.) Mill.

≡ *Statice* subg. *Pterocladus* Spach, Hist. Nat. Vég. 10: 348. 1841 syn. sec. Mabberley & Malécot (2023)
≡ *Limonium* subg. *Pterocladus* (Spach) Pignatti in Bot. J. Linn. Soc. 64(4): 361. 1971 syn. sec. Malekmohammadi & al. (2024)

***Limonium* sect. *Pterocladus* (Spach) Bokhari in Notes Roy. Bot. Gard. Edinburgh 32(1): 59. 1972.** Sec. Lledó & al. (2005)

Type: *Limonium sinuatum* (L.) Mill.
≡ *Statice* subg. *Pterocladus* Spach, Hist. Nat. Vég. 10: 348. 1841 syn. sec. Malekmohammadi & al. (2024)
≡ *Statice* sect. *Pterocladus* (Spach) Boiss. in Candolle, Prodr. 12: 635. 1848 [as " *Pteroclados* "] syn. sec. Malekmohammadi & al. (2024)
≡ *Statice* sect. *Pteroclados* (Spach) Boiss. in Candolle, Prodr. 12: 635. 1848, orth. var. syn. sec. Lledó & al. (2005)

***Limonium* subsect. *Nobilia* (Boiss.) Koutr. in Ecol. Evol. 8(24): 12421. 2018 [as " *Nobiles* "].** Sec. Koutroumpa & al. (2018)

Type: *Limonium arboreum* (Willd.) H. Arnaud
≡ *Statice* subsect. *Nobiles* Boiss. in Candolle, Prodr. 12: 636. 1848 syn. sec. Koutroumpa & al. (2018)
≡ *Limonium* subsect. *Nobiles* (Boiss.) Koutr., orth. var. syn. sec. Koutroumpa & al. (2018)

Notes. – This subsection comprise subshrubs, characterized by spikelets with truncate inner bracts and more inconspicuously winged stems compared to *L.* subsect. *Odontolepidea* (Karis 2004). It consists exclusively of Canarian endemics and forms a well-supported monophyletic group in the phylogeny of Koutroumpa et al. (2018) that sampled all species of this subsection.

***Limonium arboreum* (Willd.) H. Arnaud in Ser., Fl. Jard. 3: 304. 1849.** Sec. Mabberley & Malécot (2023)

≡ *Statice arborea* Willd., Enum. Pl. 1: 337. 1809 syn. sec. Mabberley & Malécot (2023) ≡ *Limonium arborescens* (Brouss.) Kuntze, Revis. Gen. Pl. 2: 395. 1891, nom. illeg. syn. sec. Mabberley & Malécot (2023) − *Limonium arboreum* (Willd.) Erben, A.Santos & Reyes-Bet. in Fl. Medit. 22: 65. 2012, nom. inval. syn. sec. Malekmohammadi & al. (2024) [is later isonym of *Limonium arboreum* (Willd.) H. Arnaud]
= *Statice arborescens* Brouss. ex Webb & Berthel., Hist. Nat. Iles Canaries 3(2): 180. 1846, nom. illeg. syn. sec. Domina (2011+)
= *Statice fruticans* Webb ex Boiss. in Candolle, Prodr. 12: 636. 1848 syn. sec. Domina (2011+) ≡ *Limonium fruticans* (Webb ex Boiss.) Kuntze, Revis. Gen. Pl. 2: 395. 1891, nom. illeg. syn. sec. Domina (2011+)
= *Statice arborea* var. *typica* Stapf in Ann. Bot. (Oxford) 20: 212. 1906 syn. sec. Malekmohammadi & al. (2024)
− *Statice arborescens* Brouss., Elench. Horti Bot. Monspel.: 58. 1805, nom. nud. syn. sec. Domina (2011+)

***Limonium benmageci* Marrero Rodr. in Vieraea 31: 396. 2003.** Sec. Domina (2011+)

***Limonium bourgeaui* (Webb ex Boiss.) Kuntze, Revis. Gen. Pl. 2: 395. 1891.** Sec. Domina (2011+)

≡ *Statice bourgaei* Webb ex Boiss. in Candolle, Prodr. 12: 638. 1848 syn. sec. Kuntze (1891) ≡ *Statice puberula* var. *bourgaei* (Webb ex Boiss.) Stapf in Ann. Bot. (Oxford) 20: 308. 1906 syn. sec. Domina (2011+)

***Limonium brassicifolium* (Webb & Berthel.) Kuntze, Revis. Gen. Pl. 2: 395. 1891.** Sec. Domina (2011+)

≡ *Statice brassicifolia* Webb & Berthel., Hist. Nat. Iles Canaries (Phytogr.) 3: 181. 1846 [as " *brassicaefolia* "] syn. sec. Domina (2011+) − *Statice brassicaefolia* Webb & Berthel., Hist. Nat. Iles Canaries (Phytogr.) 3: 181. 1846, orth. var. syn. sec. Domina (2011+)
− *Statice brassicifolia* var. *typica* Stapf in Ann. Bot. (Oxford) 20: 305. 1906, nom. inval. syn. sec. G.Kunkel & Sunding (1967)

***Limonium ×christii* G.Kunkel, Kanarischen Ins. Pflanzenwelt: 157. 1980.** Sec. G.Kunkel (1980)

Notes. – Hybrid Parentage: *Limonium macrophyllum* (Willd. ex Spreng.) H.Arnaud × *Limonium arboreum* (Willd.) H. Arnaud.

***Limonium frutescens* (Lem.) Erben, A.Santos & Reyes-Bet. in Fl. Medit. 22: 65. 2012.** Sec. Domina (2011+)

≡ *Statice frutescens* Lem., Fl. Serres Jard. Eur. 4: t. 325. 1848 syn. sec. Domina (2011+) ≡ *Statice arborea* f. *frutescens* (Lem.) Stapf. in Ann. Bot. (Oxford) 20: 212. 1906 syn. sec. G.Kunkel & Sunding (1967)

***Limonium imbricatum* (Webb ex Girard) H.Arnaud in Ser., Fl. Jard. 3: 309. 1849.** Sec. Mabberley & Malécot (2023)

≡ *Statice imbricata* Webb ex Girard in Ann. Sci. Nat., Bot., sér. 3, 2: 330. 1844 syn. sec. Mabberley & Malécot (2023) − *Limonium imbricatum* (Webb ex Girard) F.T.Hubb. ex L.H.Bailey in Rhodora 18(211): 158. 1916 syn. sec. Mabberley & Malécot (2023) [is later isonym of *Limonium imbricatum* (Webb ex Girard) H.Arnaud]

***Limonium macrophyllum* (Willd. ex Spreng.) H.Arnaud in Ser., Fl. Jard. 3: 305. 1849.** Sec. Mabberley & Malécot (2023)

≡ *Statice macrophylla* Willd. ex Spreng., Syst. Veg., ed. 16, 1: 959. 1825 syn. sec. Mabberley & Malécot (2023) ≡ *Taxanthema macrophylla* (Willd. ex Spreng.) Sweet, Hort. Brit.: 333. 1826 syn. sec. Malekmohammadi & al. (2024) − *Limonium macrophyllum* (Willd. ex Spreng.) Kuntze, Revis. Gen. Pl. 2: 395. 1891 syn. sec. Mabberley & Malécot (2023) [is later isonym of *Limonium macrophyllum* (Willd. ex Spreng.) H.Arnaud]
= *Statice macrophylla* var. *sinuata* Boiss. in Candolle, Prodr. 12: 637. 1848 syn. sec. Malekmohammadi & al. (2024)
= *Statice halfordii* Hovey, Nursery Cat. 1882: 47. 1882 syn. sec. Malekmohammadi & al. (2024)
= *Statice macrophylla* var. *atrocoerulea* J.R.Duncan & V.C.Davies, Nursery Cat. 1925: (app.) xvi. 1925 syn. sec. Malekmohammadi & al. (2024)
− *Statice holfordii* hort. in Bot. Mag. 107: t. 6537. 1881, nom. nud. syn. sec. G.Kunkel & Sunding (1967)

Limonium macropterum **(Webb & Berthel.) Kuntze, Revis. Gen. Pl. 2: 395. 1891.** Sec. Domina (2011+)

≡ *Statice macroptera* Webb & Berthel., Hist. Nat. Iles Canaries 3: 182. 1846 syn. sec. Kuntze (1891) ≡ *Limonium brassicifolium* subsp. *macropterum* (Webb & Berthel.) G.Kunkel, Kanarischen Ins. Pflanzenwelt: 157. 1980 syn. sec. Domina (2011+) ≡ *Statice brassicifolia* var. *macroptera* (Webb & Berthel.) Burchard syn. sec. Domina (2011+)

Limonium perezii **(Stapf) F.T.Hubb. ex L.H.Bailey in Rhodora 18(211): 158. 1916.** Sec. Domina (2011+)

≡ *Statice perezii* Stapf in Ann. Bot. (Oxford) 22(85): 116. 1908 syn. sec. Bailey (1916)

Limonium preauxii **(Webb & Berthel.) Kuntze, Revis. Gen. Pl. 2: 396. 1891.** Sec. Domina (2011+)

≡ *Statice preauxii* Webb & Berthel., Hist. Nat. Iles Canaries 3: 181. 1846 syn. sec. Kuntze (1891)
= *Statice rumicifolia* Svent. in Bol. Inst. Nac. Invest. Agron. 14(30): 38. 1954 syn. sec. Domina (2011+) ≡ *Limonium rumicifolium* (Svent.) G.Kunkel & Sunding, Cuad. Bot. Cuadernos Bot., Las Palmas 2: 13. 1967 syn. sec. Domina (2011+)

Limonium ×profusum **(hort.) F.T.Hubb. ex L.H.Bailey in Rhodora 18(211): 158. 1916.** Sec. Bailey (1916)

≡ *Statice profusa* hort., cf. Garten 33: 457. 1885 syn. sec. Bailey (1916)
= *Statice ×profusa* T.Moore in Fl. Mag. (London) 1: t. 40. 1861 syn. sec. Malekmohammadi & al. (2024)

Limonium puberulum **(Webb ex Lindl.) H.Arnaud in Ser., Fl. Jard. 3: 306. 1849.** Sec. Mabberley & Malécot (2023)

≡ *Statice puberula* Webb ex Lindl. in Edwards's Bot. Reg. 17: t. 1450. 1831 syn. sec. Mabberley & Malécot (2023) − *Limonium puberulum* (Webb ex Lindl.) Kuntze, Revis. Gen. Pl. 2: 395. 1891 syn. sec. Mabberley & Malécot (2023) [is later isonym of *Limonium puberulum* (Webb ex Lindl.) H.Arnaud]
= *Limonium incertum* H.Arnaud in Ser., Fl. Jard. 3: 306. 1849 syn. sec. Mabberley & Malécot (2023)

Limonium redivivum **(Svent.) G.Kunkel & Sunding, Cuad. Bot. Cuadernos Bot., Las Palmas 2: 12. 1967.** Sec. Domina (2011+)

≡ *Statice rediviva* Svent. in Bol. Inst. Nac. Invest. Agron. 14(30): 35. 1954 syn. sec. G.Kunkel & Sunding (1967)

Limonium redivivum **var.** ***pilosum*** **(Svent.) G.Kunkel & Sunding, Cuad. Bot. Cuadernos Bot., Las Palmas 2: 12. 1967.** Sec. Domina (2011+)

≡ *Statice rediviva* var. *pilosa* Svent. in Bol. Inst. Nac. Invest. Agron. 14(30), fasc. 200: 34. 1954 syn. sec. Malekmohammadi & al. (2024)

Limonium redivivum **var.** ***redivivum*****.** Sec. Domina (2011+)

Limonium relicticum **R.Mesa & A.Santos in Vieraea 29: 112. 2001.** Sec. Domina (2011+)

Limonium spectabile **(Svent.) G.Kunkel & Sunding, Cuad. Bot. Cuadernos Bot., Las Palmas 2: 10. 1967.** Sec. Domina (2011+)

≡ *Statice spectabilis* Svent. in Bol. Inst. Nac. Invest. Agron. 11(20): 204. 1954 syn. sec. G.Kunkel & Sunding (1967)

Limonium sventenii **A.Santos & M.L.Fernández in Anales Jard. Bot. Madrid 40(1): 90. 1983.** Sec. Domina (2011+)

Limonium vigaroense **Marrero Rodr. & R.S.Almeida in Vieraea 31: 393. 2003.** Sec. Domina (2011+)

Limonium subsect. *Odontolepidea* (Boiss.) Sauvage & Vindt in Trav. Inst. Sci. Chérifien, Sér. Bot. 4: 48. 1952. Sec. Sauvage & Vindt (1952)

Type: *Limonium sinuatum* (L.) Mill.
≡ *Statice* subsect. *Odontolepideae* Boiss. in Candolle, Prodr. 12: 635. 1848 syn. sec. Koutroumpa & al. (2018)
≡ *Limonium* subsect. *Odontolepidea* (Boiss.) Koutr. in Ecol. Evol. 8(24): 12421. 2018 [as " *sect. Odontolepideae* "], nom. inval. syn. sec. Malekmohammadi & al. (2024)
≡ *Limonium* sect. *Odontolepideae* (Boiss.) Koutr., nom. inval., orth. var. syn. sec. Malekmohammadi & al. (2024)

Notes. – This subsection is characterized by spikelets with cuspidate inner bracts and usually conspicuously winged stems (Karis 2004). It is a well-supported monophyletic group (Koutroumpa et al. 2018) comprising species endemic to North Africa and species widespread in the circum-Mediterranean area.

Limonium beaumierianum **(Coss. ex Maire) Maire in Mém. Soc. Hist. Nat. Afrique N. 3: 165. 1933.** Sec. Maire (1934)

≡ *Statice beaumieriana* Coss. ex Maire in Bull. Soc. Hist. Nat. Afrique N. 16: 93. 1925 [as " *beaumierana* "] syn. sec. Maire (1934) ≡ *Limonium sinuatum* subsp. *beaumierianum* (Coss. ex Maire) Sauvage & Vindt in Trav. Inst. Sci. Chérifien, Sér. Bot. 4: 50. 1952 syn. sec. Domina (2011+) − *Statice beaumierana* Coss. in Bull. Soc. Bot. France 22: 66. 1875, nom. nud. syn. sec. Maire (1934) − *Statice beaumierana* Coss. ex Maire in Bull. Soc. Hist. Nat. Afrique N. 16: 93. 1925, orth. var. syn. sec. Maire (1934)
= *Statice akkensis* Coss. ex Batt., Contr. Fl. Atl.: 77. 1919 syn. sec. Maire (1934) ≡ *Statice beaumieriana* var. *akkensis* (Coss. ex Batt.) Maire in Bull. Soc. Hist. Nat. Afrique N. 16: 95. 1925 syn. sec. Maire (1934) ≡ *Limonium beaumierianum* var. *akkense* (Coss. ex Batt.) Maire in Jahandiez & al., Cat. Pl. Maroc 3: 568. 1934 syn. sec. Malekmohammadi & al. (2024) ≡ *Limonium sinuatum* var. *akkense* (Coss. ex Batt.) Sauvage & Vindt in Trav. Inst. Sci. Chérifien, Sér. Bot. 4: 50. 1952 syn. sec. Malekmohammadi & al. (2024)
= *Statice beaumieriana* var. *tripeaui* Maire in Bull. Soc. Hist. Nat. Afrique N. 16: 95. 1925 syn. sec. Maire (1934) ≡ *Limonium beaumierianum* var. *tripeaui* (Maire) Maire in Bull. Soc. Hist. Nat. Afrique N. 27: 245. 1936 syn. sec. Malekmohammadi & al. (2024) ≡ *Limonium sinuatum* var. *tripeaui* (Maire) Sauvage & Vindt in Trav. Inst. Sci. Chérifien, Sér. Bot. 4: 51. 1952 syn. sec. Malekmohammadi & al. (2024)

≡ *Statice beaumieriana* f. *leucocalyx* Maire in Bull. Soc. Hist. Nat. Afrique N. 18: 10. 1927 syn. sec. Malekmohammadi & al. (2024) ≡ *Limonium beaumierianum* var. *leucocalyx* (Maire) Maire in Bull. Soc. Hist. Nat. Afrique N. 27: 245. 1936 syn. sec. Emberger & Maire (1941) ≡ *Limonium sinuatum* f. *leucocalyx* (Maire) Sauvage & Vindt in Trav. Inst. Sci. Chérifien, Sér. Bot. 4: 50. 1952 syn. sec. Malekmohammadi & al. (2024)
= *Limonium sinuatum* var. *annuum* Maire in Bull. Soc. Hist. Nat. Afrique N. 24: 222. 1933 syn. sec. Emberger & Maire (1941) ≡ *Limonium beaumierianum* var. *annuum* (Maire) Maire in Bull. Soc. Hist. Nat. Afrique N. 27: 245. 1936 syn. sec. Malekmohammadi & al. (2024) ≡ *Limonium sinuatum* subvar. *annuum* (Maire) Sauvage & Vindt in Trav. Inst. Sci. Chérifien, Sér. Bot. 4: 51. 1952 syn. sec. Malekmohammadi & al. (2024)
= *Limonium beaumierianum* var. *dubium* Maire in Bull. Soc. Hist. Nat. Afrique N. 27: 245. 1936 syn. sec. Malekmohammadi & al. (2024)
= *Limonium beaumierianum* var. *glabrescens* Maire in Bull. Soc. Hist. Nat. Afrique N. 27: 245. 1936 syn. sec. Malekmohammadi & al. (2024) ≡ *Limonium sinuatum* subvar. *glabrescens* (Maire) Sauvage & Vindt in Trav. Inst. Sci. Chérifien, Sér. Bot. 4: 50. 1952 syn. sec. Malekmohammadi & al. (2024)
= *Limonium beaumierianum* var. *violascens* Maire in Bull. Soc. Hist. Nat. Afrique N. 27: 246. 1936 syn. sec. Emberger & Maire (1941)

***Limonium bonduellei* (T.Lestib.) Kuntze, Revis. Gen. Pl. 2: 395. 1891.** Sec. Domina (2011+)

≡ *Statice bonduellei* T.Lestib. in Ann. Sci. Nat., Bot., sér. 3, 16: 81. 1851 [as " *bonduellii* "] syn. sec. Kuntze (1891) ≡ *Limonium sinuatum* subsp. *bonduellei* (T.Lestib.) Sauvage & Vindt in Trav. Inst. Sci. Chérifien, Sér. Bot. 4: 51. 1952 syn. sec. Domina (2011+) − *Statice bonduellii* T.Lestib. in Ann. Sci. Nat., Bot., sér. 3, 16: 81. 1851, orth. var. syn. sec. Domina (2011+)
= *Limonium bonduellei* f. *gigantifolia* Corti in Flora e Vegetazione del Fezzan edella Regione di Gat. Reale Società Geografica Italiana 1: 1-203. 1942 syn. sec. Malekmohammadi & al. (2024)

Notes. – This species was described to honour Mr. Bonduelle. The correct name should be “bonduellei” in any rank.

***Limonium lobatum* (L.f.) Chaz. in Miller, Suppl. Dict. Jard. 2: 36. 1790.** Sec. Domina (2011+)

≡ *Statice lobata* L.f., Suppl. Pl. Suppl. Pl.: 187. 1782 syn. sec. Kuntze (1891) ≡ *Limonium lobatum* (L.f.) Kuntze, Revis. Gen. Pl. 2: 395. 1891 syn. sec. Malekmohammadi & al. (2024)
= *Statice aegyptiaca* Pers., Syn. Pl. 1: 334. 1805 syn. sec. Boissier (1848) ≡ *Taxanthema aegyptiaca* (Pers.) Sweet, Hort. Brit.: 333. 1826 syn. sec. POWO (2017+) − *Taxanthema aegyptiacum* (Pers.) Sweet, Hort. Brit.: 333. 1826, orth. var. syn. sec. Govaerts, R. (ed.) (2023) [is misspelling for *Taxanthema aegyptiaca* (Pers.) Sweet]
= *Statice alata* Willd., Enum. Pl., Suppl.: 15. 1814 syn. sec. Boissier (1848) ≡ *Taxanthema alata* (Willd.) Sweet, Hort. Brit.: 333. 1826 syn. sec. POWO (2017+)
= *Statice tripteris* Poir., Encycl. Suppl. 5: 237. 1817 syn. sec. Boissier (1848)
= *Statice thouinii* Viv., Fl. Libyc. Spec.: 18. 1824 syn. sec. Malekmohammadi & al. (2024) ≡ *Taxanthema thouinii* (Viv.) Sweet, Hort. Brit.: 333. 1826 syn. sec. POWO (2017+) ≡ *Limonium thouinii* (Viv.) Kuntze, Revis. Gen. Pl. 2: 396. 1891 syn. sec. Malekmohammadi & al. (2024) ≡ *Linczevskia thouinii* (Viv.) Tzvelev, Konspekt Fl. Kavkaza 3(2): 283. 2012 syn. sec. Malekmohammadi & al. (2017)
= *Statice cuneata* Sm. ex Link, Handbuch 2: 264. 1831 syn. sec. Boissier (1848)
− *Statice acutifolia* Ehrenb. ex Boiss. in Candolle, Prodr. 12: 636. 1848, pro syn. syn. sec. Boissier (1848)

***Limonium mouretii* (Pit.) Maire in Jahandiez & al., Cat. Pl. Maroc 3: 569. 1934.** Sec. Maire (1934)

≡ *Statice mouretii* Pit., Contrib. Etude Fl. Maroc: 33. 1918 syn. sec. Maire (1934)
= *Limonium mouretii* var. *coloratum* Maire in Bull. Soc. Hist. Nat. Afrique N. 30: 354. 1939 syn. sec. Malekmohammadi & al. (2024)
= *Limonium mouretii* var. *pubicalyx* (Stearn) Emberger, L. & Maire, Cat. Pl. Maroc 4: 1094. 1941 syn. sec. Malekmohammadi & al. (2024)
= *Limonium heterobracteatum* Erben in Mitt. Bot. Staatssamml. München 17: 489. 1981 syn. sec. Domina (2011+)

***Limonium romanum* (Täckh. & Boulos) Domina in Willdenowia 41(1): 131. 2011.** Sec. Domina (2011+)

≡ *Limonium sinuatum* subsp. *romanum* Täckh. & Boulos, Publ. Cairo Univ. Herb. 5: 90. 1974 syn. sec. Domina (2011+)

***Limonium sinuatum* (L.) Mill., Gard. Dict., ed. 8: no. 6. 1768.** Sec. Mill. (1768)

≡ *Statice sinuata* L., Sp. Pl.: 276. 1753 syn. sec. Mill. (1768) ≡ *Taxanthema sinuata* (L.) Sweet, Hort. Brit.: 333. 1826 syn. sec. POWO (2017+) ≡ *Linczevskia sinuata* (L.) Tzvelev, Konspekt Fl. Kavkaza 3(2): 283. 2012 syn. sec. Malekmohammadi & al. (2017) ≡ *Limonium sinuatum* (L.) Mill. subsp. *sinuatum* syn. sec. Malekmohammadi & al. (2024) − *Limonium sinuatum* var. *typicum* Maire in Jahandiez & al., Cat. Pl. Maroc 3: 567. 1934, nom. inval. syn. sec. Emberger & Maire (1941)
= *Limonium africanum* Mill., Gard. Dict., ed. 8: no. 8. 1768 syn. sec. Malekmohammadi & al. (2024)
= *Statice hirsuta* C.Presl in Abh. Königl. Böhm. Ges. Wiss. ser. 5, 3: 535. 1845 syn. sec. Boissier (1848)
= *Statice sinuata* var. *integrifolia* Boiss. in Candolle, Prodr. 12: 635. 1848 syn. sec. POWO (2017+)
= *Statice floribunda* Lem. ex Huber in Wochenschrift 9: 81. 1866 syn. sec. POWO (2017+)
= *Statice sinuata* var. *subglabra* H.Lindb. in Acta Soc. Sci. Fenn., Ser. B, Opera Biol. 1(2): 117. 1932 syn. sec. POWO (2017+) ≡ *Limonium sinuatum* var. *subglabrum* (H.Lindb.) Maire in Jahandiez & al., Cat. Pl. Maroc 3: 567. 1934 syn. sec. Malekmohammadi & al. (2024)
= *Limonium sinuatum* f. *pallidum* Maire, Cat. Pl. Maroc 4: 1093. 1941 syn. sec. Malekmohammadi & al. (2024)

***Limonium sinuatum* var. *candidissimum* (hort.) F.T.Hubb. in Rhodora 18(211): 159. 1916.** Sec. Bailey (1916)

≡ *Statice sinuata* var. *candidissima* hort. syn. sec. Bailey (1916)

***Limonium sinuatum* (L.) Mill. var. *sinuatum*.** Sec. Mill. (1768)

***Limonium* sect. *Tenuiramosa* Koutr. in Ecol. Evol. 8(24): 12420. 2018 [as " *Tenuiramosum* "].** Sec. Koutroumpa & al. (2018)

Type: *Limonium anthericoides* (Schltr.) R.A.Dyer
= *Limonium* sect. *Tenuiramosum* Koutr. syn. sec. Malekmohammadi & al. (2024)

Notes. – Because Tenuiramosum" is a neuter singular adjective, its correct neuter plural form is "Tenuiramosa".

***Limonium anthericoides* (Schltr.) R.A.Dyer in Bull. Misc. Inform. Kew 1932(3): 155. 1932.** Sec. Koutroumpa & al. (2018)

≡ *Statice anthericoides* Schltr. in Bot. Jahrb. Syst 24(3): 450. 1897 syn. sec. Malekmohammadi & al. (2024)

***Muellerolimon* Lincz. in Bot. Zhurn. (Moscow & Leningrad) 67(5): 675. 1982.** Sec. Malekmohammadi & al. (2017)

Type: *Muellerolimon salicorniaceum* (F.Muell.) Lincz.

Notes. – A monotypic genus with *M. salicorniaceum* (F.Muell.) Lincz. as its only representative occurring in western Australia. *Muellerolimon* is a leafless plant with articulate, succulent stems growing in coastal and mangrove habitats (Keighery & Muir 2008). Based on molecular phylogenetic studies, it is accepted as a distinct genus sister to *Bakerolimon* and forming a well-supported clade sister to *Myriolimon* (Malekmohammadi et al. 2017; Koutroumpa et al. 2021). The placement of this genus in the *Goniolimon* clade by Lledó et al. (2005) might have been due to misidentified samples and/or mislabeled sequences (Koutroumpa et al. 2018).

***Muellerolimon salicorniaceum* (F.Muell.) Lincz. in Bot. Zhurn. (Moscow & Leningrad) 67(5): 676. 1982.** Sec. Koutroumpa & al. (2018)

≡ *Statice salicorniacea* F.Muell., Fragm. 11: 7. 1878 syn. sec. Christenh. & Byng (2018) ≡ *Limonium salicorniacea* (F.Muell.) Kuntze, Revis. Gen. Pl. 2: 396. 1891 syn. sec. Malekmohammadi & al. (2024) ≡ *Goniolimon salicorniaceum* (F.Muell.) Christenh. & Byng, Global Fl. 4: 144. 2018 syn. sec. Malekmohammadi & al. (2024)

***Myriolimon* Lledó, Erben & M.B.Crespo in Taxon 54(3): 811. 2005.** Sec. Malekmohammadi & al. (2017)

Type: *Myriolimon ferulaceum* (L.) Lledó, Erben & M.B.Crespo
≡ *Statice* sect. *Myriolepis* Boiss. in Candolle, Prodr. 12: 667. 1848 syn. sec. Lledó & al. (2005)
≡ *Limonium* sect. *Myriolepis* (Boiss.) Sauvage & Vindt in Trav. Inst. Sci. Chérifien, Sér. Bot. 4: 74. 1952 syn. sec. Lledó & al. (2005)
≡ *Limonium* subg. *Myriolepis* (Boiss.) Pignatti in Bot. J. Linn. Soc. 64(4): 361. 1971 syn. sec. Lledó & al. (2005)
≡ *Myriolepis* (Boiss.) Lledó, Erben & M.B.Crespo in Taxon 52(1): 71. 2003, nom. illeg. syn. sec. Lledó & al. (2005)

Notes. – Two species distributed along the central and western coasts of the Mediterranean basin. Initially it was described as *Myriolepis*, but due to the similarity with the earlier validly published generic name *Myrialepis* Becc. (*Arecaceae*), *Myriolepis* was considered an illegitimate homonymous name and replaced by *Myriolimon* (Lledó et al. 2003, 2005). Its monophyly is confirmed in the phylogenetic study of Malekmohammadi et al. (2017) by sampling both species of the genus.

***Myriolimon diffusum* (Pourr.) Lledó, Erben & M.B.Crespo in Taxon 54(3): 811. 2005.** Sec. Lledó & al. (2005)

≡ *Statice diffusa* Pourr. in Mém. Acad. Sci. Toulouse 3: 330. 1788 syn. sec. Malekmohammadi & al. (2024) ≡ *Limonium diffusum* (Pourr.) Kuntze, Revis. Gen. Pl. 2: 395. 1891 syn. sec. Malekmohammadi & al. (2024) ≡ *Myriolepis diffusa* (Pourr.) Lledó, Erben & M.B.Crespo in Taxon 52(1): 71. 2003 syn. sec. Lledó & al. (2005)

***Myriolimon ferulaceum* (L.) Lledó, Erben & M.B.Crespo in Taxon 54(3): 811. 2005.** Sec. Lledó & al. (2005)

≡ *Statice ferulacea* L., Sp. Pl., ed. 2, 1: 396. 1762 syn. sec. Malekmohammadi & al. (2024) ≡ *Limonium ferulaceum* (L.) Chaz. in Miller, Suppl. Dict. Jard. 2: 35. 1790 syn. sec. Malekmohammadi & al. (2024) ≡ *Taxanthema ferulacea* (L.) Sweet, Hort. Brit.: 333. 1826 syn. sec. Malekmohammadi & al. (2024) ≡ *Limonium ferulaceum* (L.) Kuntze, Revis. Gen. Pl. 2: 395. 1891 syn. sec. Malekmohammadi & al. (2024) ≡ *Myriolepis ferulacea* (L.) Lledó, Erben & M.B.Crespo in Taxon 52(1): 71. 2003 syn. sec. Lledó & al. (2005)
= *Statice setigera* Bubani, Fl. Pyren. 1: 197. 1897, nom. illeg. syn. sec. Malekmohammadi & al. (2024)

Notes. – Describing *Statice setigera*, Bubani I1897) included *Statice ferulacea* L. in synonymy, which makes his *S. setigera* illegitimate.

***Neogontscharovia* Lincz. in Bot. Zhurn. (Moscow & Leningrad) 56(11): 1633. 1971.** Sec. Kubitzki (1993)

Type: *Neogontscharovia miranda* (Lincz.) Lincz.
≡ *Acantholimon* sect. *Gontscharovia* Lincz. in Schischkin & Bobrov, Flora URSS 18: 744. 1952 syn. sec. Moharrek & al. (2014)

Notes. – Composed of three species from woody habitats that grow only in Afghanistan and Tadzhikistan (Kubitzki 1993). Its monophyly is not yet tested in phylogenetic studies due to the absence of molecular data for the genus.

***Neogontscharovia mira* (Lincz.) Lincz. in Bot. Zhurn. (Moscow & Leningrad) 56(11): 1634. 1971.** Sec. Linczevski (1971)

≡ *Acantholimon mirum* Lincz. in Schischkin & Bobrov, Flora URSS 18: 743. 1952 syn. sec. Linczevski (1971)

***Neogontscharovia miranda* (Lincz.) Lincz. in Bot. Zhurn. (Moscow & Leningrad) 56(11): 1634. 1971.** Sec. Linczevski (1971)

≡ *Acantholimon mirandum* Lincz. in Schischkin & Bobrov, Flora URSS 18: 742. 1952 syn. sec. Linczevski (1971)

***Neogontscharovia saxifragifolia* (Rech.f. & Köie) Lincz. in Bot. Zhurn. (Moscow & Leningrad) 56(11): 1634. 1971.** Sec. Linczevski (1971)

≡ *Acantholimon saxifragifolium* Rech.f. & Köie in Biol. Skr. 13(4) (Symb. Afgan. 5): 154. 1963 syn. sec. Linczevski (1971)

Popoviolimon Lincz. in Bot. Zhurn. (Moscow & Leningrad) 56(11): 1633. 1971. Sec. Moharrek & al. (2017)

Type: *Popoviolimon turcomanicum* (Popov ex Lincz.) Lincz.

Notes. – Comprising a single species distributed in Iran and Turkmenistan, this group is morphologically and phylogenetically related to *Cephalorhizum* and *Bamiania*, which all together constitute a well-supported clade (Moharrek et al. 2017).

***Popoviolimon turcomanicum* (Popov ex Lincz.) Lincz. in Bot. Zhurn. (Moscow & Leningrad) 56(11): 1633. 1971.** Sec. Malekmohammadi & al. (2024)

≡ *Cephalorhizum turcomanicum* Popov ex Lincz. in Schischkin & Bobrov, Flora URSS 18: 746. 1952 syn. sec. Malekmohammadi & al. (2024)

Notes. – The monophyly of the species was confirmed in the phylogenetic study of Moharrek et al. (2017).
Distribution (general). – Iran, Turkmenistan

Psylliostachys (Jaub. & Spach) Nevski in Trudy Bot. Inst. Akad. Nauk S.S.S.R., ser. 1, Fl. Sist. Vyssh. Rast. 4: 314. 1937. Sec. Moharrek & al. (2017)

Type: *Psylliostachys spicata* (Willd.) Nevski
≡ *Statice* subg. *Psylliostachys* Jaub. & Spach, Ill. Pl. Orient. 1: 158. 1844 syn. sec. Linczevski (1952)

Notes. – Ten annual species, distributed in southwestern and Central Asia (Kubitzki 1993; Celep et al. 2016). In phylogenetic trees the species formed a well-supported clade sister to *Armeria* (Lledó et al. 2001, 2005a; Moharrek et al. 2017; Koutroumpa et al. 2018).

***Psylliostachys ×afghanica* Roshkova in Novosti Sist. Vyssh. Rast. 1965: 214. 1965.** Sec. Malekmohammadi & al. (2024)

Notes. – Psylliostachys is a feminine name.

***Psylliostachys anceps* (Regel) Roshkova in Schischkin & Bobrov, Flora URSS 18 18: 473. 1952.** Sec. Rechinger & Schiman-Czeika (1974)

≡ *Statice anceps* Regel, Descr. Pl. Nov. Rar. Fedtsch.: 74. 1882 syn. sec. Roshkova (1952) ≡ *Limonium anceps* (Regel) Kuntze, Revis. Gen. Pl. 2: 395. 1891 syn. sec. Roshkova (1952)
= *Statice albertii* Regel in Trudy Imp. S.-Peterburgsk. Bot. Sada 9: 615. 1886 syn. sec. Roshkova (1952)

***Psylliostachys ×androssovii* Roshkova in Schischkin & Bobrov, Flora URSS 18 18: 749. 1952.** Sec. Roshkova (1952)

***Psylliostachys beludshistanica* Roshkova in Bot. Mater. Gerb. Bot. Inst. Komarova Akad. Nauk S.S.S.R. 16: 260. 1954.** Sec. Rechinger & Schiman-Czeika (1974)

= *Psylliostachys hymenostegia* Rech.f. in Biol. Skr. 13(4) (Symb. Afgan. 5): 143. 1963 syn. sec. Rechinger & Schiman-Czeika (1974)
= *Psylliostachys koelzii* Rech.f. in Biol. Skr. 13(4) (Symb. Afgan. 5): 145. 1963 syn. sec. Rechinger & Schiman-Czeika (1974)

***Psylliostachys leptostachya* (Boiss.) Roshkova in Schischkin & Bobrov, Flora URSS 18 18: 468. 1952.** Sec. Rechinger & Schiman-Czeika (1974)

≡ *Statice leptostachya* Boiss., Pl. Pers. aust.: 2. 1845 syn. sec. Roshkova (1952) ≡ *Limonium leptostachyus* (Boiss.) Kuntze, Revis. Gen. Pl. 2: 395. 1891 syn. sec. Roshkova (1952) − *Psylliostachys leptostachyus* (Boiss.) Roshkova, nom. inval. syn. sec. Rechinger & Schiman-Czeika (1974)
= *Statice leptostachya* var. *scapis-filiformibus* Aitch. in Trans. Linn. Soc. London, Bot. 3(1): 86. 1888 syn. sec. Malekmohammadi & al. (2024)
= *Statice leptostachya* f. *pomeliana* Rouy in Rev. Bot. Syst. Geogr. Bot. 1(11): 155. 1903 syn. sec. Malekmohammadi & al. (2024)
= *Statice turkestanica* Gand. in Bull. Soc. Bot. France 65: 221. 1919 syn. sec. Roshkova (1952)

***Psylliostachys ×myosuroides* (Regel) Roshkova in Schischkin & Bobrov, Flora URSS 18 18: 470. 1952.** Sec. Roshkova (1952)

≡ *Statice ×myosuroides* Regel, Descr. Pl. Nov. Rar. Fedtsch.: 74. 1882 syn. sec. Roshkova (1952) ≡ *Limonium myosuroides* (Regel) Kuntze, Revis. Gen. Pl. 2: 395. 1891 syn. sec. Roshkova (1952)
= *Statice superba* Regel ex F.Haage & M.Schmidt in Gartenflora 36: 666. 1887 syn. sec. Roshkova (1952) ≡ *Limonium superbum* (Regel) F.T.Hubb. ex L.H.Bailey in Rhodora 18(211): 159. 1916 syn. sec. Roshkova (1952)
= *Statice superba* var. *flora-alba* Benary in Gartenflora 45: 635. 1896 syn. sec. Malekmohammadi & al. (2024) ≡ *Limonium superbum* var. *flore-albo* (Benary) L.H.Bailey in Rhodora 18(211): 159. 1916 syn. sec. Malekmohammadi & al. (2024)
= *Statice superba* Regel ex F.Haage & M.Schmidt in Gartenflora 36: 666. 1887 syn. sec. Roshkova (1952) ≡ *Limonium superbum* (Regel) F.T.Hubb. ex L.H.Bailey in Rhodora 18(211): 159. 1916 syn. sec. Roshkova (1952)

***Psylliostachys spicata* (Willd.) Nevski in Trudy Bot. Inst. Akad. Nauk S.S.S.R., ser. 1, Fl. Sist. Vyssh. Rast. 4: 314. 1937.** Sec. Rechinger & Schiman-Czeika (1974)

= *Statice spicata* Willd., Sp. Pl. 1(2): 1533. 1798 syn. sec. Rechinger & Schiman-Czeika (1974); ≡ *Limonium spicatum* (Willd.) Kuntze, Revis. Gen. Pl. 2: 396. 1891 syn. sec. Rechinger & Schiman-Czeika (1974)
= *Statice lyrata* M.Bieb., Tabl. Prov. Mer Casp.: 114. 1798 syn. sec. Rechinger & Schiman-Czeika (1974)
= *Statice spicata* Hohenacker, Enum. Pl. Talysch: 32. 1838 syn. sec. Malekmohammadi & al. (2024)
= *Statice plantaginiflora* Jaub. & Spach, Ill. Pl. Orient. 1(9): 159, t. 88. 1844 syn. sec. Malekmohammadi & al. (2024) ≡ *Psylliostachys plantaginiflora* (Jaub. & Spach) Roshkova in Bot. Mater. Gerb. Bot. Inst. Komarova Akad. Nauk S.S.S.R. 16: 262. 1954 syn. sec. Malekmohammadi & al. (2024)
= *Statice sisymbriifolia* Jaub. & Spach, Ill. Pl. Orient. 1(9): 158, t. 87. 1844 syn. sec. Rechinger & Schiman-Czeika (1974) ≡ *Psylliostachys sisymbriifolia* (Jaub. & Spach) Rech.f. in Biol. Skr. 13(4) (Symb. Afghan. 5): 146. 1963 syn. sec. Malekmohammadi & al. (2024)
= *Statice spicata* var. *foliis-subintegris* Aitch. in Trans. Linn. Soc. London, Bot. 3(1): 86. 1888 syn. sec. Malekmohammadi & al. (2024)

***Psylliostachys suworowii* (Regel) Roshkova in Schischkin & Bobrov, Flora URSS 18 18: 469. 1952.** Sec. Rechinger & Schiman-Czeika (1974)

≡ *Statice suworowii* Regel in Trudy Imp. S.-Peterburgsk. Bot. Sada 7: 550. 1880 syn. sec. Rechinger & Schiman-Czeika (1974) ≡ *Limonium suworowii* (Regel) Kuntze, Revis. Gen. Pl. 2: 396. 1891 syn. sec. Rechinger & Schiman-Czeika (1974) − *Limonium suwarowii* (Regel) Kuntze, orth. var. syn. sec. Malekmohammadi & al. (2024) [is misspelling for *Limonium suworowii* (Regel) Kuntze]
= *Statice spicata* var. *glabra* Regel in Izv. obsh. ljub. Estv. Antr. Etnogr. 34(2): 74. 1882 syn. sec. Rechinger & Schiman-Czeika (1974)
= *Statice macphersonii* F.Muell., The Chemist and Druggist of Australasia: 10. 1895 syn. sec. Malekmohammadi & al. (2024)
= *Statice suworowii* var. *alba* hort., cf. Garden Chron. ser. 3, 53: 426. 1913 syn. sec. Malekmohammadi & al. (2024) ≡ *Limonium suworowii* var. *album* (hort.) F.T.Hubb. in Rhodora 18(211): 159. 1916 syn. sec. Malekmohammadi & al. (2024)
− *Psylliostachys suvorovii* (Regel) Roshk., Fl. URSS 18: 469. 1952 syn. sec. Malekmohammadi & al. (2024) [is misspelling for *Psylliostachys suworowii* (Regel) Roshkova]
− *Psylliostachys suworowi* (Regel) Roshkova, nom. inval. syn. sec. Rechinger & Schiman-Czeika (1974)

***Psylliostachys volkii* Rech.f. in Biol. Skr. 13(4) (Symb. Afgan. 5): 140. 1963.** Sec. Rechinger & Schiman-Czeika (1974)

Saharanthus M.B.Crespo & Lledó in Bot. J. Linn. Soc. 132: 169. 2000. Sec. Koutroumpa & al. (2018)

Type: *Saharanthus ifniensis* (Caball.) M.B.Crespo & Lledó
≡ *Lerrouxia* Caball. in Trab. Mus. Nac. Ci. Nat., Ser. Bot., 28: 13. 1935, nom. illeg. syn. sec. Crespo & Lledó (2000). Type: *Lerrouxia ifniensis* Caball.
≡ *Caballeroa* Font Quer in Cavanillesia 7: 150. 1935, nom. inval. syn. sec. Crespo & Lledó (2000). Type: *Caballeroa ifniensis* (Caball.) Font Quer

Notes. – Monotypic genus that is segregated from *Limoniastrum* based on phylogenetic results from Lledó et al. (2000), which strongly differs from *Limoniastrum* based on its leaves bright green, smooth, lacking chalk depositions, with open sheath; outer bract equalling in length the inner one; stamens adnate to the petal base (Crespo & Lledó 2000). *Saharanthus ifniensis* (Caball.) M.B.Crespo & Lledó is endemic to the sublittoral areas of southern Morocco and northern Sahara (northwestern Africa), in the Saharan Province of the Saharo-Arabian Region (Crespo & Lledó 2000). It forms a clade together with *Muellerolimon* Lincz., *Bakerolimon* Lincz., and *Myriolimon* Lledó, Erben & M.B.Crespo in molecular phylogenetic trees constructed from nuclear and plastid markers (Koutroumpa et al. 2018).

***Saharanthus ifniensis* (Caball.) M.B.Crespo & Lledó in Bot. J. Linn. Soc. 132: 169. 2000.** Sec. Crespo & Lledó (2000)

≡ *Lerrouxia ifniensis* Caball. in Trab. Mus. Nac. Ci. Nat., Ser. Bot., 28: 15. 1935 syn. sec. Crespo & Lledó (2000) ≡ *Limoniastrum ifniense* (Caball.) Font Quer in Cavanillesia 7: 150. 1935 syn. sec. Crespo & Lledó (2000) − *Caballeroa ifniensis* (Caball.) Font Quer in Cavanillesia 7: 150. 1935, comb. inval. syn. sec. Crespo & Lledó (2000)
= *Limoniastrum malenconianum* Maire in Bull. Soc. Hist. Nat. Afrique N. 26: 129. 1935 syn. sec. Crespo & Lledó (2000)

Distribution (general). – Endemic to the sublittoral areas of southern Morocco and northern Sahara (northwestern Africa), in the Saharan Province of the Saharo-Arabian Region.

Vassilczenkoa Lincz. in Novosti Sist. Vyssh. Rast. 16: 166. 1979. Sec. Moharrek & al. (2017)

Type: *Vassilczenkoa sogdiana* (Lincz.) Lincz.

Notes. – Monotypic genus distributed in Afghanistan and Tadzhikistan. It is morphologically and phylogenetically related to *Chaetolimon* (Moharrek et al. 2017).

***Vassilczenkoa sogdiana* (Lincz.) Lincz. in Novosti Sist. Vyssh. Rast. 16: 166. 1979.** Sec. Malekmohammadi & al. (2024)

≡ *Chaetolimon sogdianum* Lincz. in Trudy Tadzikisk. Bazy 8: 587. 1940 syn. sec. Malekmohammadi & al. (2024) ≡ *Acantholimon sogdianum* (Lincz.) Sennikov, Fl. Uzbekist. 3: 2. 2019 syn. sec. Malekmohammadi & al. (2024)

Plumbagineae **Dumort., Anal. Fam. Pl.: 27. 1829.** Sec. Malekmohammadi & al. (2024)

Type: *Plumbago* Tourn. ex L.
= *Plumbagineae* Bartl., Ord. Nat. Pl.: 127. 1830 syn. sec. Lledó & al. (2001)

Notes. – The tribe comprises four genera: *Ceratostigma*, *Plumbagella*, *Plumbago* and *Dyerophytum* (Kubitzki 1993; Hernández-Ledesma et al. 2015). The species have mostly a pantropical distribution. Koutroumpa et al. (2018) showed that the phylogenetic trees reconstructed from ITS and plastid markers (trnL-F, matK and rbcL) do not confirm monophyly of Plumbago, which is the most species-rich genus in the subfamily. However, lacking an alternative, in this checklist the traditional generic treatment is maintained. Further phylogenetic studies and subsequent taxonomic modifications are required to clarify the phylogenetic circumscriptions of the genera in this subfamily.

Ceratostigma **Bunge, Enum. Pl. China Bor.: 55. 1833.** Sec. Koutroumpa & al. (2018)

Type: *Ceratostigma plumbaginoides* Bunge
= *Valoradia* Hochst. in Flora 25(1): 239. 1842 syn. sec. Kubitzki (1993)

Notes. – Seven accepted species, distributed in Asia, especially in China and the Himalayas; one species in East Africa (Kubitzki 1993). In the phylogeny by Koutroumpa et al. (2018), two representatives of this genus formed a well-supported clade sister to the rest of the *Plumbagineae* genera. The monophyly of the genus was also supported in Zhao et al.'s (2023) phylogenetic inference based on chloroplast genomes of five *Ceratostigma* species.

Ceratostigma abyssinicum **(Hochst.) Asch., Beitr. Fl. Aethiop.: 288. 1867.** Sec. Cufodontis (1969)

≡ *Valoradia abyssinica* Hochst. in Flora 25(1): 239. 1842 syn. sec. POWO (2017+)
= *Plumbago eglandulosa* R.Br., Voy. Abyss.: Append. p. lxiv. 1814 syn. sec. Cufodontis (1969)
= *Plumbago glandulosa* Willd. ex Roem. & Schult., Syst. Veg., ed. 15, 4: 711. 1819 syn. sec. POWO (2017+)
= *Valoradia patula* Hochst. in Flora 25(1): 240. 1842 syn. sec. Cufodontis (1969)
= *Ceratostigma speciosum* Prain in J. Bot. 44: 8. 1906 syn. sec. Malekmohammadi & al. (2024)

Ceratostigma asperrimum **Stapf ex Prain in J. Bot. 44: 6. 1906.** Sec. Malekmohammadi & al. (2024)

= *Ceratostigma stapfianum* Hosseus in Beih. Bot. Centralbl. 28(2): 423. 1911 syn. sec. Malekmohammadi & al. (2024)

Ceratostigma griffithii **C.B.Clarke, Fl. Brit. India 3(9): 481. 1882.** Sec. Zhao & al. (2023)

Notes. – Two individuals of the species were sampled in Zhao et al.'s (2023) phylogenomic study and were recovered as monophyletic with highest support, sister to individuals of *Ceratostigma minus*.

Ceratostigma minus **Stapf ex Prain in J. Bot. 44(1): 7. 1906.** Sec. Peng & Kamelin (1996)

= *Ceratostigma polhillii* hort. ex Bulley, Gard. Chron. ser. 3, 30: 6. 1901 syn. sec. Malekmohammadi & al. (2024)
= *Ceratostigma minus* f. *lasaense* T.X.Peng in Guihaia 3(4): 291. 1983 syn. sec. POWO (2017+)

Ceratostigma plumbaginoides **Bunge, Enum. Pl. China Bor.: 55. 1833.** Sec. Zhao & al. (2023)

≡ *Valoradia plumbaginoides* (Bunge) Boiss. in Candolle, Prodr. 12: 695. 1848 syn. sec. POWO (2017+)
= *Plumbago larpentiae* Lindl. in Gard. Chron. 7: 732. 1847 [as " *larpentae* "] syn. sec. Govaerts, R. (ed.) (2023) − *Plumbago larpentae* Lindl. in Gard. Chron. 7: 732. 1847, orth. var. syn. sec. Malekmohammadi & al. (2024)
= *Ceratostigma plantaginoides* J.W.C.Kirk, Brit. Gard. Fl.: 368, in obs. 1927 syn. sec. POWO (2017+)

Notes. – Two individuals of the species were sampled in Zhao et al.'s (2023) phylogenomic study and were recovered as monophyletic with highest support, sister to *Ceratostigma willmottianum*.

Ceratostigma ulicinum **Prain in J. Bot. 44(1): 7. 1906.** Sec. Zhao & al. (2023)

Notes. – Two individuals of the species were sampled in Zhao et al.'s (2023) phylogenomic study and were recovered as monophyletic with highest support, sister to a clade comprising *Ceratostigma plumbaginoides, C. willmottianum, C. minus* and *C. griffthii*.

Ceratostigma willmottianum **Stapf in Bot. Mag. 140: t. 8591. 1914.** Sec. Zhao & al. (2023)

Notes. – Two individuals of the species were sampled in Zhao et al.'s (2023) phylogenomic study and were recovered as monophyletic with highest support, sister to *Ceratostigma plumbaginoides*.

Dyerophytum **(Lam.) Kuntze, Revis. Gen. Pl. 2: 394. 1891.** Sec. Koutroumpa & al. (2018)

Type: *Dyerophytum africanum* (Lam.) Kuntze
≡ *Vogelia* Lam., Tabl. Encycl. 2: 147. 1792, nom. illeg. syn. sec. Koutroumpa & al. (2018). Type: *Vogelia africana* Lam.

Notes. – A genus with three species of shrubs or subshrubs from India, Arabia, Socotra, and Southern Africa (Kubitzki 1993). In the phylogeny by Koutroumpa et al. (2018), it forms a well-supported clade sister to four *Plumbago* species, yet the type species of *Plumbago* is placed in a separate clade. Christenhusz & Byng (2018) merged species of *Dyerophytum* into *Plumbago* based on an older phylogenetic study by Lledó et al. (2005) that included only two species of *Plumbago* and one species of *Dyerophytum* and none of the species of *Plumbagella*. In addition, despite finding *Plumbago* non-monophyletic, neither Lledó et al. (2005) nor Koutroumpa et al. (2018) suggested the merging of *Dyerophytum* (and *Plumbagella*) into *Plumbago*. The morphological differences between both genera are remarkable, and therefore their separation is maintained in this checklist, awaiting further analyses.

Dyerophytum africanum **(Lam.) Kuntze, Revis. Gen. Pl. 2: 394. 1891.** Sec. Graham (2014)

≡ *Vogelia africana* Lam., Tabl. Encycl. 2: 148. t. 149. 1792 syn. sec. Graham (2014) ≡ *Plumbago africana* (Lam.) Christenh. & Byng, Global Fl. 4: 144. 2018 syn. sec. Malekmohammadi & al. (2024)

= *Plumbago dyerophyta* Christenh. & Byng, Global Fl. 4: 144. 2018 syn. sec. POWO (2017+)

Notes. – Graham 2014: "Species separated from all other *Dyerophytum* based on its geographic range and its very distinct flowers which have a shorter corolla and much broader and more deeply wrinkled calyx lobes. Leaf shape is quite similar to *D. pendulum* but is more cuneate."

***Dyerophytum indicum* (Gibbs ex Wight) Kuntze, Revis. Gen. Pl. 2: 394. 1891.** Sec. Graham (2014)

≡ *Vogelia indica* Gibson ex Wight in Calcutta J. Nat. Hist. 7: 17. 1847 syn. sec. Graham (2014)
= *Vogelia perfoliata* Stocks ex Wight in Calcutta J. Nat. Hist. 7: 17. 1847 syn. sec. Graham (2014)
= *Vogelia arabica* Boiss. in Candolle, Prodr. 12: 696. 1848 syn. sec. Christenh. & Byng (2018) ≡ *Dyerophytum arabicum* (Boiss.) M.R.Almeida, Fl. Maharashtra 3A: 156. 2001 syn. sec. Christenh. & Byng (2018) ≡ *Plumbago arabica* (Boiss.) Christenh. & Byng, Global Fl. 4: 144. 2018 syn. sec. Christenh. & Byng (2018)

***Dyerophytum pendulum* (Balf.f.) Kuntze, Revis. Gen. Pl. 2: 394. 1891.** Sec. Graham (2014)

≡ *Vogelia pendula* Balf.f., Proc. Roy. Soc. Edinb. 12: 76. 1884 syn. sec. Christenh. & Byng (2018) ≡ *Plumbago pendula* (Balf.f.) Christenh. & Byng, Global Fl. 4: 144. 2018 syn. sec. POWO (2017+)

Notes. –

***Dyerophytum socotranum* (Balf.f.) J.R.Edm. ex J.R.Edm., M.Malekm. & Koutr., Phytokeys: 2024.** Sec. Malekmohammadi & al. (2024)

≡ *Vogelia indica* var. *socotrana* Balf.f. in Proc. Roy. Soc. Edinburgh 12: 406. 1884 syn. sec. Malekmohammadi & al. (2024)

Plumbagella Spach, Hist. Nat. Vég. 10: 333. 1841. Sec. Koutroumpa & al. (2018)

Type: *Plumbagella micrantha* (Ledeb.) Spach

Notes. – A monotypic genus with one annual herb species which occurs in Central Asia (Kubitzki 1993). In the phylogenetic studies by Koutroumpa et al. (2018), *Plumbagella* was found in a clade with *Plumbago europaea* L., the type species of *Plumbago*.

***Plumbagella micrantha* (Ledeb.) Spach, Hist. Nat. Vég. 10: 333. 1841.** Sec. Linczevski (1952)

≡ *Plumbago micrantha* Ledeb., Fl. Altaic. 1: 171. 1829 syn. sec. Linczevski (1952)
= *Plumbago spinosa* K.S.Hao in Repert. Spec. Nov. Regni Veg. 36(942-950): 222. 1934 syn. sec. Malekmohammadi & al. (2024)

***Plumbagella micrantha* var. *himalaica* W.W.Sm. in Trans. & Proc. Bot. Soc. Edinburgh 26: 278. 1917.** Sec. POWO (2017+)

***Plumbagella micrantha* var. *micrantha*.** Sec. POWO (2017+)

Plumbago Tourn. ex L., Sp. Pl.: 151. 1753. Sec. Koutroumpa & al. (2018)

Type: *Plumbago europaea* L.
= *Thela* Lour., Fl. Cochinch. 1: 119. 1790 syn. sec. Lindley (1846)
= *Findlaya* Bowdich, Exc. Madeira: 258. 1825 syn. sec. POWO (2017+). Type: *Findlaya alba* Bowdich
= *Plumbago Tourn. ex L. sect. Plumbago* syn. sec. Koutroumpa & al. (2018)
= *Molubda* Raf., Sylva Tellur.: 123. 1838 syn. sec. POWO (2017+). Type: *Molubda scandens* (L.) Raf.
= *Plumbagidium* Spach, Hist. Nat. Vég. 10: 338. 1841 syn. sec. Boissier (1848). Type: *Plumbagidium auriculatum* (Lam.) Spach
= *Plumbago* sect. *Plumbagidium* Boiss. in Candolle, Prodr. 12: 691. 1848 syn. sec. Koutroumpa & al. (2018)

Notes. – A genus of 19 species ("leadworts") with pantropical distribution except for *Plumbago europaea* that is found in temperate regions of Eurasia and North Africa. *Plumbago* is the largest genus in the subfamily *Plumbaginoideae*. Koutroumpa et al. (2018) showed that this genus is polyphyletic and its type species (*P. europaea*) is sister to the monotypic *Plumbagella*, while *Dyerophytum* is sister to the rest of the sampled *Plumbago* taxa. *Plumbago*, *Plumbagella* and *Dyerophytum* are considered as separate genera here but further morphological and phylogenetic studies are necessary to resolve their circumscription.

***Plumbago amplexicaulis* Oliv. in J. Linn. Soc., Bot. 15: 96. 1876.** Sec. POWO (2017+)

***Plumbago aphylla* Bojer ex Boiss. in Candolle, Prodr. 12: 694. 1848.** Sec. POWO (2017+)

= *Plumbago parvifolia* Hemsl. in J. Bot. 54: 362. 1916 syn. sec. POWO (2017+)

***Plumbago auriculata* Lam., Encycl. 2(1): 270. 1786.** Sec. Pignatti (1972)

≡ *Plumbagidium auriculatum* (Lam.) Spach, Hist. Nat. Vég. 10: 339. 1841 syn. sec. POWO (2017+: 11 May 2022) ≡ *Plumbago auriculata* f. *auriculata* syn. sec. Malekmohammadi & al. (2024)
= *Plumbago capensis* Thunb., Prodr. Pl. Cap. 1: 33. 1794 syn. sec. Pignatti (1972)
= *Plumbago grandiflora* Ten., Cat. Orto Nap.: 91. 1845 syn. sec. POWO (2017+)
= *Plumbago capensis* Willd. ex Boiss. in Candolle, Prodr. 12: 694. 1848 syn. sec. Malekmohammadi & al. (2024)
= *Plumbago alba* hort. ex Pasq., Cat. Ort. Bot. Napoli: 82. 1867 syn. sec. POWO (2017+: 11 May 2022) ≡ *Plumbago auriculata* f. *alba* (Pasq.) T.H.Peng, Fl. Reipubl. Popularis Sin. 60(1): 7. 1987 syn. sec. Malekmohammadi & al. (2024)

***Plumbago caerulea* Kunth, Nov. Gen. Sp. (quarto ed.) 2: 220. 1818.** Sec. POWO (2017+)

= *Plumbago humboldtiana* Roem. & Schult., Syst. Veg. ed. 15[bis] 4: 711. 1819 syn. sec. POWO (2017+)
= *Plumbago rhomboidea* Hook. in Bot. Mag. 56: t. 2917. 1829 syn. sec. POWO (2017+)

***Plumbago ciliata* Engl. ex Wilmot-Dear in Kew Bull. 31: 848. 1977.** Sec. POWO (2017+)

Plumbago dawei **Rolfe in J. Linn. Soc., Bot. 37: 522. 1906.** Sec. Friis & al. (2018)

≡ *Plumbago zeylanica* var. *dawei* (Rolfe) Mildbr. in Wiss. Ergebn. Zweit. Deut. Zentr.-Afr. Exped. 2: 518. 1913 syn. sec. Friis & al. (2018)

Plumbago europaea **L., Sp. Pl.: 151. 1753.** Sec. Pignatti (1972)

= *Plumbago purpurea* Salisb., Prodr. Stirp. Chap. Allerton.: 122. 1796 syn. sec. POWO (2017+)
= *Plumbago lapathifolia* Willd., Sp. Pl. 1(2): 837. 1798 syn. sec. Linczevski (1952)
= *Plumbago undulata* Moench, Suppl. Meth.: 153. 1802 syn. sec. POWO (2017+)
= *Plumbago angustifolia* Spach, Hist. Nat. Vég. 10: 337. 1841 syn. sec. Linczevski (1952)
= *Plumbago denticulata* St.-Lag., Étude Fl., ed. 8, 2: 690. 1889 syn. sec. POWO (2017+)

Plumbago glandulicaulis **Wilmot-Dear in Kew Bull. 31: 848. 1977.** Sec. POWO (2017+)

Plumbago hunsbergensis **van Jaarsv., Swanepoel & A.E.van Wyk, Fl. Pl. Africa 62: 89, fig. 2273. 2011.** Sec. POWO (2017+)

Plumbago indica **L., Herb. Amboin.: 24. 1754.** Sec. POWO (2017+)

= *Plumbago rosea* L., Sp. Pl., ed. 2, 1: 215. 1762 syn. sec. POWO (2017+) ≡ *Plumbagidium roseum* (L.) Spach, Hist. Nat. Vég. 10: 339. 1841 syn. sec. POWO (2017+) ≡ *Plumbago zeylanica* var. *rosea* (L.) Williams syn. sec. Malekmohammadi & al. (2024)
= *Thela coccinea* Lour., Fl. Cochinch. 1: 119. 1790 syn. sec. POWO (2017+) ≡ *Plumbago coccinea* Salisb., Prodr. Stirp. Chap. Allerton: 122. 1796 syn. sec. POWO (2017+) ≡ *Plumbago rosea* var. *coccinea* (Lour.) Hook. in Bot. Mag. 89: t. 5363. 1863 syn. sec. Malekmohammadi & al. (2024)

Plumbago ituriensis **Ntore, Fl. Afrique Centr., Plumbagin.: 9. 2015.** Sec. POWO (2017+)

Plumbago madagascariensis **M.Peltier, Fl. Madagasc. 163: 22. 1981.** Sec. POWO (2017+)

Plumbago montis-elgonis **Bullock in Bull. Misc. Inform. Kew 1932(10): 501. 1932.** Sec. Friis & al. (2018)

Plumbago pearsonii **L.Bolus in Ann. Bolus Herb. 3: 7. 1920.** Sec. POWO (2017+)

= *Plumbago suffruticosa* Schinz in Vierteljahrsschr. Naturf. Ges. Zürich 70: 219. 1925 syn. sec. POWO (2017+)

Plumbago pulchella **Boiss. in Candolle, Prodr. 12: 692. 1848.** Sec. POWO (2017+)

= *Plumbago rhomboidea* G.Lodd., Bot. Cab.: t. 1536. 1830 syn. sec. POWO (2017+)
= *Plumbago lanceolata* Sessé & Moc., Fl. Mexic.: 31. 1893 syn. sec. Malekmohammadi & al. (2024)

Plumbago stenophylla **Wilmot-Dear in Kew Bull. 31: 847. 1977.** Sec. POWO (2017+)

Plumbago tristis **W.T.Aiton, Hortus Kew., ed. 2, 1: 324. 1810.** Sec. POWO (2017+)

= *Plumbago vogeliifolia* Eckl. & Zeyh. ex Boiss. in Candolle, Prodr. 12: 694. 1848 [as " *vogeliaefolia* "] syn. sec. POWO (2017+) − *Plumbago vogeliaefolia* Eckl. & Zeyh. ex Boiss. in Candolle, Prodr. 12: 694. 1848, orth. var. syn. sec. POWO (2017+: 11 May 2022)

Plumbago wissii **Friedrich in Senckenberg. Biol. 38: 417. 1957.** Sec. POWO (2017+)

Plumbago zeylanica **L., Sp. Pl.: 151. 1753.** Sec. Rechinger & Schiman-Czeika (1974)

= *Plumbago americana* L., Fl. Jamaic.: 14. 1759 syn. sec. Malekmohammadi & al. (2024)
= *Plumbago scandens* L., Sp. Pl., ed. 2, 1: 215. 1762 syn. sec. POWO (2017+) ≡ *Molubda scandens* (L.) Raf., Sylva Tellur.: 123. 1838 syn. sec. POWO (2017+) ≡ *Plumbagidium scandens* (L.) Spach, Hist. Nat. Vég. 10: 339. 1841 syn. sec. POWO (2017+)
= *Plumbago americana* Weigel, Hort. Gryph.: 12. 1782 syn. sec. POWO (2017+)
= *Thela alba* Lour., Fl. Cochinch. 1: 119. 1790 syn. sec. POWO (2017+)
= *Plumbago sarmentosa* Lam., Tabl. Encycl. 2(2): 470. 1793 syn. sec. POWO (2017+)
= *Plumbago flaccida* Moench, Methodus: 429. 1794 syn. sec. POWO (2017+)
= *Plumbago lactea* Salisb., Prodr. Stirp. Chap. Allerton: 122. 1796 syn. sec. POWO (2017+)
= *Plumbago mexicana* Kunth, Nov. Gen. Sp. (quarto ed.) 2: 221; folio ed.: 179. 1818 syn. sec. POWO (2017+)
= *Plumbago floridana* Nutt. in Amer. J. Sci. Arts 5: 290. 1822 syn. sec. POWO (2017+)
= *Findlaya alba* Bowdich, Exc. Madeira: 258. 1825 syn. sec. Malekmohammadi & al. (2024)
= *Plumbago auriculata* Blume, Bijdr. Fl. Ned. Ind. 14: 736. 1826, nom. illeg. syn. sec. Malekmohammadi & al. (2024)
= *Plumbago occidentalis* Sweet, Hort. Brit.: 333. 1826 syn. sec. POWO (2017+)
= *Plumbago juncea* Bojer, Hortus Maurit.: 263. 1837 syn. sec. POWO (2017+)
= *Plumbago viscosa* Blanco, Fl. Filip. 1: 78. 1837 syn. sec. POWO (2017+)
= *Plumbago floridana* Raf., New Fl. 4: 14. 1838 syn. sec. POWO (2017+)
= *Plumbago virginica* L. in Trans. Linn. Soc. London 20(2): 194. 1847 syn. sec. Malekmohammadi & al. (2024)
= *Plumbago littoralis* Mure, Doctrine Ecole Rio de Janeiro Pathog. Brésil.: 240. 1849 syn. sec. POWO (2017+)
= *Plumbago toxicaria* Bertol., Mem. Acc. Sc. Bolog. 2: 564. 1850 syn. sec. POWO (2017+)
= *Plumbago scandens* var. *densiflora* Kuntze, Revis. Gen. Pl. 2: 396. 1891 syn. sec. POWO (2017+)
= *Plumbago scandens* var. *normalis* Kuntze, Revis. Gen. Pl. 2: 396. 1891 syn. sec. POWO (2017+)
= *Plumbago scandens* f. *erecta* Chodat & Hassl. in Bull. Herb. Boissier, sér. 2, 3(10): 912. 1903 syn. sec. Malekmohammadi & al. (2024)
= *Plumbago scandens* var. *erecta* Chodat & Hassl. in Bull. Herb. Boissier, sér. 2, 3: 912. 1903 syn. sec. POWO (2017+)
= *Plumbago maximowiczii* Gand. in Bull. Soc. Bot. France 66: 221. 1919 syn. sec. POWO (2017+)

Plumbago zeylanica* var. *glaucescens **Boiss. in Candolle, Prodr. 12: 693. 1848.** Sec. Malekmohammadi & al. (2024)

Plumbago zeylanica* var. *oxypetala **Boiss. in Candolle, Prodr. 12: 693. 1848.** Sec. Malekmohammadi & al. (2024)

***Plumbago zeylanica* L. var. *zeylanica*.** Sec. Malekmohammadi & al. (2024)

Unplaced taxa

***Limonium* ×*garciae* Pignatti in Arch. Bot. (Forlì) 31: 98. 1955, nom. inval.** Sec. Pignatti (1955)

The name is invalid due to lack of a latin description, but the taxon is listed in later publications.

***Limonium* ×*virgolsii* f. *pseudovirgatum* Pignatti in Arch. Bot. (Forlì) 31: 97. 1955, nom. inval.** Sec. Erben (1993)

This is the hybrid *L. carregadorense* × *L. connivens* according to Erben (1993), so it cannot be treated under *L.* ×*escarrei* as suggested by Bolos & al. (1990). However, the nothotaxon name was published without a latin description and is thus invalid. [Malekmohammadi & al. 2024]

Unplaced generic subdivisions

***Acantholimon* Boiss. sect. *Acantholimon*.** Sec. Malekmohammadi & al. (2024)

***Acantholimon* sect. *Acmostegia* Bunge, Mém. Acad. Imp. Sci. Saint Pétersbourg Sér. 7, 18(2): 16. 1872.** Sec. Malekmohammadi & al. (2024)

***Acantholimon* sect. *Armeriopsis* Boiss., Diagn. Pl. Orient. ser. 1, 7: 70. 1846.** Sec. Malekmohammadi & al. (2024)

***Acantholimon* sect. *Bromeliopsis* Rech.f. & Schiman-Czeika, Fl. Iranica 108: 31. 1974.** Sec. Malekmohammadi & al. (2024)

***Acantholimon* sect. *Dracogyna* Mobayen, Revis. Taxon. Acanth.: 69. 1964.** Sec. Malekmohammadi & al. (2024)

***Acantholimon* sect. *Glumaria* Boiss. in Candolle, Prodr. 12: 623. 1848.** Sec. Malekmohammadi & al. (2024)

***Acantholimon* sect. *Inermia* Rech.f. & Köie, Dan. Biol. Skr. 13, 4: 149. 1963.** Sec. Malekmohammadi & al. (2024)

***Acantholimon* sect. *Microstegia* Bornm. in Feddes Repert. 8: 547. 1910.** Sec. Malekmohammadi & al. (2024)

***Acantholimon* sect. *Physostegia* Rech.f. & Schiman-Czeika, Fl. Iranica 108: 77. 1974.** Sec. Malekmohammadi & al. (2024)

***Acantholimon* sect. *Platystegia* Rech.f. & Schiman-Czeika, Fl. Iranica 108: 151. 1974.** Sec. Malekmohammadi & al. (2024)

***Acantholimon* sect. *Poicilocephala* Rech.f. & Schiman-Czeika, Fl. Iranica 108: 153. 1974.** Sec. Malekmohammadi & al. (2024)

***Acantholimon* sect. *Pterostegia* Bunge, Mém. Acad. Imp. Sci. Saint Pétersbourg Sér. 7, 18(2): 15. 1872.** Sec. Malekmohammadi & al. (2024)

= *Acantholimon* sect. *Cymaria* Bunge, Mem. Acad. Scienc. Petersbg. Ser. 7, 18(2): 15. 1872 syn. sec. Malekmohammadi & al. (2024)

***Acantholimon* sect. *Pulvinaria* Boiss., Fl. Orient. 4: 824. 1879.** Sec. Malekmohammadi & al. (2024)

***Acantholimon* sect. *Schizostegia* Rech.f. & Schiman-Czeika, Fl. Iranica 108: 76. 1974.** Sec. Malekmohammadi & al. (2024)

***Acantholimon* sect. *Staticopsis* Boiss., Diagn. Pl. Orient. ser. 1, 7: 71. 1846.** Sec. Malekmohammadi & al. (2024)

***Acantholimon* sect. *Stereophylla* Rech.f. & Schiman-Czeika, Fl. Iranica 108: 37. 1974.** Sec. Malekmohammadi & al. (2024)

***Acantholimon* sect. *Tragacanthina* Bunge, Mém. Acad. Imp. Sci. Saint Pétersbourg Sér. 7, 18(2): 54. 1872.** Sec. Malekmohammadi & al. (2024)

***Acantholimon* subsect. *Caryophyllacea* Boiss., Fl. Orient. 4: 824. 1879.** Sec. Malekmohammadi & al. (2024)

***Acantholimon* subsect. *Erythrostoma* Bunge, Mém. Acad. Imp. Sci. Saint Pétersbourg Sér. 7, 18(2): 66. 1872.** Sec. Malekmohammadi & al. (2024)

***Acantholimon* subsect. *Eurystomata* Bunge, Mém. Acad. Imp. Sci. Saint Pétersbourg Sér. 7, 18(2): 58. 1872.** Sec. Malekmohammadi & al. (2024)

***Acantholimon* subsect. *Exacantha* Yıldırım & M.B.Crespo in Phytotaxa 175(2): 81. 2014.** Sec. Malekmohammadi & al. (2024)

***Acantholimon* subsect. *Halophiliacea* Muvaffak & Doğan in Israel J. Pl. Sci. 49(4): 300. 2001.** Sec. Malekmohammadi & al. (2024)

***Acantholimon* subsect. *Microcalycina* (Bunge) Boiss., Fl. Orient. 4: 825. 1879.** Sec. Malekmohammadi & al. (2024)

***Acantholimon* subsect. *Rhodocalycina* Bunge, Mém. Acad. Imp. Sci. Saint Pétersbourg Sér. 7, 18(2): 25. 1872.** Sec. Malekmohammadi & al. (2024)

***Acantholimon* subsect. *Stenostoma* Bunge, Mém. Acad. Imp. Sci. Saint Pétersbourg Sér. 7, 18(2): 55. 1872.** Sec. Malekmohammadi & al. (2024)

***Armeria* sect. *Macrocentron* Boiss. in Candolle, Prodr. 12: 674. 1848.** Sec. Malekmohammadi & al. (2024)

***Armeria* sect. *Plagiobasis* Boiss. in Candolle, Prodr. 12: 677. 1848.** Sec. Malekmohammadi & al. (2024)

***Armeria* subsect. *Astegiae* Boiss. in Candolle, Prodr. 12: 674. 1848.** Sec. Malekmohammadi & al. (2024)

***Armeria* subsect. *Holotricae* Boiss. in Candolle, Prodr. 12: 677. 1848.** Sec. Malekmohammadi & al. (2024)

***Armeria* subsect. *Macrostegiae* Boiss. in Candolle, Prodr. 12: 676. 1848.** Sec. Malekmohammadi & al. (2024)

***Armeria* subsect. *Microstegiae* Boiss. in Candolle, Prodr. 12: 675. 1848.** Sec. Malekmohammadi & al. (2024)

***Armeria* subsect. *Pleurotrichae* Boiss. in Candolle, Prodr. 12: 679. 1848.** Sec. Malekmohammadi & al. (2024)

***Goniolimon* sect. *Tricuspidaria* Lincz., Fl. URSS 18: 745. 1952.** Sec. Malekmohammadi & al. (2024)

***Goniolimon* sect. *Unicuspidaria* Lincz., Fl. URSS 18: 745. 1952.** Sec. Malekmohammadi & al. (2024)

***Goniolimon* subsect. *Platycalyx* Lincz., Fl. URSS 18: 745. 1952.** Sec. Malekmohammadi & al. (2024)

***Goniolimon* subsect. *Stenocalyx* Lincz., Fl. URSS 18: 746. 1952.** Sec. Malekmohammadi & al. (2024)

Names of verified uncertain application

***Limonium brizoides* Brullo ex Erben, Del Guacchio & P.Caputo in Phytotaxa, ser. 3, 369: 187, 191. 2018, ined.** Sec. Brullo

Limonium brizoides Brullo is mentioned as a pro parte synonym of *Limonium densiflorum* and *Limonium pavonianum* by Erben & al. 2018. However, as they indicate, this name was never published, they took it from annotations of herbarium labels. Since they consider Brullo as an eminent scholar, they acknowledged this by citing the name in the synonymy. [Malekmohammadi & al. 2024]

***Limonium ikonnikovii-galitzkyi* A.V.Grebenjuk, Konspekt Fl. Aziatsk. Rossii: 117. 2012, nom. inval.** Sec. Malekmohammadi & al. (2024)

Invalid name (nomen nudum) placed in section Plathyhymenium by Grebenjuk (2012)

***Limonium oleifolium* Mill., Gard. Dict., ed. 8, n. 3. 1768, nom. rej.** Sec. Del Guacchio & al. (2018)

≡ *Statice oleifolia* (Mill.) Scop., Delic. Fl. Faun. Insubr. 1: 24. 1786 syn. sec. Domina (2011+) ≡ *Taxanthema oleifolia* (Mill.) Sweet, Hort. Brit.: 332. 1826 syn. sec. Domina (2011+) ≡ *Limonium oleifolium* Pignatti subsp. *oleifolium* in Tutin & al., Flora Europaea 3 3: 46. 1972 syn. sec. Domina (2011+)

Notes. – The name *L. oleifolium* Mill. has been misapplied for a long time, and commonly referred to as the priority name for *L. virgatum* (Willd.) Fourr. However, the type material is heterogeneous and belong partly to *L. dodartii* (Girard) Kunze and *L. binervosum* (G.E. Sm.) C.E. Salmon. To preserve stability of all concerned names, *L. oleifolium* has been proposed for rejection by Guacchio et al. (2018).

***Limonium plutosianum* Artell.** Sec. Malekmohammadi & al. (2024)

This name was cited in The Plant List (2011), probably coming from the name in Tropicos, but could not be traced any further.
Notes. – This name was cited in The Plant List (2011), probably coming from the name in Tropicos, but could not be traced any further.

***Statice reticulata* Gouan, Herbor. Montpellier: 139, 140, 271. 1793, nom. nud.** Sec. POWO (2017+)

No publication cited, unclear if referring to the earlier (1764) misapplication or to the Linnean name.

Invalid horticultural designations

***Armeria cephalotes* var. *alba* hort. ex F.T.Hubb. in Rhodora 18(211): 158. 1916, nom. nud.** Sec. Malekmohammadi & al. (2024)

− *Statice pseudoarmeria* var. *alba* F.T.Hubb. in Rhodora 18(211): 158. 1916, nom. nud. syn. sec. Malekmohammadi & al. (2024)

***Armeria cephalotes* var. *grandiflora* hort. ex F.T.Hubb. in Rhodora 18(211): 158. 1916, nom. nud.** Sec. Malekmohammadi & al. (2024)

− *Statice pseudoarmeria* var. *grandiflora* F.T.Hubb. in Rhodora 18(211): 158. 1916, nom. nud. syn. sec. Malekmohammadi & al. (2024)

***Armeria cephalotes* var. *rubra* hort. ex F.T.Hubb. in Rhodora 18(211): 158. 1916, nom. nud.** Sec. Malekmohammadi & al. (2024)

− *Statice pseudoarmeria* var. *rubra* F.T.Hubb. in Rhodora 18(211): 158. 1916, nom. nud. syn. sec. Malekmohammadi & al. (2024)

***Armeria cephalotes* var. *splendens* hort. ex F.T.Hubb. in Rhodora 18(211): 158. 1916, nom. nud.** Sec. Malekmohammadi & al. (2024)

− *Statice pseudoarmeria* var. *splendens* (hort.) F.T.Hubb. in Rhodora 18(211): 158. 1916, nom. nud. syn. sec. Malekmohammadi & al. (2024)

***Armeria plantaginea* var. *alba* hort. ex F.T.Hubb. in Rhodora 18(211): 158. 1916, nom. nud.** Sec. Malekmohammadi & al. (2024)

− *Statice plantaginea* var. *alba* F.T.Hubb. in Rhodora 18(211): 157. 1916, nom. nud. syn. sec. Malekmohammadi & al. (2024)

***Armeria plantaginea* var. *gigantea* hort. ex F.T.Hubb. in Rhodora 18(211): 157. 1916, nom. nud.** Sec. Malekmohammadi & al. (2024)

− *Statice plantaginea* var. *gigantea* F.T.Hubb. in Rhodora 18(211): 157. 1916, nom. nud. syn. sec. Malekmohammadi & al. (2024)

***Armeria plantaginea* var. *grandiflora* hort. ex F.T.Hubb. in Rhodora 18(211): 157. 1916, nom. nud.** Sec. Malekmohammadi & al. (2024)

− *Statice plantaginea* var. *grandiflora* F.T.Hubb. in Rhodora 18(211): 157. 1916, nom. nud. syn. sec. Malekmohammadi & al. (2024)

***Armeria plantaginea* var. *rosea* hort. ex F.T.Hubb. in Rhodora 18(211): 158. 1916, nom. nud.** Sec. Malekmohammadi & al. (2024)

− *Statice plantaginea* var. *rosea* F.T.Hubb. in Rhodora 18(211): 158. 1916, nom. nud. syn. sec. Malekmohammadi & al. (2024)

***Armeria plantaginea* var. *rubra* hort. ex F.T.Hubb. in Rhodora 18(211): 158. 1916, nom. nud.** Sec. Malekmohammadi & al. (2024)

− *Statice plantaginea* var. *rubra* F.T.Hubb. in Rhodora 18(211): 158. 1916, nom. nud. syn. sec. Malekmohammadi & al. (2024)

***Armeria plantaginea* var. *splendens* hort. ex F.T.Hubb. in Rhodora 18(211): 158. 1916, nom. nud.** Sec. Malekmohammadi & al. (2024)

− *Statice plantaginea* var. *splendens* F.T.Hubb. in Rhodora 18(211): 158. 1916, nom. nud. syn. sec. Malekmohammadi & al. (2024)

***Limonium eximum* var. *album* F.T.Hubb. in Rhodora 18(211): 158. 1916, nom. nud.** Sec. Malekmohammadi & al. (2024)

− *Statice eximum* var. *alba* hort. ex F.T.Hubb. in Rhodora 18(211): 158. 1916, nom. nud. syn. sec. Malekmohammadi & al. (2024)
− *Statice exima* var. *flore-alba* hort. ex F.T.Hubb. in Rhodora 18(211): 158. 1916, nom. nud. syn. sec. Malekmohammadi & al. (2024)

***Limonium eximum* var. *superbum* F.T.Hubb. in Rhodora 18(211): 158. 1916, nom. nud.** Sec. Malekmohammadi & al. (2024)

− *Statice exima* var. *superba* hort. ex F.T.Hubb. in Rhodora 18(211): 158. 1916, nom. nud. syn. sec. Malekmohammadi & al. (2024)

***Limonium latifolium* var. *album* F.T.Hubb. in Rhodora 18(211): 158. 1916, nom. nud.** Sec. Malekmohammadi & al. (2024)

− *Statice latifolia* var. *alba* hort. ex F.T.Hubb. in Rhodora 18(211): 158. 1916, nom. nud., pro syn. syn. sec. Malekmohammadi & al. (2024)

***Limonium tataricum* var. *coccineum* F.T.Hubb. in Rhodora 18(211): 159. 1916, nom. nud.** Sec. Malekmohammadi & al. (2024)

− *Statice incana* var. *coccinea* hort. ex F.T.Hubb. in Rhodora 18(211): 159. 1916, nom. nud. syn. sec. Malekmohammadi & al. (2024)

***Limonium tataricum* var. *nanum* F.T.Hubb. in Rhodora 18(211): 159. 1916, nom. nud.** Sec. Malekmohammadi & al. (2024)

− *Statice tatarica* var. *nana* hort. ex hort. in Rhodora 18(211): 159. 1916, nom. nud. syn. sec. Malekmohammadi & al. (2024)
− *Statice incana* var. *hybrida-nana* hort. ex F.T.Hubb., nom. nud. syn. sec. Malekmohammadi & al. (2024)

***Plumbago capensis* f. *alba* hort. ex Carrière in Rev. Hort. (Paris) 1888: 285. 1888.** Sec. Malekmohammadi & al. (2024)

***Plumbago tomentosa* var. *pumila* Hook.f., Trans. Linn. Soc. London 20(2): 194. 1847.** Sec. Malekmohammadi & al. (2024)

On p. 262, Hook.f. corrected Plumbago tomentosa Lam.? to Plantago tomentosa Lam.? [IPNI 2000+: 12 May 2022]

***Statice altaica* hort. ex G.Don, Hort. Brit. [Loudon]: 115. 1830.** Sec. Malekmohammadi & al. (2024)

***Statice auriculata* hort. ex Schult. in Roemer & Schultes, Syst. Veg. ed. 15[bis] 6: 799. 1820.** Sec. Malekmohammadi & al. (2024)

***Statice cuneata* hort. ex Schult. in Roemer & Schultes, Syst. Veg. ed. 15[bis] 6: 799. 1820.** Sec. Boissier (1848)

***Statice dentata* hort. ex Schult. in Roemer & Schultes, Syst. Veg. ed. 15[bis] 6: 799. 1820, nom. nud.** Sec. Boissier (1848)

***Statice dicksoniana* hort. ex W.H.Baxter, Suppl. Hort. Brit.: 643. 1850.** Sec. Malekmohammadi & al. (2024)

***Statice grandiflora* hort. Viln. ex Schult. in Roemer & Schultes, Syst. Veg. ed. 15[bis] 6: 777. 1820.** Sec. Malekmohammadi & al. (2024)

***Statice maurocenia* hort. Par. ex Dum.Cours., Bot. Cult. 1: 661. 1802.** Sec. Malekmohammadi & al. (2024)

***Statice montana* var. *alba* F.T.Hubb. in Rhodora 18(211): 157. 1916, nom. nud.** Sec. Malekmohammadi & al. (2024)

− *Armeria alpina* var. *alba* hort. ex F.T.Hubb. in Rhodora 18(211): 157. 1916, nom. nud. syn. sec. Malekmohammadi & al. (2024)

***Statice reinwardtii* hort. ex Lanza, Lav. Reale Ist. Bot. Palermo 3: 51, in obs. 1932.** Sec. Malekmohammadi & al. (2024)

= *Limoniastrum reinwardtii* Lanza, Lav. Ist. Bot. Palermo 3: 51. 1932. in obs syn. sec. Malekmohammadi & al. (2024)

***Statice scoparia* hort. ex E.Vilm., Fl. Pleine Terre ed. 3,: 1098. 1870.** Sec. Malekmohammadi & al. (2024)

Other excluded designations

***Acantholimon assyricum* var. *micracme* Nábelek & Bornm., nom. inval.** Sec. WFO 2019

Misspelling for *A. assyricum* var. *micacme*

***Acantholimon bracteatum* Girard, nom. inval.** Sec. TPL

Erroneous author citation for *A. bracteatum* (Girard) Boiss.

***Armeria alliacea* Griseb., Spic. Fl. Rumel. 2: 296. 1846 ["1844"], nom. inval.** Sec. TPL

Grisebach cites Cav. as the (original) author, so this was not intended as a new name. [Malekmohammadi & al. 2024]

***Armeria ×intermedia* Szafer, nom. inval.** Sec. TPL

A name originally from Tropicos, probably in error for *A. intermedia* (T.Marsson) Szafer (not published as a hybrid).

***Armeria vulgaris* var. *nana* Bolzon, nom. inval.** Sec. Arrigoni (2015)

Arrigoni cites this name, but in Bolzon's publication it does not exist - probably a erroneous citation of *A. alpina* var. *nana* Bolzon. [Berendsohn 2024]

***Statice alliacea* Sm., Fl. Graec. 3: t. 294. 1821.** Sec. IPNI (2000+)

No new name intended, cites *St. alliaceae* Cav. and Willd. - but misapplied the name

***Statice caespitosa* d'Urv. in Mém. Soc. Linn. Paris 4: 606. 1826, nom. inval.** Sec. IPNI (2000+)

d'Urville cites *St. "cespitosa"* Poir. - no new name intended

***Statice echinus* M.Bieb.** Sec. POWO (2017+)

S. echinus Willd. misapplied name for *Acantholimon hohenackeri*, used in TPL as a synonym.

***Statice globulariifolia* DC., Fl. Franç., ed. 3, 5: 379. 1815.** Sec. IPNI (2000+)

Later citation of *Statice globulariifolia* Desf.

***Statice globulariifolia* Webb, Iter Hispan.: 18. 1838.** Sec. IPNI (2000+)

A later citation of *Statice globulariifolia* Desf., no new name intended

***Statice juncea* (Wallr.) F.T.Hubb. ex L.H.Bailey in Rhodora 18(211): 157. 1916.** Sec. POWO (2017+)

In the introduction of the article, Bailey clearly indicated Hubbart as the author of the combination. Moreover, the combination in Bailey's publication is based on *Statice juncacea* Girard, a synonym of *Armeria girardii* (Bernis) Litard [Malekmohammadi & al. 2024]

***Statice rarida* Vis., Stirp. Dalmat. Spec.: 48. 1826, nom. inval.** Sec. TPL

Misspelling for Statice rorida Vis.

***Statice reticulata* Gouan, Fl. Monsp.: 231. 1764, nom. inval.** Sec. Malekmohammadi & al. (2024)

A later citation of the Linnean name (misapplied)

***Statice reticulata* M.Bieb., Fl. Taur.-Caucas. 1: 250. 1808, nom. inval.** Sec. IPNI (2000+)

Nomenclaturally, only a later citation of Linnean name (via Willdenow, Sp. Pl.)

***Statice vulgaris* Druce in Rep. Bot. Soc. Exch. Club Brit. Isles 3(5): 438. 1913.** Sec. IPNI (2000+)

Druce only cites *Statice vulgaris* Hill; no new name or combination intended

Excluded names

***Aegialitis* Trin., Fund. Agrost. (Trinius): 127, t. 9. 1820.** Sec. Malekmohammadi & al. (2024)

Synonym of the genus Rostraria Trin. in the family Poaceae

***Aegialitis tenuis* Trin., Fund. Agrost. (Trinius): 127, t. 9. 1820.** Sec. Malekmohammadi & al. (2024)

Synonym of Rostraria litorea (All.) Holub in the family Poaceae

***Armeria adsurgens* (Torr. ex A.Gray) Kuntze, Revis. Gen. Pl. 2: 432. 1891.** Sec. Malekmohammadi & al. (2024)

Synonym of Phlox adsurgens Torr. ex A.Gray in the family Polemoniaceae
Notes. – Synonym of *Phlox adsurgens* Torr. ex A.Gray in the family Polemoniaceae.

***Armeria amoena* (Sims) Kuntze, Revis. Gen. Pl. 2: 432. 1891.** Sec. Malekmohammadi & al. (2024)

Synonym of Phlox amoena Sims in the family Polemoniaceae

***Armeria bifida* (Beck) Kuntze, Revis. Gen. Pl. 2: 432. 1891.** Sec. Malekmohammadi & al. (2024)

Synonym of Phlox bifida L.C.Beck in the family Polemoniaceae

***Armeria bryoides* (Nutt.) Kuntze, Revis. Gen. Pl. 2: 432. 1891.** Sec. Malekmohammadi & al. (2024)

Synonym of Phlox hoodii subsp. muscoides (Nutt.) Wherry in the family Polemoniaceae

− *Armeria bryodes* (Nutt.) Kuntze, orth. var. syn. sec. TPL

***Armeria canescens* (Torr. & A.Gray) Kuntze, nom. illeg.** Sec. Malekmohammadi & al. (2024)

Synonym of Phlox canescens Torr. & A.Gray in the family Polemoniaceae

***Armeria divaricata* (L.) Kuntze, Revis. Gen. Pl. 2: 432. 1891.** Sec. Malekmohammadi & al. (2024)

Synonym of Phlox divaricata L. in the family Polemoniaceae

***Armeria douglasii* (Hook.) Kuntze, Revis. Gen. Pl. 2: 432. 1891.** Sec. Malekmohammadi & al. (2024)

Synonym of Phlox douglasii Hook. in the family Polemoniaceae

***Armeria floridana* (Benth.) Kuntze, Revis. Gen. Pl. 2: 432. 1891.** Sec. Malekmohammadi & al. (2024)

Synonym of Phlox floridana Benth. in the family Polemoniaceae

***Armeria glaberrima* (L.) Kuntze, Revis. Gen. Pl. 2: 432. 1891.** Sec. Malekmohammadi & al. (2024)

Synonym of Phlox glaberrima L. in the family Polemoniaceae

***Armeria hordii* (Rich.) Kuntze, Revis. Gen. Pl. 2: 432. 1891.** Sec. Malekmohammadi & al. (2024)

Synonym of Phlox hoodii Richardson in the family Polemoniaceae

***Armeria linearifolia* (Hook.) Kuntze, Revis. Gen. Pl. 2: 432. 1891.** Sec. Malekmohammadi & al. (2024)

Synonym of Phlox longifolia Nutt. in the family Polemoniaceae

***Armeria longifolia* (Nutt.) Kuntze, Revis. Gen. Pl. 2: 432. 1891.** Sec. Malekmohammadi & al. (2024)

Based on *Phlox longifolia<i> Nutt., Polemoniacae [IPNI 2000+]*

***<i>Armeria maculata* (L.) Kuntze, Revis. Gen. Pl. 2: 432. 1891, nom. illeg.** Sec. Malekmohammadi & al. (2024)

A synonym of *Phlox maculata* L. in the family Polemoniaceae

***Armeria muscodes* (Nutt.) Kuntze, Revis. Gen. Pl. 2: 432. 1891.** Sec. Malekmohammadi & al. (2024)

Synonym of Phlox hoodii subsp. muscoides (Nutt.) Wherry in the family Polemoniaceae

***Armeria nana* (Nutt.) Kuntze, Revis. Gen. Pl. 2: 432. 1891.** Sec. Malekmohammadi & al. (2024)

Synonym of Phlox nana Nutt. in the family Polemoniaceae

***Armeria ovata* (L.) Kuntze, Revis. Gen. Pl. 2: 432. 1891.** Sec. Malekmohammadi & al. (2024)

Synonym of *Phlox ovata* L. in the family Polemoniaceae

***Armeria paniculata* (L.) Kuntze, Revis. Gen. Pl. 2: 432. 1891.** Sec. Malekmohammadi & al. (2024)

Synonym of Phlox paniculata L. in the family Polemoniaceae

***Armeria pilosa* (L.) Kuntze, Revis. Gen. Pl. 2: 432. 1891.** Sec. Malekmohammadi & al. (2024)

Synonym of Phlox pilosa L. in the family Polemoniaceae

***Armeria reptans* (Michx.) Kuntze, Revis. Gen. Pl. 2: 432. 1891.** Sec. IPNI (2000+)

Synonym of Phlox stolonifera Sims in the family Polemoniaceae

***Armeria richardsonii* (Hook.) Kuntze, Revis. Gen. Pl. 2: 432. 1891.** Sec. Malekmohammadi & al. (2024)

Synonym of Phlox richardsonii Hook. in the family Polemoniaceae

***Armeria roemeriana* (Scheele) Kuntze, Revis. Gen. Pl. 2: 432. 1891.** Sec. Malekmohammadi & al. (2024)

Synonym of Phlox roemeriana Scheele in the family Polemoniaceae

***Armeria sibirica* (L.) Kuntze, Revis. Gen. Pl. 2: 432. 1891.** Sec. Malekmohammadi & al. (2024)

Synonym of Phlox sibirica L. in the family Polemoniaceae

***Armeria speciosa* (Pursh) Kuntze, Revis. Gen. Pl. 2: 432. 1891.** Sec. Malekmohammadi & al. (2024)

Synonym of Phlox speciosa Pursh in the family Polemoniaceae

***Armeria stellaria* (A.Gray) Kuntze, Revis. Gen. Pl. 2: 432. 1891.** Sec. Malekmohammadi & al. (2024)

Synonym of Phlox bifida subsp. stellaria (A.Gray) Wherry in the family Polemoniaceae

***Armeria subulata* (L.) Kuntze, Revis. Gen. Pl. 2: 432. 1891.** Sec. Malekmohammadi & al. (2024)

Synonym of Phlox subulata L. in the family Polemoniaceae

***Plumbago esquirolii* H.Lév. in Repert. Spec. Nov. Regni Veg. 11: 492. 1913.** Sec. Malekmohammadi & al. (2024)

Synonym of *Anisadenia pubescens* Griff. in the family Linaceae [Govaerts & al. 2021]

Unresolved names

***Acantholimon anatolicum* Doğan & Akaydın in Bot. J. Linn. Soc. 140(4): 445. 2002, nom. inval.** Sec. Malekmohammadi & al. (2024)

***Acantholimon coarctatum* Trautv., nom. nud.** Sec. Malekmohammadi & al. (2024)

Notes. – Notes from Bokhari and Edmondson, Fl. of Turkey vol. 7, page 501: "Recorded from 'Turkish Armenia' by Sapozhnikov (80, p. 24). The species does not appear to have been validly published."

***Acantholimon hellwigii* Lincz. & N.I.Akshigitova in Novosti Sist. Vyssh. Rast. 17: 210. 1980.** Sec. Malekmohammadi & al. (2024)

***Acantholimon hellwigii* var. *escaposum* Lincz. & N.I.Akshigitova in Novosti Sist. Vyssh. Rast. 17: 210. 1980.** Sec. Malekmohammadi & al. (2024)

***Acantholimon pulchellum* f. *condensatum* Korovin in Bot. Mater. Gerb. Glavn. Bot. Sada R.S.F.S.R. 3: 191. 1922.** Sec. Malekmohammadi & al. (2024)

***Acantholimon spinosum* Rob., Gard. Chron. 96: 31. 1934.** Sec. Malekmohammadi & al. (2024)

***Armeria arctica* Sternb., ign., nom. illeg.** Sec. TROPICOS (1988+)

A name from Tropicos, no publication was found.

***Armeria major* (Jacq.) Grande in Bull. Orto Bot. Regia Univ. Napoli 8: 19. 1926.** Sec. Malekmohammadi & al. (2024)

***Armeria maritima* f. *onsiensis* Bernis in Anales Inst. Bot. Cavanilles 14: 362. 1957, nom. inval.** Sec. Nieto Feliner (1987)

***Armeria maritima* var. *genuina* Gren & Godr., Fl. France 2: 733. 1853.** Sec. Daveau (1888)

***Armeria scorzonerifolia* Balb. & Nocca, Fl. Ticin. 1: t. 6. 1816.** Sec. Malekmohammadi & al. (2024)

***Plumbago cocanex* hort. ex Gentil, Pl. Cult. Serres Jard. Bot. Brux. 157. 1907.** Sec. APG [Angiosperm Phylogeny Group] (2009)

***Plumbago coerulea* Auct., not _stated.** Sec. APG [Angiosperm Phylogeny Group] (2009)

***Plumbago rhombifolia* Steud., Nomencl. Bot., ed. 2. ii. 357, sphalm.** Sec. Malekmohammadi & al. (2024)

***Statice algeriensis* Rouy in Rev. Bot. Syst. Geogr. Bot. 1(11): 157. 1903.** Sec. Malekmohammadi & al. (2024)

***Statice armeria* var. *variegata* F.T.Hubb. in Rhodora 18(211): 157. 1916, nom. nud.** Sec. Malekmohammadi & al. (2024)

− *Armeria maritima* var. *variegata* hort. ex F.T.Hubb. in Rhodora 18(211): 157. 1916, nom. nud. syn. sec. Bailey (1916)

***Statice bracteosa* Viv., Pl. Aegypt. Dec.: 16. 1836.** Sec. Malekmohammadi & al. (2024)

***Statice depressa* Bubani, Fl. Pyren. (Bubani) 1: 194. 1897.** Sec. Malekmohammadi & al. (2024)

***Statice drepanense* Tineo ex Guss., Fl. Sicul. Syn. 2: 805. 1845.** Sec. Malekmohammadi & al. (2024)

Notes. – Sometimes treated as a synonym of *Limonium vulgare*, but the protologue does not support this.

***Statice foliosa* Penny ex W.H.Baxter, Hort. Brit., ed. 3: 675. 1839.** Sec. Malekmohammadi & al. (2024)

***Statice frondosa* Lojac., Fl. Sicul. 2(2): 25. 1907.** Sec. Malekmohammadi & al. (2024)

***Statice gougetiana* Reverch., nom. inval.** Sec. Malekmohammadi & al. (2024)

***Statice ×gracillima* (J.J.Rodr.) Rouy in Rev. Bot. Syst. Geogr. Bot. 1(12): 181. 1904.** Sec. Malekmohammadi & al. (2024)

≡ *Statice virgata* var. *gracillima* J.J.Rodr., Fl. Menorca: 111. 1904 syn. sec. Malekmohammadi & al. (2024)

***Statice leucocoleum* Stapf & Wettst. ex Stapf in Denkschr. Kaiserl. Akad. Wiss., Wien. Math.-Naturwiss. Kl. 2: 42. 1886.** Sec. Malekmohammadi & al. (2024)

***Statice longearistata* Font Quer & Rothm., Sched. Fl. Iber Select., Cent. 2-3: 169. 1935.** Sec. Malekmohammadi & al. (2024)

***Statice maravignae* Tineo ex Lojac., Fl. Sicul. 2(2): 20. 1907.** Sec. Malekmohammadi & al. (2024)

***Statice micrantha* Lafont in Actes Soc. Linn. Bordeaux 27: 159. t. 9. f. 10. 1869.** Sec. Malekmohammadi & al. (2024)

***Statice minuta* Falk, Beytr. Topogr. Kenntn. Russ. Reiches 2: 153. 1786, nom. illeg.** Sec. Govaerts, R. (ed.) (2023)

***Statice minuta* Willk., Strand-Steppengeb. Iber. Halbins. [Thes.]: 137. 1852, nom. inval.** Sec. Govaerts, R. (ed.) (2023)

***Statice mongolica* Fisch. ex Girard in Ann. Sci. Nat., Bot., ser. 3, 2: 329. 1844.** Sec. Malekmohammadi & al. (2024)

***Statice nana* Penny ex G.Don, Hort. Brit. ed. 2, : 601. 1832.** Sec. Boissier (1848)

***Statice neglecta* Andrz., Rys. Bot. Wilno: 48. 1823.** Sec. Malekmohammadi & al. (2024)

***Statice nicotrae* Lojac. in Boll. Reale Orto Bot. Palermo 5: 101. 1906.** Sec. Malekmohammadi & al. (2024)

***Statice ocimifolia* Poir.** Sec. Malekmohammadi & al. (2024)

***Statice ocimifolia* Schult. in Roemer & Schultes, Syst. Veg., ed. 15, 6: 780. 1820.** Sec. Malekmohammadi & al. (2024)

***Statice ×pinto-silvae* Rothm., Broteria, Sér. Trimestr. 9: 11. 1940.** Sec. Malekmohammadi & al. (2024)

***Statice polianthemum* Neck., Delic. Gallo-Belg. 1: 160. 1768.** Sec. Malekmohammadi & al. (2024)

***Statice preciosa* Tchertkoff in Bull. Soc. Imp. Naturalistes Moscou 6: 15. 1833.** Sec. Malekmohammadi & al. (2024)

***Statice pumila* Gasp. ex Lojac., Fl. Sicul. 2(2): 20. 1907.** Sec. Malekmohammadi & al. (2024)

***Statice spathulata* Nyman, Syll. Fl. Eur.: 143. 1855.** Sec. Malekmohammadi & al. (2024)

***Statice stricta* Gueldenst., Reis. Russland, 2: 225; ex Ledeb. Fl. Ross. 3: 470.** Sec. Malekmohammadi & al. (2024)

***Statice tetragona* Drège ex Boiss. in Candolle, Prodr. 12: 659. 1848.** Sec. Malekmohammadi & al. (2024)

Notes. – This name was included by Boissier (1848) in synonymy of *Limonium linifolium*, but further check is needed before full amd certain synonymisation.

***Statice trinervia* Schult. in Roemer & Schultes, Syst. Veg., ed. 15, 6: 799. 1820.** Sec. Malekmohammadi & al. (2024)

Index to scientific names

Acantholimon ×baubaschatense Lazkov 5
Acantholimon acanthobryum Rech.f. & Schiman-Czeika 3
Acantholimon acerosum (Willd.) Boiss. 3
Acantholimon acerosum subsp. *acerosum* (Willd.) Boiss. 3
Acantholimon acerosum subsp. *brachystachyum* (Boiss.) Doğan & Akaydın 3
Acantholimon acerosum subsp. *longibracteolatum* Doğan & Akaydın 3
Acantholimon acerosum var. *acerosum* (Willd.) Boiss. ... 3
Acantholimon acerosum var. *brachystachyum* Boiss. 3
Acantholimon acerosum var. *parvifolium* Bokhari 3
Acantholimon acerosum var. *persicum* Mobayen 3
Acantholimon acerosum var. *pinardii* (Boiss.) Mobayen 3
Acantholimon acmostegium Boiss. & Buhse 3
Acantholimon aegaeum F.K.Mey. 3
Acantholimon afanassievii Lincz. 3
Acantholimon agropyroideum Mobayen 3
Acantholimon ahangarense Rech.f. & Schiman-Czeika .. 3
Acantholimon akaydinii Özüdoğru 3
Acantholimon alaicum Czerniak. 3
Acantholimon alatavicum Bunge 3
Acantholimon alatavicum O.Fedtsch. & B.Fedtsch. .. 3, 13
Acantholimon alatavicum var. *korolkowi* Regel 11
Acantholimon alatavicum var. *laevigatum* T.X.Peng 11
Acantholimon alatavicum var. *puberulum* Bunge ex Regel 18
Acantholimon alatavicum var. *subsessile* (Trautv.) Herder 3
Acantholimon alatavicum var. *typicum* Regel 3
Acantholimon alavae Rech.f. & Schiman-Czeika 3
Acantholimon albanicum O.Schwarz & F.K.Mey. 3
Acantholimon alberti Regel 4
Acantholimon albertii Regel 3
Acantholimon albocalycinum Assadi & Mirtadz. 4
Acantholimon alexandri Fed. 4
Acantholimon alexeenkoanum Czerniak. ex Ikonn. 4
Acantholimon amoenum Rech.f. & Schiman-Czeika 4
Acantholimon anatolicum Doğan & Akaydın 100
Acantholimon anatolicum Yıld. 4
Acantholimon androsaceum (Jaub. & Spach) Boiss. 17
Acantholimon androsaceum subsp. *lycaonicum* (Boiss. & Heldr.) Bokhari 12
Acantholimon androsaceum var. *androsaceum* (Jaub. & Spach) Boiss. 17
Acantholimon androsaceum var. *creticum* (Boiss.) Critop. 17
Acantholimon androsaceum var. *creticum* Boiss. 17
Acantholimon androsaceum var. *latifolium* Boiss. 17
Acantholimon androsaceum var. *majus* Boiss. 17
Acantholimon androsaceum var. *olympicum* Boiss. 17
Acantholimon androsaceum var. *purpurascens* Bokhari 17
Acantholimon anisophyllum Rech.f. & Schiman-Czeika .. 4
Acantholimon annae Lincz. 4
Acantholimon antilibanoticum Mouterde 4
Acantholimon anzobicum Lincz 4
Acantholimon anzobicum var. *albiflorum* Lincz. 4
Acantholimon ararati hort. ex Jahand. 9
Acantholimon araxanum Bunge 4
Acantholimon argyrostachyum Rech.f. & Schiman-Czeika 4
Acantholimon aristulatum Bunge 4
Acantholimon armenum Boiss. & A.Huet 4
Acantholimon armenum var. *armenum* Boiss. & A.Huet 4
Acantholimon armenum var. *balansae* Boiss. & A.Huet . 4
Acantholimon armenum var. *balansae* Kusn. 4
Acantholimon armenum var. *puberulum* Trautv. 4
Acantholimon armenum var. *typicum* Trautv. 4
Acantholimon artosense Doğan & Akaydın 4
Acantholimon arundoscapum Mobayen 4
Acantholimon aspadanum Bunge 4
Acantholimon asphodelinum Mobayen 4
Acantholimon assadii Mirtadz. & Bordbar 4
Acantholimon assyriacum Boiss. 18
Acantholimon assyriacum var. *micacme* Nábělek & Bornm. 3
Acantholimon assyricum var. *micracme* Nábelek & Bornm. 99
Acantholimon astragalinum Mobayen 4
Acantholimon atrofuscum Rech.f. 4
Acantholimon atropatanum Bunge 4
Acantholimon auganum Bunge 4
Acantholimon aulieatense Czerniak. 5
Acantholimon austro-iranicum Rech.f. & Schiman-Czeika 5
Acantholimon avanosicum Doğan & Akaydın 5
Acantholimon avenaceum Bunge 5
Acantholimon avenaceum f. *simplicior* Bornm. ex Lincz. 5
Acantholimon avenaceum var. *khorassanicum* (Czerniak.) Mobayen 10
Acantholimon azizae Mobayen 5
Acantholimon bakhtiaricum Assadi 5
Acantholimon balansae (Kusn.) Grossh. 4
Acantholimon balansae Boiss. ex Bunge 4
Acantholimon balchanicum Korovin 5
Acantholimon baltanense Boiss. & Hausskn. ex Boiss. 4
Acantholimon bashkaleicum Doğan & Akaydın 5
Acantholimon birandii Doğan & Akaydın 5
Acantholimon blakelackii Mobayen 5
Acantholimon blakelockii Mobayen 5
Acantholimon blandum Czerniak. 5
Acantholimon bobrovii Czerniak. 12
Acantholimon bodeanum Bunge 5
Acantholimon bodeanum subsp. *bodeanum* Bunge 5
Acantholimon bodeanum subsp. *pilosum* Assadi 5
Acantholimon bodeanum var. *faustii* (Trautv.) Mobayen 8
Acantholimon Boiss. 2, 96
Acantholimon bonesseae Parsa 5
Acantholimon borodinii Krasn. 5
Acantholimon brachyphyllum Boiss. 5
Acantholimon brachystachyum Boiss. ex Bunge 5
Acantholimon brachystachyum var. *brachyphyllum* Boiss. 5
Acantholimon bracteatum (Girard) Boiss. 5
Acantholimon bracteatum Girard 99
Acantholimon bracteatum var. *bracteatum* (Girard) Boiss. 5

Acantholimon bracteatum var. *capitatum* (Sosn.) Bokhari 6
Acantholimon bracteatum var. *intermedium* Bordz. 5
Acantholimon bracteatum var. *splendidum* (Bunge) Boiss. 5
Acantholimon brecklei Rech.f. & Schiman-Czeika 5
Acantholimon breviscapum Boiss. & Hausskn. ex Boiss. 11
Acantholimon bromifolium Boiss. ex Bunge 5, 6
Acantholimon bromifolium f. *breviscapa* Parsa 5
Acantholimon bromifolium var. *approximatum* Bornm. 5
Acantholimon bromifolium var. *bromifolium* Boiss. ex Bunge 6
Acantholimon bromifolium var. *ilamicum* Mobayen 6
Acantholimon bromifolium var. *iranicum* (Bornm.) Rech.f. & Schiman-Czeika 6
Acantholimon bromifolium var. *lolioides* Rech.f. & Schiman-Czeika 6
Acantholimon bromifolium var. *platyphyllum* Bornm. 6
Acantholimon butkovii Lincz. 6
Acantholimon cabulicum Boiss. 6
Acantholimon caesareum Boiss. & Balansa 6
Acantholimon caesareum var. *elongatum* Mobayen 6
Acantholimon calocephalum Aitch. & Hemsl. 6
Acantholimon calvertii Boiss. 6
Acantholimon calvertii var. *calvertii* Boiss. 6
Acantholimon calvertii var. *glabrum* Akaydın & Dogan 6
Acantholimon calvertii var. *sanguineum* Mobayen 6
Acantholimon capitatum Sosn. 6
Acantholimon capitatum subsp. *capitatum* Sosn. 6
Acantholimon capitatum subsp. *sivasicum* Doğan & H.Duman 6
Acantholimon carinatum Rech.f. & Schiman-Czeika 6
Acantholimon caryophyllaceum Boiss. 6
Acantholimon caryophyllaceum Hausskn. ex Bunge 4
Acantholimon caryophyllaceum subsp. *caryophyllaceum* Boiss. 6
Acantholimon caryophyllaceum subsp. *parviflorum* Bokhari 7
Acantholimon caryophyllaceum var. *brachystachyum* Boiss. 11
Acantholimon cataonicum Bunge 13
Acantholimon catenatum Rech.f. & Schiman-Czeika 6
Acantholimon cephalotes Boiss. 6
Acantholimon cephalotoides Rech.f. 6
Acantholimon cephalotum St.-Lag. 6
Acantholimon chitralicum Rech.f. & Schiman-Czeika 6
Acantholimon chlorostegium Rech.f. & Schiman-Czeika 6
Acantholimon chrysostegium Rech.f. & Schiman-Czeika 6
Acantholimon cleistocalyx Hand.-Mazz. 14
Acantholimon coarctatum Trautv. 100
Acantholimon collare Köie & Rech.f. 6
Acantholimon compactum Korovin 6
Acantholimon confertiflorum Bokhari 6
Acantholimon creticum (Boiss.) Rech.f. 17
Acantholimon cupreo-olivascens Rech.f. & Schiman-Czeika 6
Acantholimon curviflorum Bunge 14
Acantholimon cymosum Bunge 7
Acantholimon damassanum Mobayen 7
Acantholimon damassanum var. *damassanum* 7
Acantholimon damassanum var. *lancibracteatum* Bokhari 7
Acantholimon davisii Akaydın & M.B.Crespo 7
Acantholimon demavendicum Bornm. 7
Acantholimon demawendicum Bornm. 7
Acantholimon densiflorum Assadi 7
Acantholimon desertorum Regel 14
Acantholimon dianthifolium Bokhari 7
Acantholimon diapensioides Boiss. 7
Acantholimon diapensioides Herder 17
Acantholimon diapensioides var. *longifolium* O.Fedsch. 9
Acantholimon distachyum Boiss. 7
Acantholimon distichum Rech.f. & Schiman-Czeika 7
Acantholimon diversifolium O.Schwarz & F.K.Mey. 7
Acantholimon doganii Bağcı, Doğu & Akaydın 7
Acantholimon ecae Aitch. & Hemsl. 7
Acantholimon echinus (L.) Boiss. 16
Acantholimon echinus (L.) Bunge 16
Acantholimon echinus f. *disticum* Mobayen 17
Acantholimon echinus subsp. *creticum* (Boiss.) Papan. & Kokkini 17
Acantholimon echinus subsp. *lycaonicum* (Boiss. & Heldr.) Papan. & Kokkini 12
Acantholimon echinus var. *glaberrimum* Mobayen 17
Acantholimon echinus var. *puberulum* Boiss. 13
Acantholimon edmondsonii Rech.f. & Schiman-Czeika 7
Acantholimon ekatherinae (B.Fedtsch.) Czerniak. 7
Acantholimon ekbergianum Rech.f. & Schiman-Czeika 7
Acantholimon ekimii Doğan & Akaydın 7
Acantholimon embergeri Mobayen 7
Acantholimon erinaceum (Jaub. & Spach) Lincz. 7
Acantholimon erythraeum Bunge 7
Acantholimon eschkerense Boiss. & Hausskn. ex Boiss. 8
Acantholimon esfandiarii Rech.f. & Schiman-Czeika 8
Acantholimon eubergeri Mobayen 7
Acantholimon evrenii Doğan & Akaydın 8
Acantholimon fasciculare Boiss. 8
Acantholimon faustii Trautv. 8
Acantholimon fedorovii Tamamsch. & Mirzoeva 8
Acantholimon ferox (Jaub. & Spach) Boiss. 15
Acantholimon festucaceum (Jaub. & Spach) Boiss. 8
Acantholimon festucaceum var. *festucaceum* (Jaub. & Spach) Boiss. 8
Acantholimon festucaceum var. *laxiflora* Boiss. 8
Acantholimon fetisowi Regel 8
Acantholimon fetissovii Regel 8
Acantholimon flabellum Assadi 8
Acantholimon flexuosum Boiss. ex Bunge 8
Acantholimon flexuosum var. *laxiflorum* Mobayen 8
Acantholimon fominii Kusn. 8
Acantholimon gabrieljaniae Mirzoeva 8
Acantholimon gabrieljanii Mirzoeva 8
Acantholimon gadukense Mobayen 8
Acantholimon gaudanense Czerniak. 8
Acantholimon gemicianum Kaptaner İğci, Körüklü & Aytaç 8
Acantholimon genistioides var. *khossrovii* Mobayen 8
Acantholimon genistoides (Jaub. & Spach) Boiss. 8
Acantholimon genistoides var. *genistoides* (Jaub. & Spach) Boiss. 8
Acantholimon ghoranum Rech.f. & Schiman-Czeika 8
Acantholimon gilliatii Turrill 8
Acantholimon gilliatii var. *yamense* (Turrill) Mobayen 18
Acantholimon gillii Rech.f. & Köie 8
Acantholimon giselae Bornm. 3

Acantholimon glabratum Assadi....8
Acantholimon glabratum subsp. *glabratum* Assadi....8
Acantholimon glabratum subsp. *kashanense* Batuli & Assadi....8
Acantholimon glumaceum (Jaub. & Spach) Boiss. 2, 8
Acantholimon glumaceum var. *breviscapum* Trautv. 9
Acantholimon glumaceum var. *glabra* Mobayen....6
Acantholimon glumaceum var. *sahendicum* (Boiss. & Buhse) Kusn. 14
Acantholimon glumaceum var. *typicum* Trautv. 9
Acantholimon glutinosum Rech.f. & Köie 9
Acantholimon goeksunicum Doğan & Akaydın 9
Acantholimon gontscharovii Czerniak....9
Acantholimon gorganense Mobayen....9
Acantholimon gracillimum Rech.f. & Schiman-Czeika 9
Acantholimon graecum F.K.Mey....9
Acantholimon gramineum Korovin....5
Acantholimon grammophyllum Rech.f. & Köie....9
Acantholimon griffithianum Boiss....9
Acantholimon gulistanum Bunge....9
Acantholimon gulistanum var. *glabrescens* Mobayen....9
Acantholimon haesarense Bornm. ex Rech.f. & Schiman-Czeika....9
Acantholimon halophilum Bokhari....9
Acantholimon halophilum var. *coloratum* Doğan & Akaydın 9
Acantholimon halophilum var. *halophilum* Bokhari....9
Acantholimon hamadannicum Assadi & Mahmoodi 9
Acantholimon hariabense Rech.f. & Köie....9
Acantholimon hausknechti Bunge 4
Acantholimon haussknechtii Bunge 4
Acantholimon hedinii Ostenf. 9
Acantholimon hellwigii Lincz. & N.I.Akshigitova 100
Acantholimon hellwigii var. *escaposum* Lincz. & N.I.Akshigitova 100
Acantholimon heratense Bunge....9
Acantholimon heweri Rech.f. & Schiman-Czeika 9
Acantholimon hilariae Ikonn....9
Acantholimon hindukushum Mobayen....9
Acantholimon hissaricum Lincz....9
Acantholimon hohenackeri (Jaub. & Spach) Boiss. 9
Acantholimon hohenackeri Ledeb. 9
Acantholimon hohenackeri var. *subsessile* Trautv. 3
Acantholimon hohenackeri var. *virens* Rupr. 14
Acantholimon homophyllum Rech.f. & Schiman-Czeika .9
Acantholimon hormozganense Assadi....9
Acantholimon horridum Bunge....10
Acantholimon hoshapicum Doğan & Akaydın....10
Acantholimon huetii Boiss....10
Acantholimon huetii var. *breviscapum* Akaydın & Dogan 10
Acantholimon huetii var. *huetii* Boiss....10
Acantholimon hyalinum Rech.f. & Köie 10
Acantholimon hypochaerum Bokhari....10
Acantholimon hypochoerum Mobayen....10
Acantholimon hypochoerum var. *erythraeum* Mobayen 10
Acantholimon hystrix (Jaub. & Spach) Boiss. 17
Acantholimon hystrix Stapf....10
Acantholimon ibrahimii Akaydın....10
Acantholimon iconicum Boiss. & Heldr. ex Boiss. 10
Acantholimon incomptum Boiss. & Buhse 10
Acantholimon incomptum var. *straussii* Bornm....14
Acantholimon inerme Rech.f. & Köie 10
Acantholimon iranicum Bornm. 6
Acantholimon iskanderi Lipsky ex O.Fedtsch. & B.Fedtsch....16
Acantholimon jarmilae Halda....10
Acantholimon kandaharense Rech.f. 10
Acantholimon karabajeviorum Lazkov....10
Acantholimon karadarjense Lincz. 10
Acantholimon karamanicum Akaydın & Dogan 10
Acantholimon karatavicum Pavlov 10
Acantholimon karelinii (Stschegl.) Bunge....10
Acantholimon kaschgaricum Lincz....10
Acantholimon katrantavicum Lincz....10
Acantholimon kermanense Assadi & Mirtadz. 10
Acantholimon khorassanicum Czerniak. 10
Acantholimon khorassanicum var. *kopetdaghense* Czerniak. 10
Acantholimon kjurendaghi Mesczer. 10
Acantholimon knorringianum Lincz. 10
Acantholimon koeiei Rech.f. & Schiman-Czeika 10
Acantholimon koelzii Rech.f. & Köie 11
Acantholimon koeycegizicum Doğan & Akaydın 11
Acantholimon kokandense Bunge....11
Acantholimon komarovii Czerniak. 11
Acantholimon korolkovii (Regel) Korovin....11
Acantholimon korovini Czerniak. 11
Acantholimon korovinii Czerniak....11
Acantholimon kotschyi (Jaub. & Spach) Boiss. 11
Acantholimon kotschyi subsp. *kotschyi*....11
Acantholimon kotschyi subsp. *laxispicatum* Bokhari 11
Acantholimon kotschyi var. *cataonicum* Bunge 11
Acantholimon kotschyi var. *iconicum* Boiss....10
Acantholimon kotschyi var. *libanoticum* Boiss....11
Acantholimon kuramense Lincz. 11
Acantholimon kurdicum Bunge....5
Acantholimon kutschanense Rech.f. 14
Acantholimon laevigatum (T.X.Peng) Kamelin....11
Acantholimon langaricum O.Fedtsch. & B.Fedtsch....11
Acantholimon latifolium Boiss. 11
Acantholimon latifolium Rupr. 14
Acantholimon laxiflorum Boiss....11
Acantholimon laxiflorum Boiss. ex Bunge....11
Acantholimon laxiusculum F.O.Khass. & I.I.Malzev....11
Acantholimon laxum Czerniak....11
Acantholimon leptostachyum Aitch. & Hemsl....16
Acantholimon lepturoides (Jaub. & Spach) Boiss. 11
Acantholimon leucacanthum (Jaub. & Spach) Boiss. 11
Acantholimon leucacanthum var. *orshanum* Mobayen 11
Acantholimon leucochlorum Rech.f. & Schiman-Czeika 11
Acantholimon libanoticum Boiss....11
Acantholimon libanoticum var. *ulicinum* (Willd. ex Schult.) Boiss....17
Acantholimon limbatum (Lincz.) Sennikov....41
Acantholimon linczevskianum Lazkov....11
Acantholimon linczevskii Pavlov....11
Acantholimon listoniae Boiss. 3
Acantholimon litvinovii Lincz....12
Acantholimon longiflorum Boiss. 12
Acantholimon longiscapum Bokhari 12
Acantholimon lycaonicum Boiss. & Heldr. 12
Acantholimon lycaonicum subsp. *cappadocicum* Doğan & Akaydın....12

Acantholimon lycaonicum subsp. *lycaonicum* Boiss. & Heldr. 12
Acantholimon lycaonicum var. *cataonicum* (Bunge) Mobayen 12
Acantholimon lycopodioides (Girard) Boiss. 12
Acantholimon macranthum Rech.f. & Köie 12
Acantholimon macranthum subsp. *dubium* Rech.f. & Köie 12
Acantholimon macropetalum Rech.f. & Schiman-Czeika 12
Acantholimon macrostachyum Rech.f. & Schiman-Czeika 12
Acantholimon maewskianum Regel 12
Acantholimon majewianum O.Fedtsch. & B.Fedtsch. 12
Acantholimon majewianum Regel 12
Acantholimon manakyanii Ogan. 12
Acantholimon margaritae Korovin 12
Acantholimon marmoreum Korovin 8
Acantholimon melananthum (Boiss.) Boiss. 12
Acantholimon microstegium Bornm. 16
Acantholimon mikeschinii Lincz. 12
Acantholimon minshelkense Pavlov 12
Acantholimon mirandum Lincz. 91
Acantholimon mirtadzadinii Assadi 12
Acantholimon mirum Lincz. 90
Acantholimon mishaudaghense Mobayen 12
Acantholimon mobayenii Assadi & Ghahr. 12
Acantholimon modestum Bornm. ex Rech.f. & Schiman-Czeika 12
Acantholimon moradii Assadi 12
Acantholimon movdarinum Parsa 18
Acantholimon muchamedshanovii Lincz. 12
Acantholimon multiflorum (Bokhari) Doğan & Akaydın 12
Acantholimon munroanum Aitch. & Hemsl. 5
Acantholimon muradicum O.Schwarz & F.K.Mey. 12
Acantholimon murorum Korovin 8
Acantholimon nabievii Lincz. 12
Acantholimon narynense Lazkov 12
Acantholimon nawaricum Rech.f. & Schiman-Czeika ... 13
Acantholimon nigricans Mobayen 13
Acantholimon nikitinii Lincz. 13
Acantholimon nuratavicum Zakirov 13
Acantholimon nuristanicum Kitam. 16
Acantholimon oliganthum Boiss. 13
Acantholimon olivieri (Jaub. & Spach) Boiss. 13
Acantholimon olympicum (Boiss.) F.K.Mey. 17
Acantholimon oopodum (Popov & Korovin) Sennikov .. 40
Acantholimon ophiocladum Rech.f. & Schiman-Czeika 13
Acantholimon pamiricum Czerniak. 13
Acantholimon paniculatum Rech.f. 7
Acantholimon parsanum Mobayen 8
Acantholimon parsianum Lincz. 7
Acantholimon parviflorum (Bokhari) Akaydın & Dogan .. 7
Acantholimon parviflorum Regel 13
Acantholimon pavlovii Lincz. 13
Acantholimon peculiare Rech.f. 13
Acantholimon peronini Boiss. 13
Acantholimon peroninii Boiss. 13
Acantholimon petraeum Boiss. ex Bunge 13
Acantholimon petuniiflorum Mobayen 13
Acantholimon phrygium Boiss. 3
Acantholimon physostegium Rech.f. & Schiman-Czeika 13
Acantholimon pinardi Boiss. 3
Acantholimon pinardii Boiss. 3
Acantholimon podlechii Rech.f. & Schiman-Czeika 13
Acantholimon poliochlorum Rech.f. & Schiman-Czeika 13
Acantholimon polystachyum Boiss. 13
Acantholimon popovii Czerniak. 13
Acantholimon procumbens Czerniak. 13
Acantholimon pskemense Lincz. 13
Acantholimon pterostegium Bunge 13
Acantholimon puberulum Boiss. & Balansa 13, 14
Acantholimon puberulum subsp. *longiscapum* (Bokhari) Doğan & Akaydın 13
Acantholimon puberulum subsp. *peronini* (Boiss.) Akaydın & Dogan 13
Acantholimon puberulum subsp. *peroninii* (Boiss.) Akaydın & Dogan 13
Acantholimon puberulum subsp. *puberulum* Boiss. & Balansa 14
Acantholimon puberulum var. *glabrum* Bokhari 13
Acantholimon puberulum var. *longiscapum* Bokhari 13
Acantholimon pulchellum f. *condensatum* Korovin 101
Acantholimon pulchellum Korovin 14
Acantholimon purpureum Korovin 14
Acantholimon purpureum Parsa 7
Acantholimon quettense Rech.f. & Schiman-Czeika 14
Acantholimon quettensis Rech.f. & Schiman-Czeika 14
Acantholimon quinquelobum Bunge 14
Acantholimon quinquelobum var. *curviflorum* (Bunge) Doğan & Akaydın 14
Acantholimon quinquelobum var. *quinquelobum* Bunge 14
Acantholimon raddeanum Czerniak. 14
Acantholimon raikoviae Czerniak. ex Lincz. 14
Acantholimon rechingeri Freitag 42
Acantholimon reflexifolium Bokhari 14
Acantholimon restiaceum Bunge 14
Acantholimon revolutum Rech.f. & Köie 14
Acantholimon rhodopolium Rech.f. & Schiman-Czeika . 14
Acantholimon riyatguelii Yıldırım 14
Acantholimon roborowskii Czerniak. 5
Acantholimon roseum Boiss. 7
Acantholimon roseum var. *erinaceum* (Jaub. & Spach) Boiss. 7
Acantholimon roseum var. *pubescens* Czebnjak. 7
Acantholimon roseum var. *pungens* Boiss. 7
Acantholimon rubellum Boiss. ex Bunge 7
Acantholimon rubricosum Mobayen 14
Acantholimon rudbaricum Bornm. 14
Acantholimon ruprechti Bunge 14
Acantholimon ruprechtii Bunge 14
Acantholimon saadii Assadi & Zeraatkar 14
Acantholimon sackeni Bunge 14
Acantholimon sackenii Bunge 14
Acantholimon sahendicum Boiss. & Buhse 14
Acantholimon salangense Bokhari 14
Acantholimon salangensis Bokhari 14
Acantholimon saravschanicum Regel 15
Acantholimon sarawschanicum Regel 14, 15
Acantholimon sarytavicum Lincz. 15
Acantholimon saxifragifolium Rech.f. & Köie 91
Acantholimon saxifragiforme Hausskn. & Sint. ex Bokhari 15
Acantholimon saxifragiforme Hausskn. ex Mobayen 15
Acantholimon scabiosum Mobayen 6

Acantholimon scabiosum var. *nudicalyx* Mobayen.........6
Acantholimon scabrellum Boiss. & Hausskn. ex Boiss...15
Acantholimon scabrellum var. *kandilum* Mobayen 15
Acantholimon scabrellum var. *scabrellum* Boiss. & Hausskn. ex Boiss...15
Acantholimon schachimardanicum Lincz......................15
Acantholimon schahrudicum Bunge 15
Acantholimon schemachense Grossh. 15
Acantholimon schirazianum Boiss. 15
Acantholimon schizostegium Rech.f. & Schiman-Czeika 15
Acantholimon scirpinum Bunge 15
Acantholimon sclerophyllum Rech.f. & Schiman-Czeika15
Acantholimon scorpioideum St.-Lag..............................15
Acantholimon scorpius (Jaub. & Spach) Boiss. 15
Acantholimon scorpius var. *balutchii* Mobayen............15
Acantholimon scorpius var. *incomptum* (Boiss. & Buhse) Boiss...10
Acantholimon scorpius var. *leucacanthum* Boiss. 11
Acantholimon sect. *Acantholimon* Boiss.......................96
Acantholimon sect. *Acmostegia* Bunge.........................96
Acantholimon sect. *Armeriopsis* Boiss. 96
Acantholimon sect. *Bromeliopsis* Rech.f. & Schiman-Czeika...96
Acantholimon sect. *Chaetolimon* Bunge.......................41
Acantholimon sect. *Cymaria* Bunge..............................96
Acantholimon sect. *Dracogyna* Mobayen.....................96
Acantholimon sect. *Glumaria* Boiss. 96
Acantholimon sect. *Gontscharovia* Lincz. 90
Acantholimon sect. *Inermia* Rech.f. & Köie...................96
Acantholimon sect. *Microstegia* Bornm........................96
Acantholimon sect. *Physostegia* Rech.f. & Schiman-Czeika...96
Acantholimon sect. *Platystegia* Rech.f. & Schiman-Czeika 96
Acantholimon sect. *Poicilocephala* Rech.f. & Schiman-Czeika...96
Acantholimon sect. *Pterostegia* Bunge 96
Acantholimon sect. *Pulvinaria* Boiss. 96
Acantholimon sect. *Schizostegia* Rech.f. & Schiman-Czeika...96
Acantholimon sect. *Staticopsis* Boiss. 96
Acantholimon sect. *Stereophylla* Rech.f. & Schiman-Czeika...96
Acantholimon sect. *Tragacanthina* Bunge....................96
Acantholimon senganense Bunge................................15
Acantholimon senganense subsp. *senganense* Bunge..15
Acantholimon senganense subsp. *tehranense* Assadi...15
Acantholimon senganense var. *glaucum* Parsa............15
Acantholimon serotinum Rech.f. & Schiman-Czeika 15
Acantholimon setiferum Bunge 41
Acantholimon shahrudicum Bunge..............................15
Acantholimon sintenisii Hausskn. 10
Acantholimon sirchense Assadi & Mirtadz....................15
Acantholimon sogdianum (Lincz.) Sennikov 92
Acantholimon solidum Rech.f. & Köie...........................15
Acantholimon sorchense Rech.f...................................15
Acantholimon speciosissimum Aitch. & Hemsl. 15
Acantholimon spinicalyx Köie & Rech.f.........................16
Acantholimon spinosum Rob. 101
Acantholimon spirizianum Mobayen 16
Acantholimon spirizianum subsp. *spirizianum*.............16
Acantholimon spirizianum var. *multiflorum* Bokhari 12
Acantholimon splendidum Bunge 5
Acantholimon squarrosum Pavlov 16
Acantholimon stanjukoviczii Lincz. ex Ikonn. 16
Acantholimon stapfianum Rech.f. & Schiman-Czeika ...16
Acantholimon stenorhaphium Rech.f...........................16
Acantholimon stereophyllum Rech.f. & Schiman-Czeika 16
Acantholimon stocksii Boiss. 16
Acantholimon stocksii var. *auriculatum* Mobayen........16
Acantholimon straussii Bornm.....................................14
Acantholimon strictiforme Nikitina ex Lazkov..............16
Acantholimon strictum Czerniak. 16
Acantholimon strigillosum Bokhari 16
Acantholimon stroterophyllum Rech.f. & Schiman-Czeika 16
Acantholimon subavenaceum Lincz. 16
Acantholimon subflavescens Rech.f. & Schiman-Czeika16
Acantholimon subsect. *Caryophyllacea* Boiss. 96
Acantholimon subsect. *Erythrostoma* Bunge 96
Acantholimon subsect. *Eurystomata* Bunge 96
Acantholimon subsect. *Exacantha* Yıldırım & M.B.Crespo 96
Acantholimon subsect. *Halophiliacea* Muvaffak & Doğan 96
Acantholimon subsect. *Microcalycina* (Bunge) Boiss. ...96
Acantholimon subsect. *Rhodocalycina* Bunge..............96
Acantholimon subsect. *Stenostoma* Bunge..................96
Acantholimon subsimile Rech.f. & Schiman-Czeika.......16
Acantholimon subulatum Boiss...................................16
Acantholimon szovitzii Boiss. & Buhse 10
Acantholimon takhtajanii Ogan. 16
Acantholimon talagonicum Boiss.................................16
Acantholimon talagonicum var. *microstegium* (Bornm.) Mobayen 16
Acantholimon talassicum Korovin................................11
Acantholimon tarbagataicum Gamajunova..................16
Acantholimon taschkurganicum Lincz. & N.I.Akshigitova 16
Acantholimon taschkurganicum var. *escaposum* Lincz. & N.I.Akshigitova 16
Acantholimon taschkurganicum var. *taschkurganicum* Lincz. & N.I.Akshigitova..16
Acantholimon tataricum Boiss. 16
Acantholimon tchihatcheffii Fisch. & C.A.Mey. 3
Acantholimon tenuiflorum Boiss. 16
Acantholimon termei Rech.f. & Schiman-Czeika 17
Acantholimon tianschanicum Czerniak. 17
Acantholimon tibeticum Hook.f. & Thomson ex C.B.Clarke 12
Acantholimon titovii Lincz..17
Acantholimon tomentellum Boiss. 17
Acantholimon tournefortii (Jaub. & Spach) Boiss. 17
Acantholimon tragacanthinum (Jaub. & Spach) Boiss. .17
Acantholimon tragacanthinum Griff. ex Bunge 8
Acantholimon tragacanthium Griff. ex Bunge 8
Acantholimon trautvetteri Kusn....................................4
Acantholimon tricolor Rech.f. & Köie 17
Acantholimon trojanum F.K.Mey. 17
Acantholimon truncatum Bunge 17
Acantholimon truncatum subsp. *rudbaricum* Bornm....14
Acantholimon tschimganicum Korovin 7
Acantholimon tulakense Rech.f. & Schiman-Czeika 17
Acantholimon tulakensis Rech.f. & Schiman-Czeika 17

Acantholimon turcicum Doğan & Akaydın 17
Acantholimon ulicinum (Willd. ex Schult.) Boiss. 17
Acantholimon ulicinum subsp. *lycaonicum* (Boiss. & Heldr.) Bokhari & J.R.Edm. 12
Acantholimon ulicinum var. *creticum* (Boiss.) Bokhari & J.R.Edm. 17
Acantholimon ulicinum var. *creticum* (Boiss.) Greuter .17
Acantholimon ulicinum var. *purpurascens* (Bokhari) Bokhari & J.R.Edm. 17
Acantholimon ulicinum var. *ulicinum* (Willd. ex Schult.) Boiss. 17
Acantholimon vacillans Rech.f. & Schiman-Czeika........17
Acantholimon varivtzevae Czerniak. 18
Acantholimon vedicum Mirzoeva 18
Acantholimon velutinum Czerniak. 18
Acantholimon venustum (Fenzl ex Boiss.) Boiss. 18
Acantholimon venustum var. *assyriacum* (Boiss.) Boiss. 18
Acantholimon venustum var. *laxiflorum* (Boiss. ex Bunge) Bokhari 11
Acantholimon venustum var. *olivieri* Boiss. 13
Acantholimon venustum var. *venustum* (Fenzl ex Boiss.) Boiss. 18
Acantholimon virens Czerniak. 18
Acantholimon viscidulum Boiss. 18
Acantholimon vvedenskyi Lincz. 18
Acantholimon wendelboi Rech.f. & Schiman-Czeika 18
Acantholimon wiedemanni Bunge 18
Acantholimon wiedemannii Bunge 18
Acantholimon wilhelminae Rech.f. & Schiman-Czeika ..18
Acantholimon xanthacanthum Rech.f. & Köie 18
Acantholimon yamense Turrill 18
Acantholimon yildizelicum Akaydın 18
Acantholimon zaeifii Assadi 18
Acantholimon zakirovii Beshko 18
Acantholimon zaprjagaevii Lincz. 18
Aegialinites annulatus (R.Br.) C.Presl 2
Aegialinites C.Presl 2
Aegialinites rotundifolius (Roxb.) C.Presl 2
Aegialitidaceae Lincz. 1
Aegialitideae Z.X.Peng 1
Aegialitis annulata Kurz 2
Aegialitis annulata R.Br. 1, 2
Aegialitis R.Br. 1
Aegialitis rotundifolia Roxb. 2
Aegialitis tenuis Trin. 99
Aegialitis Trin. 99
Aeoniopsis cabulica (Boiss.) Rech.f. 40
Aeoniopsis Rech.f. 40
Afrolimon amoenum (C.H.Wright) Lincz. 73
Afrolimon capense (L.Bolus) Lincz. 73
Afrolimon Lincz. 45, 73
Afrolimon longifolium (Thunb.) Lincz. 73
Afrolimon namaquanum (L.Bolus) Lincz. 73
Afrolimon peregrinum (P.J.Bergius) Lincz. 45, 73
Afrolimon purpuratum (L.) Lincz. 73
Afrolimon teretifolium (L.Bolus) Lincz. 73
Androsace macloviana Cham. 32
Armeria ×carnotana Blanco-Dios 25
Armeria ×cintrana Taul.Gomes 26
Armeria ×intermedia Szafer 99
Armeria ×nieto-felineri Rivas Mart. & al. 36
Armeria ×pilariae Sánchez Gullón, Muñoz Rodr. & Polo Ávila 35
Armeria ×salmantica (Bernis) Nieto Fel. 37
Armeria adamovicii Halácsy 25
Armeria adsurgens (Torr. ex A.Gray) Kuntze.................99
Armeria aegialea Phil. 26
Armeria albi (Bernis) Nieto 18
Armeria alboi (Bernis) Nieto Fel. 18
Armeria alliacea .18, 19, 20, 21, 22, 25, 27, 29, 34, 37, 99
Armeria alliacea (Cav.) Hoffmanns. & Link 18, 19
Armeria alliacea Griseb. 99
Armeria alliacea Mutel, A. 19
Armeria alliacea subsp. *alliacea* (Cav.) Hoffmanns. & Link 19
Armeria alliacea subsp. *bupleuroides* (Gren. & Godr.) O.Bolòs & Vigo 21
Armeria alliacea subsp. *capitellata* (Pau) Rivas Mart....25
Armeria alliacea subsp. *loscosii* Romo 19
Armeria alliacea subsp. *matritensis* (Pau) Borja, Rivas Goday & Rivas Mart. 20
Armeria alliacea subsp. *plantaginea* (All.) O.Bolòs & Vigo 21
Armeria alliacea subsp. *praecox* (Jord.) Jovet & R.Vilm.22
Armeria alliacea subsp. *ruscinonensis* (Girard) O.Bolòs & Vigo 37
Armeria alliacea subvar. *sicorisiensis* (Sennen) Bernis..21
Armeria alliacea var. *allioides* Pau 19
Armeria alliacea var. *bilbilitana* (Bernis) O.Bolòs & Vigo 21
Armeria alliacea var. *bupleuroides* (Gren. & Godr.) O.Bolòs & Vigo 21
Armeria alliacea var. *daveaui* Cout. 27
Armeria alliacea var. *heterophylla* Pau ex Viciosa 19
Armeria alliacea var. *javalambrica* Bernis 19
Armeria alliacea var. *littorifuga* (Bernis) O.Bolòs & Vigo 37
Armeria alliacea var. *palearensis* O.Bolòs & Vigo 19
Armeria alliacea var. *plantaginea* (All.) O.Bolòs & Vigo 21
Armeria alliacea var. *pubescens* Mutel, A. 29
Armeria alliacea var. *rumelicina* (Bernis) O.Bolòs & Vigo 19
Armeria alliacea var. *sicorisiensis* (Sennen) O.Bolòs & Vigo 19
Armeria alliacea var. *yebalica* Pau 34
Armeria alliacea Webb ex Ball 34
Armeria allioides Boiss. 19
Armeria allioides Willk. & Lange 39
Armeria alpina f. *basitricha* Simonk. 20
Armeria alpina f. *conchensis* Bernis 21
Armeria alpina f. *conquensis* Bernis 21
Armeria alpina f. *glabra* Novák 20
Armeria alpina f. *hirsuta* Novák 20
Armeria alpina f. *hirtifolia* Novák 20
Armeria alpina f. *pumila* (Fuss ex Jáv.) Novák 20
Armeria alpina Friv. ex Griseb. 25
Armeria alpina Guss. 24
Armeria alpina Hoppe ex Ebel 20
Armeria alpina subsp. *alpina* Willd. 20
Armeria alpina subsp. *barcensis* (Simonk.) Jáv. 32
Armeria alpina subsp. *bubanii* (G.H.M.Lawr.) Malag. ...20
Armeria alpina subsp. *bubanii* (G.H.M.Lawr.) Rivas Mart. 20

Armeria alpina subsp. *cantabrica* (Boiss. & Reut. ex Willk. & Lange) Malag. 24
Armeria alpina subsp. *confusa* (Bernis) Malag. 20
Armeria alpina subsp. *fontqueri* (Pau) Malag. 28
Armeria alpina subsp. *godayana* (Font Quer) Malag. 28
Armeria alpina subsp. *halleri* (Wallr.) Nyman 20
Armeria alpina subsp. *microcephala* (Willk.) Malag. 23
Armeria alpina subsp. *muelleri* (A.Huet) Malag. 35
Armeria alpina subsp. *occasiana* (Bernis) Rivas Mart. 20
Armeria alpina subsp. *pubinervis* (Boiss.) Malag. 36
Armeria alpina subsp. *pumila* Fuss ex Jáv. 20
Armeria alpina subsp. *trachyphylla* (Lange) Malag. 38
Armeria alpina subsp. *vasconica* (Sennen ex Losa) Malag. 24
Armeria alpina subsp. *vasconica* (Sennen ex Losa) Rivas Mart. & al. 24
Armeria alpina subvar. *confusa* Bernis 20
Armeria alpina Ten. ex Boiss. 28
Armeria alpina Turcz. 34
Armeria alpina var. *alba* hort. ex F.T.Hubb. 98
Armeria alpina var. *eliator* Conill & Despaty 20
Armeria alpina var. *lancifolia* Freyn 23
Armeria alpina var. *microcephala* Willk. 23
Armeria alpina var. *nana* Bolzon 20
Armeria alpina var. *pumila* Fuss ex Jáv. 20
Armeria alpina var. *purpurea* (W.D.J.Koch) E.Baumann 33
Armeria alpina var. *seticeps* (Rchb.) Ebel 37
Armeria alpina Willd. 20
Armeria alpinifolia Pau & Font Quer 20
Armeria ambifaria Focke 32
Armeria amoena (Sims) Kuntze 99
Armeria amplifoliata Pau 37
Armeria ancarensis Merino 27
Armeria andicola Gay ex Boiss. 26
Armeria andina Poepp. ex Boiss. 26
Armeria andina var. *californica* Boiss. 32
Armeria androsacea Boiss. 26
Armeria apollinaris Sennen & Mauricio 20
Armeria arborea L.H.Bailey 35
Armeria arctica (Cham.) Wallr. 34
Armeria arctica Sternb. 101
Armeria arctica subsp. *californica* (Boiss.) Abrams 32
Armeria arctica var. *californica* (Boiss.) S.F.Blake 32
Armeria arcuata Welw. ex Boiss. & Reut. 20
Armeria arenaria (Pers.) F.Dietr. 20, 21
Armeria arenaria subsp. *anomala* (Bernis) Catalán ex Uribe-Ech. 21
Armeria arenaria subsp. *apennina* Arrigoni 21
Armeria arenaria subsp. *arenaria* (Pers.) F.Dietr. 21
Armeria arenaria subsp. *bilbilitana* (Bernis) Nieto Fel. 21
Armeria arenaria subsp. *bupleuroides* (Godr. & Gren.) Greuter & Raus 21
Armeria arenaria subsp. *burgalensis* (Sennen & Elias) Uribe-Ech. 21
Armeria arenaria subsp. *confusa* (Bernis) Nieto Fel. 21
Armeria arenaria subsp. *madoricola* M.B.Crespo & Mateo 21
Armeria arenaria subsp. *marginata* (Levier) Arrigoni 21
Armeria arenaria subsp. *peirescii* Baumel, Auda & Médail 22
Armeria arenaria subsp. *pradetensis* Médail, Baumel & Auda 21
Armeria arenaria subsp. *praecox* (Jord.) Kerguélen 21, 22
Armeria arenaria subsp. *praecox* (Jord.) Kerguélen ex Greuter, Burdet & G.Long 21
Armeria arenaria subsp. *segoviensis* (Gand. ex Bernis) Nieto Fel. 22
Armeria arenaria subsp. *vestita* (Willk.) Nieto Fel. 22
Armeria argyrocephala f. *longifolia* Hausskn. 23
Armeria argyrocephala f. *minor* Hausskn. 24
Armeria argyrocephala var. *graeca* Beck 24
Armeria argyrocephala Wallr. 38
Armeria armeria (L.) H.Karst. 32
Armeria asperrima (Sennen) Sennen 22
Armeria aspromontana Brullo, Scelsi & Spamp. 22
Armeria atlantica Pomel 22
Armeria australis Boiss. 22
Armeria australis var. *splendens* (Lag. & Rodr.) Boiss. 38
Armeria baetica Boiss. 29
Armeria baetica var. *africana* Boiss. 34
Armeria baetica var. *stenophylla* Boiss. 30
Armeria baltica Tzvelev 32
Armeria barcensis Simonk. 32
Armeria beirana Franco 22
Armeria beirana subsp. *altimontana* Franco 38
Armeria beirana subsp. *monchiquensis* (Bernis) Franco 22
Armeria beirana subvar. *monchiquensis* Bernis 22
Armeria beirana subvar. *sublittorea* Bernis 22
Armeria beirana var. *subrinhoi* Bernis 22
Armeria belgenciensis Donad. 22
Armeria belgenciensis Donad. ex Guin. & Vilmorin 22
Armeria belgenciensis Donad. ex Kerguélen 22
Armeria bella Albov 26
Armeria berlengensis Daveau 22
Armeria berlengensis var. *villosa* Daveau 36
Armeria bifida (Beck) Kuntze 99
Armeria bigerrensis (C.Vicioso & Beltrán) Pau ex Rivas Mart. 22, 23
Armeria bigerrensis subsp. *bigerrensis* (C.Vicioso & Beltrán) Pau ex Rivas Mart. 23
Armeria bigerrensis subsp. *legionensis* (Bernis) Rivas Mart. & al. 25
Armeria bigerrensis subsp. *losae* (Bernis) Rivas Mart., T.E.Díaz, Fern.Prieto, Loidi & Penas 23
Armeria bigerrensis subsp. *microcephala* (Willk.) Nieto Fel. 23
Armeria biguerrensis (C.Vicioso & Beltrán) Pau ex Rivas Mart. 23
Armeria boetica Boiss. 29
Armeria boissieriana Coss. 34
Armeria bottendorfensis A.G.Schulz 20
Armeria bourgaei Boiss. ex Merino 23
Armeria bourgaei subsp. *bourgaei* Boiss. ex Merino 23
Armeria bourgaei subsp. *lanceobracteata* (G.H.M.Lawr.) Nieto Fel. 23
Armeria bourgaei subsp. *willkommiana* (Bernis) Nieto Fel. 23
Armeria brachyphylla Boiss. 26
Armeria brevifolia Kunze ex Boiss. 26
Armeria brutia Brullo, Gangale & Uzunov 23
Armeria bryodes (Nutt.) Kuntze 99
Armeria bryoides (Nutt.) Kuntze 99
Armeria bubanii G.H.M.Lawr. 20
Armeria bupleuroides Cutanda 19
Armeria bupleuroides Gren. & Godr. 21
Armeria bupleuroides var. *reducta* Rouy 19

Armeria burgalensis Sennen & Elias 21
Armeria caballeroi (Bernis) Donad. 23
Armeria caespitosa (Ortega) Boiss. 23
Armeria caespitosa var. *bigerrensis* Vicioso & Beltrán . 23
Armeria caespitosa var. *isernii* Vicioso & Beltrán 29
Armeria caespitosa var. *splendens* (Lag. & Rodr.) Vicioso & Beltrán 38
Armeria campestris var. *hoffmannii* Wallr. 32
Armeria campestris var. *kochii* Wallr. 33
Armeria campestris var. *linkii* Wallr. 31
Armeria campestris Wallr. 33
Armeria canescens (Host) Boiss. 23, 24
Armeria canescens (Host) Ebel 23
Armeria canescens (Torr. & A.Gray) Kuntze 99
Armeria canescens f. *albanica* Beck 24
Armeria canescens f. *albiflora* Novák 24
Armeria canescens f. *angustifolia* Novák 24
Armeria canescens f. *dasyphylla* Rohlena 24
Armeria canescens f. *dasypoda* Murb. 23
Armeria canescens f. *dolichophylla* Novák 24
Armeria canescens f. *latifolia* (Vis.) Novák 23
Armeria canescens f. *microphylla* Novák 24
Armeria canescens f. *pantoscekii* (Strobl) Novák 24
Armeria canescens f. *platyphylla* Novák 24
Armeria canescens f. *pseudodalmatica* Novák 24
Armeria canescens f. *pubiscapa* Novák 24
Armeria canescens f. *rosea* Beck ex Novak 24
Armeria canescens f. *submajellensis* Novák 24
Armeria canescens subsp. *canescens* (Host) Boiss. 24
Armeria canescens subsp. *dalmatica* (Beck) Trinajstić . 24
Armeria canescens subsp. *gracilis* (Ten.) Bianchini 28
Armeria canescens subsp. *littorifuga* (Bernis) Malag. ... 37
Armeria canescens subsp. *nebrodensis* (Guss.) P.Silva . 24
Armeria canescens subsp. *ruscinonensis* (Girard) Malag. 37
Armeria canescens var. *brachyphylla* (Boiss.) G.H.M.Lawr. 23
Armeria canescens var. *dalmatica* (Beck) Novák 24
Armeria canescens var. *latifolia* Vis. 23
Armeria canescens var. *leucantha* (Boiss.) G.H.M.Lawr. 23
Armeria cantabrica Boiss. & Reut. ex Willk. & Lange 24
Armeria cantabrica Rouy 36
Armeria cantabrica subsp. *gracilifolia* (Bernis) Donad. 25
Armeria cantabrica subsp. *vasconica* (Sennen ex Losa) Uribe-Ech. 24
Armeria cantabrica subvar. *asturica* (Boiss. & Reut. ex Willk. & Lange) Bernis 25
Armeria cantabrica var. *maritima* Rouy 27
Armeria cantabrica var. *montana* Rouy 20
Armeria cantabrica var. *vasconica* (Sennen ex Losa) López Fern. 24
Armeria capitella Pau 25
Armeria caput-alba (Rothm.) Rothm. 25
Armeria cariensis Boiss. 25
Armeria cariensis var. *cariensis* Boiss. 25
Armeria cariensis var. *rumelica* (Boiss.) Boiss. 25
Armeria cariensis var. *thessala* Boiss. 25
Armeria carpetana subsp. *anisophylla* (Bernis) Franco 22
Armeria carpetana subsp. *carpetana* 38
Armeria carpetana Villar 38
Armeria carratracensis (Bernis) Rivas Mart. 39
Armeria castellana Boiss. & Reut. ex Leresche 25
Armeria castellana Boiss. & Reut. ex Rothm. 25
Armeria castrovalnerana Alejandre, Barredo & M.J.Escal. 25
Armeria castroviejoi Nieto Fel. 25
Armeria cephalotes Hoffmanns. & Link 35
Armeria cephalotes Hook. 34
Armeria cephalotes Schousb. 19, 34
Armeria cephalotes var. *alba* hort. ex F.T.Hubb. 97
Armeria cephalotes var. *grandiflora* hort. ex F.T.Hubb. 97
Armeria cephalotes var. *rubra* hort. ex F.T.Hubb. 97
Armeria cephalotes var. *splendens* hort. ex F.T.Hubb. .. 97
Armeria cephalotus Boiss. 35
Armeria cespitosa (Cav.) Boiss. 29
Armeria cespitosa subsp. *cespitosa* 29
Armeria chilensis Boiss. 26
Armeria chilensis subsp. *andina* (Poepp. ex Boiss.) Reiche 26
Armeria chilensis subsp. *macloviana* (Cham.) Reiche ... 26
Armeria chilensis var. *andina* (Poepp. ex Boiss.) Reiche 26
Armeria chilensis var. *bella* (Albov) Reiche 26
Armeria chilensis var. *brevifolia* Boiss. 26
Armeria chilensis var. *curvifolia* (Bertero) Boiss. 26
Armeria chilensis var. *genuina* Reiche 26
Armeria chilensis var. *macloviana* (Cham.) Reiche 26
Armeria chilensis var. *magellanica* Boiss. 26, 27
Armeria chilensis var. *majellanica* Boiss. 27
Armeria chouletteana Pomel 26
Armeria choulettiana Pomel 25, 26
Armeria ciliata (Lange) Nieto Fel. 26
Armeria cinerea Boiss. & Welw. ex Boiss. & Reut. 39
Armeria colorata Pau 26
Armeria curvifolia Bertero 26
Armeria curvifolia Colla 26
Armeria dalmatica Beck 24
Armeria daveaui (Cout.) P.Silva 27
Armeria daveaui (Cout.) Rivas Mart. 27
Armeria daveaui subsp. *daveaui* (Cout.) P.Silva 27
Armeria daveaui subsp. *matritensis* (Bernis) Franco 20
Armeria delfinii Phil. 26
Armeria denticulata (Bertol.) Bertol. 27
Armeria denticulata (Bertol.) DC. 27
Armeria denticulata Portensch. 23
Armeria denticulata Tratt. 24
Armeria dianthoides Hornem. & Spreng. ex Boiss. 19
Armeria divaricata (L.) Kuntze 100
Armeria douglasii (Hook.) Kuntze 100
Armeria duriaei Boiss. 27, 39
Armeria duriaei Cout. 39
Armeria duriaei subsp. *bourgaei* Boiss. ex Nyman 23
Armeria duriaei var. *ciliata* Lange 26
Armeria duriaei var. *seticaulis* Debeaux ex E.Rev. 23
Armeria duriensis Franco 38
Armeria duriensis subsp. *monticola* Franco 38
Armeria duriensis subsp. *oretana* (Bernis) Franco 22
Armeria duriensis subsp. *sublittorea* (Bernis) Franco 22
Armeria duriuscula Bab. 33
Armeria ebracteata Pomel 27
Armeria ebracteata var. *laevis* Maire 27
Armeria elongata (Hoffm.) M.Loehr 32
Armeria elongata (Hoffm.) W.D.J.Koch 32
Armeria elongata f. *bella* (Albov) Skottsb. 26
Armeria elongata f. *rostellata* Szafer 32
Armeria elongata Hoffm. 32

Armeria elongata var. *alpina* (DC.) Ces. 20
Armeria elongata var. *chilensis* (Boiss.) Skottsb. 26
Armeria elongata var. *intermedia* T.Marsson 32
Armeria elongata var. *macloviana* (Cham.) Skottsb. 26
Armeria elongata var. *maritima* (Mill.) Skottsb. 31
Armeria elongata var. *purpurea* (W.D.J.Koch) Boiss. 33
Armeria elongata var. *scabra* (Pall. ex Schult.) Regel 34
Armeria elongata var. *sibirica* (Turcz. ex Boiss.) Hartm. 33
Armeria elongata W.D.J.Koch 32
Armeria eriophila Willk. 27
Armeria eriophylla var. *marizii* Daveau 29
Armeria eriophylla Willk. 27
Armeria euscadiensis Donad. & Vivant 27
Armeria exaristata Phil. 26
Armeria expansa Wallr. 36
Armeria fasciculata (Vent.) Willd. 36
Armeria fasciculata var. *aristata* Mutel, A. 38
Armeria fasciculata var. *intermedia* Daveau 36
Armeria fibrosa Pomel 22
Armeria filicaulis (Boiss.) Boiss. 27
Armeria filicaulis Rouy 22
Armeria filicaulis subsp. *alfacarensis* Nieto Fel., Gut.Larena & Fuertes 27
Armeria filicaulis subsp. *filicaulis* (Boiss.) Boiss. 27
Armeria filicaulis subsp. *nevadensis* Nieto Fel., Rosselló & Fuertes 28
Armeria filicaulis subsp. *trevenqueana* Nieto Fel. 28
Armeria filicaulis subsp. *valentina* (Pau ex C.Vicioso) Mateo 28
Armeria filicaulis subsp. *willkommiana* (Bernis) Molero 27
Armeria filicaulis var. *bourgaei* (Boiss. ex Merino) Pau 23
Armeria filicaulis var. *bourgeaui* Pau 27
Armeria filicaulis var. *longifolia* Willk. 30
Armeria filicaulis var. *major* Boiss. 27
Armeria filicaulis var. *maroccana* Pau & Font Quer 27
Armeria filicaulis var. *minor* Boiss. 28
Armeria filicaulis var. *valentina* Pau ex C.Vicioso 28
Armeria floridana (Benth.) Kuntze 100
Armeria fontqueri Pau 28
Armeria formosa Heynh. 34
Armeria formosa hort. ex H.Vilm. 35
Armeria foucaudii Beck 37
Armeria fruticosa G.Lodd. 36
Armeria gaditana Boiss. 28
Armeria gaditana var. *boissieriana* (Coss.) G.H.M.Lawr. 34
Armeria gaditana var. *simplex* (Pomel) G.H.M.Lawr. 37
Armeria gaditana var. *spinulosa* (Boiss.) G.H.M.Lawr. 38
Armeria gaditiana var. *chamaeropicola* Pau 37
Armeria gaditiana var. *tingitana* (Boiss. & Reut.) Ball 38
Armeria garganica Arrigoni 30
Armeria genesiana Nieto Fel. 28
Armeria genesiana subsp. *belmonteae* (P.Silva) Nieto Fel. 28
Armeria genesiana subsp. *belmontei* (P.Silva) Nieto Fel. 28
Armeria genesiana subsp. *genesiana* Nieto Fel. 28
Armeria girardi (Bernis) Litard. 28
Armeria girardii (Bernis) Litard. 28
Armeria glaberrima (L.) Kuntze 100
Armeria glauca Wallr. 28
Armeria glaucescens Desf. 38
Armeria globosa Link ex Boiss. 35
Armeria godayana Font Quer 28
Armeria gracilis subsp. *gracilis* Ten. 28
Armeria gracilis subsp. *majellensis* (Boiss.) Arrigoni 28
Armeria gracilis Ten. 28
Armeria gracilis var. *humilis* Ten. 23
Armeria grajoana Casim.-Sor.Solanas & Cabezudo 28
Armeria grandiflora Boiss. 35
Armeria grosii Pau 29
Armeria gussonei Boiss. 35
Armeria halleri Wallr. 20
Armeria helodes F.Martini & Poldini 28
Armeria henriquesii Daveau 36
Armeria heterophylla Wallr. 24
Armeria hirta Pourr. ex Willk. & Lange 36
Armeria hirta subsp. *hispalensis* (Pau) Malag. 29
Armeria hirta subsp. *spinulosa* (Boiss.) Bernis 38
Armeria hirta var. *glauca* (Wallr.) Bernis 28
Armeria hirta var. *hirta* Willd. 29
Armeria hirta var. *perplexans* Bernis 29
Armeria hirta var. *tingitana* (Boiss. & Reut.) Bernis 38
Armeria hirta Willd. 28, 29
Armeria hispalensis Pau 29
Armeria hordii (Rich.) Kuntze 100
Armeria humilis (Link) Schult. 29
Armeria humilis subsp. *humilis* (Link) Schult. 29
Armeria humilis subsp. *odorata* (Samp.) P.Silva 29
Armeria icarica Edm. 29
Armeria intermedia (T.Marsson) Szafer 32
Armeria intermedia Link ex Boiss. 31
Armeria japonica Rippa 35
Armeria johnsenii Papan. & Kokkini 29
Armeria juncea Girard 28
Armeria juncea Wallr. 19
Armeria juniperifolia (Vahl) Hoffmanns. & Link 29
Armeria juniperifolia Ebel 27
Armeria juniperifolia Hoffmanns. & Link 29
Armeria juniperifolia J.Gay ex Boiss. 27
Armeria juniperifolia var. *bigerrensis* (Vicioso & Beltrán) G.H.M.Lawr. 29
Armeria juniperifolia var. *isernii* (Vicioso & Beltrán) G.H.M.Lawr. 29
Armeria juniperifolia var. *splendens* (Lag. & Rodr.) G.H.M.Lawr. 38
Armeria juniperifolia W.D.J.Koch 29
Armeria kochii Boiss. 29
Armeria labradorica f. *glabriscapa* (S.F.Blake) Malte 34
Armeria labradorica f. *glabriscapa* H.F.Lewis 34
Armeria labradorica f. *pubiscapa* Malte 34
Armeria labradorica subsp. *sibirica* (Turcz. ex Boiss.) Kamelin 33
Armeria labradorica var. *genuina* Malte 34
Armeria labradorica var. *submutica* H.F.Lewis 34
Armeria labradorica Wallr. 34
Armeria lacaitae (Villar) Rivas Mart. 22
Armeria lachnolepis Pomel 27
Armeria lacmonica Hausskn. 24
Armeria lanceobracteata G.H.M.Lawr. 23
Armeria langeana Henriq. 36
Armeria langeana var. *genuina* Daveau 36
Armeria langeana var. *glabra* Daveau 36
Armeria langei Boiss. ex Lange 29
Armeria langei subsp. *belmonteae* P.Silva 28

Armeria langei subsp. *belmontei* P.Silva 28
Armeria langei subsp. *deveaui* (Cout.) P.Silva 27
Armeria langei subsp. *langei* Boiss. 29
Armeria latifolia Moris 34
Armeria latifolia Willd. 35
Armeria laucheana J.N.Haage & E.Schmidt 33
Armeria leonis Sennen 29
Armeria leucantha (Boiss.) Mathon 19
Armeria leucantha Salzm. ex Boiss. 19
Armeria leucocephala Salzm. ex W.D.J.Koch 29
Armeria leucocephala subsp. *breviaristata* Arrigoni 30
Armeria leucocephala subsp. *leucocephala* 30
Armeria leucocephala subsp. *multiceps* (Wallr.) Arcang. 29
Armeria leucocephala subsp. *pubescens* (Salis) Arrigoni 29
Armeria leucocephala subsp. *soleirolii* (Duby) Arcang. . 38
Armeria leucocephala subsp. *thomasii* Nyman 30
Armeria leucocephala var. *alpina* Boiss. 29
Armeria leucocephala var. *glabra* Fiori 30
Armeria leucocephala var. *hirticula* Boenn. 30
Armeria leucocephala var. *kochii* (Boiss.) G.H.M.Lawr. 30
Armeria leucocephala var. *multiceps* (Wallr.) Mori 29
Armeria leucocephala var. *procera* Boiss. 29
Armeria leucocephala var. *pubescens* (Salis) G.H.M.Lawr. 29
Armeria leucocephala var. *soleirolii* (Duby) Boiss. 38
Armeria linearifolia (Hook.) Kuntze 100
Armeria linkiana Nieto Fel. 30
Armeria littoralis Boiss. 39
Armeria littoralis H.J.Coste 27
Armeria littoralis Hoffmanns. & Link 30
Armeria littoralis var. *ancarensis* (Merino) G.H.M.Lawr. 27
Armeria littoralis var. *hispida* Daveau 30
Armeria littoralis Willd. 30
Armeria longearistata Bourg. ex Reut. 19
Armeria longevaginata Batt. 25
Armeria longiaristata Boiss. & Reut. 39
Armeria longiaristata subsp. *vestita* (Willk.) Nyman 22
Armeria longifolia (Nutt.) Kuntze 100
Armeria longifolia Desf. 35
Armeria longivaginata Batt. 25
Armeria lusitanica Link ex Boiss. 35
Armeria macloviana Cham. 26
Armeria macloviana subsp. *andina* (Poepp. ex Boiss.) Iversen 26
Armeria macloviana subsp. *californica* (Boiss.) Iversen 32
Armeria macrophylla Boiss. & Reut. 30
Armeria macropoda Boiss. 30
Armeria maculata (L.) Kuntze 100
Armeria maculata Poepp. ex Boiss. 26
Armeria maderensis Lowe 30
Armeria maghrebensis Donad. 37
Armeria maghrebensis var. *ebracteolata* Donad. 37
Armeria maghrebensis var. *mamorensis* Donad. 38
Armeria magna Sennen 30
Armeria majellensis Boiss. 28
Armeria majellensis subsp. *ausonia* Bianchini 28
Armeria majellensis subsp. *majellensis* 28
Armeria majellensis subsp. *orphanidis* (Boiss.) Nyman. 23
Armeria majellensis var. *brachyphylla* Boiss. 23
Armeria majellensis var. *elatior* Levier ex Arch. 23
Armeria majellensis var. *leucantha* Boiss. 23
Armeria majellensis var. *marginata* Levier 21
Armeria majellensis var. *rhodopaea* Velen. 25
Armeria majellensis var. *stenophylla* Beck 24
Armeria majellensis var. *subalpina* Levier 28
Armeria major (Jacq.) Grande 101
Armeria malacitana Nieto Fel. 30
Armeria malinvaudii H.J.Coste & Soulié 30
Armeria marginata (Levier) Bianchini 21
Armeria mariae Sennen 23
Armeria maritima (Mill.) Willd. 30, 33
Armeria maritima f. *anisophylla* Bernis 22
Armeria maritima f. *aristulata* Bernis 38
Armeria maritima f. *elongata* (Hoffm.) Blytt 32
Armeria maritima f. *laucheana* (J.N.Haage & E.Schmidt) Voss 33
Armeria maritima f. *legionensis* Bernis 25
Armeria maritima f. *littorea* Bernis 36
Armeria maritima f. *losae* Bernis 23
Armeria maritima f. *occasiana* Bernis 20
Armeria maritima f. *onsiensis* Bernis 101
Armeria maritima Girard ex Boiss. 37
Armeria maritima subsp. *alpina* (DC.) P.Silva 20
Armeria maritima subsp. *andina* (Poepp. ex Boiss.) D.M.Moore & B.Yates 26
Armeria maritima subsp. *anglica* Wallr. 31
Armeria maritima subsp. *arctica* (Cham.) Hultén 34
Armeria maritima subsp. *azorica* Franco 32
Armeria maritima subsp. *barcensis* (Simonk.) P.Silva ... 32
Armeria maritima subsp. *belgica* Wallr. 31
Armeria maritima subsp. *bottendorfensis* (A.G.Schulz) Rothm. 20
Armeria maritima subsp. *bourgaei* (Boiss. ex Nyman) Bernis 23
Armeria maritima subsp. *californica* (Boiss.) Porsild 32
Armeria maritima subsp. *depilata* (Bernis) Malag. 32
Armeria maritima subsp. *eifeliaca* (Petri) Lefebvre 32
Armeria maritima subsp. *elegans* (Bernis) Malag. 31
Armeria maritima subsp. *elongata* (Hoffm.) Bonnier ... 32
Armeria maritima subsp. *fontqueri* 32
Armeria maritima subsp. *gallica* Wallr. 31
Armeria maritima subsp. *germanica* Wallr. 31
Armeria maritima subsp. *glabrescens* (Lange ex Daveau) Malag. 36
Armeria maritima subsp. *halleri* (Wallr.) Rothm. 20
Armeria maritima subsp. *hornburgensis* (A.G.Schulz) Rothm. 20
Armeria maritima subsp. *interior* (Raup) A.E.Porsild 32
Armeria maritima subsp. *interior* (Raup) Lefebvre & Vekem. 32
Armeria maritima subsp. *intermedia* (T.Marsson) C.Lefèbvre ex Buttler 32
Armeria maritima subsp. *itala* Wallr. 31
Armeria maritima subsp. *juniperifolia* (Bernis) Malag. . 32
Armeria maritima subsp. *labradorica* (Wallr.) Hultén ... 34
Armeria maritima subsp. *legionensis* (Bernis) M.Laínz . 25
Armeria maritima subsp. *maderensis* (Loew) Bernis 30
Armeria maritima subsp. *majuscula* (Samp.) Malag. 33
Armeria maritima subsp. *maritima* (Mill.) Willd. 33
Armeria maritima subsp. *miscella* (Merino) Malag. 33
Armeria maritima subsp. *mulleri* (A.Huet) O.Bolòs & Vigo 35
Armeria maritima subsp. *odorata* (Samp.) Bernis 29

Armeria maritima subsp. *planifolia* (Syme) Á.Löve & D.Löve 33
Armeria maritima subsp. *pubigera* (Desf.) Malag. 36
Armeria maritima subsp. *purpurea* (W.D.J.Koch) Á.Löve & D.Löve 33
Armeria maritima subsp. *serpentini* (Gauckler) Rothm. 20
Armeria maritima subsp. *sibirica* (Turcz. ex Boiss.) Nyman 33
Armeria maritima subsp. *smolikana* Babal. 24
Armeria maritima subvar. *carratracensis* Bernis 39
Armeria maritima subvar. *confusa* Bernis 21
Armeria maritima subvar. *elegans* Bernis 31
Armeria maritima subvar. *gracilifolia* Bernis 25
Armeria maritima subvar. *javalambrica* Bernis 19
Armeria maritima subvar. *juniperifolia* Bernis 32
Armeria maritima subvar. *littorifuga* Bernis 37
Armeria maritima subvar. *marginata* (Levier) Bernis ... 21
Armeria maritima subvar. *protypica* Bernis 37
Armeria maritima subvar. *salvadorii* Bernis 35
Armeria maritima var. *albi* Bernis 18
Armeria maritima var. *alboi* Bernis 18
Armeria maritima var. *allioides* Bernis 39
Armeria maritima var. *alpina* (DC.) Bernis 20
Armeria maritima var. *alpina* (Willd.) G.H.M.Lawr. 20
Armeria maritima var. *alpinifolia* (Pau & Font Quer) G.H.M.Lawr. 31
Armeria maritima var. *ambifaria* (Focke) G.H.M.Lawr. 31
Armeria maritima var. *andina* (Poepp. ex Boiss.) G.H.M.Lawr. 26
Armeria maritima var. *anomala* Bernis 21
Armeria maritima var. *arctica* (Cham.) Bernis 34
Armeria maritima var. *barcensis* (Simonk.) G.H.M.Lawr. 32
Armeria maritima var. *bilbilitana* Bernis 21
Armeria maritima var. *bourgaei* (Boiss. ex Nyman) Bernis 23
Armeria maritima var. *caballeroi* Bernis 23
Armeria maritima var. *californica* (Boiss.) G.H.M.Lawr. 32
Armeria maritima var. *canescens* (Host) Bernis 23
Armeria maritima var. *curvifolia* (Bertero) G.H.M.Lawr. 26
Armeria maritima var. *denticulata* (Bertol.) Bernis 27
Armeria maritima var. *depilata* Bernis 32
Armeria maritima var. *duriuscula* (Bab.) Bab. 31
Armeria maritima var. *genuina* Gren & Godr. 101
Armeria maritima var. *girardii* Bernis 28
Armeria maritima var. *goodalliana* T.R.Dudley 27
Armeria maritima var. *hortensis* Wallr. 31
Armeria maritima var. *interior* (Raup) G.H.M.Lawr. 32
Armeria maritima var. *labradorica* (Wallr.) G.H.M.Lawr. 34
Armeria maritima var. *leucocephala* (Salzm. ex W.D.J.Koch) Bernis 29
Armeria maritima var. *linkii* Gren & Godr. 31
Armeria maritima var. *macloviana* (Cham.) G.H.M.Lawr. 26
Armeria maritima var. *macropoda* (Boiss.) Bernis 30
Armeria maritima var. *magellanica* (Boiss.) G.H.M.Lawr. 26
Armeria maritima var. *majellensis* (Boiss.) Bernis 28
Armeria maritima var. *maria* G.H.M.Lawr. 31
Armeria maritima var. *maritima* (Mill.) Willd. 33
Armeria maritima var. *maroccana* (Font Quer) G.H.M.Lawr. 27
Armeria maritima var. *matritensis* (Pau) Bernis 20
Armeria maritima var. *merinoi* Bernis 34
Armeria maritima var. *morisii* (Boiss.) Bernis 35
Armeria maritima var. *multiceps* (Wallr.) Bernis 29
Armeria maritima var. *occasiana* (Bernis) O.Bolòs & Vigo 20
Armeria maritima var. *patagonica* (Phil.) G.H.M.Lawr. 27
Armeria maritima var. *pauana* Bernis 35
Armeria maritima var. *planifolia* (Syme) Bab. 31
Armeria maritima var. *profilicaulis* Bernis 33
Armeria maritima var. *provillosa* Bernis 39
Armeria maritima var. *pubescens* (Link) Bab. 33
Armeria maritima var. *pubescens* (Sowerby) Rchb. 33
Armeria maritima var. *pubigera* (Boiss.) Bab. 31
Armeria maritima var. *purpurea* (W.D.J.Koch) G.H.M.Lawr. 33
Armeria maritima var. *rumelicina* Bernis 19
Armeria maritima var. *ruscinonensis* (Girard) G.H.M.Lawr. 37
Armeria maritima var. *salmantica* Bernis 37
Armeria maritima var. *sampaioi* Bernis 37
Armeria maritima var. *sardoa* (Spreng.) Bernis 37
Armeria maritima var. *scotica* (Boiss.) Bab. 33
Armeria maritima var. *scotica* (Boiss.) P.D.Sell 32, 33
Armeria maritima var. *segoviensis* Gand. ex Bernis 22
Armeria maritima var. *serpentini* Gauckler 20
Armeria maritima var. *sibirica* (Turcz. ex Boiss.) A.Blytt 33
Armeria maritima var. *soleirolii* (Duby) Bernis 38
Armeria maritima var. *sylvestris* Wallr. 31
Armeria maritima var. *variegata* hort. ex F.T.Hubb. ... 101
Armeria maritima var. *viciosoi* Bernis 29
Armeria maritima var. *willkommiana* Bernis 23
Armeria masguindalii (Pau) Nieto Fel. 34
Armeria matritensis Pau 19
Armeria mauritanica Boiss. 37
Armeria mauritanica var. *amplifoliata* (Pau) G.H.M.Lawr. 37
Armeria mauritanica var. *boissieriana* (Coss.) Quézel & Santa 34
Armeria mauritanica var. *calva* Boiss. 37
Armeria mauritanica var. *chamaeropicola* (Pau) Bernis 37
Armeria mauritanica var. *ciliolata* Boiss. 37
Armeria mauritanica var. *minor* Batt. 37
Armeria mauritanica var. *safiensis* Maire 38
Armeria mauritanica var. *simplex* (Pomel) Faure 37
Armeria mauritanica var. *soloitana* Maire 38
Armeria mauritanica Wallr. 34
Armeria meridionalis Poepp. ex Boiss. 26
Armeria merinoi (Bernis) Nieto Fel. & Silva Pando 34
Armeria microcephala Welw. 30
Armeria miscella Merino 33
Armeria montana (Mill.) F.Herm. 20
Armeria montana f. *sicorisiensis* Sennen 19
Armeria montana G.Don ex Loudon 33
Armeria montana Ray ex Wallr. 19
Armeria montana var. *stenophylla* Rouy 19
Armeria montcaunica Pau ex Losa 21
Armeria montiberica García Cardo, Fabado & Mateo ... 34
Armeria morisii Boiss. 34
Armeria morisii var. *gussonei* (Boiss.) Parl. 35
Armeria morisii var. *macropoda* (Boiss.) Parl. 30

Armeria morisii var. *maderensis* (Lowe ex Boiss.) G.H.M.Lawr. 30
Armeria muelleri A.Huet 35
Armeria mulleri A.Huet 35
Armeria multiceps subsp. *meridionalis* Arrigoni 30
Armeria multiceps subsp. *multiceps* 30
Armeria multiceps Wallr. 29
Armeria muscodes (Nutt.) Kuntze 100
Armeria nana (Nutt.) Kuntze 100
Armeria nebrodensis (Guss.) Boiss. 24
Armeria neglecta Girard 30
Armeria nuriensis Sennen 35
Armeria odorata Samp. 29
Armeria orophila Sennen 35
Armeria orphanidis Boiss. 23
Armeria ovata (L.) Kuntze 100
Armeria paniculata (L.) Kuntze 100
Armeria pantocsekii (Strobl) K.Malý 24
Armeria parvula Franco 36
Armeria patagonica Phil. 26
Armeria pauana (Bernis) Nieto Fel. 35
Armeria petri-ludovicii Sennen 21
Armeria petteriana C.Presl 23
Armeria pilosa (L.) Kuntze 100
Armeria pinifolia (Brot.) Hoffmanns. & Link 35
Armeria pinifolia Bourg. ex Nyman 30
Armeria pinifolia subsp. *macrophylla* (Boiss. & Reut.) Bernis 30
Armeria piorum Sennen 21
Armeria planifolia Nyman 31
Armeria plantaginea 18, 19, 21, 22, 25, 28, 30, 34, 35, 97, 98
Armeria plantaginea (All.) Willd. 21
Armeria plantaginea Bertol. 21
Armeria plantaginea Boiss. 28
Armeria plantaginea subsp. *alliacea* (Cav.) Malag. 18
Armeria plantaginea subsp. *bupleuroides* (Gren. & Godr.) Nyman 21
Armeria plantaginea subsp. *burgalensis* (Sennen & Elias) Malag. 21
Armeria plantaginea subsp. *castellana* (Boiss. & Reut. ex Leresche) Nyman 19
Armeria plantaginea subsp. *choulettiana* (Pomel) Sauvage & Vindt 25
Armeria plantaginea subsp. *leucantha* (Boiss.) Sauvage & Vindt 22
Armeria plantaginea subsp. *magna* (Sennen) Malag. 30
Armeria plantaginea subsp. *montcaunica* (Pau ex Losa) Malag. 21
Armeria plantaginea subsp. *oretana* (Bernis) Malag. 22
Armeria plantaginea subsp. *piorum* (Sennen) Malag. 21
Armeria plantaginea subsp. *plantaginea* 21
Armeria plantaginea subsp. *praecox* (Jord.) Nyman 22
Armeria plantaginea subsp. *rigida* (Wallr.) Malag. 19
Armeria plantaginea subsp. *segoviensis* (Gand. ex Bernis) Rivas Mart. 22
Armeria plantaginea subsp. *sicorisiensis* (Sennen) Malag. 19
Armeria plantaginea var. *alba* hort. ex F.T.Hubb. 97
Armeria plantaginea var. *brachylepis* Boiss. 19
Armeria plantaginea var. *brachyphylla* Boiss. 19
Armeria plantaginea var. *bupleuroides* (Gren. & Godr.) G.H.M.Lawr. 21
Armeria plantaginea var. *gigantea* hort. ex F.T.Hubb. 97
Armeria plantaginea var. *grandiflora* hort. ex F.T.Hubb. 98
Armeria plantaginea var. *leucantha* Boiss. 19
Armeria plantaginea var. *longibracteata* Boiss. 19
Armeria plantaginea var. *masguindalii* Pau 34
Armeria plantaginea var. *rosea* hort. ex F.T.Hubb. 98
Armeria plantaginea var. *rubra* hort. ex F.T.Hubb. 98
Armeria plantaginea var. *scorzonerifolia* Boiss. 19
Armeria plantaginea var. *splendens* hort. ex F.T.Hubb. 98
Armeria plantaginea Webb 35
Armeria platyphylla (Daveau) Franco 35
Armeria pocutica Pawł. 35
Armeria pocutica subsp. *alpina* (Willd.) P.Silva 20
Armeria portensis G.H.M.Lawr. 36
Armeria praecox Jord. In Boreau 22
Armeria pseudarmeria (Murray) Mansf. 35
Armeria pseudarmeria Murray 35
Armeria pseud-armeria Murray 35
Armeria pseudarmeria subsp. *alboi* (Bernis) Malag. 18
Armeria pseudarmeria subsp. *allioides* (Boiss.) Malag. 19
Armeria pseudarmeria subsp. *bourgaei* (Boiss. ex Merino) Malag. 23
Armeria pseudarmeria subsp. *carratracensis* (Bernis) Malag. 39
Armeria pseudarmeria subsp. *castellata* (Boiss. & Reut. ex Leresche) Malag. 25
Armeria pseudarmeria subsp. *ciliata* (Lange) Malag. 26
Armeria pseudarmeria subsp. *eriophylla* (Willk.) Malag. 27
Armeria pseudarmeria subsp. *littoralis* (Hoffmanns. & Link) Malag. 30
Armeria pseudarmeria subsp. *longifolia* (Willk.) Malag. 30
Armeria pseudarmeria subsp. *monchiquensis* (Bernis) Malag. 35
Armeria pseudarmeria subsp. *pauana* (Bernis) Malag. 35
Armeria pseudarmeria subsp. *provillosa* (Bernis) Malag. 35
Armeria pseudarmeria subsp. *pseudarmeria* (Murray) Mansf. 35
Armeria pseudarmeria subsp. *sampaioi* (Bernis) Malag. 37
Armeria pseudarmeria subsp. *sobrinhoi* (Bernis) Malag. 35
Armeria pseudarmeria subsp. *tejedensis* (Bernis) Malag. 36
Armeria pseudarmeria subsp. *transmontana* (Samp.) Malag. 38
Armeria pseudarmeria subsp. *villosa* (Girard) Malag. 39
Armeria pseudarmeria subvar. *carratracensis* Bernis 39
Armeria pseudarmeria subvar. *tejedensis* Bernis 36
Armeria pseudarmeria var. *alboi* Bernis 18
Armeria pseudarmeria var. *pauana* Bernis 35
Armeria pseudarmeria var. *provillosa* Bernis 35
Armeria pseudarmeria var. *sampaioi* Bernis 37
Armeria pseudarmeria var. *sobrinhoi* Bernis 35
Armeria pseudoarmeria Brot. 35
Armeria pubescens B.Heyne ex Steud. 31
Armeria pubescens Link 33
Armeria pubescens subsp. *expansa* (Wallr.) Nyman 36
Armeria pubescens subsp. *pubescens* Link 33
Armeria pubigera (Desf.) Boiss. 36

Armeria pubigera var. *glabrescens* Lange ex Daveau ... 36
Armeria pubigera var. *hirta* Lange ex Daveau 36
Armeria pubigera var. *scotica* Boiss. 31
Armeria pubinervis Boiss. 36
Armeria pubinervis subsp. *orissonensis* Donad. 36
Armeria pungens (Brot.) Hoffmanns. & Link 36
Armeria pungens subsp. *bernisiana* Malag. 36
Armeria pungens subsp. *major* (Daveau) Franco 36
Armeria pungens var. *aciculifolia* Franco 36
Armeria pungens var. *major* Daveau 36
Armeria pungens var. *pungens* (Brot.) Hoffmanns. & Link 36
Armeria pungens var. *velutina* Cout. 36
Armeria purpurea W.D.J.Koch 33
Armeria quichiotis (Gonz.Albo) A.W.Hill 36
Armeria reptans (Michx.) Kuntze 100
Armeria rhenana Gremli 33
Armeria rhodopea Velen. 25
Armeria richardsonii (Hook.) Kuntze 100
Armeria rigida f. *asperrima* Sennen 22
Armeria rigida f. *elongata* Sennen 30
Armeria rigida subsp. *latifolia* Wallr. 19
Armeria rigida Wallr. 19
Armeria rivasmartinezii Sard.Rosc. & Nieto Fel. 36
Armeria roemeriana (Scheele) Kuntze 100
Armeria rothmaleri Nieto Fel. 36
Armeria rouyana Daveau 36, 37
Armeria rouyana f. *littorea* (Bernis) Bernis 36
Armeria rouyana subsp. *littorea* (Bernis) Malag. 36
Armeria rouyana subsp. *rouyana* Daveau 37
Armeria rumelica Boiss. 25
Armeria rumelica f. *adamovicii* (Halácsy) Hayek 25
Armeria rumelica f. *isophylla* Stoj. & Jordanov ex Novák 25
Armeria rumelica f. *rhodopaea* (Velen.) Ančev 25
Armeria rumelica f. *rhodopaea* (Velen.) Hayek 25
Armeria rumelica f. *setacea* Novák 25
Armeria rumelica f. *temskyana* (Degen & Dörfl.) Micevski 25
Armeria rumelica var. *adamovicii* (Halácsy) Novák 25
Armeria rumelica var. *pseudocanescens* (Halácsy) Novák 25
Armeria rumelica var. *rhodopaea* (Velen.) Beck 25
Armeria rumelica var. *tempskyana* (Degen & Dörfl.) Vandas 25
Armeria ruscinonensis Girard 37
Armeria ruscinonensis subsp. *littorifuga* (Bernis) Malag. 37
Armeria ruscinonensis subsp. *ruscinonensis* Girard 37
Armeria ruscinonensis subvar. *littorifuga* Bernis 37
Armeria sabulosa Jord. ex Boreau 19
Armeria sabulosa var. *serpentini* Legrand 19
Armeria sampaioi (Bernis) Nieto Fel. 37
Armeria sancta Janka 37
Armeria sanguinolenta Wallr. 32
Armeria sardoa Spreng. 37
Armeria sardoa subsp. *genargentea* Arrigoni 37
Armeria sardoa subsp. *sardoa* Spreng. 37
Armeria sardoa var. *nebrodensis* (Guss.) Parl. 24
Armeria saviana Selvi 37
Armeria scabra Kunze ex Boiss. 27
Armeria scabra Pall. ex Schult. 33
Armeria scabra subsp. *arctica* (Cham.) Iversen 34
Armeria scabra subsp. *labradorica* (Wallr.) Iversen 34
Armeria scabra subsp. *sibirica* (Turcz. ex Boiss.) Hyl. 33
Armeria scabra var. *glabricalyx* Tzvelev 34
Armeria scabra var. *labradorica* (Wallr.) Tzvelev 34
Armeria scabra Willd. 37
Armeria scabriuscula Kunze ex Boiss. 26
Armeria scorzonerifolia Balb. & Nocca 101
Armeria scorzonerifolia Friv. ex Nyman 25
Armeria scorzonerifolia Link. 22
Armeria scorzonerifolia Ten. 30
Armeria scorzonerifolia Willd. 19
Armeria sect. *Macrocentron* Boiss. 96
Armeria sect. *Plagiobasis* Boiss. 96
Armeria segoviensis Gand. 19
Armeria segoviensis subsp. *lacaitae* (Villar) Rivas Mart. 22
Armeria sennenii G.H.M.Lawr. 21
Armeria setacea Delile ex Nyman 28
Armeria seticeps Rchb. 19, 37
Armeria seticeps Rchb. f. 19
Armeria sibirica (L.) Kuntze 100
Armeria sibirica Turcz. ex Boiss. 33
Armeria sicorisiana Sennen 19
Armeria sicula Heldr. ex Boiss. 24
Armeria simplex Pomel 37
Armeria sobrinhoi subvar. *monchiquensis* Bernis 35
Armeria soleirolii (Duby) Godr. 38
Armeria speciosa (Pursh) Kuntze 100
Armeria spinulosa Boiss. 38
Armeria splendens (Lag. & Rodr.) Webb 38
Armeria splendens Boiss. 38
Armeria splendens subsp. *bigerrensis* (Vicioso & Beltrán) P.Silva 23
Armeria splendens subsp. *splendens* 38
Armeria stellaria (A.Gray) Kuntze 100
Armeria stenophylla Girard 19
Armeria subsect. *Astegiae* Boiss. 96
Armeria subsect. *Holotricae* Boiss. 96
Armeria subsect. *Macrostegiae* Boiss. 97
Armeria subsect. *Microstegiae* Boiss. 97
Armeria subsect. *Pleurotrichae* Boiss. 97
Armeria subulata (L.) Kuntze 100
Armeria sulcitana Arrigoni 38
Armeria tenorei (Fiori) Lacaita 24
Armeria tenuifolia Phil. 26
Armeria tenuis Balb. ex Boiss. 37
Armeria tenuis var. *elata* Wallr. 27
Armeria tenuis var. *humilis* Wallr. 19
Armeria tenuis Wallr. 27
Armeria thessala (Boiss.) Boiss. & Heldr. 25
Armeria thomasii Nyman 30
Armeria tingitana Boiss. & Reut. 38
Armeria tingitana var. *chamaeropicola* (Pau) Donad. .. 37
Armeria trachyphylla Lange 38
Armeria transmontana (Samp.) G.H.M.Lawr. 38
Armeria transmontana subsp. *aristulata* (Bernis) Bernis 38
Armeria transmontana subsp. *pseudotransmontana* Franco 38
Armeria transmontana subsp. *transmontana* (Samp.) G.H.M.Lawr. 38
Armeria trianoi Nieto Fel. 38
Armeria trigoloides Ebel 19

Armeria trigonoides Ebel 19
Armeria trojana Bokhari & Quézel 38
Armeria undulata (Bory & Chaub.) Boiss. 38
Armeria undulata f. *brevifolia* Novák 38
Armeria undulata f. *longifolia* Novák 39
Armeria undulata var. *capitella* (Pau) Rivas Goday & Bellot 25
Armeria vandasii Hayek 39
Armeria vasconica Sennen 24
Armeria vasconica Sennen ex Losa 24
Armeria velutina Welw. ex Boiss. & Reut. 39
Armeria vestita Willk. 22
Armeria villosa Cout. 27
Armeria villosa Girard 39
Armeria villosa subsp. *alcaracensis* Nieto Fel. 39
Armeria villosa subsp. *bernisii* Nieto Fel. 39
Armeria villosa subsp. *carratracensis* (Bernis) Nieto Fel. 39
Armeria villosa subsp. *enritrianoi* Blanca, Cueto, J.Fuentes & S.Tello 39
Armeria villosa subsp. *longiaristata* (Boiss. & Reut.) Nieto Fel. 39
Armeria villosa subsp. *longiristata* (Boiss. & Reut.) Nieto Fel. 39
Armeria villosa subsp. *provillosa* (Bernis) Nieto Fel. 39
Armeria villosa subsp. *serpentinicola* Cabezudo, Casim.-Sor.Solanas & Pérez Lat. 39
Armeria villosa subsp. *villosa* Girard 39
Armeria vulgaris f. *ambifaria* (Focke) W.F.Christ. 31
Armeria vulgaris f. *arctica* Cham. 34
Armeria vulgaris f. *longiscapa* F.Petri 31
Armeria vulgaris f. *persicina* W.F.Christ. 32
Armeria vulgaris f. *purpurea* T.Marsson 31
Armeria vulgaris f. *tenorei* Fiori 24
Armeria vulgaris Moris ex Nyman 24
Armeria vulgaris subsp. *arctica* (Cham.) Hultén 34
Armeria vulgaris subsp. *arctica* (Cham.) Nyman 34
Armeria vulgaris subsp. *elongata* (Hoffm.) F.Petri 32
Armeria vulgaris subsp. *intermedia* (T.Marsson) Nordh. 31
Armeria vulgaris subsp. *maritima* (Mill.) F.Petri 31
Armeria vulgaris subsp. *plantaginea* (All.) Syme 21
Armeria vulgaris subsp. *purpurea* (W.D.J.Koch) Nyman 33
Armeria vulgaris subsp. *rhenana* (Gremli) Nyman 33
Armeria vulgaris subsp. *serpentini* (Gauckler) Holub 20
Armeria vulgaris subsp. *vulgaris* Willd. 33
Armeria vulgaris var. *alba* L.H.Bailey 31
Armeria vulgaris var. *alpina* (Willd.) F.Petri 20
Armeria vulgaris var. *alpina* Fiori 20
Armeria vulgaris var. *calaminaria* F.Petri 31
Armeria vulgaris var. *denticulata* (Bertol.) Fiori 27
Armeria vulgaris var. *eifeliaca* F.Petri 31
Armeria vulgaris var. *elongata* (Hoffm.) F.Petri 32
Armeria vulgaris var. *elongata* (Hoffm.) Mert. & W.D.J.Koch 32
Armeria vulgaris var. *elongata* (Hoffm.) T.Marsson 32
Armeria vulgaris var. *glabra* T.Marsson 31
Armeria vulgaris var. *grandiflora* L.H.Bailey 31
Armeria vulgaris var. *halleri* (Wallr.) F.Petri 20
Armeria vulgaris var. *intermedia* T.Marsson 32
Armeria vulgaris var. *labradorica* (Wallr.) F.Petri 34
Armeria vulgaris var. *laucheana* (J.N.Haage & E.Schmidt) L.H.Bailey 33
Armeria vulgaris var. *longiinvolucrata* F.Petri 31
Armeria vulgaris var. *marginata* (Levier) Fiori 21
Armeria vulgaris var. *maritima* (Mill.) T.Marsson 31
Armeria vulgaris var. *maritima* Rosenv. 31
Armeria vulgaris var. *nana* Bolzon 99
Armeria vulgaris var. *palatina* F.Petri 31
Armeria vulgaris var. *planifolia* Syme 31
Armeria vulgaris var. *plantaginea* (Willd.) F.Petri 21
Armeria vulgaris var. *pubescens* (Sowerby) F.Petri 31
Armeria vulgaris var. *purpurea* (W.D.J.Koch) Mert. & W.D.J.Koch 33
Armeria vulgaris var. *purpurea* hort. ex L.H.Bailey 33
Armeria vulgaris var. *rubra* hort. ex L.H.Bailey 31
Armeria vulgaris var. *rugica* F.Petri 31
Armeria vulgaris var. *sardoa* (Spreng.) Fiori 37
Armeria vulgaris var. *sibirica* (Turcz. ex Boiss.) Rosenv. 33
Armeria vulgaris var. *splendens* L.H.Bailey 31
Armeria vulgaris Vis. 23
Armeria vulgaris Willd. 18, 33
Armeria welwitschii Boiss. 39
Armeria welwitschii var. *cinerea* (Boiss. & Welw.) Govaerts 39
Armeria welwitschii var. *diversifolia* Franco 39
Armeria welwitschii var. *longibracteata* Daveau 39
Armeria welwitschii var. *platyphylla* Daveau 35
Armeria welwitschii var. *stenophylla* Daveau 39
Armeria Willd. 2, 18
Armeria willkommii Henriq. 29
Armeria willkommii var. *odorata* Samp. 29
Armeriaceae Horan. 1
Armeriastrum (Jaub. & Spach) Lindl. 2
Armeriastrum acerosum (Boiss.) Kuntze 3
Armeriastrum acmostegium (Boiss. & Buhse) Kuntze 3
Armeriastrum araxanum (Bunge) Kuntze 4
Armeriastrum aristulatum (Bunge) Kuntze 4
Armeriastrum armenum (Boiss.) Kuntze 4
Armeriastrum aspadanum (Bunge) Kuntze 4
Armeriastrum assyriacum (Boiss.) Kuntze 18
Armeriastrum atropatanum (Bunge) Kuntze 4
Armeriastrum auganum (Bunge) Kuntze 5
Armeriastrum avenaceum (Bunge) Kuntze 5
Armeriastrum balansae (Boiss. ex Bunge) Kuntze 4
Armeriastrum baltanense (Boiss. & Hausskn. ex Boiss.) Kuntze 4
Armeriastrum bodeanum (Bunge) Kuntze 5
Armeriastrum brachyphyllum (Boiss.) Kuntze 5
Armeriastrum brachystachyum (Boiss. ex Bunge) Kuntze 5
Armeriastrum bracteatum (Boiss.) Kuntze 5
Armeriastrum bromifolium (Boiss. ex Bunge) Kuntze 5
Armeriastrum cabulicum (Boiss.) Kuntze 6
Armeriastrum caesareum (Boiss. & Balansa) Kuntze 6
Armeriastrum calvertii (Boiss.) Kuntze 6
Armeriastrum caryophyllaceum (Boiss.) Kuntze 6
Armeriastrum cataonicum (Bunge) Kuntze 13
Armeriastrum cephalotes (Boiss.) Kuntze 6
Armeriastrum curviflorum (Bunge) Kuntze 14
Armeriastrum cymosum (Bunge) Kuntze 7
Armeriastrum dianthifolium (Jaub. & Spach) Kuntze 18
Armeriastrum diapensioides (Boiss.) Kuntze 7
Armeriastrum distachyum (Boiss.) Kuntze 7
Armeriastrum echinus (L.) Kuntze 16
Armeriastrum erinaceum (Jaub. & Spach) Kuntze 7

Armeriastrum eschkerense (Boiss. & Hausskn. ex Boiss.) Kuntze 8
Armeriastrum fasciculare (Boiss.) Kuntze 8
Armeriastrum faustii (Trautv.) Kuntze 8
Armeriastrum ferox (Boiss.) Kuntze 15
Armeriastrum festucaceum (Jaub. & Spach) Kuntze 8
Armeriastrum flexuosum (Boiss. ex Bunge) Kuntze 8
Armeriastrum genistoides (Jaub. & Spach) Kuntze 8
Armeriastrum glumaceum (Boiss.) Kuntze 9
Armeriastrum griffithianum (Boiss.) Kuntze 9
Armeriastrum gulistanum (Bunge) Kuntze 9
Armeriastrum hausknechtii (Bunge) Kuntze 4
Armeriastrum heratense (Bunge) Kuntze 9
Armeriastrum horridum (Bunge) Kuntze 10
Armeriastrum huetii (Boiss.) Kuntze 10
Armeriastrum iconicum (Boiss. & Heldr.) Kuntze 10
Armeriastrum incomptum (Boiss. & Buhse) Kuntze 10
Armeriastrum karelinii (Bunge) Kuntze 10
Armeriastrum kotschyi (Boiss.) Kuntze 11
Armeriastrum kurdicum (Bunge) Kuntze 5
Armeriastrum latifolium (Boiss.) Kuntze 11
Armeriastrum laxiflorum (Boiss.) Kuntze 11
Armeriastrum lepturoides (Boiss.) Kuntze 11
Armeriastrum leucacanthum (Boiss.) Kuntze 11
Armeriastrum libanoticum (Boiss.) Kuntze 11
Armeriastrum listoniae (Boiss.) Kuntze 3
Armeriastrum longiflorum (Boiss.) Kuntze 12
Armeriastrum lycaonicum (Boiss. & Heldr.) Kuntze 12
Armeriastrum lycopodioides (Girard) Kuntze 12
Armeriastrum melananthum (Boiss.) Kuntze 12
Armeriastrum oliganthum (Boiss.) Kuntze 13
Armeriastrum olivieri (Boiss.) Kuntze 13
Armeriastrum peronini (Boiss.) Kuntze 13
Armeriastrum peroninii (Boiss.) Kuntze 13
Armeriastrum petraeum (Boiss. ex Bunge) Kuntze 13
Armeriastrum pinardii (Boiss.) Kuntze 3
Armeriastrum polystachyum (Boiss.) Kuntze 13
Armeriastrum pterostegium (Bunge) Kuntze 13
Armeriastrum puberulum (Boiss. & Balansa) Kuntze 13
Armeriastrum quinquelobum (Bunge) Kuntze 14
Armeriastrum restiaceum (Bunge) Kuntze 14
Armeriastrum ruprechtii (Bunge) Kuntze 14
Armeriastrum sahendicum (Boiss. & Buhse) Kuntze 14
Armeriastrum scabrellum (Boiss. & Hausskn. ex Boiss.) Kuntze 15
Armeriastrum schahrudicum (Bunge) Kuntze 15
Armeriastrum schirazianum (Boiss.) Kuntze 15
Armeriastrum scirpinum (Bunge) Kuntze 15
Armeriastrum senganense (Bunge) Kuntze 15
Armeriastrum setiferum (Bunge) Kuntze 41
Armeriastrum splendidum (Bunge) Kuntze 5
Armeriastrum stocksii (Boiss.) Kuntze 16
Armeriastrum subsessile (Trautv.) Kuntze 3
Armeriastrum subulatum (Boiss.) Kuntze 16
Armeriastrum talagonicum (Boiss.) Kuntze 16
Armeriastrum tartaricum (Boiss.) Kuntze 16
Armeriastrum tenuiflorum (Boiss.) Kuntze 16
Armeriastrum tenuifolium (Jaub. & Spach) Kuntze 9
Armeriastrum tomentellum (Boiss.) Kuntze 17
Armeriastrum tragacanthium (Boiss.) Kuntze 17
Armeriastrum truncatum (Bunge) Kuntze 17
Armeriastrum ulicinum (Boiss.) Kuntze 17
Armeriastrum viscidulum (Boiss.) Kuntze 18
Armeriastrum wiedemannii (Bunge) Kuntze 18
Armerieae Dumort. 2
Bakerolimon Lincz. 39, 92
Bakerolimon peruvianum (Kuntze) Lincz. 39
Bakerolimon plumosum (Phil.) Lincz. 39
Bamiania Lincz. 40
Bamiania pachycorma (Rech.f.) Lincz. 40
Bubania feei Girard 41
Bubania Girard 40, 45
Bubania migiurtina Chiov. 41
Bubania monopetala (L.) Girard 40, 45
Bukiniczia cabulica (Boiss.) Lincz. 40
Bukiniczia Lincz. 40
Caballeroa Font Quer 92
Caballeroa ifniensis (Caball.) Font Quer 92
Cephalorhizum coelicolor (Rech.f.) Rech.f. 40
Cephalorhizum micranthum Lincz. 40
Cephalorhizum oopodum Popov & Korovin 40
Cephalorhizum pachycormum Rech.f. 40
Cephalorhizum Popov & Korovin 40
Cephalorhizum popovii Lincz. 40
Cephalorhizum sect. *Sarcophyllastrum* Rech.f. 2
Cephalorhizum setiferum (Bunge) Popov & Korovin 41
Cephalorhizum turcomanicum Popov ex Lincz. 91
Cephalorrhizum Popov & Korovin 40
Ceratolimon feei (Girard) M.B.Crespo & Lledó 40, 41
Ceratolimon feei var. *feei* 41
Ceratolimon feei var. *grandiflorum* (Maire & Wilcz.) M.B.Crespo & Lledó 41
Ceratolimon M.B.Crespo & Lledó 40
Ceratolimon migiurtinum (Chiov.) M.B.Crespo & Lledó 41
Ceratolimon rechingeri (J.R.Edm.) M.B.Crespo & Lledó 41
Ceratolimon weygandiorum (Maire & Wilczek) M.B.Crespo & Lledó 41
Ceratostigma abyssinicum (Hochst.) Asch. 93
Ceratostigma asperrimum Stapf ex Prain 93
Ceratostigma Bunge 93
Ceratostigma griffithii C.B.Clarke 93
Ceratostigma minus f. *lasaense* T.X.Peng 93
Ceratostigma minus Stapf ex Prain 93
Ceratostigma plantaginoides J.W.C.Kirk 93
Ceratostigma plumbaginoides Bunge 93
Ceratostigma polhillii hort. ex Bulley 93
Ceratostigma speciosum Prain 93
Ceratostigma stapfianum Hosseus 93
Ceratostigma ulicinum Prain 93
Ceratostigma willmottianum Stapf 93
Chaetolimon (Bunge) Lincz. 41
Chaetolimon limbatum Lincz. 41
Chaetolimon setiferum (Bunge) Lincz. 41
Chaetolimon sogdianum Lincz. 41, 92
Chomutowia B.Fedtsch 2
Chomutowia ekatherinae B.Fedtsch. 2, 7
Dictyolimon gilesii (Hemsl.) Rech.f. 41
Dictyolimon griffithii (Aitch. & Hemsl.) Rech.f. 41
Dictyolimon macrorrhabdos (Boiss.) Rech.f. 41
Dictyolimon Rech.f. 41
Dictyolimon thomsonii (C.B.Clarke) Rech.f. 42
Dyerophytum (Lam.) Kuntze 93
Dyerophytum africanum (Lam.) Kuntze 93
Dyerophytum arabicum (Boiss.) M.R.Almeida 94
Dyerophytum indicum (Gibbs ex Wight) Kuntze 94
Dyerophytum pendulum (Balf.f.) Kuntze 94

Dyerophytum socotranum (Balf.f.) J.R.Edm. ex J.R.Edm., M.Malekm. & Koutr. 94
Eremolimon botschantzevii Lincz. 85
Eremolimon drepanostachyum (Ikonn.-Gal.) Lincz. 86
Eremolimon fajzievii (Zakirov ex Lincz.) Lincz. 86
Eremolimon jarmolenkoi Lincz. 86
Eremolimon kurgantjubense Lincz. 86
Eremolimon Lincz. 45, 85
Eremolimon piptopodum (Nevski) Lincz. 86
Eremolimon sogdianum (Ikonn.-Gal.) Lincz. 45, 85, 86
Eurychiton adensis Nimmo 45, 84
Eurychiton Nimmo 45
Findlaya alba Bowdich 94, 95
Findlaya Bowdich 94
Ghaznianthus Lincz. 42
Ghaznianthus rechingeri (Freitag) Lincz. 42
Gladiolimon Mobayen 2
Gladiolimon speciosissimum (Aitch. & Hemsl.) Mobayen 2, 15
Goniolimon africanum Buzurović, Bogdanović & Brullo 42
Goniolimon beckerianum Janka 44
Goniolimon besserianum Nyman 42
Goniolimon Boiss. 42
Goniolimon cabulicum (Boiss.) Mobayen 40
Goniolimon callicomum (C.A.Mey.) Boiss. 42
Goniolimon callicomum var. *callicomum* (C.A.Mey.) Boiss. 42
Goniolimon callicomum var. *gorczakovskyi* (Knjaz.) Knjaz. & Golovanov 42
Goniolimon caucasicum Klokov 42
Goniolimon collinum (Griseb.) Boiss. 43
Goniolimon collinum var. *bulgaricum* Novák 44
Goniolimon crispum (Regel) Lipsch. 44
Goniolimon cuspidatum Gamajun. 42
Goniolimon dalmaticum (C.Presl) Rchb. 42
Goniolimon desertorum (Trautv.) Klokov 43
Goniolimon dshungaricum (Regel) O.Fedtsch. & B.Fedtsch. 42
Goniolimon elatum (Fisch. ex Spreng.) Boiss. 43
Goniolimon eximium (Schenk) Boiss. 43
Goniolimon glaberrimum Klokov 45
Goniolimon gorczakovskyi Knjaz. 42
Goniolimon graminifolium (Aiton) Boiss. 43
Goniolimon griffithianum (Aitch. & Hemsl.) Mobayen . 41
Goniolimon heldreichii Halácsy 43
Goniolimon incanum (L.) Hepper 43
Goniolimon italicum Tammaro, Pignatti & Frizzi 44
Goniolimon kaufmannianum (Regel) O.Fedtsch. & B.Fedtsch. 43
Goniolimon kaufmannianum (Regel) Voss 43
Goniolimon krylovii A.V.Grebenjuk 43
Goniolimon orae-syvashicae Klokov 44
Goniolimon orthocladum Rupr. 43
Goniolimon platypterum Klokov 45
Goniolimon rubellum (S.G.Gmel.) Klokov 43
Goniolimon salicorniaceum (F.Muell.) Christenh. & Byng 90
Goniolimon sartorii Boiss. 44
Goniolimon sect. *Tricuspidaria* Lincz. 97
Goniolimon sect. *Unicuspidaria* Lincz. 97
Goniolimon serbicum Vis. 44
Goniolimon severzovii Herder 44
Goniolimon sewerzovii Herder 44
Goniolimon sewerzowii Herder 44
Goniolimon speciosum (L.) Boiss. 44
Goniolimon speciosum var. *alpinum* Herd. 43
Goniolimon speciosum var. *crispum* O.Fedtsch & B.Fedtsch. 43
Goniolimon speciosum var. *genuinum* Herd. 44
Goniolimon speciosum var. *lanceolatum* (Regel) O.Fedtsch. & B.Fedtsch. 42
Goniolimon speciosum var. *speciosum* (L.) Boiss. 44
Goniolimon speciosum var. *strictum* (Regel) T.H.Peng. .44
Goniolimon speciosum var. *typicum* O.Fedtsch & B.Fedtsch. 44
Goniolimon strictum (Regel) Lincz. 44
Goniolimon subsect. *Platycalyx* Lincz. 97
Goniolimon subsect. *Stenocalyx* Lincz. 97
Goniolimon tarbagataicum Gamajun. 43
Goniolimon tataricum (L.) Boiss. 42, 44, 45
Goniolimon tataricum f. *bulgaricum* (Novák) Ančev 44
Goniolimon tataricum f. *ciliatum* Ančev 44
Goniolimon tataricum subsp. *croaticum* Buzurović & Bogdanović 44
Goniolimon tataricum subsp. *graecum* Buzurović 44
Goniolimon tataricum subsp. *italicum* (Tammaro, Pignatti & Frizzi) Buzurović 44
Goniolimon tataricum subsp. *tataricum* (L.) Boiss. 45
Goniolimon tataricum var. *angustifolium* Boiss. 42
Goniolimon tataricum var. *besserianum* O.Fedtsch. & B.Fedtsch. 42
Goniolimon tataricum var. *desertorum* Trautv. 43
Goniolimon tataricum var. *graminifolium* Trautv. 43
Goniolimon tataricum var. *kluchoricum* Tzvelev 44
Goniolimon tataricum var. *laxiflorum* Boiss. 43
Goniolimon tataricum var. *platypterum* (Klokov) Tzvelev 45
Goniolimon tataricum var. *puberulum* Trautv. 42
Goniolimon tataricum var. *rubellum* Trautv. 43
Goniolimon tataricum var. *tauricum* (Klokov) Tzvelev ..45
Goniolimon tauricum Klokov 45
Ikonnikovia kaufmanniana (Regel) Lincz. 42, 43
Ikonnikovia kaufmanniana var. *latifolia* Z.B.Kubanskaya ex Lincz. 43
Ikonnikovia Lincz. 42
Lerrouxia Caball. 92
Lerrouxia ifniensis Caball. 92
Limoniaceae Lincz. 1
Limoniaceae Ser. 1
Limoniastrum articulatum Moench 45
Limoniastrum feei (Girard) Hook.f. ex Pax 41
Limoniastrum feei var. *grandiflorum* Maire & Wilcz. 41
Limoniastrum guyonianum Durieu ex Boiss. 45
Limoniastrum Heist. ex Fabr. 45
Limoniastrum ifniense (Caball.) Font Quer 92
Limoniastrum majus Lanza 45
Limoniastrum malenconianum Maire 92
Limoniastrum migiurtinum (Chiov.) Chiov. ex Maire 41
Limoniastrum migiurtinum Chiov. 41
Limoniastrum monopetalum (L.) Boiss. 45
Limoniastrum monopetalum subsp. *multiflorum* Bonhomme & P.Fourn. 45
Limoniastrum multiflorum C.Bonhomme & P.Fourn. 45
Limoniastrum ouarglense Pomel 45
Limoniastrum rechingeri J.R.Edm. 41
Limoniastrum reinwardtii Lanza 99

Limoniastrum sect. *Bubania* Batt. 40
Limoniastrum speciosum (L.) Moench 44
Limoniastrum subg. Bubania (Batt.) Maire 40
Limoniastrum weygandiorum Maire & Wilczek 41
Limonieae Reveal 2
Limoniodes guyonianum Siegesb. ex Kuntze 45
Limoniodes monopetalum Kuntze 45
Limoniodes Siegesb. ex Kuntze 45
Limoniopsis davisii Bokhari 45
Limoniopsis Lincz. 45
Limoniopsis owerinii (Boiss.) Lincz. 45
Limonium 46
Limonium ×abnorme (Rouy) P.Fourn. 48
Limonium ×albuferae Ferrer-Gallego, P. P., R.Roselló, M.Rosato, Rosselló & E.Laguna 49
Limonium ×ambiguum (Rouy) P.Fourn. 49
Limonium ×capdeperae Pignatti 52
Limonium ×castellonense Erben 53
Limonium ×christii G.Kunkel 87
Limonium ×coincyi Sennen 53
Limonium ×coriacifolium (Sennen) M.B.Crespo & Serra 54
Limonium ×coriacifolium Sennen 54
Limonium ×dolcheri Pignatti 57
Limonium ×dolcheri Pignatti ex Dolcher & Pignatti 57
Limonium ×erectiflorum (B.Fedtsch. & Gontsch.) A.V.Grebenjuk 58
Limonium ×escarrei L.Llorens & Tébar 58
Limonium ×eugeniae Sennen 58
Limonium ×fraternum (Sennen & Pau) M.B.Crespo 58
Limonium ×garciae Pignatti 96
Limonium ×glaucophyllum Pignatti 59
Limonium ×gougemolsii Pignatti 59
Limonium ×interjectum J.X.Soler & Rosselló 60
Limonium ×lucentinum Pignatti & Freitag 62
Limonium ×multirameum Sennen 53
Limonium ×neumanii C.E.Salmon 64
Limonium ×profusum (hort.) F.T.Hubb. ex L.H.Bailey 88
Limonium ×pseudoconfusum (Rouy) P.Fourn. 66
Limonium ×pseudodivaricatum Pignatti 66
Limonium ×pseudosmithii Pignatti 66
Limonium ×rossmaessleri (Willk.) M.B.Crespo 47
Limonium ×sennenii (Rouy) P.Fourn. 69
Limonium ×tibulatium Pignatti 71
Limonium ×valentinum (Huter, Porta & Rigo) M.B.Crespo & Lledó 71
Limonium ×viretianum Pignatti 72
Limonium ×virgatoformis (Rouy) B.Bock 72
Limonium ×virgitanum Pignatti 72
Limonium ×virgolsii f. *pseudovirgatum* Pignatti 96
Limonium ×virgolsii Pignatti 58
Limonium ×virgutiflorum Pignatti 72
Limonium acuminatum L.Bolus 48
Limonium acutifolium (Badarò ex Rchb.) C.E.Salmon 48
Limonium acutifolium subsp. *acutifolium* 48
Limonium acutifolium subsp. *acutifolium* (Badarò ex Rchb.) C.E.Salmon 48
Limonium acutifolium subsp. *bosanum* (Arrigoni & Diana) Arrigoni 52
Limonium acutifolium subsp. *cornusianum* (Arrigoni & Diana) Arrigoni 54
Limonium acutifolium subsp. *nymphaeum* (Erben) Arrigoni 64
Limonium acutifolium subsp. *obtusifolium* (Rouy) Diana 64
Limonium acutifolium subsp. *tenuifolium* (Bertol. ex Moris) Arrigoni 70
Limonium acutifolium subsp. *tharrosianum* (Arrigoni & Diana) Arrigoni 71
Limonium acutifolium var. *obtusifolium* (Rouy) C.E.Salmon 64
Limonium adilguneri Yıld. & Doğru-Koca 81
Limonium admirabile Terrones, J.Moreno, M.Á.Alonso, Juan & M.B.Crespo 49
Limonium aegaeum Erben & Brullo 49
Limonium aegusae Brullo 49
Limonium afghanicum Erben & Podlech 80
Limonium africanum Mill. 89
Limonium afrum (Pignatti) Domina 49
Limonium albarracinense Pau ex Ferrer-Gallego, P. P. & R.Roselló 49
Limonium albidum (Guss.) Pignatti 49
Limonium albidum subsp. *cyprium* Meikle 55
Limonium albomarginatum Brullo 49
Limonium album (Coincy) Sennen 49
Limonium alcudianum Erben 49
Limonium algarvense Erben 49
Limonium algusae (Brullo) Greuter 49
Limonium alicunense Gómiz 49
Limonium alleizettei (Pau) Brullo 47
Limonium alleizettii Balls 47
Limonium almeriense Pount 58
Limonium altum P.D.Sell 49
Limonium alutaceum (Steven) Kuntze 49
Limonium amblyolobum Ikonn.-Gal. 83
Limonium ammochostianum Erben, Christodoulou, Hand & Kefalas 49
Limonium ammophilon (Papatsou & Phitos) Domina 49
Limonium amoenum (C.H.Wright) R.A.Dyer 73
Limonium amopicum Erben & Brullo 49
Limonium ampuriense Arrigoni & Diana 49
Limonium amynclaeum Pignatti 49
Limonium anatolicum Hedge 75
Limonium anceps (Regel) Kuntze 91
Limonium anfractum (C.E.Salmon) C.E.Salmon 56
Limonium anglicum (Ingr.) P.D.Sell 49
Limonium angustatum (A. Gray) Small 77
Limonium angustebracteatum Erben 49
Limonium angustibracteatum Erben 49
Limonium angustifolium (Tausch) Degen 76
Limonium angustifolium (Tausch) Turrill 76
Limonium anthericoides (Schltr.) R.A.Dyer 90
Limonium antipaxorum R.Artelari 49
Limonium antonii-llorensii L.Llorens 49
Limonium aphroditae R.Artelari & Georgiou 49
Limonium apulum Brullo 49
Limonium arabicum (Jaub. & Spach) Kuntze 84
Limonium aragonense (Debeaux ex Willk.) Font Quer 49
Limonium aragonense (Debeaux ex Willk.) Pignatti 50
Limonium aragonense subsp. *ruizii* (Font Quer) Fern.Casas & Muñoz Garm. 68
Limonium arborescens (Brouss.) Kuntze 87
Limonium arboreum (Willd.) Erben, A.Santos & Reyes-Bet. 87
Limonium arboreum (Willd.) H. Arnaud 87
Limonium arbusculum (Maxim.) Makino 84

Limonium arbusculum var. *luteum* H.Hara 84
Limonium archaeothirae Erben & Brullo 50
Limonium archeothirae Erben & Brullo.........................50
Limonium arcuatum R.Artelari.....................................50
Limonium arenosum Erben..50
Limonium argentarium Pignatti...................................63
Limonium artelariae Koutr...50
Limonium articulatum (Loisel.) Kuntze 50
Limonium articulatum subsp. *dictyocladum*................70
Limonium articulatum subsp. *pseudarticulatum* (Erben) O.Bolòs & Vigo 66
Limonium artruchium Erben 50
Limonium asparagoides (Coss. & Durieu ex Batt.) Maire 47
Limonium asperrimum Maire 48
Limonium asterotrichum (C.E.Salmon) C.E.Salmon.......76
Limonium astipaleanum Erben & Brullo 50
Limonium astypaleanum Erben & Brullo 50
Limonium athinense Erben & Brullo 50
Limonium atticum Erben & Brullo................................50
Limonium aucheri (Girard) Greuter & Raus 50
Limonium augustatum (A.Gray) Small 77
Limonium aureum (L.) Chaz. 81
Limonium aureum (L.) Hill..82
Limonium aureum (L.) Hill ex Kuntze 82
Limonium aureum var. *aureum*....................................82
Limonium aureum var. *dielsianum* (Wangerin) T.H.Peng 82
Limonium aureum var. *maduoensis* Y.H.Wu 82
Limonium aureum var. *potaninii* (Ikonn.-Gal.) T.H.Peng 83
Limonium auriculae-ursifolium (Pourr.) Druce.............50
Limonium auriculae-ursifolium subsp. *lusitanicum* (Pignatti) Pignatti 65
Limonium auriculae-ursifolium subsp. *multillorum* (Pignatti) Pignatti 63
Limonium auriculifolium (Vahl) Druce 50
Limonium auriculifolium var. *dodarti* (Girard) Druce....57
Limonium aurigniense (Ingr.) P.D.Sell 50
Limonium australe (R.Br.) Kuntze 82
Limonium australe var. *australe* 82
Limonium australe var. *baudinii* (Lincz.) A.M.Gray 82
Limonium avei (De Not.) Brullo....................................48
Limonium avenaceum (C.H.Wright) R.A.Dyer 50
Limonium axillare (Forssk.) Kuntze 84
Limonium bahamense (Griseb.) Britton........................73
Limonium bahamense var. *haitense* (S.F.Blake) Alain...74
Limonium bahamense var. *haitiense* (S.F.Blake) Alain..74
Limonium balearicum (Pignatti) Brullo 50
Limonium barceloi Gil & L.Llorens................................50
Limonium battandieri Greuter & Raus..........................50
Limonium baudinii Lincz...82
Limonium beaumierianum (Coss. ex Maire) Maire.......88
Limonium beaumierianum var. *akkense* (Coss. ex Batt.) Maire 88
Limonium beaumierianum var. *annuum* (Maire) Maire 89
Limonium beaumierianum var. *dubium* Maire.............89
Limonium beaumierianum var. *glabrescens* Maire.......89
Limonium beaumierianum var. *leucocalyx* (Maire) Maire 89
Limonium beaumierianum var. *tripeaui* (Maire) Maire 88
Limonium beaumierianum var. *violascens* Maire 89
Limonium behen (Drejer) Kuntze 79, 80
Limonium bellidifolium (Gouan) Dumort. 80
Limonium bellidifolium subsp. *caspium* (Willd.) P.Fourn. 80
Limonium bellidifolium subsp. *dubyi* (Gren. & Godr.) P.Fourn. 81
Limonium bellidifolium var. *bellidifolium* 80
Limonium bellidifolium var. *prostratum* (Beauverd) Rech.f. 80
Limonium benmageci Marrero Rodr. 87
Limonium besserianum (Schult. ex Rchb.) Kuntze.........42
Limonium bianorii (Sennen & Pau) Erben 51
Limonium bicolor (Bunge) Kuntze 82
Limonium biflorum (Pignatti) Pignatti 51
Limonium billardierei (Girard) Kuntze 51
Limonium binervosum (G.E.Sm.) C.E.Salmon 51
Limonium binervosum subsp. *anglicum* Ingr.................49
Limonium binervosum subsp. *binervosum*....................51
Limonium binervosum subsp. *cantianum* Ingr. 52
Limonium binervosum subsp. *dodartii* (Girard) P.Fourn. 57
Limonium binervosum subsp. *lychnidifolium* P.Fourn. ..50
Limonium binervosum subsp. *multiflorum* Pignatti 63
Limonium binervosum subsp. *mutatum* Ingr. 64
Limonium binervosum subsp. *occidentale* (J.Lloyd) P.Fourn. 51
Limonium binervosum subsp. *sarniense* Ingr. 68
Limonium binervosum subsp. *saxonicum* Ingr. 68
Limonium binervosum var. *aurigniense* Ingr. 50
Limonium binervosum var. *humilis* C.E.Salmon.............51
Limonium binervosum var. *sarniense* Ingr.....................68
Limonium binervosum var. *sercquense* Ingr..................69
Limonium bocconei (Lojac.) Litard................................51
Limonium boirae L.Llorens & Tébar 51
Limonium boitardii Maire 51
Limonium bollei (Webb ex Wangerin) Erben 51
Limonium bolosii Gil & L.Llorens..................................51
Limonium bonafei Erben..51
Limonium bonduellei (T.Lestib.) Kuntze 89
Limonium bonduellei f. *gigantifolia* Corti......................89
Limonium bonifaciense Arrigoni & Diana......................51
Limonium bonnetii (Sennen) Erben...............................51
Limonium bosanum Arrigoni & Diana 52
Limonium botschantzevii (Lincz.) M.Malekm., Akhani & Borsch 85
Limonium bourgeaui (Webb ex Boiss.) Kuntze..............87
Limonium brasiliense (Boiss.) Kuntze 76
Limonium brasiliense (Boiss.) Small 76
Limonium brasiliense Small..76
Limonium brasiliense var. *brasiliense*............................76
Limonium brasiliense var. *patagonicum* (Speg.) Burkart 76
Limonium brasiliensis A.Heller 76
Limonium brassicifolium (Webb & Berthel.) Kuntze 87
Limonium brassicifolium subsp. *macropterum* (Webb & Berthel.) G.Kunkel 88
Limonium braunii (Bolle) A.Chev..................................74
Limonium brevipetiolatum R.Artelari & Erben 76
Limonium britannicum Ingr..52
Limonium britannicum subsp. *celticum* Ingr. 53
Limonium britannicum subsp. *coombense* Ingr.............53
Limonium britannicum subsp. *transcanalis* Ingr. 71
Limonium britannicum var. *celticum* Ingr......................53
Limonium britannicum var. *coombense* Ingr.................53

Limonium britannicum var. *grandicaule* Ingr. 59
Limonium britannicum var. *kelseyanum* Ingr. 61
Limonium britannicum var. *pharense* Ingr. 65
Limonium brizoides Brullo ex Erben, Del Guacchio & P.Caputo 97
Limonium brunneri (Webb) Kuntze 74
Limonium brusnicense (Trinajstić) Bogdanović & Brullo 52
Limonium brutium Brullo 52
Limonium bulgaricum Ančev.. 76
Limonium bungei (Claus) Gamajun. 76
Limonium busianum Bogdanović & Brullo 52
Limonium byzacium Brullo 52
Limonium cabulicum (Boiss.) Kuntze 40
Limonium caesium (Girard) Kuntze 46
Limonium calabrum Brullo 52
Limonium calaminare Pignatti 53
Limonium calanchicola Erben 52
Limonium calcarae (Tod. ex Janka) Pignatti 52
Limonium calcicola P.D.Sell 52
Limonium californicum (Boiss.) A.Heller 76
Limonium californicum Small 76
Limonium californicum var. *californicum* (Boiss.) A.Heller 76
Limonium californicum var. *mexicanum* (S.F.Blake) Munz 76
Limonium callianthum (T.X.Peng) Kamelin 85
Limonium callicomum (C.A.Mey.) Kuntze 42
Limonium calliopsium Alf.Mayer 52
Limonium cambrense (Ingr.) P.D.Sell 52
Limonium camposanum Erben 52
Limonium cancellatum (Bernh. ex Bertol.) Kuntze 52
Limonium cantianum (Ingr.) P.D.Sell 52
Limonium capense (L.Bolus) L.Bolus 73
Limonium capitis-eliae Erben 52
Limonium capitis-marci Arrigoni & Diana 52
Limonium caprariae Rizzotto 52
Limonium caprariense (Font Quer & Marcos) Pignatti . 52
Limonium caprariense subsp. *caprariense* 52
Limonium caprariense subsp. *multiflorum* Pignatti 69
Limonium caralitanum Erben 68
Limonium carinense (Chiov.) Verdc. & Hemming ex Cufod. 85
Limonium carisae Erben 52
Limonium carminis B.Díez & Erben 52
Limonium carnosum (Boiss.) Kuntze 75
Limonium carolinianum (Walter) Britton 76, 77
Limonium carolinianum var. *angustatum* (A.Gray) S.F.Blake 77
Limonium carolinianum var. *carolinianum* (Walter) Britton 77
Limonium carolinianum var. *compactum* Shinners 77
Limonium carolinianum var. *nashii* (Small) B.Boivin 77
Limonium carolinianum var. *obtusilobum* (S.F.Blake) H.E.Ahles 77
Limonium carolinianum var. *trichogonum* (S.F.Blake) B.Boivin 77
Limonium carpathum (Rech.f.) Rech.f. 52
Limonium carpetanicum Erben 52
Limonium carregadorense Erben 52
Limonium carthaginense (Rouy) C.E.Hubb. & Sandwith 53
Limonium carvalhoi Rosselló, L.Sáez & Carvalho, A.C. .. 53
Limonium caspium (Willd.) Gams 80
Limonium caspium (Willd.) P.Fourn. 80
Limonium caspium var. *patens* (Fisch. ex Boiss.) A.V.Grebenjuk 81
Limonium castellonense Socorro & S.Tárrega 56
Limonium catalaunicum (Willk. & Costa) Pignatti 53
Limonium catalaunicum subsp. *catalaunicum* 53
Limonium catalaunicum subsp. *procerum* (Willk.) Pignatti 60
Limonium catalaunicum subsp. *viciosoi* (Pau) Pignatti . 72
Limonium catanense (Tineo ex Lojac.) Brullo 53
Limonium catanzaroi Brullo 53
Limonium cavanillesii Erben 57
Limonium cazzae Bogdanović & Brullo 53
Limonium cedrorum Domina & Raimondo 53
Limonium celticum (Ingr.) P.D.Sell 53
Limonium cephalonicum R.Artelari 53
Limonium cercinense Brullo 53
Limonium chazaliei (H.Boissieu) Maire 74
Limonium chersonesum Erben & Brullo 53
Limonium chodshamumynense Lincz. & Czukav. 85
Limonium chrisianum Brullo & Guarino 60
Limonium chrysocephalum (Regel) Lincz. 82
Limonium chrysocomum (Kar. & Kir.) Kuntze 82
Limonium chrysocomum subsp. *chrysocomum* (Kar. & Kir.) Kuntze 82
Limonium chrysocomum subsp. *semenovii* (Herder) Kamelin 83
Limonium chrysocomum var. *chrysocephalum* (Regel) T.H.Peng 82
Limonium chrysocomum var. *pubescens* Lincz. 82
Limonium chrysocomum var. *sedoides* (Regel) T.H.Peng 82
Limonium chrysocomum var. *semenovii* (Herder) T.H.Peng 83
Limonium chrysopotamicum Maire 74
Limonium cimmericum (Lipsky) Klokov 81
Limonium circaei Pignatti 53
Limonium clupeanum Brullo 53
Limonium coelicolor Rech.f. 40
Limonium cofrentanum Erben 53
Limonium collinum (Griseb.) F.T.Hubb. ex L.H.Bailey 43
Limonium commune Gray 80
Limonium commune subsp. *californicum* (Boiss.) A.E.Murray 76
Limonium commune var. *californicum* (Boiss.) Greene . 76
Limonium commune var. *mexicanum* (S.F.Blake) Jeps. . 76
Limonium commune var. *minus* Gray 80
Limonium commune var. *obtusum* Gray 80
Limonium commune var. *serotinum* Gray 80
Limonium comosum Erben 53
Limonium compactum Erben & Brullo 77
Limonium companyonis (Gren. & Billot) Kuntze 58
Limonium confertum Brullo 53
Limonium confusum (Gren. & Godr.) Fourr. 53
Limonium confusum (Gren. & Godr.) Kuntze 53
Limonium confusum subsp. *densissimum* Pignatti 56
Limonium confusum subsp. *duriusculum* (Girard) P.Fourn. 57
Limonium confusum subsp. *psilocladum* (Boiss.) P.Fourn. 67
Limonium confusum subsp. *raddianum* (Boiss.) P.Fourn. 67
Limonium congestum (Ledeb.) Kuntze 82
Limonium connivens Erben 53

Limonium contortirameum (Mabille) Erben 53
Limonium contractum Erben & Brullo 53
Limonium coombense (Ingr.) P.D.Sell...........................53
Limonium cophanense C.Brullo, Brullo, Cambria, del Galdo & Ilardi 54
Limonium coralliforme Alf.Mayer 71
Limonium coralloides (Tausch) Lincz.............................81
Limonium corculum (Webb & Berthel.) Kuntze 74
Limonium cordatum (L.) Mill.......................................54
Limonium cordovillense Stübing & Cirujano 54
Limonium coriarium H.Arnaud.....................................77
Limonium corinthiacum (Boiss. & Heldr.) Kuntze..........54
Limonium cornarianum Kypr. & R.Artelari...................54
Limonium cornubiense (Ingr.) P.D.Sell 54
Limonium cornusianum Arrigoni & Diana 54
Limonium coronense R.Artelari....................................54
Limonium corsicum Erben...54
Limonium corymbulosum (Boiss.) Kuntze 54
Limonium cossonianum (Nyman) Kuntze......................54
Limonium cossonianum f. *tabernense* (Erben) G.Kunkel 70
Limonium costae (Willk.) Pignatti 54
Limonium cosyrense (Guss.) Kuntze.............................54
Limonium cosyrense var. *maior* (Boiss.) Rech.f.............55
Limonium crateriforme Erben & Brullo.........................55
Limonium cretaceum Cherkasova................................81
Limonium creticum R.Artelari 55
Limonium crispum (Pers.) H.Arnaud 74
Limonium croaticum Bogdanović & Brullo...................55
Limonium cumanum (Ten.) Kuntze 55
Limonium cumanum var. *cumanum* (Ten.) Kuntze 55
Limonium cumanum var. *glabrescens* (Lacaita) Vallariello, Iamonico & Del Guacchio........................55
Limonium cunicularium Arrigoni & Diana 55
Limonium cuspidatum (Delort.) Erben..........................55
Limonium cylindrifolium (Forssk.) Verdc. ex Cufod.......85
Limonium cymuliferum (Boiss.) Sauvage & Vindt 55
Limonium cymuliferum subsp. *cymuliferum* 55
Limonium cymuliferum subsp. *mauritii* Sennen............55
Limonium cymuliferum var. *corymbulosum* Pignatti.....54
Limonium cymuliferum var. *cymuliferum* (Boiss.) Sauvage & Vindt..55
Limonium cymuliferum var. *sebkarum* (Pomel) Sauvage & Vindt 55
Limonium cymuliferum var. *uniflorum* Pignatti.............55
Limonium cyprium (Meikle) Hand................................55
Limonium cyrenaicum (Rouy) Brullo 55
Limonium cyrtostachyum (Girard) Brullo......................55
Limonium cythereum R.Artelari & Georgiou.................55
Limonium czurjukiense (Klokov) Lavrenko 79
Limonium dagmarae Mucina..73
Limonium dagmariae Mucina.......................................73
Limonium dalmaticum (C.Presl) Kuntze........................42
Limonium damboldtianum Phitos & R.Artelari 55
Limonium danubiale Klokov...80
Limonium daveaui Erben 56
Limonium davisii Doğan 86
Limonium decipiens (Ledeb.) Kuntze 81
Limonium decumbens (Boiss.) Kuntze...........................56
Limonium delicatulum (Girard) Kuntze 56
Limonium delicatulum subsp. *afrum* Pignatti 49
Limonium delicatulum subsp. *angustebracteatum* (Erben) Rivas Mart. & M.J.Costa 49
Limonium delicatulum subsp. *angustibracteatum* (Erben) Rivas Mart. & M.J.Costa...49
Limonium delicatulum subsp. *biflorum* (Pignatti) O.Bolòs, Vigo, Masalles & Ninot..51
Limonium delicatulum subsp. *delicatulum*....................56
Limonium delicatulum subsp. *eu-delicatulum* Maire 56
Limonium delicatulum subsp. *formenterae* (L.Llorens) O.Bolòs, Vigo, Masalles & Ninot...............................56
Limonium delicatulum subsp. *gallicum* Pignatti............59
Limonium delicatulum subsp. *latebracteatum* (Erben) Castrov. & Cirujano 61
Limonium delicatulum subsp. *latibracteatum* (Erben) Castrov. & Cirujano 61
Limonium delicatulum subsp. *migjornense* (L.Llorens) O.Bolòs, Vigo, Masalles & Ninot...............................63
Limonium delicatulum subsp. *orientale* Pignatti...........56
Limonium delicatulum subsp. *retusum* (L.Llorens) O.Bolòs, Vigo, Masalles & Ninot...............................68
Limonium delicatulum subsp. *santapolense* (Erben) O.Bolòs, Vigo, Masalles & Ninot...............................68
Limonium delicatulum subsp. *tournefortii* (Girard) Pignatti..71
Limonium delicatulum subsp. *valentinum* Pignatti 49
Limonium delicatulum var. *leptostachys* (Pomel) Maire 66
Limonium delicatulum var. *typicum* Maire....................56
Limonium dendroides Svent...75
Limonium densiflorum (Guss.) Kuntze..........................56
Limonium densiflorum Maire & Petitm.........................59
Limonium densissimum (Pignatti) Pignatti 56
Limonium depauperatum (Boiss.) R.A.Dyer 51
Limonium desertorum (Trautv.) Kuntze 43
Limonium devoniense (Ingr.) P.D.Sell 56
Limonium dianiae (Pau) A.Barber, M.B.Crespo & Lledó60
Limonium dianium Pignatti 56
Limonium dichotomum (Cav.) Kuntze 56
Limonium dichroanthum (Rupr.) Ikonn.-Gal. ex Lincz. ..82
Limonium dictyocladum (Boiss.) Kuntze.......................70
Limonium dictyophorum (Tausch) Degen......................56
Limonium didimense Doğan & Akaydın.........................56
Limonium diegoi Sennen...70
Limonium dielsianum (Wangerin) Kamelin 82
Limonium diffusum (Pourr.) Kuntze 90
Limonium diomedeum Brullo 56
Limonium dissitiflorum (Boiss.) Kerguélen 56
Limonium distichum Wilmot-Dear 85
Limonium divaricatum (Rouy) Brullo............................56
Limonium dodartiforme Ingr..57
Limonium dodartii (Girard) Kuntze 57
Limonium dodartii subsp. *lusitanicum* (Daveau) Franco63
Limonium doerfleri (Halácsy) Rech.f. 57
Limonium dolihiense Erben & Brullo 57
Limonium donegalense (Ingr.) P.D.Sell..........................57
Limonium donetzicum Klokov 79
Limonium doriae (Sommier) Pignatti 57
Limonium dragonericum Erben....................................57
Limonium dregeanum (C.Presl) Kuntze.........................57
Limonium drepanostachyum Ikonn.-Gal. 85
Limonium drepanostachyum subsp. *callianthum* T.X.Peng 85
Limonium dubium (Andrews ex Guss.) Litard................57
Limonium dubium Gamajun. ex Klokov.........................79
Limonium dubyi (Gren. & Godr.) Kuntze 81

Limonium dufourii (Girard) Kuntze 57, 71
Limonium duriaei (Girard) Kuntze 57
Limonium durieui (Girard) Kuntze 57
Limonium durisculum subsp. *cavanillesii* (Erben) O.Bolòs, Vigo, Masalles & Ninot 57
Limonium duriusculum (Girard) Fourr. 57
Limonium duriusculum (Girard) Kuntze 57
Limonium duriusculum subsp. *bianorii* (Sennen & Pau) Malag. 51
Limonium duriusculum subsp. *cavanillesii* (Erben) O.Bolòs, Vigo, Masalles & Ninot 57
Limonium duriusculum subsp. *companyonis* (Gren. & Billot) O.Bolòs, Vigo, Masalles & Ninot 57
Limonium duriusculum subsp. *duriusculum* 58
Limonium duriusculum subsp. *thiniense* (Erben) O.Bolòs, Vigo, Masalles & Ninot 71
Limonium duriusculum var. *robustior* Pignatti 51
Limonium dyeri Lincz. 58
Limonium ebusitanum (Font Quer) Font Quer 58
Limonium echinus (L.) Chaz. 16
Limonium echioides (L.) Mill. 47, 48
Limonium echioides subsp. *eu-echioides* Maire 48
Limonium echioides subsp. *exaristatum* (Murb.) Maire 48
Limonium echoideum (L.) Mill. 48
Limonium effusum (Boiss.) Kuntze 77
Limonium ejulabilis Rosselló, Mus & J.X.Soler 58
Limonium elaphonisicum Alf.Mayer 58
Limonium elatum (Fisch. ex Spreng.) Kuntze 43
Limonium elfahsianum Brullo & Giusso 58
Limonium emarginatum (Willd.) Kuntze 58
Limonium emporitanum Fern.Casas & Molero 68
Limonium endlichianum (Wangerin) S.F.Blake 77
Limonium equisetinum (Boiss.) R.A.Dyer 58
Limonium equisetinum var. *depauperatum* (Boiss.) Steenis 51
Limonium erectum Erben 58
Limonium erythrorrhizum Ikonn.-Gal. ex Lincz. 82
Limonium erythrorrhizum var. *eriocalyx* Lincz. 82
Limonium estevei Fern.Casas 58
Limonium etruscum Arrigoni & Rizzotto 58
Limonium exaristatum (Murb.) P.Fourn. 48
Limonium eximium (Schrenk ex Fisch. & C.A.Mey.) H.Arnaud 43
Limonium eximium (Schrenk) Kuntze 43
Limonium eximum var. *album* F.T.Hubb. 98
Limonium eximum var. *superbum* F.T.Hubb. 98
Limonium failachicum Erben & Mucina 75
Limonium fajzievii Zakirov ex Lincz. 86
Limonium fallax (Coss. ex Wangerin) Maire 74
Limonium fallax var. *trachycladum* (Maire & Wilczek) Maire 75
Limonium faustii Sennen & Mauricio 54
Limonium ferganense Ikonn.-Gal. 86
Limonium fergusoniae L.Bolus 73
Limonium ferulaceum (L.) Chaz. 90
Limonium ferulaceum (L.) Kuntze 90
Limonium fesianum Erben 47
Limonium fischeri (Trautv.) Lincz. 82
Limonium flagellare (Lojac.) Brullo 58
Limonium flexuosum (L.) Chaz. 81, 82
Limonium flexuosum (L.) Kuntze 82
Limonium flexuosum Sennen 53
Limonium florentinum Arrigoni & Diana 58
Limonium fontqueri (Pau) L.Llorens ex Greuter 58
Limonium formenterae L.Llorens 56
Limonium formosum Bartolo, Brullo & Giusso 58
Limonium fortunei (Lindl.) H.Arnaud 84
Limonium fradinianum (Pomel) Erben 58
Limonium fragile Erben & Brullo 58
Limonium franchetii (Debeaux) Kuntze 83
Limonium frederici (Barbey) Rech.f. 58
Limonium frutescens (Lem.) Erben, A.Santos & Reyes-Bet. 87
Limonium fruticans (Webb ex Boiss.) Kuntze 87
Limonium fruticosum Mill. 85
Limonium furfuraceum (Lag.) Kuntze 58
Limonium furfuraceum subsp. *lucentinum* (Pignatti & Freitag) O.Bolòs & Vigo 62
Limonium furnarii Brullo 59
Limonium gabrieli (Bornm.) Rech.f. 75
Limonium galilaeum Domina, Danin & Raimondo 59
Limonium gallicum (Pignatti) Domina 59
Limonium gallurense Arrigoni & Diana 59
Limonium gavilae Sennen & Mauricio 54
Limonium gerberi Soldano 77
Limonium geronense Erben 59
Limonium giberti (Sennen) Sennen 59
Limonium gibertii (Sennen) Sennen 59
Limonium ginae P.D.Sell 59
Limonium ginzbergeri Bogdanović & Brullo 59
Limonium girardianum (Guss.) Fourr. 59
Limonium girardianum (Guss.) Kuntze 59
Limonium girardianum subsp. *balearicum* (Pignatti) O.Bolòs, Vigo, Masalles & Ninot 50
Limonium girardianum subsp. *grosii* (L.Llorens) O.Bolòs, Vigo, Masalles & Ninot 59
Limonium girardianum subsp. *majoricum* (Pignatti) O.Bolòs, Vigo, Masalles & Ninot 62
Limonium girardianum subsp. *willdenowii* P.Fourn. 50
Limonium globulariifolium (Desf.) Kuntze 67
Limonium globulariifolium subsp. *globulariifolium* (Desf.) Kuntze 67
Limonium globulariifolium subsp. *lusitanicum* Pignatti 56
Limonium globulariifolium subsp. *provinciale* Pignatti . 67
Limonium globulariifolium subsp. *tommasinii* Pignatti . 56
Limonium globuliferum (Boiss. & Heldr. ex Boiss.) Kuntze 86
Limonium glomeratum (Tausch) Erben 59
Limonium gmelini (Willd.) Kuntze 77
Limonium gmelini f. *laxiflorum* C.E.Salmon 78
Limonium gmelini var. *meyeri* C.E.Salmon 78
Limonium gmelinii (Willd.) Kuntze 77
Limonium gmelinii subsp. *hungaricum* (Klokov) Soó 78
Limonium gmelinii subsp. *hypanicum* (Klokov) Sóo 78
Limonium gmelinii var. *hypanicum* Pawł. 78
Limonium gobicum Ikonn.-Gal. 83
Limonium gomezi-jordanae Sennen & Mauricio 54
Limonium gorgonae Pignatti 59
Limonium gougetianum (Girard) Kuntze 59
Limonium gougetianum subsp. *balearicum* Pignatti 50
Limonium gougetianum subsp. *multiceps* (Pomel) Quézel & Santa ex Greuter & Raus 63
Limonium gougetianum var. *multiceps* (Pomel) Maire . 63
Limonium grabusae Erben & Brullo 59
Limonium graeca Post 63
Limonium graecum (Poir.) Kuntze 59

Limonium graecum (Poir.) Rech.f. 59
Limonium graecum Mouterde 63
Limonium graecum subsp. *ammophilon* Papatsou & Phitos 49
Limonium graecum subsp. *divaricatum* (Pignatti) Pignatti 57
Limonium graecum subsp. *graecum* 59
Limonium graecum var. *hyssopifolium* (Girard) Bokhari 68
Limonium graecum var. *palmare* (Sm.) Rech.f. 64
Limonium graecum var. *stenotatum* Rech.f. 69
Limonium graminifolium (Aitch.) Kuntze 43
Limonium grandicaule (Ingr.) P.D.Sell 59
Limonium greuteri Erben 59
Limonium griffithii (Aitch. & Hemsl.) Kuntze 41
Limonium grosii L.Llorens 59
Limonium grubovii Lincz. 83
Limonium guaicuru (Molina) Kuntze 78
Limonium gueneri Doğan, H.Duman & Akaydın 59
Limonium guigliae Raimondo & Domina 85
Limonium gummiferum (Durieu ex Boiss. & Reut.) Kuntze 59
Limonium gummiferum subsp. *battandieri* Sauvage & Vindt 51
Limonium gummiferum subsp. *cymuliferum* 51
Limonium gummiferum subsp. *eu-gummiferum* Maire 59
Limonium gummiferum subsp. *gummiferum* 59
Limonium gummiferum subsp. *sebkarum* (Pomel) Maire 69
Limonium gummiferum var. *corymbulosum* (Coss.) Maire 54
Limonium gummiferum var. *muticum* Maire & Sennen 60
Limonium gummiferum var. *typicum* Maire 60
Limonium gussonei (Tineo ex Lojac.) Giardina & Raimondo 71
Limonium gymnesicum Erben 60
Limonium haitense S.F.Blake 74
Limonium haitiense S.F.Blake 74
Limonium halophilum Pignatti ex Brullo 60
Limonium helenae Erben & Brullo 60
Limonium heraionense Erben & Brullo 60
Limonium herculis Pignatti 63
Limonium hermaeum (Pignatti) Pignatti 60
Limonium heterobracteatum Erben 89
Limonium heterospicatum Erben 60
Limonium hibericum Erben 60
Limonium hibernicum (Ingr.) P.D.Sell 60
Limonium hierapetrae Rech.f. 60
Limonium himariense F.K.Mey. 60
Limonium hipponense Brullo 60
Limonium hirsuticalyx Pignatti 78
Limonium hoeltzeri (Regel) Ikonn.-Gal. 83
Limonium humboldtii (Bolle) Kuntze 74
Limonium humile Mill. 78
Limonium hungaricum Klokov 78
Limonium hyblaeum Brullo 60
Limonium hypanicum Klokov 78
Limonium hyssopifolium (Girard) Rech.f. 68
Limonium iconicum (Boiss. & Heldr.) Kuntze 81
Limonium iconium (Boiss. & Heldr.) Kuntze 81
Limonium ifniense Caball. 74
Limonium ifniensis Caball. 74
Limonium ikaricum Erben & Brullo 60
Limonium ikonnikovii-galitzkyi A.V.Grebenjuk 97
Limonium ilerdense Erben 71
Limonium ilergabonum López-Alvarado, Cobacho, Arán & L.Sáez 60
Limonium iljinii Sobolevsk. 82
Limonium ilvae Pignatti 60
Limonium imbricatum (Webb ex Girard) F.T.Hubb. ex L.H.Bailey 87
Limonium imbricatum (Webb ex Girard) H.Arnaud 87
Limonium inarimense (Guss.) Pignatti 60
Limonium inarimense subsp. *ebusitanum* (Font Quer) Pignatti 58
Limonium incanum (L.) Chaz. 43
Limonium incanum (L.) Kuntze 43
Limonium incertum H.Arnaud 88
Limonium inexpectans L.Sáez & Rosselló 60
Limonium insigne (Coss.) Kuntze 46
Limonium insigne subsp. *carthaginensis* Pignatti 46
Limonium insigne subsp. *carthaginiense* Pignatti 46
Limonium insigne subsp. *insigne* 46
Limonium insigne var. *insigne* 46
Limonium insigne var. *paui* Pignatti 46
Limonium insigne var. *rossmaessleri* (Willk.) Pignatti 47
Limonium insulare (Bég. & Landi) Arrigoni & Diana 60
Limonium intercedens P.D.Sell 60
Limonium intermedium (Guss.) Brullo 60
Limonium intricatum Brullo 60
Limonium ionicum Brullo 60
Limonium iranicum (Bornm.) Lincz. 75
Limonium irtaense Ferrer-Gallego, P. P., A.Navarro, P.Pérez, R.Roselló, Rosselló, M.Rosato & E.Laguna 61
Limonium isidorum Erben & Brullo 61
Limonium issaeum Bogdanović & Brullo 61
Limonium istriacum Bogdanović & Brullo 61
Limonium ithacense R.Artelari 61
Limonium jankae (Lojac.) Giardina & Raimondo 61
Limonium japonicum (Siebold & Zucc.) Kuntze 84
Limonium japygicum (E.Groves) Pignatti 61
Limonium japygicum (E.Groves) Pignatti, Galasso & Nicolella 61
Limonium jarmolenkoi (Lincz.) M.Malekm., Akhani & Borsch 86
Limonium johannis Pignatti 55
Limonium jovibarba (Webb ex Boiss.) Kuntze 75
Limonium kairouanum Brullo 61
Limonium kardamylii R.Artelari & Kamari 61
Limonium karpasiticum Kefalas, Erben, Christodoulou & Hand 61
Limonium kaschgaricum (Rupr.) Ikonn.-Gal. 83
Limonium kaufmannianum (Regel) Kuntze 43
Limonium kelseyanum (Ingr.) P.D.Sell 61
Limonium kerryense (Ingr.) P.D.Sell 61
Limonium kimmericum (Lipsky) Klokov 81
Limonium kirikosicum Erben & Brullo 61
Limonium klementzii Ikonn.-Gal. 83
Limonium kobstanicum Tzvelev 75
Limonium komarovii Ikonn.-Gal. 86
Limonium komarovii Ikonn.-Gal. ex Lincz. & Czukav. 86
Limonium korakonisicum Valli 61
Limonium korbousense Brullo 61
Limonium kossmatii (R.Wagner & Vierh.) Verdc. & Hemming ex Cufod. 85
Limonium kraussianum (Buchinger ex Boiss.) Kuntze 61

Limonium ksamilum Bogdanović, Shuka, Giusso & Brullo 61
Limonium kurgantjubense (Lincz.) M.Malekm., Akhani & Borsch 86
Limonium lacertosum Brullo 61
Limonium lacinium Arrigoni 61
Limonium lacostei (Danguy) Kamelin 83
Limonium ladikanum Erben & Brullo 67
Limonium laetum (Nyman) Pignatti 61
Limonium laetum subsp. *insulare* (Bég. & Landi) Atzei & V.Picci 60
Limonium lagostanum Bogdanović & Brullo 61
Limonium lajaconi Brullo 62
Limonium lambinonii Erben 61
Limonium lanceolatum (Hoffmanns. & Link) Franco 61
Limonium lanfrancoi Agius, M.E.Galea, Cambria, del Galdo & Brullo 61
Limonium latebracteatum Erben 61
Limonium latibracteatum Erben 61
Limonium latifolium (Sm.) Kuntze 77
Limonium latifolium Moench 44
Limonium latifolium var. *album* F.T.Hubb. 98
Limonium lausianum Pignatti 61
Limonium laxiusculum Franco 61
Limonium lefroyi (Hemsl.) Britton 77
Limonium legrandii (Gaut. & Timb.-Lagr.) Erben 61
Limonium leonardi-llorensii L.Sáez, Á.C.Carvalho & Rosselló 62
Limonium leprosorum Bogdanović & Brullo 62
Limonium leptolobum (Regel) Kuntze 83
Limonium leptophyllum (Schrenk) Kuntze 75
Limonium leptostachyus (Boiss.) Kuntze 91
Limonium lessingianum Lincz. 75
Limonium letourneuxii (Coss. ex Batt.) A.Pons & Quézel 62
Limonium letourneuxii (Coss. ex Batt.) Greuter & Raus 62
Limonium liberianum Bogdanović & Brullo 62
Limonium liburnicum Bogdanović & Brullo 62
Limonium lilacinum (Boiss. & Balansa) Wagenitz 86
Limonium lilacinum var. *laxiflorum* Doğan & Akaydın .. 86
Limonium lilacinum var. *lilacinum* 86
Limonium lilybaeum Brullo 62
Limonium limbatum Small 78
Limonium limbatum var. *glabrescens* Correll 78
Limonium limonium (L.) A.Lyons 79
Limonium limonium Druce 80
Limonium linguum (Pomel) Pons, Quézel, Quezel & Santa 55
Limonium linifolium (L.f.) Chaz. 62
Limonium linifolium (L.f.) Kuntze 62
Limonium linifolium var. *linifolium* (L.f.) Chaz. 62
Limonium linifolium var. *maritimum* (Eckl. & Zeyh. ex Boiss.) R.A.Dyer 62
Limonium lobatum (L.f.) Chaz. 89
Limonium lobatum (L.f.) Kuntze 89
Limonium lobetanicum Erben 62
Limonium lobinii N.Kilian & T.Leyens 75
Limonium loganicum Ingr. 62
Limonium lojaconoi Brullo 62
Limonium longibracteatum Erben 62
Limonium longifolium (Thunb.) R.A.Dyer 73
Limonium longispicatum Erben 48
Limonium lopadusanum Brullo 62
Limonium lovricii Bogdanović & Brullo 62
Limonium lowei R.Jardim, M.Seq., Capelo, J.C.Costa & Rivas Mart. 62
Limonium lychnidifolium (Girard) Kuntze 50
Limonium lychnidifolium var. *corymbosum* (Boiss.) C.E.Salmon 64
Limonium macrophyllum (Willd. ex Spreng.) H.Arnaud 87
Limonium macrophyllum (Willd. ex Spreng.) Kuntze 87
Limonium macropterum (Webb & Berthel.) Kuntze 88
Limonium macrorhabdon (Boiss.) Kuntze 41
Limonium macrorrhizum (Ledeb.) Kuntze 81
Limonium magallufianum L.Llorens 62
Limonium majoricum Pignatti 62
Limonium majus (Boiss.) Erben 62
Limonium malacitanum B.Díez 62
Limonium malfatanicum Erben 62
Limonium mansanetianum M.B.Crespo & Lledó 62
Limonium mareoticum El Garf ex Hadidi & Fayed 79
Limonium marisolii L.Llorens 62
Limonium maritimum Caperta, Cortinhas, A.P.Paes, Guara, Esp.Santo & Erben 80
Limonium marmarisense Doğan & Akaydın 78
Limonium maroccanum (Batt. & Trab.) Domina 48
Limonium mateoi Erben & Arán 62
Limonium mauritanicum Hutch. & Dalziel 74
Limonium maurocordatae (Schweinf. & Volkens) Cufod. 85
Limonium mazarae Pignatti ex Brullo 62
Limonium meandrinum Erben & Brullo 62
Limonium medium (Ingr.) P.D.Sell 62
Limonium melancholicum Brullo, Marcenò & S.Romano 62
Limonium melitense Brullo 62
Limonium melium (Nyman) Pignatti 72
Limonium membranaceum (Czern. ex Trautv.) Klokov . 76
Limonium membranaceum R.A.Dyer 58
Limonium menigense Brullo 63
Limonium merxmuelleri Erben 63
Limonium merxmuelleri subsp. *merxmuelleri* 63
Limonium merxmuelleri subsp. *oristanum* (Alf.Mayer) Arrigoni 64
Limonium merxmuelleri subsp. *sulcitanum* (Arrigoni) Arrigoni 70
Limonium merxmuelleri subsp. *tigulianum* (Arrigoni & Diana) Arrigoni 71
Limonium messeniacum R.Artelari & Kamari 63
Limonium mexicanum S.F.Blake 76
Limonium meyeri (Boiss.) Kuntze 78
Limonium michelsonii Lincz. 83
Limonium microcycladicum Erben & Brullo 63
Limonium migjornense L.Llorens 63
Limonium Mill. 2, 45
Limonium milleri Ghaz. & J.R.Edm. 85
Limonium milovicii Bogdanović & Brullo 63
Limonium minoicum Erben & Brullo 60
Limonium minoricense Erben 63
Limonium minus (Boiss.) Erben 63
Limonium minutiflorum (Guss.) Kuntze 63
Limonium minutiflorum subsp. *balearicum* Pignatti 50
Limonium minutum (L.) Chaz. 63
Limonium minutum (L.) Fourr. 63
Limonium minutum (L.) Kuntze 63
Limonium minutum f. *puberulum* C.E.Salmon 63

Limonium minutum subsp. *acutifolium* (Rchb.) P.Fourn. 48
Limonium minutum subsp. *aragonense* De Litard. 50
Limonium minutum subsp. *caprariense* Font Quer & Marcos 52
Limonium minutum subsp. *escarrei* (L.Llorens & Tébar) O.Bolòs, Vigo, Masalles & Ninot 58
Limonium minutum subsp. *minutum* 63
Limonium minutum subsp. *pericotii* O.Bolòs & Vigo 65
Limonium minutum subsp. *pseudominutum* (Erben) O.Bolòs & Vigo 66
Limonium minutum subsp. *revolutum* (Erben) O.Bolòs & Vigo 68
Limonium minutum subsp. *tremolsii* (Rouy) P.Fourn. ... 71
Limonium minutum var. *dissitiflorum* (Boiss.) C.E.Salmon 56
Limonium minutum var. *medense* O.Bolòs & Vigo 63
Limonium minutum var. *microphyllum* C.E.Salmon 66
Limonium minutum var. *pseudebusitanum* (Erben) O.Bolòs & Vigo 66
Limonium molesii Sennen & Mauricio 54
Limonium monolithicum Erben & Brullo 63
Limonium monopetalum (L.) Hill 45
Limonium montis-christi Rizzotto 63
Limonium morisianum Arrigoni 63
Limonium motianum Brullo ex Erben, Del Guacchio & P.Caputo 56
Limonium mouretii (Pit.) Maire 89
Limonium mouretii var. *coloratum* Maire 89
Limonium mouretii var. *pubicalyx* (Stearn) Emberger, L. & Maire 89
Limonium mouterdei Domina, Erben & Raimondo 63
Limonium mucronatum (L.f.) Chaz. 73, 74
Limonium mucronatum (L.f.) Kuntze 74
Limonium mucronulatum (H.Lindb.) Greuter & Raus.... 63
Limonium multiceps (Pomel) Erben 63
Limonium multiflorum Erben 63
Limonium multiforme (Martelli) Pignatti 63
Limonium multifurcatum Erben 64
Limonium muradense Erben 64
Limonium mutabile (Ingr.) P.D.Sell 64
Limonium mutatum (Ingr.) P.D.Sell 64
Limonium myosuroides (Regel) Kuntze 91
Limonium myrianthum (Schrenk ex Fisch. & C.A.Mey.) Kuntze 81
Limonium namaquanum L.Bolus 73
Limonium naniforme P.D.Sell 64
Limonium narbonense Mill. 78
Limonium narynense Lincz. 86
Limonium nashii f. *albiflorum* House 77
Limonium nashii Small 77
Limonium nashii var. *angustatum* (A.Gray) H.E.Ahles .. 77
Limonium nashii var. *trichogonum* S.F.Blake 77
Limonium neapolense Brullo 64
Limonium neocastellonense Fern.Casas 56
Limonium neoscoparium Klokov 78
Limonium nogalense (Chiov.) Verdc. & Hemming ex Cufod. 85
Limonium normannicum Ingr. 64
Limonium nudum (Boiss. & Buhse) Kuntze 83
Limonium nydeggeri Erben 50
Limonium nymphaeum Erben 64
Limonium obesifolium P.D.Sell 64
Limonium oblanceolatum Brullo 64
Limonium oblongifolium (Kotov) Loscot & Trautv. 49
Limonium obovatum (Ledeb.) Kuntze 78
Limonium obtusifolium (Rouy) Erben 64
Limonium obtusilobum S.F.Blake 77
Limonium occidentale (J.Lloyd) Kuntze 51
Limonium occidentale var. *procerum* C.E.Salmon 66
Limonium ochranthum (Kar. & Kir.) Kuntze 44
Limonium ocymifolium (Poir.) Kuntze 64
Limonium ocymifolium subsp. *bellidifolium* (Sm.) Meikle 50
Limonium ocymifolium var. *bellidifolium* (Sm.) Rech.f. . 50
Limonium oleifolium Mill. 97
Limonium oleifolium subsp. *algusae* Brullo 49
Limonium oleifolium subsp. *dictyocladum* 70
Limonium oleifolium subsp. *oleifolium* Pignatti 97
Limonium oleifolium subsp. *opulentum* (Lojac.) Brullo . 64
Limonium oleifolium subsp. *pseudodictyocladum* (Pignatti) Pignatti 66
Limonium oleifolium subsp. *sardoum* (Pignatti) Pignatti 68
Limonium oleifolium var. *majus* (Guss.) Pignatti 57
Limonium oligotrichum Erben & Brullo 64
Limonium omissae Bogdanović & Brullo 64
Limonium optimae Raimondo 64
Limonium opulentum (Lojac.) Brullo 64
Limonium opulentum (Lojac.) Greuter 64
Limonium orellii Erben 64
Limonium oristanum Alf.Mayer 64
Limonium ornatum (Ball) Kuntze 47
Limonium otolepis (Schrenk) Kuntze 81
Limonium oudayense Sauvage & Vindt 47
Limonium ovalifolium (Poir.) Kuntze 64
Limonium ovalifolium f. *biflorum* Pignatti 51
Limonium ovalifolium subsp. *biflorum* (Pignatti) Pignatti 51
Limonium ovalifolium subsp. *canariense* Pignatti 51
Limonium ovalifolium subsp. *gallicum* Pignatti 59
Limonium ovalifolium subsp. *lanceolatum* (Hoffmanns. & Link) Franco 61
Limonium ovalifolium subsp. *lusitanicum* Pignatti 64
Limonium ovalifolium subsp. *ovalifolium* 64
Limonium ovalifolium subsp. *pyramidatum* (Lowe) A.Hansen & Sunding 62
Limonium ovczinnikovii Lincz. & Czukav. 86
Limonium owerinii (Boiss.) Kuntze 45
Limonium pachynense Brullo 64
Limonium pagasaeum Erben & Brullo 79
Limonium palmare (Sm.) Rech.f. 64
Limonium palmyrense (Post) Dinsm. 75
Limonium pandatariae Pignatti 64
Limonium panormitanum (Tod.) Pignatti 65
Limonium papillatum (Webb & Berthel.) Kuntze 74
Limonium papillatum var. *callibotryum* Svent. 74
Limonium papillatum var. *papillatum* 74
Limonium paradoxum Pugsley 65
Limonium paradoxum var. *mutabile* Ingr. 64
Limonium paralimniticum Christodoulou, Erben, Hand & Kefalas 65
Limonium paramedium (Ingr.) P.D.Sell 65
Limonium parosicum Erben & Brullo 65
Limonium parvibracteatum Pignatti 65
Limonium parvifolium (Tineo) Pignatti 65

Limonium parvum Ingr. 65
Limonium patagonicum Macloskie 76
Limonium patrimoniense Arrigoni & Diana 65
Limonium paui Cámara & Sennen 65
Limonium paulayanum (Vierh.) Ghaz. & J.R.Edm. 85
Limonium pavonianum Brullo 65
Limonium pectinatum (Aiton) Kuntze 74
Limonium pectinatum var. *corculum* (Webb & Berthel.) G.Kunkel & Sunding 74
Limonium pectinatum var. *divaricatum* (Pit.) G.Kunkel & Sunding 74
Limonium pectinatum var. *incomptum* (Webb & Berthel.) G.Kunkel & Sunding 74
Limonium pectinatum var. *pectinatum* 74
Limonium pectinatum var. *solandri* (Webb & Berthel.) Kuntze 74
Limonium pedicellatum (Wallr. ex Boiss.) Kuntze 65
Limonium pelagosae Bogdanović & Brullo 65
Limonium penicillatum Adamson 65
Limonium peregrinum (P.J.Bergius) R.A.Dyer 72, 73
Limonium perezii (Stapf) F.T.Hubb. ex L.H.Bailey 88
Limonium perfoliatum (C.A.Mey. ex Boiss.) Kuntze 81
Limonium pericotii (O.Bolòs & Vigo) Greuter & Raus 65
Limonium perplexum L.Sáez & Rosselló 65
Limonium peruvianum Kuntze 39
Limonium pescadense Greuter & Raus 65
Limonium peucetium Pignatti 65
Limonium pharense (Ingr.) P.D.Sell 65
Limonium pharosianum Bogdanović & Brullo 65
Limonium phitosianum R.Artelari 65
Limonium pigadiense (Rech.f.) Rech.f. 65
Limonium pignattii Brullo & Di Martino 57
Limonium pignattii Brullo & Di Martino ex Brullo 57
Limonium pinillense Roselló, Stübing, Peris J.B. & Cirujano 65
Limonium piptopodum Nevski 86
Limonium planesiae Pignatti 65
Limonium platyphyllum Lincz. 77
Limonium plumosum (Phil.) Kuntze 40
Limonium plurisquamatum Erben 65
Limonium plutosianum Artell 97
Limonium poimenum Ilardi, Brullo, D.Cusimano & G.Giusso 65
Limonium pomelianum (Rouy) Erben 66
Limonium pomoense Bogdanović & Brullo 66
Limonium pontium Pignatti 66
Limonium pontium subsp. *pandatariae* (Pignatti) Iamonico, Iberite, De Castro & Nicolella 65
Limonium pontium subsp. *pontium* 66
Limonium pontium subsp. *terracinense* Iberite, Iamonico, De Castro, Nicolella Iamonico, De Castro & Nicolella 49
Limonium ponzoi (Fiori & Bég.) Brullo 66
Limonium popovii Kubansk. 81
Limonium portlandicum (Ingr.) P.D.Sell 66
Limonium portopetranum Erben 66
Limonium portovecchiense Erben 66
Limonium postii Domina, Erben & Raimondo 66
Limonium potaninii Ikonn.-Gal. 83
Limonium preauxii (Webb & Berthel.) Kuntze 88
Limonium procerum (C.E.Salmon) Ingr. 66
Limonium procerum subsp. *cambrense* Ingr. 52
Limonium procerum subsp. *devoniense* Ingr. 56
Limonium procerum var. *cornubiense* Ingr. 54
Limonium procerum var. *hibernicum* Ingr. 60
Limonium procerum var. *medium* Ingr. 62
Limonium procerum var. *paramedium* Ingr. 65
Limonium procerum var. *wessexense* Ingr. 72
Limonium proliferum (d'Urv.) Erben & Brullo 66
Limonium protohermaeum Arrigoni & Diana 66
Limonium pruinosum (L.) Chaz. 47
Limonium pruinosum (L.) Kuntze 47
Limonium pruinosum subsp. *alleizettei* (Pau) Maire 47
Limonium pruinosum var. *glabrum* Maire & Weiller 47
Limonium pruinosum var. *hirtiflorum* (Cavara) Täckh. ex Feinbrun 47
Limonium pseudarticulatum Erben 66
Limonium pseudebusitanum Erben 66
Limonium pseudodictyocladum (Pignatti) L.Llorens 66
Limonium pseudodictyocladum Greuter & Raus 66
Limonium pseudolaetum Arrigoni & Diana 66
Limonium pseudominutum Erben 66
Limonium pseudoparadoxum (Ingr.) P.D.Sell 66
Limonium pseudotranswallianum (Ingr.) P.D.Sell 66
Limonium psilocladum (Boiss.) Kuntze 67
Limonium puberulum (Webb ex Lindl.) H.Arnaud 88
Limonium puberulum (Webb ex Lindl.) Kuntze 88
Limonium pubescens (DC.) P.Fourn. 54
Limonium pujosii Sauvage & Vindt 67
Limonium pulviniforme Arrigoni & Diana 67
Limonium punicum Brullo 60
Limonium purpuratum (L.) Chaz. 73
Limonium purpuratum (L.) F.T.Hubb. 73
Limonium purpuratum var. *longifolium* (Thunb.) F.T.Hubb. 73
Limonium pusillum Erben & Brullo 67
Limonium pycnanthum (K.Koch) Kuntze 86
Limonium pylium R.Artelari 67
Limonium pyramidatum Brullo 67
Limonium quesadense Erben 67
Limonium quinnii M.B.Crespo & Pena-Martín 67
Limonium racemosum (Lojac.) Diana 67
Limonium raddianum (Boiss.) Pignatti ex Brullo 67
Limonium ramosissimum (Poir.) Maire 67
Limonium ramosissimum subsp. *confusum* (Gren. & Godr.) Pignatti 53
Limonium ramosissimum subsp. *doerfleri* (Halácsy) Pignatti 57
Limonium ramosissimum subsp. *provinciale* (Pignatti) Pignatti 67
Limonium ramosissimum subsp. *ramosissimum* 67
Limonium ramosissimum subsp. *siculum* Pignatti 60
Limonium ramosissimum subsp. *tommasinii* (Pignatti) Pignatti 56
Limonium rariflorum (Drejer) Kuntze 78
Limonium recticaule Erben & Brullo 67
Limonium recurviforme (Ingr.) P.D.Sell 67
Limonium recurvum C.E.Salmon 67
Limonium recurvum subsp. *crigyllensis* I.Rees 67
Limonium recurvum subsp. *humile* (Girard) Ingr. 67
Limonium recurvum subsp. *portlandicum* Ingr. 66
Limonium recurvum subsp. *pseudotranswallianum* Ingr. 66
Limonium recurvum subsp. *recurvum* 67
Limonium recurvum var. *donegalense* Ingr. 57
Limonium recurvum var. *humile* (Girard) Ingr. 67

Limonium recurvum var. *kerryense* Ingr. 61
Limonium recurvum var. *portlandicum* Ingr. 66
Limonium recurvum var. *pseudoparadoxum* Ingr. 66
Limonium recurvum var. *recurviforme* Ingr. 67
Limonium redivivum (Svent.) G.Kunkel & Sunding 88
Limonium redivivum var. *pilosum* (Svent.) G.Kunkel & Sunding 88
Limonium redivivum var. *redivivum* 88
Limonium relicticum R.Mesa & A.Santos 88
Limonium remotispiculum (Lacaita) Pignatti 67
Limonium reniforme (Girard) Lincz. 80, 81
Limonium reticulatum (L.) Mill. 67
Limonium retirameum Greuter & Raus 67
Limonium retirameum subsp. *caralitanum* (Erben) Arrigoni 68
Limonium retirameum subsp. *retirameum* 67
Limonium retusum L.Llorens 67
Limonium revolutum Erben 68
Limonium rezniczenkoanum Lincz. 83
Limonium rhodense M.B.Crespo & Pena-Martín 49
Limonium rigidum Alf.Mayer 55
Limonium rigualii M.B.Crespo & Erben 68
Limonium roborowskii Ikonn.-Gal. 83
Limonium romanum (Täckh. & Boulos) Domina 89
Limonium roridum (Sibth. & Sm.) Brullo & Guarino 68
Limonium roseum (Sm.) Kuntze 73
Limonium rosselloi Ferrer-Gallego, P. P., R.Roselló & E.Laguna 68
Limonium rubescens Brullo 68
Limonium ruizii (Font Quer) Fern.Casas 68
Limonium rumicifolium (Svent.) G.Kunkel & Sunding 88
Limonium runemarkii Rech.f. 69
Limonium rungsii Sauvage & Vindt 68
Limonium rupicola (Badarò ex Rchb.) Kuntze 48
Limonium rytidophyllum (Hook.) H.Arnaud 73
Limonium sabulicola P.D.Sell 68
Limonium salicorniacea (F.Muell.) Kuntze 90
Limonium salmonis (Sennen & Elías) Pignatti 68
Limonium salsuginosum (Boiss.) Kuntze 70
Limonium samium Erben & Brullo 68
Limonium sanctamargaritense P.D.Sell 68
Limonium sanjurjoi Sennen & Mauricio 54
Limonium santapolense Erben 68
Limonium saracinatum R.Artelari 68
Limonium sarcophyllum Ghaz. & J.R.Edm. 85
Limonium sardoum (Pignatti) Erben 68
Limonium sareptanum (A.K.Becker) Gams 79
Limonium sarniense (Ingr.) P.D.Sell 68
Limonium sartorianum Erben & Brullo 68
Limonium sartorii (Nyman) Kuntze 44
Limonium savianum Pignatti 59
Limonium saxicola Erben 68
Limonium saxonicum (Ingr.) P.D.Sell 68
Limonium scabrum (Thunb.) Kuntze 68
Limonium scabrum var. *avenaceum* (C.H.Wright) R.A.Dyer 50
Limonium scabrum var. *corymbulosum* (Boiss.) R.A.Dyer 54
Limonium scabrum var. *scabrum* (Thunb.) Kuntze 68
Limonium schinousae Erben & Brullo 68
Limonium schrenkianum (Fisch. & C.A.Mey.) Kuntze 82
Limonium scoparium (M.Bieb.) Stankov 78
Limonium scoparium (Pall. ex Willd.) H.Arnaud 78
Limonium scoparium Klokov 78
Limonium scoparium var. *meyeri* (Boiss.) Tzvelev 78
Limonium scopulorum M.B.Crespo & Lledó 68
Limonium scorpioides Erben 68
Limonium sebkarum (Pomel) Maire 69
Limonium sect. *Circinaria* (Boiss.) M.Malekm. 72
Limonium sect. *Ctenostachys* (Boiss.) Sauvage & Vindt 73
Limonium sect. *Iranolimon* M.Malekm., Akhani & Borsch 75
Limonium sect. *Jovibarba* (Boiss.) M.Malekm. & Koutr. 75
Limonium sect. *Limoniodendron* Svent. 75
Limonium sect. *Limonium* 75, 86
Limonium sect. *Myriolepis* (Boiss.) Sauvage & Vindt 90
Limonium sect. *Nephrophyllum* Rech.f. 80
Limonium sect. *Odontolepideae* (Boiss.) Koutr. 88
Limonium sect. *Plathymenium* (Boiss.) Lincz. 81
Limonium sect. *Polyarthrion* (Boiss.) Sauvage & Vindt 46
Limonium sect. *Pruinosa* (Batt.) Koutr. 47
Limonium sect. *Pruinosum* (Batt.) Koutr. 47
Limonium sect. *Pterocladus* (Spach) Bokhari 87
Limonium sect. *Sarcophylla* (Boiss.) Lincz. 84
Limonium sect. *Sarcophyllum* (Boiss.) Lincz. 84
Limonium sect. *Schizhymenium* (Boiss.) Sauvage & Vindt 47
Limonium sect. *Siphonantha* (Boiss.) Sauvage & Vindt 48
Limonium sect. *Siphonocalyx* Lincz. 85
Limonium sect. *Sphaerostachys* (Boiss.) Bokhari 86
Limonium sect. *Tenuiramosa* Koutr. 90
Limonium sect. *Tenuiramosum* Koutr. 90
Limonium secundirameum (Lojac.) Brullo 69
Limonium secundirameum (Lojac.) Greuter & Raus 69
Limonium secundirameum (Lojac.) Pignatti 69
Limonium sedodes (Regel) Kuntze 82
Limonium selinuntinum Brullo 69
Limonium semenowii (Herder) Kuntze 83
Limonium semenowii var. *chrysocephalum* (Regel) Grubov 82
Limonium semenowii var. *sedoides* (Regel) Grubov 82
Limonium senkakuense T.Yamaz. 83
Limonium serbicum (Nyman) Kuntze 44
Limonium sercquense (Ingr.) P.D.Sell 69
Limonium serotinum (Rchb.) Erben 79
Limonium serotinum (Rchb.) Pignatti 79
Limonium serpentinicum R.Pino, Silva Pando & J.J.Pino 69
Limonium serratum Brullo 69
Limonium sewerzowii (Herder) Kuntze 44
Limonium sibthorpianum (Guss.) Kuntze 69
Limonium sibthorpianum subsp. *vaccarii* Pignatii ex Brullo 71
Limonium siculum Mill. 45
Limonium sieberi (Boiss.) Kuntze 69
Limonium silvestrei Aparicio 69
Limonium sinense (Girard) Kuntze 83
Limonium sinense var. *spinulosum* Y.Huang 84
Limonium sinisicum Erben 71
Limonium sinuatum (L.) Mill. 86, 87, 88, 89
Limonium sinuatum f. *leucocalyx* (Maire) Sauvage & Vindt 89
Limonium sinuatum f. *pallidum* Maire 89
Limonium sinuatum subsp. *beaumierianum* (Coss. ex Maire) Sauvage & Vindt 88
Limonium sinuatum subsp. *bonduellei* (T.Lestib.) Sauvage & Vindt 89

Limonium sinuatum subsp. *romanum* Täckh. & Boulos 89
Limonium sinuatum subsp. *sinuatum* (L.) Mill. 89
Limonium sinuatum subvar. *annuum* (Maire) Sauvage & Vindt 89
Limonium sinuatum subvar. *glabrescens* (Maire) Sauvage & Vindt 89
Limonium sinuatum var. *akkense* (Coss. ex Batt.) Sauvage & Vindt 88
Limonium sinuatum var. *annuum* Maire 89
Limonium sinuatum var. *candidissimum* (hort.) F.T.Hubb. 89
Limonium sinuatum var. *sinuatum* (L.) Mill. 89
Limonium sinuatum var. *subglabrum* (H.Lindb.) Maire 89
Limonium sinuatum var. *tripeaui* (Maire) Sauvage & Vindt 88
Limonium sinuatum var. *typicum* Maire 89
Limonium sirinicum Erben & Brullo 69
Limonium sitiacum Rech.f. 69
Limonium smithii Akaydın 81
Limonium soboliferum Erben 69
Limonium sogdianum (Popov) Ikonn.-Gal. 85, 86
Limonium sokotranum (Vierh.) Radcl.-Sm. 85
Limonium solanderi Lincz. 84
Limonium solandri (Webb & Berthel.) G.Kunkel 74
Limonium somalorum (Vierh.) Hutch. & E.A.Bruce 85
Limonium sommierianum (Fiori) Arrigoni 69
Limonium sougiae Erben & Brullo 69
Limonium spathulatum (Desf.) Kuntze 69
Limonium spathulatum subsp. *rusicadense* (Maire) Quézel & Santa 69
Limonium spathulatum var. *emarginatum* (Willd.) C.E.Hubb. & Sandwith 58
Limonium speciosum (L.) Chaz. 44
Limonium speciosum (L.) Kuntze 44
Limonium spectabile (Svent.) G.Kunkel & Sunding 88
Limonium spicatum (Willd.) Kuntze 92
Limonium spreitzenhoferi Erben & Brullo 69
Limonium squarrosum Erben 69
Limonium stenophyllum Erben 69
Limonium stenotatum (Rech.f.) Erben & Brullo 69
Limonium stephanii Sennen 50
Limonium steppicum Sefi, Ghrabi-Gammar & Brullo 69
Limonium stocksii (Boiss.) Kuntze 85
Limonium strictissimum (Salzm.) Arrigoni 70
Limonium subanfractum Trinajstić 70
Limonium subg. Limonium 46, 75
Limonium subg. Myriolepis (Boiss.) Pignatti 90
Limonium subg. Pterocladus (Spach) H. Arnaud 86
Limonium subg. Pterocladus (Spach) Pignatti 87
Limonium subglabrum Erben 70
Limonium subnudum Bogdanović & Brullo 70
Limonium subrotundifolium (Bég. & A.Vacc.) Brullo 70
Limonium subsect. *Nobiles* (Boiss.) Koutr. 87
Limonium subsect. *Nobilia* (Boiss.) Koutr. 87
Limonium subsect. *Odontolepidea* (Boiss.) Koutr. 88
Limonium subsect. *Odontolepidea* (Boiss.) Sauvage & Vindt 88
Limonium subsect. *Pruinosa* (Batt.) Sauvage & Vindt ... 47
Limonium subviolaceum Q.Z.Han & S.D.Zhao 83
Limonium sucronicum Erben 70
Limonium suffruticosum (L.) Kuntze 75
Limonium sulcitanum Arrigoni 70
Limonium sundingii Leyens, Lobin, N.Kilian & Erben 75
Limonium superbum (Regel) F.T.Hubb. ex L.H.Bailey 91
Limonium superbum var. *flore-albo* (Benary) L.H.Bailey 91
Limonium supinum (Girard) Pignatti 70
Limonium supinum Erben 65
Limonium supinum var. *diegoi* (Sennen) Pignatti 70
Limonium supinum var. *supinum* 70
Limonium suwarowii (Regel) Kuntze 92
Limonium suworowii (Regel) Kuntze 92
Limonium suworowii var. *album* (hort.) F.T.Hubb. 92
Limonium sventenii A.Santos & M.L.Fernández 88
Limonium syracusanum Brullo 70
Limonium tabernense Erben 70
Limonium tabulare Bogdanović & Brullo 70
Limonium tacapense Brullo 70
Limonium taenari Erben & Brullo 70
Limonium tamaricoides Bokkari 81
Limonium tamarindanum Erben 70
Limonium tanaiticum Gamajun. 78
Limonium tarcoense Arrigoni & Diana 70
Limonium tataricum (L.) Mill. 44
Limonium tataricum var. *angustifolium* (Boiss.) F.T.Hubb. 42
Limonium tataricum var. *coccineum* F.T.Hubb. 98
Limonium tataricum var. *nanum* F.T.Hubb. 98
Limonium tauromenitanum Brullo 70
Limonium tenellum (Turcz.) Ikonn.-Gal. 84
Limonium tenellum (Turcz.) Kuntze 84
Limonium tenium (Heldr.) Rech.f. 72
Limonium tenoreanum (Guss.) Pignatti 70
Limonium tenuicaule Erben 70
Limonium tenuicolum (Tineo ex Guss.) Pignatti 70
Limonium tenuiculum (Tineo ex Cuss.) Desole 70
Limonium tenuiculum (Tineo ex Guss.) Desole & Pignatti 70
Limonium tenuiculum (Tineo ex Guss.) Pignatti 70
Limonium tenuiculum subsp. *hermaeum* Pignatti 60
Limonium tenuifolium (Bertol. ex Moris) Erben 70
Limonium teretifolium (Baker ex Oliv.) Cufod. 85
Limonium teretifolium L.Bolus 73
Limonium teretiscaposum S.D.Zhao 83
Limonium teretiscaposum var. *microphyllum* S.D.Zhao 83
Limonium tetragonum (Thunb.) Bullock 84
Limonium teuchirae Brullo 70
Limonium thaenicum Brullo 70
Limonium tharrosianum Arrigoni & Diana 70
Limonium thiniense Erben 71
Limonium thirae Erben & Brullo 71
Limonium thouinii (Viv.) Kuntze 89
Limonium thymoides (Girard) M.B.Crespo 47
Limonium tianschanicum Lincz. 81
Limonium tibulatium Pignatti 71
Limonium tigulianum Arrigoni & Diana 71
Limonium tineoi (Lojac.) Giardina & Raimondo 71
Limonium tobarrense J.Moreno, Terrones, M.Á.Alonso, Juan & M.B.Crespo 71
Limonium todaroanum Raimondo & Pignatti 71
Limonium toletanum Erben 71
Limonium tomentellum (Boiss.) Kuntze 79
Limonium tomentellum subsp. *alutaceum* (Steven) Moysiyenko 49
Limonium tomentellum subsp. *donetzicum* (Klokov) Moysiyenko 79

Limonium tomentellum subsp. *hypanicum* (Klokov) Moysiyenko ... 78
Limonium tomentellum var. *sareptanum* C.E.Salmon ... 79
Limonium tommasinii (Pignatti) Brullo ... 56
Limonium Tourn. ex Mill. ... 45
Limonium tournefortii (Girard) Erben ... 71
Limonium trachycladum Maire & Wilczek ... 74
Limonium transcanalis (Ingr.) P.D.Sell ... 71
Limonium transwallianum (Pugsley) Pugsley ... 71
Limonium tremolsii (Rouy) P.Fourn. ... 71
Limonium tremolsii (Rouy) P.Fourn. ex Erben ... 71
Limonium trichogonum f. *albiflorum* House ... 77
Limonium trichogonum S.F.Blake ... 77
Limonium trigonodes Kuntze ... 43
Limonium trinajsticii Bogdanović & Brullo ... 71
Limonium tritonianum Brullo ... 71
Limonium trojae Pignatti ... 63
Limonium tschurjukiense (Klokov) Lavr. ex Klokov ... 79
Limonium tuberculatum (Boiss.) Kuntze ... 47
Limonium tubiflorum (Delile) Kuntze ... 48
Limonium tubiflorum subsp. *maroccanum* (Batt. & Trab.) Maire & Weiller ... 48
Limonium tubiflorum subsp. *zanonii* (Pamp.) Brullo ... 48
Limonium tubiflorum var. *zanonii* (Pamp.) Maire ... 48
Limonium tunetanum (Barratte & Bonnet) Maire ... 71
Limonium tunetanum subsp. *eugeniae* (Sennen) Pignatti ... 58
Limonium turritanum Diana ... 61
Limonium tyrrhenicum Arrigoni & Diana ... 71
Limonium ugijarense Erben ... 71
Limonium ursanum Erben ... 71
Limonium usticanum Giardina & Raimondo ... 71
Limonium vaccarii Pignatti ex Brullo ... 71
Limonium validum Erben ... 71
Limonium vanandense Erben & Brullo ... 72
Limonium vanense Kit Tan & Sorger ... 79
Limonium velutinum Bogdanović & Brullo ... 72
Limonium vestitum (C.E.Salmon) C.E.Salmon ... 72
Limonium vestitum subsp. *brusnicense* Trinajstić ... 52
Limonium vestitum subsp. *vestitum* ... 72
Limonium viciosoi (Pau) Erben ... 72
Limonium vigaroense Marrero Rodr. & R.S.Almeida ... 88
Limonium vigoi L.Sáez, Curcó & Rosselló ... 72
Limonium viniolae Arrigoni & Diana ... 72
Limonium virgatum (Willd.) Fourr. ... 72
Limonium virgatum subsp. *divaricatum* Pignatti ... 57
Limonium virgatum subsp. *fontqueri* (Pau) O.Bolòs, Vigo, Masalles & Ninot ... 58
Limonium virgatum subsp. *pseudodictyocladum* (Pignatti) O.Bolòs, Vigo, Masalles & Ninot ... 66
Limonium virgatum subsp. *pseudodictyocladum* Pignatti ... 66
Limonium virgatum subsp. *sardoum* Pignatti ... 68
Limonium vravronense Erben & Brullo ... 72
Limonium vulgare f. *pyramidale* C.E.Salmon ... 80
Limonium vulgare Mill. ... 45, 46, 75, 79
Limonium vulgare subsp. *angustifolium* (Tausch) P.Fourn. ... 76
Limonium vulgare subsp. *bahusiense* (Fr.) Bech. ... 78
Limonium vulgare subsp. *behen* (Drejer) Bech. ... 79, 80
Limonium vulgare subsp. *humile* (Mill.) Gams ... 78
Limonium vulgare subsp. *pseudolimonium* (Rchb.) Gams ... 80
Limonium vulgare subsp. *serotinum* (Rchb.) Gams ... 79
Limonium vulgare var. *album* F.T.Hubb. ... 80
Limonium vulgare var. *macroclada* (Boiss.) F.T.Hubb. ... 76
Limonium wendelboi Bokhari ... 84
Limonium wessexense (Ingr.) P.D.Sell ... 72
Limonium wiedmannii Erben ... 72
Limonium willdenowii (Poir.) P.Fourn. ... 50
Limonium willdenowii Pignatti ... 52
Limonium woolacombense P.D.Sell ... 72
Limonium wrightii (Hance) Kuntze ... 84
Limonium wrightii f. *albescens* Hatus. ... 84
Limonium wrightii f. *albolutescens* Hatus. ... 84
Limonium wrightii f. *arbusculum* (Maxim.) Hatus. ... 84
Limonium wrightii var. *luteum* (H.Hara) H.Hara ... 84
Limonium wrightii var. *roseum* H.Hara ... 84
Limonium wrightii var. *wrightii* (Hance) Kuntze ... 84
Limonium xerocamposicum Erben & Brullo ... 72
Limonium xerophilum Brullo ... 72
Limonium xiliense Erben & Brullo ... 72
Limonium xipholepis (Baker) Hutch. & E.A.Bruce ... 85
Limonium zacynthium R.Artelari ... 72
Limonium zankii Bogdanović & Brullo ... 72
Limonium zanonii (Pamp.) Domina ... 48
Limonium zembrae Pignatti ... 72
Limonium zeraphae Brullo ... 72
Limonium zeugitanum Brullo ... 72
Linczevskia sinuata (L.) Tzvelev ... 45, 89
Linczevskia thouinii (Viv.) Tzvelev ... 89
Linczevskia Tzvelev ... 45
Molubda Raf. ... 94
Molubda scandens (L.) Raf. ... 94, 95
Muellerolimon Lincz. ... 90, 92
Muellerolimon salicorniaceum (F.Muell.) Lincz. ... 90
Myriolepis (Boiss.) Lledó, Erben & M.B.Crespo ... 90
Myriolepis diffusa (Pourr.) Lledó, Erben & M.B.Crespo 90
Myriolepis ferulacea (L.) Lledó, Erben & M.B.Crespo ... 90
Myriolimon diffusum (Pourr.) Lledó, Erben & M.B.Crespo ... 90
Myriolimon ferulaceum (L.) Lledó, Erben & M.B.Crespo ... 90
Myriolimon Lledó, Erben & M.B.Crespo ... 90, 92
Neogontscharovia Lincz. ... 90
Neogontscharovia mira (Lincz.) Lincz. ... 90
Neogontscharovia miranda (Lincz.) Lincz. ... 90, 91
Neogontscharovia saxifragifolia (Rech.f. & Köie) Lincz. 91
Plegorhiza adstringens Willd. ... 78
Plegorhiza guaicuru Molina ... 45, 78
Plegorhiza Molina ... 45
Plegorhiza quicura Steud. ... 78
Plumbagella micrantha (Ledeb.) Spach ... 94
Plumbagella micrantha var. *himalaica* W.W.Sm. ... 94
Plumbagella micrantha var. *micrantha* ... 94
Plumbagella Spach ... 94
Plumbagidium auriculatum (Lam.) Spach ... 94
Plumbagidium roseum (L.) Spach ... 95
Plumbagidium scandens (L.) Spach ... 95
Plumbagidium Spach ... 94
Plumbaginaceae Juss. ... 1
Plumbagineae Bartl. ... 93
Plumbagineae Dumort. ... 93
Plumbago africana (Lam.) Christenh. & Byng ... 93
Plumbago alba hort. ex Pasq. ... 94
Plumbago americana L. ... 95

Plumbago americana Weigel....95
Plumbago amplexicaulis Oliv....94
Plumbago angustifolia Spach....95
Plumbago aphylla Bojer ex Boiss....94
Plumbago arabica (Boiss.) Christenh. & Byng....94
Plumbago auriculata Blume....95
Plumbago auriculata f. *alba* (Pasq.) T.H.Peng....94
Plumbago auriculata f. *auriculata*....94
Plumbago auriculata Lam....94
Plumbago caerulea Kunth....94
Plumbago capensis f. *alba* hort. ex Carrière....98
Plumbago capensis Thunb....94
Plumbago capensis Willd. ex Boiss....94
Plumbago ciliata Engl. ex Wilmot-Dear....94
Plumbago cocanex hort. ex Gentil....101
Plumbago coccinea Salisb....95
Plumbago coerulea Auct....101
Plumbago dawei Rolfe....95
Plumbago denticulata St.-Lag....95
Plumbago dyerophyta Christenh. & Byng....94
Plumbago eglandulosa R.Br....93
Plumbago esquirolii H.Lév....100
Plumbago europaea L....94, 95
Plumbago flaccida Moench....95
Plumbago floridana Nutt....95
Plumbago floridana Raf....95
Plumbago glandulicaulis Wilmot-Dear....95
Plumbago glandulosa Willd. ex Roem. & Schult....93
Plumbago grandiflora Ten....94
Plumbago humboldtiana Roem. & Schult....94
Plumbago hunsbergensis van Jaarsv., Swanepoel & A.E.van Wyk....95
Plumbago indica L....95
Plumbago ituriensis Ntore....95
Plumbago juncea Bojer....95
Plumbago lactea Salisb....95
Plumbago lanceolata Sessé & Moc....95
Plumbago lapathifolia Willd....95
Plumbago larpentae Lindl....93
Plumbago larpentiae Lindl....93
Plumbago littoralis Mure....95
Plumbago madagascariensis M.Peltier....95
Plumbago maximowiczii Gand....95
Plumbago mexicana Kunth....95
Plumbago micrantha Ledeb....94
Plumbago montis-elgonis Bullock....95
Plumbago occidentalis Sweet....95
Plumbago parvifolia Hemsl....94
Plumbago pearsonii L.Bolus....95
Plumbago pendula (Balf.f.) Christenh. & Byng....94
Plumbago pulchella Boiss....95
Plumbago purpurea Salisb....95
Plumbago rhombifolia Steud....101
Plumbago rhomboidea G.Lodd....95
Plumbago rhomboidea Hook....94
Plumbago rosea L....95
Plumbago rosea var. *coccinea* (Lour.) Hook....95
Plumbago sarmentosa Lam....95
Plumbago scandens f. *erecta* Chodat & Hassl....95
Plumbago scandens L....95
Plumbago scandens var. *densiflora* Kuntze....95
Plumbago scandens var. *erecta* Chodat & Hassl....95
Plumbago scandens var. *normalis* Kuntze....95
Plumbago sect. *Plumbagidium* Boiss....94
Plumbago spinosa K.S.Hao....94
Plumbago stenophylla Wilmot-Dear....95
Plumbago suffruticosa Schinz....95
Plumbago tomentosa var. *pumila* Hook.f....98
Plumbago Tourn. ex L....1, 93, 94
Plumbago Tourn. ex L. sect. *Plumbago*....94
Plumbago toxicaria Bertol....95
Plumbago tristis W.T.Aiton....95
Plumbago undulata Moench....95
Plumbago virginica L....95
Plumbago viscosa Blanco....95
Plumbago vogeliaefolia Eckl. & Zeyh. ex Boiss....95
Plumbago vogeliifolia Eckl. & Zeyh. ex Boiss....95
Plumbago wissii Friedrich....95
Plumbago zeylanica L....95, 96
Plumbago zeylanica var. *dawei* (Rolfe) Mildbr....95
Plumbago zeylanica var. *glaucescens* Boiss....95
Plumbago zeylanica var. *oxypetala* Boiss....95
Plumbago zeylanica var. *rosea* (L.) Williams....95
Plumbago zeylanica var. *zeylanica* L....96
Polyanthemum Medik....18
Popoviolimon Lincz....91
Popoviolimon turcomanicum (Popov ex Lincz.) Lincz....91
Psylliostachys (Jaub. & Spach) Nevski....91
Psylliostachys ×afghanica Roshkova....91
Psylliostachys ×androssovii Roshkova....91
Psylliostachys ×myosuroides (Regel) Roshkova....91
Psylliostachys anceps (Regel) Roshkova....91
Psylliostachys beludshistanica Roshkova....91
Psylliostachys hymenostegia Rech.f....91
Psylliostachys koelzii Rech.f....91
Psylliostachys leptostachya (Boiss.) Roshkova....91
Psylliostachys leptostachyus (Boiss.) Roshkova....91
Psylliostachys plantaginiflora (Jaub. & Spach) Roshkova....92
Psylliostachys sisymbriifolia (Jaub. & Spach) Rech.f....92
Psylliostachys spicata (Willd.) Nevski....91, 92
Psylliostachys suvorovii (Regel) Roshk....92
Psylliostachys suworowi (Regel) Roshkova....92
Psylliostachys suworowii (Regel) Roshkova....92
Psylliostachys volkii Rech.f....92
Reverchonia baetica (Boiss.) Gand....29
Reverchonia Gand....18
Saharanthus ifniensis (Caball.) M.B.Crespo & Lledó....92
Saharanthus M.B.Crespo & Lledó....92
Statice (L.) Mill....18
Statice ×abnormis Rouy....48
Statice ×ambigua Rouy....49
Statice ×coincyi Sennen....53
Statice ×erectiflora B.Fedtsch. & Gontsch....58
Statice ×girardianoides H.Lév....66
Statice ×gracillima (J.J.Rodr.) Rouy....101
Statice ×intermedia Czern....79
Statice ×multiramea Sennen....53
Statice ×myosuroides Regel....91
Statice ×neumani Rouy....64
Statice ×pinto-silvae Rothm....101
Statice ×profusa T.Moore....88
Statice ×pseudoconfusa Rouy....66
Statice ×sennenii Rouy....69
Statice ×valentina Huter, Porta & Rigo....71
Statice ×virgatoformis Rouy....72

Statice acerosa Willd. 3
Statice aciphylla Jaub. & Spach 9
Statice acutifolia Badarò ex Rchb. 48
Statice acutifolia Ehrenb. ex Boiss. 89
Statice acutifolia var. *obtusifolia* Rouy 64
Statice adensis (Nimmo) Boiss. 84
Statice aegyptiaca Pers. 89
Statice aggregata Rouy 79
Statice airoides Tausch 80
Statice akkensis Coss. ex Batt. 88
Statice alata Willd. 89
Statice alatavica Regel & Schmalh. 44
Statice alba Coincy 49
Statice alba Pau 51
Statice albertii Regel 91
Statice albicans Ledeb. 42
Statice albida Guss. 49
Statice algeriensis Rouy 101
Statice alleizettei Pau 47
Statice alliacea Cav. 18, 30
Statice alliacea Guss. ex Nyman 30
Statice alliacea Sm. 99
Statice alliacea var. *apollinaris* (Sennen & Mauricio) Maire 20
Statice alliacea var. *matritensis* Pau 20
Statice alliacea Willd. 21
Statice allioides Braun-Blanq. 19
Statice allioides var. *yebalica* (Pau) Maire 34
Statice alpina (DC.) Poir. 20
Statice alpinifolia (Pau & Font Quer) Maire 20
Statice altaica hort. ex G.Don 98
Statice alutacea Steven 49
Statice ambigua f. *major* Lojac. 71
Statice ambigua Tineo ex Lojac. 51
Statice ambigua var. *ponzoi* Fiori & Bég. 66
Statice amblyoloba Ikonn.-Gal. 83
Statice amblyoloba Popov 83
Statice americana Larrañaga 76
Statice amoena C.H.Wright 73
Statice amplifoliata (Pau) Maire 37
Statice ancarensis (Merino) Rothm. 27
Statice anceps Regel 91
Statice androsacea Jaub. & Spach 17
Statice anfracta C.E.Salmon 56
Statice angustata (Small) Wangerin 77
Statice angustifolia Tausch 76
Statice annulata (R.Br.) Spreng. 2
Statice anthericoides Schltr. 90
Statice aphylla Forssk. 47
Statice aphylla Poir. 81
Statice arabica f. *glomerata* N.Terrac. 84
Statice arabica Jaub. & Spach 84
Statice aragonensis Debeaux ex Willk. 50
Statice ararati Planch. 9
Statice arborea f. *frutescens* (Lem.) Stapf. 87
Statice arborea var. *typica* Stapf 87
Statice arborea Willd. 87
Statice arborescens Brouss. 87
Statice arborescens Brouss. ex Webb & Berthel. 87
Statice arbuscula Maxim. 84
Statice arctica (Cham.) S.F.Blake 34
Statice arctica subsp. *californica* (Boiss.) A.E.Murray 32
Statice arctica var. *californica* (Boiss.) S.F.Blake 32
Statice aretiifolia Fr. ex Boiss. 61
Statice argentea Pall. ex Siev. 42
Statice aristata Sm. 48
Statice armeria Brot. 39
Statice armeria L. 18, 30, 45
Statice armeria subsp. *armeria* 33
Statice armeria subsp. *elongata* (Hoffm.) P.Fourn. 32
Statice armeria subsp. *major* Ehrh. 32
Statice armeria subsp. *maritima* (Mill.) P.Fourn. 33
Statice armeria var. *alba* (L.H.Bailey) F.T.Hubb. 31
Statice armeria var. *alpina* DC. 20
Statice armeria var. *arenaria* Dumort. 31
Statice armeria var. *caudiculata* Dumort. 31
Statice armeria var. *elongata* Dumort. 32
Statice armeria var. *grandiflora* (L.H.Bailey) F.T.Hubb. 31
Statice armeria var. *hortensis* Gaudin 31
Statice armeria var. *laucheana* (J.N.Haage & E.Schmidt) F.T.Hubb. 33
Statice armeria var. *maritima* (Mill.) Wahlenb. 30
Statice armeria var. *minor* Dumort. 31
Statice armeria var. *mixta* Dumort. 31
Statice armeria var. *pubescens* Salis 29
Statice armeria var. *pubescens* Sowerby ex Sm. 30
Statice armeria var. *purpurea* (L.H.Bailey) F.T.Hubb. 31
Statice armeria var. *rubra* (L.H.Bailey) F.T.Hubb. 31
Statice armeria var. *splendens* (L.H.Bailey) F.T.Hubb. 31
Statice armeria var. *tenuifolia* DC. 32
Statice armeria var. *variegata* F.T.Hubb. 101
Statice articulata Loisel. 50
Statice articulata Schimp. ex Nyman 52
Statice articulata var. *strictissima* Salzm. 70
Statice articulata Vis. 55
Statice asparagoides Coss. & Durieu ex Batt. 47
Statice aspera Lam. 48
Statice asterotricha C.E.Salmon 76
Statice asturiana Rothm. 27
Statice attenuata Schult. 84
Statice aucheri Girard 50
Statice aurea L. 82
Statice auriculae-ursifolia Pourr. 50
Statice auriculata hort. ex Schult. 98
Statice auriculifolia Benth. 50
Statice auriculifolia Brot. 50
Statice auriculifolia DC. 50
Statice auriculifolia Poir. 50
Statice auriculifolia Vahl 50
Statice australis (R.Br.) Spreng. 82
Statice australis Moris 61
Statice avei De Not. 48
Statice avenacea C.H.Wright 50
Statice axillaris Balf.f. 85
Statice axillaris f. *attenuata* N.Terrac. 84
Statice axillaris Forssk. 84
Statice axillaris var. *attenuata* (N.Terrac.) Fiori 84
Statice axillaris var. *ellenbeckii* Engl. 85
Statice axillaris var. *glomerata* (N.Terrac.) Fiori 84
Statice baetica (Boiss.) Font Quer & Rothm. 29
Statice bahamensis Griseb. 73
Statice bahusiensis Fr. 78
Statice balansae Boiss. 86
Statice barulensis A.Bruni 79
Statice bayonnensis Gren. ex Boiss. 51
Statice beaumierana Coss. 88

Statice beaumierana Coss. ex Maire 88
Statice beaumieriana Coss. ex Maire 88
Statice beaumieriana f. *leucocalyx* Maire 89
Statice beaumieriana var. *akkensis* (Coss. ex Batt.) Maire 88
Statice beaumieriana var. *tripeaui* Maire 88
Statice behen Drejer 79, 80
Statice bella (Albov) Macloskie 26
Statice bellidifolia (Gouan) DC. 80
Statice bellidifolia Auct. ex Boiss. 50
Statice bellidifolia Bertol. 67
Statice bellidifolia Guss. 60
Statice bellidifolia Sm. 50
Statice bellidifolia var. *divaricata* DC. 80
Statice bellidifolia var. *prostrata* Beauverd 80
Statice besseriana Friv. ex Boiss. 43
Statice besseriana Schult. ex Rchb. 42
Statice bianorii Sennen & Pau 51
Statice bicolor Bunge 82
Statice billardierei Girard 51
Statice binervosa G.E.Sm. 51
Statice binervosa var. *dodarti* (Girard) Hook 57
Statice binervosa var. *dodarti* (Girard) Syme 57
Statice binervosa var. *intermedia* Syme 57
Statice binervosa var. *occidentalis* (J.Lloyd) Syme 51
Statice bocconei Lojac. 51
Statice boissieri Lafont 76
Statice bollei Webb ex Wangerin 51
Statice bonduellei T.Lestib. 89
Statice bonduellii T.Lestib. 89
Statice bonnetii Sennen 52
Statice bottendorfensis (A.G.Schulz) O.Schwarz 20
Statice bourgaei Webb ex Boiss. 87
Statice bovei Jaub. & Spach 84, 85
Statice bracteata Girard 5
Statice bracteosa Viv. 101
Statice brasiliense var. *angustata* A.Gray 77
Statice brasiliense var. *antarctica* Boiss. 76
Statice brasiliense var. *augustata* A.Gray 77
Statice brasiliense var. *uruguayensis* (Arechav.) Hauman 76
Statice brasiliensis A.Gray 77
Statice brasiliensis Boiss. 76
Statice brasiliensis Chapm. 77
Statice brasiliensis var. *angustata* A.Gray 77
Statice brasiliensis var. *patagonica* Hosseus 76
Statice brassicaefolia Webb & Berthel. 87
Statice brassicifolia var. *macroptera* (Webb & Berthel.) Burchard 88
Statice brassicifolia var. *typica* Stapf 87
Statice brassicifolia Webb & Berthel. 87
Statice braunii Bolle 74
Statice brunii Guss. ex A.Bruni 79
Statice brunneri Webb 74
Statice bubanii Girard 51
Statice bungeana Boiss. 82
Statice bungei Claus 76
Statice cabulica Boiss. 40
Statice caesia Girard 46
Statice caesia var. *major* Rouy 46
Statice caespitosa Cav. 29
Statice caespitosa d'Urv. 99
Statice caespitosa Ortega 23, 26
Statice caespitosa Poir. 26
Statice calcarae Tod. ex Janka 52
Statice calcarae Tod. ex Lojac. 52
Statice californica Boiss. 76
Statice callicoma C.A.Mey. 42
Statice cancellata Bernh. ex Bertol. 52
Statice cancellata Bernh. ex Mutel, A. 52
Statice cancellata var. *glabra* Boiss. 56
Statice cancellata var. *japygica* E.Groves 61
Statice canescens Host 23
Statice cantabrica (Boiss. & Reut. ex Willk. & Lange) P.Fourn. 24
Statice cantabrica subsp. *pubinervis* (Boiss.) P.Fourn. .. 36
Statice capensis L.Bolus 73
Statice capillifolia Poir. 35
Statice capitata Lam. 30
Statice capitella (Pau) Font Quer & Rothm. 25
Statice caput-alba Rothm. 29
Statice carinensis Chiov. 85
Statice carnosa Boiss. 75
Statice caroliniana var. *albiflora* Raf. 77
Statice caroliniana var. *cespitosa* Raf. 77
Statice caroliniana var. *longifolia* Raf. 77
Statice caroliniana var. *pumila* Raf. 77
Statice caroliniana var. *ramosissima* Raf. 77
Statice caroliniana Walter 77
Statice carpatha Rech.f. 52
Statice carpetana (Villar) Villar 38
Statice carthaginensis Rouy 53
Statice caryophyllacea Boiss. 3, 6
Statice caryophyllacea Boiss. & Hohen. ex Boiss. 6
Statice caspia Pollini 81
Statice caspia var. *kimmerica* Lipsky 81
Statice caspia var. *patens* (Fisch. ex Boiss.) Boiss. 81
Statice caspia var. *urumiensis* Bornm. 81
Statice caspia Willd. 80
Statice castellana (Boiss. & Reut. ex Leresche) Rothm. 25
Statice catalaunica Willk. & Costa 53
Statice catanensis Janka 72
Statice catanensis Tineo ex Lojac. 53
Statice cephalotes Aiton 35
Statice cephalotes Bertol. 34
Statice cephalotes Poir. 34
Statice cespitosa Poir. 26
Statice chazaliei H.Boissieu 74
Statice chilensis (Boiss.) Macloskie 26
Statice chilensis Phil. 78
Statice chrysocephala Regel 82
Statice chrysocoma Kar. & Kir. 82
Statice ciliata (Lange) Rothm. 57
Statice cinerea Poir. 68
Statice collina Griseb. 43
Statice collina Nyman 42
Statice companyonis Gren. & Billot 58
Statice confusa Gren. & Godr. 53
Statice confusa Rchb.f. 56
Statice confusa var. *intermedia* Daveau 56
Statice congesta Ledeb. 82
Statice conspicua Sims 44
Statice contortiramea Mabille 53
Statice coralloides Tausch 81
Statice corculum Christ 74
Statice cordata Desf. 72

Statice cordata G.E.Sm. ... 51
Statice cordata Guss. ... 51
Statice cordata Hayek ... 52
Statice cordata L. ... 54
Statice cordata Poir. ... 69
Statice cordata var. *genuina* Boiss. ... 51
Statice cordata var. *maior* Boiss. ... 55
Statice coriacea hort. ex Schult. ... 77
Statice coriacifolia Sennen ... 54
Statice coriaria Pall. ... 77
Statice coriaria Pall. ex M.Bieb. ... 77
Statice corinthiaca Boiss. & Heldr. ... 54
Statice corymbulosa (Coss.) Nyman ... 54
Statice corymbulosa Boiss. ... 54
Statice corymbulosa Coss. ... 55
Statice costae Willk. ... 54
Statice cosyrensis Guss. ... 55
Statice cosyrensis subsp. *cosyrensis* ... 55
Statice cosyrensis subsp. *melia* Nyman ... 72
Statice crispa L. ex B.D.Jacks. ... 74
Statice crispa Pers. ... 74
Statice crouanii Lenorm. ex Nyman ... 78
Statice cumana Ten. ... 55
Statice cumana var. *glabrescens* Lacaita ... 55
Statice cumana var. *tenuicula* (Tineo ex Cuss.) Boiss. ... 70
Statice cuneata hort. ex Schult. ... 98
Statice cuneata Sm. ex Link ... 89
Statice cuspidata Delort. ... 55
Statice cylindrica Steud. ... 85
Statice cylindrifolia Forssk. ... 85
Statice cymulifera Boiss. ... 55
Statice cymulifera subsp. *mauritii* Sennen ... 55
Statice cyrenaica Rouy ... 55
Statice cyrtostachya Girard ... 55
Statice czurjukiensis Klokov ... 79
Statice dalmatica C.Presl ... 42
Statice davaei (Samp.) Samp. ... 30
Statice davurica Pall. ... 82
Statice decipiens Ledeb. ... 81
Statice decumbens Boiss. ... 56
Statice delicatula Bég. & Vacc. ... 71
Statice delicatula f. *pomeliana* Rouy ... 66
Statice delicatula Girard ... 56
Statice delicatula subsp. *raddiana* (Boiss.) Rouy ... 67
Statice delicatula var. *minor* (Boiss.) Bég. & A.Vacc. ... 63
Statice delicatula var. *subrotundifolia* Bég. & A.Vacc. ... 70
Statice delicatula var. *tournefortii* (Girard) Boiss. ... 71
Statice delilei Aubouy ex Rouy ... 72
Statice densiflora Girard ... 59
Statice densiflora Guss. ... 56
Statice densiflora var. *humilis* Guss. ... 65
Statice densiflora var. *lusitanica* Daveau ... 63
Statice densiflora var. *obtusifolia* Guss. ... 56
Statice dentata hort. ex Schult. ... 98
Statice denticulata Bertol. ... 27
Statice denudata Regel & Körn. ... 45
Statice depressa Bubani ... 101
Statice dertosensis Font Quer & Rothm. ... 28
Statice desertorum Trautv. ... 43
Statice dianiae Pau ... 60
Statice dianthifolia Jaub. & Spach ... 18
Statice dianthodes Voss ... 19
Statice dichotoma Cav. ... 52, 55, 56, 59, 80
Statice dichotoma Duby ... 80
Statice dichotoma Guss. ... 52
Statice dichotoma Mutel, A. ... 57
Statice dichotoma Rchb. ... 59
Statice dichotoma Sm. ... 55
Statice dichotoma Willk. ... 56
Statice dichroantha Rupr. ... 82
Statice dickensonii Poit. ... 73
Statice dickinsonii hort. Angl. ex F.Cels ... 73
Statice dicksoniana hort. ex W.H.Baxter ... 98
Statice dictyoclada Boiss. ... 70
Statice dictyoclada subsp. *calcarae* (Tod. ex Janka) Nyman ... 52
Statice dictyoclada subsp. *pygmaea* (Tineo) Nyman ... 55
Statice dictyoclada var. *dubia* (Andrews ex Guss) Boiss. ... 57
Statice dictyoclada var. *pygmaea* Boiss. ... 55
Statice dictyoclada var. *reticulata* Boiss. ... 67
Statice dictyophora Tausch ... 56
Statice diegoi Sennen ... 70
Statice dielsiana Wangerin ... 82
Statice diffusa Laterr. ex Boiss. ... 80
Statice diffusa Pourr. ... 90
Statice divaricata Cav. ex Schult. ... 70
Statice dodartii Girard ... 57
Statice dodartii var. *humile* Girard ... 67
Statice doerfleri Halácsy ... 57
Statice donetzica Klokov ... 79
Statice doriae Sommier ... 57
Statice dregeana C.Presl ... 57
Statice drepanense Tineo ex Guss. ... 101
Statice drepanensis Tineo ex Guss. ... 79
Statice drepanostachya Ikonn.-Gal. ... 86
Statice dschungarica Regel ... 42
Statice dubia Andrews ex Guss. ... 57
Statice dubyeana Bubani ... 81
Statice dubyi Gren. & Godr. ... 81
Statice dufourii Girard ... 57
Statice durieui Girard ... 57
Statice duriuscula Girard ... 57
Statice duriuscula subsp. *catalaunica* (Willk. & Costa) Nyman ... 53
Statice duriuscula subsp. *laeta* Nyman ... 61
Statice duriuscula var. *catalaunica* Costa ... 53
Statice duriuscula var. *procera* Willk. ... 60
Statice duriuscula var. *valentina* Sennen & Pau ... 57
Statice ebracteata (Pomel) Maire ... 27
Statice ebracteata var. *laevis* (Maire) Maire ... 27
Statice ebusitana Font Quer ... 58
Statice echinata Schult. ... 27
Statice echinus L. ... 16
Statice echinus M.Bieb. ... 99
Statice echioides L. ... 48
Statice echioides subsp. *echioides* L. ... 48
Statice echioides subsp. *exaristata* (Murb.) Hayek ... 48
Statice echioides subsp. *exaristata* (Murb.) Pamp. ... 48
Statice echioides subsp. *exaristata* Murb. ... 48
Statice echioides var. *segobricensis* Pau ... 48
Statice effusa Boiss. ... 77
Statice elata Fisch. ex Spreng. ... 43
Statice elegans Coss. ex Nyman ... 46
Statice elongata Hoffm. ... 32
Statice emarginata Schur ... 78

Statice emarginata Willd. 58
Statice endlichiana Wangerin 77
Statice equisetina Boiss. 58
Statice equisetina var. *depauperata* Boiss. 51
Statice erinacea Boiss. 15
Statice erinacea Jaub. & Spach 7
Statice eriophylla (Willk.) Samp. 27
Statice eugeniae Sennen 58
Statice exaristata Murb. 48
Statice exima var. *flore-alba* hort. ex F.T.Hubb. 98
Statice exima var. *superba* hort. ex F.T.Hubb. 98
Statice eximia Schrenk 43
Statice eximia var. *turkestanica* Regel 43
Statice eximum var. *alba* hort. ex F.T.Hubb. 98
Statice fallax Coss. ex Wangerin 74
Statice fasciculata var. *pungens* Samp. 36
Statice fasciculata Vent. 36
Statice faustii Sennen & Mauricio 54
Statice ferganensis Ikonn.-Gal. 86
Statice ferox Jaub. & Spach 15
Statice ferulacea L. 90
Statice festucacea Jaub. & Spach 8
Statice filicaulis (Boiss.) Jahand. & Maire 27
Statice filicaulis Boiss. 27
Statice filicaulis var. *marizii* (Daveau) Samp. 29
Statice filicaulis var. *maroccana* (Pau & Font Quer) Maire 27
Statice fischeri Trautv. 82
Statice flagellaris Lojac. 58
Statice flexuosa L. 82
Statice flexuosa Less. 43
Statice flexuosa O.Fedtsch. & B.Fedtsch. 86
Statice flexuosa Sennen 53
Statice floribunda Lem. ex Huber 89
Statice florida Kitag. 82
Statice foliosa Penny ex W.H.Baxter 101
Statice fontqueri Pau 58
Statice formosa hort. ex E.Vilm. 35
Statice fortunei Lindl. 84
Statice fortuni Lindl. 84
Statice fradiniana Pomel 58
Statice franchetii Debeaux 83
Statice fraterna Sennen & Pau 58
Statice frederici Barbey 58
Statice frondosa Lojac. 101
Statice frutescens Lem. 87
Statice fruticans Webb ex Boiss. 87
Statice fruticosa Lepech. 75
Statice furfuracea Lag. 59
Statice furfuracea Rchb. 52
Statice gabrieli Bornm. 75
Statice gaditana (Boiss.) Jahand. & Maire 28
Statice gaditana (Boiss.) Samp. 28
Statice gaditana var. *chamaeropicola* (Pau) Maire 37
Statice gaditana var. *tingitana* (Boiss. & Reut.) Ball 38
Statice gaillardotii Lafont 76
Statice gavilae Sennen & Mauricio 54
Statice genistoides Jaub. & Spach 8
Statice giberti Sennen 59
Statice gibertii Sennen 59
Statice gilesii Hemsl. 41
Statice girardiana Guss. 59
Statice girardiana subsp. *costae* (Willk.) Nyman 54
Statice girardiana subsp. *girardiana* 59
Statice glauca (Boiss.) Cuatrec. 79
Statice glauca Less. 75
Statice glauca Pers. 69
Statice glauca Willd. ex Schult. 77
Statice globulariaefolia Desf. 67
Statice globulariaefolia var. *major* Boiss. 62
Statice globulariaefolia var. *minor* Boiss. 63, 70
Statice globulariifolia DC. 99
Statice globulariifolia Desf. 67, 99
Statice globulariifolia var. *glauca* Boiss. 56
Statice globulariifolia var. *major* Boiss. 62
Statice globulariifolia var. *minor* Boiss. 63
Statice globulariifolia Webb 99
Statice globulifera Boiss. & Heldr. ex Boiss. 86
Statice glomerata Tausch 59
Statice glumacea Jaub. & Spach 9
Statice gmelini f. *steiroclada* Wangerin 76
Statice gmelini M.Bieb. 79
Statice gmelini Rchb. 79
Statice gmelini subsp. *genuina* Wangerin 78
Statice gmelini subsp. *scoparia* Wangerin 78
Statice gmelini var. *genuina* Boiss. 77
Statice gmelini var. *grandis* Popov ex Andross. 78
Statice gmelini var. *laxiflora* Boiss. 78
Statice gmelini var. *steiroclada* Trautv. 76
Statice gmelini var. *typica* Trautv. 77
Statice gmelini Willd. 77, 78
Statice gmelinii subsp. *tomentella* (Boiss.) Wangerin 79
Statice gmelinii var. *laxiflora* Boiss. 78
Statice gmelinii var. *lilacina* (Boiss. & Balansa) Boiss. 86
Statice gmelinii var. *limonioides* Wangerin 78
Statice gmelinii var. *scoparia* (Pall. ex Willd.) Schmalh. 77
Statice gmelinii var. *scoparia* Trautv. 78
Statice gmelinii var. *tomentella* (Boiss.) Trautv. 79
Statice gmelinii W.D.J.Koch 76
Statice gmelinii Willd. 78
Statice gobica Ikonn.-Gal. 83
Statice gomezi-jordanae Sennen 54
Statice gougetiana Girard 59
Statice gougetiana Reverch. 101
Statice gracilis Fisch. ex Boiss. 76
Statice gracilis O.Fedtch. & B.Fedtsch. 86
Statice gracilis Tineo 55
Statice graeca Boiss. 59
Statice graeca Poir. 59
Statice graeca var. *hyssopifolia* (Girard) Boiss. 68
Statice graeca var. *microphylla* Boiss. 59
Statice graeca var. *palmaris* (Sm.) Hay 64
Statice graeca var. *sieberi* (Boiss.) Boiss. 69
Statice graminifolia Aiton 43
Statice graminifolia Besser 42
Statice graminifolia var. *desertorum* Regel 43
Statice grandiflora hort. Viln. ex Schult. 98
Statice griffithii Aitch. & Hemsl. 41
Statice guaicuru (Molina) I.M.Johnst. 78
Statice guaicuru (Molina) Larrañaga 78
Statice gummifera Durieu ex Boiss. & Reut. 59
Statice gummifera f. *corymbulosa* (Coss.) Batt. 51
Statice gummifera subsp. *cymulifera* 51
Statice gummifera subsp. *cymulifera* (Boiss.) Batt. 51
Statice gummifera var. *corymbulosa* Coss. 54
Statice gummifera var. *cymulifera* (Boiss.) Batt. 55

Statice gussoneana Steud. 69
Statice gussonei Tineo ex Lojac. 71
Statice halfordii Hovey 87
Statice halleri Garcke 20
Statice halochrysa Fisch. ex Boiss. 82
Statice heldreichii Halácsy 43
Statice hieronymi Sennen 70
Statice hirsuta C.Presl 89
Statice hirta (Willd.) Rothm. 28
Statice hirta (Willd.) Steud. 28
Statice hirta subsp. *baetica* (Boiss.) Rothm. 29
Statice histrix Jaub. & Spach 17
Statice hoeltzeri Regel 83
Statice hohenackeri Jaub. & Spach 9
Statice hohenackeri Ledeb. 9
Statice holfordii hort. 87
Statice horrida Girard 9
Statice humboldtii Bolle 74
Statice humilis (Mill.) C.E.Salmon 78
Statice humilis Link 29
Statice humilis var. *odorata* (Samp.) Samp. 29
Statice hybrida Mont. ex J.Lloyd 64
Statice hypanica Klokov 78
Statice hyssopifolia Girard 68
Statice hystrix Jaub. & Spach 17
Statice iconia Boiss. & Heldr. 81
Statice ifniensis Caball. 74
Statice imbricata Webb ex Girard 87
Statice inarimensis Guss. 60
Statice incana Becker 44
Statice incana d'Urv. ex Boiss. 43
Statice incana L. 43
Statice incana Ledeb. 42
Statice incana M.Bieb. 42
Statice incana var. *coccinea* hort. ex F.T.Hubb. 98
Statice incana var. *hybrida* hort. ex F.T.Hubb. 42
Statice incana var. *hybrida-nana* hort. ex F.T.Hubb. 98
Statice incana Vis. 42
Statice insigne var. *rossmaessleri* Willk. 47
Statice insignis Coss. 46
Statice interior Raup 32
Statice intermedia Guss. 60
Statice isernii (Vicioso & Beltrán) Font Quer & Rothm. 29
Statice jankae Lojac. 61
Statice japonica Siebold & Zucc. 84
Statice jaubertii Girard 7
Statice jovibarba Webb ex Boiss. 75
Statice juncea (Wallr.) F.T.Hubb. ex L.H.Bailey 99
Statice juncea (Wallr.) Samp. 19
Statice juncea F.T.Hubb. 28
Statice juncea race eriophylla (Willk.) Samp. 27
Statice juncea Tatarinow ex Wlangali 83
Statice juniperifolia 9, 20, 29, 38
Statice juniperifolia Boiss. 38
Statice juniperifolia Pall. ex Steud. 9
Statice juniperifolia Samp. 20
Statice juniperifolia Vahl 29
Statice juniperina Willd. ex Steud. 7
Statice karelinii Stschegl. 10
Statice kaschgarica Rupr. 83
Statice kaufmanniana Regel 43
Statice klementzii Ikonn.-Gal. 83
Statice kossmatii R.Wagner & Vierh. 85
Statice kotschyi Jaub. & Spach 11
Statice kraussiana Buchinger ex Boiss. 61
Statice L. 18, 45
Statice labradorica (Wallr.) F.T.Hubb. & S.F.Blake 34
Statice labradorica f. *glabriscapa* S.F.Blake 34
Statice labradorica f. *pubiscapa* S.F.Blake 34
Statice labradorica var. *submutica* S.F.Blake 34
Statice lacaitae Villar 22
Statice lachnolepis (Pomel) Maire 27
Statice lacostei Danguy 83
Statice laeta Ball 47
Statice laeta Moris 61
Statice laeta Salisb. 74
Statice laeta subsp. *insularis* Bég. & Landi 60
Statice lanceolata Edgew. 85
Statice lanceolata Hoffmanns. & Link 61
Statice lanceolata hort. ex E.Vilm. 76
Statice lanceolata Rchb. 51
Statice laneolata Hoffmanns. & Link 61
Statice langeana (Henriq.) Rothm. 36
Statice langei (Boiss.) Rothm. 29
Statice latifolia Sm. 77
Statice latifolia var. *alba* hort. ex F.T.Hubb. 98
Statice latissima Kar. & Kir. 81
Statice laxiflora (Boiss.) Novopokr. 78
Statice laxissima Rouy 67
Statice lefroyi Hemsl. 77
Statice legrandii Gaut. & Timb.-Lagr. 61
Statice leonis Sennen 29
Statice lepidorachis Pomel 55
Statice leptoloba Michelson 83
Statice leptoloba Regel 83
Statice leptoloba var. *subaphylla* Regel 83
Statice leptophylla Schrenk 75
Statice leptophylla var. *iranica* Bornm. 75
Statice leptostachya Boiss. 91
Statice leptostachya f. *pomeliana* Rouy 91
Statice leptostachya var. *scapis-filiformibus* Aitch. 91
Statice leptostachys Pomel 66
Statice lepturoides Jaub. & Spach 11
Statice lespinassi Lafont 78
Statice letourneuxii Coss. ex Batt. 62
Statice leucacantha Jaub. & Spach 11
Statice leucantha Pouzolz 29
Statice leucocephala (Salzm. ex W.D.J.Koch) F.T.Hubb. 29
Statice leucocoleum Stapf & Wettst. ex Stapf 101
Statice lilacina Boiss. & Balansa 86
Statice limbata (Small) K.Schum. 78
Statice limonia St.-Lag. 79
Statice limonioides Bernh. ex Link 76
Statice limonium Bigelow 77
Statice limonium L. 79
Statice limonium Pall. 76, 77
Statice limonium Rattan 76
Statice limonium subsp. *aggregata* (Rouy) Rouy 79
Statice limonium subsp. *angustifolia* (Tausch) Rouy 76
Statice limonium subsp. *bahusiensis* (Fr.) Hook.f. 78
Statice limonium subsp. *bahusiensis* Syme 78
Statice limonium subsp. *behen* Syme 79, 80
Statice limonium subsp. *rariflora* (Drejer) Hook.f. 78
Statice limonium subsp. *remotiflora* (Rouy) Rouy 79
Statice limonium subsp. *serotina* (Rchb.) Nyman 79
Statice limonium Thunb. 84

Statice limonium var. *alba* hort. ex F.T.Hubb. 80
Statice limonium var. *behen* Rouy 80
Statice limonium var. *bellidifolia* Gouan 80
Statice limonium var. *californica* (Boiss.) A.Gray 76
Statice limonium var. *carolinianum* (Walter) A.Gray 77
Statice limonium var. *genuina* Boiss. 80
Statice limonium var. *longidentata* (Lafont) Rouy 79
Statice limonium var. *macroclada* Boiss. 76
Statice limonium var. *minor* Roth 80
Statice limonium var. *pseudolimonium* Rouy 80
Statice limonium var. *puberula* Regel 79
Statice limonium var. *scanica* Fries 80
Statice limonium var. *typica* Rouy 80
Statice limonoides Biv. ex Link 78
Statice linearifolia Laterr. 30
Statice linearifolia Loisel. 32
Statice lingua Pomel 55
Statice linifolia Drege ex Boiss. 65
Statice linifolia L.f. 62
Statice linifolia var. *aretiifolia* Boiss. 61
Statice linifolia var. *brachyphylla* Boiss. 62
Statice linifolia var. *maritima* Eckl. & Zeyh. ex Boiss. 62
Statice linifolia var. *robusta* C.H.Wright 62
Statice littoralis (Willd.) Steud. 30
Statice littoralis P.Fourn. 30
Statice littoralis Poir. 30
Statice littoralis subsp. *filicaulis* (Boiss.) P.Fourn. 27
Statice littoralis subsp. *juncea* P.Fourn. 28
Statice littoralis subsp. *littoralis* (Willd.) Steud. 30
Statice littoralis var. *davaei* Samp. 30
Statice lobata L.f. 89
Statice longearistata Font Quer & Rothm. 101
Statice longearistata var. *cuspidata* Faure & Maire 22
Statice longiaristata (Boiss. & Reut.) Maire 39
Statice longidentata Lafont 79
Statice longifolia Thunb. 73
Statice lusitanica Poir. 34
Statice lychnidifolia Girard 50
Statice lychnidifolia Porta 51
Statice lychnidifolia var. *corymbosa* Boiss. 64
Statice lycopodioides Girard 12
Statice lyrata M.Bieb. 92
Statice macloviana (Cham.) Macloskie 26
Statice macphersonii F.Muell. 92
Statice macrophylla (Boiss. & Reut.) Font Quer & Rothm. 30
Statice macrophylla (Boiss. & Reut.) Rothm. 30
Statice macrophylla var. *atrocoerulea* J.R.Duncan & V.C.Davies 87
Statice macrophylla var. *sinuata* Boiss. 87
Statice macrophylla Willd. ex Spreng. 87
Statice macroptera Webb & Berthel. 88
Statice macrorrhabdos Boiss. 41
Statice macrorrhabdos var. *thomsoni* C.B.Clarke 42
Statice macrorrhabdos var. *thomsonii* C.B.Clarke 42
Statice macrorrhiza Ledeb. 81
Statice maeotica Klokov 79
Statice majellensis (Boiss.) F.T.Hubb. 28
Statice majellensis subsp. *eumajellensis* P.Fourn. 37
Statice majellensis subsp. *majellensis* 28
Statice majellensis subsp. *malinvaudii* (H. J. Coste & Soulié) P.Fourn. 37
Statice major Garsault 21
Statice major race gaditana (Boiss.) Samp. 28
Statice major Samp. 35
Statice malinvaudii (H.J.Coste & Soulié) P.Fourn. 30
Statice manricarum Bolle 47
Statice manricarum Bolle ex Linding. 47
Statice manricarum Bolle ex Pitard & Proust 47
Statice manriqueorum Bolle ex Linding. 47
Statice manriquorum Bolle ex Pitard & Proust 47
Statice maravignae Tineo ex Lojac. 101
Statice marginata Schott ex Steud. 76
Statice maritima Lam. 79
Statice maritima Mill. 30
Statice maritima var. *elongata* (Hoffm.) Samp. 32
Statice maritima var. *longeana* (Henriq.) Samp. 36
Statice maritima var. *majuscula* Samp. 33
Statice maritima var. *pubigera* (Desf.) Samp. 36
Statice mauritanica (Wallr.) F.T.Hubb. 34
Statice mauritanica var. *safiensis* Maire 37
Statice mauritanica var. *soloitana* Maire 37
Statice maurocenia hort. Par. ex Dum.Cours. 98
Statice maurocordatae Schweinf. & Volkens 85
Statice melanantha Boiss. 12
Statice melia Nyman 72
Statice membranacea Czern. ex Trautv. 76
Statice meyeri Boiss. 78
Statice micrantha Lafont 101
Statice minuta Desf. 59
Statice minuta f. *puberula* C.E.Salmon 63
Statice minuta Falk 101
Statice minuta L. 63
Statice minuta Moritzi ex Rchb. 48
Statice minuta Ten. ex Nyman 63
Statice minuta Tomm. ex Nyman 52
Statice minuta var. *acutifolia* Boiss. 48
Statice minuta var. *calcarae* (Tod. ex Janka) Fiori 52
Statice minuta var. *cancellata* (Bernh. ex Bertol.) Fiori 52
Statice minuta var. *cumana* (Ten.) Fiori 55
Statice minuta var. *dissitiflora* Boiss. 56
Statice minuta var. *inarimensis* (Guss.) Fiori 60
Statice minuta var. *microphylla* Boiss. 66
Statice minuta var. *multiformis* Martelli 63
Statice minuta var. *pubescens* Boiss. 52
Statice minuta var. *remotispicula* (Lacaita) Fiori 67
Statice minuta var. *sommierana* Fiori 63
Statice minuta var. *sommieriana* Fiori 69
Statice minuta Vis. 54
Statice minuta Willk. 101
Statice minutiflora Guss. 63
Statice minutiflora subsp. *minutiflora* 63
Statice minutiflora subsp. *panormitana* (Tod.) Nyman 65
Statice molesii Sennen 54
Statice mongolica Fisch. ex Girard 101
Statice monopetala L. 45
Statice monrealensis Pau 50
Statice monregalensis Pau 50
Statice montana Mill. 20
Statice montana Soleirol ex Boiss. 30
Statice montana var. *alba* F.T.Hubb. 98
Statice mouretii Pit. 89
Statice mucosa Salzm. ex Boiss. 64
Statice mucronata L.f. 74
Statice mucronulata H.Lindb. 63
Statice muelleri (A.Huet) P.Fourn. 35

Statice multiceps Pomel 63
Statice multiramea Sennen 53
Statice myriantha Schrenk ex Fisch. & C.A.Mey. 81
Statice nana Penny ex G.Don 101
Statice nashii (Small) Wangerin 77
Statice nebrodensis Guss. 24
Statice nebrodensis var. *duriaei* (Boiss.) Samp. 27
Statice neglecta (Girard) Samp. 30
Statice neglecta Andrz. 101
Statice nicotrae Lojac. 101
Statice nigricans Lafont 79
Statice nogalensis Chiov. 85
Statice nuda Boiss. & Buhse 83
Statice nuda Grossh. 82
Statice oblongifolia Kotov 49
Statice obovata Ledeb. 78
Statice obtusa Dum.Cours. 54
Statice occidentalis J.Lloyd 51
Statice ochrantha Kar. & Kir. 44
Statice ocimifolia Poir. 101
Statice ocimifolia Schult. 101
Statice ocymifolia Poir. 64
Statice ocymifolia subsp. *ocymifolia* 64
Statice ocymifolia var. *bellidifolia* (Sibth. & Sm.) Boiss. 50
Statice oleifolia (Mill.) Scop. 97
Statice oleifolia Host 81
Statice oleifolia Pourr. ex DC. 72
Statice oleifolia Sm. 72
Statice oleifolia var. *densiflora* (Guss.) Fiori 56
Statice oleifolia var. *fradiniana* (Pomel) Batt. 58
Statice oleifolia Willd. 72
Statice oligantha Boiss. 13
Statice oliveriana Andrz. ex Boiss. 68
Statice olivieri Jaub. & Spach 13
Statice opulenta Lojac. 64
Statice ornata Ball 47
Statice otolepis Schrenk 81
Statice ovalifolia Poir. 64
Statice ovalifolia var. *balleariea* Wanger 51
Statice ovalifolia var. *lanceolata* Rouy 61
Statice ovalifolia var. *major* Rouy 64
Statice ovalifolia var. *minor* Boiss. 64
Statice ovalifolia var. *nana* Rouy 64
Statice ovalifolia var. *normalis* Rouy 64
Statice ovalifolia var. *paniculata* Rouy 64
Statice ovalifolia var. *pyramidata* (Lowe) Menezes 62
Statice owerinii Boiss. 45
Statice oxylepis Boiss. 56
Statice palmaris Sm. 64
Statice palmyrensis Post 75
Statice panormitana Tod. 65
Statice papillata Webb & Berthel. 74
Statice parvifolia Tineo 65
Statice patagonica Speg. 76
Statice patens Fisch. ex Boiss. 81
Statice pauciflora Jaub. & Spach 17
Statice pauі Cámara & Sennen 65
Statice paui Vicioso 47
Statice paulayana Vierh. 85
Statice pectinata Aiton 74
Statice pectinata var. *corculum* Webb & Berthel. 74
Statice pectinata var. *divaricata* Pit. 74
Statice pectinata var. *incompta* Webb & Berthel. 74
Statice pectinata var. *solandri* Webb & Berthel. 74
Statice pedicellata Wallr. ex Boiss. 65
Statice peregrina P.J.Bergius 73
Statice perezii Stapf 88
Statice perfoliata C.A.Mey. ex Boiss. 81
Statice perfoliata var. *reniformis* Boiss. 81
Statice petteriana (C.Presl) Degen 24
Statice pigadiensis Rech.f. 65
Statice pinifolia Brot. 35
Statice pinifolia var. *capillifolia* Samp. 35
Statice planifolia (Syme) Druce 31
Statice plantaginea All. 21
Statice plantaginea Guss. 34
Statice plantaginea proles rigida (Wallr.) Samp. 19
Statice plantaginea subsp. *choulettiana* (Pomel) Maire 25
Statice plantaginea subsp. *leucantha* (Boiss.) Maire 22
Statice plantaginea subsp. *medians* Maire 22
Statice plantaginea var. *alba* F.T.Hubb. 97
Statice plantaginea var. *atlantica* (Pomel) Maire 22
Statice plantaginea var. *barbata* Maire 26
Statice plantaginea var. *brachylepis* (Batt.) Maire 26
Statice plantaginea var. *cuspidata* (Faure & Maire) Maire 22
Statice plantaginea var. *djurdjurae* Maire 26
Statice plantaginea var. *gigantea* F.T.Hubb. 98
Statice plantaginea var. *grandiflora* F.T.Hubb. 98
Statice plantaginea var. *leucantha* (Boiss.) F.T.Hubb. 19
Statice plantaginea var. *masguindalii* (Pau) Maire 34
Statice plantaginea var. *microcephala* Maire 26
Statice plantaginea var. *minor* Gaudin 20
Statice plantaginea var. *rosea* F.T.Hubb. 98
Statice plantaginea var. *rubra* F.T.Hubb. 98
Statice plantaginea var. *splendens* F.T.Hubb. 98
Statice plantaginea var. *subcuspidata* Maire 22
Statice plantaginea var. *zaianica* (Emberger, L. & Maire) Maire 25
Statice plantaginiflora Jaub. & Spach 92
Statice plumosa Phil. 40
Statice polianthemum Neck. 101
Statice pomeliana Rouy 66
Statice potaninii Ikonn.-Gal. 83
Statice preauxii Webb & Berthel. 88
Statice preciosa Tchertkoff 101
Statice profusa hort. 88
Statice prolifera d'Urv. 66
Statice pruinosa Cav. ex Willk. & Lange 46
Statice pruinosa Forssk. ex Schult. 59
Statice pruinosa L. 47
Statice pruinosa var. *hirtiflora* Cavara & Grande 47
Statice pruinosa Webb & Berthel. ex Boiss. 74
Statice pseudarmeria Murray 35
Statice pseud-armeria Murray 35
Statice pseudoarmeria Brot. 35
Statice pseudoarmeria Cav. 28
Statice pseudoarmeria var. *alba* F.T.Hubb. 97
Statice pseudoarmeria var. *grandiflora* F.T.Hubb. 97
Statice pseudoarmeria var. *rubra* F.T.Hubb. 97
Statice pseudoarmeria var. *splendens* (hort.) F.T.Hubb. 97
Statice pseudolimonium Rchb. 80
Statice psiloclada Boiss. 67
Statice psiloclada var. *albida* (Guss.) Boiss. 49
Statice psiloclada var. *genuina* Boiss. 65

Statice psiloclada var. *gracilis* Boiss. 60
Statice psiloclada var. *intermedia* (Guss.) Boiss. 60
Statice psiloclada var. *spathulaefolia* Lojac. 60
Statice puberula var. *bourgaei* (Webb ex Boiss.) Stapf 87
Statice puberula Webb ex Lindl. 88
Statice pubescens (Sowerby) Druce 31
Statice pubescens DC. 54
Statice pubescens Rchb. 63
Statice pubescens Salis 29
Statice pubescens Sm. ex Schult. 33
Statice pubescens W.D.J.Koch 52
Statice pubigera Desf. 36
Statice pubinervis (Boiss.) Vines & Druce 36
Statice pulchella Gray 31
Statice pumila Gasp. ex Lojac. 101
Statice pungens Brot. 36
Statice pungens Jaub. & Spach 7
Statice punicea Rendle 26
Statice purpurata L. 68, 73
Statice purpurata var. *genuina* Boiss. 73
Statice purpurata var. *longifolia* (Thunb.) Boiss. 73
Statice purpurata Willd. 73
Statice purpurea (W.D.J.Koch) W.D.J.Koch 33
Statice pusilla (Salis) P.Fourn. 30
Statice pycnantha K.Koch 86
Statice pygmaea Tineo 55
Statice pyramidata Lowe 62
Statice pyrrholepis Pomel 56
Statice quichiotis Gonz.Albo 36
Statice racemosa Lojac. 67
Statice raddiana Boiss. 67
Statice ramosissima Poir. 67
Statice rarida Vis. 99
Statice rariflora Drejer 78
Statice recurva (C.E.Salmon) C.E.Salmon 67
Statice rediviva Svent. 88
Statice rediviva var. *pilosa* Svent. 88
Statice reinwardtii hort. ex Lanza 98
Statice remotiflora Rouy 79
Statice remotispicula Lacaita 67
Statice reniformis Girard 81
Statice reticulata Gouan 97, 99
Statice reticulata Hook. 51
Statice reticulata L. 67
Statice reticulata M.Bieb. 99
Statice reticulata Sievers ex Boiss. 81
Statice rigida (Wallr.) Samp. 19
Statice roborowskii Ikonn.-Gal. 83
Statice rorida Sibth. & Sm. 68
Statice rorida Vis. 52, 99
Statice rosea (Boiss.) Boiss. 7
Statice rosea Pall. 82
Statice rosea Sm. 73
Statice rouyana (Daveau) Samp. 36
Statice rubella S.G.Gmel. 43
Statice ruizii Font Quer 68
Statice rumelica (Boiss.) Degen & Dörfl. 25
Statice rumelica var. *tempskyana* Degen & Dörfl. 25
Statice rumicifolia Svent. 88
Statice rupicola Badarò ex Rchb. 48
Statice ruscinonensis (Girard) P.Fourn. 37
Statice rytidophylla Hook. 73
Statice sabulosa Fourr. 19
Statice salicorniacea F.Muell. 90
Statice salmonis Sennen & Elías 68
Statice salsuginosa Boiss. 70
Statice salsuginosa var. *hirtula* Willk. 70
Statice sanjurjensis Sennen 54
Statice sanjurjoi Sennen & Mauricio 54
Statice sareptana A.K.Becker 79
Statice sartorii (Boiss.) Nyman 44
Statice scabra Drege ex Boiss. 68
Statice scabra Krauss 54
Statice scabra Pall. ex Boiss. 34
Statice scabra Pers. 45
Statice scabra Thunb. 68
Statice scabrida C.Presl 58
Statice scanica Warm. 79
Statice schirasiana Boiss. 15
Statice schrenkiana Fisch. & C.A.Mey. 82
Statice scoparia C.A.Mey. ex Boiss. 78
Statice scoparia hort. ex E.Vilm. 99
Statice scoparia M.Bieb. 78
Statice scoparia Pall. ex Willd. 78, 79
Statice scoparia Rchb. ex Nyman 79
Statice scopoliana Bertol. 59
Statice scorpius Jaub. & Spach 15
Statice scorzonerifolia Link 19
Statice sebkarum f. *glomerata* Batt. 55
Statice sebkarum f. *macrolepis* Batt. 55
Statice sebkarum Pomel 69
Statice sebkarum var. *mauritii* Sennen 69
Statice sect. *Circinaria* Boiss. 72
Statice sect. *Ctenostachys* Boiss. 73
Statice sect. *Goniolimon* (Boiss.) Hook. 42
Statice sect. *Jovibarba* Boiss. 75
Statice sect. *Myriolepis* Boiss. 90
Statice sect. *Plathymenium* Boiss. 81
Statice sect. *Polyarthrion* Boiss. 46
Statice sect. *Pteroclados* (Spach) Boiss. 87
Statice sect. *Pterocladus* (Spach) Boiss. 87
Statice sect. *Schizhymenium* Boiss. 47
Statice sect. *Schizopetalum* Boiss. 41
Statice sect. *Siphonantha* Boiss. 48
Statice sect. *Tropidice* Griseb. 42
Statice secundiramea Lojac. 69
Statice sedoides Regel 82
Statice semenovii Herder 83
Statice serbica (Vis.) Nyman 44
Statice serotina Rchb. 79
Statice setigera Bubani 90
Statice sewerzowii (Herder) Regel 44
Statice sewerzowii var. *alatavica* Regel 44
Statice sewerzowii var. *typica* Regel 44
Statice sibirica (Turcz. ex Boiss.) Ledeb. 33
Statice sibirica Dum.Cours. 48
Statice sibthorpiana Guss. 69
Statice sicula Tineo ex Lojac. 62
Statice sieberi Boiss. 69
Statice simplex (Pomel) Santa 37
Statice sinensis Girard 83
Statice sinensium Gand. 82
Statice sinuata L. 89
Statice sinuata var. *candidissima* hort. 89
Statice sinuata var. *integrifolia* Boiss. 89
Statice sinuata var. *subglabra* H.Lindb. 89

Statice sisymbriifolia Jaub. & Spach 92
Statice smithii Ten. 72
Statice sogdiana Popov 86
Statice sokotrana Vierh. 85
Statice soleirolii Duby 38
Statice somalorum Vierh. 85
Statice sommieriana (Fiori) Sommier 69
Statice spachii Girard 15, 17
Statice spathulata Desf. 69
Statice spathulata Heldr. ex Nyman 68
Statice spathulata Hook. 51
Statice spathulata Jan ex Nyman 56
Statice spathulata Nyman 101
Statice spathulata Sieber ex Boiss. 69
Statice spathulata var. *emarginata* (Willd.) Boiss. 58
Statice spathulata var. *spathulata* 69
Statice spathulata Willd. ex Boiss. 69
Statice speciosa Forssk. 43
Statice speciosa L. 44
Statice speciosa var. *crispa* Regel 43
Statice speciosa var. *genuina* Kryl. 44
Statice speciosa var. *lanceolata* Regel 42
Statice speciosa var. *lepidota* Regel 43
Statice speciosa var. *multicaulis* Krylov 43
Statice speciosa var. *stricta* Regel 44
Statice speciosa var. *typica* Regel 44
Statice spectabilis Svent. 88
Statice spicata Hohenacker 92
Statice spicata var. *foliis-subintegris* Aitch. 92
Statice spicata var. *glabra* Regel 92
Statice spicata Willd. 92
Statice spinulosa (Boiss.) Maire 38
Statice spinulosa Janka 79
Statice spiridonowii B.Fedtsch. 86
Statice splendens Lag. & Rodr. 38
Statice squamata Poir. 48
Statice stephani Sennen 50
Statice stocksii Boiss. 85
Statice stricta Gueldenst. 101
Statice subg. Armeriastrum Jaub. & Spach 2
Statice subg. Limonium Spach 46
Statice subg. Psylliostachys Jaub. & Spach 91
Statice subg. Pterocladus Spach 87
Statice subg. Schizopetalum (Boiss.) Melchior 41
Statice subsect. *Genuinae* Boiss. 75
Statice subsect. *Hyalolepideae* Boiss. 47, 80
Statice subsect. *Nobiles* Boiss. 87
Statice subsect. *Odontolepideae* Boiss. 88
Statice subsect. *Pruinosae* Batt. 47
Statice subsect. *Sarcophyllae* Boiss. 75, 84
Statice subsect. *Sphaerostachys* Boiss. 86
Statice suffruticosa L. 75
Statice suffruticosa var. *carnosa* (Boiss.) Kusn. 75
Statice suffruticosa var. *leptophylla* Trautv. 75
Statice suffruticosa var. *typica* Trautv. 75
Statice superba Regel ex F.Haage & M.Schmidt 91
Statice superba var. *flora-alba* Benary 91
Statice supina Girard 70
Statice suworowii Regel 92
Statice suworowii var. *alba* hort. 92
Statice tatarica L. 44
Statice tatarica var. *angustifolium* hort. ex F.T.Hubb. 42
Statice tatarica var. *nana* hort. ex hort. 98
Statice tatarica var. *trigonoides* Poir. 43
Statice tatarica var. *typica* Regel 44
Statice taubertii Hausskn. 72
Statice taxanthema Schult. 82
Statice tchefouensis Gand. 83
Statice tenella Turcz. 84
Statice tenoreana Guss. 70
Statice tenuicula Tineo ex Guss. 70
Statice tenuifolia Bertol. ex Moris 70
Statice tenuifolia Jaub. & Spach 9
Statice terekiensis Gueldenst. 44
Statice teretifolia Baker ex Oliv. 85
Statice tetragona Drège ex Boiss. 101
Statice tetragona Thunb. 84
Statice thouinii Viv. 89
Statice thymoides Girard 47
Statice tineoi J.Woods 52
Statice tineoi Lojac. 71
Statice tineoi var. *gracilis* (Tineo) J.Woods 55
Statice tineoi var. *parvifolia* (Tineo) J.Woods 65
Statice tineoi var. *pygmaea* (Tineo) J.Woods 55
Statice tomentella Boiss. 17, 79
Statice tomentella subsp. *sareptana* Nyman 79
Statice tournefortii Girard 71
Statice tournefortii Jaub. & Spach 17
Statice tracyi Gand. 77
Statice tragacanthina Jaub. & Spach 17
Statice transmontana Samp. 38
Statice transwalliana Pugsley 71
Statice tremolsii Rouy 71
Statice tremolsii var. *delilei* (Aubouy) Rouy 72
Statice trigona Pall. 44
Statice trigonoides Boiss. 43
Statice trigonoides Pall. 43
Statice trinervia Schult. 101
Statice tripteris Poir. 89
Statice triquetra Boeber 44
Statice tschurjukiensis Klokov 79
Statice tuberculata Boiss. 47
Statice tubiflora Delile 48
Statice tubiflora Sieber ex Schult. 47
Statice tubiflora var. *maroccana* Batt. & Trab. 48
Statice tubiflora var. *zanonii* Pamp. 48
Statice tunetana Barratte & Bonnet 71
Statice turkestanica Gand. 91
Statice ulicina Willd. ex Schult. 17
Statice undulata Bory & Chaub. 38
Statice urgellensis Sennen 53
Statice uruguayensis Arechav. 76
Statice valentina Sennen & Pau 57
Statice varia Hance 82
Statice variabilis Salisb. 35
Statice velutina (Welw. ex Boiss. & Reut.) Samp. 39
Statice venusta Fenzl ex Boiss. 18
Statice verrucosa Willd. ex Boiss. 59
Statice vestita C.E.Salmon 72
Statice viciosoi Pau 72
Statice villosa (Girard) Jahand. & Maire 39
Statice villosa var. *zaianica* Emberger, L. & Maire 25
Statice viminea Schrad. ex Hornem. 72
Statice virgata Herb. ex Nyman 53
Statice virgata Orph. ex Nyman 69
Statice virgata var. *divaricata* Rouy 57

Statice virgata var. *gracillima* J.J.Rodr. 101
Statice virgata var. *macroclada* Pau 60
Statice virgata var. *minuta* (L.) Knoche........................ 63
Statice virgata var. *pumila* Boiss.................................. 48
Statice virgata var. *reticulata* Boiss. 57
Statice virgata var. *tenia* Heldr. 72
Statice virgata Willd.. 72
Statice vulgaris Druce 99
Statice vulgaris Hill... 33, 99
Statice vulgaris Sweet.. 33
Statice vulgaris var. *elongata* (Hoffm.) Samp. 32
Statice vulgaris var. *langeana* (Henriq.) Samp. 36
Statice vulgaris var. *majuscula* (Samp.) Pires de Lima .. 33
Statice vulgaris var. *pubigera* (Desf.) Pires de Lima 36
Statice welwitschii (Boiss.) F.T.Hubb. ex L.H.Bailey 39
Statice welwitschii (Boiss.) Samp. 39
Statice welwitschii var. *berlengensis* (Daveau) Samp.... 22
Statice welwitschii var. *cinerea* (Boiss. & Reut.) Samp.. 39
Statice welwitschii var. *platyphylla* (Daveau) Samp. 35
Statice willdenovii Poir.. 50
Statice willdenowiana Herb. ex Nyman 56
Statice willdenowiana Rchb. 57
Statice willdenowii Loisel. 50
Statice wrightii Hance 84
Statice xipholepis Baker 85
Staticeae Bartl.. 2
Taxanthema aegyptiaca (Pers.) Sweet 89
Taxanthema aegyptiacum (Pers.) Sweet 89
Taxanthema alata (Willd.) Sweet 89
Taxanthema armeria Neck. 32
Taxanthema attenuata R.Br. 85
Taxanthema auriculifolia (Vahl) Sweet 50
Taxanthema australis R.Br.................................... 45, 82
Taxanthema bellidifolia (Gouan) Sweet........................ 80
Taxanthema caroliniana (Walter) Sweet 77
Taxanthema carolinianum (Walter) Sweet 77
Taxanthema caspia (Willd.) Sweet 80
Taxanthema conspicua (Sims) Sweet............................ 44
Taxanthema cordata (L.) Sweet.................................... 54
Taxanthema dichotoma (Cav.) Sweet 56
Taxanthema dichotomum (Cav.) Sweet 56
Taxanthema echioides Sweet 48
Taxanthema elata (Fisch. ex Spreng.) Sweet 43
Taxanthema emarginata (Willd.) Sweet 58
Taxanthema ferulacea (L.) Sweet 90
Taxanthema flexuosa (L.) Sweet 82
Taxanthema globulariifolia Sweet 67
Taxanthema gmelini Sweet... 77
Taxanthema graminifolia (Aiton) Sweet 43
Taxanthema incana (L.) Sweet...................................... 43
Taxanthema latifolia (Sm.) Sweet................................. 77
Taxanthema limonium (L.) Sweet 79
Taxanthema macrophylla (Willd. ex Spreng.) Sweet 87
Taxanthema minuta (L.) Sweet 63
Taxanthema monopetala (L.) Sweet 45
Taxanthema mucronata (L.f.) Sweet............................. 74
Taxanthema Neck. ex R.Br. 45
Taxanthema oleifolia (Mill.) Sweet 97
Taxanthema pectinata (Aiton) Sweet 74
Taxanthema purpurata (L.) Sweet 73
Taxanthema reticulata (L.) Sweet................................. 67
Taxanthema scabra (Thunb.) Sweet 68
Taxanthema scoparia Sweet... 77
Taxanthema sinuata (L.) Sweet 89
Taxanthema spathulata (Desf.) Sweet.......................... 69
Taxanthema speciosa (L.) Sweet................................... 44
Taxanthema suffruticosa (L.) Sweet.............................. 75
Taxanthema tatarica (L.) Sweet.................................... 44
Taxanthema tetragona (Thunb.) Sweet 84
Taxanthema thouinii (Viv.) Sweet 89
Taxanthema viminea Sweet.. 72
Taxanthema virgata (Willd.) Sweet 72
Taxanthema willdenowiana Sweet 69
Thela alba Lour. 95
Thela coccinea Lour.. 95
Thela Lour. 94
Valoradia abyssinica Hochst. 93
Valoradia Hochst.. 93
Valoradia patula Hochst. 93
Valoradia plumbaginoides (Bunge) Boiss...................... 93
Vassilczenkoa Lincz. 92
Vassilczenkoa sogdiana (Lincz.) Lincz............................ 92
Vogelia africana Lam. 93
Vogelia arabica Boiss. 94
Vogelia indica Gibson ex Wight 94
Vogelia indica var. *socotrana* Balf.f............................... 94
Vogelia Lam. 93
Vogelia pendula Balf.f. 94
Vogelia perfoliata Stocks ex Wight 94

Alphabetical list of names with WFO name identifier and link to original publication

Acantholimon ×baubaschatense Lazkov – – wfo-0000743294
Acantholimon acanthobryum Rech.f. & Schiman-Czeika – – wfo-0000513145
Acantholimon acerosum (Willd.) Boiss. – – wfo-0000513146 – https://hdl.handle.net/2027/hvd.32044106337678?urlappend=%3Bseq=658
Acantholimon acerosum subsp. *acerosum* (Willd.) Boiss. – – wfo-0001303059 – https://doi.org/10.1111/j.1095-8339.2007.00663.x
Acantholimon acerosum var. *acerosum* (Willd.) Boiss. – – wfo-0001303055
Acantholimon acerosum subsp. *brachystachyum* (Boiss.) Doğan & Akaydın – – wfo-0001303056 – https://doi.org/10.1111/j.1095-8339.2007.00663.x
Acantholimon acerosum subsp. *longibracteolatum* Doğan & Akaydın – – wfo-0001303060 – https://doi.org/10.1111/j.1095-8339.2007.00663.x
Acantholimon acerosum var. *brachystachyum* Boiss. – – wfo-0001303061 – https://biodiversitylibrary.org/page/18115510
Acantholimon acerosum var. *parvifolium* Bokhari – – wfo-0001303062
Acantholimon acerosum var. *persicum* Mobayen – – wfo-0000513147 – https://vdoc.pub/download/revision-taxonomique-du-genre-acantholimon-montpellier-6usvmobjili0
Acantholimon acerosum var. *pinardii* (Boiss.) Mobayen – – wfo-0000513148 – https://vdoc.pub/download/revision-taxonomique-du-genre-acantholimon-montpellier-6usvmobjili0
Acantholimon acmostegium Boiss. & Buhse – – wfo-0000513150 – https://bibdigital.rjb.csic.es/viewer/14783/?offset=#page=351&viewer=picture&o=bookmark&n=0&q=
Acantholimon aegaeum F.K.Mey. – – wfo-0000513151
Acantholimon afanassievii Lincz. – – wfo-0000513152
Acantholimon agropyroideum Mobayen – – wfo-0000513153 – https://vdoc.pub/download/revision-taxonomique-du-genre-acantholimon-montpellier-6usvmobjili0
Acantholimon ahangarense Rech.f. & Schiman-Czeika – – wfo-0000513154
Acantholimon akaydinii Özüdoğru – – wfo-0001303063 – https://doi.org/10.11646/phytotaxa.539.3.5
Acantholimon alaicum Czerniak. – – wfo-0000513155 – https://biodiversitylibrary.org/page/30218696
Acantholimon alatavicum Bunge – – wfo-0000513156 – https://biodiversitylibrary.org/page/46698045
Acantholimon alatavicum O.Fedtsch. & B.Fedtsch. – – wfo-0001303064
Acantholimon alatavicum var. *korolkowi* Regel – – wfo-0000513157 – https://biodiversitylibrary.org/page/15733852
Acantholimon alatavicum var. *laevigatum* T.X.Peng – – wfo-0000513158
Acantholimon alatavicum var. *puberulum* Bunge ex Regel – – wfo-0001303065 – https://biodiversitylibrary.org/page/15733852
Acantholimon alatavicum var. *subsessile* (Trautv.) Herder – – wfo-0001303066 – http://www.biodiversitylibrary.org/item/210808#page/405/mode/1up
Acantholimon alatavicum var. *typicum* Regel – – wfo-0001303067 – https://biodiversitylibrary.org/page/15733852
Acantholimon alavae Rech.f. & Schiman-Czeika – – wfo-0000513159
Acantholimon albanicum O.Schwarz & F.K.Mey. – – wfo-0000513161
Acantholimon alberti Regel – – wfo-0001304469 – https://biodiversitylibrary.org/page/15733851
Acantholimon albertii Regel – – wfo-0000513162 – https://biodiversitylibrary.org/page/15733851
Acantholimon albocalycinum Assadi & Mirtadz. – – wfo-0000513163 – http://ijb.areeo.ac.ir/article_102876_en.html
Acantholimon alexandri Fed. – – wfo-0000513164
Acantholimon alexeenkoanum Czerniak. ex Ikonn. – – wfo-0000513165
Acantholimon amoenum Rech.f. & Schiman-Czeika – – wfo-0000513166
Acantholimon anatolicum Doğan & Akaydın – – wfo-0000513167 – https://doi.org/10.1046/j.1095-8339.2002.00095.x
Acantholimon anatolicum Yıld. – – wfo-0000745824 – http://docplayer.biz.tr/6409902-Ot-sistematik-botanik-dergisi-the-herb-journal-of-systematic-botany.html
Acantholimon androsaceum (Jaub. & Spach) Boiss. – – wfo-0000513168 –

https://babel.hathitrust.org/cgi/pt?id=hvd.32044106337678&view=1up&seq=655&skin=2021
Acantholimon androsaceum var. *androsaceum* (Jaub. & Spach) Boiss. – – wfo-0001303068
Acantholimon androsaceum subsp. *lycaonicum* (Boiss. & Heldr.) Bokhari – – wfo-0000513170
Acantholimon androsaceum var. *creticum* (Boiss.) Critop. – – wfo-0001303069
Acantholimon androsaceum var. *creticum* Boiss. – – wfo-0000513169 – https://babel.hathitrust.org/cgi/pt?id=hvd.32044106337678;view=1up;seq=651
Acantholimon androsaceum var. *latifolium* Boiss. – – wfo-0001303070 – https://hdl.handle.net/2027/hvd.32044106337678?urlappend=%3Bseq=651
Acantholimon androsaceum var. *majus* Boiss. – – wfo-0000513172 – https://babel.hathitrust.org/cgi/pt?id=hvd.32044106337678;view=1up;seq=651
Acantholimon androsaceum var. *olympicum* Boiss. – – wfo-0000513173 – https://babel.hathitrust.org/cgi/pt?id=hvd.32044106337678;view=1up;seq=651
Acantholimon androsaceum var. *purpurascens* Bokhari – – wfo-0000513174
Acantholimon anisophyllum Rech.f. & Schiman-Czeika – – wfo-0000513175
Acantholimon annae Lincz. – – wfo-0000513176
Acantholimon antilibanoticum Mouterde – – wfo-0000513177
Acantholimon anzobicum Lincz. – – wfo-0000513178
Acantholimon anzobicum var. *albiflorum* Lincz. – – wfo-0000513179
Acantholimon ararati hort. ex Jahand. – – wfo-0000513180
Acantholimon araxanum Bunge – – wfo-0000513181 – https://biodiversitylibrary.org/page/46698038
Acantholimon argyrostachyum Rech.f. & Schiman-Czeika – – wfo-0000513184
Acantholimon aristulatum Bunge – – wfo-0000513185 – https://biodiversitylibrary.org/page/46698065
Acantholimon armenum Boiss. & A.Huet – – wfo-0000513186 – http://bibdigital.rjb.csic.es/ing/Libro.php?Libro=1418&Pagina=66
Acantholimon armenum var. *armenum* Boiss. & A.Huet – – wfo-0001303071
Acantholimon armenum var. *balansae* Boiss. & A.Huet – – wfo-0000513187
Acantholimon armenum var. *balansae* Kusn. – – wfo-0001304457 – http://www.biodiversitylibrary.org/item/40121#page/281/mode/1up
Acantholimon armenum var. *puberulum* Trautv. – – wfo-0001303072 – http://www.biodiversitylibrary.org/item/53380#page/585/mode/1up
Acantholimon armenum var. *typicum* Trautv. – – wfo-0001303073 – http://www.biodiversitylibrary.org/item/53380#page/584/mode/1up
Acantholimon artosense Doğan & Akaydın – – wfo-0000513188 – https://doi.org/10.1111/j.1095-8339.2003.00267.x
Acantholimon arundoscapum Mobayen – – wfo-0000513189 – https://vdoc.pub/download/revision-taxonomique-du-genre-acantholimon-montpellier-6usvmobjili0
Acantholimon aspadanum Bunge – – wfo-0000513190 – https://biodiversitylibrary.org/page/46698055
Acantholimon asphodelinum Mobayen – – wfo-0000513191 – https://vdoc.pub/download/revision-taxonomique-du-genre-acantholimon-montpellier-6usvmobjili0
Acantholimon assadii Mirtadz. & Bordbar – – wfo-1000041675 – ttps://doi.org/10.11646/phytotaxa.574.1.7
Acantholimon assyriacum Boiss. – – wfo-0000513192 – https://babel.hathitrust.org/cgi/pt?id=hvd.32044106337678;view=1up;seq=652
Acantholimon assyriacum var. *micacme* Nábělek & Bornm. – – wfo-0000513193
Acantholimon assyricum var. *micracme* Nábelek & Bornm. – – wfo-0000513195
Acantholimon astragalinum Mobayen – – wfo-0000513196 – https://vdoc.pub/download/revision-taxonomique-du-genre-acantholimon-montpellier-6usvmobjili0
Acantholimon atrofuscum Rech.f. – – wfo-0000513197
Acantholimon atropatanum Bunge – – wfo-0000513198 – https://biodiversitylibrary.org/page/46698034
Acantholimon auganum Bunge – – wfo-0000513199 – https://biodiversitylibrary.org/page/46698034
Acantholimon aulieatense Czerniak. – – wfo-0000513200
Acantholimon austro-iranicum Rech.f. & Schiman-Czeika – – wfo-0000513201

Acantholimon avanosicum Doğan & Akaydın – – wfo-0000513202 – https://doi.org/10.1046/j.1095-8339.2002.00027.x
Acantholimon avenaceum Bunge – – wfo-0000513203 – https://biodiversitylibrary.org/page/46698030
Acantholimon avenaceum f. *simplicior* Bornm. ex Lincz. – – wfo-0000513205
Acantholimon avenaceum var. *khorassanicum* (Czerniak.) Mobayen – – wfo-0000513204 – https://vdoc.pub/download/revision-taxonomique-du-genre-acantholimon-montpellier-6usvmobjili0
Acantholimon azizae Mobayen – – wfo-0000513206 – https://vdoc.pub/download/revision-taxonomique-du-genre-acantholimon-montpellier-6usvmobjili0
Acantholimon bakhtiaricum Assadi – – wfo-0000513207 – http://ijb.areeo.ac.ir/article_103506_en.html
Acantholimon balansae (Kusn.) Grossh. – – wfo-0001303074
Acantholimon balansae Boiss. ex Bunge – – wfo-0000513208 – https://biodiversitylibrary.org/page/46698041
Acantholimon balchanicum Korovin – – wfo-0000513209
Acantholimon baltanense Boiss. & Hausskn. ex Boiss. – – wfo-0000513210
Acantholimon bashkaleicum Doğan & Akaydın – – wfo-0000513211 – https://doi.org/10.1111/j.1095-8339.2003.00267.x
Acantholimon birandii Doğan & Akaydın – – wfo-0000513212 – http://onlinelibrary.wiley.com/doi/10.1111/j.1756-1051.2001.tb00800.x/full
Acantholimon blakelackii Mobayen – – wfo-0001303075
Acantholimon blakelockii Mobayen – – wfo-0000513213 – https://vdoc.pub/download/revision-taxonomique-du-genre-acantholimon-montpellier-6usvmobjili0
Acantholimon blandum Czerniak. – – wfo-0000513214
Acantholimon bobrovii Czerniak. – – wfo-0000513215
Acantholimon bodeanum Bunge – – wfo-0000513216 – https://biodiversitylibrary.org/page/46698047
Acantholimon bodeanum subsp. *bodeanum* Bunge – – wfo-0001303076
Acantholimon bodeanum subsp. *pilosum* Assadi – – wfo-0001303077
Acantholimon bodeanum var. *faustii* (Trautv.) Mobayen – – wfo-0000513217 – https://vdoc.pub/download/revision-taxonomique-du-genre-acantholimon-montpellier-6usvmobjili0
Acantholimon Boiss. – – wfo-4000000124 – https://www.digitale-sammlungen.de/en/view/bsb10301127?page=69
Acantholimon sect. *Acantholimon* Boiss. – – wfo-0001304475
Acantholimon bonesseae Parsa – – wfo-0000513218
Acantholimon borodinii Krasn. – – wfo-0000513219
Acantholimon brachyphyllum Boiss. – – wfo-0000513220 – https://www.biodiversitylibrary.org/page/160852
Acantholimon brachystachyum Boiss. ex Bunge – – wfo-0000513221 – https://biodiversitylibrary.org/page/46698056
Acantholimon brachystachyum var. *brachyphyllum* Boiss. – – wfo-0001303078 – https://www.biodiversitylibrary.org/page/18115517
Acantholimon bracteatum (Girard) Boiss. – – wfo-0000513222 – https://babel.hathitrust.org/cgi/pt?id=hvd.32044106337678;view=1up;seq=648
Acantholimon bracteatum var. *bracteatum* (Girard) Boiss. – – wfo-0001303079
Acantholimon bracteatum Girard – – wfo-0001095217
Acantholimon bracteatum var. *capitatum* (Sosn.) Bokhari – – wfo-0000513223
Acantholimon bracteatum var. *intermedium* Bordz. – – wfo-0001303080
Acantholimon bracteatum var. *splendidum* (Bunge) Boiss. – – wfo-0000513224 – https://biodiversitylibrary.org/page/18115499
Acantholimon brecklei Rech.f. & Schiman-Czeika – – wfo-0000513225
Acantholimon breviscapum Boiss. & Hausskn. ex Boiss. – – wfo-0000513226
Acantholimon bromifolium Boiss. ex Bunge – – wfo-0000513227 – https://biodiversitylibrary.org/page/46698028
Acantholimon bromifolium var. *bromifolium* Boiss. ex Bunge – – wfo-0001303081
Acantholimon bromifolium f. *breviscapa* Parsa – – wfo-0000513228
Acantholimon bromifolium var. *approximatum* Bornm. – – wfo-0001303082 – https://www.biodiversitylibrary.org/page/13744550
Acantholimon bromifolium var. *ilamicum* Mobayen – – wfo-0001303083 – https://vdoc.pub/download/revision-

taxonomique-du-genre-acantholimon-montpellier-6usvmobjili0
Acantholimon bromifolium var. *iranicum* (Bornm.) Rech.f. & Schiman-Czeika – – wfo-0000513229
Acantholimon bromifolium var. *lolioides* Rech.f. & Schiman-Czeika – – wfo-0001303084
Acantholimon bromifolium var. *platyphyllum* Bornm. – – wfo-0001303085
Acantholimon butkovii Lincz. – – wfo-0000513230
Acantholimon cabulicum Boiss. – – wfo-0000513231 – https://www.biodiversitylibrary.org/page/160847
Acantholimon caesareum Boiss. & Balansa – – wfo-0000513232 – http://bibdigital.rjb.csic.es/ing/Libro.php?Libro=1418&Pagina=68
Acantholimon caesareum var. *elongatum* Mobayen – – wfo-0000513233 – https://vdoc.pub/download/revision-taxonomique-du-genre-acantholimon-montpellier-6usvmobjili0
Acantholimon calocephalum Aitch. & Hemsl. – – wfo-0000513234 – https://biodiversitylibrary.org/page/230605
Acantholimon calvertii Boiss. – – wfo-0000513235 – http://bibdigital.rjb.csic.es/ing/Libro.php?Libro=1418&Pagina=67
Acantholimon calvertii var. *calvertii* Boiss. – – wfo-0001303086
Acantholimon calvertii var. *glabrum* Akaydın & Dogan – – wfo-0001303087 – https://doi.org/10.1111/j.1095-8339.2007.00663.x
Acantholimon calvertii var. *sanguineum* Mobayen – – wfo-0000513236 – https://vdoc.pub/download/revision-taxonomique-du-genre-acantholimon-montpellier-6usvmobjili0
Acantholimon capitatum Sosn. – – wfo-0000513237
Acantholimon capitatum subsp. *capitatum* Sosn. – – wfo-0001303088
Acantholimon capitatum subsp. *sivasicum* Doğan & H.Duman – – wfo-0001303089 – https://doi.org/10.1111/j.1095-8339.2007.00663.x
Acantholimon carinatum Rech.f. & Schiman-Czeika – – wfo-0000513238
Acantholimon caryophyllaceum Boiss. – – wfo-0000513239 – https://hdl.handle.net/2027/hvd.32044106337678?urlappend=%3Bseq=656
Acantholimon caryophyllaceum subsp. *caryophyllaceum* Boiss. – – wfo-0001303090
Acantholimon caryophyllaceum Hausskn. ex Bunge – – wfo-0000513240 – https://biodiversitylibrary.org/page/46698042
Acantholimon caryophyllaceum subsp. *parviflorum* Bokhari – – wfo-0001303091
Acantholimon caryophyllaceum var. *brachystachyum* Boiss. – – wfo-0000513241
Acantholimon cataonicum Bunge – – wfo-0000513242 – https://biodiversitylibrary.org/page/46698048
Acantholimon catenatum Rech.f. & Schiman-Czeika – – wfo-0000513243
Acantholimon cephalotes Boiss. – – wfo-0000513244 – https://www.biodiversitylibrary.org/page/160846
Acantholimon cephalotoides Rech.f. – – wfo-0000513245
Acantholimon cephalotum St.-Lag. – – wfo-0000513246 – https://www.biodiversitylibrary.org/page/55494275
Acantholimon chitralicum Rech.f. & Schiman-Czeika – – wfo-0000513247
Acantholimon chlorostegium Rech.f. & Schiman-Czeika – – wfo-0000513248
Acantholimon chrysostegium Rech.f. & Schiman-Czeika – – wfo-0000513249
Acantholimon cleistocalyx Hand.-Mazz. – – wfo-0000513251 – https://www.biodiversitylibrary.org/page/30939556
Acantholimon coarctatum Trautv. – – wfo-0001303092
Acantholimon collare Köie & Rech.f. – – wfo-0000513252
Acantholimon compactum Korovin – – wfo-0000513253
Acantholimon confertiflorum Bokhari – – wfo-0000513254
Acantholimon creticum (Boiss.) Rech.f. – – wfo-0000513255
Acantholimon cupreo-olivascens Rech.f. & Schiman-Czeika – – wfo-0000513256
Acantholimon curviflorum Bunge – – wfo-0000513257 – https://biodiversitylibrary.org/page/46698062
Acantholimon cymosum Bunge – – wfo-0000513258 – https://biodiversitylibrary.org/page/46698020
Acantholimon damassanum Mobayen – – wfo-0000513259 – https://vdoc.pub/download/revision-taxonomique-du-genre-acantholimon-montpellier-6usvmobjili0
Acantholimon damassanum var. *damassanum* – – wfo-0001303093

Acantholimon damassanum var. *lancibracteatum* Bokhari – – wfo-0000513260
Acantholimon davisii Akaydın & M.B.Crespo – – wfo-0001303094 – https://doi.org/10.11646/phytotaxa.334.1.13
Acantholimon demavendicum Bornm. – – wfo-0000513262 – https://www.biodiversitylibrary.org/page/33632844
Acantholimon demawendicum Bornm. – – wfo-0001303095
Acantholimon densiflorum Assadi – – wfo-0000513263 – http://ijb.areeo.ac.ir/article_102860_en.html
Acantholimon desertorum Regel – – wfo-0000513264 – https://biodiversitylibrary.org/page/15733853
Acantholimon dianthifolium Bokhari – – wfo-0000513265
Acantholimon diapensioides Boiss. – – wfo-0000513266 – https://www.biodiversitylibrary.org/page/160848
Acantholimon diapensioides Herder – – wfo-0001303096 – https://biodiversitylibrary.org/page/51627646
Acantholimon diapensioides var. *longifolium* O.Fedsch – – wfo-0000741783 – https://biodiversitylibrary.org/page/15572446
Acantholimon distachyum Boiss. – – wfo-0000513267 – https://www.biodiversitylibrary.org/page/160847
Acantholimon distichum Rech.f. & Schiman-Czeika – – wfo-0000513268
Acantholimon diversifolium O.Schwarz & F.K.Mey. – – wfo-0000513269
Acantholimon doganii Bağcı, Doğu & Akaydın – – wfo-0000743295 – http://onlinelibrary.wiley.com/doi/10.1111/j.1756-1051.2008.00390.x/full
Acantholimon ecae Aitch. & Hemsl. – – wfo-0000513270 – https://biodiversitylibrary.org/page/2925512
Acantholimon echinus (L.) Boiss. – – wfo-0000513271
Acantholimon echinus (L.) Bunge – – wfo-0001303097 – https://biodiversitylibrary.org/page/46698051
Acantholimon echinus f. *disticum* Mobayen – – wfo-0000513274 – https://vdoc.pub/download/revision-taxonomique-du-genre-acantholimon-montpellier-6usvmobjili0
Acantholimon echinus subsp. *creticum* (Boiss.) Papan. & Kokkini – – wfo-0000513273
Acantholimon echinus subsp. *lycaonicum* (Boiss. & Heldr.) Papan. & Kokkini – – wfo-0001303098
Acantholimon echinus var. *glaberrimum* Mobayen – – wfo-0000513275 – https://vdoc.pub/download/revision-taxonomique-du-genre-acantholimon-montpellier-6usvmobjili0
Acantholimon echinus var. *puberulum* Boiss. – – wfo-0000513276
Acantholimon edmondsonii Rech.f. & Schiman-Czeika – – wfo-0000513277
Acantholimon ekatherinae (B.Fedtsch.) Czerniak. – – wfo-0000513278 – https://biodiversitylibrary.org/page/30218398
Acantholimon ekbergianum Rech.f. & Schiman-Czeika – – wfo-0000513279
Acantholimon ekimii Doğan & Akaydın – – wfo-0000506252 – https://doi.org/10.1111/j.1095-8339.2007.00663.x
Acantholimon embergeri Mobayen – – wfo-0000513280 – https://vdoc.pub/download/revision-taxonomique-du-genre-acantholimon-montpellier-6usvmobjili0
Acantholimon erinaceum (Jaub. & Spach) Lincz. – – wfo-0000513281 – https://biodiversitylibrary.org/page/30218430
Acantholimon erythraeum Bunge – – wfo-0000513284
Acantholimon eschkerense Boiss. & Hausskn. ex Boiss. – – wfo-0000513285
Acantholimon esfandiarii Rech.f. & Schiman-Czeika – – wfo-0000513286
Acantholimon eubergeri Mobayen – – wfo-0001095276 – https://vdoc.pub/download/revision-taxonomique-du-genre-acantholimon-montpellier-6usvmobjili0
Acantholimon evrenii Doğan & Akaydın – – wfo-0000513287 – https://doi.org/10.1111/j.1095-8339.2005.00438.x
Acantholimon fasciculare Boiss. – – wfo-0000513288 – https://www.biodiversitylibrary.org/page/160850
Acantholimon faustii Trautv. – – wfo-0000513289 – https://www.biodiversitylibrary.org/page/15639252
Acantholimon fedorovii Tamamsch. & Mirzoeva – – wfo-0000513290
Acantholimon ferox (Jaub. & Spach) Boiss. – – wfo-0000513291 – https://hdl.handle.net/2027/hvd.32044106337678?urlappend=%3Bseq=659
Acantholimon festucaceum (Jaub. & Spach) Boiss. – – wfo-0000513292 – https://babel.hathitrust.org/cgi/pt?id=hvd.32044106337678;view=1up;seq=652
Acantholimon festucaceum var. *festucaceum* (Jaub. & Spach) Boiss. – – wfo-0001303099
Acantholimon festucaceum var. *laxiflora* Boiss. – – wfo-0001303100

Acantholimon fetisowi Regel – – wfo-0001095218
Acantholimon fetissovii Regel – – wfo-0000513293
Acantholimon flabellum Assadi – – wfo-0000513295 – http://dx.doi.org/10.11646/phytotaxa.227.1.11
Acantholimon flabellum Assadi – – wfo-0001303038 – http://ijb.areeo.ac.ir/article_102860_en.html
Acantholimon flexuosum Boiss. ex Bunge – – wfo-0000513296 – https://biodiversitylibrary.org/page/46698056
Acantholimon flexuosum var. *laxiflorum* Mobayen – – wfo-0000513297 – https://vdoc.pub/download/revision-taxonomique-du-genre-acantholimon-montpellier-6usvmobjili0
Acantholimon fominii Kusn. – – wfo-0000513298
Acantholimon gabrieljaniae Mirzoeva – – wfo-0000513299
Acantholimon gabrieljanii Mirzoeva – – wfo-0001304470
Acantholimon gadukense Mobayen – – wfo-0000513300 – https://vdoc.pub/download/revision-taxonomique-du-genre-acantholimon-montpellier-6usvmobjili0
Acantholimon gaudanense Czerniak. – – wfo-0000513301
Acantholimon gemicianum Kaptaner İğci, Körüklü & Aytaç – – wfo-0001303101 – http://www.sekj.org/AnnBot.html
Acantholimon genistioides var. *khossrovii* Mobayen – – wfo-0001303102 – https://vdoc.pub/download/revision-taxonomique-du-genre-acantholimon-montpellier-6usvmobjili0
Acantholimon genistoides (Jaub. & Spach) Boiss. – – wfo-0000513302 – https://babel.hathitrust.org/cgi/pt?id=hvd.32044106337678;view=1up;seq=652
Acantholimon genistoides var. *genistoides* (Jaub. & Spach) Boiss. – – wfo-0001303103
Acantholimon ghoranum Rech.f. & Schiman-Czeika – – wfo-0000513303
Acantholimon gilliatii Turrill – – wfo-0000513305
Acantholimon gilliatii var. *yamense* (Turrill) Mobayen – – wfo-0000513306 – https://vdoc.pub/download/revision-taxonomique-du-genre-acantholimon-montpellier-6usvmobjili0
Acantholimon gillii Rech.f. & Köie – – wfo-0000513307
Acantholimon giselae Bornm. – – wfo-0000513308 – https://bibdigital.rjb.csic.es/viewer/14783/?offset=#page=351&viewer=picture&o=bookmark&n=0&q=
Acantholimon glabratum Assadi – – wfo-0000513309 – http://ijb.areeo.ac.ir/article_102860_en.html
Acantholimon glabratum subsp. *glabratum* Assadi – – wfo-0001303104
Acantholimon glabratum subsp. *kashanense* Batuli & Assadi – – wfo-0001303105
Acantholimon glumaceum (Jaub. & Spach) Boiss. – – wfo-0000513310 – https://babel.hathitrust.org/cgi/pt?id=hvd.32044106337678;view=1up;seq=652
Acantholimon glumaceum var. *breviscapum* Trautv. – – wfo-0001303106
Acantholimon glumaceum var. *glabra* Mobayen – – wfo-0000513311 – https://vdoc.pub/download/revision-taxonomique-du-genre-acantholimon-montpellier-6usvmobjili0
Acantholimon glumaceum var. *sahendicum* (Boiss. & Buhse) Kusn. – – wfo-0001303107 – http://www.biodiversitylibrary.org/item/40121#page/287/mode/1up
Acantholimon glumaceum var. *typicum* Trautv. – – wfo-0001303108
Acantholimon glutinosum Rech.f. & Köie – – wfo-0000513312
Acantholimon goeksunicum Doğan & Akaydın – – wfo-0000513314
Acantholimon gontscharovii Czerniak. – – wfo-0000513315
Acantholimon gorganense Mobayen – – wfo-0000513316 – https://vdoc.pub/download/revision-taxonomique-du-genre-acantholimon-montpellier-6usvmobjili0
Acantholimon gracillimum Rech.f. & Schiman-Czeika – – wfo-0000513317
Acantholimon graecum F.K.Mey. – – wfo-0000513318
Acantholimon gramineum Korovin – – wfo-0000513319
Acantholimon grammophyllum Rech.f. & Köie – – wfo-0000513320
Acantholimon griffithianum Boiss. – – wfo-0000513321 – https://www.biodiversitylibrary.org/page/160847
Acantholimon gulistanum Bunge – – wfo-0000513322 – https://biodiversitylibrary.org/page/46698072
Acantholimon gulistanum var. *glabrescens* Mobayen – – wfo-0000513323 – https://vdoc.pub/download/revision-taxonomique-du-genre-acantholimon-montpellier-6usvmobjili0

Acantholimon haesarense Bornm. ex Rech.f. & Schiman-Czeika – – wfo-0000513324
Acantholimon halophilum Bokhari – – wfo-0000513325
Acantholimon halophilum var. *halophilum* Bokhari – – wfo-0001303109
Acantholimon halophilum var. *coloratum* Doğan & Akaydın – – wfo-0001303110 – https://doi.org/10.1111/j.1095-8339.2007.00663.x
Acantholimon hamadannicum Assadi & Mahmoodi – – wfo-0001303111 – https://ijb.areeo.ac.ir/article_125619.html?lang=en
Acantholimon hariabense Rech.f. & Köie – – wfo-0000513326
Acantholimon hausknechti Bunge – – wfo-0001303112
Acantholimon haussknechtii Bunge – – wfo-0000513327 – https://biodiversitylibrary.org/page/46698042
Acantholimon hedinii Ostenf. – – wfo-0000513328
Acantholimon hellwigii Lincz. & N.I.Akshigitova – – wfo-0001303113
Acantholimon hellwigii var. *escaposum* Lincz. & N.I.Akshigitova – – wfo-0001303114
Acantholimon heratense Bunge – – wfo-0000513329 – https://biodiversitylibrary.org/page/46698060
Acantholimon heweri Rech.f. & Schiman-Czeika – – wfo-0000513330
Acantholimon hilariae Ikonn. – – wfo-0000513331
Acantholimon hindukushum Mobayen – – wfo-0000513332 – https://vdoc.pub/download/revision-taxonomique-du-genre-acantholimon-montpellier-6usvmobjili0
Acantholimon hissaricum Lincz. – – wfo-0000513334 – https://biodiversitylibrary.org/page/30218700
Acantholimon hohenackeri (Jaub. & Spach) Boiss. – – wfo-0000513335 – https://babel.hathitrust.org/cgi/pt?id=hvd.32044106337678;view=1up;seq=652
Acantholimon hohenackeri Ledeb. – – wfo-0000513336
Acantholimon hohenackeri var. *subsessile* Trautv. – – wfo-0000513337
Acantholimon hohenackeri var. *virens* Rupr. – – wfo-0001303115 – https://biodiversitylibrary.org/page/46560801
Acantholimon homophyllum Rech.f. & Schiman-Czeika – – wfo-0000513338
Acantholimon hormozganense Assadi – – wfo-0000513339 – http://ijb.areeo.ac.ir/article_103338_en.html
Acantholimon horridum Bunge – – wfo-0000513340 – https://biodiversitylibrary.org/page/46698069
Acantholimon hoshapicum Doğan & Akaydın – – wfo-0000513341 – https://doi.org/10.1111/j.1095-8339.2003.00267.x
Acantholimon huetii Boiss. – – wfo-0000513342 – http://bibdigital.rjb.csic.es/ing/Libro.php?Libro=1418&Pagina=67
Acantholimon huetii var. *huetii* Boiss. – – wfo-0001303116 – https://doi.org/10.1111/j.1095-8339.2007.00663.x
Acantholimon huetii var. *breviscapum* Akaydın & Dogan – – wfo-0001303117 – https://doi.org/10.1111/j.1095-8339.2007.00663.x
Acantholimon hyalinum Rech.f. & Köie – – wfo-0000513343
Acantholimon hypochaerum Bokhari – – wfo-0000513344
Acantholimon hypochoerum Mobayen – – wfo-0000513345 – https://vdoc.pub/download/revision-taxonomique-du-genre-acantholimon-montpellier-6usvmobjili0
Acantholimon hypochoerum var. *erythraeum* Mobayen – – wfo-0000513346 – https://vdoc.pub/download/revision-taxonomique-du-genre-acantholimon-montpellier-6usvmobjili0
Acantholimon hystrix (Jaub. & Spach) Boiss. – – wfo-0001303118 – https://hdl.handle.net/2027/hvd.32044106337678?urlappend=%3Bseq=653
Acantholimon hystrix Stapf – – wfo-0000513347 – https://www.biodiversitylibrary.org/page/7216668
Acantholimon ibrahimii Akaydın – – wfo-0001303119 – https://doi.org/10.11646/phytotaxa.340.1.2
Acantholimon iconicum Boiss. & Heldr. ex Boiss. – – wfo-0000513348
Acantholimon incomptum Boiss. & Buhse – – wfo-0000513349
Acantholimon incomptum var. *straussii* Bornm. – – wfo-0001303120 – https://www.biodiversitylibrary.org/page/3884697
Acantholimon inerme Rech.f. & Köie – – wfo-0000513350
Acantholimon iranicum Bornm. – – wfo-0000513351 – https://www.biodiversitylibrary.org/page/3884695
Acantholimon iskanderi Lipsky ex O.Fedtsch. & B.Fedtsch. – – wfo-0000513352

Acantholimon jarmilae Halda – – wfo-0000513353
Acantholimon kandaharense Rech.f. – – wfo-0000513354
Acantholimon karabajeviorum Lazkov – – wfo-0001303121 – http://old.ssbg.asu.ru/eng/turczaninowia.php?pages=npc/search.php&god=2011
Acantholimon karadarjense Lincz. – – wfo-0000513355
Acantholimon karamanicum Akaydın & Dogan – – wfo-0000513356 – http://www.tandfonline.com/doi/abs/10.1560/54A2-0BEP-BTYH-A3WC
Acantholimon karatavicum Pavlov – – wfo-0000513357
Acantholimon karelinii (Stschegl.) Bunge – – wfo-0000513358 – https://biodiversitylibrary.org/page/46698063
Acantholimon kaschgaricum Lincz. – – wfo-0000513359
Acantholimon katrantavicum Lincz. – – wfo-0000513360
Acantholimon kermanense Assadi & Mirtadz. – – wfo-0000513361 – http://ijb.areeo.ac.ir/article_102876_en.html
Acantholimon khorassanicum Czerniak. – – wfo-0000513362
Acantholimon khorassanicum var. *kopetdaghense* Czerniak. – – wfo-0001303122
Acantholimon kjurendaghi Mesczer. – – wfo-0000513363
Acantholimon knorringianum Lincz. – – wfo-0000513364 – https://biodiversitylibrary.org/page/30218704
Acantholimon koeiei Rech.f. & Schiman-Czeika – – wfo-0000513365
Acantholimon koelzii Rech.f. & Köie – – wfo-0000513366
Acantholimon koeycegizicum Doğan & Akaydın – – wfo-0000513367
Acantholimon kokandense Bunge – – wfo-0000513368
Acantholimon komarovii Czerniak. – – wfo-0000513369 – https://biodiversitylibrary.org/page/30218705
Acantholimon korolkovii (Regel) Korovin – – wfo-0000513370 – https://biodiversitylibrary.org/page/30218425
Acantholimon korovini Czerniak. – – wfo-0001304471
Acantholimon korovinii Czerniak. – – wfo-0000513371
Acantholimon kotschyi (Jaub. & Spach) Boiss. – – wfo-0000513372 – https://babel.hathitrust.org/cgi/pt?id=hvd.32044106337678;view=1up;seq=652
Acantholimon kotschyi subsp. *kotschyi* – – wfo-0001303123
Acantholimon kotschyi subsp. *laxispicatum* Bokhari – – wfo-0000513374
Acantholimon kotschyi var. *cataonicum* Bunge – – wfo-0001303124
Acantholimon kotschyi var. *iconicum* Boiss. – – wfo-0000513373 – http://www.biodiversitylibrary.org/openurl?pid=title:286&volume=12&issue=&spage=628&date=1848
Acantholimon kotschyi var. *libanoticum* Boiss. – – wfo-0001303125
Acantholimon kuramense Lincz. – – wfo-0000513375
Acantholimon kurdicum Bunge – – wfo-0000513376 – https://biodiversitylibrary.org/page/46698057
Acantholimon kutschanense Rech.f. – – wfo-0000513377
Acantholimon laevigatum (T.X.Peng) Kamelin – – wfo-0000513378 – https://biodiversitylibrary.org/page/639168
Acantholimon langaricum O.Fedtsch. & B.Fedtsch. – – wfo-0000513379
Acantholimon latifolium Boiss. – – wfo-0000513380 – http://bibdigital.rjb.csic.es/ing/Libro.php?Libro=1418&Pagina=63
Acantholimon latifolium Rupr. – – wfo-0000513381 – https://biodiversitylibrary.org/page/46560801
Acantholimon laxiflorum Boiss. – – wfo-0001303126 – https://biodiversitylibrary.org/page/18115504
Acantholimon laxiflorum Boiss. ex Bunge – – wfo-0000513382 – https://biodiversitylibrary.org/page/46698033
Acantholimon laxiusculum F.O.Khass. & I.I.Malzev – – wfo-0001303127
Acantholimon laxum Czerniak. – – wfo-0000513383
Acantholimon leptostachyum Aitch. & Hemsl. – – wfo-0000513385 – https://biodiversitylibrary.org/page/230604
Acantholimon lepturoides (Jaub. & Spach) Boiss. – – wfo-0000513386 – https://babel.hathitrust.org/cgi/pt?id=hvd.32044106337678;view=1up;seq=652
Acantholimon leucacanthum (Jaub. & Spach) Boiss. – – wfo-0000513387 –

https://babel.hathitrust.org/cgi/pt?id=hvd.32044106337678;view=1up;seq=652
Acantholimon leucacanthum var. *orshanum* Mobayen – – wfo-0000513388 – https://vdoc.pub/download/revision-taxonomique-du-genre-acantholimon-montpellier-6usvmobjili0
Acantholimon leucochlorum Rech.f. & Schiman-Czeika – – wfo-0000513389
Acantholimon libanoticum Boiss. – – wfo-0000513390 – https://www.biodiversitylibrary.org/page/160854
Acantholimon libanoticum var. *ulicinum* (Willd. ex Schult.) Boiss. – – wfo-0000513391 – http://www.biodiversitylibrary.org/openurl?pid=title:286&volume=12&issue=&spage=627&date=1848
Acantholimon limbatum (Lincz.) Sennikov – – wfo-0001303128
Acantholimon linczevskianum Lazkov – – wfo-0000513392
Acantholimon linczevskii Pavlov – – wfo-0000513393
Acantholimon listoniae Boiss. – – wfo-0000513394
Acantholimon litvinovii Lincz. – – wfo-0000513395
Acantholimon longiflorum Boiss. – – wfo-0000513396 – http://bibdigital.rjb.csic.es/ing/Libro.php?Libro=1418&Pagina=65
Acantholimon longiscapum Bokhari – – wfo-0000513397
Acantholimon lycaonicum Boiss. & Heldr. – – wfo-0000513398
Acantholimon lycaonicum subsp. *lycaonicum* Boiss. & Heldr. – – wfo-0001303129 – https://doi.org/10.1111/j.1095-8339.2007.00663.x
Acantholimon lycaonicum subsp. *cappadocicum* Doğan & Akaydın – – wfo-0001303130 – https://doi.org/10.1111/j.1095-8339.2007.00663.x
Acantholimon lycaonicum var. *cataonicum* (Bunge) Mobayen – – wfo-0000513399 – https://vdoc.pub/download/revision-taxonomique-du-genre-acantholimon-montpellier-6usvmobjili0
Acantholimon lycopodioides (Girard) Boiss. – – wfo-0000513400
Acantholimon macranthum Rech.f. & Köie – – wfo-0000513401
Acantholimon macranthum subsp. *dubium* Rech.f. & Köie – – wfo-0001303131
Acantholimon macropetalum Rech.f. & Schiman-Czeika – – wfo-0000513402
Acantholimon macrostachyum Rech.f. & Schiman-Czeika – – wfo-0000513403
Acantholimon maewskianum Regel – – wfo-0001303132 – https://biodiversitylibrary.org/page/15733853
Acantholimon majewianum O.Fedtsch. & B.Fedtsch. – – wfo-0001304458
Acantholimon majewianum Regel – – wfo-0000513404 – https://biodiversitylibrary.org/page/15733853
Acantholimon manakyanii Ogan. – – wfo-0001303133 – http://takhtajania.asj-oa.am/id/eprint/12
Acantholimon margaritae Korovin – – wfo-0000513406 – https://biodiversitylibrary.org/page/30218699
Acantholimon marmoreum Korovin – – wfo-0000513407
Acantholimon melananthum (Boiss.) Boiss. – – wfo-0000513408 – https://babel.hathitrust.org/cgi/pt?id=hvd.32044106337678;view=1up;seq=652
Acantholimon microstegium Bornm. – – wfo-0000513409
Acantholimon mikeschinii Lincz. – – wfo-0000513410 – https://biodiversitylibrary.org/page/30218697
Acantholimon minshelkense Pavlov – – wfo-0000513411
Acantholimon mirandum Lincz. – – wfo-0000513412 – https://biodiversitylibrary.org/page/30218706
Acantholimon mirtadzadinii Assadi – – wfo-0000513413 – http://ijb.areeo.ac.ir/article_103506_en.html
Acantholimon mirum Lincz. – – wfo-0000513414 – https://biodiversitylibrary.org/page/30218707
Acantholimon mishaudaghense Mobayen – – wfo-0000513415 – https://vdoc.pub/download/revision-taxonomique-du-genre-acantholimon-montpellier-6usvmobjili0
Acantholimon mobayenii Assadi & Ghahr. – – wfo-0000506254
Acantholimon modestum Bornm. ex Rech.f. & Schiman-Czeika – – wfo-0000513417
Acantholimon moradii Assadi – – wfo-0000513418 – http://ijb.areeo.ac.ir/article_102860_en.html
Acantholimon moradii Assadi – – wfo-0001303037 – http://dx.doi.org/10.11646/phytotaxa.227.1.11
Acantholimon movdarinum Parsa – – wfo-0000513419
Acantholimon muchamedshanovii Lincz. – – wfo-0000513420

Acantholimon multiflorum (Bokhari) Doğan & Akaydın – – wfo-0000506255 – https://doi.org/10.1111/j.1095-8339.2007.00663.x
Acantholimon munroanum Aitch. & Hemsl. – – wfo-0000513421 – https://biodiversitylibrary.org/page/230604
Acantholimon muradicum O.Schwarz & F.K.Mey. – – wfo-0000513422
Acantholimon murorum Korovin – – wfo-0000513423
Acantholimon nabievii Lincz. – – wfo-0000513424
Acantholimon narynense Lazkov – – wfo-0000513425
Acantholimon nawaricum Rech.f. & Schiman-Czeika – – wfo-0000513426
Acantholimon nigricans Mobayen – – wfo-0000513428 – https://vdoc.pub/download/revision-taxonomique-du-genre-acantholimon-montpellier-6usvmobjili0
Acantholimon nikitinii Lincz. – – wfo-0000513429 – https://biodiversitylibrary.org/page/30218706
Acantholimon nuratavicum Zakirov – – wfo-0000513430 – https://biodiversitylibrary.org/page/30218700
Acantholimon nuristanicum Kitam. – – wfo-0000513431
Acantholimon oliganthum Boiss. – – wfo-0000513432 – https://babel.hathitrust.org/cgi/pt?id=hvd.32044106337678;view=1up;seq=652
Acantholimon olivieri (Jaub. & Spach) Boiss. – – wfo-0000513433 – https://babel.hathitrust.org/cgi/pt?id=hvd.32044106337678&view=1up&seq=660
Acantholimon olympicum (Boiss.) F.K.Mey. – – wfo-0000513434
Acantholimon oopodum (Popov & Korovin) Sennikov – – wfo-0001303134
Acantholimon ophiocladum Rech.f. & Schiman-Czeika – – wfo-0000513435
Acantholimon pamiricum Czerniak. – – wfo-0000513436 – https://biodiversitylibrary.org/page/30218702
Acantholimon paniculatum Rech.f. – – wfo-0000513437
Acantholimon parsanum Mobayen – – wfo-0000513439 – https://vdoc.pub/download/revision-taxonomique-du-genre-acantholimon-montpellier-6usvmobjili0
Acantholimon parsianum Lincz. – – wfo-0000513440
Acantholimon parviflorum (Bokhari) Akaydın & Dogan – – wfo-0000506256 – https://doi.org/10.1111/j.1095-8339.2007.00663.x
Acantholimon parviflorum Regel – – wfo-0000513441
Acantholimon pavlovii Lincz. – – wfo-0000513442
Acantholimon peculiare Rech.f. – – wfo-0000513443
Acantholimon peronini Boiss. – – wfo-0000513444 – https://biodiversitylibrary.org/page/18115514
Acantholimon peroninii Boiss. – – wfo-0001303135
Acantholimon petraeum Boiss. ex Bunge – – wfo-0000513445 – https://biodiversitylibrary.org/page/46698033
Acantholimon petuniiflorum Mobayen – – wfo-0000513446 – https://vdoc.pub/download/revision-taxonomique-du-genre-acantholimon-montpellier-6usvmobjili0
Acantholimon phrygium Boiss. – – wfo-0000513447 – https://babel.hathitrust.org/cgi/pt?id=hvd.32044106337678;view=1up;seq=652
Acantholimon physostegium Rech.f. & Schiman-Czeika – – wfo-0000513448
Acantholimon pinardi Boiss. – – wfo-0001303136
Acantholimon pinardii Boiss. – – wfo-0000513450 – https://babel.hathitrust.org/cgi/pt?id=hvd.32044106337678&view=1up&seq=663&skin=2021
Acantholimon podlechii Rech.f. & Schiman-Czeika – – wfo-0000513451
Acantholimon poliochlorum Rech.f. & Schiman-Czeika – – wfo-0000513452
Acantholimon polystachyum Boiss. – – wfo-0000513453
Acantholimon popovii Czerniak. – – wfo-0000513454
Acantholimon procumbens Czerniak. – – wfo-0000513455
Acantholimon pskemense Lincz. – – wfo-0000513456 – https://biodiversitylibrary.org/page/30218698
Acantholimon pterostegium Bunge – – wfo-0000513457 – https://biodiversitylibrary.org/page/46698020
Acantholimon puberulum Boiss. & Balansa – – wfo-0000513458 –

http://bibdigital.rjb.csic.es/ing/Libro.php?Libro=1418&Pagina=64
Acantholimon puberulum subsp. *puberulum* Boiss. & Balansa – – wfo-0001303137 – https://doi.org/10.1111/j.1095-8339.2007.00663.x
Acantholimon puberulum var. *puberulum* Boiss. & Balansa – – wfo-0001303138
Acantholimon puberulum subsp. *longiscapum* (Bokhari) Doğan & Akaydın – – wfo-0000506257 – https://doi.org/10.1111/j.1095-8339.2007.00663.x
Acantholimon puberulum subsp. *peronini* (Boiss.) Akaydın & Dogan – – wfo-0001303139 – https://doi.org/10.1111/j.1095-8339.2007.00663.x
Acantholimon puberulum subsp. *peroninii* (Boiss.) Akaydın & Dogan – – wfo-0001303140
Acantholimon puberulum var. *glabrum* Bokhari – – wfo-0000513459
Acantholimon puberulum var. *longiscapum* Bokhari – – wfo-0000513461
Acantholimon pulchellum f. *condensatum* Korovin – – wfo-0001303141
Acantholimon pulchellum Korovin – – wfo-0000513462
Acantholimon purpureum Korovin – – wfo-0000513463
Acantholimon purpureum Parsa – – wfo-0000513464
Acantholimon quettense Rech.f. & Schiman-Czeika – – wfo-0000513465
Acantholimon quettensis Rech.f. & Schiman-Czeika – – wfo-0001303039
Acantholimon quinquelobum Bunge – – wfo-0000513466 – https://biodiversitylibrary.org/page/46698061
Acantholimon quinquelobum var. *quinquelobum* Bunge – – wfo-0001303142 – https://doi.org/10.1111/j.1095-8339.2007.00663.x
Acantholimon quinquelobum var. *curviflorum* (Bunge) Doğan & Akaydın – – wfo-0000506258 – https://doi.org/10.1111/j.1095-8339.2007.00663.x
Acantholimon raddeanum Czerniak. – – wfo-0000513467
Acantholimon raikoviae Czerniak. ex Lincz. – – wfo-0000513468
Acantholimon rechingeri Freitag – – wfo-0000513469
Acantholimon reflexifolium Bokhari – – wfo-0000513470
Acantholimon restiaceum Bunge – – wfo-0000513472 – https://biodiversitylibrary.org/page/46698027
Acantholimon revolutum Rech.f. & Köie – – wfo-0000513473
Acantholimon rhodopolium Rech.f. & Schiman-Czeika – – wfo-0000513474
Acantholimon riyatguelii Yıldırım – – wfo-0001303143 – http://dx.doi.org/10.11646/phytotaxa.175.2.2
Acantholimon roborowskii Czerniak. – – wfo-0000513475
Acantholimon roseum Boiss. – – wfo-0000513476 – https://babel.hathitrust.org/cgi/pt?id=hvd.32044106337678;view=1up;seq=649
Acantholimon roseum var. *erinaceum* (Jaub. & Spach) Boiss. – – wfo-0001303144
Acantholimon roseum var. *pubescens* Czebnjak. – – wfo-0001303145
Acantholimon roseum var. *pungens* Boiss. – – wfo-0001303146
Acantholimon rubellum Boiss. ex Bunge – – wfo-0000513477 – https://biodiversitylibrary.org/page/46698071
Acantholimon rubricosum Mobayen – – wfo-0000513478 – https://vdoc.pub/download/revision-taxonomique-du-genre-acantholimon-montpellier-6usvmobjili0
Acantholimon rudbaricum Bornm. – – wfo-0000513479 – https://www.biodiversitylibrary.org/page/33632845
Acantholimon ruprechti Bunge – – wfo-0001304472
Acantholimon ruprechtii Bunge – – wfo-0000513480 – https://biodiversitylibrary.org/page/46698025
Acantholimon saadii Assadi & Zeraatkar – – wfo-0001303147
Acantholimon sackeni Bunge – – wfo-0001304473
Acantholimon sackenii Bunge – – wfo-0000513482 – https://www.biodiversitylibrary.org/page/46698073
Acantholimon sahendicum Boiss. & Buhse – – wfo-0000513483
Acantholimon salangense Bokhari – – wfo-0000513484
Acantholimon salangensis Bokhari – – wfo-0001304454

Acantholimon saravschanicum Regel – – wfo-0001303041
Acantholimon sarawschanicum Regel – – wfo-0000513485
Acantholimon sarytavicum Lincz. – – wfo-0000513486
Acantholimon saxifragifolium Rech.f. & Köie – – wfo-0000513487
Acantholimon saxifragiforme Hausskn. & Sint. ex Bokhari – – wfo-0000513489
Acantholimon saxifragiforme Hausskn. ex Mobayen – – wfo-0000513488 – https://vdoc.pub/download/revision-taxonomique-du-genre-acantholimon-montpellier-6usvmobjili0
Acantholimon scabiosum Mobayen – – wfo-0000513490 – https://vdoc.pub/download/revision-taxonomique-du-genre-acantholimon-montpellier-6usvmobjili0
Acantholimon scabiosum var. *nudicalyx* Mobayen – – wfo-0000513491 – https://vdoc.pub/download/revision-taxonomique-du-genre-acantholimon-montpellier-6usvmobjili0
Acantholimon scabrellum Boiss. & Hausskn. ex Boiss. – – wfo-0000513493
Acantholimon scabrellum var. *scabrellum* Boiss. & Hausskn. ex Boiss. – – wfo-0001303148
Acantholimon scabrellum var. *kandilum* Mobayen – – wfo-0001303149 – https://vdoc.pub/download/revision-taxonomique-du-genre-acantholimon-montpellier-6usvmobjili0
Acantholimon schachimardanicum Lincz. – – wfo-0000513494
Acantholimon schahrudicum Bunge – – wfo-0000513495 – https://biodiversitylibrary.org/page/46698070
Acantholimon schemachense Grossh. – – wfo-0000513496
Acantholimon schirazianum Boiss. – – wfo-0000513497 – https://babel.hathitrust.org/cgi/pt?id=hvd.32044106337678;view=1up;seq=650
Acantholimon schizostegium Rech.f. & Schiman-Czeika – – wfo-0000513498
Acantholimon scirpinum Bunge – – wfo-0000513499 – https://biodiversitylibrary.org/page/46698027
Acantholimon sclerophyllum Rech.f. & Schiman-Czeika – – wfo-0000513500
Acantholimon scorpioideum St.-Lag. – – wfo-0000513501 – https://www.biodiversitylibrary.org/page/55494275
Acantholimon scorpius (Jaub. & Spach) Boiss. – – wfo-0000513502 – https://babel.hathitrust.org/cgi/pt?id=hvd.32044106337678;view=1up;seq=652
Acantholimon scorpius var. *balutchii* Mobayen – – wfo-0000513503 – https://vdoc.pub/download/revision-taxonomique-du-genre-acantholimon-montpellier-6usvmobjili0
Acantholimon scorpius var. *incomptum* (Boiss. & Buhse) Boiss. – – wfo-0000513504
Acantholimon scorpius var. *leucacanthum* Boiss. – – wfo-0001303150
Acantholimon sect. *Acmostegia* Bunge – – wfo-0001304476
Acantholimon sect. *Armeriopsis* Boiss. – – wfo-0001304477
Acantholimon sect. *Bromeliopsis* Rech.f. & Schiman-Czeika – – wfo-0001304478
Acantholimon sect. *Chaetolimon* Bunge – – wfo-0001303151
Acantholimon sect. *Cymaria* Bunge – – wfo-0001304502 – https://www.biodiversitylibrary.org/page/28687076
Acantholimon sect. *Dracogyna* Mobayen – – wfo-0001304480
Acantholimon sect. *Glumaria* Boiss. – – wfo-0001304481 – https://www.biodiversitylibrary.org/page/160847
Acantholimon sect. *Gontscharovia* Lincz. – – wfo-0001304483
Acantholimon sect. *Inermia* Rech.f. & Köie – – wfo-0001304482
Acantholimon sect. *Microstegia* Bornm. – – wfo-0001304484
Acantholimon sect. *Physostegia* Rech.f. & Schiman-Czeika – – wfo-0001304485
Acantholimon sect. *Platystegia* Rech.f. & Schiman-Czeika – – wfo-0001304486
Acantholimon sect. *Poicilocephala* Rech.f. & Schiman-Czeika – – wfo-0001304487
Acantholimon sect. *Pterostegia* Bunge – – wfo-0001304488 – https://www.biodiversitylibrary.org/page/28687076
Acantholimon sect. *Pulvinaria* Boiss. – – wfo-0001304489 – https://www.biodiversitylibrary.org/page/18115496
Acantholimon sect. *Schizostegia* Rech.f. & Schiman-Czeika – – wfo-0001304490
Acantholimon sect. *Staticopsis* Boiss. – – wfo-0001304491
Acantholimon sect. *Stereophylla* Rech.f. & Schiman-Czeika – – wfo-0001304492

Acantholimon sect. *Tragacanthina* Bunge – – wfo-0001304493
Acantholimon senganense Bunge – – wfo-0000513505 – https://biodiversitylibrary.org/page/46698034
Acantholimon senganense subsp. *senganense* Bunge – – wfo-0001303152
Acantholimon senganense subsp. *tehranense* Assadi – – wfo-0001303153
Acantholimon senganense var. *glaucum* Parsa – – wfo-0000513506
Acantholimon serotinum Rech.f. & Schiman-Czeika – – wfo-0000513507
Acantholimon setiferum Bunge – – wfo-0000513508 – https://biodiversitylibrary.org/page/46698073
Acantholimon shahrudicum Bunge – – wfo-0001303154
Acantholimon sintenisii Hausskn. – – wfo-0000513509
Acantholimon sirchense Assadi & Mirtadz. – – wfo-0000513510 – http://ijb.areeo.ac.ir/article_102876_en.html
Acantholimon sogdianum (Lincz.) Sennikov – – wfo-0001303155
Acantholimon solidum Rech.f. & Köie – – wfo-0000513512
Acantholimon sorchense Rech.f. – – wfo-0000513513
Acantholimon speciosissimum Aitch. & Hemsl. – – wfo-0000513514 – https://biodiversitylibrary.org/page/2925513
Acantholimon spinicalyx Köie & Rech.f. – – wfo-0000513515
Acantholimon spinosum Rob. – – wfo-0001303156
Acantholimon spirizianum Mobayen – – wfo-0000513516 – https://vdoc.pub/download/revision-taxonomique-du-genre-acantholimon-montpellier-6usvmobjili0
Acantholimon spirizianum subsp. *spirizianum* – – wfo-0001303157 – https://vdoc.pub/download/revision-taxonomique-du-genre-acantholimon-montpellier-6usvmobjili0
Acantholimon spirizianum var. *multiflorum* Bokhari – – wfo-0000513517
Acantholimon splendidum Bunge – – wfo-0000513518 – https://biodiversitylibrary.org/page/46698022
Acantholimon squarrosum Pavlov – – wfo-0000513519
Acantholimon stanjukoviczii Lincz. ex Ikonn. – – wfo-0000513520
Acantholimon stapfianum Rech.f. & Schiman-Czeika – – wfo-0000513521
Acantholimon stenorhaphium Rech.f. – – wfo-0000513523
Acantholimon stereophyllum Rech.f. & Schiman-Czeika – – wfo-0000513524
Acantholimon stocksii Boiss. – – wfo-0000513525 – http://bibdigital.rjb.csic.es/ing/Libro.php?Libro=1418&Pagina=65
Acantholimon stocksii var. *auriculatum* Mobayen – – wfo-0000513526 – https://vdoc.pub/download/revision-taxonomique-du-genre-acantholimon-montpellier-6usvmobjili0
Acantholimon straussii Bornm. – – wfo-0000513527 – https://www.biodiversitylibrary.org/page/3884697
Acantholimon strictiforme Nikitina ex Lazkov – – wfo-0000513528
Acantholimon strictum Czerniak. – – wfo-0000513529 – https://biodiversitylibrary.org/page/30218697
Acantholimon strigillosum Bokhari – – wfo-0000513530
Acantholimon stroterophyllum Rech.f. & Schiman-Czeika – – wfo-0000513531
Acantholimon subavenaceum Lincz. – – wfo-0000513532
Acantholimon subflavescens Rech.f. & Schiman-Czeika – – wfo-0000513534
Acantholimon subsect. *Caryophyllacea* Boiss. – – wfo-0001304494
Acantholimon subsect. *Erythrostoma* Bunge – – wfo-0001304495
Acantholimon subsect. *Eurystomata* Bunge – – wfo-0001304496
Acantholimon subsect. *Exacantha* Yıldırım & M.B.Crespo – – wfo-0001304497
Acantholimon subsect. *Halophiliacea* Muvaffak & Doğan – – wfo-0001304498
Acantholimon subsect. *Microcalycina* (Bunge) Boiss. – – wfo-0001304499 – https://www.biodiversitylibrary.org/page/18115497
Acantholimon subsect. *Rhodocalycina* Bunge – – wfo-0001304500
Acantholimon subsect. *Stenostoma* Bunge – – wfo-0001304501

Acantholimon subsimile Rech.f. & Schiman-Czeika – – wfo-0000513535
Acantholimon subulatum Boiss. – – wfo-0000513536
Acantholimon szovitzii Boiss. & Buhse – – wfo-0000513537 – https://biodiversitylibrary.org/page/11820162
Acantholimon takhtajanii Ogan. – – wfo-0001303158 – http://takhtajania.asj-oa.am/id/eprint/12
Acantholimon talagonicum Boiss. – – wfo-0000513538
Acantholimon talagonicum var. *microstegium* (Bornm.) Mobayen – – wfo-0000513539 – https://vdoc.pub/download/revision-taxonomique-du-genre-acantholimon-montpellier-6usvmobjili0
Acantholimon talassicum Korovin – – wfo-0000513540
Acantholimon tarbagataicum Gamajunova – – wfo-0000513541
Acantholimon taschkurganicum Lincz. & N.I.Akshigitova – – wfo-0000513542
Acantholimon taschkurganicum var. *taschkurganicum* Lincz. & N.I.Akshigitova – – wfo-0001303159
Acantholimon taschkurganicum var. *escaposum* Lincz. & N.I.Akshigitova – – wfo-0001303160
Acantholimon tataricum Boiss. – – wfo-0000513544 – https://biodiversitylibrary.org/page/160847
Acantholimon tchihatcheffii Fisch. & C.A.Mey. – – wfo-0000513545 – https://biodiversitylibrary.org/page/41555551
Acantholimon tenuiflorum Boiss. – – wfo-0000513546 – https://hdl.handle.net/2027/hvd.32044106337678?urlappend=%3Bseq=656
Acantholimon termei Rech.f. & Schiman-Czeika – – wfo-0000513547
Acantholimon tianschanicum Czerniak. – – wfo-0000513548
Acantholimon tibeticum Hook.f. & Thomson ex C.B.Clarke – – wfo-0000513549 – https://biodiversitylibrary.org/page/456075
Acantholimon titovii Lincz. – – wfo-0000513550 – https://biodiversitylibrary.org/page/30218703
Acantholimon tomentellum Boiss. – – wfo-0000513551 – https://babel.hathitrust.org/cgi/pt?id=hvd.32044106337678;view=1up;seq=650
Acantholimon tournefortii (Jaub. & Spach) Boiss. – – wfo-0000513552 – https://babel.hathitrust.org/cgi/pt?id=hvd.32044106337678;view=1up;seq=652
Acantholimon tragacanthinum (Jaub. & Spach) Boiss. – – wfo-0000513553 – https://babel.hathitrust.org/cgi/pt?id=hvd.32044106337678;view=1up;seq=652
Acantholimon tragacanthinum Griff. ex Bunge – – wfo-0000513555 – https://biodiversitylibrary.org/page/46698069
Acantholimon tragacanthium Griff. ex Bunge – – wfo-0001303042
Acantholimon trautvetteri Kusn. – – wfo-0000513556
Acantholimon tricolor Rech.f. & Köie – – wfo-0000513557
Acantholimon trojanum F.K.Mey. – – wfo-0000513558
Acantholimon truncatum Bunge – – wfo-0000513559 – https://biodiversitylibrary.org/page/46698061
Acantholimon truncatum subsp. *rudbaricum* Bornm. – – wfo-0001303161
Acantholimon tschimganicum Korovin – – wfo-0000513560
Acantholimon tulakense Rech.f. & Schiman-Czeika – – wfo-0000513561
Acantholimon tulakensis Rech.f. & Schiman-Czeika – – wfo-0001304463
Acantholimon turcicum Doğan & Akaydın – – wfo-0000513562 – http://www.tandfonline.com/doi/abs/10.1560/NUGH-WE3X-6F8P-6NT5
Acantholimon ulicinum (Willd. ex Schult.) Boiss. – – wfo-0000513563
Acantholimon ulicinum subsp. *ulicinum* (Willd. ex Schult.) Boiss. – – wfo-0001303162
Acantholimon ulicinum var. *ulicinum* (Willd. ex Schult.) Boiss. – – wfo-0001303163
Acantholimon ulicinum subsp. *lycaonicum* (Boiss. & Heldr.) Bokhari & J.R.Edm. – – wfo-0000513566
Acantholimon ulicinum var. *creticum* (Boiss.) Bokhari & J.R.Edm. – – wfo-0000513564
Acantholimon ulicinum var. *creticum* (Boiss.) Greuter – – wfo-0001303164
Acantholimon ulicinum var. *purpurascens* (Bokhari) Bokhari & J.R.Edm. – – wfo-0000513567
Acantholimon vacillans Rech.f. & Schiman-Czeika – – wfo-0000513568
Acantholimon varivtzevae Czerniak. – – wfo-0000513569 – https://biodiversitylibrary.org/page/30218702

Acantholimon vedicum Mirzoeva – – wfo-0000513570
Acantholimon velutinum Czerniak. – – wfo-0000513571 – https://biodiversitylibrary.org/page/30218703
Acantholimon venustum (Fenzl ex Boiss.) Boiss. – – wfo-0000513572 – https://babel.hathitrust.org/cgi/pt?id=hvd.32044106337678;view=1up;seq=652
Acantholimon venustum var. *venustum* (Fenzl ex Boiss.) Boiss. – – wfo-0001303165
Acantholimon venustum var. *assyriacum* (Boiss.) Boiss. – – wfo-0000513573
Acantholimon venustum var. *laxiflorum* (Boiss. ex Bunge) Bokhari – – wfo-0000513574
Acantholimon venustum var. *olivieri* Boiss. – – wfo-0000513575
Acantholimon virens Czerniak. – – wfo-0000513577 – https://biodiversitylibrary.org/page/30218701
Acantholimon viscidulum Boiss. – – wfo-0000513578 – https://babel.hathitrust.org/cgi/pt?id=hvd.32044106337678;view=1up;seq=649
Acantholimon vvedenskyi Lincz. – – wfo-0000513579
Acantholimon wendelboi Rech.f. & Schiman-Czeika – – wfo-0000513580
Acantholimon wiedemanni Bunge – – wfo-0001304474
Acantholimon wiedemannii Bunge – – wfo-0000513581 – https://biodiversitylibrary.org/page/46698050
Acantholimon wilhelminae Rech.f. & Schiman-Czeika – – wfo-0000513582
Acantholimon xanthacanthum Rech.f. & Köie – – wfo-0000513583
Acantholimon yamense Turrill – – wfo-0000513584
Acantholimon yildizelicum Akaydın – – wfo-0000513585 – http://onlinelibrary.wiley.com/doi/10.1111/j.1756-1051.2002.tb01926.x/full
Acantholimon zaeifii Assadi – – wfo-0000513586 – http://ijb.areeo.ac.ir/article_103338_en.html
Acantholimon zakirovii Beshko – – wfo-0001303166 – http://old.ssbg.asu.ru/eng/turczaninowia.php?pages=npc/tom&tom=t18v1
Acantholimon zaprjagaevii Lincz. – – wfo-0000513588 – https://biodiversitylibrary.org/page/30218696
Aegialinites annulatus (R.Br.) C.Presl – – wfo-0000521483 – https://biodiversitylibrary.org/page/45617014
Aegialinites C.Presl – – wfo-4000000741 – https://www.biodiversitylibrary.org/page/45617014
Aegialinites rotundifolius (Roxb.) C.Presl – – wfo-0000521484 – http://bibdigital.rjb.csic.es/ing/Libro.php?Libro=1626&Pagina=290
Aegialitidaceae Lincz. – – wfo-7000000675
Aegialitideae Z.X.Peng – – wfo-0001303167
Aegialitis annulata Kurz – – wfo-0000521487 – https://biodiversitylibrary.org/page/35548303
Aegialitis annulata R.Br. – – wfo-0000521485 – http://bibdigital.rjb.csic.es/ing/Libro.php?Libro=1626&Pagina=290
Aegialitis R.Br. – – wfo-4000000742 – https://www.biodiversitylibrary.org/page/2954582
Aegialitis rotundifolia Roxb. – – wfo-0000521488 – https://biodiversitylibrary.org/page/45617014
Aegialitis tenuis Trin. – – wfo-0000841441
Aegialitis Trin. – – wfo-0001303168
Aeoniopsis cabulica (Boiss.) Rech.f. – – wfo-0000521601
Aeoniopsis Rech.f. – – wfo-4000000783
Afrolimon amoenum (C.H.Wright) Lincz. – – wfo-0000522670
Afrolimon capense (L.Bolus) Lincz. – – wfo-0000522671
Afrolimon Lincz. – – wfo-4000000858
Afrolimon longifolium (Thunb.) Lincz. – – wfo-0000522672
Afrolimon namaquanum (L.Bolus) Lincz. – – wfo-0000522673
Afrolimon peregrinum (P.J.Bergius) Lincz. – – wfo-0001285624
Afrolimon purpuratum (L.) Lincz. – – wfo-0000522675
Afrolimon teretifolium (L.Bolus) Lincz. – – wfo-0000522676
Androsace macloviana Cham. – – wfo-0001236407

Armeria ×carnotana Blanco-Dios – – wfo-0001303169 – https://revistas.uma.es/index.php/abm/article/view/2661
Armeria ×cintrana Taul.Gomes – – wfo-0001303170 – https://revistas.uma.es/index.php/abm/article/view/2832
Armeria ×intermedia Szafer – – wfo-0001095220
Armeria ×nieto-felineri Rivas Mart. & al. – – wfo-0000549128
Armeria ×pilariae Sánchez Gullón, Muñoz Rodr. & Polo Ávila – – wfo-0001303171
Armeria ×salmantica (Bernis) Nieto Fel. – – wfo-0000549212 – http://www.rjb.csic.es/jardinbotanico/ficheros/documentos/pdf/anales/1987/Anales_44(2)_319_348.pdf
Armeria adamovicii Halácsy – – wfo-0000548636 – https://biodiversitylibrary.org/page/28307382
Armeria adsurgens (Torr. ex A.Gray) Kuntze – – wfo-0000548637
Armeria aegialea Phil. – – wfo-0000548638
Armeria albi (Bernis) Nieto – – wfo-0001303172
Armeria alboi (Bernis) Nieto Fel. – – wfo-0000548639 – http://www.rjb.csic.es/jardinbotanico/jardin/contenido.php?Pag=219&tipo=volumenanales&vol=44(2)
Armeria alliacea – – wfo-0001303173
Armeria alliacea (Cav.) Hoffmanns. & Link – – wfo-0000548640
Armeria alliacea (Cav.) Hoffmanns. & Link – – wfo-0000548642
Armeria alliacea subsp. *alliacea* (Cav.) Hoffmanns. & Link – – wfo-0001303174
Armeria alliacea Griseb. – – wfo-0000548642 – https://www.digitale-sammlungen.de/en/view/bsb10301664?page=364
Armeria alliacea Mutel, A. – – wfo-0000548641 – http://www.biodiversitylibrary.org/openurl?pid=title:9461&volume=3&issue=&spage=86&date=1836
Armeria alliacea subsp. *bupleuroides* (Gren. & Godr.) O.Bolòs & Vigo – – wfo-0000548647
Armeria alliacea subsp. *capitellata* (Pau) Rivas Mart. – – wfo-0000548648
Armeria alliacea subsp. *loscosii* Romo – – wfo-0000548653
Armeria alliacea subsp. *matritensis* (Pau) Borja, Rivas Goday & Rivas Mart. – – wfo-0000548656
Armeria alliacea subsp. *plantaginea* (All.) O.Bolòs & Vigo – – wfo-0000548658
Armeria alliacea subsp. *praecox* (Jord.) Jovet & R.Vilm. – – wfo-0001303175
Armeria alliacea subsp. *ruscinonensis* (Girard) O.Bolòs & Vigo – – wfo-0000548660
Armeria alliacea subvar. *sicorisiensis* (Sennen) Bernis – – wfo-0001303176
Armeria alliacea var. *allioides* Pau – – wfo-0000548645 – http://www.biodiversitylibrary.org/openurl?pid=title:276&volume=8&issue=&spage=136&date=1910
Armeria alliacea var. *bilbilitana* (Bernis) O.Bolòs & Vigo – – wfo-0000548646
Armeria alliacea var. *bupleuroides* (Gren. & Godr.) O.Bolòs & Vigo – – wfo-0001303177
Armeria alliacea var. *daveaui* Cout. – – wfo-0000548649 – https://biodiversitylibrary.org/page/11470335
Armeria alliacea var. *heterophylla* Pau ex Viciosa – – wfo-0000548650
Armeria alliacea var. *javalambrica* Bernis – – wfo-0000548651
Armeria alliacea var. *littorifuga* (Bernis) O.Bolòs & Vigo – – wfo-0000548652
Armeria alliacea var. *palearensis* O.Bolòs & Vigo – – wfo-0000548657
Armeria alliacea var. *plantaginea* (All.) O.Bolòs & Vigo – – wfo-0001303045
Armeria alliacea var. *pubescens* Mutel, A. – – wfo-0001303178
Armeria alliacea var. *rumelicina* (Bernis) O.Bolòs & Vigo – – wfo-0000548659
Armeria alliacea var. *sicorisiensis* (Sennen) O.Bolòs & Vigo – – wfo-0000548661
Armeria alliacea var. *yebalica* Pau – – wfo-0000548662
Armeria alliacea Webb ex Ball – – wfo-0000548644 – http://www.biodiversitylibrary.org/openurl?pid=title:350&volume=16&issue=&spage=560&date=1878
Armeria allioides Boiss. – – wfo-0000548663 – http://bibdigital.rjb.csic.es/ing/Libro.php?Libro=353&Pagina=529
Armeria allioides Willk. & Lange – – wfo-0000548664 – http://www.biodiversitylibrary.org/openurl?pid=title:37768&volume=2&issue=&spage=366&date=1868

Armeria alpina f. *basitricha* Simonk. – – wfo-0000548673
Armeria alpina f. *conchensis* Bernis – – wfo-0001303179
Armeria alpina f. *conquensis* Bernis – – wfo-0001303180
Armeria alpina f. *glabra* Novák – – wfo-0000548675
Armeria alpina f. *hirsuta* Novák – – wfo-0000548678
Armeria alpina f. *hirtifolia* Novák – – wfo-0000548679
Armeria alpina f. *pumila* (Fuss ex Jáv.) Novák – – wfo-0000548685
Armeria alpina Friv. ex Griseb. – – wfo-0000548670
Armeria alpina Guss. – – wfo-0000548667
Armeria alpina Hoppe ex Ebel – – wfo-0000548669
Armeria alpina subsp. *barcensis* (Simonk.) Jáv. – – wfo-0000548672
Armeria alpina subsp. *bubanii* (G.H.M.Lawr.) Malag. – – wfo-0001303181 – https://bibdigital.rjb.csic.es/viewer/11793/?offset=#page=23&viewer=picture&o=bookmark&n=0&q=
Armeria alpina subsp. *bubanii* (G.H.M.Lawr.) Rivas Mart. – – wfo-0001303182
Armeria alpina subsp. *cantabrica* (Boiss. & Reut. ex Willk. & Lange) Malag. – – wfo-0001303183 – https://bibdigital.rjb.csic.es/viewer/11793/?offset=#page=24&viewer=picture&o=bookmark&n=0&q=
Armeria alpina subsp. *confusa* (Bernis) Malag. – – wfo-0001303184
Armeria alpina subsp. *fontqueri* (Pau) Malag. – – wfo-0001303185 – https://bibdigital.rjb.csic.es/viewer/11793/?offset=#page=23&viewer=picture&o=bookmark&n=0&q=
Armeria alpina subsp. *godayana* (Font Quer) Malag. – – wfo-0001303186
Armeria alpina subsp. *halleri* (Wallr.) Nyman – – wfo-0000548677 – http://www.biodiversitylibrary.org/openurl?pid=title:10533&volume=&issue=&spage=616&date=1881
Armeria alpina subsp. *microcephala* (Willk.) Malag. – – wfo-0001303187
Armeria alpina subsp. *muelleri* (A.Huet) Malag. – – wfo-0001303188
Armeria alpina subsp. *occasiana* (Bernis) Rivas Mart. – – wfo-0000548683
Armeria alpina subsp. *pubinervis* (Boiss.) Malag. – – wfo-0001303189
Armeria alpina subsp. *pumila* Fuss ex Jáv. – – wfo-0001303046
Armeria alpina subsp. *trachyphylla* (Lange) Malag. – – wfo-0001303190 – https://bibdigital.rjb.csic.es/viewer/11793/?offset=#page=23&viewer=picture&o=bookmark&n=0&q=
Armeria alpina subsp. *vasconica* (Sennen ex Losa) Malag. – – wfo-0001303191
Armeria alpina subsp. *vasconica* (Sennen ex Losa) Rivas Mart. & al. – – wfo-0000548688
Armeria alpina subvar. *confusa* Bernis – – wfo-0001303192
Armeria alpina Ten. ex Boiss. – – wfo-0000548671
Armeria alpina Turcz. – – wfo-0000548668
Armeria alpina var. *alba* hort. ex F.T.Hubb. – – wfo-0001303193
Armeria alpina var. *eliator* Conill & Despaty – – wfo-0000548674 – http://www.biodiversitylibrary.org/openurl?pid=title:359&volume=67&issue=&spage=146&date=1920
Armeria alpina var. *lancifolia* Freyn – – wfo-0000548680 – http://www.biodiversitylibrary.org/openurl?pid=title:13275&volume=38&issue=&spage=38&date=1888
Armeria alpina var. *microcephala* Willk. – – wfo-0000548681 – http://www.biodiversitylibrary.org/openurl?pid=title:37768&volume=2&issue=&spage=368&date=1868
Armeria alpina var. *nana* Bolzon – – wfo-0000548682
Armeria alpina var. *pumila* Fuss ex Jáv. – – wfo-0000548684
Armeria alpina var. *purpurea* (W.D.J.Koch) E.Baumann – – wfo-0000548686 – http://www.biodiversitylibrary.org/openurl?pid=title:4266&volume=9&issue=&spage=&date=1911
Armeria alpina var. *seticeps* (Rchb.) Ebel – – wfo-0001303194
Armeria alpina Willd. – – wfo-0000548665
Armeria alpina subsp. *alpina* Willd. – – wfo-0001303195
Armeria alpinifolia Pau & Font Quer – – wfo-0000548689

Armeria ambifaria Focke – – wfo-0000548690 – http://www.biodiversitylibrary.org/openurl?pid=title:4220&volume=17&issue=&spage=445&date=1903
Armeria amoena (Sims) Kuntze – – wfo-0000548691 – https://biodiversitylibrary.org/page/3854
Armeria amplifoliata Pau – – wfo-0000548692
Armeria ancarensis Merino – – wfo-0000548693
Armeria andicola Gay ex Boiss. – – wfo-0000548694 – http://www.biodiversitylibrary.org/openurl?pid=title:286&volume=12&issue=&spage=682&date=1848
Armeria andina Poepp. ex Boiss. – – wfo-0000548695 – http://www.biodiversitylibrary.org/openurl?pid=title:286&volume=12&issue=&spage=682&date=1848
Armeria andina var. *californica* Boiss. – – wfo-0000548696
Armeria androsacea Boiss. – – wfo-0000548697 – http://www.biodiversitylibrary.org/openurl?pid=title:286&volume=12&issue=&spage=679&date=1848
Armeria apollinaris Sennen & Mauricio – – wfo-0000548699
Armeria arborea L.H.Bailey – – wfo-0000746041
Armeria arctica (Cham.) Wallr. – – wfo-0000548701
Armeria arctica Sternb. – – wfo-0000548700
Armeria arctica subsp. *californica* (Boiss.) Abrams – – wfo-0000548703
Armeria arctica var. *californica* (Boiss.) S.F.Blake – – wfo-0000548702 – http://www.biodiversitylibrary.org/openurl?pid=title:721&volume=19&issue=&spage=18&date=1917
Armeria arcuata Welw. ex Boiss. & Reut. – – wfo-0000548704
Armeria arenaria (Pers.) Ebel – – wfo-0001303196
Armeria arenaria (Pers.) F.Dietr. – – wfo-0001303197
Armeria arenaria subsp. *arenaria* (Pers.) F.Dietr. – – wfo-0001443421
Armeria arenaria (Pers.) Schult. – – wfo-0000548705
Armeria arenaria subsp. *anomala* (Bernis) Catalán ex Uribe-Ech. – – wfo-0000548706
Armeria arenaria subsp. *apennina* Arrigoni – – wfo-0001303198 – https://www.herbmedit.org/flora/FL25SI_007-032.pdf
Armeria arenaria subsp. *bilbilitana* (Bernis) Nieto Fel. – – wfo-0000548707 – http://www.rjb.csic.es/jardinbotanico/jardin/contenido.php?Pag=219&tipo=volumenanales&vol=44(2)
Armeria arenaria subsp. *bupleuroides* (Godr. & Gren.) Greuter & Raus – – wfo-0000548708 – https://www.jstor.org/stable/3996917?seq=1#page_scan_tab_contents
Armeria arenaria subsp. *burgalensis* (Sennen & Elias) Uribe-Ech. – – wfo-0000548710
Armeria arenaria subsp. *confusa* (Bernis) Nieto Fel. – – wfo-0001303199 – http://www.rjb.csic.es/jardinbotanico/jardin/contenido.php?Pag=219&tipo=volumenanales&vol=44(2)
Armeria arenaria subsp. *madoricola* M.B.Crespo & Mateo – – wfo-0001303200
Armeria arenaria subsp. *marginata* (Levier) Arrigoni – – wfo-0001303201 – https://www.herbmedit.org/flora/FL25SI_007-032.pdf
Armeria arenaria subsp. *peirescii* Baumel, Auda & Médail – – wfo-0001303202 – http://onlinelibrary.wiley.com/doi/10.1111/j.1095-8339.2008.00925.x/full
Armeria arenaria subsp. *pradetensis* Médail, Baumel & Auda – – wfo-0001303203 – http://onlinelibrary.wiley.com/doi/10.1111/j.1095-8339.2008.00925.x/full
Armeria arenaria subsp. *praecox* (Jord.) Kerguélen – – wfo-0001304459
Armeria arenaria subsp. *praecox* (Jord.) Kerguélen ex Greuter, Burdet & G.Long – – wfo-0000548711
Armeria arenaria subsp. *segoviensis* (Gand. ex Bernis) Nieto Fel. – – wfo-0000548712 – http://www.rjb.csic.es/jardinbotanico/jardin/contenido.php?Pag=219&tipo=volumenanales&vol=44(2)
Armeria arenaria subsp. *vestita* (Willk.) Nieto Fel. – – wfo-0000548713 – http://www.rjb.csic.es/jardinbotanico/jardin/contenido.php?Pag=219&tipo=volumenanales&vol=44(2)
Armeria argyrocephala f. *longifolia* Hausskn. – – wfo-0000548716 – https://www.biodiversitylibrary.org/page/14241215
Armeria argyrocephala f. *minor* Hausskn. – – wfo-0000548717 – https://www.biodiversitylibrary.org/page/14241215
Armeria argyrocephala var. *graeca* Beck – – wfo-0000548715 – https://www.biodiversitylibrary.org/page/5373848
Armeria argyrocephala Wallr. – – wfo-0000548714
Armeria armeria (L.) H.Karst. – – wfo-0000548718

Armeria asperrima (Sennen) Sennen – – wfo-0001303204
Armeria aspromontana Brullo, Scelsi & Spamp. – – wfo-0000548720
Armeria atlantica Pomel – – wfo-0000548721
Armeria australis Boiss. – – wfo-0000548722 – http://bibdigital.rjb.csic.es/ing/Libro.php?Libro=353&Pagina=530
Armeria australis var. *splendens* (Lag. & Rodr.) Boiss. – – wfo-0000548723
Armeria baetica Boiss. – – wfo-0000548724 – https://bibdigital.rjb.csic.es/viewer/9792/?offset=#page=753&viewer=picture&o=bookmark&n=0&q=
Armeria baetica var. *africana* Boiss. – – wfo-0000548725 – http://www.biodiversitylibrary.org/openurl?pid=title:286&volume=12&issue=&spage=676&date=1848
Armeria baetica var. *stenophylla* Boiss. – – wfo-0000548726 – http://www.biodiversitylibrary.org/openurl?pid=title:286&volume=12&issue=&spage=677&date=1848
Armeria baltica Tzvelev – – wfo-0001303205
Armeria barcensis Simonk. – – wfo-0000548727 – http://www.biodiversitylibrary.org/openurl?pid=title:115966&volume=6&issue=&spage=13&date=1907
Armeria beirana Franco – – wfo-0000548728
Armeria beirana subsp. *altimontana* Franco – – wfo-0000548729
Armeria beirana subsp. *monchiquensis* (Bernis) Franco – – wfo-0000548731
Armeria beirana subvar. *monchiquensis* Bernis – – wfo-0001303206
Armeria beirana subvar. *sublittorea* Bernis – – wfo-0001303207
Armeria beirana var. *subrinhoi* Bernis – – wfo-0001303208
Armeria belgenciensis Donad. – – wfo-0001303209
Armeria belgenciensis Donad. ex Guin. & Vilmorin – – wfo-0001303210
Armeria belgenciensis Donad. ex Kerguélen – – wfo-0000548732
Armeria bella Albov – – wfo-0000548733
Armeria berlengensis Daveau – – wfo-0000548734 – http://www.biodiversitylibrary.org/openurl?pid=title:5931&volume=2&issue=&spage=24&date=1884
Armeria berlengensis var. *villosa* Daveau – – wfo-0000548735 – http://www.biodiversitylibrary.org/openurl?pid=title:5931&volume=2&issue=&spage=25&date=1884
Armeria bifida (Beck) Kuntze – – wfo-0000548736 – https://biodiversitylibrary.org/page/3854
Armeria bigerrensis (C.Vicioso & Beltrán) Pau ex Rivas Mart. – – wfo-0000548737
Armeria bigerrensis subsp. *bigerrensis* (C.Vicioso & Beltrán) Pau ex Rivas Mart. – – wfo-0001303211
Armeria bigerrensis subsp. *legionensis* (Bernis) Rivas Mart. & al. – – wfo-0000548738
Armeria bigerrensis subsp. *losae* (Bernis) Rivas Mart., T.E.Díaz, Fern.Prieto, Loidi & Penas – – wfo-0000548739
Armeria bigerrensis subsp. *microcephala* (Willk.) Nieto Fel. – – wfo-0000548740 – http://www.rjb.csic.es/jardinbotanico/jardin/contenido.php?Pag=219&tipo=volumenanales&vol=44(2)
Armeria biguerrensis (C.Vicioso & Beltrán) Pau ex Rivas Mart. – – wfo-0001303212
Armeria boetica Boiss. – – wfo-0001303213
Armeria boissieriana Coss. – – wfo-0000548742
Armeria bottendorfensis A.G.Schulz – – wfo-0000548743
Armeria bourgaei Boiss. ex Merino – – wfo-0000548744 – https://biodiversitylibrary.org/page/11015759
Armeria bourgaei subsp. *bourgaei* Boiss. ex Merino – – wfo-0001303214
Armeria bourgaei subsp. *lanceobracteata* (G.H.M.Lawr.) Nieto Fel. – – wfo-0000548745 – http://www.rjb.csic.es/jardinbotanico/jardin/contenido.php?Pag=219&tipo=volumenanales&vol=44(2)
Armeria bourgaei subsp. *willkommiana* (Bernis) Nieto Fel. – – wfo-0000548746 – http://www.rjb.csic.es/jardinbotanico/jardin/contenido.php?Pag=219&tipo=volumenanales&vol=44(2)
Armeria brachyphylla Boiss. – – wfo-0000548747 – http://www.biodiversitylibrary.org/openurl?pid=title:286&volume=12&issue=&spage=682&date=1848
Armeria brevifolia Kunze ex Boiss. – – wfo-0000548748 – http://www.biodiversitylibrary.org/openurl?pid=title:286&volume=12&issue=&spage=682&date=1848
Armeria brutia Brullo, Gangale & Uzunov – – wfo-0000548749

Armeria bryodes (Nutt.) Kuntze – – wfo-0001304512
Armeria bryoides (Nutt.) Kuntze – – wfo-0000548750 – https://biodiversitylibrary.org/page/3854
Armeria bubanii G.H.M.Lawr. – – wfo-0000548751
Armeria bupleuroides Cutanda – – wfo-0001303215
Armeria bupleuroides Gren. & Godr. – – wfo-0000548753 – http://www.biodiversitylibrary.org/openurl?pid=title:6635&volume=2&issue=&spage=736&date=1853
Armeria bupleuroides var. *reducta* Rouy – – wfo-0000548754
Armeria burgalensis Sennen & Elias – – wfo-0001251973
Armeria caballeroi (Bernis) Donad. – – wfo-0000548755
Armeria caespitosa (Ortega) Boiss. – – wfo-0000548756 – http://www.biodiversitylibrary.org/openurl?pid=title:286&volume=12&issue=&spage=679&date=1848
Armeria caespitosa var. *bigerrensis* Vicioso & Beltrán – – wfo-0000548757
Armeria caespitosa var. *isernii* Vicioso & Beltrán – – wfo-0000548758
Armeria caespitosa var. *splendens* (Lag. & Rodr.) Vicioso & Beltrán – – wfo-0000548759
Armeria campestris var. *hoffmannii* Wallr. – – wfo-0000548761
Armeria campestris var. *kochii* Wallr. – – wfo-0001304467
Armeria campestris var. *linkii* Wallr. – – wfo-0000548764
Armeria campestris Wallr. – – wfo-0000548760
Armeria canescens (Host) Boiss. – – wfo-0000548765
Armeria canescens subsp. *canescens* (Host) Boiss. – – wfo-0001303216
Armeria canescens (Host) Ebel – – wfo-0001303217
Armeria canescens (Torr. & A.Gray) Kuntze – – wfo-0001419108
Armeria canescens f. *albanica* Beck – – wfo-0000548766 – https://www.biodiversitylibrary.org/page/5373850
Armeria canescens f. *albiflora* Novák – – wfo-0000548767
Armeria canescens f. *angustifolia* Novák – – wfo-0000548768
Armeria canescens f. *dasyphylla* Rohlena – – wfo-0000548771
Armeria canescens f. *dasypoda* Murb. – – wfo-0000548772
Armeria canescens f. *dolichophylla* Novák – – wfo-0000548773
Armeria canescens f. *latifolia* (Vis.) Novák – – wfo-0000548777
Armeria canescens f. *microphylla* Novák – – wfo-0000548779
Armeria canescens f. *pantoscekii* (Strobl) Novák – – wfo-0000548781
Armeria canescens f. *platyphylla* Novák – – wfo-0000548782
Armeria canescens f. *pseudodalmatica* Novák – – wfo-0000548783
Armeria canescens f. *pubiscapa* Novák – – wfo-0000548784
Armeria canescens f. *rosea* Beck ex Novak – – wfo-0000548786
Armeria canescens f. *submajellensis* Novák – – wfo-0000548787
Armeria canescens subsp. *dalmatica* (Beck) Trinajstić – – wfo-0001303218
Armeria canescens subsp. *gracilis* (Ten.) Bianchini – – wfo-0000548775
Armeria canescens subsp. *littorifuga* (Bernis) Malag. – – wfo-0001303219
Armeria canescens subsp. *nebrodensis* (Guss.) P.Silva – – wfo-0000548780
Armeria canescens subsp. *ruscinonensis* (Girard) Malag. – – wfo-0001303220
Armeria canescens var. *brachyphylla* (Boiss.) G.H.M.Lawr. – – wfo-0000548769
Armeria canescens var. *dalmatica* (Beck) Novák – – wfo-0000548770
Armeria canescens var. *latifolia* Vis. – – wfo-0000548776
Armeria canescens var. *leucantha* (Boiss.) G.H.M.Lawr. – – wfo-0000548778

Armeria cantabrica Boiss. & Reut. ex Willk. & Lange – – wfo-0000548789 – http://www.biodiversitylibrary.org/openurl?pid=title:37768&volume=2&issue=&spage=366&date=1868
Armeria cantabrica Rouy – – wfo-0000548788
Armeria cantabrica subsp. *gracilifolia* (Bernis) Donad. – – wfo-0000548790
Armeria cantabrica subsp. *vasconica* (Sennen ex Losa) Uribe-Ech. – – wfo-0000548794
Armeria cantabrica subvar. *asturica* (Boiss. & Reut. ex Willk. & Lange) Bernis – – wfo-0001303221
Armeria cantabrica var. *maritima* Rouy – – wfo-0000548791
Armeria cantabrica var. *montana* Rouy – – wfo-0000548792
Armeria cantabrica var. *vasconica* (Sennen ex Losa) López Fern. – – wfo-0000548793
Armeria capitella Pau – – wfo-0000548795
Armeria caput-alba (Rothm.) Rothm. – – wfo-0000548797
Armeria cariensis Boiss. – – wfo-0000548798 – http://www.biodiversitylibrary.org/openurl?pid=title:286&volume=12&issue=&spage=677&date=1848
Armeria cariensis var. *cariensis* Boiss. – – wfo-0001303222
Armeria cariensis var. *rumelica* (Boiss.) Boiss. – – wfo-0000548799 – https://www.biodiversitylibrary.org/page/18115546
Armeria cariensis var. *thessala* Boiss. – – wfo-0001303223 – https://www.biodiversitylibrary.org/page/18115546
Armeria carpetana subsp. *anisophylla* (Bernis) Franco – – wfo-0000548801
Armeria carpetana subsp. *carpetana* – – wfo-0001303224
Armeria carpetana Villar – – wfo-0000548800
Armeria carratracensis (Bernis) Rivas Mart. – – wfo-0000548802
Armeria castellana Boiss. & Reut. ex Leresche – – wfo-0000548803
Armeria castellana Boiss. & Reut. ex Rothm. – – wfo-0000548805
Armeria castrovalnerana Alejandre, Barredo & M.J.Escal. – – wfo-0001303225
Armeria castroviejoi Nieto Fel. – – wfo-0000548806 – http://www.rjb.csic.es/jardinbotanico/jardin/contenido.php?Pag=219&tipo=volumenanales&vol=44(2)
Armeria cephalotes Hoffmanns. & Link – – wfo-0000548808 – https://bibdigital.rjb.csic.es/viewer/9783/?offset=#page=444&viewer=picture&o=bookmark&n=0&q=
Armeria cephalotes Hook. – – wfo-0000548809 – https://www.biodiversitylibrary.org/page/434273
Armeria cephalotes Schousb. – – wfo-0000548807
Armeria cephalotes var. *alba* hort. ex F.T.Hubb. – – wfo-0001303226
Armeria cephalotes var. *grandiflora* hort. ex F.T.Hubb. – – wfo-0001303227
Armeria cephalotes var. *rubra* hort. ex F.T.Hubb. – – wfo-0001303228
Armeria cephalotes var. *splendens* hort. ex F.T.Hubb. – – wfo-0001303229
Armeria cephalotus Boiss. – – wfo-0000548810 – http://www.biodiversitylibrary.org/openurl?pid=title:286&volume=12&issue=&spage=675&date=1848
Armeria cespitosa (Cav.) Boiss. – – wfo-0001303230
Armeria cespitosa subsp. *cespitosa* – – wfo-0001303231
Armeria chilensis Boiss. – – wfo-0000548811
Armeria chilensis subsp. *andina* (Poepp. ex Boiss.) Reiche – – wfo-0000548812
Armeria chilensis subsp. *macloviana* (Cham.) Reiche – – wfo-0000548818
Armeria chilensis var. *andina* (Poepp. ex Boiss.) Reiche – – wfo-0000548813
Armeria chilensis var. *bella* (Albov) Reiche – – wfo-0000548815
Armeria chilensis var. *brevifolia* Boiss. – – wfo-0000548816
Armeria chilensis var. *curvifolia* (Bertero) Boiss. – – wfo-0000548817 – http://www.biodiversitylibrary.org/openurl?pid=title:286&volume=12&issue=&spage=682&date=1848
Armeria chilensis var. *genuina* Reiche – – wfo-0001303232
Armeria chilensis var. *macloviana* (Cham.) Reiche – – wfo-0000548819

Armeria chilensis var. *magellanica* Boiss. – – wfo-0000548820
Armeria chilensis var. *majellanica* Boiss. – – wfo-0000548821 – http://www.biodiversitylibrary.org/openurl?pid=title:286&volume=12&issue=&spage=682&date=1848
Armeria chouletteana Pomel – – wfo-0001303233
Armeria choulettiana Pomel – – wfo-0000548822
Armeria ciliata (Lange) Nieto Fel. – – wfo-0000548823
Armeria cinerea Boiss. & Welw. ex Boiss. & Reut. – – wfo-0000548825
Armeria colorata Pau – – wfo-0000548826
Armeria curvifolia Bertero – – wfo-0000548827
Armeria curvifolia Colla – – wfo-0000548828
Armeria dalmatica Beck – – wfo-0000548829 – https://www.biodiversitylibrary.org/page/5373849
Armeria daveaui (Cout.) P.Silva – – wfo-0000548830
Armeria daveaui subsp. *daveaui* (Cout.) P.Silva – – wfo-0001303234
Armeria daveaui (Cout.) Rivas Mart. – – wfo-0001303235 – https://floramontiberica.files.wordpress.com/2013/01/itinerageobotanica_182_2011.pdf
Armeria daveaui subsp. *matritensis* (Bernis) Franco – – wfo-0000548831
Armeria delfinii Phil. – – wfo-0000548832
Armeria denticulata (Bertol.) Bertol. – – wfo-0001303236
Armeria denticulata (Bertol.) DC. – – wfo-0000548833 – http://bibdigital.rjb.csic.es/ing/Libro.php?Libro=932&Pagina=15
Armeria denticulata Portensch. – – wfo-0000548834
Armeria denticulata Tratt. – – wfo-0001304460
Armeria dianthoides Hornem. & Spreng. ex Boiss. – – wfo-0000548836 – http://www.biodiversitylibrary.org/openurl?pid=title:286&volume=12&issue=&spage=683&date=1848
Armeria divaricata (L.) Kuntze – – wfo-0000548837 – https://biodiversitylibrary.org/page/3854
Armeria douglasii (Hook.) Kuntze – – wfo-0000548838 – https://biodiversitylibrary.org/page/3854
Armeria duriaei Boiss. – – wfo-0000548840 – http://www.biodiversitylibrary.org/openurl?pid=title:286&volume=12&issue=&spage=684&date=1848
Armeria duriaei Cout. – – wfo-0001303237
Armeria duriaei subsp. *bourgaei* Boiss. ex Nyman – – wfo-0001303238
Armeria duriaei var. *ciliata* Lange – – wfo-0000548841 – http://www.biodiversitylibrary.org/openurl?pid=title:7547&volume=1881&issue=&spage=101&date=1882
Armeria duriaei var. *seticaulis* Debeaux ex E.Rev. – – wfo-0001303239
Armeria duriensis Franco – – wfo-0000548842
Armeria duriensis subsp. *monticola* Franco – – wfo-0000548843
Armeria duriensis subsp. *oretana* (Bernis) Franco – – wfo-0000548844
Armeria duriensis subsp. *sublittorea* (Bernis) Franco – – wfo-0000548845
Armeria duriuscula Bab. – – wfo-0000548847
Armeria ebracteata Pomel – – wfo-0000548848
Armeria ebracteata var. *laevis* Maire – – wfo-0000548849
Armeria elongata (Hoffm.) M.Loehr – – wfo-0001303240
Armeria elongata (Hoffm.) W.D.J.Koch – – wfo-0000548851
Armeria elongata f. *bella* (Albov) Skottsb. – – wfo-0000548853
Armeria elongata f. *rostellata* Szafer – – wfo-0001303241 – https://pbsociety.org.pl/journals/index.php/asbp/article/view/asbp.1946.001/6567
Armeria elongata Hoffm. – – wfo-0000548850
Armeria elongata var. *alpina* (DC.) Ces. – – wfo-0000548852
Armeria elongata var. *chilensis* (Boiss.) Skottsb. – – wfo-0000548854

Armeria elongata var. *intermedia* T.Marsson – – wfo-0000548855
Armeria elongata var. *macloviana* (Cham.) Skottsb. – – wfo-0000548856
Armeria elongata var. *maritima* (Mill.) Skottsb. – – wfo-0000548858
Armeria elongata var. *purpurea* (W.D.J.Koch) Boiss. – – wfo-0000548859 – http://www.biodiversitylibrary.org/openurl?pid=title:286&volume=12&issue=&spage=681&date=1848
Armeria elongata var. *scabra* (Pall. ex Schult.) Regel – – wfo-0000736836
Armeria elongata var. *sibirica* (Turcz. ex Boiss.) Hartm. – – wfo-0000548860
Armeria elongata W.D.J.Koch – – wfo-0001095219
Armeria eriophila Willk. – – wfo-0001303242
Armeria eriophylla var. *marizii* Daveau – – wfo-0000548862 – https://bibdigital.rjb.csic.es/viewer/10622/?offset=#page=186&viewer=picture&o=bookmark&n=0&q=
Armeria eriophylla Willk. – – wfo-0000548861 – http://www.biodiversitylibrary.org/openurl?pid=title:5931&volume=2&issue=&spage=145&date=1884
Armeria euscadiensis Donad. & Vivant – – wfo-0000548863
Armeria exaristata Phil. – – wfo-0000548864
Armeria expansa Wallr. – – wfo-0000548865
Armeria fasciculata (Vent.) Willd. – – wfo-0000548866 – http://www.biodiversitylibrary.org/openurl?pid=title:6648&volume=&issue=&spage=334&date=1809
Armeria fasciculata var. *aristata* Mutel, A. – – wfo-0000496406 – http://www.biodiversitylibrary.org/openurl?pid=title:9461&volume=3&issue=&spage=86&date=1836
Armeria fasciculata var. *intermedia* Daveau – – wfo-0000549178
Armeria fibrosa Pomel – – wfo-0000548867
Armeria filicaulis (Boiss.) Boiss. – – wfo-0000548870 – https://bibdigital.rjb.csic.es/viewer/9792/?offset=#page=531&viewer=picture&o=bookmark&n=0&q=
Armeria filicaulis subsp. *filicaulis* (Boiss.) Boiss. – – wfo-0001303243
Armeria filicaulis Rouy – – wfo-0001303244
Armeria filicaulis subsp. *alfacarensis* Nieto Fel., Gut.Larena & Fuertes – – wfo-0001303245 – http://www.rjb.csic.es/jardinbotanico/jardin/contenido.php?Pag=219&tipo=volumenanales&vol=61(1)
Armeria filicaulis subsp. *nevadensis* Nieto Fel., Rosselló & Fuertes – – wfo-0001303246 – http://www.rjb.csic.es/jardinbotanico/jardin/contenido.php?Pag=219&tipo=volumenanales&vol=56(1)
Armeria filicaulis subsp. *trevenqueana* Nieto Fel. – – wfo-0001303247 – http://www.rjb.csic.es/jardinbotanico/jardin/contenido.php?Pag=219&tipo=volumenanales&vol=47(1)
Armeria filicaulis subsp. *valentina* (Pau ex C.Vicioso) Mateo – – wfo-0000508418
Armeria filicaulis subsp. *willkommiana* (Bernis) Molero – – wfo-0000548876
Armeria filicaulis var. *bourgaei* (Boiss. ex Merino) Pau – – wfo-0001303248
Armeria filicaulis var. *bourgeaui* Pau – – wfo-0000548871
Armeria filicaulis var. *longifolia* Willk. – – wfo-0000548872
Armeria filicaulis var. *major* Boiss. – – wfo-0000548873
Armeria filicaulis var. *maroccana* Pau & Font Quer – – wfo-0000548874
Armeria filicaulis var. *minor* Boiss. – – wfo-0000548875
Armeria filicaulis var. *valentina* Pau ex C.Vicioso – – wfo-0000508419
Armeria floridana (Benth.) Kuntze – – wfo-0000548878 – https://biodiversitylibrary.org/page/3854
Armeria fontqueri Pau – – wfo-0000548879
Armeria formosa Heynh. – – wfo-0000548880
Armeria formosa hort. ex H.Vilm. – – wfo-0000548881
Armeria foucaudii Beck – – wfo-0000548882 – https://www.biodiversitylibrary.org/page/5373849
Armeria fruticosa G.Lodd. – – wfo-0000548883
Armeria gaditana Boiss. – – wfo-0000548884 – https://biodiversitylibrary.org/page/160897
Armeria gaditana var. *boissieriana* (Coss.) G.H.M.Lawr. – – wfo-0000548885

Armeria gaditana var. *simplex* (Pomel) G.H.M.Lawr. – – wfo-0000548887
Armeria gaditana var. *spinulosa* (Boiss.) G.H.M.Lawr. – – wfo-0000548889
Armeria gaditiana var. *chamaeropicola* Pau – – wfo-0000548886
Armeria gaditiana var. *tingitana* (Boiss. & Reut.) Ball – – wfo-0000548890 – http://www.biodiversitylibrary.org/openurl?pid=title:350&volume=16&issue=&spage=560&date=1878
Armeria garganica Arrigoni – – wfo-0001443422 – https://www.herbmedit.org/flora/FL25SI_007-032.pdf
Armeria genesiana Nieto Fel. – – wfo-0000548891 – https://rjb.csic.es//jardinbotanico/ficheros/documentos/pdf/anales/1987/Anales_44(2)_319_348.pdf
Armeria genesiana subsp. *genesiana* Nieto Fel. – – wfo-0001303249
Armeria genesiana subsp. *belmonteae* (P.Silva) Nieto Fel. – – wfo-0000548892 – http://www.rjb.csic.es/jardinbotanico/ficheros/documentos/pdf/anales/1987/Anales_44(2)_319_348.pdf
Armeria genesiana subsp. *belmontei* (P.Silva) Nieto Fel. – – wfo-0001303047
Armeria girardi (Bernis) Litard. – – wfo-0000548893
Armeria girardii (Bernis) Litard. – – wfo-0001033897
Armeria glaberrima (L.) Kuntze – – wfo-0000548894 – https://biodiversitylibrary.org/page/3854
Armeria glauca Wallr. – – wfo-0000548895
Armeria glaucescens Desf. – – wfo-0000548896
Armeria globosa Link ex Boiss. – – wfo-0000548897 – http://www.biodiversitylibrary.org/openurl?pid=title:286&volume=12&issue=&spage=684&date=1848
Armeria godayana Font Quer – – wfo-0000548898
Armeria gracilis subsp. *majellensis* (Boiss.) Arrigoni – – wfo-1200027297
Armeria gracilis Ten. – – wfo-0000548900
Armeria gracilis subsp. *gracilis* Ten. – – wfo-0001303250
Armeria gracilis var. *humilis* Ten. – – wfo-0000548901
Armeria grajoana Casim.-Sor.Solanas & Cabezudo – – wfo-0001303251 – https://revistas.uma.es/index.php/abm/article/view/2493/2287
Armeria grandiflora Boiss. – – wfo-0000548902 – http://www.biodiversitylibrary.org/openurl?pid=title:286&volume=12&issue=&spage=684&date=1848
Armeria grosii Pau – – wfo-0000548903
Armeria gussonei Boiss. – – wfo-0000548904 – https://biodiversitylibrary.org/page/160909
Armeria halleri Wallr. – – wfo-0000548905
Armeria helodes F.Martini & Poldini – – wfo-0000548906
Armeria henriquesii Daveau – – wfo-0000548907
Armeria heterophylla Wallr. – – wfo-0000548908
Armeria hirta Pourr. ex Willk. & Lange – – wfo-0000548911 – http://www.biodiversitylibrary.org/openurl?pid=title:37768&volume=2&issue=&spage=369&date=1868
Armeria hirta subsp. *hispalensis* (Pau) Malag. – – wfo-0001303252
Armeria hirta subsp. *spinulosa* (Boiss.) Bernis – – wfo-0001303253
Armeria hirta var. *glauca* (Wallr.) Bernis – – wfo-0000548912
Armeria hirta var. *perplexans* Bernis – – wfo-0001303254
Armeria hirta var. *tingitana* (Boiss. & Reut.) Bernis – – wfo-0001303255
Armeria hirta Willd. – – wfo-0000548909
Armeria hirta var. *hirta* Willd. – – wfo-0001303256
Armeria hispalensis Pau – – wfo-0000548913
Armeria hordii (Rich.) Kuntze – – wfo-0000548914 – https://biodiversitylibrary.org/page/3854
Armeria humilis (Link) Schult. – – wfo-0000548915 – http://www.biodiversitylibrary.org/openurl?pid=title:825&volume=6&issue=&spage=772&date=1820
Armeria humilis subsp. *humilis* (Link) Schult. – – wfo-0001303257

Armeria humilis Link – – wfo-0000548916 – https://www.biodiversitylibrary.org/page/53266256
Armeria humilis subsp. *odorata* (Samp.) P.Silva – – wfo-0000548917
Armeria icarica Edm. – – wfo-0000548918
Armeria intermedia (T.Marsson) Szafer – – wfo-0000548921 – https://pbsociety.org.pl/journals/index.php/asbp/article/view/asbp.1946.001/6567
Armeria intermedia Link ex Boiss. – – wfo-0000548919 – http://www.biodiversitylibrary.org/openurl?pid=title:286&volume=12&issue=&spage=681&date=1848
Armeria japonica Rippa – – wfo-0000746040
Armeria johnsenii Papan. & Kokkini – – wfo-0000548922
Armeria juncea Girard – – wfo-0000548924 – https://biodiversitylibrary.org/page/41531990
Armeria juncea Wallr. – – wfo-0000548923
Armeria juniperifolia (Vahl) Hoffmanns. & Link – – wfo-0000548925 – http://www.biodiversitylibrary.org/openurl?pid=title:64&volume=6&issue=&spage=711&date=1823
Armeria juniperifolia Ebel – – wfo-0000548927
Armeria juniperifolia Hoffmanns. & Link – – wfo-0001303258
Armeria juniperifolia J.Gay ex Boiss. – – wfo-0000548928 – http://www.biodiversitylibrary.org/openurl?pid=title:286&volume=12&issue=&spage=684&date=1848
Armeria juniperifolia var. *bigerrensis* (Vicioso & Beltrán) G.H.M.Lawr. – – wfo-0000548929
Armeria juniperifolia var. *isernii* (Vicioso & Beltrán) G.H.M.Lawr. – – wfo-0000548930
Armeria juniperifolia var. *splendens* (Lag. & Rodr.) G.H.M.Lawr. – – wfo-0000548932
Armeria juniperifolia W.D.J.Koch – – wfo-0000548926 – http://www.biodiversitylibrary.org/openurl?pid=title:64&volume=6&issue=&spage=711&date=1823
Armeria kochii Boiss. – – wfo-0000548933 – http://www.biodiversitylibrary.org/openurl?pid=title:286&volume=12&issue=&spage=686&date=1848
Armeria labradorica f. *glabriscapa* (S.F.Blake) Malte – – wfo-0001244937
Armeria labradorica f. *glabriscapa* H.F.Lewis – – wfo-0000548935
Armeria labradorica f. *pubiscapa* Malte – – wfo-0000548936
Armeria labradorica subsp. *sibirica* (Turcz. ex Boiss.) Kamelin – – wfo-0000548937 – https://biodiversitylibrary.org/page/639168
Armeria labradorica var. *genuina* Malte – – wfo-0001304468
Armeria labradorica var. *submutica* H.F.Lewis – – wfo-0000548938 – http://www.biodiversitylibrary.org/openurl?pid=title:39970&volume=46&issue=4&spage=92&date=1932
Armeria labradorica Wallr. – – wfo-0000548934
Armeria lacaitae (Villar) Rivas Mart. – – wfo-0000548939
Armeria lachnolepis Pomel – – wfo-0000548940
Armeria lacmonica Hausskn. – – wfo-0000548942
Armeria lanceobracteata G.H.M.Lawr. – – wfo-0000548943
Armeria langeana Henriq. – – wfo-0000548944
Armeria langeana var. *genuina* Daveau – – wfo-0001303259 – https://bibdigital.rjb.csic.es/viewer/10622/?offset=#page=182&viewer=picture&o=bookmark&n=0&q=
Armeria langeana var. *glabra* Daveau – – wfo-0001303260 – https://bibdigital.rjb.csic.es/viewer/10622/?offset=#page=182&viewer=picture&o=bookmark&n=0&q=
Armeria langei Boiss. ex Lange – – wfo-0000548945 – http://www.biodiversitylibrary.org/openurl?pid=title:7547&volume=1861&issue=&spage=59&date=1861
Armeria langei subsp. *langei* Boiss. – – wfo-0001303261
Armeria langei subsp. *belmonteae* P.Silva – – wfo-0000548946
Armeria langei subsp. *belmontei* P.Silva – – wfo-0001303262
Armeria langei subsp. *deveaui* (Cout.) P.Silva – – wfo-0000548947
Armeria latifolia Moris – – wfo-0000548949
Armeria latifolia Willd. – – wfo-0000548948

Armeria laucheana J.N.Haage & E.Schmidt – – wfo-0001304513
Armeria leonis Sennen – – wfo-0000548950
Armeria leucantha (Boiss.) Mathon – – wfo-0000548953
Armeria leucantha Salzm. ex Boiss. – – wfo-0000548951
Armeria leucocephala Salzm. ex W.D.J.Koch – – wfo-0000548954 – http://www.biodiversitylibrary.org/openurl?pid=title:64&volume=6&issue=&spage=712&date=1823
Armeria leucocephala subsp. *breviaristata* Arrigoni – – wfo-0000496407 – https://doi.org/10.1080/00837792.1970.10669931
Armeria leucocephala subsp. *leucocephala* – – wfo-0001303263 – https://doi.org/10.1080/00837792.1970.10669931
Armeria leucocephala subsp. *multiceps* (Wallr.) Arcang. – – wfo-0000496409
Armeria leucocephala subsp. *pubescens* (Salis) Arrigoni – – wfo-0000496410 – https://doi.org/10.1080/00837792.1970.10669931
Armeria leucocephala subsp. *soleirolii* (Duby) Arcang. – – wfo-0000496412
Armeria leucocephala subsp. *thomasii* Nyman – – wfo-0000548962 – http://www.biodiversitylibrary.org/openurl?pid=title:10533&volume=&issue=&spage=651&date=1881
Armeria leucocephala var. *alpina* Boiss. – – wfo-0000548955 – http://www.biodiversitylibrary.org/openurl?pid=title:286&volume=12&issue=&spage=687&date=1848
Armeria leucocephala var. *glabra* Fiori – – wfo-0000548956
Armeria leucocephala var. *hirticula* Boenn. – – wfo-0000548957 – http://www.biodiversitylibrary.org/openurl?pid=title:359&volume=67&issue=&spage=263&date=1920
Armeria leucocephala var. *kochii* (Boiss.) G.H.M.Lawr. – – wfo-0000548958
Armeria leucocephala var. *multiceps* (Wallr.) Mori – – wfo-0000496408 – http://www.biodiversitylibrary.org/openurl?pid=title:6341&volume=8&issue=&spage=592&date=1889
Armeria leucocephala var. *procera* Boiss. – – wfo-0000548959 – http://www.biodiversitylibrary.org/openurl?pid=title:286&volume=12&issue=&spage=687&date=1848
Armeria leucocephala var. *pubescens* (Salis) G.H.M.Lawr. – – wfo-0000548960
Armeria leucocephala var. *soleirolii* (Duby) Boiss. – – wfo-0000548961 – http://www.biodiversitylibrary.org/openurl?pid=title:286&volume=12&issue=&spage=687&date=1848
Armeria linearifolia (Hook.) Kuntze – – wfo-0000548964 – https://biodiversitylibrary.org/page/3854
Armeria linkiana Nieto Fel. – – wfo-0000548965
Armeria littoralis Boiss. – – wfo-0000548969 – http://www.biodiversitylibrary.org/openurl?pid=title:286&volume=12&issue=&spage=676&date=1848
Armeria littoralis H.J.Coste – – wfo-0000548966
Armeria littoralis Hoffmanns. & Link – – wfo-0000548968
Armeria littoralis var. *ancarensis* (Merino) G.H.M.Lawr. – – wfo-0000548970
Armeria littoralis var. *hispida* Daveau – – wfo-0000548971
Armeria littoralis Willd. – – wfo-0000548967
Armeria longearistata Bourg. ex Reut. – – wfo-0000548973 – http://www.biodiversitylibrary.org/openurl?pid=title:359&volume=2&issue=&spage=643&date=1855
Armeria longevaginata Batt. – – wfo-0000548977
Armeria longiaristata Boiss. & Reut. – – wfo-0000548972 – http://bibdigital.rjb.csic.es/ing/Libro.php?Libro=20&Pagina=103
Armeria longiaristata subsp. *vestita* (Willk.) Nyman – – wfo-0000548976 – http://www.biodiversitylibrary.org/openurl?pid=title:10533&volume=&issue=&spage=615&date=1881
Armeria longifolia (Nutt.) Kuntze – – wfo-0001417142
Armeria longifolia Desf. – – wfo-0001303264
Armeria longivaginata Batt. – – wfo-0001304461
Armeria lusitanica Link ex Boiss. – – wfo-0000548978 – http://www.biodiversitylibrary.org/openurl?pid=title:286&volume=12&issue=&spage=675&date=1848
Armeria macloviana Cham. – – wfo-0000548979
Armeria macloviana subsp. *andina* (Poepp. ex Boiss.) Iversen – – wfo-0000548980
Armeria macloviana subsp. *californica* (Boiss.) Iversen – – wfo-0000548981

Armeria macrophylla Boiss. & Reut. – – wfo-0000548982
Armeria macropoda Boiss. – – wfo-0000548983 – http://www.biodiversitylibrary.org/openurl?pid=title:286&volume=12&issue=&spage=688&date=1848
Armeria maculata (L.) Kuntze – – wfo-0001418424
Armeria maculata Poepp. ex Boiss. – – wfo-0000548984 – http://www.biodiversitylibrary.org/openurl?pid=title:286&volume=12&issue=&spage=682&date=1848
Armeria maderensis Lowe – – wfo-0000548985 – http://www.biodiversitylibrary.org/openurl?pid=title:2348&volume=6&issue=&spage=12&date=1833
Armeria maghrebensis Donad. – – wfo-0000548987
Armeria maghrebensis var. *ebracteolata* Donad. – – wfo-0000548988
Armeria maghrebensis var. *mamorensis* Donad. – – wfo-0000548989
Armeria magna Sennen – – wfo-0001303265
Armeria majellensis Boiss. – – wfo-0000548990 – http://www.biodiversitylibrary.org/openurl?pid=title:286&volume=12&issue=&spage=685&date=
Armeria majellensis subsp. *ausonia* Bianchini – – wfo-0000548991
Armeria majellensis subsp. *majellensis* – – wfo-0001303266
Armeria majellensis subsp. *orphanidis* (Boiss.) Nyman – – wfo-0000548996 – http://www.biodiversitylibrary.org/openurl?pid=title:10533&volume=&issue=&spage=614&date=1881
Armeria majellensis var. *brachyphylla* Boiss. – – wfo-0000548992
Armeria majellensis var. *elatior* Levier ex Arch. – – wfo-0000548993
Armeria majellensis var. *leucantha* Boiss. – – wfo-0000548994
Armeria majellensis var. *marginata* Levier – – wfo-0000548995
Armeria majellensis var. *rhodopaea* Velen. – – wfo-0000548998
Armeria majellensis var. *stenophylla* Beck – – wfo-0000548999
Armeria majellensis var. *subalpina* Levier – – wfo-0001303267
Armeria major (Jacq.) Grande – – wfo-0000549000
Armeria malacitana Nieto Fel. – – wfo-0000549001 – http://www.rjb.csic.es/jardinbotanico/ficheros/documentos/pdf/anales/1987/Anales_44(2)_319_348.pdf
Armeria malinvaudii H.J.Coste & Soulié – – wfo-0000549002 – http://www.biodiversitylibrary.org/openurl?pid=title:359&volume=58&issue=&spage=362&date=1911
Armeria marginata (Levier) Bianchini – – wfo-0000549003
Armeria mariae Sennen – – wfo-0000549004
Armeria maritima (Mill.) Willd. – – wfo-0000549005
Armeria maritima subsp. *maritima* (Mill.) Willd. – – wfo-0001260090
Armeria maritima var. *maritima* (Mill.) Willd. – – wfo-0001095143
Armeria maritima f. *anisophylla* Bernis – – wfo-0000549017
Armeria maritima f. *aristulata* Bernis – – wfo-0000549022
Armeria maritima f. *elongata* (Hoffm.) Blytt – – wfo-0000549035
Armeria maritima f. *laucheana* (J.N.Haage & E.Schmidt) Voss – – wfo-0000549050 – https://www.biodiversitylibrary.org/page/42525307
Armeria maritima f. *legionensis* Bernis – – wfo-0000549051
Armeria maritima f. *littorea* Bernis – – wfo-0000549053
Armeria maritima f. *losae* Bernis – – wfo-0000549054
Armeria maritima f. *occasiana* Bernis – – wfo-0000549066
Armeria maritima f. *onsiensis* Bernis – – wfo-0001303268
Armeria maritima Girard ex Boiss. – – wfo-0000549006 – http://www.biodiversitylibrary.org/openurl?pid=title:286&volume=12&issue=&spage=680&date=1848
Armeria maritima subsp. *alpina* (DC.) P.Silva – – wfo-0000549011 – http://onlinelibrary.wiley.com/doi/10.1111/boj.1971.64.issue-4/issuetoc

Armeria maritima subsp. *andina* (Poepp. ex Boiss.) D.M.Moore & B.Yates – – wfo-0000549015
Armeria maritima subsp. *anglica* Wallr. – – wfo-0000549016
Armeria maritima subsp. *arctica* (Cham.) Hultén – – wfo-0000549020
Armeria maritima subsp. *azorica* Franco – – wfo-0001303269
Armeria maritima subsp. *barcensis* (Simonk.) P.Silva – – wfo-0000549024
Armeria maritima subsp. *belgica* Wallr. – – wfo-0000549025
Armeria maritima subsp. *bottendorfensis* (A.G.Schulz) Rothm. – – wfo-0000549027
Armeria maritima subsp. *bourgaei* (Boiss. ex Nyman) Bernis – – wfo-0001303270
Armeria maritima subsp. *californica* (Boiss.) Porsild – – wfo-0000549032
Armeria maritima subsp. *depilata* (Bernis) Malag. – – wfo-0001303271 – https://bibdigital.rjb.csic.es/viewer/11793/?offset=#page=24&viewer=picture&o=bookmark&n=0&q=
Armeria maritima subsp. *eifeliaca* (Petri) Lefebvre – – wfo-0001303272
Armeria maritima subsp. *elegans* (Bernis) Malag. – – wfo-0001303273
Armeria maritima subsp. *elongata* (Hoffm.) Bonnier – – wfo-0000549037
Armeria maritima subsp. *fontqueri* – – wfo-0001303274
Armeria maritima subsp. *gallica* Wallr. – – wfo-0000549038
Armeria maritima subsp. *germanica* Wallr. – – wfo-0000549039
Armeria maritima subsp. *glabrescens* (Lange ex Daveau) Malag. – – wfo-0001303275
Armeria maritima subsp. *halleri* (Wallr.) Rothm. – – wfo-0000549041
Armeria maritima subsp. *hornburgensis* (A.G.Schulz) Rothm. – – wfo-0000549042
Armeria maritima subsp. *interior* (Raup) A.E.Porsild – – wfo-0000549045
Armeria maritima subsp. *interior* (Raup) Lefebvre & Vekem. – – wfo-0001303276
Armeria maritima subsp. *intermedia* (T.Marsson) C.Lefèbvre ex Buttler – – wfo-0001303277
Armeria maritima subsp. *itala* Wallr. – – wfo-0000549047
Armeria maritima subsp. *juniperifolia* (Bernis) Malag. – – wfo-0001303278 – https://bibdigital.rjb.csic.es/viewer/11793/?offset=#page=24&viewer=picture&o=bookmark&n=0&q=
Armeria maritima subsp. *labradorica* (Wallr.) Hultén – – wfo-0000549049
Armeria maritima subsp. *legionensis* (Bernis) M.Laínz – – wfo-0000549052
Armeria maritima subsp. *maderensis* (Loew) Bernis – – wfo-0000549057
Armeria maritima subsp. *majuscula* (Samp.) Malag. – – wfo-0001303279 – https://bibdigital.rjb.csic.es/viewer/11793/?offset=#page=24&viewer=picture&o=bookmark&n=0&q=
Armeria maritima subsp. *miscella* (Merino) Malag. – – wfo-0000549063
Armeria maritima subsp. *mulleri* (A.Huet) O.Bolòs & Vigo – – wfo-0000549064
Armeria maritima subsp. *odorata* (Samp.) Bernis – – wfo-0000549068
Armeria maritima subsp. *planifolia* (Syme) Á.Löve & D.Löve – – wfo-0001303280
Armeria maritima subsp. *pubigera* (Desf.) Malag. – – wfo-0001303281
Armeria maritima subsp. *purpurea* (W.D.J.Koch) Á.Löve & D.Löve – – wfo-0000549079
Armeria maritima subsp. *serpentini* (Gauckler) Rothm. – – wfo-0000549086
Armeria maritima subsp. *sibirica* (Turcz. ex Boiss.) Nyman – – wfo-0000549088 – https://biodiversitylibrary.org/page/11015760
Armeria maritima subsp. *smolikana* Babal. – – wfo-0001303282
Armeria maritima subvar. *carratracensis* Bernis – – wfo-0001303283
Armeria maritima subvar. *confusa* Bernis – – wfo-0001303284
Armeria maritima subvar. *elegans* Bernis – – wfo-0001303285
Armeria maritima subvar. *gracilifolia* Bernis – – wfo-0001303286
Armeria maritima subvar. *javalambrica* Bernis – – wfo-0001303287

Armeria maritima subvar. *juniperifolia* Bernis – – wfo-0001303288
Armeria maritima subvar. *littorifuga* Bernis – – wfo-1200093585
Armeria maritima subvar. *marginata* (Levier) Bernis – – wfo-0001303289
Armeria maritima subvar. *protypica* Bernis – – wfo-0001303290
Armeria maritima subvar. *salvadorii* Bernis – – wfo-0001303291
Armeria maritima var. *albi* Bernis – – wfo-0001303292
Armeria maritima var. *alboi* Bernis – – wfo-0000549007
Armeria maritima var. *allioides* Bernis – – wfo-0000549009
Armeria maritima var. *alpina* (DC.) Bernis – – wfo-0001303293
Armeria maritima var. *alpina* (Willd.) G.H.M.Lawr. – – wfo-0000549010
Armeria maritima var. *alpinifolia* (Pau & Font Quer) G.H.M.Lawr. – – wfo-0000549012
Armeria maritima var. *ambifaria* (Focke) G.H.M.Lawr. – – wfo-0000549013
Armeria maritima var. *andina* (Poepp. ex Boiss.) G.H.M.Lawr. – – wfo-0000549014
Armeria maritima var. *anomala* Bernis – – wfo-0000549018
Armeria maritima var. *arctica* (Cham.) Bernis – – wfo-0000549021 – http://www.rjb.csic.es/jardinbotanico/jardin/contenido.php?Pag=219&tipo=volumenanales&vol=11(2)
Armeria maritima var. *barcensis* (Simonk.) G.H.M.Lawr. – – wfo-0000549023
Armeria maritima var. *bilbilitana* Bernis – – wfo-0000549026
Armeria maritima var. *bourgaei* (Boiss. ex Nyman) Bernis – – wfo-0000549029
Armeria maritima var. *caballeroi* Bernis – – wfo-0000549030
Armeria maritima var. *californica* (Boiss.) G.H.M.Lawr. – – wfo-0000549031
Armeria maritima var. *canescens* (Host) Bernis – – wfo-0001303294
Armeria maritima var. *curvifolia* (Bertero) G.H.M.Lawr. – – wfo-0000549033
Armeria maritima var. *denticulata* (Bertol.) Bernis – – wfo-0001303295
Armeria maritima var. *depilata* Bernis – – wfo-0001303296
Armeria maritima var. *duriuscula* (Bab.) Bab. – – wfo-0000549034
Armeria maritima var. *genuina* Gren & Godr. – – wfo-0001303297
Armeria maritima var. *girardii* Bernis – – wfo-0000747215
Armeria maritima var. *goodalliana* T.R.Dudley – – wfo-0000549040 – http://www.biodiversitylibrary.org/openurl?pid=title:721&volume=83&issue=836&spage=488&date=1981
Armeria maritima var. *hortensis* Wallr. – – wfo-0000549043
Armeria maritima var. *interior* (Raup) G.H.M.Lawr. – – wfo-0000549044
Armeria maritima var. *labradorica* (Wallr.) G.H.M.Lawr. – – wfo-0000549048
Armeria maritima var. *leucocephala* (Salzm. ex W.D.J.Koch) Bernis – – wfo-0000496413
Armeria maritima var. *linkii* Gren & Godr. – – wfo-0001303298
Armeria maritima var. *macloviana* (Cham.) G.H.M.Lawr. – – wfo-0000549055
Armeria maritima var. *macropoda* (Boiss.) Bernis – – wfo-0001303299
Armeria maritima var. *magellanica* (Boiss.) G.H.M.Lawr. – – wfo-0000549058
Armeria maritima var. *majellensis* (Boiss.) Bernis – – wfo-0001303300
Armeria maritima var. *maria* G.H.M.Lawr. – – wfo-0000549059
Armeria maritima var. *maroccana* (Font Quer) G.H.M.Lawr. – – wfo-0000549060
Armeria maritima var. *matritensis* (Pau) Bernis – – wfo-0000549061
Armeria maritima var. *merinoi* Bernis – – wfo-0000549062
Armeria maritima var. *morisii* (Boiss.) Bernis – – wfo-0000496414
Armeria maritima var. *multiceps* (Wallr.) Bernis – – wfo-0000496415

Armeria maritima var. *occasiana* (Bernis) O.Bolòs & Vigo – – wfo-0000549067
Armeria maritima var. *patagonica* (Phil.) G.H.M.Lawr. – – wfo-0000549069
Armeria maritima var. *pauana* Bernis – – wfo-0000549070
Armeria maritima var. *planifolia* (Syme) Bab. – – wfo-0000549071
Armeria maritima var. *profilicaulis* Bernis – – wfo-0000549072
Armeria maritima var. *provillosa* Bernis – – wfo-0000549075
Armeria maritima var. *pubescens* (Link) Bab. – – wfo-0000549076
Armeria maritima var. *pubescens* (Sowerby) Rchb.
Armeria maritima var. *pubigera* (Boiss.) Bab. – – wfo-0000549077
Armeria maritima var. *purpurea* (W.D.J.Koch) G.H.M.Lawr. – – wfo-0000549078
Armeria maritima var. *rumelicina* Bernis – – wfo-0000549080
Armeria maritima var. *ruscinonensis* (Girard) G.H.M.Lawr. – – wfo-0000549081
Armeria maritima var. *salmantica* Bernis – – wfo-0000549082
Armeria maritima var. *sampaioi* Bernis – – wfo-0000549083
Armeria maritima var. *sardoa* (Spreng.) Bernis – – wfo-0000496416
Armeria maritima var. *scotica* (Boiss.) Bab. – – wfo-0001303301
Armeria maritima var. *scotica* (Boiss.) P.D.Sell – – wfo-0001303302
Armeria maritima var. *segoviensis* Gand. ex Bernis – – wfo-0000549085
Armeria maritima var. *serpentini* Gauckler – – wfo-0000549087
Armeria maritima var. *sibirica* (Turcz. ex Boiss.) A.Blytt – – wfo-0000741784
Armeria maritima var. *soleirolii* (Duby) Bernis – – wfo-0000496417
Armeria maritima var. *sylvestris* Wallr. – – wfo-0000549089
Armeria maritima var. *variegata* hort. ex F.T.Hubb. – – wfo-0001303303
Armeria maritima var. *viciosoi* Bernis – – wfo-0000549090
Armeria maritima var. *willkommiana* Bernis – – wfo-0000549091
Armeria masguindalii (Pau) Nieto Fel. – – wfo-0000549093
Armeria matritensis Pau – – wfo-0000549094
Armeria mauritanica Boiss. – – wfo-0000549096 – http://www.biodiversitylibrary.org/openurl?pid=title:286&volume=12&issue=&spage=674&date=1848
Armeria mauritanica var. *amplifoliata* (Pau) G.H.M.Lawr. – – wfo-0000549097
Armeria mauritanica var. *boissieriana* (Coss.) Quézel & Santa – – wfo-0000549098
Armeria mauritanica var. *calva* Boiss. – – wfo-0000549099 – http://www.biodiversitylibrary.org/openurl?pid=title:286&volume=12&issue=&spage=677&date=1848
Armeria mauritanica var. *chamaeropicola* (Pau) Bernis – – wfo-0000549100 – http://www.rjb.csic.es/jardinbotanico/ficheros/documentos/pdf/anales/1954/Anales_11(2)_005_287.pdf
Armeria mauritanica var. *ciliolata* Boiss. – – wfo-0000549101 – http://www.biodiversitylibrary.org/openurl?pid=title:286&volume=12&issue=&spage=677&date=1848
Armeria mauritanica var. *minor* Batt. – – wfo-0000549103
Armeria mauritanica var. *safiensis* Maire – – wfo-0000549104
Armeria mauritanica var. *simplex* (Pomel) Faure – – wfo-0000549105
Armeria mauritanica var. *soloitana* Maire – – wfo-0000549106
Armeria mauritanica Wallr. – – wfo-0000549095
Armeria meridionalis Poepp. ex Boiss. – – wfo-0000549107 – http://www.biodiversitylibrary.org/openurl?pid=title:286&volume=12&issue=&spage=682&date=1848
Armeria merinoi (Bernis) Nieto Fel. & Silva Pando – – wfo-0000549108 – http://www.rjb.csic.es/jardinbotanico/ficheros/documentos/pdf/anales/1987/Anales_44(2)_319_348.pdf
Armeria microcephala Welw. – – wfo-0000549109

Armeria miscella Merino – – wfo-0000549110
Armeria montana (Mill.) F.Herm. – – wfo-0001303304
Armeria montana f. *sicorisiensis* Sennen – – wfo-0000549114
Armeria montana G.Don ex Loudon – – wfo-0000549112 – https://www.biodiversitylibrary.org/page/10904315
Armeria montana Ray ex Wallr. – – wfo-0000549113 – https://www.digitale-sammlungen.de/en/view/bsb10303815?page=228
Armeria montana var. *stenophylla* Rouy – – wfo-0000549115
Armeria montcaunica Pau ex Losa – – wfo-0000549116
Armeria montiberica García Cardo, Fabado & Mateo – – wfo-1000055380
Armeria morisii Boiss. – – wfo-0000549117 – http://www.biodiversitylibrary.org/openurl?pid=title:286&volume=12&issue=&spage=687&date=1848
Armeria morisii var. *gussonei* (Boiss.) Parl. – – wfo-0000549118 – http://www.biodiversitylibrary.org/openurl?pid=title:6341&volume=8&issue=&spage=600&date=1889
Armeria morisii var. *macropoda* (Boiss.) Parl. – – wfo-0000549119 – http://www.biodiversitylibrary.org/openurl?pid=title:6341&volume=8&issue=&spage=600&date=1889
Armeria morisii var. *maderensis* (Lowe ex Boiss.) G.H.M.Lawr. – – wfo-0000549120
Armeria muelleri A.Huet – – wfo-0000549122 – http://www.biodiversitylibrary.org/openurl?pid=title:5010&volume=19&issue=&spage=255&date=1853
Armeria mulleri A.Huet – – wfo-0001303305 – https://www.biodiversitylibrary.org/page/41552274
Armeria multiceps subsp. *meridionalis* Arrigoni – – wfo-0000496418 – https://doi.org/10.1080/00837792.1970.10669931
Armeria multiceps subsp. *multiceps* – – wfo-0001303306 – https://doi.org/10.1080/00837792.1970.10669931
Armeria multiceps Wallr. – – wfo-0000549123
Armeria muscodes (Nutt.) Kuntze – – wfo-0000549124 – https://biodiversitylibrary.org/page/3854
Armeria nana (Nutt.) Kuntze – – wfo-0000549125 – https://biodiversitylibrary.org/page/3854
Armeria nebrodensis (Guss.) Boiss. – – wfo-0000549126
Armeria neglecta Girard – – wfo-0000549127 – https://biodiversitylibrary.org/page/41531990
Armeria nuriensis Sennen – – wfo-0001303307
Armeria odorata Samp. – – wfo-0000549129
Armeria orophila Sennen – – wfo-0001303308
Armeria orphanidis Boiss. – – wfo-0000549130 – http://bibdigital.rjb.csic.es/ing/Libro.php?Libro=1418&Pagina=73
Armeria ovata (L.) Kuntze – – wfo-0000549131
Armeria paniculata (L.) Kuntze – – wfo-0000549133 – https://biodiversitylibrary.org/page/3854
Armeria pantocsekii (Strobl) K.Malý – – wfo-0001303309
Armeria parvula Franco – – wfo-0000549134
Armeria patagonica Phil. – – wfo-0000549135
Armeria pauana (Bernis) Nieto Fel. – – wfo-0000549136 – http://www.rjb.csic.es/jardinbotanico/ficheros/documentos/pdf/anales/1987/Anales_44(2)_319_348.pdf
Armeria petri-ludovicii Sennen – – wfo-0000549137
Armeria petteriana C.Presl – – wfo-0000549138 – https://biodiversitylibrary.org/page/45617016
Armeria pilosa (L.) Kuntze – – wfo-0000549139 – https://biodiversitylibrary.org/page/3854
Armeria pinifolia (Brot.) Hoffmanns. & Link – – wfo-0000549140
Armeria pinifolia Bourg. ex Nyman – – wfo-0000549141 – https://biodiversitylibrary.org/page/11015758
Armeria pinifolia subsp. *macrophylla* (Boiss. & Reut.) Bernis – – wfo-0001303310
Armeria piorum Sennen – – wfo-0001303311
Armeria planifolia Nyman – – wfo-0000549142 – https://biodiversitylibrary.org/page/11015760
Armeria plantaginea – – wfo-0001303312
Armeria plantaginea (All.) Willd. – – wfo-0000549144 – http://bibdigital.rjb.csic.es/ing/Libro.php?Libro=1684&Pagina=341

Armeria plantaginea Bertol. – – wfo-0001303313
Armeria plantaginea Boiss. – – wfo-0000549146
Armeria plantaginea subsp. *alliacea* (Cav.) Malag. – – wfo-0001303314
Armeria plantaginea subsp. *bupleuroides* (Gren. & Godr.) Nyman – – wfo-0000549149 – https://biodiversitylibrary.org/page/11015760
Armeria plantaginea subsp. *burgalensis* (Sennen & Elias) Malag. – – wfo-0001303315
Armeria plantaginea subsp. *castellana* (Boiss. & Reut. ex Leresche) Nyman – – wfo-0000549151 – https://www.biodiversitylibrary.org/search?SearchTerm=Consp.+fl.+eur.&lname=&vol=&ed=&yr=&subj=&lang=&col=&SearchCat=T&return=ADV#/titles
Armeria plantaginea subsp. *choulettiana* (Pomel) Sauvage & Vindt – – wfo-0000549152
Armeria plantaginea subsp. *leucantha* (Boiss.) Sauvage & Vindt – – wfo-0001303316
Armeria plantaginea subsp. *magna* (Sennen) Malag. – – wfo-0001303317
Armeria plantaginea subsp. *montcaunica* (Pau ex Losa) Malag. – – wfo-0001303318
Armeria plantaginea subsp. *oretana* (Bernis) Malag. – – wfo-0000549157 – https://bibdigital.rjb.csic.es/viewer/11793/?offset=#page=26&viewer=picture&o=bookmark&n=0&q=
Armeria plantaginea subsp. *piorum* (Sennen) Malag. – – wfo-0001303319
Armeria plantaginea subsp. *plantaginea* – – wfo-0001303320
Armeria plantaginea subsp. *praecox* (Jord.) Nyman – – wfo-0000549158 – http://www.biodiversitylibrary.org/openurl?pid=title:10533&volume=&issue=&spage=616&date=1881
Armeria plantaginea subsp. *rigida* (Wallr.) Malag. – – wfo-0001303321
Armeria plantaginea subsp. *segoviensis* (Gand. ex Bernis) Rivas Mart. – – wfo-0000549160
Armeria plantaginea subsp. *sicorisiensis* (Sennen) Malag. – – wfo-0001303322 – https://bibdigital.rjb.csic.es/viewer/11793/?offset=#page=26&viewer=picture&o=bookmark&n=0&q=
Armeria plantaginea var. *alba* hort. ex F.T.Hubb. – – wfo-0001303323
Armeria plantaginea var. *brachylepis* Boiss. – – wfo-0000549147 – http://www.biodiversitylibrary.org/openurl?pid=title:286&volume=12&issue=&spage=683&date=1848
Armeria plantaginea var. *brachyphylla* Boiss. – – wfo-0000549148 – http://www.biodiversitylibrary.org/openurl?pid=title:286&volume=12&issue=&spage=683&date=1848
Armeria plantaginea var. *bupleuroides* (Gren. & Godr.) G.H.M.Lawr. – – wfo-0000549150
Armeria plantaginea var. *gigantea* hort. ex F.T.Hubb. – – wfo-0001303324
Armeria plantaginea var. *grandiflora* hort. ex F.T.Hubb. – – wfo-0001303325
Armeria plantaginea var. *leucantha* Boiss. – – wfo-0000549153 – http://www.biodiversitylibrary.org/openurl?pid=title:286&volume=12&issue=&spage=683&date=1848
Armeria plantaginea var. *longibracteata* Boiss. – – wfo-0000549155 – http://www.biodiversitylibrary.org/openurl?pid=title:286&volume=12&issue=&spage=683&date=1848
Armeria plantaginea var. *masguindalii* Pau – – wfo-0000549156
Armeria plantaginea var. *rosea* hort. ex F.T.Hubb. – – wfo-0001303326
Armeria plantaginea var. *rubra* hort. ex F.T.Hubb. – – wfo-0001303327
Armeria plantaginea var. *scorzonerifolia* Boiss. – – wfo-0000549159 – http://www.biodiversitylibrary.org/openurl?pid=title:286&volume=12&issue=&spage=683&date=1848
Armeria plantaginea var. *splendens* hort. ex F.T.Hubb. – – wfo-0001303328
Armeria plantaginea Webb – – wfo-0000549145
Armeria platyphylla (Daveau) Franco – – wfo-0000549161
Armeria pocutica Pawł. – – wfo-0000549162
Armeria pocutica subsp. *alpina* (Willd.) P.Silva – – wfo-0001303329
Armeria portensis G.H.M.Lawr. – – wfo-0000549163
Armeria praecox Jord. In Boreau – – wfo-0000549164 – https://babel.hathitrust.org/cgi/pt?id=hvd.32044106333941&view=1up&seq=101&skin=2021
Armeria pseudarmeria (Murray) Mansf. – – wfo-0000549166
Armeria pseudarmeria subsp. *pseudarmeria* (Murray) Mansf. – – wfo-0001303330

Armeria pseudarmeria Murray – – wfo-0001303331
Armeria pseud-armeria Murray – – wfo-0001303332
Armeria pseudarmeria subsp. *alboi* (Bernis) Malag. – – wfo-0001303333
Armeria pseudarmeria subsp. *allioides* (Boiss.) Malag. – – wfo-0001303334
Armeria pseudarmeria subsp. *bourgaei* (Boiss. ex Merino) Malag. – – wfo-0001303335
Armeria pseudarmeria subsp. *carratracensis* (Bernis) Malag. – – wfo-0001303336
Armeria pseudarmeria subsp. *castellata* (Boiss. & Reut. ex Leresche) Malag. – – wfo-0001303337
Armeria pseudarmeria subsp. *ciliata* (Lange) Malag. – – wfo-0001303338
Armeria pseudarmeria subsp. *eriophylla* (Willk.) Malag. – – wfo-0001303339
Armeria pseudarmeria subsp. *littoralis* (Hoffmanns. & Link) Malag. – – wfo-0001303340 – https://bibdigital.rjb.csic.es/viewer/11793/?offset=#page=27&viewer=picture&o=bookmark&n=0&q=
Armeria pseudarmeria subsp. *longifolia* (Willk.) Malag. – – wfo-0001303341
Armeria pseudarmeria subsp. *monchiquensis* (Bernis) Malag. – – wfo-0001303342
Armeria pseudarmeria subsp. *pauana* (Bernis) Malag. – – wfo-0001303343 – https://bibdigital.rjb.csic.es/viewer/11793/?offset=#page=25&viewer=picture&o=bookmark&n=0&q=
Armeria pseudarmeria subsp. *provillosa* (Bernis) Malag. – – wfo-0001303344
Armeria pseudarmeria subsp. *sampaioi* (Bernis) Malag. – – wfo-0001303345
Armeria pseudarmeria subsp. *sobrinhoi* (Bernis) Malag. – – wfo-0001303346 – https://bibdigital.rjb.csic.es/viewer/11793/?offset=#page=25&viewer=picture&o=bookmark&n=0&q=
Armeria pseudarmeria subsp. *tejedensis* (Bernis) Malag. – – wfo-0001303347 – https://bibdigital.rjb.csic.es/viewer/11793/?offset=#page=25&viewer=picture&o=bookmark&n=0&q=
Armeria pseudarmeria subsp. *transmontana* (Samp.) Malag. – – wfo-0001303348
Armeria pseudarmeria subsp. *villosa* (Girard) Malag. – – wfo-0001303349 – https://bibdigital.rjb.csic.es/viewer/11793/?offset=#page=25&viewer=picture&o=bookmark&n=0&q=
Armeria pseudarmeria subvar. *carratracensis* Bernis – – wfo-0001303350
Armeria pseudarmeria subvar. *tejedensis* Bernis – – wfo-0001303351
Armeria pseudarmeria var. *alboi* Bernis – – wfo-0001303352
Armeria pseudarmeria var. *pauana* Bernis – – wfo-0001303353
Armeria pseudarmeria var. *provillosa* Bernis – – wfo-0001303354
Armeria pseudarmeria var. *sampaioi* Bernis – – wfo-0001303355
Armeria pseudarmeria var. *sobrinhoi* Bernis – – wfo-0001303356
Armeria pseudoarmeria Brot. – – wfo-0001033898 – https://biodiversitylibrary.org/page/31323713
Armeria pubescens B.Heyne ex Steud. – – wfo-0000549168
Armeria pubescens Link – – wfo-0000549167
Armeria pubescens subsp. *pubescens* Link – – wfo-0001303357
Armeria pubescens subsp. *expansa* (Wallr.) Nyman – – wfo-0000549169 – http://www.biodiversitylibrary.org/openurl?pid=title:10533&volume=&issue=&spage=616&date=1881
Armeria pubigera (Desf.) Boiss. – – wfo-0000549170 – https://biodiversitylibrary.org/page/160900
Armeria pubigera var. *glabrescens* Lange ex Daveau – – wfo-0001303358 – https://bibdigital.rjb.csic.es/viewer/10622/?offset=#page=182&viewer=picture&o=bookmark&n=0&q=
Armeria pubigera var. *hirta* Lange ex Daveau – – wfo-0001303359 – https://bibdigital.rjb.csic.es/viewer/10622/?offset=#page=182&viewer=picture&o=bookmark&n=0&q=
Armeria pubigera var. *scotica* Boiss. – – wfo-0000549171 – http://www.biodiversitylibrary.org/openurl?pid=title:286&volume=12&issue=&spage=678&date=1848
Armeria pubinervis Boiss. – – wfo-0000549172 – http://www.biodiversitylibrary.org/openurl?pid=title:286&volume=12&issue=&spage=688&date=1848
Armeria pubinervis subsp. *orissonensis* Donad. – – wfo-0000549173
Armeria pungens (Brot.) Hoffmanns. & Link – – wfo-0000549174
Armeria pungens var. *pungens* (Brot.) Hoffmanns. & Link – – wfo-0001303360

Armeria pungens subsp. *bernisiana* Malag. – – wfo-0001303361 – https://bibdigital.rjb.csic.es/viewer/11793/?offset=#page=27&viewer=picture&o=bookmark&n=0&q=
Armeria pungens subsp. *major* (Daveau) Franco – – wfo-0000549180
Armeria pungens var. *aciculifolia* Franco – – wfo-0000549177
Armeria pungens var. *major* Daveau – – wfo-0000549179 – https://bibdigital.rjb.csic.es/viewer/10622/?offset=#page=174&viewer=picture&o=bookmark&n=0&q=
Armeria pungens var. *velutina* Cout. – – wfo-0001303362 – https://biodiversitylibrary.org/page/11470332
Armeria purpurea W.D.J.Koch – – wfo-0000549181 – http://www.biodiversitylibrary.org/openurl?pid=title:64&volume=6&issue=2&spage=710&date=1823
Armeria quichiotis (Gonz.Albo) A.W.Hill – – wfo-0000549182
Armeria reptans (Michx.) Kuntze – – wfo-0000549183
Armeria rhenana Gremli – – wfo-0000549184
Armeria rhodopea Velen. – – wfo-0000549185 – https://www.biodiversitylibrary.org/page/33897584
Armeria richardsonii (Hook.) Kuntze – – wfo-0000549187 – https://biodiversitylibrary.org/page/3854
Armeria rigida f. *asperrima* Sennen – – wfo-0001303363
Armeria rigida f. *elongata* Sennen – – wfo-0001303364
Armeria rigida subsp. *latifolia* Wallr. – – wfo-0000549189
Armeria rigida Wallr. – – wfo-0000549188
Armeria rivasmartinezii Sard.Rosc. & Nieto Fel. – – wfo-0000549190
Armeria roemeriana (Scheele) Kuntze – – wfo-0000549191 – https://biodiversitylibrary.org/page/3854
Armeria rothmaleri Nieto Fel. – – wfo-0000549192
Armeria rouyana Daveau – – wfo-0000549193 – https://bibdigital.rjb.csic.es/viewer/10622/?offset=#page=177&viewer=picture&o=bookmark&n=0&q=
Armeria rouyana subsp. *rouyana* Daveau – – wfo-0001303365
Armeria rouyana f. *littorea* (Bernis) Bernis – – wfo-0000549194
Armeria rouyana subsp. *littorea* (Bernis) Malag. – – wfo-0000549195 – https://bibdigital.rjb.csic.es/viewer/11793/?offset=#page=24&viewer=picture&o=bookmark&n=0&q=
Armeria rumelica Boiss. – – wfo-0000549196 – http://www.biodiversitylibrary.org/openurl?pid=title:286&volume=12&issue=&spage=677&date=1848
Armeria rumelica f. *adamovicii* (Halácsy) Hayek – – wfo-0000549198
Armeria rumelica f. *isophylla* Stoj. & Jordanov ex Novák – – wfo-0000549200
Armeria rumelica f. *rhodopaea* (Velen.) Ančev – – wfo-0000549204
Armeria rumelica f. *rhodopaea* (Velen.) Hayek – – wfo-0000549203
Armeria rumelica f. *setacea* Novák – – wfo-0000549205
Armeria rumelica f. *temskyana* (Degen & Dörfl.) Micevski – – wfo-0001303366
Armeria rumelica var. *adamovicii* (Halácsy) Novák – – wfo-0000549199
Armeria rumelica var. *pseudocanescens* (Halácsy) Novák – – wfo-0000549201
Armeria rumelica var. *rhodopaea* (Velen.) Beck – – wfo-0000549202 – https://www.biodiversitylibrary.org/page/5373845
Armeria rumelica var. *tempskyana* (Degen & Dörfl.) Vandas – – wfo-0000549206
Armeria ruscinonensis Girard – – wfo-0000549207 – https://biodiversitylibrary.org/page/41531990
Armeria ruscinonensis subsp. *ruscinonensis* Girard – – wfo-0001303367
Armeria ruscinonensis subsp. *littorifuga* (Bernis) Malag. – – wfo-0000549209
Armeria ruscinonensis subvar. *littorifuga* Bernis – – wfo-0001303368
Armeria sabulosa Jord. ex Boreau – – wfo-0000549210
Armeria sabulosa var. *serpentini* Legrand – – wfo-0000549211
Armeria sampaioi (Bernis) Nieto Fel. – – wfo-0000549213 – http://www.rjb.csic.es/jardinbotanico/ficheros/documentos/pdf/anales/1987/Anales_44(2)_319_348.pdf
Armeria sancta Janka – – wfo-0000549214

Armeria sanguinolenta Wallr. – – wfo-0000549215
Armeria sardoa Spreng. – – wfo-0000549216
Armeria sardoa subsp. *sardoa* Spreng. – – wfo-0001303369 – https://doi.org/10.1080/00837792.1970.10669931
Armeria sardoa subsp. *genargentea* Arrigoni – – wfo-0001303370 – https://doi.org/10.1080/00837792.1970.10669931
Armeria sardoa var. *nebrodensis* (Guss.) Parl. – – wfo-0000549217
Armeria saviana Selvi – – wfo-0000743319
Armeria scabra Kunze ex Boiss. – – wfo-0000549220 – https://biodiversitylibrary.org/page/160904
Armeria scabra Pall. ex Schult. – – wfo-0000549218 – http://www.biodiversitylibrary.org/openurl?pid=title:825&volume=6&issue=&spage=776&date=1820
Armeria scabra subsp. *arctica* (Cham.) Iversen – – wfo-0000549221
Armeria scabra subsp. *labradorica* (Wallr.) Iversen – – wfo-0000549223
Armeria scabra subsp. *sibirica* (Turcz. ex Boiss.) Hyl. – – wfo-0000549225
Armeria scabra var. *glabricalyx* Tzvelev – – wfo-0000549222
Armeria scabra var. *labradorica* (Wallr.) Tzvelev – – wfo-0000549224
Armeria scabra Willd. – – wfo-0001095142
Armeria scabriuscula Kunze ex Boiss. – – wfo-0000549226 – https://biodiversitylibrary.org/page/160904
Armeria scorzonerifolia Balb. & Nocca – – wfo-0001303371
Armeria scorzonerifolia Friv. ex Nyman – – wfo-0000549231 – https://biodiversitylibrary.org/page/11015759
Armeria scorzonerifolia Link – – wfo-0000549227
Armeria scorzonerifolia Ten. – – wfo-0000549230
Armeria scorzonerifolia Willd. – – wfo-0000549229
Armeria sect. *Macrocentron* Boiss. – – wfo-0001304503
Armeria sect. *Plagiobasis* Boiss. – – wfo-0001304504
Armeria segoviensis Gand. – – wfo-0000549232
Armeria segoviensis subsp. *lacaitae* (Villar) Rivas Mart. – – wfo-0001303372
Armeria sennenii G.H.M.Lawr. – – wfo-0000549233 – https://hdl.handle.net/2027/inu.30000046319335?urlappend=%3Bseq=427
Armeria setacea Delile ex Nyman – – wfo-0000549234 – https://biodiversitylibrary.org/page/11015760
Armeria seticeps Rchb. – – wfo-0000549235
Armeria seticeps Rchb. f. – – wfo-0001303373
Armeria sibirica (L.) Kuntze – – wfo-0001419129
Armeria sibirica Turcz. ex Boiss. – – wfo-0000549236 – http://www.biodiversitylibrary.org/openurl?pid=title:286&volume=12&issue=&spage=678&date=1848
Armeria sicorisiana Sennen – – wfo-0000549237
Armeria sicula Heldr. ex Boiss. – – wfo-0000549239 – http://www.biodiversitylibrary.org/openurl?pid=title:286&volume=12&issue=&spage=685&date=1848
Armeria simplex Pomel – – wfo-0000549240
Armeria sobrinhoi subvar. *monchiquensis* Bernis – – wfo-0001303374
Armeria soleirolii (Duby) Godr. – – wfo-0000549241
Armeria speciosa (Pursh) Kuntze – – wfo-0000549242 – https://biodiversitylibrary.org/page/3854
Armeria spinulosa Boiss. – – wfo-0000549243 – http://www.biodiversitylibrary.org/openurl?pid=title:286&volume=12&issue=&spage=675&date=1848
Armeria splendens (Lag. & Rodr.) Webb – – wfo-0001303049
Armeria splendens Boiss. – – wfo-0000549244
Armeria splendens subsp. *bigerrensis* (Vicioso & Beltrán) P.Silva – – wfo-0000549245
Armeria splendens subsp. *splendens* – – wfo-0001303375
Armeria stellaria (A.Gray) Kuntze – – wfo-0000549246 – https://biodiversitylibrary.org/page/3854

Armeria stenophylla Girard – – wfo-0000549247 – https://biodiversitylibrary.org/page/41531990
Armeria subsect. *Astegiae* Boiss. – – wfo-0001304505
Armeria subsect. *Holotricae* Boiss. – – wfo-0001304506
Armeria subsect. *Macrostegiae* Boiss. – – wfo-0001304507
Armeria subsect. *Microstegiae* Boiss. – – wfo-0001304508
Armeria subsect. *Pleurotrichae* Boiss. – – wfo-0001304509
Armeria subulata (L.) Kuntze – – wfo-0000549248 – https://biodiversitylibrary.org/page/3854
Armeria sulcitana Arrigoni – – wfo-0000549250 – https://doi.org/10.1080/00837792.1970.10669931
Armeria tenorei (Fiori) Lacaita – – wfo-0000549251
Armeria tenuifolia Phil. – – wfo-0000549252
Armeria tenuis Balb. ex Boiss. – – wfo-0000549254 – http://www.biodiversitylibrary.org/openurl?pid=title:286&volume=12&issue=&spage=685&date=1848
Armeria tenuis var. *elata* Wallr. – – wfo-0000549255
Armeria tenuis var. *humilis* Wallr. – – wfo-0000549256
Armeria tenuis Wallr. – – wfo-0000549253
Armeria thessala (Boiss.) Boiss. & Heldr. – – wfo-0000549257 – http://bibdigital.rjb.csic.es/ing/Libro.php?Libro=1418&Pagina=72
Armeria thomasii Nyman – – wfo-0000549258 – https://biodiversitylibrary.org/page/11015759
Armeria tingitana Boiss. & Reut. – – wfo-0000549259
Armeria tingitana var. *chamaeropicola* (Pau) Donad. – – wfo-0000549261
Armeria trachyphylla Lange – – wfo-0000549262
Armeria transmontana (Samp.) G.H.M.Lawr. – – wfo-0000549263
Armeria transmontana subsp. *transmontana* (Samp.) G.H.M.Lawr. – – wfo-0001303377
Armeria transmontana subsp. *aristulata* (Bernis) Bernis – – wfo-0000549264
Armeria transmontana subsp. *pseudotransmontana* Franco – – wfo-0000549265
Armeria trianoi Nieto Fel. – – wfo-0000549266
Armeria trigoloides Ebel – – wfo-0000549267
Armeria trigonoides Ebel – – wfo-0001303378 – https://books.google.de/books?id=tR4yAQAAMAAJ&pg=PT5&lpg=PT5&dq=Armeria+glaucescens+Desf.&source=bl&ots=qx29_HFQbO&sig=ACfU3U27TNZ9UURsHXHlmHxqPpxPCB42Ow&hl=en&sa=X&ved=2ahUKEwiJyNGQ_9L3AhW0RPEDHV9ZBuUQ6AF6BAgiEAM#v=onepage&q=Armeria%20glaucescens%20Desf.
Armeria trojana Bokhari & Quézel – – wfo-0000549268
Armeria undulata (Bory & Chaub.) Boiss. – – wfo-0000549269 – http://www.biodiversitylibrary.org/openurl?pid=title:286&volume=12&issue=&spage=685&date=1848
Armeria undulata f. *brevifolia* Novák – – wfo-0000549270
Armeria undulata f. *longifolia* Novák – – wfo-0000549272
Armeria undulata var. *capitella* (Pau) Rivas Goday & Bellot – – wfo-0001303379
Armeria vandasii Hayek – – wfo-0000549273 – http://www.biodiversitylibrary.org/openurl?pid=title:276&volume=21&issue=&spage=257&date=1925
Armeria vasconica Sennen – – wfo-0001251971
Armeria vasconica Sennen ex Losa – – wfo-0000549274
Armeria velutina Welw. ex Boiss. & Reut. – – wfo-0000549275
Armeria vestita Willk. – – wfo-0000549276 – http://www.biodiversitylibrary.org/openurl?pid=title:37768&volume=2&issue=&spage=366&date=1868
Armeria villosa Cout. – – wfo-0000549277 – https://biodiversitylibrary.org/page/41531990
Armeria villosa Girard – – wfo-0000549278 – http://www.biodiversitylibrary.org/openurl?pid=title:5010&volume=2&issue=&spage=323&date=1844
Armeria villosa subsp. *villosa* Girard – – wfo-0001303380

Armeria villosa subsp. *alcaracensis* Nieto Fel. – – wfo-0001303381 – http://www.rjb.csic.es/jardinbotanico/ficheros/documentos/pdf/anales/1987/Anales_44(2)_319_348.pdf
Armeria villosa subsp. *bernisii* Nieto Fel. – – wfo-0000549279
Armeria villosa subsp. *carratracensis* (Bernis) Nieto Fel. – – wfo-0000549280
Armeria villosa subsp. *enritrianoi* Blanca, Cueto, J.Fuentes & S.Tello – – wfo-0001303382 – https://revistas.uma.es/index.php/abm/article/view/2882/3932
Armeria villosa subsp. *longiaristata* (Boiss. & Reut.) Nieto Fel. – – wfo-0000549283 – http://www.rjb.csic.es/jardinbotanico/jardin/contenido.php?Pag=219&tipo=volumenanales&vol=44(2)
Armeria villosa subsp. *longiristata* (Boiss. & Reut.) Nieto Fel. – – wfo-0001303383
Armeria villosa subsp. *provillosa* (Bernis) Nieto Fel. – – wfo-0000549284
Armeria villosa subsp. *serpentinicola* Cabezudo, Casim.-Sor.Solanas & Pérez Lat. – – wfo-0001303384 – https://revistas.uma.es/index.php/abm/article/view/2493
Armeria vulgaris f. *ambifaria* (Focke) W.F.Christ. – – wfo-0000549288
Armeria vulgaris f. *arctica* Cham. – – wfo-0000549289
Armeria vulgaris f. *longiscapa* F.Petri – – wfo-0000549301
Armeria vulgaris f. *persicina* W.F.Christ. – – wfo-0000549304
Armeria vulgaris f. *purpurea* T.Marsson – – wfo-0000549308
Armeria vulgaris f. *tenorei* Fiori – – wfo-0000549314
Armeria vulgaris Moris ex Nyman – – wfo-0001303385 – https://biodiversitylibrary.org/page/11015759
Armeria vulgaris subsp. *arctica* (Cham.) Hultén – – wfo-0000549290
Armeria vulgaris subsp. *arctica* (Cham.) Nyman – – wfo-0001095221 – https://biodiversitylibrary.org/page/11015760
Armeria vulgaris subsp. *elongata* (Hoffm.) F.Petri – – wfo-0000549295
Armeria vulgaris subsp. *intermedia* (T.Marsson) Nordh. – – wfo-0000549298
Armeria vulgaris subsp. *maritima* (Mill.) F.Petri – – wfo-0000549302
Armeria vulgaris subsp. *plantaginea* (All.) Syme – – wfo-0001303386
Armeria vulgaris subsp. *purpurea* (W.D.J.Koch) Nyman – – wfo-0000549309 – http://www.biodiversitylibrary.org/openurl?pid=title:10533&volume=&issue=&spage=616&date=1881
Armeria vulgaris subsp. *rhenana* (Gremli) Nyman – – wfo-0001303387
Armeria vulgaris subsp. *serpentini* (Gauckler) Holub – – wfo-0000549311
Armeria vulgaris var. *alba* L.H.Bailey – – wfo-0000745671
Armeria vulgaris var. *alpina* (Willd.) F.Petri – – wfo-0000549287
Armeria vulgaris var. *alpina* Fiori – – wfo-0001303389
Armeria vulgaris var. *calaminaria* F.Petri – – wfo-0000549291
Armeria vulgaris var. *denticulata* (Bertol.) Fiori – – wfo-0001303390
Armeria vulgaris var. *eifeliaca* F.Petri – – wfo-0000549293
Armeria vulgaris var. *elongata* (Hoffm.) F.Petri – – wfo-0001303391
Armeria vulgaris var. *elongata* (Hoffm.) Mert. & W.D.J.Koch – – wfo-0001303392
Armeria vulgaris var. *elongata* (Hoffm.) T.Marsson – – wfo-0000549294
Armeria vulgaris var. *glabra* T.Marsson – – wfo-0000743320
Armeria vulgaris var. *grandiflora* L.H.Bailey – – wfo-0000746044
Armeria vulgaris var. *halleri* (Wallr.) F.Petri – – wfo-0000549296
Armeria vulgaris var. *intermedia* T.Marsson – – wfo-0000549297
Armeria vulgaris var. *labradorica* (Wallr.) F.Petri – – wfo-0000549299
Armeria vulgaris var. *laucheana* (J.N.Haage & E.Schmidt) L.H.Bailey – – wfo-0000746038 – https://www.biodiversitylibrary.org/page/19594058
Armeria vulgaris var. *longiinvolucrata* F.Petri – – wfo-0000549300
Armeria vulgaris var. *marginata* (Levier) Fiori – – wfo-0001303393

Armeria vulgaris var. *maritima* (Mill.) T.Marsson – – wfo-0000743321 – http://www.biodiversitylibrary.org/openurl?pid=title:14642&volume=3&issue=&spage=683&date=1891
Armeria vulgaris var. *maritima* Rosenv. – – wfo-0001303394
Armeria vulgaris var. *nana* Bolzon – – wfo-0001303395
Armeria vulgaris var. *palatina* F.Petri – – wfo-0001303396
Armeria vulgaris var. *planifolia* Syme – – wfo-0000549305
Armeria vulgaris var. *plantaginea* (Willd.) F.Petri – – wfo-0001303397
Armeria vulgaris var. *pubescens* (Sowerby) F.Petri – – wfo-0000549306
Armeria vulgaris var. *purpurea* (W.D.J.Koch) Mert. & W.D.J.Koch – – wfo-0000549307
Armeria vulgaris var. *purpurea* hort. ex L.H.Bailey – – wfo-0001303398
Armeria vulgaris var. *rubra* hort. ex L.H.Bailey – – wfo-0001303399
Armeria vulgaris var. *rugica* F.Petri – – wfo-0000549310
Armeria vulgaris var. *sardoa* (Spreng.) Fiori – – wfo-0000496419
Armeria vulgaris var. *sibirica* (Turcz. ex Boiss.) Rosenv. – – wfo-0000549313 – http://www.biodiversitylibrary.org/openurl?pid=title:14642&volume=3&issue=&spage=683&date=1891
Armeria vulgaris var. *splendens* L.H.Bailey – – wfo-0000746037
Armeria vulgaris Vis. – – wfo-0000549286
Armeria vulgaris Willd. – – wfo-0000549285
Armeria vulgaris subsp. *vulgaris* Willd. – – wfo-0001303388
Armeria welwitschii Boiss. – – wfo-0000549315 – http://www.biodiversitylibrary.org/openurl?pid=title:286&volume=12&issue=&spage=676&date=1848
Armeria welwitschii var. *cinerea* (Boiss. & Welw.) Govaerts – – wfo-0000549316
Armeria welwitschii var. *diversifolia* Franco – – wfo-0000549317
Armeria welwitschii var. *longibracteata* Daveau – – wfo-0000549318 – https://bibdigital.rjb.csic.es/viewer/10622/?offset=#page=178&viewer=picture&o=bookmark&n=0&q=
Armeria welwitschii var. *platyphylla* Daveau – – wfo-0000549319 – https://bibdigital.rjb.csic.es/viewer/10622/?offset=#page=178&viewer=picture&o=bookmark&n=0&q=
Armeria welwitschii var. *stenophylla* Daveau – – wfo-0000549320 – https://bibdigital.rjb.csic.es/viewer/10622/?offset=#page=178&viewer=picture&o=bookmark&n=0&q=
Armeria Willd. – – wfo-4000003077 – https://bibdigital.rjb.csic.es/viewer/10963/?offset=#page=340&viewer=picture&o=search&n=0&q=statice
Armeria willkommii Henriq. – – wfo-0000549321
Armeria willkommii var. *odorata* Samp. – – wfo-0000549322
Armeriaceae Horan. – – wfo-0001303400
Armeriastrum (Jaub. & Spach) Lindl. – – wfo-4000003079 – http://www.biodiversitylibrary.org/openurl?pid=title:7756&volume=&issue=&spage=641&date=1846
Armeriastrum acerosum (Boiss.) Kuntze – – wfo-0000549324 – http://www.biodiversitylibrary.org/openurl?pid=title:327&volume=2&issue=&spage=393&date=1891
Armeriastrum acmostegium (Boiss. & Buhse) Kuntze – – wfo-0000549325 – http://www.biodiversitylibrary.org/openurl?pid=title:327&volume=2&issue=&spage=393&date=1891
Armeriastrum araxanum (Bunge) Kuntze – – wfo-0000549326 – http://www.biodiversitylibrary.org/openurl?pid=title:327&volume=2&issue=&spage=393&date=1891
Armeriastrum aristulatum (Bunge) Kuntze – – wfo-0000549327 – http://www.biodiversitylibrary.org/openurl?pid=title:327&volume=2&issue=&spage=393&date=1891
Armeriastrum armenum (Boiss.) Kuntze – – wfo-0000549328 – http://www.biodiversitylibrary.org/openurl?pid=title:327&volume=2&issue=&spage=393&date=1891
Armeriastrum aspadanum (Bunge) Kuntze – – wfo-0000549329 – http://www.biodiversitylibrary.org/openurl?pid=title:327&volume=2&issue=&spage=393&date=1891
Armeriastrum assyriacum (Boiss.) Kuntze – – wfo-0000549330 – http://www.biodiversitylibrary.org/openurl?pid=title:327&volume=2&issue=&spage=393&date=1891
Armeriastrum atropatanum (Bunge) Kuntze – – wfo-0000549331 – http://www.biodiversitylibrary.org/openurl?pid=title:327&volume=2&issue=&spage=393&date=1891
Armeriastrum auganum (Bunge) Kuntze – – wfo-0000549332 –

http://www.biodiversitylibrary.org/openurl?pid=title:327&volume=2&issue=&spage=393&date=1891
Armeriastrum avenaceum (Bunge) Kuntze – – wfo-0000549334 – http://www.biodiversitylibrary.org/openurl?pid=title:327&volume=2&issue=&spage=393&date=1891
Armeriastrum balansae (Boiss. ex Bunge) Kuntze – – wfo-0000549335 – http://www.biodiversitylibrary.org/openurl?pid=title:327&volume=2&issue=&spage=393&date=1891
Armeriastrum baltanense (Boiss. & Hausskn. ex Boiss.) Kuntze – – wfo-0000549336 – https://biodiversitylibrary.org/page/3816
Armeriastrum bodeanum (Bunge) Kuntze – – wfo-0000549337 – http://www.biodiversitylibrary.org/openurl?pid=title:327&volume=2&issue=&spage=393&date=1891
Armeriastrum brachyphyllum (Boiss.) Kuntze – – wfo-0000549338 – http://www.biodiversitylibrary.org/openurl?pid=title:327&volume=2&issue=&spage=394&date=1891
Armeriastrum brachystachyum (Boiss. ex Bunge) Kuntze – – wfo-0000549339 – http://www.biodiversitylibrary.org/openurl?pid=title:327&volume=2&issue=&spage=393&date=1891
Armeriastrum bracteatum (Boiss.) Kuntze – – wfo-0000549340 – http://www.biodiversitylibrary.org/openurl?pid=title:327&volume=2&issue=&spage=393&date=1891
Armeriastrum bromifolium (Boiss. ex Bunge) Kuntze – – wfo-0000549341 – http://www.biodiversitylibrary.org/openurl?pid=title:327&volume=2&issue=&spage=393&date=1891
Armeriastrum cabulicum (Boiss.) Kuntze – – wfo-0000549342 – http://www.biodiversitylibrary.org/openurl?pid=title:327&volume=2&issue=&spage=393&date=1891
Armeriastrum caesareum (Boiss. & Balansa) Kuntze – – wfo-0000549343 – http://www.biodiversitylibrary.org/openurl?pid=title:327&volume=2&issue=&spage=393&date=1891
Armeriastrum calvertii (Boiss.) Kuntze – – wfo-0000549345 – http://www.biodiversitylibrary.org/openurl?pid=title:327&volume=2&issue=&spage=393&date=1891
Armeriastrum caryophyllaceum (Boiss.) Kuntze – – wfo-0000549346 – http://www.biodiversitylibrary.org/openurl?pid=title:327&volume=2&issue=&spage=393&date=1891
Armeriastrum cataonicum (Bunge) Kuntze – – wfo-0000549347 – https://biodiversitylibrary.org/page/3815
Armeriastrum cephalotes (Boiss.) Kuntze – – wfo-0000549348 – http://www.biodiversitylibrary.org/openurl?pid=title:327&volume=2&issue=&spage=393&date=1891
Armeriastrum curviflorum (Bunge) Kuntze – – wfo-0000549349 – https://biodiversitylibrary.org/page/3815
Armeriastrum cymosum (Bunge) Kuntze – – wfo-0000549350 – http://www.biodiversitylibrary.org/openurl?pid=title:327&volume=2&issue=&spage=393&date=1891
Armeriastrum dianthifolium (Jaub. & Spach) Kuntze – – wfo-0000549351 – http://www.biodiversitylibrary.org/openurl?pid=title:327&volume=2&issue=&spage=393&date=1891
Armeriastrum diapensioides (Boiss.) Kuntze – – wfo-0000549352 – http://www.biodiversitylibrary.org/openurl?pid=title:327&volume=2&issue=&spage=393&date=1891
Armeriastrum distachyum (Boiss.) Kuntze – – wfo-0000549353 – http://www.biodiversitylibrary.org/openurl?pid=title:327&volume=2&issue=&spage=393&date=1891
Armeriastrum echinus (L.) Kuntze – – wfo-0000549354 – https://biodiversitylibrary.org/page/358295
Armeriastrum erinaceum (Jaub. & Spach) Kuntze – – wfo-0000549356 – https://biodiversitylibrary.org/page/3815
Armeriastrum eschkerense (Boiss. & Hausskn. ex Boiss.) Kuntze – – wfo-0000549357 – http://www.biodiversitylibrary.org/openurl?pid=title:327&volume=2&issue=&spage=394&date=1891
Armeriastrum fasciculare (Boiss.) Kuntze – – wfo-0000549358 – http://www.biodiversitylibrary.org/openurl?pid=title:327&volume=2&issue=&spage=393&date=1891
Armeriastrum faustii (Trautv.) Kuntze – – wfo-0000549359 – http://www.biodiversitylibrary.org/openurl?pid=title:327&volume=2&issue=&spage=394&date=1891
Armeriastrum ferox (Boiss.) Kuntze – – wfo-0000549360 – http://www.biodiversitylibrary.org/openurl?pid=title:327&volume=2&issue=&spage=393&date=1891
Armeriastrum festucaceum (Jaub. & Spach) Kuntze – – wfo-0000549361 – http://www.biodiversitylibrary.org/openurl?pid=title:327&volume=2&issue=&spage=393&date=1891
Armeriastrum flexuosum (Boiss. ex Bunge) Kuntze – – wfo-0000549362 – http://www.biodiversitylibrary.org/openurl?pid=title:327&volume=2&issue=&spage=393&date=1891
Armeriastrum genistoides (Jaub. & Spach) Kuntze – – wfo-0000549363 – http://www.biodiversitylibrary.org/openurl?pid=title:327&volume=2&issue=&spage=393&date=1891
Armeriastrum glumaceum (Boiss.) Kuntze – – wfo-0000549364 – http://www.biodiversitylibrary.org/openurl?pid=title:327&volume=2&issue=&spage=393&date=1891
Armeriastrum griffithianum (Boiss.) Kuntze – – wfo-0000549365 – http://www.biodiversitylibrary.org/openurl?pid=title:327&volume=2&issue=&spage=393&date=1891
Armeriastrum gulistanum (Bunge) Kuntze – – wfo-0000549367 – http://www.biodiversitylibrary.org/openurl?pid=title:327&volume=2&issue=&spage=393&date=1891

Armeriastrum hausknechtii (Bunge) Kuntze – – wfo-0000549368 – http://www.biodiversitylibrary.org/openurl?pid=title:327&volume=2&issue=&spage=393&date=1891
Armeriastrum heratense (Bunge) Kuntze – – wfo-0000549369 – http://www.biodiversitylibrary.org/openurl?pid=title:327&volume=2&issue=&spage=393&date=1891
Armeriastrum horridum (Bunge) Kuntze – – wfo-0000549370 – http://www.biodiversitylibrary.org/openurl?pid=title:327&volume=2&issue=&spage=393&date=1891
Armeriastrum huetii (Boiss.) Kuntze – – wfo-0000549371 – http://www.biodiversitylibrary.org/openurl?pid=title:327&volume=2&issue=&spage=393&date=1891
Armeriastrum iconicum (Boiss. & Heldr.) Kuntze – – wfo-0000549372 – http://www.biodiversitylibrary.org/openurl?pid=title:327&volume=2&issue=&spage=394&date=1891
Armeriastrum incomptum (Boiss. & Buhse) Kuntze – – wfo-0000549373 – http://www.biodiversitylibrary.org/openurl?pid=title:327&volume=2&issue=&spage=393&date=1891
Armeriastrum karelinii (Bunge) Kuntze – – wfo-0000549374 – http://www.biodiversitylibrary.org/openurl?pid=title:327&volume=2&issue=&spage=393&date=1891
Armeriastrum kotschyi (Boiss.) Kuntze – – wfo-0000549375 – http://www.biodiversitylibrary.org/openurl?pid=title:327&volume=2&issue=&spage=393&date=1891
Armeriastrum kurdicum (Bunge) Kuntze – – wfo-0000549376 – http://www.biodiversitylibrary.org/openurl?pid=title:327&volume=2&issue=&spage=393&date=1891
Armeriastrum latifolium (Boiss.) Kuntze – – wfo-0000549378 – http://www.biodiversitylibrary.org/openurl?pid=title:327&volume=2&issue=&spage=393&date=1891
Armeriastrum laxiflorum (Boiss.) Kuntze – – wfo-0000549379 – http://www.biodiversitylibrary.org/openurl?pid=title:327&volume=2&issue=&spage=393&date=1891
Armeriastrum lepturoides (Boiss.) Kuntze – – wfo-0000549380 – http://www.biodiversitylibrary.org/openurl?pid=title:327&volume=2&issue=&spage=393&date=1891
Armeriastrum leucacanthum (Boiss.) Kuntze – – wfo-0000549381 – http://www.biodiversitylibrary.org/openurl?pid=title:327&volume=2&issue=&spage=393&date=1891
Armeriastrum libanoticum (Boiss.) Kuntze – – wfo-0000549382 – http://www.biodiversitylibrary.org/openurl?pid=title:327&volume=2&issue=&spage=393&date=1891
Armeriastrum listoniae (Boiss.) Kuntze – – wfo-0000549383 – http://www.biodiversitylibrary.org/openurl?pid=title:327&volume=2&issue=&spage=393&date=1891
Armeriastrum longiflorum (Boiss.) Kuntze – – wfo-0000549384 – http://www.biodiversitylibrary.org/openurl?pid=title:327&volume=2&issue=&spage=394&date=1891
Armeriastrum lycaonicum (Boiss. & Heldr.) Kuntze – – wfo-0000549385 – http://www.biodiversitylibrary.org/openurl?pid=title:327&volume=2&issue=&spage=394&date=1891
Armeriastrum lycopodioides (Girard) Kuntze – – wfo-0000549386 – http://www.biodiversitylibrary.org/openurl?pid=title:327&volume=2&issue=&spage=393&date=1891
Armeriastrum melananthum (Boiss.) Kuntze – – wfo-0000549387 – http://www.biodiversitylibrary.org/openurl?pid=title:327&volume=2&issue=&spage=394&date=1891
Armeriastrum oliganthum (Boiss.) Kuntze – – wfo-0000549390 – http://www.biodiversitylibrary.org/openurl?pid=title:327&volume=2&issue=&spage=394&date=1891
Armeriastrum olivieri (Boiss.) Kuntze – – wfo-0000549391 – http://www.biodiversitylibrary.org/openurl?pid=title:327&volume=2&issue=&spage=394&date=1891
Armeriastrum peronini (Boiss.) Kuntze – – wfo-0000549392 – https://www.biodiversitylibrary.org/page/3816
Armeriastrum peroninii (Boiss.) Kuntze – – wfo-0001303401
Armeriastrum petraeum (Boiss. ex Bunge) Kuntze – – wfo-0000549393 – http://www.biodiversitylibrary.org/openurl?pid=title:327&volume=2&issue=&spage=394&date=1891
Armeriastrum pinardii (Boiss.) Kuntze – – wfo-0000549394 – https://www.biodiversitylibrary.org/page/3816
Armeriastrum polystachyum (Boiss.) Kuntze – – wfo-0000549395 – http://www.biodiversitylibrary.org/openurl?pid=title:327&volume=2&issue=&spage=394&date=1891
Armeriastrum pterostegium (Bunge) Kuntze – – wfo-0000549396 – http://www.biodiversitylibrary.org/openurl?pid=title:327&volume=2&issue=&spage=394&date=1891
Armeriastrum puberulum (Boiss. & Balansa) Kuntze – – wfo-0000549397 – https://biodiversitylibrary.org/page/3815
Armeriastrum quinquelobum (Bunge) Kuntze – – wfo-0000549398 – http://www.biodiversitylibrary.org/openurl?pid=title:327&volume=2&issue=&spage=394&date=1891
Armeriastrum restiaceum (Bunge) Kuntze – – wfo-0000549399 – http://www.biodiversitylibrary.org/openurl?pid=title:327&volume=2&issue=&spage=394&date=1891
Armeriastrum ruprechtii (Bunge) Kuntze – – wfo-0000549401 – http://www.biodiversitylibrary.org/openurl?pid=title:327&volume=2&issue=&spage=394&date=1891
Armeriastrum sahendicum (Boiss. & Buhse) Kuntze – – wfo-0000549402 –

http://www.biodiversitylibrary.org/openurl?pid=title:327&volume=2&issue=&spage=394&date=1891
Armeriastrum scabrellum (Boiss. & Hausskn. ex Boiss.) Kuntze – – wfo-0000549403 – http://www.biodiversitylibrary.org/openurl?pid=title:327&volume=2&issue=&spage=393&date=1891
Armeriastrum schahrudicum (Bunge) Kuntze – – wfo-0000549404 – http://www.biodiversitylibrary.org/openurl?pid=title:327&volume=2&issue=&spage=394&date=1891
Armeriastrum schirazianum (Boiss.) Kuntze – – wfo-0000549405 – http://www.biodiversitylibrary.org/openurl?pid=title:327&volume=2&issue=&spage=394&date=1891
Armeriastrum scirpinum (Bunge) Kuntze – – wfo-0000549406 – http://www.biodiversitylibrary.org/openurl?pid=title:327&volume=2&issue=&spage=394&date=1891
Armeriastrum senganense (Bunge) Kuntze – – wfo-0000549407 – http://www.biodiversitylibrary.org/openurl?pid=title:327&volume=2&issue=&spage=394&date=1891
Armeriastrum setiferum (Bunge) Kuntze – – wfo-0000549408 – http://bibdigital.rjb.csic.es/ing/Libro.php?Libro=5480&Pagina=20
Armeriastrum splendidum (Bunge) Kuntze – – wfo-0000549409 – https://biodiversitylibrary.org/page/3816
Armeriastrum stocksii (Boiss.) Kuntze – – wfo-0000549410 – https://biodiversitylibrary.org/page/3816
Armeriastrum subsessile (Trautv.) Kuntze – – wfo-0000549412 – http://www.biodiversitylibrary.org/openurl?pid=title:327&volume=2&issue=&spage=393&date=1891
Armeriastrum subulatum (Boiss.) Kuntze – – wfo-0000549413 – http://www.biodiversitylibrary.org/openurl?pid=title:327&volume=2&issue=&spage=394&date=1891
Armeriastrum talagonicum (Boiss.) Kuntze – – wfo-0000549414 – http://www.biodiversitylibrary.org/openurl?pid=title:327&volume=2&issue=&spage=394&date=1891
Armeriastrum tartaricum (Boiss.) Kuntze – – wfo-0000549415 – https://biodiversitylibrary.org/page/3816
Armeriastrum tenuiflorum (Boiss.) Kuntze – – wfo-0000549416 – http://www.biodiversitylibrary.org/openurl?pid=title:327&volume=2&issue=&spage=394&date=1891
Armeriastrum tenuifolium (Jaub. & Spach) Kuntze – – wfo-0000549417 – http://www.biodiversitylibrary.org/openurl?pid=title:327&volume=2&issue=&spage=393&date=1891
Armeriastrum tomentellum (Boiss.) Kuntze – – wfo-0000549418 – http://www.biodiversitylibrary.org/openurl?pid=title:327&volume=2&issue=&spage=394&date=1891
Armeriastrum tragacanthium (Boiss.) Kuntze – – wfo-0000549419 – https://biodiversitylibrary.org/page/3816
Armeriastrum truncatum (Bunge) Kuntze – – wfo-0000549420 – http://www.biodiversitylibrary.org/openurl?pid=title:327&volume=2&issue=&spage=394&date=1891
Armeriastrum ulicinum (Boiss.) Kuntze – – wfo-0000549421 – http://www.biodiversitylibrary.org/openurl?pid=title:327&volume=2&issue=&spage=394&date=1891
Armeriastrum viscidulum (Boiss.) Kuntze – – wfo-0000549423 – http://www.biodiversitylibrary.org/openurl?pid=title:327&volume=2&issue=&spage=394&date=1891
Armeriastrum wiedemannii (Bunge) Kuntze – – wfo-0000549424 – https://biodiversitylibrary.org/page/3816
Armerieae Dumort. – – wfo-0001303402 – https://www.biodiversitylibrary.org/page/33110212
Bakerolimon Lincz. – – wfo-4000003915
Bakerolimon peruvianum (Kuntze) Lincz. – – wfo-0000558553
Bakerolimon plumosum (Phil.) Lincz. – – wfo-0001261789
Bamiania Lincz. – – wfo-4000004003
Bamiania pachycorma (Rech.f.) Lincz. – – wfo-0000558968
Bubania feei Girard – – wfo-0000573210 – http://gallica.bnf.fr/ark:/12148/bpt6k209710c/f84.image
Bubania Girard – – wfo-4000005631 – http://gallica.bnf.fr/ark:/12148/bpt6k209710c/f81.image
Bubania migiurtina Chiov. – – wfo-0000573211
Bubania monopetala (L.) Girard – – wfo-0000573212 – http://gallica.bnf.fr/ark:/12148/bpt6k209710c/f82.image
Bukiniczia cabulica (Boiss.) Lincz. – – wfo-0000574491
Bukiniczia Lincz. – – wfo-4000005715
Caballeroa Font Quer – – wfo-0001303403
Caballeroa ifniensis (Caball.) Font Quer – – wfo-0000577388
Cephalorhizum coelicolor (Rech.f.) Rech.f. – – wfo-0000594929
Cephalorhizum micranthum Lincz. – – wfo-0000594930

Cephalorhizum oopodum Popov & Korovin – – wfo-0000594931
Cephalorhizum pachycormum Rech.f. – – wfo-0000594932
Cephalorhizum Popov & Korovin – – wfo-4000007275
Cephalorhizum popovii Lincz. – – wfo-0000594933
Cephalorhizum sect. *Sarcophyllastrum* Rech.f. – – wfo-0001303404
Cephalorhizum setiferum (Bunge) Popov & Korovin – – wfo-0000594934
Cephalorhizum turcomanicum Popov ex Lincz. – – wfo-0000594935 – http://biodiversitylibrary.org/page/30218710
Cephalorrhizum Popov & Korovin – – wfo-0001303405
Ceratolimon feei (Girard) M.B.Crespo & Lledó – – wfo-0000596392 – https://doi.org/10.1111/j.1095-8339.2000.tb01212.x
Ceratolimon feei var. *feei* – – wfo-0001303406 – http://onlinelibrary.wiley.com/doi/10.1111/j.1095-8339.2000.tb01212.x/pdf
Ceratolimon feei var. *grandiflorum* (Maire & Wilcz.) M.B.Crespo & Lledó – – wfo-0000596393
Ceratolimon M.B.Crespo & Lledó – – wfo-4000007364 – http://onlinelibrary.wiley.com/doi/10.1111/j.1095-8339.2000.tb01212.x/pdf
Ceratolimon migiurtinum (Chiov.) M.B.Crespo & Lledó – – wfo-0000596394 – http://onlinelibrary.wiley.com/doi/10.1111/j.1095-8339.2000.tb01212.x/pdf
Ceratolimon rechingeri (J.R.Edm.) M.B.Crespo & Lledó – – wfo-0000596395
Ceratolimon weygandiorum (Maire & Wilczek) M.B.Crespo & Lledó – – wfo-0000596396 – http://onlinelibrary.wiley.com/doi/10.1111/j.1095-8339.2000.tb01212.x/pdf
Ceratostigma abyssinicum (Hochst.) Asch. – – wfo-0000596549
Ceratostigma asperrimum Stapf ex Prain – – wfo-0000596550 – https://biodiversitylibrary.org/page/35239239
Ceratostigma Bunge – – wfo-4000007387 – https://biodiversitylibrary.org/page/38131
Ceratostigma griffithii C.B.Clarke – – wfo-0000596551 – http://www.biodiversitylibrary.org/openurl?pid=title:678&volume=3&issue=9&spage=481&date=1882
Ceratostigma minus f. *lasaense* T.X.Peng – – wfo-0000596553
Ceratostigma minus Stapf ex Prain – – wfo-0000596552 – https://biodiversitylibrary.org/page/35239240
Ceratostigma plantaginoides J.W.C.Kirk – – wfo-0000596555
Ceratostigma plumbaginoides Bunge – – wfo-0000596556
Ceratostigma polhillii hort. ex Bulley – – wfo-0000596557
Ceratostigma speciosum Prain – – wfo-0000596558 – https://biodiversitylibrary.org/page/35239240
Ceratostigma stapfianum Hosseus – – wfo-0000596559 – https://biodiversitylibrary.org/page/33511137
Ceratostigma ulicinum Prain – – wfo-0000596560 – https://biodiversitylibrary.org/page/35239240
Ceratostigma willmottianum Stapf – – wfo-0000596561 – https://biodiversitylibrary.org/page/476863
Chaetolimon (Bunge) Lincz. – – wfo-4000007514
Chaetolimon limbatum Lincz. – – wfo-0000599170
Chaetolimon setiferum (Bunge) Lincz. – – wfo-0000599171
Chaetolimon sogdianum Lincz. – – wfo-0000599172
Chomutowia B.Fedtsch – – wfo-4000008042
Chomutowia ekatherinae B.Fedtsch. – – wfo-0000603208
Dictyolimon gilesii (Hemsl.) Rech.f. – – wfo-0000444663
Dictyolimon griffithii (Aitch. & Hemsl.) Rech.f. – – wfo-0000646348
Dictyolimon macrorrhabdos (Boiss.) Rech.f. – – wfo-0000646349
Dictyolimon Rech.f. – – wfo-4000011574
Dictyolimon thomsonii (C.B.Clarke) Rech.f. – – wfo-0001303408
Dyerophytum (Lam.) Kuntze – – wfo-4000012816 – http://bibdigital.rjb.csic.es/ing/Libro.php?Libro=5480&Pagina=20
Dyerophytum africanum (Lam.) Kuntze – – wfo-0000658244 – http://www.biodiversitylibrary.org/openurl?pid=title:327&volume=2&issue=&spage=394&date=1891

Dyerophytum arabicum (Boiss.) M.R.Almeida – – wfo-0000658245
Dyerophytum indicum (Gibbs ex Wight) Kuntze – – wfo-0000658246 – http://www.biodiversitylibrary.org/openurl?pid=title:327&volume=2&issue=&spage=394&date=1891
Dyerophytum pendulum (Balf.f.) Kuntze – – wfo-0000658248 – http://www.biodiversitylibrary.org/openurl?pid=title:327&volume=2&issue=&spage=394&date=1891
Dyerophytum socotranum (Balf.f.) J.R.Edm. ex J.R.Edm., M.Malekm. & Koutr. – – wfo-0001303409
Eremolimon botschantzevii Lincz. – – wfo-0000670913
Eremolimon drepanostachyum (Ikonn.-Gal.) Lincz. – – wfo-0000670915
Eremolimon fajzievii (Zakirov ex Lincz.) Lincz. – – wfo-0000670916
Eremolimon jarmolenkoi Lincz. – – wfo-0000670917
Eremolimon kurgantjubense Lincz. – – wfo-0000670918
Eremolimon Lincz. – – wfo-4000013721
Eremolimon piptopodum (Nevski) Lincz. – – wfo-0000670919
Eremolimon sogdianum (Ikonn.-Gal.) Lincz. – – wfo-0000670920
Eurychiton adensis Nimmo – – wfo-0000683340 – https://biodiversitylibrary.org/page/31389107
Eurychiton Nimmo – – wfo-4000014287 – https://biodiversitylibrary.org/page/31389107
Findlaya alba Bowdich – – wfo-0000690993
Findlaya Bowdich – – wfo-0001303410
Ghaznianthus Lincz. – – wfo-4000015606
Ghaznianthus rechingeri (Freitag) Lincz. – – wfo-0000702481
Gladiolimon Mobayen – – wfo-4000015693
Gladiolimon speciosissimum (Aitch. & Hemsl.) Mobayen – – wfo-0000703473
Goniolimon africanum Buzurović, Bogdanović & Brullo – – wfo-0001303411 – https://doi.org/10.11646/phytotaxa.349.3.10
Goniolimon beckerianum Janka – – wfo-0000706382 – https://biodiversitylibrary.org/page/30862189
Goniolimon besserianum Nyman – – wfo-0000706383 – https://biodiversitylibrary.org/page/11015757
Goniolimon Boiss. – – wfo-4000015998 – https://biodiversitylibrary.org/page/160856
Goniolimon cabulicum (Boiss.) Mobayen – – wfo-0000706384
Goniolimon callicomum (C.A.Mey.) Boiss. – – wfo-0000706385 – http://www.biodiversitylibrary.org/openurl?pid=title:286&volume=12&issue=&spage=633&date=1848
Goniolimon callicomum var. *callicomum* (C.A.Mey.) Boiss. – – wfo-0001303412
Goniolimon callicomum var. *gorczakovskyi* (Knjaz.) Knjaz. & Golovanov – – wfo-0001303413
Goniolimon caucasicum Klokov – – wfo-0000706386
Goniolimon collinum (Griseb.) Boiss. – – wfo-0000706387 – https://biodiversitylibrary.org/page/160857
Goniolimon collinum var. *bulgaricum* Novák – – wfo-0000706388
Goniolimon crispum (Regel) Lipsch. – – wfo-0000706389 – https://biodiversitylibrary.org/page/30218448
Goniolimon cuspidatum Gamajun. – – wfo-0000706390
Goniolimon dalmaticum (C.Presl) Rchb. – – wfo-0000706391
Goniolimon desertorum (Trautv.) Klokov – – wfo-0000706393 – https://biodiversitylibrary.org/page/45215726
Goniolimon dshungaricum (Regel) O.Fedtsch. & B.Fedtsch. – – wfo-0000706394
Goniolimon elatum (Fisch. ex Spreng.) Boiss. – – wfo-0000706395 – https://biodiversitylibrary.org/page/160858
Goniolimon eximium (Schenk) Boiss. – – wfo-0000706396 – http://www.biodiversitylibrary.org/openurl?pid=title:286&volume=12&issue=&spage=634&date=1848
Goniolimon glaberrimum Klokov – – wfo-0000706397
Goniolimon gorczakovskyi Knjaz. – – wfo-0001303414
Goniolimon graminifolium (Aiton) Boiss. – – wfo-0000706398 – https://biodiversitylibrary.org/page/160857
Goniolimon griffithianum (Aitch. & Hemsl.) Mobayen – – wfo-0000706399

Goniolimon heldreichii Halácsy – – wfo-0000706400 – http://www.biodiversitylibrary.org/openurl?pid=title:13275&volume=36&issue=&spage=241&date=1886
Goniolimon incanum (L.) Hepper – – wfo-0000706401
Goniolimon italicum Tammaro, Pignatti & Frizzi – – wfo-0000706402 – https://doi.org/10.1080/00837792.1982.10670238
Goniolimon kaufmannianum (Regel) O.Fedtsch. & B.Fedtsch. – – wfo-0001303415
Goniolimon kaufmannianum (Regel) Voss – – wfo-0000706405 – https://biodiversitylibrary.org/page/42525303
Goniolimon krylovii A.V.Grebenjuk – – wfo-0001303416
Goniolimon orae-syvashicae Klokov – – wfo-0001095223 – https://biodiversitylibrary.org/page/45215726
Goniolimon orthocladum Rupr. – – wfo-0000706406 – https://biodiversitylibrary.org/page/46560801
Goniolimon platypterum Klokov – – wfo-0000706407
Goniolimon rubellum (S.G.Gmel.) Klokov – – wfo-0000706408
Goniolimon salicorniaceum (F.Muell.) Christenh. & Byng – – wfo-0001303417 – http://www.plantgateway.com/wp-content/uploads/lana-downloads/2018/02/Global-Flora-Vol-4.pdf
Goniolimon sartorii Boiss. – – wfo-0000706409 – http://bibdigital.rjb.csic.es/ing/Libro.php?Libro=1418&Pagina=69
Goniolimon sect. *Tricuspidaria* Lincz. – – wfo-1000045085
Goniolimon sect. *Unicuspidaria* Lincz. – – wfo-1000045084
Goniolimon serbicum Vis. – – wfo-0000706410
Goniolimon severzovii Herder – – wfo-0001303418
Goniolimon sewerzovii Herder – – wfo-0001303419
Goniolimon sewerzowii Herder – – wfo-0000706411 – http://www.biodiversitylibrary.org/openurl?pid=title:4951&volume=41&issue=1&spage=396&date=1868
Goniolimon speciosum (L.) Boiss. – – wfo-0000706412 – http://www.biodiversitylibrary.org/openurl?pid=title:286&volume=12&issue=&spage=634&date=1848
Goniolimon speciosum var. *speciosum* (L.) Boiss. – – wfo-0001095291
Goniolimon speciosum var. *alpinum* Herd. – – wfo-0001303420
Goniolimon speciosum var. *crispum* O.Fedtsch & B.Fedtsch. – – wfo-0001303421
Goniolimon speciosum var. *genuinum* Herd. – – wfo-0001303422
Goniolimon speciosum var. *lanceolatum* (Regel) O.Fedtsch. & B.Fedtsch. – – wfo-0001303423
Goniolimon speciosum var. *strictum* (Regel) T.H.Peng – – wfo-0000706413
Goniolimon speciosum var. *typicum* O.Fedtsch & B.Fedtsch – – wfo-0001303424
Goniolimon strictum (Regel) Lincz. – – wfo-0000706414 – https://biodiversitylibrary.org/page/30218448
Goniolimon subsect. *Platycalyx* Lincz. – – wfo-1000045086
Goniolimon subsect. *Stenocalyx* Lincz. – – wfo-1000045087
Goniolimon tarbagataicum Gamajun. – – wfo-0001095293
Goniolimon tataricum (L.) Boiss. – – wfo-0000706416 – https://biodiversitylibrary.org/page/160856
Goniolimon tataricum subsp. *tataricum* (L.) Boiss. – – wfo-0001303425
Goniolimon tataricum f. *bulgaricum* (Novák) Ančev – – wfo-0000706417
Goniolimon tataricum f. *ciliatum* Ančev – – wfo-0000706418
Goniolimon tataricum subsp. *croaticum* Buzurović & Bogdanović – – wfo-0001303426
Goniolimon tataricum subsp. *graecum* Buzurović – – wfo-0001303427
Goniolimon tataricum subsp. *italicum* (Tammaro, Pignatti & Frizzi) Buzurović – – wfo-0001303428
Goniolimon tataricum var. *angustifolium* Boiss. – – wfo-0001303429 – https://biodiversitylibrary.org/page/160857
Goniolimon tataricum var. *besserianum* O.Fedtsch. & B.Fedtsch. – – wfo-0001303430
Goniolimon tataricum var. *desertorum* Trautv. – – wfo-0001303431
Goniolimon tataricum var. *graminifolium* Trautv. – – wfo-0001303432
Goniolimon tataricum var. *kluchoricum* Tzvelev – – wfo-0001303433

Goniolimon tataricum var. *laxiflorum* Boiss. – – wfo-0001303434 – https://biodiversitylibrary.org/page/160857
Goniolimon tataricum var. *platypterum* (Klokov) Tzvelev – – wfo-0001303435
Goniolimon tataricum var. *puberulum* Trautv. – – wfo-0001303436
Goniolimon tataricum var. *rubellum* Trautv. – – wfo-0001303437
Goniolimon tataricum var. *tauricum* (Klokov) Tzvelev – – wfo-0000706419
Goniolimon tauricum Klokov – – wfo-0000706420
Ikonnikovia kaufmanniana (Regel) Lincz. – – wfo-0001095214 – https://biodiversitylibrary.org/page/30218437
Ikonnikovia kaufmanniana var. *latifolia* Z.B.Kubanskaya ex Lincz. – – wfo-0001303438 – https://www.biodiversitylibrary.org/page/30218439
Ikonnikovia Lincz. – – wfo-4000018988 – https://biodiversitylibrary.org/page/30218709
Lerrouxia Caball. – – wfo-4000021414 – URL:http://bibdigital.rjb.csic.es/ing/Libro.php?Libro=708&Pagina=11
Lerrouxia ifniensis Caball. – – wfo-0000444447 – http://bibdigital.rjb.csic.es/ing/Libro.php?Libro=708&Pagina=13
Limoniaceae Lincz. – – wfo-7000000677
Limoniaceae Ser. – – wfo-0001303439
Limoniastrum articulatum Moench – – wfo-0000444467 – http://www.biodiversitylibrary.org/openurl?pid=title:304&volume=&issue=&spage=423&date=1794
Limoniastrum feei (Girard) Hook.f. ex Pax – – wfo-0001038957
Limoniastrum feei var. *grandiflorum* Maire & Wilcz. – – wfo-0001038958
Limoniastrum guyonianum Durieu ex Boiss. – – wfo-0000444466
Limoniastrum Heist. ex Fabr. – – wfo-4000021805 – http://www.digibib.tu-bs.de/view_page.php?page=31&nav=+%3E+&minpage=1&max_page=246&suffix=gif&url=http%3A%2F%2Fdigisrv-1.biblio.etc.tu-bs.de%2Fdocportal%2Fservlets%2FMCRFileNodeServlet%2FDocPortal_derivate_00000100&offset=0
Limoniastrum ifniense (Caball.) Font Quer – – wfo-0000444465
Limoniastrum majus Lanza – – wfo-0000444462
Limoniastrum malenconianum Maire – – wfo-0000444461
Limoniastrum migiurtinum (Chiov.) Chiov. ex Maire – – wfo-0000444460
Limoniastrum migiurtinum Chiov. – – wfo-0000444452
Limoniastrum monopetalum (L.) Boiss. – – wfo-0000444468 – http://www.biodiversitylibrary.org/openurl?pid=title:286&volume=12&issue=&spage=689&date=1848
Limoniastrum monopetalum subsp. *multiflorum* Bonhomme & P.Fourn. – – wfo-0001303440
Limoniastrum multiflorum C.Bonhomme & P.Fourn. – – wfo-0000444459
Limoniastrum ouarglense Pomel – – wfo-0000444458 – https://bibdigital.rjb.csic.es/viewer/10313/?offset=#page=133&viewer=picture&o=bookmark&n=0&q=
Limoniastrum rechingeri J.R.Edm. – – wfo-0001038955
Limoniastrum reinwardtii Lanza – – wfo-0000444457
Limoniastrum sect. *Bubania* Batt. – – wfo-0001303441
Limoniastrum speciosum (L.) Moench – – wfo-0000444456
Limoniastrum subg. Bubania (Batt.) Maire – – wfo-0001303442
Limoniastrum weygandiorum Maire & Wilczek – – wfo-0000444454
Limonieae Reveal – – wfo-0001303443 – http://www.phytoneuron.net/PhytoN-InfrafamNames.pdf
Limoniodes guyonianum Siegesb. ex Kuntze – – wfo-0000444442
Limoniodes monopetalum Kuntze – – wfo-0000444423
Limoniodes Siegesb. ex Kuntze – – wfo-4000021806 – http://www.biodiversitylibrary.org/openurl?pid=title:327&volume=2&issue=&spage=394&date=1891
Limoniopsis davisii Bokhari – – wfo-0000444422
Limoniopsis Lincz. – – wfo-4000021807 – https://biodiversitylibrary.org/page/30218708
Limoniopsis owerinii (Boiss.) Lincz. – – wfo-0001095224 – https://biodiversitylibrary.org/page/30218436

Limonium "Mediterranean lineage" – DUMMY_Limonium_Mediterranean lineage
Limonium ×abnorme (Rouy) P.Fourn. – – wfo-0000444421
Limonium ×albuferae Ferrer-Gallego, P. P., R.Roselló, M.Rosato, Rosselló & E.Laguna – – wfo-0001303444 – http://dx.doi.org/10.11646/phytotaxa.252.2.3
Limonium ×ambiguum (Rouy) P.Fourn. – – wfo-0000444428
Limonium ×capdeperae Pignatti – – wfo-0000444356
Limonium ×castellonense Erben – – wfo-0000444310 – https://biodiversitylibrary.org/page/15043341
Limonium ×christii G.Kunkel – – wfo-0000444301
Limonium ×coincyi Sennen – – wfo-0000444319
Limonium ×coriacifolium (Sennen) M.B.Crespo & Serra – – wfo-0000509734
Limonium ×coriacifolium Sennen – – wfo-0000444398 – http://bibdigital.rjb.csic.es/ing/Libro.php?Libro=15&Pagina=99
Limonium ×dolcheri Pignatti – – wfo-0000443464 – https://doi.org/10.1080/00837792.1982.10670239
Limonium ×dolcheri Pignatti ex Dolcher & Pignatti – – wfo-0001303445
Limonium ×erectiflorum (B.Fedtsch. & Gontsch.) A.V.Grebenjuk – – wfo-0000507524
Limonium ×escarrei L.Llorens & Tébar – – wfo-0001260274
Limonium ×eugeniae Sennen – – wfo-0000444679 – http://bibdigital.rjb.csic.es/ing/Libro.php?Libro=15&Pagina=99
Limonium ×fraternum (Sennen & Pau) M.B.Crespo – – wfo-0001303446
Limonium ×garciae Pignatti – – wfo-0000444697
Limonium ×glaucophyllum Pignatti – – wfo-0000444660 – http://bibdigital.rjb.csic.es/ing/Libro.php?Libro=2834&Pagina=419
Limonium ×gougemolsii Pignatti – – wfo-0000444652
Limonium ×interjectum J.X.Soler & Rosselló – – wfo-0000444777 – http://dx.doi.org/10.3989/ajbm.1997.v55.i1.256
Limonium ×lucentinum Pignatti & Freitag – – wfo-0000444565 – http://onlinelibrary.wiley.com/doi/10.1111/j.1095-8339.1971.tb02152.x/full
Limonium ×multirameum Sennen – – wfo-0000444543 – http://bibdigital.rjb.csic.es/ing/Libro.php?Libro=15&Pagina=98
Limonium ×neumanii C.E.Salmon – – wfo-0000444531 – https://biodiversitylibrary.org/page/35302666
Limonium ×profusum (hort.) F.T.Hubb. ex L.H.Bailey – – wfo-0000444061 – https://biodiversitylibrary.org/page/568409
Limonium ×pseudoconfusum (Rouy) P.Fourn. – – wfo-0000444056
Limonium ×pseudodivaricatum Pignatti – – wfo-0000444054
Limonium ×pseudosmithii Pignatti – – wfo-0000444059
Limonium ×rossmaessleri (Willk.) M.B.Crespo – – wfo-0001303447
Limonium ×sennenii (Rouy) P.Fourn. – – wfo-0000444117
Limonium ×tibulatium Pignatti – – wfo-0000443946
Limonium ×valentinum (Huter, Porta & Rigo) M.B.Crespo & Lledó – – wfo-0000443932
Limonium ×viretianum Pignatti – – wfo-0000443924
Limonium ×virgatoformis (Rouy) B.Bock – – wfo-0001303448
Limonium ×virgitanum Pignatti – – wfo-0000443921
Limonium ×virgolsii f. *pseudovirgatum* Pignatti – – wfo-0001303449
Limonium ×virgolsii Pignatti – – wfo-0000443920
Limonium ×virgutiflorum Pignatti – – wfo-0000443919
Limonium acuminatum L.Bolus – – wfo-0001095170
Limonium acutifolium (Badarò ex Rchb.) C.E.Salmon – – wfo-0000444419 – http://archive.bsbi.org.uk/journal_of_botany.html
Limonium acutifolium subsp. *acutifolium* (Badarò ex Rchb.) C.E.Salmon – – wfo-0001303450
Limonium acutifolium subsp. *acutifolium* – – wfo-0001303451
Limonium acutifolium subsp. *bosanum* (Arrigoni & Diana) Arrigoni – – wfo-0001303452

Limonium acutifolium subsp. *cornusianum* (Arrigoni & Diana) Arrigoni – – wfo-0001303453
Limonium acutifolium subsp. *nymphaeum* (Erben) Arrigoni – – wfo-0001303454
Limonium acutifolium subsp. *obtusifolium* (Rouy) Diana – – wfo-0001303455
Limonium acutifolium subsp. *tenuifolium* (Bertol. ex Moris) Arrigoni – – wfo-0001303456
Limonium acutifolium subsp. *tharrosianum* (Arrigoni & Diana) Arrigoni – – wfo-0001303457
Limonium acutifolium var. *obtusifolium* (Rouy) C.E.Salmon – – wfo-0001303458 – http://archive.bsbi.org.uk/journal_of_botany.html
Limonium adilguneri Yıld. & Doğru-Koca – – wfo-0000507522
Limonium admirabile Terrones, J.Moreno, M.Á.Alonso, Juan & M.B.Crespo – – wfo-0001303459 – https://doi.org/10.11646/phytotaxa.333.1.3
Limonium aegaeum Erben & Brullo – – wfo-0001303460 – http://dx.doi.org/10.11646/phytotaxa.240.1
Limonium aegusae Brullo – – wfo-0000444416 – http://journals.lub.lu.se/index.php/bn/article/view/11412/10558
Limonium afghanicum Erben & Podlech – – wfo-0000444431 – https://biodiversitylibrary.org/page/15043336
Limonium africanum Mill. – – wfo-0000444433 – https://biodiversitylibrary.org/page/395149
Limonium afrum (Pignatti) Domina – – wfo-0000749420 – http://www.bioone.org/doi/abs/10.3372/wi.41.41117
Limonium albarracinense Pau ex Ferrer-Gallego, P. P. & R.Roselló – – wfo-0001303461 – http://www.bioone.org/doi/full/10.3417/2017041
Limonium albidum (Guss.) Pignatti – – wfo-0000444420 – http://onlinelibrary.wiley.com/doi/10.1111/j.1095-8339.1971.tb02152.x/full
Limonium albidum subsp. *cyprium* Meikle – – wfo-0000444439
Limonium albomarginatum Brullo – – wfo-0000444438 – http://www.jstor.org/stable/3996200
Limonium album (Coincy) Sennen – – wfo-0000745500 – http://bibdigital.rjb.csic.es/ing/Libro.php?Libro=15&Pagina=73
Limonium alcudianum Erben – – wfo-0000444437 – http://www.biodiversitylibrary.org/openurl?pid=title:14894&volume=28&issue=&spage=313&date=1989
Limonium algarvense Erben – – wfo-0000444436 – https://biodiversitylibrary.org/page/15235838
Limonium algusae (Brullo) Greuter – – wfo-0000444435 – http://www.jstor.org/stable/3996512
Limonium alicunense Gómiz – – wfo-0000444434
Limonium alleizettei (Pau) Brullo – – wfo-0000444424 – https://biodiversitylibrary.org/page/27802342
Limonium alleizettii Balls – – wfo-0000444432
Limonium almeriense Pount – – wfo-0000444440
Limonium altum P.D.Sell – – wfo-0001303462
Limonium alutaceum (Steven) Kuntze – – wfo-0000444429 – https://biodiversitylibrary.org/page/3817
Limonium amblyolobum Ikonn.-Gal. – – wfo-0000444427
Limonium ammochostianum Erben, Christodoulou, Hand & Kefalas – – wfo-1000047127
Limonium ammophilon (Papatsou & Phitos) Domina – – wfo-0000749413 – http://www.bioone.org/doi/abs/10.3372/wi.41.41117
Limonium amoenum (C.H.Wright) R.A.Dyer – – wfo-0001095171 – https://www.jstor.org/stable/4115039?seq=1#page_scan_tab_contents
Limonium amopicum Erben & Brullo – – wfo-0001303463 – http://dx.doi.org/10.11646/phytotaxa.240.1
Limonium ampuriense Arrigoni & Diana – – wfo-0000444426
Limonium amynclaeum Pignatti – – wfo-0000444425 – https://doi.org/10.1080/00837792.1982.10670239
Limonium anatolicum Hedge – – wfo-0000444475
Limonium anceps (Regel) Kuntze – – wfo-0000444418 – https://biodiversitylibrary.org/page/3817
Limonium anfractum (C.E.Salmon) C.E.Salmon – – wfo-0000444453 – http://archive.bsbi.org.uk/journal_of_botany.html
Limonium anglicum (Ingr.) P.D.Sell – – wfo-0001303464
Limonium angustatum (A. Gray) Small – – wfo-0001095213 – http://www.biodiversitylibrary.org/openurl?pid=title:340&volume=24&issue=11&spage=488&date=1897
Limonium angustebracteatum Erben – – wfo-0001303465

Limonium angustibracteatum Erben – – wfo-0000444499 – https://biodiversitylibrary.org/page/15235847
Limonium angustifolium (Tausch) Degen – – wfo-0001303466
Limonium angustifolium (Tausch) Turrill – – wfo-0000444498
Limonium anthericoides (Schltr.) R.A.Dyer – – wfo-0001095173 – https://www.jstor.org/stable/4115039?seq=1#page_scan_tab_contents
Limonium antipaxorum R.Artelari – – wfo-0000444497
Limonium antonii-llorensii L.Llorens – – wfo-0000444496
Limonium aphroditae R.Artelari & Georgiou – – wfo-0000444490 – http://onlinelibrary.wiley.com/doi/10.1111/j.1095-8339.1999.tb01522.x/full
Limonium apulum Brullo – – wfo-0001443426
Limonium arabicum (Jaub. & Spach) Kuntze – – wfo-0000444495 – https://biodiversitylibrary.org/page/3817
Limonium aragonense (Debeaux ex Willk.) Font Quer – – wfo-0001303467
Limonium aragonense (Debeaux ex Willk.) Pignatti – – wfo-0000444500 – http://bibdigital.rjb.csic.es/ing/Libro.php?Libro=2837&Pagina=309
Limonium aragonense subsp. *ruizii* (Font Quer) Fern.Casas & Muñoz Garm. – – wfo-0001303468
Limonium arborescens (Brouss.) Kuntze – – wfo-0001095314 – http://www.biodiversitylibrary.org/openurl?pid=title:327&volume=2&issue=&spage=395&date=1891
Limonium arboreum (Willd.) Erben, A.Santos & Reyes-Bet. – – wfo-0001303469 – http://www.herbmedit.org/flora22.html
Limonium arboreum (Willd.) H. Arnaud – – wfo-0001303470
Limonium arbusculum (Maxim.) Makino – – wfo-0001095325
Limonium arbusculum var. *luteum* H.Hara – – wfo-0001095326
Limonium archaeothirae Erben & Brullo – – wfo-0001303471 – http://dx.doi.org/10.11646/phytotaxa.240.1
Limonium archeothirae Erben & Brullo – – wfo-0001303472
Limonium arcuatum R.Artelari – – wfo-0000444492
Limonium arenosum Erben – – wfo-0000444491 – https://biodiversitylibrary.org/page/15235885
Limonium argentarium Pignatti – – wfo-0000444507 – https://doi.org/10.1080/00837792.1982.10670239
Limonium artelariae Koutr. – – wfo-0001304455
Limonium articulatum (Loisel.) Kuntze – – wfo-0000444494 – https://biodiversitylibrary.org/page/3817
Limonium articulatum subsp. *dictyocladum* – – wfo-0001303473
Limonium articulatum subsp. *pseudarticulatum* (Erben) O.Bolòs & Vigo – – wfo-0001303474
Limonium artruchium Erben – – wfo-0000444516 – https://biodiversitylibrary.org/page/27802209
Limonium asparagoides (Coss. & Durieu ex Batt.) Maire – – wfo-0000444515 – http://bibdigital.rjb.csic.es/ing/Libro.php?Libro=1866&Pagina=55
Limonium asperrimum Maire – – wfo-0000444514
Limonium asterotrichum (C.E.Salmon) C.E.Salmon – – wfo-0000444513 – http://archive.bsbi.org.uk/journal_of_botany.html
Limonium astipaleanum Erben & Brullo – – wfo-0001303475
Limonium astypaleanum Erben & Brullo – – wfo-0001303476 – http://dx.doi.org/10.11646/phytotaxa.240.1
Limonium athinense Erben & Brullo – – wfo-0001303477 – http://dx.doi.org/10.11646/phytotaxa.240.1
Limonium atticum Erben & Brullo – – wfo-0001303478 – http://dx.doi.org/10.11646/phytotaxa.240.1
Limonium aucheri (Girard) Greuter & Raus – – wfo-0001244266 – https://www.jstor.org/stable/3996917?seq=1#page_scan_tab_contents
Limonium augustatum (A.Gray) Small – – wfo-0001303479
Limonium aureum (L.) Chaz. – – wfo-0001303480 – https://gallica.bnf.fr/ark:/12148/bpt6k15115421/f43.item
Limonium aureum (L.) Hill – – wfo-0001303481 – http://bibdigital.rjb.csic.es/ing/Libro.php?Libro=5656&Pagina=80
Limonium aureum (L.) Hill ex Kuntze – – wfo-0001095225 – https://biodiversitylibrary.org/page/3817
Limonium aureum var. *aureum* – – wfo-0001303482

Limonium aureum var. *dielsianum* (Wangerin) T.H.Peng – – wfo-0001095344
Limonium aureum var. *maduoensis* Y.H.Wu – – wfo-0001303483 – https://www.oriprobe.com/journals/whzwxyj/2006_4.html
Limonium aureum var. *potaninii* (Ikonn.-Gal.) T.H.Peng – – wfo-0001095343
Limonium auriculae-ursifolium (Pourr.) Druce – – wfo-0000732970
Limonium auriculae-ursifolium subsp. *lusitanicum* (Pignatti) Pignatti – – wfo-0000443117 – http://onlinelibrary.wiley.com/doi/10.1111/j.1095-8339.1971.tb02152.x/full
Limonium auriculae-ursifolium subsp. *multillorum* (Pignatti) Pignatti – – wfo-0000443116 – http://onlinelibrary.wiley.com/doi/10.1111/j.1095-8339.1971.tb02152.x/full
Limonium auriculifolium (Vahl) Druce – – wfo-0000443115 – https://biodiversitylibrary.org/page/335139
Limonium auriculifolium var. *dodarti* (Girard) Druce – – wfo-0001303484
Limonium aurigniense (Ingr.) P.D.Sell – – wfo-0001303485
Limonium australe (R.Br.) Kuntze – – wfo-0000443155 – https://biodiversitylibrary.org/page/3817
Limonium australe var. *australe* – – wfo-0001303486
Limonium australe var. *baudinii* (Lincz.) A.M.Gray – – wfo-0001303487 – https://www.tmag.tas.gov.au/__data/assets/pdf_file/0003/128568/KANUNNAH4.pdf
Limonium avei (De Not.) Brullo – – wfo-0000443180 – http://www.jstor.org/stable/3996200
Limonium avenaceum (C.H.Wright) R.A.Dyer – – wfo-0000444512 – https://www.jstor.org/stable/4115039?seq=1#page_scan_tab_contents
Limonium axillare (Forssk.) Kuntze – – wfo-0001095147 – http://www.biodiversitylibrary.org/openurl?pid=title:327&volume=2&issue=&spage=395&date=1891
Limonium bahamense (Griseb.) Britton – – wfo-0000444511 – http://www.biodiversitylibrary.org/openurl?pid=title:44786&volume=4&issue=&spage=142&date=1906
Limonium bahamense var. *haitense* (S.F.Blake) Alain – – wfo-0001033128
Limonium bahamense var. *haitiense* (S.F.Blake) Alain – – wfo-0001303488 – https://nybgshop.org/a-biosystematic-study-of-north-american-thlaspi-montanum-its-allies-mem-21-2/
Limonium balearicum (Pignatti) Brullo – – wfo-0000444508 – http://journals.lub.lu.se/index.php/bn/article/view/11412/10558
Limonium barceloi Gil & L.Llorens – – wfo-0000444517 – http://www.rjb.csic.es/jardinbotanico/jardin/contenido.php?Pag=219&tipo=volumenanales&vol=49(1)
Limonium battandieri Greuter & Raus – – wfo-0000444506 – http://www.jstor.org/stable/3996512
Limonium baudinii Lincz. – – wfo-0000444505
Limonium beaumierianum (Coss. ex Maire) Maire – – wfo-0000444504 – https://bibdigital.rjb.csic.es/viewer/13176/?offset=#page=20&viewer=picture&o=bookmark&n=0&q=
Limonium beaumierianum var. *akkense* (Coss. ex Batt.) Maire – – wfo-0001303489
Limonium beaumierianum var. *annuum* (Maire) Maire – – wfo-0001303490
Limonium beaumierianum var. *dubium* Maire – – wfo-0001303491
Limonium beaumierianum var. *glabrescens* Maire – – wfo-0001303492
Limonium beaumierianum var. *leucocalyx* (Maire) Maire – – wfo-0001303493 – https://bibdigital.rjb.csic.es/viewer/13179/?offset=#page=203&viewer=picture&o=bookmark&n=0&q=
Limonium beaumierianum var. *tripeaui* (Maire) Maire – – wfo-0001303494
Limonium beaumierianum var. *violascens* Maire – – wfo-0001303495 – https://bibdigital.rjb.csic.es/viewer/13179/?offset=#page=203&viewer=picture&o=bookmark&n=0&q=
Limonium behen (Drejer) Kuntze – – wfo-0000444503 – https://biodiversitylibrary.org/page/3817
Limonium bellidifolium (Gouan) Dumort. – – wfo-0000444502
Limonium bellidifolium (Guan) Dumort. – – wfo-0001303496
Limonium bellidifolium subsp. *caspium* (Willd.) P.Fourn. – – wfo-0001303497
Limonium bellidifolium subsp. *dubyi* (Gren. & Godr.) P.Fourn. – – wfo-0000747449
Limonium bellidifolium var. *bellidifolium* – – wfo-0001303498
Limonium bellidifolium var. *prostratum* (Beauverd) Rech.f. – – wfo-0001303499 – https://www.zobodat.at/publikation_volumes.php?id=31166
Limonium benmageci Marrero Rodr. – – wfo-0000507523 – https://dialnet.unirioja.es/servlet/articulo?codigo=2242681

Limonium besserianum (Schult. ex Rchb.) Kuntze – – wfo-0000444488 – https://biodiversitylibrary.org/page/3817
Limonium bianorii (Sennen & Pau) Erben – – wfo-0000444510 – https://biodiversitylibrary.org/page/27802199
Limonium bicolor (Bunge) Kuntze – – wfo-0001095308 – http://www.biodiversitylibrary.org/openurl?pid=title:327&volume=2&issue=&spage=395&date=1891
Limonium biflorum (Pignatti) Pignatti – – wfo-0000444479 – http://onlinelibrary.wiley.com/doi/10.1111/j.1095-8339.1971.tb02152.x/full
Limonium billardierei (Girard) Kuntze – – wfo-0000444480 – https://biodiversitylibrary.org/page/3817
Limonium binervosum (G.E.Sm.) C.E.Salmon – – wfo-0000444478
Limonium binervosum subsp. *anglicum* Ingr. – – wfo-0001303500 – https://doi.org/10.1111/j.1095-8339.1986.tb01428.x
Limonium binervosum subsp. *binervosum* – – wfo-0001303501
Limonium binervosum subsp. *cantianum* Ingr. – – wfo-0001303502 – https://doi.org/10.1111/j.1095-8339.1986.tb01428.x
Limonium binervosum subsp. *dodartii* (Girard) P.Fourn. – – wfo-0001303503
Limonium binervosum subsp. *lychnidifolium* P.Fourn. – – wfo-0001303504
Limonium binervosum subsp. *multiflorum* Pignatti – – wfo-0000745497
Limonium binervosum subsp. *mutatum* Ingr. – – wfo-0001303505 – https://doi.org/10.1111/j.1095-8339.1986.tb01428.x
Limonium binervosum subsp. *occidentale* (J.Lloyd) P.Fourn. – – wfo-0001303506
Limonium binervosum subsp. *sarniense* Ingr. – – wfo-0001303507 – https://doi.org/10.1111/j.1095-8339.1986.tb01428.x
Limonium binervosum subsp. *saxonicum* Ingr. – – wfo-0001303508 – https://doi.org/10.1111/j.1095-8339.1986.tb01428.x
Limonium binervosum var. *aurigniense* Ingr. – – wfo-0001303509 – https://doi.org/10.1111/j.1095-8339.1986.tb01428.x
Limonium binervosum var. *humilis* C.E.Salmon – – wfo-0001303510 – https://www.biodiversitylibrary.org/page/35439571
Limonium binervosum var. *sarniense* Ingr. – – wfo-0001303511
Limonium binervosum var. *sercquense* Ingr. – – wfo-0001303512 – https://doi.org/10.1111/j.1095-8339.1986.tb01428.x
Limonium bocconei (Lojac.) Litard. – – wfo-0000745477
Limonium boirae L.Llorens & Tébar – – wfo-0000444474
Limonium boitardii Maire – – wfo-0000444473
Limonium bollei (Webb ex Wangerin) Erben – – wfo-0000444471
Limonium bolosii Gil & L.Llorens – – wfo-0000444470 – http://www.rjb.csic.es/jardinbotanico/jardin/contenido.php?Pag=219&tipo=volumenanales&vol=49(1)
Limonium bonafei Erben – – wfo-0000444469 – https://biodiversitylibrary.org/page/27803562
Limonium bonduellei (T.Lestib.) Kuntze – – wfo-0000444483 – https://biodiversitylibrary.org/page/3817
Limonium bonduellei f. *gigantifolia* Corti – – wfo-0001303513
Limonium bonifaciense Arrigoni & Diana – – wfo-0000444484
Limonium bonnetii (Sennen) Erben – – wfo-0000444476 – https://biodiversitylibrary.org/page/27802262
Limonium bosanum Arrigoni & Diana – – wfo-0000444487
Limonium botschantzevii (Lincz.) M.Malekm., Akhani & Borsch – – wfo-0001303514 – https://doi.org/10.12705/665.8
Limonium bourgeaui (Webb ex Boiss.) Kuntze – – wfo-0001095255 – http://www.biodiversitylibrary.org/openurl?pid=title:327&volume=2&issue=&spage=395&date=1891
Limonium brasiliense (Boiss.) Kuntze – – wfo-0001095271 – http://www.biodiversitylibrary.org/openurl?pid=title:327&volume=2&issue=&spage=395&date=1891
Limonium brasiliense (Boiss.) Small – – wfo-0001303515 – https://www.biodiversitylibrary.org/page/715985
Limonium brasiliense Small – – wfo-0001303516 – https://www.biodiversitylibrary.org/page/2141626
Limonium brasiliense var. *brasiliense* – – wfo-0001236408
Limonium brasiliense var. *patagonicum* (Speg.) Burkart – – wfo-0001095269
Limonium brasiliensis A.Heller – – wfo-0000447141
Limonium brassicifolium (Webb & Berthel.) Kuntze – – wfo-0000444485 – https://biodiversitylibrary.org/page/3817

Limonium brassicifolium subsp. *macropterum* (Webb & Berthel.) G.Kunkel – – wfo-0001303517
Limonium braunii (Bolle) A.Chev. – – wfo-0001095250 – http://gallica.bnf.fr/ark:/12148/bpt6k12571595/f196.item.r=928
Limonium brevipetiolatum R.Artelari & Erben – – wfo-0000444489 – http://www.biodiversitylibrary.org/openurl?pid=title:14894&volume=22&issue=&spage=507&date=1986
Limonium britannicum Ingr. – – wfo-0000444482 – http://onlinelibrary.wiley.com/doi/10.1111/j.1095-8339.1986.tb01428.x/full
Limonium britannicum subsp. *celticum* Ingr. – – wfo-0001303518 – http://onlinelibrary.wiley.com/doi/10.1111/j.1095-8339.1986.tb01428.x/full
Limonium britannicum subsp. *coombense* Ingr. – – wfo-0001303519 – https://doi.org/10.1111/j.1095-8339.1986.tb01428.x
Limonium britannicum subsp. *transcanalis* Ingr. – – wfo-0001303520 – https://doi.org/10.1111/j.1095-8339.1986.tb01428.x
Limonium britannicum var. *celticum* Ingr. – – wfo-0001303521 – https://doi.org/10.1111/j.1095-8339.1986.tb01428.x
Limonium britannicum var. *coombense* Ingr. – – wfo-0001303522 – https://doi.org/10.1111/j.1095-8339.1986.tb01428.x
Limonium britannicum var. *grandicaule* Ingr. – – wfo-0001303523 – https://doi.org/10.1111/j.1095-8339.1986.tb01428.x
Limonium britannicum var. *kelseyanum* Ingr. – – wfo-0001303524 – https://doi.org/10.1111/j.1095-8339.1986.tb01428.x
Limonium britannicum var. *pharense* Ingr. – – wfo-0001303525
Limonium brizoides Brullo ex Erben, Del Guacchio & P.Caputo – – wfo-0001303527
Limonium brunneri (Webb) Kuntze – – wfo-0001095144 – https://biodiversitylibrary.org/page/3817
Limonium brusnicense (Trinajstić) Bogdanović & Brullo – – wfo-0001303528 – http://dx.doi.org/10.11646/phytotaxa.215.1.1
Limonium brutium Brullo – – wfo-0000444337 – http://www.herbmedit.org/flora02.html
Limonium bulgaricum Ančev – – wfo-0000444336
Limonium bungei (Claus) Gamajun. – – wfo-0000748100
Limonium busianum Bogdanović & Brullo – – wfo-0001303529 – http://dx.doi.org/10.11646/phytotaxa.215.1.1
Limonium byzacium Brullo – – wfo-0000444335 – http://www.biodiversitylibrary.org/openurl?pid=title:14894&volume=28&issue=&spage=419&date=1989
Limonium cabulicum (Boiss.) Kuntze – – wfo-0001264529 – http://www.biodiversitylibrary.org/openurl?pid=title:327&volume=2&issue=&spage=395&date=1891
Limonium caesium (Girard) Kuntze – – wfo-0000444334 – http://www.biodiversitylibrary.org/openurl?pid=title:327&volume=2&issue=&spage=395&date=1891
Limonium calabrum Brullo – – wfo-0000444333 – http://journals.lub.lu.se/index.php/bn/article/view/11412/10558
Limonium calaminare Pignatti – – wfo-0000444332 – http://bibdigital.rjb.csic.es/ing/Libro.php?Libro=2838&Pagina=955
Limonium calanchicola Erben – – wfo-0000444340 – https://biodiversitylibrary.org/page/28677725
Limonium calcarae (Tod. ex Janka) Pignatti – – wfo-0000444330 – http://onlinelibrary.wiley.com/doi/10.1111/j.1095-8339.1971.tb02152.x/full
Limonium calcicola P.D.Sell – – wfo-0001303530
Limonium californicum (Boiss.) A.Heller – – wfo-0001095155
Limonium californicum var. *californicum* (Boiss.) A.Heller – – wfo-0001095209
Limonium californicum Small – – wfo-0001303531
Limonium californicum var. *mexicanum* (S.F.Blake) Munz – – wfo-0001095156
Limonium callianthum (T.X.Peng) Kamelin – – wfo-0001095280 – https://biodiversitylibrary.org/page/639169
Limonium callicomum (C.A.Mey.) Kuntze – – wfo-0001095295 – http://www.biodiversitylibrary.org/openurl?pid=title:327&volume=2&issue=&spage=395&date=1891
Limonium calliopsium Alf.Mayer – – wfo-0000444328
Limonium cambrense (Ingr.) P.D.Sell – – wfo-0001303532
Limonium camposanum Erben – – wfo-0000444348 – http://www.biodiversitylibrary.org/openurl?pid=title:14894&volume=28&issue=&spage=313&date=1989
Limonium cancellatum (Bernh. ex Bertol.) Kuntze – – wfo-0000444331 – https://biodiversitylibrary.org/page/3817
Limonium cantianum (Ingr.) P.D.Sell – – wfo-0001303533

Limonium capense (L.Bolus) L.Bolus – – wfo-0001095175
Limonium capitis-eliae Erben – – wfo-0000444355 – https://biodiversitylibrary.org/page/15042580
Limonium capitis-marci Arrigoni & Diana – – wfo-0000444354
Limonium caprariae Rizzotto – – wfo-0000444353 – https://doi.org/10.1080/00837792.1999.10670663
Limonium caprariense (Font Quer & Marcos) Pignatti – – wfo-0000444352
Limonium caprariense subsp. *caprariense* – – wfo-0001303534
Limonium caprariense subsp. *multiflorum* Pignatti – – wfo-0001303535
Limonium caralitanum Erben – – wfo-0000444351 – https://biodiversitylibrary.org/page/15042572
Limonium carinense (Chiov.) Verdc. & Hemming ex Cufod. – – wfo-0000444349 – http://www.jstor.org/stable/3667449, http://www.jstor.org/stable/3667348
Limonium carisae Erben – – wfo-0000444358 – https://biodiversitylibrary.org/page/15042587
Limonium carminis B.Díez & Erben – – wfo-1000047223 – http://www.floramontiberica.org/FM/082/Flora_Montib_082_113-115_2022.pdf
Limonium carnosum (Boiss.) Kuntze – – wfo-0000444346
Limonium carolinianum (Walter) Britton – – wfo-0001095157 – http://www.biodiversitylibrary.org/openurl?pid=title:45469&volume=5&issue=17&spage=255&date=1894
Limonium carolinianum var. *carolinianum* (Walter) Britton – – wfo-0001095210
Limonium carolinianum var. *angustatum* (A.Gray) S.F.Blake – – wfo-0001095211 – http://www.biodiversitylibrary.org/openurl?pid=title:721&volume=25&issue=292&spage=56&date=1923
Limonium carolinianum var. *compactum* Shinners – – wfo-0001273547
Limonium carolinianum var. *nashii* (Small) B.Boivin – – wfo-0001303536
Limonium carolinianum var. *obtusilobum* (S.F.Blake) H.E.Ahles – – wfo-0001095158
Limonium carolinianum var. *trichogonum* (S.F.Blake) B.Boivin – – wfo-0001242844
Limonium carpathum (Rech.f.) Rech.f. – – wfo-0000444345 – https://www.zobodat.at/publikation_volumes.php?id=31166
Limonium carpetanicum Erben – – wfo-0000444344 – https://biodiversitylibrary.org/page/15148007
Limonium carregadorense Erben – – wfo-0000444343 – http://www.biodiversitylibrary.org/openurl?pid=title:14894&volume=28&issue=&spage=313&date=1989
Limonium carthaginense (Rouy) C.E.Hubb. & Sandwith – – wfo-0000444342
Limonium carvalhoi Rosselló, L.Sáez & Carvalho, A.C. – – wfo-0000444326 – http://dx.doi.org/10.3989/ajbm.1998.v56.i1.217
Limonium caspium (Willd.) Gams – – wfo-0001303537 – https://bibdigital.rjb.csic.es/viewer/16557/?offset=#page=346
Limonium caspium (Willd.) P.Fourn. – – wfo-0000444350
Limonium caspium var. *patens* (Fisch. ex Boiss.) A.V.Grebenjuk – – wfo-0001303538
Limonium castellonense Socorro & S.Tárrega – – wfo-0000444327 – http://www.rjb.csic.es/jardinbotanico/jardin/contenido.php?Pag=219&tipo=volumenanales&vol=40(1)
Limonium catalaunicum (Willk. & Costa) Pignatti – – wfo-0000444309 – http://bibdigital.rjb.csic.es/ing/Libro.php?Libro=2837&Pagina=308
Limonium catalaunicum subsp. *catalaunicum* – – wfo-0001303539
Limonium catalaunicum subsp. *procerum* (Willk.) Pignatti – – wfo-0000748111
Limonium catalaunicum subsp. *viciosoi* (Pau) Pignatti – – wfo-0000748112 – http://bibdigital.rjb.csic.es/ing/Libro.php?Libro=2837&Pagina=308
Limonium catanense (Tineo ex Lojac.) Brullo – – wfo-0000444308
Limonium catanzaroi Brullo – – wfo-0000444306 – http://journals.lub.lu.se/index.php/bn/article/view/11412/10558
Limonium cavanillesii Erben – – wfo-0001260264 – https://biodiversitylibrary.org/page/15043345
Limonium cazzae Bogdanović & Brullo – – wfo-0001303540 – http://dx.doi.org/10.11646/phytotaxa.215.1.1
Limonium cedrorum Domina & Raimondo – – wfo-0001303541
Limonium celticum (Ingr.) P.D.Sell – – wfo-0001303542 – https://doi.org/10.1111/j.1095-8339.1986.tb01428.x
Limonium cephalonicum R.Artelari – – wfo-0000444305

Limonium cercinense Brullo – – wfo-0000444299 – http://www.biodiversitylibrary.org/openurl?pid=title:14894&volume=28&issue=&spage=419&date=1989
Limonium chazaliei (H.Boissieu) Maire – – wfo-0000444303
Limonium chersonesum Erben & Brullo – – wfo-0001303543 – http://dx.doi.org/10.11646/phytotaxa.240.1
Limonium chodshamumynense Lincz. & Czukav. – – wfo-0000444311
Limonium chrisianum Brullo & Guarino – – wfo-0000444302 – http://www.herbmedit.org/flora10.html
Limonium chrysocephalum (Regel) Lincz. – – wfo-0001095365 – https://biodiversitylibrary.org/page/30218477
Limonium chrysocomum (Kar. & Kir.) Kuntze – – wfo-0001095226 – http://www.biodiversitylibrary.org/openurl?pid=title:327&volume=2&issue=&spage=395&date=1891
Limonium chrysocomum subsp. *chrysocomum* (Kar. & Kir.) Kuntze – – wfo-0001095345
Limonium chrysocomum subsp. *semenovii* (Herder) Kamelin – – wfo-0001095350 – https://biodiversitylibrary.org/page/639168
Limonium chrysocomum var. *chrysocephalum* (Regel) T.H.Peng – – wfo-0001095346
Limonium chrysocomum var. *pubescens* Lincz. – – wfo-0001095347
Limonium chrysocomum var. *sedoides* (Regel) T.H.Peng – – wfo-0001095348
Limonium chrysocomum var. *semenovii* (Herder) T.H.Peng – – wfo-0001095351
Limonium chrysopotamicum Maire – – wfo-0000444304
Limonium cimmericum (Lipsky) Klokov – – wfo-0000444325
Limonium circaei Pignatti – – wfo-0000444323 – https://doi.org/10.1080/00837792.1982.10670239
Limonium clupeanum Brullo – – wfo-0000444322 – http://www.biodiversitylibrary.org/openurl?pid=title:14894&volume=28&issue=&spage=419&date=1989
Limonium coelicolor Rech.f. – – wfo-0000444321
Limonium cofrentanum Erben – – wfo-0000444320 – http://www.biodiversitylibrary.org/openurl?pid=title:14894&volume=28&issue=&spage=313&date=1989
Limonium collinum (Griseb.) F.T.Hubb. ex L.H.Bailey – – wfo-0000444312 – https://biodiversitylibrary.org/page/568409
Limonium commune Gray – – wfo-0000444318 – https://biodiversitylibrary.org/page/30086162
Limonium commune subsp. *californicum* (Boiss.) A.E.Murray – – wfo-0001303544
Limonium commune var. *californicum* (Boiss.) Greene – – wfo-0001262698 – https://biodiversitylibrary.org/page/38425951
Limonium commune var. *mexicanum* (S.F.Blake) Jeps. – – wfo-0001235720 – https://biodiversitylibrary.org/page/7368651
Limonium commune var. *minus* Gray – – wfo-0001303545 – https://www.biodiversitylibrary.org/page/49762993
Limonium commune var. *obtusum* Gray – – wfo-0001303546 – https://www.biodiversitylibrary.org/page/49762993
Limonium commune var. *serotinum* Gray – – wfo-0001303547 – https://www.biodiversitylibrary.org/page/49762993
Limonium comosum Erben – – wfo-0000444317 – https://biodiversitylibrary.org/page/15042595
Limonium compactum Erben & Brullo – – wfo-0001303548 – http://dx.doi.org/10.11646/phytotaxa.240.1
Limonium companyonis (Gren. & Billot) Kuntze – – wfo-0000444316 – https://biodiversitylibrary.org/page/3817
Limonium confertum Brullo – – wfo-0000444314 – http://www.biodiversitylibrary.org/openurl?pid=title:14894&volume=28&issue=&spage=419&date=1989
Limonium confusum (Gren. & Godr.) Fourr. – – wfo-0000444313 – https://biodiversitylibrary.org/page/54553850
Limonium confusum (Gren. & Godr.) Kuntze – – wfo-0001303549 – https://biodiversitylibrary.org/page/3817
Limonium confusum subsp. *densissimum* Pignatti – – wfo-0000745495 – http://bibdigital.rjb.csic.es/ing/Libro.php?Libro=2834&Pagina=418
Limonium confusum subsp. *duriusculum* (Girard) P.Fourn. – – wfo-0001303550
Limonium confusum subsp. *psilocladum* (Boiss.) P.Fourn. – – wfo-0001303551
Limonium confusum subsp. *raddianum* (Boiss.) P.Fourn. – – wfo-0001303552
Limonium congestum (Ledeb.) Kuntze – – wfo-0001095227 – http://www.biodiversitylibrary.org/openurl?pid=title:327&volume=2&issue=&spage=395&date=1891
Limonium connivens Erben – – wfo-0000444363 – http://www.biodiversitylibrary.org/openurl?pid=title:14894&volume=28&issue=&spage=313&date=1989

Limonium contortirameum (Mabille) Erben – – wfo-0001238363 – https://biodiversitylibrary.org/page/27803575
Limonium contractum Erben & Brullo – – wfo-0001303553 – http://dx.doi.org/10.11646/phytotaxa.240.1
Limonium coombense (Ingr.) P.D.Sell – – wfo-0001303554
Limonium cophanense C.Brullo, Brullo, Cambria, del Galdo & Ilardi – – wfo-0001443423 – http://dx.doi.org/10.11646/phytotaxa.255.2.4
Limonium coralliforme Alf.Mayer – – wfo-0000444341
Limonium coralloides (Tausch) Lincz. – – wfo-0001095228 – https://biodiversitylibrary.org/page/30218489
Limonium corculum (Webb & Berthel.) Kuntze – – wfo-0000444401 – https://biodiversitylibrary.org/page/3817
Limonium cordatum (L.) Mill. – – wfo-0000444400 – https://biodiversitylibrary.org/page/395149
Limonium cordovillense Stübing & Cirujano – – wfo-0000444399 – http://rjb.revistas.csic.es/index.php/rjb/article/view/287/282
Limonium coriarium H.Arnaud – – wfo-1000049848
Limonium corinthiacum (Boiss. & Heldr.) Kuntze – – wfo-0000444397 – https://biodiversitylibrary.org/page/3817
Limonium cornarianum Kypr. & R.Artelari – – wfo-0000444395
Limonium cornubiense (Ingr.) P.D.Sell – – wfo-0001303555
Limonium cornusianum Arrigoni & Diana – – wfo-0000444384
Limonium coronense R.Artelari – – wfo-0000444393
Limonium corsicum Erben – – wfo-0000444391 – http://www.biodiversitylibrary.org/openurl?pid=title:14894&volume=30&issue=&spage=174&date=1991
Limonium corymbulosum (Boiss.) Kuntze – – wfo-0000444390 – https://biodiversitylibrary.org/page/3817
Limonium cossonianum (Nyman) Kuntze – – wfo-0000444389 – https://biodiversitylibrary.org/page/3817
Limonium cossonianum (Nyman) Kuntze – – wfo-0001303556
Limonium cossonianum f. *tabernense* (Erben) G.Kunkel – – wfo-0001303557
Limonium costae (Willk.) Pignatti – – wfo-0000444388 – http://bibdigital.rjb.csic.es/ing/Libro.php?Libro=2837&Pagina=310
Limonium cosyrense (Guss.) Kuntze – – wfo-0000444387 – https://biodiversitylibrary.org/page/3817
Limonium cosyrense var. *maior* (Boiss.) Rech.f. – – wfo-0001303558 – https://www.zobodat.at/publikation_volumes.php?id=31166
Limonium crateriforme Erben & Brullo – – wfo-0001303559 – http://dx.doi.org/10.11646/phytotaxa.240.1.1
Limonium cretaceum Cherkasova – – wfo-0000444407
Limonium creticum R.Artelari – – wfo-0000444392
Limonium crispum (Pers.) H.Arnaud – – wfo-1000055774 – https://www.biodiversitylibrary.org/page/45269412
Limonium croaticum Bogdanović & Brullo – – wfo-0001303560 – http://dx.doi.org/10.11646/phytotaxa.215.1.1
Limonium cumanum (Ten.) Kuntze – – wfo-0000444394 – https://biodiversitylibrary.org/page/3817
Limonium cumanum var. *cumanum* (Ten.) Kuntze – – wfo-0001303561
Limonium cumanum var. *glabrescens* (Lacaita) Vallariello, Iamonico & Del Guacchio – – wfo-0001303562 – http://dx.doi.org/10.11646/phytotaxa.263.2.5
Limonium cunicularium Arrigoni & Diana – – wfo-0000444414
Limonium cuspidatum (Delort.) Erben – – wfo-0000444413 – https://biodiversitylibrary.org/page/15235807
Limonium cylindrifolium (Forssk.) Verdc. ex Cufod. – – wfo-0001095258 – http://www.jstor.org/stable/3667348, http://www.jstor.org/stable/3667449
Limonium cymuliferum (Boiss.) Sauvage & Vindt – – wfo-0000444411
Limonium cymuliferum var. *cymuliferum* (Boiss.) Sauvage & Vindt – – wfo-0001303563
Limonium cymuliferum subsp. *cymuliferum* – – wfo-0001303564
Limonium cymuliferum subsp. *mauritii* Sennen – – wfo-0001303565 – http://bibdigital.rjb.csic.es/ing/Libro.php?Libro=42&Pagina=116
Limonium cymuliferum var. *corymbulosum* Pignatti – – wfo-0001303566
Limonium cymuliferum var. *sebkarum* (Pomel) Sauvage & Vindt – – wfo-0001303567

Limonium cymuliferum var. *uniflorum* Pignatti – – wfo-0001303568
Limonium cyprium (Meikle) Hand – – wfo-0000447136 – https://doi.org/10.3372/wi.33.33209
Limonium cyrenaicum (Rouy) Brullo – – wfo-0000444410 – https://doi.org/10.1080/00837792.1978.10670114
Limonium cyrtostachyum (Girard) Brullo – – wfo-0000444409 – http://journals.lub.lu.se/index.php/bn/article/view/11412/10558
Limonium cythereum R.Artelari & Georgiou – – wfo-0000444402 – http://onlinelibrary.wiley.com/doi/10.1111/j.1095-8339.1999.tb01522.x/full
Limonium czurjukiense (Klokov) Lavrenko – – wfo-0000748101
Limonium dagmarae Mucina – – wfo-0001303569
Limonium dagmarae Mucina – – wfo-0001303570 – https://doi.org/10.11646/phytotaxa.403.2.1
Limonium dagmariae Mucina – – wfo-0001442002 – https://doi.org/10.11646/phytotaxa.403.2.1
Limonium dalmaticum (C.Presl) Kuntze – – wfo-0000444408 – https://biodiversitylibrary.org/page/3817
Limonium damboldtianum Phitos & R.Artelari – – wfo-0000444415
Limonium danubiale Klokov – – wfo-0000444406
Limonium daveaui Erben – – wfo-0000444405 – https://biodiversitylibrary.org/page/15235812
Limonium davisii Doğan – – wfo-0001303571 – https://doi.org/10.1007/s00606-020-01706-3
Limonium decipiens (Ledeb.) Kuntze – – wfo-0001095362 – http://www.biodiversitylibrary.org/openurl?pid=title:327&volume=2&issue=&spage=395&date=1891
Limonium decumbens (Boiss.) Kuntze – – wfo-0001095177 – https://biodiversitylibrary.org/page/3817
Limonium delicatulum (Girard) Kuntze – – wfo-0000444403 – https://biodiversitylibrary.org/page/3817
Limonium delicatulum subsp. *afrum* Pignatti – – wfo-0000749433
Limonium delicatulum subsp. *angustebracteatum* (Erben) Rivas Mart. & M.J.Costa – – wfo-0001303572
Limonium delicatulum subsp. *angustibracteatum* (Erben) Rivas Mart. & M.J.Costa – – wfo-0001303573
Limonium delicatulum subsp. *biflorum* (Pignatti) O.Bolòs, Vigo, Masalles & Ninot – – wfo-0001260257
Limonium delicatulum subsp. *delicatulum* – – wfo-0001303574
Limonium delicatulum subsp. *eu-delicatulum* Maire – – wfo-0001303575 – https://bibdigital.rjb.csic.es/viewer/13176/?offset=#page=22&viewer=picture&o=bookmark&n=0&q=
Limonium delicatulum subsp. *formenterae* (L.Llorens) O.Bolòs, Vigo, Masalles & Ninot – – wfo-0001260258
Limonium delicatulum subsp. *gallicum* Pignatti – – wfo-0000749419
Limonium delicatulum subsp. *latebracteatum* (Erben) Castrov. & Cirujano – – wfo-0001303576
Limonium delicatulum subsp. *latibracteatum* (Erben) Castrov. & Cirujano – – wfo-0001303577 – https://biodiversitylibrary.org/page/15235852
Limonium delicatulum subsp. *migjornense* (L.Llorens) O.Bolòs, Vigo, Masalles & Ninot – – wfo-0001260259
Limonium delicatulum subsp. *orientale* Pignatti – – wfo-0001303578
Limonium delicatulum subsp. *retusum* (L.Llorens) O.Bolòs, Vigo, Masalles & Ninot – – wfo-0001260260
Limonium delicatulum subsp. *santapolense* (Erben) O.Bolòs, Vigo, Masalles & Ninot – – wfo-0001260261
Limonium delicatulum subsp. *tournefortii* (Girard) Pignatti – – wfo-0001303579
Limonium delicatulum subsp. *valentinum* Pignatti – – wfo-0001303580
Limonium delicatulum var. *leptostachys* (Pomel) Maire – – wfo-0001303581 – https://bibdigital.rjb.csic.es/viewer/13176/?offset=#page=22&viewer=picture&o=&n=0&q=statice
Limonium delicatulum var. *typicum* Maire – – wfo-0001303582 – https://bibdigital.rjb.csic.es/viewer/13176/?offset=#page=22&viewer=picture&o=&n=0&q=statice
Limonium dendroides Svent. – – wfo-0000444369
Limonium densiflorum (Guss.) Kuntze – – wfo-0000444368 – http://www.biodiversitylibrary.org/openurl?pid=title:327&volume=2&issue=&spage=395&date=1891
Limonium densiflorum Maire & Petitm. – – wfo-0001303583
Limonium densissimum (Pignatti) Pignatti – – wfo-0000444367 – http://onlinelibrary.wiley.com/doi/10.1111/j.1095-8339.1971.tb02152.x/full
Limonium depauperatum (Boiss.) R.A.Dyer – – wfo-0001095179 – https://doi.org/10.4102/abc.v7i3.1673

Limonium desertorum (Trautv.) Kuntze – – wfo-0000444364 – https://biodiversitylibrary.org/page/3817
Limonium devoniense (Ingr.) P.D.Sell – – wfo-0001303584
Limonium dianiae (Pau) A.Barber, M.B.Crespo & Lledó – – wfo-0000443104
Limonium dianium Pignatti – – wfo-0000443103 – https://doi.org/10.1080/00837792.1982.10670239
Limonium dichotomum (Cav.) Kuntze – – wfo-0000443102
Limonium dichroanthum (Rupr.) Ikonn.-Gal. ex Lincz. – – wfo-0001095230 – https://biodiversitylibrary.org/page/30218472
Limonium dictyocladum (Boiss.) Kuntze – – wfo-0000443120 – https://biodiversitylibrary.org/page/3817
Limonium dictyophorum (Tausch) Degen – – wfo-0000443108
Limonium didimense Doğan & Akaydın – – wfo-0001303585
Limonium diegoi Sennen – – wfo-0000443125
Limonium dielsianum (Wangerin) Kamelin – – wfo-0001095279 – https://biodiversitylibrary.org/page/639168
Limonium diffusum (Pourr.) Kuntze – – wfo-0000443124 – http://www.biodiversitylibrary.org/openurl?pid=title:327&volume=2&issue=&spage=395&date=1891
Limonium diomedeum Brullo – – wfo-0000443122 – http://www.jstor.org/stable/3996200
Limonium dissitiflorum (Boiss.) Kerguélen – – wfo-0001260263
Limonium distichum Wilmot-Dear – – wfo-0000443121 – https://www.jstor.org/stable/4109559?seq=1#page_scan_tab_contents
Limonium divaricatum (Rouy) Brullo – – wfo-0000443114 – http://journals.lub.lu.se/index.php/bn/article/view/11412/10558
Limonium dodartiforme Ingr. – – wfo-0000443119 – http://onlinelibrary.wiley.com/doi/10.1111/j.1095-8339.1986.tb01428.x/full
Limonium dodartii (Girard) Kuntze – – wfo-0000443126 – https://biodiversitylibrary.org/page/3817
Limonium dodartii subsp. *lusitanicum* (Daveau) Franco – – wfo-0001303586
Limonium doerfleri (Halácsy) Rech.f. – – wfo-0000443453 – https://www.zobodat.at/publikation_volumes.php?id=31166
Limonium dolihiense Erben & Brullo – – wfo-0001303587 – http://dx.doi.org/10.11646/phytotaxa.240.1.1
Limonium donegalense (Ingr.) P.D.Sell – – wfo-0001303588
Limonium donetzicum Klokov – – wfo-0000444362
Limonium doriae (Sommier) Pignatti – – wfo-0000444361 – https://doi.org/10.1080/00837792.1982.10670239
Limonium dragonericum Erben – – wfo-0000444360 – https://biodiversitylibrary.org/page/27802194
Limonium dregeanum (C.Presl) Kuntze – – wfo-0001095181 – https://biodiversitylibrary.org/page/3817
Limonium drepanostachyum Ikonn.-Gal. – – wfo-0000444359
Limonium drepanostachyum subsp. *callianthum* T.X.Peng – – wfo-0001095281
Limonium dubium (Andrews ex Guss.) Litard. – – wfo-0000444378
Limonium dubium Gamajun. ex Klokov – – wfo-0000444379
Limonium dubyi (Gren. & Godr.) Kuntze – – wfo-0000444366 – https://biodiversitylibrary.org/page/3817
Limonium dufourii (Girard) Kuntze – – wfo-0000444383 – https://biodiversitylibrary.org/page/3817
Limonium duriaei (Girard) Kuntze – – wfo-0001303589
Limonium durieui (Girard) Kuntze – – wfo-0000444382 – https://biodiversitylibrary.org/page/3817
Limonium durisculum subsp. *cavanillesii* (Erben) O.Bolòs, Vigo, Masalles & Ninot – – wfo-0001260265
Limonium duriusculum (Girard) Fourr. – – wfo-0000444381 – https://biodiversitylibrary.org/page/54553850
Limonium duriusculum (Girard) Kuntze – – wfo-0001303590 – https://biodiversitylibrary.org/page/3817
Limonium duriusculum subsp. *bianorii* (Sennen & Pau) Malag. – – wfo-0001303591
Limonium duriusculum subsp. *cavanillesii* (Erben) O.Bolòs, Vigo, Masalles & Ninot – – wfo-0001303592
Limonium duriusculum subsp. *companyonis* (Gren. & Billot) O.Bolòs, Vigo, Masalles & Ninot – – wfo-0001260267
Limonium duriusculum subsp. *duriusculum* – – wfo-0001303593
Limonium duriusculum subsp. *thiniense* (Erben) O.Bolòs, Vigo, Masalles & Ninot – – wfo-0001260268

Limonium duriusculum var. *robustior* Pignatti – – wfo-0001303594
Limonium dyeri Lincz. – – wfo-0001095183
Limonium ebusitanum (Font Quer) Font Quer – – wfo-0000444377
Limonium echinus (L.) Chaz. – – wfo-0001303595 – https://books.google.de/books?id=QjM_AAAAcAAJ&printsec=frontcover&hl=de&source=gbs_ge_summary_r&cad=0#v=onepage&q&f=false
Limonium echioides (L.) Mill. – – wfo-0000444376 – https://biodiversitylibrary.org/page/395149
Limonium echioides subsp. *eu-echioides* Maire – – wfo-0001303596 – https://bibdigital.rjb.csic.es/viewer/13176/?offset=#page=23&viewer=picture&o=search&n=0&q=statice
Limonium echioides subsp. *exaristatum* (Murb.) Maire – – wfo-0001303597 – https://bibdigital.rjb.csic.es/viewer/13176/?offset=#page=23&viewer=picture&o=search&n=0&q=statice
Limonium echoideum (L.) Mill. – – wfo-0001303598 – https://www.biodiversitylibrary.org/page/395149
Limonium effusum (Boiss.) Kuntze – – wfo-0000444375 – https://biodiversitylibrary.org/page/3817
Limonium ejulabilis Rosselló, Mus & J.X.Soler – – wfo-0000444374 – http://www.rjb.csic.es/jardinbotanico/jardin/contenido.php?Pag=219&tipo=volumenanales&vol=51(2)
Limonium elaphonisicum Alf.Mayer – – wfo-0000444373
Limonium elatum (Fisch. ex Spreng.) Kuntze – – wfo-0000444372 – https://biodiversitylibrary.org/page/3817
Limonium elfahsianum Brullo & Giusso – – wfo-0000509735 – http://ortobotanico.unipa.it/publications/bocconea19.html
Limonium emarginatum (Willd.) Kuntze – – wfo-0000444568 – https://biodiversitylibrary.org/page/3817
Limonium emporitanum Fern.Casas & Molero – – wfo-0000444339 – http://bibdigital.rjb.csic.es/ing/Libro.php?Libro=2931&Pagina=21
Limonium endlichianum (Wangerin) S.F.Blake – – wfo-0001286744 – http://www.biodiversitylibrary.org/openurl?pid=title:721&volume=18&issue=&spage=60&date=1916
Limonium equisetinum (Boiss.) R.A.Dyer – – wfo-0001095185 – https://www.jstor.org/stable/4115039?seq=1#page_scan_tab_contents
Limonium equisetinum var. *depauperatum* (Boiss.) Steenis – – wfo-0001304515
Limonium erectum Erben – – wfo-0000444693 – https://biodiversitylibrary.org/page/15235766
Limonium erythrorrhizum Ikonn.-Gal. ex Lincz. – – wfo-0001095342
Limonium erythrorrhizum var. *eriocalyx* Lincz. – – wfo-0001303599
Limonium estevei Fern.Casas – – wfo-0000444692
Limonium etruscum Arrigoni & Rizzotto – – wfo-0000444691 – https://doi.org/10.1080/00837792.1985.10670362
Limonium exaristatum (Murb.) P.Fourn. – – wfo-0000444689
Limonium eximium (Schrenk ex Fisch. & C.A.Mey.) H.Arnaud – – wfo-1200068001 – https://www.biodiversitylibrary.org/page/45269409
Limonium eximium (Schrenk) Kuntze – – wfo-0001095298 – http://www.biodiversitylibrary.org/openurl?pid=title:327&volume=2&issue=&spage=395&date=1891
Limonium eximum var. *album* F.T.Hubb. – – wfo-0001303600
Limonium eximum var. *superbum* F.T.Hubb. – – wfo-0001303601 – https://biodiversitylibrary.org/page/568409
Limonium failachicum Erben & Mucina – – wfo-0000444687 – https://link.springer.com/article/10.1007/BF02806481
Limonium fajzievii Zakirov ex Lincz. – – wfo-0000444685 – https://biodiversitylibrary.org/page/30218712
Limonium fallax (Coss. ex Wangerin) Maire – – wfo-0000444684 – https://bibdigital.rjb.csic.es/viewer/13176/?offset=#page=20&viewer=picture&o=bookmark&n=0&q=
Limonium fallax var. *trachycladum* (Maire & Wilczek) Maire – – wfo-0001303602
Limonium faustii Sennen & Mauricio – – wfo-0000444683 – http://bibdigital.rjb.csic.es/ing/Libro.php?Libro=42&Pagina=115
Limonium ferganense Ikonn.-Gal. – – wfo-0000444682
Limonium fergusoniae L.Bolus – – wfo-0000444681
Limonium ferulaceum (L.) Chaz. – – wfo-0001095145 – https://gallica.bnf.fr/ark:/12148/bpt6k15115421/f43.item
Limonium ferulaceum (L.) Kuntze – – wfo-0000444704 – https://biodiversitylibrary.org/page/3817
Limonium fesianum Erben – – wfo-0000444688 – https://biodiversitylibrary.org/page/15042591

Limonium fischeri (Trautv.) Lincz. – – wfo-0000444690 – https://biodiversitylibrary.org/page/30218472
Limonium flagellare (Lojac.) Brullo – – wfo-0000444710 – http://journals.lub.lu.se/index.php/bn/article/view/11412/10558
Limonium flexuosum (L.) Chaz. – – wfo-0001303603 – https://gallica.bnf.fr/ark:/12148/bpt6k15115421/f42.item
Limonium flexuosum (L.) Kuntze – – wfo-0000444709 – http://www.biodiversitylibrary.org/openurl?pid=title:327&volume=2&issue=&spage=395&date=1891
Limonium flexuosum Sennen – – wfo-0001303604 – https://bibdigital.rjb.csic.es/viewer/9472/?offset=#page=74&viewer=picture&o=bookmark&n=0&q=
Limonium florentinum Arrigoni & Diana – – wfo-0000444708
Limonium fontqueri (Pau) L.Llorens ex Greuter – – wfo-0000444707 – http://bibdigital.rjb.csic.es/spa/Libro.php?Libro=2743&Pagina=57
Limonium formenterae L.Llorens – – wfo-0001095268
Limonium formosum Bartolo, Brullo & Giusso – – wfo-0000447140 – http://www.herbmedit.org/bocconea16.html
Limonium fortunei (Lindl.) H.Arnaud – – wfo-1000055773 – https://www.biodiversitylibrary.org/page/45269408
Limonium fradinianum (Pomel) Erben – – wfo-0001303605 – http://www.herbmedit.org/flora22.html
Limonium fragile Erben & Brullo – – wfo-0001303606 – http://dx.doi.org/10.11646/phytotaxa.240.1.1
Limonium franchetii (Debeaux) Kuntze – – wfo-0001095310 – http://www.biodiversitylibrary.org/openurl?pid=title:327&volume=2&issue=&spage=395&date=1891
Limonium frederici (Barbey) Rech.f. – – wfo-0000444695 – https://www.zobodat.at/publikation_volumes.php?id=31166
Limonium frutescens (Lem.) Erben, A.Santos & Reyes-Bet. – – wfo-0000444705 – http://www.herbmedit.org/flora22.html
Limonium fruticans (Webb ex Boiss.) Kuntze – – wfo-0001303607 – https://biodiversitylibrary.org/page/3817
Limonium fruticosum Mill. – – wfo-0000444712 – https://biodiversitylibrary.org/page/395149
Limonium furfuraceum (Lag.) Kuntze – – wfo-0000444702 – https://biodiversitylibrary.org/page/3817
Limonium furfuraceum subsp. *lucentinum* (Pignatti & Freitag) O.Bolòs & Vigo – – wfo-0000444701
Limonium furnarii Brullo – – wfo-0000444700 – http://journals.lub.lu.se/index.php/bn/article/view/11412/10558
Limonium gabrieli (Bornm.) Rech.f. – – wfo-0000444699
Limonium galilaeum Domina, Danin & Raimondo – – wfo-0000507525 – http://www.herbmedit.org/flora16.html
Limonium gallicum (Pignatti) Domina – – wfo-0000749414 – http://www.bioone.org/doi/abs/10.3372/wi.41.41117
Limonium gallurense Arrigoni & Diana – – wfo-0000444698
Limonium gavilae Sennen & Mauricio – – wfo-0000444678
Limonium gerberi Soldano – – wfo-0000444706
Limonium geronense Erben – – wfo-0000444680 – https://biodiversitylibrary.org/page/15235802
Limonium giberti (Sennen) Sennen – – wfo-0001303608
Limonium gibertii (Sennen) Sennen – – wfo-0000745499 – https://bibdigital.rjb.csic.es/viewer/9472/?offset=#page=272&viewer=picture&o=bookmark&n=0&q=
Limonium ginae P.D.Sell – – wfo-0001303610
Limonium ginzbergeri Bogdanović & Brullo – – wfo-0001303611 – http://dx.doi.org/10.11646/phytotaxa.215.1.1
Limonium girardianum (Guss.) Fourr. – – wfo-0000444662 – https://biodiversitylibrary.org/page/54553850
Limonium girardianum (Guss.) Kuntze – – wfo-0001303612 – https://biodiversitylibrary.org/page/3817
Limonium girardianum subsp. *balearicum* (Pignatti) O.Bolòs, Vigo, Masalles & Ninot – – wfo-0001260270
Limonium girardianum subsp. *grosii* (L.Llorens) O.Bolòs, Vigo, Masalles & Ninot – – wfo-0001260271
Limonium girardianum subsp. *majoricum* (Pignatti) O.Bolòs, Vigo, Masalles & Ninot – – wfo-0001260272
Limonium girardianum subsp. *willdenowii* P.Fourn. – – wfo-0001303613
Limonium globulariifolium (Desf.) Kuntze – – wfo-0000444650 – https://biodiversitylibrary.org/page/3817
Limonium globulariifolium subsp. *globulariifolium* (Desf.) Kuntze – – wfo-0001303614
Limonium globulariifolium subsp. *lusitanicum* Pignatti – – wfo-0000745496
Limonium globulariifolium subsp. *provinciale* Pignatti – – wfo-0000745493

Limonium globulariifolium subsp. *tommasinii* Pignatti – – wfo-0000745494
Limonium globuliferum (Boiss. & Heldr. ex Boiss.) Kuntze – – wfo-0000444659 – https://biodiversitylibrary.org/page/3817
Limonium glomeratum (Tausch) Erben – – wfo-0000444664 – https://biodiversitylibrary.org/page/15043350
Limonium gmelini (Willd.) Kuntze – – wfo-0000444657 – http://www.biodiversitylibrary.org/openurl?pid=title:327&volume=2&issue=&spage=395&date=1891
Limonium gmelini f. *laxiflorum* C.E.Salmon – – wfo-0001303615 – https://www.biodiversitylibrary.org/page/35193373
Limonium gmelini var. *meyeri* C.E.Salmon – – wfo-0001303616 – https://www.biodiversitylibrary.org/page/35193373
Limonium gmelinii (Willd.) Kuntze – – wfo-0001303617 – https://biodiversitylibrary.org/page/3817
Limonium gmelinii subsp. *hungaricum* (Klokov) Soó – – wfo-0001303618
Limonium gmelinii subsp. *hypanicum* (Klokov) Sóo – – wfo-0001095231
Limonium gmelinii var. *hypanicum* Pawł. – – wfo-0001095232
Limonium gobicum Ikonn.-Gal. – – wfo-0000444656
Limonium gomezi-jordanae Sennen & Mauricio – – wfo-0000444655 – http://bibdigital.rjb.csic.es/ing/Libro.php?Libro=42&Pagina=115
Limonium gorgonae Pignatti – – wfo-0000444654 – https://doi.org/10.1080/00837792.1982.10670239
Limonium gougetianum (Girard) Kuntze – – wfo-0000444651 – https://biodiversitylibrary.org/page/3817
Limonium gougetianum subsp. *balearicum* Pignatti – – wfo-0001260269
Limonium gougetianum subsp. *multiceps* (Pomel) Quézel & Santa ex Greuter & Raus – – wfo-0001303619 – http://www.jstor.org/stable/3996512
Limonium gougetianum var. *multiceps* (Pomel) Maire – – wfo-0001303620
Limonium grabusae Erben & Brullo – – wfo-0001303621 – http://dx.doi.org/10.11646/phytotaxa.240.1.1
Limonium graeca Post – – wfo-0001303622
Limonium graecum (Poir.) Kuntze – – wfo-0000444672 – https://biodiversitylibrary.org/page/53057190
Limonium graecum (Poir.) Rech.f. – – wfo-0001303623
Limonium graecum Mouterde – – wfo-0001303624
Limonium graecum subsp. *ammophilon* Papatsou & Phitos – – wfo-0000749416
Limonium graecum subsp. *divaricatum* (Pignatti) Pignatti – – wfo-0000444676
Limonium graecum subsp. *graecum* – – wfo-0001303625
Limonium graecum var. *hyssopifolium* (Girard) Bokhari – – wfo-0000444675
Limonium graecum var. *palmare* (Sm.) Rech.f. – – wfo-0001303626 – https://www.zobodat.at/publikation_volumes.php?id=31166
Limonium graecum var. *stenotatum* Rech.f. – – wfo-0001303627
Limonium graminifolium (Aitch.) Kuntze – – wfo-0000444674 – https://biodiversitylibrary.org/page/3817
Limonium grandicaule (Ingr.) P.D.Sell – – wfo-0001303628
Limonium greuteri Erben – – wfo-0000507526 – https://doi.org/10.3372/wi.36.36111
Limonium griffithii (Aitch. & Hemsl.) Kuntze – – wfo-0000444673 – http://bibdigital.rjb.csic.es/ing/Libro.php?Libro=5480&Pagina=21
Limonium grosii L.Llorens – – wfo-0001095266
Limonium grubovii Lincz. – – wfo-0000444671
Limonium guaicuru (Molina) Kuntze – – wfo-0001095206 – https://biodiversitylibrary.org/page/3817
Limonium gueneri Doğan, H.Duman & Akaydın – – wfo-0000509736
Limonium guigliae Raimondo & Domina – – wfo-0000746746
Limonium gummiferum (Durieu ex Boiss. & Reut.) Kuntze – – wfo-0000444670 – https://biodiversitylibrary.org/page/3817
Limonium gummiferum subsp. *battandieri* Sauvage & Vindt – – wfo-0001303629
Limonium gummiferum subsp. *cymuliferum* – – wfo-0001303630
Limonium gummiferum subsp. *eu-gummiferum* Maire – – wfo-0001303631

Limonium gummiferum subsp. *gummiferum* – – wfo-0001303632
Limonium gummiferum subsp. *sebkarum* (Pomel) Maire – – wfo-0001303633
Limonium gummiferum var. *corymbulosum* (Coss.) Maire – – wfo-0001303634 – https://bibdigital.rjb.csic.es/viewer/13176/?offset=#page=22&viewer=picture&o=bookmark&n=0&q=
Limonium gummiferum var. *muticum* Maire & Sennen – – wfo-0001303635
Limonium gummiferum var. *typicum* Maire – – wfo-0001303636
Limonium gussonei (Tineo ex Lojac.) Giardina & Raimondo – – wfo-0000509738 – http://www.herbmedit.org/bocconea20.html
Limonium gymnesicum Erben – – wfo-0000444669 – https://biodiversitylibrary.org/page/14863475
Limonium haitense S.F.Blake – – wfo-0001033127
Limonium haitiense S.F.Blake – – wfo-0001303637 – https://www.biodiversitylibrary.org/page/39914355
Limonium halophilum Pignatti ex Brullo – – wfo-0000444667 – http://journals.lub.lu.se/index.php/bn/article/view/11412/10558
Limonium helenae Erben & Brullo – – wfo-0001303638 – http://dx.doi.org/10.11646/phytotaxa.240.1.1
Limonium heraionense Erben & Brullo – – wfo-0001303639 – http://dx.doi.org/10.11646/phytotaxa.240.1.1
Limonium herculis Pignatti – – wfo-0000444666 – https://doi.org/10.1080/00837792.1982.10670239
Limonium hermaeum (Pignatti) Pignatti – – wfo-0000444665 – http://onlinelibrary.wiley.com/doi/10.1111/j.1095-8339.1971.tb02152.x/full
Limonium heterobracteatum Erben – – wfo-0000444718 – https://biodiversitylibrary.org/page/15206062
Limonium heterospicatum Erben – – wfo-0000444658 – https://biodiversitylibrary.org/page/27802232
Limonium hibericum Erben – – wfo-0000444696 – https://biodiversitylibrary.org/page/15148004
Limonium hibernicum (Ingr.) P.D.Sell – – wfo-0001303640
Limonium hierapetrae Rech.f. – – wfo-0000444760
Limonium himariense F.K.Mey. – – wfo-0000749683
Limonium hipponense Brullo – – wfo-0000444759 – https://biodiversitylibrary.org/page/27802281
Limonium hirsuticalyx Pignatti – – wfo-0000444758 – http://onlinelibrary.wiley.com/doi/10.1111/j.1095-8339.1971.tb02152.x/full
Limonium hoeltzeri (Regel) Ikonn.-Gal. – – wfo-0001095364 – https://biodiversitylibrary.org/page/30218471
Limonium humboldtii (Bolle) Kuntze – – wfo-0000444757 – https://biodiversitylibrary.org/page/3817
Limonium humile Mill. – – wfo-0000444756 – https://biodiversitylibrary.org/page/395148
Limonium hungaricum Klokov – – wfo-0000444754
Limonium hyblaeum Brullo – – wfo-0000444743 – http://journals.lub.lu.se/index.php/bn/article/view/11412/10558
Limonium hypanicum Klokov – – wfo-0000444752
Limonium hyssopifolium (Girard) Rech.f. – – wfo-0000444761 – https://www.zobodat.at/publikation_volumes.php?id=31166
Limonium iconicum (Boiss. & Heldr.) Kuntze – – wfo-0001303641 – http://www.biodiversitylibrary.org/openurl?pid=title:327&volume=2&issue=&spage=395&date=1891
Limonium iconium (Boiss. & Heldr.) Kuntze – – wfo-0001095256
Limonium ifniense Caball. – – wfo-0000444750 – URL:http://bibdigital.rjb.csic.es/ing/Libro.php?Libro=708&Pagina=9
Limonium ifniensis Caball. – – wfo-0001303642
Limonium ikaricum Erben & Brullo – – wfo-0001303643 – http://dx.doi.org/10.11646/phytotaxa.240.1.1
Limonium ikonnikovii-galitzkyi A.V.Grebenjuk – – wfo-0001303644
Limonium ilerdense Erben – – wfo-0000444749 – https://biodiversitylibrary.org/page/15148005
Limonium ilergabonum López-Alvarado, Cobacho, Arán & L.Sáez – – wfo-0001303645 – https://doi.org/10.11646/phytotaxa.331.2.4
Limonium iljinii Sobolevsk. – – wfo-0000444748
Limonium ilvae Pignatti – – wfo-0000444747 – https://doi.org/10.1080/00837792.1982.10670239
Limonium imbricatum (Webb ex Girard) F.T.Hubb. ex L.H.Bailey – – wfo-0000444746 – https://biodiversitylibrary.org/page/568409

Limonium imbricatum (Webb ex Girard) H.Arnaud – – wfo-0001303646
Limonium inarimense (Guss.) Pignatti – – wfo-0000444770
Limonium inarimense subsp. *ebusitanum* (Font Quer) Pignatti – – wfo-0000745483
Limonium incanum (L.) Chaz. – – wfo-0001303647 – https://gallica.bnf.fr/ark:/12148/bpt6k15115421/f42.item
Limonium incanum (L.) Kuntze – – wfo-0000444751 – https://biodiversitylibrary.org/page/3817
Limonium incertum H.Arnaud – – wfo-0001303648
Limonium inexpectans L.Sáez & Rosselló – – wfo-0000444753 – http://www.rjb.csic.es/jardinbotanico/jardin/contenido.php?Pag=219&tipo=volumenanales&vol=54(1)
Limonium insigne (Coss.) Kuntze – – wfo-0000444778 – http://www.biodiversitylibrary.org/openurl?pid=title:327&volume=2&issue=&spage=395&date=1891
Limonium insigne subsp. *carthaginensis* Pignatti – – wfo-0001303649
Limonium insigne subsp. *carthaginiense* Pignatti – – wfo-0001303650
Limonium insigne subsp. *insigne* – – wfo-0001303651 – http://bibdigital.rjb.csic.es/ing/Libro.php?Libro=2837&Pagina=303
Limonium insigne var. *insigne* – – wfo-0001303652 – http://bibdigital.rjb.csic.es/ing/Libro.php?Libro=2837&Pagina=304
Limonium insigne var. *paui* Pignatti – – wfo-0001303653 – http://bibdigital.rjb.csic.es/ing/Libro.php?Libro=2837&Pagina=303
Limonium insigne var. *rossmaessleri* (Willk.) Pignatti – – wfo-0001303654 – http://bibdigital.rjb.csic.es/ing/Libro.php?Libro=2837&Pagina=304
Limonium insulare (Bég. & Landi) Arrigoni & Diana – – wfo-0001260136
Limonium intercedens P.D.Sell – – wfo-0001303655
Limonium intermedium (Guss.) Brullo – – wfo-0000444776 – http://journals.lub.lu.se/index.php/bn/article/view/11412/10558
Limonium intricatum Brullo – – wfo-0000444774 – http://www.biodiversitylibrary.org/openurl?pid=title:14894&volume=28&issue=&spage=419&date=1989
Limonium ionicum Brullo – – wfo-0000444773 – http://journals.lub.lu.se/index.php/bn/article/view/11412/10558
Limonium iranicum (Bornm.) Lincz. – – wfo-0000444772 – https://biodiversitylibrary.org/page/30218496
Limonium irtaense Ferrer-Gallego, P. P., A.Navarro, P.Pérez, R.Roselló, Rosselló, M.Rosato & E.Laguna – – wfo-0001303656 – http://dx.doi.org/10.11646/phytotaxa.234.3.7
Limonium isidorum Erben & Brullo – – wfo-0001303657 – http://dx.doi.org/10.11646/phytotaxa.240.1.1
Limonium issaeum Bogdanović & Brullo – – wfo-0001303658 – http://dx.doi.org/10.11646/phytotaxa.215.1.1
Limonium istriacum Bogdanović & Brullo – – wfo-0001303659 – http://dx.doi.org/10.11646/phytotaxa.215.1.1
Limonium ithacense R.Artelari – – wfo-0000444762
Limonium jankae (Lojac.) Giardina & Raimondo – – wfo-0000509739 – http://www.herbmedit.org/bocconea20.html
Limonium japonicum (Siebold & Zucc.) Kuntze – – wfo-0000444779 – https://biodiversitylibrary.org/page/3817
Limonium japygicum (E.Groves) Pignatti – – wfo-0000444769 – http://onlinelibrary.wiley.com/doi/10.1111/j.1095-8339.1971.tb02152.x/full
Limonium japygicum (E.Groves) Pignatti, Galasso & Nicolella – – wfo-0001303660 – http://www.societabotanicaitaliana.it/SBI/fascicolo.asp?cartella=IBI_46_(1)_2014
Limonium jarmolenkoi (Lincz.) M.Malekm., Akhani & Borsch – – wfo-0001303661 – https://doi.org/10.12705/665.8
Limonium johannis Pignatti – – wfo-0000444768
Limonium jovibarba (Webb ex Boiss.) Kuntze – – wfo-0000444767 – https://biodiversitylibrary.org/page/3817
Limonium kairouanum Brullo – – wfo-0000444766 – https://www.biodiversitylibrary.org/page/27802287
Limonium kardamylii R.Artelari & Kamari – – wfo-0000444765
Limonium karpasiticum Kefalas, Erben, Christodoulou & Hand – – wfo-1000047128 – https://doi.org/10.7320/FlMedit32.035
Limonium kaschgaricum (Rupr.) Ikonn.-Gal. – – wfo-0001095233
Limonium kaufmannianum (Regel) Kuntze – – wfo-0001095290 – http://www.biodiversitylibrary.org/openurl?pid=title:327&volume=2&issue=&spage=395&date=1891
Limonium kelseyanum (Ingr.) P.D.Sell – – wfo-0001303662

Limonium kerryense (Ingr.) P.D.Sell – – wfo-0001303663
Limonium kimmericum (Lipsky) Klokov – – wfo-0001303664
Limonium kirikosicum Erben & Brullo – – wfo-0001303665 – http://dx.doi.org/10.11646/phytotaxa.240.1.1
Limonium klementzii Ikonn.-Gal. – – wfo-0000444771
Limonium kobstanicum Tzvelev – – wfo-0001303666
Limonium komarovii Ikonn.-Gal. – – wfo-0000444725
Limonium komarovii Ikonn.-Gal. ex Lincz. & Czukav. – – wfo-0000444744
Limonium korakonisicum Valli – – wfo-0001303667 – http://dx.doi.org/10.11646/phytotaxa.217.1.5
Limonium korbousense Brullo – – wfo-0000444724 – http://www.biodiversitylibrary.org/openurl?pid=title:14894&volume=28&issue=&spage=419&date=1989
Limonium kossmatii (R.Wagner & Vierh.) Verdc. & Hemming ex Cufod. – – wfo-0000444723 – https://www.jstor.org/stable/3667348
Limonium kraussianum (Buchinger ex Boiss.) Kuntze – – wfo-0001095187 – https://biodiversitylibrary.org/page/3817
Limonium ksamilum Bogdanović, Shuka, Giusso & Brullo – – wfo-0001303668
Limonium kurgantjubense (Lincz.) M.Malekm., Akhani & Borsch – – wfo-0001303669 – https://doi.org/10.12705/665.8
Limonium lacertosum Brullo – – wfo-0000444722 – http://www.biodiversitylibrary.org/openurl?pid=title:14894&volume=28&issue=&spage=419&date=1989
Limonium lacinium Arrigoni – – wfo-0000444721
Limonium lacostei (Danguy) Kamelin – – wfo-0001095278 – https://biodiversitylibrary.org/page/639168
Limonium ladikanum Erben & Brullo – – wfo-0001303670
Limonium laetum (Nyman) Pignatti – – wfo-0000444719 – http://onlinelibrary.wiley.com/doi/10.1111/j.1095-8339.1971.tb02152.x/full
Limonium laetum subsp. *insulare* (Bég. & Landi) Atzei & V.Picci – – wfo-0001303671
Limonium lagostanum Bogdanović & Brullo – – wfo-0001303672 – http://dx.doi.org/10.11646/phytotaxa.215.1.1
Limonium lajaconi Brullo – – wfo-0001303673
Limonium lambinonii Erben – – wfo-0000444726 – https://biodiversitylibrary.org/page/28677727
Limonium lanceolatum (Hoffmanns. & Link) Franco – – wfo-0000444717
Limonium lanfrancoi Agius, M.E.Galea, Cambria, del Galdo & Brullo – – wfo-1000055932
Limonium latebracteatum Erben – – wfo-0001303674
Limonium latibracteatum Erben – – wfo-0000444716 – https://biodiversitylibrary.org/page/15235851
Limonium latifolium (Sm.) Kuntze – – wfo-0000444715 – https://biodiversitylibrary.org/page/3817
Limonium latifolium Moench – – wfo-0001303675
Limonium latifolium var. *album* F.T.Hubb. – – wfo-0001303676 – https://biodiversitylibrary.org/page/568409
Limonium lausianum Pignatti – – wfo-0000444714 – http://onlinelibrary.wiley.com/doi/10.1111/j.1095-8339.1971.tb02152.x/full
Limonium laxiusculum Franco – – wfo-0000444713
Limonium lefroyi (Hemsl.) Britton – – wfo-0000447135
Limonium legrandii (Gaut. & Timb.-Lagr.) Erben – – wfo-0000444734 – https://biodiversitylibrary.org/page/15235810
Limonium leonardi-llorensii L.Sáez, Á.C.Carvalho & Rosselló – – wfo-0000444735 – http://rjb.revistas.csic.es/index.php/rjb/article/view/218/215
Limonium leprosorum Bogdanović & Brullo – – wfo-0001303677 – http://dx.doi.org/10.11646/phytotaxa.215.1.1
Limonium leptolobum (Regel) Kuntze – – wfo-0001095234 – https://biodiversitylibrary.org/page/3817
Limonium leptophyllum (Schrenk) Kuntze – – wfo-0000444742 – https://biodiversitylibrary.org/page/3817
Limonium leptostachyus (Boiss.) Kuntze – – wfo-0000444741 – https://biodiversitylibrary.org/page/3817
Limonium lessingianum Lincz. – – wfo-0000444740
Limonium letourneuxii (Coss. ex Batt.) A.Pons & Quézel – – wfo-0000444739
Limonium letourneuxii (Coss. ex Batt.) Greuter & Raus – – wfo-0000444738 – http://www.jstor.org/stable/3996512

Limonium liberianum Bogdanović & Brullo – – wfo-0001303678 – http://dx.doi.org/10.11646/phytotaxa.215.1.1
Limonium liburnicum Bogdanović & Brullo – – wfo-0001303679 – http://dx.doi.org/10.11646/phytotaxa.215.1.1
Limonium lilacinum (Boiss. & Balansa) Wagenitz – – wfo-0000444737 – https://www.jstor.org/stable/3995371?seq=1#page_scan_tab_contents
Limonium lilacinum var. *laxiflorum* Doğan & Akaydın – – wfo-0001303680
Limonium lilacinum var. *lilacinum* – – wfo-0001303681
Limonium lilybaeum Brullo – – wfo-0000444727 – http://journals.lub.lu.se/index.php/bn/article/view/11412/10558
Limonium limbatum Small – – wfo-0001095159 – http://www.biodiversitylibrary.org/openurl?pid=title:340&volume=25&issue=6&spage=317&date=1898
Limonium limbatum var. *glabrescens* Correll – – wfo-0001095160 – http://www.biodiversitylibrary.org/openurl?pid=title:721&volume=68&issue=776&spage=425&date=1966
Limonium limonium (L.) A.Lyons – – wfo-0000444733 – https://biodiversitylibrary.org/page/11442879
Limonium limonium Druce – – wfo-0001303682 – http://archive.bsbi.org.uk/bec_reports.html
Limonium linguum (Pomel) Pons, Quézel, Quezel & Santa – – wfo-0000444732
Limonium linifolium (L.f.) Chaz. – – wfo-0001303683 – https://gallica.bnf.fr/ark:/12148/bpt6k15115421/f43.item
Limonium linifolium var. *linifolium* (L.f.) Chaz. – – wfo-0001095189
Limonium linifolium (L.f.) Kuntze – – wfo-0001095150 – https://biodiversitylibrary.org/page/3817
Limonium linifolium var. *maritimum* (Eckl. & Zeyh. ex Boiss.) R.A.Dyer – – wfo-0001095190 – https://doi.org/10.4102/abc.v7i3.1673
Limonium lobatum (L.f.) Chaz. – – wfo-0001303684 – https://gallica.bnf.fr/ark:/12148/bpt6k15115421/f44.item
Limonium lobatum (L.f.) Kuntze – – wfo-0000444731 – https://biodiversitylibrary.org/page/3817
Limonium lobetanicum Erben – – wfo-0000444730 – http://www.biodiversitylibrary.org/openurl?pid=title:14894&volume=28&issue=&spage=313&date=1989
Limonium lobinii N.Kilian & T.Leyens – – wfo-0001239902 – https://www.jstor.org/stable/3996679?seq=1#page_scan_tab_contents
Limonium loganicum Ingr. – – wfo-0000444729 – http://onlinelibrary.wiley.com/doi/10.1111/j.1095-8339.1986.tb01428.x/full
Limonium lojaconoi Brullo – – wfo-0000444632 – http://journals.lub.lu.se/index.php/bn/article/view/11412/10558
Limonium longibracteatum Erben – – wfo-0000444530 – https://biodiversitylibrary.org/page/15148012
Limonium longifolium (Thunb.) R.A.Dyer – – wfo-0001095192 – https://doi.org/10.4102/abc.v7i3.1673
Limonium longispicatum Erben – – wfo-0000444567 – https://biodiversitylibrary.org/page/15235890
Limonium lopadusanum Brullo – – wfo-0000444566 – http://journals.lub.lu.se/index.php/bn/article/view/11412/10558
Limonium lovricii Bogdanović & Brullo – – wfo-0001303685 – http://dx.doi.org/10.11646/phytotaxa.215.1.1
Limonium lowei R.Jardim, M.Seq., Capelo, J.C.Costa & Rivas Mart. – – wfo-0000746705
Limonium lychnidifolium (Girard) Kuntze – – wfo-0000444563 – https://biodiversitylibrary.org/page/3817
Limonium lychnidifolium var. *corymbosum* (Boiss.) C.E.Salmon – – wfo-0001303686
Limonium macrophyllum (Willd. ex Spreng.) H.Arnaud – – wfo-0001303687
Limonium macrophyllum (Willd. ex Spreng.) Kuntze – – wfo-0000444562 – https://biodiversitylibrary.org/page/3817
Limonium macropterum (Webb & Berthel.) Kuntze – – wfo-0000444561 – https://biodiversitylibrary.org/page/3817
Limonium macrorhabdon (Boiss.) Kuntze – – wfo-0000444549 – http://bibdigital.rjb.csic.es/ing/Libro.php?Libro=5480&Pagina=21
Limonium macrorrhizum (Ledeb.) Kuntze – – wfo-0000444559 – https://biodiversitylibrary.org/page/3817
Limonium magallufianum L.Llorens – – wfo-0000444569
Limonium majoricum Pignatti – – wfo-0000444557
Limonium majus (Boiss.) Erben – – wfo-0000444556 – https://biodiversitylibrary.org/page/15235887
Limonium malacitanum B.Díez – – wfo-0000444555
Limonium malfatanicum Erben – – wfo-0000444554 – https://biodiversitylibrary.org/page/15042583
Limonium mansanetianum M.B.Crespo & Lledó – – wfo-0000444552

Limonium mareoticum El Garf ex Hadidi & Fayed – – wfo-0000444551
Limonium marisolii L.Llorens – – wfo-0000444574
Limonium maritimum Caperta, Cortinhas, A.P.Paes, Guara, Esp.Santo & Erben – – wfo-0001303688 – https://watermark.silverchair.com/mcu186.pdf?token=AQECAHi208BE49Ooan9kkhW_Ercy7Dm3ZL_9Cf3qfKAc485ysgAAA2QwggNgBgkqhkiG9w0BBwagggNRMIIDTQIBADCCA0YGCSqGSIb3DQEHATAeBglghkgBZQMEAS4wEQQMx2ooofNRiQ5wqIg_AgEQgIIDFwF7iJ2oFOb0tTuhr_tiOUDMmtYLnDosonOhfpKM0UBOonP6
Limonium marmarisense Doğan & Akaydın – – wfo-0001303689
Limonium maroccanum (Batt. & Trab.) Domina – – wfo-0000749440 – http://www.bioone.org/doi/abs/10.3372/wi.41.41117
Limonium mateoi Erben & Arán – – wfo-0000444558
Limonium mauritanicum Hutch. & Dalziel – – wfo-0000444560
Limonium maurocordatae (Schweinf. & Volkens) Cufod. – – wfo-0000444581 – http://www.jstor.org/stable/3667449, http://www.jstor.org/stable/3667348
Limonium mazarae Pignatti ex Brullo – – wfo-0000444580 – http://journals.lub.lu.se/index.php/bn/article/view/11412/10558
Limonium meandrinum Erben & Brullo – – wfo-0001303690 – http://dx.doi.org/10.11646/phytotaxa.240.1.1
Limonium medium (Ingr.) P.D.Sell – – wfo-0001303691
Limonium melancholicum Brullo, Marcenò & S.Romano – – wfo-0000444578
Limonium melitense Brullo – – wfo-0000444577 – http://www.jstor.org/stable/3996200
Limonium melium (Nyman) Pignatti – – wfo-0000444576 – http://onlinelibrary.wiley.com/doi/10.1111/j.1095-8339.1971.tb02152.x/full
Limonium membranaceum (Czern. ex Trautv.) Klokov – – wfo-0001095235
Limonium membranaceum R.A.Dyer – – wfo-0001095184 – https://doi.org/10.4102/abc.v7i3.1673
Limonium menigense Brullo – – wfo-0000444575 – http://www.biodiversitylibrary.org/openurl?pid=title:14894&volume=28&issue=&spage=419&date=1989
Limonium merxmuelleri Erben – – wfo-0000444582
Limonium merxmuelleri subsp. *merxmuelleri* – – wfo-0001303692
Limonium merxmuelleri subsp. *oristanum* (Alf.Mayer) Arrigoni – – wfo-0001303693
Limonium merxmuelleri subsp. *sulcitanum* (Arrigoni) Arrigoni – – wfo-0001303694
Limonium merxmuelleri subsp. *tigulianum* (Arrigoni & Diana) Arrigoni – – wfo-0001303695
Limonium messeniacum R.Artelari & Kamari – – wfo-0000444572
Limonium mexicanum S.F.Blake – – wfo-0001095208 – http://www.biodiversitylibrary.org/openurl?pid=title:721&volume=18&issue=207&spage=59&date=1916
Limonium meyeri (Boiss.) Kuntze – – wfo-0000444571 – https://biodiversitylibrary.org/page/3817
Limonium michelsonii Lincz. – – wfo-0000444547 – https://biodiversitylibrary.org/page/30218710
Limonium microcycladicum Erben & Brullo – – wfo-0001303696 – http://dx.doi.org/10.11646/phytotaxa.240.1.1
Limonium migjornense L.Llorens – – wfo-0001095265
Limonium Mill. – – wfo-4000021808 – https://biodiversitylibrary.org/page/44046355
Limonium milleri Ghaz. & J.R.Edm. – – wfo-0000444550 – https://doi.org/10.1017/S0960428603000027
Limonium milovicii Bogdanović & Brullo – – wfo-0001303697 – http://dx.doi.org/10.11646/phytotaxa.215.1.1
Limonium minoicum Erben & Brullo – – wfo-0001303698 – http://dx.doi.org/10.11646/phytotaxa.240.1.1
Limonium minoricense Erben – – wfo-0000444529 – https://biodiversitylibrary.org/page/27802217
Limonium minus (Boiss.) Erben – – wfo-0000444528 – https://biodiversitylibrary.org/page/15148001
Limonium minutiflorum (Guss.) Kuntze – – wfo-0000444527 – https://biodiversitylibrary.org/page/3817
Limonium minutiflorum subsp. *balearicum* Pignatti – – wfo-0001303699
Limonium minutum (L.) Chaz. – – wfo-0001303700 – https://gallica.bnf.fr/ark:/12148/bpt6k15115421/f43.item
Limonium minutum (L.) Fourr. – – wfo-0000444526 – https://biodiversitylibrary.org/page/54553850
Limonium minutum (L.) Kuntze – – wfo-0001303701 – https://biodiversitylibrary.org/page/3817

Limonium minutum f. *puberulum* C.E.Salmon – – wfo-0001303702 – http://archive.bsbi.org.uk/journal_of_botany.html
Limonium minutum subsp. *acutifolium* (Rchb.) P.Fourn. – – wfo-0001303703
Limonium minutum subsp. *aragonense* De Litard. – – wfo-0001303704
Limonium minutum subsp. *caprariense* Font Quer & Marcos – – wfo-0000748115
Limonium minutum subsp. *escarrei* (L.Llorens & Tébar) O.Bolòs, Vigo, Masalles & Ninot – – wfo-0001260275
Limonium minutum subsp. *minutum* – – wfo-0001303705
Limonium minutum subsp. *pericotii* O.Bolòs & Vigo – – wfo-0000444518
Limonium minutum subsp. *pseudominutum* (Erben) O.Bolòs & Vigo – – wfo-0001303706
Limonium minutum subsp. *revolutum* (Erben) O.Bolòs & Vigo – – wfo-0001260277
Limonium minutum subsp. *tremolsii* (Rouy) P.Fourn. – – wfo-0000443965
Limonium minutum var. *dissitiflorum* (Boiss.) C.E.Salmon – – wfo-0001303707 – http://archive.bsbi.org.uk/journal_of_botany.html
Limonium minutum var. *medense* O.Bolòs & Vigo – – wfo-0001303708
Limonium minutum var. *microphyllum* C.E.Salmon – – wfo-0001303709 – http://archive.bsbi.org.uk/journal_of_botany.html
Limonium minutum var. *pseudebusitanum* (Erben) O.Bolòs & Vigo – – wfo-0001303710
Limonium molesii Sennen & Mauricio – – wfo-0000444523 – http://bibdigital.rjb.csic.es/ing/Libro.php?Libro=42&Pagina=115
Limonium monolithicum Erben & Brullo – – wfo-0001303711 – http://dx.doi.org/10.11646/phytotaxa.240.1.1
Limonium monopetalum (L.) Hill – – wfo-0000444522
Limonium montis-christi Rizzotto – – wfo-0000444521 – https://doi.org/10.1080/00837792.1999.10670663
Limonium morisianum Arrigoni – – wfo-0000444520
Limonium motianum Brullo ex Erben, Del Guacchio & P.Caputo – – wfo-0001303712
Limonium mouretii (Pit.) Maire – – wfo-0000444538 – http://bibdigital.rjb.csic.es/ing/Libro.php?Libro=4268&Pagina=21
Limonium mouretii var. *coloratum* Maire – – wfo-0001303713
Limonium mouretii var. *pubicalyx* (Stearn) Emberger, L. & Maire – – wfo-0001303714
Limonium mouterdei Domina, Erben & Raimondo – – wfo-0000744155 – http://www.herbmedit.org/flora18.html
Limonium mucronatum (L.f.) Chaz. – – wfo-0001303715 – https://gallica.bnf.fr/ark:/12148/bpt6k15115421/f44.item
Limonium mucronatum (L.f.) Kuntze – – wfo-0000444539 – https://biodiversitylibrary.org/page/3817
Limonium mucronulatum (H.Lindb.) Greuter & Raus – – wfo-0000444524 – https://www.jstor.org/stable/3996917?seq=1#page_scan_tab_contents
Limonium multiceps (Pomel) Erben – – wfo-0001303716 – http://www.herbmedit.org/flora22.html
Limonium multiflorum Erben – – wfo-0000444546 – https://biodiversitylibrary.org/page/15235832
Limonium multiforme (Martelli) Pignatti – – wfo-0000444545
Limonium multiforme (Martelli) Pignatti – – wfo-0001303050 – http://onlinelibrary.wiley.com/doi/10.1111/j.1095-8339.1971.tb02152.x/full
Limonium multifurcatum Erben – – wfo-0000444544 – https://biodiversitylibrary.org/page/15042576
Limonium muradense Erben – – wfo-0000444541 – http://www.biodiversitylibrary.org/openurl?pid=title:14894&volume=30&issue=&spage=648&date=1991
Limonium mutabile (Ingr.) P.D.Sell – – wfo-0001303717
Limonium mutatum (Ingr.) P.D.Sell – – wfo-0001303718
Limonium myosuroides (Regel) Kuntze – – wfo-0000444540 – https://biodiversitylibrary.org/page/3817
Limonium myrianthum (Schrenk ex Fisch. & C.A.Mey.) Kuntze – – wfo-0001095236 – https://biodiversitylibrary.org/page/3817
Limonium namaquanum L.Bolus – – wfo-0001095194
Limonium naniforme P.D.Sell – – wfo-0001303719
Limonium narbonense Mill. – – wfo-0000444548 – https://biodiversitylibrary.org/page/395149
Limonium narynense Lincz. – – wfo-0000444537

Limonium nashii f. *albiflorum* House – – wfo-0001303720
Limonium nashii Small – – wfo-0001095161 – http://www.biodiversitylibrary.org/openurl?pid=title:340&volume=24&issue=11&spage=491&date=1897
Limonium nashii var. *angustatum* (A.Gray) H.E.Ahles – – wfo-0001095162
Limonium nashii var. *trichogonum* S.F.Blake – – wfo-0001303721
Limonium neapolense Brullo – – wfo-0000444536 – http://www.biodiversitylibrary.org/openurl?pid=title:14894&volume=28&issue=&spage=419&date=1989
Limonium neocastellonense Fern.Casas – – wfo-0000444535 – https://bibdigital.rjb.csic.es/viewer/12086/?offset=#page=39&viewer=picture&o=bookmark&n=0&q=
Limonium neoscoparium Klokov – – wfo-0000444534
Limonium nogalense (Chiov.) Verdc. & Hemming ex Cufod. – – wfo-0000444588 – http://www.jstor.org/stable/3667348, http://www.jstor.org/stable/3667449
Limonium normannicum Ingr. – – wfo-0000444570 – http://archive.bsbi.org.uk/watsonia_15.html
Limonium nudum (Boiss. & Buhse) Kuntze – – wfo-0000444630 – https://biodiversitylibrary.org/page/3817
Limonium nudum (Boiss. & Buhse) Kuntze – – wfo-0001303722
Limonium nydeggeri Erben – – wfo-0000444629 – https://biodiversitylibrary.org/page/27804243
Limonium nymphaeum Erben – – wfo-0000444628 – https://biodiversitylibrary.org/page/15206067
Limonium obesifolium P.D.Sell – – wfo-0001303723
Limonium oblanceolatum Brullo – – wfo-0000444627 – http://www.biodiversitylibrary.org/openurl?pid=title:14894&volume=28&issue=&spage=419&date=1989
Limonium oblongifolium (Kotov) Loscot & Trautv. – – wfo-0001303724
Limonium obovatum (Ledeb.) Kuntze – – wfo-0000444626 – https://biodiversitylibrary.org/page/3817
Limonium obtusifolium (Rouy) Erben – – wfo-0000444625 – https://biodiversitylibrary.org/page/15235784
Limonium obtusilobum S.F.Blake – – wfo-0001095212 – http://www.biodiversitylibrary.org/openurl?pid=title:721&volume=18&issue=207&spage=63&date=1916
Limonium occidentale (J.Lloyd) Kuntze – – wfo-0000444615 – https://biodiversitylibrary.org/page/3817
Limonium occidentale var. *procerum* C.E.Salmon – – wfo-0001303725 – https://doi.org/10.1111/j.1095-8339.1986.tb01428.x
Limonium ochranthum (Kar. & Kir.) Kuntze – – wfo-0000444631 – http://www.biodiversitylibrary.org/openurl?pid=title:327&volume=2&issue=&spage=396&date=1891
Limonium ocymifolium (Poir.) Kuntze – – wfo-0000444620 – https://biodiversitylibrary.org/page/3818
Limonium ocymifolium subsp. *bellidifolium* (Sm.) Meikle – – wfo-0001303726
Limonium ocymifolium var. *bellidifolium* (Sm.) Rech.f. – – wfo-0001303727 – https://www.zobodat.at/publikation_volumes.php?id=31166
Limonium oleifolium Mill. – – wfo-0000444619 – https://biodiversitylibrary.org/page/395149
Limonium oleifolium subsp. *oleifolium* Pignatti – – wfo-0001303728
Limonium oleifolium subsp. *algusae* Brullo – – wfo-0000444618 – http://journals.lub.lu.se/index.php/bn/article/view/11412/10558
Limonium oleifolium subsp. *dictyocladum* – – wfo-0001303729
Limonium oleifolium subsp. *opulentum* (Lojac.) Brullo – – wfo-0001303730 – http://journals.lub.lu.se/index.php/bn/article/view/11412/10558
Limonium oleifolium subsp. *pseudodictyocladum* (Pignatti) Pignatti – – wfo-0001303731 – http://onlinelibrary.wiley.com/doi/10.1111/j.1095-8339.1971.tb02152.x/full
Limonium oleifolium subsp. *sardoum* (Pignatti) Pignatti – – wfo-0000444640 – http://onlinelibrary.wiley.com/doi/10.1111/j.1095-8339.1971.tb02152.x/full
Limonium oleifolium var. *majus* (Guss.) Pignatti – – wfo-0001303732
Limonium oligotrichum Erben & Brullo – – wfo-0001303733 – http://dx.doi.org/10.11646/phytotaxa.240.1.1
Limonium omissae Bogdanović & Brullo – – wfo-0001303734 – http://dx.doi.org/10.11646/phytotaxa.215.1.1
Limonium optimae Raimondo – – wfo-0000444621 – http://www.herbmedit.org/flora03.html
Limonium opulentum (Lojac.) Brullo – – wfo-0000444622 – https://doi.org/10.1080/11263508009426433
Limonium opulentum (Lojac.) Greuter – – wfo-0000444648 – http://www.jstor.org/stable/3996512

Limonium orellii Erben – – wfo-0000444647 – http://www.biodiversitylibrary.org/openurl?pid=title:14894&volume=30&issue=&spage=462&date=1991
Limonium oristanum Alf.Mayer – – wfo-0000444646
Limonium ornatum (Ball) Kuntze – – wfo-0000444645 – https://biodiversitylibrary.org/page/3818
Limonium otolepis (Schrenk) Kuntze – – wfo-0001095237 – https://biodiversitylibrary.org/page/3818
Limonium oudayense Sauvage & Vindt – – wfo-0000444644
Limonium ovalifolium (Poir.) Kuntze – – wfo-0000444642 – https://biodiversitylibrary.org/page/3818
Limonium ovalifolium f. *biflorum* Pignatti – – wfo-0000745498
Limonium ovalifolium subsp. *biflorum* (Pignatti) Pignatti – – wfo-0001303735 – https://bibdigital.rjb.csic.es/viewer/11985/?offset=#page=324&viewer=picture&o=bookmark&n=0&q=
Limonium ovalifolium subsp. *canariense* Pignatti – – wfo-0001303736
Limonium ovalifolium subsp. *gallicum* Pignatti – – wfo-0001303737 – https://bibdigital.rjb.csic.es/viewer/11985/?offset=#page=324&viewer=picture&o=bookmark&n=0&q=
Limonium ovalifolium subsp. *lanceolatum* (Hoffmanns. & Link) Franco – – wfo-0001303738
Limonium ovalifolium subsp. *lusitanicum* Pignatti – – wfo-0001303739 – https://bibdigital.rjb.csic.es/viewer/11985/?offset=#page=326&viewer=picture&o=bookmark&n=0&q=
Limonium ovalifolium subsp. *ovalifolium* – – wfo-0001303740 – https://bibdigital.rjb.csic.es/viewer/11985/?offset=#page=324&viewer=picture&o=bookmark&n=0&q=
Limonium ovalifolium subsp. *pyramidatum* (Lowe) A.Hansen & Sunding – – wfo-0001303741
Limonium ovczinnikovii Lincz. & Czukav. – – wfo-0000444641
Limonium owerinii (Boiss.) Kuntze – – wfo-0000444649 – https://biodiversitylibrary.org/page/3818
Limonium pachynense Brullo – – wfo-0000444639 – http://journals.lub.lu.se/index.php/bn/article/view/11412/10558
Limonium pagasaeum Erben & Brullo – – wfo-0001303742 – http://dx.doi.org/10.11646/phytotaxa.240.1.1
Limonium palmare (Sm.) Rech.f. – – wfo-0000444638
Limonium palmyrense (Post) Dinsm. – – wfo-0000444637
Limonium pandatariae Pignatti – – wfo-0000444636 – https://doi.org/10.1080/00837792.1982.10670239
Limonium panormitanum (Tod.) Pignatti – – wfo-0000444635 – http://onlinelibrary.wiley.com/doi/10.1111/j.1095-8339.1971.tb02152.x/full
Limonium papillatum (Webb & Berthel.) Kuntze – – wfo-0000444634 – https://biodiversitylibrary.org/page/3818
Limonium papillatum var. *callibotryum* Svent. – – wfo-0001303743
Limonium papillatum var. *papillatum* – – wfo-0001303744
Limonium paradoxum Pugsley – – wfo-0000444613
Limonium paradoxum var. *mutabile* Ingr. – – wfo-0001303745 – https://doi.org/10.1111/j.1095-8339.1986.tb01428.x
Limonium paralimniticum Christodoulou, Erben, Hand & Kefalas – – wfo-1000047129 – https://doi.org/10.7320/FlMedit32.035
Limonium paramedium (Ingr.) P.D.Sell – – wfo-0001303746
Limonium parosicum Erben & Brullo – – wfo-0001303747 – http://dx.doi.org/10.11646/phytotaxa.240.1.1
Limonium parvibracteatum Pignatti – – wfo-0000444617 – http://onlinelibrary.wiley.com/doi/10.1111/j.1095-8339.1971.tb02152.x/full
Limonium parvifolium (Tineo) Pignatti – – wfo-0000444598 – http://onlinelibrary.wiley.com/doi/10.1111/j.1095-8339.1971.tb02152.x/full
Limonium parvum Ingr. – – wfo-0000444596 – http://onlinelibrary.wiley.com/doi/10.1111/j.1095-8339.1986.tb01428.x/full
Limonium patagonicum Macloskie – – wfo-0000444595
Limonium patrimoniense Arrigoni & Diana – – wfo-0000444594
Limonium paui Cámara & Sennen – – wfo-0001303748 – https://bibdigital.rjb.csic.es/viewer/9472/?offset=#page=270&viewer=picture&o=bookmark&n=0&q=
Limonium paulayanum (Vierh.) Ghaz. & J.R.Edm. – – wfo-0000444593 – https://doi.org/10.1017/S0960428603000027
Limonium pavonianum Brullo – – wfo-0000444592 – http://www.jstor.org/stable/3996200
Limonium pectinatum (Aiton) Kuntze – – wfo-0000444591

Limonium pectinatum var. *corculum* (Webb & Berthel.) G.Kunkel & Sunding – – wfo-0001303749 – http://bibdigital.rjb.csic.es/ing/FichaLibro.php?Libro=7205
Limonium pectinatum var. *divaricatum* (Pit.) G.Kunkel & Sunding – – wfo-0001303750 – http://mdc.ulpgc.es/cdm/singleitem/collection/cbotanica/id/13/rec/3
Limonium pectinatum var. *incomptum* (Webb & Berthel.) G.Kunkel & Sunding – – wfo-0001303751
Limonium pectinatum var. *pectinatum* – – wfo-0001303752
Limonium pectinatum var. *solandri* (Webb & Berthel.) Kuntze – – wfo-0001303753 – https://biodiversitylibrary.org/page/3816
Limonium pedicellatum (Wallr. ex Boiss.) Kuntze – – wfo-0000444583 – https://biodiversitylibrary.org/page/3818
Limonium pelagosae Bogdanović & Brullo – – wfo-0001303754 – http://dx.doi.org/10.11646/phytotaxa.215.1.1
Limonium penicillatum Adamson – – wfo-0000444589 – https://archive.org/details/journalofsouthaf07unse/page/202/mode/2up
Limonium peregrinum (P.J.Bergius) R.A.Dyer – – wfo-0001095195 – https://doi.org/10.4102/abc.v7i3.1673
Limonium perezii (Stapf) F.T.Hubb. ex L.H.Bailey – – wfo-0001095316 – http://www.biodiversitylibrary.org/openurl?pid=title:721&volume=18&issue=211&spage=158&date=1916
Limonium perfoliatum (C.A.Mey. ex Boiss.) Kuntze – – wfo-0001095163 – https://biodiversitylibrary.org/page/3818
Limonium pericotii (O.Bolòs & Vigo) Greuter & Raus – – wfo-0000444586 – https://www.jstor.org/stable/3996917?seq=1#page_scan_tab_contents
Limonium perplexum L.Sáez & Rosselló – – wfo-0000444585 – http://www.rjb.csic.es/jardinbotanico/jardin/contenido.php?Pag=219&tipo=volumenanales&vol=57(1)
Limonium peruvianum Kuntze – – wfo-0001095264 – https://www.biodiversitylibrary.org/page/3817
Limonium pescadense Greuter & Raus – – wfo-0000444584 – http://www.jstor.org/stable/3996512
Limonium peucetium Pignatti – – wfo-0000444607 – https://doi.org/10.1080/00837792.1982.10670239
Limonium pharense (Ingr.) P.D.Sell – – wfo-0001303755
Limonium pharosianum Bogdanović & Brullo – – wfo-0001303756 – http://dx.doi.org/10.11646/phytotaxa.215.1.1
Limonium phitosianum R.Artelari – – wfo-0000444608 – https://biodiversitylibrary.org/page/27801773
Limonium pigadiense (Rech.f.) Rech.f. – – wfo-0000444590 – https://www.zobodat.at/publikation_volumes.php?id=31166
Limonium pignattii Brullo & Di Martino – – wfo-0001303757
Limonium pignattii Brullo & Di Martino ex Brullo – – wfo-0000444612 – http://journals.lub.lu.se/index.php/bn/article/view/11412/10558
Limonium pinillense Roselló, Stübing, Peris J.B. & Cirujano – – wfo-0000444611 – http://rjb.revistas.csic.es/index.php/rjb/article/view/287/282
Limonium piptopodum Nevski – – wfo-0000444610
Limonium planesiae Pignatti – – wfo-0000444609 – https://doi.org/10.1080/00837792.1982.10670239
Limonium platyphyllum Lincz. – – wfo-0000444599
Limonium plumosum (Phil.) Kuntze – – wfo-0001095207 – https://biodiversitylibrary.org/page/3817
Limonium plurisquamatum Erben – – wfo-0000444614 – https://biodiversitylibrary.org/page/15235815
Limonium plutosianum Artell – – wfo-0001095253
Limonium poimenum Ilardi, Brullo, D.Cusimano & G.Giusso – – wfo-0001443424 – http://dx.doi.org/10.11646/phytotaxa.188.5.4
Limonium pomelianum (Rouy) Erben – – wfo-0001303758 – http://www.herbmedit.org/flora22.html
Limonium pomoense Bogdanović & Brullo – – wfo-0001303759 – http://dx.doi.org/10.11646/phytotaxa.215.1.1
Limonium pontium Pignatti – – wfo-0000444605 – http://onlinelibrary.wiley.com/doi/10.1111/j.1095-8339.1971.tb02152.x/full
Limonium pontium subsp. *pandatariae* (Pignatti) Iamonico, Iberite, De Castro & Nicolella – – wfo-1200069340 – https://doi.org/10.3390/plants11223163
Limonium pontium subsp. *pontium* – – wfo-0001303760 – https://doi.org/10.3390/plants11223163
Limonium pontium subsp. *terracinense* Iberite, Iamonico, De Castro, Nicolella Iamonico, De Castro & Nicolella – – wfo-1200069339
Limonium ponzoi (Fiori & Bég.) Brullo – – wfo-0000444604 – http://journals.lub.lu.se/index.php/bn/article/view/11412/10558

Limonium popovii Kubansk. – – wfo-0000444603
Limonium portlandicum (Ingr.) P.D.Sell – – wfo-0001303761
Limonium portopetranum Erben – – wfo-0000444602 – http://www.biodiversitylibrary.org/openurl?pid=title:14894&volume=30&issue=&spage=471&date=1991
Limonium portovecchiense Erben – – wfo-0000444601 – https://biodiversitylibrary.org/page/15042568
Limonium postii Domina, Erben & Raimondo – – wfo-0000744156 – http://www.herbmedit.org/flora18.html
Limonium potaninii Ikonn.-Gal. – – wfo-0001095341
Limonium preauxii (Webb & Berthel.) Kuntze – – wfo-0000444600 – https://biodiversitylibrary.org/page/3818
Limonium procerum (C.E.Salmon) Ingr. – – wfo-0000444287 – http://onlinelibrary.wiley.com/doi/10.1111/j.1095-8339.1986.tb01428.x/full
Limonium procerum subsp. *cambrense* Ingr. – – wfo-0001303762 – https://doi.org/10.1111/j.1095-8339.1986.tb01428.x
Limonium procerum subsp. *devoniense* Ingr. – – wfo-0001303763 – https://doi.org/10.1111/j.1095-8339.1986.tb01428.x
Limonium procerum var. *cornubiense* Ingr. – – wfo-0001303764 – https://doi.org/10.1111/j.1095-8339.1986.tb01428.x
Limonium procerum var. *hibernicum* Ingr. – – wfo-0001303765 – https://doi.org/10.1111/j.1095-8339.1986.tb01428.x
Limonium procerum var. *medium* Ingr. – – wfo-0001303766 – https://doi.org/10.1111/j.1095-8339.1986.tb01428.x
Limonium procerum var. *paramedium* Ingr. – – wfo-0001303767 – https://doi.org/10.1111/j.1095-8339.1986.tb01428.x
Limonium procerum var. *wessexense* Ingr. – – wfo-0001303768 – https://doi.org/10.1111/j.1095-8339.1986.tb01428.x
Limonium proliferum (d'Urv.) Erben & Brullo – – wfo-0001303769 – http://dx.doi.org/10.11646/phytotaxa.240.1
Limonium protohermaeum Arrigoni & Diana – – wfo-0000444052
Limonium pruinosum (L.) Chaz. – – wfo-0001303770 – https://gallica.bnf.fr/ark:/12148/bpt6k15115421/f44.image
Limonium pruinosum (L.) Kuntze – – wfo-0000444060 – https://biodiversitylibrary.org/page/3818
Limonium pruinosum subsp. *alleizettei* (Pau) Maire – – wfo-0001303771 – http://bibdigital.rjb.csic.es/ing/Libro.php?Libro=1866&Pagina=320
Limonium pruinosum var. *glabrum* Maire & Weiller – – wfo-0001303772
Limonium pruinosum var. *hirtiflorum* (Cavara) Täckh. ex Feinbrun – – wfo-0001303773
Limonium pseudarticulatum Erben – – wfo-0000444064 – http://www.biodiversitylibrary.org/openurl?pid=title:14894&volume=28&issue=&spage=313&date=1989
Limonium pseudebusitanum Erben – – wfo-0000444058 – http://www.biodiversitylibrary.org/openurl?pid=title:14894&volume=28&issue=&spage=313&date=1989
Limonium pseudodictyocladum (Pignatti) L.Llorens – – wfo-0000444055
Limonium pseudodictyocladum Greuter & Raus – – wfo-0001303774 – http://www.jstor.org/stable/3996512
Limonium pseudolaetum Arrigoni & Diana – – wfo-0000444053
Limonium pseudominutum Erben – – wfo-0000444071 – https://biodiversitylibrary.org/page/15148016
Limonium pseudoparadoxum (Ingr.) P.D.Sell – – wfo-0001303775
Limonium pseudotranswallianum (Ingr.) P.D.Sell – – wfo-0001303776
Limonium psilocladum (Boiss.) Kuntze – – wfo-0001095254 – https://biodiversitylibrary.org/page/3818
Limonium puberulum (Webb ex Lindl.) H.Arnaud – – wfo-0001303777
Limonium puberulum (Webb ex Lindl.) Kuntze – – wfo-0000444038 – https://biodiversitylibrary.org/page/3817
Limonium pubescens (DC.) P.Fourn. – – wfo-0001303778
Limonium pujosii Sauvage & Vindt – – wfo-0000444037
Limonium pulviniforme Arrigoni & Diana – – wfo-0000444036
Limonium punicum Brullo – – wfo-0000444034 – http://www.biodiversitylibrary.org/openurl?pid=title:14894&volume=28&issue=&spage=419&date=1989
Limonium purpuratum (L.) Chaz. – – wfo-0001303779 – https://gallica.bnf.fr/ark:/12148/bpt6k15115421/f43.item
Limonium purpuratum (L.) F.T.Hubb. – – wfo-0001095197 – https://biodiversitylibrary.org/page/568409
Limonium purpuratum var. *longifolium* (Thunb.) F.T.Hubb. – – wfo-0001303780 – https://biodiversitylibrary.org/page/568409
Limonium pusillum Erben & Brullo – – wfo-0001303781 – http://dx.doi.org/10.11646/phytotaxa.240.1.1

Limonium pycnanthum (K.Koch) Kuntze – – wfo-0001095363 – http://www.biodiversitylibrary.org/openurl?pid=title:327&volume=2&issue=&spage=396&date=1891
Limonium pylium R.Artelari – – wfo-0000444033
Limonium pyramidatum Brullo – – wfo-0000444031 – https://biodiversitylibrary.org/page/27802305
Limonium quesadense Erben – – wfo-0000444039 – https://biodiversitylibrary.org/page/15148009
Limonium quinnii M.B.Crespo & Pena-Martín – – wfo-0001303782 – http://dx.doi.org/10.11646/phytotaxa.94.2.1
Limonium racemosum (Lojac.) Diana – – wfo-0000444030
Limonium raddianum (Boiss.) Pignatti ex Brullo – – wfo-0000444029 – https://doi.org/10.1080/00837792.1978.10670114
Limonium ramosissimum (Poir.) Maire – – wfo-0000444028
Limonium ramosissimum subsp. *confusum* (Gren. & Godr.) Pignatti – – wfo-0000444026 – http://onlinelibrary.wiley.com/doi/10.1111/j.1095-8339.1971.tb02152.x/full
Limonium ramosissimum subsp. *doerfleri* (Halácsy) Pignatti – – wfo-0000444025 – http://onlinelibrary.wiley.com/doi/10.1111/j.1095-8339.1971.tb02152.x/full
Limonium ramosissimum subsp. *provinciale* (Pignatti) Pignatti – – wfo-0000444024 – http://onlinelibrary.wiley.com/doi/10.1111/j.1095-8339.1971.tb02152.x/full
Limonium ramosissimum subsp. *ramosissimum* – – wfo-0001303783
Limonium ramosissimum subsp. *siculum* Pignatti – – wfo-0001303784 – http://onlinelibrary.wiley.com/doi/10.1111/j.1095-8339.1971.tb02152.x/full
Limonium ramosissimum subsp. *tommasinii* (Pignatti) Pignatti – – wfo-0000444047
Limonium rariflorum (Drejer) Kuntze – – wfo-0000444032 – https://biodiversitylibrary.org/page/3817
Limonium recticaule Erben & Brullo – – wfo-0001303785 – http://dx.doi.org/10.11646/phytotaxa.240.1.1
Limonium recurviforme (Ingr.) P.D.Sell – – wfo-0001303786
Limonium recurvum C.E.Salmon – – wfo-0000444050 – https://www.biodiversitylibrary.org/page/35445941
Limonium recurvum subsp. *crigyllensis* I.Rees – – wfo-1200040728 – https://britishandirishbotany.org/index.php/bib/article/view/102/147
Limonium recurvum subsp. *humile* (Girard) Ingr. – – wfo-0001303787 – https://doi.org/10.1111/j.1095-8339.1986.tb01428.x
Limonium recurvum subsp. *portlandicum* Ingr. – – wfo-0001303788 – https://doi.org/10.1111/j.1095-8339.1986.tb01428.x
Limonium recurvum subsp. *pseudotranswallianum* Ingr. – – wfo-0001303789 – https://doi.org/10.1111/j.1095-8339.1986.tb01428.x
Limonium recurvum subsp. *recurvum* – – wfo-0001303790
Limonium recurvum var. *donegalense* Ingr. – – wfo-0001303791 – https://doi.org/10.1111/j.1095-8339.1986.tb01428.x
Limonium recurvum var. *humile* (Girard) Ingr. – – wfo-0001303792 – https://doi.org/10.1111/j.1095-8339.1986.tb01428.x
Limonium recurvum var. *kerryense* Ingr. – – wfo-0001303793 – https://doi.org/10.1111/j.1095-8339.1986.tb01428.x
Limonium recurvum var. *portlandicum* Ingr. – – wfo-0001303794 – https://doi.org/10.1111/j.1095-8339.1986.tb01428.x
Limonium recurvum var. *pseudoparadoxum* Ingr. – – wfo-0001303795 – https://doi.org/10.1111/j.1095-8339.1986.tb01428.x
Limonium recurvum var. *recurviforme* Ingr. – – wfo-0001303796 – https://doi.org/10.1111/j.1095-8339.1986.tb01428.x
Limonium redivivum (Svent.) G.Kunkel & Sunding – – wfo-0000444046 – http://mdc.ulpgc.es/cdm/singleitem/collection/cbotanica/id/13/rec/3
Limonium redivivum var. *pilosum* (Svent.) G.Kunkel & Sunding – – wfo-0001303797
Limonium redivivum var. *redivivum* – – wfo-0001303798
Limonium relicticum R.Mesa & A.Santos – – wfo-0000444044 – https://dialnet.unirioja.es/servlet/articulo?codigo=2279895
Limonium remotispiculum (Lacaita) Pignatti – – wfo-0000444043 – http://onlinelibrary.wiley.com/doi/10.1111/j.1095-8339.1971.tb02152.x/full
Limonium reniforme (Girard) Lincz. – – wfo-0000444042 – https://biodiversitylibrary.org/page/30218492
Limonium reticulatum (L.) Mill. – – wfo-0000444041 – https://biodiversitylibrary.org/page/395149
Limonium retirameum Greuter & Raus – – wfo-0000444088 – http://www.jstor.org/stable/3996512
Limonium retirameum subsp. *caralitanum* (Erben) Arrigoni – – wfo-0001303799

Limonium retirameum subsp. *retirameum* – – wfo-0001303800
Limonium retusum L.Llorens – – wfo-0001095267
Limonium revolutum Erben – – wfo-0001260276 – https://biodiversitylibrary.org/page/15235786
Limonium rezniczenkoanum Lincz. – – wfo-0001095238 – https://biodiversitylibrary.org/page/30218477
Limonium rhodense M.B.Crespo & Pena-Martín – – wfo-0001303801 – http://dx.doi.org/10.11646/phytotaxa.94.2.1
Limonium rigidum Alf.Mayer – – wfo-0000444132
Limonium rigualii M.B.Crespo & Erben – – wfo-0000444131 – http://www.biodiversitylibrary.org/openurl?pid=title:14894&volume=30&issue=&spage=459&date=1991
Limonium roborowskii Ikonn.-Gal. – – wfo-0001095335
Limonium romanum (Täckh. & Boulos) Domina – – wfo-0000749441 – http://www.bioone.org/doi/abs/10.3372/wi.41.41117
Limonium roridum (Sibth. & Sm.) Brullo & Guarino – – wfo-0000444129 – http://www.herbmedit.org/flora10.html
Limonium roseum (Sm.) Kuntze – – wfo-0000444128 – https://biodiversitylibrary.org/page/3818
Limonium rosselloi Ferrer-Gallego, P. P., R.Roselló & E.Laguna – – wfo-0001303802 – http://collectaneabotanica.revistas.csic.es/index.php/collectaneabotanica/article/view/208/211
Limonium rubescens Brullo – – wfo-0000444116 – http://www.biodiversitylibrary.org/openurl?pid=title:14894&volume=28&issue=&spage=419&date=1989
Limonium ruizii (Font Quer) Fern.Casas – – wfo-0000444126
Limonium rumicifolium (Svent.) G.Kunkel & Sunding – – wfo-0000444133 – http://mdc.ulpgc.es/cdm/singleitem/collection/cbotanica/id/13/rec/3
Limonium runemarkii Rech.f. – – wfo-0000444124
Limonium rungsii Sauvage & Vindt – – wfo-0000444123
Limonium rupicola (Badarò ex Rchb.) Kuntze – – wfo-0000444122 – https://biodiversitylibrary.org/page/3818
Limonium rytidophyllum (Hook.) H.Arnaud – – wfo-1000055772 – https://www.biodiversitylibrary.org/page/45269408
Limonium sabulicola P.D.Sell – – wfo-0001303803
Limonium salicorniacea (F.Muell.) Kuntze – – wfo-0000444121 – https://biodiversitylibrary.org/page/3818
Limonium salmonis (Sennen & Elías) Pignatti – – wfo-0000444120
Limonium salsuginosum (Boiss.) Kuntze – – wfo-0000444119 – https://biodiversitylibrary.org/page/3818
Limonium samium Erben & Brullo – – wfo-0001303804 – http://dx.doi.org/10.11646/phytotaxa.240.1.1
Limonium sanctamargaritense P.D.Sell – – wfo-0001303805
Limonium sanjurjoi Sennen & Mauricio – – wfo-0000444140 – http://bibdigital.rjb.csic.es/ing/Libro.php?Libro=42&Pagina=115
Limonium santapolense Erben – – wfo-0000444125 – https://biodiversitylibrary.org/page/15148011
Limonium saracinatum R.Artelari – – wfo-0000444127
Limonium sarcophyllum Ghaz. & J.R.Edm. – – wfo-0000444148 – https://doi.org/10.1017/S0960428603000027
Limonium sardoum (Pignatti) Erben – – wfo-0000444147 – https://biodiversitylibrary.org/page/15043350
Limonium sareptanum (A.K.Becker) Gams – – wfo-0000748099 – https://bibdigital.rjb.csic.es/viewer/16557/?offset=#page=346
Limonium sarniense (Ingr.) P.D.Sell – – wfo-0001303806
Limonium sartorianum Erben & Brullo – – wfo-0001303807 – http://dx.doi.org/10.11646/phytotaxa.240.1.1
Limonium sartorii (Nyman) Kuntze – – wfo-0000444146 – https://biodiversitylibrary.org/page/3818
Limonium savianum Pignatti – – wfo-0000444145
Limonium saxicola Erben – – wfo-0000444143 – https://biodiversitylibrary.org/page/27802251
Limonium saxonicum (Ingr.) P.D.Sell – – wfo-0001303808
Limonium scabrum (Thunb.) Kuntze – – wfo-0001095199 – https://biodiversitylibrary.org/page/3818
Limonium scabrum var. *scabrum* (Thunb.) Kuntze – – wfo-0001095202
Limonium scabrum var. *avenaceum* (C.H.Wright) R.A.Dyer – – wfo-0001095200 – https://doi.org/10.4102/abc.v7i3.1673
Limonium scabrum var. *corymbulosum* (Boiss.) R.A.Dyer – – wfo-0001442003 – https://doi.org/10.4102/abc.v7i3.1673

Limonium schinousae Erben & Brullo – – wfo-0001303809 – http://dx.doi.org/10.11646/phytotaxa.240.1.1
Limonium schrenkianum (Fisch. & C.A.Mey.) Kuntze – – wfo-0000444134 – https://biodiversitylibrary.org/page/3818
Limonium scoparium (M.Bieb.) Stankov – – wfo-0000444141 – https://books.google.com.sv/books?redir_esc=y&id=N6poHAAACAAJ&focus=searchwithinvolume&q=scoparium
Limonium scoparium (Pall. ex Willd.) H.Arnaud – – wfo-1200068002
Limonium scoparium Klokov – – wfo-0001303810
Limonium scoparium var. *meyeri* (Boiss.) Tzvelev – – wfo-0001303811
Limonium scopulorum M.B.Crespo & Lledó – – wfo-0000744157 – http://www.jstor.org/stable/23064502
Limonium scorpioides Erben – – wfo-0000444149 – http://www.biodiversitylibrary.org/openurl?pid=title:14894&volume=28&issue=&spage=313&date=1989
Limonium sebkarum (Pomel) Maire – – wfo-0000444139
Limonium sect. *Circinaria* (Boiss.) M.Malekm. – – wfo-0001303812 – https://doi.org/10.12705/665.8
Limonium sect. *Ctenostachys* (Boiss.) Sauvage & Vindt – – wfo-0001303813
Limonium sect. *Iranolimon* M.Malekm., Akhani & Borsch – – wfo-0001303814 – https://doi.org/10.12705/665.8
Limonium sect. *Jovibarba* (Boiss.) M.Malekm. & Koutr. – – wfo-0001303815
Limonium sect. *Limoniodendron* Svent. – – wfo-0001303816
Limonium sect. *Limonium* – – wfo-0001303817
Limonium sect. *Myriolepis* (Boiss.) Sauvage & Vindt – – wfo-0001303818
Limonium sect. *Nephrophyllum* Rech.f. – – wfo-0001303819
Limonium sect. *Odontolepideae* (Boiss.) Koutr. – – wfo-1000045204
Limonium sect. *Plathymenium* (Boiss.) Lincz. – – wfo-0001303820
Limonium sect. *Polyarthrion* (Boiss.) Sauvage & Vindt – – wfo-0001303821
Limonium sect. *Pruinosa* (Batt.) Koutr. – – wfo-0001303822 – https://doi.org/10.1002/ece3.4553
Limonium sect. *Pruinosum* (Batt.) Koutr. – – wfo-1200028578
Limonium sect. *Pterocladus* (Spach) Bokhari – – wfo-0001303823
Limonium sect. *Sarcophylla* (Boiss.) Lincz. – – wfo-0001303824
Limonium sect. *Sarcophyllum* (Boiss.) Lincz. – – wfo-0001303825
Limonium sect. *Schizhymenium* (Boiss.) Sauvage & Vindt – – wfo-0001303826
Limonium sect. *Siphonantha* (Boiss.) Sauvage & Vindt – – wfo-0001303827
Limonium sect. *Siphonocalyx* Lincz. – – wfo-1000049111
Limonium sect. *Sphaerostachys* (Boiss.) Bokhari – – wfo-0001303828
Limonium sect. *Tenuiramosa* Koutr. – – wfo-0001303829 – https://doi.org/10.1002/ece3.4553
Limonium sect. *Tenuiramosum* Koutr. – – wfo-1000045200
Limonium secundirameum (Lojac.) Brullo – – wfo-0001303830
Limonium secundirameum (Lojac.) Greuter & Raus – – wfo-0000444138 – http://www.jstor.org/stable/3996512
Limonium secundirameum (Lojac.) Pignatti – – wfo-0001303831
Limonium sedodes (Regel) Kuntze – – wfo-0001095349 – http://www.biodiversitylibrary.org/openurl?pid=title:327&volume=2&issue=&spage=396&date=1891
Limonium selinuntinum Brullo – – wfo-0000444137 – http://journals.lub.lu.se/index.php/bn/article/view/11412/10558
Limonium semenowii (Herder) Kuntze – – wfo-0001095239 – http://www.biodiversitylibrary.org/openurl?pid=title:327&volume=2&issue=&spage=396&date=1891
Limonium semenowii var. *chrysocephalum* (Regel) Grubov – – wfo-0001095284 – http://www.biodiversitylibrary.org/openurl?pid=title:744&volume=4&issue=1&spage=31&date=1994
Limonium semenowii var. *sedoides* (Regel) Grubov – – wfo-0001095285 – http://www.biodiversitylibrary.org/openurl?pid=title:744&volume=4&issue=1&spage=31&date=1994
Limonium senkakuense T.Yamaz. – – wfo-0000444142 – http://www.jjbotany.com/
Limonium serbicum (Nyman) Kuntze – – wfo-0000444097 – https://biodiversitylibrary.org/page/3818

Limonium sercquense (Ingr.) P.D.Sell – – wfo-0001303832
Limonium serotinum (Rchb.) Erben – – wfo-0001303833 – https://biodiversitylibrary.org/page/15235746
Limonium serotinum (Rchb.) Pignatti – – wfo-0000444096 – https://doi.org/10.1080/11263507309426323
Limonium serpentinicum R.Pino, Silva Pando & J.J.Pino – – wfo-0001303834 – https://doi.org/10.3417/2015026
Limonium serratum Brullo – – wfo-0000444095 – http://www.biodiversitylibrary.org/openurl?pid=title:14894&volume=28&issue=&spage=419&date=1989
Limonium sewerzowii (Herder) Kuntze – – wfo-0000444094 – https://biodiversitylibrary.org/page/3818
Limonium sibthorpianum (Guss.) Kuntze – – wfo-0000444093 – https://biodiversitylibrary.org/page/3818
Limonium sibthorpianum subsp. *vaccarii* Pignatii ex Brullo – – wfo-0001303835 – https://doi.org/10.1080/00837792.1978.10670114
Limonium siculum Mill. – – wfo-0000444092 – https://biodiversitylibrary.org/page/395149
Limonium sieberi (Boiss.) Kuntze – – wfo-0000444082
Limonium silvestrei Aparicio – – wfo-0000444089 – http://www.jstor.org/stable/23726732
Limonium sinense (Girard) Kuntze – – wfo-0001095301 – http://www.biodiversitylibrary.org/openurl?pid=title:327&volume=2&issue=&spage=396&date=1891
Limonium sinense var. *spinulosum* Y.Huang – – wfo-0001303836
Limonium sinisicum Erben – – wfo-0000444087 – http://www.biodiversitylibrary.org/openurl?pid=title:14894&volume=22&issue=&spage=210&date=1986
Limonium sinuatum (L.) Mill. – – wfo-0000444086 – https://biodiversitylibrary.org/page/395149
Limonium sinuatum subsp. *sinuatum* (L.) Mill. – – wfo-0001442004
Limonium sinuatum var. *sinuatum* (L.) Mill. – – wfo-0001303837
Limonium sinuatum f. *leucocalyx* (Maire) Sauvage & Vindt – – wfo-0001303838
Limonium sinuatum f. *pallidum* Maire – – wfo-0001303839 – https://bibdigital.rjb.csic.es/viewer/13179/?offset=#page=203&viewer=picture&o=bookmark&n=0&q=
Limonium sinuatum subsp. *beaumierianum* (Coss. ex Maire) Sauvage & Vindt – – wfo-0001303840
Limonium sinuatum subsp. *bonduellei* (T.Lestib.) Sauvage & Vindt – – wfo-0001303841
Limonium sinuatum subsp. *romanum* Täckh. & Boulos – – wfo-0000444085
Limonium sinuatum subvar. *annuum* (Maire) Sauvage & Vindt – – wfo-0001303842
Limonium sinuatum subvar. *glabrescens* (Maire) Sauvage & Vindt – – wfo-0001303843
Limonium sinuatum var. *akkense* (Coss. ex Batt.) Sauvage & Vindt – – wfo-0001303844
Limonium sinuatum var. *annuum* Maire – – wfo-0001303845 – https://bibdigital.rjb.csic.es/viewer/13176/?offset=#page=19&viewer=picture&o=bookmark&n=0&q=statice
Limonium sinuatum var. *candidissimum* (hort.) F.T.Hubb. – – wfo-0001303846 – https://biodiversitylibrary.org/page/568410
Limonium sinuatum var. *subglabrum* (H.Lindb.) Maire – – wfo-0001303847
Limonium sinuatum var. *tripeaui* (Maire) Sauvage & Vindt – – wfo-0001303848
Limonium sinuatum var. *typicum* Maire – – wfo-0001303849 – https://bibdigital.rjb.csic.es/viewer/13176/?offset=#page=19&viewer=picture&o=search&n=0&q=statice
Limonium sirinicum Erben & Brullo – – wfo-0001303850 – http://dx.doi.org/10.11646/phytotaxa.240.1.1
Limonium sitiacum Rech.f. – – wfo-0000444084
Limonium smithii Akaydın – – wfo-0001303851 – http://idosi.org/wasj/wasj2(4)2007.htm
Limonium soboliferum Erben – – wfo-0000444083 – http://www.biodiversitylibrary.org/openurl?pid=title:14894&volume=28&issue=&spage=313&date=1989
Limonium sogdianum (Popov) Ikonn.-Gal. – – wfo-0001095240
Limonium sokotranum (Vierh.) Radcl.-Sm. – – wfo-0000444107 – http://www.jstor.org/stable/4103205
Limonium solanderi Lincz. – – wfo-0000444091
Limonium solandri (Webb & Berthel.) G.Kunkel – – wfo-0000444114 – http://mdc.ulpgc.es/cdm/singleitem/collection/cbotanica/id/170/rec/4
Limonium somalorum (Vierh.) Hutch. & E.A.Bruce – – wfo-0000444113

Limonium sommierianum (Fiori) Arrigoni – – wfo-0000444112
Limonium sougiae Erben & Brullo – – wfo-0001303852 – http://dx.doi.org/10.11646/phytotaxa.240.1.1
Limonium spathulatum (Desf.) Kuntze – – wfo-0000444111 – https://biodiversitylibrary.org/page/3818
Limonium spathulatum subsp. *rusicadense* (Maire) Quézel & Santa – – wfo-0001303853
Limonium spathulatum var. *emarginatum* (Willd.) C.E.Hubb. & Sandwith – – wfo-0001303854
Limonium speciosum (L.) Chaz. – – wfo-0001303855 – https://gallica.bnf.fr/ark:/12148/bpt6k15115421/f42.item
Limonium speciosum (L.) Kuntze – – wfo-0000444110 – http://www.biodiversitylibrary.org/openurl?pid=title:327&volume=2&issue=&spage=396&date=1891
Limonium spectabile (Svent.) G.Kunkel & Sunding – – wfo-0000444109 – http://mdc.ulpgc.es/cdm/singleitem/collection/cbotanica/id/13/rec/3
Limonium spicatum (Willd.) Kuntze – – wfo-0000444098 – https://biodiversitylibrary.org/page/3818
Limonium spreitzenhoferi Erben & Brullo – – wfo-0001303856 – http://dx.doi.org/10.11646/phytotaxa.240.1.1
Limonium squarrosum Erben – – wfo-0000444106 – http://www.biodiversitylibrary.org/openurl?pid=title:14894&volume=28&issue=&spage=313&date=1989
Limonium stenophyllum Erben – – wfo-0000444115 – http://www.biodiversitylibrary.org/openurl?pid=title:14894&volume=28&issue=&spage=313&date=1989
Limonium stenotatum (Rech.f.) Erben & Brullo – – wfo-0001303857 – http://dx.doi.org/10.11646/phytotaxa.240.1.1
Limonium stephanii Sennen – – wfo-0000444105 – http://bibdigital.rjb.csic.es/ing/Libro.php?Libro=15&Pagina=263
Limonium steppicum Sefi, Ghrabi-Gammar & Brullo – – wfo-0001303858 – https://doi.org/10.11646/phytotaxa.446.5.2
Limonium stocksii (Boiss.) Kuntze – – wfo-0000444104 – https://biodiversitylibrary.org/page/3818
Limonium strictissimum (Salzm.) Arrigoni – – wfo-0000444103
Limonium subanfractum Trinajstić – – wfo-0001303859
Limonium subg. Limonium – – wfo-0001303860
Limonium subg. Myriolepis (Boiss.) Pignatti – – wfo-0001303861 – http://onlinelibrary.wiley.com/doi/10.1111/j.1095-8339.1971.tb02152.x/full
Limonium subg. Pterocladus (Spach) H. Arnaud – – wfo-0001303862
Limonium subg. Pterocladus (Spach) Pignatti – – wfo-0001303863 – http://onlinelibrary.wiley.com/doi/10.1111/j.1095-8339.1971.tb02152.x/full
Limonium subglabrum Erben – – wfo-0000444102 – https://biodiversitylibrary.org/page/15235857
Limonium subnudum Bogdanović & Brullo – – wfo-0001303864 – http://dx.doi.org/10.11646/phytotaxa.215.1.1
Limonium subrotundifolium (Bég. & A.Vacc.) Brullo – – wfo-0000444101 – https://doi.org/10.1080/00837792.1978.10670114
Limonium subsect. *Nobiles* (Boiss.) Koutr. – – wfo-1000045202
Limonium subsect. *Nobilia* (Boiss.) Koutr. – – wfo-0001303865 – https://doi.org/10.1002/ece3.4553
Limonium subsect. *Odontolepidea* (Boiss.) Koutr. – – wfo-0001303866 – https://doi.org/10.1002/ece3.4553
Limonium subsect. *Odontolepidea* (Boiss.) Sauvage & Vindt – – wfo-0001303867
Limonium subsect. *Pruinosa* (Batt.) Sauvage & Vindt – – wfo-0001303868
Limonium subviolaceum Q.Z.Han & S.D.Zhao – – wfo-0001095311
Limonium sucronicum Erben – – wfo-0000444100 – http://www.biodiversitylibrary.org/openurl?pid=title:14894&volume=28&issue=&spage=313&date=1989
Limonium suffruticosum (L.) Kuntze – – wfo-0000444011 – http://www.biodiversitylibrary.org/openurl?pid=title:327&volume=2&issue=&spage=396&date=1891
Limonium sulcitanum Arrigoni – – wfo-0000443935
Limonium sundingii Leyens, Lobin, N.Kilian & Erben – – wfo-0001260278 – http://www.jstor.org/stable/3996981
Limonium superbum (Regel) F.T.Hubb. ex L.H.Bailey – – wfo-0000444079 – https://biodiversitylibrary.org/page/568410
Limonium superbum var. *flore-albo* (Benary) L.H.Bailey – – wfo-0001303869 – https://biodiversitylibrary.org/page/568410
Limonium supinum (Girard) Pignatti – – wfo-0000444078 – http://bibdigital.rjb.csic.es/ing/Libro.php?Libro=2837&Pagina=317
Limonium supinum Erben – – wfo-0001303870

Limonium supinum var. *diegoi* (Sennen) Pignatti – – wfo-0001303871
Limonium supinum var. *supinum* – – wfo-0001303872
Limonium suwarowii (Regel) Kuntze – – wfo-0001303873
Limonium suworowii (Regel) Kuntze – – wfo-0000444077 – https://biodiversitylibrary.org/page/3817
Limonium suworowii var. *album* (hort.) F.T.Hubb. – – wfo-0001303874 – https://biodiversitylibrary.org/page/568410
Limonium sventenii A.Santos & M.L.Fernández – – wfo-0000444076 – http://www.rjb.csic.es/jardinbotanico/jardin/contenido.php?Pag=219&tipo=volumenanales&vol=40(1)
Limonium syracusanum Brullo – – wfo-0000444075 – http://journals.lub.lu.se/index.php/bn/article/view/11412/10558
Limonium tabernense Erben – – wfo-0000444074 – https://biodiversitylibrary.org/page/15235878
Limonium tabulare Bogdanović & Brullo – – wfo-0001303875 – http://dx.doi.org/10.11646/phytotaxa.215.1.1
Limonium tacapense Brullo – – wfo-0000444073 – https://www.biodiversitylibrary.org/page/27802315
Limonium taenari Erben & Brullo – – wfo-0001303876 – http://dx.doi.org/10.11646/phytotaxa.240.1.1
Limonium tamaricoides Bokkari – – wfo-0000444065
Limonium tamarindanum Erben – – wfo-0000444072 – http://www.biodiversitylibrary.org/openurl?pid=title:14894&volume=28&issue=&spage=313&date=1989
Limonium tanaiticum Gamajun. – – wfo-0001303877
Limonium tarcoense Arrigoni & Diana – – wfo-0000444081
Limonium tataricum (L.) Mill. – – wfo-0000444069 – https://biodiversitylibrary.org/page/395149
Limonium tataricum var. *angustifolium* (Boiss.) F.T.Hubb. – – wfo-0001303878 – https://biodiversitylibrary.org/page/568410
Limonium tataricum var. *coccineum* F.T.Hubb. – – wfo-0001303879 – https://biodiversitylibrary.org/page/568410
Limonium tataricum var. *nanum* F.T.Hubb. – – wfo-0001303880
Limonium tauromenitanum Brullo – – wfo-0000444068 – http://journals.lub.lu.se/index.php/bn/article/view/11412/10558
Limonium tenellum (Turcz.) Ikonn.-Gal. – – wfo-0001303881
Limonium tenellum (Turcz.) Kuntze – – wfo-0001095334 – http://www.biodiversitylibrary.org/openurl?pid=title:327&volume=2&issue=&spage=396&date=1891
Limonium tenium (Heldr.) Rech.f. – – wfo-0000444067
Limonium tenoreanum (Guss.) Pignatti – – wfo-0000444066 – https://doi.org/10.1111/njb.01313
Limonium tenuicaule Erben – – wfo-0000444051 – http://www.biodiversitylibrary.org/openurl?pid=title:14894&volume=28&issue=&spage=313&date=1989
Limonium tenuicolum (Tineo ex Guss.) Pignatti – – wfo-0001303882
Limonium tenuiculum (Tineo ex Cuss.) Desole – – wfo-0001303883 – https://doi.org/10.1080/00837792.1960.10669709
Limonium tenuiculum (Tineo ex Guss.) Desole & Pignatti – – wfo-0001303884
Limonium tenuiculum (Tineo ex Guss.) Pignatti – – wfo-0000444040 – http://onlinelibrary.wiley.com/doi/10.1111/j.1095-8339.1971.tb02152.x/full
Limonium tenuiculum subsp. *hermaeum* Pignatti – – wfo-0000745482
Limonium tenuifolium (Bertol. ex Moris) Erben – – wfo-0000443954 – https://biodiversitylibrary.org/page/15206078
Limonium teretifolium (Baker ex Oliv.) Cufod. – – wfo-0001095204 – http://www.jstor.org/stable/3667449, http://www.jstor.org/stable/3667348
Limonium teretifolium L.Bolus – – wfo-0001303885
Limonium teretiscaposum S.D.Zhao – – wfo-0001095312
Limonium teretiscaposum var. *microphyllum* S.D.Zhao – – wfo-0001303886
Limonium tetragonum (Thunb.) Bullock – – wfo-0001285526 – http://www.jstor.org/stable/4108842
Limonium teuchirae Brullo – – wfo-0000443955 – https://doi.org/10.1080/00837792.1978.10670114
Limonium thaenicum Brullo – – wfo-0000443950 – https://biodiversitylibrary.org/page/27802318
Limonium tharrosianum Arrigoni & Diana – – wfo-0000443949
Limonium thiniense Erben – – wfo-0001095146 – https://biodiversitylibrary.org/page/15206058

Limonium thirae Erben & Brullo – – wfo-0001303887 – http://dx.doi.org/10.11646/phytotaxa.240.1.1
Limonium thouinii (Viv.) Kuntze – – wfo-0000443948 – https://biodiversitylibrary.org/page/3817
Limonium thymoides (Girard) M.B.Crespo – – wfo-0001303888
Limonium tianschanicum Lincz. – – wfo-0000443947
Limonium tibulatium Pignatti – – wfo-0000443962 – https://doi.org/10.1080/00837792.1982.10670239
Limonium tigulianum Arrigoni & Diana – – wfo-0000443951
Limonium tineoi (Lojac.) Giardina & Raimondo – – wfo-0001303889 – http://dx.doi.org/10.11646/phytotaxa.196.1.1
Limonium tobarrense J.Moreno, Terrones, M.Á.Alonso, Juan & M.B.Crespo – – wfo-0001303890 – http://dx.doi.org/10.11646/phytotaxa.257.1.4
Limonium todaroanum Raimondo & Pignatti – – wfo-0000443952 – https://doi.org/10.1080/00837792.1986.10670381
Limonium toletanum Erben – – wfo-0000443971 – http://www.biodiversitylibrary.org/openurl?pid=title:14894&volume=28&issue=&spage=313&date=1989
Limonium tomentellum (Boiss.) Kuntze – – wfo-0000443970 – https://biodiversitylibrary.org/page/3818
Limonium tomentellum subsp. *alutaceum* (Steven) Moysiyenko – – wfo-0001303891
Limonium tomentellum subsp. *donetzicum* (Klokov) Moysiyenko – – wfo-0001303892
Limonium tomentellum subsp. *hypanicum* (Klokov) Moysiyenko – – wfo-0001303893
Limonium tomentellum var. *sareptanum* C.E.Salmon – – wfo-0001303894 – https://www.biodiversitylibrary.org/page/9103689
Limonium tommasinii (Pignatti) Brullo – – wfo-0001443425
Limonium Tourn. ex Mill. – – wfo-0001303051 – https://bibdigital.rjb.csic.es/viewer/14683/?offset=#page=415
Limonium tournefortii (Girard) Erben – – wfo-0000443968 – https://biodiversitylibrary.org/page/15235849
Limonium trachycladum Maire & Wilczek – – wfo-0000443967
Limonium transcanalis (Ingr.) P.D.Sell – – wfo-0001303895
Limonium transwallianum (Pugsley) Pugsley – – wfo-0000443966
Limonium tremolsii (Rouy) P.Fourn. – – wfo-0001304462
Limonium tremolsii (Rouy) P.Fourn. ex Erben – – wfo-0000745481 – https://www.biodiversitylibrary.org/page/15235779
Limonium trichogonum f. *albiflorum* House – – wfo-0001303896
Limonium trichogonum S.F.Blake – – wfo-0001242843 – http://www.biodiversitylibrary.org/openurl?pid=title:721&volume=18&issue=207&spage=61&date=1916
Limonium trigonodes Kuntze – – wfo-0000443963 – https://biodiversitylibrary.org/page/3817
Limonium trinajsticii Bogdanović & Brullo – – wfo-0001303897 – http://dx.doi.org/10.11646/phytotaxa.215.1.1
Limonium tritonianum Brullo – – wfo-0000443972 – http://www.biodiversitylibrary.org/openurl?pid=title:14894&volume=28&issue=&spage=419&date=1989
Limonium trojae Pignatti – – wfo-0000443961 – https://doi.org/10.1080/00837792.1982.10670239
Limonium tschurjukiense (Klokov) Lavr. ex Klokov – – wfo-0001095241
Limonium tuberculatum (Boiss.) Kuntze – – wfo-0000443960 – https://biodiversitylibrary.org/page/3818
Limonium tubiflorum (Delile) Kuntze – – wfo-0000443959 – https://biodiversitylibrary.org/page/3818
Limonium tubiflorum subsp. *maroccanum* (Batt. & Trab.) Maire & Weiller – – wfo-0001303898
Limonium tubiflorum subsp. *zanonii* (Pamp.) Brullo – – wfo-0001303899 – https://doi.org/10.1080/00837792.1978.10670114
Limonium tubiflorum var. *zanonii* (Pamp.) Maire – – wfo-0001303900
Limonium tunetanum (Barratte & Bonnet) Maire – – wfo-0000443957
Limonium tunetanum subsp. *eugeniae* (Sennen) Pignatti – – wfo-0001303901
Limonium turritanum Diana – – wfo-0000443956
Limonium tyrrhenicum Arrigoni & Diana – – wfo-0000443944
Limonium ugijarense Erben – – wfo-0000443964 – http://www.biodiversitylibrary.org/openurl?pid=title:14894&volume=28&issue=&spage=313&date=1989
Limonium ursanum Erben – – wfo-0000443945 –

http://www.biodiversitylibrary.org/openurl?pid=title:14894&volume=22&issue=&spage=214&date=1986
Limonium usticanum Giardina & Raimondo – – wfo-0000509740 – http://www.herbmedit.org/bocconea20.html
Limonium usticanum Giardina & Raimondo – – wfo-0001303036 – http://dx.doi.org/10.11646/phytotaxa.196.1.1
Limonium vaccarii Pignatti ex Brullo – – wfo-0000443933 – https://doi.org/10.1080/00837792.1978.10670114
Limonium validum Erben – – wfo-0000443931 – http://www.biodiversitylibrary.org/openurl?pid=title:14894&volume=28&issue=&spage=313&date=1989
Limonium vanandense Erben & Brullo – – wfo-0001303902 – http://dx.doi.org/10.11646/phytotaxa.240.1.1
Limonium vanense Kit Tan & Sorger – – wfo-0000443930
Limonium velutinum Bogdanović & Brullo – – wfo-0001303903 – http://dx.doi.org/10.11646/phytotaxa.215.1.1
Limonium vestitum (C.E.Salmon) C.E.Salmon – – wfo-0000443929 – http://archive.bsbi.org.uk/journal_of_botany.html
Limonium vestitum subsp. *brusnicense* Trinajstić – – wfo-0001303904
Limonium vestitum subsp. *vestitum* – – wfo-0001303905
Limonium viciosoi (Pau) Erben – – wfo-0000443928 – https://biodiversitylibrary.org/page/15235772
Limonium vigaroense Marrero Rodr. & R.S.Almeida – – wfo-0000507527 – https://dialnet.unirioja.es/servlet/articulo?codigo=2242681
Limonium vigoi L.Sáez, Curcó & Rosselló – – wfo-0000443926 – http://www.rjb.csic.es/jardinbotanico/jardin/contenido.php?Pag=219&tipo=volumenanales&vol=56(2)
Limonium viniolae Arrigoni & Diana – – wfo-0000443917
Limonium virgatum (Willd.) Fourr. – – wfo-0000443934 – https://biodiversitylibrary.org/page/54553850
Limonium virgatum subsp. *dictyocladum* (Arcang.) Pignatti – – wfo-0001303906
Limonium virgatum subsp. *divaricatum* Pignatti – – wfo-0000745490
Limonium virgatum subsp. *fontqueri* (Pau) O.Bolòs, Vigo, Masalles & Ninot – – wfo-0001260280
Limonium virgatum subsp. *pseudodictyocladum* (Pignatti) O.Bolòs, Vigo, Masalles & Ninot – – wfo-0000443922
Limonium virgatum subsp. *pseudodictyocladum* Pignatti – – wfo-0000745492
Limonium virgatum subsp. *sardoum* Pignatti – – wfo-0000745491
Limonium vravronense Erben & Brullo – – wfo-0001303907 – http://dx.doi.org/10.11646/phytotaxa.240.1.1
Limonium vulgare f. *pyramidale* C.E.Salmon – – wfo-0001303908
Limonium vulgare Mill. – – wfo-0000443938 – https://biodiversitylibrary.org/page/395149
Limonium vulgare subsp. *angustifolium* (Tausch) P.Fourn. – – wfo-0001303909
Limonium vulgare subsp. *bahusiense* (Fr.) Bech. – – wfo-0001303910 – https://bibdigital.rjb.csic.es/viewer/14716/?offset=#page=22&viewer=picture&o=bookmark&n=0&q=
Limonium vulgare subsp. *behen* (Drejer) Bech. – – wfo-0001303911 – https://bibdigital.rjb.csic.es/viewer/14716/?offset=#page=22&viewer=picture&o=bookmark&n=0&q=
Limonium vulgare subsp. *humile* (Mill.) Gams – – wfo-0001303912 – https://bibdigital.rjb.csic.es/viewer/16557/?offset=#page=353&viewer=picture&o=bookmark&n=0&q=
Limonium vulgare subsp. *pseudolimonium* (Rchb.) Gams – – wfo-0001303913 – https://bibdigital.rjb.csic.es/viewer/16557/?offset=#page=353
Limonium vulgare subsp. *serotinum* (Rchb.) Gams – – wfo-0000748107 – https://bibdigital.rjb.csic.es/viewer/16557/?offset=#page=354&viewer=picture&o=bookmark&n=0&q=
Limonium vulgare var. *album* F.T.Hubb. – – wfo-0001303914 – https://biodiversitylibrary.org/page/568410
Limonium vulgare var. *macroclada* (Boiss.) F.T.Hubb. – – wfo-0001303915 – https://biodiversitylibrary.org/page/568410
Limonium wendelboi Bokhari – – wfo-0000443925
Limonium wessexense (Ingr.) P.D.Sell – – wfo-0001303916
Limonium wiedmannii Erben – – wfo-0000443943 – http://www.biodiversitylibrary.org/openurl?pid=title:14894&volume=22&issue=&spage=206&date=1986
Limonium willdenowii (Poir.) P.Fourn. – – wfo-0000443942
Limonium willdenowii Pignatti – – wfo-0001303917
Limonium woolacombense P.D.Sell – – wfo-0001303918
Limonium wrightii (Hance) Kuntze – – wfo-0001095319 –

http://www.biodiversitylibrary.org/openurl?pid=title:327&volume=2&issue=&spage=396&date=1891
Limonium wrightii f. *wrightii* (Hance) Kuntze – – wfo-0001303919
Limonium wrightii var. *wrightii* (Hance) Kuntze – – wfo-0001095320
Limonium wrightii f. *albescens* Hatus. – – wfo-0001303920
Limonium wrightii f. *albolutescens* Hatus. – – wfo-0001303921
Limonium wrightii f. *arbusculum* (Maxim.) Hatus. – – wfo-0001303922
Limonium wrightii var. *luteum* (H.Hara) H.Hara – – wfo-0001095321
Limonium wrightii var. *roseum* H.Hara – – wfo-0001095322
Limonium xerocamposicum Erben & Brullo – – wfo-0001303923 – http://dx.doi.org/10.11646/phytotaxa.240.1.1
Limonium xerophilum Brullo – – wfo-0000444006 – http://www.biodiversitylibrary.org/openurl?pid=title:14894&volume=28&issue=&spage=419&date=1989
Limonium xiliense Erben & Brullo – – wfo-0001303924 – http://dx.doi.org/10.11646/phytotaxa.240.1.1
Limonium xipholepis (Baker) Hutch. & E.A.Bruce – – wfo-0000444022
Limonium zacynthium R.Artelari – – wfo-0000444021 – https://biodiversitylibrary.org/page/27801772
Limonium zankii Bogdanović & Brullo – – wfo-0001303925 – http://dx.doi.org/10.11646/phytotaxa.215.1.1
Limonium zanonii (Pamp.) Domina – – wfo-0000749442 – http://www.bioone.org/doi/abs/10.3372/wi.41.41117
Limonium zembrae Pignatti – – wfo-0000444020 – https://doi.org/10.1080/00837792.1982.10670239
Limonium zeraphae Brullo – – wfo-0000444019 – http://journals.lub.lu.se/index.php/bn/article/view/11412/10558
Limonium zeugitanum Brullo – – wfo-0000444018 – http://www.biodiversitylibrary.org/openurl?pid=title:14894&volume=28&issue=&spage=419&date=1989
Linczevskia sinuata (L.) Tzvelev – – wfo-0001303926
Linczevskia thouinii (Viv.) Tzvelev – – wfo-0001303927
Linczevskia Tzvelev – – wfo-0001303928
Molubda Raf. – – wfo-4000024594
Molubda scandens (L.) Raf. – – wfo-0000452569 – https://biodiversitylibrary.org/page/404679
Muellerolimon Lincz. – – wfo-4000024951
Muellerolimon salicorniaceum (F.Muell.) Lincz. – – wfo-0001038948
Myriolepis (Boiss.) Lledó, Erben & M.B.Crespo – – wfo-0001303929 – http://www.ingentaconnect.com/contentone/iapt/tax/2003/00000052/00000001/art00007
Myriolepis diffusa (Pourr.) Lledó, Erben & M.B.Crespo – – wfo-0001269877 – http://www.ingentaconnect.com/contentone/iapt/tax/2003/00000052/00000001/art00007
Myriolepis ferulacea (L.) Lledó, Erben & M.B.Crespo – – wfo-0001269876 – https://onlinelibrary.wiley.com/doi/epdf/10.2307/3647438
Myriolimon diffusum (Pourr.) Lledó, Erben & M.B.Crespo – – wfo-0000452572 – http://www.ingentaconnect.com/contentone/iapt/tax/2005/00000054/00000003/art00027#
Myriolimon ferulaceum (L.) Lledó, Erben & M.B.Crespo – – wfo-0000452571 – http://www.ingentaconnect.com/contentone/iapt/tax/2005/00000054/00000003/art00027#
Myriolimon Lledó, Erben & M.B.Crespo – – wfo-4000025142 – http://www.ingentaconnect.com/contentone/iapt/tax/2005/00000054/00000003/art00027#
Neogontscharovia Lincz. – – wfo-4000025696
Neogontscharovia mira (Lincz.) Lincz. – – wfo-0001095242
Neogontscharovia miranda (Lincz.) Lincz. – – wfo-0001095243
Neogontscharovia saxifragifolia (Rech.f. & Köie) Lincz. – – wfo-0000380899
Plegorhiza adstringens Willd. – – wfo-0000487077 – http://www.biodiversitylibrary.org/openurl?pid=title:727&volume=2&issue=1&spage=487&date=
Plegorhiza guaicuru Molina – – wfo-0000487099 – http://www.biodiversitylibrary.org/openurl?pid=title:62689&volume=&issue=&spage=164&date=1782
Plegorhiza Molina – – wfo-4000030222 – http://www.biodiversitylibrary.org/openurl?pid=title:62689&volume=&issue=&spage=164&date=1782
Plegorhiza quicura Steud. – – wfo-0000487075 – https://www.biodiversitylibrary.org/page/392431

Plumbagella micrantha (Ledeb.) Spach – – wfo-0001095249 – https://biodiversitylibrary.org/page/32442011
Plumbagella micrantha var. *himalaica* W.W.Sm. – – wfo-0001303930
Plumbagella micrantha var. *micrantha* – – wfo-0001303931
Plumbagella Spach – – wfo-4000030422 – https://biodiversitylibrary.org/page/32442011
Plumbagidium auriculatum (Lam.) Spach – – wfo-0000487108 – https://biodiversitylibrary.org/page/32442017
Plumbagidium roseum (L.) Spach – – wfo-0000487109 – https://biodiversitylibrary.org/page/32442017
Plumbagidium scandens (L.) Spach – – wfo-0000487110 – https://biodiversitylibrary.org/page/32442017
Plumbagidium Spach – – wfo-4000030423 – http://www.biodiversitylibrary.org/openurl?pid=title:44839&volume=10&issue=&spage=338&date=1841
Plumbaginaceae Juss. – – wfo-7000000482
Plumbagineae Bartl. – – wfo-0001303932
Plumbagineae Dumort. – – wfo-0001303933 – https://www.biodiversitylibrary.org/page/33110212
Plumbago africana (Lam.) Christenh. & Byng – – wfo-0001303934 – http://www.plantgateway.com/wp-content/uploads/lana-downloads/2018/02/Global-Flora-Vol-4.pdf
Plumbago alba hort. ex Pasq. – – wfo-0001095368
Plumbago americana L. – – wfo-0001303935
Plumbago americana Weigel – – wfo-0000487120 – https://www.jstor.org/stable/1220984?seq=1#page_scan_tab_contents
Plumbago amplexicaulis Oliv. – – wfo-0001095259 – http://www.biodiversitylibrary.org/openurl?pid=title:350&volume=15&issue=&spage=96&date=1876
Plumbago angustifolia Spach – – wfo-0000487121 – https://biodiversitylibrary.org/page/32442015
Plumbago aphylla Bojer ex Boiss. – – wfo-0001095152 – http://www.biodiversitylibrary.org/openurl?pid=title:286&volume=12&issue=&spage=694&date=1848
Plumbago arabica (Boiss.) Christenh. & Byng – – wfo-0001303936
Plumbago auriculata Blume – – wfo-0001303937 – https://biodiversitylibrary.org/page/429430
Plumbago auriculata f. *alba* (Pasq.) T.H.Peng – – wfo-0001095367
Plumbago auriculata f. *auriculata* – – wfo-0001095366
Plumbago auriculata Lam. – – wfo-0000487119 – http://www.biodiversitylibrary.org/openurl?pid=title:824&volume=2&issue=1&spage=270&date=1786
Plumbago caerulea Kunth – – wfo-0000487097 – http://www.biodiversitylibrary.org/openurl?pid=title:640&volume=2&issue=&spage=220&date=1818
Plumbago capensis f. *alba* hort. ex Carrière – – wfo-0001303938
Plumbago capensis Thunb. – – wfo-0000487098 – http://www.biodiversitylibrary.org/openurl?pid=title:84&volume=&issue=&spage=33&date=1794
Plumbago capensis Willd. ex Boiss. – – wfo-0001249146 – https://biodiversitylibrary.org/page/160915
Plumbago ciliata Engl. ex Wilmot-Dear – – wfo-0001095263 – https://www.jstor.org/stable/4109559?seq=1#page_scan_tab_contents
Plumbago cocanex hort. ex Gentil – – wfo-0001303939
Plumbago coccinea Salisb. – – wfo-0000487106 – http://www.biodiversitylibrary.org/openurl?pid=title:427&volume=&issue=&spage=122&date=1796
Plumbago coerulea Auct. – – wfo-0001303940
Plumbago dawei Rolfe – – wfo-0001095153 – http://www.biodiversitylibrary.org/openurl?pid=title:350&volume=37&issue=&spage=522&date=1906
Plumbago denticulata St.-Lag. – – wfo-0000487101
Plumbago dyerophyta Christenh. & Byng – – wfo-0001303941
Plumbago eglandulosa R.Br. – – wfo-0000487102 – https://biodiversitylibrary.org/page/47589044
Plumbago esquirolii H.Lév. – – wfo-0001095370 – https://www.biodiversitylibrary.org/page/237664
Plumbago europaea L. – – wfo-0000487103 – http://www.biodiversitylibrary.org/openurl?pid=title:669&volume=1&issue=&spage=151&date=1753
Plumbago flaccida Moench – – wfo-0000487104 – https://biodiversitylibrary.org/page/449060

Plumbago floridana Nutt. – – wfo-0000487105
Plumbago floridana Raf. – – wfo-0000487071
Plumbago glandulicaulis Wilmot-Dear – – wfo-0001095262 – https://www.jstor.org/stable/4109559?seq=1#page_scan_tab_contents
Plumbago glandulosa Willd. ex Roem. & Schult. – – wfo-0000487078 – https://www.biodiversitylibrary.org/page/770025
Plumbago grandiflora Ten. – – wfo-0000487038
Plumbago humboldtiana Roem. & Schult. – – wfo-0000487039
Plumbago hunsbergensis van Jaarsv., Swanepoel & A.E.van Wyk – – wfo-0001303942
Plumbago indica L. – – wfo-0001095164 – http://www.biodiversitylibrary.org/openurl?pid=title:78821&volume=&issue=&spage=24&date=1754
Plumbago ituriensis Ntore – – wfo-0001303943
Plumbago juncea Bojer – – wfo-0000487040
Plumbago lactea Salisb. – – wfo-0000487048 – https://biodiversitylibrary.org/page/1485092
Plumbago lanceolata Sessé & Moc. – – wfo-0000487042 – http://www.biodiversitylibrary.org/openurl?pid=title:58596&volume=&issue=&spage=31&date=1893
Plumbago lapathifolia Willd. – – wfo-0000487036
Plumbago larpentae Lindl. – – wfo-0001095317 – https://biodiversitylibrary.org/page/31037232
Plumbago larpentiae Lindl. – – wfo-0001304464
Plumbago littoralis Mure – – wfo-0001303944
Plumbago madagascariensis M.Peltier – – wfo-0001095154 – http://www.biodiversitylibrary.org/openurl?pid=title:6600&volume=163&issue=&spage=22&date=1981
Plumbago maximowiczii Gand. – – wfo-0000487045 – https://biodiversitylibrary.org/page/345071
Plumbago mexicana Kunth – – wfo-0000487046 – https://www.biodiversitylibrary.org/page/5196, https://www.biodiversitylibrary.org/page/58729016
Plumbago micrantha Ledeb. – – wfo-0001095216 – http://www.biodiversitylibrary.org/openurl?pid=title:6618&volume=1&issue=&spage=171&date=1829
Plumbago montis-elgonis Bullock – – wfo-0001095261 – https://www.jstor.org/stable/4113427?seq=1#page_scan_tab_contents
Plumbago occidentalis Sweet – – wfo-0000487043 – https://biodiversitylibrary.org/page/30078653
Plumbago parvifolia Hemsl. – – wfo-0000487041 – http://www.biodiversitylibrary.org/openurl?pid=title:15787&volume=54&issue=&spage=362&date=1916
Plumbago pearsonii L.Bolus – – wfo-0001095167
Plumbago pendula (Balf.f.) Christenh. & Byng – – wfo-0001303945
Plumbago pulchella Boiss. – – wfo-0001095277 – http://www.biodiversitylibrary.org/openurl?pid=title:286&volume=12&issue=&spage=692&date=1848
Plumbago purpurea Salisb. – – wfo-0000487028
Plumbago rhombifolia Steud. – – wfo-0001303946
Plumbago rhomboidea G.Lodd. – – wfo-0000487033 – https://biodiversitylibrary.org/page/28550771
Plumbago rhomboidea Hook. – – wfo-0000487030
Plumbago rosea L. – – wfo-0001095165 – http://www.biodiversitylibrary.org/openurl?pid=title:11179&volume=1&issue=&spage=215&date=1762
Plumbago rosea var. *coccinea* (Lour.) Hook. – – wfo-0001303947 – https://www.biodiversitylibrary.org/page/438955
Plumbago sarmentosa Lam. – – wfo-0000487027 – https://biodiversitylibrary.org/page/794655
Plumbago scandens f. *erecta* Chodat & Hassl. – – wfo-0001303948 – https://biodiversitylibrary.org/page/33743184
Plumbago scandens L. – – wfo-0000487031 – http://www.biodiversitylibrary.org/openurl?pid=title:11179&volume=1&issue=&spage=215&date=1762
Plumbago scandens var. *densiflora* Kuntze – – wfo-0001303949 – https://biodiversitylibrary.org/page/3818
Plumbago scandens var. *erecta* Chodat & Hassl. – – wfo-0001303950
Plumbago scandens var. *normalis* Kuntze – – wfo-0001303951 – https://biodiversitylibrary.org/page/3818
Plumbago sect. *Plumbagidium* Boiss. – – wfo-0001304511

Plumbago spinosa K.S.Hao – – wfo-0001095287
Plumbago stenophylla Wilmot-Dear – – wfo-0001095260 – https://www.jstor.org/stable/4109559?seq=1#page_scan_tab_contents
Plumbago suffruticosa Schinz – – wfo-0000487032
Plumbago tomentosa var. *pumila* Hook.f. – – wfo-0001303952
Plumbago Tourn. ex L. – – wfo-4000030425 – https://biodiversitylibrary.org/page/358170
Plumbago Tourn. ex L. sect. *Plumbago* – – wfo-0001304510
Plumbago toxicaria Bertol. – – wfo-0000487055
Plumbago tristis W.T.Aiton – – wfo-0001095168 – https://biodiversitylibrary.org/page/48083718
Plumbago undulata Moench – – wfo-0000487047
Plumbago virginica L. – – wfo-0001303953
Plumbago viscosa Blanco – – wfo-0001095286 – http://bibdigital.rjb.csic.es/ing/Libro.php?Libro=36&Pagina=157
Plumbago vogeliaefolia Eckl. & Zeyh. ex Boiss. – – wfo-0000487061 – https://biodiversitylibrary.org/page/160915
Plumbago vogeliifolia Eckl. & Zeyh. ex Boiss. – – wfo-0001304465 – https://biodiversitylibrary.org/page/160915
Plumbago wissii Friedrich – – wfo-0001095169
Plumbago zeylanica L. – – wfo-0000487062 – http://www.biodiversitylibrary.org/openurl?pid=title:669&volume=1&issue=&spage=151&date=1753
Plumbago zeylanica var. *zeylanica* L. – – wfo-0001095273
Plumbago zeylanica var. *dawei* (Rolfe) Mildbr. – – wfo-0001303954 – https://www.biodiversitylibrary.org/page/6559531
Plumbago zeylanica var. *glaucescens* Boiss. – – wfo-0001303955 – https://biodiversitylibrary.org/page/160914
Plumbago zeylanica var. *oxypetala* Boiss. – – wfo-0001303956 – https://www.biodiversitylibrary.org/page/160915
Plumbago zeylanica var. *rosea* (L.) Williams – – wfo-0001303957 – https://biodiversitylibrary.org/page/33511137
Polyanthemum Medik. – – wfo-0001303958
Popoviolimon Lincz. – – wfo-4000030918
Popoviolimon turcomanicum (Popov ex Lincz.) Lincz. – – wfo-0001095244
Psylliostachys (Jaub. & Spach) Nevski – – wfo-4000031852
Psylliostachys ×afghanica Roshkova – – wfo-0000487066
Psylliostachys ×androssovii Roshkova – – wfo-0000487068 – https://biodiversitylibrary.org/page/30218713
Psylliostachys ×myosuroides (Regel) Roshkova – – wfo-0000487063 – https://biodiversitylibrary.org/page/30218502
Psylliostachys anceps (Regel) Roshkova – – wfo-0000487067 – https://biodiversitylibrary.org/page/30218504
Psylliostachys beludshistanica Roshkova – – wfo-0000487069
Psylliostachys hymenostegia Rech.f. – – wfo-0000487070
Psylliostachys koelzii Rech.f. – – wfo-0000487057
Psylliostachys leptostachya (Boiss.) Roshkova – – wfo-0001095245 – https://biodiversitylibrary.org/page/30218501
Psylliostachys leptostachyus (Boiss.) Roshkova – – wfo-0000487065
Psylliostachys plantaginiflora (Jaub. & Spach) Roshkova – – wfo-0000487049
Psylliostachys sisymbriifolia (Jaub. & Spach) Rech.f. – – wfo-0000487050
Psylliostachys spicata (Willd.) Nevski – – wfo-0000487052
Psylliostachys suvorovii (Regel) Roshk. – – wfo-0001303959
Psylliostachys suworowi (Regel) Roshkova – – wfo-0000487053
Psylliostachys suworowii (Regel) Roshkova – – wfo-0001095246 – https://biodiversitylibrary.org/page/30218501
Psylliostachys volkii Rech.f. – – wfo-0000487054
Reverchonia baetica (Boiss.) Gand. – – wfo-0001303960
Reverchonia Gand. – – wfo-0001303961
Saharanthus ifniensis (Caball.) M.B.Crespo & Lledó – – wfo-0000503719 –

http://onlinelibrary.wiley.com/doi/10.1111/j.1095-8339.2000.tb01212.x/full
Saharanthus M.B.Crespo & Lledó – – wfo-4000033798 – http://onlinelibrary.wiley.com/doi/10.1111/j.1095-8339.2000.tb01212.x/full
Statice (L.) Mill. – – wfo-0001303962
Statice ×abnormis Rouy – – wfo-0000503717
Statice ×ambigua Rouy – – wfo-0000503808 – https://www.biodiversitylibrary.org/page/14597830
Statice ×coincyi Sennen – – wfo-0000503680
Statice ×erectiflora B.Fedtsch. & Gontsch. – – wfo-0000503945
Statice ×girardianoides H.Lév. – – wfo-0001303963
Statice ×gracillima (J.J.Rodr.) Rouy – – wfo-0000504023 – https://biodiversitylibrary.org/page/14597830
Statice ×intermedia Czern. – – wfo-0000503857
Statice ×multiramea Sennen – – wfo-0000503898
Statice ×myosuroides Regel – – wfo-0000503882
Statice ×neumani Rouy – – wfo-0000503900
Statice ×pinto-silvae Rothm. – – wfo-0000503326
Statice ×profusa T.Moore – – wfo-0001303964
Statice ×pseudoconfusa Rouy – – wfo-0000503299
Statice ×sennenii Rouy – – wfo-0000503362
Statice ×valentina Huter, Porta & Rigo – – wfo-0000503219
Statice ×virgatoformis Rouy – – wfo-0000503251
Statice acerosa Willd. – – wfo-0001095247
Statice aciphylla Jaub. & Spach – – wfo-0000503715
Statice acutifolia Badarò ex Rchb. – – wfo-0000503714 – http://bibdigital.rjb.csic.es/ing/Libro.php?Libro=1064&Pagina=29
Statice acutifolia Ehrenb. ex Boiss. – – wfo-0001304456 – https://biodiversitylibrary.org/page/160860
Statice acutifolia var. *obtusifolia* Rouy – – wfo-0001303965
Statice adensis (Nimmo) Boiss. – – wfo-0000503711 – https://biodiversitylibrary.org/page/160892
Statice aegyptiaca Pers. – – wfo-0000503751 – https://biodiversitylibrary.org/page/235119
Statice aggregata Rouy – – wfo-0000503795 – https://www.biodiversitylibrary.org/page/12880587
Statice airoides Tausch – – wfo-0000503794
Statice akkensis Coss. ex Batt. – – wfo-0000503792
Statice alata Willd. – – wfo-0000503791
Statice alatavica Regel & Schmalh. – – wfo-0000503790 – https://biodiversitylibrary.org/page/14579535
Statice alba Coincy – – wfo-0000745501 – https://biodiversitylibrary.org/page/8855590
Statice alba Pau – – wfo-0001303966
Statice albertii Regel – – wfo-0000503789 – https://biodiversitylibrary.org/page/27410283
Statice albicans Ledeb. – – wfo-0000503778
Statice albida Guss. – – wfo-0000503787 – https://biodiversitylibrary.org/page/42982951
Statice algeriensis Rouy – – wfo-0000503796 – https://www.biodiversitylibrary.org/page/14597796
Statice alleizettei Pau – – wfo-0001303967 – http://bibdigital.rjb.csic.es/ing/Libro.php?Libro=808&Pagina=96
Statice alliacea Cav. – – wfo-0000503785 – https://bibdigital.rjb.csic.es/viewer/9680/?offset=#page=10
Statice alliacea Guss. ex Nyman – – wfo-0000503783 – https://biodiversitylibrary.org/page/11015758
Statice alliacea Sm. – – wfo-0000503784 – https://www.biodiversitylibrary.org/page/54121987
Statice alliacea var. *apollinaris* (Sennen & Mauricio) Maire – – wfo-0001303968
Statice alliacea var. *matritensis* Pau – – wfo-0001303969

Statice alliacea Willd. – – wfo-0000503781 – https://www.biodiversitylibrary.org/page/630840
Statice allioides Braun-Blanq. – – wfo-0000503780
Statice allioides var. *yebalica* (Pau) Maire – – wfo-0001303970
Statice alpina (DC.) Poir. – – wfo-0000503779 – https://biodiversitylibrary.org/page/782264
Statice alpinifolia (Pau & Font Quer) Maire – – wfo-0000503803 – https://bibdigital.rjb.csic.es/viewer/13176/?offset=#page=19&viewer=picture&o=bookmark&n=0&q=
Statice altaica hort. ex G.Don – – wfo-0000503805
Statice alutacea Steven – – wfo-0000503788 – https://biodiversitylibrary.org/page/44192716
Statice ambigua f. *major* Lojac. – – wfo-0000510382
Statice ambigua Tineo ex Lojac. – – wfo-0000503809
Statice ambigua var. *ponzoi* Fiori & Bég. – – wfo-0000745479
Statice amblyoloba Ikonn.-Gal. – – wfo-0001303971
Statice amblyoloba Popov – – wfo-0001303972
Statice americana Larrañaga – – wfo-0000503807
Statice amoena C.H.Wright – – wfo-0001095172 – https://biodiversitylibrary.org/page/713907
Statice amplifoliata (Pau) Maire – – wfo-0000503806
Statice ancarensis (Merino) Rothm. – – wfo-0000503797
Statice anceps Regel – – wfo-0000503804
Statice androsacea Jaub. & Spach – – wfo-0000503812 – https://babel.hathitrust.org/cgi/pt?id=mdp.39015061880194;view=1up;seq=179;size=125
Statice anfracta C.E.Salmon – – wfo-0000503802 – https://biodiversitylibrary.org/page/8074422
Statice angustata (Small) Wangerin – – wfo-0000503801
Statice angustifolia Tausch – – wfo-0000503799
Statice annulata (R.Br.) Spreng. – – wfo-0000503798 – http://bibdigital.rjb.csic.es/ing/Libro.php?Libro=1733&Pagina=966
Statice anthericoides Schltr. – – wfo-0001095174 – https://biodiversitylibrary.org/page/208455
Statice aphylla Forssk. – – wfo-0001303973
Statice aphylla Poir. – – wfo-0001095354 – http://www.biodiversitylibrary.org/openurl?pid=title:824&volume=7&issue=1&spage=408&date=1806
Statice arabica f. *glomerata* N.Terrac. – – wfo-0001303974
Statice arabica Jaub. & Spach – – wfo-0000503757 – https://babel.hathitrust.org/cgi/pt?id=mdp.39015061880194;view=1up;seq=174;size=125
Statice aragonensis Debeaux ex Willk. – – wfo-0000503786 – https://biodiversitylibrary.org/page/11376634
Statice ararati Planch. – – wfo-0000503762
Statice arborea f. *frutescens* (Lem.) Stapf. – – wfo-0001303975
Statice arborea var. *typica* Stapf – – wfo-0001303976
Statice arborea Willd. – – wfo-0000503761 – https://bibdigital.rjb.csic.es/viewer/10963/?offset=#page=344&viewer=picture&o=bookmark&n=0&q=
Statice arborescens Brouss. – – wfo-0000503760 – http://gallica.bnf.fr/ark:/12148/bpt6k97650q/f66.image
Statice arborescens Brouss. ex Webb & Berthel. – – wfo-0001303977
Statice arbuscula Maxim. – – wfo-0001095324
Statice arctica (Cham.) S.F.Blake – – wfo-0001245900 – http://www.biodiversitylibrary.org/openurl?pid=title:721&volume=19&issue=217&spage=8&date=1917
Statice arctica subsp. *californica* (Boiss.) A.E.Murray – – wfo-0001303978
Statice arctica var. *californica* (Boiss.) S.F.Blake – – wfo-0001273465 – http://www.biodiversitylibrary.org/openurl?pid=title:721&volume=19&issue=217&spage=9&date=1917
Statice arenaria Pers. – – wfo-0000503754 – https://biodiversitylibrary.org/page/235117
Statice aretiifolia Fr. ex Boiss. – – wfo-0000503758 – https://biodiversitylibrary.org/page/160879
Statice argentea Pall. ex Siev. – – wfo-0001095296

Statice aristata Sm. – – wfo-0000503756
Statice armeria Brot. – – wfo-0001303979
Statice armeria L. – – wfo-0000503755 – http://www.biodiversitylibrary.org/openurl?pid=title:669&volume=1&issue=&spage=274&date=1753
Statice armeria subsp. *armeria* – – wfo-0001303980
Statice armeria subsp. *elongata* (Hoffm.) P.Fourn. – – wfo-0001303981
Statice armeria subsp. *major* Ehrh. – – wfo-0001303982
Statice armeria subsp. *maritima* (Mill.) P.Fourn. – – wfo-0001303983
Statice armeria var. *alba* (L.H.Bailey) F.T.Hubb. – – wfo-0001303984 – https://biodiversitylibrary.org/page/568407
Statice armeria var. *alpina* DC. – – wfo-0000733944
Statice armeria var. *arenaria* Dumort. – – wfo-0001303985
Statice armeria var. *caudiculata* Dumort. – – wfo-0001303986
Statice armeria var. *elongata* Dumort. – – wfo-0001303987
Statice armeria var. *grandiflora* (L.H.Bailey) F.T.Hubb. – – wfo-0001303988 – https://biodiversitylibrary.org/page/568408
Statice armeria var. *hortensis* Gaudin – – wfo-0001303989
Statice armeria var. *laucheana* (J.N.Haage & E.Schmidt) F.T.Hubb. – – wfo-0001303990 – https://biodiversitylibrary.org/page/568408
Statice armeria var. *maritima* (Mill.) Wahlenb. – – wfo-0001303991
Statice armeria var. *minor* Dumort. – – wfo-0001303992
Statice armeria var. *mixta* Dumort. – – wfo-0001303993
Statice armeria var. *pubescens* Salis – – wfo-0000733946 – http://www.biodiversitylibrary.org/openurl?pid=title:64&volume=17&issue=2&spage=13&date=1834
Statice armeria var. *pubescens* Sowerby ex Sm. – – wfo-0000733945
Statice armeria var. *purpurea* (L.H.Bailey) F.T.Hubb. – – wfo-0001303994
Statice armeria var. *rubra* (L.H.Bailey) F.T.Hubb. – – wfo-0001303995 – https://biodiversitylibrary.org/page/568408
Statice armeria var. *splendens* (L.H.Bailey) F.T.Hubb. – – wfo-0001303996
Statice armeria var. *tenuifolia* DC. – – wfo-0001303997
Statice armeria var. *variegata* F.T.Hubb. – – wfo-0001303998 – https://biodiversitylibrary.org/page/568408
Statice articulata Loisel. – – wfo-0000503777
Statice articulata Schimp. ex Nyman – – wfo-0001303999 – https://biodiversitylibrary.org/page/11015756
Statice articulata var. *strictissima* Salzm. – – wfo-0001304000
Statice articulata Vis. – – wfo-0001304001
Statice asparagoides Coss. & Durieu ex Batt. – – wfo-0000503776 – http://bibdigital.rjb.csic.es/ing/Libro.php?Libro=1454&Pagina=739
Statice aspera Lam. – – wfo-0000503775
Statice asterotricha C.E.Salmon – – wfo-0000503774 – https://biodiversitylibrary.org/page/9105241
Statice asturiana Rothm. – – wfo-0000503771
Statice attenuata Schult. – – wfo-0000503763
Statice aucheri Girard – – wfo-0001244265 – https://biodiversitylibrary.org/page/41531995
Statice aurea L. – – wfo-0001095336 – http://www.biodiversitylibrary.org/openurl?pid=title:669&volume=1&issue=&spage=276&date=1753
Statice auriculae-ursifolia Pourr. – – wfo-0000503770 – https://archive.org/stream/HistoireEtMemoiresToulouse3/Histoire_et_memoires_Toulouse_3#page/n407/mode/2up
Statice auriculata hort. ex Schult. – – wfo-0000503702 – https://www.biodiversitylibrary.org/page/732556
Statice auriculifolia Benth. – – wfo-0000503765
Statice auriculifolia Brot. – – wfo-0000503767 – https://biodiversitylibrary.org/page/31323715
Statice auriculifolia DC. – – wfo-0000503769

Statice auriculifolia Poir. – – wfo-0000503766 – https://biodiversitylibrary.org/page/724427
Statice auriculifolia Vahl – – wfo-0000503768
Statice australis (R.Br.) Spreng. – – wfo-0000503713 – http://bibdigital.rjb.csic.es/ing/Libro.php?Libro=1733&Pagina=965
Statice australis Moris – – wfo-0000503604
Statice avei De Not. – – wfo-0000503629
Statice avenacea C.H.Wright – – wfo-0001095201 – https://biodiversitylibrary.org/page/713909
Statice axillaris Balf.f. – – wfo-0001304002
Statice axillaris f. *attenuata* N.Terrac. – – wfo-0001304003
Statice axillaris Forssk. – – wfo-0001095148
Statice axillaris var. *attenuata* (N.Terrac.) Fiori – – wfo-0001304004
Statice axillaris var. *ellenbeckii* Engl. – – wfo-0001304005
Statice axillaris var. *glomerata* (N.Terrac.) Fiori – – wfo-0001304006
Statice baetica (Boiss.) Font Quer & Rothm. – – wfo-0000503628
Statice bahamensis Griseb. – – wfo-0000503627 – http://www.biodiversitylibrary.org/openurl?pid=title:143&volume=&issue=&spage=389&date=1864http://www.biodiversitylibrary.org/openurl?pid=title:56664&volume=&issue=&spage=389&date=1864
Statice bahusiensis Fr. – – wfo-0000503617
Statice balansae Boiss. – – wfo-0000503626 – http://bibdigital.rjb.csic.es/ing/Libro.php?Libro=1418&Pagina=71
Statice barulensis A.Bruni – – wfo-0000503630
Statice bayonnensis Gren. ex Boiss. – – wfo-0000503622 – https://biodiversitylibrary.org/page/37172644
Statice beaumierana Coss. – – wfo-0001304007
Statice beaumierana Coss. ex Maire – – wfo-0001304008
Statice beaumieriana Coss. ex Maire – – wfo-0000503621 – https://bibdigital.rjb.csic.es/viewer/11128/?offset=#page=101
Statice beaumieriana f. *leucocalyx* Maire – – wfo-0001304009
Statice beaumieriana var. *akkensis* (Coss. ex Batt.) Maire – – wfo-0001304010 – http://bibdigital.rjb.csic.es/ing/Libro.php?Libro=1853&Pagina=103
Statice beaumieriana var. *tripeaui* Maire – – wfo-0001304011 – http://bibdigital.rjb.csic.es/ing/Libro.php?Libro=1853&Pagina=103
Statice behen Drejer – – wfo-0000503620
Statice bella (Albov) Macloskie – – wfo-0000503619
Statice bellidifolia (Gouan) DC. – – wfo-0000503642 – http://bibdigital.rjb.csic.es/ing/Libro.php?Libro=442&Pagina=425
Statice bellidifolia Auct. ex Boiss. – – wfo-0001304012 – https://biodiversitylibrary.org/page/37172643
Statice bellidifolia Bertol. – – wfo-0001304013
Statice bellidifolia Guss. – – wfo-0001304015
Statice bellidifolia Guss. – – wfo-0001304014
Statice bellidifolia Sm. – – wfo-0001304016
Statice bellidifolia var. *divaricata* DC. – – wfo-0001304017
Statice bellidifolia var. *prostrata* Beauverd – – wfo-0001304018
Statice besseriana Friv. ex Boiss. – – wfo-0001304019 – https://biodiversitylibrary.org/page/160857
Statice besseriana Schult. ex Rchb. – – wfo-0000503641
Statice bianorii Sennen & Pau – – wfo-0000503640
Statice bicolor Bunge – – wfo-0001095303
Statice billardierei Girard – – wfo-0000503638 – https://biodiversitylibrary.org/page/41531992
Statice binervosa G.E.Sm. – – wfo-0000503644
Statice binervosa var. *dodarti* (Girard) Hook – – wfo-0001304020

Statice binervosa var. *dodarti* (Girard) Syme – – wfo-0001304021
Statice binervosa var. *intermedia* Syme – – wfo-0001304022
Statice binervosa var. *occidentalis* (J.Lloyd) Syme – – wfo-0001304023
Statice bocconei Lojac. – – wfo-0000503637
Statice boissieri Lafont – – wfo-0000503636 – https://www.biodiversitylibrary.org/page/35747625
Statice bollei Webb ex Wangerin – – wfo-0000503635 – https://biodiversitylibrary.org/page/181583
Statice bonduellei T.Lestib. – – wfo-0000503634 – https://biodiversitylibrary.org/page/41552532
Statice bonduellii T.Lestib. – – wfo-0001304024
Statice bonnetii Sennen – – wfo-0000503633
Statice bottendorfensis (A.G.Schulz) O.Schwarz – – wfo-0000503632
Statice bourgaei Webb ex Boiss. – – wfo-0001280051 – http://www.biodiversitylibrary.org/openurl?pid=title:286&volume=12&issue=&spage=638&date=1848
Statice bovei Jaub. & Spach – – wfo-0001095149 – https://babel.hathitrust.org/cgi/pt?id=mdp.39015061880194;view=1up;seq=175;size=125
Statice bracteata Girard – – wfo-0000503592 – https://biodiversitylibrary.org/page/41531997
Statice bracteosa Viv. – – wfo-0000503625
Statice brasiliense var. *angustata* A.Gray – – wfo-0001243417 – https://www.biodiversitylibrary.org/page/11284314
Statice brasiliense var. *antarctica* Boiss. – – wfo-0001236411 – https://biodiversitylibrary.org/page/160868
Statice brasiliense var. *augustata* A.Gray – – wfo-0001304025
Statice brasiliense var. *uruguayensis* (Arechav.) Hauman – – wfo-0001236410 – https://biodiversitylibrary.org/page/14714345
Statice brasiliensis A.Gray – – wfo-0001304026
Statice brasiliensis Boiss. – – wfo-0001095272 – http://www.biodiversitylibrary.org/openurl?pid=title:286&volume=12&issue=&spage=644&date=1848
Statice brasiliensis Chapm. – – wfo-0001304027
Statice brasiliensis var. *angustata* A.Gray – – wfo-0001304028
Statice brasiliensis var. *patagonica* Hosseus – – wfo-0001304029
Statice brassicaefolia Webb & Berthel. – – wfo-0000503600 – https://biodiversitylibrary.org/page/41422877
Statice brassicifolia var. *macroptera* (Webb & Berthel.) Burchard – – wfo-0001304030
Statice brassicifolia var. *typica* Stapf – – wfo-0001304031 – https://www.biodiversitylibrary.org/page/54255360
Statice brassicifolia Webb & Berthel. – – wfo-0001303043
Statice braunii Bolle – – wfo-0000503599 – https://biodiversitylibrary.org/page/44923888
Statice brunii Guss. ex A.Bruni – – wfo-0000503597 – https://biodiversitylibrary.org/page/14626823
Statice brunneri Webb – – wfo-0000503596 – https://biodiversitylibrary.org/page/551967
Statice bubanii Girard – – wfo-0000503595 – https://biodiversitylibrary.org/page/41531993
Statice bungeana Boiss. – – wfo-0001095304 – http://www.biodiversitylibrary.org/openurl?pid=title:286&volume=12&issue=&spage=642&date=1848
Statice bungei Claus – – wfo-0000503593 – https://biodiversitylibrary.org/page/43059160
Statice cabulica Boiss. – – wfo-0001285492 – http://www.biodiversitylibrary.org/openurl?pid=title:286&volume=12&issue=&spage=666&date=1848
Statice caesia Girard – – wfo-0000503591 – https://biodiversitylibrary.org/page/41531992
Statice caesia var. *major* Rouy – – wfo-0001304032
Statice caespitosa Cav. – – wfo-0000503588 – https://bibdigital.rjb.csic.es/viewer/9679/?offset=#page=42&viewer=picture&o=bookmark&n=0&q=
Statice caespitosa d'Urv. – – wfo-0000503589 – https://biodiversitylibrary.org/page/44510022
Statice caespitosa Ortega – – wfo-0001304033
Statice caespitosa Poir. – – wfo-0000503590 – https://biodiversitylibrary.org/page/724422
Statice calcarae Tod. ex Janka – – wfo-0000503586 – https://biodiversitylibrary.org/page/30862195

Statice calcarae Tod. ex Lojac. – – wfo-0001304034
Statice californica Boiss. – – wfo-0001095315 – http://www.biodiversitylibrary.org/openurl?pid=title:286&volume=12&issue=&spage=643&date=1848
Statice callicoma C.A.Mey. – – wfo-0001095294 – http://www.biodiversitylibrary.org/openurl?pid=title:53549&volume=4&issue=&spage=212&date=1841
Statice cancellata Bernh. ex Bertol. – – wfo-0000503594
Statice cancellata Bernh. ex Mutel, A. – – wfo-0000503615 – https://www.digitale-sammlungen.de/en/view/bsb10302586?page=96
Statice cancellata var. *glabra* Boiss. – – wfo-0001304035
Statice cancellata var. *japygica* E.Groves – – wfo-0000748114
Statice canescens Host – – wfo-0000503613
Statice cantabrica (Boiss. & Reut. ex Willk. & Lange) P.Fourn. – – wfo-0000503612
Statice cantabrica subsp. *pubinervis* (Boiss.) P.Fourn. – – wfo-0001304036
Statice capensis L.Bolus – – wfo-0001095176
Statice capillifolia Poir. – – wfo-0000503611 – https://biodiversitylibrary.org/page/724423
Statice capitata Lam. – – wfo-0000503603
Statice capitella (Pau) Font Quer & Rothm. – – wfo-0000503616
Statice caput-alba Rothm. – – wfo-0000503610
Statice carinensis Chiov. – – wfo-0000503609
Statice carnosa Boiss. – – wfo-0000503608
Statice caroliniana var. *albiflora* Raf. – – wfo-0001304037
Statice caroliniana var. *cespitosa* Raf. – – wfo-0001304038
Statice caroliniana var. *longifolia* Raf. – – wfo-0001304039
Statice caroliniana var. *pumila* Raf. – – wfo-0001304040
Statice caroliniana var. *ramosissima* Raf. – – wfo-0001304041
Statice caroliniana Walter – – wfo-0001246030 – http://www.biodiversitylibrary.org/openurl?pid=title:9458&volume=&issue=&spage=118&date=1788
Statice carpatha Rech.f. – – wfo-0000503606
Statice carpetana (Villar) Villar – – wfo-0000503605
Statice carthaginensis Rouy – – wfo-0000503661
Statice caryophyllacea Boiss. – – wfo-0001303052 – https://babel.hathitrust.org/cgi/pt?id=hvd.32044106337678&seq=660
Statice caryophyllacea Boiss. & Hohen. ex Boiss. – – wfo-0000503602 – https://babel.hathitrust.org/cgi/pt?id=hvd.32044106337678&seq=659
Statice caspia Pollini – – wfo-0000503643 – https://books.google.de/books?id=02z1yrHXatwC&pg=PP5&source=gbs_selected_pages&cad=3#v=onepage&q&f=false
Statice caspia var. *kimmerica* Lipsky – – wfo-0001304042
Statice caspia var. *patens* (Fisch. ex Boiss.) Boiss. – – wfo-0001304043 – https://biodiversitylibrary.org/page/160883
Statice caspia var. *urumiensis* Bornm. – – wfo-0001304044 – https://www.biodiversitylibrary.org/page/13744551
Statice caspia Willd. – – wfo-0000503692 – https://bibdigital.rjb.csic.es/viewer/10963/?offset=#page=343&viewer=picture&o=bookmark&n=0&q=
Statice castellana (Boiss. & Reut. ex Leresche) Rothm. – – wfo-0000503691
Statice catalaunica Willk. & Costa – – wfo-0000503690 – https://biodiversitylibrary.org/page/116606
Statice catanensis Janka – – wfo-0001304045
Statice catanensis Tineo ex Lojac. – – wfo-0000503689
Statice cephalotes Aiton – – wfo-0000503688 – https://biodiversitylibrary.org/page/3650141
Statice cephalotes Bertol. – – wfo-0000503687
Statice cephalotes Poir. – – wfo-0000503686 – https://biodiversitylibrary.org/page/724420

Statice cespitosa Poir. – – wfo-0001304046
Statice chazaliei H.Boissieu – – wfo-0000503685
Statice chilensis (Boiss.) Macloskie – – wfo-0000503683
Statice chilensis Phil. – – wfo-0000503693 – https://biodiversitylibrary.org/page/50421835
Statice chrysocephala Regel – – wfo-0001095283 – http://www.biodiversitylibrary.org/openurl?pid=title:14650&volume=6&issue=2&spage=383&date=1880
Statice chrysocoma Kar. & Kir. – – wfo-0001095338 – http://www.biodiversitylibrary.org/openurl?pid=title:4951&volume=15&issue=&spage=429&date=1842
Statice ciliata (Lange) Rothm. – – wfo-0000503682
Statice cinerea Poir. – – wfo-0000503681 – https://biodiversitylibrary.org/page/724433
Statice collina Griseb. – – wfo-0000503699
Statice collina Nyman – – wfo-0001304047 – https://biodiversitylibrary.org/page/11015757
Statice companyonis Gren. & Billot – – wfo-0001260266
Statice confusa Gren. & Godr. – – wfo-0000503701
Statice confusa Rchb.f. – – wfo-0001304048
Statice confusa var. *intermedia* Daveau – – wfo-0001304049
Statice congesta Ledeb. – – wfo-0001095330 – http://www.biodiversitylibrary.org/openurl?pid=title:6618&volume=1&issue=&spage=437&date=1829
Statice conspicua Sims – – wfo-0000503700
Statice contortiramea Mabille – – wfo-0001260256
Statice coralloides Tausch – – wfo-0001095353
Statice corculum Christ – – wfo-0000503694 – https://biodiversitylibrary.org/page/197898
Statice cordata Desf. – – wfo-0001304050 – https://biodiversitylibrary.org/page/305832
Statice cordata G.E.Sm. – – wfo-0001304051
Statice cordata Guss. – – wfo-0001304052
Statice cordata Hayek – – wfo-0001304053
Statice cordata L. – – wfo-0000503698 – https://biodiversitylibrary.org/page/358294
Statice cordata Poir. – – wfo-0001304054
Statice cordata var. *genuina* Boiss. – – wfo-0001304055 – https://biodiversitylibrary.org/page/160878
Statice cordata var. *maior* Boiss. – – wfo-0001304056
Statice coriacea hort. ex Schult. – – wfo-0000503697
Statice coriacifolia Sennen – – wfo-0000503696 – http://bibdigital.rjb.csic.es/ing/Libro.php?Libro=15&Pagina=99
Statice coriaria Pall. – – wfo-0000503695 – https://biodiversitylibrary.org/page/11268329
Statice coriaria Pall. ex M.Bieb. – – wfo-0001304057
Statice corinthiaca Boiss. & Heldr. – – wfo-0000503678 – http://bibdigital.rjb.csic.es/ing/Libro.php?Libro=1418&Pagina=71
Statice corymbulosa (Coss.) Nyman – – wfo-0000503653 – https://biodiversitylibrary.org/page/11015753
Statice corymbulosa Boiss. – – wfo-0000503684 – https://biodiversitylibrary.org/page/160880
Statice corymbulosa Coss. – – wfo-0001304058
Statice costae Willk. – – wfo-0000503660 – https://biodiversitylibrary.org/page/116605
Statice cosyrensis Guss. – – wfo-0000503659
Statice cosyrensis subsp. *cosyrensis* – – wfo-0001304059
Statice cosyrensis subsp. *melia* Nyman – – wfo-0000745480 – https://biodiversitylibrary.org/page/11015756
Statice crispa L. ex B.D.Jacks. – – wfo-0001304060 – https://www.biodiversitylibrary.org/page/36184648
Statice crispa Pers. – – wfo-0000503658 – https://biodiversitylibrary.org/page/235119
Statice crouanii Lenorm. ex Nyman – – wfo-0000503657 – https://biodiversitylibrary.org/page/11015753

Statice cumana Ten. – – wfo-0000503655
Statice cumana var. *glabrescens* Lacaita – – wfo-0001304061
Statice cumana var. *tenuicula* (Tineo ex Cuss.) Boiss. – – wfo-0001304062 – https://biodiversitylibrary.org/page/160879
Statice cuneata hort. ex Schult. – – wfo-0001304063
Statice cuneata Sm. ex Link – – wfo-0000503645 – https://biodiversitylibrary.org/page/53266257
Statice cuspidata Delort. – – wfo-0000503654
Statice cylindrica Steud. – – wfo-0001304064
Statice cylindrifolia Forssk. – – wfo-0000503652
Statice cymulifera Boiss. – – wfo-0000503651
Statice cymulifera subsp. *mauritii* Sennen – – wfo-0001304065 – http://bibdigital.rjb.csic.es/ing/Libro.php?Libro=42&Pagina=116
Statice cyrenaica Rouy – – wfo-0000503650 – https://biodiversitylibrary.org/page/14597794
Statice cyrtostachya Girard – – wfo-0000503649 – https://biodiversitylibrary.org/page/41531994
Statice czurjukiensis Klokov – – wfo-0001304066
Statice dalmatica C.Presl – – wfo-0000503648 – https://biodiversitylibrary.org/page/45617016
Statice davaei (Samp.) Samp. – – wfo-0000503647
Statice davurica Pall. – – wfo-0000503671
Statice decipiens Ledeb. – – wfo-0001095355 – http://www.biodiversitylibrary.org/openurl?pid=title:6618&volume=1&issue=&spage=433&date=1829
Statice decumbens Boiss. – – wfo-0001095178 – https://biodiversitylibrary.org/page/160881
Statice delicatula Bég. & Vacc. – – wfo-0001304067
Statice delicatula f. *pomeliana* Rouy – – wfo-0001304068 – https://www.biodiversitylibrary.org/page/14597794
Statice delicatula Girard – – wfo-0000503677 – https://biodiversitylibrary.org/page/41531994
Statice delicatula subsp. *raddiana* (Boiss.) Rouy – – wfo-0001304069 – https://www.biodiversitylibrary.org/page/14597794
Statice delicatula var. *ambigua* (Bonnet & Barratte) Rouy – – wfo-0001304070
Statice delicatula var. *minor* (Boiss.) Bég. & A.Vacc. – – wfo-0001304071
Statice delicatula var. *subrotundifolia* Bég. & A.Vacc. – – wfo-0001304072
Statice delicatula var. *tournefortii* (Girard) Boiss. – – wfo-0001304073 – https://biodiversitylibrary.org/page/160875
Statice delilei Aubouy ex Rouy – – wfo-0001304074
Statice densiflora Girard – – wfo-0001304075 – https://biodiversitylibrary.org/page/36219379
Statice densiflora Guss. – – wfo-0000503675 – https://www.biodiversitylibrary.org/page/42982949
Statice densiflora var. *humilis* Guss. – – wfo-0001304076 – https://www.biodiversitylibrary.org/page/42982950
Statice densiflora var. *lusitanica* Daveau – – wfo-0001304077
Statice densiflora var. *obtusifolia* Guss. – – wfo-0001304078 – https://www.biodiversitylibrary.org/page/42982950
Statice dentata hort. ex Schult. – – wfo-0000503674 – https://www.biodiversitylibrary.org/page/732556
Statice denticulata Bertol. – – wfo-0000503673
Statice denudata Regel & Körn. – – wfo-0000503672
Statice depressa Bubani – – wfo-0000503662 – https://www.biodiversitylibrary.org/page/6718528
Statice dertosensis Font Quer & Rothm. – – wfo-0000503679
Statice desertorum Trautv. – – wfo-0000503670
Statice dianiae Pau – – wfo-0001304079
Statice dianthifolia Jaub. & Spach – – wfo-0000503669 – https://babel.hathitrust.org/cgi/pt?id=mdp.39015061880194;view=1up;seq=180;size=125
Statice dianthodes Voss – – wfo-0000503668 – https://biodiversitylibrary.org/page/42525308
Statice dichotoma Cav. – – wfo-0000503811

Statice dichotoma Duby – – wfo-0000503831
Statice dichotoma Guss. – – wfo-0000504012 – https://biodiversitylibrary.org/page/42982954
Statice dichotoma Mutel, A. – – wfo-0000503665
Statice dichotoma Rchb. – – wfo-0000503664
Statice dichotoma Sm. – – wfo-0000503663
Statice dichotoma Willk. – – wfo-0000503666 – https://biodiversitylibrary.org/page/40337612
Statice dichroantha Ik.-Gal. – – wfo-0001304080
Statice dichroantha Rupr. – – wfo-0001095327 – http://www.biodiversitylibrary.org/openurl?pid=title:96968&volume=14&issue=4&spage=69&date=1869
Statice dickensonii Poit. – – wfo-0000503975 – https://www.biodiversitylibrary.org/page/49661854
Statice dickinsonii hort. Angl. ex F.Cels – – wfo-1000055928
Statice dicksoniana hort. ex W.H.Baxter – – wfo-0000503974
Statice dictyoclada Boiss. – – wfo-0000503972 – https://biodiversitylibrary.org/page/160876
Statice dictyoclada subsp. *calcarae* (Tod. ex Janka) Nyman – – wfo-0001304081 – https://biodiversitylibrary.org/page/11015755
Statice dictyoclada subsp. *pygmaea* (Tineo) Nyman – – wfo-0001304082 – https://biodiversitylibrary.org/page/11015755
Statice dictyoclada var. *dubia* (Andrews ex Guss) Boiss. – – wfo-0001304083
Statice dictyoclada var. *pygmaea* Boiss. – – wfo-0001304084 – https://biodiversitylibrary.org/page/160876
Statice dictyoclada var. *reticulata* Boiss. – – wfo-0001304085 – http://www.jstor.org/stable/3996512
Statice dictyophora Tausch – – wfo-0000503971
Statice diegoi Sennen – – wfo-0000503970
Statice dielsiana Wangerin – – wfo-0001095337 – http://www.biodiversitylibrary.org/openurl?pid=title:276&volume=17&issue=492-503&spage=399&date=1921
Statice diffusa Laterr. ex Boiss. – – wfo-0001304086 – https://biodiversitylibrary.org/page/160883
Statice diffusa Pourr. – – wfo-0000503977 – https://archive.org/stream/HistoireEtMemoiresToulouse3/Histoire_et_memoires_Toulouse_3#page/n407/mode/2up
Statice divaricata Cav. ex Schult. – – wfo-0000503968 – https://biodiversitylibrary.org/page/732604
Statice dodartii Girard – – wfo-0000503967 – https://biodiversitylibrary.org/page/36219373
Statice dodartii var. *humile* Girard – – wfo-0001304087 – https://www.biodiversitylibrary.org/page/36219371
Statice doerfleri Halácsy – – wfo-0000503966 – https://biodiversitylibrary.org/page/10085740
Statice donetzica Klokov – – wfo-0001304088
Statice doriae Sommier – – wfo-0000503965
Statice dregeana C.Presl – – wfo-0001095182 – https://biodiversitylibrary.org/page/45617016
Statice drepanense Tineo ex Guss. – – wfo-0001304089
Statice drepanensis Tineo ex Guss. – – wfo-0000503963
Statice drepanostachya Ikonn.-Gal. – – wfo-0001304090
Statice dschungarica Regel – – wfo-0001095292 – http://www.biodiversitylibrary.org/openurl?pid=title:14650&volume=6&issue=2&spage=386&date=1880
Statice dubia Andrews ex Guss. – – wfo-0000503969
Statice dubyeana Bubani – – wfo-0000503991
Statice dubyi Gren. & Godr. – – wfo-0000503989
Statice dufourii Girard – – wfo-0000503988 – https://biodiversitylibrary.org/page/36219338
Statice durieui Girard – – wfo-0000503987 – http://gallica.bnf.fr/ark:/12148/bpt6k209710c/f86.image
Statice duriuscula Girard – – wfo-0000503986 – https://biodiversitylibrary.org/page/41531994
Statice duriuscula subsp. *catalaunica* (Willk. & Costa) Nyman – – wfo-0001304091 – https://biodiversitylibrary.org/page/11015755
Statice duriuscula subsp. *duriuscula* – – wfo-0001304092

Statice duriuscula subsp. *laeta* Nyman – – wfo-0000745485
Statice duriuscula var. *catalaunica* Costa – – wfo-0001304093
Statice duriuscula var. *procera* Willk. – – wfo-0000748113
Statice duriuscula var. *valentina* Sennen & Pau – – wfo-0001304094
Statice ebracteata (Pomel) Maire – – wfo-0000503985
Statice ebracteata var. *laevis* (Maire) Maire – – wfo-0001304095
Statice ebusitana Font Quer – – wfo-0000503984
Statice echinata Schult. – – wfo-0000503993
Statice echinus L. – – wfo-0000503983 – https://www.biodiversitylibrary.org/page/358295
Statice echinus M.Bieb. – – wfo-0000503982
Statice echioides L. – – wfo-0000503981 – https://biodiversitylibrary.org/page/358294
Statice echioides subsp. *echioides* L. – – wfo-0001304096
Statice echioides subsp. *exaristata* (Murb.) Hayek – – wfo-0001304097
Statice echioides subsp. *exaristata* (Murb.) Pamp. – – wfo-0001304098
Statice echioides subsp. *exaristata* Murb. – – wfo-0001304099
Statice echioides var. *segobricensis* Pau – – wfo-0001304100
Statice effusa Boiss. – – wfo-0000503979 – https://biodiversitylibrary.org/page/160870
Statice elata Fisch. ex Spreng. – – wfo-0000503978 – https://biodiversitylibrary.org/page/6721023
Statice elegans Coss. ex Nyman – – wfo-0000503961 – https://biodiversitylibrary.org/page/11015757
Statice elongata Hoffm. – – wfo-0000503940
Statice emarginata Schur – – wfo-0001304101
Statice emarginata Willd. – – wfo-0000503947
Statice endlichiana Wangerin – – wfo-0000503946
Statice equisetina Boiss. – – wfo-0001095186 – https://biodiversitylibrary.org/page/160880
Statice equisetina var. *depauperata* Boiss. – – wfo-0001095180 – https://biodiversitylibrary.org/page/160880
Statice erinacea Boiss. – – wfo-0001304102
Statice erinacea Jaub. & Spach – – wfo-0000503944 – https://babel.hathitrust.org/cgi/pt?id=mdp.39015061880194;view=1up;seq=181;size=125
Statice eriophylla (Willk.) Samp. – – wfo-0000503943
Statice eugeniae Sennen – – wfo-0000503935 – http://bibdigital.rjb.csic.es/ing/Libro.php?Libro=15&Pagina=99
Statice exaristata Murb. – – wfo-0000503941
Statice exima var. *flore-alba* hort. ex F.T.Hubb. – – wfo-0001304103
Statice exima var. *superba* hort. ex F.T.Hubb. – – wfo-0001304104
Statice eximia Schrenk – – wfo-0001095297 – https://www.biodiversitylibrary.org/page/31312369
Statice eximia var. *turkestanica* Regel – – wfo-0001095299
Statice eximum var. *alba* hort. ex F.T.Hubb. – – wfo-0001304105
Statice fallax Coss. ex Wangerin – – wfo-0000503939
Statice fasciculata var. *pungens* Samp. – – wfo-0001304106
Statice fasciculata Vent. – – wfo-0000503938
Statice faustii Sennen & Mauricio – – wfo-0001304107 – http://bibdigital.rjb.csic.es/ing/Libro.php?Libro=42&Pagina=115
Statice ferganensis Ikonn.-Gal. – – wfo-0001304108
Statice ferox Jaub. & Spach – – wfo-0000503937 – https://hdl.handle.net/2027/mdp.39015061880194?urlappend=%3Bseq=181
Statice ferulacea L. – – wfo-0000503936 – http://www.biodiversitylibrary.org/openurl?pid=title:11179&volume=2&issue=&spage=396&date=1762

Statice festucacea Jaub. & Spach – – wfo-0000503955 – https://babel.hathitrust.org/cgi/pt?id=mdp.39015061880194;view=1up;seq=181;size=125
Statice filicaulis (Boiss.) Jahand. & Maire – – wfo-0001304109 – https://bibdigital.rjb.csic.es/viewer/13176/?offset=#page=18&viewer=picture&o=search&n=0&q=statice
Statice filicaulis Boiss. – – wfo-0000503956
Statice filicaulis var. *marizii* (Daveau) Samp. – – wfo-0001304110 – https://bibdigital.rjb.csic.es/viewer/9516/?offset=#page=102&viewer=picture&o=bookmark&n=0&q=
Statice filicaulis var. *maroccana* (Pau & Font Quer) Maire – – wfo-0001304111
Statice fischeri Trautv. – – wfo-0000503960
Statice flagellaris Lojac. – – wfo-0000503959
Statice flexuosa L. – – wfo-0000503958 – http://www.biodiversitylibrary.org/openurl?pid=title:669&volume=1&issue=&spage=276&date=1753
Statice flexuosa Less. – – wfo-0001304112 – https://biodiversitylibrary.org/page/1924
Statice flexuosa O.Fedtsch. & B.Fedtsch. – – wfo-0001304113
Statice flexuosa Sennen – – wfo-0001304114
Statice floribunda Lem. ex Huber – – wfo-0000503949
Statice florida Kitag. – – wfo-0001095305 – https://doi.org/10.15281/jplantres1887.48.91
Statice foliosa Penny ex W.H.Baxter – – wfo-0000503962
Statice fontqueri Pau – – wfo-0001260279 – https://biodiversitylibrary.org/page/30165841
Statice formosa hort. ex E.Vilm. – – wfo-0000503954
Statice fortunei Lindl. – – wfo-0001303032
Statice fortuni Lindl. – – wfo-0001095302 – https://www.biodiversitylibrary.org/page/243079
Statice fradiniana Pomel – – wfo-0000503953
Statice franchetii Debeaux – – wfo-0001095309 – http://www.biodiversitylibrary.org/openurl?pid=title:16235&volume=31&issue=&spage=348&date=1876
Statice fraterna Sennen & Pau – – wfo-0000503952
Statice frederici Barbey – – wfo-0000504002
Statice frondosa Lojac. – – wfo-0000504039
Statice frutescens Lem. – – wfo-0000503992
Statice fruticans Webb ex Boiss. – – wfo-0000504037 – https://biodiversitylibrary.org/page/160860
Statice fruticosa Lepech. – – wfo-0000504036
Statice furfuracea Lag. – – wfo-0000504035
Statice furfuracea Rchb. – – wfo-0001304115
Statice gabrieli Bornm. – – wfo-0000504034
Statice gaditana (Boiss.) Jahand. & Maire – – wfo-0000504026 – https://bibdigital.rjb.csic.es/viewer/13176/?offset=#page=17&viewer=picture&o=search&n=0&q=statice
Statice gaditana (Boiss.) Samp. – – wfo-0001304116
Statice gaditana var. *chamaeropicola* (Pau) Maire – – wfo-0001304117 – https://bibdigital.rjb.csic.es/viewer/13176/?offset=#page=19&viewer=picture&o=bookmark&n=0&q=
Statice gaditana var. *tingitana* (Boiss. & Reut.) Ball – – wfo-0001304118
Statice gaillardotii Lafont – – wfo-0000504031 – https://www.biodiversitylibrary.org/page/35747627
Statice gavilae Sennen & Mauricio – – wfo-0000504038 – http://bibdigital.rjb.csic.es/ing/Libro.php?Libro=42&Pagina=115
Statice genistoides Jaub. & Spach – – wfo-0000504030 – https://babel.hathitrust.org/cgi/pt?id=mdp.39015061880194;view=1up;seq=180;size=125
Statice giberti Sennen – – wfo-0001304119
Statice gibertii Sennen – – wfo-0000504029 – https://bibdigital.rjb.csic.es/viewer/9472/?offset=#page=272&viewer=picture&o=bookmark&n=0&q=
Statice gilesii Hemsl. – – wfo-0000504028 – https://biodiversitylibrary.org/page/16160007

Statice girardiana Guss. – – wfo-0000504027
Statice girardiana subsp. *costae* (Willk.) Nyman – – wfo-0001304120 – https://biodiversitylibrary.org/page/11015754
Statice girardiana subsp. *girardiana* – – wfo-0001304121
Statice glauca (Boiss.) Cuatrec. – – wfo-0001304122
Statice glauca Less. – – wfo-0001304123 – https://biodiversitylibrary.org/page/1924
Statice glauca Pers. – – wfo-0001304124
Statice glauca Willd. ex Schult. – – wfo-0001095358
Statice globulariaefolia Desf. – – wfo-0001304125
Statice globulariaefolia var. *major* Boiss. – – wfo-0001304126
Statice globulariaefolia var. *minor* Boiss. – – wfo-0001304127
Statice globulariifolia DC. – – wfo-0001304128 – https://www.biodiversitylibrary.org/page/50110593
Statice globulariifolia Desf. – – wfo-0000504050 – https://biodiversitylibrary.org/page/305833
Statice globulariifolia var. *glauca* Boiss. – – wfo-0001304129
Statice globulariifolia var. *major* Boiss. – – wfo-0001304130
Statice globulariifolia var. *minor* Boiss. – – wfo-0001304131 – https://bibdigital.rjb.csic.es/viewer/9792/?offset=#page=535
Statice globulariifolia Webb – – wfo-0001304132 – https://bibdigital.rjb.csic.es/viewer/9510/?offset=#page=27
Statice globulifera Boiss. & Heldr. ex Boiss. – – wfo-0000504048 – https://biodiversitylibrary.org/page/160887
Statice glomerata Tausch – – wfo-0000504047
Statice glumacea Jaub. & Spach – – wfo-0001095215 – https://babel.hathitrust.org/cgi/pt?id=mdp.39015061880194;view=1up;seq=180;size=125
Statice gmelini f. *steiroclada* Wangerin – – wfo-0001304133
Statice gmelini M.Bieb. – – wfo-0001304134
Statice gmelini Rchb. – – wfo-0001304135
Statice gmelini subsp. *genuina* Wangerin – – wfo-0001304136
Statice gmelini subsp. *scoparia* Wangerin – – wfo-0001304137
Statice gmelini var. *genuina* Boiss. – – wfo-0001304138
Statice gmelini var. *grandis* Popov ex Andross. – – wfo-0001304139
Statice gmelini var. *laxiflora* Boiss. – – wfo-0001304140 – https://biodiversitylibrary.org/page/160870
Statice gmelini var. *steiroclada* Trautv. – – wfo-0001304141
Statice gmelini var. *typica* Trautv. – – wfo-0001304142
Statice gmelini Willd. – – wfo-0000504045 – https://www.biodiversitylibrary.org/page/630841
Statice gmelinii subsp. *tomentella* (Boiss.) Wangerin – – wfo-0001304143
Statice gmelinii var. *laxiflora* Boiss. – – wfo-0001304144
Statice gmelinii var. *lilacina* (Boiss. & Balansa) Boiss. – – wfo-0001304145 – https://biodiversitylibrary.org/page/18115531
Statice gmelinii var. *limonioides* Wangerin – – wfo-0001304146
Statice gmelinii var. *scoparia* (Pall. ex Willd.) Schmalh. – – wfo-0001095361 – http://www.biodiversitylibrary.org/openurl?pid=title:825&volume=6&issue=&spage=799&date=1820
Statice gmelinii var. *scoparia* Trautv. – – wfo-0001304147
Statice gmelinii var. *tomentella* (Boiss.) Trautv. – – wfo-0001304148
Statice gmelinii W.D.J.Koch – – wfo-0001304149
Statice gmelinii Willd. – – wfo-0001303033
Statice gobica Ikonn.-Gal. – – wfo-0001304150
Statice gomezi-jordanae Sennen – – wfo-0000504044 – http://bibdigital.rjb.csic.es/ing/Libro.php?Libro=15&Pagina=166
Statice gougetiana Girard – – wfo-0000504043 – https://www.biodiversitylibrary.org/page/41531995

Statice gougetiana Reverch. – – wfo-0001304151
Statice gracilis Fisch. ex Boiss. – – wfo-0000504042 – https://biodiversitylibrary.org/page/160882
Statice gracilis O.Fedtch. & B.Fedtsch. – – wfo-0001304152
Statice gracilis Tineo – – wfo-0000504041
Statice graeca Boiss. – – wfo-0001304153 – https://biodiversitylibrary.org/page/160872
Statice graeca Poir. – – wfo-0000504046 – https://biodiversitylibrary.org/page/782267
Statice graeca var. *hyssopifolia* (Girard) Boiss. – – wfo-0001304154 – https://biodiversitylibrary.org/page/160872
Statice graeca var. *microphylla* Boiss. – – wfo-1200068050 – https://biodiversitylibrary.org/page/160872
Statice graeca var. *palmaris* (Sm.) Hay – – wfo-0001304155
Statice graeca var. *sieberi* (Boiss.) Boiss. – – wfo-1200068049 – https://biodiversitylibrary.org/page/160872
Statice graminifolia Aiton – – wfo-0000504009 – https://biodiversitylibrary.org/page/3650141
Statice graminifolia Besser – – wfo-0001304156 – https://biodiversitylibrary.org/page/6089433
Statice graminifolia var. *desertorum* Regel – – wfo-0001304157
Statice grandiflora hort. Viln. ex Schult. – – wfo-0000504008 – https://www.biodiversitylibrary.org/page/732534
Statice griffithii Aitch. & Hemsl. – – wfo-0000504007 – https://biodiversitylibrary.org/page/228998
Statice guaicuru (Molina) I.M.Johnst. – – wfo-0000504006
Statice guaicuru (Molina) Larrañaga – – wfo-0000504005
Statice gummifera Durieu ex Boiss. & Reut. – – wfo-0000504004
Statice gummifera f. *corymbulosa* (Coss.) Batt. – – wfo-0001304158
Statice gummifera subsp. *cymulifera* – – wfo-0001304159
Statice gummifera subsp. *cymulifera* (Boiss.) Batt. – – wfo-0001304160
Statice gummifera var. *corymbulosa* Coss. – – wfo-0001304161 – http://bibdigital.rjb.csic.es/ing/Libro.php?Libro=914&Pagina=175
Statice gummifera var. *cymulifera* (Boiss.) Batt. – – wfo-0001304162
Statice gussoneana Steud. – – wfo-0000504003
Statice gussonei Tineo ex Lojac. – – wfo-0000503994
Statice halfordii Hovey – – wfo-0001304163 – https://www.biodiversitylibrary.org/page/43999244
Statice halleri Garcke – – wfo-0000504011
Statice halochrysa Fisch. ex Boiss. – – wfo-0000504000 – https://biodiversitylibrary.org/page/160865
Statice heldreichii Halácsy – – wfo-0000503999 – http://www.biodiversitylibrary.org/openurl?pid=title:13275&volume=36&issue=&spage=241&date=1886
Statice hieronymi Sennen – – wfo-0000503998
Statice hirsuta C.Presl – – wfo-0000503997 – https://biodiversitylibrary.org/page/45617016
Statice hirta (Willd.) Rothm. – – wfo-0000503995
Statice hirta (Willd.) Steud. – – wfo-0000503996
Statice hirta subsp. *baetica* (Boiss.) Rothm. – – wfo-0001304164
Statice histrix Jaub. & Spach – – wfo-0000504015 – https://babel.hathitrust.org/cgi/pt?id=mdp.39015061880194&seq=178
Statice hoeltzeri Regel – – wfo-0001095369 – http://www.biodiversitylibrary.org/openurl?pid=title:14650&volume=5&issue=&spage=259&date=1877
Statice hohenackeri Jaub. & Spach – – wfo-0001042000 – https://babel.hathitrust.org/cgi/pt?id=mdp.39015061880194;view=1up;seq=180;size=125
Statice hohenackeri Ledeb. – – wfo-0001304166
Statice hohenackeri Ledeb. – – wfo-0001304165 – http://www.biodiversitylibrary.org/item/29508#page/493/mode/1up
Statice holfordii hort. – – wfo-0001304167
Statice horrida Girard – – wfo-0000504022 – https://biodiversitylibrary.org/page/41531998
Statice humboldtii Bolle – – wfo-0000504021 – https://biodiversitylibrary.org/page/44923888

Statice humilis (Mill.) C.E.Salmon – – wfo-0000504020
Statice humilis Link – – wfo-0001304168
Statice humilis var. *odorata* (Samp.) Samp. – – wfo-0001304169
Statice hybrida Mont. ex J.Lloyd – – wfo-0000504019
Statice hypanica Klokov – – wfo-0001304171
Statice hypanica Klokov – – wfo-0001304170
Statice hyssopifolia Girard – – wfo-0000504018 – https://biodiversitylibrary.org/page/41531996
Statice hystrix Jaub. & Spach – – wfo-0001304172 – https://babel.hathitrust.org/cgi/pt?id=mdp.39015061880194&seq=178
Statice iconia Boiss. & Heldr. – – wfo-0001041257 – https://www.biodiversitylibrary.org/page/160883
Statice ifniensis Caball. – – wfo-0000504017 – http://bibdigital.rjb.csic.es/ing/Libro.php?Libro=708&Pagina=9
Statice imbricata Webb ex Girard – – wfo-0000504024 – https://biodiversitylibrary.org/page/41531997
Statice inarimensis Guss. – – wfo-0000504014
Statice incana Becker – – wfo-0001304173
Statice incana d'Urv. ex Boiss. – – wfo-0001304174 – https://biodiversitylibrary.org/page/160894
Statice incana L. – – wfo-0000504013 – https://biodiversitylibrary.org/page/44260800
Statice incana Ledeb. – – wfo-0001304175
Statice incana M.Bieb. – – wfo-0001304176 – https://biodiversitylibrary.org/page/11268331
Statice incana var. *coccinea* hort. ex F.T.Hubb. – – wfo-0001304177
Statice incana var. *hybrida* hort. ex F.T.Hubb. – – wfo-0001304178
Statice incana var. *hybrida-nana* hort. ex F.T.Hubb. – – wfo-0001304179
Statice incana Vis. – – wfo-0001304180
Statice insigne var. *rossmaessleri* Willk. – – wfo-0001304181
Statice insignis Coss. – – wfo-0000503933
Statice interior Raup – – wfo-0001251977 – http://www.biodiversitylibrary.org/openurl?pid=title:480&volume=17&issue=4&spage=289&date=1936
Statice intermedia Guss. – – wfo-0000503948
Statice isernii (Vicioso & Beltrán) Font Quer & Rothm. – – wfo-0000503856
Statice jankae Lojac. – – wfo-0000503855
Statice japonica Siebold & Zucc. – – wfo-0000503853
Statice jaubertii Girard – – wfo-0000503852 – https://biodiversitylibrary.org/page/41531998
Statice jovibarba Webb ex Boiss. – – wfo-0000503851 – https://biodiversitylibrary.org/page/551967, https://biodiversitylibrary.org/page/160887
Statice juncea (Wallr.) F.T.Hubb. ex L.H.Bailey – – wfo-0001304182
Statice juncea (Wallr.) Samp. – – wfo-0000503858 – https://bibdigital.rjb.csic.es/viewer/9516/?offset=#page=102&viewer=picture&o=bookmark&n=0&q=
Statice juncea F.T.Hubb. – – wfo-0000503849 – https://biodiversitylibrary.org/page/568408
Statice juncea race eriophylla (Willk.) Samp. – – wfo-0001304183 – https://bibdigital.rjb.csic.es/viewer/9516/?offset=#page=102&viewer=picture&o=bookmark&n=0&q=
Statice juncea Tatarinow ex Wlangali – – wfo-0001304184
Statice juniperifolia – – wfo-0001304185
Statice juniperifolia Boiss. – – wfo-0000503845 – https://biodiversitylibrary.org/page/160902
Statice juniperifolia Pall. ex Steud. – – wfo-0000503847 – https://biodiversitylibrary.org/page/392710
Statice juniperifolia Samp. – – wfo-0001304186
Statice juniperifolia Vahl – – wfo-0000503846 – http://www.biodiversitylibrary.org/openurl?pid=title:57641&volume=&issue=&spage=25&date=1790
Statice juniperina Willd. ex Steud. – – wfo-0000503844

Statice karelinii Stschegl. – – wfo-0000503842
Statice kaschgarica Rupr. – – wfo-0001095332 – http://www.biodiversitylibrary.org/openurl?pid=title:96968&volume=14&issue=4&spage=69&date=1869
Statice kaufmanniana Regel – – wfo-0001095289 – http://www.biodiversitylibrary.org/openurl?pid=title:14650&volume=6&issue=2&spage=300&date=1880
Statice klementzii Ikonn.-Gal. – – wfo-0001304187
Statice kossmatii R.Wagner & Vierh. – – wfo-0000503850
Statice kotschyi Jaub. & Spach – – wfo-0000503869 – https://babel.hathitrust.org/cgi/pt?id=mdp.39015061880194;view=1up;seq=180;size=125
Statice kraussiana Buchinger ex Boiss. – – wfo-0001095188 – https://biodiversitylibrary.org/page/40078
Statice L. – – wfo-4000036306 – https://biodiversitylibrary.org/page/358293
Statice labradorica (Wallr.) F.T.Hubb. & S.F.Blake – – wfo-0001244933 – http://www.biodiversitylibrary.org/openurl?pid=title:721&volume=19&issue=217&spage=6&date=1917
Statice labradorica f. *glabriscapa* S.F.Blake – – wfo-0001244935
Statice labradorica f. *pubiscapa* S.F.Blake – – wfo-0001244936
Statice labradorica var. *submutica* S.F.Blake – – wfo-0001244934 – http://www.biodiversitylibrary.org/openurl?pid=title:721&volume=19&issue=217&spage=7&date=1917
Statice lacaitae Villar – – wfo-0001251976
Statice lachnolepis (Pomel) Maire – – wfo-0000503868
Statice lacostei Danguy – – wfo-0001095333
Statice laeta Ball – – wfo-0001304188 – https://biodiversitylibrary.org/page/8955108
Statice laeta Moris – – wfo-0000503872
Statice laeta Salisb. – – wfo-0001304189 – https://www.biodiversitylibrary.org/page/1485147
Statice laeta subsp. *insularis* Bég. & Landi – – wfo-0001260273
Statice lanceolata Edgew. – – wfo-0000503863 – https://biodiversitylibrary.org/page/40126797
Statice lanceolata Hoffmanns. & Link – – wfo-0000503867
Statice lanceolata hort. ex E.Vilm. – – wfo-0001304190 – https://gallica.bnf.fr/ark:/12148/bpt6k30755973/f1117.item
Statice lanceolata Rchb. – – wfo-0000503866 – http://bibdigital.rjb.csic.es/ing/Libro.php?Libro=1069&Pagina=13
Statice laneolata Hoffmanns. & Link – – wfo-0000503862
Statice langeana (Henriq.) Rothm. – – wfo-0000503861
Statice langei (Boiss.) Rothm. – – wfo-0000503860
Statice latifolia Sm. – – wfo-0000503859
Statice latifolia var. *alba* hort. ex F.T.Hubb. – – wfo-0001304191
Statice latissima Kar. & Kir. – – wfo-0001095357 – http://www.biodiversitylibrary.org/openurl?pid=item:128700&spage=729&date=1841
Statice laxiflora (Boiss.) Novopokr. – – wfo-0000503820
Statice laxissima Rouy – – wfo-0000503848
Statice lefroyi Hemsl. – – wfo-0000503826
Statice legrandii Gaut. & Timb.-Lagr. – – wfo-0000503825
Statice leonis Sennen – – wfo-0000503824
Statice lepidorachis Pomel – – wfo-0000503823 – https://bibdigital.rjb.csic.es/viewer/10313/?offset=#page=134&viewer=picture&o=info&n=0&q=
Statice leptoloba Michelson – – wfo-0001304192
Statice leptoloba Regel – – wfo-0001095328 – http://www.biodiversitylibrary.org/openurl?pid=title:14650&volume=6&issue=2&spage=385&date=1880
Statice leptoloba var. *subaphylla* Regel – – wfo-0001095329
Statice leptophylla Schrenk – – wfo-0000503813
Statice leptophylla var. *iranica* Bornm. – – wfo-0001304193 – https://biodiversitylibrary.org/page/3884697

Statice leptostachya Boiss. – – wfo-0000503822 – https://hdl.handle.net/2027/hvd.32044106337678?urlappend=%3Bseq=646, https://biodiversitylibrary.org/page/34838820
Statice leptostachya f. *pomeliana* Rouy – – wfo-0001304194
Statice leptostachya var. *scapis-filiformibus* Aitch. – – wfo-0001304195 – https://biodiversitylibrary.org/page/2925514
Statice leptostachys Pomel – – wfo-0000503828
Statice lepturoides Jaub. & Spach – – wfo-0000503819 – https://babel.hathitrust.org/cgi/pt?id=mdp.39015061880194;view=1up;seq=181;size=125
Statice lespinassi Lafont – – wfo-0000503818 – https://www.biodiversitylibrary.org/page/35747625
Statice letourneuxii Coss. ex Batt. – – wfo-0000503817
Statice leucacantha Jaub. & Spach – – wfo-0000503816 – https://babel.hathitrust.org/cgi/pt?id=mdp.39015061880194;view=1up;seq=182;size=125
Statice leucantha Pouzolz – – wfo-0000503815 – https://biodiversitylibrary.org/page/44509976
Statice leucocephala (Salzm. ex W.D.J.Koch) F.T.Hubb. – – wfo-0000503814 – https://biodiversitylibrary.org/page/568408
Statice leucocoleum Stapf & Wettst. ex Stapf – – wfo-0000503835
Statice lilacina Boiss. & Balansa – – wfo-0000503837 – http://bibdigital.rjb.csic.es/ing/Libro.php?Libro=1418&Pagina=70
Statice limbata (Small) K.Schum. – – wfo-0001273549 – https://biodiversitylibrary.org/page/5728303
Statice limonia St.-Lag. – – wfo-0000503840
Statice limonioides Bernh. ex Link. – – wfo-0001304196
Statice limonium Bigelow – – wfo-0001304197
Statice limonium L. – – wfo-0000503839 – http://www.biodiversitylibrary.org/openurl?pid=title:669&volume=1&issue=&spage=274&date=1753
Statice limonium Pall. – – wfo-0001304198
Statice limonium Rattan – – wfo-0001304199
Statice limonium subsp. *aggregata* (Rouy) Rouy – – wfo-0001304200
Statice limonium subsp. *angustifolia* (Tausch) Rouy – – wfo-0001304201
Statice limonium subsp. *bahusiensis* (Fr.) Hook.f. – – wfo-0001304202 – https://www.biodiversitylibrary.org/page/17930552
Statice limonium subsp. *bahusiensis* Syme – – wfo-0001304203
Statice limonium subsp. *behen* Syme – – wfo-0001304204
Statice limonium subsp. *rariflora* (Drejer) Hook.f. – – wfo-0001304205
Statice limonium subsp. *remotiflora* (Rouy) Rouy – – wfo-0001304206 – https://www.biodiversitylibrary.org/page/14597828, https://biodiversitylibrary.org/page/12880587
Statice limonium subsp. *serotina* (Rchb.) Nyman – – wfo-0001304207 – https://biodiversitylibrary.org/page/11015753
Statice limonium Thunb. – – wfo-0001304208 – https://biodiversitylibrary.org/page/50709895
Statice limonium var. *alba* hort. ex F.T.Hubb. – – wfo-0001304209
Statice limonium var. *behen* Rouy – – wfo-0001304210
Statice limonium var. *bellidifolia* Gouan – – wfo-0000503618 – https://www.biodiversitylibrary.org/page/6085130
Statice limonium var. *californica* (Boiss.) A.Gray – – wfo-0001262697 – https://biodiversitylibrary.org/page/32611630
Statice limonium var. *carolinianum* (Walter) A.Gray – – wfo-0001246031 – http://www.biodiversitylibrary.org/openurl?pid=title:50405&volume=&issue=&spage=270&date=1856
Statice limonium var. *genuina* Boiss. – – wfo-0001304211
Statice limonium var. *longidentata* (Lafont) Rouy – – wfo-0001304212
Statice limonium var. *macroclada* Boiss. – – wfo-0001304213 – https://biodiversitylibrary.org/page/160869
Statice limonium var. *minor* Roth – – wfo-0001304214
Statice limonium var. *pseudolimonium* Rouy – – wfo-0001304215
Statice limonium var. *puberula* Regel – – wfo-0001304216
Statice limonium var. *scanica* Fries – – wfo-0001304217

Statice limonium var. *typica* Rouy – – wfo-0001304218
Statice limonoides Biv. ex Link – – wfo-0000503830
Statice linearifolia Laterr. – – wfo-0000503841
Statice linearifolia Loisel. – – wfo-0000503836
Statice lingua Pomel – – wfo-0000503834
Statice linifolia Drege ex Boiss. – – wfo-0001304219 – https://biodiversitylibrary.org/page/160880
Statice linifolia L.f. – – wfo-0001095151 – http://www.biodiversitylibrary.org/openurl?pid=title:555&volume=&issue=&spage=187&date=1781
Statice linifolia var. *aretiifolia* Boiss. – – wfo-0001304220 – https://biodiversitylibrary.org/page/160879
Statice linifolia var. *brachyphylla* Boiss. – – wfo-0001304221 – https://biodiversitylibrary.org/page/160879
Statice linifolia var. *maritima* Eckl. & Zeyh. ex Boiss. – – wfo-0001095191 – https://biodiversitylibrary.org/page/160879
Statice linifolia var. *robusta* C.H.Wright – – wfo-0001304222 – https://www.biodiversitylibrary.org/page/713908
Statice littoralis (Willd.) Steud. – – wfo-0001304223
Statice littoralis subsp. *littoralis* (Willd.) Steud. – – wfo-0001304224
Statice littoralis P.Fourn. – – wfo-0001304225
Statice littoralis Poir. – – wfo-0000503833 – https://biodiversitylibrary.org/page/782264
Statice littoralis subsp. *filicaulis* (Boiss.) P.Fourn. – – wfo-0001304226
Statice littoralis subsp. *juncea* P.Fourn. – – wfo-0001304227
Statice littoralis var. *davaei* Samp. – – wfo-0001304228
Statice lobata L.f. – – wfo-0000503832 – https://biodiversitylibrary.org/page/411220
Statice longearistata Font Quer & Rothm. – – wfo-0001095205
Statice longearistata var. *cuspidata* Faure & Maire – – wfo-0001304229
Statice longiaristata (Boiss. & Reut.) Maire – – wfo-0000503827
Statice longidentata Lafont – – wfo-0000503871 – https://www.biodiversitylibrary.org/page/35747625
Statice longifolia Thunb. – – wfo-0001095193 – https://biodiversitylibrary.org/page/3553174
Statice lusitanica Poir. – – wfo-0000503918
Statice lychnidifolia Girard – – wfo-0000503917 – https://biodiversitylibrary.org/page/36219386
Statice lychnidifolia Porta – – wfo-0001304230
Statice lychnidifolia var. *corymbosa* Boiss. – – wfo-0001304231
Statice lycopodioides Girard – – wfo-0001095288 – https://biodiversitylibrary.org/page/41531997
Statice lyrata M.Bieb. – – wfo-0000503915
Statice macloviana (Cham.) Macloskie – – wfo-0000503914
Statice macphersonii F.Muell. – – wfo-0000503906
Statice macrophylla (Boiss. & Reut.) Font Quer & Rothm. – – wfo-0000503911
Statice macrophylla (Boiss. & Reut.) Rothm. – – wfo-0000503919
Statice macrophylla var. *atrocoerulea* J.R.Duncan & V.C.Davies – – wfo-0001304232
Statice macrophylla var. *sinuata* Boiss. – – wfo-0001304233 – https://biodiversitylibrary.org/page/160861
Statice macrophylla Willd. ex Spreng. – – wfo-0000503912 – https://biodiversitylibrary.org/page/6721025
Statice macroptera Webb & Berthel. – – wfo-0000503910 – https://biodiversitylibrary.org/page/41422878
Statice macrorrhabdos Boiss. – – wfo-0001038946 – http://bibdigital.rjb.csic.es/ing/Libro.php?Libro=1418&Pagina=69
Statice macrorrhabdos var. *thomsoni* C.B.Clarke – – wfo-0001304234
Statice macrorrhabdos var. *thomsonii* C.B.Clarke – – wfo-1200029706
Statice macrorrhiza Ledeb. – – wfo-0000503909
Statice maeotica Klokov – – wfo-0001304235

Statice majellensis (Boiss.) F.T.Hubb. – – wfo-0000503907 – https://biodiversitylibrary.org/page/568408
Statice majellensis subsp. *eumajellensis* P.Fourn. – – wfo-0001304236
Statice majellensis subsp. *majellensis* – – wfo-0001304237
Statice majellensis subsp. *malinvaudii* (H. J. Coste & Soulié) P.Fourn. – – wfo-0001304238
Statice major Garsault – – wfo-0000503926
Statice major race gaditana (Boiss.) Samp. – – wfo-0001304239 – https://bibdigital.rjb.csic.es/viewer/9516/?offset=#page=102&viewer=picture&o=bookmark&n=0&q=
Statice major Samp. – – wfo-0001304240 – https://bibdigital.rjb.csic.es/viewer/9516/?offset=#page=102&viewer=picture&o=bookmark&n=0&q=
Statice malinvaudii (H.J.Coste & Soulié) P.Fourn. – – wfo-0000503913
Statice manricarum Bolle – – wfo-0001304241 – https://www.biodiversitylibrary.org/page/42973213
Statice manricarum Bolle ex Linding. – – wfo-0001304242 – https://bibdigital.rjb.csic.es/viewer/15616/?offset=#page=271&viewer=picture&o=bookmark&n=0&q=
Statice manricarum Bolle ex Pitard & Proust – – wfo-0001304243
Statice manriqueorum Bolle ex Linding. – – wfo-0001304244 – https://bibdigital.rjb.csic.es/viewer/15616/?offset=#page=271&viewer=picture&o=bookmark&n=0&q=
Statice manriquorum Bolle ex Pitard & Proust – – wfo-0001304245 – https://bibdigital.rjb.csic.es/viewer/12453/?offset=#page=323&viewer=picture&o=bookmark&n=0&q=
Statice maravignae Tineo ex Lojac. – – wfo-0000503932
Statice marginata Schott ex Steud. – – wfo-0000503930
Statice maritima Lam. – – wfo-0001304246
Statice maritima Mill. – – wfo-0000503929 – https://biodiversitylibrary.org/page/395592
Statice maritima var. *elongata* (Hoffm.) Samp. – – wfo-0001304247 – https://bibdigital.rjb.csic.es/viewer/9516/?offset=#page=101&viewer=picture&o=bookmark&n=0&q=
Statice maritima var. *longeana* (Henriq.) Samp. – – wfo-0001304248 – https://bibdigital.rjb.csic.es/viewer/9516/?offset=#page=101&viewer=picture&o=bookmark&n=0&q=
Statice maritima var. *majuscula* Samp. – – wfo-0001304249
Statice maritima var. *pubigera* (Desf.) Samp. – – wfo-0001304250
Statice mauritanica (Wallr.) F.T.Hubb. – – wfo-0000503928 – https://biodiversitylibrary.org/page/568408
Statice mauritanica var. *safiensis* Maire – – wfo-0001304251 – https://bibdigital.rjb.csic.es/viewer/13176/?offset=#page=19&viewer=picture&o=bookmark&n=0&q=
Statice mauritanica var. *soloitana* Maire – – wfo-0001304252
Statice maurocenia hort. Par. ex Dum.Cours. – – wfo-0000503920
Statice maurocordatae Schweinf. & Volkens – – wfo-0000503927
Statice melanantha Boiss. – – wfo-0001304253 – https://biodiversitylibrary.org/page/34838820
Statice melia Nyman – – wfo-0000503934 – https://biodiversitylibrary.org/page/11015756
Statice membranacea Czern. ex Trautv. – – wfo-0000503925
Statice meyeri Boiss. – – wfo-0000503924 – https://biodiversitylibrary.org/page/160869
Statice micrantha Lafont – – wfo-0000503923 – https://www.biodiversitylibrary.org/page/35747628
Statice minuta Desf. – – wfo-0001304254 – https://biodiversitylibrary.org/page/305834
Statice minuta f. *puberula* C.E.Salmon – – wfo-0001304255
Statice minuta Falk – – wfo-0001304256
Statice minuta L. – – wfo-0000503921 – https://biodiversitylibrary.org/page/44260800
Statice minuta Moritzi ex Rchb. – – wfo-0001304257
Statice minuta Ten. ex Nyman – – wfo-0001304258 – https://biodiversitylibrary.org/page/11015754
Statice minuta Tomm. ex Nyman – – wfo-0001304259 – https://biodiversitylibrary.org/page/11015756
Statice minuta var. *acutifolia* Boiss. – – wfo-0001304260 – https://biodiversitylibrary.org/page/160877
Statice minuta var. *calcarae* (Tod. ex Janka) Fiori – – wfo-0001304261

Statice minuta var. *cancellata* (Bernh. ex Bertol.) Fiori – – wfo-0001304262
Statice minuta var. *cumana* (Ten.) Fiori – – wfo-0001304263
Statice minuta var. *dissitiflora* Boiss. – – wfo-0001260262 – https://biodiversitylibrary.org/page/160877
Statice minuta var. *inarimensis* (Guss.) Fiori – – wfo-0001304264
Statice minuta var. *microphylla* Boiss. – – wfo-0001304265 – https://biodiversitylibrary.org/page/160877
Statice minuta var. *multiformis* Martelli – – wfo-0000745476
Statice minuta var. *pubescens* Boiss. – – wfo-0001304266 – https://biodiversitylibrary.org/page/160877
Statice minuta var. *remotispicula* (Lacaita) Fiori – – wfo-0001304267
Statice minuta var. *sommierana* Fiori – – wfo-0001304268
Statice minuta var. *sommieriana* Fiori – – wfo-0001304269
Statice minuta Vis. – – wfo-0001304270
Statice minuta Willk. – – wfo-0001304271
Statice minutiflora Guss. – – wfo-0000503885
Statice minutiflora subsp. *minutiflora* – – wfo-0001304272
Statice minutiflora subsp. *panormitana* (Tod.) Nyman – – wfo-0001304273 – https://biodiversitylibrary.org/page/11015755
Statice molesii Sennen – – wfo-0000503884 – http://bibdigital.rjb.csic.es/ing/Libro.php?Libro=15&Pagina=165
Statice mongolica Fisch. ex Girard – – wfo-0000503883 – https://biodiversitylibrary.org/page/41531996
Statice monopetala L. – – wfo-0000503873
Statice monrealensis Pau – – wfo-0000503881
Statice monregalensis Pau – – wfo-0001304274
Statice montana Mill. – – wfo-0000503886 – https://biodiversitylibrary.org/page/395592
Statice montana Soleirol ex Boiss. – – wfo-0000503879 – https://biodiversitylibrary.org/page/160908
Statice montana var. *alba* F.T.Hubb. – – wfo-0001304275
Statice mouretii Pit. – – wfo-0000503878
Statice mucosa Salzm. ex Boiss. – – wfo-0000503877
Statice mucronata L.f. – – wfo-0000503876 – https://biodiversitylibrary.org/page/411220
Statice mucronulata H.Lindb. – – wfo-0000503875
Statice muelleri (A.Huet) P.Fourn. – – wfo-0000503874
Statice multiceps Pomel – – wfo-0000503896
Statice multiramea Sennen – – wfo-0001303034 – https://bibdigital.rjb.csic.es/viewer/9472/?offset=#page=98
Statice myriantha Schrenk ex Fisch. & C.A.Mey. – – wfo-0001095356 – http://www.biodiversitylibrary.org/openurl?pid=title:45100&volume=1&issue=&spage=14&date=1841
Statice nana Penny ex G.Don – – wfo-0000503904 – https://biodiversitylibrary.org/page/46026193
Statice nashii (Small) Wangerin – – wfo-0000503903
Statice nebrodensis Guss. – – wfo-0001304276 – https://www.biodiversitylibrary.org/page/42982948
Statice nebrodensis var. *duriaei* (Boiss.) Samp. – – wfo-0001304277
Statice neglecta (Girard) Samp. – – wfo-0000503901
Statice neglecta Andrz. – – wfo-0000503902
Statice nicotrae Lojac. – – wfo-0000503887
Statice nigricans Lafont – – wfo-0000503897 – https://www.biodiversitylibrary.org/page/35747624
Statice nogalensis Chiov. – – wfo-0000503905
Statice nuda Boiss. & Buhse – – wfo-0000503895 – https://biodiversitylibrary.org/page/11820162
Statice nuda Grossh. – – wfo-0001304278
Statice oblongifolia Kotov – – wfo-0000503894

Statice obovata Ledeb. – – wfo-0000503893 – https://biodiversitylibrary.org/page/6120224
Statice obtusa Dum.Cours. – – wfo-0000503892
Statice occidentalis J.Lloyd – – wfo-0000503891
Statice ochrantha Kar. & Kir. – – wfo-0000503890 – http://www.biodiversitylibrary.org/openurl?pid=item:128700&spage=730&date=1841
Statice ocimifolia Poir. – – wfo-0001304279
Statice ocimifolia Schult. – – wfo-0000503889 – https://biodiversitylibrary.org/page/732537
Statice ocymifolia Poir. – – wfo-0000503523 – https://biodiversitylibrary.org/page/782268
Statice ocymifolia subsp. *ocymifolia* – – wfo-0001304280
Statice ocymifolia var. *bellidifolia* (Sibth. & Sm.) Boiss. – – wfo-0001304281 – https://biodiversitylibrary.org/page/18115533
Statice oleifolia (Mill.) Scop. – – wfo-0000503322
Statice oleifolia Host – – wfo-0001304282 – https://www.biodiversitylibrary.org/page/52522365
Statice oleifolia Pourr. ex DC. – – wfo-0001304283 – https://biodiversitylibrary.org/page/49713140
Statice oleifolia Sm. – – wfo-0001304284 – https://biodiversitylibrary.org/page/6114642
Statice oleifolia var. *densiflora* (Guss.) Fiori – – wfo-0001304285
Statice oleifolia var. *fradiniana* (Pomel) Batt. – – wfo-0001304286 – https://bibdigital.rjb.csic.es/viewer/10763/?offset=#page=743&viewer=picture&o=bookmark&n=0&q=
Statice oleifolia Willd. – – wfo-0001304287 – https://biodiversitylibrary.org/page/630842
Statice oligantha Boiss. – – wfo-0001304288 – https://www.biodiversitylibrary.org/page/34838823
Statice oliveriana Andrz. ex Boiss. – – wfo-0000503321 – https://biodiversitylibrary.org/page/160872
Statice olivieri Jaub. & Spach – – wfo-0000503319 – https://babel.hathitrust.org/cgi/pt?id=mdp.39015061880194;view=1up;seq=181;size=125
Statice opulenta Lojac. – – wfo-0000503310
Statice ornata Ball – – wfo-0000503317
Statice otolepis Schrenk – – wfo-0001095352
Statice ovalifolia Poir. – – wfo-0000503315 – https://biodiversitylibrary.org/page/782267
Statice ovalifolia var. *baleariea* Wanger – – wfo-0001304289 – https://biodiversitylibrary.org/page/181582
Statice ovalifolia var. *lanceolata* Rouy – – wfo-0001304290
Statice ovalifolia var. *major* Rouy – – wfo-0001304291 – https://www.biodiversitylibrary.org/page/12880584
Statice ovalifolia var. *minor* Boiss. – – wfo-0001304292
Statice ovalifolia var. *nana* Rouy – – wfo-0001304293 – https://www.biodiversitylibrary.org/page/12880584
Statice ovalifolia var. *normalis* Rouy – – wfo-0001304294 – https://www.biodiversitylibrary.org/page/12880584
Statice ovalifolia var. *paniculata* Rouy – – wfo-0001304295 – https://www.biodiversitylibrary.org/page/12880584
Statice ovalifolia var. *pyramidata* (Lowe) Menezes – – wfo-0001304296
Statice owerinii Boiss. – – wfo-0001038947 – http://www.biodiversitylibrary.org/openurl?pid=title:20323&volume=4&issue=2&spage=870&date=1879
Statice oxylepis Boiss. – – wfo-0000503314
Statice palmaris Sm. – – wfo-0000503313
Statice palmyrensis Post – – wfo-0000503312 – https://www.biodiversitylibrary.org/page/18085595
Statice panormitana Tod. – – wfo-0000503331
Statice papillata Webb & Berthel. – – wfo-0000503332 – https://biodiversitylibrary.org/page/41422863
Statice parvifolia Tineo – – wfo-0000503318
Statice patagonica Speg. – – wfo-0001095270
Statice patens Fisch. ex Boiss. – – wfo-0000503338 – https://biodiversitylibrary.org/page/160883
Statice pauciflora Jaub. & Spach – – wfo-0000503337
Statice paui Cámara & Sennen – – wfo-0000503335 –

https://bibdigital.rjb.csic.es/viewer/9472/?offset=#page=270&viewer=picture&o=bookmark&n=0&q=
Statice paui Vicioso – – wfo-0001304297
Statice paulayana Vierh. – – wfo-0000503334
Statice pectinata Aiton – – wfo-0000503333 – https://biodiversitylibrary.org/page/3650142
Statice pectinata var. *corculum* Webb & Berthel. – – wfo-0001304298 – https://biodiversitylibrary.org/page/41422863
Statice pectinata var. *divaricata* Pit. – – wfo-0001304299 – https://bibdigital.rjb.csic.es/viewer/12453/?offset=#page=323&viewer=picture&o=bookmark&n=0&q=
Statice pectinata var. *incompta* Webb & Berthel. – – wfo-0001304300 – https://biodiversitylibrary.org/page/41422863
Statice pectinata var. *solandri* Webb & Berthel. – – wfo-0001304301 – https://biodiversitylibrary.org/page/41422863
Statice pedicellata Wallr. ex Boiss. – – wfo-0000503324 – https://biodiversitylibrary.org/page/160880
Statice peregrina P.J.Bergius – – wfo-0001095196
Statice perezii Stapf – – wfo-0001268689 – https://doi.org/10.1093/oxfordjournals.aob.a089156
Statice perfoliata C.A.Mey. ex Boiss. – – wfo-0001268623 – http://www.biodiversitylibrary.org/openurl?pid=title:286&volume=12&issue=&spage=663&date=
Statice perfoliata var. *reniformis* Boiss. – – wfo-0001304302 – https://biodiversitylibrary.org/page/160885
Statice petteriana (C.Presl) Degen – – wfo-0000503330
Statice pigadiensis Rech.f. – – wfo-0000503329
Statice pinifolia Brot. – – wfo-0000503328 – https://biodiversitylibrary.org/page/31323712
Statice pinifolia var. *capillifolia* Samp. – – wfo-0001304303
Statice planifolia (Syme) Druce – – wfo-0000503325
Statice plantaginea All. – – wfo-0000503283
Statice plantaginea Guss. – – wfo-0001304304
Statice plantaginea proles rigida (Wallr.) Samp. – – wfo-0001304305 – https://bibdigital.rjb.csic.es/viewer/9516/?offset=#page=102&viewer=picture&o=bookmark&n=0&q=
Statice plantaginea subsp. *choulettiana* (Pomel) Maire – – wfo-0001304306
Statice plantaginea subsp. *leucantha* (Boiss.) Maire – – wfo-0001304307
Statice plantaginea subsp. *medians* Maire – – wfo-0001304308
Statice plantaginea var. *alba* F.T.Hubb. – – wfo-0001304309
Statice plantaginea var. *atlantica* (Pomel) Maire – – wfo-0001304310
Statice plantaginea var. *barbata* Maire – – wfo-0001304311
Statice plantaginea var. *brachylepis* (Batt.) Maire – – wfo-0001304312
Statice plantaginea var. *cuspidata* (Faure & Maire) Maire – – wfo-0001304313
Statice plantaginea var. *djurdjurae* Maire – – wfo-0001304314
Statice plantaginea var. *gigantea* F.T.Hubb. – – wfo-0001304315
Statice plantaginea var. *grandiflora* F.T.Hubb. – – wfo-0001304316
Statice plantaginea var. *leucantha* (Boiss.) F.T.Hubb. – – wfo-0001304317
Statice plantaginea var. *masguindalii* (Pau) Maire – – wfo-0001304318
Statice plantaginea var. *microcephala* Maire – – wfo-0001304319
Statice plantaginea var. *minor* Gaudin – – wfo-0001304320
Statice plantaginea var. *rosea* F.T.Hubb. – – wfo-0001304321
Statice plantaginea var. *rubra* F.T.Hubb. – – wfo-0001304322
Statice plantaginea var. *splendens* F.T.Hubb. – – wfo-0001304323
Statice plantaginea var. *subcuspidata* Maire – – wfo-0001304324
Statice plantaginea var. *zaianica* (Emberger, L. & Maire) Maire – – wfo-0001304325
Statice plantaginiflora Jaub. & Spach – – wfo-0000503316 – https://babel.hathitrust.org/cgi/pt?id=mdp.39015061880194;view=1up;seq=178;size=125

Statice plumosa Phil. – – wfo-0001261788
Statice polianthemum Neck. – – wfo-0000503288
Statice pomeliana Rouy – – wfo-0000503287 – https://biodiversitylibrary.org/page/14597794
Statice potaninii Ikonn.-Gal. – – wfo-0001304326
Statice preauxii Webb & Berthel. – – wfo-0000503286 – https://biodiversitylibrary.org/page/41422877
Statice preciosa Tchertkoff – – wfo-0000503278 – https://biodiversitylibrary.org/page/45989151
Statice profusa hort. – – wfo-0001304327
Statice prolifera d'Urv. – – wfo-0000503284
Statice pruinosa Cav. ex Willk. & Lange – – wfo-0001304328 – https://biodiversitylibrary.org/page/26145028
Statice pruinosa Forssk. ex Schult. – – wfo-0000503290 – https://biodiversitylibrary.org/page/732551
Statice pruinosa L. – – wfo-0000503280 – https://biodiversitylibrary.org/page/44260800
Statice pruinosa var. *hirtiflora* Cavara & Grande – – wfo-0001304329
Statice pruinosa Webb & Berthel. ex Boiss. – – wfo-0000503281 – https://biodiversitylibrary.org/page/160864
Statice pseudarmeria Murray – – wfo-0000503279 – https://biodiversitylibrary.org/page/1479752
Statice pseud-armeria Murray – – wfo-0001304330
Statice pseudoarmeria Brot. – – wfo-0001304331 – https://biodiversitylibrary.org/page/31323713
Statice pseudoarmeria Cav. – – wfo-0001304332
Statice pseudoarmeria var. *alba* F.T.Hubb. – – wfo-0001304333 – https://biodiversitylibrary.org/page/568409
Statice pseudoarmeria var. *grandiflora* F.T.Hubb. – – wfo-0001304334 – https://biodiversitylibrary.org/page/568409
Statice pseudoarmeria var. *rubra* F.T.Hubb. – – wfo-0001304335 – https://biodiversitylibrary.org/page/568409
Statice pseudoarmeria var. *splendens* (hort.) F.T.Hubb. – – wfo-0001304336 – https://biodiversitylibrary.org/page/568409
Statice pseudolimonium Rchb. – – wfo-0000503302
Statice psiloclada Boiss. – – wfo-0000503285 – http://www.biodiversitylibrary.org/openurl?pid=title:286&volume=12&issue=&spage=651&date=1848
Statice psiloclada var. *albida* (Guss.) Boiss. – – wfo-0001304337
Statice psiloclada var. *genuina* Boiss. – – wfo-0001304339
Statice psiloclada var. *gracilis* Boiss. – – wfo-0001304341 – https://biodiversitylibrary.org/page/160873
Statice psiloclada var. *gracilis* Boiss. – – wfo-0001304340 – https://biodiversitylibrary.org/page/160873
Statice psiloclada var. *intermedia* (Guss.) Boiss. – – wfo-0001304342 – https://biodiversitylibrary.org/page/160873
Statice psiloclada var. *spathulaefolia* Lojac. – – wfo-0001304344
Statice psiloclada var. *spathulaefolia* Lojac. – – wfo-0001304343
Statice puberula var. *bourgaei* (Webb ex Boiss.) Stapf – – wfo-0001304345
Statice puberula Webb ex Lindl. – – wfo-0000503308 – https://biodiversitylibrary.org/page/41422864, https://biodiversitylibrary.org/page/239129
Statice pubescens (Sowerby) Druce – – wfo-0000503292
Statice pubescens DC. – – wfo-0000503303
Statice pubescens Rchb. – – wfo-0000503307
Statice pubescens Salis – – wfo-0000503305
Statice pubescens Sm. ex Schult. – – wfo-0000503304 – https://biodiversitylibrary.org/page/732529
Statice pubescens W.D.J.Koch – – wfo-0000503306 – https://biodiversitylibrary.org/page/6133676
Statice pubigera Desf. – – wfo-0000503300 – https://biodiversitylibrary.org/page/42985065
Statice pubinervis (Boiss.) Vines & Druce – – wfo-0000503309
Statice pulchella Gray – – wfo-0000503298 – https://biodiversitylibrary.org/page/30086162
Statice pumila Gasp. ex Lojac. – – wfo-0000503297

Statice pungens Brot. – – wfo-0000503296 – https://biodiversitylibrary.org/page/31323713
Statice pungens Jaub. & Spach – – wfo-0000503295 – https://babel.hathitrust.org/cgi/pt?id=mdp.39015061880194;view=1up;seq=181;size=125
Statice punicea Rendle – – wfo-0000503294
Statice purpurata L. – – wfo-0001095198 – https://biodiversitylibrary.org/page/44260800
Statice purpurata var. *genuina* Boiss. – – wfo-0001304346 – https://biodiversitylibrary.org/page/160888
Statice purpurata var. *longifolia* (Thunb.) Boiss. – – wfo-0001304347 – https://biodiversitylibrary.org/page/160888
Statice purpurata Willd. – – wfo-0001304348 – https://biodiversitylibrary.org/page/630845
Statice purpurea (W.D.J.Koch) W.D.J.Koch – – wfo-0000503289
Statice pusilla (Salis) P.Fourn. – – wfo-0000503339
Statice pycnantha K.Koch – – wfo-0001095359 – http://www.biodiversitylibrary.org/openurl?pid=title:626&volume=21&issue=&spage=716&date=1848
Statice pygmaea Tineo – – wfo-0000503382
Statice pyramidata Lowe – – wfo-0000503381
Statice pyrrholepis Pomel – – wfo-0000503380 – https://bibdigital.rjb.csic.es/viewer/10313/?offset=#page=138&viewer=picture&o=bookmark&n=0&q=
Statice quichiotis Gonz.Albo – – wfo-0000503379
Statice racemosa Lojac. – – wfo-0000503378
Statice raddiana Boiss. – – wfo-0000503377 – https://biodiversitylibrary.org/page/160875
Statice ramosissima Poir. – – wfo-0000503368
Statice rarida Vis. – – wfo-0000503375
Statice rariflora Drejer – – wfo-0000503383
Statice recurva (C.E.Salmon) C.E.Salmon – – wfo-0000503373 – https://biodiversitylibrary.org/page/8964603
Statice rediviva Svent. – – wfo-0000503372
Statice rediviva var. *pilosa* Svent. – – wfo-0001304349
Statice reinwardtii hort. ex Lanza – – wfo-0000503371
Statice remotiflora Rouy – – wfo-0000503370
Statice remotispicula Lacaita – – wfo-0000503369
Statice reniformis Girard – – wfo-0000503392 – https://biodiversitylibrary.org/page/41531992
Statice reticulata Gouan – – wfo-0000503397 – https://www.biodiversitylibrary.org/page/6085129
Statice reticulata Gouan – – wfo-0001304350 – https://books.google.com.sv/books?id=P0M-AAAAcAAJ&pg=PA135&source=gbs_toc_r&cad=2#v=onepage&q&f=false
Statice reticulata Hook. – – wfo-0001304351
Statice reticulata L. – – wfo-0000503376 – https://www.biodiversitylibrary.org/page/26068244
Statice reticulata M.Bieb. – – wfo-0000503396 – https://www.biodiversitylibrary.org/page/11268330
Statice reticulata Sievers ex Boiss. – – wfo-0000503398 – https://biodiversitylibrary.org/page/160883
Statice rigida (Wallr.) Samp. – – wfo-0001304352
Statice roborowskii Ikonn.-Gal. – – wfo-0001304353
Statice rorida Sibth. & Sm. – – wfo-0000503395
Statice rorida Vis. – – wfo-0001304354
Statice rosea (Boiss.) Boiss. – – wfo-0000503394 – https://biodiversitylibrary.org/page/160848
Statice rosea Pall. – – wfo-0000503384
Statice rosea Sm. – – wfo-0000503393 – https://biodiversitylibrary.org/page/38756811
Statice rouyana (Daveau) Samp. – – wfo-0000503399
Statice rubella S.G.Gmel. – – wfo-0000503391
Statice ruizii Font Quer – – wfo-0000503390

Statice rumelica (Boiss.) Degen & Dörfl. – – wfo-0001038950 – http://www.biodiversitylibrary.org/openurl?pid=title:6733&volume=64&issue=&spage=734&date=1897
Statice rumelica var. *tempskyana* Degen & Dörfl. – – wfo-0000733947 – http://www.biodiversitylibrary.org/openurl?pid=title:6733&volume=64&issue=&spage=734&date=1897
Statice rumicifolia Svent. – – wfo-0000503389
Statice rupicola Badarò ex Rchb. – – wfo-0000503388 – https://biodiversitylibrary.org/page/6026253
Statice ruscinonensis (Girard) P.Fourn. – – wfo-0001304355
Statice rytidophylla Hook. – – wfo-0000503387
Statice sabulosa Fourr. – – wfo-0000503386
Statice salicorniacea F.Muell. – – wfo-0001038949 – http://www.biodiversitylibrary.org/openurl?pid=title:287&volume=11&issue=88&spage=7&date=1878
Statice salmonis Sennen & Elías – – wfo-0000503345
Statice salsuginosa Boiss. – – wfo-0000503374 – https://biodiversitylibrary.org/page/160875
Statice salsuginosa var. *hirtula* Willk. – – wfo-0001304356
Statice sanjurjensis Sennen – – wfo-0000503349
Statice sanjurjoi Sennen & Mauricio – – wfo-0001304357
Statice sareptana A.K.Becker – – wfo-0000503348 – https://biodiversitylibrary.org/page/44169109, https://biodiversitylibrary.org/page/44169165
Statice sartorii (Boiss.) Nyman – – wfo-0000503347
Statice scabra Drege ex Boiss. – – wfo-0001304358
Statice scabra Krauss – – wfo-0001304359 – https://biodiversitylibrary.org/page/40858
Statice scabra Pall. ex Boiss. – – wfo-0001304360 – https://biodiversitylibrary.org/page/160903
Statice scabra Pers. – – wfo-0001304361 – https://biodiversitylibrary.org/page/235118
Statice scabra Thunb. – – wfo-0001095203 – https://biodiversitylibrary.org/page/3553174
Statice scabrida C.Presl – – wfo-0000503344
Statice scanica Warm. – – wfo-0000503343
Statice schirasiana Boiss. – – wfo-0001304362 – https://biodiversitylibrary.org/page/34838820
Statice schrenkiana Fisch. & C.A.Mey. – – wfo-0001095339
Statice scoparia C.A.Mey. ex Boiss. – – wfo-0001304363 – https://biodiversitylibrary.org/page/160869
Statice scoparia hort. ex E.Vilm. – – wfo-0001304364
Statice scoparia M.Bieb. – – wfo-0001304365
Statice scoparia Pall. ex Willd. – – wfo-0001095360
Statice scoparia Rchb. ex Nyman – – wfo-0001304366 – https://biodiversitylibrary.org/page/11015753
Statice scopoliana Bertol. – – wfo-0000503361
Statice scorpius Jaub. & Spach – – wfo-0000503346 – https://babel.hathitrust.org/cgi/pt?id=mdp.39015061880194&seq=179
Statice scorzonerifolia Link – – wfo-0000503365
Statice sebkarum f. *glomerata* Batt. – – wfo-0001304367
Statice sebkarum f. *macrolepis* Batt. – – wfo-0001304368
Statice sebkarum Pomel – – wfo-0000503364 – https://bibdigital.rjb.csic.es/viewer/10313/?offset=#page=137&viewer=picture&o=bookmark&n=0&q=
Statice sebkarum var. *mauritii* Sennen – – wfo-0001304369 – https://bibdigital.rjb.csic.es/viewer/9472/?offset=#page=242&viewer=picture&o=search&n=0&q=mauritii
Statice sect. *Circinaria* Boiss. – – wfo-0001304370 – https://www.biodiversitylibrary.org/page/160888
Statice sect. *Ctenostachys* Boiss. – – wfo-0001304371 – https://www.biodiversitylibrary.org/page/160863
Statice sect. *Goniolimon* (Boiss.) Hook. – – wfo-0001304372
Statice sect. *Jovibarba* Boiss. – – wfo-0001304373 – https://www.biodiversitylibrary.org/page/160887
Statice sect. *Myriolepis* Boiss. – – wfo-0001304374 – https://biodiversitylibrary.org/page/160889

Statice sect. *Plathymenium* Boiss. – – wfo-0001304375 – https://www.biodiversitylibrary.org/page/160864
Statice sect. *Polyarthrion* Boiss. – – wfo-0001304376 – https://www.biodiversitylibrary.org/page/160889
Statice sect. *Pteroclados* (Spach) Boiss. – – wfo-0001304377 – https://biodiversitylibrary.org/page/160859
Statice sect. *Pterocladus* (Spach) Boiss. – – wfo-0001304378 – https://www.biodiversitylibrary.org/page/160859
Statice sect. *Schizhymenium* Boiss. – – wfo-0001304379 – https://www.biodiversitylibrary.org/page/160887
Statice sect. *Schizopetalum* Boiss. – – wfo-0001304380 – http://bibdigital.rjb.csic.es/ing/Libro.php?Libro=1418&Pagina=69
Statice sect. *Siphonantha* Boiss. – – wfo-0001304381 – https://www.biodiversitylibrary.org/page/160890
Statice sect. *Tropidice* Griseb. – – wfo-0001304382
Statice secundiramea Lojac. – – wfo-0000503363
Statice sedoides Regel – – wfo-0001095282 – http://www.biodiversitylibrary.org/openurl?pid=title:14650&volume=6&issue=2&spage=384&date=1880
Statice semenovii Herder – – wfo-0001095340 – http://www.biodiversitylibrary.org/openurl?pid=title:4951&volume=41&issue=2&spage=398&date=1868
Statice serbica (Vis.) Nyman – – wfo-0000503351
Statice serotina Rchb. – – wfo-0000503360 – https://biodiversitylibrary.org/page/6026253
Statice setigera Bubani – – wfo-0000503367 – https://biodiversitylibrary.org/page/6718531
Statice sewerzowii (Herder) Regel – – wfo-0000503358 – https://biodiversitylibrary.org/page/15733848
Statice sewerzowii var. *alatavica* Regel – – wfo-0001304383 – https://biodiversitylibrary.org/page/15733849
Statice sewerzowii var. *typica* Regel – – wfo-0001304384 – https://biodiversitylibrary.org/page/15733849
Statice sibirica (Turcz. ex Boiss.) Ledeb. – – wfo-0000503357
Statice sibirica Dum.Cours. – – wfo-0000503356
Statice sibthorpiana Guss. – – wfo-0000503355
Statice sicula Tineo ex Lojac. – – wfo-0000503354
Statice sieberi Boiss. – – wfo-0000503353
Statice simplex (Pomel) Santa – – wfo-0000503352
Statice sinensis Girard – – wfo-0001095300 – https://biodiversitylibrary.org/page/41531996
Statice sinensium Gand. – – wfo-0001095306 – http://www.biodiversitylibrary.org/openurl?pid=title:359&volume=66&issue=&spage=221&date=1919
Statice sinuata L. – – wfo-0000503293 – http://www.biodiversitylibrary.org/openurl?pid=title:669&volume=1&issue=&spage=276&date=1753
Statice sinuata var. *candidissima* hort. – – wfo-0001304385
Statice sinuata var. *integrifolia* Boiss. – – wfo-0001304386
Statice sinuata var. *subglabra* H.Lindb. – – wfo-0001304387
Statice sisymbriifolia Jaub. & Spach – – wfo-0000503203 – https://babel.hathitrust.org/cgi/pt?id=mdp.39015061880194;view=1up;seq=176;size=125
Statice smithii Ten. – – wfo-0000503202
Statice sogdiana Popov – – wfo-0000503201
Statice sokotrana Vierh. – – wfo-0000503200
Statice soleirolii Duby – – wfo-0001304388
Statice somalorum Vierh. – – wfo-0000503199
Statice sommieriana (Fiori) Sommier – – wfo-0001304389
Statice spachii Girard – – wfo-0000503198 – https://biodiversitylibrary.org/page/41531998
Statice spathulata Desf. – – wfo-0000503196 – https://biodiversitylibrary.org/page/305834
Statice spathulata Heldr. ex Nyman – – wfo-0001304390 – https://biodiversitylibrary.org/page/11015755
Statice spathulata Hook. – – wfo-0001304391
Statice spathulata Hook. – – wfo-0001304392

Statice spathulata Jan ex Nyman – – wfo-0001304393 – https://biodiversitylibrary.org/page/11015754
Statice spathulata Nyman – – wfo-0001304394 – https://biodiversitylibrary.org/page/38621622
Statice spathulata Sieber ex Boiss. – – wfo-0001304395 – https://biodiversitylibrary.org/page/160872
Statice spathulata var. *emarginata* (Willd.) Boiss. – – wfo-0001304396 – https://biodiversitylibrary.org/page/160872
Statice spathulata var. *spathulata* – – wfo-0001304397
Statice spathulata Willd. ex Boiss. – – wfo-0001304398 – https://biodiversitylibrary.org/page/160874, https://biodiversitylibrary.org/page/160872
Statice speciosa Forssk. – – wfo-0001304399 – https://biodiversitylibrary.org/page/737832
Statice speciosa L. – – wfo-0000503211 – http://www.biodiversitylibrary.org/openurl?pid=title:669&volume=1&issue=&spage=275&date=1753
Statice speciosa var. *crispa* Regel – – wfo-0000741787 – http://www.biodiversitylibrary.org/openurl?pid=title:14650&volume=6&issue=2&spage=387&date=1880
Statice speciosa var. *genuina* Kryl. – – wfo-0001304400
Statice speciosa var. *lanceolata* Regel – – wfo-0001304401
Statice speciosa var. *lepidota* Regel – – wfo-0001304402
Statice speciosa var. *multicaulis* Krylov – – wfo-0001304403
Statice speciosa var. *stricta* Regel – – wfo-0000741789 – http://www.biodiversitylibrary.org/openurl?pid=title:14650&volume=6&issue=2&spage=387&date=1880
Statice speciosa var. *typica* Regel – – wfo-0001304404
Statice spectabilis Svent. – – wfo-0000503197
Statice spicata Hohenacker – – wfo-0001304405
Statice spicata var. *foliis-subintegris* Aitch. – – wfo-0001304406 – https://biodiversitylibrary.org/page/2925514
Statice spicata var. *glabra* Regel – – wfo-0001304407
Statice spicata Willd. – – wfo-0000503218 – http://www.biodiversitylibrary.org/openurl?pid=title:727&volume=1&issue=2&spage=1533&date=1798
Statice spinulosa (Boiss.) Maire – – wfo-0000503216
Statice spinulosa Janka – – wfo-0000503217 – https://biodiversitylibrary.org/page/30862190
Statice spiridonowii B.Fedtsch. – – wfo-0001304408
Statice splendens Lag. & Rodr. – – wfo-0000503215 – http://www.biodiversitylibrary.org/openurl?pid=title:14767&volume=5&issue=15&spage=274&date=1802http://www.biodiversitylibrary.org/openurl?pid=title:162&volume=5&issue=15&spage=274&date=1802
Statice squamata Poir. – – wfo-0000503214 – https://biodiversitylibrary.org/page/782267
Statice stephani Sennen – – wfo-0001304409 – http://bibdigital.rjb.csic.es/ing/Libro.php?Libro=15&Pagina=263
Statice stocksii Boiss. – – wfo-0000503212 – http://www.biodiversitylibrary.org/openurl?pid=title:286&volume=12&issue=&spage=664&date=1848
Statice stricta Gueldenst. – – wfo-0000503204
Statice subg. Armeriastrum Jaub. & Spach – – wfo-0001304410
Statice subg. Limonium Spach – – wfo-0001304411 – https://www.biodiversitylibrary.org/page/31486452
Statice subg. Psylliostachys Jaub. & Spach – – wfo-0001304412 – https://babel.hathitrust.org/cgi/pt?id=mdp.39015061880194;view=1up;seq=176;size=125
Statice subg. Pterocladus Spach – – wfo-0001304414 – https://www.biodiversitylibrary.org/page/31486455
Statice subg. Pterocladus Spach – – wfo-0001304413 – https://www.biodiversitylibrary.org/page/31486455
Statice subg. Schizopetalum (Boiss.) Melchior – – wfo-0001304415
Statice subsect. *Genuinae* Boiss. – – wfo-0001304416 – https://www.biodiversitylibrary.org/page/160867
Statice subsect. *Hyalolepideae* Boiss. – – wfo-0001304417 – https://www.biodiversitylibrary.org/page/160881
Statice subsect. *Nobiles* Boiss. – – wfo-1000045201 – https://www.biodiversitylibrary.org/page/160860
Statice subsect. *Odontolepideae* Boiss. – – wfo-1000045203 – https://www.biodiversitylibrary.org/page/160859
Statice subsect. *Pruinosae* Batt. – – wfo-1000045205
Statice subsect. *Sarcophyllae* Boiss. – – wfo-0001304418 – https://www.biodiversitylibrary.org/page/160885

Statice subsect. *Sphaerostachys* Boiss. – – wfo-0001304419 – https://www.biodiversitylibrary.org/page/160886
Statice suffruticosa L. – – wfo-0000503210 – http://www.biodiversitylibrary.org/openurl?pid=title:669&volume=1&issue=&spage=276&date=1753
Statice suffruticosa var. *carnosa* (Boiss.) Kusn. – – wfo-0001304420
Statice suffruticosa var. *leptophylla* Trautv. – – wfo-0001304421
Statice suffruticosa var. *typica* Trautv. – – wfo-0001304422
Statice superba Regel ex F.Haage & M.Schmidt – – wfo-0000503220 – https://biodiversitylibrary.org/page/42128199, https://biodiversitylibrary.org/page/43059964
Statice superba var. *flora-alba* Benary – – wfo-0001304423
Statice supina Girard – – wfo-0000503209 – https://biodiversitylibrary.org/page/41531992
Statice suworowii Regel – – wfo-0000503208 – http://www.biodiversitylibrary.org/openurl?pid=title:14650&volume=7&issue=&spage=550&date=1880
Statice suworowii var. *alba* hort. – – wfo-0001304424 – https://www.biodiversitylibrary.org/page/25916220
Statice tatarica L. – – wfo-0000503207 – https://biodiversitylibrary.org/page/358294
Statice tatarica var. *angustifolium* hort. ex F.T.Hubb. – – wfo-0001304425
Statice tatarica var. *nana* hort. ex hort. – – wfo-0001304426
Statice tatarica var. *trigonoides* Poir. – – wfo-0001304427 – https://biodiversitylibrary.org/page/724426
Statice tatarica var. *typica* Regel – – wfo-0001304428
Statice taubertii Hausskn. – – wfo-0000503206 – https://www.biodiversitylibrary.org/page/14241214
Statice taxanthema Schult. – – wfo-0000503205
Statice tchefouensis Gand. – – wfo-0001095313 – http://www.biodiversitylibrary.org/openurl?pid=title:359&volume=66&issue=&spage=221&date=1919
Statice tenella Regel – – wfo-0001304429
Statice tenella Turcz. – – wfo-0001095331 – http://www.biodiversitylibrary.org/openurl?pid=title:4951&volume=5&issue=&spage=203&date=1832
Statice tenoreana Guss. – – wfo-0000745484
Statice tenuicula Tineo ex Guss. – – wfo-0000503167
Statice tenuifolia Bertol. ex Moris – – wfo-0000503195
Statice tenuifolia Jaub. & Spach – – wfo-0000503173 – https://babel.hathitrust.org/cgi/pt?id=mdp.39015061880194;view=1up;seq=180;size=125
Statice terekiensis Gueldenst. – – wfo-0000503172
Statice teretifolia Baker ex Oliv. – – wfo-0000503171 – https://biodiversitylibrary.org/page/40892791
Statice tetragona Drège ex Boiss. – – wfo-0000503169 – https://biodiversitylibrary.org/page/160881
Statice tetragona Thunb. – – wfo-0000503170 – https://biodiversitylibrary.org/page/3553174
Statice thouinii Viv. – – wfo-0000503161
Statice thymoides Girard – – wfo-0000503168 – http://gallica.bnf.fr/ark:/12148/bpt6k209710c/f88.image
Statice tineoi J.Woods – – wfo-0000503166
Statice tineoi Lojac. – – wfo-0000503176
Statice tineoi var. *gracilis* (Tineo) J.Woods – – wfo-0001304430 – https://www.biodiversitylibrary.org/page/33502160
Statice tineoi var. *parvifolia* (Tineo) J.Woods – – wfo-0001304431 – https://www.biodiversitylibrary.org/page/33502160
Statice tineoi var. *pygmaea* (Tineo) J.Woods – – wfo-0001304432 – https://www.biodiversitylibrary.org/page/33502160
Statice tomentella Boiss. – – wfo-0000503165 – https://www.biodiversitylibrary.org/page/160869
Statice tomentella Boiss. – – wfo-0001303035 – https://biodiversitylibrary.org/page/34838820
Statice tomentella subsp. *sareptana* Nyman – – wfo-0001304433
Statice tournefortii Girard – – wfo-0000503163 – https://biodiversitylibrary.org/page/41531992
Statice tournefortii Jaub. & Spach – – wfo-0000503164 – https://babel.hathitrust.org/cgi/pt?id=mdp.39015061880194;view=1up;seq=183;size=125
Statice tracyi Gand. – – wfo-0000503162 – https://biodiversitylibrary.org/page/345071

Statice tragacanthina Jaub. & Spach – – wfo-0001042001 – https://babel.hathitrust.org/cgi/pt?id=mdp.39015061880194&seq=180
Statice transmontana Samp. – – wfo-0000503186
Statice transwalliana Pugsley – – wfo-0000503192
Statice tremolsii Rouy – – wfo-0000503191
Statice tremolsii var. *delilei* (Aubouy) Rouy – – wfo-0001304434
Statice trigona Pall. – – wfo-0000503190
Statice trigonoides Boiss. – – wfo-0000503189 – https://biodiversitylibrary.org/page/160857
Statice trigonoides Pall. – – wfo-0000503188
Statice trinervia Schult. – – wfo-0000503187
Statice tripteris Poir. – – wfo-0000503177 – https://biodiversitylibrary.org/page/782267
Statice triquetra Boeber – – wfo-0000503185
Statice tschurjukiensis Klokov – – wfo-0001095248
Statice tuberculata Boiss. – – wfo-0000503193 – https://biodiversitylibrary.org/page/160884
Statice tubiflora Delile – – wfo-0000503183
Statice tubiflora Sieber ex Schult. – – wfo-0000503182 – https://biodiversitylibrary.org/page/732555
Statice tubiflora var. *maroccana* Batt. & Trab. – – wfo-0000750396
Statice tubiflora var. *zanonii* Pamp. – – wfo-0000750390
Statice tunetana Barratte & Bonnet – – wfo-0000503181
Statice turkestanica Gand. – – wfo-0000503180 – https://biodiversitylibrary.org/page/345071
Statice ulicina Willd. ex Schult. – – wfo-0000503179
Statice undulata Bory & Chaub. – – wfo-0000503178
Statice urgellensis Sennen – – wfo-0000503232
Statice uruguayensis Arechav. – – wfo-0001236409
Statice valentina Sennen & Pau – – wfo-0000503263
Statice varia Hance – – wfo-0001095307 – http://www.biodiversitylibrary.org/openurl?pid=title:15787&volume=20&issue=238&spage=290&date=1882
Statice variabilis Salisb. – – wfo-0000503262
Statice velutina (Welw. ex Boiss. & Reut.) Samp. – – wfo-0000503261
Statice venusta Fenzl ex Boiss. – – wfo-0000503260 – https://biodiversitylibrary.org/page/18115504
Statice verrucosa Willd. ex Boiss. – – wfo-0000503259 – https://biodiversitylibrary.org/page/160872
Statice vestita C.E.Salmon – – wfo-0000503258 – https://www.biodiversitylibrary.org/page/58633732
Statice viciosoi Pau – – wfo-0000503249
Statice villosa (Girard) Jahand. & Maire – – wfo-0000503254 – https://bibdigital.rjb.csic.es/viewer/13176/?offset=#page=19&viewer=picture&o=search&n=0&q=statice
Statice villosa var. *zaianica* Emberger, L. & Maire – – wfo-0001304435
Statice viminea Schrad. ex Hornem. – – wfo-0000503264
Statice virgata Herb. ex Nyman – – wfo-0001304436 – https://biodiversitylibrary.org/page/11015755
Statice virgata Orph. ex Nyman – – wfo-0001304437 – https://biodiversitylibrary.org/page/11015754
Statice virgata var. *divaricata* Rouy – – wfo-0000745486
Statice virgata var. *gracillima* J.J.Rodr. – – wfo-0001304438 – https://www.biodiversitylibrary.org/page/14597830
Statice virgata var. *macroclada* Pau – – wfo-0001304439
Statice virgata var. *minuta* (L.) Knoche – – wfo-0001304440
Statice virgata var. *pumila* Boiss. – – wfo-0000745489 – https://biodiversitylibrary.org/page/160876
Statice virgata var. *reticulata* Boiss. – – wfo-0000745487 – https://biodiversitylibrary.org/page/160876

Statice virgata var. *tenia* Heldr. – – wfo-0001304441
Statice virgata Willd. – – wfo-0000503252
Statice vulgaris Druce – – wfo-0001304442
Statice vulgaris Hill – – wfo-0000503268
Statice vulgaris Sweet – – wfo-0000503250 – https://biodiversitylibrary.org/page/30078652
Statice vulgaris var. *elongata* (Hoffm.) Samp. – – wfo-0001304443
Statice vulgaris var. *langeana* (Henriq.) Samp. – – wfo-0001304444
Statice vulgaris var. *majuscula* (Samp.) Pires de Lima – – wfo-0001304445
Statice vulgaris var. *pubigera* (Desf.) Pires de Lima – – wfo-0001304446
Statice welwitschii (Boiss.) F.T.Hubb. ex L.H.Bailey – – wfo-0000503270 – https://biodiversitylibrary.org/page/568409
Statice welwitschii (Boiss.) Samp. – – wfo-0000503255
Statice welwitschii var. *berlengensis* (Daveau) Samp. – – wfo-0001304447
Statice welwitschii var. *cinerea* (Boiss. & Reut.) Samp. – – wfo-0001304448
Statice welwitschii var. *platyphylla* (Daveau) Samp. – – wfo-0001304449
Statice willdenovii Poir. – – wfo-0000503276 – https://biodiversitylibrary.org/page/782266
Statice willdenowiana Herb. ex Nyman – – wfo-0000503275 – https://biodiversitylibrary.org/page/11015754
Statice willdenowiana Rchb. – – wfo-0000503273
Statice willdenowii Loisel. – – wfo-0000503272
Statice wrightii Hance – – wfo-0001095323 – http://www.biodiversitylibrary.org/openurl?pid=title:5010&volume=5&issue=&spage=236&date=1866
Statice xipholepis Baker – – wfo-0000503271 – https://biodiversitylibrary.org/page/40984770
Staticeae Bartl. – – wfo-0001304450
Taxanthema aegyptiaca (Pers.) Sweet – – wfo-0000456373 – https://biodiversitylibrary.org/page/30078653
Taxanthema aegyptiacum (Pers.) Sweet – – wfo-0001304451
Taxanthema alata (Willd.) Sweet – – wfo-0000456372 – https://biodiversitylibrary.org/page/30078653
Taxanthema armeria Neck. – – wfo-0000456371 – https://www.biodiversitylibrary.org/page/3589377
Taxanthema attenuata R.Br. – – wfo-0000456370 – https://biodiversitylibrary.org/page/47589045
Taxanthema auriculifolia (Vahl) Sweet – – wfo-0000456369 – https://biodiversitylibrary.org/page/30078652
Taxanthema australis R.Br. – – wfo-0000455322 – https://biodiversitylibrary.org/page/2954582
Taxanthema bellidifolia (Gouan) Sweet – – wfo-0000456645 – https://biodiversitylibrary.org/page/30078652
Taxanthema caroliniana (Walter) Sweet – – wfo-0000455469 – https://biodiversitylibrary.org/page/30078652
Taxanthema carolinianum (Walter) Sweet – – wfo-0001304452
Taxanthema caspia (Willd.) Sweet – – wfo-0000454429 – https://biodiversitylibrary.org/page/30078652
Taxanthema conspicua (Sims) Sweet – – wfo-0000454428 – https://biodiversitylibrary.org/page/30078653
Taxanthema cordata (L.) Sweet – – wfo-0000454427 – https://biodiversitylibrary.org/page/30078652
Taxanthema dichotoma (Cav.) Sweet – – wfo-0001304466 – https://biodiversitylibrary.org/page/30078653
Taxanthema dichotomum (Cav.) Sweet – – wfo-0000454426 – https://biodiversitylibrary.org/page/30078653
Taxanthema echioides Sweet – – wfo-0000454425 – https://biodiversitylibrary.org/page/30078653
Taxanthema elata (Fisch. ex Spreng.) Sweet – – wfo-0000454424 – https://biodiversitylibrary.org/page/30078652
Taxanthema emarginata (Willd.) Sweet – – wfo-0000454422 – https://biodiversitylibrary.org/page/30078652
Taxanthema ferulacea (L.) Sweet – – wfo-0000454421 – https://biodiversitylibrary.org/page/30078653
Taxanthema flexuosa (L.) Sweet – – wfo-0000454408 – https://biodiversitylibrary.org/page/30078653
Taxanthema globulariifolia Sweet – – wfo-0000454419 – https://biodiversitylibrary.org/page/30078652
Taxanthema gmelini Sweet – – wfo-0000454430 – https://biodiversitylibrary.org/page/30078652

Taxanthema graminifolia (Aiton) Sweet – – wfo-0000454417 – https://biodiversitylibrary.org/page/30078652
Taxanthema incana (L.) Sweet – – wfo-0000454416 – https://biodiversitylibrary.org/page/30078652
Taxanthema latifolia (Sm.) Sweet – – wfo-0000454415 – https://biodiversitylibrary.org/page/30078652
Taxanthema limonium (L.) Sweet – – wfo-0000454414 – https://biodiversitylibrary.org/page/30078652
Taxanthema macrophylla (Willd. ex Spreng.) Sweet – – wfo-0000454413 – https://biodiversitylibrary.org/page/30078653
Taxanthema minuta (L.) Sweet – – wfo-0000454411 – https://biodiversitylibrary.org/page/30078653
Taxanthema monopetala (L.) Sweet – – wfo-0000454410 – https://biodiversitylibrary.org/page/30078653
Taxanthema mucronata (L.f.) Sweet – – wfo-0000454409 – https://biodiversitylibrary.org/page/30078653
Taxanthema Neck. ex R.Br. – – wfo-4000037627 – https://biodiversitylibrary.org/page/3589377, https://biodiversitylibrary.org/page/2954582
Taxanthema oleifolia (Mill.) Sweet – – wfo-0000454436 – https://biodiversitylibrary.org/page/30078652
Taxanthema pectinata (Aiton) Sweet – – wfo-0000454438 – https://biodiversitylibrary.org/page/30078653
Taxanthema purpurata (L.) Sweet – – wfo-0000454420 – https://biodiversitylibrary.org/page/30078652
Taxanthema reticulata (L.) Sweet – – wfo-0000454447 – https://biodiversitylibrary.org/page/30078653
Taxanthema scabra (Thunb.) Sweet – – wfo-0000454446 – https://biodiversitylibrary.org/page/30078652
Taxanthema scoparia Sweet – – wfo-0000454445 – https://biodiversitylibrary.org/page/30078652
Taxanthema sinuata (L.) Sweet – – wfo-0000454444 – https://biodiversitylibrary.org/page/30078653
Taxanthema spathulata (Desf.) Sweet – – wfo-0000454443 – https://biodiversitylibrary.org/page/30078652
Taxanthema speciosa (L.) Sweet – – wfo-0000454442 – https://biodiversitylibrary.org/page/30078653
Taxanthema suffruticosa (L.) Sweet – – wfo-0000454441 – https://biodiversitylibrary.org/page/30078653
Taxanthema tatarica (L.) Sweet – – wfo-0000454439 – https://biodiversitylibrary.org/page/30078653
Taxanthema tetragona (Thunb.) Sweet – – wfo-0000454431 – https://biodiversitylibrary.org/page/30078652
Taxanthema thouinii (Viv.) Sweet – – wfo-0000454437 – https://biodiversitylibrary.org/page/30078653
Taxanthema viminea Sweet – – wfo-0000454449 – https://biodiversitylibrary.org/page/30078653
Taxanthema virgata (Willd.) Sweet – – wfo-0000454435 – https://biodiversitylibrary.org/page/30078653
Taxanthema willdenowiana Sweet – – wfo-0000454434 – https://biodiversitylibrary.org/page/30078652
Thela alba Lour. – – wfo-0001095274 – https://www.biodiversitylibrary.org/page/48860058
Thela coccinea Lour. – – wfo-0001095275 – https://www.biodiversitylibrary.org/page/48860058
Thela Lour. – – wfo-4000038119 – https://www.biodiversitylibrary.org/page/653318
Valoradia abyssinica Hochst. – – wfo-0001244060 – http://www.biodiversitylibrary.org/openurl?pid=title:64&volume=25&issue=&spage=239&date=1842
Valoradia Hochst. – – wfo-4000039976 – https://www.biodiversitylibrary.org/page/38131
Valoradia patula Hochst. – – wfo-0000418245 – https://biodiversitylibrary.org/page/38132
Valoradia plumbaginoides (Bunge) Boiss. – – wfo-0001095318 – http://www.biodiversitylibrary.org/openurl?pid=title:286&volume=12&issue=&spage=695&date=1848
Vassilczenkoa Lincz. – – wfo-4000040055
Vassilczenkoa sogdiana (Lincz.) Lincz. – – wfo-0001095252
Vogelia africana Lam. – – wfo-0000418250 – https://biodiversitylibrary.org/page/33349484, https://biodiversitylibrary.org/page/33348769
Vogelia arabica Boiss. – – wfo-0000418251 – https://biodiversitylibrary.org/page/160918
Vogelia indica Gibson ex Wight – – wfo-0000418252 – https://biodiversitylibrary.org/page/40384807
Vogelia indica var. *socotrana* Balf.f. – – wfo-0001304453 – https://www.biodiversitylibrary.org/page/48552994
Vogelia Lam. – – wfo-4000040408 – https://biodiversitylibrary.org/page/33348768
Vogelia pendula Balf.f. – – wfo-0000418253 – https://biodiversitylibrary.org/page/48552646
Vogelia perfoliata Stocks ex Wight – – wfo-0000418254 – https://biodiversitylibrary.org/page/40384806

References cited in the checklist

Abdulina SA (1999) Spisok sosudistykn rastenii Kazakhstana [Checklist of Vascular Plants of Kazakhstan]. Academy of Sciences, Almaty, Kazakhstan, 187 pp.

Adamson RS, Compton RH, Barker WF, Bond P (1941) Plantae novae Africanae. Journal of South African Botany 7: 187–208.

Agius DR, Galea ME, Cambria S, del Galdo GG, Brullo S (2023) Taxonomic survey on the genus *Limonium* in the Maltese islands, with description of a new species. Phytotaxa 622(2): 099–114. https://doi.org/10.11646/phytotaxa.622.2.1

Akaydın G (2018) *Acantholimon ibrahimii* (Plumbaginaceae), a new species of *A.* section *Staticopsis* from the Mediterranean part of Turkey. Phytotaxa 340(1): 048–054. https://doi.org/10.11646/phytotaxa.340.1.2

Akaydın G, Crespo MB (2018) *Acantholimon davisii* (Plumbaginaceae), a new specific name for the Turkish endemic *A. caryophyllaceum* subsp. *Parviflorum.* Phytotaxa 334(1): 080–082. https://doi.org/10.11646/phytotaxa.334.1.13

Akhani H, Malekmohammadi M, Madhavi P, Gharibiyan A, Chase MW (2013) Phylogenetics of the Irano-Turanian taxa of *Limonium* (Plumbaginaceae) based on ITS nrDNA sequences and leaf anatomy provides evidence for species delimitation and relationships of lineages. Botanical Journal of the Linnean Society 171(3): 519–550. https://doi.org/10.1111/boj.12015

APG III [Angiosperm Phylogeny Group] (2009) An update of the Angiosperm Phylogeny Group classification for the orders and families of flowering plants: APG III. Botanical Journal of the Linnean Society 161: 105–121. https://doi.org/10.1111/j.1095-8339.2009.00996.x

APG IV [Angiosperm Phylogeny Group] (2016) An update of the Angiosperm Phylogeny Group classification for the orders and families of flowering plants: APG IV. Botanical Journal of the Linnean Society 181(1): 1–20. https://doi.org/10.1111/boj.12385

Arrigoni PV (2015) Contribution to the study of the genus *Armeria* (Plumbaginaceae) in the Italian peninsula. Flora Mediterranea 25 (Special issue): 7–32. https://doi.org/10.7320/FlMedit25SI.007

Arrigoni PV, Diana S (1990) Le piante endemiche della Sardegna, 192–197. Bollettino della Società Sarda di Scienze Naturali 27: 259–282.

Assadi M (2003) Two new species of the genus *Acantholimon* Boiss. (Plumbaginaceae) from Iran. The Iranian Journal of Botany 10(1): 25–29. https://doi.org/20.1001.1.1029788.1382.10.1.4.7

Assadi M (2004) *Acantholimon hormozganense* and *A. zaeifii* (Plumbaginaceae), two new species from Iran. The Iranian Journal of Botany 10(2): 153–157. https://doi.org/20.1001.1.1029788.1383.10.2.7.6

Assadi M (2005) Four new species of the genus *Acantholimon* (Plumbaginaceae) from Iran. The Iranian Journal of Botany 11(1): 31–39. https://doi.org/20.1001.1.1029788.1384.11.1.5.9

Assadi M (2005) Plumbaginaceae. *In*: Assadi M, Jamzad Z, Maassoumi AA (Eds) *Flora of Iran,* vol. 51. Research Institute of Forest and Rangeland, Tehran. 230 pp.

Assadi M, Mirtadzadidni SM (2006) Three new species of the genus *Acantholimon* (Plumbaginaceae) from Iran. The Iranian Journal of Botany 11(2): 129–136. https://doi.org/20.1001.1.1029788.1384.11.2.1.7

Assadi M, Zeraatkar A (2020) *Acantholimon saadii* (Plumbaginaceae), a new species from Iran. The Iranian Journal of Botany 26(1): 01–05. https://doi.org/10.22092/ijb.2020.342569.1277

Bailey LH (1916) Nomenclatorial transferes. Rhodora 18(211): 152–160.

Baker HG (1953) Dimorphism and monomorphism in the Plumbaginaceae III. Correlation of geographical distribution patterns with dimorphism and monomorphism in *Limonium*. Annals of Botany 17(4): 615–628. https://doi.org/10.1093/oxfordjournals.aob.a083374

Baker WJ, Bailey P, Barber V, Barker A, Bellot S, Bishop D, Botigué LR, Brewer G, Carruthers T, Clarkson JJ, Cook J, Cowan RS, Dodsworth S, Epitawalage N, Françoso E, Gallego B, Johnson MG, Kim JT, Leempoel K, Maurin O, Mcginnie C, Pokorny L, Roy S, Stone M, Toledo E, Wickett NJ, Zuntini AR, Eiserhardt WL, Kersey PJ, Leitch IJ, Forest F (2022) A comprehensive phylogenomic platform for exploring the Angiosperm tree of life. Systematic Biology 71(2): 301−319. https://doi.org/10.1093/sysbio/syab035

Battandier JA (1890) Plumbaginaceae. In: Battandier JA (Ed) Flore de l'Algérie, Dicotylédones [1](4), Libraire F. Savy, Paris, 724–739.

Baumel A, Médail F, Juin M, Paquier T, Clares M, Laffargue P, Lutard H, Dixon L, Pires M (2020) Population genetic structure and management perspectives for *Armeria belgenciensis,* a narrow endemic plant from Provence (France). Plant Ecology and Evolution 153(2): 219–228. https://doi.org/10.5091/plecevo.2020.1702

Becherer A (1928) Zur Nomenklatur einiger Sippen mitteleuropäischer Gefäßpflanzen. Repertorium Specierum Novarum Regni Vegetabilis 25: 10-15.

Bernis F (1954) Revision del género *Armeria* Willd. con especial referencia a los grupos ibericas. Anales del Instituto Botánico A. J. Cavanilles 11(2): 5–287.

Bernis F (1955) Revision del género *Armeria* Willd. con especial referencia a los grupos ibericas II. Anales del Instituto Botánico A. J. Cavanilles 12(2): 77–252.

Bernis F (1957) Revision del género *Armeria* Willd. con especial referencia a los grupos ibericas, Parte Secunda (conclusión). Anales del Instituto Botánico A. J. Cavanilles 14(1): 259–433.

Blake SF (1916) *Limonium* in North America and Mexico. Rhodora 18(207): 53–66.

Blanca G, Cueto M, Fuentes J, Tello S (2017) *Armeria villosa* subsp. *Enritrianoi subsp. nov.* (Plumbaginaceae) en el sureste de España (provincia de Jaén). Acta Botanica Malacitana 42(2): 287–291. https://doi.org/10.24310/abm.v42i2.2882

Bogdanović S, Brullo S (2015) Taxonomic revision of the *Limonium cancellatum* group (Plumbaginaceae) in Croatia. Phytotaxa 215(1): 001–087. https://doi.org/10.11646/phytotaxa.215.1.1

Bogdanović S, Shuka L, del Galdo GG, Brullo S (2022) *Limonium ksamilum* (Plumbaginaceae), a new species from Albania. Phytotaxa 554(1): 85–92. https://doi.org/10.11646/phytotaxa.554.1.7

Boissier E (1848) Plumbaginales. In: de Candolle AP (Ed) Prodromus systematis naturalis regni vegetabilis, Treuttel et Wurz, Paris, France 12: 617–696.

Boissier PE (1846) Diagnoses plantarum orientalem novarum, ser. 1, vol. 7. B. Hermann, Lipsiae, 130 pp.

Boissier PE (1879) Flora orientalis sive enumeration plantarum in Oriente, a Graecia et Aegypto ad Indiae fines hucusque observatarum, seri 2, vol. 4. H. Georg, Bibliopolam, Genevae et Basilae, pp. 281–1276.

Bokhari MH, Edmonson JR (1982) Plumbaginaceae. In: Davis PH (Ed) Flora of Turkey and the East Aegean Islands, vol. 7. Edinburgh University Press, Edinburgh, 463–504.

Bolòs o, Vigo J, Masalles RM, Ninot JM (1990) Flora Manual dels Països Catalans. Editorial Pòrtic, Barcelona.

Bordbar F, Mirtadzadini M (2022) *Acantholimon assadii* (Plumbaginaceae), a new species from flora of Iran. Phytotaxa 574(1): 99–104. https://doi.org/10.11646/phytotaxa.574.1.7

Boulos L (2000) Flora of Egypt, Geraniaceae-Boraginaceae, vol. 2. Al Hadara Publishing, Cairo, Egypt, 352pp.

Bredenkamp CL (2003) Plumbaginaceae. In: Germishuizen G, Meyer NL (Eds) Plant of southern Africa: an annotated checklist. Strelitzia 14: 778–780. National Botanical Institute, Pretoria.

Brenan JP (1981) Index Kewensis Supplementum 16. Oxford University Press, New York.

Britton NL (1906) Contributions to the flora of Bahama Islands III. Bulletin of the New York Botanical Garden 4: 137–143.

Brullo S (1978) Il genere *Limonium* Miller in Cirenaica. Webbia 33: 137–158. http://dx.doi.org/10.1080/00837792.1978.10670114

Brullo S (1980) Taxonomic and nomenclatural notes on the genus *Limonium* in Sicily. Botaniska Notisier 133: 281–293.

Brullo S (1988) Miscellaneous notes on the genus *Limonium* (Plumbaginaceae). Willdenowia 17: 11–18. https://www.jstor.org/stable/3996200

Brullo S, Erben M (2016) The genus *Limonium* (Plumbaginaceae) in Greece. Phytotaxa 240(1): 001–212. https://doi.org/10.11646/phytotaxa.240.1.1

Brullo S, Guarino R (2000) Contribution to the knowledge of flora and vegetation of Khrisi islet (Crete, SE Mediterranean Sea). Flora Mediterranea 10: 265–282.

Brullo S, Guarino R (2017) *Limonium* Mill. In: Pignatti S, Guarino R, La Rosa M, (Eds) Flora d'Italia, vol. 2, 2nd ed. Edagricole, Milan, 18–48.

Bullock AA (1948) On the Identity of *Statice tetragona* Thunb. Kew Bulletin 3(3): 368. https://doi.org/10.2307/4108842

Bunge A (1872) Die Gattung *Acantholimon* Boiss. [Mémoires de l'Académie Impériale des Sciences de St.-Pétersbourg, ser. VII, 18 (1)]. Commissionaries de l'Académie Impériale des Sciences, St.-Pétersbourg, 72 pp. https://doi.org/10.5962/bhl.title.41374

Buzurović U, Stevanović V, Niketić M, Jakovljević K, Tomović G (2013) On the distribution of *Goniolimon tataricum* (Plumbaginaceae) in Serbia. Botanica Serbica 37(2): 167–172.

Buzurović U, Tomović G, Niketić M, Bogdanović S, Aleksić JM (2020) Phylogeographic and taxonomic considerations on *Goniolimon tataricum* (Plumbaginaceae) and its relatives from south-eastern Europe and the Apennine Peninsula. Plant Systematics and Evolution 306, 29 (2020). https://doi.org/10.1007/s00606-020-01636-0

Cabezudo B, Casimiro-Soriguer Solanas F, Pérez-Latorre AV (2015) Sobre el género *Armeria* en la provincia de Málaga (Andalucía, España). Acta Botanica Malacitana 40: 57–70. https://doi.org/10.24310/abm.v40i0.2493

Celep F, Karabacak O, Malekmohammadi M, Fidan M, Doğan M (2016) First record of *Psylliostachys spicata* (Plumbaginaceae), confirmation of *Salvia pratensis* (Lamiaceae) from Turkey, and taxonomic status of *Salvia ertekinii*. Turkish Journal of Botany 40(2): 226–230. https://10.3906/bot-1503-48

Chevalier AJB (1935) Catalogue des plantes spontanées, introduites ou cultivées. Revue de Votanique Appliquee et d'Agriculture Tropicale 15: 873–1074.

Christenhusz MJM, Byng JW (2018) 282. Plumbaginaceae. In Christenhusz MJM, Fay MF, Byng JW (Eds) Plant gateway's, the global flora, a particular flora to vascular plant species of the world, special edition, GLOVAP Nomenclatural part 1, 4. Plant Gateway Ltd., Bradford, United Kingdom, 144.

Crespo MB, Lledó MD (1998) El género *Limonium* Mill. (Plumbaginaceae) en la Comunidad Valenciana: taxonomía y conservación. Generalitat Valenciana, Consellería de Medio Ambiente, Valencia, 116 pp.

Crespo MB, Lledó MD (2000) Two new North African genera related to *Limoniastrum* (Plumbaginaceae). Botanical Journal of the Linnean Society 132(2): 165−174. https://doi.org/10.1111/j.1095-8339.2000.tb01212.x

Cuccuini P, Nepi C, Abuhadra MN, Banfi E, Domina G, Luccioli E, Miranda S, Pagitz K, Thiv M, Vela E (2016) The Libyan collections in FI (Herbarium Centrale Italicum and Webb Herbarium) and studies on the Libyan flora by R. Pampanini, Part 2. Flora Mediterranea 26: 81−143. https://doi.org/10.7320/FlMedit26.081

Cufodontis G (1960) Enumeratio plantarum aethiopiae spermatophyta (sequentia). Bulletin du Jardin botanique de l'État a Bruxelles 30(4): 653−708.

Daveau J (1888) Contributions pour l'étude de la flore Portugaise, Plumbaginées du Portugal, 1st partie. Boletim da Sociedade Broteriana 6: 145−187.

Guacchio del E, Erben M, Caputo P (2019) The neglected name *Statice auriculifolia* (Plumbaginaceae) and its related names: A long history of nomenclatural intricacy. Taxon, 68(5): 1093−1100. https://doi.org/10.1002/tax.12125

Didukh Ya, Korotchenko I, Fitsailo T, Burda R, Moysiyenko I, Pashkevich N, Yakushenko D, Shevera M (2010) Family Limoniaceae Lincz. (Plumbaginaceae Lindl.). In: Didukh Ya (Ed) Ecoflora of Ukraine, vol. 6. Phytosociocentre, 43 pp.

Dimopoulos P, Raus T, Bergmeier E, Constantinidis T, Iatrou G, Kokkini S, Strid A, Tzanoudakis D (2013) Vascular plants of Greece: an annotated checklist. Berlin: Botanischer Garten und Botanisches Museum Berlin-Dahlem; Athens: Hellenic Botanical Society. [Englera 31].

Dimopoulos P, Raus T, Bergmeier E, Constantinidis T, Iatrou G, Kokkini S, Strid A, Tzanoudakis D (2016) Vascular plants of Greece: an annotated checklist. Supplement. Willdenowia 46(3): 301−347. https://doi.org/10.3372/wi.46.46303

Dobignard A, Chatelain C (2013) Plumbaginaceae. In: Index synonymique de la flore d'Afrique du Nord, vol. 5. Ville de Genève: Éditions des Conservatoire et Jardin Botaniques, pp. 118−142.

Doğan M, Akaydın G (2007) Synopsis of Turkish *Acantholimon* Boiss. (Plumbaginaceae). Botanical Journal of the Linnean Society 154(3): 397−419. https://doi.org/10.1111/j.1095-8339.2007.00663.x

Doğan M, Akaydın G (2017) Two new species of *Limonium* Mill. (Plumbaginaceae) from southwest Anatolia, Turkey. Ot Sistematik Botanik Dergisi 24(2):13−28.

Doğan M, Akaydın G, Erdal J (2020) Phytogeography, ecology and conservation of the genus *Limonium* (Plumbaginaceae) in Turkey and a taxonomic revision. Plant Systematics and Evolution 306, 89 (2020). https://doi.org/10.1007/s00606-020-01706-3

Domina G (2011+) Plumbaginaceae. In: Greuter W, Raab-Straube E von (Eds) Euro+Med Notulae, vol. 5. Willdenowia 41: 131. Available online: https://europlusmed.org. [Accessed 19 February 2024].

Domina G, Erben M, Raimondo FM (2008) Two new species of *Limonium* (Plumbaginaceae) from Lebanon. Flora Mediterranea 18: 333−339.

Dyer RA (1961) Plumbaginaceae. In: Various Authors, Notes and New Records of African Plants. Bothalia 7(3): 488–491. https://doi.org/10.4102/abc.v7i3.1673

Dyer RA (1961) Plumbaginaceae. In: *Various authors.* Notes and new records of African plants. Bothalia 7(3): 488−491. https://doi.org/10.4102/abc.v7i3.1673

Emberger L, Maire R (1941) Catalogue des plantes du Maroc, vol. 4. Algiers: Imprimerie Minerva, Algiers.

Erben M (1978) Die Gattung *Limonium* im südwestmediterranen Raum. Mitteilungen der Botanischen Staatssammlung München 14: 361–631

Erben M (1988) Bemerkungen zur Taxonomie der Gattung *Limonium*. IV. Mitteilungen der Botanischen Staatssammlung München 27: 381–406.

Erben M (1989) Bemerkungen zur Taxonomie der Gattung *Limonium*. V. Mitteilungen der Botanischen Staatssammlung München 28: 313–417.

Erben M (1991) Bemerkungen zur Taxonomie der Gattung *Limonium* VI. Mitteilungen der Botanischen Staatssammlung München 30: 459–478.

Erben M (1993) *Limonium* Mill. In: Castroviejo S, et al. (Eds) Flora iberica, vol. 3. Consejo Superior de Investigaciones Cientificas, C.S.I.C., Madrid, 2–143.

Erben M (2001) Bemerkungen zur Taxonomie der Gattung *Limonium* VII. Sendtnera - Mitteilungen der Botanischen Staatssammlung und des Instituts fuer Systematische Botanik der Universitaet Muenchen 7: 53–84.

Erben M (2012) Contributions to the taxonomy of the genus *Limonium* IX. Flora Mediterranea 22: 63–66. https://doi.org/10.7320/FlMedit22.063

Erben M, Christodoulou CS, Hand R, Kefalas K (2022) *Limonium ammochostianum*, *L. karpasiticum* and *L. paralimniticum* (Plumbaginaceae), three new endemic species from the eastern part of Cyprus. Flora Mediterranea 32: 35–45. https://doi.org/10.7320/FlMedit32.035

Erben M, Del Guacchio E, Caputo P (2018) A nomenclatural study on *Statice densiflora* (Plumbaginaceae) and its linked names. Phytotaxa, 369(3): 185–199. https://doi.org/10.11646/phytotaxa.369.3.2

Ferrer-Gallego PP, Iamonico D, Iberite M, Laguna E, Crespo MB (2014) Lectotypification of two names in *Limoniastrum* (Plumbaginaceae). Taxon 63(6): 1342–1346. http://dx.doi.Org/10.12705/6

Ferrer-Gallego PP, Laguna E, Roselló R, Peris JB, Gómez-Navarro J (2018) *Limonium albarracinense* (Plumbaginaceae), una nueva especie para la flora Ibérica (España). Novon 26(1): 28–36. https://doi.org/10.3417/2017041

Ferrer-Gallego PP, Roselló R, Laguna E, Peris JB (2018) Sobre el lectótipo de *Limonium aragonense (Plumbaginaceae).* Collectanea Botanica 37 (2018): e010. https://doi.org/10.3989/collectbot.2018.v37.010

Fournier PV (1937) Les quatre flores de la France. Poinson-les-Grancey, France, 1091 pp.

Friis I, Wilmot-Dear M, Edmondson JR, Wondafrash M, Demissew S (2018) The genus *Plumbago* (Plumbaginaceae) in Ethiopia and Eritrea II. Webbia 67: 157–177. https://doi.org/10.1080/00837792.2012.10670916

Fuertes Aguilar J, Nieto Feliner G (2003) Additive polymorphisms and reticulation in an ITS phylogeny of thrifts (*Armeria*, Plumbaginaceae). Molecular Phylogenetics and Evolution 28(3): 430–447. https://doi.org/10.1016/S1055-7903(02)00301-9

Gams H (1927) Plumbaginaceae. In: Hedgi G (Ed) Illustrierte Flora von Mittel-Europa. 5(3): 1877–1897.

Ghazanfar SA, Edmondson JR (2003) Two new species of *Limonium* (Plumbaginaceae) from the sultanate of Oman, Arabia. Edinburgh Journal of Botany 60(1): 11–18. https://doi.org/10.1017/S0960428603000027

Giardina G, Raimondo FM, Spadaro V (2007). A catalogue of plants growing in Sicily. Bocconea 20: 5–582.

Govaerts R (ed.) (2023) WCVP: World Checklist of Vascular Plants version 12. Facilitated by the Royal Botanic Gardens, Kew. https://doi.org/10.34885/jdh2-dr22

Graham R (2014) Systematics of *Dyerophytum* (Plumbaginaceae) with a focus on heterostyly and the evolution of endemic taxa from Socotra. Thesis submitted in partial fulfilment for the MSc in the Biodiversity and Taxonomy of Plants. Royal Botanic Garden of Edinburgh, Edinburgh, 117 pp.

Gray AM, Duretto MF (2011) *Limonium australe* var. *baudinii* (Lincz.) A.M.Gray, a new combination in Plumbaginaceae. Kanunnah 4: 117–118.

Greuter W, Burdet H, Long G (1989) Med-Checklist, vol. 4 (Lauraceae-Rhamnaceae). Editions des Conservatoire et Jardin Botaniques de Genève, Geneva.

Greuter W, Raus T (1987) Med-Checklist Notulae, 14. Willdenowia 16(2): 439–452.

Greuter W, Raus T (1989) Med-Checklist Notulae, 15. Willdenowia 19(1): 22–48.

Guacchio del E, Erben M, Iamonico D (2018) The Linnaean names in *Statice* (Plumbaginaceae). Taxon 67(3): 606−610. https://doi.org/10.12705/673.1

Hansen A, Sunding P (1993) Flora of Macaronesia. Checklist of vascular plants 4, revised edition. Sommerfeltia 17: 1–295. https://doi.org/10.2478/som-1993-0003

Hanson AD, Rathinassabapathi B, Rivoal J, Burnet M, Dillon M0, Gage DA (1994) Osmoprotective compounds in the Plumbaginaceae: A natural experiment in metabolic engineering of stress tolerance. Proceedings of the National Academy of Sciences of the United States of America 91: 306–310.

Harborne JB (1967) Comparative biochemistry of the flavonoids. IV. Correlations between chemistry, pollen morphology and systematics in the family Plumbaginaceae. Phytochemistry 6: 1415−1428. https://doi.org/10.1016/S0031-9422(00)82884-8

Hernández-Ledesma P, Berendsohn WG, Borsch T, Mering S von, Akhani H, Arias S, Castañeda-Noa I, Eggli U, Eriksson R, Flores-Olvera H, Fuentes-Bazán S, Kadereit G, Klak C, Korotkova N, Nyffeler R, Ocampo G, Ochoterena H, Oxelman B, Rabeler RK, Sanchez A, Schlumpberger BO, Uotila P (2015) A taxonomic backbone for the global synthesis of species diversity in the angiosperm order Caryophyllales. Willdenowia 45(3): 281−383. https://doi.org/10.3372/wi.45.45301

Iamonico D (2024) Epitypification of the Linnaean *Statice echioides* (Plumbaginaceae) and notes on Smith's concepts of *S. aristata* and *S. rorida*. Taxon 2024, 1-3. https://doi.org/10.1002/tax.13165

Iamonico D, De Castro O, Di Iorio E, Nicolella G, Iberite M (2022) Taxonomy complexity of some Tyrrhenian endemic *Limonium* species belonging to *L. multiforme* group (Plumbaginaceae): new insights from molecular and morphometric analyses. Plants 11(22): 3163. https://doi.org/10.3390/plants11223163

Ingrouille MJ (1985) The *Limonium auriculae-ursifolium* (Pourret) Druce group (Plumbaginaceae) in the Channel Isles. Watsonia 15(3): 221–229.

Ingrouille MJ, Stace CA (1986) The *Limonium binervosum* aggregate (Plumbaginaceae) in the British Isles. Botanical journal of the Linnean Society 92(3): 177–217. https://doi.org/10.1111/j.1095-8339.1986.tb01428.x

IPNI (2000+) International Plant Names Index. The Royal Botanic Gardens, Kew, Harvard University Herbaria & Libraries and Australian National Botanic Gardens. http://www.ipni.org

Karis PO (2004) Taxonomy, phylogeny and biogeography of *Limonium* sect. *Pteroclados* (Plumbaginaceae), based on morphological data. Botanical Journal of the Linnean Society 144: 461–482. https://doi.org/10.1111/j.1095-8339.2003.00260.x

Keighery G, Muir W (2008) Vegetation and vascular flora of Faure Island, Shark Bay, Western Australia. Records of the Western Australian Museum Supplement 75: 11–19.

Koutroumpa K, Theodoridis S, Warren BH, Jiménez A, Celep F, Doğan M, Romeiras MM, Santos-Guerra A, Fernández-Palacios JM, Caujapé-Castells J, Moura M, Menezes de Sequeira M, Conti E (2018) An expanded molecular phylogeny of Plumbaginaceae, with emphasis on *Limonium* (sea lavenders): taxonomic implications and biogeographic considerations. Ecology and Evolution 8 (24): 12397–12424. https://doi.org/10.1002/ece3.4553

Koutroumpa K, Warren BH, Theodoridis S, Coiro M, Romeiras MM, Jiménez A, Conti E (2021) Geo-Climatic changes and apomixis as major drivers of diversification in the Mediterranean Sea Lavenders (*Limonium* Mill.). Frontiers in Plant Science 11:612258. https://doi.org/10.3389/fpls.2020.612258

Kubitzki K (1993) Plumbaginaceae. In: Kubitzki K, Rohwer JG, Bittrich V (Eds) The families and genera of vascular plants, vol. 2. Springer, Berlin, Heidelberg, 523–530.

Kunkel G (1980) Die Kanarischen Inseln und ihre Pflanzenwelt. Gustav Fischer V. Stuttgart, Germany.

Kunkel G, Sunding P (1967) Enumeration of *Limonium* Mill. (Plumbaginaceae) in the Canary Islands, with nomenclatural notes. Cuadernos de Botanica 2: 9–13.

Kuntze CEO (1891) Plumbaginaceae. In: Kuntze CEO (Ed) Revisio generum plantarum, vol. 2. Arthur Felix, Leipzig, 393–397.

Lazkov GA, Sultanova BA (2011) Checklist of vascular plants of Kyrgyzstan. Norrlinia 24: 1–166.

Linczevski IA (1952) Plumbaginaceae Lindl, except the genera *Plumbaginella*, *Armeria*, *Psylliostachys*. In: Shishkin BK, Bobrov EG (Eds) Flora SSSR, vol. 18. Botanicheskii Institut Akademii Nauk SSSR, Moskva-Lenjingrad, 293–474. [English translation: Israel Program for Scientific Translations, Jerusalem, 1967]

Linczevski IA (1961) Generis Acantholimon Boiss. Species novae ex Asia Madia. Botanicheskie materialy gerbariya Botanicheskogo Instituta Imeni VL Komarova Akademii Nauk SSSR, Leningrad [St. Petersburg] 21: 485–498.

Linczevski IA (1971) Notes on Limoniaceae 3. Botanicheskii Zhurnal 56(11): 1633–1635.

Linczevski IA (1979) Notes on Limoniaceae 4. Novosti Sistematiki Vysshikh Rastenii, 16: 166–168.

Linczevski IA (1980) Notes on Limoniaceae 5. Novosti Sistematiki Vysshikh Rastenii 17: 208–211.

Linczevski IA (1986) Limoniaceae Lincz. In: Nikiforova GT (Ed) Flora Tadzhikskoĭ SSR, vol. 8. Nauka, Leningrad, 10–52. [In Russian]

Lindley J (1846) The vegetable kingdom; or the structure, classification and uses of plants illustrated upon the natural system, vol. 1. Bradbury and Evans, London, 194 pp.

Lledó MD, Crespo MB, Cameron KM, Fay MF, Chase MW (1998) Systematics of Plumbaginaceae based upon cladistic analysis of rbcL sequence data. Systematic Botany 23(1): 21−29. https://doi.org/10.2307/2419571

Lledó MD, Crespo MB, Cox AV, Chase MW, Fay M (2000) Polyphyly of *Limoniastrum* (Plumbaginaceae): evidence from DNA sequences of plastid *rbc*L, *trn*L intron and *trn*L-F intergene spacer. Botanical Journal of the Linnean Society 132(2): 175–191. https://doi.org/10.1006/boj1.1999.030

Lledó MD, Crespo MB, Fay M, Chase MW (2005a) Molecular phylogenetics of *Limonium* and related genera (Plumbaginaceae): biogeographical and systematics implications. American Journal of Botany 92(7): 1189–1198. https://doi.org/10.3732/ajb.92.7.1189

Lledó MD, Erben M, Crespo MB (2003) *Myriolepis*, a new genus segregated from *Limonium* (Plumbaginaceae) Taxon 52(1): 67−73. https://doi.org/10.2307/3647438

Lledó MD, Erben M, Crespo MB (2005b) *Myriolimon*, a new name for the recently published *Myriolepis* (Plumbaginaceae). Taxon 54(3): 811–812. https://doi.org/10.2307/25065443

Lledó MD, Karis PO, Crespo MB, Fay M, Chase MW (2001) Phylogenetic position and taxonomic status of the genus *Aegialitis* and subfamilies Staticoideae and Plumbaginoideae (Plumbaginaceae): evidence from plastid DNA sequences and morphology. Plant Systematics and Evolution 229: 107–124. https://doi.org/10.1007/s006060170021

López-Alvarado J, Cobacho I, Aran VJ, Rosato M, Rossello JA, Saez L (2017) *Limonium ilergabonum* (Plumbaginaceae), a new species from northeastern Iberian Peninsula. Phytotaxa 331: 199–212. https://doi.org/10.11646/phytotaxa.331.2.4

Luteyn JL (1976) Revision of *Limonium* (Plumbaginaceae) in eastern North America. Brittonia 28 303–317. https://doi.org/10.2307/2805792

Mabberley DJ, Malécot V (2023) Hippolyte Arnaud's account of *Limonium* (Plumbaginaceae) in cultivation and the further nomenclatural importance of both Seringe's and Jacques' Flore des Jardins. Lejeunia Revue de Botanique, Nouvelle série 209: 2–31. https://institut-agro-rennes-angers.hal.science/hal-04098697

Mahmoodi M, Assadi M (2021) A new species of the genus *Acantholimon* (Plumbaginaceae) from Iran. The Iranian Journal of Botany 27(2): 71–77. https://doi.org/10.22092/ijb.2021.356512.1341

Mahmoodi M, Ghahremaninejad F, Maassoumi AA (2015) Validation of the names of four Iranian plant taxa. Phytotaxa 227(1): 99–100. https://doi.org/10.11646/phytotaxa.227.1.11

Maire R (1934) Plumbaginaceae. In: Jahandiez E, Maire R (Eds) Catalogue des plantes du Maroc, vol. 3. Algiers: Imprimerie Minerva, Algiers, Algiers, 564–572.

Malagarriga RP (1968) Notas Fitocorologicas, primera serie. Acta Phytotaxonomica Barcinonensia 1: 1–100.

Malekmohammadi M, Akhani H, Borsch T (2017) Phylogenetic relationships of *Limonium* (Plumbaginaceae) inferred from multiple chloroplast and nuclear loci. Taxon 66: 1128–1146. https://doi.org/10.12705/665.8

Malekmohammadi M, Lack HW, Lomonosova MN, Akhani H (2017) The discovery, naming and typification of *Limonium gmelini* (Plumbaginaceae). Willdenowia 47(2): 99–106. https://doi.org/10.3372/wi.47.47201

Malekmohammadi, M., Koutroumpa, K., Crespo, M. B., Domina, G., Korotkova, N., Akhani, H., Mering S von, Borsch, T. & Berendsohn, W. G. (Submitted) A species level taxonomic backbone for the Plumbaginaceae (Caryophyllales). Current study.

Maury P (1886) Études sur l'organisation et la distribution géographique des Plumbaginacées. Annales des Sciences Naturelles 7(4): 1–134.

Memariani F, Akhani H, Joharchi MR (2016) Endemic plants of Khorassan-Kopet Dagh floristic province in Irano-Turanian region: diversity, distribution patterns and conservation status. Phytotaxa 249(1): 31–117. https://doi.org/10.11646/phytotaxa.249.1.5

Miller P (1768) The gardeners dictionary, ed. 8. Printed for the author, and sold by John and Francis Rivington [and 23 others], London. https://doi.org/10.5962/bhl.title.541

Mobayen S (1964) Revision taxonomique du genre *Acantholimon*. PhD Thesis, Université de Montpellier, France.

Moharrek F, Kazempour Osaloo S, Assadi M (2014) Molecular phylogeny of Plumbaginaceae with emphasis on *Acantholimon* Boiss. based on nuclear and plastid DNA sequences in Iran. Biochemical Systematics and Ecology 57: 117–127. https://doi.org/10.1016/j.bse.2014.07.02

Moharrek F, Kazempour-Osaloo S, Assadi M, Nieto Feliner G (2017). Molecular phylogenetic evidence for a wide circumscription of a characteristic Irano-Turanian element: *Acantholimon* (Plumbaginaceae: Limonioideae). Botanical Journal of the Linnean Society 184(3): 366–386. https://doi.org/10.1093/botlinnean/box033

Moreno J, Terrones A, Alonso MÁ, Juan A, Crespo MB (2016). *Limonium tobarrense* (Plumbaginaceae), a new species from the southeastern Iberian Peninsula. Phytotaxa 257(1): 61–70. https://doi.org/10.11646/phytotaxa.257.1.4

Moreno J, Terrones A, Alonso MA, Juan A, Crespo MB (2018). Taxonomic revision of the *Limonium latebracteatum* group (Plumbaginaceae), with the description of a new species. Phytotaxa 333(1): 41–57. https://doi.org/10.11646/phytotaxa.333.1.3

Morin NR (2005) Plumbaginaceae. In: Flora of North America Editorial Committee (Eds.) Flora of North America North of Mexico, vol. 5. Oxford University Press, New York and Oxford, 602–611. Available online at: http://floranorthamerica.org

Mucina L, Hammer TA (2019) *Limonium dagmarae* (Plumbaginaceae), a new species from Namaqualand Coast, South Africa. Phytotaxa 403(2): 071–085. https://doi.org/10.11646/phytotaxa.403.2.1

Nieto Feliner G (1985) Estudio critico de la flora orofila del suroeste de Leon: Montes Aquilianos, Sierra del Teleno y Sierra de la Cabrera. Ruizia 2: 1–239.

Nieto Feliner G (1987) The genus *Armeria* (Plumbaginaceae) in the Iberian Peninsula: comments and new data for a review. Anales Jardin Botanico de Madrid 44(2): 319–348.

Nieto Feliner G (1990) *Armeria*. In: Castroviejo S (Ed) Flora iberica, vol. 2. Real Jardín Botánico, Consejo Superior de Investigaciones Cientificas, C.S.I.C, Madrid, 642–721.

Nyman CF (1881) Plumbaginaceae. In: Conspectus florae europaeae, vol. 3. Typis Officinae Bohlinianae, Örebro (Sueciae), 602–616. https://doi.org/10.5962/bhl.title.10533

Oganesian ME (2011) Genus *Acantholimon* Boiss. (Plumbaginaceae) in South Transcaucasia (Armenia, Nakhichevan). Takhtajania 1: 51–59.

Peng TH, Kamelin RV (1996) Plumbaginaceae. In: Wu ZY, Raven PH (Eds) Flora of China (Myrsinaceae through Loganiaceae), vol. 15. Science Press, Beijing, and Missouri Botanical Garden Press, St. Louis, 190–204.

Peruzzi L, Domina G, Bartolucci F, Galasso G, Peccenini S, Raimondo FM, Albano A, Alessandrini A, Banfi E, Barberis G, Bernardo L, Bovio M, Brullo S, Brundu G, Brunu A, Camarda I, Carta L, Conti F, Croce A, Iamonico D, Iberite M, Iiriti G, Longo D, Marsili S, Medagli P, Pistarino A, Salmeri C, Santangelo A, Scassellati E, Selvi F, Soldano A, Stinca A, Villani M, Wagensommer RP, Passalacqua NG (2015) An inventory of the names of vascular plants endemic to Italy, their loci classici and types. Phytotaxa 196(1): 1–217. https://doi.org/10.11646/phytotaxa.196.1.1

Pignatti S (1955) Studi sulla flora e vegetazione dell'isola di Mallorca (Baleari), II. I *Limonium* della flora balearica. Archivio Botanico (Forlì) 31: 65–100.

Pignatti S (1962) Studi sui *Limonium*, V. Note sulla sistematica delle specie iberiche di *Limonium*. Collectanea Botanica (Barcelona) 6(6): 293–330.

Pignatti S (1971) Studi sui *Limonium*, VIII. Botanical Journal of the Linnean Society 64(4): 361–370.

Pignatti S (1972) Plumbaginaceae. In: Tutin TG, Heywood VH, Burges NA, Moore DM, Valentine DH, Walters SM, Webb DA (Eds) Flora Europaea, vol. 3. Cambridge University Press, Cambridge, 29–51.

Pignatti S (1982) New species of *Limonium* from Italy and Tunesia. Webbia 36(1): 47–56. https://doi.org/10.1080/00837792.1982.10670239

POWO (2017+) http://www.plantsoftheworldonline.org. Plants of the World Online. Facilitated by the Royal Botanic Gardens, Kew [Accessed 1 August 2023]

Raimondoa FM, Domina G (2009) new species of Limonium (Plumbaginaceae) from Soqotra (Yemen). Plant Biosystems 143(30): 504–508. https://doi.org/10.1080/11263500903086278

Rechinger KH (1943) Flora Aegea. Flora der Inseln und Halbinseln des Ägäischen Meeres. Denkschriften Akademie der Wissenschaften in Wien, Mathematisch-Naturwissenschaftliche Klasse 105(1).

Rechinger KH, Schiman-Czeika H (1974) Plumbaginaceae. In: Rechinger H (Ed), Flora Iranica, vol. 108. Graz: Akademische Druck-u. Verlagsanstalt.

Reveal JL (2012) Newly required infrafamilial names mandated by changes in the code of nomenclature for algae, fungi, and plants. Phytoneuron 33: 1–32.

Roshkova OI (1952) *Psylliostachys*. In: Shishkin BK, Bobrov EG (Eds.) Flora SSSR, vol. 18. Botanicheskii Institut Akademii Nauk SSSR, Moskva-Lenjingrad, 467–474. [English translation: Israel Program for Scientific Translations, Jerusalem, 1967]

Rosselló JA, Mus M, Soler JX (1994) *Limonium ejulabilis*, a new endagered endemic species from Majorca (Balearic Islands, Spain). Anales del Jardin Botanico de Madrid 51: 199–204.

Sáez L, Curcó A, Rosselló JA (1998) *Limonium vigoi* (Plumbaginaceae) a new tetraploid species from the Northeast of the Iberian Peninsula. Anales del Jardin Botánico de Madrid 56(2): 269–278. https://doi.org/10.3989/ajbm.1998.v56.i2.233

Salmon CE (1907) Notes on *Limonium*. Journal of Botany 45: 24–25.

Salmon CE (1911) Notes on *Limonium.* Journal of Botany 49: 73–77.

Salmon CE (1924) Short notes. *Statice* v. *Limonium*. Journal of Botany 62: 335–336.

Sampaio G (1913) Lista das espécies representadas no herbário Português. Tipografia Costa Carregal, Porto, 148 pp.

Sauvage C, Vindt J (1952) Flore du Maroc: analytique, descriptive et illustrée. Editions Internationales, Série Botanique 4, Travaux de l'Institut Scientifique Chérifien, Tanger, 148 pp.

Schultes JA (1820) *Armeria, Statice* and *Aegialitis*. In: Schultes JA (Ed) Systema Vegetabilium, vol. 6. Stuttgardtiae: Sumtibus JG Cottae, Stuttgart, 771– 799.

Sefi O, Ghrabi-Gammar Z, Ben Haj Jilani I, Daoud-Bouattour A, Brullo S (2020) A new species of *Limonium* from Tunisia. Phytotaxa 446: 281–290. https://doi.org/10.11646/phytotaxa.446.5.2

Sell PD (2018) Plumbaginaceae. In: Sell PD, Murrell G (Eds) Flora of Great Britain and Ireland, vol. 1. Cambridge University Press, Cambridge, 536–559.

Sennen F (1927) Nos découvertes en Cerdagne. Bulletin de la Société Botanique de France 73: 641–680. https://doi.org/10.1080/00378941.1926.10833624

Sennen F (1936) Diagnoses des nouveautés parues dans les exsiccata plantes d'Espagne et du Maroc de 1928 à 1935. Imp. Anglada.-Vic, Vic, 308.

Sennen F, Mauricio H (1933) Catálogo de la flora del Rif oriental y principalmente de las Cabilas limitrofes con Melilla. Gráficas La Ibérica, Marina, Melilla, 159 pp.

Serra-Laliga L (2007) Estudio crítico de la flora vascular de la Provincia de Alicante aspectos nomenclaturales, biogeográficos y de conservación. Ruizia, Monografías del Jardin Botanico 19: 1–1414.

Soler JX, Rosello JA (1997) On the status of *Statice dianiae* Pau (Plumbaginaceae). Anales del Jardín Botánico de Madrid 55(1): 9–15. https://doi.org/10.3989/ajbm.1997.v55.i1.256

Stace CA (1975) Hybridization and the flora of the British Isles, Academic Press. 1–626.

Sventenius ER (1960) Additamentum ad floram canariensem. Agronomiarum Investigationem Nationale Hispanicum Institutum, Madrid, 136 pp.

Thulin M (2006) Plumbaginaceae. In: Thulin M (Ed) Flora of Somalia, vol. 3, Royal Botanic Gardens, Kew, 25–30.

Tiburtini M, Astuti G, Bartolucci F, Casazza G, Varaldo L, De Luca D, Bottigliero MV, Bacchetta G, Porceddu M, Domina G, Orsenigo S (2022) Integrative taxonomy of *Armeria arenaria* (Plumbaginaceae), with a special focus on the putative subspecies endemic to the Apennines. Biology 11: 1060. https://doi.org/10.3390/biology11071060

Tiburtini M, Bacchetta G, Sarigu M, Cambria S, Caputo P, De Luca D, Domina G, Turini A, Peruzzi L (2023) Integrative taxonomy of *Armeria* taxa (Plumbaginaceae) endemic to Sardinia and Corsica. Plants 12: 2229. https://doi.org/10.3390/plants12112229

TPL (2013) The Plant List Version 1.1. Published on the Internet. http://www.theplantlist.org/

Tzvelev NN (2012) Plumbaginaceae. In: Takhtajan AL (Ed) Konspekt flory Kavkaza, vol. 3(2). KMK Scientific Press, St. Petersburg & Moscow, 272–284.

Vallariello R, Iamonico D, Del Guacchio E (2016) Typification of three accepted names in *Limonium* (Plumbaginaceae). Phytotaxa 263: 131–138. https://doi.org/10.11646/phytotaxa.263.2.5

Vilmorin-Andrieux E (1870) Les fleurs de pleine terre comprenant la description et la culture des fleurs annuelles, vivaces et bulbeuses de pleine terre suivies de classements divers indiquant l'emploi de ces plantes et l'époque de leur floraison de plans de jardins avec des examples de leur ornementation en divers genres, etc. Premiere Partie, ed. 3. Chez Vilmorin-Andrieux & Cie, Paris. 1–990.

Wagenitz G (1962) Zur Kenntnis der Flora und Vegetation Anatoliens (Ergebnisse einer Reise im Herbst 1957). Willdenowia 3(2): 221–288.

Wangerin W (1921) Generis *Statice* species et varietates novae, I. Repertorium Novarum Specierum Regni Vegetabilis 17(19-30): 398–402.

Weber-El Ghobary MO (1984) The systematic relationships of *Aegialitis* (Plumbaginaceae) as revealed by pollen morphology. Plant Systematics and Evolution 144: 53−58.

WFO (2018) World Flora Online taxonomic backbone data export for Caryophyllales, dated 2 feb 2018, received from William Ulate, Missouri Botanical Garden

Wu YH, Yang YC (2006) A new variety of the genus *Limonium* from Qinghai Province, China. Wuhan Botanical Research 24(4): 323–323.

Yildirim H, Crespo MB (2014) *Acantholimon riyatguelii* (Plumbaginaceae), a threatened new unarmed species from Central Anatolia, Turkey. Phytotaxa 175(2): 073–084. https://doi.org/10.11646/phytotaxa.175.2.2

Yıldırımlı Ş (2009) Miscellaneous nomenclatural and taxonomic corrections on Turkish plants, 2. Ot Sistematik Botanik Dergisi 16(1): 9–14.

Zhao YJ, Liu J, Yin GS, Gong X (2023) Characteristics of plastid genomes in the genus *Ceratostigma* inhabiting arid habitats in China and their phylogenomic implications. BMC Plant Biology 23: 303. https://doi.org/10.1186/s12870-023-04323-7

Zuloaga FO, Belgrano MJ, Zanotti CA (2019) Actualización del catálogo de las plantas vasculares del Cono Sur. Darwiniana, nueva serie 7(2): 208–278. http://dx.doi.org/10.14522/darwiniana.2019.72.861
