# Supplementary figures and images for: ﻿A taxonomic backbone for the Plumbaginaceae (Caryophyllales)
Source: PhytoKeys. 2024 Jun 20;243:67–103. doi: 10.3897/phytokeys.243.122784 (PMC11211657; doi:10.3897/phytokeys.243.122784)

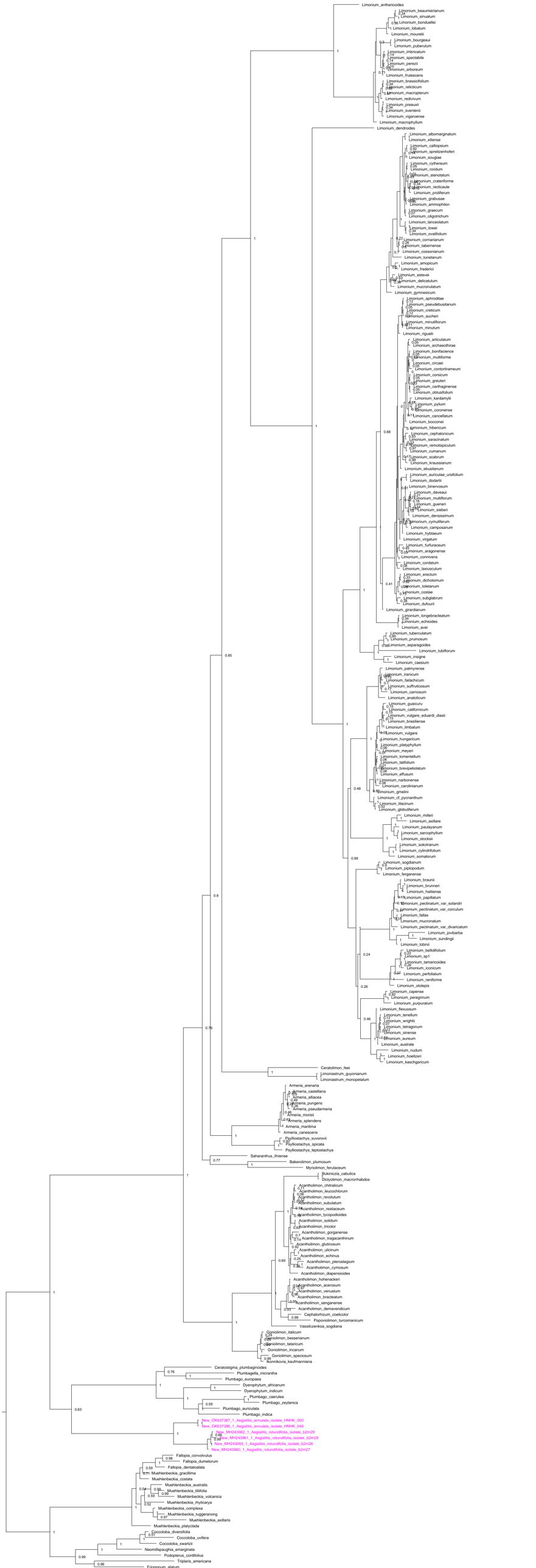

Supplement: Supplementary material 2 — Phylogenetic relationship of Plumbaginaceae using Polygonaceae outgroups [file phytokeys-243-067_article-122784__-s002.pdf]
